# Conserved organ-specific microbial assemblages in different populations of a terrestrial crab

**DOI:** 10.3389/fmicb.2023.1113617

**Published:** 2023-06-12

**Authors:** Giovanni Bacci, Sara Fratini, Niccolò Meriggi, Christine L. Y. Cheng, Ka Hei Ng, Massimo Pindo, Alessio Iannucci, Alessio Mengoni, Duccio Cavalieri, Stefano Cannicci

**Affiliations:** ^1^Department of Biology, University of Florence, Sesto Fiorentino, Italy; ^2^NBFC, National Biodiversity Future Center, Palermo, Italy; ^3^The Swire Institute of Marine Science, The University of Hong Kong, Pokfulam, Hong Kong SAR, China; ^4^Research and Innovation Centre, Fondazione Edmund Mach, San Michele all’Adige, Italy

**Keywords:** bacterial communities, organ-associated microbiota, sesarmid crab, coastal forests, host-microbe associations

## Abstract

Microorganisms are ubiquitous in the environment and provide genetic and physiological functions to multicellular organisms. Knowledge on the associated microbiota is becoming highly relevant to understand the host’s ecology and biology. Among invertebrates, many examples of endosymbiosis have been described, such as those in corals, ants, and termites. At present, however, little is known on the presence, diversity, and putative roles of the microbiota associated to brachyuran crabs in relation to their environment. In this work we investigated the associated microbiota of three populations of the terrestrial brachyuran crab *Chiromantes haematocheir* to find evidence of a conserved organ-specific microbiome unrelated to the population of origin and dissimilar from environmental microbial assemblages. Bacterial 16S rRNA gene and fungal ITS sequences were obtained from selected crab organs and environmental matrices to profile microbial communities. Despite the presence of truly marine larval stages and the absence of a gregarious behaviour, favouring microbiota exchanges, we found common, organ-specific microbiota, associated with the gut and the gills of crabs from the different populations (with more than 15% of the genera detected specifically enriched only in one organ). These findings suggest the presence of possible functional roles of the organ-specific microbiota.

## 1. Introduction

Microorganisms are ubiquitous in natural environments and play critical roles in many ecological and biogeochemical processes. They can form strict interactions with multicellular organisms and can represent a critical component of the organism’s ecology and biology (Jones, 2013). In this view, host and microbes can be considered as inextricable parts of the same meta-organism, contributing to its fitness and its adaptation to the surrounding environment (Theis et al., 2016). Since microorganisms perform a large variety of metabolic processes that are absent in the host, these biological interactions often lay the foundation for an evolutionarily stable unit (the meta-organism) positively influencing its development, physiology, survival, and fitness (Theis et al., 2016).

Within this framework, the concepts of holobiont as originally described (Margulis and Fester, 1991) and hologenome (Rosenberg et al., 2007; Zilber-Rosenberg and Rosenberg, 2008; Bordenstein and Theis, 2015) have recurrently emerged to study the interactions between hosts and their associated microbial communities. Rosenberg et al. (2009) argued that the holobiont (and consequently the hologenome) highlights Lamarckian aspects contextualized in a Darwinian framework and theorised that microorganisms can be lost and acquired from the environment and then transmitted to the offspring according to the Lamarckian principle of ‘inheritance of acquired characteristics’. An organism can create stable associations with some environmental microorganisms, which it interacts with.

The first critical step to disentangle the evolutionary development of these ecological interactions is to define how much the microbial signature is shared between the organism and its environment. Many studies on animal microbiomes performed to date focused on model species, such as humans and mice. From an ecological perspective, our understanding of invertebrate–microbiome interactions has advanced mostly in arthropods, with regard to their gut microbiomes (Engel and Moran, 2013). Famous examples are the studies on termites’ ability to degrade lignocellulose through mutualistic associations with microbial symbionts (Scharf, 2015); the studies on ants in relation to food supplementations (Zientz et al., 2005; Russell et al., 2009; Anderson et al., 2012) and those on the species belonging to the order Hemiptera that feed on nutritionally poor food items as plant sap or animal fluids (Kuechler et al., 2012).

Terrestrial isopods of the family Oniscidea are another example of extensively studied arthropods in the context of animal-microbe interactions of ecological relevance—once again with a focus on the gut—due to the numerous ecological functions that these species play in terrestrial ecosystems (Bouchon et al., 2016). These fully terrestrial arthropods base their diet on difficult-to-digest vascular plant compounds (i.e., cellulose, lignin, and polyphenols) whose digestion and assimilation are guaranteed by bacterial symbionts located in their digestive tissues, specifically those present in the hepatopancreas (the digestive midgut glands) (Bouchon et al., 2016).

Decapoda represents the most specious crustacean order with more than 2,700 genera and 17,000 species distributed worldwide, in marine and freshwater ecosystems (De Grave et al., 2009). Many Decapods, among them a wide range of species of lobsters, shrimps, prawns and swimming crabs, are also economically important for fisheries and aquaculture. Due to such diversity, ecological plasticity and economic importance, their biology has been extensively studied. Only a few studies, however, have been performed on the microbiomes associated to their organs, with some exception represented by the cultured species belonging to the genera *Penaeus*, *Litopenaeus*, *Macrobrachium* and *Portunus* (Chen et al., 2021; Garibay-Valdez et al., 2021; Tang et al., 2021; Gao et al., 2022; Zhang and Sun, 2022). Among the other few available studies on brachyuran crabs, recent papers on the Chinese mitten crab *Eriochioner sinensis* (Brachyura; Varunidae), a commercially important estuarine crab, that record a low microbial diversity associated to its gills and intestine in comparison to the microbial diversity present in the environment and specific organ-associated microbial communities (Chen et al., 2015; Zhang et al., 2016, 2017; Sun et al., 2020). Biswas et al. (2022) analysed the gut microbiota of three crab species, *Scylla serrata*, *Episesarma versicolor* and *Uca rosea,* to find a close association between their associated microbiomes and the environment. Béziat et al. (2021) profiled the microbial communities harboured on the gills of two Caribbean mangrove crabs, *Aratus pisonii* and *Minuca rapax*, and found that their associated bacterial communities were species-specific despite living in the same habitat.

In this study we aimed to investigate the presence of host- and organ-specific microbiota in a terrestrial brachyuran crab. Our model species was *Chiromantes haematocheir*, a common inhabitant of lowland forests of East Asia (Abd El-Wakeil, 2009; Miyake et al., 2019), of which we sampled different populations and applied targeted metabarcoding approaches to profile both prokaryotic and fungal communities from different key organs (i.e., gills, gonads, and guts) hypothesising the presence of stable host-microbe associations different from the microbial communities harboured in their environment (i.e., sediment, leaf litter, freshwater, and freshwater debris). Our null hypothesis was thus that microbial composition of crab organs could show the same microbial signature displayed by the matrices from the surrounding environments. This hypothesis implies that the crab organs would host bacterial and fungal populations acquired from the environment without any specific selection and could be ultimately explained by a dynamic “loss and acquire” balance preventing the development of stable host-microbe associations. Within this theoretical framework, we could expect that the gut microbiome would be mainly shared with those present on leaf litter and in sediment (which represent the main food for this species), and the gill microbiome could be similar to the ones found in water and water debris. Our alternative hypothesis was that crab organs could harbour exclusive bacterial and/or fungal communities, in terms of diversity and composition, when compared to those present in the environment. This working hypothesis implies the presence of an active selection process in favour of specific microbial assemblages conserved at organ level, likely associated with the ecology of this sesarmid crab.

## 2. Materials and methods

### 2.1. Study species

*Chiromantes haematocheir* (Decapoda; Brachyura; Sesarmidae) is a semi-terrestrial crab colonising the coastal vegetated areas from Taiwan to South East Asia (Abd El-Wakeil, 2009; Schubart and Ng, 2020). In Hong Kong, it lives in areas of lowland secondary forest adjacent to mangroves and in pockets of riverine forests, where it was also observed climbing trees (pers. obs.). Very little is known about its ecology, apart from the fact that it digs deep burrows that maintains throughout its life, it mainly feeds on decay leaves and leaf litter and, as many sesarmids, it releases pelagic larvae into the ocean (Abd El-Wakeil, 2009; Miyake et al., 2019).

### 2.2. Sample collection and DNA extraction

**Figure 1:**
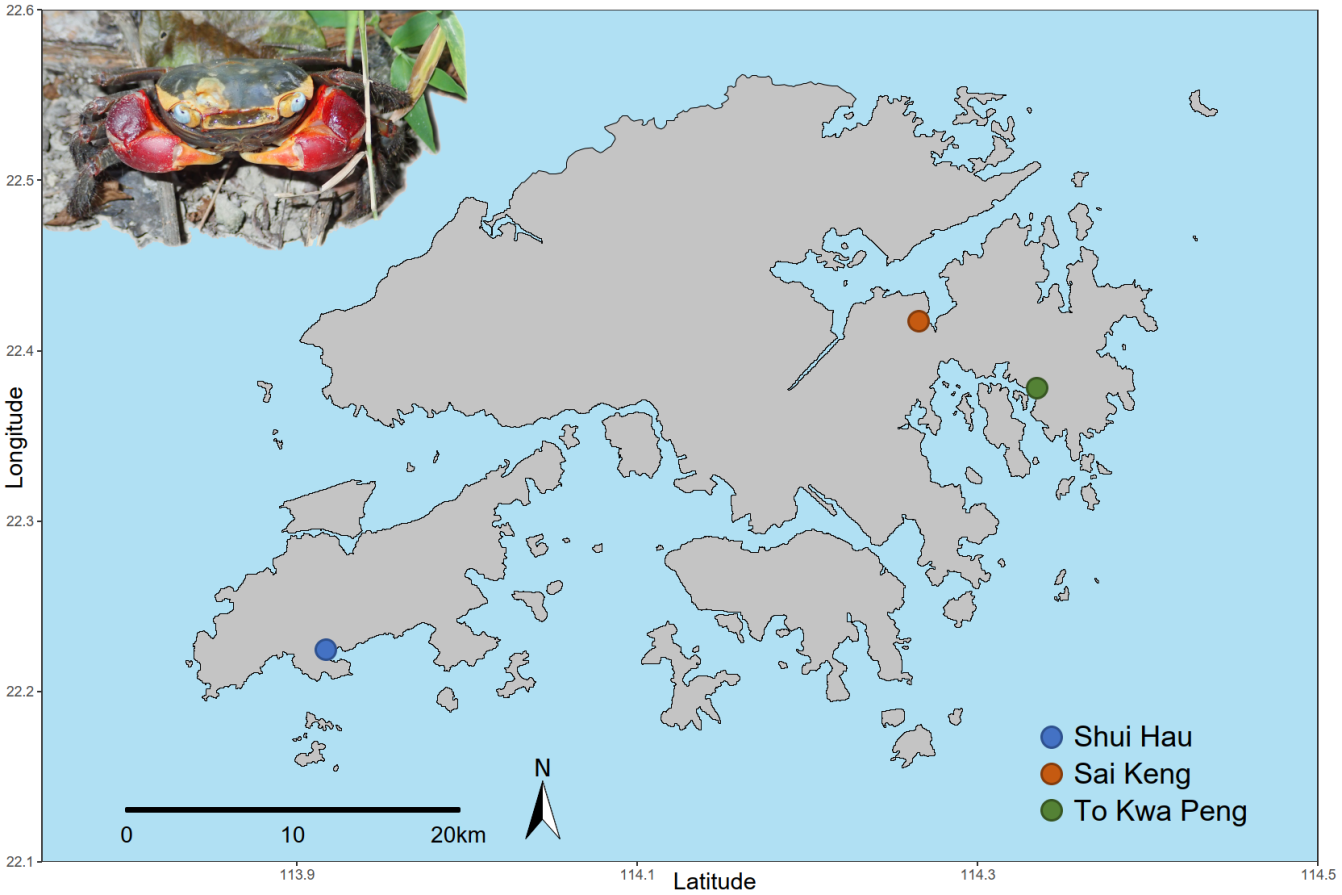
Geographical distribution of the sampled populations of *C. haematocheir*. The map shows the names and location of the three sampling sites visited for the study in the New Territories and on Lantau Island and an adult male *C. haematocheir* in its natural environment (top left).

We selected three large populations of *C. haematocheir* that colonised distant catchments across the Hong Kong territory. The selected populations were sampled at Shui Hau (Southern Lantau Island, 22.219 N, 113.919 E), To Kwa Peng (Eastern coast of Sai Kung Country Park, New Territories, 22.488 N, 114,333 E), and Sai Keng (Three Fathoms Cove, Tolo Harbour, New Territories, 22.421 N, 114.268 E) (Figure 1). From each site, eleven sexually mature adult crabs (carapace width range between 13.4 mm and 32.2 mm) were collected in October 2018. Animal samplings were performed in compliance with local and institutional laws.

To explore differences in microbial community composition across the crab’s organs and the surrounding environment, we collected and analysed four organ samples (gills, hindgut, midgut, and gonads) and four environmental matrices (sediment, leaf litter, freshwater, and freshwater debris: five samples for each environmental matrix per population). Soil samples (topsoil layer) were taken at 5 cm depth (Pérez-Valera et al., 2020). A total of 100 mL of freshwater per sample was collected and stored (Rud et al., 2017), then filtered by using 0.2 μm Nalgene™ Sterile Analytical Filter Units (Thermo Fisher Scientific). Freshwater sediment debris were taken at 0–5 cm depth underwater (Song et al., 2020). All samples were collected using sterile instruments and placed in sterile tubes (Nunc™ 15 mL and 50 mL Conical Sterile Polypropylene Centrifuge Tubes – Thermo Fisher Scientific) to avoid contaminations. Due to the intensive sampling, all environmental samples and crabs were immediately frozen and subsequently transported to the laboratories of the Swire Institute of Marine Science (The University of Hong Kong). The dissections were then performed under sterile conditions. All dissection instruments were sterilized over an open flame to eliminate residual DNA and washed with 75% EtOH to prevent cross-contamination. After removing the carapace, gills, gonads, hindgut and midgut from each crab were excised under a stereomicroscope and stored at −20°C in RNAlater (Thermo Fisher Scientific) stabilization solution until DNA extraction.

Total DNA extraction from crab organs, sediment, leaf litter and freshwater debris was performed using the DNeasy PowerLyzer PowerSoil Kit (QIAGEN) following manufacturer’s protocol. Total DNA extraction from water samples was performed using the DNeasy PowerWater Kit (QIAGEN) following manufacturer’s protocol. Extracted DNA samples were stored at −20°C. Before the DNA library’s preparation, DNAs were quantified fluorometrically by using Qubit dsDNA HS Assay Kit (Thermo Fisher Scientific).

### 2.3. 16S (V3-V4) rRNA gene amplification and sequencing

The preparation and sequencing of the 16S rRNA library were performed at the Laboratory of Advanced Genomics, Department of Biology, University of Florence (Firenze, Italy). PCR amplifications of the bacterial V3-V4 16S rRNA gene fragments were performed using KAPA HiFi HotStart ReadyMix (Roche) and the primer pair 341F (5’-CCTACGGGNGGCWGCAG-3′) and 805R (5’-GACTACNVGGGTWTCTAATCC-3′) (Rosso et al., 2018) with overhang Illumina adapters. Amplification of the 16S rRNA was performed using 2× KAPA HiFi HotStart ReadyMix (Roche) in a GeneAmp PCR System 2700 (Thermo Fisher Scientific) according to manufacturer specification. All PCR products were checked through electrophoresis on 1.5% agarose gel and then purified using KAPA Pure Beads (Roche) following the manufacturer’s instructions. To apply the Illumina adapters sequencing indexing using Nextera XT Index Kit V2 (Illumina), a second PCR amplification was then performed by preparing a reaction mix in accordance with the 16 S metagenomic library preparation protocol (Illumina, 2013). An indexing step was made for all samples by seven PCR cycles. Amplicon products from indexing PCR were purified using KAPA Pure Beads (Roche) and their quality check was performed using Agilent 2100 Bioanalyzer (Agilent Technologies) with Agilent DNA 1000 Kit (Agilent Technologies). Subsequently, concentration check was performed by Qubit dsDNA HS Assay Kit (Thermo Fisher Scientific). Finally, the barcoded libraries were balanced and pooled at equimolar concentration, before being sequenced on an Illumina MiSeq (PE300) platform (MiSeq Control Software 2.6.2.1).

### 2.4. ITS1 rDNA region sequencing

ITS1 library preparation and sequencing were performed at the Research and Innovation Centre, Fondazione Edmund Mach (FEM) (S. Michele all’Adige, Trento, Italy). Fungal ITS1 fragments were amplified by PCR using the FastStart High Fidelity PCR System (Roche) for environment matrixes and the Hot Start High-Fidelity DNA Polymerase (NEB) for animal matrixes following the manufacturer instructions using the primers ITS1F (5′- CTTGGTCATTTAGAGGAAGTAA-3′) (Gardes and Bruns, 1993) and ITS2 (5’-GCTGCGTTCTTCATCGATGC-3′) (White et al., 1990) with overhang Illumina adapters. ITS1 PCRs were performed in a GeneAmp PCR System 9,700 (Thermo Fisher Scientific) according to manufacturer specification.

All PCR products were checked on 1.5% agarose gel and purified using the CleanNGS kit (CleanNA, the Netherlands) following the manufacturer’s instructions. Subsequently a second PCR was performed to apply the Illumina sequencing adapters Nextera XT Index Primer (Illumina) (Illumina, 2013). The indexing step was made for all samples by seven PCR cycles. After Indexing PCR amplicon libraries were purified using the CleanNGS kit (CleanNA, the Netherlands), and the quality control was performed on a Typestation 2,200 platform (Agilent Technologies, Santa Clara, CA, United States). Afterwards all barcoded libraries were mixed at equimolar concentration, quantified by qPCR Kapa Library quantification kit (Roche) and sequenced on an Illumina MiSeq (PE300) platform (MiSeq Control Software 2.5.0.5 and Real-Time Analysis software 1.18.54.0).

### 2.5. Amplicon sequence variant inference

The DADA2 pipeline version 1.14.1 (Callahan et al., 2016) was used to infer amplicon sequence variants (ASVs) from raw sequences. Primers used for PCR amplification were removed using cutadapt version 1.15 (Martin, 2011) in paired-end mode. If a primer was not found, the sequence was discarded together with its mate to reduce possible contamination. For ITS amplicon sequences reads containing both the forward and reverse primers were considered valid only if concordant (one of the two primers must be present but reverse and complemented) with a cut-off length of 70 bp. Low quality reads were discarded using the “filterAndTrim” function with an expected error threshold of 2 for both forward and reverse read pairs (namely only reads with more than 2 expected errors were removed). Denoising was performed using the “dada” function after error rate modelling (“learnErrors” function). Denoised reads were merged discarding those with any mismatches and/or an overlap length shorter than 20 bp (“mergePairs” function). Chimeric sequences were removed using the “removeBimeraDenovo” function.

Taxonomical classification was performed using DECIPHER package version 2.14.0 against the latest version of the pre-formatted Silva small-subunit reference database (Quast et al., 2012) (SSU version 138 available at: http://www2.decipher.codes/Downloads.html) and the Warcup database for fungal ITS1 (Deshpande et al., 2016). All variants not classified as Bacteria, Archaea or Fungi were removed together with sequences classified as chloroplasts or mitochondria (16S rRNA sequences only). Additional information on the sequence variant inference pipeline used were reported in Supplementary materials. The number of reads retained in each step of the pipeline described above was reported in Supplementary Figure S1.

### 2.6. Inferring functional content of amplicon variants

The genome content of bacterial ASVs was inferred using PICRUSt2 pipeline (Douglas et al., 2020). Enzyme Commission Numbers (EC numbers) were converted into Gene Ontology terms (GO terms) using the mapping file available at: http://www.geneontology.org/external2go/ec2go. Gene abundance was retrieved using the “--strtified” option to report gene abundances at species level (ASVs). Additional information about functional content inference was reported in Supplementary material.

### 2.7. Statistical analyses

All statistical analyses were performed in the R environment (version 3.6). Briefly, alpha- and beta-diversity analyses were conducted using the vegan package version 2.5 (Oksanen et al., 2019) in combination with the iNEXT package version 2.0 (Hsieh et al., 2016). Differences between sample types and sampling sites were assessed by using permutational multivariate analysis of variance (PERMANOVA) based on Bray-Curtis diversity index whereas differences in dispersion were tested using one-way analysis of variance (ANOVA). To dampen the hypothesis that extremely rare species may be responsible for major differences in community distribution, we did not consider ASVs that appeared only once in a sample when estimating beta-diversity indices. Principal coordinate analysis (also called PCoA or classical multidimensional scaling, MDS) was used to inspect the distribution of samples according to different sampling sites and sample types. Normalization and differential abundance analyses were performed with DESeq2 version 1.28 (Love et al., 2014) whereas enrichment analysis of taxa and functions was performed using hypergeometric test (“phyper” function of R stats package). For additional details about tests data manipulation see Supplementary materials.

## 3. Results

### 3.1. Microbial composition of crab’s organs and environmental samples

Sequencing produced 25,875,448 sequences for prokaryotic 16S rRNA amplicon and 15,788,842 sequences for the fungal internal transcribed region 1 (ITS1) that were clustered according to DADA2 pipeline (see Supplementary methods for additional details). After the clustering pipeline two fungal samples (namely GI_TKP_5 and MG_SH_1) produced no counts in any of the fungal amplicon sequence variants (ASVs) and were therefore removed from subsequent analyses. Since several fungal samples reported a low number of reads in crab’s organs, two samples were replicated and tested for correlation and accuracy to evaluate the presence of fungal cells in the crabs’ organs (Supplementary Figure S1 and Supplementary Table S1). Technical replicates showed an average correlation coefficient (Spearman’s rho) between replicates ranging between 0.74 and 0.99, with an accuracy higher than 75% in all contrasts. These results mean that the low recovery in biotic samples was not due to technical or analytical issues but corresponded to the scarce presence of fungal DNA (Supplementary Figure S2; Supplementary Table S2; and Supplementary method section).

**Figure 2:**
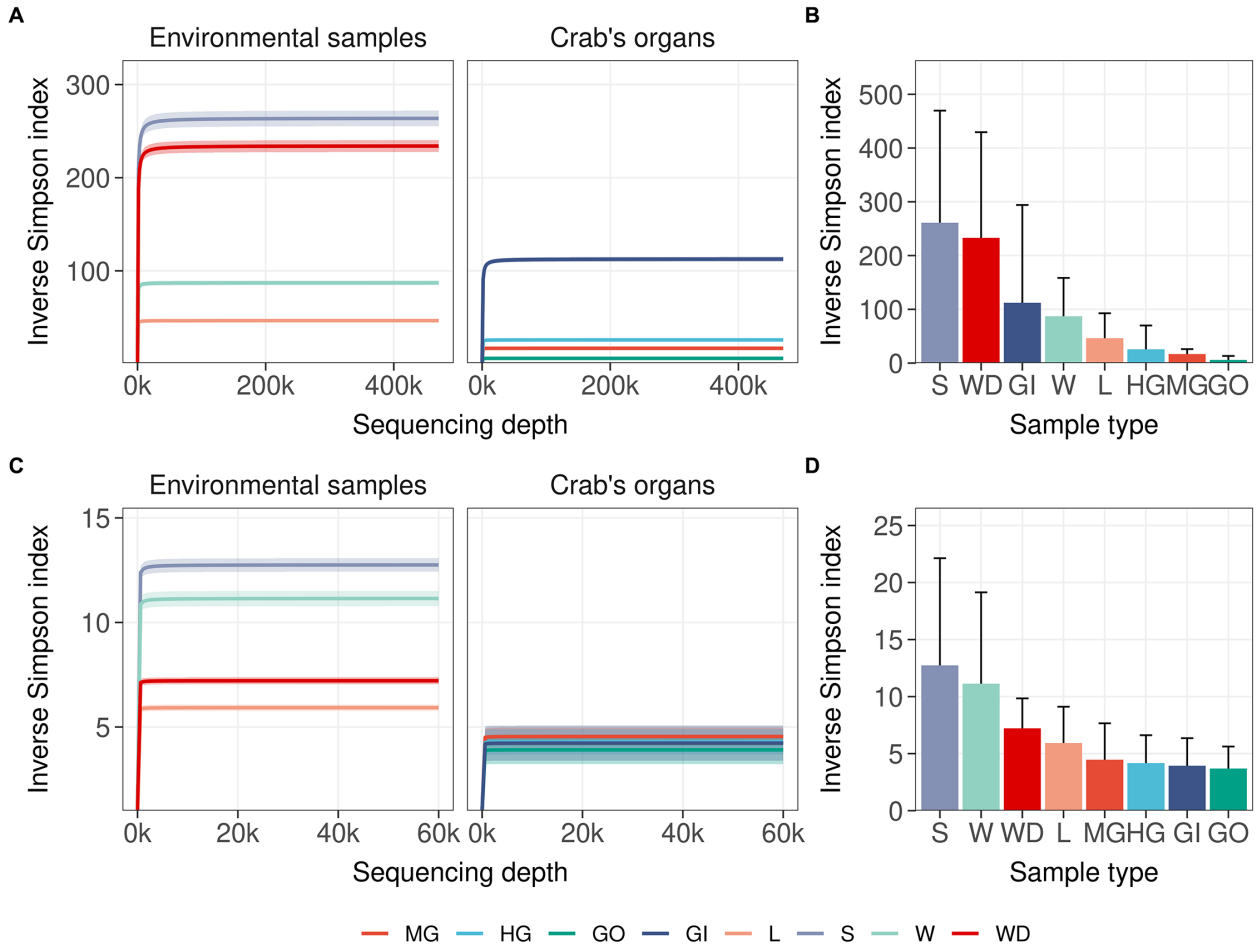
Microbial diversity in crab’s organs and environmental samples. The average of the inverse Simpson index was reported with increasing sampling effort for all types of samples. Interpolated and extrapolated diversity was reported in panels **(A)** and **(C)** (16S rRNA gene and ITS1 region, respectively) together with the 98% confidence limits of diversity (iNEXT package). Observed diversity was reported in panels **(B)** and **(D)** (16S rRNA gene and ITS1 region respectively) with error bars representing the standard deviation of the observed values of diversity. Colours and acronyms on the x-axis correspond to different sample types (MG, midgut; HG, hindgut; GO, gonads; GI, gills; L, litter; S, soil; W, water; WD, water debris).

Single nucleotide clustering detected a total of 56,233 ASVs including both 16S rRNA gene and ITS1 amplicons. The 16S rRNA gene amplicons produced 55,036 ASVs (97.87%), whereas the ITS1 region was clustered into 1,197 ASVs (2.13%). Most variants were assigned to the Bacteria domain (54,800 ASVs, 97.45% of the total ASVs detected) and Fungi kingdom, even if a sporadic presence of Archaea was found (236 ASVs, 0.42% of the total ASVs detected). In general, microbial diversity was higher in the environmental samples than in crab’s organs, except for the gills where we found a high diversity of 16S rRNA amplicons (Figures 2A,B; Supplementary Tables S3, S4). The fungal diversity was low in all crab’s organs, whereas environmental samples were characterised by a highly diverse fungal assemblage, with soil, water, and water debris reporting the highest diversities (Figures 2C,D). For each sample category and at each site, the difference between extrapolated diversity (namely the inverse Simpson index calculated by simulating a higher sequencing depth than the observed depth for each sample) and observed diversity was low (Table 1), meaning that our sampling effort was effective in assessing the natural microbial diversity for each category. Even the Good’s coverage estimator was higher than 99.9% in all sample types highlighting that only 0.1% of clustered sequences came from ASVs detected only once in the whole dataset (Table 1 and Supplementary Table S3). Microbial diversity was rather uniform across sampling sites, except for To Kwa Peng (TKP), which showed a significantly lower diversity with respect to the other sites in terms of ITS1 data (Supplementary Figure S3 and Supplementary Table S4).

**Table 1:** The differences between extrapolated Simpson diversity (Inverse Simpson index computed for a sequencing depth higher than the observed one) and observed diversity (Inverse Simpson index computed for a sequencing depth equal to the real sequencing depth of the sample) was reported using the average value ± the standard error on the mean for each sample type and site.

		Inverse Simpson (ext – obs)	Good’s coverage estimator
Sample type			
	MG	0.051 ± 0.040	99.995 ± 0.001
	HG	0.132 ± 0.126	99.997 ± 0.001
	GO	0.085 ± 0.029	99.987 ± 0.005
	GI	0.575 ± 0.230	99.994 ± 0.002
	L	0.185 ± 0.114	99.963 ± 0.007
	S	2.741 ± 0.809	99.993 ± 0.001
	W	0.246 ± 0.066	99.997 ± 0.001
	WD	1.149 ± 0.293	99.992 ± 0.002
Site			
	SH	0.705 ± 0.236	99.987 ± 0.003
	SK	0.502 ± 0.146	99.990 ± 0.002
	TKP	0.240 ± 0.067	99.996 ± 0.001

Good’s coverage estimator was also reported using the same notation used for Simpson diversity. Sample type abbreviations: MG, midgut; HG, hindgut; GO, gonads; GI, gills; L, litter; S, soil; W, water; WD, water debris. Site abbreviations: SH, Shui Hau; Sai Keng, SK; To Kwa Peng, TKP.

### 3.2. Microbial distribution across sample types

**Figure 3:**
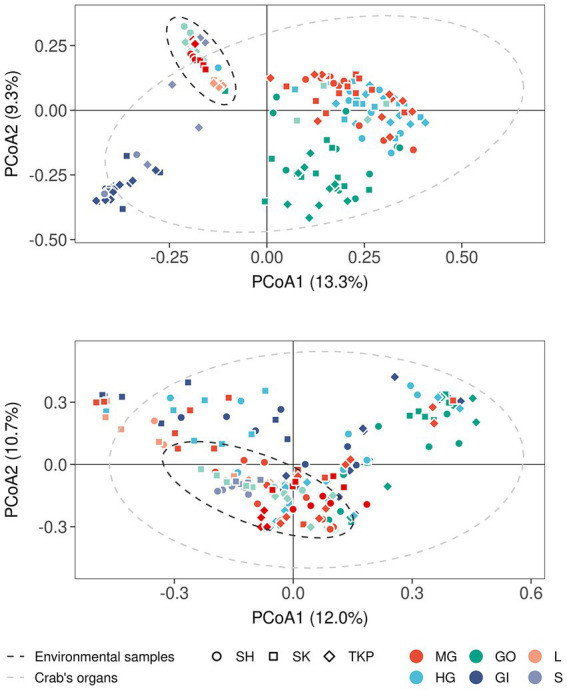
Microbial distribution according to Bray-Curtis distance. Principal coordinates analyses on Bray-Curtis distances inferred form 16S rRNA **(A)** and ITS1 metabarcoding **(B)**. The percentage of variance explained by each axis was reported between round brackets whereas different sample types were reported using different colors (MG, midgut; HG, hindgut; GO, gonads; GI, gills; L, litter; S, soil; W, water; WD, water debris). Sample collected from different sites were reported by using different shapes (SH, Shui Hau; SK, Sai Keng; TKP, To Kwa Peng) whereas the 95% confidence intervals of the distribution of environmental samples (dark gray ellipse) and crab’s organs (light gray ellipse) were reported by using dotted lines assuming a multivariate t-distribution.

The multidimensional ordination showed that both environmental samples and crab’s organs contributed to shape microbial community distribution, but their effect varied according to both the amplicon type and the category of sample considered (Figure 3 and Supplementary Figure S4A). In terms of composition, the microbial communities found in the environmental samples overlapped with each other more than the ones characteristic of the crab’s organs (Figures 3A,B). The PCoA built on 16S rRNA gene amplicons (Bacteria and Archaea) showed that soil and litter, and water and water debris, respectively, formed two distinct clusters (Figure 3A). The observed intra-cluster variability is due to differences in microbial communities found across the three Hong Kong sites (Figure 3A). The same 16S rRNA amplicon dataset showed a clear separation across crab’s organs, with a very limited, but significant, influence of the collection sites (Figure 3A and Supplementary Figure S4A). The gut’s microbiome composition was similar across the two sampled sections (mid- and hindgut), while the gills and gonads sharply separated from each other and the gut itself. These significant differences were highlighted also by the permutational analysis of variance (Supplementary Figure S4A and Supplementary Table S5). In contrast to 16S rRNA data, fungal distribution produced more overlap, especially across crab’s organs, with the gonads being the only organ to show significant differences when compared to the other organs (Figure 3B; Supplementary Figure S4A; and Supplementary Table S4).

**Figure 4:**
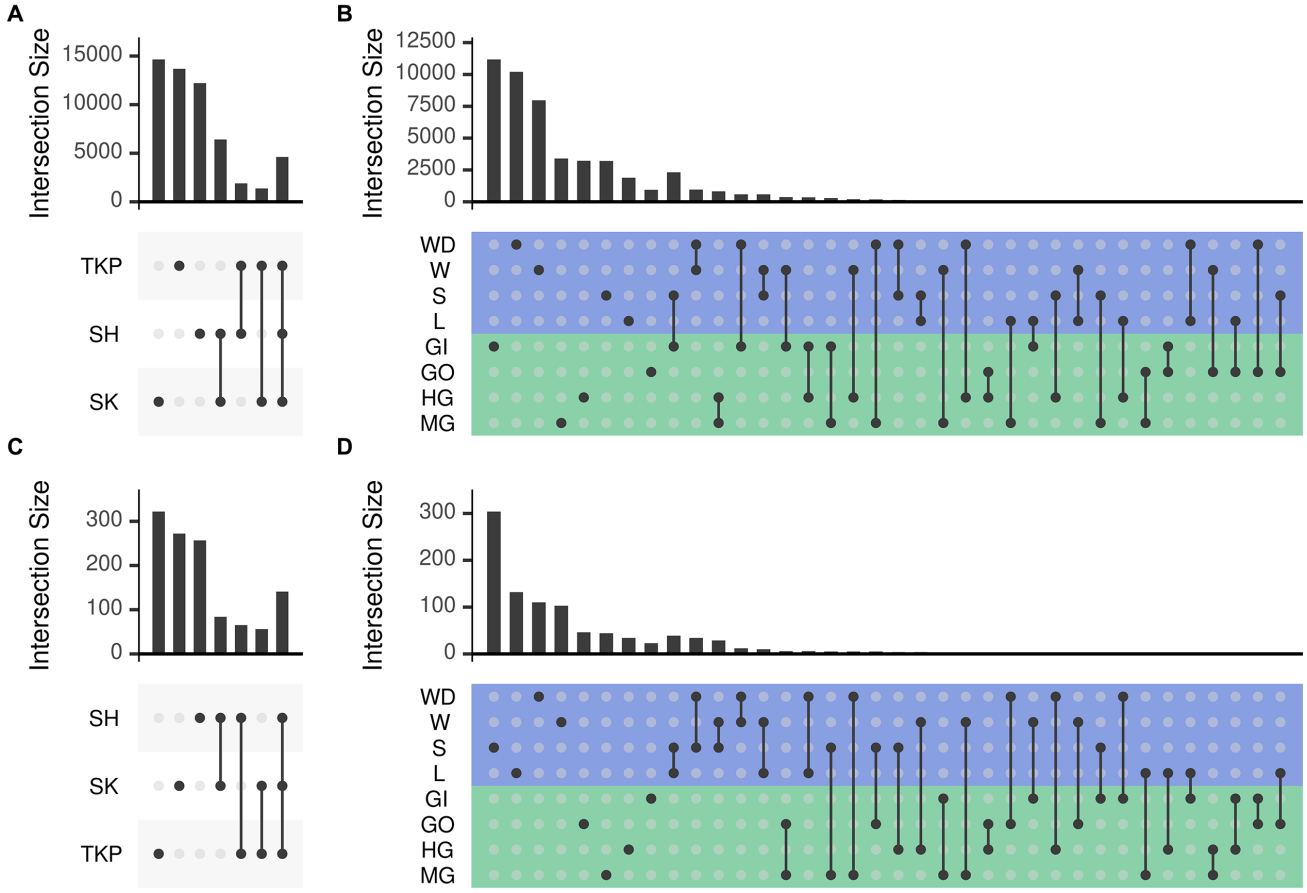
Number of ASVs shared across sampling sites and sample types. The number of ASVs in common (namely simultaneously detected with abundance higher than zero) between different sets was reported following the upset representation. This representation is conceptually similar to a Venn diagram, but intersections are reported as a matrix instead of using different shapes depending on the number of sets, which become very complex to understand with more than three or four sets. Panels a and b report the number of shared ASVs obtained from 16 s rRNA amplicon sequencing whereas panels b and d report those obtained from ITS1 sequencing. Set intersections were displayed in a matrix layout where each row is a different site (panels **A** and **C**) or sample type (panels **B** and **D**) and each column corresponds to a different intersection. In panels **(B)** and **(D)** the rows of the matrix were colored according to the sample type: green for crab’s organs and blue for environmental samples. Intersected sets are reported using points connected by a straight line. The number of ASVs contained in each intersection is reported using bars on top of the intersection considered. Intersections are mutually exclusive so that if an ASV is present in a given intersection it is excluded from the others. Sites and sample types were abbreviated as follows: TKP, To Kwa Peng; SK, Sai Keng; SH, Shui Hau; MG, midgut; HG, hindgut; GO, gonads; GI, gills; L, litter; S, soil; W, water; WD, water debris.

Both environmental samples and crab’s organs possessed a large set of microbial ASVs which were unique to the category considered (Figure 4) but the number of shared taxa across samples from the same organ was limited (from 0.2 to 3% for the 16S rRNA gene and from 0 to 0.4% for the ITS-1 region, Supplementary Figure S5). As already mentioned, sampling sites also contributed to shape microbial communities, but they predominantly affected environmental samples distribution and showed a low effect on crab’s organs (Supplementary Figure S4A; Figures 4A,C). In addition, the community composition of both prokaryotic and fungal communities was more variable and dispersed at site level than at organ level (Supplementary Figures S4, S5 and Supplementary Table S6), showing a higher specificity of such assemblages within the different organs and across sites (Figures 4B,D). Gills exchanged microbes with environmental matrices in direct contact with them (soil and water) and even with the gut, which is in turn connected with the same environmental matrices through defecation (Figures 4B,D).

### 3.3. Defining characteristic patterns across sample types

**Figure 5:**
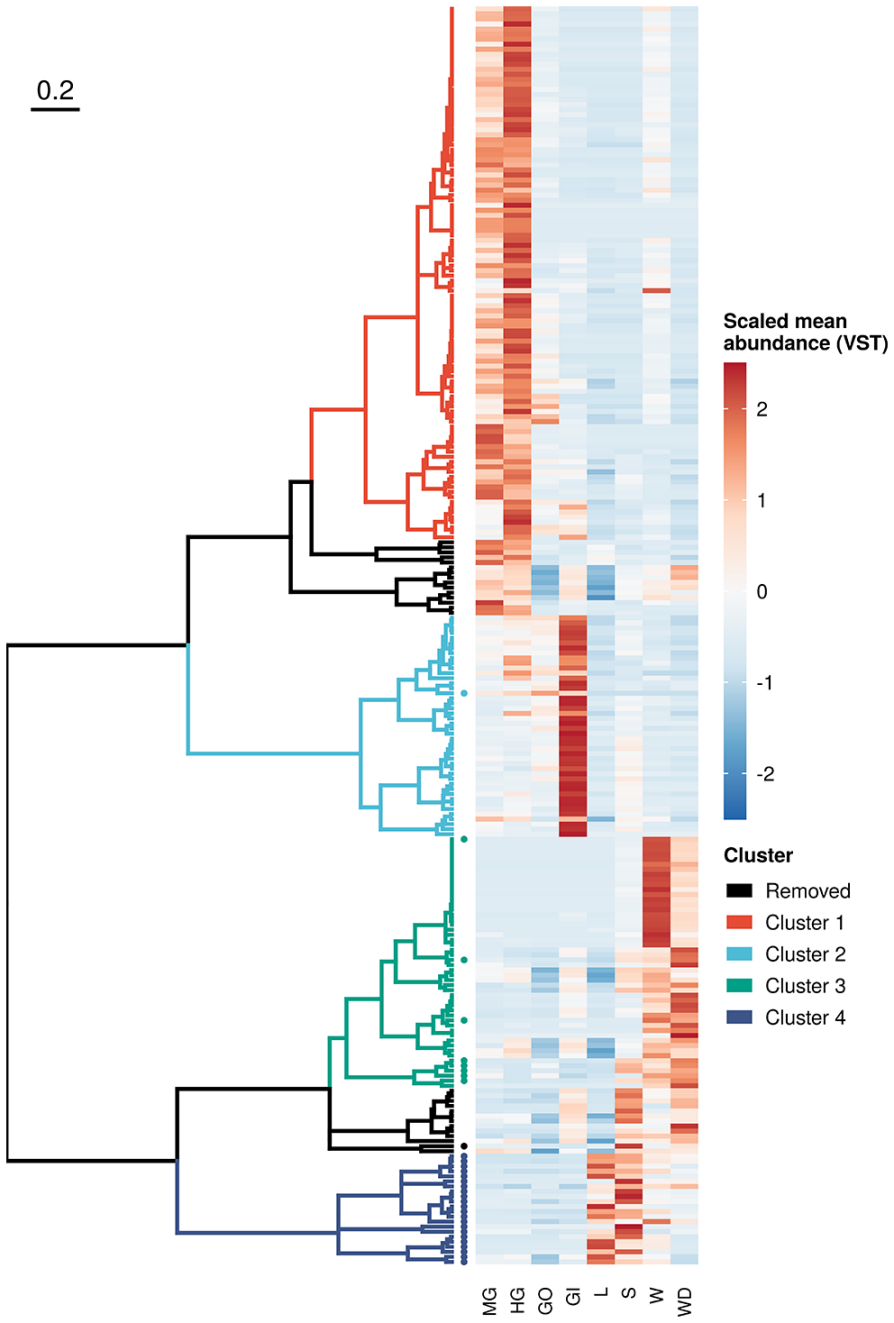
Sequence variant clustering according to their abundance along sample types. Amplicon sequence variants reporting a different abundance pattern in one or more sample types (loglikelihood ratio test of DESeq2) were clustered according to their mean variance-stabilized abundance. Abundance values were reported using different colours after clustering based on Kendall correlation (right side of the plot). Clusters were coloured according to the scheme reported in the legend whereas removed clusters (namely those composed with less than 10 variants) where reported in black. Sequence variants inferred from ITS-1 amplicon sequencing were reported using a solid dot.

To inspect microbial distribution in different sample categories, we performed log-likelihood ratio test on both prokaryotic and fungal communities using DESeq2, as detailed in Supplementary materials. We found 250 ASVs (218 bacteria and 32 fungi) reporting a different distribution across crab’s organs and/or environmental samples (Figure 5 and Supplementary Table S7). Even if significant ASVs corresponded to 0.45% of the ASVs profiled in the whole community (250 on 55,819), they accounted for more than 50% of the total microbial abundance (with a mean in each sample of 56.5% and a standard error of 2.10%) reflecting the presence of many rare and sporadic species throughout sample types and sites. Divisive analysis of hierarchical clustering obtained using variance-stabilized counts produced four distinct clusters, which show a peculiar pattern of abundance of ASVs clearly linked to the ecological and biological settings of the study. Indeed, clusters 1 and 2 (Figure 5 and Supplementary Figure S7) were composed by microbial assemblages highly represented in the crab’s organs, while the other two clusters, (namely, cluster 3 and 4) represented the microorganisms mostly found in the environmental samples (Figure 5 and Supplementary Figure S7). The clusters representing the microorganisms more abundant in the crab’s organs included bacterial ASVs only. These were split into two groups, formed by the bacteria of the gut (cluster 1) and those of the gills (cluster 2), respectively, with the latter being the smallest one. We found a very limited number of genera shared across organs/environments (from 0.2 to 3% of total genera inferred from 16S rRNA gene and from 0 to 0.4% of fungal genera), which can represent a soft-core set (sharing more than 70%) of genera from a given organs/environments (Supplementary Figure S5). This result is in line with the knowledge that host associated microbiota share a great number of microbial species with the environment, but those species may include some strains which can establish symbiotic relationships with the host and others which act as mere commensals (Theis et al., 2016).

### 3.4. Taxonomic and functional enrichments in crab’s organs and environmental samples

**Figure 6:**
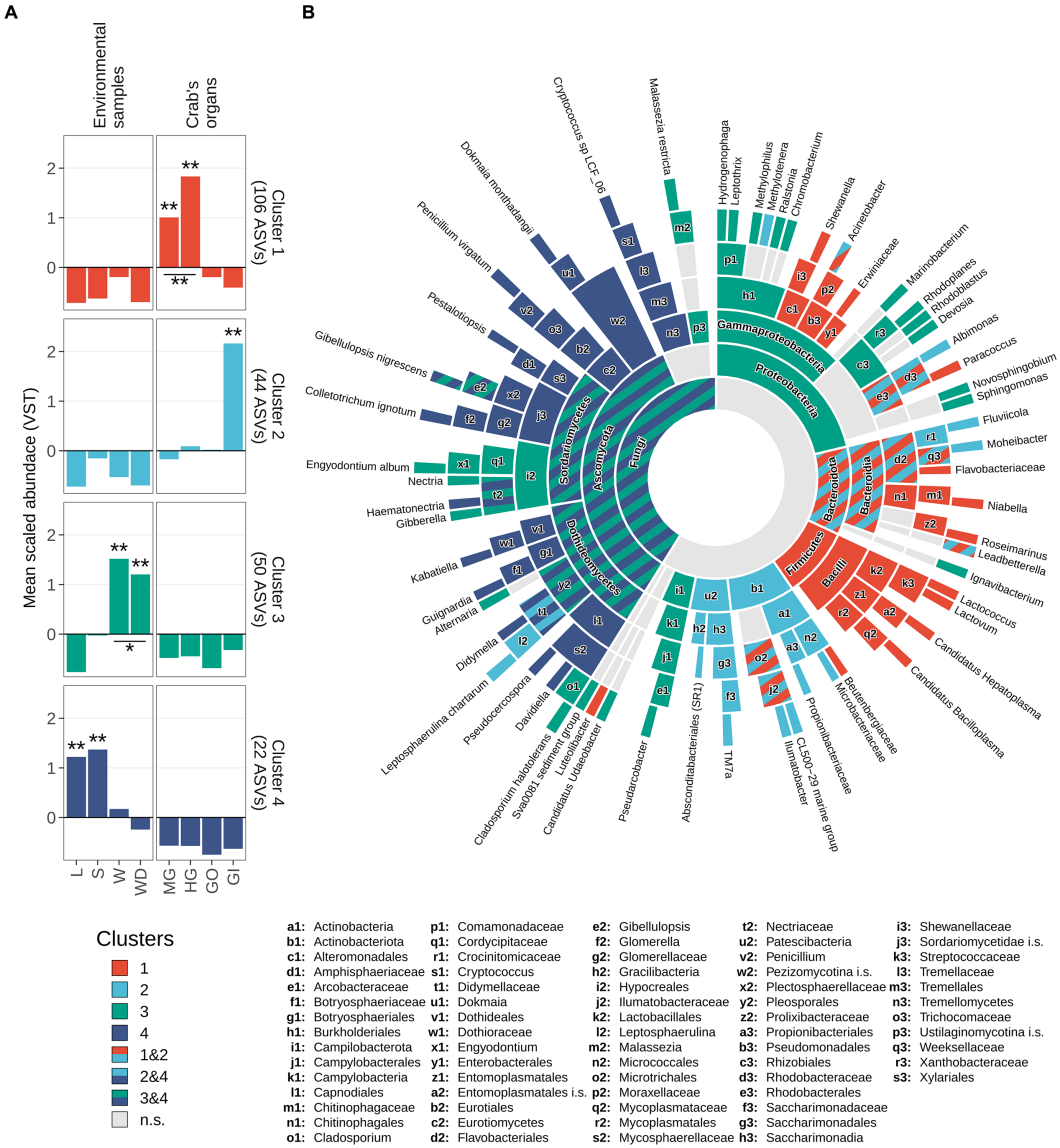
Taxonomic enrichment in clusters. **(A)** Scaled variance-stabilized counts were tested for differences along sample types within all clusters detected. A pairwise Wilcoxon test was performed, and results were reported highlighting significant differences with one asterisk (*p*-value <0.05) or two (*p*-value <0.01). A complete overview of significant differences was reported in Supplementary Figure S7. **(B)** Sunburst plot of taxa significantly enriched in clusters. Sector dimension is proportional to the number of ASVs detected whereas colours correspond to different clusters following the same colour scale in panel a. Taxa significantly enriched in more than one cluster were coloured using a striped pattern (as reported in the legend). Taxonomic ranks from the innermost circle (Kingdom level) to the leaves were reported in the legend. Leaves represent the most specific level at which a given variant has been classified, namely genus level for 16S rRNA amplicons (Bacteria and Archaea) and species level for ITS1 region amplicons (Fungi). To avoid overlaps, small sectors were named by using lowercase letters followed by a number. Complete taxonomical names of these sectors were reported in bottom part of the figure.

An exploration of the distribution of the scaled variance-stabilized counts within each sample group is shown in Figure 6A. Pairwise Wilcoxon test revealed that the ASVs present in the cluster 1 were significantly enriched in the gut, while ASVs included in cluster 2 were significantly enriched in the gills (Figure 6A). Clusters 3 and 4 were significantly related to environmental matrices, cluster 3 to the water and water debris samples, while cluster 4 with litter and soil samples (Figure 6A).

Enrichment analysis estimated using log_2_ fold-changes allowed to assign a fair number of ASVs to a specific taxon present in the previously described clusters (Figure 6B; Supplementary Table S8). Proteobacteria was the most represented phylum of the whole 16S dataset and was comprised of ASVs belonging to clusters 1, 2, and 3 (Figure 6). The results of this analysis are displayed in the sunburst plot of Figure 6B, which shows that some taxa were enriched in one or more clusters. Significantly enriched taxa were reported from the highest taxonomic rank considered (namely the domain level, the centre of the plot) to the lowest available taxonomic level (namely genus, for bacteria/archaea and species for fungi) and were hierarchically ordered (Supplementary Table S8). The intestinal cluster (i.e., cluster 1) was enriched in members of the Firmicutes phylum, more specifically members of Bacilli class including *Candidatus Hepatoplasma*, *Candidatus Bacilloplasma*, *Lactovum* and *Lactococcus* (Figure 6B). Members of the phylum Bacteroidota and some taxa related to Protebacteria and Actinobacteriota were enriched in both clusters 1 and 2. Within the phylum Bacteroidota, genera *Roseimarinus* and *Niabella* were associated with cluster 1, *Fluviicola* and *Moheibacter* were related to cluster 2, and *Leadbetterella* was shared between both clusters (Figure 6B). Other clear associations were highlighted, with cluster 3 mainly associated with Proteobacteria and Ascomycota, while cluster 2 mainly related to Actinobacteriota. The genera of these latter phyla are not exclusively associated to a single cluster and are present in multiple clusters. Cluster 4 was taxonomically assigned totally to the Fungi kingdom, specifically to the phylum Ascomycota (Supplementary Table S8).

Molecular functions inferred from 16S rRNA gene amplicons showed that bacterial structures associated with crab’s organs are not only taxonomically defined but also functionally defined (Supplementary Figure S8A). Clusters of bacterial variants were mainly enriched/depleted by a unique set of molecular functions that were absent in other clusters. The inferred genomic content of ASVs detected in crab’s gut (Cluster 1) was enriched by 359 GO terms and depleted by 21, with more than a half (218 terms, 18 depleted and 200 enriched, Supplementary Table S9) peculiar only to this cluster (Supplementary Figure S8B). Enzymes involved in the hydrolysis of complex carbohydrates—such as cellulases and xylanases—and commonly associated to the gut of model herbivorous arthropods were not enriched in the gut microbiome of the crab (Supplementary Table S10). The gills (Cluster 2) had a population of bacteria enriched by 231 GO terms and depleted by 14 terms, with roughly one third (88 terms, 12 depleted and 76 enriched) significantly found only in these organs (Supplementary Figure S8B). Functions associated to biofilm formation such as cellulose synthase (Flemming and Wingender, 2010; Augimeri et al., 2015) were enriched only in cluster 2 (GO:0016760), whereas functions associated to the nitrogen cycle—such as nitric- and nitrous-oxide reductase activity—were shared between the gills (cluster 2) and the environmental cluster 3. The latter, mainly composed of ASVs mostly found in water and water debris samples, had the largest set of GO terms (401 terms, 22 depleted and 379 enriched) with more than a half (223 terms, 19 depleted and 204 enriched) exclusively present in the inferred genome of ASVs highly abundant in cluster 3 (Supplementary Figure S8B). Only 4.87% of the total GO terms was significantly enriched in all clusters (37 terms out of 759, with no terms significantly depleted), indicating a functional role of microbial communities both in crab’s organs and in environmental samples (Supplementary Figures S8B,C and Supplementary Table S9). ASVs enriched in crab’s gut and gills (cluster 1) shared more functions (GO terms) with the environmental cluster 3 (water and water debris) than between themselves (Supplementary Figure S8B). In particular, 73 and 68 functions (with only 2 and 1 depleted terms) were shared between the gut and the gills (cluster 1 and 2), respectively, and the ASVs detected in the environmental cluster 3 (Supplementary Figure S8B and Supplementary Table S9). Considering the total number of terms significantly enriched/depleted, the gills were the organs more impacted by the environment with roughly one third (27.8%) of molecular functions shared with cluster 3. In contrast, the more populated organ, the gut, shared only 19.2% of the total number of functions with the environmental cluster 3.

## 4. Discussion

Our results show that different organs host specific microbiomes in the terrestrial brachyuran crab *C. haematocheir*, significantly different from those found in the surrounding environment. This difference is more pronounced for Bacteria than for Fungi. The microbiome profiling also revealed consistent differences among the organ-specific microbial communities. In particular, we defined stable, organ-specific microbiota in both the gut and gills of individuals belonging to different populations, suggesting a stable association between those microbial communities and *C. haematocheir*. The organ specificity is supported by the observation that such microbiota were consistently different among the different organs but their composition did not significantly change across sampling sites, while environmental microbial assemblages proved to be site-specific and different from those associated with the crabs. Another crucial result is that fungal communities are exclusive to the environmental matrices and almost absent in the *C. haematocheir* organs. This suggests a specific selection process that favours bacteria versus yeasts and fungi, possibly due to the fact that several fungi are known pathogens for arthropods and crabs (Vicente et al., 2012; Flórez et al., 2018).

The present integrated set of results put forward the idea that the microbial communities could be selected by this crab species in response to its ecological and physiological needs. The transition from a marine to a terrestrial lifestyle is marked by remarkable morphological and physiological changes evolved to tackle challenges related to respiration, feeding, reproduction and the immune system (Cannicci et al., 2020). We hypothesise that the symbiotic microbiota could help to cope with the challenges of the terrestrial lifestyle, as known for other terrestrial arthropods (Bouchon et al., 2016). For example, host-associated microbiota are known to play a crucial role in the digestive processes of terrestrial insects and isopods (Brune and Dietrich, 2015). It is not surprising that the highly diverse microbial community found in *C. haematocheir* gut is clearly distinguished from all the other internal and environmental assemblages. Moreover, the most enriched microorganisms in the gut microbiota of our species are shared with terrestrial isopods, which moved to the land roughly 300 million years ago. These shared microorganisms are supposed to play a central role in the isopods’ adaptation to a diet based on vascular plant tissues (Fraune and Zimmer, 2008). *Candidatus hepatoplasma*, for instance, was detected in *Porcellio scaber* specimens collected from Germany and western Canada (i.e, very distant geographical areas), proving a strong host-microbe association (Wang et al., 2004, 2007). More detailed studies, aimed to compare the gut microbiota of terrestrial versus marine brachyuran crabs, are still needed to shed light on the role of the associated microbiome in their ecology and environmental adaptations.

The digestive system of brachyuran crabs comprises different tracts according to their embryological origin and functional role. We intentionally focused on the midgut, where central digestion and absorption occur, and the hindgut, which plays a role in water and ion transport (Burggren and McMahon, 1988; Lindquist et al., 2009). Notwithstanding their different functions the selected intestinal tracts of *C. haematocheir* were homogeneous in terms of microbial communities, in contrast to what is known for the Chinese mitten crab, *Eriocheir sinensis* (Dong et al., 2018). Our results suggest that mid- and hindgut microbiomes play similar role, at least in this terrestrial crab.

The potential for a vertical transmission vs. an acquisition from the environment of this specialized gut microbiota deserve a deeper discussion and further experiments. First, land and terrestrial crabs are neither social or gregarious and only few of them show some basic degrees of parental care (Anger, 1995; Hornung, 2011). Second, *C. haematocheir*, as well as the most land-adapted family of brachyuran crabs, the Gecarcinidae, still retains an indirect development strategy and releases planktonic larvae in coastal waters. Consequently, *C. haematoicher*, as well as the land crabs with planktonic larvae in general, may have developed intimate gut-specific symbiotic associations only through pseudo-vertical transmission (Wilkinson, 1997), i.e., by acquiring and selecting their gut microbiota directly from food or, possibly, through consumption of adults’ faeces (coprophagy).

The gonads host a low microbial diversity with respect to the other organs and environmental matrices we analysed, as hypothesised for this internal organ in no direct contact with the environment and not morphologically connected to the gut and the gills. Their microbial community, moreover, is shared with both the other organs and the environment, determining the absence of a specific taxonomic and functional cluster associated with them. Microorganisms are known to be associated with the reproductive system of arthropods, in both insects (Gibson and Hunter, 2010) and crustaceans (Perlmutter and Bordenstein, 2020). Few studies, however, have specifically characterised these gonad-associated microbiota and most of them were focused on specific pathogens of sexually transmitted infections or reproductive parasites, with the most notable example being *Wolbachia* (Chevalier et al., 2012). When present, the vertical transmission of specific microbiota during oogenesis or at birth is known to determine a colonization of the gonads that ultimately affects the microbial composition of the offspring (Perlmutter and Bordenstein, 2020). In this view, the absence of an exclusive microbiota in *C. haematoicher* gonads can also be explained by the lack of vertical transmission through the parental-offspring pathways, due to the presence of planktonic stages.

Although protected in the gill chambers, *C. haematocheir* gills are in direct contact with the environment, but they do show a unique resident microbiota different from that detected in the environmental matrices. These gill-associated microbiota are only enriched in prokaryotes, unlike the soil/litter and the water communities, which are composed almost entirely by fungi and a mix of Proteobacteria and fungi, respectively. The gill-associated microbial cluster is characterised by a strong uniformity in terms of both taxa and genes. Actinobacteria associated to the gills include Microbacteriacea and *Illumatobacter*, which are known to play an important role by producing bioactive compounds crucial in the defence against pathogens in marine organisms (Valliappan et al., 2014). Some of the bacteria detected on the gills of *C. haematocheir*, such as *Ilumatobacter* and *Albimonas*, were also found in the gills of *E. sinensis* (Dong et al., 2018). In contrast to the gut, genes associated with cellulose synthase activity were found in the microbiota associated with the gills (GO:0016760). This activity is typical of biofilm-forming bacteria that use cellulose both as a physical barrier against harmful molecules, and as a molecular glue to help their interaction with the host (Flemming and Wingender, 2010). Since the gills of *C. haematocheir* are exposed to external perturbations, the presence of biofilm-forming functions may help to boost the resilience of bacteria stabilizing host-microbiome interactions. Genes related to the reduction of nitric compounds (namely: nitric oxide reductase activity, GO:0016966, and nitrous-oxide reductase activity, GO:0050304) were enriched in both the gills and environmental water and water debris samples. Besides their role in the anaerobic metabolism of nitrogen, these functions are involved in pathogenesis and antibiotic resistance in bacteria (Vázquez-Torres and Bäumler, 2016) and may help tissue colonization. We speculate that this mechanism may be used by bacteria inhabiting the gills of *C. haematocheir* as a possible molecular defence against external pathogens. Further experiments and comparative investigations are indeed needed to support this hypothesis.

Our results show that fungi are strongly depleted in all crab’s organs and that gills should be considered an efficient selective filter between the environment and the host. In our opinion, the substantial absence of fungal associations can be put in relation to the existence of defences from potential pathogens. Pathogenic fungi have been reported in intertidal and terrestrial crabs, such as the case of the swamp ghost crab, *U. cordatus*, infected by species of black yeast, *Exophiala cancerae*, and *Fonsecaea brasiliensis*, which cause a condition called “lethargic crab disease” (Boeger et al., 2005; Vicente et al., 2012). The role of prokaryotic associations in the defence against pathogenic fungi can thus be of critical importance for terrestrial crabs, since the immune molecules of crustaceans are less efficient than those of insects against fungal infections (Ng and Kurtz, 2020).

In conclusion, we found clear differentiations among the microbiota associated with different crab’s organs and the ones found in the environmental matrices, suggesting a selective process towards specific microbial taxa operated by the crabs in the different districts of its body. The differences found among the organ-related and environmental clusters were not merely taxonomical since the different clusters also harbour different metabolic profiles. These results indicate the presence of metabolic complementation mechanisms between the crab and its microbiota and suggest the possible evolution of crab-microbe interaction associated with the ecology and physiology of the host, as theorized by the holobiont theory of evolution. Further phylosymbiosis studies across crab species belonging to different families and inhabiting marine and terrestrial habitats are needed to confirm the role of microbial communities in driving host adaptations to different environments.

## Data availability statement

The datasets presented in this study can be found in online repositories. The names of the repository/repositories and accession number(s) can be found in the article/Supplementary material.

## Ethics statement

This research has been performed under the respect of all local, national and international regulations and conventions, and normal scientific ethical practices.

## Author contributions

GB performed bioinformatics analyses and wrote the manuscript. SF supervised lab work and wrote the manuscript. NM performed 16S rRNA gene laboratory analyses and wrote the manuscript. CC and KN carried out the samplings and performed DNA extractions. DC and AM helped in conceiving the original idea. SC conceived the original idea, collected the samples and wrote the manuscript. SC and DC provided funding for the experiments. All authors contributed to the article and approved the submitted version.

## Funding

This work was supported by the project “The sky’s the limit: the irresistible ascent to land and trees by crabs,” sponsored by TUYF Charitable Trust funds, Hong Kong, (HKU no 260008686.088562.26000.400.01) and by the Eighth Government Matching Grant, Hong Kong Government (HKU no 207080320.088562.26020.430.01) to SC and by the RAE Improvement Fund from the Faculty of Science, HKU (HKU no 000250449.088562.26000.100.01) to SC and DC. DC was also supported by the Tuscany Region (Italy) with the project “PO FEAMP 2014 - 2020 - Misura 1.39 SSL FLAG Costa degli Etruschi” (grant no 2/SSL/16/TO-1/ICR/21/TO).

## Conflict of interest

The authors declare that the research was conducted in the absence of any commercial or financial relationships that could be construed as a potential conflict of interest.

## Publisher’s note

All claims expressed in this article are solely those of the authors and do not necessarily represent those of their affiliated organizations, or those of the publisher, the editors and the reviewers. Any product that may be evaluated in this article, or claim that may be made by its manufacturer, is not guaranteed or endorsed by the publisher.
