# Supplementary material for: Conserved organ-specific microbial assemblages in different populations of a terrestrial crab
Source: Front Microbiol. 2023 Jun 12;14:1113617. doi: 10.3389/fmicb.2023.1113617 (PMC10291174; doi:10.3389/fmicb.2023.1113617)
Supplement: Supplementary file 2 [file Table_1.pdf]

Table S1: Number of samples sequenced for 16S rRNA gene and ITS1 region. Column one reported the sample id of each condition whereas column two reported the biological matrix sampled (MG, midgut; HG, hindgut; GO, gonads; GI, gills; L, litter; S, soil; W, water; WD, water debris). The sampling site was reported in column three together with the crab's number (reported in column four). Samples with the same crab's number within the same sample site are sample coming from the same specimen (experimental unit)–this information was not reported for environmental samples which, in this study, did not share a common experimental unit. The number of samples sequenced was reported in column five and six for 16S rRNA and ITS1 amplicons, respectively. The last column of the table identifies the technical replicates used for ITS1 validation.

sample.id	env	site	crab	16S	its	technical replicates (ITS only)
MG_SH_1	MG	SH	1	2	1	
MG_SH_2	MG	SH	2	1	2	
MG_SH_3	MG	SH	3	1	2	yes
MG_SH_4	MG	SH	4	1	2	
MG_SH_5	MG	SH	5	1	1	
MG_SH_6	MG	SH	6	1	2	
MG_SH_7	MG	SH	7	1	2	
MG_SH_8	MG	SH	8	1	1	
MG_SH_9	MG	SH	9	1	2	
MG_SH_10	MG	SH	10	1	2	
MG_SH_11	MG	SH	11	1	3	
MG_SK_1	MG	SK	1	1	2	
MG_SK_2	MG	SK	2	1	2	
MG_SK_3	MG	SK	3	1	2	
MG_SK_4	MG	SK	4	1	1	
MG_SK_5	MG	SK	5	1	1	
MG_SK_6	MG	SK	6	1	3	
MG_SK_7	MG	SK	7	1	1	
MG_SK_8	MG	SK	8	1	1	
MG_SK_9	MG	SK	9	2	2	
MG_SK_10	MG	SK	10	1	2	
MG_SK_11	MG	SK	11	1	2	yes
MG_TKP_1	MG	TKP	1	2	1	
MG_TKP_2	MG	TKP	2	1	2	
MG_TKP_3	MG	TKP	3	1	2	
MG_TKP_4	MG	TKP	4	1	3	
MG_TKP_5	MG	TKP	5	1	2	
MG_TKP_6	MG	TKP	6	1	1	
MG_TKP_7	MG	TKP	7	1	1	
MG_TKP_8	MG	TKP	8	1	2	
MG_TKP_9	MG	TKP	9	1	1	
MG_TKP_10	MG	TKP	10	1	2	
MG_TKP_11	MG	TKP	11	1	1	
HG_SH_1	HG	SH	1	2	2	yes
HG_SH_2	HG	SH	2	1	1	
HG_SH_3	HG	SH	3	1	2	
HG_SH_4	HG	SH	4	1	2	
HG_SH_5	HG	SH	5	2	1	
HG_SH_6	HG	SH	6	1	1	
HG_SH_7	HG	SH	7	1	1	
HG_SH_8	HG	SH	8	1	1	
HG_SH_9	HG	SH	9	1	1	
HG_SH_10	HG	SH	10	1	1	
HG_SH_11	HG	SH	11	1	1	
HG_SK_1	HG	SK	1	1	2	
HG_SK_2	HG	SK	2	1	1	
HG_SK_3	HG	SK	3	1	1	
HG_SK_4	HG	SK	4	1	1	
HG_SK_5	HG	SK	5	1	2	

HG_SK_6	HG	SK	6	1	2	yes
HG_SK_7	HG	SK	7	1	1	
HG_SK_8	HG	SK	8	1	1	
HG_SK_9	HG	SK	9	1	2	
HG_SK_10	HG	SK	10	1	1	
HG_SK_11	HG	SK	11	1	1	
HG_TKP_1	HG	TKP	1	1	2	
HG_TKP_2	HG	TKP	2	1	1	
HG_TKP_3	HG	TKP	3	2	1	
HG_TKP_4	HG	TKP	4	1	1	
HG_TKP_5	HG	TKP	5	1	2	
HG_TKP_6	HG	TKP	6	1	2	
HG_TKP_7	HG	TKP	7	1	1	
HG_TKP_8	HG	TKP	8	1	2	
HG_TKP_9	HG	TKP	9	1	1	
HG_TKP_10	HG	TKP	10	1	2	
HG_TKP_11	HG	TKP	11	1	1	
GO_SH_1	GO	SH	1	1	1	
GO_SH_2	GO	SH	2	1	1	
GO_SH_3	GO	SH	3	1	2	
GO_SH_4	GO	SH	4	1	2	
GO_SH_5	GO	SH	5	1	2	
GO_SH_6	GO	SH	6	1	1	
GO_SH_7	GO	SH	7	1	1	
GO_SH_8	GO	SH	8	1	2	yes
GO_SH_9	GO	SH	9	1	1	
GO_SH_10	GO	SH	10	1	1	
GO_SH_11	GO	SH	11	1	1	
GO_SK_1	GO	SK	1	1	1	
GO_SK_2	GO	SK	2	1	1	
GO_SK_3	GO	SK	3	1	1	
GO_SK_4	GO	SK	4	1	1	
GO_SK_5	GO	SK	5	1	1	
GO_SK_6	GO	SK	6	1	1	
GO_SK_7	GO	SK	7	1	1	
GO_SK_8	GO	SK	8	1	1	
GO_SK_9	GO	SK	9	1	1	
GO_SK_10	GO	SK	10	1	1	
GO_SK_11	GO	SK	11	1	1	
GO_TKP_1	GO	TKP	1	1	2	
GO_TKP_2	GO	TKP	2	1	1	
GO_TKP_3	GO	TKP	3	1	1	
GO_TKP_4	GO	TKP	4	1	1	
GO_TKP_5	GO	TKP	5	1	1	
GO_TKP_6	GO	TKP	6	1	2	
GO_TKP_7	GO	TKP	7	1	2	yes
GO_TKP_8	GO	TKP	8	1	1	
GO_TKP_9	GO	TKP	9	1	1	
GO_TKP_10	GO	TKP	10	1	1	
GO_TKP_11	GO	TKP	11	1	2	

GI_SH_1	GI	SH	1	1	1	
GI_SH_2	GI	SH	2	2	1	
GI_SH_3	GI	SH	3	1	1	
GI_SH_4	GI	SH	4	2	2	yes
GI_SH_5	GI	SH	5	1	1	
GI_SH_6	GI	SH	6	1	1	
GI_SH_7	GI	SH	7	1	1	
GI_SH_8	GI	SH	8	1	1	
GI_SH_9	GI	SH	9	1	2	yes
GI_SH_10	GI	SH	10	1	1	
GI_SH_11	GI	SH	11	1	1	
GI_SK_1	GI	SK	1	2	1	
GI_SK_2	GI	SK	2	2	1	
GI_SK_3	GI	SK	3	2	1	
GI_SK_4	GI	SK	4	2	1	
GI_SK_5	GI	SK	5	2	1	
GI_SK_6	GI	SK	6	2	1	
GI_SK_7	GI	SK	7	2	1	
GI_SK_8	GI	SK	8	2	1	
GI_SK_9	GI	SK	9	2	1	
GI_SK_10	GI	SK	10	2	1	
GI_SK_11	GI	SK	11	1	1	
GI_TKP_1	GI	TKP	1	2	1	
GI_TKP_2	GI	TKP	2	1	1	
GI_TKP_3	GI	TKP	3	2	1	
GI_TKP_4	GI	TKP	4	2	1	
GI_TKP_5	GI	TKP	5	2	1	
GI_TKP_6	GI	TKP	6	1	1	
GI_TKP_7	GI	TKP	7	2	1	
GI_TKP_8	GI	TKP	8	2	1	
GI_TKP_9	GI	TKP	9	1	1	
GI_TKP_10	GI	TKP	10	1	1	
GI_TKP_11	GI	TKP	11	1	1	
L_SH_1	L	SH		1	1	
L_SH_2	L	SH		1	1	
L_SH_3	L	SH		1	1	
L_SH_4	L	SH		1	2	
L_SH_5	L	SH		1	1	
L_SK_1	L	SK		2	1	
L_SK_2	L	SK		2	1	
L_SK_3	L	SK		2	1	
L_SK_4	L	SK		1	1	
L_SK_5	L	SK		1	1	
L_TKP_1	L	TKP		2	1	
L_TKP_2	L	TKP		2	1	
L_TKP_3	L	TKP		1	1	
L_TKP_4	L	TKP		1	1	
L_TKP_5	L	TKP		1	1	
S_SH_1	S	SH		2	1	
S_SH_2	S	SH		2	1	

S_SH_3	S	SH	2	1
S_SH_4	S	SH	1	1
S_SH_5	S	SH	2	1
S_SK_1	S	SK	2	1
S_SK_2	S	SK	2	1
S_SK_3	S	SK	2	1
S_SK_4	S	SK	2	1
S_SK_5	S	SK	2	1
S_TKP_1	S	TKP	1	1
S_TKP_2	S	TKP	2	1
S_TKP_3	S	TKP	2	1
S_TKP_4	S	TKP	2	1
S_TKP_5	S	TKP	2	1
W_SH_1	W	SH	2	1
W_SH_2	W	SH	2	1
W_SH_3	W	SH	2	1
W_SH_4	W	SH	2	1
W_SH_5	W	SH	2	1
W_SK_1	W	SK	2	1
W_SK_2	W	SK	2	1
W_SK_3	W	SK	2	1
W_SK_4	W	SK	2	1
W_SK_5	W	SK	2	1
W_TKP_1	W	TKP	2	1
W_TKP_2	W	TKP	2	2
W_TKP_3	W	TKP	2	2
W_TKP_4	W	TKP	2	1
W_TKP_5	W	TKP	2	1
WD_SH_1	WD	SH	1	2
WD_SH_2	WD	SH	1	2
WD_SH_3	WD	SH	1	2
WD_SH_4	WD	SH	1	2
WD_SH_5	WD	SH	1	2
WD_SK_1	WD	SK	1	2
WD_SK_2	WD	SK	1	2
WD_SK_3	WD	SK	1	2
WD_SK_4	WD	SK	1	2
WD_SK_5	WD	SK	1	2
WD_TKP_1	WD	TKP	1	1
WD_TKP_2	WD	TKP	1	1
WD_TKP_3	WD	TKP	1	1
WD_TKP_4	WD	TKP	1	1
WD_TKP_5	WD	TKP	1	1

Table S2: Accuracy and correlation between replicates. Sample IDs were reported in the first column whereas crab's organs were reported in the second one. The number of fungal ASVs detected in all organs (GI, gills; GO, gonads; HG and MG, hind- and midgut) was reported in the "N" column. Spearman's rank correlation coefficient was reported in the ρ column whereas accuracy was reported in the last column of the table. Other columns report values used to calculate the accuracy as explained in the "Fungal amplicon sequence variant evaluation" section.

Sample ID	Organ	N	ρ	TP	TN	FP + FN	Accuracy
GI_SH_4	GI	82	0.8	10	66	6	0.93
GI_SH_9	GI	82	0.67	5	71	6	0.93
GO_SH_8	GO	82	1	2	60	20	0.76
GO_TKP_7	GO	82	0.97	11	70	1	0.99
HG_SH_1	HG	82	1	5	71	6	0.93
HG_SK_6	HG	82	0.98	12	59	11	0.87
MG_SH_3	MG	82	0.87	6	70	6	0.93
MG_SK_11	MG	82	1	4	71	7	0.91

Table S3: Microbial diversity of samples included in the study. Microbial diversity was calculated considering counts from both 16S rRNA and ITS1 amplicon sequencing. Sample IDs were reported in the first column (called ID) whereas sample types and sampling sites were reported in the second and the third ones, respectively. The number of ASVs detected in each sample (also known as the "Richness") was reported together with the number of singletons and doubletons detected (defined as the number of ASVs with a count of one and two across all samples, respectively). The last two columns of the table reported the inverse Simpson index and the Good's coverage for all samples (for additional information on how these indices were computed see "Microbial diversity within and between sample types" section).

ID	Sample type	Site	Number of ASVs	Number of singletons	Number of doubletons	Inverse Simpson index	Good's coverage estimator
MG_SH_1	MG	SH	355	0	5	4.818706231	100
MG_SH_10	MG	SH	409	4	7	27.03786021	99.9856511
MG_SH_11	MG	SH	283	2	2	11.66302146	99.99524545
MG_SH_2	MG	SH	379	5	6	22.03412969	99.97415958
MG_SH_3	MG	SH	601	3	4	22.3623928	99.99410868
MG_SH_4	MG	SH	287	0	0	10.48148819	100
MG_SH_5	MG	SH	321	0	2	8.412803658	100
MG_SH_6	MG	SH	349	1	0	12.27959394	99.99906696
MG_SH_7	MG	SH	252	2	0	13.75124693	99.99693438
MG_SH_8	MG	SH	426	0	0	5.33375842	100
MG_SH_9	MG	SH	429	3	4	17.72186224	99.98739614
MG_SK_1	MG	SK	186	0	0	21.1856249	100
MG_SK_10	MG	SK	363	5	10	30.44687129	99.96150112
MG_SK_11	MG	SK	470	2	4	26.5393647	99.99489136
MG_SK_2	MG	SK	275	1	0	5.671059412	99.99909447
MG_SK_3	MG	SK	542	2	5	37.40671634	99.99453337
MG_SK_4	MG	SK	394	0	2	18.0872314	100
MG_SK_5	MG	SK	411	0	1	13.90561351	100
MG_SK_6	MG	SK	658	5	7	35.39470196	99.98775102
MG_SK_7	MG	SK	559	0	0	11.24145972	100
MG_SK_8	MG	SK	362	0	6	18.5515724	100
MG_SK_9	MG	SK	730	0	1	20.13511029	100
MG_TKP_1	MG	TKP	854	1	9	14.1473312	99.99852363
MG_TKP_10	MG	TKP	782	1	11	19.68293752	99.99837438
MG_TKP_11	MG	TKP	488	2	8	23.20893597	99.99816511
MG_TKP_2	MG	TKP	592	1	6	19.83853392	99.99765465
MG_TKP_3	MG	TKP	430	5	7	13.48972354	99.98552119
MG_TKP_4	MG	TKP	384	0	1	15.5623347	100
MG_TKP_5	MG	TKP	833	3	1	30.64169241	99.9951857
MG_TKP_6	MG	TKP	485	3	7	15.81227072	99.99198904
MG_TKP_7	MG	TKP	393	0	0	19.85198873	100
MG_TKP_8	MG	TKP	238	1	1	3.918777089	99.99722703
MG_TKP_9	MG	TKP	194	0	0	8.494027457	100
HG_SH_1	HG	SH	254	1	0	4.564213113	99.99955309
HG_SH_10	HG	SH	460	2	5	16.23235827	99.99711357
HG_SH_11	HG	SH	566	7	7	26.71816502	99.98352915
HG_SH_2	HG	SH	464	5	16	12.97688791	99.99500114
HG_SH_3	HG	SH	692	3	6	45.17913289	99.99498326
HG_SH_4	HG	SH	330	0	0	10.13476703	100
HG_SH_5	HG	SH	618	0	0	265.8918303	100
HG_SH_6	HG	SH	295	0	2	15.28308989	100
HG_SH_7	HG	SH	371	1	2	7.779032915	99.99864594
HG_SH_8	HG	SH	268	2	8	8.302922738	99.9981124
HG_SH_9	HG	SH	623	7	5	13.16694071	99.98497681
HG_SK_1	HG	SK	649	2	5	26.95881626	99.99739245
HG_SK_10	HG	SK	570	4	19	15.22326085	99.99206771
HG_SK_11	HG	SK	305	1	0	12.95790294	99.99808561
HG_SK_2	HG	SK	524	0	0	16.04426432	100
HG_SK_3	HG	SK	731	6	16	38.1089613	99.99083768
HG_SK_4	HG	SK	568	2	0	27.27063351	99.99739827
HG_SK_5	HG	SK	540	3	5	30.7080746	99.99088424
HG_SK_6	HG	SK	503	0	5	17.70261096	100
HG_SK_7	HG	SK	476	1	9	6.970532973	99.99903272
HG_SK_8	HG	SK	235	0	0	10.43029724	100
HG_SK_9	HG	SK	372	0	1	11.1799848	100
HG_TKP_1	HG	TKP	293	0	0	9.567358711	100
HG_TKP_10	HG	TKP	813	2	2	28.87181711	99.99699725
HG_TKP_11	HG	TKP	466	1	0	11.72743476	99.99934608
HG_TKP_2	HG	TKP	626	2	8	18.55507036	99.9964843
HG_TKP_3	HG	TKP	294	0	0	7.344452561	100
HG_TKP_4	HG	TKP	532	2	0	13.44776479	99.99791029
HG_TKP_5	HG	TKP	613	0	5	13.90994856	100
HG_TKP_6	HG	TKP	837	2	9	37.32583202	99.99746098
HG_TKP_7	HG	TKP	561	1	9	36.28835372	99.99877768
HG_TKP_8	HG	TKP	902	5	12	39.82095153	99.99357096

HG_TKP_9	HG	TKP	305	1	8	9.220143786	99.9987925
GO_SH_1	GO	SH	248	0	0	6.786578525	100
GO_SH_10	GO	SH	8	0	0	4.87960396	100
GO_SH_11	GO	SH	65	8	3	1.642661962	99.94636802
GO_SH_2	GO	SH	15	0	0	6.991360158	100
GO_SH_3	GO	SH	58	0	3	6.897210981	100
GO_SH_4	GO	SH	80	0	0	10.06046153	100
GO_SH_5	GO	SH	38	0	0	7.602746791	100
GO_SH_6	GO	SH	141	7	3	18.86112154	99.93577061
GO_SH_7	GO	SH	86	0	0	2.232154107	100
GO_SH_8	GO	SH	87	8	5	9.097225582	99.90433536
GO_SH_9	GO	SH	48	0	0	1.478507632	100
GO_SK_1	GO	SK	133	1	7	1.738520331	99.99510553
GO_SK_10	GO	SK	149	0	10	6.87797961	100
GO_SK_11	GO	SK	155	0	3	4.73212502	100
GO_SK_2	GO	SK	139	0	1	7.136700186	100
GO_SK_3	GO	SK	50	0	0	1.418786235	100
GO_SK_4	GO	SK	156	3	1	2.003806205	99.98317875
GO_SK_5	GO	SK	11	0	0	4.888260255	100
GO_SK_6	GO	SK	116	1	1	2.790798986	99.99403291
GO_SK_7	GO	SK	87	7	5	9.706186979	99.89617167
GO_SK_8	GO	SK	213	8	11	27.24675722	99.91806819
GO_SK_9	GO	SK	118	0	0	1.983700928	100
GO_TKP_1	GO	TKP	148	0	0	3.327560388	100
GO_TKP_10	GO	TKP	304	1	2	7.863097569	99.99650031
GO_TKP_11	GO	TKP	15	0	0	6.285752779	100
GO_TKP_2	GO	TKP	173	0	0	3.647923199	100
GO_TKP_3	GO	TKP	116	1	6	1.356976999	99.99764343
GO_TKP_4	GO	TKP	115	0	1	2.526557592	100
GO_TKP_5	GO	TKP	135	0	0	1.585009434	100
GO_TKP_6	GO	TKP	157	0	0	10.99292462	100
GO_TKP_7	GO	TKP	188	0	0	2.145117077	100
GO_TKP_8	GO	TKP	330	0	10	4.201787869	100
GO_TKP_9	GO	TKP	82	0	0	1.170411259	100
GI_SH_1	GI	SH	318	12	15	44.45981897	99.95295381
GI_SH_10	GI	SH	5517	28	152	195.4047557	99.9907388
GI_SH_11	GI	SH	1599	4	29	206.9616715	99.99232945
GI_SH_2	GI	SH	70	4	5	6.711664078	99.98342664
GI_SH_3	GI	SH	1033	4	0	346.4219903	99.98545936
GI_SH_4	GI	SH	501	1	0	12.2802025	99.99920005
GI_SH_5	GI	SH	2520	8	39	537.8104584	99.98944162
GI_SH_6	GI	SH	2465	11	23	623.1416422	99.98455607
GI_SH_7	GI	SH	2709	13	44	546.6640852	99.98449673
GI_SH_8	GI	SH	2236	9	22	265.4986009	99.98800418
GI_SH_9	GI	SH	6004	26	43	323.564784	99.98895319
GI_SK_1	GI	SK	412	2	0	22.27594957	99.99649021
GI_SK_10	GI	SK	106	0	0	11.88147467	100
GI_SK_11	GI	SK	8110	30	189	316.8538822	99.99170301
GI_SK_2	GI	SK	100	0	6	4.87147279	100
GI_SK_3	GI	SK	196	0	0	13.28176627	100
GI_SK_4	GI	SK	339	1	15	13.59608544	99.99904286
GI_SK_5	GI	SK	106	0	0	7.602865069	100
GI_SK_6	GI	SK	141	0	0	11.77085152	100
GI_SK_7	GI	SK	116	0	0	9.129463368	100
GI_SK_8	GI	SK	690	2	0	11.75406376	99.99716438
GI_SK_9	GI	SK	76	5	4	7.406711538	99.98032117
GI_TKP_1	GI	TKP	242	0	0	8.470114606	100
GI_TKP_10	GI	TKP	157	0	1	16.21312176	100
GI_TKP_11	GI	TKP	173	0	0	13.01292865	100
GI_TKP_2	GI	TKP	190	0	0	11.51358029	100
GI_TKP_3	GI	TKP	203	0	9	15.60133616	100
GI_TKP_4	GI	TKP	296	0	0	14.22255956	100
GI_TKP_5	GI	TKP	266	0	16	10.40935667	100
GI_TKP_6	GI	TKP	166	0	0	14.78461487	100
GI_TKP_7	GI	TKP	165	0	0	8.509072812	100
GI_TKP_8	GI	TKP	517	2	5	21.44907895	99.99721016
GI_TKP_9	GI	TKP	198	1	0	8.134201012	99.99848557

L_SH_1	L	SH	354	28	20	28.13404566	99.9384296
L_SH_2	L	SH	315	15	26	45.45411645	99.97184144
L_SH_3	L	SH	96	1	8	6.276289819	99.99339061
L_SH_4	L	SH	140	17	6	10.21267203	99.88984315
L_SH_5	L	SH	140	14	13	15.78586133	99.94473746
L_SK_1	L	SK	1018	20	34	70.91582835	99.97752192
L_SK_2	L	SK	881	17	40	61.73500358	99.96933552
L_SK_3	L	SK	868	14	37	61.20524562	99.97282555
L_SK_4	L	SK	310	19	28	32.12721063	99.9525447
L_SK_5	L	SK	286	17	18	26.72637423	99.93327768
L_TKP_1	L	TKP	685	1	22	6.488833714	99.998274
L_TKP_2	L	TKP	502	16	27	50.23624715	99.96808149
L_TKP_3	L	TKP	106	10	10	21.10578315	99.98262904
L_TKP_4	L	TKP	58	4	5	8.130822914	99.9600368
L_TKP_5	L	TKP	182	3	10	5.890670047	99.9856369
S_SH_1	S	SH	511	2	6	36.63341386	99.99281863
S_SH_2	S	SH	1374	4	24	63.78773963	99.99127508
S_SH_3	S	SH	1865	4	55	240.0080686	99.99351054
S_SH_4	S	SH	131	2	3	19.81877493	99.98710576
S_SH_5	S	SH	988	1	18	57.9784605	99.99822825
S_SK_1	S	SK	950	5	16	34.71418707	99.98798354
S_SK_2	S	SK	2002	12	35	18.94529601	99.98731726
S_SK_3	S	SK	1743	7	35	308.760236	99.98849956
S_SK_4	S	SK	1134	4	12	219.9309371	99.98974744
S_SK_5	S	SK	1703	7	40	202.1880481	99.98821482
S_TKP_1	S	TKP	401	0	8	16.86696092	100
S_TKP_2	S	TKP	456	0	3	11.24043437	100
S_TKP_3	S	TKP	1259	3	25	28.43293575	99.99566746
S_TKP_4	S	TKP	983	0	2	9.856846746	100
S_TKP_5	S	TKP	1202	2	21	12.03280726	99.99717502
W_SH_1	W	SH	799	0	7	127.2805405	100
W_SH_2	W	SH	1205	0	7	151.0226808	100
W_SH_3	W	SH	1210	6	0	158.1309967	99.98779476
W_SH_4	W	SH	373	0	29	24.56079362	100
W_SH_5	W	SH	680	0	30	75.90200743	100
W_SK_1	W	SK	761	1	16	19.43223473	99.99784419
W_SK_2	W	SK	592	0	0	22.84512791	100
W_SK_3	W	SK	195	0	0	3.204749823	100
W_SK_4	W	SK	3567	23	267	244.8521176	99.98340367
W_SK_5	W	SK	484	0	0	25.76002911	100
W_TKP_1	W	TKP	741	0	3	16.6556518	100
W_TKP_2	W	TKP	1498	2	0	98.99462578	99.99725314
W_TKP_3	W	TKP	3390	8	208	45.38607668	99.99764277
W_TKP_4	W	TKP	573	0	1	63.13150886	100
W_TKP_5	W	TKP	1755	33	64	45.41702799	99.99296514
WD_SH_1	WD	SH	470	0	4	21.81797564	100
WD_SH_2	WD	SH	524	3	6	26.24533418	99.99210661
WD_SH_3	WD	SH	775	3	11	22.17023698	99.99359646
WD_SH_4	WD	SH	405	1	5	16.8986672	99.99730084
WD_SH_5	WD	SH	337	0	3	7.774626473	100
WD_SK_1	WD	SK	1739	5	130	79.2014175	99.99308103
WD_SK_2	WD	SK	1948	8	103	290.4342669	99.98775157
WD_SK_3	WD	SK	1157	0	5	31.33554017	100
WD_SK_4	WD	SK	3353	16	212	347.3035972	99.98717574
WD_SK_5	WD	SK	664	0	5	17.20298842	100
WD_TKP_1	WD	TKP	2501	9	164	370.3107527	99.99000447
WD_TKP_2	WD	TKP	3123	14	236	385.8381324	99.98890488
WD_TKP_3	WD	TKP	3377	30	184	226.7327169	99.97729126
WD_TKP_4	WD	TKP	1411	5	0	74.08000193	99.99072005
WD_TKP_5	WD	TKP	1412	7	0	100.2788867	99.98763874

Table S4: Wilcoxon test on alpha diversity. Results of pairwise Wilcoxon test on crab's organs and environmental samples. P-values were corrected by using the "Benjamini-Hochberg" method also known as "False Discovery Rate" (FDR). Target, the target used for metabarcoding analyses; Type, the type of samples (if crab's organs and environmental samples or sampling sites); Group 1, the first group tested; Group 2, the second group tested; Q-value, the adjusted P-value.

Target	Type	Group 1	Group 2	Q-value	Sign
16S	Sample types	WD	S	0.598	
		GI	S	0.008	**
		W	S	0.033	*
		L	S	0.004	**
		HG	S	0.000	**
		MG	S	0.000	**
		GO	S	0.000	**
		GI	WD	0.110	
		W	WD	0.152	
		L	WD	0.072	
		HG	WD	0.012	*
		MG	WD	0.012	*
		GO	WD	0.000	**
		W	GI	0.110	
		L	GI	0.464	
		HG	GI	0.776	
		MG	GI	0.776	
		GO	GI	0.000	**
		L	W	0.135	
		HG	W	0.001	**
		MG	W	0.000	**
		GO	W	0.000	**
		HG	L	0.100	
		MG	L	0.025	*
		GO	L	0.000	**
		MG	HG	0.788	
		GO	HG	0.000	**
		GO	MG	0.000	**
	Sites	SK	SH	0.930	
		TKP	SH	0.930	
		TKP	SK	0.930	
ITS	Sample types	W	S	0.789	
		WD	S	0.476	
		L	S	0.124	
		MG	S	0.002	**
		HG	S	0.002	**

	GI	S	0.002	**
	GO	S	0.002	**
	WD	W	0.482	
	L	W	0.138	
	MG	W	0.006	**
	HG	W	0.006	**
	GI	W	0.003	**
	GO	W	0.003	**
	L	WD	0.293	
	MG	WD	0.002	**
	HG	WD	0.002	**
	GI	WD	0.002	**
	GO	WD	0.001	**
	MG	L	0.124	
	HG	L	0.124	
	GI	L	0.062	
	GO	L	0.053	
	HG	MG	0.974	
	GI	MG	0.739	
	GO	MG	0.713	
	GI	HG	0.789	
	GO	HG	0.696	
	GO	GI	0.907	
Sites	SK	SH	0.539	
	TKP	SH	0.030	*
	TKP	SK	0.049	*

Table S5: Variance explained by crab, sample type, and sampling site with permutational multivariate analysis of variance (PERMANOVA). The variance explained by each factor (also called R squared) was reported separately for 16S and ITS1 amplicons. The type columns reports if the PERMANOVA was computed on crab's samples (midgut, hindgut, gills, and gonads) or on environmental samples (litter, soil, water, and water debris). The crab was included in the model to account for variability across different animals collected. Asterisks were used to report significance based on p-values (*, p-value < 0.05; ** p-value < 0.01). The proportion of variance unexplained by the factor considered was reported in the Residual column.

Amplicon	Type	Crab	Sample Type	Site	Residual
16S	Crab's organs	0.203**	0.260**	0.043**	0.494
	Environmental samples	-	0.160**	0.088**	0.752
ITS	Crab's organs	0.226	0.053**	0.055**	0.666
	Environmental samples	-	0.174**	0.132**	0.694

Table S6: Analysis of variance (ANOVA) on euclidean distances around centroids. The homogeneity of dispersion across groups was tested using ANOVA on euclidean distances from group centroids. P-values were reported in the table using asterisks to report statistical significance (*, p-value < 0.05; ** p-value < 0.01).

Amplicon	Type	Sample Type	Site
16S	Crab's organs	0.672	0.012*
	Environmental samples	0.206	0.979
ITS	Crab's organs	0.067	0.024*
	Environmental samples	0.087	0.479

Table S7: Results of the likelihood ratio test (LRT) on sequence variants in different sample types. All columns returned from the LRT were reported even if the "log2FoldChange" column is not representative of a given contrast in this type of test. Columns are: asv, the name of the ASV tested; baseMean, the mean overall abundance of the each ASV tested; log2FoldChange, the logarithm of the ratio between two levels of the factor considered (since in LRT no contrast is provided, this column is not directly associated with the actual hypothesis test); lfcSE, the standard error of the log2FoldChange; stat, the difference in deviance between the reduced model and the full model; pvalue, the pvalue obtained by comparing stat to a chi-squared distribution; padj, adjusted p-values using the Benjamini–Hochberg method.

asv	baseMean	log2FoldChange	lfcSE	stat	pvalue	padj
ASV_16s_1	4193.925376	0.291294328	0.700618258	122.634551	2.16E-23	4.03E-21
ASV_16s_2	2974.042659	1.607565663	0.550839046	189.5774702	1.84E-37	2.23E-34
ASV_16s_3	2070.721697	0.018198732	0.684138918	135.0956634	5.41E-26	1.19E-23
ASV_16s_4	1782.252285	0.268915547	0.563220198	234.0417475	6.87E-47	1.66E-43
ASV_16s_5	2000.777163	0.003280727	0.720204251	143.0129591	1.19E-27	4.79E-25
ASV_16s_6	1323.431652	1.237259215	0.780931039	137.8100229	1.46E-26	3.93E-24
ASV_16s_7	1167.835274	2.774387931	0.665632684	179.9703419	1.98E-35	1.59E-32
ASV_16s_8	1085.69007	-0.675968088	0.799376444	95.86037298	7.71E-18	8.11E-16
ASV_16s_9	935.4725098	0.9077719	0.867358202	105.7160025	7.09E-20	1.07E-17
ASV_16s_10	794.9096287	2.404929393	0.693879963	140.8050448	3.45E-27	1.04E-24
ASV_16s_11	470.2334213	1.874036395	0.754795379	167.1691431	9.92E-33	6.00E-30
ASV_16s_12	377.7578079	1.23638359	0.807675489	73.56362708	2.81E-13	2.00E-11
ASV_16s_13	440.4518065	0.118524009	0.733370371	141.0183516	3.11E-27	1.04E-24
ASV_16s_14	433.9113438	5.917177117	0.817305923	41.03165421	7.98E-07	1.95E-05
ASV_16s_15	310.0818619	3.734246693	0.914789155	57.08347738	5.75E-10	2.32E-08
ASV_16s_16	378.197037	13.42414978	1.265589317	39.0576093	1.91E-06	4.31E-05
ASV_16s_17	479.0655672	2.404718759	0.94987472	75.94560532	9.21E-14	6.97E-12
ASV_16s_18	343.9012014	-0.4235691	0.862188286	111.0950016	5.44E-21	8.78E-19
ASV_16s_19	312.6063985	-5.018168812	0.95597808	65.28624478	1.32E-11	6.64E-10
ASV_16s_20	356.6465872	2.463998378	1.033193824	70.28455046	1.29E-12	7.83E-11
ASV_16s_21	297.9990391	2.069708084	0.920340511	87.7189494	3.64E-16	3.39E-14
ASV_16s_22	338.083022	-1.044090365	1.405334982	34.62703278	NA	NA
ASV_16s_23	229.6771001	0.94216184	0.871603846	67.89718994	3.93E-12	2.16E-10
ASV_16s_24	222.5022874	0.606809962	0.914264128	98.58186075	2.12E-18	2.44E-16
ASV_16s_25	180.8966598	1.616592344	0.973085154	71.98224842	5.87E-13	3.74E-11
ASV_16s_26	288.5115396	2.652946229	0.979885216	71.18018631	8.53E-13	5.29E-11
ASV_16s_27	292.1742582	-8.347071537	1.491711254	28.65794008	1.67E-04	0.002511345
ASV_16s_28	269.6337811	0.644903033	1.882991064	39.746861	1.41E-06	3.31E-05
ASV_16s_29	197.8823357	0.557676563	0.91310122	55.98127401	9.53E-10	3.66E-08
ASV_16s_30	195.4341619	-0.384343988	1.628639698	47.23127818	5.03E-08	1.45E-06
ASV_16s_31	265.9280636	-6.572505705	1.816483764	39.40184163	1.64E-06	3.78E-05
ASV_16s_32	281.3461091	2.858447537	0.842539248	102.2033073	3.78E-19	5.09E-17
ASV_16s_33	184.264651	1.306883617	0.959242501	76.41421672	7.40E-14	5.97E-12
ASV_16s_34	240.7294395	-0.570695193	0.911732757	96.52005594	5.64E-18	6.20E-16
ASV_16s_35	149.5418473	4.192684173	0.815160795	88.25709475	2.82E-16	2.73E-14
ASV_16s_36	67.4682983	-12.96087257	3.54988791	8.175642694	0.31736114	1
ASV_16s_37	173.8750968	4.281099471	1.142427188	39.26964351	1.74E-06	3.97E-05
ASV_16s_38	248.7349105	12.68042642	1.471647987	22.85944806	0.001803368	0.02040165
ASV_16s_39	275.3205525	2.144720981	0.979277728	85.25968652	1.16E-15	1.04E-13
ASV_16s_40	187.8488015	-2.811699731	1.258413702	50.07645311	1.40E-08	4.50E-07
ASV_16s_41	207.852463	-0.448069366	0.749085764	137.1285394	2.03E-26	4.91E-24
ASV_16s_42	233.8042313	2.187597109	0.680072201	123.5806343	1.37E-23	2.77E-21
ASV_16s_43	153.472643	4.193987517	0.773789611	56.5805242	7.24E-10	2.83E-08
ASV_16s_44	217.788306	-5.790611713	3.549643256	4.051361452	NA	NA
ASV_16s_45	166.9370268	2.479680722	0.5830744	101.901205	4.37E-19	5.56E-17
ASV_16s_46	151.0153322	6.031212634	1.068070647	48.73032324	2.56E-08	8.06E-07
ASV_16s_47	117.0885388	1.355793902	0.884052983	59.95810809	1.54E-10	6.77E-09
ASV_16s_48	222.9749782	7.425079606	1.161707537	76.23227515	8.06E-14	6.29E-12
ASV_16s_49	220.2448575	-3.491914225	1.251135679	45.28291075	NA	NA
ASV_16s_50	133.8619021	7.347607908	1.043220841	51.60345035	6.99E-09	2.45E-07
ASV_16s_51	140.7222212	2.927129385	0.679926219	66.96083269	6.06E-12	3.26E-10
ASV_16s_52	159.0278817	1.966066687	1.151776554	46.66698083	6.48E-08	1.80E-06
ASV_16s_53	165.531134	-7.903593784	1.800053254	19.45861289	0.006865807	0.061563401
ASV_16s_54	150.6245856	0.40706154	2.106616406	28.77836453	1.59E-04	0.002402654
ASV_16s_55	42.10738035	-0.336746521	3.550717738	7.540663116	0.374832165	1
ASV_16s_56	163.1414911	8.689392718	1.236345508	34.51259801	1.38E-05	2.79E-04
ASV_16s_57	148.3510771	-3.986332352	1.519578076	32.68599169	3.03E-05	5.60E-04
ASV_16s_58	159.7859853	1.635214469	0.838248011	72.22373256	5.24E-13	3.43E-11
ASV_16s_59	73.35437056	0.177603944	2.37341622	6.91752804	NA	NA
ASV_16s_60	98.18216444	1.602844453	0.950233449	62.83914621	4.08E-11	1.90E-09
ASV_16s_61	99.9562784	3.392880625	0.931178446	61.66422693	7.02E-11	3.15E-09
ASV_16s_62	80.58139714	4.290745369	1.445313243	24.17516775	0.001061316	0.012595328
ASV_16s_63	114.5930605	1.484599422	0.778857506	64.38954146	1.99E-11	9.85E-10
ASV_16s_64	85.74149799	3.408466288	1.16440725	33.1951178	2.43E-05	4.64E-04
ASV_16s_65	115.813847	-5.871187606	1.526312969	34.6244741	1.32E-05	2.68E-04
ASV_16s_66	30.43471246	-2.01565948	2.976732894	9.116256966	0.24441673	0.961181421

ASV_16s_67	65.7103055	-6.102988615	2.498662071	8.533780635	NA	NA
ASV_16s_68	133.4604682	11.24303777	1.05876325	45.59858409	1.05E-07	2.85E-06
ASV_16s_69	27.83351883	-7.484669527	3.550697117	7.842848058	0.346655308	1
ASV_16s_70	128.2235943	2.593552636	1.356723396	39.46351692	1.59E-06	3.71E-05
ASV_16s_71	29.89637842	-6.990308832	3.550389835	7.305537665	0.397775538	1
ASV_16s_72	81.76448706	-1.632456789	1.587601029	23.61995465	0.001328445	0.015612394
ASV_16s_73	111.5017579	0.427251229	1.06343681	50.64262612	1.08E-08	3.58E-07
ASV_16s_74	107.167734	1.504323746	0.870345407	25.86568759	5.32E-04	0.007159013
ASV_16s_75	16.93858428	-27.53545452	3.551437631	3.304981645	0.855429351	1
ASV_16s_76	97.21177708	4.135766	1.594041111	20.09555013	0.005366953	0.051561082
ASV_16s_77	114.7470113	-0.407345545	1.643281124	45.96074245	8.90E-08	2.45E-06
ASV_16s_78	82.02671683	10.04949116	1.52362038	22.34341696	0.002216132	0.024498889
ASV_16s_79	109.1089527	-3.60429205	0.930052398	68.35340932	3.18E-12	1.79E-10
ASV_16s_80	28.29874765	-4.686865151	3.550725889	7.352250232	0.393147481	1
ASV_16s_81	91.58333077	0.393333527	1.044968485	41.47656938	6.56E-07	1.65E-05
ASV_16s_82	93.43312519	8.260628265	2.18499804	13.0435773	0.07105373	0.41279216
ASV_16s_83	37.38652435	17.080293	3.551437934	3.510370922	0.834125788	1
ASV_16s_84	77.20334925	2.991057798	0.958560047	56.94799322	6.12E-10	2.43E-08
ASV_16s_85	111.5353199	2.063086655	0.883793264	72.62811075	4.34E-13	2.92E-11
ASV_16s_86	90.4001389	-1.434955437	1.254803013	48.82969657	2.45E-08	7.80E-07
ASV_16s_87	79.42747318	1.778052407	1.067305748	28.9369814	1.49E-04	0.002276439
ASV_16s_88	72.1037424	6.773978863	1.303647779	33.88489881	1.81E-05	3.54E-04
ASV_16s_89	30.40980122	20.05977569	3.551437771	3.555424529	0.82932059	1
ASV_16s_90	109.8628954	3.836448697	1.096335304	59.31736556	2.07E-10	8.62E-09
ASV_16s_91	21.1111176	-27.47572807	3.551437607	3.440301482	NA	NA
ASV_16s_92	20.48298897	-4.191057005	3.550721815	7.120495645	0.416442758	1
ASV_16s_93	93.99801464	1.140688173	1.890473831	30.42683389	7.93E-05	0.001332869
ASV_16s_94	107.5379097	1.739502028	1.189663464	68.90567438	2.46E-12	1.45E-10
ASV_16s_95	22.17799641	7.194320456	3.550315442	7.399417172	0.388509366	1
ASV_16s_96	67.39989913	0.823300349	0.865712128	25.90532598	5.24E-04	0.00712245
ASV_16s_97	68.57268716	-2.279235056	0.958524828	62.54720151	4.67E-11	2.13E-09
ASV_16s_98	94.0539742	4.70179665	1.104496552	59.83161134	1.63E-10	7.05E-09
ASV_16s_99	29.26373194	-4.360649179	3.549673578	5.662552722	0.579656174	1
ASV_16s_100	30.26480261	20.09050601	3.551287035	3.487630521	0.836533903	1
ASV_16s_101	77.95981565	1.791002212	1.126587995	47.42442672	4.61E-08	1.35E-06
ASV_16s_102	20.57948985	-3.65798922	3.550719407	7.325390684	0.3958044	1
ASV_16s_103	67.12996012	-10.99818157	2.783950994	5.432688392	0.607314851	1
ASV_16s_104	17.30994291	-27.0599505	3.551437629	3.318306439	0.854078916	1
ASV_16s_105	16.39034989	-26.98326386	3.551437644	5.164107672	0.639944121	1
ASV_16s_106	61.28641363	7.033517492	1.267062552	34.92481249	1.16E-05	2.37E-04
ASV_16s_107	61.05185033	7.104062059	1.090280822	32.10803379	3.88E-05	7.01E-04
ASV_16s_108	104.8974794	-9.597065212	1.775222365	17.06667699	0.016971259	0.132113882
ASV_16s_109	58.08696383	7.352397312	1.567213815	22.003465	0.002536922	0.027791351
ASV_16s_110	85.33703806	2.957816653	1.043287938	59.20674382	2.17E-10	8.92E-09
ASV_16s_111	17.12773712	-9.27547804	3.550526627	6.717388523	0.45888636	1
ASV_16s_112	63.70541371	-3.180971597	1.150315376	19.98158193	0.005609603	0.053258234
ASV_16s_113	56.5305513	-2.742606687	0.958298679	45.21595635	1.24E-07	3.34E-06
ASV_16s_114	80.69741301	2.412936081	1.228585978	44.11270184	2.03E-07	5.35E-06
ASV_16s_115	102.850309	29.99526575	3.549019801	3.222633773	0.863671408	1
ASV_16s_116	68.98098302	8.209652475	1.056094592	19.185953	0.007624352	0.0661597
ASV_16s_117	80.88724665	2.770854003	1.076244891	43.22544231	3.02E-07	7.85E-06
ASV_16s_118	15.654311	-1.488475869	3.54952693	5.36021814	0.616093278	1
ASV_16s_119	44.44723289	-1.018499291	0.901732082	51.61702892	6.95E-09	2.45E-07
ASV_16s_120	43.98575402	1.502818912	1.195349907	33.88376239	1.81E-05	3.54E-04
ASV_16s_121	57.22749602	2.914659975	0.970591873	19.70285304	0.006248574	0.057420468
ASV_16s_122	21.76350293	19.86681196	3.551437872	3.350438811	0.850803669	1
ASV_16s_123	69.24052707	1.160676674	2.050061117	25.64714441	5.82E-04	0.007702826
ASV_16s_124	73.01152389	4.724358818	1.397373848	42.40485116	4.35E-07	1.11E-05
ASV_16s_125	16.00069785	6.051928199	3.548569425	3.671930407	0.816693536	1
ASV_16s_126	50.38351944	-2.432512986	1.734187275	2.995195182	0.88544636	1
ASV_16s_127	59.19475968	1.645639128	1.441499609	31.48780338	5.05E-05	8.93E-04
ASV_16s_128	70.4946555	0.183566143	1.423285797	42.4676526	4.23E-07	1.09E-05
ASV_16s_129	18.02817827	-1.175289816	3.548574077	6.532800054	0.47908661	1
ASV_16s_130	59.39612014	20.37481652	3.549342419	3.847568926	0.797157651	1
ASV_16s_131	49.33553492	2.79829905	1.149224315	24.53326732	9.18E-04	0.011164744
ASV_16s_132	64.10944263	-2.600862223	1.128431232	48.63465091	2.68E-08	8.30E-07
ASV_16s_133	16.24804818	-8.865835837	3.550189354	7.002773409	0.428591148	1

ASV_16s_134	40.20439879	1.121563661	1.053096194	50.52808561	1.14E-08	3.72E-07
ASV_16s_135	14.98079097	-5.990677459	3.550797991	5.84197687	0.558317525	1
ASV_16s_136	69.38222663	-6.803384219	2.192018115	9.238048315	0.236014052	0.949152858
ASV_16s_137	19.32280795	23.89709886	3.550720051	2.470084991	0.929332798	1
ASV_16s_138	59.76363759	-6.335974482	2.690689045	6.603667367	0.471277376	1
ASV_16s_139	16.51971401	-15.24604755	3.548805158	5.473425765	0.602391134	1
ASV_16s_140	18.60105252	10.47189019	3.550867719	3.368306634	NA	NA
ASV_16s_141	35.70302087	-1.864715176	1.108654854	10.49105352	0.162411081	0.72839759
ASV_16s_142	65.07565608	-5.885500734	3.546123185	2.499073219	NA	NA
ASV_16s_143	43.81454322	-16.21985506	2.320694943	11.56931859	0.115641561	0.574017711
ASV_16s_144	18.25429078	19.76708533	3.551437939	3.242667542	NA	NA
ASV_16s_145	54.23435003	0.330125657	1.384971132	20.22922299	0.005095275	0.049540807
ASV_16s_146	56.83395646	0.901023934	1.96461295	25.3178077	6.66E-04	0.008535888
ASV_16s_147	83.79446315	-10.77989346	3.149453316	5.256508548	NA	NA
ASV_16s_148	16.98805889	28.3110158	3.5513614	2.684486003	NA	NA
ASV_16s_149	20.24915135	10.3910371	3.550847318	3.437127332	0.841839335	1
ASV_16s_150	59.13065404	3.42229588	0.91632392	52.60611001	4.43E-09	1.63E-07
ASV_16s_151	46.30363044	3.438297314	0.836999092	104.4722701	1.28E-19	1.83E-17
ASV_16s_152	41.41355643	11.20840314	1.366248535	30.0600654	9.26E-05	0.00150428
ASV_16s_153	12.22631124	6.319774065	3.550542113	6.156089548	0.521646567	1
ASV_16s_154	25.75854943	-8.35618173	3.074072753	2.170907373	0.949778358	1
ASV_16s_155	12.04089214	-25.98833559	3.551437943	5.007086886	0.659098336	1
ASV_16s_156	40.30740792	7.986927331	1.194749033	36.11115818	6.91E-06	1.44E-04
ASV_16s_157	16.73684108	19.71184106	3.551437973	3.283142685	0.857632695	1
ASV_16s_158	12.42601391	-9.852224567	3.550681139	5.559294393	0.592041814	1
ASV_16s_159	34.68604283	2.094278941	1.19950027	20.6412088	0.004339108	0.043053195
ASV_16s_160	39.43771886	-0.2975863	1.386069125	22.74483796	0.001888011	0.021259883
ASV_16s_161	26.47551578	-5.553902368	3.547624464	3.923121185	NA	NA
ASV_16s_162	33.78882528	1.017921732	0.899794225	41.30148255	7.09E-07	1.75E-05
ASV_16s_163	37.64887174	0.338221871	1.028496769	59.628229	1.79E-10	7.61E-09
ASV_16s_164	59.77740441	-30	3.546714059	3.427728718	NA	NA
ASV_16s_165	33.62520054	0.205233165	0.917896989	15.85310867	0.026492791	0.192609753
ASV_16s_166	46.4942745	2.623011321	2.654305835	8.103787567	0.323531107	1
ASV_16s_167	42.07164078	-14.95867274	2.899519023	3.153008013	0.870496092	1
ASV_16s_168	11.32953876	-2.78830189	3.550719903	6.52332543	0.480135647	1
ASV_16s_169	34.42785375	0.507576509	0.864642446	21.94939182	0.00259195	0.028266263
ASV_16s_170	13.42438182	-3.882120265	3.004893901	7.232533304	0.405076942	1
ASV_16s_171	40.42797346	1.826365728	1.005028708	37.17088366	4.35E-06	9.41E-05
ASV_16s_172	31.75492066	-2.921117029	0.941711931	19.9400719	0.005700591	0.053700901
ASV_16s_173	56.29704818	2.23076722	1.142934352	68.60819078	2.82E-12	1.63E-10
ASV_16s_174	40.69965498	0.294192241	0.884597297	40.16566213	1.17E-06	2.80E-05
ASV_16s_175	45.30437603	-0.648464089	1.857443335	26.01242962	5.01E-04	0.006854021
ASV_16s_176	41.67030513	0.456443486	1.31214324	48.05988569	3.47E-08	1.04E-06
ASV_16s_177	24.62237513	0.643805955	1.237504738	32.76002098	2.93E-05	5.46E-04
ASV_16s_178	13.03940901	-5.290153819	3.550800379	5.501561754	0.5989955	1
ASV_16s_179	32.44382764	7.017102783	1.375754431	24.6397367	8.79E-04	0.010745517
ASV_16s_180	42.10314304	0.576170615	0.825166298	146.556634	2.14E-28	1.04E-25
ASV_16s_181	28.87140255	4.451151254	1.067288666	54.96218276	1.52E-09	5.74E-08
ASV_16s_182	35.56931226	-5.357922732	1.513928116	15.09701897	0.034775679	0.239863019
ASV_16s_183	32.63558012	7.638178247	1.307264465	30.45040791	7.85E-05	0.00132886
ASV_16s_184	29.27878925	0.315560116	1.359724182	21.54815606	0.003038156	0.031704204
ASV_16s_185	44.10798651	3.463684991	1.181502456	52.2585466	5.19E-09	1.88E-07
ASV_16s_186	12.99480393	19.56241586	3.551438104	3.03430212	NA	NA
ASV_16s_187	23.10411114	0.158584457	2.591395327	7.20675875	0.407674548	1
ASV_16s_188	41.445297	0.060435951	1.145804199	63.36774433	3.20E-11	1.52E-09
ASV_16s_189	30.8376768	1.349909204	0.951811115	29.20322062	1.33E-04	0.002061674
ASV_16s_190	33.56021782	-0.572443995	1.112180902	31.08253105	6.00E-05	0.001038126
ASV_16s_191	39.90985332	0.588309597	0.918583031	116.092927	4.98E-22	8.61E-20
ASV_16s_192	41.46110906	2.367881257	2.001788159	22.65928231	0.00195371	0.021897837
ASV_16s_193	39.7192056	0.35246178	1.213788948	65.57447437	1.15E-11	5.94E-10
ASV_16s_194	37.51525162	0.333151015	1.352907032	44.22998179	1.93E-07	5.13E-06
ASV_16s_195	10.72050292	-30	3.551334269	4.773536185	0.687577063	1
ASV_16s_196	35.10486366	-1.656964965	1.640385232	27.58376961	2.62E-04	0.003791469
ASV_16s_197	30.56258361	1.410629299	1.563505458	14.94490663	0.036712142	0.250366464
ASV_16s_198	5.132284278	-3.533973363	3.550076079	2.244194251	NA	NA
ASV_16s_199	10.17874876	-10.81284307	3.550051128	5.427860679	0.607898895	1
ASV_16s_200	5.773688431	-26.22912934	3.55143786	2.643648199	0.915897537	1

ASV_16s_201	45.68375894	5.413508261	3.546370151	5.235116653	NA	NA
ASV_16s_202	12.29239621	29.81065883	3.54970579	2.445777225	NA	NA
ASV_16s_203	49.68670093	-1.461863335	3.548422697	7.082588043	0.420331829	1
ASV_16s_204	34.25480931	-0.885361983	0.799880009	83.59296878	2.54E-15	2.20E-13
ASV_16s_205	21.81317141	-0.210872244	1.636147024	10.48991749	0.162467864	0.72839759
ASV_16s_206	12.65776699	-4.659345046	3.549783588	2.207430426	0.947473154	1
ASV_16s_207	9.111474397	-0.378610242	3.546471772	2.322884407	NA	NA
ASV_16s_208	44.74386522	1.38717141	2.013803538	20.20040766	0.00515269	0.049898653
ASV_16s_209	35.09547128	6.90716263	1.187737169	25.83936257	5.38E-04	0.007196899
ASV_16s_210	28.18227711	10.61170869	1.860546199	13.93345558	0.052377647	0.329366973
ASV_16s_211	24.89727646	1.485271632	1.388293017	28.81158454	1.57E-04	0.002384318
ASV_16s_212	8.684936791	-4.407797532	3.550738822	5.48901063	0.600509726	1
ASV_16s_213	26.15478048	10.35977063	2.067556479	9.426863175	0.223443799	0.915325611
ASV_16s_214	30.07483766	8.585827035	1.622754158	19.22311744	0.007516396	0.065693843
ASV_16s_215	4.684129143	-25.9460162	3.550202514	2.218631628	NA	NA
ASV_16s_216	30.19104513	0.669066925	1.119960399	11.69925579	0.110892939	0.556995447
ASV_16s_217	34.44209178	-3.008386164	2.052368121	14.71541878	0.03982535	0.263434898
ASV_16s_218	26.00221413	-18.81370175	2.65572355	3.360385901	NA	NA
ASV_16s_219	33.94433757	-6.213482963	2.905430373	6.681090467	0.462822256	1
ASV_16s_220	27.75613586	3.675686786	1.330005876	19.95718589	0.005662907	0.053554286
ASV_16s_221	8.262487342	-29.91446599	3.549864035	2.077743574	NA	NA
ASV_16s_222	37.5829011	0.675823081	1.658777993	30.10760245	9.07E-05	0.001484328
ASV_16s_223	8.372561022	-25.70755465	3.551437882	4.829171951	0.680801033	1
ASV_16s_224	8.673531746	-8.083820157	3.5506244	5.931128817	0.547813784	1
ASV_16s_225	25.29713947	1.570000465	1.212327594	27.2107635	3.05E-04	0.004374777
ASV_16s_226	25.55412277	15.09026357	3.551236643	1.051835011	0.993948471	1
ASV_16s_227	18.75297662	-4.771263439	2.052817677	8.203617143	0.314982189	1
ASV_16s_228	21.17820207	1.280858303	1.637347522	13.06094582	NA	NA
ASV_16s_229	25.78881927	9.557681555	1.40729075	21.70924155	0.002850697	0.030133107
ASV_16s_230	33.06348801	3.751146401	1.867223583	27.73305614	2.46E-04	0.0036062
ASV_16s_231	22.50898518	3.739633495	1.994264627	11.01967059	0.137763298	0.651415906
ASV_16s_232	31.30061029	22.18532714	3.304046271	4.396773078	0.733109222	1
ASV_16s_233	32.56774451	0.600795688	1.919549158	25.3288253	6.63E-04	0.008535888
ASV_16s_234	31.46662278	3.353307509	1.174136021	32.88325568	2.78E-05	5.22E-04
ASV_16s_235	23.38862075	2.857475879	1.746107861	12.02795304	0.099643357	0.523289732
ASV_16s_236	22.10967401	-2.385134238	1.210356597	19.16492214	0.007686103	0.066457344
ASV_16s_237	24.27839976	1.209914179	0.998223009	37.482546	3.80E-06	8.29E-05
ASV_16s_238	7.668340337	-24.57153654	3.55058036	2.518100689	NA	NA
ASV_16s_239	22.92013053	11.12404494	1.925630285	12.09919698	0.097343552	0.519090802
ASV_16s_240	6.439634604	2.94581071	2.70522641	1.96517617	0.961742593	1
ASV_16s_241	27.01603834	0.233267238	1.303711719	19.90524323	0.005778033	0.053990899
ASV_16s_242	7.07450506	6.749540786	3.550713495	2.187078901	NA	NA
ASV_16s_243	23.72369	11.42591723	1.577151661	16.71522142	0.019327711	0.148547265
ASV_16s_244	27.82924367	8.195041176	1.137894376	5.746509377	0.569640243	1
ASV_16s_245	28.59358054	1.702968825	1.188083434	36.43351236	6.00E-06	1.26E-04
ASV_16s_246	23.35142737	0.964350518	1.239377245	34.22973987	1.56E-05	3.12E-04
ASV_16s_247	28.36043204	1.284096482	1.171455594	36.98920014	4.71E-06	1.01E-04
ASV_16s_248	30.52121576	-10.38362608	3.202697069	7.046662814	0.424037583	1
ASV_16s_249	22.50592515	10.47245167	1.659101498	16.61476261	0.020056469	0.153660479
ASV_16s_250	4.014660339	-29.8037477	3.550365977	1.11213081	NA	NA
ASV_16s_251	36.60095503	-7.474509775	3.23663759	7.373349886	NA	NA
ASV_16s_252	6.876566873	0.525387166	3.550729129	4.73816473	0.691880545	1
ASV_16s_253	26.89487212	0.055000607	1.810454988	9.079403275	0.247005397	0.964516234
ASV_16s_254	20.61832406	9.421804413	3.545213187	4.316463351	0.742690388	1
ASV_16s_255	22.04270368	9.411529863	1.513650575	15.75678343	0.027433725	0.198259845
ASV_16s_256	15.68102448	-3.759727135	3.54631868	3.638411243	NA	NA
ASV_16s_257	31.78737728	1.446098828	1.473171845	33.76451109	1.91E-05	3.69E-04
ASV_16s_258	5.95717331	-25.48355584	3.551437917	4.139738549	NA	NA
ASV_16s_259	24.20847681	-30	3.182252952	6.39383653	0.494587275	1
ASV_16s_260	27.56407663	1.768086862	1.012931692	32.26159053	3.63E-05	6.61E-04
ASV_16s_261	20.71026954	0.403452806	1.195157562	30.23279658	8.61E-05	0.001417389
ASV_16s_262	22.3485473	0.351735575	1.079513077	32.99624106	2.65E-05	5.02E-04
ASV_16s_263	25.99904857	3.792516448	0.917897437	37.60295923	3.60E-06	7.93E-05
ASV_16s_264	24.93432567	20.01764209	3.549562353	3.889635019	0.792397999	1
ASV_16s_265	6.43235831	2.227437387	3.550687285	1.911760068	NA	NA
ASV_16s_266	40.45744883	-11.4418624	3.548502954	3.531048056	NA	NA
ASV_16s_267	19.03954258	2.288818442	1.170657421	25.02618136	7.51E-04	0.009501403

ASV_16s_268	20.28924897	12.13931468	2.129412758	8.594180933	0.28311938	1
ASV_16s_269	21.08931631	11.22363917	1.427237365	19.62955898	0.00642794	0.058327015
ASV_16s_270	23.16078378	1.686322277	1.18101615	22.41261661	0.002155854	0.023941842
ASV_16s_271	16.85754406	6.770892017	1.828207339	17.72898637	0.01325447	0.109146503
ASV_16s_272	31.69344354	9.044439355	2.395222284	6.77646912	0.452519144	1
ASV_16s_273	17.14367868	-3.453550232	1.27548411	11.80603276	0.107120742	0.548286079
ASV_16s_274	24.3592624	-3.111828205	1.092736702	14.48013834	0.043271631	0.278580897
ASV_16s_275	28.87633068	2.490759515	3.009535153	6.143868662	0.523054735	1
ASV_16s_276	30.43094628	19.41849134	3.550576922	3.637918377	0.820408781	1
ASV_16s_277	27.67737665	1.04878785	2.024153606	21.77915086	0.002772888	0.029443688
ASV_16s_278	22.57640392	8.422595848	1.405394878	17.13574722	0.016541519	0.129989756
ASV_16s_279	22.23659684	0.740191864	1.203796221	29.77368767	1.04E-04	0.001664081
ASV_16s_280	19.73118973	11.100338	1.709505002	15.6317617	0.028702017	0.205584565
ASV_16s_281	25.03773257	0.335677534	2.034593084	21.00972307	0.003755786	0.038366068
ASV_16s_282	19.0752775	10.11921265	1.87384793	14.24376243	0.047011814	0.298728613
ASV_16s_283	19.25917513	5.762173421	1.964448957	12.03666243	0.099359632	0.523289732
ASV_16s_284	5.10934035	-6.941052731	3.550864262	3.187065043	0.867174716	1
ASV_16s_285	5.975942346	-25.52097972	3.551437849	2.66479261	NA	NA
ASV_16s_286	27.4925237	4.915373353	1.68065053	29.52433825	1.16E-04	0.001824488
ASV_16s_287	9.486350968	10.25650294	3.546952936	1.498575935	0.98235892	1
ASV_16s_288	22.81457032	-24.51603591	2.695013056	7.046588925	0.424045225	1
ASV_16s_289	5.641543492	0.249847566	3.543779383	1.136312752	0.992320134	1
ASV_16s_290	24.81468016	0.944541063	1.754325159	29.50395556	1.17E-04	0.001828345
ASV_16s_291	20.72176177	1.954258225	1.112124274	28.46305673	1.81E-04	0.002708055
ASV_16s_292	19.01901864	9.946837852	1.851200773	13.03455144	0.071271013	0.41279216
ASV_16s_293	18.73630152	9.112003374	2.089684724	11.16861304	0.131431436	0.628844875
ASV_16s_294	6.271207975	-25.04579481	3.551438052	4.504124996	NA	NA
ASV_16s_295	16.09333476	-0.35382509	1.443847907	11.65657284	0.112433439	0.562399495
ASV_16s_296	18.63233563	7.641441729	3.54958264	3.280815903	NA	NA
ASV_16s_297	22.23196216	1.940725551	1.63947159	17.38146035	0.015095307	0.121819129
ASV_16s_298	19.18367442	-14.75926615	2.585983118	4.962331482	0.664560244	1
ASV_16s_299	14.72755253	4.42627486	2.101458086	9.783222928	0.201194471	0.847116199
ASV_16s_300	15.49020873	2.001664744	1.237642456	25.40365641	6.43E-04	0.00839082
ASV_16s_301	18.01751492	10.04917253	2.616912869	7.374542126	0.390951039	1
ASV_16s_302	23.52904506	2.877266461	1.480964802	20.49321926	0.004597291	0.045428746
ASV_16s_303	4.716171858	-2.885981606	3.549684513	2.209936114	0.947313025	1
ASV_16s_304	23.38892395	0.063223924	1.794224622	26.9212841	3.44E-04	0.004874964
ASV_16s_305	5.839496027	-24.70017553	3.550582176	2.36772595	NA	NA
ASV_16s_306	7.516250495	19.23357019	3.551438507	2.698561503	NA	NA
ASV_16s_307	17.83571883	9.888398785	2.819154309	5.985317423	0.54146481	1
ASV_16s_308	19.6851738	1.352421971	1.344741076	19.9214413	0.005741891	0.053880303
ASV_16s_309	20.97531836	2.404636659	1.000800112	30.52924463	7.59E-05	0.001294276
ASV_16s_310	24.53889445	3.914849856	1.616168779	16.76325561	0.018988216	0.146402771
ASV_16s_311	19.32431695	6.497278121	1.857252297	6.254110161	0.510411435	1
ASV_16s_312	14.77427917	2.12212128	1.20288299	8.681449822	0.276342782	1
ASV_16s_313	17.93802202	-5.183414789	1.378339875	10.83221602	0.146106969	0.676918169
ASV_16s_314	22.32176216	1.389344915	1.206924086	25.38567059	6.48E-04	0.00839082
ASV_16s_315	19.42310182	-6.726940584	2.920285026	5.668739433	0.578916359	1
ASV_16s_316	17.53190121	10.41951871	1.456080894	19.07201945	0.007964701	0.068377804
ASV_16s_317	17.37682729	-7.530606769	1.599027916	12.57078878	0.083282683	0.459287872
ASV_16s_318	13.91204105	-2.480859337	1.853050117	11.1182955	0.133541618	0.636075028
ASV_16s_319	15.97977493	1.830529624	1.360510174	17.19858245	0.016159597	0.128270111
ASV_16s_320	26.54115123	0.545748624	1.379336592	41.32055902	7.03E-07	1.75E-05
ASV_16s_321	5.529265397	-24.97842477	3.551438102	4.338241665	NA	NA
ASV_16s_322	16.56250705	5.770606275	1.413559036	14.59475104	0.041559534	0.272671087
ASV_16s_323	24.83251295	-16.19746737	3.546054004	3.035900781	NA	NA
ASV_16s_324	25.92971262	1.723813931	1.369371715	33.46440056	2.17E-05	4.17E-04
ASV_16s_325	16.27887758	8.330103734	2.237067902	10.44252633	0.164851729	0.736358
ASV_16s_326	16.48200848	9.643078898	1.951955428	12.05987663	0.098606908	0.521238697
ASV_16s_327	16.13459979	4.261761379	1.649251485	17.71925273	0.013302972	0.109174562
ASV_16s_328	16.86410302	10.13241632	2.492812335	7.096995934	0.418851105	1
ASV_16s_329	16.33832279	10.98286518	1.598308448	17.13126584	0.016569083	0.129989756
ASV_16s_330	15.54768242	-7.642058589	1.706629942	12.37981628	0.088740117	0.482786118
ASV_16s_331	15.25603727	12.24959955	2.858038361	5.485783232	0.600899232	1
ASV_16s_332	12.84522967	3.492259086	1.244786423	20.46883758	0.004641232	0.045624643
ASV_16s_333	9.316204579	-9.383317506	3.552157344	2.920925584	NA	NA
ASV_16s_334	21.55433712	0.734753302	2.544269264	12.97751614	0.072658105	0.418822075

ASV_16s_335	17.54935312	10.87970427	1.599416841	15.44678728	0.030680177	0.216550173
ASV_16s_336	17.56330152	11.16171804	1.449990989	19.25217912	0.007433005	0.06520038
ASV_16s_337	5.498203989	-11.85705101	3.549483421	4.614042538	0.706944673	1
ASV_16s_338	13.54444631	1.682300661	3.546138321	4.876534272	NA	NA
ASV_16s_339	16.14809307	8.244248133	1.681057549	10.25404205	0.174628408	0.76652072
ASV_16s_340	0.083053082	0.077649298	3.551802732	0.234135117	0.999956904	NA
ASV_16s_341	16.07680311	5.429199526	1.575839111	15.27076721	0.032681026	0.22735852
ASV_16s_342	15.79505586	12.54980023	1.881201753	14.65276417	0.040717189	0.267870421
ASV_16s_343	11.88581964	3.934491504	1.770368194	9.9749554	0.189997171	0.814129472
ASV_16s_344	4.064490224	-24.17720378	3.551438079	3.541177967	0.830844909	1
ASV_16s_345	4.839660653	-2.528738358	3.545224531	2.7257547	NA	NA
ASV_16s_346	23.61182147	-0.337461831	1.512728684	27.39527994	2.83E-04	0.004076185
ASV_16s_347	19.2788076	-0.373226793	1.601664085	24.29091289	0.001012649	0.012136745
ASV_16s_348	23.14090424	8.440996273	3.549741169	5.104246184	0.647243147	1
ASV_16s_349	14.89225233	9.27588759	2.066708236	8.931287005	0.257627096	0.998771776
ASV_16s_350	15.91163246	11.0251403	1.696587977	13.44614849	0.061955918	0.378775951
ASV_16s_351	15.06932072	0.843244515	1.316518035	33.95928706	1.75E-05	3.48E-04
ASV_16s_352	15.08045428	0.736094832	1.369512761	26.02615729	4.98E-04	0.006854021
ASV_16s_353	25.27355743	1.952541113	1.560882293	24.43674159	9.54E-04	0.0115534
ASV_16s_354	10.7318184	17.5617619	3.549937607	3.288534518	NA	NA
ASV_16s_355	15.66708211	-0.274387707	1.190170175	15.95296888	0.025549534	0.187440675
ASV_16s_356	13.96301121	12.38859157	2.555276886	7.893526967	0.342076238	1
ASV_16s_357	5.97511732	-13.87202563	3.552157344	2.648456255	NA	NA
ASV_16s_358	4.241949823	-25.00175574	3.551437987	2.454240268	NA	NA
ASV_16s_359	20.1790243	4.707424482	1.550232206	23.6079566	0.001334889	0.015612394
ASV_16s_360	14.36318065	4.229169967	2.050121225	9.945878751	0.191661275	0.816922443
ASV_16s_361	14.80359611	3.599508876	1.490067071	11.43596195	0.120700743	0.59514562
ASV_16s_362	14.53048685	11.50819152	2.504876108	6.726038355	0.457951116	1
ASV_16s_363	21.91421123	4.058300549	1.580915324	18.34574993	0.010503718	0.088052868
ASV_16s_364	4.344341501	-4.991615617	3.550775583	4.366271525	0.736754795	1
ASV_16s_365	18.58222355	1.287135627	0.851319559	63.45949846	3.07E-11	1.48E-09
ASV_16s_366	14.94507369	10.12604322	1.415844873	21.47404283	0.003128377	0.032366672
ASV_16s_367	16.64406498	1.788117837	1.380776226	30.87454748	6.56E-05	0.001125935
ASV_16s_368	14.83676979	0.703841755	2.15463177	9.903760095	0.194093221	0.82294166
ASV_16s_369	19.59901871	1.363550676	1.909172888	20.46135045	0.004654807	0.045624643
ASV_16s_370	11.85613838	-2.837080608	1.973403342	12.09028639	0.097628573	0.519090802
ASV_16s_371	18.74278265	-2.253561759	1.571820958	15.59897053	0.029043701	0.207418288
ASV_16s_372	18.67913459	0.906228974	1.08982959	48.60033045	2.72E-08	8.33E-07
ASV_16s_373	14.44461047	10.13850557	2.063874684	9.108492678	0.244960321	0.961181421
ASV_16s_374	19.69797258	1.196153114	2.196903283	14.68082664	0.040315463	0.265950235
ASV_16s_375	25.94051919	7.55165111	1.639168348	19.7649315	0.006100445	0.056370909
ASV_16s_376	12.12501692	1.200076149	1.5993543	7.64864839	0.364592352	1
ASV_16s_377	14.09914824	12.2068011	2.249628963	10.21684672	0.176614395	0.771147377
ASV_16s_378	16.98744485	2.505602917	1.202891113	17.0814515	0.01687845	0.13181525
ASV_16s_379	9.556543226	18.18890085	3.551439046	2.887744759	NA	NA
ASV_16s_380	4.454001216	-24.21607951	3.550584542	2.218255323	NA	NA
ASV_16s_381	13.68199403	12.07425544	2.194960637	10.88617807	0.143661515	0.673682217
ASV_16s_382	14.37591841	-2.604921791	0.981802967	17.73331153	0.013232972	0.109146503
ASV_16s_383	15.28898399	-6.09148861	1.692622575	20.08392019	0.005391241	0.051589704
ASV_16s_384	15.74365924	-6.23437293	1.751490021	12.14246858	0.095969985	0.515173686
ASV_16s_385	13.88060309	-0.891466523	1.620696352	14.75058176	0.039332852	0.262951231
ASV_16s_386	9.227217469	-7.240457069	2.052971335	6.427086172	0.490856371	1
ASV_16s_387	25.1128971	29.9621421	3.549051403	2.495645651	NA	NA
ASV_16s_388	13.52732866	10.05513636	2.475246824	6.856619302	0.443959888	1
ASV_16s_389	11.5167151	-21.68526524	3.55019716	2.556525204	NA	NA
ASV_16s_390	0.795672241	2.147404639	3.548677439	1.33134918	NA	NA
ASV_16s_391	13.33652294	-17.85762943	3.552157344	2.051969965	0.956907982	1
ASV_16s_392	13.91213088	10.34480103	1.58928558	14.97276662	0.036350086	0.249301861
ASV_16s_393	9.744631222	1.098551897	1.937627264	9.66086775	NA	NA
ASV_16s_394	15.74249038	3.612486689	1.086021911	21.67069983	0.002894498	0.030335844
ASV_16s_395	21.08172104	-9.190928098	3.552157344	2.064505207	0.956183963	1
ASV_16s_396	13.61558417	1.433267006	1.268595526	17.61846129	0.013815293	0.112996031
ASV_16s_397	17.72982954	-7.510351387	2.885037497	5.331564145	0.6195703	1
ASV_16s_398	12.5264141	-10.39821831	3.549052196	4.539290562	0.715981008	1
ASV_16s_399	17.39441148	-0.070630852	1.694267138	23.02820737	0.001685434	0.019338555
ASV_16s_400	13.93005389	1.908666356	1.03369029	31.86291286	4.31E-05	7.67E-04
ASV_16s_401	17.06742194	4.79562401	1.585172628	12.77784444	0.077709755	0.437523994

ASV_16s_402	14.51712651	-0.5351074	1.015106097	50.79736686	1.01E-08	3.43E-07
ASV_16s_403	17.53378781	0.235423032	1.906356853	14.74387173	0.039426393	0.262951231
ASV_16s_404	18.98792327	-4.927780822	2.523761739	8.269994188	0.309389324	1
ASV_16s_405	15.257897	-28.65749968	2.036778105	10.86290423	0.144711875	0.673745093
ASV_16s_406	4.6687653	17.30307237	3.551442105	3.950351832	0.785477845	1
ASV_16s_407	13.37746795	5.506883854	2.112008005	11.05002622	0.136451738	0.647744428
ASV_16s_408	11.16507021	3.606974831	1.825304805	9.198943392	0.238686629	0.955141038
ASV_16s_409	3.888379732	-25.16722707	3.55143803	2.400774264	NA	NA
ASV_16s_410	12.51974938	-12.2412324	3.548496558	4.54557786	0.715222208	1
ASV_16s_411	16.78493886	1.549573446	1.021184367	47.1684994	5.18E-08	1.47E-06
ASV_16s_412	18.49027987	17.44308981	3.550586118	5.028542595	NA	NA
ASV_16s_413	5.316396925	-8.838182159	3.549163	1.762579153	NA	NA
ASV_16s_414	2.425180764	-24.52889019	3.551438342	2.110828484	0.953452241	1
ASV_16s_415	9.159035743	7.554368164	3.544805908	2.224719804	NA	NA
ASV_16s_416	13.29296421	-4.302065405	1.177259724	19.34635763	0.007168849	0.063570108
ASV_16s_417	16.36696613	-3.731886928	2.049005958	12.65960414	0.080849372	0.448936538
ASV_16s_418	13.52343031	-2.386343977	1.402874425	21.69861637	0.002862707	0.030133107
ASV_16s_419	12.53995036	7.755320908	3.548945898	2.026220433	0.95837487	1
ASV_16s_420	15.23729054	2.431641034	1.100663892	31.90697127	4.23E-05	7.58E-04
ASV_16s_421	16.29632777	-1.62979713	3.104478282	6.379314212	0.496221069	1
ASV_16s_422	4.777513167	-1.281670279	3.550723785	5.158970497	0.640570269	1
ASV_16s_423	5.797844272	19.07418766	3.551438786	2.53930047	0.92410874	1
ASV_16s_424	8.130705533	-30	3.550111564	3.021405499	0.883015008	1
ASV_16s_425	3.52008502	-25.06728581	3.551438118	2.998996863	NA	NA
ASV_16s_426	13.63591998	-9.342507349	1.756983597	9.121167214	0.244073446	0.961181421
ASV_16s_427	13.70340478	-30	2.377092017	8.723847048	0.273095431	1
ASV_16s_428	13.17320143	1.779902196	1.313887036	11.96120158	0.101842017	0.529097687
ASV_16s_429	14.81319852	6.409951628	2.939324474	4.507357248	0.719831157	1
ASV_16s_430	11.67602901	10.66352502	1.780460468	11.73106838	0.109756949	0.554325566
ASV_16s_431	3.642259576	-24.98993267	3.551026057	2.20456042	NA	NA
ASV_16s_432	10.17217161	4.49159693	2.153358845	2.966416511	0.888091493	1
ASV_16s_433	15.64609129	-3.015271585	1.508394958	21.4130506	0.003204574	0.033013932
ASV_16s_434	15.99489498	-7.798409925	2.425651192	11.771688	0.108321462	0.549782513
ASV_16s_435	12.36268082	-0.555240991	1.284888455	27.03124618	3.29E-04	0.004685228
ASV_16s_436	12.13190726	4.378903861	1.696705673	9.130891257	0.243394746	0.961181421
ASV_16s_437	15.58621612	0.355342405	2.44354731	8.463683431	0.293485737	1
ASV_16s_438	14.63335087	1.300814729	1.06258284	52.66263379	4.32E-09	1.61E-07
ASV_16s_439	13.47531248	4.363664956	1.019270904	26.50686041	4.09E-04	0.005752761
ASV_16s_440	12.77374947	-1.761468343	1.475163107	14.54440695	0.042303658	0.274867208
ASV_16s_441	17.09499838	1.211169095	1.108750362	48.12504309	3.37E-08	1.02E-06
ASV_16s_442	5.189409391	-3.077313721	3.54788833	1.673970465	0.975669068	1
ASV_16s_443	7.523802453	-24.76758301	3.550935172	2.576386837	0.921236071	1
ASV_16s_444	15.95081663	1.280149055	2.556479921	11.78997366	0.107680703	0.549323381
ASV_16s_445	16.68797226	4.546633762	1.858962235	21.81611197	0.002732589	0.029272552
ASV_16s_446	15.74554775	-3.951527998	2.253115463	6.900919066	0.439268622	1
ASV_16s_447	14.97293264	-14.61731015	3.016279239	2.379456383	0.935897383	1
ASV_16s_448	11.99222637	1.460288517	1.931333998	9.326446957	0.230060362	0.934523719
ASV_16s_449	9.046860128	-3.171227255	3.37971464	3.106861859	0.87494373	1
ASV_16s_450	11.46941476	-2.936544655	1.901854278	6.166642018	0.520431944	1
ASV_16s_451	8.830292102	8.739784666	3.547050108	2.889145177	0.895064276	1
ASV_16s_452	4.559947053	1.343936108	3.550449057	3.642720413	0.819885653	1
ASV_16s_453	11.45186475	-21.53275367	3.552157344	1.93365287	0.963421011	1
ASV_16s_454	5.13666717	22.66437848	3.550046693	2.647329875	0.915600597	1
ASV_16s_455	12.54390376	-4.384018633	2.160079656	10.41491929	0.166254054	0.739891661
ASV_16s_456	12.3470056	-29.83172293	1.857769052	9.964085982	0.190617836	0.815345903
ASV_16s_457	11.38207101	0.355580656	2.421771749	3.789567152	0.803671099	1
ASV_16s_458	11.91962225	0.880186398	1.530104887	18.90902352	0.008477161	0.072520167
ASV_16s_459	8.28793767	-7.498155876	3.546874461	2.900384456	NA	NA
ASV_16s_460	1.948836631	-24.23936706	3.551438545	1.976558813	NA	NA
ASV_16s_461	11.89960565	-8.847359181	3.549011045	2.053314906	0.95683061	1
ASV_16s_462	15.04885776	5.215340871	1.137282763	24.9013085	7.90E-04	0.009858455
ASV_16s_463	7.21423863	-29.99779181	3.550662938	2.381450502	0.935756353	1
ASV_16s_464	3.564367705	-23.78750672	3.550587041	2.095301501	NA	NA
ASV_16s_465	4.150575424	-6.961656964	2.516096861	1.651721913	0.976587758	1
ASV_16s_466	13.99224033	1.266746876	2.247173963	11.11381908	0.133730768	0.636075028
ASV_16s_467	11.37929198	-8.536580648	1.732567671	15.92159399	0.025842423	0.189016632
ASV_16s_468	3.785230934	-0.900288035	3.548371803	5.263230549	0.627874289	1

ASV_16s_469	13.54711023	21.03668342	3.550607272	3.842948969	0.797678586	1
ASV_16s_470	14.62874614	2.202296703	2.83904739	8.377284537	0.300503288	1
ASV_16s_471	8.103676688	0.93046372	2.532853163	5.442080346	0.60617896	1
ASV_16s_472	10.86092841	9.801822817	2.438344426	6.634757083	0.467872456	1
ASV_16s_473	11.45679476	8.540581596	1.698144926	12.64049878	0.081367315	0.450778647
ASV_16s_474	13.5667232	-0.034673394	1.337267162	29.80275412	1.03E-04	0.001654688
ASV_16s_475	11.00748661	9.786703541	1.478603512	16.54466892	0.020580324	0.157176542
ASV_16s_476	12.98854273	0.877118384	1.411399302	20.93751866	0.003863734	0.039302944
ASV_16s_477	7.989452003	-0.958808896	2.498347451	5.239750973	NA	NA
ASV_16s_478	11.39213787	7.393104717	1.943744262	10.452799	0.164332488	0.735395476
ASV_16s_479	11.64602864	7.622383759	1.300089799	24.83864235	8.10E-04	0.010061742
ASV_16s_480	6.213895416	-20.15241128	3.551456506	3.172023425	0.868645658	1
ASV_16s_481	12.4137296	6.173600802	1.53531185	9.199437717	0.238652695	0.955141038
ASV_16s_482	10.84433079	3.335028992	1.215133917	23.27330168	0.001527453	0.017693605
ASV_16s_483	12.46094884	-30	2.742850797	7.261233708	0.402196601	1
ASV_16s_484	12.19837264	-2.408663408	1.702884349	11.78721364	0.107777202	0.549323381
ASV_16s_485	11.9280937	3.246668671	1.757584622	10.49145728	0.162390904	0.72839759
ASV_16s_486	11.59002926	0.820365064	1.433739303	23.32932228	0.001493427	0.017382628
ASV_16s_487	15.50769899	0.477713997	2.18155799	19.34276553	0.007178756	0.063570108
ASV_16s_488	10.55049016	-11.67905295	3.54853585	4.299108775	0.744753124	1
ASV_16s_489	3.449152873	-23.95244417	3.550587458	2.077171058	NA	NA
ASV_16s_490	16.30525758	0.87949389	1.64990677	16.22066726	0.023175029	0.174244548
ASV_16s_491	4.505639111	-23.18267357	3.551439733	3.189756663	0.866910832	1
ASV_16s_492	10.64531039	10.11815225	1.861927093	10.87774576	0.144041306	0.673682217
ASV_16s_493	13.92022473	0.258765376	2.14517148	16.85655308	0.018344942	0.141894906
ASV_16s_494	10.16015974	9.208290615	2.46312072	5.209110117	0.634461321	1
ASV_16s_495	10.57565226	-0.977238368	1.556266093	6.237369935	0.512322583	1
ASV_16s_496	3.377690509	-25.25119744	3.550587824	2.065614827	NA	NA
ASV_16s_497	15.06961833	0.901258642	1.967827425	23.00678493	0.001699977	0.019413411
ASV_16s_498	11.25262256	-2.94222615	1.914530199	12.95172855	0.073293287	0.421479922
ASV_16s_499	11.74657816	3.508325182	1.052654977	21.8203757	0.002727977	0.029272552
ASV_16s_500	17.23614904	1.693961109	1.478875858	40.22968385	1.14E-06	2.75E-05
ASV_16s_501	5.647688761	-30	3.55033691	3.050814425	0.880262008	1
ASV_16s_502	11.48956678	-2.699874712	1.545157274	14.95261002	0.036611696	0.250366464
ASV_16s_503	13.81419205	-0.603765882	3.549158979	4.985175428	0.661772436	1
ASV_16s_504	11.1922574	10.61087356	1.523902862	13.35598528	0.063895482	0.385763
ASV_16s_505	15.99558254	-11.653244	3.552157344	2.569166025	NA	NA
ASV_16s_506	12.29094013	-0.556146245	1.984699747	9.359099956	0.227891787	0.928831678
ASV_16s_507	11.63328739	1.606291685	1.811079721	17.29164941	0.015609336	0.124720138
ASV_16s_508	12.74307262	-1.024619134	1.866083787	9.635237296	0.210203555	0.87128452
ASV_16s_509	9.56299137	9.625756168	2.128400668	8.369315753	0.30115674	1
ASV_16s_510	6.526215591	-10.24862431	3.547449423	2.278888322	0.9428075	1
ASV_16s_511	9.541074082	-8.102789299	1.966602896	3.543391114	0.830608404	1
ASV_16s_512	17.42618514	28.32555168	3.549045479	2.594650633	0.919802762	1
ASV_16s_513	13.35447994	6.327943614	1.548774797	8.114561816	0.322600508	1
ASV_16s_514	4.190592927	18.86646021	3.551439244	2.340125153	NA	NA
ASV_16s_515	10.26521261	-0.99762808	1.50612914	9.753969462	0.202949779	0.853023291
ASV_16s_516	10.23762763	4.552752402	2.14510876	7.478086289	0.380852223	1
ASV_16s_517	14.32596939	-4.716171203	2.508014793	7.802069572	0.350370511	1
ASV_16s_518	4.793469515	1.439191893	3.55071622	3.954304884	NA	NA
ASV_16s_519	11.6408213	-6.730568429	1.776643494	17.50529378	0.014412992	0.117487724
ASV_16s_520	12.7229443	-0.511117266	1.369555184	19.69751878	0.006261464	0.057420468
ASV_16s_521	7.721643584	-1.180703241	2.792185558	4.362067091	0.73725669	1
ASV_16s_522	10.52215335	-8.385076116	3.547133363	1.945521416	NA	NA
ASV_16s_523	10.34194229	9.866296094	2.782417378	4.920916592	0.669613675	1
ASV_16s_524	10.58614376	0.23057469	1.585503919	20.86348243	0.003977545	0.040123484
ASV_16s_525	9.3458789	-2.751702221	2.051520324	10.60252398	0.156920938	0.712768465
ASV_16s_526	13.34488719	0.739290383	2.357444038	13.20471311	0.067274923	0.39919752
ASV_16s_527	10.12817354	9.288158157	2.118433476	8.466537858	0.293255994	1
ASV_16s_528	10.40582722	8.437193812	1.602138956	14.72016635	0.03975852	0.263434898
ASV_16s_529	9.405334884	11.30277169	3.17407048	4.802760781	0.684018691	1
ASV_16s_530	7.183632215	-13.17250722	3.54713314	2.124929911	NA	NA
ASV_16s_531	12.21283747	2.907655693	1.375032888	11.30100193	0.126017724	0.611400623
ASV_16s_532	10.74545406	10.87041932	1.993050884	7.84486956	0.346471846	1
ASV_16s_533	10.94307556	-0.308375447	1.483910691	18.79486116	0.008854833	0.074956474
ASV_16s_534	13.05115847	3.204492444	3.548081521	5.242495831	0.630396967	1
ASV_16s_535	10.66036665	6.600721437	2.432260316	9.086624197	0.246496494	0.964516234

ASV_16s_536	10.96796508	5.265243807	1.580983412	12.91844467	0.07412058	0.425227307
ASV_16s_537	11.94992753	-4.453283382	1.696311451	13.04172191	0.071098346	0.41279216
ASV_16s_538	3.461260515	0.813007335	3.550773739	3.276854904	0.858264754	1
ASV_16s_539	2.638046981	-4.221725846	3.550772733	3.689506512	0.814764616	1
ASV_16s_540	9.097990444	10.36717073	2.780840977	4.861280456	0.676887269	1
ASV_16s_541	12.41651058	2.773615763	1.65767846	9.624915064	0.210844059	0.87128452
ASV_16s_542	10.75268898	0.082467167	1.711622069	12.54927803	0.083881888	0.461541025
ASV_16s_543	10.03567051	-30	3.548310742	1.506219905	NA	NA
ASV_16s_544	13.28262137	4.56631752	3.420545241	5.725661849	0.572122442	1
ASV_16s_545	8.737169487	11.43805159	3.54628115	3.785572767	0.804117484	1
ASV_16s_546	10.15751446	-5.049889816	2.020362765	8.138330488	0.320554346	1
ASV_16s_547	3.874811642	-10.13612062	3.552157344	2.382724381	0.93566618	1
ASV_16s_548	2.526885372	-7.064293425	3.551355802	2.678133387	0.913097198	1
ASV_16s_549	10.83817305	-26.78380727	2.000802595	10.16324365	0.179509809	0.780239225
ASV_16s_550	9.003654509	-2.317926602	1.780172902	13.87420022	0.053464599	0.334416596
ASV_16s_551	9.156442794	24.61013682	3.252568676	3.994867005	0.780368441	1
ASV_16s_552	11.2032653	8.79514589	1.81914929	10.86473494	0.144629014	0.673745093
ASV_16s_553	12.77622383	-0.105053741	3.549157083	4.895788061	0.672679087	1
ASV_16s_554	12.77552491	-0.14302098	3.549181731	4.761803447	0.689004961	1
ASV_16s_555	9.127156626	5.8526163	3.11665172	4.975480582	0.662955587	1
ASV_16s_556	10.12376308	-0.512904085	1.494374878	26.25311248	4.54E-04	0.006314261
ASV_16s_557	9.090086589	-5.955721689	1.701922202	6.180646404	0.518821866	1
ASV_16s_558	11.05556592	-5.569931273	2.099235218	11.38021202	0.122872764	0.602176034
ASV_16s_559	11.7542897	-0.726517918	1.845019241	5.370453239	0.614852097	1
ASV_16s_560	12.26674735	0.663039191	2.261691606	12.68345441	0.080206984	0.44639335
ASV_16s_561	8.899978176	9.675533883	3.260052643	3.620528908	0.82229922	1
ASV_16s_562	9.971773569	6.832254394	1.636235655	13.40538068	0.062826117	0.383128535
ASV_16s_563	11.06456176	0.830932868	2.055721357	13.36146588	0.063776015	0.385763
ASV_16s_564	6.492751064	-5.389555911	2.15974097	3.219485348	0.863982913	1
ASV_16s_565	11.90715681	-0.191471504	2.104107349	17.46304034	0.014642427	0.118559581
ASV_16s_566	2.944541079	-23.48255728	3.550589674	1.989897442	NA	NA
ASV_16s_567	3.317885173	-12.65725705	3.549484695	3.384004064	0.847354601	1
ASV_16s_568	5.702994188	-15.1746007	3.552157344	2.61984609	0.917805484	1
ASV_16s_569	9.977249346	7.693295386	2.058819084	7.208948844	0.407453426	1
ASV_16s_570	11.72376072	-4.224801368	2.748171393	4.924393152	0.669189519	1
ASV_16s_571	8.206328897	1.60133605	1.851975059	10.65919558	0.154191042	0.703006614
ASV_16s_572	2.886204455	-23.50663126	3.550590023	1.978856108	NA	NA
ASV_16s_573	10.14529494	10.67439293	2.093976612	6.661962924	0.464903568	1
ASV_16s_574	8.804476382	8.724673897	3.545620726	3.322670112	0.853635674	1
ASV_16s_575	11.8114345	1.439899141	2.574239483	10.6897256	0.152737318	0.700762408
ASV_16s_576	8.328100125	0.471012789	1.116708304	26.44027219	4.20E-04	0.00587893
ASV_16s_577	9.069464996	1.965169271	1.992226161	8.068016921	0.32663449	1
ASV_16s_578	3.744135251	-7.355992744	3.550322884	1.672421913	0.975733675	1
ASV_16s_579	10.16824752	-3.229868709	1.204499379	11.2348727	0.128697055	0.623151142
ASV_16s_580	8.994001988	10.78930144	1.902408385	7.82893392	0.34791991	1
ASV_16s_581	6.455068043	-10.4485621	3.546729272	2.244767638	NA	NA
ASV_16s_582	10.61907559	9.444456258	3.112424311	1.596731081	0.978770672	1
ASV_16s_583	8.805621358	8.601118008	2.94580874	4.324514894	0.741732427	1
ASV_16s_584	8.691701254	8.746391403	3.200073789	4.059636284	0.772883427	1
ASV_16s_585	10.02736943	-0.849112242	1.757526837	16.99543124	0.017425667	0.135216471
ASV_16s_586	7.516574818	2.151947457	1.411192864	15.71774153	0.027824001	0.199886966
ASV_16s_587	10.10502349	-30	2.274090804	10.26405519	0.174096994	0.766343316
ASV_16s_588	10.84371994	-3.629315813	1.353742492	13.18916565	0.06763136	0.40033135
ASV_16s_589	3.223210789	-1.43161667	3.550723068	4.509742076	NA	NA
ASV_16s_590	11.29184846	0.065411119	3.031249209	5.679250235	0.577660085	1
ASV_16s_591	12.53388463	5.307337232	1.256608928	6.380746509	0.496059818	1
ASV_16s_592	6.050567893	23.9945398	3.548675055	1.923911397	NA	NA
ASV_16s_593	14.2266873	14.38175804	3.549150663	3.853713846	0.796464207	1
ASV_16s_594	11.65567406	5.750261285	3.545349957	1.997745674	NA	NA
ASV_16s_595	1.489271936	-23.75038644	3.551438862	1.811453293	NA	NA
ASV_16s_596	5.822759335	2.529682619	3.224108439	3.302261877	0.855704428	1
ASV_16s_597	7.974252993	-4.764920393	2.225531989	4.315249465	0.742834762	1
ASV_16s_598	9.952098	-9.297027474	1.964914742	9.162797808	0.241178258	0.960445384
ASV_16s_599	10.75362802	-1.087549737	1.618090098	24.35538208	9.86E-04	0.011882115
ASV_16s_600	9.77893936	-30	3.098152432	4.505930818	0.720002986	1
ASV_16s_601	2.756405467	-23.44336815	3.550590707	1.953470381	NA	NA
ASV_16s_603	8.008250564	0.75937592	1.426393414	23.7879291	0.001241359	0.014660153

ASV_16s_604	11.744279	0.037327298	2.787775668	6.61701888	0.469813543	1
ASV_16s_605	8.782062147	6.148069732	3.546362787	3.358602204	0.849967411	1
ASV_16s_606	8.648020998	-11.80920324	3.548504754	4.161549215	0.760993484	1
ASV_16s_607	7.083978972	3.806557958	1.971333147	4.846149913	0.678731808	1
ASV_16s_608	8.438373609	5.459021268	2.397688591	6.261371829	0.509583402	1
ASV_16s_609	12.39854817	0.848242022	1.788456937	6.708950717	0.459799676	1
ASV_16s_610	8.172248848	-6.908257134	3.54926238	4.515508074	NA	NA
ASV_16s_611	8.639741234	4.767700406	3.475845499	3.816880626	0.800611152	1
ASV_16s_612	11.72855361	-29.26582508	3.549059199	2.527590542	0.925005179	1
ASV_16s_613	7.426209065	-1.434935373	1.529345163	16.2510052	0.022919508	0.172860213
ASV_16s_614	11.72816137	15.45068781	2.453053958	2.435295999	0.931890026	1
ASV_16s_615	10.55633882	4.118153818	1.101423674	17.47173321	0.014594941	0.118559581
ASV_16s_616	11.10971927	0.445739896	2.075671902	21.86840708	0.002676543	0.02905789
ASV_16s_617	7.587329699	8.700745278	3.542908851	1.937514283	0.963217641	1
ASV_16s_618	12.63749697	30	3.548301231	2.631506056	NA	NA
ASV_16s_619	5.487797643	-5.733903768	3.550577038	0.911481023	0.99613295	1
ASV_16s_620	8.787979025	7.007377812	1.763478458	7.899073391	0.341577662	1
ASV_16s_621	14.49184437	21.19611102	3.549361557	3.495662514	NA	NA
ASV_16s_622	11.0177377	18.80627217	2.119363149	5.027900631	0.656558238	1
ASV_16s_623	8.61082247	-11.87828532	3.548524706	3.860573393	0.79568937	1
ASV_16s_624	9.799849936	-27.05278005	3.151741909	4.778483775	0.686974811	1
ASV_16s_625	7.317649712	0.820470148	2.995194073	3.729742666	0.810326272	1
ASV_16s_626	2.128968799	-24.54390982	3.551438457	2.030821681	NA	NA
ASV_16s_627	9.34776423	-0.37540706	1.615951889	10.6169337	0.156222928	0.710931784
ASV_16s_628	8.893582	-0.061744846	1.942222101	7.934275975	0.338425082	1
ASV_16s_629	8.227717691	6.150133651	3.114138177	4.59258929	0.709541186	1
ASV_16s_630	11.4676028	0.783736945	2.501639586	11.592129	0.114795173	0.571850026
ASV_16s_631	10.34169247	-0.184296288	1.564253542	19.11928447	0.007821768	0.067389681
ASV_16s_632	2.953230532	14.58867364	3.551477683	2.690073079	0.91211779	1
ASV_16s_633	8.74294219	-7.135163913	2.396012971	3.618580842	0.82251061	1
ASV_16s_634	6.899010295	6.382651045	2.317099869	4.315221344	0.742838106	1
ASV_16s_635	7.425126643	0.152101524	1.722443955	9.191405754	0.23920453	0.95563394
ASV_16s_636	7.809602662	-9.641528941	3.54920884	4.173930127	0.759540338	1
ASV_16s_637	7.623558961	-3.538545417	1.812719576	8.510316396	0.289749203	1
ASV_16s_638	7.985914865	10.30333038	2.151509671	7.20751678	0.407598005	1
ASV_16s_639	11.98607304	-9.12714136	3.548451767	2.791680262	0.903581627	1
ASV_16s_640	4.115695192	-10.78660876	3.550291668	3.605206161	0.823959803	1
ASV_16s_641	10.42925262	0.353363905	1.798780139	12.9893646	0.072367945	0.418145095
ASV_16s_642	9.019611105	1.030957283	1.464523942	25.86909728	5.32E-04	0.007159013
ASV_16s_643	10.07567891	0.924046285	1.238573002	32.62068455	3.11E-05	5.71E-04
ASV_16s_644	2.074705495	-23.85803647	3.551438485	2.014966756	NA	NA
ASV_16s_645	12.30396647	0.603470972	3.550161135	2.82452978	NA	NA
ASV_16s_646	5.673622561	19.06523206	3.551438811	2.526008823	NA	NA
ASV_16s_647	8.813075025	1.855029041	1.057733804	25.01710961	7.54E-04	0.009501403
ASV_16s_648	7.476224507	-29.33895984	2.266253633	4.50302563	0.720352906	1
ASV_16s_649	11.00379248	1.468969551	2.520779285	10.21374247	0.176780997	0.771147377
ASV_16s_650	9.598591462	-1.016134324	2.716522621	4.58365275	0.710622066	1
ASV_16s_651	1.780670069	1.983910371	3.546756177	1.038864734	0.994176995	NA
ASV_16s_652	11.15137017	2.574354445	2.263690527	11.72694916	0.109903458	0.554325566
ASV_16s_653	8.533086378	1.617942672	1.281683208	3.037557531	0.881506241	1
ASV_16s_654	8.052186232	8.523723109	2.681802467	5.10786603	0.64680162	1
ASV_16s_655	11.56854256	-25.39817815	3.549013803	2.130921286	NA	NA
ASV_16s_656	7.442818409	2.113614631	2.127942082	6.745499931	0.455850674	1
ASV_16s_657	8.250915076	0.284522156	2.717870466	5.575183398	0.590131502	1
ASV_16s_658	8.310706384	-2.120225227	1.212264631	7.1778065	0.410604653	1
ASV_16s_659	6.136834255	-0.221790377	3.546242071	1.409357414	0.985282931	1
ASV_16s_660	7.980295855	3.188975374	1.702121588	11.36543873	0.123454049	0.603802533
ASV_16s_661	9.20112292	-30	2.612832541	4.903883346	0.671691629	1
ASV_16s_662	5.029768176	-24.30782988	3.551177299	2.196665646	0.948158203	1
ASV_16s_663	7.802683653	7.345897196	3.546440017	3.430866597	0.842492857	1
ASV_16s_664	8.410723855	-1.484613948	2.11104608	8.758538683	0.270460042	1
ASV_16s_665	6.995922085	2.251480952	1.555611661	12.13182759	0.096306143	0.515834453
ASV_16s_666	2.549310453	-23.47811506	3.550592036	1.910381093	NA	NA
ASV_16s_667	2.406029839	-24.05783283	3.551438348	2.105939051	0.953744738	1
ASV_16s_668	10.46942837	0.863739526	1.980650544	10.55507538	0.159238215	0.72059013
ASV_16s_669	2.539101544	-23.64506164	3.550592102	1.908167547	NA	NA
ASV_16s_670	7.632537991	22.90547313	3.549076105	3.11121767	0.87452654	1

ASV_16s_671	8.04865488	9.128486156	1.669088691	12.72924621	0.078986555	0.443680855
ASV_16s_672	2.55890732	-23.9132068	3.551439092	3.421282634	0.843491461	1
ASV_16s_673	8.52399902	-1.433436957	2.011398182	10.12175519	0.181778076	0.788682297
ASV_16s_674	9.772877195	-0.371146498	1.559990104	14.76818675	0.039088416	0.262425576
ASV_16s_675	10.404345	0.215876992	2.181580458	14.89823887	0.037326183	0.253127981
ASV_16s_676	6.600146416	-0.269784578	1.981982248	6.413093584	0.492424799	1
ASV_16s_677	11.1313252	27.48046659	2.891101279	2.122526129	0.952748483	1
ASV_16s_678	10.72288791	1.244330417	2.879430181	7.342291734	0.394131239	1
ASV_16s_679	9.239380763	3.156875784	1.428267338	14.25689773	0.046796337	0.298141923
ASV_16s_680	2.647753607	-1.585309297	3.550738748	3.903817408	0.790786782	1
ASV_16s_681	6.787095064	-6.590389458	1.904990686	3.743712931	0.808778056	1
ASV_16s_682	7.95535587	-4.370717395	1.7609383	9.666290314	0.208286252	0.864941709
ASV_16s_683	9.473911786	-0.906362189	2.741952054	3.878574944	0.793652241	1
ASV_16s_684	11.00858752	0.421884966	2.715125956	5.421532951	0.608664575	1
ASV_16s_685	8.875584609	0.26883004	1.231113301	30.23931401	8.58E-05	0.001417389
ASV_16s_686	11.90038087	-8.155903303	3.552157344	2.380062773	NA	NA
ASV_16s_687	2.531589164	-30	3.550316075	2.822704939	NA	NA
ASV_16s_688	8.64891721	6.795900925	1.71768762	5.941395923	0.54660873	1
ASV_16s_689	7.596302342	-13.26463653	3.546499613	2.739924894	NA	NA
ASV_16s_690	2.063788553	-2.185283719	3.035523071	2.294625486	0.94175255	1
ASV_16s_691	9.442478404	23.56502344	3.344654751	2.831357998	0.90015237	1
ASV_16s_692	8.290980623	7.452314216	1.760767897	6.445602987	0.488784568	1
ASV_16s_693	7.361945226	10.4964844	3.228801437	3.688459397	0.814879704	1
ASV_16s_694	2.070726949	-3.19159367	3.55074429	2.619659483	NA	NA
ASV_16s_695	8.23102138	-1.340205666	1.624980852	8.210676798	0.314383882	1
ASV_16s_696	4.055998984	0.186652871	2.711835817	4.22348783	0.753706217	1
ASV_16s_697	5.066538841	-30	3.550640582	2.219290704	0.94671297	1
ASV_16s_698	10.11801847	0.682784214	2.294301313	12.84534635	0.07596736	0.43074494
ASV_16s_699	8.951644153	-1.423153162	1.499484434	21.85404495	0.002691822	0.029093313
ASV_16s_700	9.386148511	-0.036821223	1.993360931	13.52248464	0.060356182	0.370868823
ASV_16s_701	6.44865683	-2.4650542	2.127954666	6.940011603	0.435152483	1
ASV_16s_702	8.78338679	6.486505817	1.752390899	11.48378757	0.11886441	0.588488215
ASV_16s_703	7.42443443	11.25470245	3.503202848	3.741480101	0.809025749	1
ASV_16s_704	7.297336668	2.500538513	1.656379473	6.90654583	0.438674793	1
ASV_16s_705	9.665414691	29.95812224	1.793041531	7.500596085	0.378679474	1
ASV_16s_706	7.557386041	3.214721528	1.66879007	13.09053151	0.06993309	0.411813304
ASV_16s_707	8.216672436	2.408776682	1.399456172	10.36911042	0.168603265	0.74623127
ASV_16s_708	8.231361352	1.821857467	1.470756405	16.10203936	0.02420029	0.180273546
ASV_16s_709	11.72580229	-25.64295709	3.549013571	2.14181103	NA	NA
ASV_16s_710	9.823884964	0.151265232	2.097980849	15.48235345	0.030290133	0.214721581
ASV_16s_711	7.022671031	6.307721022	3.547371663	3.66460662	0.817495497	1
ASV_16s_712	9.883974673	5.607884251	1.482428637	9.162450334	0.24120231	0.960445384
ASV_16s_713	7.378638677	-0.029766728	1.459350075	11.47708248	0.119120372	0.588551877
ASV_16s_714	2.018832361	-23.89417711	3.551438696	2.769123955	NA	NA
ASV_16s_715	9.830044929	0.09166916	1.825255005	21.80238973	0.002747483	0.029302452
ASV_16s_716	10.36431624	6.239991414	2.362695551	12.60371762	0.082372902	0.455307754
ASV_16s_717	7.212883182	10.29951738	3.546736328	3.620904933	0.822258408	1
ASV_16s_718	7.255862724	5.896757344	2.870969453	4.857673807	0.677326988	1
ASV_16s_719	7.72940262	9.001210673	1.933215527	8.982507347	0.25391439	0.988306654
ASV_16s_720	9.746094755	0.160305408	1.931027107	19.6629612	0.00634559	0.057972352
ASV_16s_721	12.51051886	-1.086619167	3.122295795	4.91475607	0.670365258	1
ASV_16s_722	7.470443993	8.213645527	1.972068472	6.523690083	0.480095251	1
ASV_16s_723	13.75398784	30	3.548332504	2.823771022	NA	NA
ASV_16s_724	9.752831129	1.51943791	2.185923246	12.48696493	0.085639714	0.469080875
ASV_16s_725	9.480790813	30	1.870679068	6.846093107	0.445078755	1
ASV_16s_726	2.068314091	-22.9416683	3.551440059	2.47205039	NA	NA
ASV_16s_727	1.99150582	-26.48620775	3.550874281	2.535773003	NA	NA
ASV_16s_728	7.267991201	0.035813843	1.502295232	18.2923462	0.010718365	0.089172374
ASV_16s_729	15.48700549	18.95409804	3.550579124	2.787893427	NA	NA
ASV_16s_730	10.99411496	3.347442099	3.546753452	3.619851102	0.82237278	1
ASV_16s_731	2.216964405	-5.303120793	3.550846121	3.019449381	0.883197191	1
ASV_16s_732	10.93925767	-23.04434141	3.551050613	2.426429271	NA	NA
ASV_16s_733	6.289684858	1.338765321	1.980125619	7.545422522	0.374376876	1
ASV_16s_734	6.980240036	-0.020742985	3.546483904	3.839082219	0.798114313	1
ASV_16s_735	8.257749944	-24.71203193	2.655148032	4.121128	0.765724951	1
ASV_16s_736	8.473593388	-30	2.726176481	5.578218926	0.589766722	1
ASV_16s_737	2.20946408	-24.3345658	3.551438423	2.053599511	NA	NA

ASV_16s_738	7.028330977	9.247854817	1.643095164	13.3079851	0.064950594	0.389708047
ASV_16s_739	7.374333612	5.207921161	1.998285881	11.02576317	0.137499185	0.651415906
ASV_16s_740	2.599770463	0.158773527	3.550573501	4.70969946	0.695340757	1
ASV_16s_741	6.545913831	-9.814699057	3.549178717	2.950622444	NA	NA
ASV_16s_742	7.989183149	-0.831595812	1.864152628	5.731181065	0.571464977	1
ASV_16s_743	2.227915683	0.062933626	3.550546149	5.569847881	0.590772811	1
ASV_16s_744	7.557517944	10.12632247	2.330677355	5.800987218	0.563169919	1
ASV_16s_745	8.649694819	0.214523893	1.990957892	10.55982085	0.159005151	0.72059013
ASV_16s_746	9.651640803	5.370712411	1.41804487	8.500771375	0.290511099	1
ASV_16s_747	9.658550734	-0.757086334	3.545902182	3.304214451	0.855506964	1
ASV_16s_748	5.409327655	-3.528962862	2.978100471	3.770925104	0.805751953	1
ASV_16s_749	7.900356484	-4.987500742	1.916492992	12.36203861	0.089264184	0.483506725
ASV_16s_750	8.266816086	-0.475251677	1.423067736	24.96062348	7.71E-04	0.009672623
ASV_16s_751	6.917942373	9.500297172	2.698035266	4.877383936	0.674923685	1
ASV_16s_752	10.13255893	0.662250612	2.204846735	11.70335354	0.110746031	0.556995447
ASV_16s_753	7.307534927	-9.172960794	2.079794784	5.05363799	0.65341748	1
ASV_16s_754	6.753446477	-3.755074326	3.048726976	3.840814032	0.797919194	1
ASV_16s_755	8.256471848	-30	3.549158083	2.546176212	0.923579998	1
ASV_16s_756	9.682697311	-10.73604582	3.552157344	2.625473906	0.917356211	1
ASV_16s_757	8.347409443	6.467505717	3.439973213	2.257752833	0.944208802	1
ASV_16s_758	2.817903795	-13.32336681	3.552157344	2.187248067	NA	NA
ASV_16s_759	6.730987134	9.204578387	3.547781326	3.783576181	0.804340502	1
ASV_16s_760	2.269321167	-12.11942661	3.549705926	1.334220674	NA	NA
ASV_16s_761	7.545431634	6.173706317	2.022832708	5.159594794	0.640494174	1
ASV_16s_762	6.798727747	11.05550806	3.134077993	4.462663664	0.725208411	1
ASV_16s_763	7.527178395	9.713268561	2.071396682	7.579620168	0.371116253	1
ASV_16s_764	7.133733793	11.69493139	2.176187839	8.086418571	0.325035357	1
ASV_16s_765	4.340676148	-12.81059345	3.552157344	2.452347549	0.930642393	1
ASV_16s_766	7.393326078	10.04278477	2.05944484	7.322570613	0.396084018	1
ASV_16s_767	2.499091644	8.877219927	3.550185423	3.294707051	0.856467512	1
ASV_16s_768	3.162518315	-6.196139412	3.549969116	1.353423479	0.986955342	1
ASV_16s_769	8.846079992	-1.115976525	2.204955158	13.36431333	0.063714027	0.385763
ASV_16s_770	2.905995575	15.12454334	3.551442881	1.597853755	NA	NA
ASV_16s_771	7.913258026	0.223732279	1.649672843	20.69973563	0.004240928	0.042252206
ASV_16s_772	10.15273518	-1.696733786	1.989843212	6.997458151	NA	NA
ASV_16s_773	6.340908796	-3.43447537	3.545175917	2.233323787	NA	NA
ASV_16s_774	7.532570395	-8.010337915	2.265955351	5.379516016	0.613753445	1
ASV_16s_775	7.520879604	3.067836132	1.727622381	7.007405655	0.42810919	1
ASV_16s_776	5.678694978	17.05486808	3.549009076	2.031158724	0.958095674	1
ASV_16s_777	6.089185755	0.464414287	1.763609129	6.595812352	0.472139694	1
ASV_16s_778	7.561917036	0.675311517	2.19734492	10.28288219	0.173101503	0.764742224
ASV_16s_779	7.911441718	-30	3.089867517	4.226262416	0.753378777	1
ASV_16s_780	7.319558056	-6.758976671	2.345492922	6.033516582	0.53584129	1
ASV_16s_781	6.469899501	-9.418942465	3.549256313	3.728364735	0.810478779	1
ASV_16s_782	7.321402994	4.805579274	1.872811674	7.261142716	0.402205713	1
ASV_16s_783	6.051723659	3.718441404	3.54683261	3.656971295	0.818330432	1
ASV_16s_784	8.592446521	-7.522958136	3.438334497	1.955523299	0.962260945	1
ASV_16s_785	2.898041792	-10.73418869	3.547981015	2.341042353	0.938583829	1
ASV_16s_786	9.217750938	-1.13799684	1.872644448	16.18167609	0.023507393	0.175652465
ASV_16s_787	2.180331309	-23.11922972	3.550595007	1.824135056	NA	NA
ASV_16s_788	9.055347105	0.211361524	2.029775702	14.54790503	0.042251555	0.274867208
ASV_16s_789	10.83661805	8.766326326	2.560412607	8.229214529	0.312816737	1
ASV_16s_790	6.597370885	-6.923178808	3.09391458	4.32392096	0.741803113	1
ASV_16s_791	6.572893223	1.500030276	2.43623955	3.509548332	0.834213101	1
ASV_16s_792	7.58427634	-9.27673168	1.976437428	5.862205816	0.555928042	1
ASV_16s_793	7.054095513	8.103115211	1.787672606	9.2687877	0.233929966	0.948650667
ASV_16s_794	7.868304194	0.434083822	1.737281448	8.653713817	0.27848304	1
ASV_16s_795	4.191416442	-14.01575632	3.552157344	2.43087425	0.932211738	1
ASV_16s_796	6.464163728	-9.007928472	3.549233082	3.726279771	0.810709471	1
ASV_16s_797	9.106190942	-5.725721776	2.573590251	4.257032567	0.749741967	1
ASV_16s_798	6.419939638	1.850492872	1.960576608	5.14318131	0.64249505	1
ASV_16s_799	2.054316759	-24.46701064	3.551438493	2.008890747	NA	NA
ASV_16s_800	1.9541754	-2.758935206	3.549977724	1.858899105	NA	NA
ASV_16s_801	6.150507822	5.884248975	2.728087583	5.842288869	0.558280644	1
ASV_16s_802	9.32676077	-0.011165674	1.86094145	12.71009114	0.079495008	0.444115414
ASV_16s_803	1.156754137	-23.16211601	3.551439253	1.656390076	NA	NA
ASV_16s_804	10.54539313	1.707118463	2.271072723	10.67420177	0.15347503	0.702387612

ASV_16s_805	10.40164406	-13.88850478	3.552157344	2.638230724	NA	NA
ASV_16s_806	6.69900918	1.792973423	1.187026235	24.7687593	8.34E-04	0.010299594
ASV_16s_807	6.158000482	1.437485148	1.873754512	11.1810314	0.130915135	0.627614935
ASV_16s_808	8.060842564	1.113052761	2.577435081	5.489876536	0.600405232	1
ASV_16s_809	8.803434548	4.246158253	2.322729093	5.194259466	0.636270129	1
ASV_16s_810	9.292203426	-5.629899105	2.359412039	7.647682232	0.36468313	1
ASV_16s_811	6.522648171	-2.305174831	2.615413941	5.982674416	0.54177383	1
ASV_16s_812	7.528660595	0.037222135	2.239745474	8.157791342	0.318885995	1
ASV_16s_813	9.152161697	0.220232753	3.42395386	2.935533934	0.890901111	1
ASV_16s_814	7.571297572	0.896469107	1.63770533	14.90740492	0.037204826	0.253013718
ASV_16s_815	6.629283373	6.091360895	1.790179321	9.330580527	0.22978493	0.934523719
ASV_16s_816	8.877567398	-0.764904681	1.321631759	29.06173062	1.41E-04	0.002174031
ASV_16s_817	2.371376954	0.335326478	3.550589244	4.584290302	0.710544968	1
ASV_16s_818	8.218611127	0.229219628	1.940994677	11.87422739	0.104771573	0.539685062
ASV_16s_819	7.56796102	-28.2860914	2.818213626	4.335700974	0.740400525	1
ASV_16s_820	6.272731556	-9.312030392	3.54920621	3.971207036	0.78308777	1
ASV_16s_821	6.605205702	9.155252948	2.307016564	6.320002555	0.502920235	1
ASV_16s_822	8.062838986	-0.655987929	2.026104242	11.96234746	0.101803912	0.529097687
ASV_16s_823	7.347422803	-5.733954496	2.106084384	11.21899513	0.129347722	0.623806443
ASV_16s_824	8.13357986	3.514453023	1.970737248	8.30814052	0.306208148	1
ASV_16s_825	7.226474006	1.681727887	2.293017771	9.717967948	0.205127236	0.854755659
ASV_16s_826	7.798077435	-19.41140685	2.811748478	3.041640117	0.881123632	1
ASV_16s_827	5.2407506	2.490604204	2.000797027	7.009562812	0.42788486	1
ASV_16s_828	3.401155861	18.7323211	3.551439625	2.212024428	0.947179375	1
ASV_16s_829	6.323558764	-11.27434674	3.548594475	3.576456202	0.827062234	1
ASV_16s_830	6.446493821	4.992450502	2.358238705	5.413839522	0.609595764	1
ASV_16s_831	10.16814639	1.054601353	2.192785407	16.48719258	0.021019545	0.15952451
ASV_16s_832	8.301201449	2.808475086	2.446944027	7.265270713	0.401792481	1
ASV_16s_833	9.018986603	-20.15296822	3.552157344	2.586292458	0.920460213	1
ASV_16s_834	7.318998066	3.616353414	1.739320098	5.707006291	0.574346441	1
ASV_16s_835	6.585610762	0.897365695	1.702256289	17.36288477	0.01520028	0.122258733
ASV_16s_836	7.491671895	-0.209842675	1.811188487	13.82334577	0.054414017	0.337785474
ASV_16s_837	8.04787955	-1.268667451	1.61616323	14.32690434	0.045663299	0.29169089
ASV_16s_838	7.274975047	4.057342214	1.30359108	17.23580409	0.015937339	0.126922031
ASV_16s_839	8.899811189	-0.233800269	2.184239613	9.03802976	0.249937189	0.974392809
ASV_16s_840	2.148054303	-25.23551196	3.549519716	1.522236736	NA	NA
ASV_16s_841	7.242992851	-1.814364395	1.760312409	14.11594197	0.049157222	0.310730118
ASV_16s_842	4.18096654	-13.02706928	3.552157344	1.924255562	0.963913346	1
ASV_16s_843	7.831452216	20.28619943	3.550150675	2.650073403	0.915379006	1
ASV_16s_844	5.572892839	9.195935635	1.979582372	7.468800415	0.381750904	1
ASV_16s_845	7.213943639	0.825276769	1.232825814	10.81576469	0.146859612	0.678525039
ASV_16s_846	8.907088315	0.496071319	2.130113765	14.86692249	0.037743606	0.255243772
ASV_16s_847	6.661919363	10.32910373	2.066807863	6.893585818	0.440043237	1
ASV_16s_848	6.176079842	1.026054554	1.774775907	14.56377961	0.042015858	0.274867208
ASV_16s_849	6.728779745	4.824926871	1.594650743	15.96086439	0.025476324	0.187440675
ASV_16s_850	9.523855445	-24.13046595	3.552157344	2.04561264	NA	NA
ASV_16s_851	2.44221579	-22.6062625	3.551440886	2.523748591	0.92529818	1
ASV_16s_852	6.065102904	9.878983381	3.547617596	3.570919628	0.82765767	1
ASV_16s_853	6.312406884	10.05009518	1.921636351	7.688841235	0.360829379	1
ASV_16s_854	5.236932172	1.999277753	2.620210798	3.927513374	0.788087615	1
ASV_16s_855	1.905925034	0.022790624	3.550551302	3.950248804	0.785489636	NA
ASV_16s_856	7.915138344	2.399151616	1.292584537	15.3611152	0.031639195	0.222024614
ASV_16s_857	7.60758716	-0.24772998	1.943269339	8.31530068	0.305613717	1
ASV_16s_858	4.228024027	-26.9302288	3.54951909	1.919195271	0.964176933	1
ASV_16s_859	7.858176449	-0.707432085	2.154316006	13.07712071	0.070251505	0.411813304
ASV_16s_860	6.432275104	1.535383128	1.716795943	15.72530571	0.027747981	0.199886966
ASV_16s_861	6.828487562	5.132489942	1.781409294	13.36095623	0.063787116	0.385763
ASV_16s_862	6.502266545	-7.50049607	3.549809216	1.567634883	0.979875342	1
ASV_16s_863	5.085694052	2.587460135	2.030711328	6.424202547	0.491179398	1
ASV_16s_864	9.495540923	1.281208119	1.77718229	21.50895683	0.003085555	0.032060639
ASV_16s_865	5.96938298	10.20607147	3.547023157	3.32122808	0.853782203	1
ASV_16s_866	7.561042161	8.401092092	3.335210276	2.685240613	0.912514803	1
ASV_16s_867	1.872448315	-23.68016496	3.551439001	2.909727109	0.893225694	NA
ASV_16s_868	5.078092921	4.9779859	3.539620524	2.942591121	0.890261725	1
ASV_16s_869	0.192405096	0.845470819	3.551280309	0.607431288	0.998950359	NA
ASV_16s_870	6.97607776	-0.584523598	2.434616413	7.99137245	0.333355288	1
ASV_16s_871	6.266489946	-7.17817832	3.5498633	2.180799335	0.949159352	1

ASV_16s_872	6.448017677	-2.861269835	1.657435363	9.151552939	0.241957589	0.961181421
ASV_16s_873	5.658458627	1.771471245	2.307839494	4.078873254	0.770649266	1
ASV_16s_874	2.553258962	18.55014555	3.551440275	2.036131443	NA	NA
ASV_16s_875	6.040227477	11.36525034	2.854992982	4.985839122	0.661691439	1
ASV_16s_876	6.657553935	-2.820094063	1.844221852	12.70561515	0.079614246	0.444115414
ASV_16s_877	6.614447267	5.336329995	2.670250371	5.141134866	0.642744553	1
ASV_16s_878	5.463874804	2.400751806	2.455729712	5.104409803	0.647223189	1
ASV_16s_879	5.147333572	7.340693879	3.018685509	1.924703616	0.963889955	1
ASV_16s_880	6.490245006	1.235097631	1.653180065	11.3255225	0.125036709	0.607859181
ASV_16s_881	4.594359869	-0.316542065	2.576858022	4.306588679	0.743864428	1
ASV_16s_882	3.879635984	-24.11029627	3.550982472	2.142926116	0.951507774	1
ASV_16s_883	1.510310243	0.737892815	3.547600617	1.885193761	0.965920335	NA
ASV_16s_884	3.144769695	-21.52548059	3.551443663	2.416829169	0.933228631	1
ASV_16s_885	3.127991831	-20.49702339	3.551445857	2.246466783	0.944949762	1
ASV_16s_886	6.323343046	10.0519848	3.233437314	3.20715843	0.865199924	1
ASV_16s_887	7.051548957	-2.442108906	1.621523937	12.09192068	0.097576241	0.519090802
ASV_16s_888	6.205416982	6.403432192	1.320736947	15.47847706	0.030332417	0.214721581
ASV_16s_889	7.184168543	2.487999562	2.285654166	3.047933586	0.880532841	1
ASV_16s_890	3.964233076	-9.362523121	3.547782358	2.401585181	0.93432372	1
ASV_16s_891	4.790505106	9.445167209	3.500409713	2.11139343	0.953418381	1
ASV_16s_892	6.081862552	9.337974144	2.130431898	7.317095145	0.396627282	1
ASV_16s_893	6.187277466	0.911807238	2.457508594	3.680533847	0.815750096	1
ASV_16s_894	1.923650165	-22.42596876	3.55059769	1.755050619	NA	NA
ASV_16s_895	6.348645447	0.187393501	3.549390143	2.93893908	0.8905928	1
ASV_16s_896	8.04171169	-9.670303324	3.15222446	2.668657089	0.913870938	1
ASV_16s_897	7.095862008	-1.027546126	1.43340613	18.41861497	0.010217497	0.086190103
ASV_16s_898	5.702194277	10.00111164	3.54713891	3.322921057	0.85361017	1
ASV_16s_899	6.36492535	9.364702384	1.722685237	9.747268502	0.203353626	0.853239389
ASV_16s_900	4.186693314	-1.495440718	1.931301095	3.407907861	0.844881359	1
ASV_16s_901	1.910524425	-22.93626362	3.550597884	1.751274046	NA	NA
ASV_16s_902	1.827556076	-5.272139995	3.550890622	2.929546719	0.891442315	NA
ASV_16s_903	6.46169703	-1.971306897	1.963363919	9.201027536	0.238543587	0.955141038
ASV_16s_904	5.641780332	9.097456999	1.960142046	8.199911555	0.315296568	1
ASV_16s_905	6.483088545	8.726133541	2.488995516	3.421797437	0.843437877	1
ASV_16s_906	7.422203017	8.83164328	3.380409101	2.272168046	0.943254994	1
ASV_16s_907	6.68980692	9.2945253	1.546921457	12.0708043	0.098254342	0.520511512
ASV_16s_908	6.333084597	-2.180202736	1.665309958	4.200417882	0.756425528	1
ASV_16s_909	1.893023437	-22.83617857	3.550598077	1.746198872	NA	NA
ASV_16s_910	5.761384371	11.5151877	2.881585478	4.551398847	0.714519473	1
ASV_16s_911	6.644282758	5.180511836	2.135138883	7.355766586	0.39280049	1
ASV_16s_912	7.16023489	4.427304384	1.222553047	10.23909522	0.175424201	0.767996367
ASV_16s_913	9.237644882	25.61232665	3.549059977	2.32498507	NA	NA
ASV_16s_914	5.546542811	-11.52631601	2.786432882	3.859041198	0.795862511	1
ASV_16s_915	6.024549762	1.28461639	1.64994926	19.98167199	0.005609407	0.053258234
ASV_16s_916	1.075114593	-23.45933392	3.550811101	0.161352125	NA	NA
ASV_16s_917	6.211492996	9.388788362	2.247726258	5.44388992	0.605960155	1
ASV_16s_918	3.990678498	17.60096803	3.551441076	2.351444388	NA	NA
ASV_16s_919	5.492041426	-0.545771105	1.905592052	12.17037226	0.095093463	0.511602833
ASV_16s_920	3.984476978	17.59967657	3.551441081	2.350490013	NA	NA
ASV_16s_921	5.260455533	1.466711271	1.994935199	13.08349509	0.070099993	0.411813304
ASV_16s_922	5.732749724	6.38398463	3.451424781	3.049862235	0.880351553	1
ASV_16s_923	5.812215144	8.62879715	2.291495253	6.170360067	0.520004273	1
ASV_16s_924	6.261204841	-1.447282608	2.049186537	10.25136859	0.174770524	0.76652072
ASV_16s_925	6.395063966	-1.342254159	2.363485943	7.668155197	0.36276278	1
ASV_16s_926	8.821840869	-17.08187859	3.552157344	2.034093112	NA	NA
ASV_16s_927	6.142304395	4.59610835	2.019853013	6.024939679	0.536840308	1
ASV_16s_928	5.672318208	8.885506635	3.156453871	3.185215501	0.867355926	1
ASV_16s_929	5.971898072	2.63124015	1.780837839	8.023689977	0.330509581	1
ASV_16s_930	5.820617885	-11.23492819	3.54862086	3.253001435	0.860653019	1
ASV_16s_931	7.429177549	0.653224003	1.512529153	22.25909669	0.002291805	0.025220268
ASV_16s_932	5.221790025	0.158822435	1.711520855	6.4510513	0.488175785	1
ASV_16s_933	7.225788231	6.025688716	1.68938856	4.111853453	0.766807742	1
ASV_16s_934	7.139471912	-4.399451986	3.549632594	2.713720874	0.910163134	1
ASV_16s_935	6.109287874	-1.645119911	1.439748375	15.31960072	0.032113977	0.224057459
ASV_16s_936	6.378984336	-11.20587989	2.597490284	4.669185763	0.700260256	1
ASV_16s_937	7.196255917	-0.361040903	2.094381715	7.89348712	0.342079822	1
ASV_16s_938	4.851407722	3.38111848	1.854743857	7.099415937	0.418602706	1

ASV_16s_939	7.39707626	-0.040933383	1.899225259	10.66606617	0.153862861	0.702833938
ASV_16s_940	3.130414518	0.840051764	2.616375045	4.644772106	0.70322141	1
ASV_16s_941	3.341985078	0.026354757	1.668822718	8.587600006	0.283635456	1
ASV_16s_942	6.547847483	-30	2.713193743	5.172056102	0.638975422	1
ASV_16s_943	5.842140408	7.89132023	2.084598101	6.305288052	0.504588708	1
ASV_16s_944	6.653009525	4.138559561	1.574029658	12.7995162	0.07714644	0.435364875
ASV_16s_945	6.944456214	-1.451224035	1.228391666	25.1614901	7.10E-04	0.009051527
ASV_16s_946	7.140503078	-10.8798067	3.552157344	2.23876231	0.945452649	1
ASV_16s_947	5.673233951	3.319466709	1.348035099	14.47297206	0.043380834	0.278580897
ASV_16s_948	7.869787666	2.977158868	2.618269256	6.263651382	0.509323593	1
ASV_16s_949	6.659654521	2.009076977	1.306255424	29.88026842	9.99E-05	0.001612118
ASV_16s_950	7.099033906	-15.90505576	3.552157344	1.642458771	0.976964211	1
ASV_16s_951	3.650122034	0.951801937	1.911497103	12.80953314	0.076887323	0.434916377
ASV_16s_952	6.170836367	-25.29750039	3.549027954	1.85027126	0.967660689	1
ASV_16s_953	6.345714955	-3.057246539	1.876147272	5.522845731	0.596429692	1
ASV_16s_955	6.750666946	5.972929835	2.040431396	7.047676183	0.423932786	1
ASV_16s_956	8.157782192	16.79501277	3.549589391	2.749666364	NA	NA
ASV_16s_957	5.953056762	11.40543771	2.436324308	6.816145882	0.448270629	1
ASV_16s_958	7.549090923	1.587449503	2.793207504	7.461154644	0.382491894	1
ASV_16s_959	5.478597611	11.14650469	2.486876866	5.840628289	0.558476946	1
ASV_16s_960	5.398614241	-10.8661727	3.548714294	3.359541798	0.849871051	1
ASV_16s_961	5.99605441	-0.473288911	1.551699311	20.74758343	0.004162265	0.041639853
ASV_16s_962	2.948014095	0.071026746	3.549214159	0.734139912	NA	NA
ASV_16s_963	4.991405681	11.20979852	3.547304364	2.817682787	0.901340263	1
ASV_16s_964	6.008850721	-1.04502423	2.018449539	6.28643726	0.506729907	1
ASV_16s_965	6.017548282	-3.033327658	1.457476687	8.965426894	0.2551478	0.99151336
ASV_16s_966	6.675993884	0.355442566	2.24685195	14.38549926	0.044734603	0.286514478
ASV_16s_967	5.534306532	7.057225524	3.432530016	3.13417513	0.872318634	1
ASV_16s_968	5.293473143	1.634274201	2.331385318	2.076983299	0.955456809	1
ASV_16s_969	1.758392653	10.07435599	3.549346474	2.215756517	NA	NA
ASV_16s_970	6.035121442	-3.364921746	2.33052323	3.829255657	0.799220475	1
ASV_16s_971	6.168963171	1.236140631	1.880892946	14.51824176	0.042695282	0.275640743
ASV_16s_972	1.391218765	-23.82285913	3.551438957	1.76962132	NA	NA
ASV_16s_973	7.344307188	3.694139508	3.550744318	5.271780654	0.626834421	1
ASV_16s_974	7.582279539	8.245603344	2.583883835	6.480239096	0.484920777	1
ASV_16s_975	5.977306919	7.786811856	2.257053939	5.263246827	0.627872309	1
ASV_16s_976	5.296712848	-9.062204678	3.549195009	3.858342925	0.795941404	1
ASV_16s_977	5.725796651	5.639921497	2.548018281	6.764689555	0.453784754	1
ASV_16s_978	5.523781212	-3.100260336	2.555944336	5.821958679	0.560685544	1
ASV_16s_979	5.424166328	7.517072781	2.349201865	4.225179037	0.753506641	1
ASV_16s_980	5.732642816	-11.60165786	3.54852357	3.349477881	0.850901997	1
ASV_16s_981	8.631597448	-5.337052575	3.549580358	3.772743695	0.805549235	1
ASV_16s_982	0.142133901	0.077675893	3.551026924	0.675411997	NA	NA
ASV_16s_983	3.379643345	-9.052486779	3.552157344	2.298767997	0.941473219	1
ASV_16s_984	5.164413529	9.300614705	3.547799906	3.103010016	0.875312191	1
ASV_16s_985	5.227324958	9.796627442	2.564560157	4.25346006	0.750164722	1
ASV_16s_986	7.418812393	4.37515022	1.855108671	12.89742615	0.074647374	0.426229462
ASV_16s_987	1.885646463	0.086907721	3.550563621	3.846723055	0.797253057	NA
ASV_16s_988	5.927612321	6.870629659	2.926587059	3.168665146	0.868973202	1
ASV_16s_989	5.545611587	4.628864604	2.273251111	5.755843343	0.568529983	1
ASV_16s_990	5.065883571	1.280716145	1.375556607	25.38947098	6.47E-04	0.00839082
ASV_16s_991	3.950465792	0.436430719	3.357323225	1.428389369	NA	NA
ASV_16s_992	6.907812423	-9.996792022	3.550022633	5.157209255	0.640784951	1
ASV_16s_993	6.898556008	-0.629619001	2.285630098	13.31763844	0.064737127	0.389708047
ASV_16s_994	4.965170226	-26.87758078	3.550588882	3.083438437	0.877177655	1
ASV_16s_995	5.562713001	9.433086685	2.241654302	5.729767039	0.571633398	1
ASV_16s_996	4.638599509	-0.622427369	2.309222856	8.555111847	0.28619358	1
ASV_16s_997	6.605578018	0.479352444	1.349451726	19.89621231	0.005798279	0.053990899
ASV_16s_998	6.69764454	0.862974151	2.927379219	4.553377439	0.714280563	1
ASV_16s_999	1.413782429	-23.37197269	3.551439478	1.911190319	NA	NA
ASV_16s_1000	6.506426829	11.17035516	2.972396548	4.159481851	0.761235955	1
ASV_16s_1001	5.290945157	5.175090236	2.58678699	5.843608874	0.558124619	1
ASV_16s_1002	6.830749714	5.746954247	2.678883639	4.901597686	0.671970441	1
ASV_16s_1003	6.753348173	-9.785230209	3.548281462	1.204883736	NA	NA
ASV_16s_1004	1.852625339	-23.46071875	3.55143944	2.364469171	0.936952361	NA
ASV_16s_1005	5.159266943	10.15977023	2.68847876	4.21512939	0.754692124	1
ASV_16s_1006	5.991130928	-1.014713767	2.504664571	5.866297414	0.555445165	1

ASV_16s_1007	5.437638534	3.880658796	1.49824789	9.952839894	0.19126178	0.816657443
ASV_16s_1008	6.055081	-3.338371839	1.984293877	8.370587857	0.301052355	1
ASV_16s_1009	7.331716069	8.466747209	2.94611638	4.784008352	0.686302244	1
ASV_16s_1010	7.182763334	2.556666669	3.54815317	4.419906493	0.730339181	1
ASV_16s_1011	2.183111953	18.45128564	3.551440722	1.940023522	NA	NA
ASV_16s_1012	1.044958918	-23.31348735	3.551439437	0.3001291	NA	NA
ASV_16s_1013	5.267171438	4.771857256	2.028403159	10.92615323	0.141872776	0.668237336
ASV_16s_1014	6.005194662	-29.99430068	2.53812185	4.99146247	0.661005162	1
ASV_16s_1015	6.621518506	-5.603990282	2.429770497	6.236784852	0.512389437	1
ASV_16s_1016	3.155242562	18.68787749	3.551439775	2.165985717	0.950084857	1
ASV_16s_1017	7.016884834	1.372895112	2.99418381	5.560534175	0.591892701	1
ASV_16s_1018	6.505449344	0.334850498	2.535772554	4.868444076	0.676013823	1
ASV_16s_1019	5.317488516	0.350168764	2.419542214	4.486822461	0.722303486	1
ASV_16s_1020	4.798590617	6.830835994	1.907906202	3.241396324	0.86180945	1
ASV_16s_1021	5.676085724	-0.309901504	1.853426101	12.40246282	0.08807653	0.481339231
ASV_16s_1022	5.35815965	-0.727380639	3.552157344	1.572549585	NA	NA
ASV_16s_1023	5.568399754	-5.729873582	2.065844644	9.738197502	0.203901358	0.854057419
ASV_16s_1024	6.598030896	1.004711694	1.818704884	15.79780333	0.02702924	0.195921527
ASV_16s_1025	5.384992167	9.359560188	1.901439192	7.708624671	0.358986868	1
ASV_16s_1026	5.708267185	-1.16639138	2.23168923	6.548607141	0.477339035	1
ASV_16s_1027	5.041687546	-3.681075058	1.93409041	3.006999939	0.884353925	1
ASV_16s_1028	5.977854805	-30	3.074424668	3.868467925	0.794796652	1
ASV_16s_1029	5.929303469	-30	3.549881479	2.872763475	0.896517815	1
ASV_16s_1030	6.076602146	2.261842158	1.821317975	8.249040234	0.311147005	1
ASV_16s_1031	6.154137779	-0.045197013	2.634367582	6.782579204	0.451863438	1
ASV_16s_1032	6.458052747	-5.384289643	2.718128693	3.951107678	0.785391337	1
ASV_16s_1033	4.296241309	-0.086283212	1.581130409	17.35155941	0.015264623	0.122369709
ASV_16s_1034	4.780311545	3.77095424	2.210830976	5.063172165	0.652254112	1
ASV_16s_1035	4.964209005	7.880436322	3.546550845	2.649731905	0.915406603	1
ASV_16s_1036	4.987732125	7.02615669	2.084102067	5.998921897	0.539875241	1
ASV_16s_1037	6.146566171	9.711488687	3.01050135	3.427648586	0.842828404	1
ASV_16s_1038	5.84119282	-2.892234978	2.265424483	4.287417417	0.7461411	1
ASV_16s_1039	6.353804559	2.135599295	1.584021336	7.923539719	0.339384405	1
ASV_16s_1040	4.790197402	-7.948864029	3.438422276	0.792419272	NA	NA
ASV_16s_1041	6.710862704	-24.27435797	3.55072921	3.248388067	NA	NA
ASV_16s_1042	4.411654897	18.27324814	3.550727716	3.43803966	NA	NA
ASV_16s_1043	5.040848331	11.43082462	2.907592204	3.811791533	0.801182281	1
ASV_16s_1044	5.709022671	-9.233345288	2.790507758	4.626789389	0.705400794	1
ASV_16s_1045	2.361056313	-2.292963017	3.550785813	3.031655483	0.882058472	1
ASV_16s_1046	5.354460687	-9.794283156	2.748555692	4.2086689	0.755453636	1
ASV_16s_1047	5.04215385	9.205170924	2.347854552	4.814625829	0.68257338	1
ASV_16s_1048	1.623216553	-22.8583552	3.550601981	1.661400376	NA	NA
ASV_16s_1049	6.517930784	-0.152797758	2.035577339	7.288443212	0.399477744	1
ASV_16s_1050	5.298407147	0.85459373	1.78047436	13.86259337	0.053679934	0.334416596
ASV_16s_1051	5.634778026	-1.050018176	2.401586573	5.974655817	0.542711775	1
ASV_16s_1052	1.929775982	-20.59844958	3.551451388	1.649923475	NA	NA
ASV_16s_1053	4.794252809	8.493108175	3.54795809	3.356901345	0.850141785	1
ASV_16s_1054	5.23579184	9.778187633	2.145555466	5.610444647	0.585897739	1
ASV_16s_1055	3.224058898	-8.585403065	3.552157344	2.269855623	0.943408558	1
ASV_16s_1056	5.950044078	-30	2.768704733	4.368682026	0.736466977	1
ASV_16s_1057	6.382396156	-1.131261965	2.141973232	5.401269744	0.611117758	1
ASV_16s_1058	1.098985236	0.648344977	3.549944945	1.8820086	0.966081179	NA
ASV_16s_1059	4.831353497	-8.92189799	3.549204672	3.706045257	0.812944034	1
ASV_16s_1060	3.229251287	0.267284547	3.550111682	1.7328647	0.973137983	1
ASV_16s_1061	6.27382758	1.634574115	2.83430483	4.653409068	0.702174131	1
ASV_16s_1062	5.138164359	9.434487444	2.274275151	4.644918057	0.703203716	1
ASV_16s_1063	7.222276023	-10.34211365	3.552157344	2.455239237	NA	NA
ASV_16s_1064	7.458500861	2.199486042	2.05865039	10.18409391	0.178378867	0.776718052
ASV_16s_1065	4.898386433	11.40317303	3.545686358	2.840056149	0.89939356	1
ASV_16s_1066	5.039491843	11.0556903	2.281435543	6.52822099	0.479593462	1
ASV_16s_1067	5.578040465	0.66159333	1.611612203	9.696693964	0.206422915	0.858676764
ASV_16s_1068	5.393561137	4.738225664	1.795853373	13.30431929	0.065031826	0.389708047
ASV_16s_1069	5.034934169	11.79291834	2.505223855	5.360197558	0.616095774	1
ASV_16s_1070	0.990675338	-23.34095722	3.551439542	0.267403087	NA	NA
ASV_16s_1071	3.158699854	-30	3.55092242	1.786168643	0.970722581	1
ASV_16s_1072	1.336444431	-8.729459128	3.552157344	1.486056287	NA	NA
ASV_16s_1073	5.154998435	-11.31280811	3.548624352	2.955698257	0.889070023	1

ASV_16s_1074	3.222371539	-12.16199669	3.552157344	2.269504073	0.943431885	1
ASV_16s_1075	6.563946465	5.237716624	2.475796906	5.611854175	0.585728665	1
ASV_16s_1076	1.404623167	-0.086807762	3.550535993	3.547543908	0.830164331	NA
ASV_16s_1077	5.520651196	6.286339445	2.537673157	6.566872757	0.475323719	1
ASV_16s_1078	6.1750294	3.090058525	1.544897921	11.92742942	0.102970791	0.532675821
ASV_16s_1079	6.156538867	-1.926283038	1.975748573	15.32485765	0.032053488	0.224057459
ASV_16s_1080	2.669224202	-0.298938872	3.550794276	2.456885482	0.930308488	1
ASV_16s_1081	6.735723368	-3.902430049	2.65171688	3.563917926	0.82840973	1
ASV_16s_1082	4.440655465	11.02136239	3.546416387	2.92374162	0.891965964	1
ASV_16s_1083	4.548129719	2.252598072	1.684295253	10.02901301	0.18693528	0.803223968
ASV_16s_1084	4.771600152	9.49429901	3.547386053	3.216076732	0.864319856	1
ASV_16s_1085	4.632138724	-1.470586989	2.200667668	3.724882197	0.81086406	1
ASV_16s_1086	5.497238653	-0.158592791	2.070973925	13.98981334	0.05136283	0.323826592
ASV_16s_1087	5.699756523	-1.596270122	1.607228785	10.68776199	0.152830463	0.700762408
ASV_16s_1088	5.765546019	-30	3.074991525	3.810823937	0.801290819	1
ASV_16s_1089	6.024106199	0.669008358	2.610340084	6.430919922	0.49042707	1
ASV_16s_1090	6.037873301	-22.34581356	3.096513385	2.329441455	0.939383752	1
ASV_16s_1091	5.556249065	-0.303861932	1.98327809	8.333691045	0.304090847	1
ASV_16s_1092	5.755418659	-0.247759617	1.868494888	14.76764098	0.039095972	0.262425576
ASV_16s_1093	4.141684354	-12.76670968	3.546432236	2.674770195	0.913372168	1
ASV_16s_1094	6.544647017	-30	3.546830441	1.347840528	NA	NA
ASV_16s_1095	1.131207928	-24.17382148	3.55143929	0.348768865	0.999833682	NA
ASV_16s_1096	4.461560605	3.136760499	3.014349318	0.46898423	0.99955231	1
ASV_16s_1097	5.736108005	-1.323475227	2.368416374	1.400079975	0.985568815	1
ASV_16s_1098	5.407700252	-10.88311346	2.385553568	6.275748949	0.507945795	1
ASV_16s_1099	5.471649111	0.150597074	2.372279973	11.1875731	0.130643872	0.627557169
ASV_16s_1100	4.642550707	-8.797779019	3.549233657	3.548260177	0.830087699	1
ASV_16s_1101	4.680282591	1.669367544	3.072086452	5.733050481	0.57124234	1
ASV_16s_1102	6.557607611	4.440419485	3.550809939	5.047553912	0.654159896	1
ASV_16s_1103	5.669380985	-30	3.075798894	3.774020168	0.805406911	1
ASV_16s_1104	5.974157641	3.08807074	2.123690383	9.245261095	0.235523714	0.949152858
ASV_16s_1105	6.061226327	1.976117317	2.764246476	6.686879827	0.462193288	1
ASV_16s_1106	5.529594324	-0.11667365	1.971249238	10.83333543	0.146055878	0.676918169
ASV_16s_1107	6.897293812	0.407941341	2.521791004	12.30198534	0.091055131	0.492063551
ASV_16s_1108	5.39899236	-1.489348374	1.668788827	13.28760467	0.06540339	0.390254648
ASV_16s_1109	3.283705151	17.43116726	3.551441835	2.231680171	NA	NA
ASV_16s_1110	5.69490969	-29.9951614	2.56448068	4.836926389	0.679856017	1
ASV_16s_1111	4.503886588	10.76004332	3.546387103	2.816928093	0.901405637	1
ASV_16s_1112	1.45312089	-23.69576517	3.551438897	1.796324309	NA	NA
ASV_16s_1113	1.978345874	18.38247994	3.551441035	1.879600786	0.966202487	NA
ASV_16s_1114	4.622392989	11.20509865	3.546398303	2.997032659	0.885276599	1
ASV_16s_1115	3.447418515	-8.015625696	2.802805332	2.773209637	0.90515984	1
ASV_16s_1116	1.536730902	-23.23197169	3.54971275	1.366204043	NA	NA
ASV_16s_1117	5.390152602	-3.219241454	2.65295249	2.310995344	0.940644755	1
ASV_16s_1118	6.545716189	7.561101648	2.400877317	6.425776477	0.491003072	1
ASV_16s_1119	0.937996401	-0.170447516	3.550534443	1.586909725	0.979147433	NA
ASV_16s_1120	4.732955037	8.297772547	2.082277322	5.963527349	0.5440145	1
ASV_16s_1121	4.834509209	-10.8878677	3.548602418	3.332956386	0.852588911	1
ASV_16s_1122	4.436149756	-10.51765838	3.548674563	3.342793046	0.85158538	1
ASV_16s_1123	5.41617599	-0.925506445	2.643502919	5.894713439	0.552095647	1
ASV_16s_1124	4.387885128	9.58043549	3.547568331	3.100659312	0.875536842	1
ASV_16s_1125	6.887104106	4.713950315	1.320905223	11.65932429	0.112333567	0.562399495
ASV_16s_1126	5.227365146	1.736054051	1.781369143	6.220015112	0.51430725	1
ASV_16s_1127	4.886309381	4.158590275	2.38584585	8.201704038	0.315144466	1
ASV_16s_1128	1.721493236	-23.0312413	3.551439919	2.282637027	0.942557098	NA
ASV_16s_1129	5.002699144	3.941274082	2.041871451	10.41901548	0.166045347	0.739891661
ASV_16s_1130	6.157808888	1.682869365	3.131449223	5.408135356	0.610286356	1
ASV_16s_1131	1.863481776	-11.97510717	3.552157344	1.933462386	NA	NA
ASV_16s_1132	4.684155607	-1.971183082	1.840645312	5.723333835	0.572399831	1
ASV_16s_1133	7.085437305	-9.431959589	3.552157344	2.453154282	NA	NA
ASV_16s_1134	4.835982099	30	3.547830358	1.943222283	NA	NA
ASV_16s_1135	6.574175146	5.51144254	2.032333931	4.053134828	0.77363738	1
ASV_16s_1136	4.261402737	1.325990669	1.743796523	5.869323238	0.55508816	1
ASV_16s_1137	4.470261502	-8.963306366	3.549242062	3.360845953	0.849737268	1
ASV_16s_1138	4.435205183	11.37381229	3.509698068	2.771834712	0.905276854	1
ASV_16s_1139	0.469266533	2.454787886	3.548576241	0.316609992	0.999879971	NA
ASV_16s_1140	5.596513099	0.587945705	2.733368354	4.509774064	0.719539994	1

ASV_16s_1141	7.127076268	1.189540228	3.549015726	3.34591423	NA	NA
ASV_16s_1142	4.549279303	10.82568976	3.546902433	3.05958163	0.879436255	1
ASV_16s_1143	1.380988119	7.472514573	3.550174136	1.166693062	NA	NA
ASV_16s_1144	5.303662426	-5.774286859	2.93731732	3.051983382	0.880152041	1
ASV_16s_1145	5.333624076	-30	3.071095742	3.702191927	0.813368681	1
ASV_16s_1146	6.134237265	-0.547163571	2.376138805	8.791383174	0.267982985	1
ASV_16s_1147	4.325911938	-8.773343253	3.549263435	3.322908872	0.853611408	1
ASV_16s_1148	5.384030059	0.859711237	1.466126467	11.22305752	0.129180971	0.623806443
ASV_16s_1149	4.534201037	9.460832844	2.796131547	4.199157652	0.756573905	1
ASV_16s_1150	4.694690525	3.82937437	2.22798243	8.421430908	0.296902216	1
ASV_16s_1151	2.875623283	-23.10909781	3.551450767	1.893690068	0.965489217	1
ASV_16s_1152	3.063317005	15.28594128	3.550849539	2.505632297	0.926672351	1
ASV_16s_1153	4.556440143	-7.635830741	2.567028076	4.990234201	0.661155061	1
ASV_16s_1154	5.539737845	9.31544546	3.547670967	4.002698176	NA	NA
ASV_16s_1155	2.891944262	-29.92610756	3.55132116	1.616534681	0.977998916	1
ASV_16s_1156	5.882895265	-0.675905948	2.876047005	6.797310919	0.450284665	1
ASV_16s_1157	4.823180754	2.642038926	2.523299523	7.375222675	0.390884107	1
ASV_16s_1158	5.843730599	-26.10289679	3.551524097	1.788447241	0.970616686	1
ASV_16s_1159	2.361322095	-1.992825666	3.550903792	2.085263033	0.954970772	1
ASV_16s_1160	5.460051772	7.924455477	3.163047677	1.16822465	0.99164021	1
ASV_16s_1161	4.39198037	4.895793123	3.443987781	2.875858211	0.896243892	1
ASV_16s_1162	4.75903059	8.701230502	2.634128619	4.30073615	0.744559819	1
ASV_16s_1163	3.664112814	9.99392652	3.408634774	1.510679006	0.981937649	1
ASV_16s_1164	5.8684545	1.180752194	1.433325413	18.85110617	0.008666784	0.073622048
ASV_16s_1165	5.126488131	-7.685593584	2.340465358	5.737923711	0.570662088	1
ASV_16s_1166	3.842174422	1.368281301	2.33537964	7.523925403	0.376436209	1
ASV_16s_1167	4.137908444	17.85217453	3.549887513	1.933585183	0.963424571	1
ASV_16s_1168	4.804485355	9.389310459	3.203677332	2.884996951	0.895433173	1
ASV_16s_1169	4.524725735	9.074222176	2.34997486	4.680907113	0.698837641	1
ASV_16s_1170	1.347732491	-7.203115471	3.552157344	1.734710167	NA	NA
ASV_16s_1171	6.223751923	0.08863382	2.854288053	4.838017278	0.679723063	1
ASV_16s_1172	4.030585829	8.299331456	2.499822662	4.635666713	0.704325114	1
ASV_16s_1173	4.883834396	-0.008922889	2.443438409	8.928357526	0.257840711	0.998771776
ASV_16s_1174	1.426330448	-22.16507969	3.550605707	1.590103461	NA	NA
ASV_16s_1175	2.586218586	-14.17969863	3.552157344	2.134562747	NA	NA
ASV_16s_1176	4.554909266	2.018791743	2.168349391	7.62416792	0.366897168	1
ASV_16s_1177	4.674113971	3.262500677	2.544761952	6.271138719	0.508470658	1
ASV_16s_1178	4.481838061	0.294482345	3.549455291	2.170385916	0.949810879	1
ASV_16s_1179	2.971787688	-5.692293999	3.550705199	1.58690103	0.979147765	1
ASV_16s_1180	4.618487923	12.35444764	3.543181176	2.542397662	0.923870784	1
ASV_16s_1181	4.407895247	0.242313986	3.549583233	1.605011629	0.978449938	1
ASV_16s_1182	1.131138657	-23.4582937	3.551439294	0.348724742	0.999833753	NA
ASV_16s_1183	4.439392138	-10.68730835	3.548646912	3.171617847	0.868685232	1
ASV_16s_1184	5.55563816	-4.884584072	2.416181714	5.35366388	0.616888319	1
ASV_16s_1185	3.762474569	1.435467246	2.514188385	3.345867123	0.851271261	1
ASV_16s_1186	5.109756562	-0.874101175	2.410342456	3.031019602	0.882117906	1
ASV_16s_1187	4.127478723	10.3051878	2.706214794	3.419541863	0.843672605	1
ASV_16s_1188	5.540355818	7.060874108	3.440750965	2.596111294	0.919687605	1
ASV_16s_1189	5.306088812	-0.033796177	1.573455781	19.19245329	0.007605363	0.0661597
ASV_16s_1190	4.647929063	0.166621451	2.868032048	2.955434223	0.889094083	1
ASV_16s_1191	3.837403669	13.78418079	3.551334612	1.903482677	0.964988583	1
ASV_16s_1192	0.873965641	-23.00779993	3.551439811	0.190520935	NA	NA
ASV_16s_1193	4.164910462	-5.305248132	3.54819454	3.307555607	0.855168844	1
ASV_16s_1194	4.445634716	-10.77115659	3.548635007	3.066696806	0.87876441	1
ASV_16s_1195	0.75679169	-23.13964176	3.551440174	0.102268053	NA	NA
ASV_16s_1196	4.568170803	9.160299555	3.514936713	2.378023676	0.935998612	1
ASV_16s_1197	5.111084906	-0.93497885	2.691747216	5.130869421	0.643996226	1
ASV_16s_1198	3.55911435	5.240828346	3.546393593	2.333849461	NA	NA
ASV_16s_1199	5.106790871	1.475535348	2.526260207	7.881162576	0.343189516	1
ASV_16s_1200	2.743559833	-8.147227351	3.552157344	2.170806614	0.949784643	1
ASV_16s_1201	5.507076499	6.12996835	3.457283413	2.922252299	0.892100134	1
ASV_16s_1202	3.323988999	17.70831132	3.548073777	1.383044084	NA	NA
ASV_16s_1203	4.650671751	-3.972446262	1.951340092	3.361165845	0.849704446	1
ASV_16s_1204	4.456476164	3.553625445	1.904139174	11.34727832	0.124171911	0.606089103
ASV_16s_1205	3.951166541	1.42393384	1.690111654	8.007420704	0.331940007	1
ASV_16s_1206	4.512376947	-11.00647623	3.548602169	2.98119403	0.886736468	1
ASV_16s_1207	4.315697597	0.084615242	1.642370453	16.42634597	0.021494205	0.162617091

ASV_16s_1208	4.33209772	-15.9273108	3.546268769	0.56497125	0.999172102	1
ASV_16s_1209	1.353107612	-30	3.549945037	0.743025474	0.997982868	NA
ASV_16s_1210	2.884994393	-20.8359173	3.551088751	0.972266026	NA	NA
ASV_16s_1211	5.285459798	1.32378079	2.54211714	5.936627631	0.547168265	1
ASV_16s_1212	1.310676709	-12.94327835	3.552157344	0.450096445	NA	NA
ASV_16s_1213	2.017467885	-22.40407306	3.551442595	2.193478672	0.948360117	1
ASV_16s_1214	4.643971681	-7.610135674	3.15440121	2.883107057	0.895601053	1
ASV_16s_1215	3.072600199	0.482804747	2.710068355	8.71308399	0.273917038	1
ASV_16s_1216	4.675755975	0.470070745	1.980242976	10.03428759	0.186638734	0.803223968
ASV_16s_1217	7.510305074	4.017799161	2.393219762	5.02612438	0.656775007	1
ASV_16s_1218	2.94882304	17.37754164	3.551442286	0.90740224	NA	NA
ASV_16s_1219	1.314596497	-23.71381683	3.551439043	0.440944816	NA	NA
ASV_16s_1220	5.073817736	-0.799691991	3.244195578	4.344606912	0.739339297	1
ASV_16s_1221	4.706551195	4.433734303	1.982300215	6.733925944	0.457099193	1
ASV_16s_1222	4.722837229	6.486726581	2.103960648	4.373319604	0.735913103	1
ASV_16s_1223	4.904602921	-0.931750711	2.651627154	5.572067772	0.590505967	1
ASV_16s_1224	4.779902632	-1.207356779	2.132544974	5.516512277	0.597192931	1
ASV_16s_1225	4.615204652	6.298077923	2.73097503	5.62986106	0.583569878	1
ASV_16s_1226	4.651049045	-0.924698502	1.476975669	10.90763351	0.142699057	0.670824112
ASV_16s_1227	4.630564983	-1.163238464	1.846084003	7.952056023	0.336840557	1
ASV_16s_1228	4.41363814	2.118691068	1.80201358	3.162308852	0.869592283	1
ASV_16s_1229	1.309055521	-23.46443124	3.551439043	0.43835296	NA	NA
ASV_16s_1230	4.307900419	-0.402134046	1.731377932	6.745333261	0.45586864	1
ASV_16s_1231	5.764257052	1.155994292	3.1517146	5.647507097	0.581456461	1
ASV_16s_1232	5.288845413	2.850863249	2.070547347	12.87255181	0.075275203	0.428802981
ASV_16s_1233	4.966173787	-30	3.071683706	3.564003066	0.828400591	1
ASV_16s_1234	4.536647345	-25.63516333	3.549044772	0.43665074	0.999646977	1
ASV_16s_1235	5.045835355	-1.393034889	2.375497598	8.389919862	0.299469323	1
ASV_16s_1236	0.735617207	-22.6144539	3.551440244	0.084871541	NA	NA
ASV_16s_1237	3.561905312	3.09255865	2.817913115	4.846311719	0.678712085	1
ASV_16s_1238	5.453213741	-0.8094731	2.502278309	5.741064089	0.570288262	1
ASV_16s_1239	5.107373993	-0.187929009	2.988744801	4.687690205	0.698014118	1
ASV_16s_1240	3.902852476	-8.817641135	3.549206484	3.237317075	NA	NA
ASV_16s_1241	5.436801829	1.294838827	2.002306513	13.52826629	0.060236578	0.370868823
ASV_16s_1242	6.121516416	15.66660944	3.55064495	2.911193353	0.893094192	1
ASV_16s_1243	5.775523854	8.240985346	2.94753527	3.230453477	0.862896558	1
ASV_16s_1244	4.416728275	-10.76703876	3.548568069	3.023231077	0.882844878	1
ASV_16s_1245	5.701686204	3.035387551	1.561999832	10.02572512	0.18712033	0.803223968
ASV_16s_1246	4.192725668	7.339601488	3.546255652	2.418565375	0.933103337	1
ASV_16s_1247	6.721019742	0.286296478	3.210710686	5.045572055	0.654401738	1
ASV_16s_1248	5.467937256	0.397119173	2.370604051	11.85111947	0.105562416	0.542604265
ASV_16s_1249	4.580364189	6.244278786	3.042291454	4.221165821	0.753980182	1
ASV_16s_1250	2.630803841	-8.670911685	3.552157344	0.877545329	0.996569713	1
ASV_16s_1251	4.417195572	1.092882421	2.058077014	11.33110281	0.124814389	0.607859181
ASV_16s_1252	4.737518012	1.174488068	1.928884962	12.72029448	0.079223802	0.443983392
ASV_16s_1253	1.344659175	-22.12696583	3.55060762	0.263708056	NA	NA
ASV_16s_1254	4.611384261	1.611232978	2.373661939	7.926860042	0.339087519	1
ASV_16s_1255	4.807933681	-14.96906057	3.335480755	-0.368637884	1	1
ASV_16s_1256	1.894212938	18.35658391	3.551441189	0.594665272	0.999020772	NA
ASV_16s_1257	3.602529989	0.668502191	2.094024905	7.155603326	0.412860462	1
ASV_16s_1258	4.272059761	4.033896139	2.84857153	5.672054546	0.578520045	1
ASV_16s_1259	4.622296935	-0.866026931	2.534962889	5.736084263	0.570881088	1
ASV_16s_1260	2.923369746	13.10297259	3.551503915	1.711550074	0.974070675	1
ASV_16s_1261	4.0461733	7.601110796	2.140554462	2.930947539	0.891315792	1
ASV_16s_1262	5.327943872	17.92190399	3.550589526	2.723460252	0.909352407	1
ASV_16s_1263	3.147953226	-2.305842674	2.771825082	3.056057229	0.879768483	1
ASV_16s_1264	4.973635198	0.517449856	2.419025322	9.242358407	0.235720947	0.949152858
ASV_16s_1265	3.977410939	-8.63296503	3.549218604	3.428843791	0.842703807	1
ASV_16s_1266	4.368781141	10.70692764	3.385949784	3.293935701	0.856545339	1
ASV_16s_1267	5.079707117	-18.34452606	3.048145761	0.741065242	0.997999931	1
ASV_16s_1268	0.718363925	-22.93278364	3.551440312	0.070322625	NA	NA
ASV_16s_1269	4.277343627	8.950716739	2.413058857	3.147241017	0.871055266	1
ASV_16s_1270	4.375809089	-0.331825991	2.172834801	2.78677329	0.904002033	1
ASV_16s_1271	5.303728595	0.397313308	2.245047111	1.430806179	0.984608961	1
ASV_16s_1272	5.364265276	-4.218047728	3.065373527	2.63671159	0.916455675	1
ASV_16s_1273	4.329727977	-11.08868373	3.548682563	2.662355311	0.914383709	1
ASV_16s_1274	5.137993208	18.07171506	3.551439903	1.242740204	0.989909291	1

ASV_16s_1275	6.102792989	-15.69458112	3.547037838	2.004198259	NA	NA
ASV_16s_1276	4.499598286	7.530995966	2.418847909	5.261604514	0.628072074	1
ASV_16s_1277	5.486696924	0.934064917	1.79870049	15.21728403	0.033312845	0.231008289
ASV_16s_1278	5.197560815	-18.07545609	3.552157344	0.78276043	0.997615934	1
ASV_16s_1279	4.839902392	-0.832984811	2.864115597	5.508670841	0.59813821	1
ASV_16s_1280	4.446189569	3.957012504	1.727871352	11.91338224	0.103443564	0.533980528
ASV_16s_1281	5.309035066	-0.691068597	3.549042799	4.952355394	0.665777629	1
ASV_16s_1282	4.057018813	9.080297515	3.545983551	2.193955491	0.948329934	1
ASV_16s_1283	4.564268921	4.395186022	3.544450851	0.749835186	0.997922842	1
ASV_16s_1284	1.467495718	-21.56874253	3.551444859	1.780904797	0.970966383	NA
ASV_16s_1285	3.664827684	-5.496451923	3.492134612	1.689402401	0.975019793	1
ASV_16s_1286	5.427145887	0.542322449	2.211802274	13.04021157	0.071134684	0.41279216
ASV_16s_1287	1.044857247	-23.51996907	3.551439436	0.300043153	NA	NA
ASV_16s_1288	4.286781214	-1.815956451	1.74027734	7.576440803	0.371418598	1
ASV_16s_1289	4.943843134	-0.536842557	1.931175071	11.98204926	0.101150736	0.527771404
ASV_16s_1290	4.11974995	-2.116815453	3.54677478	2.60012386	0.919370857	1
ASV_16s_1291	5.321188787	-26.1368967	3.55059611	3.214292484	0.864496102	1
ASV_16s_1292	3.062272025	-11.58566131	3.549205353	2.138149459	NA	NA
ASV_16s_1293	5.399380832	1.179034726	3.061937967	5.37795106	0.613943135	1
ASV_16s_1294	4.654988709	-1.19236308	2.982946034	5.38518849	0.613065967	1
ASV_16s_1295	5.015042243	-1.330217559	1.579775761	18.31525837	0.010625763	0.0887068
ASV_16s_1296	3.985060325	10.45462807	2.400878426	5.657405946	0.580271835	1
ASV_16s_1297	2.598214245	-13.83924539	3.550995472	0.735370485	0.998048961	1
ASV_16s_1298	4.195492631	8.148050354	2.602920727	4.860762561	0.676950412	1
ASV_16s_1299	5.526288686	8.3216501	3.425991647	2.311616481	0.940602512	1
ASV_16s_1300	4.278608157	-21.74650801	2.622653866	1.581970133	0.979335427	1
ASV_16s_1301	5.13313156	1.662363196	1.99787224	9.412462137	0.224383192	0.917621126
ASV_16s_1302	3.417707002	1.550825814	3.361806356	1.93884576	0.963147371	1
ASV_16s_1303	3.595962037	3.282009553	1.71794116	6.84035852	0.445688974	1
ASV_16s_1304	1.241178568	-23.76756897	3.551439133	0.405674522	NA	NA
ASV_16s_1305	4.408954417	6.19261046	3.02822131	4.335464503	0.740428693	1
ASV_16s_1306	5.331018984	2.639058966	2.489069665	8.761003033	0.270273579	1
ASV_16s_1307	4.044797792	-23.83738354	3.551439595	1.205916056	0.990790043	1
ASV_16s_1308	0.807920618	-23.22487909	3.55144	0.142334906	NA	NA
ASV_16s_1309	4.446939026	-1.204461757	2.267972997	6.582014734	0.47365637	1
ASV_16s_1310	5.349520369	-5.280752809	3.411697385	3.288010275	0.857142682	1
ASV_16s_1311	4.08798256	6.450749254	2.739286348	4.917294941	0.670055521	1
ASV_16s_1312	4.595254823	-7.604816201	3.552157344	1.059090076	0.993818191	1
ASV_16s_1313	3.890352261	10.89212077	3.546408582	2.689852245	0.91213595	1
ASV_16s_1314	3.867793646	-9.919540942	2.143689559	3.92837318	0.78798951	1
ASV_16s_1315	3.468595066	-3.199789055	1.740342394	5.995909381	0.540227074	1
ASV_16s_1316	3.875644303	-8.379915107	3.549270286	3.255393595	0.860414195	1
ASV_16s_1317	4.678138627	-0.799680255	3.23879174	4.265760194	0.748708625	1
ASV_16s_1318	2.762146493	18.41075176	3.55144035	2.229217892	0.946072328	1
ASV_16s_1319	4.850432071	0.663974202	1.846780868	15.96068594	0.025477977	0.187440675
ASV_16s_1320	4.362927732	0.638050289	2.612511406	6.941323892	0.435014697	1
ASV_16s_1321	3.752024203	10.16384435	3.546638444	2.639222645	0.916253829	1
ASV_16s_1322	3.700607279	11.2274698	3.545845796	2.460455925	NA	NA
ASV_16s_1323	4.846859969	0.378626227	3.438488022	1.544201211	0.980739841	1
ASV_16s_1324	4.666646047	-0.709276674	2.273938667	6.960058758	0.433050361	1
ASV_16s_1325	5.283144264	0.560088625	2.560322525	11.1564208	0.131940061	0.630033311
ASV_16s_1326	5.825335384	0.832505252	2.693818842	7.26576283	0.401743235	1
ASV_16s_1327	3.94953102	5.363112149	2.811129187	4.243906164	0.751294636	1
ASV_16s_1328	3.550122396	-0.773077612	2.692537699	5.078867989	0.65033905	1
ASV_16s_1329	4.902095987	0.432985147	2.627753663	5.095322278	0.648331699	1
ASV_16s_1330	1.010221095	-23.33051732	3.551439503	0.279361167	NA	NA
ASV_16s_1331	4.538899564	-0.243889879	1.609471537	15.54646038	0.029598845	0.210761189
ASV_16s_1332	5.451861983	0.567103406	2.161134428	10.87551694	0.144141837	0.673682217
ASV_16s_1333	5.598282418	0.453387403	2.433581958	12.08581903	0.097771749	0.519090802
ASV_16s_1334	4.637092401	-0.797775607	3.547526202	3.541104941	0.830852711	1
ASV_16s_1335	4.081273836	9.51647867	2.566709181	3.842131647	0.797770708	1
ASV_16s_1336	4.621020659	6.95973726	3.465980811	2.110963575	NA	NA
ASV_16s_1337	4.82777791	0.402493064	3.03615238	7.64592793	0.364848	1
ASV_16s_1338	3.921201883	-10.80373934	3.548735762	2.593928381	0.919859675	1
ASV_16s_1339	4.222128791	0.223627051	2.575327711	4.218267601	0.75432205	1
ASV_16s_1340	4.772597171	1.322947554	3.548622215	2.599467973	0.919422672	1
ASV_16s_1341	3.736185728	9.771522101	3.547299778	2.957425331	0.888912593	1

ASV_16s_1342	4.696890479	0.614447006	2.952512356	3.998286152	0.77997479	1
ASV_16s_1343	3.247195743	-29.46325202	3.549076035	1.60528985	NA	NA
ASV_16s_1344	2.436068259	-29.78115867	3.551260737	0.512808404	0.999398218	1
ASV_16s_1345	3.790670633	6.654436748	3.548472725	2.367846495	0.93671539	1
ASV_16s_1346	5.223379847	3.243233036	3.548640658	4.082220767	0.770259991	1
ASV_16s_1347	4.473295888	-0.832313649	2.944388639	4.24976842	0.750601435	1
ASV_16s_1348	4.872007178	1.656292598	3.546102857	3.286904222	0.857254081	1
ASV_16s_1349	5.131220765	-8.938989817	3.550065113	4.259050199	0.749503151	1
ASV_16s_1350	5.097120287	-9.169140297	2.594514381	3.619388941	0.82242293	1
ASV_16s_1351	1.187097151	-29.94811685	3.550509642	1.238975184	NA	NA
ASV_16s_1352	2.494174046	0.747791707	2.431965435	4.4423302	0.727650104	1
ASV_16s_1353	3.947787497	5.035941882	3.546836707	2.323891887	0.939764541	1
ASV_16s_1354	3.778801908	5.770752915	3.356270731	3.142066681	0.871556168	1
ASV_16s_1355	3.992941382	-10.21613561	3.547828271	1.707977944	0.974225135	1
ASV_16s_1356	4.887905784	5.037872988	2.126760219	1.54960859	0.980542345	1
ASV_16s_1357	4.010354804	3.155259287	2.04497024	9.507060931	0.218270278	0.898694462
ASV_16s_1358	4.543073762	5.834452028	3.520318863	2.289158409	0.942120155	1
ASV_16s_1359	4.063273181	0.640493213	1.621619178	13.64360774	0.057895584	0.358478795
ASV_16s_1360	4.814866673	-0.11770338	2.043279276	5.912507459	0.550001853	1
ASV_16s_1361	4.500361411	-0.799578236	3.227623466	4.295955364	0.745127622	1
ASV_16s_1362	4.309718719	-0.389060194	2.111366111	9.121361747	0.244059854	0.961181421
ASV_16s_1363	4.85671536	0.595505188	1.499000075	10.26598807	0.17399457	0.766343316
ASV_16s_1364	4.429585935	-0.818682915	2.860534141	5.417759297	0.609121292	1
ASV_16s_1365	2.897863877	0.696192853	2.903865976	3.530771345	0.831955551	1
ASV_16s_1366	3.267884514	-1.789479867	2.96670415	2.77982613	0.904595832	1
ASV_16s_1367	4.043127482	-10.91012046	3.548641543	2.764014775	0.905941148	1
ASV_16s_1368	4.63031326	6.363988444	2.772172036	4.819592419	0.681968288	1
ASV_16s_1369	4.406218934	-30	3.54883869	2.289369806	0.942105963	1
ASV_16s_1370	5.095515807	0.637785344	2.69636385	4.796693493	0.684757627	1
ASV_16s_1371	3.606082034	10.42590648	3.061036221	3.369748195	0.84882292	1
ASV_16s_1372	4.864657131	-1.001289169	2.3607189	3.960167251	0.784353781	1
ASV_16s_1373	3.759603842	4.172048998	2.67401496	5.603906184	0.586682201	1
ASV_16s_1374	3.553190959	-13.22293918	3.545803706	1.207891392	NA	NA
ASV_16s_1375	2.854495282	12.08670713	3.55145155	1.829667985	NA	NA
ASV_16s_1376	1.188224384	-0.626388604	3.344704029	2.319462833	0.940067573	NA
ASV_16s_1377	5.200166487	-0.310148833	3.523685428	3.983119864	0.781719611	1
ASV_16s_1378	7.32214615	-30	3.548354094	2.559515866	NA	NA
ASV_16s_1379	3.726978912	-10.05923119	3.548901196	3.082948819	0.877224178	1
ASV_16s_1380	4.786510704	5.735554562	1.970981147	1.011697343	0.994637629	1
ASV_16s_1381	4.990576421	0.778428098	3.551112958	0.958304829	NA	NA
ASV_16s_1382	1.225069097	-22.455864	3.550610888	0.212360949	NA	NA
ASV_16s_1383	1.854554527	-1.024432496	3.545697123	2.535840352	0.924374157	NA
ASV_16s_1384	5.697450196	2.110099036	2.884162562	4.94003674	0.667280796	1
ASV_16s_1385	4.30869925	-1.758740041	2.06549823	11.38718348	0.122599292	0.602054536
ASV_16s_1386	4.225212036	-1.263067308	2.29481468	6.490752429	0.483750983	1
ASV_16s_1387	3.680489615	1.938614391	1.816175827	7.416566637	0.386831738	1
ASV_16s_1388	3.573806807	0.757216126	2.453317938	6.028610543	0.536412645	1
ASV_16s_1389	3.86907836	10.76836116	2.413271355	5.282807244	0.6254937	1
ASV_16s_1390	6.126420968	1.170528377	3.549032116	5.087337646	NA	NA
ASV_16s_1391	3.719387186	-8.55769254	3.549227125	3.306157189	0.855310397	1
ASV_16s_1392	5.653764347	7.871813527	3.5500381	2.880988266	0.895789129	1
ASV_16s_1393	4.578839864	0.199689972	2.270250311	6.69338758	0.461486819	1
ASV_16s_1394	1.375749592	-22.97347496	3.551440032	1.993551603	NA	NA
ASV_16s_1395	3.659057266	-8.634972822	3.549271261	3.073683604	0.87810322	1
ASV_16s_1396	4.833405344	0.039600978	2.52572132	7.785317812	0.351904628	1
ASV_16s_1397	4.837378718	-10.49961645	3.550028068	4.712986867	0.694941292	1
ASV_16s_1398	5.637990279	-10.4802239	3.023929125	3.2125572	0.864667428	1
ASV_16s_1399	4.946156701	5.440266727	2.961026288	0.937312114	0.9957774	1
ASV_16s_1400	0.712260172	-0.170410889	3.550543468	1.446770879	0.984095456	NA
ASV_16s_1401	3.636346088	8.198516466	3.545982073	2.01813019	0.958830086	1
ASV_16s_1402	3.992290982	-29.90087814	2.27516566	4.441750262	0.727719699	1
ASV_16s_1403	2.542728314	-18.5838525	3.55153106	0.841646136	0.996995348	1
ASV_16s_1404	2.338696099	-12.17083732	3.552157344	0.805311922	0.997389365	1
ASV_16s_1405	5.09183762	0.22049179	3.541690472	2.419824926	0.933012369	1
ASV_16s_1406	3.872920482	9.450739556	3.54473058	1.803084573	0.969931261	1
ASV_16s_1407	4.055816338	0.046967418	2.231344029	4.119540239	0.765910396	1
ASV_16s_1408	3.661462311	-8.397445674	3.549202425	3.438855792	0.841658746	1

ASV_16s_1409	3.701138233	2.509997781	3.542883579	3.309462755	0.854975714	1
ASV_16s_1410	3.201645217	0.813824287	1.836443577	7.131913834	0.41527561	1
ASV_16s_1411	4.103524918	1.30007028	2.688198737	3.675703669	0.816279946	1
ASV_16s_1412	3.481860057	-2.687050078	2.65816865	3.382193696	0.847541343	1
ASV_16s_1413	3.870947701	-0.300146083	2.501714912	7.816258786	0.349074675	1
ASV_16s_1414	5.003319724	1.258648312	3.548849346	2.308370777	0.940823081	1
ASV_16s_1415	4.719071366	0.463680031	2.889470604	3.398736661	0.84583192	1
ASV_16s_1416	2.315609451	-7.903122567	3.552157344	0.799222532	0.99745188	1
ASV_16s_1417	4.691850828	1.170831233	3.23924863	2.286736803	0.942282604	1
ASV_16s_1418	5.085600391	11.24800097	3.550124546	4.240824734	0.751658863	1
ASV_16s_1419	4.214914293	0.16412411	2.791245178	5.147188364	0.642006528	1
ASV_16s_1420	4.723015367	1.421621799	3.30491829	3.9297566	0.787831637	1
ASV_16s_1421	5.70347487	-6.055176545	3.550380368	0.531430623	NA	NA
ASV_16s_1422	4.467176189	-1.801678047	2.654187003	4.348312796	0.738897495	1
ASV_16s_1423	4.570368187	-1.203696606	2.481459167	3.298953624	0.856038765	1
ASV_16s_1424	4.675762421	-0.670242073	2.76304844	4.173217995	0.759623969	1
ASV_16s_1425	3.279462952	-6.676863917	3.546707696	2.445649026	NA	NA
ASV_16s_1426	1.464293859	-14.7709842	3.552157344	0.518062132	NA	NA
ASV_16s_1427	3.992689793	2.176998413	2.87497814	4.220805681	0.754022669	1
ASV_16s_1428	1.173292356	-12.53040087	3.552157344	-0.177014967	NA	NA
ASV_16s_1429	3.041946398	0.626556391	2.188533196	9.109073272	0.24491964	0.961181421
ASV_16s_1430	1.896762953	-20.94312789	3.551449084	1.733452882	0.973111977	NA
ASV_16s_1431	2.307060675	-12.13459899	3.552157344	0.796953362	0.997474921	1
ASV_16s_1432	3.762512774	0.722259941	2.497931354	6.933270684	NA	NA
ASV_16s_1433	0.803766532	8.077838946	3.550189668	0.168156681	NA	NA
ASV_16s_1434	3.488306025	8.254092416	3.548145422	2.225987565	0.94628123	1
ASV_16s_1435	2.51989934	-1.023795771	3.506708076	1.470139315	0.983325467	1
ASV_16s_1436	4.24065004	-0.824086939	2.911515412	4.531122671	0.71696641	1
ASV_16s_1437	2.460708256	11.13122368	3.551539345	1.646328646	0.976807371	1
ASV_16s_1438	4.372817382	-5.58142894	2.232076642	5.112356444	0.646253931	1
ASV_16s_1439	3.455572033	-10.16574205	3.548867236	2.851021436	0.898433389	1
ASV_16s_1440	4.458346544	7.85616209	3.218077436	-0.215349749	1	1
ASV_16s_1441	3.860220926	0.010144565	2.186693418	9.919471628	0.193183059	0.820519624
ASV_16s_1442	3.351228242	-8.413225832	3.549404506	2.721545754	0.909512035	1
ASV_16s_1443	4.22876925	-2.919924306	2.00501874	5.005906804	0.659242355	1
ASV_16s_1444	0.980337225	-21.8313463	3.550647271	0.116799055	NA	NA
ASV_16s_1445	4.341878576	0.511058164	2.217170178	12.25151753	0.092585099	0.499217205
ASV_16s_1446	1.155065148	-21.74464097	3.550613105	0.17992776	NA	NA
ASV_16s_1447	4.271286221	-0.943889344	2.563845228	5.046713704	0.654262425	1
ASV_16s_1448	4.532623605	1.838355274	3.549415339	2.206499013	0.947532612	1
ASV_16s_1449	6.326880204	1.47573765	3.55073055	4.010418869	0.778576577	1
ASV_16s_1450	3.667190759	-10.82198654	3.54860458	2.765656519	0.905801858	1
ASV_16s_1451	4.127034759	-0.457988413	2.060160501	5.795492569	0.563821438	1
ASV_16s_1452	4.657922073	-10.87301566	3.548946665	3.909895172	0.790095321	1
ASV_16s_1453	1.079240177	-19.71809929	3.551467753	0.902499738	NA	NA
ASV_16s_1454	3.149106087	1.126966023	2.094470568	8.210625004	0.314388269	1
ASV_16s_1455	1.149231486	-22.29612424	3.550613279	0.177137099	NA	NA
ASV_16s_1456	3.534716261	-0.006787987	1.964438156	11.92976004	0.102892538	0.532675821
ASV_16s_1457	3.408768536	-7.6236063	3.54759164	2.730036339	0.908803128	1
ASV_16s_1458	4.709552832	-0.544966322	3.549576078	3.44479894	0.84103727	1
ASV_16s_1459	4.825401207	-9.374504998	3.550033947	4.5528329	0.714346317	1
ASV_16s_1460	4.866396824	13.34823794	3.547233784	0.330963288	NA	NA
ASV_16s_1461	3.858932346	-0.725049765	2.151189698	5.010362661	0.658698557	1
ASV_16s_1462	4.647011409	-9.237214009	3.550040467	4.353372348	0.738294115	1
ASV_16s_1463	3.190969863	3.550662901	2.642572723	4.352242232	0.738428907	1
ASV_16s_1464	6.257498109	21.02132318	3.549842324	1.875058082	NA	NA
ASV_16s_1465	2.387007084	-22.75188091	3.552157344	0.131248915	0.999994085	1
ASV_16s_1466	0.900539948	-23.2737596	3.551439745	0.208860835	NA	NA
ASV_16s_1467	3.545452439	-1.184251609	3.168599379	2.374750459	0.936229585	1
ASV_16s_1468	1.079255011	0.008336939	3.550550898	3.992549232	0.780635193	NA
ASV_16s_1469	3.501267318	3.716529666	2.71210998	5.194358907	0.636258015	1
ASV_16s_1470	2.024001987	0.334423523	3.548507194	3.890742454	0.792272304	1
ASV_16s_1471	3.441724273	7.165511782	3.546705612	1.938976381	NA	NA
ASV_16s_1472	4.211941402	-27.21679088	3.551507455	1.432430092	0.984557191	1
ASV_16s_1473	5.345848071	14.89002249	3.550699165	2.553637525	0.92300424	1
ASV_16s_1474	4.1859548	1.090148046	3.547402411	3.028641834	0.882340039	1
ASV_16s_1475	4.199357528	2.532593815	2.37366286	3.832066445	0.798904238	1

ASV_16s_1476	2.044117553	-13.18356776	3.552157344	0.722700412	NA	NA	
ASV_16s_1477	3.900838973	-0.298076301	1.654439297	20.89936623	0.003921983	0.039728538	
ASV_16s_1478	5.236553016	-10.14776198	3.547776278	2.416801247	NA	NA	
ASV_16s_1479	4.293119049	-0.940598697	2.587198457	5.681058573	0.577444027		1
ASV_16s_1480	4.79518725	2.533650061	2.868637658	2.569237827	0.921793777		1
ASV_16s_1481	0.980076508	-23.19816103	3.551439564	0.26076293	0.999937815	NA	
ASV_16s_1482	3.307000712	-8.408131375	3.549240959	3.111595395	0.874490337		1
ASV_16s_1483	5.161194837	-2.062776618	3.552157344	0.488648555	NA	NA	
ASV_16s_1484	3.526177574	-29.09668944	3.549162639	0.426460072	NA	NA	
ASV_16s_1485	0.601512151	-22.71239573	3.551440855	-0.038473204	NA	NA	
ASV_16s_1486	1.0624821	-23.49946851	3.551439405	0.310282014	NA	NA	
ASV_16s_1487	3.547689452	-0.87198405	3.330984185	4.4179121	0.730578162		1
ASV_16s_1488	4.766591915	2.201902805	2.745010612	6.140084313	0.523491118		1
ASV_16s_1489	2.184782219	-7.938192065	3.552157344	0.763531616	0.997798559		1
ASV_16s_1490	3.327461804	8.987745286	3.545683711	1.720470453	0.973682636		1
ASV_16s_1491	3.81393269	-1.383854809	1.965320205	4.789075624	0.685685275		1
ASV_16s_1492	3.736550903	0.911143865	2.983608423	1.056918051	NA	NA	
ASV_16s_1493	3.688423964	2.699127947	3.54758755	1.585091831	0.979216736		1
ASV_16s_1494	4.812223967	0.758056816	2.624638189	7.058020483	0.422863916		1
ASV_16s_1495	0.993129093	-23.30258556	3.551439537	0.268876608	NA	NA	
ASV_16s_1496	4.161708138	-30	3.549883935	2.46849877	NA	NA	
ASV_16s_1497	2.19845647	-14.03181514	3.552157344	0.767360791	0.997762955		1
ASV_16s_1498	1.112481114	0.215538438	3.550591857	3.513165517	0.833829045	NA	
ASV_16s_1499	3.85031462	-0.666310913	2.269213564	7.610870032	0.368153233		1
ASV_16s_1500	3.750913076	-0.283693039	2.639228164	7.258883135	0.40243202		1
ASV_16s_1501	3.037856766	17.56135324	3.549904188	0.416498215	0.999698454		1
ASV_16s_1502	3.55566744	3.209054572	2.293193752	8.446360077	0.294882919		1
ASV_16s_1503	2.635620627	0.151878026	3.545109	0.873497444	NA	NA	
ASV_16s_1504	4.600615557	-8.606630146	3.550074843	3.97896924	0.782196538		1
ASV_16s_1505	3.35521519	-8.263157635	3.549298184	2.997398394	0.885242797		1
ASV_16s_1506	3.422559485	8.921119493	2.467063561	3.858331495	0.795942696		1
ASV_16s_1507	3.335889319	-1.582746348	3.544737102	2.981715322	0.886688544		1
ASV_16s_1508	4.211474897	0.362837544	1.696668874	19.42272304	0.006961325	0.062189545	
ASV_16s_1509	1.105479018	-22.27457423	3.55061489	0.155743654	NA	NA	
ASV_16s_1510	0.986621039	-23.27358413	3.551439551	0.264843155	NA	NA	
ASV_16s_1511	4.162407237	-0.145497881	2.146347486	9.726119774	0.204632524	0.854755659	
ASV_16s_1512	4.599729853	4.005555203	2.765507645	2.767208591	0.90567009		1
ASV_16s_1513	4.392774268	-23.70691226	3.550598678	3.002676233	NA	NA	
ASV_16s_1514	5.118579568	0.916396992	1.860921876	12.36177426	0.089271998	0.483506725	
ASV_16s_1515	1.427709893	18.1739564	3.551442323	0.421239953	NA	NA	
ASV_16s_1516	3.328130509	10.22381922	3.049213469	3.217220239	0.864206855		1
ASV_16s_1517	3.539962035	10.98861274	3.546438714	2.557343931	0.922717466		1
ASV_16s_1518	4.86599273	0.192333068	3.191344427	3.521949566	0.83289517		1
ASV_16s_1519	3.192486849	2.098264332	3.480633365	1.559634965	0.980172991		1
ASV_16s_1520	3.906383213	4.266299017	2.329623361	2.7801894	0.904564823		1
ASV_16s_1521	0.747994569	-22.92706321	3.551440192	0.095065155	0.99999806	NA	
ASV_16s_1522	2.929041532	1.059909136	2.774390091	2.979219335	0.886917932		1
ASV_16s_1523	3.218632455	10.54097499	3.546425391	2.425025593	NA	NA	
ASV_16s_1524	3.528366307	10.37462928	3.546604124	2.541204515	0.923962495		1
ASV_16s_1525	3.584333669	7.528665123	2.802011895	3.039772286	0.881298743		1
ASV_16s_1526	3.882158396	-0.847889866	2.828422684	5.473430836	0.602390521		1
ASV_16s_1527	3.733435227	-1.021359561	2.30807273	6.660397578	0.465074118		1
ASV_16s_1528	2.382731617	18.50580716	3.551440466	0.735402169	NA	NA	
ASV_16s_1529	3.287109419	-2.911482464	2.147933731	5.454964885	0.604621372		1
ASV_16s_1530	4.215961447	-0.274383002	2.476811985	8.873234163	0.26188595		1
ASV_16s_1531	3.252230077	-0.114695471	2.695627859	4.008171877	0.778835686		1
ASV_16s_1532	3.320879323	-8.502806379	3.549235779	3.070086794	0.878443783		1
ASV_16s_1533	3.706652085	-4.649861733	2.712400173	2.379689228	0.935880923		1
ASV_16s_1534	5.774641211	-0.563821326	3.099490599	3.788309556	0.80381167		1
ASV_16s_1535	3.058189299	0.890451178	2.311684861	3.042400441	0.881052321		1
ASV_16s_1536	3.928961979	-0.365181793	1.97423383	7.579722541	0.371106521		1
ASV_16s_1537	3.728101776	-6.106174132	2.642172738	4.335119013	0.740469846		1
ASV_16s_1538	3.958766467	-30	3.549888417	2.432849911	0.932068088		1
ASV_16s_1539	3.814956807	-0.988931453	2.370812487	4.532118563	0.716846285		1
ASV_16s_1540	4.310546163	0.480316016	2.777132862	4.317484932	0.742568875		1
ASV_16s_1541	3.183674579	1.385833323	2.41661139	7.774018659	0.352941998		1
ASV_16s_1542	3.527525473	3.74484267	1.955334918	6.187263257	0.518061877		1

ASV_16s_1543	3.167808745	-3.267924004	3.086951236	3.181936837	0.867676922	1
ASV_16s_1544	5.894878214	-9.33417602	3.547533396	3.328707587	NA	NA
ASV_16s_1545	3.527490501	-13.85513277	3.547032343	0.185529978	0.99998055	1
ASV_16s_1546	0.969700098	-23.75653671	3.551439611	0.254224602	NA	NA
ASV_16s_1547	0.584258869	-22.60157136	3.551440954	-0.056302768	NA	NA
ASV_16s_1548	2.920911569	-2.64493161	3.549219565	1.309616328	0.98818028	1
ASV_16s_1549	3.779939319	-1.058253846	2.074466644	5.709920968	0.5739988	1
ASV_16s_1550	3.717338639	-3.172481447	1.746344189	4.079456124	0.770581496	1
ASV_16s_1551	2.329785081	-19.52053325	3.552157344	0.156226658	0.999989222	1
ASV_16s_1552	3.359224576	4.45579866	3.547345102	2.839216227	0.899466943	1
ASV_16s_1553	3.453615249	-1.366589192	2.644212749	1.38408784	0.98605366	1
ASV_16s_1554	4.345255744	0.336522151	2.39082714	9.893070759	0.194714476	0.824132425
ASV_16s_1555	2.030918863	8.215891054	3.547625945	0.721300443	0.998166667	1
ASV_16s_1556	5.107902	-9.830408116	3.552157344	0.957906968	NA	NA
ASV_16s_1557	3.914940386	0.564176698	2.139724418	9.145878643	0.242351604	0.961181421
ASV_16s_1558	3.466056137	0.113473127	3.549450996	1.566367446	0.979922674	1
ASV_16s_1559	4.161451635	1.942119157	3.549479266	1.942672378	0.962945008	1
ASV_16s_1560	3.503463263	5.787593236	3.016353511	3.462811431	0.839148618	1
ASV_16s_1561	2.450829459	-9.767417107	3.54754792	1.325807386	NA	NA
ASV_16s_1562	3.945566978	-24.66810438	3.549933196	0.688143244	NA	NA
ASV_16s_1563	3.222851783	2.300434048	3.547569142	3.056574827	0.879719715	1
ASV_16s_1564	1.066101797	-22.08497199	3.550616356	0.135754802	NA	NA
ASV_16s_1565	3.455688532	8.803628768	3.29242036	3.305578256	0.855368984	1
ASV_16s_1566	1.769210852	-1.869268601	3.546623275	1.924051865	0.963923977	NA
ASV_16s_1567	2.923288534	1.394856703	3.545421612	1.309125389	NA	NA
ASV_16s_1568	3.264405943	-0.112752979	2.983431768	5.863380078	0.555789445	1
ASV_16s_1569	4.039156514	-0.082893058	2.810735183	7.679827706	0.361670962	1
ASV_16s_1570	2.942329477	9.109470987	2.812160868	2.599016573	NA	NA
ASV_16s_1571	3.247803996	7.769321439	3.548078786	2.489116204	0.927914393	1
ASV_16s_1572	5.108070266	1.051534168	2.66281336	6.550011606	0.47718392	1
ASV_16s_1573	4.044852428	0.245800991	3.539081544	4.405968708	0.732008635	1
ASV_16s_1574	1.058809719	-22.21584453	3.550616598	0.131972644	NA	NA
ASV_16s_1575	3.209180397	-8.394297338	3.549263815	2.986036759	0.886290933	1
ASV_16s_1576	3.874753107	2.959178708	2.178383304	3.262118498	0.859741986	1
ASV_16s_1577	1.367277728	18.14325984	3.551442522	0.39471712	NA	NA
ASV_16s_1578	4.553379808	0.987096337	3.411332132	3.383122666	0.847445528	1
ASV_16s_1579	3.017393311	9.635587576	3.54677666	2.241839859	0.945252057	1
ASV_16s_1580	5.097782151	16.20526359	3.017141312	4.08216512	0.770266463	1
ASV_16s_1581	4.460929066	3.054450505	3.54880673	3.255831974	0.860370412	1
ASV_16s_1582	3.320014505	0.149707133	3.401921796	4.361632224	0.737308592	1
ASV_16s_1583	3.649406761	0.18006746	1.898444554	5.771000117	0.566728563	1
ASV_16s_1584	4.486683773	0.034142193	1.989358109	16.51684863	0.020791821	0.158292451
ASV_16s_1585	3.050013103	-8.280827686	3.549304304	2.832957912	0.900012986	1
ASV_16s_1586	3.380234801	0.84945649	2.274637934	6.155139249	0.521756009	1
ASV_16s_1587	4.090698063	0.412781727	2.339199832	12.38730828	0.088520091	0.482673737
ASV_16s_1588	3.418179221	-0.966050352	2.720699191	4.776241012	0.687247823	1
ASV_16s_1589	2.051316294	-20.75429796	3.551434521	0.599524062	0.99899436	1
ASV_16s_1590	3.089706191	-0.26007109	2.141850438	8.712613431	0.273953001	1
ASV_16s_1591	2.460635625	1.867724943	3.547899133	2.190280079	NA	NA
ASV_16s_1592	0.824340414	-23.12865109	3.551439951	0.154638807	NA	NA
ASV_16s_1593	0.871434999	-9.76624357	3.552157344	-0.017087604	NA	NA
ASV_16s_1594	1.039850317	-22.05358522	3.550617329	0.12201542	NA	NA
ASV_16s_1595	3.921295679	-1.485607514	1.555755828	8.77945489	0.268880566	1
ASV_16s_1596	2.018270865	-21.05224221	3.552157344	0.600704281	0.998987873	1
ASV_16s_1597	2.835189058	-7.106203793	2.677250153	3.127642784	0.872948424	1
ASV_16s_1598	0.87909333	-22.04806479	3.550655441	0.056743097	NA	NA
ASV_16s_1599	4.963314275	0.175018768	2.564001913	6.686862057	0.462195217	1
ASV_16s_1600	3.110698811	-8.319739045	3.549243269	3.005744666	0.884470295	1
ASV_16s_1601	3.530075259	3.823759986	2.540637663	6.492736884	0.483530335	1
ASV_16s_1602	1.03547507	-21.82907936	3.550617699	0.119690301	NA	NA
ASV_16s_1603	3.135599463	0.390736972	3.549555395	1.658563059	0.976307456	1
ASV_16s_1604	3.136222814	3.413991151	2.834189491	5.34028534	0.618511686	1
ASV_16s_1605	2.730946242	0.924756407	2.409929475	7.859412332	0.345153998	1
ASV_16s_1606	3.257797393	-15.05557661	3.54739728	1.195560175	NA	NA
ASV_16s_1607	3.142847866	-1.041604187	2.948169863	4.200005941	0.756474031	1
ASV_16s_1608	3.382997	-10.54880871	3.548648615	2.704548656	0.910923615	1
ASV_16s_1609	3.650466667	-12.72244414	2.662497623	6.529323512	0.4794714	1

ASV_16s_1610	4.856430768	-29.71616192	3.549038768	0.458537101	0.999584586	1
ASV_16s_1611	3.717936262	0.302120227	2.541437789	6.416747501	0.492014998	1
ASV_16s_1612	3.628909376	-0.432102169	1.940911973	8.062187841	0.327142215	1
ASV_16s_1613	4.040067003	0.132670012	3.082779315	3.413785623	0.844271077	1
ASV_16s_1614	3.453826876	-0.262892949	3.546337492	2.328645577	0.939438437	1
ASV_16s_1615	3.215100384	0.473491336	3.549266153	0.828926945	NA	NA
ASV_16s_1616	3.134072148	2.850594104	3.5445344	3.556442413	NA	NA
ASV_16s_1617	3.52651237	0.412759834	2.790962181	6.028965607	0.536371286	1
ASV_16s_1618	2.294361132	-9.539007651	3.54936541	1.041276059	0.994134934	1
ASV_16s_1619	3.334716531	1.471245559	1.618965263	13.11707836	0.069306662	0.409247386
ASV_16s_1620	3.624170685	-0.771323346	2.856993973	5.133143197	0.643718968	1
ASV_16s_1621	3.538421941	0.206615076	2.416677303	7.893227934	0.342103133	1
ASV_16s_1622	4.288532721	-1.142605754	3.545011815	3.88340644	NA	NA
ASV_16s_1623	4.218504883	1.053613757	3.156010926	5.072306234	0.65113963	1
ASV_16s_1624	3.381168545	0.972023908	2.333996747	7.408153763	0.387654134	1
ASV_16s_1625	3.626420112	-0.995553236	2.942786556	3.750339767	0.808042383	1
ASV_16s_1626	1.019432498	-22.11252333	3.550618323	0.111086529	NA	NA
ASV_16s_1627	3.910177752	4.074147085	1.461213828	11.77383453	0.108246069	0.549782513
ASV_16s_1628	4.132745335	0.751387149	2.331346206	8.11224104	0.322800797	1
ASV_16s_1629	3.728249006	-0.799652341	3.220657512	4.043131257	0.774796343	1
ASV_16s_1630	2.669334057	10.66807947	3.547251015	-0.230978176	1	1
ASV_16s_1631	3.354161196	-1.259840296	2.538488239	7.020605806	0.426737548	1
ASV_16s_1632	3.36164722	-0.831309263	3.10629307	4.675702046	0.699469449	1
ASV_16s_1633	4.324404523	2.2199204	3.548530923	3.549378378	0.829968044	1
ASV_16s_1634	3.352255428	-1.037956764	2.078118772	7.8908092	0.342320727	1
ASV_16s_1635	3.296866376	1.620973547	2.738821417	4.697366472	0.69683902	1
ASV_16s_1636	3.748281753	-1.264487206	3.426657916	1.643910589	0.976905443	1
ASV_16s_1637	4.526814824	-0.791128231	2.171270929	11.42009013	0.12131565	0.596961766
ASV_16s_1638	2.93555208	-8.236789017	3.549296537	2.79650767	0.90316724	1
ASV_16s_1639	2.908059559	-1.296010039	2.083700411	3.724730137	NA	NA
ASV_16s_1640	3.110517026	1.965993977	2.326558177	7.543704407	0.374541191	1
ASV_16s_1641	0.542694144	-22.50464482	3.551441217	-0.101507112	NA	NA
ASV_16s_1642	3.061848231	-9.170189217	3.548914738	2.542958489	0.923827658	1
ASV_16s_1643	4.256572956	1.065335207	1.517086152	20.14625868	0.005262282	0.050756909
ASV_16s_1644	3.092876482	2.633834753	3.038723896	5.005931558	0.659239334	1
ASV_16s_1645	3.684365721	-0.345329034	3.290699781	5.114564766	0.645984595	1
ASV_16s_1646	3.082771323	-28.85792356	3.549453633	0.776229535	0.997679038	1
ASV_16s_1647	1.077015502	-23.3674372	3.549373743	0.772278455	NA	NA
ASV_16s_1648	0.891993866	-22.08453613	3.551442646	1.467912831	0.983399772	NA
ASV_16s_1649	3.217981037	-3.778667865	2.412768976	5.806449876	0.562522438	1
ASV_16s_1650	4.852856246	15.73891626	3.55062507	2.785193675	NA	NA
ASV_16s_1651	1.003389927	-22.04696301	3.55061892	0.102346603	NA	NA
ASV_16s_1652	0.939437394	-18.99863607	3.551490439	0.607441583	NA	NA
ASV_16s_1653	1.814055163	-7.109142884	3.552157344	0.64943104	NA	NA
ASV_16s_1654	3.181449044	2.029094079	2.750978972	4.460024719	0.725525474	1
ASV_16s_1655	4.127385819	1.877274255	3.548400408	3.381552308	0.847607484	1
ASV_16s_1656	3.617599535	4.948894878	2.078941417	4.466437638	0.724754891	1
ASV_16s_1657	3.711879167	0.133494211	3.278009714	2.125063228	0.952595106	1
ASV_16s_1658	3.514818749	19.6453447	3.546534041	-0.365723838	1	1
ASV_16s_1659	3.110523246	-0.503427078	1.824571512	5.417760206	0.609121182	1
ASV_16s_1660	3.088657853	3.379140133	3.303412084	2.879995792	0.895877175	1
ASV_16s_1661	3.467538919	-0.591162324	2.006568788	5.430238283	0.607611245	1
ASV_16s_1662	1.83351747	-2.030951004	3.355539701	2.970771906	NA	NA
ASV_16s_1663	3.643204064	-20.72117904	3.550881976	1.615166225	0.978052765	1
ASV_16s_1664	3.136783338	-9.69652616	3.28953421	3.012946658	0.883801981	1
ASV_16s_1665	2.796834776	1.792230232	3.545885686	3.810192651	0.801361623	1
ASV_16s_1666	3.3768773	1.390588237	3.546595902	3.645738186	0.819556661	1
ASV_16s_1667	3.509255987	-0.922739052	3.547273544	3.035648285	0.881684997	1
ASV_16s_1668	3.115037843	3.373908408	3.336839276	3.045146279	0.880794643	1
ASV_16s_1669	2.375392149	13.43904849	3.551446424	1.639166928	NA	NA
ASV_16s_1670	2.513549382	2.036980454	3.547415976	2.575333352	NA	NA
ASV_16s_1671	2.114699381	0.906528065	3.549484558	0.923202635	0.995974117	1
ASV_16s_1672	3.039983268	1.783616877	2.998230993	5.297512566	0.623706302	1
ASV_16s_1673	1.981769512	-30	3.550600713	0.89030542	0.996409471	NA
ASV_16s_1674	1.308080946	-6.604955124	3.552157344	0.448831921	0.99961311	NA
ASV_16s_1675	2.902312878	-6.583895369	3.550595344	2.554080873	0.922969964	1
ASV_16s_1676	2.914768536	8.136762278	3.548206739	1.915090167	0.964389981	1

ASV_16s_1677	4.905227832	-25.82623111	3.548089572	0.21129989	NA		NA		
ASV_16s_1678	3.800890169	0.348476027	2.547724173	10.03798949		0.186430841		0.803223968	
ASV_16s_1679	2.949098886	-8.227121184	3.549271376	2.896828462		0.894379533		1	
ASV_16s_1680	1.889551253	-12.12697742	3.552157344	0.674440405		0.998524509	NA		
ASV_16s_1681	3.547978628	0.025250374	2.870107108	6.511374966		0.481460466		1	
ASV_16s_1682	3.20813071	-0.811795184	2.04563794	9.080731265		0.246911743		0.964516234	
ASV_16s_1683	3.21434747	2.569561955	1.998891215	9.114561016		0.244535385		0.961181421	
ASV_16s_1684	2.920811689	-22.06630643	3.550598587	2.493078036		0.927617393		1	
ASV_16s_1685	2.922621152	2.425526399	3.545526434	3.794472386		0.803122534		1	
ASV_16s_1686	3.053433336	-8.379193178	3.549471607	1.862877815		0.967038322		1	
ASV_16s_1687	0.920904787	-14.20330122	3.552157344	0.233639418	NA		NA		
ASV_16s_1688	3.544093114	-0.967998265	2.576148763	5.222937989		0.632777571		1	
ASV_16s_1689	3.255607603	-0.935943346	2.24087103	6.965255797		0.432506371		1	
ASV_16s_1690	3.722669139	0.368344696	2.695723959	0.807985293		0.997361604		1	
ASV_16s_1691	1.951070612	-5.32997065	3.550653705	1.413087984		0.985167016	NA		
ASV_16s_1692	0.977138446	-22.0367925	3.550620222	0.087737487	NA		NA		
ASV_16s_1693	3.333123083	0.216615431	2.509134058	8.198266306		0.315436222		1	
ASV_16s_1694	3.246083147	-8.850922996	3.546551793	1.059062865		0.993818683		1	
ASV_16s_1695	2.825701992	8.885421403	3.464099192	1.655850863		0.976418815		1	
ASV_16s_1696	3.935479471	1.8348278	3.549429756	2.077739863		0.955412515		1	
ASV_16s_1697	0.605261919	-22.62012481	3.551440836	-0.03468763	NA		NA		
ASV_16s_1698	3.154351821	6.470753862	2.639432294	4.733766365		0.692415392		1	
ASV_16s_1699	4.334864086	11.72231555	3.544459944	1.52161693		0.981551832		1	
ASV_16s_1700	3.398199784	-10.25163423	3.547539092	1.476019866		0.983128257		1	
ASV_16s_1701	4.081512069	1.049088884	2.908882546	3.949667989		0.785556105		1	
ASV_16s_1702	0.974221615	-22.0250425	3.55062033	0.086090488	NA		NA		
ASV_16s_1703	0.810168823	-22.71865389	3.551440361	0.912465412	NA		NA		
ASV_16s_1704	3.492141287	5.922758527	2.734844716	4.706054273		0.69578365		1	
ASV_16s_1705	3.129998031	1.434912205	2.734821496	5.189529218		0.63684638		1	
ASV_16s_1706	4.115382216	-7.492687163	3.184707692	4.557502463		0.713782402		1	
ASV_16s_1707	2.986346246	1.073346862	3.05003897	4.719999701		0.694089003		1	
ASV_16s_1708	3.510018994	-0.928168026	3.26640254	3.470359488		0.838354931		1	
ASV_16s_1709	2.32857394	3.062209313	3.546082276	1.481126609	NA		NA		
ASV_16s_1710	1.872416602	-16.06376256	3.552157344	0.305314732		0.999893839	NA		
ASV_16s_1711	3.368321417	-1.653438858	3.390513348	2.725142505		0.909212037		1	
ASV_16s_1712	4.299745187	0.761933481	3.546881173	5.007040411	NA		NA		
ASV_16s_1713	0.945746138	-0.014677687	3.550557508	3.094942213		0.876082536	NA		
ASV_16s_1714	4.699112297	-4.678781134	3.552157344	0.932101978	NA		NA		
ASV_16s_1715	2.045118652	-21.94012476	3.552042038	0.394351631		0.999748806		1	
ASV_16s_1716	3.031967472	6.881219377	3.548103827	1.49002975		0.982652823		1	
ASV_16s_1717	5.741871329	-8.700935797	3.547551004	3.066395654	NA		NA		
ASV_16s_1718	3.531468873	0.600870957	2.766036868	6.156651118		0.521581898		1	
ASV_16s_1719	0.966929537	-21.96392304	3.550620658	0.081950836	NA		NA		
ASV_16s_1720	2.960304812	-8.143968448	3.549502455	2.620659603		0.917740612		1	
ASV_16s_1721	3.397222409	-1.608967231	3.549265157	3.635056082		0.820720372		1	
ASV_16s_1722	3.62585119	2.242529081	2.415510354	5.341758429		0.618332905		1	
ASV_16s_1723	3.536268359	-0.797537556	3.547585967	3.306500194		0.855275682		1	
ASV_16s_1724	2.712738168	-0.273100228	2.489772728	2.704363586		0.910938929		1	
ASV_16s_1725	3.423111969	-0.584080032	1.530979916	10.5321138		0.160370043		0.723009076	
ASV_16s_1726	2.649279137	0.425391979	1.491795686	4.965582007		0.664163571		1	
ASV_16s_1727	3.577115847	-30	3.549887972	2.303454277		0.9411564		1	
ASV_16s_1728	3.495222743	-30	3.549892196	2.298111132		0.941517557		1	
ASV_16s_1729	4.292298025	29.47610553	3.548243848	0.188524126	NA		NA		
ASV_16s_1730	3.527479958	-0.866680194	3.255844928	3.590570541		0.825541332		1	
ASV_16s_1731	3.100822137	-29.38043068	3.549231358	1.786623778		0.970701446		1	
ASV_16s_1732	2.998733242	-0.629902612	2.779579194	4.872544401		0.67551384		1	
ASV_16s_1733	3.643668883	2.492279928	3.546677572	1.549045674	NA		NA		
ASV_16s_1734	3.10052032	1.154889867	2.62137315	7.61013322		0.368222913		1	
ASV_16s_1735	0.024511954	0.028553451	3.551938175	0.112869894		0.999996487	NA		
ASV_16s_1736	3.4616759	-0.303884994	2.305648533	10.54003805		0.159978667		0.722590209	
ASV_16s_1737	3.224124574	-1.175714329	2.485201225	4.708460686		0.695491275		1	
ASV_16s_1738	3.965736055	0.560208893	3.549217564	3.710034383		0.812504121		1	
ASV_16s_1739	3.10251543	1.002640256	3.545181356	3.568208811		0.827948967		1	
ASV_16s_1740	3.513811507	-0.931499842	2.936168538	4.009524814		0.778679683		1	
ASV_16s_1741	3.404222405	-2.124242287	1.983343249	6.073898448		0.531147636		1	
ASV_16s_1742	4.864011091	1.809599933	2.777479721	3.229924954		0.862948982		1	
ASV_16s_1743	3.876264832	0.603832413	2.236403832	9.468211063		0.220764242		0.907419746	

ASV_16s_1744	0.816736388	-6.609873296	3.550662441	0.081803711	NA	NA
ASV_16s_1745	3.207149803	-2.683571888	2.78184082	3.675198948	0.816335285	1
ASV_16s_1746	2.84162373	-1.844463013	2.57369732	1.823849837	0.968943525	1
ASV_16s_1747	4.032277695	0.94405686	2.920982895	4.392201226	0.733656151	1
ASV_16s_1748	4.988421885	-1.539874456	3.550121793	2.47407762	NA	NA
ASV_16s_1749	4.792341706	2.810029894	3.546034922	1.496657302	NA	NA
ASV_16s_1750	2.624509661	3.907603442	2.42457108	3.073234523	0.878145762	1
ASV_16s_1751	3.695963383	0.399954452	3.550019464	1.292225203	0.988646172	1
ASV_16s_1752	2.896477934	10.26731069	3.547186786	2.574768458	0.921362488	1
ASV_16s_1753	3.778268227	0.959480472	2.65930351	6.765645041	0.453682023	1
ASV_16s_1754	3.677565325	0.94394031	3.549383407	1.970897879	0.961433511	1
ASV_16s_1755	3.250497826	-0.34702614	2.194015321	10.03823178	0.186417241	0.803223968
ASV_16s_1756	3.196170883	0.57786087	2.762936983	7.482192699	0.380455247	1
ASV_16s_1757	2.977450045	3.04365458	2.671121952	5.795525295	0.563817557	1
ASV_16s_1758	3.534421235	1.7530131	3.100459703	4.19031896	0.757614047	1
ASV_16s_1759	1.466206787	-21.05855265	3.551447436	1.633981201	0.977305628	NA
ASV_16s_1760	2.844985884	-8.162738042	3.549331308	2.851031774	0.898432481	1
ASV_16s_1761	2.556103828	2.339331534	3.545735971	3.545906169	NA	NA
ASV_16s_1762	3.713368712	-0.958489891	2.555649185	6.086338391	0.529705068	1
ASV_16s_1763	3.83304434	-0.230054788	3.545925044	3.043135465	0.880983367	1
ASV_16s_1764	3.961132076	17.8957096	3.550749769	0.864177485	0.996732514	1
ASV_16s_1765	3.503678087	-0.153467657	3.269362267	5.041217517	0.654933123	1
ASV_16s_1766	3.016820671	-26.76910157	3.551452209	2.027501325	0.958302549	1
ASV_16s_1767	4.028944097	0.730618391	3.53947439	3.62865848	0.821416213	1
ASV_16s_1768	0.5066191	-22.36090992	3.551441481	-0.143629019	NA	NA
ASV_16s_1769	2.818472073	-8.254586985	3.549399798	2.517504847	0.925773174	1
ASV_16s_1770	3.813941063	-0.455886972	2.705320564	7.610724096	0.368167033	1
ASV_16s_1771	3.36877222	1.790898493	1.638568286	14.54140646	0.042348397	0.274867208
ASV_16s_1772	0.940678056	-21.8418254	3.550621882	0.06678551	NA	NA
ASV_16s_1773	3.21480407	-1.088425802	2.350514622	5.245740216	0.630002158	1
ASV_16s_1774	3.116404765	-10.10079543	3.548000013	2.784127112	0.904228405	1
ASV_16s_1775	3.957676927	0.492983644	3.180390176	6.566916396	0.475318909	1
ASV_16s_1776	3.901084335	-2.568622744	3.549836177	2.330300499	0.9393247	1
ASV_16s_1777	3.389581816	-0.868606931	2.922715305	4.088725313	0.769503171	1
ASV_16s_1778	3.687960349	-14.34322222	3.552157344	1.138641592	0.992271737	1
ASV_16s_1779	3.382874231	-0.799620584	3.227379874	3.760664818	0.806894536	1
ASV_16s_1780	2.887652179	-1.153953635	2.662120399	2.825545615	0.900658025	1
ASV_16s_1781	3.55571428	0.088519718	3.547950837	4.206322239	0.755730128	1
ASV_16s_1782	1.832600491	-14.42871048	3.552157344	0.655658437	0.998653724	NA
ASV_16s_1783	2.638678153	1.668108878	2.041034394	5.450223729	0.605194426	1
ASV_16s_1784	2.288621409	-4.861808499	3.547673584	1.477582053	0.983075635	1
ASV_16s_1785	0.933385978	-22.02889104	3.550622242	0.06249786	NA	NA
ASV_16s_1786	3.31942007	-0.312053931	2.946142421	6.693772031	0.461445102	1
ASV_16s_1787	2.644146801	3.597655964	3.450990232	3.026488872	0.882541024	1
ASV_16s_1788	3.122446258	-1.667357569	3.549516886	0.925473644	NA	NA
ASV_16s_1789	2.915596272	0.466088635	3.549339006	0.830034619	NA	NA
ASV_16s_1790	4.140114494	0.849802127	3.539515216	3.568110403	0.827959539	1
ASV_16s_1791	3.281106736	-0.770741927	2.903189219	4.058760383	0.772985035	1
ASV_16s_1792	2.608211631	2.659945643	2.21060922	3.860070928	0.795746154	1
ASV_16s_1793	3.561691329	1.187328951	3.158605645	2.782812918	0.904340742	1
ASV_16s_1794	1.803347638	-13.17741935	3.552157344	0.645788045	0.998718491	NA
ASV_16s_1795	2.794417213	3.270376139	2.945433876	2.483527624	0.928332335	1
ASV_16s_1796	3.342271241	0.147412726	1.987879973	12.52364089	0.08460112	0.464442884
ASV_16s_1797	3.422608772	-0.799429352	3.212314641	4.027022628	0.776659719	1
ASV_16s_1798	2.697332784	9.177376196	3.547820525	2.667774773	0.913942817	1
ASV_16s_1799	2.955765159	2.335634825	2.458916036	6.135161137	0.52405905	1
ASV_16s_1800	3.177089065	0.077790943	1.528215636	12.01602586	0.100033079	0.524199319
ASV_16s_1801	3.617435833	0.857243871	3.546105483	4.308967502	NA	NA
ASV_16s_1802	4.02123382	-17.80127936	3.552157344	0.349830364	NA	NA
ASV_16s_1803	2.867521918	-1.210944932	3.545776127	2.840273916	NA	NA
ASV_16s_1804	0.551361388	-0.170366648	3.550554409	1.316534049	0.987991752	NA
ASV_16s_1805	2.957964509	-1.302239537	2.682794928	2.763493719	0.905985337	1
ASV_16s_1806	3.637527635	4.768761641	2.589082726	2.216474052	0.946894017	1
ASV_16s_1807	2.02022543	-9.227482324	3.549548128	1.041485873	NA	NA
ASV_16s_1808	3.654398767	0.249792669	3.547962689	2.249539187	0.944748556	1
ASV_16s_1809	0.861890025	-12.60327427	3.552157344	0.193034624	NA	NA
ASV_16s_1810	2.696808915	10.12862261	3.546820834	2.229025094	0.946084808	1

ASV_16s_1811	3.120131456	1.009121769	2.4390043	7.149291109	0.413503155	1
ASV_16s_1812	3.688918363	1.282527019	1.467010402	14.53320492	0.042470912	0.274925339
ASV_16s_1813	3.202476399	-0.248168951	2.698394864	2.983403151	0.886533318	1
ASV_16s_1814	0.96609018	-14.23281284	3.552157344	0.262978878	0.999936	NA
ASV_16s_1815	1.137685563	-11.95778404	3.552157344	0.363234851	NA	NA
ASV_16s_1816	4.302286435	0.383869715	2.776569201	6.222472831	0.514025983	1
ASV_16s_1817	2.627914063	0.317907865	2.363587882	6.464642386	0.486658775	1
ASV_16s_1818	2.514419946	-23.43824656	3.551440881	0.913880921	0.996100767	1
ASV_16s_1819	3.628142876	0.315939287	3.550607018	2.415052018	0.933356759	1
ASV_16s_1820	3.269803479	1.325632593	3.035138154	5.090869498	0.64887489	1
ASV_16s_1821	2.455528155	2.394790337	3.54465248	2.184532505	NA	NA
ASV_16s_1822	2.930633718	0.562843805	2.399545943	2.367732463	0.936723398	1
ASV_16s_1823	2.999543341	2.671080905	1.713806484	8.832331475	0.264919236	1
ASV_16s_1824	3.852423282	-0.306134198	3.546326858	2.641410059	NA	NA
ASV_16s_1825	3.521336815	17.70977872	3.550590497	0.698189882	0.998349606	1
ASV_16s_1826	2.773116807	-10.03182574	3.549120307	2.016330454	0.958930984	1
ASV_16s_1827	3.287414514	-30	3.549887725	2.18008027	0.949204483	1
ASV_16s_1828	3.087868541	-0.456017932	1.738125214	13.05028003	0.070892763	0.41279216
ASV_16s_1829	0.863540044	0.291659516	3.54987334	3.073328891	0.878136823	NA
ASV_16s_1830	2.667320705	1.876520748	3.364480883	4.010713731	0.77854257	1
ASV_16s_1831	3.046302213	-0.279203556	2.808853945	6.227038866	0.513503617	1
ASV_16s_1832	3.610862048	11.20348403	3.547752913	2.399937391	0.934441555	1
ASV_16s_1833	3.107712227	1.056358551	2.263342504	3.630617076	0.821203274	1
ASV_16s_1834	3.582638657	1.334087036	2.321451409	8.479772654	NA	NA
ASV_16s_1835	2.844171275	0.362661812	2.23030337	4.488144528	0.722144399	1
ASV_16s_1836	3.617527194	0.694121679	3.550572771	2.347881773	0.938109748	1
ASV_16s_1837	3.078854588	-0.712598092	1.757941673	12.8451667	0.075971949	0.43074494
ASV_16s_1838	3.278898375	-0.797372622	3.547618343	3.260988976	0.859854975	1
ASV_16s_1839	3.211845197	-2.525321729	3.546013531	2.818089804	0.901304998	1
ASV_16s_1840	3.320603043	-0.416051009	1.285252604	22.48059676	0.002098194	0.023408884
ASV_16s_1841	1.556913205	2.609734851	3.54677309	0.631525204	0.998808343	NA
ASV_16s_1842	3.103458015	-0.762430982	2.577494852	5.925949148	0.548422093	1
ASV_16s_1843	3.579961337	0.363516111	3.549507302	1.861268903	0.967118122	1
ASV_16s_1844	2.758323075	-23.31467702	3.550601058	2.443782928	0.931270442	1
ASV_16s_1845	1.035010067	0.074133582	3.550571908	3.176629298	0.868195916	NA
ASV_16s_1846	2.635144989	1.530564969	3.54509188	3.326282507	0.853268374	1
ASV_16s_1847	3.337908081	-0.931010157	2.898601127	4.33083615	0.740979911	1
ASV_16s_1848	2.555132868	3.212761955	3.421321183	3.437366219	0.84181438	1
ASV_16s_1849	3.182976273	-0.504519755	1.883722886	10.95712912	0.140499991	0.663061361
ASV_16s_1850	1.625343904	-0.882970272	3.549012773	1.276326322	0.989062041	NA
ASV_16s_1851	2.060081615	-0.551162587	2.858701858	2.181014477	0.949145845	1
ASV_16s_1852	1.771907617	-15.2880032	3.552157344	0.634965663	0.998787069	NA
ASV_16s_1853	2.554280756	-23.19881969	3.550599611	2.29459978	NA	NA
ASV_16s_1854	2.197444457	2.005249777	3.547380366	2.42494105	NA	NA
ASV_16s_1855	2.295548308	0.776710059	3.546902942	3.889431448	NA	NA
ASV_16s_1856	4.006735631	12.46014303	3.549464249	1.259572745	0.989489951	1
ASV_16s_1857	3.213848232	-0.661730779	2.316638236	6.967673441	0.432253445	1
ASV_16s_1858	2.829191059	2.203726839	2.171210015	2.817852808	0.901325532	1
ASV_16s_1859	1.780693941	18.31734711	3.551441409	0.556709022	NA	NA
ASV_16s_1860	2.932022451	-10.76764655	3.54874444	2.068490917	NA	NA
ASV_16s_1861	2.002516319	21.08422275	3.551444139	0.954457964	NA	NA
ASV_16s_1862	2.736117481	-11.67916082	3.549207898	2.003814968	NA	NA
ASV_16s_1863	4.118032274	0.415900965	3.175061593	4.471413884	0.724156736	1
ASV_16s_1864	2.676856197	2.061874268	3.360306977	4.168328764	0.760197992	1
ASV_16s_1865	3.222061961	-0.984861314	2.553982621	5.955109073	0.545000743	1
ASV_16s_1866	1.98525163	-29.21509813	3.549698006	0.986415083	0.995044713	NA
ASV_16s_1867	3.465630011	0.107525132	2.815343598	3.884194808	0.793015183	1
ASV_16s_1868	2.43552595	2.068114711	2.835819909	3.399858027	0.845715805	1
ASV_16s_1869	3.986317535	0.132411852	3.143076528	3.866220346	0.795050915	1
ASV_16s_1870	3.37986528	-0.108812996	3.548996505	2.348885458	0.938040022	1
ASV_16s_1871	0.894008757	-21.95743469	3.550624343	0.038750695	NA	NA
ASV_16s_1872	3.356751376	0.021590474	3.550492506	4.735768368	0.692171953	1
ASV_16s_1873	3.309053447	-0.83729577	3.261801937	3.403753726	0.845312173	1
ASV_16s_1874	2.864876299	-1.028040086	2.601207913	5.085632892	0.649513728	1
ASV_16s_1875	1.881785572	0.007303809	3.550367618	2.057260452	0.956603202	NA
ASV_16s_1876	3.190735623	3.916661305	2.813945525	1.847777022	NA	NA
ASV_16s_1877	2.520220326	1.818733439	2.523443112	2.525297932	0.925180088	1

ASV_16s_1878	2.27995683	0.669397116	3.387224352	0.837728949	0.997039581	1
ASV_16s_1879	3.350086733	1.67367413	3.548389913	2.810425932	0.901968081	1
ASV_16s_1880	3.648012464	0.914025238	3.549290358	2.94075028	0.890428659	1
ASV_16s_1881	3.340357622	0.099465047	3.201822045	5.886184356	0.553100257	1
ASV_16s_1882	3.73533064	0.363503361	3.550424812	1.168707452	0.991629644	1
ASV_16s_1883	3.10769563	1.233918304	2.533180128	7.269535109	0.401365874	1
ASV_16s_1884	1.73308522	-15.3774852	3.552157344	0.621372967	0.998869659	NA
ASV_16s_1885	3.328824619	-14.5201006	3.552157344	1.075713158	0.993513	1
ASV_16s_1886	3.717139502	17.8380718	3.550749179	0.82419068	0.99718917	1
ASV_16s_1887	3.177995757	-0.796866714	3.547730685	3.276420631	0.858308369	1
ASV_16s_1888	2.296931765	-7.219023389	3.547958104	1.841420295	0.968093679	1
ASV_16s_1889	3.287565507	-1.058658515	2.476007431	5.047707277	0.654141181	1
ASV_16s_1890	3.626741823	0.017740352	3.413259316	2.300943243	0.941326268	1
ASV_16s_1891	3.806943968	2.107595656	3.549209125	3.871945155	0.794403118	1
ASV_16s_1892	3.074588679	1.753512484	3.54288905	2.630436986	NA	NA
ASV_16s_1893	4.187067226	-3.249739392	3.544135332	1.949801689	NA	NA
ASV_16s_1894	3.22621357	-0.151287546	2.651048287	4.22749486	0.753233305	1
ASV_16s_1895	3.565169077	-9.03586608	3.550052565	3.906851158	0.790441709	1
ASV_16s_1896	3.20506174	-0.797314082	3.547630911	3.248590993	0.86109294	1
ASV_16s_1897	3.453327007	0.213790902	2.592194892	7.929897364	0.338816097	1
ASV_16s_1898	2.823482019	0.026549197	3.416931272	3.292257872	0.856714575	1
ASV_16s_1899	3.815686105	0.459224479	2.493088518	6.48806307	0.484050088	1
ASV_16s_1900	3.508364054	0.267219342	3.029910797	6.035670627	0.535590507	1
ASV_16s_1901	3.111075004	-0.005958633	2.39250265	5.064453145	0.652097811	1
ASV_16s_1902	2.678567433	0.775117487	2.627108623	4.247333537	0.750889399	1
ASV_16s_1903	3.824876644	0.624417364	2.577455348	10.80452982	0.14737552	0.679611685
ASV_16s_1904	2.946879013	-24.91151077	3.54992328	2.051153626	0.956954907	1
ASV_16s_1905	1.236246179	0.248851309	3.545223136	1.155734289	0.99191065	NA
ASV_16s_1906	3.227141421	-0.933206862	2.744195091	4.41233526	0.731246248	1
ASV_16s_1907	3.302388586	-0.343116099	2.201302681	7.213530906	0.406991041	1
ASV_16s_1908	2.142411494	0.180811789	3.544055798	0.53081337	NA	NA
ASV_16s_1909	3.730656152	0.965638041	2.727335617	5.10240239	0.647468048	1
ASV_16s_1910	0.877966186	-21.92830916	3.550625245	0.028775286	NA	NA
ASV_16s_1911	2.82050767	-24.78061431	3.54996488	2.176439306	0.949432678	1
ASV_16s_1912	1.709405287	13.86727606	3.551469233	0.690159265	0.998410211	NA
ASV_16s_1913	1.966970779	-1.249494007	2.973383308	2.939438579	0.890547543	NA
ASV_16s_1914	2.992722403	-11.30877611	3.552157344	0.701626239	NA	NA
ASV_16s_1915	3.798560045	0.242539099	3.546949886	1.615373833	NA	NA
ASV_16s_1916	2.48412345	-7.868199643	3.549402537	2.454573193	NA	NA
ASV_16s_1917	3.231234306	-0.841009623	2.87999326	4.387152977	0.73425987	1
ASV_16s_1918	3.644263124	1.102934006	3.549419041	2.389408763	0.935191983	1
ASV_16s_1919	3.389334272	1.645039489	3.549325738	2.663704715	0.914274028	1
ASV_16s_1920	3.149648556	-0.799379975	3.199012571	3.978803503	0.782215576	1
ASV_16s_1921	3.048648798	-12.67881318	3.545590351	1.257015134	0.989554349	1
ASV_16s_1922	1.976489585	2.308087355	2.907447781	3.940044315	0.786656687	NA
ASV_16s_1923	2.666087387	-7.734299959	3.54930408	2.796499765	0.903167919	1
ASV_16s_1924	3.105749837	0.674946613	3.172187916	5.687934684	0.576622695	1
ASV_16s_1925	3.178739721	-0.741537747	2.857679921	4.998391952	0.660159478	1
ASV_16s_1926	0.771855245	-22.827651	3.551440118	0.114277869	NA	NA
ASV_16s_1927	1.024556534	0.113771211	3.550580366	3.029318295	0.882276861	NA
ASV_16s_1928	2.925779193	-0.519792668	2.766496966	6.149265106	0.522432725	1
ASV_16s_1929	3.43096384	1.261098939	3.546553822	2.908559836	0.893330332	1
ASV_16s_1930	3.209756879	0.108866604	2.902004969	3.755614739	0.807456204	1
ASV_16s_1931	2.623553248	-9.569717589	3.549686354	1.837305907	0.96829384	1
ASV_16s_1932	2.933950878	-0.488937095	2.826742359	4.905668796	0.67147383	1
ASV_16s_1933	2.776113592	-1.380232111	2.453196416	4.987607073	0.661475677	1
ASV_16s_1934	4.913472018	-26.85240569	3.549041464	0.46696457	NA	NA
ASV_16s_1935	3.624905428	-0.24468315	1.983710279	7.302189922	0.398108534	1
ASV_16s_1936	2.521919725	-1.378164631	3.309315351	2.548554282	0.923396716	1
ASV_16s_1937	2.922768197	-8.264619913	3.545167391	0.907337548	0.996188108	1
ASV_16s_1938	3.200156623	1.015871177	1.847246523	13.89145691	0.05314592	0.333332311
ASV_16s_1939	1.592364312	-0.409031668	3.546674614	2.854961931	0.898087368	NA
ASV_16s_1940	2.605710832	-22.90684729	3.550604766	2.398030474	0.934577789	1
ASV_16s_1941	2.740681448	2.158550277	3.544282175	3.080908563	0.877417967	1
ASV_16s_1942	2.993195849	-1.172576494	3.376665415	3.909788361	0.790107477	1
ASV_16s_1943	3.009606646	-0.533169089	1.763368748	12.03022705	0.099569207	0.523289732
ASV_16s_1944	3.413046178	0.32150857	3.549617047	4.139896844	0.763530449	1

ASV_16s_1945	2.407435533	-8.944847947	3.548428505	1.885674904	NA	NA
ASV_16s_1946	2.399232197	1.131787869	2.895155863	2.468397279	NA	NA
ASV_16s_1947	0.810921032	-10.36831065	3.549640517	0.015573538	NA	NA
ASV_16s_1948	2.925906747	-1.285553065	2.236671643	4.220118196	0.754103768	1
ASV_16s_1949	5.612297025	18.25550779	3.550586821	0.976825604	NA	NA
ASV_16s_1950	0.85025629	-21.8898321	3.550626906	0.011108681	NA	NA
ASV_16s_1951	2.65947829	0.149075664	3.547678617	4.704544172	0.695967114	1
ASV_16s_1952	3.075639029	-0.799353407	3.193085454	3.983626911	0.781661331	1
ASV_16s_1953	2.840879841	-1.775130032	2.027601022	5.635049161	0.582948295	1
ASV_16s_1954	3.32243562	0.23359886	3.066445222	4.261320874	0.749234334	1
ASV_16s_1955	0.479561093	-0.17033493	3.550561657	1.245639537	0.989837814	NA
ASV_16s_1956	2.923737031	-1.931635426	2.005224618	5.45362865	0.604782869	1
ASV_16s_1957	2.082309719	-7.553541463	3.5498997	1.758528682	NA	NA
ASV_16s_1958	0.455643494	-22.53353524	3.551441932	-0.208542514	NA	NA
ASV_16s_1959	2.746115682	-8.220366276	3.549233296	2.82710545	0.900522436	1
ASV_16s_1960	2.612454859	-5.228690275	3.552157344	0.206910939	NA	NA
ASV_16s_1961	2.115707639	-0.196240308	3.545910469	3.051293286	NA	NA
ASV_16s_1962	2.626415573	-0.953216256	3.546400795	2.022641201	0.958576598	1
ASV_16s_1963	3.593405906	-0.077473102	2.654341519	5.613810026	0.585494081	1
ASV_16s_1964	3.746030623	0.861049379	3.549217006	3.432103445	0.842363824	1
ASV_16s_1965	2.563674097	1.450606707	3.543324838	2.502886001	0.92687959	1
ASV_16s_1966	3.059778883	-0.712393745	2.704463069	6.399286164	0.493974844	1
ASV_16s_1967	2.606685785	1.742833341	2.699607952	2.731248695	0.908701699	1
ASV_16s_1968	1.673092274	-29.18975494	3.551421433	0.345832153	0.999838349	NA
ASV_16s_1969	2.661318838	8.907923603	3.547112142	1.569290105	NA	NA
ASV_16s_1970	2.877993467	1.604542634	2.147699181	5.123986252	0.644835588	1
ASV_16s_1971	3.060975131	-0.960087333	2.903383316	4.094735224	0.768803415	1
ASV_16s_1972	3.1823341	-2.425124765	3.139609646	3.132762113	0.872454969	1
ASV_16s_1973	3.620658128	-0.12691314	2.677420572	5.155104473	NA	NA
ASV_16s_1974	2.645314966	-5.085468281	3.549633594	1.564661427	NA	NA
ASV_16s_1975	2.901021449	0.534880525	2.836747967	6.367635479	0.497536817	1
ASV_16s_1976	3.313019198	1.059585626	3.548141735	3.061601328	0.8792457	1
ASV_16s_1977	3.055016544	-0.799514079	3.204129548	3.873362729	0.794242627	1
ASV_16s_1978	3.200008683	0.722135841	3.549727821	2.812092678	0.901824041	1
ASV_16s_1979	2.48228087	26.61412248	3.547200208	-0.373152063	NA	NA
ASV_16s_1980	3.341014341	0.547071234	2.773905175	7.614859683	0.367776085	1
ASV_16s_1981	3.062546514	-0.797298834	3.547638502	3.202902001	0.865619182	1
ASV_16s_1982	2.936991509	-0.95059363	2.749604885	4.448957458	0.726854622	1
ASV_16s_1983	2.938587276	-5.560450675	3.5493775	2.898301468	0.894248038	1
ASV_16s_1984	2.96633596	-18.87667604	3.551799395	0.6788169	0.998493256	1
ASV_16s_1985	0.938179404	0.162661309	3.550576173	4.129531482	0.76474293	NA
ASV_16s_1986	2.566667379	1.224443206	3.311070398	4.533328626	0.716700317	1
ASV_16s_1987	2.693223961	1.052011728	3.544913894	3.350440959	0.850803449	1
ASV_16s_1988	2.811484241	1.438867552	3.545346821	3.821759438	0.800063199	1
ASV_16s_1989	2.901914112	-24.35691845	3.088953161	2.853135565	0.898247808	1
ASV_16s_1990	2.893260284	-0.157341639	1.81520518	8.4535637	0.294301324	1
ASV_16s_1991	2.783950015	-0.683258557	3.40845228	3.171200756	0.868725925	1
ASV_16s_1992	2.680843878	-0.511111631	1.976030953	10.82947324	0.146232219	0.676918169
ASV_16s_1993	3.089803453	-0.917832663	3.236596429	3.526659301	0.832393745	1
ASV_16s_1994	2.506849193	-1.464351237	2.913709201	3.239168846	0.862031	1
ASV_16s_1995	4.799871256	-25.10235396	3.549040847	0.453014117	NA	NA
ASV_16s_1996	2.154446108	11.37524592	3.043804779	0.878059628	NA	NA
ASV_16s_1997	3.056509959	-0.799429813	3.200539153	3.92053768	0.788883123	1
ASV_16s_1998	3.001886544	-0.865304843	3.268213003	3.222824611	0.863652518	1
ASV_16s_1999	2.942900381	-0.797129293	3.547689582	3.198923178	0.866010643	1
ASV_16s_2000	0.832755303	-21.6817845	3.55062803	-3.48E-04	NA	NA
ASV_16s_2001	3.062711674	-0.821752975	3.546781216	2.68334528	0.912670289	1
ASV_16s_2002	3.000700607	-0.651434958	2.352117774	5.53661897	0.594770682	1
ASV_16s_2003	3.170439147	0.389342708	1.896268466	5.428701693	0.607797144	1
ASV_16s_2004	2.270742887	-2.892581151	2.925337767	3.509963346	0.834169052	1
ASV_16s_2005	3.306743241	0.370892019	3.550399638	1.559284247	0.98018598	1
ASV_16s_2006	3.174703112	-3.309932653	2.739486515	2.852445931	0.898308361	1
ASV_16s_2007	3.531515535	0.407035592	2.826123279	8.006468463	0.332023865	1
ASV_16s_2008	3.30757133	0.368326258	3.547276231	2.125315295	0.952579853	1
ASV_16s_2009	3.240677521	0.361096385	1.945468387	11.19166521	0.130474434	0.627557169
ASV_16s_2010	3.208610535	0.93145618	3.549561347	1.917243621	0.964278308	1
ASV_16s_2011	2.603589382	-29.63308674	3.549322638	1.726532151	0.97341706	1

ASV_16s_2012	3.255147169	3.807661561	1.827859743	7.501979984	0.378546159	1
ASV_16s_2013	2.89554099	-0.936028234	3.548466526	1.663184182	NA	NA
ASV_16s_2014	1.79767677	15.1430339	3.551446379	1.226641873	0.990300482	NA
ASV_16s_2015	2.083113261	17.82984004	3.550738917	1.849226839	NA	NA
ASV_16s_2016	2.682127565	-10.18113055	3.548772287	2.298026743	0.941523252	1
ASV_16s_2017	2.020024463	3.991426129	3.546127187	0.873983138	NA	NA
ASV_16s_2018	3.36321493	0.343219214	3.549258571	3.619705407	0.82238859	1
ASV_16s_2019	2.618637367	8.553392353	3.549608512	1.675230418	0.975616428	1
ASV_16s_2020	2.073274778	20.40540068	3.296782254	0.616061378	0.998900879	1
ASV_16s_2021	3.154482099	0.67870997	3.550578024	3.162069636	0.86961556	1
ASV_16s_2022	1.755030326	17.06100069	3.551445392	0.58880004	NA	NA
ASV_16s_2023	0.652314193	-22.73597733	3.5514406	0.011145459	NA	NA
ASV_16s_2024	2.991704502	-0.819557543	3.547556448	2.634559525	0.916628484	1
ASV_16s_2025	2.538096902	-0.92607849	1.904292509	6.188324011	0.517940087	1
ASV_16s_2026	2.422854892	0.767183045	3.547433581	3.952218642	0.78526417	1
ASV_16s_2027	2.731658658	-2.292213701	1.954052915	3.595150069	0.825046952	1
ASV_16s_2028	3.035071942	2.798837269	3.546772118	2.275881909	0.943007915	1
ASV_16s_2029	0.441527172	-22.28563344	3.551442064	-0.227799142	NA	NA
ASV_16s_2030	3.587725308	-1.506856509	2.220088271	3.799191947	0.802594329	1
ASV_16s_2031	2.07007873	0.248883404	2.861310231	4.606790996	0.707822604	1
ASV_16s_2032	0.74058836	-5.343415732	3.551067075	0.413843742	NA	NA
ASV_16s_2033	2.877142829	-0.796706414	3.54776668	3.19354658	0.866538929	1
ASV_16s_2034	1.706735013	13.82796235	3.551490024	1.531265135	0.98120748	NA
ASV_16s_2035	3.374859502	0.980079487	3.256313785	1.547810713	0.980608142	1
ASV_16s_2036	2.358640966	1.679251883	2.285503463	6.309325979	0.504130595	1
ASV_16s_2037	3.260850947	-0.122938823	2.076485998	11.98496746	0.101054307	0.527771404
ASV_16s_2038	0.374825873	-0.059358648	3.55061242	1.291878249	NA	NA
ASV_16s_2039	2.412018879	2.850028235	3.5446616	3.164824136	NA	NA
ASV_16s_2040	2.274596472	0.778891459	3.550476223	1.657102246	0.976367473	1
ASV_16s_2041	3.119743848	2.167694964	2.021107184	5.572163277	0.590494488	1
ASV_16s_2042	3.404461562	3.274664436	3.548865805	2.758395503	0.906417203	1
ASV_16s_2043	2.452928325	-0.041731546	3.547675046	4.300028229	0.744643911	1
ASV_16s_2044	3.798802981	0.206114076	2.823007982	5.487234864	0.600724032	1
ASV_16s_2045	0.641923347	-22.68181324	3.551440645	0.00130469	NA	NA
ASV_16s_2046	0.761702171	-13.83969321	3.552157344	0.117283723	NA	NA
ASV_16s_2047	2.506317287	-7.983169523	3.549316174	2.606365932	0.918876947	1
ASV_16s_2048	2.509257568	-9.761352337	3.548896848	2.328192593	0.93946955	1
ASV_16s_2049	2.986375278	-0.797330197	3.547640603	3.174010663	0.868451687	1
ASV_16s_2050	3.228870148	-0.044362068	2.570110409	8.121363387	0.322014029	1
ASV_16s_2051	2.415460462	-21.9468278	3.550606085	2.307773414	0.940863631	1
ASV_16s_2052	2.618234745	-1.61334698	3.283457692	2.807402999	0.902229084	1
ASV_16s_2053	2.948921397	-0.216719056	3.548232423	1.964492983	NA	NA
ASV_16s_2054	2.786115006	-1.240979838	3.364204425	2.064434599	0.95618806	1
ASV_16s_2055	2.820434242	0.325644933	2.55150378	7.291879127	0.39913524	1
ASV_16s_2056	1.872223741	0.869794325	3.54877134	4.323017125	0.741910675	NA
ASV_16s_2057	2.765313894	-8.587396508	3.552157344	0.658014995	NA	NA
ASV_16s_2058	3.125989771	-0.095827446	3.549036062	2.075651686	0.955534714	1
ASV_16s_2059	2.88175885	-0.797343887	3.547641119	3.126394494	0.873068633	1
ASV_16s_2060	2.894964911	-0.823398874	2.90707282	4.09355936	0.768940362	1
ASV_16s_2061	2.915105668	-1.526562123	2.367726866	4.512577533	0.719202202	1
ASV_16s_2062	3.082374984	0.96870389	3.548964512	2.336524034	0.938896013	1
ASV_16s_2063	2.769403213	0.425086181	3.012893696	5.010349174	0.658700203	1
ASV_16s_2064	2.226600224	2.948372058	3.544617737	2.986685027	0.886231236	1
ASV_16s_2065	2.845731778	0.022379646	2.533547118	5.814229394	0.561600767	1
ASV_16s_2066	2.37318238	-24.82445201	3.551447276	1.839127648	0.968205301	1
ASV_16s_2067	3.735343943	0.344940225	3.550727587	3.283184704	0.857628468	1
ASV_16s_2068	2.769089112	-0.228172571	1.851074941	9.784903995	0.201093982	0.847116199
ASV_16s_2069	3.504085815	17.82244202	3.550335878	0.673434031	0.998531634	1
ASV_16s_2070	2.345811873	-8.030209275	3.549414203	2.287601874	0.942224599	1
ASV_16s_2071	2.708932573	0.532301989	3.370498238	3.984704196	0.781537496	1
ASV_16s_2072	2.921262579	-0.799330876	3.190454648	3.916773107	0.789312118	1
ASV_16s_2073	3.26505271	1.169813238	2.147454256	8.53346486	0.287907685	1
ASV_16s_2074	2.404084051	17.40597421	3.551454404	1.21814463	0.990503103	1
ASV_16s_2075	3.40599072	0.288103531	3.106972057	3.593392401	0.825236752	1
ASV_16s_2076	4.19432579	-0.935698938	3.548929737	2.78579776	0.904085514	1
ASV_16s_2077	3.345503977	0.583846183	3.059172096	7.407485858	0.387719473	1
ASV_16s_2078	2.356658559	-7.983320745	3.549318392	2.490261519	0.927828595	1

ASV_16s_2079	2.792770194	-0.881467577	2.579568257	6.107961246	0.527201444	1
ASV_16s_2080	3.092497276	-0.981235507	2.50680125	3.020623011	0.883087899	1
ASV_16s_2081	2.294108802	-1.741056678	2.584832813	2.214843458	0.946998681	1
ASV_16s_2082	2.049836968	-30	3.549949785	1.645120972	0.976856383	1
ASV_16s_2083	0.796294913	-21.80908196	3.550630448	-0.02500693	NA	NA
ASV_16s_2084	0.660527821	-22.59520777	3.551440558	0.018804953	0.999999993	NA
ASV_16s_2085	2.992744364	0.312392603	2.367461149	9.581230103	0.213572388	0.88084966
ASV_16s_2086	3.192691038	-0.831079517	2.750670518	2.951678343	0.889436091	1
ASV_16s_2087	2.518662544	-5.076208528	3.550730112	-0.21434705	NA	NA
ASV_16s_2088	0.879625855	-0.853171563	3.548769535	1.236183541	0.990069777	NA
ASV_16s_2089	2.446885425	-23.29687137	3.550592761	0.593739783	0.999025749	1
ASV_16s_2090	2.317179216	-0.071715676	3.54696949	3.061606865	0.879245178	1
ASV_16s_2091	3.005257538	-0.924643073	2.674892891	3.06452244	0.878969883	1
ASV_16s_2092	2.622908757	0.217483309	3.549567673	1.387393175	0.985954273	1
ASV_16s_2093	1.560607031	0.345674288	3.05373109	3.704648288	0.813098017	NA
ASV_16s_2094	2.47793123	0.460224914	3.549492787	0.809821118	0.997342429	1
ASV_16s_2095	3.16287378	-24.33692585	3.550589544	0.74057598	NA	NA
ASV_16s_2096	1.675237434	17.91463178	3.551442617	1.933467364	0.963430766	NA
ASV_16s_2097	0.798118829	-23.99484547	3.549554349	0.340672044	NA	NA
ASV_16s_2098	2.332148289	0.766882922	3.546321396	3.325533269	0.853344582	1
ASV_16s_2099	2.801694219	1.058985296	2.311880514	4.607099458	0.707785265	1
ASV_16s_2100	3.114110164	0.09179916	2.610462787	7.319415382	0.396397016	1
ASV_16s_2101	0.42348965	-22.01800119	3.551442259	-0.253316914	NA	NA
ASV_16s_2102	2.982709326	-30	3.549892679	2.096833078	0.954286863	1
ASV_16s_2103	2.684244181	4.7531903	3.12391708	1.61206105	0.978174667	1
ASV_16s_2104	2.364514493	0.839664724	2.980644621	5.205503061	0.634900611	1
ASV_16s_2105	2.291932335	11.42876133	3.545629682	0.712698865	NA	NA
ASV_16s_2106	1.674410559	16.95883407	3.551445818	0.559910707	NA	NA
ASV_16s_2107	3.035295577	0.694103906	3.550576714	2.258353753	0.944169207	1
ASV_16s_2108	2.329258792	0.277899304	3.296066843	4.16430963	0.760669651	1
ASV_16s_2109	2.594821678	0.166209528	2.959861853	5.658842584	0.580099965	1
ASV_16s_2110	2.049383676	4.638312417	3.545374691	0.978434077	NA	NA
ASV_16s_2111	2.330422065	-21.80284011	3.550601142	2.167955322	0.949962317	1
ASV_16s_2112	2.511087877	0.35414277	3.549768743	1.326717563	0.987710885	1
ASV_16s_2113	2.501235647	1.936052009	3.451463015	3.071927454	0.878269548	1
ASV_16s_2114	2.856174594	-0.797451246	3.547620056	3.044890167	0.880818687	1
ASV_16s_2115	2.782650465	-0.024852188	2.452087948	7.972742965	0.335003545	1
ASV_16s_2116	1.629573204	-25.87501472	3.551565427	0.728410714	0.998107791	NA
ASV_16s_2117	0.759686653	-22.71206823	3.550434349	-0.129479196	NA	NA
ASV_16s_2118	2.869463874	-1.515303413	3.387095042	3.464656624	0.838954716	1
ASV_16s_2119	2.775769183	1.409167956	3.547535742	2.798946638	NA	NA
ASV_16s_2120	2.497914751	-0.35866822	3.54658434	3.776291716	0.805153566	1
ASV_16s_2121	2.564387923	0.178794353	3.54588025	3.853763166	0.796458639	1
ASV_16s_2122	2.380233671	0.039215579	3.547726072	4.304798002	0.744077229	1
ASV_16s_2123	2.658177798	-1.223851095	2.654795284	3.703228835	0.81325444	1
ASV_16s_2124	2.163487223	4.093449879	3.039375252	1.768498851	0.971536404	1
ASV_16s_2125	3.424190437	0.007732373	2.693435109	4.941124886	0.667148021	1
ASV_16s_2126	2.861472432	-0.667539439	2.767361433	6.005238353	0.539137827	1
ASV_16s_2127	2.844568548	0.363330558	3.550058466	1.895537939	0.965395054	1
ASV_16s_2128	2.377487506	-0.141680042	3.547655222	4.031632679	0.776126815	1
ASV_16s_2129	0.670888845	-0.134543407	3.550547848	1.788877491	0.970596666	NA
ASV_16s_2130	2.198675647	-0.135149234	3.547732233	3.792052629	NA	NA
ASV_16s_2131	2.861873749	-0.797381197	3.547636524	3.10627053	0.875000324	1
ASV_16s_2132	2.020445091	1.846703644	2.39297911	4.14410485	0.763037847	1
ASV_16s_2133	3.065806652	-0.175191694	3.549063301	2.875172023	0.896304655	1
ASV_16s_2134	3.129068112	0.540583317	3.044913805	5.294892504	0.624024711	1
ASV_16s_2135	2.305094192	-8.015408686	3.549406332	2.274371517	0.943108466	1
ASV_16s_2136	2.349467172	5.694970434	3.547824113	1.919252644	NA	NA
ASV_16s_2137	3.310134169	0.243482465	3.1052924	3.813734444	0.800964289	1
ASV_16s_2138	2.30010911	0.088286618	3.54777162	4.252551347	0.750272234	1
ASV_16s_2139	2.351293867	1.369832465	3.546843265	3.674269059	0.816437228	1
ASV_16s_2140	2.457371455	1.13777308	3.544494996	3.016674021	0.883455473	1
ASV_16s_2141	1.774845609	-0.159778916	3.340738089	2.199439502	0.947982129	NA
ASV_16s_2142	2.394069729	2.240260221	2.524778434	3.989564847	0.78097855	1
ASV_16s_2143	3.292684816	2.240758816	3.057716585	3.441080004	0.841426258	1
ASV_16s_2144	2.92366045	0.346117722	3.550611168	3.674620676	0.816398683	1
ASV_16s_2145	2.231333534	-22.62444534	3.550605324	2.186446277	0.948804205	1

ASV_16s_2146	2.191974168	-0.309144435	3.547678904	2.810774448	NA	NA	
ASV_16s_2147	2.672826382	-2.102096511	2.485611752	3.399454843	0.845757557		1
ASV_16s_2148	3.226199017	3.406444564	2.197258766	4.091936539	0.769129335		1
ASV_16s_2149	2.936983885	1.544201573	3.548596833	1.985167577	0.960656741		1
ASV_16s_2150	3.164282677	0.804867214	3.549361146	1.909126768	0.964698218		1
ASV_16s_2151	3.270608509	1.231163938	2.294383899	8.917322541	0.258646605		0.999196018
ASV_16s_2152	0.649985082	-0.016956047	3.550567819	2.754749045	0.906725544	NA	
ASV_16s_2153	2.68864357	-29.2182469	2.831961775	1.54514089	0.980705607		1
ASV_16s_2154	3.303073175	17.6948083	3.549738713	0.456741532	0.999589968		1
ASV_16s_2155	2.797212751	4.551629408	2.731868101	0.803598633	0.997407055		1
ASV_16s_2156	2.198713499	0.498978241	3.547717607	3.874993315	NA	NA	
ASV_16s_2157	2.358235117	-0.373313744	3.547619366	3.974281861	0.782734835		1
ASV_16s_2158	2.376511664	2.323060999	2.855027122	5.23500283	0.631308901		1
ASV_16s_2159	2.384167992	-0.835943658	3.268868788	2.905133131	0.893637261		1
ASV_16s_2160	2.437487628	5.171169543	3.549822	1.557225215	0.980262137		1
ASV_16s_2161	2.561830912	-0.884082569	3.546699256	2.559551232	0.922546441		1
ASV_16s_2162	2.54955137	3.204295455	2.326568317	7.103341917	0.418199915		1
ASV_16s_2163	2.738353287	0.886038354	2.36024474	6.003193568	0.539376502		1
ASV_16s_2164	2.992781889	-0.706415431	3.268588668	1.794626965	0.970328399		1
ASV_16s_2165	2.212903465	-4.711512818	3.135962261	1.793912539	NA	NA	
ASV_16s_2166	1.515853863	-15.82724619	3.552157344	0.539180564	0.999289996	NA	
ASV_16s_2167	2.675662387	-0.981153992	2.759648679	4.375615732	0.735638804		1
ASV_16s_2168	2.93249619	0.608139812	2.547692252	5.498001837	0.599424898		1
ASV_16s_2169	2.849750015	0.143055642	2.425062079	4.498458759	0.720902857		1
ASV_16s_2170	2.109358027	-0.042491115	2.908949826	3.277394994	0.858210504		1
ASV_16s_2171	2.263020027	2.502322869	2.119496571	4.475776697	0.723632172		1
ASV_16s_2172	1.618596873	16.92385049	3.551446105	0.539097149	NA	NA	
ASV_16s_2173	2.748378122	-0.797221671	3.547658476	3.089907749	0.876562279		1
ASV_16s_2174	2.67382437	-0.321944344	3.065352968	2.804448392	0.902483891		1
ASV_16s_2175	3.615783889	-8.837643594	3.552157344	0.767262981	NA	NA	
ASV_16s_2176	2.667417559	-1.603537651	2.246959991	6.986043296	0.430334474		1
ASV_16s_2177	2.724983738	-0.799031134	3.183911085	3.894407732	0.791856148		1
ASV_16s_2178	1.297957525	0.618363585	2.34613996	2.501854386	0.926957363	NA	
ASV_16s_2179	3.271821027	8.000792712	3.550154577	1.775785825	0.971202365		1
ASV_16s_2180	2.701616734	-0.909330958	2.934620503	3.818215613	0.800461257		1
ASV_16s_2181	2.490373419	1.685847568	1.755900241	7.666860822	0.36288399		1
ASV_16s_2182	3.009513189	3.417442424	2.884549151	3.037918307	0.88147245		1
ASV_16s_2183	2.993835017	0.808426647	3.549223473	3.248714564	0.861080621		1
ASV_16s_2184	2.154544013	0.124761537	3.547854882	4.09562684	NA	NA	
ASV_16s_2185	2.769834523	-0.786174048	2.892197854	4.635328213	0.704366138		1
ASV_16s_2186	2.3885401	3.870775948	3.54304225	2.523532039	0.925314679		1
ASV_16s_2187	2.318266937	-1.522967687	3.544517881	2.306699557	0.940936491		1
ASV_16s_2188	0.712238105	0.295920317	3.55104398	0.809021342	NA	NA	
ASV_16s_2189	2.833413762	0.372841032	3.550391087	1.423493215	0.984840799		1
ASV_16s_2190	2.861213146	0.4160148	2.927338097	6.378528661	0.496309519		1
ASV_16s_2191	2.711004925	-0.799074982	3.178111985	3.903058083	0.790873129		1
ASV_16s_2192	2.466963259	-1.217662997	2.959811958	2.880981365	0.895789741		1
ASV_16s_2193	2.258096574	0.210141111	3.547733603	4.42240966	0.730039191		1
ASV_16s_2194	2.753617111	-0.805194284	2.266592217	6.54974676	0.477213169		1
ASV_16s_2195	2.443693209	-9.20816142	3.548882241	1.448940941	0.984024872		1
ASV_16s_2196	1.86206556	2.035112375	3.546757922	1.935307145	0.963333961	NA	
ASV_16s_2197	2.311117825	2.029055462	3.545772309	3.25252896	0.860700171		1
ASV_16s_2198	2.547527611	-0.038265768	2.840130837	5.637316197	0.58267674		1
ASV_16s_2199	2.225361306	-1.370650225	3.546814755	2.077042043	0.955453371		1
ASV_16s_2200	3.325487273	-9.398282357	3.550051555	4.168484276	0.760179738		1
ASV_16s_2201	2.348700658	0.172429263	3.327436977	4.051470379	0.773830309		1
ASV_16s_2202	2.227027519	1.268110485	3.546027614	3.425192015	0.843084387		1
ASV_16s_2203	3.020629185	5.028355466	1.736228456	3.277107444	0.858239388		1
ASV_16s_2204	0.707992059	-13.52782608	3.552157344	0.07247031	0.999999243	NA	
ASV_16s_2205	2.254669553	0.003056667	3.547825501	4.017117085	0.777803754		1
ASV_16s_2206	2.487846694	3.246764787	2.626885207	4.789128853	0.685678793		1
ASV_16s_2207	3.17817424	2.989166248	2.295652072	2.944676109	0.890072522		1
ASV_16s_2208	2.337594177	-1.065320052	3.545824127	3.079796969	NA	NA	
ASV_16s_2209	1.791902354	-10.89421738	3.547257847	0.955305568	NA	NA	
ASV_16s_2210	3.642150496	0.841198842	3.549228912	3.875521987	0.793998103		1
ASV_16s_2211	3.244639622	0.34455682	3.547820525	3.23590226	NA	NA	
ASV_16s_2212	2.196615016	-22.22780602	3.550605663	2.16647433	0.950054473		1

ASV_16s_2213	0.767383239	0.102764743	3.550586291	2.739570575	0.908004095	NA
ASV_16s_2214	0.400746687	-21.73792634	3.55144182	-0.287077916	NA	NA
ASV_16s_2215	1.351285989	-7.277386113	3.552157344	0.468717045	NA	NA
ASV_16s_2216	2.153872244	0.588971533	3.392148396	3.162241754	0.869598812	1
ASV_16s_2217	1.616966608	18.25338178	3.551441786	0.497510178	NA	NA
ASV_16s_2218	0.663821543	-22.64579851	3.551440543	0.021839145	NA	NA
ASV_16s_2219	0.588814581	-22.52470363	3.551440932	-0.051613771	NA	NA
ASV_16s_2220	0.399962447	-22.26720758	3.55144254	-0.288276101	NA	NA
ASV_16s_2221	2.114204398	0.623372699	3.548460796	3.6962383	NA	NA
ASV_16s_2222	2.271846089	2.157207674	3.546009866	3.473724273	0.838000694	1
ASV_16s_2223	2.282992168	-1.356125022	2.977299413	2.757178649	0.90652015	1
ASV_16s_2224	0.769058758	-0.136299254	3.54959522	2.456706455	NA	NA
ASV_16s_2225	2.112835524	-23.26279555	3.550606913	2.12386071	NA	NA
ASV_16s_2226	0.587660043	-22.65696833	3.551440936	-0.052816059	NA	NA
ASV_16s_2227	2.290516928	0.032494174	3.547697055	4.348497345	0.73887549	1
ASV_16s_2228	2.316153246	0.537476108	3.047042429	3.589186133	0.825690697	1
ASV_16s_2229	2.409570765	0.105171133	2.458166613	6.656008005	0.465552553	1
ASV_16s_2230	3.156220144	3.644112953	3.545593743	0.764670464	NA	NA
ASV_16s_2231	0.893785311	0.120382743	3.548609579	2.66376611	NA	NA
ASV_16s_2232	2.187594205	-1.691314111	3.546609975	1.923294887	NA	NA
ASV_16s_2233	0.597836198	-22.0664204	3.551441319	0.536139535	NA	NA
ASV_16s_2234	2.448109313	0.357378273	2.103891441	7.741203755	0.355966584	1
ASV_16s_2235	1.992415155	-1.655089745	3.545136174	2.602706227	0.919166697	NA
ASV_16s_2236	2.956186954	-2.97599527	3.544656169	1.560113901	0.980155245	1
ASV_16s_2237	2.092320094	-22.49633355	3.55060633	2.099006126	NA	NA
ASV_16s_2238	2.408839064	-0.231831627	3.546809737	3.433714102	0.842195738	1
ASV_16s_2239	2.131791915	0.048603162	3.547739788	4.208113427	NA	NA
ASV_16s_2240	2.760752506	-0.998081423	3.54759076	2.895773502	0.894473665	1
ASV_16s_2241	2.733455638	-1.223902401	2.670769998	3.346451772	0.851211492	1
ASV_16s_2242	2.236565367	0.067426403	3.547990536	3.912412753	0.789808726	1
ASV_16s_2243	1.516708776	-0.439263657	3.550338573	3.202391592	0.865669423	NA
ASV_16s_2244	0.914024206	0.013864321	3.550774271	1.874859395	NA	NA
ASV_16s_2245	2.187110265	0.048657066	3.547944702	3.913411481	0.789695006	1
ASV_16s_2246	2.174267057	0.314534112	3.54769568	4.046967332	0.774352076	1
ASV_16s_2247	2.40285168	-0.042682225	2.689545221	6.268492749	0.508772005	1
ASV_16s_2248	2.639287306	-0.797282215	3.547654962	3.017806552	0.883350105	1
ASV_16s_2249	2.459298867	0.268212818	3.549433765	1.832364762	0.968533287	1
ASV_16s_2250	3.360304344	10.91884973	3.548196156	1.834904575	0.968410336	1
ASV_16s_2251	0.735041458	-21.36117699	3.550635113	-0.069087562	NA	NA
ASV_16s_2252	2.66940279	0.439993603	3.298804546	3.406904002	0.844985505	1
ASV_16s_2253	2.681543388	0.443691726	3.549348098	2.316491474	0.940270437	1
ASV_16s_2254	2.896255739	1.046021018	3.455231181	4.004371897	0.779273713	1
ASV_16s_2255	2.259476932	-0.336318958	3.547711193	3.952514739	0.785230274	1
ASV_16s_2256	2.650137757	-0.797687839	3.547708488	3.099975171	0.875602193	1
ASV_16s_2257	2.294969314	-1.48585667	3.05397809	3.185618536	0.867316447	1
ASV_16s_2258	2.271019381	-0.038303244	3.544991541	2.806663986	0.902292845	1
ASV_16s_2259	2.553769732	-2.101854564	2.438038288	3.299829217	0.855950304	1
ASV_16s_2260	0.540629422	18.40138035	3.548801168	-0.514408058	1	NA
ASV_16s_2261	2.165226147	0.890726348	3.547383385	3.682838433	0.815497131	1
ASV_16s_2262	2.353438282	1.678914096	3.019680435	4.368558579	0.736481718	1
ASV_16s_2263	2.235676163	3.304969674	2.878616753	4.332357555	0.740798739	1
ASV_16s_2264	2.70895672	0.659957425	3.549427911	3.262575454	0.859696266	1
ASV_16s_2265	2.927439062	-8.793846206	3.550068016	3.599728902	0.824552205	1
ASV_16s_2266	2.286926012	0.278145177	3.548802178	3.46656899	0.838753672	1
ASV_16s_2267	2.152572695	0.545046418	3.547758897	3.726979458	0.810632063	1
ASV_16s_2268	2.861699371	0.213051088	3.243901359	4.956759041	0.665240257	1
ASV_16s_2269	3.035916675	-7.750448676	2.568167903	5.263061298	0.627894876	1
ASV_16s_2270	1.428813462	-7.283885773	3.552157344	0.502924223	0.999435703	NA
ASV_16s_2271	2.115261209	-22.47603786	3.550604072	2.067599715	0.956004231	1
ASV_16s_2272	2.256053538	2.61940887	2.756864335	4.668120058	0.700389571	1
ASV_16s_2273	2.22462853	-25.99116713	3.550776737	1.950843398	0.962510856	1
ASV_16s_2274	2.986416851	-0.090946474	2.746672036	6.56506128	0.475523391	1
ASV_16s_2275	2.4155496	-0.263789183	3.54498293	2.762569459	0.906063696	1
ASV_16s_2276	1.392938863	0.817874301	3.549086316	3.338196068	NA	NA
ASV_16s_2277	2.675870854	1.03444672	3.546304444	3.071183513	NA	NA
ASV_16s_2278	0.747609433	-23.46422953	3.551441165	1.110916046	NA	NA
ASV_16s_2279	2.653977796	-0.92390007	2.924029013	4.036784827	0.775530899	1

ASV_16s_2280	1.808597252	0.205876622	2.852766339	1.745772329	0.972563966	NA
ASV_16s_2281	1.743614678	-0.209370054	3.545307979	0.562222264	NA	NA
ASV_16s_2282	2.201475275	-1.094146401	3.269152899	2.423457049	0.932749705	1
ASV_16s_2283	1.23912372	2.008137095	3.548458305	1.281211606	NA	NA
ASV_16s_2284	0.882655846	0.27784102	3.550586416	4.028381233	0.7765027	NA
ASV_16s_2285	0.687080993	-22.6834302	3.551440439	0.042935637	NA	NA
ASV_16s_2286	2.237049632	-0.760517673	2.988205163	4.823302345	0.681516262	1
ASV_16s_2287	2.442390421	1.158345814	3.401299759	3.509666632	0.834200545	1
ASV_16s_2288	2.537681464	-0.86337149	3.546828936	2.579757643	0.920972457	1
ASV_16s_2289	2.75286468	0.459311366	3.546343018	2.969516521	0.887807804	1
ASV_16s_2290	1.631024364	-28.22047244	3.549670074	0.566662865	0.999163937	NA
ASV_16s_2291	2.026400304	2.142567641	2.372784278	2.356119719	0.937536291	1
ASV_16s_2292	2.139452406	-4.402356208	3.547775271	1.489125371	0.982683752	1
ASV_16s_2293	2.110945521	1.546890466	3.546856252	3.449483816	NA	NA
ASV_16s_2294	2.244062004	2.38745705	3.039731895	4.300032284	0.74464343	1
ASV_16s_2295	2.294740045	2.250614381	3.545296945	3.305757162	0.85535088	1
ASV_16s_2296	2.322346825	1.297113527	2.220679115	4.944164157	0.666777165	1
ASV_16s_2297	2.628066242	1.589754444	2.704777331	5.59628326	0.587597122	1
ASV_16s_2298	3.043034312	16.27972018	3.550637344	2.448928635	NA	NA
ASV_16s_2299	0.726195483	-23.05392494	3.550142009	-0.231204674	NA	NA
ASV_16s_2300	2.265477348	0.896994476	3.010416046	4.962615457	0.664525589	1
ASV_16s_2301	2.707353792	0.420806804	3.125135036	5.577160764	0.589893875	1
ASV_16s_2302	0.717540471	-21.28656552	3.550636589	-0.08235665	NA	NA
ASV_16s_2303	0.690331153	0.219963335	3.550618367	1.40526505	0.985409455	NA
ASV_16s_2304	2.635507862	0.950844196	2.651671014	6.64861272	0.466359183	1
ASV_16s_2305	1.773687765	2.15960663	3.324105719	3.344420155	0.851419146	NA
ASV_16s_2306	2.528981265	-0.87548087	2.608125919	4.890492901	0.673324942	1
ASV_16s_2307	1.488204455	-21.32489007	3.551091881	0.592663193	NA	NA
ASV_16s_2308	1.795972633	10.63510531	3.551470537	1.250127559	NA	NA
ASV_16s_2309	2.920546731	0.640297435	3.435412598	3.514799275	0.833655483	1
ASV_16s_2310	2.19542969	0.38680714	3.545372423	2.583550786	0.920675315	1
ASV_16s_2311	2.371213702	1.045294034	3.546414697	3.920006085	0.788943715	1
ASV_16s_2312	2.485464368	3.073225615	2.063016923	4.111761378	0.766818486	1
ASV_16s_2313	1.85574356	0.859723849	2.667406296	3.378925082	0.8478783	NA
ASV_16s_2314	3.72434904	-1.557818621	3.547696134	1.249066864	NA	NA
ASV_16s_2315	3.073495298	17.42748892	3.551442102	0.93271463	NA	NA
ASV_16s_2316	2.618743934	-0.801624214	2.905228796	4.535733523	0.716410192	1
ASV_16s_2317	1.242124473	-0.464881255	3.548204027	2.322767405	NA	NA
ASV_16s_2318	2.60878088	0.236387211	3.3045656	4.686459404	0.698163562	1
ASV_16s_2319	4.237615608	-2.379915263	3.41638037	2.565925173	0.922051565	1
ASV_16s_2320	2.184499464	0.097242798	3.547880722	4.046664945	0.774387104	1
ASV_16s_2321	2.204797615	0.18114777	3.546798693	3.424533422	0.843152991	1
ASV_16s_2322	2.333856408	-0.338880295	3.546687639	3.675563838	0.816295278	1
ASV_16s_2323	3.133296125	0.465851297	3.185910933	4.924307459	0.669199974	1
ASV_16s_2324	2.235944923	2.115096547	2.44466187	5.829858359	0.559750661	1
ASV_16s_2325	0.382709165	-0.170278455	3.550575739	1.131124821	NA	NA
ASV_16s_2326	2.177496923	0.619826501	3.547461803	3.829027236	0.799246169	1
ASV_16s_2327	2.295314079	-0.449745792	3.546432732	3.003019511	0.884722762	1
ASV_16s_2328	2.412248917	0.967981417	2.551873043	6.687841279	0.462088878	1
ASV_16s_2329	2.268298867	0.406156184	3.000291428	4.944761498	0.666704276	1
ASV_16s_2330	2.663878846	-1.018195957	2.643582362	4.111539451	0.766844382	1
ASV_16s_2331	3.529501995	0.201374496	3.549302539	1.488467691	0.982706224	1
ASV_16s_2332	2.325886509	1.376376055	2.681001748	6.318922446	0.503042621	1
ASV_16s_2333	2.874820139	0.343327102	3.549302107	3.426186047	0.842980823	1
ASV_16s_2334	2.071546009	0.731425748	3.54798205	3.745775879	NA	NA
ASV_16s_2335	2.571867968	-0.797009479	3.547721412	3.075499278	0.877931157	1
ASV_16s_2336	2.761742511	-0.927328994	2.392967854	3.908913263	0.790207071	1
ASV_16s_2337	1.98706775	2.005869056	1.959303625	6.271316082	0.508450461	NA
ASV_16s_2338	2.161130127	-0.651972266	3.011025596	4.38292378	0.734765474	1
ASV_16s_2339	2.414454334	-3.11020901	3.544659959	0.654228554	0.998663239	1
ASV_16s_2340	1.503868743	16.93359002	3.551446716	0.493980341	NA	NA
ASV_16s_2341	0.705873146	-0.684094273	3.549366449	1.101789317	NA	NA
ASV_16s_2342	2.265788245	0.136134882	3.5465373	3.250411584	0.860911408	1
ASV_16s_2343	2.008299773	-0.342213214	3.547709877	2.339354472	NA	NA
ASV_16s_2344	2.906751857	0.015745972	3.549041255	3.799071384	0.802607827	1
ASV_16s_2345	0.704414731	-0.684088388	3.549367757	1.101122271	NA	NA
ASV_16s_2346	2.382256116	-0.034921678	2.983706932	5.450888225	0.605114103	1

ASV_16s_2347	1.991363488	1.530602113	3.547024547	3.400713195	NA	NA	
ASV_16s_2348	2.494483278	-0.816966009	2.739146921	5.092723413	0.64864873		1
ASV_16s_2349	2.467229208	-0.796480552	3.547820719	3.064609576	0.878961652		1
ASV_16s_2350	2.238480475	4.120370113	3.066954281	0.985571116	0.995057948		1
ASV_16s_2351	2.521972724	0.92646584	2.216798467	7.602270218	0.368967064		1
ASV_16s_2352	3.048419056	0.087120217	3.54844798	2.713595165	0.910173577		1
ASV_16s_2353	2.82189469	0.761408277	3.549381395	2.860845597	0.897569762		1
ASV_16s_2354	2.346390345	1.999738274	3.545625275	3.359166972	0.849909494		1
ASV_16s_2355	3.808696253	0.428814564	3.549067968	2.263631557	NA	NA	
ASV_16s_2356	0.698792558	-0.66924723	3.54931201	1.352691359	NA	NA	
ASV_16s_2357	2.160063946	0.017342948	3.548010051	3.794361906	0.803134894		1
ASV_16s_2358	2.044894786	0.090126066	3.547964561	3.887863948	0.792598977		1
ASV_16s_2359	2.183168352	1.933058493	3.54626666	3.442482823	0.841279566		1
ASV_16s_2360	2.540574335	0.154395043	1.827401064	14.76512777	0.039130786		0.262425576
ASV_16s_2361	2.245663293	-0.679401746	3.285162945	4.259047488	0.749503472		1
ASV_16s_2362	2.009727313	-0.396168026	3.143143649	2.645042653	0.915785126		1
ASV_16s_2363	2.579373848	-1.623566686	2.397789224	6.760778459	0.454205397		1
ASV_16s_2364	2.451576172	0.737504621	3.549062992	3.375655347	NA	NA	
ASV_16s_2365	2.092559431	0.569840803	3.545263614	1.939440067	0.963115982		1
ASV_16s_2366	2.720266123	0.777216169	2.677682767	7.164794652	0.411925719		1
ASV_16s_2367	0.374866764	-0.170273366	3.550577197	1.120622474	NA	NA	
ASV_16s_2368	2.307310449	-26.19290533	3.551470723	1.514311821	0.981810045		1
ASV_16s_2369	1.968115546	-0.278209995	3.549022848	3.347059811	NA	NA	
ASV_16s_2370	2.152693047	0.099460401	3.54780068	4.158057301	0.761403004		1
ASV_16s_2371	2.140699488	1.142435147	3.292394218	4.272714955	0.747884632		1
ASV_16s_2372	2.284283398	-2.529431808	2.521605224	2.839812386	0.89941486		1
ASV_16s_2373	2.459377157	-21.06587082	3.55021323	1.416662035	0.985055448		1
ASV_16s_2374	2.498444242	-2.139943926	1.877577886	5.887473727	0.552948345		1
ASV_16s_2375	3.055069568	-2.171388214	3.544141369	3.290219921	0.856920035		1
ASV_16s_2376	1.256087759	-7.214199853	3.552157344	0.423893265	NA	NA	
ASV_16s_2377	0.880179191	13.82652062	3.55156209	0.618386983	NA	NA	
ASV_16s_2378	2.517854003	-0.799113517	3.174954718	3.814879889	0.800835741		1
ASV_16s_2379	2.216985627	-2.887181964	3.544993223	0.648407136	NA	NA	
ASV_16s_2380	2.078086896	-0.311581525	2.722784221	5.548530866	0.593336767		1
ASV_16s_2381	2.389631553	2.555075581	1.786818552	8.579099429	0.284303121		1
ASV_16s_2382	2.045218247	0.550837882	3.547319249	3.815585576	0.800756533		1
ASV_16s_2383	2.151406234	2.217498812	3.040914747	4.257922845	0.749636595		1
ASV_16s_2384	2.087468039	-0.112177729	3.041234074	4.609207443	0.707530079		1
ASV_16s_2385	2.334494064	0.075874325	3.546601249	4.11334362	0.76663384		1
ASV_16s_2386	2.22833514	-8.897012687	3.546290809	1.205751108	NA	NA	
ASV_16s_2387	2.131511655	0.010081577	3.547566896	2.958064474	0.888854309		1
ASV_16s_2388	1.781032064	2.435152472	2.910742094	4.260788017	0.749297422	NA	
ASV_16s_2389	2.414076432	-7.925681555	3.548970561	2.128884728	0.952363584		1
ASV_16s_2390	2.33005143	1.439141282	3.25257524	2.726890151	0.909066106		1
ASV_16s_2391	1.408715454	0.052642513	3.55026446	3.293848543	0.856554132	NA	
ASV_16s_2392	2.857167434	2.531454533	3.547566408	1.009748698	0.99466974		1
ASV_16s_2393	1.954178162	-0.006394914	3.547842942	3.853254131	NA	NA	
ASV_16s_2394	2.053840404	0.768250701	3.547015904	3.722263146	0.81115366		1
ASV_16s_2395	2.288226599	0.014343935	3.545492598	3.342596428	0.851605462		1
ASV_16s_2396	1.844975022	2.316299075	3.545802957	3.176826771	NA	NA	
ASV_16s_2397	2.319046869	0.960530185	1.748384003	9.624122242	0.21089332		0.87128452
ASV_16s_2398	2.36294731	-0.9288769	2.795852477	4.00785243	0.778872517		1
ASV_16s_2399	2.491258047	-0.214961831	2.931030734	6.014966493	0.538002879		1
ASV_16s_2400	2.531453206	-0.890841783	3.548930222	2.435866985	0.931848428		1
ASV_16s_2401	3.034381775	0.161126547	3.1296039	5.994767577	0.540360448		1
ASV_16s_2402	2.902207432	0.811837603	3.549276761	3.08678912	0.876859084		1
ASV_16s_2403	2.120798281	-0.065258543	3.547295457	3.626527507	0.821647804		1
ASV_16s_2404	2.308147842	-0.762762479	2.841026221	2.928210302	0.891562961		1
ASV_16s_2405	2.336525335	-0.119737286	3.546569788	3.733216026	0.809941689		1
ASV_16s_2406	2.452689028	2.046087354	1.895126562	10.37915135	0.168085946		0.745304169
ASV_16s_2407	2.547834246	11.69687241	3.54893177	1.024203374	0.994428596		1
ASV_16s_2408	2.131610087	-0.205244245	3.547403635	3.206826191	0.865232668		1
ASV_16s_2409	1.960891274	0.710716789	3.547880767	3.672028047	NA	NA	
ASV_16s_2410	2.496570294	17.93052268	3.549222999	0.099218521	0.999997751		1
ASV_16s_2411	2.415421744	-0.706286593	3.546559503	2.534365874	0.924487125		1
ASV_16s_2412	1.917190592	22.08335991	3.548878749	1.080176028	NA	NA	
ASV_16s_2413	2.314029129	-0.772940602	3.547828052	2.913883498	0.892852744		1

ASV_16s_2414	2.192902659	0.134717026	3.013157438	5.121667741	0.645118332		1
ASV_16s_2415	2.518055025	-0.796965577	3.547724681	3.054318554	0.879932243		1
ASV_16s_2416	2.674660658	0.222711606	2.039523127	11.8077127	0.107062314		0.548286079
ASV_16s_2417	0.571192688	0.317236828	3.550469479	1.053836447	NA	NA	
ASV_16s_2418	2.543774733	0.723213476	3.550411973	1.987865923	0.960508905		1
ASV_16s_2419	2.819111943	0.118813041	3.549317795	5.546302563	0.593604939		1
ASV_16s_2420	2.423575155	0.311346656	3.056425723	7.274246624	0.400894868		1
ASV_16s_2421	2.126727853	-0.342254349	3.546821957	3.626829064	0.821615037		1
ASV_16s_2422	1.350756255	0.277218625	3.550596704	2.984985829	0.886387681	NA	
ASV_16s_2423	2.793755377	0.396808034	3.549788804	1.207045172	0.990763779		1
ASV_16s_2424	2.03549883	0.025688949	3.54797873	3.786174525	0.804050254		1
ASV_16s_2425	2.090658548	0.575115105	3.54764575	3.778182406	0.804942626		1
ASV_16s_2426	2.220457624	0.29717186	3.546670237	4.001946018	0.779553237		1
ASV_16s_2427	2.45748191	-0.797170893	3.547674127	2.940718139	0.890431573		1
ASV_16s_2428	2.4018031	0.320821591	2.986632946	5.76392377	0.567569379		1
ASV_16s_2429	2.262489781	-0.154764832	3.547785011	4.594916456	0.709259644		1
ASV_16s_2430	2.28144448	-1.043001417	2.625726667	4.787032964	0.685933988		1
ASV_16s_2431	2.556958505	1.207026992	3.548997625	1.463902801	0.983533098		1
ASV_16s_2432	3.401020293	-2.219776832	3.545567277	1.571110391	NA	NA	
ASV_16s_2433	2.076819691	-1.658639389	3.231590012	2.203725267	0.947709471		1
ASV_16s_2434	2.33392527	-0.306321835	3.545495245	2.876822648	0.896158463		1
ASV_16s_2435	2.556041486	-0.090783932	3.549663138	1.676035285	0.975582767		1
ASV_16s_2436	0.567531197	0.316927873	3.550476381	1.051690675	NA	NA	
ASV_16s_2437	1.313654803	-0.275464517	3.547542701	1.904317768	NA	NA	
ASV_16s_2438	1.837618541	-0.835262107	3.545301929	3.047997176	0.880526866	NA	
ASV_16s_2439	2.088838631	-0.602240303	2.98412469	4.820043785	0.681913294		1
ASV_16s_2440	2.461965827	-0.714175432	2.916768645	4.466331557	0.72476764		1
ASV_16s_2441	2.221299213	0.158905999	3.547660979	3.060180274	0.879379787		1
ASV_16s_2442	2.475152665	-0.42257523	2.627267923	6.110425425	0.526916436		1
ASV_16s_2443	1.438511348	16.86497383	3.551447195	0.556583664	NA	NA	
ASV_16s_2444	0.869632479	-1.264045308	2.973320858	1.45064549	0.983969297	NA	
ASV_16s_2445	1.822898015	2.487420379	3.094120298	2.623525629	0.917511873	NA	
ASV_16s_2446	2.063552465	0.237007086	3.548659097	3.281734838	0.857774305		1
ASV_16s_2447	2.078387288	1.161163408	3.545199036	2.932023002	0.891218614		1
ASV_16s_2448	2.381209153	-0.658430005	2.923588415	4.329564167	0.741131364		1
ASV_16s_2449	2.297077458	-1.973632735	2.672145439	2.398278788	0.934560057		1
ASV_16s_2450	1.801408298	0.999992525	2.022151858	3.516300827	0.833495913	NA	
ASV_16s_2451	2.84206366	0.47619165	2.68000505	8.884486897	0.261056195		1
ASV_16s_2452	2.09232847	-0.301574816	3.546668282	3.64805295	NA	NA	
ASV_16s_2453	2.241288093	-0.315319616	3.546559518	3.663402991	0.817627193		1
ASV_16s_2454	2.696667197	-0.855963172	2.706310573	5.57186714	0.590530083		1
ASV_16s_2455	2.791206209	0.015335951	1.933901841	11.74198674	0.109369449		0.553940245
ASV_16s_2456	1.333979936	-13.61681461	3.552157344	0.460787336	0.999577773	NA	
ASV_16s_2457	2.047451064	-0.096554514	2.492867472	4.535571818	0.716429701		1
ASV_16s_2458	0.607746624	-0.724673936	3.549134244	1.43498179	NA	NA	
ASV_16s_2459	2.444442472	0.817367878	3.548535161	3.200290016	0.865876214		1
ASV_16s_2461	1.97523471	0.079091901	3.548054833	3.741976498	0.80897069	NA	
ASV_16s_2462	2.041151561	-0.010191838	3.548001278	3.706757353	0.812865528		1
ASV_16s_2463	2.657151187	0.403762432	3.549145218	1.51266925	NA	NA	
ASV_16s_2464	3.42449631	6.839064585	3.550038556	2.599144493	0.919448221		1
ASV_16s_2465	1.981919242	2.808984958	2.732484717	4.691724342	0.697524253	NA	
ASV_16s_2466	1.846983397	-1.416866637	2.902699229	3.170166357	0.868826823	NA	
ASV_16s_2467	1.62022861	-2.129873828	3.547084503	1.06167564	0.993771335	NA	
ASV_16s_2468	2.209403775	-27.22561214	3.550230066	1.137861306	0.992287974		1
ASV_16s_2469	2.62311268	0.218606554	2.972222498	6.029732877	0.536281918		1
ASV_16s_2470	2.330516236	1.053479197	3.061082473	4.194580553	0.757112651		1
ASV_16s_2471	1.334185187	-18.58544765	3.552157344	0.170710602	0.999985382	NA	
ASV_16s_2472	2.697556363	0.568839516	3.549055086	2.047572243	0.957160451		1
ASV_16s_2473	2.658297954	1.588202257	3.548590187	2.258643951	0.94415008		1
ASV_16s_2474	0.357613482	-0.170259489	3.55058063	1.096731795	NA	NA	
ASV_16s_2475	2.190513892	1.995669833	2.544002072	4.171117562	0.759870606		1
ASV_16s_2476	1.851219431	1.444478691	2.487145655	5.689251458	0.576465449	NA	
ASV_16s_2477	1.369617329	-14.26982885	3.552157344	0.476954822	0.999526565	NA	
ASV_16s_2478	0.356829242	-0.170258876	3.550580794	1.095618956	NA	NA	
ASV_16s_2479	2.206202687	1.382538098	3.053334832	3.980409159	0.782031113		1
ASV_16s_2480	2.125651454	0.377940558	3.546678491	3.591836844	0.825404673		1
ASV_16s_2481	2.159984765	1.974744895	2.406511964	3.337328136	0.852143211		1

ASV_16s_2482	2.107611573	-2.255854849	3.544228045	1.464945128	NA	NA	
ASV_16s_2484	1.949099579	0.072605959	3.548108517	3.70122018	0.813475725	NA	
ASV_16s_2485	2.365892415	2.426449046	2.656421733	4.173704787	0.759566802		1
ASV_16s_2486	2.065322219	1.271802907	3.39429707	2.537559717	0.924242324		1
ASV_16s_2487	3.278798179	0.750403806	3.54805195	2.235665357	0.945654123		1
ASV_16s_2488	2.841887709	17.36355848	3.551442474	0.884585803	NA	NA	
ASV_16s_2489	1.04483364	-20.70764114	3.551451082	1.242713945	NA	NA	
ASV_16s_2490	2.636243082	-0.48937226	2.325587138	7.838510255	0.347049213		1
ASV_16s_2491	2.173708097	-0.721364026	3.547975717	2.959595993	0.888714595		1
ASV_16s_2492	2.072568872	2.983987043	3.454556278	2.825526446	0.900659691		1
ASV_16s_2493	2.389121917	-0.796720592	3.547776841	3.025778509	0.882607307		1
ASV_16s_2494	3.031085076	0.592093285	3.549046036	3.640182257	NA	NA	
ASV_16s_2495	1.86681681	-0.847443294	3.545557131	2.301685483	NA	NA	
ASV_16s_2496	3.408443906	-3.845355025	3.543889293	0.619556054	NA	NA	
ASV_16s_2497	3.081793906	-4.653888422	3.543855242	0.348911688	NA	NA	
ASV_16s_2498	2.116163273	-0.39576547	3.376483051	2.934644311	0.890981599		1
ASV_16s_2499	2.550432645	0.364556365	3.550492034	1.18152556	0.991346065		1
ASV_16s_2500	2.500832829	1.184346604	3.548158357	3.548324683	0.830080797		1
ASV_16s_2501	1.917486644	-0.329451252	3.549017839	3.272896629	0.858662112	NA	
ASV_16s_2502	0.352908042	-0.1702544	3.550581625	1.090018107	NA	NA	
ASV_16s_2503	1.315340371	-29.98480496	3.550786947	0.20974367	0.999970399	NA	
ASV_16s_2504	2.06410495	0.391934464	3.551318072	0.5486594	NA	NA	
ASV_16s_2505	2.793258992	1.128809377	3.549431914	3.006738504	0.884378165		1
ASV_16s_2506	2.032994019	0.269148048	3.285765923	3.880654595	0.793416555		1
ASV_16s_2507	2.727226272	0.565169915	2.864373832	7.224220057	0.405913641		1
ASV_16s_2508	2.023338613	17.47785308	3.55144303	0.665974615	NA	NA	
ASV_16s_2509	0.620589284	-22.45097793	3.551440756	-0.019449555	NA	NA	
ASV_16s_2510	2.14437175	0.125900766	3.547419553	3.094090001	0.876163797		1
ASV_16s_2511	2.065170523	-0.002706183	3.547722569	4.146672165	0.762737204		1
ASV_16s_2512	2.131388578	0.99377988	2.423615716	7.907272645	0.340841549		1
ASV_16s_2513	2.319748958	-0.79676838	3.547766272	2.990247586	0.885902941		1
ASV_16s_2514	2.962768627	1.209950988	2.852076864	6.212354623	0.515184367		1
ASV_16s_2515	1.995328087	-0.544571847	3.547093876	1.883538469	NA	NA	
ASV_16s_2516	2.870907643	0.945443858	2.811020788	4.772809443	0.687665521		1
ASV_16s_2518	2.277876463	-0.993002912	3.546865575	2.523848679	0.925290554		1
ASV_16s_2519	2.485415868	0.829662248	3.549710366	3.04196198	0.881093447		1
ASV_16s_2520	2.391567762	0.790106763	3.016337215	6.32890833	0.501911655		1
ASV_16s_2521	2.59848036	0.587627542	3.37641893	4.582898466	0.710713277		1
ASV_16s_2522	0.650453354	-0.683853144	3.549420268	1.075420162	NA	NA	
ASV_16s_2523	2.363061318	-0.797132694	3.547699619	2.938613838	0.890622264		1
ASV_16s_2524	2.1366788	0.34836631	3.549853806	1.206222349	0.990782923		1
ASV_16s_2525	2.605998699	0.271422899	3.549135517	1.503509908	NA	NA	
ASV_16s_2526	2.5451758	-0.368898479	2.374227911	3.206050108	0.865309142		1
ASV_16s_2527	2.032296935	0.477879098	3.54999083	0.706166083	NA	NA	
ASV_16s_2528	1.599911386	0.115015316	3.30061463	2.169488679	0.949866809	NA	
ASV_16s_2529	1.890283554	-0.361230867	3.549015468	3.215254897	0.864401047	NA	
ASV_16s_2530	2.051739611	-0.321703207	3.547033233	3.63629746	0.820585255		1
ASV_16s_2531	2.314221918	0.749545773	2.688606604	6.103931211	0.527667696		1
ASV_16s_2532	2.548937978	-1.006783372	3.549459232	2.007751151	0.959410114		1
ASV_16s_2533	0.646078107	-0.683832395	3.549424908	1.073243703	NA	NA	
ASV_16s_2534	0.373081931	-0.170271067	3.550577538	1.118184437	0.992690348	NA	
ASV_16s_2535	1.835147688	0.048366622	3.547883013	3.833498944	NA	NA	
ASV_16s_2536	2.084973506	-1.68164773	3.546898465	1.878775215	0.966244024		1
ASV_16s_2537	2.099531712	-1.212106964	3.081381849	2.638646828	0.916300135		1
ASV_16s_2538	2.423343288	3.353994562	3.546376925	0.771119337	NA	NA	
ASV_16s_2539	2.318681755	2.847098693	3.547164444	0.858432382	NA	NA	
ASV_16s_2540	1.29508674	-30	3.550632413	0.616382404	0.998899009	NA	
ASV_16s_2541	1.255194546	-12.18554662	3.552157344	0.423439844	0.999681349	NA	
ASV_16s_2542	2.374044554	1.273971254	3.549011111	3.313294905	0.854587361		1
ASV_16s_2543	2.809396692	1.188650428	3.549324646	1.652553347	0.976553796		1
ASV_16s_2544	2.773031399	17.37052261	3.551442571	0.869540042	NA	NA	
ASV_16s_2545	0.834719276	17.77572637	3.551445573	0.092115592	NA	NA	
ASV_16s_2546	2.271113464	-0.859453942	2.793923327	4.057554221	0.773124939		1
ASV_16s_2547	2.140975972	-0.976385241	3.546871348	2.412635811	0.933530765		1
ASV_16s_2548	2.116294442	0.355612415	3.54963087	1.379655172	0.986186273		1
ASV_16s_2549	3.108700796	0.161504589	3.547874943	3.337589242	0.852116575		1
ASV_16s_2550	2.057540865	3.02674916	2.508844896	5.107672369	0.646825242		1

ASV_16s_2551	2.277407183	0.256242942	2.569787335	6.365825932	0.497740832	1
ASV_16s_2552	2.346018178	-0.664367672	2.653971117	4.291276888	0.745683058	1
ASV_16s_2553	1.747853618	2.183798782	3.54656993	1.979036741	0.96099151	NA
ASV_16s_2554	1.921258626	-2.179639213	3.5445498	1.971235276	0.961415243	NA
ASV_16s_2555	1.36743529	16.86730319	3.551447613	0.435608933	NA	NA
ASV_16s_2556	2.316274561	-0.843243444	2.771811883	4.457227015	0.725861556	1
ASV_16s_2557	3.164377807	0.393149271	3.548055931	3.362769958	0.849539821	1
ASV_16s_2558	2.283816844	0.700293147	2.640009538	7.10293181	0.41824198	1
ASV_16s_2559	1.707213543	-1.500271887	3.545973673	1.764229373	0.971731089	NA
ASV_16s_2560	2.357286267	0.094954811	2.729746656	6.443665587	0.489001138	1
ASV_16s_2561	0.751601541	-0.450806899	3.549304227	2.725957131	NA	NA
ASV_16s_2562	1.899871341	0.067834095	3.547794232	4.046251436	0.774435001	NA
ASV_16s_2563	2.710436566	0.881728233	2.152248351	9.722852604	0.204830682	0.854755659
ASV_16s_2564	2.793757368	2.004242041	3.275461466	2.726078924	0.909133858	1
ASV_16s_2565	2.280047799	1.68318189	2.433267053	6.490853871	0.483739703	1
ASV_16s_2566	0.54079056	-0.607860836	3.549895902	1.005149785	NA	NA
ASV_16s_2567	1.924710194	0.832497999	3.546732499	3.73125379	0.810158983	NA
ASV_16s_2568	1.484376479	2.136418432	3.5470829	1.432175054	NA	NA
ASV_16s_2569	1.479763627	-22.9354016	3.551442474	0.581631783	NA	NA
ASV_16s_2570	3.349872521	0.159554248	3.547972451	3.468480757	0.838552605	1
ASV_16s_2571	3.086982101	0.786499867	3.527716758	3.733151931	0.809948787	1
ASV_16s_2572	2.68694025	12.23816043	3.550117248	1.781959907	NA	NA
ASV_16s_2573	2.588629384	17.60865456	3.550784636	0.641848046	0.99874375	1
ASV_16s_2574	2.148362564	-0.963343683	3.547229744	2.479950744	0.928599212	1
ASV_16s_2575	2.375681445	-0.80790737	3.547653718	2.59433402	0.919827713	1
ASV_16s_2576	2.250875942	0.237995362	3.545642159	3.558316339	0.829010639	1
ASV_16s_2577	1.239193959	-13.49410357	3.552157344	0.415566907	0.9997007	NA
ASV_16s_2578	1.934311031	1.866013496	3.546035728	3.172452817	0.868603755	NA
ASV_16s_2579	1.284271956	29.79700685	3.550270812	0.495406435	0.999463128	NA
ASV_16s_2580	2.505953673	3.364551682	3.548698356	2.031560799	0.958072897	1
ASV_16s_2581	1.935697452	0.561547801	3.547536796	3.238946941	0.862053064	NA
ASV_16s_2582	1.846282498	0.075167496	3.547742002	4.18006763	NA	NA
ASV_16s_2583	2.234818421	-0.707940567	3.547690443	2.492907774	0.927630169	1
ASV_16s_2584	2.287497442	-0.799026597	3.170316871	3.703638791	0.813209267	1
ASV_16s_2585	2.302190166	-0.799106446	3.167820237	3.729288476	0.810376545	1
ASV_16s_2586	2.220041875	-0.796104229	3.547903897	2.977645193	0.887062501	1
ASV_16s_2587	3.236879895	-3.758333756	3.543972526	0.621633345	NA	NA
ASV_16s_2588	2.813606784	-0.79034522	3.546585969	1.844917853	NA	NA
ASV_16s_2589	1.752719675	1.026566181	3.547505979	2.991555238	0.88578234	NA
ASV_16s_2590	2.705257434	0.498446414	3.547948281	4.609933068	0.707442232	1
ASV_16s_2591	0.562781094	-0.009497306	3.550573258	2.705147871	0.910874023	NA
ASV_16s_2592	1.877337217	-0.016839462	3.54783694	3.809290823	0.801462759	NA
ASV_16s_2593	2.231631942	-0.109550889	3.54663794	3.509034383	0.834267646	1
ASV_16s_2594	2.210292639	-0.652299073	3.547806142	2.919894761	0.892312376	1
ASV_16s_2595	1.9750453	-1.067414334	2.340435214	3.594524054	0.825114559	NA
ASV_16s_2596	3.787994173	-3.905018707	3.543714652	0.634285762	NA	NA
ASV_16s_2597	1.214837155	-13.79341292	3.552157344	0.403390976	0.999729016	NA
ASV_16s_2598	2.192381369	-0.187704848	3.214204629	3.868737012	0.794766205	1
ASV_16s_2599	2.201803877	-0.244094382	3.139976317	4.956385748	0.66528581	1
ASV_16s_2600	2.564164697	0.394987571	3.550076996	1.112854394	0.99279701	1
ASV_16s_2601	1.939046833	1.047617772	3.547716189	3.527214338	0.832334621	NA
ASV_16s_2602	2.173612396	-0.795550558	3.548021328	2.953112537	0.889305547	1
ASV_16s_2603	2.437167218	0.350506239	3.510166421	1.586257805	0.979172302	1
ASV_16s_2604	1.94999362	0.652951007	3.547611595	2.708129162	0.910627101	NA
ASV_16s_2605	2.262623329	-0.796995022	3.547771426	2.965928204	0.888136152	1
ASV_16s_2606	2.049351708	-2.3762593	3.54588824	2.108527675	0.953590003	1
ASV_16s_2607	2.355295205	15.65179286	3.551077268	0.995643773	0.994898503	1
ASV_16s_2608	2.934799828	0.27141867	3.549109656	1.541857001	NA	NA
ASV_16s_2609	2.542960842	1.518065069	2.917306337	3.814914198	0.80083189	1
ASV_16s_2610	2.530635822	0.694088305	3.550581599	2.165578759	0.950110157	1
ASV_16s_2611	2.299078412	-0.285026281	3.547312205	3.633576643	0.820881359	1
ASV_16s_2612	1.328071588	-18.53857742	3.552157344	0.123907453	0.999995151	NA
ASV_16s_2613	0.999460698	-0.294044052	3.548529926	2.01661697	0.95891493	NA
ASV_16s_2614	3.506273734	9.274880411	3.54968358	2.440652699	0.931499284	1
ASV_16s_2615	1.76839919	-0.318738651	3.549035605	3.205198158	NA	NA
ASV_16s_2616	2.105330867	-0.962754201	3.546504213	3.21760983	0.864168348	1
ASV_16s_2617	2.332991144	0.068496559	2.214085612	8.611426803	0.281770328	1

ASV_16s_2618	3.404889662	0.245560834	3.546227663	3.779910135	NA	NA
ASV_16s_2619	1.242713256	0.323344478	3.549690099	1.306015437	0.988277691	NA
ASV_16s_2620	2.375162281	0.828212426	3.54816031	2.842137777	0.89921159	1
ASV_16s_2621	1.990481086	0.9899084	3.546975657	3.694045101	0.814265538	NA
ASV_16s_2622	2.042272792	-0.381489389	3.547829572	2.820669024	0.901081398	1
ASV_16s_2623	1.926287461	-0.817123207	3.245492206	3.850963255	0.796774685	NA
ASV_16s_2624	1.561934505	-9.023503976	3.548581626	1.405182728	0.985411994	NA
ASV_16s_2625	1.815845674	-0.733714398	2.621864919	4.31705773	0.74261969	NA
ASV_16s_2626	2.411522272	0.400584019	2.743376665	5.27624112	0.626292027	1
ASV_16s_2627	2.597492055	7.381182606	3.550135599	1.712629663	0.974023889	1
ASV_16s_2628	2.12446894	-0.783950716	2.872077854	3.510174908	0.834146595	1
ASV_16s_2629	1.893430624	2.737574818	2.328292608	5.402337972	0.610988385	NA
ASV_16s_2630	2.310893665	2.796669785	2.972996473	1.714946502	0.97392332	1
ASV_16s_2631	0.81719022	-20.42370707	3.550914115	0.589697612	NA	NA
ASV_16s_2632	1.314722365	16.80289059	3.551448022	0.411478887	NA	NA
ASV_16s_2633	2.173666466	0.049068157	2.292648572	9.238906207	0.235955689	0.949152858
ASV_16s_2634	2.561729304	-7.577348178	1.950087348	8.12164514	0.321989751	1
ASV_16s_2635	3.113778707	-2.854106725	3.544778348	0.722800318	NA	NA
ASV_16s_2636	2.121815373	-0.819463851	2.157471535	7.998535611	0.332723049	1
ASV_16s_2637	2.281848916	0.1739396	3.548254857	3.673838676	0.816484405	1
ASV_16s_2638	2.154567123	0.860524319	3.048045816	2.655097143	0.914972546	1
ASV_16s_2639	1.315511379	-30	3.550166282	0.977916537	0.99517695	NA
ASV_16s_2640	2.881808156	-1.942669934	3.543730039	1.26151979	NA	NA
ASV_16s_2641	2.159192638	1.335123241	2.906174704	5.628381966	0.583747121	1
ASV_16s_2642	2.507126442	15.69423159	3.550981581	2.169016248	0.949896246	1
ASV_16s_2643	1.913765598	0.668021994	3.547659206	3.808356852	NA	NA
ASV_16s_2644	2.282966561	0.234109257	3.135941463	2.678171834	0.913094053	1
ASV_16s_2645	1.999753409	-21.57608823	3.551462129	1.34876992	0.987088981	1
ASV_16s_2646	2.361978904	0.35542742	3.55028175	1.844767201	0.967930333	1
ASV_16s_2647	0.806831806	17.79016602	3.551445827	0.071292906	0.999999285	NA
ASV_16s_2648	2.485385792	-0.091065871	2.596996794	7.806110084	0.350001172	1
ASV_16s_2649	2.331146997	0.940360691	3.549117085	2.254257168	0.944438847	1
ASV_16s_2650	1.302319323	16.83695542	3.551448114	0.405663742	NA	NA
ASV_16s_2651	1.778143995	-0.284032771	3.549041199	3.242507567	0.861698874	NA
ASV_16s_2652	1.76404963	-0.044212682	3.547940681	3.564091791	0.828391067	NA
ASV_16s_2653	1.890628789	0.069801629	3.548104995	3.668034351	0.81712029	NA
ASV_16s_2654	1.95805514	-0.191945433	3.548680782	2.600440876	0.919345807	NA
ASV_16s_2655	1.991318653	2.458725931	3.543375867	1.002045092	NA	NA
ASV_16s_2656	1.994035156	0.215645229	3.548288234	2.802596869	0.902643416	NA
ASV_16s_2657	1.961748979	3.98903356	2.337421491	1.711173485	0.974086984	NA
ASV_16s_2658	0.851871249	-1.983002252	3.54790861	0.570414653	NA	NA
ASV_16s_2659	2.590241646	0.916628557	3.549303585	2.766114836	0.905762956	1
ASV_16s_2660	1.824462937	0.309363029	3.548625209	3.1987048	0.866032116	NA
ASV_16s_2661	2.159534058	-0.79689442	3.54774953	2.88706495	0.895249338	1
ASV_16s_2662	2.226459247	1.227162405	2.869733661	4.624947955	0.705623875	1
ASV_16s_2663	1.165467416	-28.04006925	3.551319338	0.004037052	1	NA
ASV_16s_2664	2.16730667	-0.665637274	3.547069809	2.407163653	0.933924018	1
ASV_16s_2665	2.146198978	-0.796632403	3.547791584	2.919231535	0.892372052	1
ASV_16s_2666	2.222392516	-0.902149541	3.214356813	3.197802392	0.866120834	1
ASV_16s_2667	1.875193481	-1.872393578	3.256934855	2.4469076	0.931041633	NA
ASV_16s_2668	2.035538714	-1.859039233	3.546875151	0.868535193	NA	NA
ASV_16s_2669	0.325459639	-0.170228047	3.550587998	1.048996291	NA	NA
ASV_16s_2670	2.126468459	-0.590767001	2.178903108	9.393704363	0.225611542	0.921088607
ASV_16s_2671	2.295041286	-0.901036351	2.648524433	4.3168782	0.742641045	1
ASV_16s_2672	2.314020335	-0.475470173	2.692788943	6.265714969	0.509088451	1
ASV_16s_2673	2.323437807	0.120860257	3.548249819	3.118621319	0.873816169	1
ASV_16s_2674	1.232827295	-30	3.550894664	0.413723924	0.999705111	NA
ASV_16s_2675	2.107806914	-0.985476754	3.546848653	2.312992037	0.940508907	1
ASV_16s_2676	1.893321475	0.449124063	3.549730379	0.790377332	NA	NA
ASV_16s_2677	1.219184622	-29.91319215	3.550632354	0.579437283	0.999100517	NA
ASV_16s_2678	1.283714762	16.77990868	3.55144827	0.39683178	NA	NA
ASV_16s_2679	2.223101778	-2.098459345	3.263421774	2.622865833	0.917564558	1
ASV_16s_2680	2.18000112	-0.841661956	2.761644039	4.852899592	0.677909018	1
ASV_16s_2681	1.586539316	17.16912602	3.549123726	-0.107021855	NA	NA
ASV_16s_2682	1.592298907	-0.351068112	3.082217732	2.979390596	0.886902199	NA
ASV_16s_2683	2.091264	0.960105656	3.366049224	2.757913093	0.906458022	1
ASV_16s_2684	1.643449392	-1.224342177	3.54698164	1.806116739	0.969788155	NA

ASV_16s_2685	2.500766976	6.83838578	3.550147365	1.651173346	0.976610151	1
ASV_16s_2686	2.492867689	1.188191355	3.549433377	1.653492446	0.9765154	1
ASV_16s_2687	1.699110269	-0.268945123	3.54905356	3.207853918	NA	NA
ASV_16s_2688	0.599408808	-0.683592079	3.549478587	1.049067006	NA	NA
ASV_16s_2689	1.846832221	0.044161876	3.547852185	3.87664494	0.793870904	NA
ASV_16s_2690	1.781990372	-0.062120207	3.545507328	2.635406818	NA	NA
ASV_16s_2691	1.991449428	2.459351226	3.544324299	2.392691591	0.934958461	NA
ASV_16s_2692	2.415769478	-10.1230354	3.548007918	1.574678429	0.979611109	1
ASV_16s_2693	2.151972846	0.629710087	3.549465615	4.743650397	0.691213389	1
ASV_16s_2694	1.920576471	-0.200393982	3.546757391	3.622652471	0.822068697	NA
ASV_16s_2695	1.880153682	-2.355943922	3.382238787	1.934386975	0.963382396	NA
ASV_16s_2696	2.460640177	5.895143901	3.549150974	2.233816454	0.94577422	1
ASV_16s_2697	2.01185572	-0.964142586	3.547868551	3.213246008	0.864599432	1
ASV_16s_2698	2.038019444	14.60537898	3.551478935	2.032442741	0.958022914	1
ASV_16s_2699	1.744143854	-0.34572346	3.549033924	3.158313428	0.869980839	NA
ASV_16s_2700	2.117891497	-0.83010359	3.547593783	2.235837485	0.945642935	1
ASV_16s_2701	1.753349365	2.348228208	3.545654173	2.988266286	0.886085569	NA
ASV_16s_2702	2.366169601	1.228363637	2.353788412	6.858020028	0.44381112	1
ASV_16s_2703	1.240043421	14.80780311	3.550996392	0.940937466	0.995725872	NA
ASV_16s_2704	1.690788342	-0.020065152	2.550924161	2.701472761	0.91117798	NA
ASV_16s_2705	2.959914691	6.064262865	3.55004078	2.579122757	0.92102214	1
ASV_16s_2706	2.855750047	6.53566061	3.550069675	2.455798632	0.930388531	1
ASV_16s_2707	2.608306364	2.234211602	3.548525891	2.9227064	0.892059233	1
ASV_16s_2708	1.943497724	0.258348808	2.855298787	4.737410575	0.691972255	NA
ASV_16s_2709	1.926724083	-0.901518199	3.547569237	1.882775801	0.966042475	NA
ASV_16s_2710	2.033853235	-6.810686929	2.95015379	1.277563728	0.989030017	1
ASV_16s_2711	2.479856816	0.334049347	2.853728534	3.014453254	0.883661974	1
ASV_16s_2712	1.992320883	0.68634076	3.549102656	3.091916069	NA	NA
ASV_16s_2713	2.532909986	2.241765271	3.546794884	3.342933596	0.851571023	1
ASV_16s_2714	0.558581592	-6.98312679	3.552157344	-0.072766691	NA	NA
ASV_16s_2715	1.767187837	0.018741752	3.54798134	3.647461859	0.819368667	NA
ASV_16s_2716	1.933883418	-0.541376248	3.546894129	2.903809289	0.893755735	NA
ASV_16s_2717	1.73584451	1.277035737	3.547447813	3.430093038	NA	NA
ASV_16s_2718	1.972053163	-0.491617503	3.546815627	3.554877684	0.829379182	NA
ASV_16s_2719	2.145980021	-0.830060046	2.981138073	3.350985969	0.850747671	1
ASV_16s_2720	2.163903084	0.475242685	3.550617986	4.150935179	0.762237812	1
ASV_16s_2721	2.312117876	0.694079841	3.550584376	2.119517475	0.952930027	1
ASV_16s_2722	0.532445556	-1.229595412	3.548976767	0.590466511	NA	NA
ASV_16s_2723	1.923074945	1.191941009	2.59742434	5.613987392	0.585472809	NA
ASV_16s_2724	1.426114321	3.323357352	3.546938503	0.614856927	NA	NA
ASV_16s_2725	2.256785827	0.366812199	3.550465027	1.400291805	0.985562325	1
ASV_16s_2726	1.708750153	-0.306026727	3.549045067	3.184329538	0.867442696	NA
ASV_16s_2727	0.369074015	-0.170262959	3.550578317	1.112671022	0.992800662	NA
ASV_16s_2728	1.92571675	-0.474227576	3.546911935	3.639429032	0.820244263	NA
ASV_16s_2729	1.234617604	0.644578504	3.550253916	0.827729459	0.997150559	NA
ASV_16s_2730	1.985738052	1.104234797	1.694060423	11.54491094	0.116553306	NA
ASV_16s_2731	1.857822075	-0.339953837	3.547722981	3.206193192	0.865295044	NA
ASV_16s_2732	1.692292506	0.692541392	3.547953684	3.49407773	NA	NA
ASV_16s_2733	2.012034598	-0.286432056	2.303555351	6.926058559	0.436619066	1
ASV_16s_2734	2.382736309	-0.713904669	2.919902757	4.198894109	0.756604931	1
ASV_16s_2735	2.285399298	-0.074031133	2.029097849	11.64746286	0.112764673	0.562893349
ASV_16s_2736	2.111967464	0.024660095	3.547324611	1.846431415	NA	NA
ASV_16s_2737	1.43990527	1.891079666	3.546533953	1.513397314	0.981842218	NA
ASV_16s_2738	1.440687528	-1.440593322	3.548468468	0.914322131	NA	NA
ASV_16s_2739	1.86594553	0.21407053	3.546637953	3.571322751	0.827614338	NA
ASV_16s_2740	1.679678685	2.747517629	3.545105571	2.57561022	0.921296747	NA
ASV_16s_2741	2.332878383	-7.3327222	2.924828797	3.728696076	0.81044211	1
ASV_16s_2742	2.326287609	1.676855595	3.548041206	2.260500052	0.944027668	1
ASV_16s_2743	1.682752279	-11.05964588	3.549256814	1.454649981	NA	NA
ASV_16s_2744	1.444555134	-0.233462385	3.548365928	1.994537614	NA	NA
ASV_16s_2745	1.673058514	0.29329631	3.548977261	3.25085678	NA	NA
ASV_16s_2746	1.722334054	1.083201605	3.547061133	3.431149564	0.84246334	NA
ASV_16s_2747	1.840905082	-0.62630414	3.548058032	2.190720596	0.948534525	NA
ASV_16s_2748	2.107879511	0.734658973	1.9495724	7.706575266	0.359177441	1
ASV_16s_2749	2.192970397	-10.68976198	3.549444435	3.081487316	0.877363008	1
ASV_16s_2750	2.697748961	1.130612795	3.54930607	1.993627552	0.960192226	1
ASV_16s_2751	2.015533877	-15.18069893	3.552157344	0.767590068	0.997760811	1

ASV_16s_2752	1.730764489	-0.186164898	3.549042499	4.060420637	0.772792429	NA
ASV_16s_2753	1.797882551	-0.087675126	3.547188103	2.936913839	0.890776215	NA
ASV_16s_2754	2.015399179	-0.678086947	3.157253483	3.591952023	0.825392242	1
ASV_16s_2755	2.149183926	-0.278270152	2.456103436	9.248186866	0.235325046	0.949152858
ASV_16s_2756	2.209635435	-0.008676885	3.54945341	1.757787774	0.972023382	1
ASV_16s_2757	2.013457429	0.292774182	3.549225603	4.386801075	0.734301946	1
ASV_16s_2758	2.290730913	0.315939099	3.550618599	2.180424393	0.949182887	1
ASV_16s_2759	1.005773636	13.10798899	3.551629329	0.576742682	0.999114155	NA
ASV_16s_2760	1.843746051	-0.73553887	3.548196722	2.841582601	0.899260136	NA
ASV_16s_2761	1.807553895	0.115919349	3.547872703	3.917574724	0.789220787	NA
ASV_16s_2762	1.944626331	-0.733640611	2.657707276	5.85879427	0.556330774	NA
ASV_16s_2763	2.027738306	-0.863453617	3.546771566	2.350715125	0.937912814	1
ASV_16s_2764	2.314560764	-0.479015661	3.001500945	5.006429398	0.659178577	1
ASV_16s_2765	2.074770353	1.185986102	1.939256311	10.79027097	0.148032536	0.681343669
ASV_16s_2766	1.386319161	-3.799165646	3.547228731	0.280898174	NA	NA
ASV_16s_2767	1.646385109	-0.290251781	3.549056143	3.161603438	NA	NA
ASV_16s_2768	0.753513555	17.70195758	3.551446413	0.02938881	NA	NA
ASV_16s_2769	1.644976732	1.901246335	3.546662538	3.042767904	NA	NA
ASV_16s_2770	2.029462116	-0.857945563	3.197723521	3.110268082	0.874617537	1
ASV_16s_2771	2.144042453	-0.400544809	2.256461368	6.310977897	0.503943236	1
ASV_16s_2772	2.345562327	0.476480963	3.549739282	0.746574046	NA	NA
ASV_16s_2773	0.980270616	-5.618844772	3.549496517	1.022927474	NA	NA
ASV_16s_2774	1.770000696	-0.757966643	3.54878883	1.814505076	0.969390255	NA
ASV_16s_2775	1.211086252	18.77733539	3.54916382	0.424511744	NA	NA
ASV_16s_2776	2.248147477	1.571558086	3.548473782	2.576632019	0.921216911	1
ASV_16s_2777	2.353750237	0.541793328	3.549310395	3.165881746	0.869244436	1
ASV_16s_2778	1.757531029	1.862394039	3.296338146	3.69082168	0.814620037	NA
ASV_16s_2779	2.086869179	-0.796217026	3.547884373	2.92235341	0.892091028	1
ASV_16s_2780	1.907134694	0.518101136	3.546364933	3.026943615	0.882498584	NA
ASV_16s_2781	1.946824536	-0.360369917	2.849041394	4.007835848	0.778874429	NA
ASV_16s_2782	2.090830444	-0.796669304	3.547788078	2.885948392	0.895348612	1
ASV_16s_2783	2.33807094	0.867739274	3.549489358	2.480724532	0.928541519	1
ASV_16s_2784	1.845863944	0.897404845	3.336692212	3.345911995	0.851266674	NA
ASV_16s_2785	2.050715493	0.613187275	3.546603322	1.646206038	0.97681235	1
ASV_16s_2786	1.421155523	19.13198545	3.551463117	0.930699996	NA	NA
ASV_16s_2787	1.822797891	-0.168046381	3.547849827	3.613856707	0.823022912	NA
ASV_16s_2788	1.718544514	0.685835261	3.547527214	3.408518659	0.844817979	NA
ASV_16s_2789	2.131873985	-0.541586953	2.846816711	4.236956572	0.752115942	1
ASV_16s_2790	2.039722527	-1.021609691	3.428449532	2.719712835	0.909664742	1
ASV_16s_2791	2.389595853	-0.089978395	2.784579624	5.270787377	0.626955212	1
ASV_16s_2792	0.933020927	0.152022773	3.548958857	2.113375145	NA	NA
ASV_16s_2793	1.641753096	0.689163932	3.54868079	3.329431394	0.85294793	NA
ASV_16s_2794	2.277748496	0.672967664	2.658784263	6.780941996	0.452039084	1
ASV_16s_2795	2.275870902	17.51753037	3.550764927	0.544612564	0.999266178	1
ASV_16s_2796	1.906461874	0.097411713	3.281211687	3.856772225	0.796118838	NA
ASV_16s_2797	2.001968855	-0.482191208	3.260043124	4.365482244	0.736849025	1
ASV_16s_2798	1.914823733	-0.782620505	2.57926632	3.021656146	0.882991656	NA
ASV_16s_2799	2.182773397	-0.30174197	3.262288782	4.534886316	0.716512402	1
ASV_16s_2800	2.011501518	-0.53145858	2.544203301	4.369215217	0.736403307	1
ASV_16s_2801	1.794490618	3.123980026	3.54711325	0.747139349	0.997946744	NA
ASV_16s_2802	1.796985614	2.768940664	3.547560755	0.787811824	NA	NA
ASV_16s_2803	0.571698912	-0.683431059	3.549514578	1.033806071	NA	NA
ASV_16s_2804	1.758408626	0.408757014	3.548762756	3.397695535	0.8459397	NA
ASV_16s_2805	1.774951654	1.113404435	3.433356395	2.522222079	0.925414445	NA
ASV_16s_2806	1.866503939	0.36516476	3.549597336	1.402600987	0.985491466	NA
ASV_16s_2807	1.117490745	-15.31407544	3.552157344	0.352145515	0.999828202	NA
ASV_16s_2808	1.876385788	3.395027371	3.546081658	0.650986899	NA	NA
ASV_16s_2809	2.330292385	0.846301138	3.474774541	2.536133992	0.92435165	1
ASV_16s_2810	1.663424517	0.141186332	3.548411319	3.591293646	NA	NA
ASV_16s_2811	1.696909071	-0.932523556	3.547369907	2.361461901	0.937162993	NA
ASV_16s_2812	1.724314888	0.203382802	3.546706257	3.516380824	0.83348741	NA
ASV_16s_2813	1.851698917	0.80725186	3.547293717	1.884218062	0.965969651	NA
ASV_16s_2814	2.281130314	0.917773819	3.546499747	2.546809968	NA	NA
ASV_16s_2815	1.668108851	-1.107058095	2.936242303	1.478998112	0.98302785	NA
ASV_16s_2816	1.813079632	-0.429230304	2.740422475	5.178200025	0.638226732	NA
ASV_16s_2817	2.104354116	-0.696865864	2.634842035	5.895183647	0.552040282	1
ASV_16s_2818	1.661492094	0.4874961	2.368131474	1.624878724	0.977668908	NA

ASV_16s_2819	1.033933181	1.339887112	3.549339159	1.119169947	NA	NA	
ASV_16s_2820	2.147205699	1.220815088	3.549723487	2.496351017	0.927371586		1
ASV_16s_2821	1.60532427	-0.298116218	3.549060179	3.129976343	NA	NA	
ASV_16s_2822	1.58523713	0.829179398	3.548585041	2.971360132	NA	NA	
ASV_16s_2823	1.735272516	1.12330189	3.049402515	4.325560825	0.741607939	NA	
ASV_16s_2824	1.823969205	1.070501629	3.547126879	3.490081657	0.836274904	NA	
ASV_16s_2825	1.928043728	5.04E-04	2.427893809	3.859391138	0.795822971	NA	
ASV_16s_2826	1.028674386	1.700271496	3.548017046	1.755143709	NA	NA	
ASV_16s_2827	0.888516795	2.090804841	3.548933666	0.770020928	NA	NA	
ASV_16s_2828	1.206195754	16.74988576	3.551448947	0.358608892	NA	NA	
ASV_16s_2829	1.638158293	-1.009571109	3.54805933	1.891869262	NA	NA	
ASV_16s_2830	2.558490215	1.912565547	3.547485986	2.06894443	0.955926006		1
ASV_16s_2831	2.100501865	-0.071164007	3.549985891	1.395032986	0.985722914		1
ASV_16s_2832	2.200703414	0.335691693	3.55060355	1.670626767	0.975808446		1
ASV_16s_2833	1.735970503	0.072512409	3.548245109	3.514693977	0.833666671	NA	
ASV_16s_2834	1.827931497	0.018231148	2.559234449	6.170314312	0.520009535	NA	
ASV_16s_2835	1.769671793	0.014328556	2.88835438	4.300632562	0.744572124	NA	
ASV_16s_2836	1.318043841	0.943385088	3.550036281	1.227224532	0.99028649	NA	
ASV_16s_2837	2.303016806	-2.872365248	3.548786984	2.827074966	0.900525087		1
ASV_16s_2838	1.729464749	0.607205521	3.546749711	3.194989749	0.866397207	NA	
ASV_16s_2839	2.191879594	1.342551664	3.549056992	1.4252257	NA	NA	
ASV_16s_2840	2.272181189	0.574452339	1.648522229	15.85467825	0.026477714		0.192609753
ASV_16s_2841	1.852725437	0.431208704	3.549314798	2.801826454	0.902709761	NA	
ASV_16s_2842	2.247081752	0.363382922	3.549913984	1.748340824	0.972448913		1
ASV_16s_2843	1.714677457	0.112884664	3.548205479	3.554124783	0.829459841	NA	
ASV_16s_2844	1.673298213	1.408220376	3.546626701	3.222249375	0.863709455	NA	
ASV_16s_2845	1.686880407	0.867723025	3.547818295	3.370693308	0.848725732	NA	
ASV_16s_2846	1.815464031	-0.316979282	3.547022229	3.296002287	0.856336791	NA	
ASV_16s_2847	1.831354589	3.08295936	3.36452732	2.753474237	0.906833233	NA	
ASV_16s_2848	1.630031751	0.431269845	3.548787539	3.29792331	0.856142832	NA	
ASV_16s_2849	1.69885662	0.134794816	3.548004629	3.723260245	0.811043421	NA	
ASV_16s_2850	1.973402844	-2.342676041	3.544899006	2.018117594	0.958830793	NA	
ASV_16s_2851	1.673266618	-0.939149282	3.54702938	2.222742113	0.946490686	NA	
ASV_16s_2852	1.735696069	-1.236836109	2.94983622	2.846377084	0.898840556	NA	
ASV_16s_2853	1.81520406	2.675700306	3.547652779	0.839650811	NA	NA	
ASV_16s_2854	2.193923704	1.605075653	2.926002266	3.06802761	0.878638583		1
ASV_16s_2855	2.150020141	0.50599454	2.748706505	6.397326671	0.49419501		1
ASV_16s_2856	2.132264915	0.676874035	3.550589347	2.779285362	0.904641985		1
ASV_16s_2857	0.557114756	-0.683340724	3.549534949	1.025474759	NA	NA	
ASV_16s_2858	0.557114756	-0.683340724	3.549534949	1.025474759	NA	NA	
ASV_16s_2859	1.729509472	-0.264568684	3.547581945	3.479573369	0.837384291	NA	
ASV_16s_2860	1.722633344	-0.003709368	3.546809164	3.636822156	NA	NA	
ASV_16s_2861	2.215827305	0.315939155	3.550619662	2.16345683	0.950241962		1
ASV_16s_2862	1.692833322	-0.186146378	3.5480028	3.241356666	0.861813396	NA	
ASV_16s_2863	1.659667142	1.683863065	3.5467725	3.127688657	NA	NA	
ASV_16s_2864	1.935649893	0.588315145	3.003450861	5.229620884	0.631964005	NA	
ASV_16s_2865	2.059998894	-0.946392635	2.527910048	3.507915278	0.834386395		1
ASV_16s_2866	2.019282676	-0.13478062	2.742317534	6.917826262	0.437485687		1
ASV_16s_2867	1.700799042	-0.83460881	3.546779787	2.667125575	0.913995686	NA	
ASV_16s_2868	2.24087509	0.471031113	3.549729876	0.755013121	NA	NA	
ASV_16s_2869	1.744340107	-1.45271856	3.547812195	0.903291576	NA	NA	
ASV_16s_2870	1.298417255	-1.722338263	3.548249466	0.785031991	NA	NA	
ASV_16s_2871	0.554197925	-0.683321177	3.54953915	1.023782314	NA	NA	
ASV_16s_2872	1.668874511	0.071144134	3.54792416	3.724912057	0.810860757	NA	
ASV_16s_2873	1.685445116	0.250336523	3.54789451	3.312640168	0.854653739	NA	
ASV_16s_2874	1.802861454	0.112856311	3.547766445	4.177692998	0.759098336	NA	
ASV_16s_2875	2.261749588	4.885533399	1.996971231	2.762486228	0.906070751		1
ASV_16s_2876	2.132514112	0.364915858	3.550520455	1.382198115	0.986110289		1
ASV_16s_2877	0.520153375	-6.731656836	3.552157344	-0.116407699	NA	NA	
ASV_16s_2878	1.675216416	-0.183373096	3.547847841	3.386095034	0.847138814	NA	
ASV_16s_2879	1.661743348	0.469626568	3.547595592	3.27532723	0.858418161	NA	
ASV_16s_2880	1.760363605	0.210687353	3.259319336	3.804213144	0.802031933	NA	
ASV_16s_2881	1.607068168	0.150130297	3.547914619	3.791179133	0.803490874	NA	
ASV_16s_2882	2.167955552	-0.517786955	3.406790992	2.825148537	0.900692528		1
ASV_16s_2883	2.027343796	0.777809135	3.549088784	3.226897043	NA	NA	
ASV_16s_2884	1.568371598	0.121984539	2.739882435	1.904431585	0.964939859	NA	
ASV_16s_2885	2.24332087	11.68268911	3.549386047	1.445191845	0.984146698		1

ASV_16s_2886	1.568815929	-0.317062283	3.549061873	3.088666535	NA	NA	
ASV_16s_2887	0.557220725	0.077550296	3.550578851	3.130511061	NA	NA	
ASV_16s_2889	1.897243916	-0.814202202	3.547985902	2.719897881	0.90964933	NA	
ASV_16s_2890	1.656051514	0.754468828	3.547581907	3.520341526	0.833066258	NA	
ASV_16s_2891	1.759762773	-0.972611565	3.546984197	3.121805915	0.873510122	NA	
ASV_16s_2892	1.942032984	-0.464295029	2.081220925	10.50838921	0.161546672	NA	
ASV_16s_2893	2.022012724	-0.264565058	2.510104609	4.234664312	0.75238673		1
ASV_16s_2894	1.768287423	-0.930301367	2.81888875	3.666152835	0.817326274	NA	
ASV_16s_2895	1.970266949	0.732273097	3.549100148	3.153210834	NA	NA	
ASV_16s_2896	1.735211067	2.134263532	3.548316085	0.857225243	NA	NA	
ASV_16s_2897	2.09290067	0.854438267	3.486184207	3.685287941	0.815228144		1
ASV_16s_2898	2.089169713	0.53326284	3.377933471	4.500166999	0.720697164		1
ASV_16s_2899	2.14969086	0.694069543	3.550586805	2.082361029	0.955141447		1
ASV_16s_2900	1.985901656	-0.842903006	3.240644067	2.686694209	0.912395469	NA	
ASV_16s_2901	2.167083776	-0.127743868	3.549675634	1.576702964	0.979534785		1
ASV_16s_2902	2.24965073	0.784951513	3.549339039	2.836401803	0.899712665		1
ASV_16s_2903	2.046310895	-0.905021762	3.547452196	2.509494385	0.926380433		1
ASV_16s_2904	1.95058071	0.630204347	2.99894681	5.21553694	0.633678699	NA	
ASV_16s_2905	1.742213042	-1.076117328	3.547349328	2.128557282	0.952383446	NA	
ASV_16s_2906	2.068306342	-0.045870475	2.993954345	2.254238526	0.944440073		1
ASV_16s_2907	1.748308464	-1.115626152	3.547607699	2.46191389	0.929937577	NA	
ASV_16s_2908	1.688423472	-0.077312634	3.549608658	2.369758727	0.93658102	NA	
ASV_16s_2909	2.622307001	-0.444430956	3.547930021	1.766586748	NA	NA	
ASV_16s_2910	1.94268559	0.190166045	2.936557437	3.770425321	0.805807653	NA	
ASV_16s_2911	2.37470857	0.088124719	3.549255821	5.195977496	0.636060846		1
ASV_16s_2912	1.759355166	0.026231831	3.546810211	3.169349795	0.868906452	NA	
ASV_16s_2913	1.647239832	0.569594333	3.33233516	2.723401665	0.909357293	NA	
ASV_16s_2914	2.071638984	-1.006269897	2.842278882	3.098085064	0.875782671		1
ASV_16s_2915	1.980226335	-0.142140827	3.546892138	4.049778379	0.774026393	NA	
ASV_16s_2916	1.867484253	2.10346165	2.723329774	4.169646853	0.760043269	NA	
ASV_16s_2917	1.937169879	2.372865831	3.545583385	2.297280676	NA	NA	
ASV_16s_2918	2.02802543	1.106263275	3.547136166	2.279970574	0.942735266		1
ASV_16s_2919	1.70872372	0.068203959	3.547930682	3.733784613	0.809878711	NA	
ASV_16s_2920	1.598889361	0.010042773	3.547974535	3.554137304	0.8294585	NA	
ASV_16s_2921	2.040321128	-0.638019608	2.797931403	2.928799859	0.891509745		1
ASV_16s_2922	1.081132226	0.198964002	3.550557807	0.925892102	0.995937088	NA	
ASV_16s_2923	1.059374622	0.323322579	3.549813408	1.254503949	NA	NA	
ASV_16s_2924	2.155203134	0.542831141	2.972246141	2.657411677	0.914784978		1
ASV_16s_2925	0.631700451	-0.458833771	3.550741474	0.959161137	NA	NA	
ASV_16s_2926	0.516695989	-22.05679878	3.551441403	-0.131697345	NA	NA	
ASV_16s_2927	1.587445862	-0.034419667	3.548001139	3.435195053	0.842041134	NA	
ASV_16s_2928	1.706138472	0.013845927	3.547815269	3.885714912	0.792842778	NA	
ASV_16s_2929	1.662824771	-0.035910955	3.54706427	3.086180274	0.876916996	NA	
ASV_16s_2930	1.679070187	-0.747213802	2.89130038	3.465945424	0.838819236	NA	
ASV_16s_2931	1.98039641	-0.318707849	3.524005759	2.053162909	0.956839358	NA	
ASV_16s_2932	1.78221537	1.802355806	2.637613537	4.508302831	0.719717243	NA	
ASV_16s_2933	2.727195631	-0.104797432	3.546295784	2.595976222	0.919698257		1
ASV_16s_2934	2.469471247	3.22479063	3.548560235	1.589503725	0.979048309		1
ASV_16s_2935	1.685011014	0.078484118	3.547628414	3.072450288	0.878220039	NA	
ASV_16s_2936	1.762135653	0.207145972	3.545907184	3.221436875	0.863789861	NA	
ASV_16s_2937	2.113513401	-0.160282843	2.969609618	3.086017283	0.876932497		1
ASV_16s_2938	2.057246853	1.157645099	3.549867981	2.413292712	0.93348348		1
ASV_16s_2939	2.299917678	14.93081934	3.551466217	2.399575338	0.934467432		1
ASV_16s_2941	1.989395483	0.804904876	2.76184764	4.358675992	0.737661382	NA	
ASV_16s_2942	0.565207605	0.121331555	3.550587628	3.189789267	0.866907634	NA	
ASV_16s_2943	1.580381358	0.477271868	3.547943537	3.477907792	0.837559897	NA	
ASV_16s_2944	1.538256215	0.629253962	3.547520028	2.907046853	NA	NA	
ASV_16s_2945	1.054839096	-14.13761417	3.552157344	0.316750183	0.999879791	NA	
ASV_16s_2946	1.103957728	3.077408245	3.54724175	0.639386343	NA	NA	
ASV_16s_2947	2.115414239	0.167641328	2.151846995	6.446146272	0.488723845		1
ASV_16s_2948	2.512197672	-4.26417062	3.544132122	0.371625123	NA	NA	
ASV_16s_2949	2.016244018	-0.101795356	3.549485477	1.999007357	0.959895289		1
ASV_16s_2950	2.153743534	0.190081389	3.044791605	6.245169552	0.511431743		1
ASV_16s_2951	1.725899809	2.039530629	2.593061362	5.294088385	0.624122439	NA	
ASV_16s_2952	2.169785361	-9.307056994	3.54812928	1.902097118	0.965059661		1
ASV_16s_2953	1.582379643	0.880190495	2.531253025	3.710130211	0.81249355	NA	
ASV_16s_2954	1.297837825	0.321849452	3.131359843	2.914567653	0.892791302	NA	

ASV_16s_2955	1.520021021	-0.784074315	3.547417087	3.235837805	0.862362062	NA	
ASV_16s_2956	2.175915511	1.942548229	2.860227623	1.992162117	0.960272902		1
ASV_16s_2957	2.20165563	0.34644163	3.548248204	2.918331135	0.892453046		1
ASV_16s_2958	2.539476646	0.405880933	3.54833301	3.219502669	0.8639812		1
ASV_16s_2959	1.903918194	-0.808696949	3.158722113	3.594461002	0.825121368	NA	
ASV_16s_2960	1.925907573	0.674482813	3.012206299	5.20136676	0.635404396	NA	
ASV_16s_2961	1.676298204	-0.788448015	3.54758188	2.077457857	0.955429028	NA	
ASV_16s_2962	1.741963568	-28.6867756	3.551464425	1.4442802	0.984176237	NA	
ASV_16s_2963	2.208511533	-0.250273316	3.548430345	2.22234466	0.946516308		1
ASV_16s_2964	1.689965187	0.442966876	3.549817093	0.790672532	NA	NA	
ASV_16s_2965	1.164469104	17.99643635	3.551443371	0.296105884	NA	NA	
ASV_16s_2966	2.030086491	30	3.549346468	1.313504618	0.988074538		1
ASV_16s_2967	2.021143455	0.360091463	3.550227352	1.752768115	0.972249952		1
ASV_16s_2968	0.970493068	-13.36093282	3.552157344	0.265654363	NA	NA	
ASV_16s_2969	2.471671088	-1.66928837	3.54556427	1.797487285	0.970194423		1
ASV_16s_2970	1.827269981	-0.092431061	2.839336498	2.833217169	0.899990391	NA	
ASV_16s_2971	1.820258132	-0.794772648	3.548194711	2.801311962	0.902754055	NA	
ASV_16s_2972	1.658560825	-0.990728912	3.548964077	1.652126207	0.976571247	NA	
ASV_16s_2973	3.323577734	-0.568886625	3.548802233	1.578880325	NA	NA	
ASV_16s_2974	2.016371595	-0.63082429	3.548953175	2.120289021	0.952883507		1
ASV_16s_2975	2.27203448	0.390595544	3.550174601	1.091534575	0.993213801		1
ASV_16s_2976	2.049848177	0.694064152	3.550588489	2.05810797	0.956554269		1
ASV_16s_2977	1.955019964	-0.796663084	3.54779574	2.805672889	0.902378325	NA	
ASV_16s_2978	1.895544656	-0.796382897	3.547851749	2.814532819	0.901612996	NA	
ASV_16s_2979	2.138004455	0.641724933	3.163764344	3.685328503	0.815223689		1
ASV_16s_2980	1.549785578	-21.65883452	3.550765155	1.800122686	0.97007068	NA	
ASV_16s_2981	2.251841625	0.534187528	3.54800764	4.19456916	0.757113991		1
ASV_16s_2982	2.178432365	0.915230425	3.549598572	3.671728799	0.816715627		1
ASV_16s_2983	1.659957876	-0.151598757	3.547864284	3.395399188	0.846177329	NA	
ASV_16s_2984	1.684973444	0.602385936	3.546937063	3.678332585	0.815991622	NA	
ASV_16s_2985	2.067600304	0.412091092	2.87934733	3.719499856	0.811459068		1
ASV_16s_2986	1.979682246	-0.922292921	2.975299747	3.179278718	0.867936943	NA	
ASV_16s_2987	2.046928817	0.360860615	3.55056082	1.404557381	0.985431267		1
ASV_16s_2988	1.131777507	16.70476362	3.551449695	0.319530342	NA	NA	
ASV_16s_2989	1.636643027	-0.310935812	3.54905346	3.13680947	0.872064308	NA	
ASV_16s_2990	1.54082004	-0.32436862	3.549064785	3.062728313	0.879139318	NA	
ASV_16s_2991	1.700898148	1.106960842	3.547056313	3.487270999	0.83657188	NA	
ASV_16s_2992	1.528951606	2.361795352	3.312959966	3.32915935	0.852975624	NA	
ASV_16s_2993	1.943703606	-0.798787149	3.157913173	3.561704766	0.828647227	NA	
ASV_16s_2994	1.872148431	-0.795945687	3.547950255	2.832124161	0.900085633	NA	
ASV_16s_2995	1.685514161	-0.787054897	3.549243556	1.158660657	NA	NA	
ASV_16s_2996	2.008069804	-0.926569405	2.99223949	3.18027328	0.867839677		1
ASV_16s_2997	1.195944984	1.340174858	3.549275415	1.166095025	NA	NA	
ASV_16s_2998	1.68661958	-0.100280487	3.549086965	3.166532396	NA	NA	
ASV_16s_2999	0.52940486	-0.683153168	3.549576723	1.009027868	NA	NA	
ASV_16s_3000	0.082950566	0.9897469	3.551294495	0.134065902	0.999993636	NA	
ASV_16s_3001	1.586227832	0.011470143	3.548403071	3.038930304	0.881377644	NA	
ASV_16s_3002	1.643246638	1.211709976	3.271373948	3.866845727	0.794980176	NA	
ASV_16s_3003	1.645954572	0.128692554	3.546228976	1.747644743	NA	NA	
ASV_16s_3004	1.530575458	-21.94646587	3.550805903	1.873008945	NA	NA	
ASV_16s_3005	0.753837748	17.1930874	3.550786232	0.416929325	NA	NA	
ASV_16s_3006	1.540729909	1.155030445	3.547203993	3.2376901	NA	NA	
ASV_16s_3007	1.924754813	-0.796740556	3.547784163	2.76389437	0.90595136	NA	
ASV_16s_3008	1.907585069	-0.821393963	3.547759774	2.264571076	0.943758695	NA	
ASV_16s_3009	1.983103024	-0.64725706	3.54959116	1.555332736	0.98033198	NA	
ASV_16s_3010	2.005163634	0.239452907	2.474152139	2.825703788	0.90064428		1
ASV_16s_3011	1.088826669	19.91222233	3.551437823	0.195262535	0.999976826	NA	
ASV_16s_3012	1.7851589	0.748834363	2.983624826	5.743845207	0.569957265	NA	
ASV_16s_3013	1.981738252	0.271434828	3.549208206	1.41509366	NA	NA	
ASV_16s_3014	1.940057831	0.403731498	3.549232818	1.411113913	NA	NA	
ASV_16s_3015	2.549761078	-0.568734445	3.548839466	1.493328308	0.982539735		1
ASV_16s_3016	0.526488028	-0.683132336	3.549581375	1.00724695	NA	NA	
ASV_16s_3017	1.766650316	-0.469755391	3.547828476	2.617032441	0.91802967	NA	
ASV_16s_3018	1.6038228	0.812321611	3.547282806	3.454725381	0.839997401	NA	
ASV_16s_3019	1.432010651	2.067221961	3.108934754	3.552040753	0.829683041	NA	
ASV_16s_3020	1.862216823	-0.469192072	3.084986604	3.28183306	0.857764427	NA	
ASV_16s_3021	1.828574592	-0.794731092	3.548203502	2.803672085	0.902550791	NA	

ASV_16s_3022	1.521031597	-2.388373776	3.546092347	0.673060573	0.998534272	NA	
ASV_16s_3023	0.440599488	0.304772593	3.550781734	0.967227131	NA	NA	
ASV_16s_3024	2.100489311	0.566866497	3.515814983	3.34652996	0.851203498		1
ASV_16s_3025	1.52432291	1.064706315	3.547512607	3.152436814	0.870551518	NA	
ASV_16s_3026	1.725215848	-0.345920555	3.546814895	3.476605967	0.837697107	NA	
ASV_16s_3027	1.878173332	0.242050188	3.423507194	2.051694096	0.956923842	NA	
ASV_16s_3028	2.460291091	0.271425142	3.549149206	1.484898654	NA	NA	
ASV_16s_3029	1.026058465	-14.14664638	3.552157344	0.299780817	0.99990021	NA	
ASV_16s_3030	2.080166633	-0.738708604	3.299735202	2.617671172	NA	NA	
ASV_16s_3031	1.9955347	1.206795387	3.549053204	1.383849192	NA	NA	
ASV_16s_3032	0.470238381	-0.607348248	3.550049551	0.960095227	NA	NA	
ASV_16s_3033	2.031381091	0.347197408	3.550393294	1.506264358	0.982091995		1
ASV_16s_3034	2.20501949	11.65017505	3.549408172	1.296121577	0.988542792		1
ASV_16s_3035	1.578191377	-0.285654841	3.549066548	3.123355849	0.873361064	NA	
ASV_16s_3036	1.657045051	2.083602352	3.545885419	2.957024312	0.888949156	NA	
ASV_16s_3037	1.510293045	-0.762223408	2.926581353	3.531850903	0.831840447	NA	
ASV_16s_3038	1.910375013	-0.559219751	2.783926333	2.286749239	0.94228177	NA	
ASV_16s_3039	1.405278952	-0.199752519	3.549509855	0.996710187	0.994881431	NA	
ASV_16s_3040	1.647497277	3.037290367	3.546728076	0.638373855	NA	NA	
ASV_16s_3041	2.015204482	0.366266781	3.550562744	1.187229339	0.991217973		1
ASV_16s_3042	0.749304202	-21.14169403	3.551447985	1.100580768	0.993038871	NA	
ASV_16s_3043	1.558815966	1.430173474	3.547203274	3.091862527	NA	NA	
ASV_16s_3044	1.562742308	1.263724367	3.547232975	3.239362426	0.862011751	NA	
ASV_16s_3045	1.614740059	0.496296612	3.546638392	3.410881605	0.844572702	NA	
ASV_16s_3046	2.529747673	0.41942877	3.549109165	1.427142166	NA	NA	
ASV_16s_3047	1.065032036	-20.52521977	3.551062583	0.429706937	0.999665344	NA	
ASV_16s_3048	1.165474565	-8.102912235	3.549920833	0.48032777	NA	NA	
ASV_16s_3049	1.572912068	0.566515634	3.547867734	3.339307783	0.851941226	NA	
ASV_16s_3050	1.61296355	0.518485258	3.547006161	3.479287314	0.837414455	NA	
ASV_16s_3051	1.67293708	0.462164145	3.545733573	3.121799615	NA	NA	
ASV_16s_3052	1.783201141	-0.255837705	2.887602278	4.170899992	0.75989615	NA	
ASV_16s_3053	1.791427895	-0.827545906	3.54782532	1.960556614	0.961991144	NA	
ASV_16s_3054	2.037983798	0.715817654	3.546632629	3.464223998	0.839000185		1
ASV_16s_3055	2.074114501	4.520361099	2.801285619	0.479548631	0.999517974		1
ASV_16s_3056	1.532261773	1.714918943	3.546592596	2.850092537	0.898514882	NA	
ASV_16s_3057	1.794152327	-0.614012381	3.17923459	3.351103629	0.850735628	NA	
ASV_16s_3058	1.913356034	-0.798189904	3.159781951	3.586579194	0.825971853	NA	
ASV_16s_3059	1.685505317	-0.613762785	3.547296506	2.293394176	0.941835447	NA	
ASV_16s_3060	1.976841965	-0.55494158	3.549126652	1.554921323	0.980347144	NA	
ASV_16s_3061	2.100391359	0.939766365	3.060727535	4.11207568	0.76678181		1
ASV_16s_3062	1.855035253	-0.746113647	3.002045971	3.336052999	0.852273261	NA	
ASV_16s_3063	1.80124477	-0.683340538	3.547888041	2.753496918	0.906831318	NA	
ASV_16s_3064	1.363173918	-8.8384758	3.547203165	0.462294905	NA	NA	
ASV_16s_3065	2.034134706	17.40639512	3.551443369	0.673698506	0.998529764		1
ASV_16s_3066	1.813936799	-0.796121326	3.547914479	2.794322797	0.903354888	NA	
ASV_16s_3067	1.675179212	2.278074559	3.54745075	1.701527886	NA	NA	
ASV_16s_3068	0.497774081	-0.815529774	3.549322601	1.183258443	0.991307273	NA	
ASV_16s_3069	2.061286244	-0.091270093	3.549097768	1.328001176	0.987675199		1
ASV_16s_3070	1.996567318	0.694056789	3.550589457	2.044669724	0.957326643		1
ASV_16s_3071	1.475206377	0.080043212	3.548104353	3.467497077	NA	NA	
ASV_16s_3072	1.564747105	1.77049795	3.545911263	2.653674313	0.915087755	NA	
ASV_16s_3073	1.879666034	-0.927204963	3.22254005	2.859665696	0.897673653	NA	
ASV_16s_3074	1.681978332	0.17670696	3.548073804	2.59852947	0.919496786	NA	
ASV_16s_3075	2.000528747	-0.078114433	3.540981509	2.120775402	0.952854168		1
ASV_16s_3076	1.866349958	3.011446872	2.643808415	2.545734147	0.923614045	NA	
ASV_16s_3077	2.031199953	0.694062553	3.550588823	2.053435648	0.956823661		1
ASV_16s_3078	1.608901663	2.985294286	3.5443302	2.495522779	0.927433827	NA	
ASV_16s_3079	1.245010984	0.943194883	3.548248246	2.206400843	NA	NA	
ASV_16s_3080	1.006580603	-7.396857267	3.552157344	0.288030615	0.999912847	NA	
ASV_16s_3081	2.008700534	-0.386810952	2.890961162	4.606985172	0.707799099		1
ASV_16s_3082	2.146282376	-7.179981949	3.550176968	2.4939336	0.927553178		1
ASV_16s_3083	1.479749853	0.834146366	3.548018139	3.311487894	NA	NA	
ASV_16s_3084	1.524903541	0.266796786	3.54786256	3.647241924	0.819392658	NA	
ASV_16s_3085	1.793311695	-0.757314203	2.681102819	4.376962632	0.73547788	NA	
ASV_16s_3086	1.733947166	-2.309889418	3.486782202	1.120983356	0.99263394	NA	
ASV_16s_3087	1.578271187	-0.104296854	3.549298407	2.673723749	0.913457642	NA	
ASV_16s_3088	1.605928649	3.278115795	2.874339165	1.967199664	0.961633441	NA	

ASV_16s_3089	1.695303449	2.450261438	2.952546035	2.531519315	0.924704986	NA	
ASV_16s_3090	1.196654923	0.163088102	3.550597426	1.114921985	0.992755752	NA	
ASV_16s_3091	2.15656422	4.073568607	3.550875035	3.226966995	0.863242238		1
ASV_16s_3092	1.781937236	-12.33883593	3.552157344	0.571765219	0.999138981	NA	
ASV_16s_3093	0.510445457	-0.68301037	3.549607899	0.997272381	NA	NA	
ASV_16s_3094	2.045173219	3.771841117	2.69773242	2.264359443	0.943772694		1
ASV_16s_3095	1.916045271	0.271436519	3.549218605	1.404207473	NA	NA	
ASV_16s_3096	0.427174019	0.303290713	3.550823925	0.956900874	NA	NA	
ASV_16s_3097	2.037207184	1.517742671	3.549450143	1.683988333	0.975248707		1
ASV_16s_3098	1.635087785	-0.29913899	3.547479658	3.52315138	0.832767265	NA	
ASV_16s_3099	1.799596272	2.486910865	3.546749355	0.698601645	0.998346458	NA	
ASV_16s_3100	1.845969644	-0.798731098	3.171853885	3.43274898	0.842296464	NA	
ASV_16s_3101	1.777209986	0.371529008	2.335140308	7.566161469	0.372397237	NA	
ASV_16s_3102	1.863443067	-0.796139017	3.547907966	2.819447275	0.901187343	NA	
ASV_16s_3103	1.774974173	-0.773880361	3.547230027	2.822884023	0.900889196	NA	
ASV_16s_3104	1.807092192	-1.203414157	2.305695683	4.495284213	0.72128506	NA	
ASV_16s_3105	1.897658856	1.085403382	2.668194356	5.4157399	0.609365723	NA	
ASV_16s_3106	1.996029618	-0.540674299	2.874041233	5.335265338	0.619120999	NA	
ASV_16s_3107	1.534629608	1.583898383	3.544238881	1.614990477	0.978059675	NA	
ASV_16s_3108	1.99405567	-0.05284072	3.549889087	1.444566259	0.984166972	NA	
ASV_16s_3109	2.400712445	0.331084677	3.548172485	3.129849896	0.872735772		1
ASV_16s_3110	2.100372487	0.315939175	3.550621447	2.136142816	0.951922216		1
ASV_16s_3111	1.993216922	0.694059546	3.55058952	2.04381104	0.957375743	NA	
ASV_16s_3112	1.535095678	-0.021031232	3.547918775	3.526605029	0.832399526	NA	
ASV_16s_3113	1.545299888	0.497893005	3.547184629	3.391972273	0.846531711	NA	
ASV_16s_3114	0.983248629	-14.1466935	3.552157344	0.273619764	0.999926771	NA	
ASV_16s_3115	1.884600302	-13.50172382	3.552157344	0.726319546	0.998125235	NA	
ASV_16s_3116	1.509576677	-0.350409339	3.549065526	3.008991819	0.88416917	NA	
ASV_16s_3117	1.518066788	0.074974179	3.548048283	3.40303286	0.84538689	NA	
ASV_16s_3118	1.613801806	0.00454279	3.547828325	3.802614512	0.802211035	NA	
ASV_16s_3119	1.608661989	0.789118306	3.54612137	2.781035187	NA	NA	
ASV_16s_3120	2.248376679	0.409226596	3.549806639	1.002801799	0.994783213		1
ASV_16s_3121	0.429065418	-0.075478201	3.55057894	1.999965834	0.95984226	NA	
ASV_16s_3122	1.489921943	0.0638883	3.547912232	3.64401029	0.819745056	NA	
ASV_16s_3123	1.562074474	1.83331773	3.077812406	4.019878947	0.777484913	NA	
ASV_16s_3124	1.844411638	-0.796461995	3.547846003	2.777983603	0.904753044	NA	
ASV_16s_3125	1.821403047	-0.792243296	2.988633345	3.518737341	0.833236878	NA	
ASV_16s_3126	2.004248248	0.240536322	3.547156747	3.329708337	0.852919735		1
ASV_16s_3127	2.359183995	0.271426892	3.549159688	1.471346572	NA	NA	
ASV_16s_3128	1.879302365	0.083637189	3.549206181	2.8527884	0.898278293	NA	
ASV_16s_3129	2.141959499	0.79673304	3.549345029	2.784777337	0.904172802		1
ASV_16s_3130	0.477909158	-22.633382	3.55144171	-0.179480201	NA	NA	
ASV_16s_3131	1.688250723	-0.969329748	3.54810616	2.349555744	0.937993435	NA	
ASV_16s_3132	1.870578761	1.236113996	3.033480533	4.571469344	0.712094948	NA	
ASV_16s_3133	1.60371329	0.97813675	2.582391372	3.793548184	0.803225922	NA	
ASV_16s_3134	1.986312312	2.056724802	3.546491277	1.485745383	0.982799054	NA	
ASV_16s_3135	1.871049096	-0.63876012	3.548453534	2.642091919	0.91602291	NA	
ASV_16s_3136	1.861712689	0.540939663	3.548142999	4.308171411	0.743676313	NA	
ASV_16s_3137	1.991092764	17.41783936	3.551443487	0.660566677	0.998620718	NA	
ASV_16s_3138	1.404844358	-0.386059194	3.549078972	2.88011652	NA	NA	
ASV_16s_3139	0.406280047	-0.127769378	3.550575803	1.636143907	0.977218812	NA	
ASV_16s_3140	1.853733427	-0.137619021	3.093963348	3.292392863	0.856700961	NA	
ASV_16s_3141	1.66826001	-0.51752657	3.547276868	2.240975999	0.945308401	NA	
ASV_16s_3142	1.517144431	-0.04333566	3.548982298	3.035092835	0.881736981	NA	
ASV_16s_3143	1.506900053	0.454647669	3.547654573	3.265468812	0.859406644	NA	
ASV_16s_3144	1.545899561	1.021348208	3.547063989	3.180524286	0.867815124	NA	
ASV_16s_3145	1.934002084	0.613029335	3.334506596	3.080763661	0.877431725	NA	
ASV_16s_3146	1.546346929	0.084722765	3.548238759	3.425810478	0.843019955	NA	
ASV_16s_3147	1.471317232	0.630025354	3.548015044	3.289814584	0.856960887	NA	
ASV_16s_3148	1.647050423	0.116389719	3.547796751	4.061718538	0.772641834	NA	
ASV_16s_3149	1.774927484	-0.906226981	3.547388399	1.922008791	0.96403051	NA	
ASV_16s_3150	1.809898961	-0.348281453	2.395283186	6.37973269	0.496173953	NA	
ASV_16s_3151	1.853426566	2.782196121	3.545223496	2.299228938	0.941442095	NA	
ASV_16s_3152	1.566955658	-0.061816224	3.549345233	2.845690164	0.898900717	NA	
ASV_16s_3153	2.167156454	0.213678471	3.546678665	2.450126699	0.930805519		1
ASV_16s_3154	1.953134996	0.360452076	3.550364899	1.410875453	0.985235829	NA	
ASV_16s_3155	2.242802222	0.324177031	3.548160629	3.202532439	0.86565556		1

ASV_16s_3156	2.040563793	0.920089335	3.549532794	2.186367536	0.948809166	1
ASV_16s_3157	2.217806328	4.540709464	3.548738463	2.206940979	NA	NA
ASV_16s_3158	1.560851831	2.163796301	2.91347757	4.155881374	0.761658116	NA
ASV_16s_3159	1.773860099	-1.034446472	3.547146561	2.236703286	0.945586643	NA
ASV_16s_3160	1.502582587	0.787008357	2.78638655	3.762539816	NA	NA
ASV_16s_3161	1.456490492	-0.103205625	3.547093735	3.23310356	0.862633586	NA
ASV_16s_3162	2.431127349	0.800975439	2.396289196	8.440414574	0.295363583	1
ASV_16s_3163	1.831646527	-0.099036503	3.548963652	1.950051252	0.962553067	NA
ASV_16s_3164	1.490685422	-0.274886878	3.549082233	3.074686167	0.878008224	NA
ASV_16s_3165	1.541545163	-0.24687341	3.547273157	3.214284814	0.86449686	NA
ASV_16s_3166	1.548081112	0.969195035	3.547218198	3.319200087	0.853988183	NA
ASV_16s_3167	1.431522254	-0.97544967	2.721526873	1.210334772	0.990686992	NA
ASV_16s_3168	1.698501092	0.410651054	3.148118121	2.648009091	0.915545762	NA
ASV_16s_3169	1.700372526	0.280243045	2.827825543	2.488300334	0.927975482	NA
ASV_16s_3170	2.001819844	1.181885127	3.549898312	1.679946632	0.975418803	1
ASV_16s_3171	1.462393358	-0.241271572	3.549091569	3.072772811	0.878189494	NA
ASV_16s_3172	0.61511229	-14.62303516	3.552157344	-0.01379061	NA	NA
ASV_16s_3173	0.698646933	21.23520976	3.551458044	0.131958453	NA	NA
ASV_16s_3174	1.76552633	-0.330743931	2.435229783	5.106214865	0.647003017	NA
ASV_16s_3175	1.778690822	-0.796276068	3.547865234	2.747621135	0.907326951	NA
ASV_16s_3176	1.824262891	-0.943504467	3.547012716	2.2920579	0.941925342	NA
ASV_16s_3177	1.787571602	-0.796269419	3.547872161	2.758612246	0.906398861	NA
ASV_16s_3178	1.45621097	0.336963362	3.548711711	3.071123372	0.878345674	NA
ASV_16s_3179	1.871147236	0.13068461	3.546966463	2.884136459	0.895509625	NA
ASV_16s_3180	1.812545592	-0.925384903	3.546863657	2.242394747	0.945215849	NA
ASV_16s_3181	1.609283424	-1.132206383	3.22438448	2.774371134	0.905060939	NA
ASV_16s_3182	1.502883335	-9.138454129	3.552157344	0.261257939	NA	NA
ASV_16s_3183	1.532832454	-0.745665313	3.548361261	2.156505236	0.950672483	NA
ASV_16s_3184	0.958937407	-15.66968652	3.552157344	0.258260062	0.999939821	NA
ASV_16s_3185	1.292338114	0.686964766	3.549716905	0.75291728	0.99789529	NA
ASV_16s_3186	1.13073035	16.16012422	3.550223289	0.541905858	NA	NA
ASV_16s_3187	1.828675162	0.532953303	3.133070754	4.753449289	0.690021424	NA
ASV_16s_3188	1.395459413	-0.271879537	3.549099381	3.011500691	NA	NA
ASV_16s_3189	0.491486054	-0.682859255	3.549641463	0.985073828	NA	NA
ASV_16s_3190	0.407164623	-0.087753744	3.5505804	1.888759089	0.965739791	NA
ASV_16s_3191	1.512593276	0.126373446	3.547565745	3.357312198	0.85009967	NA
ASV_16s_3192	1.611887925	2.152774481	3.545755942	2.921588674	0.892159896	NA
ASV_16s_3193	1.532664683	-0.518128772	3.546781983	2.536500273	0.92432357	NA
ASV_16s_3194	1.778789929	-0.797485813	3.187326481	3.193774149	0.866516585	NA
ASV_16s_3195	1.806075937	0.403723024	3.549256883	1.38800467	NA	NA
ASV_16s_3196	1.685147252	2.022332103	2.799014593	4.070217809	0.771655108	NA
ASV_16s_3197	1.787420438	0.690998479	3.549693022	1.64285266	0.976948275	NA
ASV_16s_3198	0.465441963	-22.34981814	3.551441833	-0.195662714	NA	NA
ASV_16s_3199	1.492873698	0.774880822	3.547389209	3.225801463	0.863357724	NA
ASV_16s_3200	1.718690512	-0.184186276	3.247162502	3.654846357	0.818562589	NA
ASV_16s_3201	1.95494452	0.416124374	3.549402181	1.127896686	NA	NA
ASV_16s_3202	1.174372376	-0.490056984	3.299425745	3.088571465	0.87668949	NA
ASV_16s_3203	1.594669442	-0.678635555	3.548512556	3.622367919	0.822099592	NA
ASV_16s_3204	1.544762732	0.761743536	3.265781043	3.429129713	0.842673995	NA
ASV_16s_3205	2.003455016	-0.912608633	3.324037423	3.186563557	0.867223859	1
ASV_16s_3206	0.970695065	-0.216548102	3.549709228	2.137248821	0.95185477	NA
ASV_16s_3207	2.367411502	-3.142506363	3.54534471	0.696050534	NA	NA
ASV_16s_3208	1.462928186	-0.241387444	3.549091465	3.07307566	0.87816081	NA
ASV_16s_3209	1.442655183	0.057410782	3.54904788	2.934554528	0.890989721	NA
ASV_16s_3210	1.474355975	-0.076515355	3.548341943	3.025325189	0.882649597	NA
ASV_16s_3211	1.474412859	0.627453658	3.548273006	3.160619913	0.869756588	NA
ASV_16s_3212	1.437305513	0.119907257	3.548160824	3.441248748	0.841408615	NA
ASV_16s_3213	1.422292444	0.034390062	3.548018442	3.448827366	0.840615542	NA
ASV_16s_3214	1.511744301	-0.082480834	3.54791489	3.407394119	0.84493466	NA
ASV_16s_3215	1.500307272	1.163207536	3.547529712	3.199617077	0.865942404	NA
ASV_16s_3216	1.506376074	0.823767569	3.547348261	3.116213519	0.874047369	NA
ASV_16s_3217	0.572966728	0.885298756	3.547303623	0.933934933	NA	NA
ASV_16s_3218	1.55122546	0.970119396	3.546897306	3.038498428	0.881418107	NA
ASV_16s_3219	1.742134339	-0.695530764	3.5478252	2.65558264	0.914933217	NA
ASV_16s_3220	1.907279877	-0.904547481	2.747115752	4.20732758	0.755611682	NA
ASV_16s_3221	1.118843686	2.571702708	3.547632477	1.579705433	0.979421283	NA
ASV_16s_3222	0.88547098	1.3395269	3.549418	1.069161286	NA	NA

ASV_16s_3223	1.260473229	0.254429755	3.550563915	0.437310909	0.999645196	NA	
ASV_16s_3224	1.470213415	-22.5085071	3.550763562	1.701018833	0.974524525	NA	
ASV_16s_3225	1.647248168	1.581927	3.548286316	2.609060557	NA	NA	
ASV_16s_3226	2.03215193	0.771598429	3.549750148	2.242292229	0.94522254		1
ASV_16s_3227	1.7147048	-0.623328556	3.547047006	2.51769266	0.925758908	NA	
ASV_16s_3228	1.704581668	-0.795908109	3.54796174	2.745045957	0.907543797	NA	
ASV_16s_3229	1.632018483	2.992109362	2.048628595	7.058510498	0.422813323	NA	
ASV_16s_3230	1.902010681	-0.97337615	3.548305304	1.570394966	0.979772042	NA	
ASV_16s_3231	1.053717966	17.92964939	3.551443967	0.234801612	NA	NA	
ASV_16s_3232	1.11580162	-8.686728213	3.549623143	0.776929099	NA	NA	
ASV_16s_3233	1.820769899	1.244090592	3.547907252	1.750845387	0.97233646	NA	
ASV_16s_3234	1.385991705	-0.202823249	3.549111607	3.02603001	NA	NA	
ASV_16s_3235	1.582229949	-0.804948229	3.548327099	2.637003395	0.916432231	NA	
ASV_16s_3236	1.39037305	-0.802186162	3.547921465	2.376153965	NA	NA	
ASV_16s_3237	1.468886656	-0.826666842	3.547743591	2.321543015	0.939925345	NA	
ASV_16s_3238	1.650258688	0.154270582	3.548096278	3.656355885	0.818397677	NA	
ASV_16s_3239	1.788813406	-0.322420799	2.18240426	8.736845086	0.272105729	NA	
ASV_16s_3240	1.977133554	1.183273889	3.549619953	1.607239196	0.978363176	NA	
ASV_16s_3241	0.979237025	2.359527779	3.548637507	0.730350954	NA	NA	
ASV_16s_3242	1.778053285	3.34217483	3.546191997	0.627242705	NA	NA	
ASV_16s_3243	1.745250672	1.061008058	3.547858193	1.775270895	0.971226042	NA	
ASV_16s_3244	1.759372865	0.393716419	3.548633148	1.971057924	0.961424846	NA	
ASV_16s_3245	0.482735561	-0.682791273	3.549657838	0.9792854	NA	NA	
ASV_16s_3246	1.618698799	-0.693826675	3.547946626	2.630557535	0.916949397	NA	
ASV_16s_3247	1.311607335	0.763086195	3.547147285	3.16998163	0.868844839	NA	
ASV_16s_3248	1.516505387	-0.60840776	3.546729408	2.757274634	0.906512032	NA	
ASV_16s_3249	1.491588972	2.044354395	3.545924793	2.544906921	0.923677738	NA	
ASV_16s_3250	1.708860867	0.044090304	3.257733441	4.18734456	0.757963878	NA	
ASV_16s_3251	1.532966002	-0.50117956	2.951785863	2.314822302	0.940384243	NA	
ASV_16s_3252	0.429531587	-22.20453077	3.551442153	-0.244783119	NA	NA	
ASV_16s_3253	1.598520313	0.159233811	3.547759239	2.513609426	0.92606878	NA	
ASV_16s_3254	1.33425058	-1.02230635	3.324911945	2.871470323	0.896632182	NA	
ASV_16s_3255	1.858395527	0.465349949	3.467976372	4.311276071	0.74330724	NA	
ASV_16s_3256	1.903435259	17.34305845	3.551443774	0.632906948	0.99879983	NA	
ASV_16s_3257	0.409926708	-0.064167896	3.550583199	1.995793542	0.960072819	NA	
ASV_16s_3258	0.870006048	-7.323008476	3.552157344	0.198620056	NA	NA	
ASV_16s_3259	1.483666044	-0.417451867	3.54893242	2.70727048	0.910698252	NA	
ASV_16s_3260	1.698772371	-0.330236847	3.071296966	3.716210572	0.811822421	NA	
ASV_16s_3261	1.572148194	-0.845014461	2.688290353	3.849970671	0.796886694	NA	
ASV_16s_3262	1.716921257	-0.868265556	3.003878095	3.412587234	0.844395572	NA	
ASV_16s_3263	1.637880848	-1.872478344	3.239457922	2.405705352	0.934028623	NA	
ASV_16s_3264	1.591537959	-0.782494027	3.54933407	2.33496992	0.939003205	NA	
ASV_16s_3265	0.978733309	-16.02388628	3.552157344	0.270785532	0.999929314	NA	
ASV_16s_3266	1.396747127	-21.31037527	3.550818361	1.71642135	0.973859184	NA	
ASV_16s_3267	1.436179186	-0.012889401	3.547890892	3.534142392	0.831596039	NA	
ASV_16s_3268	1.070662408	0.771574362	3.546819583	1.64623605	0.976811131	NA	
ASV_16s_3269	1.804575931	-0.796288652	3.547881559	2.775115199	0.904997558	NA	
ASV_16s_3270	1.676771368	-1.188222988	2.416851108	4.373551381	0.735885416	NA	
ASV_16s_3271	0.93618397	0.219955078	3.55060733	1.560193587	0.980152292	NA	
ASV_16s_3272	1.015685968	0.067722984	3.549849081	2.339075604	0.938719816	NA	
ASV_16s_3273	0.588860114	0.085239296	3.550592398	2.490416953	0.927816948	NA	
ASV_16s_3274	1.548437018	-0.713833377	3.548030789	2.611555489	NA	NA	
ASV_16s_3275	1.465150627	0.503043588	3.548111284	3.324344229	0.853465495	NA	
ASV_16s_3276	1.54978565	0.160335558	3.546634727	3.170660742	0.868778603	NA	
ASV_16s_3277	1.548038526	-0.102254768	3.07907662	3.329112823	0.85298036	NA	
ASV_16s_3278	1.595494036	-0.6093725	2.757073219	3.129015916	0.872816141	NA	
ASV_16s_3279	1.910515239	0.211209598	3.545553408	3.131164481	0.872609047	NA	
ASV_16s_3280	1.787033806	-0.802502772	3.191434674	2.949805173	0.889606495	NA	
ASV_16s_3281	1.646598839	-0.506632952	3.547399086	2.833096295	0.900000926	NA	
ASV_16s_3282	1.747471842	-0.796325884	3.547878567	2.735479047	0.908347378	NA	
ASV_16s_3283	1.544450487	0.441390699	3.546073669	2.20374172	0.947708423	NA	
ASV_16s_3284	1.909847449	0.381944347	3.55026595	1.058037389	0.993837204	NA	
ASV_16s_3285	1.980420114	0.553378549	3.548287297	3.523356923	0.832745387	NA	
ASV_16s_3286	1.986114097	0.639595785	3.5493757	2.859522569	0.897686253	NA	
ASV_16s_3287	1.533446655	1.214101578	2.626660238	4.972899411	0.663270586	NA	
ASV_16s_3288	1.624347758	0.520608433	3.546742556	3.464778751	0.838941879	NA	
ASV_16s_3289	1.558991064	0.043678582	3.547856008	3.856174355	0.796186365	NA	

ASV_16s_3290	1.647617893	-1.033288188	3.032136537	2.777452051	0.904798377	NA		
ASV_16s_3291	1.373984099	0.777620991	3.320742921	3.016205571	0.883499045	NA		
ASV_16s_3292	0.986812769	-20.83927471	3.550976001	0.500290227	0.999445418	NA		
ASV_16s_3293	0.752937094	0.427914497	3.548121559	2.164709965	NA	NA		
ASV_16s_3294	1.995849003	4.071025188	3.550842644	1.596065864	NA	NA		
ASV_16s_3295	1.435523629	0.472257111	3.547278407	3.261087373	0.859845134	NA		
ASV_16s_3296	1.470889439	0.657116722	3.547205074	3.48935487	0.836351714	NA		
ASV_16s_3297	1.473232366	0.264021813	3.546974304	2.843464193	0.899095563	NA		
ASV_16s_3298	1.752835976	-0.68201544	2.419888368	7.559192704	0.373061667	NA		
ASV_16s_3299	1.411257874	0.340708115	3.546971434	3.276650827	0.858285251	NA		
ASV_16s_3300	1.317104762	0.341288508	3.54661496	2.813699705	0.901685074	NA		
ASV_16s_3301	1.653591842	-0.974740519	3.547404503	2.612748009	0.918370493	NA		
ASV_16s_3302	1.836955212	0.363361201	3.54998797	1.661136279	0.976201522	NA		
ASV_16s_3303	1.423182016	-0.275584273	3.549093548	3.028437884	0.882359085	NA		
ASV_16s_3304	1.36919132	0.294816591	3.548849664	2.923770803	NA	NA		
ASV_16s_3305	1.510534316	-0.324070451	3.546859298	3.188812538	0.867003416	NA		
ASV_16s_3306	1.522755353	0.715496955	3.281649873	3.750821111	0.807988915	NA		
ASV_16s_3307	1.452905595	-0.96614483	3.547840793	2.121909975	0.952785693	NA		
ASV_16s_3308	1.641649407	-0.134734319	2.880846823	3.055546681	0.879816579	NA		
ASV_16s_3309	1.629005828	2.305439423	2.274901911	3.892892551	0.792028208	NA		
ASV_16s_3310	1.422129597	1.613931718	2.879329779	3.363202512	0.849495419	NA		
ASV_16s_3311	1.169531978	-0.255315887	3.549453377	1.325261899	NA	NA		
ASV_16s_3312	1.228523425	-1.449397624	3.547650146	0.723598913	0.99814777	NA		
ASV_16s_3313	1.811878915	0.66875028	3.550596419	2.823753206	0.90081373	NA		
ASV_16s_3315	1.510666	0.114889793	3.547090488	3.016255428	0.883494408	NA		
ASV_16s_3316	1.729151649	-0.798451097	3.184114655	3.343353493	0.851528129	NA		
ASV_16s_3317	1.820883784	-0.866464116	3.546865466	2.836969204	0.899663147	NA		
ASV_16s_3318	1.789717471	0.199224936	2.989144297	2.09239389	0.954549916	NA		
ASV_16s_3319	0.996261757	0.635796717	3.548901549	2.874585042	0.896356621	NA		
ASV_16s_3320	1.85306218	0.341700337	3.550562002	1.542217273	0.980812003	NA		
ASV_16s_3321	1.451082816	0.179080457	3.546938747	3.260910151	0.859862859	NA		
ASV_16s_3322	2.377391922	0.271426559	3.549157753	1.473794458	NA	NA		
ASV_16s_3323	1.833406813	18.16425378	3.550354303	0.973212514	0.995249159	NA		
ASV_16s_3324	1.75936582	0.680897812	3.548092471	2.603194423	0.919128073	NA		
ASV_16s_3325	0.480011947	0.145843894	3.550603946	2.753018415	0.906871725	NA		
ASV_16s_3326	0.848850886	-13.61876446	3.552157344	0.183532384	NA	NA		
ASV_16s_3327	1.471462819	0.086481145	3.547980083	3.583941683	0.826256158	NA		
ASV_16s_3328	1.478150796	-1.29E-04	3.547902244	3.55678016	0.829175313	NA		
ASV_16s_3329	1.449673284	0.567094372	3.546967139	3.407218604	0.844952869	NA		
ASV_16s_3330	1.387841355	2.367037342	3.545202098	2.222851565	0.946483629	NA		
ASV_16s_3331	1.715500844	0.339486813	2.202855059	4.345076823	0.739283283	NA		
ASV_16s_3332	1.530430245	-0.957402031	3.54659381	1.426645501	NA	NA		
ASV_16s_3333	1.642608716	-1.032313038	3.547663836	3.199780799	NA	NA		
ASV_16s_3334	0.882558668	0.449326027	3.549219156	3.270241873	NA	NA		
ASV_16s_3335	1.349062526	-0.324742151	3.549098038	2.930987094	0.891312219	NA		
ASV_16s_3336	1.349357302	-0.051584247	2.388262136	4.226134024	0.753393931	NA		
ASV_16s_3337	2.420757386	0.508050001	3.548930558	3.611405192	0.82328858		1	
ASV_16s_3338	2.025369158	-0.535445843	2.801212599	2.63507026	NA	NA		
ASV_16s_3339	1.555490263	0.299905121	2.453385229	7.006501982	0.428203187	NA		
ASV_16s_3340	1.793833131	3.872441276	3.544902236	1.206136647	NA	NA		
ASV_16s_3341	1.744726958	-3.169691234	3.549943084	1.792179245	0.970442777	NA		
ASV_16s_3342	0.285988912	0.688156771	3.550786696	1.030750114	0.994317125	NA		
ASV_16s_3343	1.590265439	-0.269590033	2.89545292	3.546160046	0.830312354	NA		
ASV_16s_3344	1.215329165	2.168765988	3.150776571	2.779450171	0.90462792	NA		
ASV_16s_3345	1.870241802	-7.111559186	3.550186807	2.830491923	NA	NA		
ASV_16s_3346	1.889537659	0.343889946	3.549494514	2.898135161	0.894262888	NA		
ASV_16s_3347	1.353745455	-0.300533877	3.549101437	2.959356904	0.888736411	NA		
ASV_16s_3348	0.413912256	-22.24556125	3.551442381	-0.267446436	NA	NA		
ASV_16s_3349	0.367143209	-0.170266572	3.5505787	1.109959706	NA	NA		
ASV_16s_3350	0.367143209	-0.170266572	3.5505787	1.109959706	NA	NA		
ASV_16s_3351	0.436434758	-6.798686372	3.552157344	-0.223826399	NA	NA		
ASV_16s_3352	1.469829692	0.495112852	3.547476145	3.530627722	0.831970862	NA		
ASV_16s_3353	1.206051376	10.7371869	3.546994416	-0.398002161	1	NA		
ASV_16s_3354	1.68664967	-0.796256636	3.547895829	2.703986574	0.910970122	NA		
ASV_16s_3355	1.639266232	-0.939707488	3.216359712	2.859418452	0.897695417	NA		
ASV_16s_3356	2.067975375	0.502117125	3.549123429	3.094605853	NA	NA		
ASV_16s_3357	1.838561013	-0.920680359	3.548590539	1.474235013	0.983188261	NA		

ASV_16s_3358	0.973804447	-0.664481281	3.549922466	1.997595381	0.959973339	NA	
ASV_16s_3359	1.29252375	-0.898079852	3.546905193	1.824887634	0.968893687	NA	
ASV_16s_3360	1.16995655	2.40671607	3.547255524	0.33345042	0.999857033	NA	
ASV_16s_3361	1.839382529	0.087618204	3.550588256	3.088479616	0.876698232	NA	
ASV_16s_3362	1.828336889	-0.116689935	3.549805763	2.523310919	0.925331524	NA	
ASV_16s_3363	2.087154354	1.042123854	3.482911043	2.545870199	0.923603567		1
ASV_16s_3364	1.698757708	0.363427182	3.5498011	1.582170511	0.97932782	NA	
ASV_16s_3365	1.311824151	-0.29175387	3.549111654	2.935938814	NA	NA	
ASV_16s_3366	0.549112301	0.21997162	3.550629179	1.288780042	0.9887371	NA	
ASV_16s_3367	1.324541778	0.016715255	3.548118564	3.276932892	NA	NA	
ASV_16s_3368	1.558833085	0.688344457	2.878482601	5.214790594	0.633769579	NA	
ASV_16s_3369	1.414054167	-0.79798748	3.547501569	2.094286547	NA	NA	
ASV_16s_3370	1.632656083	-0.794930265	3.548168048	2.720540642	0.909595789	NA	
ASV_16s_3371	1.581302764	-1.000477555	3.547233441	2.236071078	0.94562775	NA	
ASV_16s_3372	0.909042854	0.219955633	3.550608256	1.545204701	0.980703281	NA	
ASV_16s_3373	1.432248907	-0.45840019	3.54802919	2.558237505	0.922648252	NA	
ASV_16s_3374	1.410999815	0.949612086	3.546775865	3.010148808	0.884061798	NA	
ASV_16s_3375	1.080214552	1.604049925	3.546990315	2.624973971	NA	NA	
ASV_16s_3376	1.314543348	-3.020733125	3.545829699	0.946046817	0.995652563	NA	
ASV_16s_3377	1.64300487	-0.439451134	2.116578687	5.525791344	0.596074796	NA	
ASV_16s_3378	1.333833542	-0.895582621	3.54831869	1.708047223	0.974222144	NA	
ASV_16s_3379	1.75182668	0.449170985	3.146071627	4.720032079	0.694085068	NA	
ASV_16s_3380	0.247035629	-0.170122965	3.550613998	0.909875743	NA	NA	
ASV_16s_3381	0.219730307	-0.544180806	3.550190256	0.63271416	NA	NA	
ASV_16s_3382	1.413825131	-1.151431029	3.547523449	1.57507667	NA	NA	
ASV_16s_3383	1.135146811	0.064178767	3.136344841	1.603885588	0.978493719	NA	
ASV_16s_3384	1.306150525	2.223289575	3.547320635	1.329540043	NA	NA	
ASV_16s_3385	1.285524045	0.083418147	3.548781111	3.020457289	0.883103334	NA	
ASV_16s_3386	0.911632514	0.219955718	3.550608166	1.546651073	0.980650512	NA	
ASV_16s_3387	0.830340119	-6.805358328	3.552157344	0.170000557	NA	NA	
ASV_16s_3388	0.86471388	17.83830706	3.551445285	0.113653061	0.999996402	NA	
ASV_16s_3389	1.403709462	0.031103726	3.547888622	3.601182829	0.824395015	NA	
ASV_16s_3390	1.847606422	2.79807261	3.546012539	0.668068535	0.998569234	NA	
ASV_16s_3391	1.654407211	-0.796170897	3.547918194	2.695139303	0.911700687	NA	
ASV_16s_3392	1.490106994	-1.012875379	3.099323448	1.948259829	0.962648429	NA	
ASV_16s_3393	1.69677615	0.482924791	3.549263097	2.635667021	0.916539574	NA	
ASV_16s_3394	1.356759731	-0.069781522	3.549344396	3.045827462	0.880730684	NA	
ASV_16s_3395	1.940260192	0.271435889	3.54921471	1.408233424	NA	NA	
ASV_16s_3396	1.71715382	0.399080262	3.550626371	3.645766088	0.819553619	NA	
ASV_16s_3397	1.732413627	0.606551566	3.550606783	3.554577626	0.829411329	NA	
ASV_16s_3398	1.965534671	17.11528436	3.551444612	0.658145362	NA	NA	
ASV_16s_3399	1.294993531	-0.295112364	3.549114557	2.920357882	NA	NA	
ASV_16s_3401	1.49429292	0.20860517	3.279020129	3.525155184	0.832553936	NA	
ASV_16s_3402	1.383207164	0.014510873	3.547704652	2.557850218	0.922678254	NA	
ASV_16s_3403	1.729303046	-0.796547562	3.547840117	2.680268001	0.912922467	NA	
ASV_16s_3404	1.517315583	0.182594696	3.547939892	3.83434819	0.798647422	NA	
ASV_16s_3405	1.512293273	0.740225571	3.547142161	3.381881088	0.847573581	NA	
ASV_16s_3406	1.385288514	0.257901245	3.352730537	3.009869243	NA	NA	
ASV_16s_3407	2.058721991	3.107420493	3.546953594	0.759591956	NA	NA	
ASV_16s_3408	0.914635161	0.219955561	3.550608061	1.548323111	0.980589403	NA	
ASV_16s_3409	1.298248018	1.037000389	3.213697208	1.706549511	0.974286752	NA	
ASV_16s_3410	0.537777219	0.012465731	3.549746469	2.155189218	NA	NA	
ASV_16s_3411	1.90126451	0.687839837	3.549578671	2.460203801	0.930063827	NA	
ASV_16s_3412	1.59566695	-0.796368484	3.547883592	2.613325303	0.918324609	NA	
ASV_16s_3413	1.670225929	-0.7984105	3.192649216	3.265901248	0.859363339	NA	
ASV_16s_3414	1.704746698	-0.307263992	3.547784071	2.830039098	0.900267208	NA	
ASV_16s_3415	1.608640089	-0.852020621	3.54795093	2.673692215	0.913460217	NA	
ASV_16s_3416	1.581511795	-0.832994882	3.547846501	2.095736443	0.954351922	NA	
ASV_16s_3417	1.649433222	-0.072566631	2.731690978	5.298732135	0.623558099	NA	
ASV_16s_3418	1.795700288	0.405494545	3.544496929	2.499857318	NA	NA	
ASV_16s_3419	1.420987163	0.114849814	2.922385164	4.420205308	0.730303373	NA	
ASV_16s_3420	0.896437812	0.21995598	3.550608705	1.538091069	0.980961571	NA	
ASV_16s_3421	0.987267283	17.92450184	3.551444362	0.194873219	NA	NA	
ASV_16s_3422	2.018665053	3.484499123	3.544225857	1.026251672	NA	NA	
ASV_16s_3423	1.624253156	0.309378143	3.549845743	1.570516364	0.979767491	NA	
ASV_16s_3424	2.178095376	0.78662167	3.549325535	4.13797878	0.763754913		1
ASV_16s_3425	2.050329905	9.591928391	3.55028683	1.583571561	0.979274588		1

ASV_16s_3426	2.299189532	0.731903243	3.510608701	3.652976408	0.818766813		1
ASV_16s_3427	1.40723576	-0.337683084	3.549084898	2.956829774	0.888966891	NA	
ASV_16s_3428	1.524080252	0.399375295	3.546867726	2.981926336	0.886669142	NA	
ASV_16s_3429	1.627397196	-1.734026095	2.845254365	1.753393392	0.972221787	NA	
ASV_16s_3430	1.484339937	1.186993107	3.286724645	3.311418171	0.854777598	NA	
ASV_16s_3431	0.959279081	0.478970653	3.550597855	2.867825854	0.896954206	NA	
ASV_16s_3432	1.436691512	-1.150585386	3.54754171	1.800655626	NA	NA	
ASV_16s_3433	0.403499369	-22.06885963	3.551442496	-0.283032243	NA	NA	
ASV_16s_3434	1.339055669	0.563231695	3.548244683	3.062374348	0.879172735	NA	
ASV_16s_3435	1.409107681	-0.562987881	3.548102303	2.594146233	NA	NA	
ASV_16s_3436	1.296150468	-2.53859157	3.338655295	1.621289723	0.977811206	NA	
ASV_16s_3437	1.602153004	-0.774668552	2.284529147	5.600391892	0.587103948	NA	
ASV_16s_3438	1.664411053	-0.929277684	2.853539593	3.425748092	0.843026455	NA	
ASV_16s_3439	1.622053529	2.637383671	3.138184352	2.31204414	0.940573418	NA	
ASV_16s_3440	1.388066746	0.827695167	2.931781209	4.173639621	0.759574455	NA	
ASV_16s_3441	1.520391993	-0.246204194	3.546587007	2.795609284	0.903244418	NA	
ASV_16s_3442	1.456654252	-2.664068768	3.545805583	0.608808703	NA	NA	
ASV_16s_3443	1.413993339	-0.069702231	3.549311583	3.052878094	0.880067846	NA	
ASV_16s_3444	1.798052402	-0.556207783	3.548883652	2.445616189	0.931136244	NA	
ASV_16s_3445	1.82228998	1.005253729	3.548846744	2.264953869	0.94373337	NA	
ASV_16s_3446	2.002668023	1.703486053	3.550755055	3.472109616	0.838170714		1
ASV_16s_3447	0.356752364	-0.170258858	3.550580816	1.095402016	NA	NA	
ASV_16s_3448	1.280824497	0.493341282	3.548744454	3.092937588	NA	NA	
ASV_16s_3449	1.59782781	-0.79819121	3.202092579	3.21070676	0.864850033	NA	
ASV_16s_3450	1.457847221	-0.801094308	3.247886637	2.60677867	0.918844239	NA	
ASV_16s_3451	1.435361358	-1.367535256	3.547471887	2.534233827	0.924497238	NA	
ASV_16s_3452	1.434030583	-0.17752708	3.549123043	2.415900851	0.933295575	NA	
ASV_16s_3453	1.475165561	-0.321345638	3.5478877	1.992685671	0.960244089	NA	
ASV_16s_3454	1.691108459	3.166835503	2.562313238	1.985728202	0.96062605	NA	
ASV_16s_3455	1.908856773	-0.053158222	3.54669952	3.714825832	0.811975326	NA	
ASV_16s_3456	1.823888214	0.699026206	3.549567213	1.794065666	0.970354649	NA	
ASV_16s_3457	1.734224534	0.918329025	3.420531919	3.773494226	0.805465556	NA	
ASV_16s_3458	1.416959048	1.830904628	3.54628567	2.735003276	0.908387259	NA	
ASV_16s_3459	1.558843329	-0.021756364	3.548416933	3.912406999	0.789809381	NA	
ASV_16s_3460	1.546651046	2.802156462	2.654027629	2.812214174	0.901813538	NA	
ASV_16s_3461	1.217976622	-0.557135743	3.545919298	2.484404494	NA	NA	
ASV_16s_3462	1.252443823	0.271463666	3.549384533	1.26699971	NA	NA	
ASV_16s_3463	0.282431393	-0.170175493	3.550600482	0.977227834	0.995187566	NA	
ASV_16s_3464	1.346658771	-3.75E-04	3.548028124	3.381402043	0.847622978	NA	
ASV_16s_3465	1.374923564	1.246443919	3.547714684	2.906698417	0.893497106	NA	
ASV_16s_3466	1.183076622	-1.01868185	3.547456788	2.043995384	0.957365205	NA	
ASV_16s_3467	1.452738144	2.761842573	2.905858799	1.653109225	0.976531073	NA	
ASV_16s_3468	1.182936661	-0.069405424	3.550742994	1.839774898	0.968173811	NA	
ASV_16s_3469	1.717502259	0.355509805	3.54994836	2.48351951	0.928332941	NA	
ASV_16s_3470	0.415149389	0.126260146	3.550619956	1.953115762	NA	NA	
ASV_16s_3471	1.339622343	0.806814375	3.547948828	2.929459627	0.891450179	NA	
ASV_16s_3472	1.286646745	0.878982246	3.547910128	3.047093294	NA	NA	
ASV_16s_3473	1.285223709	0.104803195	3.547946485	3.522926856	NA	NA	
ASV_16s_3474	1.396689943	1.448193045	3.546785533	3.114389595	0.874222393	NA	
ASV_16s_3475	1.640282871	-0.798148943	3.196345751	3.280112456	0.857937429	NA	
ASV_16s_3476	1.637504452	-0.79621815	3.547906656	2.674656208	0.91338148	NA	
ASV_16s_3477	1.621779625	-0.341255208	3.229861075	4.321490029	0.742092392	NA	
ASV_16s_3478	1.6410893	-0.795991742	3.547949148	2.701302844	0.911192022	NA	
ASV_16s_3479	1.273775755	0.049880323	3.339555015	2.039924891	0.957597574	NA	
ASV_16s_3480	1.743009582	-0.011914223	3.546373442	3.307179791	0.855206891	NA	
ASV_16s_3481	1.65436261	0.080232198	3.186962273	5.351204495	0.61718669	NA	
ASV_16s_3482	0.953713417	-6.94650227	3.550539743	0.257029042	NA	NA	
ASV_16s_3483	1.551975905	14.29879272	3.551469819	1.971683052	0.96139099	NA	
ASV_16s_3484	1.327539221	0.633552138	3.547153861	2.056076119	NA	NA	
ASV_16s_3485	1.693580546	-0.796707723	3.547817986	2.57138629	0.92162637	NA	
ASV_16s_3486	1.588959072	-0.783321247	3.26088353	2.149416971	0.951109441	NA	
ASV_16s_3487	1.523218116	-0.878603358	2.926860912	3.496697141	0.835575194	NA	
ASV_16s_3488	1.092460014	-0.985947466	3.343125442	1.679408309	0.975441407	NA	
ASV_16s_3489	1.529406543	0.066043197	3.276271043	2.862868991	0.897391493	NA	
ASV_16s_3490	1.503155384	1.173710836	3.547146293	2.052576533	NA	NA	
ASV_16s_3491	0.455588513	0.038732607	3.550594913	2.26985346	0.943408702	NA	
ASV_16s_3492	1.60312401	0.176498068	2.469174852	6.257154107	0.510064266	NA	

ASV_16s_3493	1.362241833	0.043183005	3.547030229	3.045442299	0.88076685	NA	
ASV_16s_3494	1.416706829	1.818533212	2.749227432	2.328340621	0.939459384	NA	
ASV_16s_3495	1.646671593	-0.246577275	2.823560401	3.145874227	0.871187652	NA	
ASV_16s_3496	0.371031148	0.294548236	3.551011303	0.909395094	NA	NA	
ASV_16s_3497	1.252165893	-0.30745413	3.549121735	2.875805328	NA	NA	
ASV_16s_3498	1.473942106	-0.489477812	3.548046335	2.613868117	0.918281454	NA	
ASV_16s_3499	1.446764348	-0.338046456	3.547231543	3.006319529	0.884417008	NA	
ASV_16s_3500	1.680959031	-0.367465024	3.547138452	2.872644136	0.896528372	NA	
ASV_16s_3501	1.333845998	2.468270654	3.114755478	3.086779012	0.876860046	NA	
ASV_16s_3502	1.60115168	0.034844282	3.548848691	3.53495495	0.831509345	NA	
ASV_16s_3503	1.434416363	-0.75072275	3.547274174	2.044060654	0.957361474	NA	
ASV_16s_3504	1.217368283	-0.54960038	3.122559601	2.100206758	0.954086408	NA	
ASV_16s_3505	1.802378662	0.178351639	3.550506553	2.252872778	0.944529817	NA	
ASV_16s_3506	0.440441509	-0.68239213	3.549746066	0.94974352	NA	NA	
ASV_16s_3507	0.798607375	-13.31951719	3.552157344	0.146091975	NA	NA	
ASV_16s_3508	1.311168385	0.826371521	3.546939682	3.273309111	NA	NA	
ASV_16s_3509	1.465463067	-1.501626068	3.546933285	1.681440286	0.975356021	NA	
ASV_16s_3510	1.45785696	-0.771623101	3.547135219	2.130802242	0.952247189	NA	
ASV_16s_3511	1.367638531	-2.144071964	3.544387574	1.617624134	NA	NA	
ASV_16s_3512	1.073816321	0.262697249	3.545265528	2.005078916	NA	NA	
ASV_16s_3513	1.746123136	1.212356809	3.548828893	1.982377611	0.960809278	NA	
ASV_16s_3514	0.936429609	16.60246281	3.551452144	0.203299281	NA	NA	
ASV_16s_3515	0.440475474	-0.020561794	3.550585858	2.194366553	NA	NA	
ASV_16s_3517	1.308973359	0.208673069	3.54858256	3.325631448	NA	NA	
ASV_16s_3518	1.356119339	0.104107411	3.548309278	3.283237508	0.857623155	NA	
ASV_16s_3519	1.610722732	-0.796728559	3.547838322	2.462501447	0.929894174	NA	
ASV_16s_3520	1.461554957	3.290435934	3.544740037	0.49772238	0.999454778	NA	
ASV_16s_3521	1.529901163	0.5501011	2.906953423	4.383492788	0.734697458	NA	
ASV_16s_3522	1.332199274	-0.479834203	3.546993227	2.310789741	0.940658734	NA	
ASV_16s_3523	1.60938446	0.389343753	3.54913234	1.919568645	0.964157521	NA	
ASV_16s_3524	1.591805497	0.087887089	3.54663175	3.360648291	0.849757547	NA	
ASV_16s_3525	0.860891288	0.219957095	3.550610043	1.517482827	0.981698226	NA	
ASV_16s_3526	0.536721909	-0.717960863	3.549911824	1.412323228	NA	NA	
ASV_16s_3527	1.827499279	-0.213825182	3.546653778	2.360769643	0.937211429	NA	
ASV_16s_3528	1.698908036	-0.091301663	3.549486436	1.520241617	0.98160061	NA	
ASV_16s_3529	0.933328848	16.57258688	3.551452217	0.201260249	NA	NA	
ASV_16s_3530	1.29129765	0.052810242	3.548124476	3.293307174	0.856608745	NA	
ASV_16s_3531	1.593978435	-0.796378381	3.547876289	2.599850247	0.919392474	NA	
ASV_16s_3532	1.482248312	-0.251189361	3.357426856	2.942030473	0.890312578	NA	
ASV_16s_3533	0.377585657	0.278442342	3.551234431	0.808353145	NA	NA	
ASV_16s_3534	1.303906151	-0.415508176	2.814113062	2.488609872	0.927952308	NA	
ASV_16s_3535	1.491911287	2.508535429	3.547500332	1.762523566	NA	NA	
ASV_16s_3536	1.756067556	0.793044873	3.548111872	2.606707232	0.9188499	NA	
ASV_16s_3537	1.284504529	-0.24440197	3.54912535	2.943542982	0.890175365	NA	
ASV_16s_3539	1.32486717	0.062173737	3.548245244	3.235672352	0.862378498	NA	
ASV_16s_3540	1.30551334	-0.068108328	3.548397446	2.998081163	0.885179683	NA	
ASV_16s_3541	1.360961239	0.561558446	3.54778285	3.357306105	0.850100295	NA	
ASV_16s_3542	1.663034106	-0.46844692	3.102825517	3.720409664	0.811358529	NA	
ASV_16s_3543	1.676100412	-0.949501502	3.052646291	1.907533709	0.964780309	NA	
ASV_16s_3544	0.733387368	0.323256919	3.550183582	1.135734679	NA	NA	
ASV_16s_3545	0.3905591	-0.606539475	3.550289071	0.900309576	NA	NA	
ASV_16s_3546	1.586734602	0.984302747	3.550275372	2.126548311	0.952505205	NA	
ASV_16s_3547	1.75006364	0.698348723	3.548885418	2.082974256	0.95510541	NA	
ASV_16s_3548	0.927127328	16.60000709	3.551452304	0.197172771	NA	NA	
ASV_16s_3551	1.346511255	0.200274277	3.548377267	2.827456658	0.900491896	NA	
ASV_16s_3552	1.353497018	-0.167772263	3.548180453	3.017921655	0.883339394	NA	
ASV_16s_3553	1.411103926	0.54737307	3.546311694	2.588428847	0.920292408	NA	
ASV_16s_3554	1.38859063	0.633194819	3.547281525	3.460221413	0.839420655	NA	
ASV_16s_3555	1.509938133	0.376249803	3.036881697	4.857648917	0.677330022	NA	
ASV_16s_3556	1.426369997	0.069944868	3.352879569	2.644375471	0.915838918	NA	
ASV_16s_3557	1.771148668	-0.054310179	2.680977395	4.855571895	0.677583239	NA	
ASV_16s_3558	2.139203598	1.615008753	3.548628564	2.266503888	0.943630763		1
ASV_16s_3559	2.007006785	0.794429662	3.54810457	2.243135217	0.945167513		1
ASV_16s_3560	1.313820373	-0.499041886	3.547556375	2.833012437	0.900008234	NA	
ASV_16s_3561	1.421033604	0.593922624	3.547646079	3.554446637	0.829425362	NA	
ASV_16s_3562	1.529224738	0.016661785	3.546803893	3.316222545	0.854290416	NA	
ASV_16s_3563	1.427277985	0.832052946	3.078951667	4.180365675	0.758784293	NA	

ASV_16s_3564	1.585613661	-0.795678233	3.548015387	2.686044032	0.912448855	NA
ASV_16s_3565	1.6415265	1.86194214	2.619680665	4.536606655	0.716304849	NA
ASV_16s_3566	1.519345086	-1.769899091	3.442570596	1.693896291	0.97482886	NA
ASV_16s_3567	1.367988444	-1.237672673	3.547876779	2.173253072	0.949631931	NA
ASV_16s_3568	1.793519428	1.212975	2.958758643	2.433817922	0.931997649	NA
ASV_16s_3569	1.345531953	0.438320152	3.549928312	0.813481779	NA	NA
ASV_16s_3570	1.388773467	-2.465925333	3.546089293	0.62163305	NA	NA
ASV_16s_3571	1.332609018	-0.053920099	3.548017018	3.327896709	0.853104138	NA
ASV_16s_3572	1.281755389	-1.39903987	3.306483881	1.616940153	0.977982946	NA
ASV_16s_3573	1.723421781	0.643687836	3.489185933	3.465744034	0.838840408	NA
ASV_16s_3574	1.333871335	17.2274488	3.551445747	0.410209286	NA	NA
ASV_16s_3575	1.613901279	17.2387034	3.551444857	0.531618295	0.999322275	NA
ASV_16s_3576	1.215056616	-0.358649449	3.549122683	2.782520514	NA	NA
ASV_16s_3577	0.266894269	-0.17015492	3.550605975	0.948715668	NA	NA
ASV_16s_3578	1.469673564	-0.901883855	2.415506638	4.604455936	0.708105248	NA
ASV_16s_3579	1.494288733	-0.793613215	3.548453084	2.630167233	0.916980664	NA
ASV_16s_3580	1.850478879	0.519598067	2.866612769	5.692417247	0.576087449	NA
ASV_16s_3581	0.782795009	0.702026183	3.548372168	2.287016997	NA	NA
ASV_16s_3582	1.107336148	1.016936782	3.547669255	1.778078496	0.971096809	NA
ASV_16s_3583	1.470228485	0.648069713	3.45556872	1.677379663	NA	NA
ASV_16s_3584	1.688745027	0.512522334	3.348540406	3.145151604	0.871257624	NA
ASV_16s_3585	2.170248741	-0.028452271	2.946043129	4.989105222	0.661292842	1
ASV_16s_3586	1.909699953	0.773880677	3.549282668	3.082821901	0.877236237	NA
ASV_16s_3587	1.727552001	1.16130802	3.550032571	1.639296043	0.977091931	NA
ASV_16s_3588	1.714891873	0.694035272	3.550595572	1.967095132	0.961639084	NA
ASV_16s_3589	1.33399054	-0.301018053	3.5491053	2.944315879	0.890105221	NA
ASV_16s_3590	0.382673596	-0.170278604	3.550575753	1.130937177	NA	NA
ASV_16s_3591	1.281132482	0.137627365	3.548457246	3.164172646	0.869410874	NA
ASV_16s_3592	1.313338664	0.038160558	3.548102326	3.171816954	0.868665805	NA
ASV_16s_3593	1.354267172	-0.405715744	3.548212708	2.542949209	0.923828372	NA
ASV_16s_3594	1.306164177	-0.306771037	3.547917591	3.335245674	0.852355578	NA
ASV_16s_3595	1.405427267	-0.449248677	3.547129459	3.257676083	0.860186177	NA
ASV_16s_3596	1.515020306	-1.257329826	2.720884016	3.131390352	0.872587268	NA
ASV_16s_3597	1.611245554	-0.796240902	3.547904177	2.651213787	0.915286819	NA
ASV_16s_3598	1.210922703	2.277474995	3.326615114	2.863368492	0.897347465	NA
ASV_16s_3599	1.755985852	-1.177597212	3.545321307	2.859165204	0.897717708	NA
ASV_16s_3600	1.537303883	-0.86186217	3.547505168	2.260645829	0.944018048	NA
ASV_16s_3601	1.512264658	-0.893440584	2.836932003	3.704475723	0.813117036	NA
ASV_16s_3602	1.552174424	-0.833640137	2.534652485	5.679036029	0.577685679	NA
ASV_16s_3603	1.532613819	2.407200484	3.547367223	1.138130421	0.992282377	NA
ASV_16s_3604	1.425026491	0.299779455	2.372044066	5.444424599	0.605895507	NA
ASV_16s_3605	2.077502279	0.419408244	3.549153081	1.363542892	NA	NA
ASV_16s_3606	1.733897578	3.437953008	3.550929343	1.629002351	NA	NA
ASV_16s_3607	1.677861581	0.282233018	3.550556243	2.139466006	0.951719413	NA
ASV_16s_3608	1.226140463	-0.149042322	3.5491582	2.889896519	NA	NA
ASV_16s_3609	1.210985269	-0.266030242	3.549139446	2.875036944	NA	NA
ASV_16s_3610	0.586359205	0.197887979	3.550599714	3.153581552	0.87044043	NA
ASV_16s_3611	1.271767712	-0.404561879	3.549115462	2.761375123	0.90616491	NA
ASV_16s_3612	1.483355145	-0.795696861	3.548018456	2.618186896	0.91793772	NA
ASV_16s_3613	1.520264026	1.393936363	2.465009036	5.520074778	0.596763589	NA
ASV_16s_3614	2.072878471	-0.728202026	3.386081059	3.30040179	0.855892446	1
ASV_16s_3615	1.883224575	1.208701389	3.547103176	2.561419112	NA	NA
ASV_16s_3616	1.520681769	-0.210319723	2.783041779	3.723137016	0.811057047	NA
ASV_16s_3617	1.378968693	0.385950607	2.59023908	4.809483138	0.683199866	NA
ASV_16s_3618	1.887074664	2.403018152	2.640345243	3.455539365	0.839912027	NA
ASV_16s_3619	1.225979445	0.720446204	3.547994414	3.074577187	NA	NA
ASV_16s_3620	1.355572776	-0.386941625	3.547225717	2.799496245	0.902910304	NA
ASV_16s_3621	1.329685543	-0.41831511	3.548281236	2.538735388	0.924152116	NA
ASV_16s_3622	1.221417076	-0.341342601	3.547900964	2.107966687	NA	NA
ASV_16s_3623	1.470422423	0.525327294	3.547006815	3.396422007	0.846071502	NA
ASV_16s_3624	1.42543144	-0.944277951	3.547131235	2.102348576	0.953958904	NA
ASV_16s_3625	1.532837671	-0.795313647	3.54809234	2.665890646	0.914096216	NA
ASV_16s_3626	1.612206915	-0.916037907	3.020891571	2.701672728	0.911161454	NA
ASV_16s_3627	1.408697526	0.219682484	3.548028647	3.555055801	NA	NA
ASV_16s_3628	1.239329503	0.057818195	3.549924887	1.523517331	0.981484304	NA
ASV_16s_3629	1.603705779	0.743525119	3.549197085	1.006607257	NA	NA
ASV_16s_3630	1.028173375	0.273965025	3.549103135	2.16373794	0.950224511	NA

ASV_16s_3631	1.447009037	-2.507834589	3.545982732	0.623351188	0.998857882	NA
ASV_16s_3632	0.850204131	0.469176372	3.550627139	1.592566758	0.978930905	NA
ASV_16s_3633	1.114525261	1.501852751	3.547224011	1.591014999	NA	NA
ASV_16s_3634	0.492372174	0.227307866	3.551113292	0.939695474	NA	NA
ASV_16s_3635	0.862288405	-0.002097968	3.549155726	2.070699385	0.955823803	NA
ASV_16s_3636	1.68503804	0.694034112	3.55059634	1.95814107	0.962120756	NA
ASV_16s_3637	0.424398937	-0.682226793	3.549784093	0.937792881	NA	NA
ASV_16s_3638	1.395616654	1.775580413	3.292785994	3.291384161	0.856802673	NA
ASV_16s_3639	1.597336058	-0.935651635	3.248128475	3.653556487	0.818703468	NA
ASV_16s_3640	1.490111413	0.24324299	3.548540214	2.747201759	NA	NA
ASV_16s_3641	1.391876969	-0.071721546	3.549481938	1.980256292	0.960925042	NA
ASV_16s_3642	1.109222795	1.267108047	3.547176034	1.972557761	NA	NA
ASV_16s_3643	1.064682403	-9.540079557	3.548371375	0.54254655	NA	NA
ASV_16s_3644	1.598203587	0.34249893	3.550629026	1.453721568	0.983868713	NA
ASV_16s_3645	1.216315193	-0.317198804	3.549128502	2.837079304	NA	NA
ASV_16s_3646	1.28716662	-0.356567384	3.54910622	2.842510694	0.899178975	NA
ASV_16s_3647	1.406997989	-0.201250472	3.54790284	3.606557044	0.8238136	NA
ASV_16s_3648	1.487763338	-0.796245056	3.54791811	2.545861645	0.923604226	NA
ASV_16s_3649	1.533348315	-0.667283663	2.271889275	5.07530207	0.650774112	NA
ASV_16s_3650	1.211817608	1.166595513	3.5488768	1.443804269	0.984191645	NA
ASV_16s_3651	1.613700732	-0.498679365	3.527917184	4.184938087	0.758246838	NA
ASV_16s_3652	1.635871449	0.403710236	3.549293138	1.356020917	NA	NA
ASV_16s_3653	1.620407093	1.043650955	3.549347605	1.66207715	0.97616272	NA
ASV_16s_3654	1.19783085	-0.328729664	3.549131186	2.808872027	NA	NA
ASV_16s_3655	1.221674061	0.272193884	3.548846853	2.856370092	0.897963591	NA
ASV_16s_3656	1.322657257	-4.28E-04	3.547956113	3.403995199	0.845287142	NA
ASV_16s_3657	1.301171169	1.117454036	3.54667427	2.948533746	0.889722094	NA
ASV_16s_3658	1.459226134	-0.178830219	3.136528996	2.843846958	0.89906207	NA
ASV_16s_3659	1.562338918	-0.499571896	3.549363942	2.244072027	0.945106328	NA
ASV_16s_3660	1.401683869	-0.312969376	3.54825004	3.077254472	0.877764733	NA
ASV_16s_3661	1.456610631	-0.568252483	3.548959337	1.312639413	NA	NA
ASV_16s_3662	0.835427166	0.222414842	3.550625797	1.366902195	0.986563519	NA
ASV_16s_3663	1.399992595	0.929971869	3.544662436	1.711661192	NA	NA
ASV_16s_3664	1.563891438	0.885800336	3.548891129	2.011947983	0.959176114	NA
ASV_16s_3665	0.42002369	-0.682178275	3.549794964	0.93445531	NA	NA
ASV_16s_3666	0.260561185	-0.170145794	3.550608403	0.936627387	NA	NA
ASV_16s_3667	1.452978697	-0.088821457	3.547068066	3.165945398	0.869238236	NA
ASV_16s_3668	1.671056703	0.086627017	3.546729785	3.503900638	0.834812159	NA
ASV_16s_3669	1.458250094	-0.805339393	3.049298527	2.840794796	0.899329006	NA
ASV_16s_3670	1.329639455	1.511773953	3.546847494	1.782169646	0.970907905	NA
ASV_16s_3671	1.085787023	-0.251312489	3.548858768	2.528600467	0.924928067	NA
ASV_16s_3672	1.353954477	0.617085605	3.549032443	2.757151728	NA	NA
ASV_16s_3673	1.569560694	0.352790224	3.550505688	1.337140426	0.987419286	NA
ASV_16s_3674	1.215636821	-0.317049648	3.549128696	2.836670045	0.899689257	NA
ASV_16s_3675	1.373982393	0.298936246	3.546942091	3.282766401	0.857670549	NA
ASV_16s_3676	1.513092432	-0.79601866	3.547954157	2.608390814	0.918716421	NA
ASV_16s_3677	1.783273361	0.490094945	3.549322855	2.692486952	0.911919168	NA
ASV_16s_3678	1.610769392	0.335864815	3.550630912	2.766239061	0.905752411	NA
ASV_16s_3679	1.214210049	0.102879809	3.548275357	3.20311246	0.865598463	NA
ASV_16s_3680	1.282258408	1.185679901	3.5468759	2.96134447	0.888554999	NA
ASV_16s_3681	0.84904653	-0.566942503	3.550204169	1.846165414	0.967861954	NA
ASV_16s_3682	1.394855863	-0.750762914	3.547260815	2.591158584	0.920077756	NA
ASV_16s_3683	1.43591478	-0.059233996	3.542469604	1.220062345	NA	NA
ASV_16s_3684	0.391144358	0.219987951	3.550650506	1.116488881	NA	NA
ASV_16s_3685	1.287558475	-0.865593458	3.547571787	2.122863407	0.952728109	NA
ASV_16s_3686	1.232549656	-1.05049527	3.546334799	2.470006276	NA	NA
ASV_16s_3687	1.524494578	-0.60339056	2.882956948	4.729524649	0.692931127	NA
ASV_16s_3688	1.072083228	-1.630246383	3.548174493	1.914650676	0.964412748	NA
ASV_16s_3689	1.288264346	2.404366984	2.470188475	4.312899818	0.743114177	NA
ASV_16s_3690	2.022513705	3.294920448	3.546752391	0.737419946	NA	NA
ASV_16s_3691	2.333769936	-0.759781108	3.546194484	2.634368655	NA	NA
ASV_16s_3692	1.239116235	0.789711689	3.549281398	1.567724759	NA	NA
ASV_16s_3693	1.308745048	-1.593694729	3.547236017	1.40767726	0.985334956	NA
ASV_16s_3694	1.504511774	0.215929092	3.549390774	4.436350079	0.728367624	NA
ASV_16s_3695	1.604249875	17.23209723	3.551444891	0.527928875	0.999337655	NA
ASV_16s_3696	0.73358026	-13.57275479	3.552157344	0.094052453	0.999998131	NA
ASV_16s_3698	1.262964626	0.044467078	3.548146582	3.283505311	0.857596211	NA

ASV_16s_3699	1.241751907	0.121887617	3.547945761	3.539233799	NA	NA
ASV_16s_3700	0.957720363	2.399263144	3.372738542	1.436174113	NA	NA
ASV_16s_3701	1.900333039	0.419397681	3.549175965	1.334774926	NA	NA
ASV_16s_3702	1.71669569	6.54886939	3.55025371	1.143249938	0.992175405	NA
ASV_16s_3703	0.412731612	-0.682091125	3.549813587	0.928814529	NA	NA
ASV_16s_3704	1.267592717	0.590396476	3.548066934	2.999630477	0.885036413	NA
ASV_16s_3705	1.29575731	0.06186484	3.547931652	3.521749396	0.83291647	NA
ASV_16s_3706	1.529351291	1.774652845	2.873400427	3.304502594	0.855477816	NA
ASV_16s_3707	1.639720902	3.165486156	3.546769671	1.21024121	0.990689181	NA
ASV_16s_3708	1.46990417	-1.748976381	2.610443557	1.492624605	0.982563897	NA
ASV_16s_3709	1.564050287	0.372005789	3.549325695	2.772911842	0.905185189	NA
ASV_16s_3710	1.300739027	0.441804956	3.55021562	0.738738308	NA	NA
ASV_16s_3711	1.005542767	1.502508774	3.548325655	1.103491332	0.992981988	NA
ASV_16s_3712	1.191794131	-0.069811226	3.549476991	2.787920687	0.903903802	NA
ASV_16s_3713	1.491070027	0.139195989	2.896118319	4.533905974	0.71663067	NA
ASV_16s_3714	1.74910272	0.655438966	3.533898557	1.743154138	0.972680961	NA
ASV_16s_3715	1.752267871	0.680489667	3.549418236	2.679538529	0.912982197	NA
ASV_16s_3716	0.415341148	0.38036709	3.55049258	2.457765657	0.930243634	NA
ASV_16s_3717	1.303025624	0.017155233	3.548023075	3.345456679	0.851313215	NA
ASV_16s_3718	1.228053356	-0.863873774	3.547315025	2.019908267	NA	NA
ASV_16s_3719	1.453842759	-0.795320894	3.548095357	2.616987131	0.918033278	NA
ASV_16s_3720	1.598156768	-0.118736234	3.549930313	2.3044868	0.941086479	NA
ASV_16s_3721	1.346204881	-1.067575092	3.547703357	1.40625934	NA	NA
ASV_16s_3722	1.863558037	0.760750757	3.549286366	3.177843081	0.868077297	NA
ASV_16s_3723	1.740030696	0.968090826	3.549353964	2.377426684	0.93604077	NA
ASV_16s_3724	1.660491421	17.30179405	3.55078781	0.371884537	0.999793648	NA
ASV_16s_3725	0.798810827	2.051493591	3.548888893	0.746145	0.997955515	NA
ASV_16s_3726	1.250140723	0.188726463	3.548150017	3.351566156	0.850688284	NA
ASV_16s_3727	1.400070895	0.369898078	3.547886403	2.580918089	0.920881607	NA
ASV_16s_3728	1.277611861	0.749896764	3.546911001	3.111749129	0.874475601	NA
ASV_16s_3729	1.297024557	-0.817621327	3.547249551	2.050515198	0.956991587	NA
ASV_16s_3730	1.560193427	-0.157424705	3.548032387	3.084936085	0.877035305	NA
ASV_16s_3731	0.851398079	-26.77177667	3.551346016	-0.062827898	1	NA
ASV_16s_3732	1.041554055	0.98248715	3.547657146	1.654912658	0.976457265	NA
ASV_16s_3733	1.072279641	0.371496889	3.547967645	2.913025384	0.892929788	NA
ASV_16s_3734	1.035231804	0.505319792	3.548942278	2.228120819	NA	NA
ASV_16s_3735	0.644004717	0.540777234	3.551363661	0.681500568	NA	NA
ASV_16s_3736	1.567613754	0.381983794	3.550286978	0.983460773	0.995090943	NA
ASV_16s_3737	1.571106545	0.334246938	3.550602301	1.771949018	0.971378522	NA
ASV_16s_3738	1.616492284	1.159372013	3.550044552	1.608669756	0.978307348	NA
ASV_16s_3739	0.380142116	0.19165599	3.55064386	1.445154819	NA	NA
ASV_16s_3740	1.231237119	0.074906395	3.548645795	3.004140793	0.88461891	NA
ASV_16s_3741	1.241043539	0.064120256	3.548569745	3.028951153	0.882311152	NA
ASV_16s_3742	1.305812015	-0.263717528	3.548292412	2.674413611	0.913401298	NA
ASV_16s_3743	1.200726679	0.136830215	3.548314713	3.185264455	0.867351131	NA
ASV_16s_3744	1.341769115	-2.330095963	3.3214539	2.532263786	0.924648037	NA
ASV_16s_3745	1.383923825	-0.398875959	3.548194025	3.447934228	0.840709076	NA
ASV_16s_3746	1.153459312	-1.39001177	3.367414719	2.137116496	0.951862842	NA
ASV_16s_3747	1.646830819	0.513457031	2.4416698	8.817126187	0.266053728	NA
ASV_16s_3748	1.586586863	1.030478635	3.550192521	3.006359962	0.88441326	NA
ASV_16s_3749	0.283133235	-0.170173123	3.550600252	0.978425328	0.995169098	NA
ASV_16s_3750	1.530504404	-0.796348649	3.547896901	2.561709889	0.92237901	NA
ASV_16s_3751	1.928621471	0.271436201	3.5492166	1.406259332	NA	NA
ASV_16s_3752	0.580752192	-0.685681555	3.549877083	1.722040025	0.973614017	NA
ASV_16s_3753	1.27598299	-4.198595913	3.55005992	0.534549525	NA	NA
ASV_16s_3754	1.734225148	0.466376457	3.547971929	2.704236144	0.910949474	NA
ASV_16s_3755	0.504428368	-12.45198107	3.552157344	-0.135304816	NA	NA
ASV_16s_3756	1.204506705	0.040653426	3.548569822	2.971782307	0.887600266	NA
ASV_16s_3757	1.30609221	-0.120054065	3.547659255	2.400610116	0.93439346	NA
ASV_16s_3758	1.151762727	0.811813273	3.547873259	2.695846174	0.911642418	NA
ASV_16s_3759	1.27760493	0.710479742	3.547669218	3.167315634	0.869104735	NA
ASV_16s_3760	1.197929608	0.854951401	3.547535762	2.967082912	0.888030535	NA
ASV_16s_3761	1.32420172	1.065489451	3.547099583	3.043750411	0.880925664	NA
ASV_16s_3762	1.279648691	0.233806573	3.282588696	3.644660445	0.819674176	NA
ASV_16s_3763	0.923415497	1.415246018	3.547326345	2.477146176	0.928808126	NA
ASV_16s_3764	1.534935715	-0.69918585	2.305111159	6.605018551	0.471129126	NA
ASV_16s_3765	1.481731917	-0.246351416	3.548464668	3.400689115	0.845629727	NA

ASV_16s_3766	1.234980109	-0.088886251	3.549373228	2.503285992	0.926849423	NA
ASV_16s_3767	1.308211459	1.982613228	2.478294187	4.479505823	0.723183694	NA
ASV_16s_3768	1.126221619	1.994652472	3.547139893	1.284388792	0.988852345	NA
ASV_16s_3769	1.776502432	0.732249493	3.549288935	3.060745061	0.879326502	NA
ASV_16s_3770	1.48234445	0.315939234	3.550635728	1.958425769	0.962105493	NA
ASV_16s_3771	1.227606478	-0.312044504	3.549126684	2.851543842	0.898387545	NA
ASV_16s_3772	0.502613877	-12.65768009	3.552157344	-0.137523539	NA	NA
ASV_16s_3773	1.228210817	1.040145002	3.547462467	2.991724221	0.885766752	NA
ASV_16s_3774	1.440218921	-0.795531218	3.54805472	2.598512319	0.919498141	NA
ASV_16s_3775	1.109213399	0.410035327	3.428339872	1.467919975	NA	NA
ASV_16s_3776	1.612672253	-1.533693789	3.546267827	2.92775297	0.891604235	NA
ASV_16s_3777	1.382590096	-0.319995857	3.546774867	3.135815743	0.872160269	NA
ASV_16s_3778	1.578354427	0.694021229	3.550599321	1.924789694	0.963885461	NA
ASV_16s_3779	1.196035963	0.031304405	3.548681612	2.929752962	0.891423691	NA
ASV_16s_3780	1.222558691	-0.001893001	3.548432541	2.989706038	0.885952871	NA
ASV_16s_3781	1.169804668	0.030991096	3.548195908	3.13545782	0.872194825	NA
ASV_16s_3782	1.214358573	0.077762121	3.548320117	2.787606048	0.903930744	NA
ASV_16s_3783	1.357963538	-0.526568446	3.54798805	2.469075435	NA	NA
ASV_16s_3784	1.469571592	-0.797800141	3.215935285	3.110034769	0.874639891	NA
ASV_16s_3785	1.490519877	-0.925013853	3.547316708	2.195647448	0.948222757	NA
ASV_16s_3786	1.448128219	-0.096316509	3.549350839	1.812933695	0.969465017	NA
ASV_16s_3787	1.198523499	-0.32104854	3.549132265	2.818249521	0.901291158	NA
ASV_16s_3788	1.140411596	-0.329655444	3.549146222	2.759243358	NA	NA
ASV_16s_3789	1.153138781	0.632616279	3.548377487	3.038320196	NA	NA
ASV_16s_3790	1.305951242	0.483965198	3.547555464	3.354630503	0.850374481	NA
ASV_16s_3791	1.453127593	-0.795961897	3.547970989	2.567918313	0.921896509	NA
ASV_16s_3792	1.430993928	-0.795878181	3.547989476	2.56127215	0.922412976	NA
ASV_16s_3793	1.440773364	-0.79560635	3.548040522	2.594378118	0.919824238	NA
ASV_16s_3794	1.438271644	-1.500210715	3.056646828	2.715195117	0.910040628	NA
ASV_16s_3795	1.599405583	-0.353770606	3.273933912	3.52394672	0.832682602	NA
ASV_16s_3796	1.429970789	0.186442591	3.548011321	3.337784332	0.852096673	NA
ASV_16s_3797	0.824864721	0.229030458	3.550622295	1.108678088	0.992879894	NA
ASV_16s_3798	2.121575579	0.40546367	3.549241093	1.37009568	NA	NA
ASV_16s_3799	0.852709081	16.51234053	3.551453587	0.145850894	NA	NA
ASV_16s_3800	1.16338283	-0.442841699	3.549129703	2.569022695	0.921810531	NA
ASV_16s_3801	0.399605872	-0.681938019	3.54984881	0.918407701	NA	NA
ASV_16s_3802	1.303085479	-1.265346971	3.547952755	2.213572077	0.947080213	NA
ASV_16s_3803	1.220280621	0.233592597	3.547325125	3.209433723	0.864975603	NA
ASV_16s_3804	1.523998495	3.560264813	2.968935821	0.939759989	NA	NA
ASV_16s_3805	1.2536192	-1.480636664	3.546698254	1.28625057	0.988803573	NA
ASV_16s_3806	1.14927563	-0.069806821	3.549541134	2.697304684	0.911522135	NA
ASV_16s_3807	1.574368538	2.248894725	2.478084647	3.311092207	0.85481063	NA
ASV_16s_3808	1.189240687	2.293418622	3.547916176	1.517104719	NA	NA
ASV_16s_3809	1.584125598	-6.57647149	3.55010872	1.469364495	0.983351347	NA
ASV_16s_3810	1.458915149	0.700001919	3.548436917	1.749628125	0.972391147	NA
ASV_16s_3811	0.215422894	-0.17005824	3.550629821	0.841489276	0.996997127	NA
ASV_16s_3812	1.341687895	0.138260167	3.103661933	3.685321337	0.815224476	NA
ASV_16s_3813	1.399191978	0.253783376	3.109703983	2.643615982	0.915900133	NA
ASV_16s_3814	1.505570013	-0.92534931	3.547038587	2.120417419	0.952875763	NA
ASV_16s_3815	1.447573023	-0.795651897	3.548030115	2.59573828	0.91971702	NA
ASV_16s_3816	1.344886961	-1.013455939	3.548225784	1.272384832	0.989163662	NA
ASV_16s_3817	1.479031062	0.964329388	2.240161426	7.366757045	0.391717218	NA
ASV_16s_3818	1.394032414	2.373924349	3.548100473	0.775471779	NA	NA
ASV_16s_3819	1.630031251	0.734644431	3.548927741	1.767301628	0.971591073	NA
ASV_16s_3820	1.762369748	0.89258529	3.549351286	2.551224049	0.923190704	NA
ASV_16s_3821	0.381968076	-0.004827067	3.550596962	2.055891281	0.956682189	NA
ASV_16s_3822	0.314034443	-0.170217154	3.550590987	1.030784703	NA	NA
ASV_16s_3823	1.153964362	0.090284442	3.548349765	3.102424185	0.875368192	NA
ASV_16s_3824	1.545961527	0.51691685	3.382663359	2.713384566	0.91019107	NA
ASV_16s_3825	1.488725505	0.39883974	3.549173588	2.903964727	0.893741828	NA
ASV_16s_3826	1.171727796	0.778346594	3.547052053	2.771421628	0.905311997	NA
ASV_16s_3827	1.194138978	1.539999239	3.547084975	2.868171558	NA	NA
ASV_16s_3828	1.712179659	2.673571243	3.547476247	1.350932078	0.987026994	NA
ASV_16s_3829	0.395230625	-0.681883329	3.549861065	0.914862779	NA	NA
ASV_16s_3830	0.697551737	-13.97346556	3.552157344	0.063163937	0.99999953	NA
ASV_16s_3831	1.257957854	0.092210092	3.54792105	3.611514816	0.823276703	NA
ASV_16s_3832	0.986698204	-1.067479384	3.16239267	2.369978812	0.936565545	NA

ASV_16s_3833	1.371447962	-0.794724092	3.54822357	2.575819353	0.92128041	NA
ASV_16s_3834	1.198398859	0.311745535	3.548288801	3.027251805	0.882469818	NA
ASV_16s_3835	1.243798371	-1.575884144	3.544654979	1.211059449	NA	NA
ASV_16s_3836	1.280144832	0.385040463	3.55158819	0.518636158	0.999375342	NA
ASV_16s_3837	1.348492564	0.831215381	3.547249279	2.486487615	0.928111119	NA
ASV_16s_3838	0.781330854	0.223986574	3.550639161	1.265879886	0.989330096	NA
ASV_16s_3839	1.43502445	-2.029210407	3.545041392	1.598091418	0.978718175	NA
ASV_16s_3840	1.24477309	-15.41375623	3.552157344	0.192280209	0.999978016	NA
ASV_16s_3841	1.358205549	0.432557479	3.550635799	3.522519905	0.832834475	NA
ASV_16s_3842	1.744755122	1.187770094	3.549530681	1.515243873	0.98177722	NA
ASV_16s_3843	1.178629719	0.166149825	3.548675009	3.035107369	0.881735621	NA
ASV_16s_3844	1.358948065	-0.04634972	2.911104094	4.468159611	0.724547926	NA
ASV_16s_3845	1.381550583	0.090597472	3.548015121	3.593519303	0.825223051	NA
ASV_16s_3846	1.3356967	-1.035429959	3.547147545	2.064108433	0.95620698	NA
ASV_16s_3847	1.425341368	-0.836905263	3.548199757	2.418240787	0.93312677	NA
ASV_16s_3848	1.35074968	0.53498413	3.55049558	2.951832097	0.889422099	NA
ASV_16s_3849	1.280342456	-1.041064952	3.275985268	3.386173208	0.847130745	NA
ASV_16s_3850	1.257656217	0.333793151	3.305128032	3.235527019	0.862392935	NA
ASV_16s_3851	1.606859254	0.57096441	3.549264454	1.604889662	0.978454683	NA
ASV_16s_3852	1.353635556	1.816018317	2.7249432	2.748063211	0.907289702	NA
ASV_16s_3853	1.608782793	0.369436086	3.550441392	1.110199608	0.992849767	NA
ASV_16s_3854	1.520263404	0.559952187	3.550619429	3.62765649	0.82152512	NA
ASV_16s_3855	1.343043003	14.4386932	3.551492255	1.592708788	0.978925452	NA
ASV_16s_3856	1.187654632	0.687404973	3.547876402	2.919406499	0.89235631	NA
ASV_16s_3857	1.425642968	-0.796535668	3.547907702	2.319207868	NA	NA
ASV_16s_3858	1.267337563	0.50403022	3.547240393	3.247993249	0.861152521	NA
ASV_16s_3859	1.426776792	-0.796006156	3.548005886	2.56780018	0.921905704	NA
ASV_16s_3860	1.345121249	0.081593725	3.547977841	3.613813064	0.823027642	NA
ASV_16s_3861	1.282567753	0.544423714	3.184120436	2.041204762	0.957524585	NA
ASV_16s_3862	1.151218425	0.53772551	3.175779943	1.693393231	0.974850276	NA
ASV_16s_3863	0.9921284	0.745557708	3.549359023	2.066196745	NA	NA
ASV_16s_3864	1.256691196	0.110807904	3.546680417	2.913438985	NA	NA
ASV_16s_3865	1.476241827	-2.20406193	3.546424189	0.671113389	NA	NA
ASV_16s_3866	1.15553385	-0.992005392	3.54731738	2.433907887	0.931991101	NA
ASV_16s_3867	0.348831713	-0.170251369	3.550582517	1.08397608	NA	NA
ASV_16s_3868	1.153826215	0.085181063	3.5486453	2.972633058	0.887522298	NA
ASV_16s_3869	1.257103522	-0.87774894	3.549302795	2.7858605	0.904080146	NA
ASV_16s_3870	0.710497673	1.338925738	3.549552977	0.998148955	NA	NA
ASV_16s_3871	1.588663082	1.197956705	3.548361412	2.435659006	NA	NA
ASV_16s_3872	0.506631784	-0.722054757	3.549801461	1.616868186	0.977985781	NA
ASV_16s_3873	1.412889854	0.17786567	3.21175576	4.445053019	0.727323319	NA
ASV_16s_3874	1.528640559	0.262929335	3.54932207	3.920571925	0.788879219	NA
ASV_16s_3875	1.546810305	1.172018989	3.549995062	1.571859919	0.979717087	NA
ASV_16s_3876	1.141698231	-0.240347392	3.549161162	2.828614721	0.900391165	NA
ASV_16s_3877	0.429301957	0.110801304	3.550607067	2.464322347	0.929759581	NA
ASV_16s_3878	0.484468972	-10.03046878	3.552157344	-0.16001234	NA	NA
ASV_16s_3879	1.192104456	0.164427785	3.548889375	2.968846477	0.887869147	NA
ASV_16s_3880	1.101103424	0.77667187	3.548003533	2.58745476	0.92036894	NA
ASV_16s_3881	1.304114266	1.847511923	3.546586529	2.832833005	0.900023871	NA
ASV_16s_3882	1.147743237	-0.368396063	3.548234404	2.357888038	NA	NA
ASV_16s_3883	1.440517969	-0.796024164	3.547961615	2.547567702	0.923472779	NA
ASV_16s_3884	1.461552486	-0.796072228	3.547950852	2.558076703	0.92266071	NA
ASV_16s_3885	1.437132464	-0.814919865	3.01789557	3.523560325	0.832723735	NA
ASV_16s_3886	1.367282923	-0.926691437	3.2582632	2.389035077	0.935218539	NA
ASV_16s_3887	1.412895403	-0.925799031	3.057703739	2.762881357	0.906037257	NA
ASV_16s_3888	1.442170317	-0.334454657	3.547555332	3.490484655	0.836232308	NA
ASV_16s_3889	1.558422339	0.034338006	2.735512763	5.27770647	0.626113854	NA
ASV_16s_3890	1.114255324	-2.619835221	3.107330386	1.786063942	0.970727442	NA
ASV_16s_3891	1.503619249	-0.966537434	2.717893309	2.598714379	0.919482187	NA
ASV_16s_3892	1.080072126	-0.836439315	3.547787134	3.155789197	NA	NA
ASV_16s_3893	1.044287689	0.137677705	3.544934615	1.922154902	NA	NA
ASV_16s_3894	1.473034625	2.767979913	3.547798594	0.769009158	NA	NA
ASV_16s_3895	1.269789367	-0.386472695	3.547183431	1.777462229	NA	NA
ASV_16s_3896	1.595655202	0.347939532	3.549765937	2.545844494	0.923605547	NA
ASV_16s_3897	0.827902998	16.48789165	3.551454036	0.1277493	NA	NA
ASV_16s_3898	1.128007856	-0.244174716	3.549164407	2.81535618	0.901541739	NA
ASV_16s_3899	0.320817341	-0.140119586	3.55059007	1.194960771	0.991042462	NA

ASV_16s_3900	1.169673821	-0.026256143	3.54805764	3.144787027	0.87129292	NA
ASV_16s_3901	1.148465808	0.492881003	3.547995424	3.154988702	0.870303824	NA
ASV_16s_3902	1.24309201	0.225903033	3.30033736	3.328653781	0.853027087	NA
ASV_16s_3903	1.238500944	-0.363505792	3.547033453	2.834703168	NA	NA
ASV_16s_3904	1.180138674	0.607914677	3.547149584	3.174855112	0.868369228	NA
ASV_16s_3905	1.388645172	-0.647030767	3.547945399	2.458866076	0.930162509	NA
ASV_16s_3906	1.416261437	-0.79603935	3.547961917	2.522360782	0.925403884	NA
ASV_16s_3907	1.368213617	-0.924485681	3.058748497	2.700243998	0.911279502	NA
ASV_16s_3908	1.297231315	2.76196453	3.547594288	0.81830925	NA	NA
ASV_16s_3909	1.642246949	-0.41206213	3.549184846	4.429267192	0.729217106	NA
ASV_16s_3910	0.65244495	0.322934368	3.550329776	1.097888173	NA	NA
ASV_16s_3911	1.844175536	0.790571422	3.549473858	4.186043222	0.758116901	NA
ASV_16s_3912	0.473126978	0.120429541	3.550610944	2.118770519	0.952975041	NA
ASV_16s_3913	1.238930166	-0.254595596	3.547632015	3.015918808	0.883525715	NA
ASV_16s_3914	1.149471464	-0.341329685	3.547943285	2.031267972	0.958089486	NA
ASV_16s_3915	1.223175495	-0.394109296	2.755894751	3.670910588	0.816805271	NA
ASV_16s_3916	1.386944847	-0.795946179	3.547984827	2.514621341	0.925992045	NA
ASV_16s_3917	1.375895325	-0.229449079	2.810057508	3.533865498	0.831625579	NA
ASV_16s_3918	1.298812292	-1.555524221	2.268788513	1.542943424	0.980785609	NA
ASV_16s_3919	1.241331177	-1.908316083	3.547134847	0.688594449	NA	NA
ASV_16s_3920	0.809911942	16.32552626	3.551459046	1.241676641	0.989935432	NA
ASV_16s_3921	1.317783202	1.763086688	2.487846709	4.146280425	0.762783083	NA
ASV_16s_3922	1.621346923	1.288613897	3.547301639	2.07085187	NA	NA
ASV_16s_3923	0.758921314	1.836399768	3.549204968	0.985974912	NA	NA
ASV_16s_3924	1.015550107	1.490126026	3.547784916	1.732893314	0.973136719	NA
ASV_16s_3925	1.575183845	0.37285826	3.550404695	1.05175159	0.993949959	NA
ASV_16s_3926	1.478358117	0.650540217	3.548143341	3.699796534	0.813632515	NA
ASV_16s_3927	1.159365677	-0.407978128	3.549132446	2.647191362	0.915611777	NA
ASV_16s_3928	1.446077294	-0.796620227	3.547912966	2.248930113	0.944788473	NA
ASV_16s_3929	1.208712374	0.178539136	3.547964717	3.587550424	0.825867123	NA
ASV_16s_3930	1.182767488	0.006562731	3.545410004	2.051784674	NA	NA
ASV_16s_3931	1.453562913	0.20898611	3.264057732	3.768070819	0.806069998	NA
ASV_16s_3932	1.422503017	-0.795889097	3.547988175	2.552520564	0.923090563	NA
ASV_16s_3933	1.431360607	-0.795794256	3.548004655	2.570934931	0.921661554	NA
ASV_16s_3934	1.112999114	-0.913313876	3.548129493	1.609448367	NA	NA
ASV_16s_3935	1.364553536	-0.795133756	3.548142784	2.563734155	0.922221846	NA
ASV_16s_3936	1.153637416	-2.761581728	3.156408314	0.992509085	NA	NA
ASV_16s_3937	1.90076068	-0.496511772	3.548071878	3.765590817	NA	NA
ASV_16s_3938	1.633607551	0.552651125	3.508770447	3.647549461	0.819359111	NA
ASV_16s_3939	1.492926561	0.694012303	3.550602015	1.896412549	0.965350436	NA
ASV_16s_3940	0.552177976	1.556178461	3.549832103	0.811812993	0.997321521	NA
ASV_16s_3941	1.121114247	0.118681875	3.548201882	3.187526371	0.867129502	NA
ASV_16s_3942	1.15170064	-0.031836683	3.548148198	3.151348019	0.870657144	NA
ASV_16s_3943	1.098476296	0.785736196	3.548732109	2.737869782	0.908146864	NA
ASV_16s_3944	1.243731826	-0.420326121	3.546254647	2.406606974	0.933963959	NA
ASV_16s_3945	1.455142659	-0.796027513	3.547959569	2.560084093	0.922505126	NA
ASV_16s_3946	1.339744278	-0.794124413	3.548351054	2.556047378	0.922817842	NA
ASV_16s_3947	1.758839732	0.589195842	3.549144825	3.098548684	NA	NA
ASV_16s_3948	1.211058965	0.230700208	3.547988631	2.718290503	NA	NA
ASV_16s_3949	1.252827668	-0.114598593	3.54932108	2.331369988	0.93925114	NA
ASV_16s_3950	1.497158097	0.749994266	3.54895754	1.862505558	0.967056795	NA
ASV_16s_3951	1.227049568	1.386743854	2.980191065	2.588585617	NA	NA
ASV_16s_3952	1.27356486	-11.54283246	3.552157344	0.4857462	0.999497017	NA
ASV_16s_3953	1.575874881	1.142090542	3.550116654	1.615030898	0.978058086	NA
ASV_16s_3954	1.652724429	17.28651869	3.550777093	0.360362687	0.999814347	NA
ASV_16s_3955	0.475396519	-9.413510422	3.552157344	-0.171602621	NA	NA
ASV_16s_3956	1.134884978	-0.372946175	3.548488422	2.421276606	0.932907449	NA
ASV_16s_3957	1.158635937	0.578834408	3.547553107	3.094462128	0.876128316	NA
ASV_16s_3958	1.242593744	-0.251625678	3.547552794	3.160645687	0.869754081	NA
ASV_16s_3959	1.188577583	0.65789144	3.547252441	3.221962897	0.863737808	NA
ASV_16s_3960	1.45651752	-0.803763989	3.548865629	2.0214959	0.958641036	NA
ASV_16s_3961	1.360618571	0.792631906	3.549133222	3.026435517	0.882546002	NA
ASV_16s_3962	1.753124141	0.41938739	3.549198498	1.308739455	NA	NA
ASV_16s_3963	0.771568517	-0.020407256	3.550146616	1.484182584	0.982852211	NA
ASV_16s_3964	1.369395514	-0.463146765	3.549002312	1.808147823	0.96969208	NA
ASV_16s_3965	1.438695238	1.502540267	3.548715685	2.655329962	0.914953687	NA
ASV_16s_3966	1.590842035	0.363410864	3.549853015	1.561090862	0.980119017	NA

ASV_16s_3967	1.588807678	1.187092488	3.549686662	1.515379254		0.981772449	NA
ASV_16s_3968	1.32413968	2.562825625	3.546945635	0.582103541		0.999086883	NA
ASV_16s_3969	1.154674624	0.049800486	3.547571935	2.092300856	NA		NA
ASV_16s_3970	1.281736451	-0.086423681	2.434934791	6.139979532		0.523503202	NA
ASV_16s_3971	1.300569302	-0.795938158	3.547997683	2.424718505	NA		NA
ASV_16s_3972	1.41769537	-0.795812123	3.548002461	2.557783272		0.92268344	NA
ASV_16s_3973	1.24105188	-1.06682619	3.547277479	2.866268748		0.897091659	NA
ASV_16s_3974	1.31489832	1.347628606	3.202899173	1.926122944		0.963815805	NA
ASV_16s_3975	1.393505447	-0.594662832	3.133905955	1.957784209		0.962139884	NA
ASV_16s_3976	1.253041772	-0.963589838	3.548006424	2.128616061		0.952379881	NA
ASV_16s_3977	1.465840514	0.678803669	3.550610538	2.071964637		0.955750041	NA
ASV_16s_3978	1.519930487	-0.067419415	3.548331455	2.239756657		0.94538788	NA
ASV_16s_3979	1.579592656	0.908415752	3.548870203	1.324689384		0.98776714	NA
ASV_16s_3980	1.424117143	1.162502264	3.549257445	1.604186824		0.978482012	NA
ASV_16s_3981	1.457897764	0.694011244	3.550603211	1.884315479		0.965964729	NA
ASV_16s_3982	1.175979938	-0.224475727	3.547676809	2.823538278		0.900832393	NA
ASV_16s_3983	1.116840531	0.893090788	3.547611175	3.119293388	NA		NA
ASV_16s_3984	1.300974414	0.151718972	3.54806429	3.538943628		0.831083569	NA
ASV_16s_3985	1.168413837	-0.493568511	3.547397099	1.640610506		0.977038899	NA
ASV_16s_3986	1.150988286	0.403653307	3.549454834	1.242577524	NA		NA
ASV_16s_3987	1.210029089	-0.554843481	3.083694427	2.429175865		0.932335108	NA
ASV_16s_3988	1.40265686	1.003828505	3.547656451	2.506743135		0.926588445	NA
ASV_16s_3989	1.681664332	0.658773839	3.549051993	2.626785502		0.917251341	NA
ASV_16s_3990	1.627512785	16.95096182	3.551446052	0.542258191	NA		NA
ASV_16s_3992	1.082191892	0.130544186	3.548268818	3.127658538	NA		NA
ASV_16s_3993	1.135496603	0.088610827	3.548156065	3.204419967		0.86546972	NA
ASV_16s_3994	1.195704364	-0.288751533	3.547853322	2.96271606		0.888429736	NA
ASV_16s_3995	1.188654702	0.60830656	3.547369309	3.160115959		0.869805598	NA
ASV_16s_3996	1.018916547	-0.411464496	3.548897961	1.941923758	NA		NA
ASV_16s_3997	1.386890464	-0.796005932	3.547974644	2.50393878		0.926800179	NA
ASV_16s_3998	1.372819072	-0.792192561	3.548130528	2.117465998		0.953053601	NA
ASV_16s_3999	1.228807698	-0.117017261	3.549329599	2.247285954		0.944896153	NA
ASV_16s_4000	1.526297724	-0.663418275	3.061371522	1.218162768		0.990502674	NA
ASV_16s_4001	1.409307163	0.199954267	3.539873979	3.224910753		0.863445956	NA
ASV_16s_4002	1.782324442	0.821733387	3.48778925	3.162239261		0.869599055	NA
ASV_16s_4003	1.452127758	0.7917861	3.548362593	2.610118819		0.918579314	NA
ASV_16s_4004	1.111509702	-0.239463028	3.549169906	2.802435574		0.902657308	NA
ASV_16s_4005	0.344097357	-0.938820329	3.549941287	0.621795398		0.998867151	NA
ASV_16s_4006	1.372324764	-0.657953485	3.547900329	2.605229765		0.918966953	NA
ASV_16s_4007	1.149914245	1.184645583	3.547620522	2.980579262	NA		NA
ASV_16s_4008	1.275740936	-0.418943743	3.547135114	3.105952785		0.87503073	NA
ASV_16s_4009	1.314587645	-0.794725245	3.548227288	2.538636226		0.924159727	NA
ASV_16s_4010	1.171738455	-0.101883133	3.549435564	2.39322105		0.934920759	NA
ASV_16s_4011	0.676405794	-0.473836225	3.549893983	1.279713102	NA		NA
ASV_16s_4012	0.847525018	0.813049426	3.550894514	0.933030846	NA		NA
ASV_16s_4013	1.413387655	0.602522076	3.231271883	1.647794605		0.976747797	NA
ASV_16s_4014	0.927243029	1.264121604	3.550175549	1.095159146	NA		NA
ASV_16s_4015	1.008426259	-2.046047324	3.550836384	1.668672029	NA		NA
ASV_16s_4016	0.679609587	-13.15114679	3.552157344	0.047188821	NA		NA
ASV_16s_4017	1.124117732	0.137519632	3.548073695	3.313799378		0.854536209	NA
ASV_16s_4018	1.234217301	-0.363221013	3.547277594	2.753048047		0.906869223	NA
ASV_16s_4019	1.221785486	0.218705909	3.546335226	2.752320729		0.906930627	NA
ASV_16s_4020	1.373023408	-0.79573205	3.54802273	2.529998041		0.924821293	NA
ASV_16s_4021	1.244577526	-0.199791214	3.549016601	2.237883091		0.945509887	NA
ASV_16s_4022	0.538447342	1.337945032	3.549770415	0.908819873	NA		NA
ASV_16s_4023	0.734952605	0.219961452	3.550615821	1.436935635		0.984413006	NA
ASV_16s_4024	0.742627108	0.219961211	3.550615413	1.442221058		0.984242835	NA
ASV_16s_4025	1.260536832	6.338129009	3.544693895	-0.466218864	NA		NA
ASV_16s_4026	0.767823551	-2.65E-04	3.549682152	2.33094461	NA		NA
ASV_16s_4027	1.294332077	-0.116837827	3.549770107	2.365603399		0.936872827	NA
ASV_16s_4028	1.742493554	0.50065115	3.548339074	2.747681241		0.907321887	NA
ASV_16s_4029	1.53722298	0.343841336	3.549491208	2.792963589		0.903471542	NA
ASV_16s_4030	1.113591087	-0.334817524	3.549153088	2.729323479		0.908862743	NA
ASV_16s_4031	0.316396211	-0.082467985	3.55059337	1.416834455	NA		NA
ASV_16s_4032	0.307293393	-0.10307631	3.55059628	1.489587339	NA		NA
ASV_16s_4033	1.070395171	0.072132846	3.548492691	2.992660222	NA		NA
ASV_16s_4034	1.12095718	0.048236565	3.54806802	3.223496567		0.863585996	NA

ASV_16s_4035	1.209999726	1.01654718	3.546275522	2.041615804	0.957501129	NA
ASV_16s_4036	1.39324433	-0.924326382	3.547640907	1.908513879	0.964729813	NA
ASV_16s_4037	1.278520656	-0.965507406	2.874673238	3.586007891	0.826033448	NA
ASV_16s_4038	1.267995309	-0.053142117	3.548757131	3.468060684	0.838596793	NA
ASV_16s_4039	1.563257789	0.612815861	2.978048765	2.538090507	0.924201604	NA
ASV_16s_4040	0.92883802	0.506966614	3.548288268	2.411117038	0.933640027	NA
ASV_16s_4041	1.066371061	1.147472773	3.548317599	1.879950239	0.966184896	NA
ASV_16s_4042	1.280354967	0.464299526	3.549590026	1.03095985	NA	NA
ASV_16s_4043	1.054406136	-0.312077448	3.54917489	2.701406539	NA	NA
ASV_16s_4044	0.371895976	-0.681570333	3.549931249	0.895270816	NA	NA
ASV_16s_4045	1.144473743	-0.949914536	3.548864507	2.299013244	0.94145666	NA
ASV_16s_4046	1.085310715	-0.689474967	3.548457777	2.012142787	NA	NA
ASV_16s_4047	1.080048446	0.040445689	3.547987015	3.297884028	NA	NA
ASV_16s_4048	1.436961321	-0.562910364	3.062836802	3.40530755	0.845151079	NA
ASV_16s_4049	1.116477691	0.533299207	3.549101268	2.680170135	NA	NA
ASV_16s_4050	1.187096324	0.169715976	3.548037921	2.730965515	NA	NA
ASV_16s_4051	0.941279412	-0.078905912	3.547887925	2.505745152	0.926663828	NA
ASV_16s_4052	1.580963039	1.067465071	3.548677051	1.621007344	0.977822379	NA
ASV_16s_4053	1.419964654	17.18681722	3.55144581	0.45301346	0.99960099	NA
ASV_16s_4054	1.054258166	-0.255064891	3.549185171	2.744006759	NA	NA
ASV_16s_4055	1.07089875	-0.29897821	3.549172012	2.729284306	0.908866019	NA
ASV_16s_4056	1.050406372	-0.356093325	3.549169823	2.642542015	NA	NA
ASV_16s_4057	0.348598832	0.219994823	3.550659551	1.058051721	NA	NA
ASV_16s_4058	1.260701621	-0.722493821	3.548151716	2.416335256	0.933264253	NA
ASV_16s_4059	1.099060502	0.663804707	3.547155539	2.70241861	NA	NA
ASV_16s_4060	1.394185732	-0.795843543	3.548007286	2.539591161	0.924086421	NA
ASV_16s_4061	0.719610741	-0.332009963	3.550313723	1.539091597	0.980925367	NA
ASV_16s_4062	1.468656249	0.69722217	3.549224644	1.738794706	0.972875131	NA
ASV_16s_4063	1.777363579	0.573062957	3.548219815	2.95502295	0.889131555	NA
ASV_16s_4064	1.499453623	0.769496571	3.549997262	2.605985596	0.918907082	NA
ASV_16s_4065	1.11477713	-0.266554782	3.549165078	2.792882688	0.903478484	NA
ASV_16s_4066	1.090581182	-0.373859826	3.54915517	2.651384288	0.915273032	NA
ASV_16s_4067	1.133029566	0.106121895	3.548097229	3.271869336	0.85876517	NA
ASV_16s_4068	1.0887303	1.03340491	3.548067739	2.709816258	0.910487231	NA
ASV_16s_4069	1.063481104	-0.174962272	3.547974313	2.818206484	NA	NA
ASV_16s_4070	1.202791499	-0.32561338	3.547091255	3.068941006	0.87855219	NA
ASV_16s_4071	1.565180111	0.056965273	3.54965626	4.115368253	0.76639752	NA
ASV_16s_4072	1.220845176	-0.110149648	3.547000042	2.608597667	0.918700015	NA
ASV_16s_4073	0.749988712	1.852226397	3.548996333	1.134190895	NA	NA
ASV_16s_4074	1.358229017	0.74367577	3.550279682	1.604701023	0.97846202	NA
ASV_16s_4075	0.784492354	16.47655861	3.551454921	0.094723671	NA	NA
ASV_16s_4076	1.148913334	0.083632829	3.548007035	3.359364397	0.849889246	NA
ASV_16s_4077	1.321773905	-0.795303812	3.548109709	2.523995049	0.925279401	NA
ASV_16s_4078	1.254902649	-0.949130728	3.54750796	1.636744135	0.977194683	NA
ASV_16s_4079	1.430180787	-0.440371924	3.547906719	2.51473687	0.925983282	NA
ASV_16s_4080	1.186173054	-0.106324656	3.549358461	2.280209736	0.942719297	NA
ASV_16s_4081	2.198720145	-3.231783532	3.544734349	0.620726498	NA	NA
ASV_16s_4082	1.129861971	-1.062024287	3.548539697	1.042042652	0.994121521	NA
ASV_16s_4083	1.117663831	2.106924209	3.54754573	1.662335587	NA	NA
ASV_16s_4084	1.055487911	-0.146190415	3.548032941	2.284113111	NA	NA
ASV_16s_4085	0.509045381	0.311686616	3.550599604	1.015278209	NA	NA
ASV_16s_4086	1.64844795	0.095912745	3.549334212	3.317010441	0.854210464	NA
ASV_16s_4087	1.593616293	10.43541573	3.550500967	1.955841006	0.962243946	NA
ASV_16s_4088	1.368436564	0.362647891	3.550182642	1.54601623	0.980673684	NA
ASV_16s_4089	1.407858389	0.329406134	3.550616426	1.675929468	0.975587194	NA
ASV_16s_4090	1.296152688	17.12755812	3.551446575	0.397046438	0.999743014	NA
ASV_16s_4091	1.099954542	-0.320344445	3.549159356	2.734257221	0.908449779	NA
ASV_16s_4092	0.344481523	0.2199956	3.550660545	1.052025007	NA	NA
ASV_16s_4093	1.088107328	-0.019577372	3.54899063	2.719638403	0.909670941	NA
ASV_16s_4094	1.075956069	0.051266327	3.54854271	2.930823377	0.891327009	NA
ASV_16s_4095	1.19864046	-0.829829941	3.548662753	2.331386876	0.939249978	NA
ASV_16s_4096	1.053176151	0.075720757	3.548342026	3.010715191	NA	NA
ASV_16s_4097	1.144849287	-0.031501447	3.307961285	2.827977492	0.900446598	NA
ASV_16s_4098	1.157771186	0.926665685	3.547421927	2.877799924	0.896071866	NA
ASV_16s_4099	1.271539264	-1.499576434	3.547174281	1.71205184	0.974048936	NA
ASV_16s_4100	1.346492193	-0.923988232	3.547251428	2.047545705	0.957161972	NA
ASV_16s_4101	1.205401299	0.336416016	3.550607396	1.244075582	NA	NA

ASV_16s_4102	1.210182909	-0.636098535	3.548262082	3.548836739	0.830026007	NA
ASV_16s_4103	1.078848424	0.790392359	3.547380488	2.721714849	0.909497941	NA
ASV_16s_4104	0.834326216	13.76059897	3.551457523	0.247319046	NA	NA
ASV_16s_4105	1.255081731	-0.442323872	3.548756339	3.525453093	0.832522212	NA
ASV_16s_4106	1.344189374	0.786242795	3.547088025	2.175086756	0.949517311	NA
ASV_16s_4107	1.481241872	0.797357237	3.5494128	2.482468506	0.928411408	NA
ASV_16s_4108	1.47711808	17.22860615	3.550923453	0.35610633	0.999821617	NA
ASV_16s_4109	1.032616465	-0.257089746	3.549192051	2.722550185	NA	NA
ASV_16s_4110	0.364603898	-0.681459989	3.549955002	0.888897618	NA	NA
ASV_16s_4111	0.407463463	0.098333843	3.550614278	2.035756933	NA	NA
ASV_16s_4112	1.154887259	-0.666071013	3.548827955	2.505557729	0.926677981	NA
ASV_16s_4113	1.05576371	0.054331073	3.54838664	2.941359155	NA	NA
ASV_16s_4114	1.356435206	-0.79614609	3.547959987	2.441624542	0.931428276	NA
ASV_16s_4115	1.289959494	-0.795609162	3.548032446	2.452227512	0.930651215	NA
ASV_16s_4116	1.036633974	-1.405336846	3.548030148	1.242808077	0.989907621	NA
ASV_16s_4117	1.516338455	1.350251267	3.548513853	2.948165656	0.889755552	NA
ASV_16s_4118	1.093568185	0.649941214	3.547924502	2.386479829	0.935399978	NA
ASV_16s_4119	1.385231725	-1.108795969	3.240992021	3.748261043	0.808273238	NA
ASV_16s_4120	1.192183958	1.358600695	3.338968148	2.837283783	0.899635689	NA
ASV_16s_4121	0.751007546	0.229251362	3.55068588	0.999203509	0.994841375	NA
ASV_16s_4122	0.817832826	2.553569408	3.548748481	0.64822253	NA	NA
ASV_16s_4123	0.809102678	-8.089658381	3.549869611	0.408457923	NA	NA
ASV_16s_4124	1.715859848	0.743012208	3.549685533	4.076513926	0.77092354	NA
ASV_16s_4125	1.536100266	0.57974121	3.549374107	2.798879292	0.90296337	NA
ASV_16s_4126	1.391610573	0.492974166	3.54933249	2.91814165	0.892470088	NA
ASV_16s_4127	1.059951846	-0.29898914	3.549175482	2.719115547	0.909714479	NA
ASV_16s_4128	0.658454425	-12.91371344	3.552157344	0.027799809	NA	NA
ASV_16s_4129	1.096930897	0.08070397	3.548535287	3.008096465	0.884252233	NA
ASV_16s_4130	1.32110921	-0.795638285	3.54804598	2.495305316	0.927450164	NA
ASV_16s_4131	1.256375435	1.979978659	3.546914162	1.938955253	0.963141589	NA
ASV_16s_4132	1.155077562	-1.415758184	3.548075102	1.785304296	0.970762694	NA
ASV_16s_4133	0.873940438	-0.850848781	3.549449341	0.967546508	0.995335205	NA
ASV_16s_4134	0.524647027	0.296106689	3.551039393	0.829913994	NA	NA
ASV_16s_4135	1.024619172	-0.215076317	3.549201884	2.727359282	NA	NA
ASV_16s_4136	1.051035365	-0.27365546	3.549183584	2.730390102	0.908773536	NA
ASV_16s_4137	0.224372131	-0.170078047	3.550624895	0.861669205	NA	NA
ASV_16s_4138	1.121195248	0.120917621	3.548290547	3.188916434	0.866993229	NA
ASV_16s_4139	1.040356226	0.203766817	3.548225464	3.147282211	0.871051275	NA
ASV_16s_4140	1.206292186	0.138858985	3.546296733	2.66125762	0.914472883	NA
ASV_16s_4141	1.335877271	-0.795577591	3.548056008	2.514765248	0.925981129	NA
ASV_16s_4142	1.182033589	-1.098186971	3.548488676	1.965097458	NA	NA
ASV_16s_4143	1.325581951	0.407172275	3.548663575	3.002061331	0.884811476	NA
ASV_16s_4144	1.506316866	-1.452557867	3.545536012	2.393557769	0.934896776	NA
ASV_16s_4145	1.328071704	0.659421475	3.550500557	2.516777523	0.925828411	NA
ASV_16s_4146	1.370113525	0.753863217	3.54638707	2.638677004	0.916297708	NA
ASV_16s_4147	1.533966176	1.187630006	3.549561003	1.46170596	0.983605865	NA
ASV_16s_4148	1.02283336	-0.356087509	3.549179136	2.616287378	NA	NA
ASV_16s_4150	1.197779239	-0.852984034	3.548298219	2.249359204	0.944760353	NA
ASV_16s_4151	1.198871372	-0.379480757	3.547538305	2.581964553	NA	NA
ASV_16s_4152	1.02794682	1.814509297	3.547062772	2.575344718	NA	NA
ASV_16s_4153	1.35451144	-0.81838274	3.270047415	2.295598286	0.941687015	NA
ASV_16s_4154	1.346806848	-0.795561836	3.548057155	2.525024786	0.925200914	NA
ASV_16s_4155	1.149942868	-1.334989677	3.548006674	2.047381562	0.957171379	NA
ASV_16s_4156	1.217178852	-0.598148192	3.547561115	2.847548015	0.898737969	NA
ASV_16s_4157	0.911215894	-0.638204788	3.346207615	1.921573887	0.964053166	NA
ASV_16s_4158	0.909376289	0.194776301	3.547306115	2.691226132	NA	NA
ASV_16s_4159	1.186957383	-0.841351494	3.546954694	1.842411055	NA	NA
ASV_16s_4160	1.407965911	1.519679392	3.548783843	2.034223686	0.957921882	NA
ASV_16s_4161	1.309105678	-0.109512005	3.548633901	1.933823906	0.963412017	NA
ASV_16s_4162	1.425868543	1.175349142	3.549976071	1.525987245	0.98139632	NA
ASV_16s_4163	1.429768052	0.694004641	3.550604214	1.874363461	0.966465512	NA
ASV_16s_4164	1.049960939	-0.031162805	3.548194227	2.93139648	0.891275231	NA
ASV_16s_4165	1.053652352	0.065225595	3.54802812	3.301304149	NA	NA
ASV_16s_4166	0.961097892	1.992512449	3.546857754	2.32786386	NA	NA
ASV_16s_4167	1.196973262	-0.547918204	3.548971952	1.812999147	0.969461905	NA
ASV_16s_4168	1.315484152	-0.356180966	3.547822293	3.20676791	0.865238411	NA
ASV_16s_4169	1.419226601	0.179189179	3.54956627	2.221952136	0.946541606	NA

ASV_16s_4170	1.145128839	2.087315463	3.547499412	2.453883317	0.930529478	NA
ASV_16s_4171	1.51425041	-0.376588996	3.546578758	2.808353176	0.902147078	NA
ASV_16s_4172	0.717173898	0.22658817	3.550662805	1.108064545	0.992892019	NA
ASV_16s_4173	1.536423164	0.821522098	3.549357044	2.633646814	0.916701724	NA
ASV_16s_4174	1.416749849	0.219040413	3.549488747	3.009389115	NA	NA
ASV_16s_4175	1.593057561	0.794364819	3.548291154	2.066947813	NA	NA
ASV_16s_4176	1.08752374	0.130033706	3.548922282	2.874365986	0.896376012	NA
ASV_16s_4177	1.222237841	-0.40892322	3.547215704	2.830075977	0.900263998	NA
ASV_16s_4178	1.122398612	1.644900262	3.547024662	2.68852403	0.912245142	NA
ASV_16s_4179	1.268652946	-0.974564372	3.547638105	2.026454179	0.958361678	NA
ASV_16s_4180	1.338091183	-0.795576986	3.548055264	2.51635937	0.925860158	NA
ASV_16s_4181	1.195205816	-0.740486698	2.572325293	4.085373362	0.769893247	NA
ASV_16s_4182	1.202689037	1.833251612	3.324254785	2.565482729	0.922085965	NA
ASV_16s_4183	1.343954169	-1.205407971	3.120609872	2.745711778	0.907487752	NA
ASV_16s_4184	1.334680837	-0.999942003	3.087329145	3.977462943	0.782369556	NA
ASV_16s_4185	1.167256999	-0.758220272	3.54669339	2.620137713	0.917782232	NA
ASV_16s_4186	1.128648893	-0.09487831	3.550043291	1.290426548	NA	NA
ASV_16s_4187	1.622575339	0.720138599	3.549256891	2.347761265	NA	NA
ASV_16s_4188	1.160055343	0.395933732	3.551399431	0.552674305	NA	NA
ASV_16s_4189	1.530701668	0.350809249	3.550370973	1.323623441	0.987796643	NA
ASV_16s_4190	1.011097456	-0.22762261	3.549204241	2.712270566	NA	NA
ASV_16s_4191	1.108142975	-0.34791477	3.549152878	2.707043533	0.910717053	NA
ASV_16s_4192	1.022994493	0.188530745	3.548961124	2.670466833	0.913723419	NA
ASV_16s_4193	1.051597213	0.068899911	3.548553343	2.898376281	0.894241358	NA
ASV_16s_4194	1.108907597	-0.400271157	3.548557401	2.418178617	0.933131258	NA
ASV_16s_4195	1.018820992	-0.341178096	3.548024851	1.927020788	NA	NA
ASV_16s_4196	1.13473258	0.205939365	3.547992139	3.533442071	0.831670747	NA
ASV_16s_4197	1.197288224	0.712859859	3.546043267	2.365223808	0.93689945	NA
ASV_16s_4198	1.09981794	0.73685216	3.547642355	2.809923528	0.902011481	NA
ASV_16s_4199	1.277087935	-0.843054662	3.235024386	2.928808771	0.891508941	NA
ASV_16s_4200	1.297883123	-1.176944199	2.865277514	3.462278404	0.839204616	NA
ASV_16s_4201	1.260703782	-0.795206706	3.548135845	2.480969642	0.928523239	NA
ASV_16s_4202	1.272935682	-0.546020857	3.546868635	3.040979601	0.881185568	NA
ASV_16s_4203	1.13180103	2.378650117	2.933694423	1.504795514	0.982143175	NA
ASV_16s_4204	1.362161518	0.514624969	3.548262799	3.150202826	0.870768204	NA
ASV_16s_4205	1.194692707	2.014496139	3.54625608	2.155705829	NA	NA
ASV_16s_4206	1.026906828	-0.389861975	3.547511298	2.595459806	0.919738977	NA
ASV_16s_4207	1.194726647	-0.080490083	3.376741798	2.002148005	0.959721386	NA
ASV_16s_4208	1.112454551	-0.14092494	3.549516087	2.239068292	0.945432723	NA
ASV_16s_4209	1.083678669	-0.104178323	3.549444529	2.29940492	NA	NA
ASV_16s_4210	1.450301697	0.441583852	3.550003415	0.767417834	NA	NA
ASV_16s_4211	1.470380541	1.036831117	3.547210366	2.827527579	NA	NA
ASV_16s_4212	0.696247772	0.219963171	3.550618017	1.409390037	0.985281919	NA
ASV_16s_4213	1.339709654	1.050011668	3.549330058	1.552679579	0.980429651	NA
ASV_16s_4214	1.261093527	0.780914579	3.4739022	3.921839587	0.788734711	NA
ASV_16s_4215	1.387829924	1.034393825	3.550172837	2.094316411	0.954436092	NA
ASV_16s_4216	0.352936573	-0.681286821	3.549995034	0.878435039	NA	NA
ASV_16s_4218	1.101202632	-0.161276792	3.548327813	2.539596582	0.924086005	NA
ASV_16s_4219	1.048664307	1.4789774	3.547252736	2.704172535	NA	NA
ASV_16s_4220	1.139143097	-0.468486387	3.54795847	2.387546812	0.935324246	NA
ASV_16s_4221	0.967695768	1.86814151	3.546678423	2.263820857	0.943808312	NA
ASV_16s_4222	1.258933401	-0.794428382	3.548293092	2.504063694	0.926790754	NA
ASV_16s_4223	1.368869941	0.161859627	3.549238062	2.603832573	0.919077572	NA
ASV_16s_4224	0.710024746	0.219962598	3.550617209	1.419356327	0.984971009	NA
ASV_16s_4225	0.782599523	-0.588335371	3.550054309	1.879864833	0.966189195	NA
ASV_16s_4226	1.046723211	-0.069707264	3.549381867	2.823229986	0.900859161	NA
ASV_16s_4227	0.925177045	-0.175229949	3.548222899	1.699270628	NA	NA
ASV_16s_4228	1.096363115	-10.99804013	3.552157344	0.401865089	NA	NA
ASV_16s_4229	1.090529943	17.06569339	3.551447556	0.286584158	NA	NA
ASV_16s_4230	1.043630092	-0.277289396	3.549185223	2.72093102	0.909563264	NA
ASV_16s_4231	0.351478157	-0.681262952	3.550000221	0.87710298	NA	NA
ASV_16s_4232	1.09120948	0.068277946	3.548271963	3.189579152	0.866928241	NA
ASV_16s_4233	1.348131071	-0.833945767	3.547740969	2.10625917	0.953725618	NA
ASV_16s_4234	1.273468682	-0.795397475	3.548102342	2.47971722	0.928616618	NA
ASV_16s_4235	1.214186863	-0.914046231	2.882928837	3.06729861	0.878707516	NA
ASV_16s_4236	1.297286887	-1.168847698	3.10083646	2.053943857	0.956794403	NA
ASV_16s_4237	1.282182406	0.254400566	3.490351651	3.278319096	0.858117664	NA

ASV_16s_4238	1.074019773	-0.649789168	3.548420825	1.859452517	0.967208081	NA
ASV_16s_4239	1.407944974	-0.113485698	3.548841492	2.38974247	0.935168264	NA
ASV_16s_4240	1.148177453	2.970377387	2.885095029	0.918369095	0.996040116	NA
ASV_16s_4241	1.47005264	0.91538449	3.549068404	2.375543933	0.936173632	NA
ASV_16s_4242	0.785162218	1.611326557	3.548715677	1.593799694	0.978883539	NA
ASV_16s_4243	0.856321594	-0.162694514	3.54953657	2.052885909	NA	NA
ASV_16s_4244	0.961087679	0.310360451	3.549176261	0.668601977	0.998565525	NA
ASV_16s_4245	0.691432407	0.219963252	3.550618307	1.40585606	0.985391224	NA
ASV_16s_4246	1.227320988	3.319210213	3.546571554	1.162887265	NA	NA
ASV_16s_4247	1.471146611	0.323440396	3.194670728	4.110602018	0.766953762	NA
ASV_16s_4248	1.324413432	0.31593918	3.550641515	1.900953813	0.965118252	NA
ASV_16s_4250	0.380884963	-0.128532544	3.549478951	0.887267494	NA	NA
ASV_16s_4251	1.075830069	0.010679882	3.548341504	2.796952448	0.903129021	NA
ASV_16s_4252	1.05355533	0.253521637	3.548047479	3.35244809	NA	NA
ASV_16s_4253	0.921683093	0.830633073	3.547165591	2.712010088	NA	NA
ASV_16s_4254	1.239467966	-0.802275012	3.548086726	2.223110915	0.946466906	NA
ASV_16s_4255	1.314893677	-0.827897145	3.548152299	2.537798548	0.924224003	NA
ASV_16s_4256	1.324530696	0.867351048	3.548295466	1.877519168	0.966307165	NA
ASV_16s_4257	1.089766442	-0.494601063	3.323689475	2.235070594	0.945692771	NA
ASV_16s_4258	1.355879904	0.377261263	2.492806412	4.463714175	0.725082181	NA
ASV_16s_4259	1.393864585	0.363155791	3.55048095	1.092648113	0.99319242	NA
ASV_16s_4260	1.241094495	1.374812424	3.549316722	2.328170134	0.939471092	NA
ASV_16s_4261	1.290145763	0.389448796	3.550639361	3.246236854	0.86132754	NA
ASV_16s_4262	0.026786389	0.497149673	3.551985684	0.092979958	0.999998204	NA
ASV_16s_4263	1.044875665	-0.271897725	3.549186007	2.725671071	0.909167912	NA
ASV_16s_4264	0.632010472	-13.75785614	3.552157344	0.002677312	NA	NA
ASV_16s_4265	1.02331543	0.041330789	3.548732386	2.781130072	0.904484505	NA
ASV_16s_4266	1.119083163	-0.248790312	3.54814031	2.753898058	0.906797437	NA
ASV_16s_4267	1.196857566	0.850007872	3.106499631	3.248329236	0.861119032	NA
ASV_16s_4268	1.212352727	-0.51604809	3.548154578	2.375809698	0.936154886	NA
ASV_16s_4269	1.243223505	-0.795021837	3.548174169	2.476912447	0.928825523	NA
ASV_16s_4270	1.478210991	-0.054476985	3.547001098	3.447445054	0.840760296	NA
ASV_16s_4271	1.31215241	-0.718732667	2.436749501	3.778347382	0.804924217	NA
ASV_16s_4272	1.209629406	0.128568259	3.546558362	2.069232017	NA	NA
ASV_16s_4273	1.087362413	0.407184394	3.54963091	1.056014169	NA	NA
ASV_16s_4274	1.061732991	0.40363717	3.549500642	1.216514635	NA	NA
ASV_16s_4275	1.03236888	0.407685317	3.547609638	2.301099387	NA	NA
ASV_16s_4276	1.890925415	0.403728511	3.549241325	1.402720346	NA	NA
ASV_16s_4277	0.766930872	0.663739571	3.549896478	2.061845974	0.956338099	NA
ASV_16s_4278	0.918836908	1.951115618	3.547982688	2.164219259	0.950194624	NA
ASV_16s_4279	1.087723984	1.308937227	2.453320543	4.019511522	0.777527337	NA
ASV_16s_4280	1.229375242	0.341481574	3.549407815	2.811411032	NA	NA
ASV_16s_4281	1.114899379	0.082847064	3.549342672	3.424907363	0.84311404	NA
ASV_16s_4282	0.98089846	-0.289148571	3.549204768	2.650997755	NA	NA
ASV_16s_4283	0.347102911	-0.681192376	3.550016046	0.873073775	NA	NA
ASV_16s_4284	0.377816913	0.050995118	3.550608441	2.045546531	0.957276476	NA
ASV_16s_4285	0.283719279	-0.143387997	3.550601767	1.209271135	NA	NA
ASV_16s_4286	1.091019623	-1.692446824	3.546221995	2.159107486	0.950511553	NA
ASV_16s_4287	1.121030422	-0.982863878	2.88932458	3.789317672	0.803698987	NA
ASV_16s_4288	1.269991935	0.00352299	3.548689536	2.06902899	0.955921084	NA
ASV_16s_4289	1.41817278	1.188157446	3.549439369	1.385236225	0.986019178	NA
ASV_16s_4290	1.399835899	0.694004539	3.550605326	1.863574295	0.967003745	NA
ASV_16s_4291	1.042667511	-0.257217432	3.549188685	2.731932794	0.908644443	NA
ASV_16s_4292	1.071608155	0.674918705	3.548243578	2.984083013	NA	NA
ASV_16s_4293	1.065664178	0.132102603	3.548340695	3.05890775	0.879499807	NA
ASV_16s_4294	1.097685649	0.54564671	3.338144021	2.392802348	0.934950575	NA
ASV_16s_4295	1.16234484	0.147118206	3.546728728	2.521401751	0.925476888	NA
ASV_16s_4296	1.216612404	-0.043311324	3.292138501	3.577251163	0.826976685	NA
ASV_16s_4297	1.27405409	-2.605260246	3.54660321	1.252744515	0.989661334	NA
ASV_16s_4298	1.178436536	-1.015181184	3.547459999	2.022213316	0.958600679	NA
ASV_16s_4299	1.115602142	2.303450246	3.548596958	0.818602208	0.997249445	NA
ASV_16s_4300	0.749943417	17.75661052	3.551446426	0.026308493	NA	NA
ASV_16s_4301	1.404632067	-0.066663665	3.549266347	2.868228279	0.896918669	NA
ASV_16s_4302	0.185080662	-0.169977362	3.550650079	0.766749082	NA	NA
ASV_16s_4303	0.624077287	-14.42999292	3.552157344	-0.005075746	NA	NA
ASV_16s_4305	1.024321678	0.076852462	3.548490787	2.931651202	NA	NA
ASV_16s_4306	1.02235125	-0.308764402	3.548746863	2.356606077	0.937502352	NA

ASV_16s_4307	1.295570982	-0.795988489	3.547988252	2.399752903	0.934454741	NA
ASV_16s_4308	0.572851002	0.895389036	3.550259115	1.102053365	NA	NA
ASV_16s_4309	1.012674113	-0.118415344	3.549141139	1.620914831	0.977826039	NA
ASV_16s_4310	1.239697384	0.298892814	3.551819311	0.386401568	0.99976537	NA
ASV_16s_4311	1.375255766	16.51189118	3.551449855	1.752710523	0.972252546	NA
ASV_16s_4312	0.982466623	-0.235604657	3.54921326	2.681876683	NA	NA
ASV_16s_4313	1.03209592	-0.294465895	3.549185529	2.696724432	0.911569997	NA
ASV_16s_4314	0.271316523	-0.170162597	3.550604355	0.956898016	NA	NA
ASV_16s_4315	1.230619279	-0.796322641	3.548156091	1.853256733	0.967513901	NA
ASV_16s_4316	1.030960173	0.162905647	3.548106461	3.282613664	NA	NA
ASV_16s_4317	1.086420888	0.041351292	3.547988275	3.296929327	0.856243203	NA
ASV_16s_4318	0.98705341	-0.340759982	3.548029584	1.996507756	NA	NA
ASV_16s_4319	1.142489262	2.285717063	3.54805592	0.709816067	NA	NA
ASV_16s_4320	1.251970953	-0.819452637	3.547943331	2.102129291	0.953971967	NA
ASV_16s_4321	1.094832041	1.920596436	3.546397764	2.231561988	NA	NA
ASV_16s_4322	1.26602452	-0.239398656	3.547617178	3.185933637	0.867285578	NA
ASV_16s_4323	0.807779829	-0.453792475	3.547360915	1.008703004	0.994686921	NA
ASV_16s_4324	0.791445732	-0.014327947	3.549136269	3.030247588	NA	NA
ASV_16s_4325	1.026515044	0.509793795	3.549101044	2.910300134	0.89317431	NA
ASV_16s_4326	1.226712055	-0.207427617	3.545634632	1.209118162	0.990715437	NA
ASV_16s_4327	1.010165677	-0.470218128	3.548211274	2.410374445	0.933693417	NA
ASV_16s_4328	1.357676701	0.830194864	3.549825826	1.971473868	0.961402321	NA
ASV_16s_4329	1.546558211	0.58891597	3.54835807	2.651153607	0.915291685	NA
ASV_16s_4330	1.425843797	0.69400742	3.550604358	1.872945244	0.966536539	NA
ASV_16s_4331	0.973749278	-0.194136699	3.549224958	2.676996311	NA	NA
ASV_16s_4332	0.964132467	-0.103551322	3.54911006	2.642721603	NA	NA
ASV_16s_4333	0.992624689	-0.17626482	3.54834253	2.401041438	NA	NA
ASV_16s_4334	1.114321801	0.213666704	3.309639326	2.989382146	0.885982729	NA
ASV_16s_4335	1.081923819	-0.494195562	3.12277276	2.71288788	0.91023232	NA
ASV_16s_4336	1.19801357	-0.634181496	3.546270398	1.797845777	0.970177608	NA
ASV_16s_4337	1.077391892	-0.521434914	3.547154781	2.289366715	NA	NA
ASV_16s_4338	1.044359575	-0.519895797	3.549747301	0.791443799	0.997530295	NA
ASV_16s_4339	1.270920656	0.334050259	3.550616048	1.681496672	0.975353649	NA
ASV_16s_4340	0.3914758	0.252213591	3.550612957	2.636213927	0.916495652	NA
ASV_16s_4341	0.319777668	0.220000521	3.550667044	1.014290635	NA	NA
ASV_16s_4342	1.02654862	-0.025760716	3.54918159	2.746130444	0.907452504	NA
ASV_16s_4343	1.022085835	0.108146853	3.548228735	3.076757967	0.87781182	NA
ASV_16s_4344	1.046300751	0.106403151	3.54809461	3.262742913	NA	NA
ASV_16s_4345	1.133998412	1.743620656	3.547812979	0.873546015	0.99661896	NA
ASV_16s_4346	1.054672462	1.59566527	3.547133039	2.504989633	NA	NA
ASV_16s_4347	1.337428341	-0.701552421	3.547291547	2.075203117	NA	NA
ASV_16s_4348	1.028835552	-1.24692358	3.546526997	1.658066169	NA	NA
ASV_16s_4349	0.799279014	-0.925232054	3.548442025	1.474322962	NA	NA
ASV_16s_4350	1.172648214	-1.294627009	3.547981999	1.554293465	0.980370273	NA
ASV_16s_4351	1.336961402	-0.105908565	3.550523327	2.00925211	0.95932651	NA
ASV_16s_4352	1.056630852	2.600206638	3.547699919	1.364475407	NA	NA
ASV_16s_4353	1.120519011	-0.836234756	3.547038626	1.818095033	0.969219067	NA
ASV_16s_4354	0.699896894	-6.800596755	3.552157344	0.06515736	NA	NA
ASV_16s_4355	1.349514316	0.932111529	3.549494794	1.505824064	0.982107346	NA
ASV_16s_4356	1.417182617	1.186914754	3.549727997	1.468617544	0.983376275	NA
ASV_16s_4357	0.947145285	-0.211287141	3.549231664	2.650108057	NA	NA
ASV_16s_4358	1.016879042	-0.326571789	3.549185167	2.649609701	0.915416477	NA
ASV_16s_4359	0.613499705	-6.732755076	3.552157344	-0.015539768	NA	NA
ASV_16s_4360	1.022883471	-0.404585444	3.54889102	2.380605017	0.935816167	NA
ASV_16s_4361	1.007535581	0.526494605	3.548594467	2.843977995	0.899050603	NA
ASV_16s_4362	1.061183521	0.001784198	3.548118973	3.094324115	0.876141476	NA
ASV_16s_4363	0.959296351	-0.342074368	3.548222037	1.64901449	NA	NA
ASV_16s_4364	1.071691118	-0.393509662	3.548185673	2.346495701	NA	NA
ASV_16s_4365	0.965796805	-0.341320284	3.548087337	1.824125764	NA	NA
ASV_16s_4366	1.209851318	-0.698544679	3.040548277	3.171658767	0.86868124	NA
ASV_16s_4367	1.267832022	-0.795771829	3.548032919	2.425869216	0.932574983	NA
ASV_16s_4368	1.219279099	0.617386443	3.547568735	3.212582191	0.864664961	NA
ASV_16s_4369	1.233392734	0.071480859	3.0973015	4.128381067	0.764877418	NA
ASV_16s_4370	0.79066654	-0.280123392	3.549558834	1.348151799	0.987106669	NA
ASV_16s_4371	0.988058401	0.124956564	3.54930044	2.538963633	NA	NA
ASV_16s_4372	0.87922762	2.158531242	3.548856227	1.129757418	0.992455337	NA
ASV_16s_4373	0.993596646	-0.22466111	3.549211111	2.695556528	0.911666296	NA

ASV_16s_4374	0.300672111	-0.170201667	3.550594764	1.008727797	NA	NA
ASV_16s_4375	1.011108399	0.035577416	3.548958333	2.704921349	0.910892772	NA
ASV_16s_4376	1.229851256	-0.795055663	3.548168333	2.463927861	0.92978875	NA
ASV_16s_4377	1.182775326	-1.329994939	3.546894304	1.190341173	NA	NA
ASV_16s_4378	0.741129181	-0.823680101	3.549824581	1.187439855	0.991213223	NA
ASV_16s_4379	1.022608823	0.403629245	3.549523231	1.204393798	NA	NA
ASV_16s_4380	1.945511422	-0.568530752	3.548889152	1.405918851	0.985389286	NA
ASV_16s_4381	0.48397407	1.337512859	3.549871255	0.874448356	NA	NA
ASV_16s_4382	0.95858454	-0.069751696	3.54971514	2.448959372	0.930891186	NA
ASV_16s_4383	1.002401106	0.361983856	3.551289085	0.564903702	NA	NA
ASV_16s_4384	0.768519508	-0.198450328	3.548659885	1.906542133	NA	NA
ASV_16s_4385	1.334806803	0.315939019	3.550641093	1.904916547	0.964914943	NA
ASV_16s_4386	1.011176833	-0.413288856	3.54917882	2.501070385	0.927016442	NA
ASV_16s_4387	1.040798712	-0.006140175	3.548238768	2.938493603	0.890633156	NA
ASV_16s_4388	1.073560265	0.749673143	3.547968191	2.807028269	0.902261418	NA
ASV_16s_4389	1.009639772	1.263061538	3.547548581	2.639435509	0.916236708	NA
ASV_16s_4390	1.241920772	-0.795970454	3.548003256	2.329195783	0.939400635	NA
ASV_16s_4391	1.060306185	0.363018044	3.547446075	2.317185362	0.940223094	NA
ASV_16s_4392	1.175768169	-0.794349361	3.548319918	2.444695811	0.931203634	NA
ASV_16s_4393	1.217180072	-0.794021933	3.548381445	2.47722579	0.9288022	NA
ASV_16s_4394	1.068623568	-0.296807005	2.969219093	2.447379381	0.931007054	NA
ASV_16s_4395	1.163574587	-1.291779069	3.122568628	1.640715341	0.977034666	NA
ASV_16s_4396	1.240970593	0.015282106	2.743912059	3.724753895	0.81087825	NA
ASV_16s_4397	1.134559394	-0.103913938	3.549741142	1.739759322	NA	NA
ASV_16s_4398	0.657515196	0.219964934	3.550620473	1.380252881	0.986168436	NA
ASV_16s_4399	0.697533928	-0.385969173	3.550240979	1.145793285	NA	NA
ASV_16s_4400	1.119413355	0.833328501	3.548416508	2.95221568	0.889387189	NA
ASV_16s_4401	1.614217386	0.702611282	3.549304069	3.112608763	0.874393188	NA
ASV_16s_4402	0.33397717	-0.68096626	3.550065977	0.860675945	NA	NA
ASV_16s_4403	0.504570319	0.366653655	3.55061232	2.815434401	NA	NA
ASV_16s_4404	0.446224807	0.219980887	3.550641362	1.183251712	0.991307424	NA
ASV_16s_4405	1.248457681	-0.796100749	3.54799126	2.292147021	0.941919349	NA
ASV_16s_4406	1.240688191	-0.795271145	3.548125593	2.459052666	0.930148748	NA
ASV_16s_4407	1.034478998	0.927654092	3.545599681	1.862271354	0.967068415	NA
ASV_16s_4408	1.11854205	1.528757884	3.546390824	1.841894254	NA	NA
ASV_16s_4409	1.319761318	0.743343885	3.549384292	2.190543526	0.948545712	NA
ASV_16s_4410	1.020090082	-0.285232899	3.5491915	2.692599263	0.911909922	NA
ASV_16s_4411	0.993300925	-0.406240582	3.549185695	2.498440788	0.927214429	NA
ASV_16s_4412	1.019868438	0.605265412	3.54796382	2.977775183	0.887050566	NA
ASV_16s_4413	1.033848567	0.972383629	3.547901919	2.621464373	0.917676413	NA
ASV_16s_4414	1.036360941	-9.53E-04	3.547772412	2.652763201	0.915161492	NA
ASV_16s_4415	1.062086649	1.201480271	3.547405764	2.458451061	0.93019311	NA
ASV_16s_4416	1.041270367	1.616011096	3.546861316	2.451334957	0.930716793	NA
ASV_16s_4417	0.999518664	-1.763947288	3.547926686	1.211783021	0.990653061	NA
ASV_16s_4418	1.059184318	2.332529033	3.546153959	2.226197418	NA	NA
ASV_16s_4419	1.326324889	-1.56182125	2.042069013	3.965108906	0.783787312	NA
ASV_16s_4420	1.175898891	1.61220385	3.148535787	2.552198716	0.923115428	NA
ASV_16s_4421	0.743120723	1.118426606	3.550352565	1.021953208	NA	NA
ASV_16s_4422	1.525619482	0.419367759	3.549241857	1.2638565	NA	NA
ASV_16s_4423	1.09721312	0.419526563	3.548557063	1.673898894	NA	NA
ASV_16s_4424	1.704450581	1.206619877	3.549096462	1.332842742	NA	NA
ASV_16s_4425	1.341120569	1.180119897	3.549325369	1.470589581	0.983310416	NA
ASV_16s_4426	1.682776502	1.206599433	3.549100279	1.328703767	NA	NA
ASV_16s_4427	0.972287689	-0.199503123	3.549224304	2.675880183	0.913281462	NA
ASV_16s_4428	1.070580687	-0.427672132	3.549157981	2.524647331	0.925229689	NA
ASV_16s_4429	0.28871181	0.205629015	3.550677272	1.083651976	0.993363938	NA
ASV_16s_4430	0.92736892	-0.68477163	3.549215552	1.189591779	NA	NA
ASV_16s_4431	0.396769094	0.250645625	3.550632005	2.247911287	0.944855211	NA
ASV_16s_4432	0.999502602	0.365891143	3.549006322	2.728372829	0.908942217	NA
ASV_16s_4433	0.943085856	0.054408282	3.548585921	2.771203671	NA	NA
ASV_16s_4434	1.040596164	0.062463877	3.548318948	2.971692146	0.887608527	NA
ASV_16s_4435	1.073839717	0.19317482	3.548142333	3.238328212	0.862114576	NA
ASV_16s_4436	1.007801693	-0.16998535	3.548035665	2.83382405	0.899937492	NA
ASV_16s_4437	1.215168109	-0.796166828	3.548009531	2.186538547	0.948798391	NA
ASV_16s_4438	0.95531093	8.78E-04	3.548251214	2.168840625	NA	NA
ASV_16s_4439	0.970465747	-0.232402644	3.546854742	1.94934782	0.962590528	NA
ASV_16s_4440	1.206262039	-0.795195319	3.548153644	2.437660269	0.931717701	NA

ASV_16s_4441	1.203422489	-0.795127476	3.54815741	2.436378113	0.93181118	NA
ASV_16s_4442	1.113859958	-0.76903869	3.078257939	2.49701192	0.927321902	NA
ASV_16s_4443	1.193288019	-0.794299211	3.548329548	2.458614288	0.930181075	NA
ASV_16s_4444	0.808804012	0.459889092	2.968199008	2.301943573	0.941258627	NA
ASV_16s_4445	1.374636489	-0.155781743	3.547815975	2.815834314	0.901500349	NA
ASV_16s_4446	1.233589685	-0.106802885	3.549445485	3.153118543	0.870485366	NA
ASV_16s_4447	1.260243251	-0.052198907	3.547754691	3.112463774	0.87440709	NA
ASV_16s_4448	1.327117136	1.668607629	3.102026761	1.216128181	0.990550796	NA
ASV_16s_4449	0.570191753	0.419125717	3.54979865	0.946580018	NA	NA
ASV_16s_4450	0.64092512	0.219965871	3.550621616	1.367250655	0.986553295	NA
ASV_16s_4451	0.718300235	0.596370182	3.550588156	1.35562815	NA	NA
ASV_16s_4452	0.637320644	0.204462328	3.550588316	1.142240442	0.992196572	NA
ASV_16s_4453	1.299546186	0.65828922	3.549408787	2.114958077	0.953204435	NA
ASV_16s_4454	0.997687956	1.481218374	3.547632993	2.378508935	NA	NA
ASV_16s_4455	0.850819767	0.336449738	3.549754792	2.280366003	NA	NA
ASV_16s_4456	0.280272733	-0.599073194	3.550819966	0.79262582	NA	NA
ASV_16s_4457	1.400977177	0.93725948	3.547373728	1.670883732	NA	NA
ASV_16s_4458	1.257025908	0.363452249	3.549757906	1.421129953	0.984915266	NA
ASV_16s_4459	1.389475318	15.56070679	3.551461051	2.031046426	0.958102034	NA
ASV_16s_4460	0.97232802	-0.305408346	3.549204914	2.627953471	0.917157902	NA
ASV_16s_4461	0.95369877	-0.341323349	3.54810017	1.807890072	NA	NA
ASV_16s_4462	1.045049742	0.811530763	3.547734293	2.737964527	0.908138913	NA
ASV_16s_4463	0.865316071	0.735441161	3.548522725	2.655898241	NA	NA
ASV_16s_4464	0.952735775	1.10446094	3.545284927	0.541307691	0.999280733	NA
ASV_16s_4465	1.199004323	-0.795047316	3.548174292	2.438070851	0.931687753	NA
ASV_16s_4466	0.760887816	0.521444746	3.549028063	1.395002113	0.985723853	NA
ASV_16s_4467	1.129385537	1.030911246	3.117289977	3.123824623	0.873315968	NA
ASV_16s_4468	0.5830231	0.702604857	3.55103563	1.159484013	NA	NA
ASV_16s_4469	1.115237485	0.277612608	3.549342124	3.605837547	NA	NA
ASV_16s_4470	1.213191232	0.315939289	3.550646494	1.856241098	0.967366795	NA
ASV_16s_4471	1.269424096	0.693981617	3.550610778	1.813729372	0.969427174	NA
ASV_16s_4472	0.96366411	-0.348397559	3.549201809	2.568500524	0.921851189	NA
ASV_16s_4473	0.308798178	0.220002947	3.550670266	0.996581422	NA	NA
ASV_16s_4474	0.976986047	-0.045035143	3.54817174	2.862386812	0.897433987	NA
ASV_16s_4475	0.995553909	0.295938048	3.548096098	2.985059465	0.886380903	NA
ASV_16s_4476	1.11989494	0.027335798	3.548059833	3.485964308	0.836709885	NA
ASV_16s_4477	1.195846427	-0.795805766	3.548048479	2.341472142	0.938554092	NA
ASV_16s_4478	1.022487207	5.899465753	3.548283374	0.272589589	0.999927703	NA
ASV_16s_4479	1.163122387	1.657243399	3.035010427	1.852433349	0.967554422	NA
ASV_16s_4480	1.202856142	-0.926100399	3.547406657	2.00074786	0.959798965	NA
ASV_16s_4481	1.142022604	-0.865639609	3.548199827	1.865168838	0.966924505	NA
ASV_16s_4482	1.211393703	-0.588431925	3.250712407	4.010576774	0.778558366	NA
ASV_16s_4483	1.694424879	-0.010679766	3.546465278	2.423606191	0.932738908	NA
ASV_16s_4484	1.23649244	1.839267438	3.548147849	1.553491482	NA	NA
ASV_16s_4485	1.128799944	1.144892982	3.548128667	3.147627412	0.87101783	NA
ASV_16s_4486	0.420421742	0.366448557	3.549167768	1.190542847	NA	NA
ASV_16s_4487	0.741678478	0.307765239	3.550151288	1.420297094	NA	NA
ASV_16s_4488	1.308881839	0.299920543	3.549729347	3.271706739	NA	NA
ASV_16s_4489	1.399441759	16.03371649	3.55145465	2.049904928	0.957026633	NA
ASV_16s_4490	0.986463558	12.86498428	3.551621254	0.926041244	NA	NA
ASV_16s_4491	1.422997982	1.188549958	3.446469506	1.473487506	0.983213352	NA
ASV_16s_4492	0.936632069	0.454567365	3.549026727	2.73132817	NA	NA
ASV_16s_4493	0.930104639	0.005775005	3.548295214	2.836758591	NA	NA
ASV_16s_4494	1.029966137	-0.224178362	3.548020485	2.160208115	NA	NA
ASV_16s_4495	0.93343792	-0.052879838	3.548183771	2.802340607	NA	NA
ASV_16s_4496	1.001902046	0.317218152	3.548062039	2.837536377	0.899613639	NA
ASV_16s_4497	1.02792775	0.779351557	3.547990851	2.933979271	0.891041752	NA
ASV_16s_4498	1.140439923	-0.640172384	3.548138641	2.316364906	NA	NA
ASV_16s_4499	0.933185419	-0.198400505	3.548681701	2.17716085	NA	NA
ASV_16s_4500	1.162460828	-1.295753704	3.547956463	2.09731747	0.954258111	NA
ASV_16s_4501	1.141199836	-0.634505114	3.27013995	2.457348871	0.930274348	NA
ASV_16s_4502	1.21217365	-0.794926136	3.548194457	2.455419416	NA	NA
ASV_16s_4503	1.037945148	0.564373696	3.547836101	2.82165555	0.900995815	NA
ASV_16s_4504	1.006386117	-0.93153372	3.54880981	1.985800341	NA	NA
ASV_16s_4505	1.057297423	0.188766201	3.548267575	2.261729544	NA	NA
ASV_16s_4506	1.206479127	-0.619531516	2.70517018	2.244558834	0.94507452	NA
ASV_16s_4507	0.978290844	-0.061614817	3.549677878	2.295591293	0.941687486	NA

ASV_16s_4508	1.144363047	-0.127290893	3.546187652	2.610990555	0.918510105	NA
ASV_16s_4509	1.55457887	0.323370191	3.549546971	1.378176317	NA	NA
ASV_16s_4510	1.313822093	0.344166559	3.549574512	2.503031397	NA	NA
ASV_16s_4511	1.422231233	-14.90975477	3.552157344	0.561434356	0.999188999	NA
ASV_16s_4512	1.009416145	0.688686788	3.550620278	1.672294797	NA	NA
ASV_16s_4513	0.988826951	-5.00E-04	3.549114142	2.656737102	0.914839665	NA
ASV_16s_4514	0.919699797	0.408770411	3.548147578	2.557069983	0.92273868	NA
ASV_16s_4515	1.039888675	0.507128626	3.547926581	3.052185197	0.880133052	NA
ASV_16s_4516	1.003298144	0.670940013	3.547512759	2.971164107	0.887656906	NA
ASV_16s_4517	0.809652241	0.627572957	3.547392586	1.522390634	NA	NA
ASV_16s_4518	1.436955162	3.832598804	2.846303009	0.583778869	0.999078245	NA
ASV_16s_4519	1.232589623	2.075070913	2.76642822	3.473474652	0.838026982	NA
ASV_16s_4520	1.352889161	1.113748117	3.549767486	1.587081453	0.979140879	NA
ASV_16s_4521	0.921760946	-0.214221735	3.549241617	2.623050683	NA	NA
ASV_16s_4522	0.926088414	-0.2283263	3.549236986	2.625444233	NA	NA
ASV_16s_4523	0.962351845	-0.361501421	3.549200867	2.547837784	0.92345196	NA
ASV_16s_4524	0.323768261	-0.680784649	3.550107583	0.850697867	NA	NA
ASV_16s_4525	0.94046756	-0.044472231	3.549161309	2.594598072	NA	NA
ASV_16s_4526	0.980667114	0.113980973	3.548430573	2.915384856	0.892717892	NA
ASV_16s_4527	1.019290352	0.123415319	3.548379453	2.983042638	0.886566481	NA
ASV_16s_4528	0.967836948	-0.276120088	3.548052547	2.728606692	0.908922669	NA
ASV_16s_4529	1.219991212	-0.796046579	3.548006564	2.269955642	0.94340192	NA
ASV_16s_4530	1.025904058	-0.077309012	3.55036079	1.116119967	NA	NA
ASV_16s_4531	0.761622821	1.339128081	3.549507369	1.020404304	NA	NA
ASV_16s_4532	1.311594471	1.177996769	3.548770845	1.466400853	NA	NA
ASV_16s_4533	1.144748376	0.346880855	3.550457948	1.226921841	0.99029376	NA
ASV_16s_4534	1.253070572	0.36586527	3.550131029	2.17821035	0.949321746	NA
ASV_16s_4535	1.242625807	0.351824791	3.550642922	2.791042183	0.90363634	NA
ASV_16s_4536	1.532391917	1.188243329	3.54941959	1.413047476	0.985168277	NA
ASV_16s_4537	0.984255593	-0.274786149	3.549206591	2.66474647	0.914189309	NA
ASV_16s_4538	0.983151195	-0.280568029	3.549205738	2.659656852	0.914602848	NA
ASV_16s_4539	0.922788139	-0.268137824	3.549232082	2.605441894	0.918950152	NA
ASV_16s_4540	0.912614793	-0.35962573	3.5492216	2.498431049	NA	NA
ASV_16s_4541	0.196307095	-0.170005243	3.550641862	0.795520045	0.997489404	NA
ASV_16s_4542	0.584411357	0.219969322	3.550625994	1.320307353	NA	NA
ASV_16s_4543	1.182987262	-0.795293716	3.548108567	2.38932978	0.935197596	NA
ASV_16s_4544	1.162009625	-0.647878876	3.547007337	2.629432533	0.917039505	NA
ASV_16s_4545	1.014807231	-0.482668994	3.548352726	2.24775205	0.944865638	NA
ASV_16s_4546	1.216773605	-0.810174517	3.549649319	2.22071861	0.946621066	NA
ASV_16s_4547	0.634348008	0.587110199	3.550425464	1.69264179	0.974882245	NA
ASV_16s_4548	1.067614062	0.300215522	3.551190541	0.622650462	0.998862063	NA
ASV_16s_4549	1.215642922	2.202184577	2.741301823	1.980338018	0.960920585	NA
ASV_16s_4550	0.676049294	0.30478238	3.551511384	0.442658847	NA	NA
ASV_16s_4551	1.286628537	0.349773164	3.549819606	2.392920391	0.93494217	NA
ASV_16s_4552	1.310633	0.344500113	3.549661214	2.426432715	NA	NA
ASV_16s_4553	0.32085143	-0.680729544	3.55011995	0.847790119	NA	NA
ASV_16s_4554	0.959238491	0.089615476	3.548421795	2.944322364	0.890104632	NA
ASV_16s_4555	0.936097539	0.634979066	3.548128109	2.351873562	NA	NA
ASV_16s_4556	1.205895608	-0.795802726	3.548032719	2.329217797	0.939399122	NA
ASV_16s_4557	1.007668457	0.293012478	2.957276309	3.036622439	0.881593804	NA
ASV_16s_4558	1.02819859	-1.261019943	3.548063145	1.701807746	0.974490686	NA
ASV_16s_4559	1.017741222	-0.869017247	3.548589227	1.486036898	0.982789128	NA
ASV_16s_4560	1.154485988	-0.793396581	3.54851775	2.432010088	0.93212917	NA
ASV_16s_4561	1.4127468	0.876950006	3.546999643	1.755523668	NA	NA
ASV_16s_4562	1.025098396	0.220156235	3.548325507	2.711991388	NA	NA
ASV_16s_4563	0.691077823	-0.164914949	3.550460729	1.309609785	0.988180457	NA
ASV_16s_4564	0.764909603	0.253211742	3.550593352	0.432440076	0.999658195	NA
ASV_16s_4565	1.298911694	0.419766189	3.549639181	1.016673263	0.994555071	NA
ASV_16s_4566	1.308936693	0.342984353	3.549317031	2.931887929	0.891230821	NA
ASV_16s_4567	1.166170711	0.693099814	3.548402197	3.188985937	0.866986413	NA
ASV_16s_4568	1.204888636	0.315939157	3.550646902	1.85272818	0.967539916	NA
ASV_16s_4569	1.228404718	0.69397799	3.550612732	1.796984683	0.97021799	NA
ASV_16s_4570	0.964207008	-0.265643295	3.549215662	2.650154998	0.915372411	NA
ASV_16s_4571	0.903795111	-0.271654238	3.549239821	2.58275343	NA	NA
ASV_16s_4572	0.982299964	0.376082292	3.548808302	2.6431683	0.915936206	NA
ASV_16s_4573	0.084048554	0.916771462	3.551704655	0.35109863	0.999829915	NA
ASV_16s_4574	0.936025468	0.104782299	3.548314948	2.953720675	0.889250173	NA

ASV_16s_4575	1.157470294	-0.795560203	3.548077339	2.321781775	0.93990901	NA
ASV_16s_4576	0.953178898	0.927859293	3.54632851	1.996095343	0.960056166	NA
ASV_16s_4577	1.070995888	-0.632140157	3.050134222	3.118741978	0.873804579	NA
ASV_16s_4578	0.898255501	1.550115167	3.54736635	2.000282561	NA	NA
ASV_16s_4579	1.053148934	0.332617321	3.548127735	3.100928662	0.875511109	NA
ASV_16s_4580	1.175540827	-0.794815556	3.548224105	2.430453451	0.932242315	NA
ASV_16s_4581	1.315356987	0.530144871	3.549001688	1.954041341	NA	NA
ASV_16s_4582	1.056401848	-0.157090426	3.549315158	2.131437868	0.952208572	NA
ASV_16s_4583	1.173681395	1.206061022	3.549230159	1.212466119	NA	NA
ASV_16s_4584	0.626127205	0.512299398	3.550542159	1.56816265	0.979855614	NA
ASV_16s_4585	0.917835566	-0.069717925	3.549465798	2.645609464	NA	NA
ASV_16s_4586	0.911210199	-0.868262684	3.548865312	2.143069937	NA	NA
ASV_16s_4587	1.28628598	0.70680107	3.294516098	1.028751459	0.994351306	NA
ASV_16s_4588	1.519878448	0.323367855	3.549560036	1.370879892	NA	NA
ASV_16s_4589	0.921785834	0.035172604	3.54882606	2.200492498	NA	NA
ASV_16s_4590	1.204339772	0.106832118	3.549326006	2.959661167	0.888708648	NA
ASV_16s_4591	1.244930177	0.638321066	3.550617976	2.900656475	0.894037662	NA
ASV_16s_4592	1.428288022	1.188027368	3.549467821	1.395841926	0.985698282	NA
ASV_16s_4593	0.317934599	-0.68067347	3.550132542	0.844856085	NA	NA
ASV_16s_4594	0.939935839	0.128678428	3.54875295	2.76246391	0.906072643	NA
ASV_16s_4595	1.102175503	-0.814183792	3.548232647	1.775638392	0.971209145	NA
ASV_16s_4596	1.005203998	2.484997506	3.546094927	2.019069392	0.958777379	NA
ASV_16s_4597	1.095988519	-1.490539173	3.54764576	2.062836849	0.9562807	NA
ASV_16s_4598	0.997351504	2.001863812	3.546252976	2.055548516	NA	NA
ASV_16s_4599	1.260233194	-1.139753164	2.981739782	2.410467975	0.933686694	NA
ASV_16s_4600	1.054635263	0.210853571	3.548315893	2.351103878	NA	NA
ASV_16s_4601	1.264585989	0.368358562	3.549070213	1.60111046	0.978601395	NA
ASV_16s_4602	1.027296763	0.171580498	3.547226225	2.558473419	NA	NA
ASV_16s_4603	0.787943288	-0.274955942	3.548837065	1.791752291	NA	NA
ASV_16s_4604	0.884063895	-1.033596384	3.548070048	2.8525117	NA	NA
ASV_16s_4605	0.266068389	0.279417522	3.551407144	0.794111468	NA	NA
ASV_16s_4606	1.253729456	0.661900091	3.548811673	2.528247247	0.924955041	NA
ASV_16s_4607	1.28825526	0.693987117	3.550609923	1.821215001	0.969069854	NA
ASV_16s_4608	0.95828857	-0.333012706	3.548452749	2.368682499	NA	NA
ASV_16s_4609	0.904075982	-0.036255393	3.548213735	2.789223111	NA	NA
ASV_16s_4610	0.915565178	1.083469698	3.547764042	2.566047135	NA	NA
ASV_16s_4611	1.086059067	-0.98004268	3.011351332	1.509810283	0.981968084	NA
ASV_16s_4612	1.556521693	2.553136338	3.547961571	0.833201322	NA	NA
ASV_16s_4613	0.454642308	1.337228667	3.549935466	0.854302766	NA	NA
ASV_16s_4614	0.454642308	1.337228667	3.549935466	0.854302766	NA	NA
ASV_16s_4615	0.61914259	0.21211897	3.550603279	1.233641036	0.990131581	NA
ASV_16s_4616	0.968885309	0.078109389	3.549669432	2.82148342	NA	NA
ASV_16s_4617	0.71003134	-0.092246671	3.549583873	1.932503343	NA	NA
ASV_16s_4618	1.203787361	0.36764395	3.550266028	1.138613444	0.992272324	NA
ASV_16s_4619	1.352805631	1.057785489	3.550150194	2.491188625	0.927759107	NA
ASV_16s_4620	1.190549257	0.346432695	3.550467333	1.538232812	0.980956445	NA
ASV_16s_4621	1.262627581	0.930403294	3.550533687	1.649770511	0.976667358	NA
ASV_16s_4622	0.890141866	-0.192253501	3.549260661	2.588253076	NA	NA
ASV_16s_4623	0.930826678	-0.407730689	3.549210525	2.431515	0.932165166	NA
ASV_16s_4624	0.962357701	0.247586606	3.548488577	2.574403842	0.921390956	NA
ASV_16s_4625	0.979606166	0.817631323	3.547910502	2.932918788	NA	NA
ASV_16s_4626	1.001403623	0.153075204	3.548052045	3.332933352	0.852591258	NA
ASV_16s_4627	1.101738001	0.629450828	3.546998511	3.054105452	NA	NA
ASV_16s_4628	0.967401724	-0.852794979	3.548093477	1.959378257	0.962054402	NA
ASV_16s_4629	1.169042134	-0.864581357	3.547971143	2.118639482	0.952982935	NA
ASV_16s_4630	1.145941015	-0.79479429	3.548233317	2.405904725	0.934014326	NA
ASV_16s_4631	0.647792522	0.730083977	3.54769274	2.178686467	NA	NA
ASV_16s_4632	1.0437248	0.223280235	3.548643177	1.724526379	0.973505107	NA
ASV_16s_4633	1.148283785	1.47791374	3.294623638	2.029719536	0.958177145	NA
ASV_16s_4634	1.235258636	2.850358279	3.547946041	0.708978134	NA	NA
ASV_16s_4635	0.919947551	0.253109529	3.550934511	0.347228464	0.999836142	NA
ASV_16s_4636	0.930546382	0.585257118	3.54796372	2.583127013	NA	NA
ASV_16s_4637	1.317843873	-0.606718241	3.548684555	2.260964411	0.943997021	NA
ASV_16s_4638	0.972357953	1.552301755	3.54835412	1.891621633	NA	NA
ASV_16s_4639	1.14980435	-0.09418312	3.550290814	2.07106811	0.955802314	NA
ASV_16s_4640	1.267552983	0.365868812	3.550130356	2.184388451	0.948933776	NA
ASV_16s_4641	1.168452542	0.332210386	3.550624221	1.654916995	0.976457087	NA

ASV_16s_4642	0.929588775	-0.251419022	3.549231599	2.62138288	0.917682915	NA
ASV_16s_4643	0.912677793	-0.274554778	3.5492353	2.590467438	0.92013213	NA
ASV_16s_4644	0.887758359	-0.296804968	3.54924175	2.54610858	NA	NA
ASV_16s_4645	0.892902512	-0.347305533	3.549231698	2.494816422	NA	NA
ASV_16s_4646	0.313559352	-0.680583015	3.550151861	0.840404178	NA	NA
ASV_16s_4647	0.301953816	0.202748146	3.550666472	1.170211685	0.991596672	NA
ASV_16s_4648	0.950650174	-0.314778058	3.549142201	2.387826092	0.935304416	NA
ASV_16s_4649	0.95696161	0.013759171	3.548308621	2.84424441	0.899027287	NA
ASV_16s_4650	0.942911341	0.540554985	3.548643396	2.787583239	0.903932696	NA
ASV_16s_4651	0.957103186	0.084174084	3.548324294	2.933440377	0.891090484	NA
ASV_16s_4652	0.99608231	-0.757880096	3.09239107	2.565532318	0.92208211	NA
ASV_16s_4653	0.905529018	2.033719891	3.546657985	2.199449511	0.947981493	NA
ASV_16s_4654	1.23356848	0.172912053	3.546144959	2.380457824	0.935826577	NA
ASV_16s_4655	1.131833393	-0.79498156	3.548195449	2.379835706	0.935870568	NA
ASV_16s_4656	1.143225049	-0.83397662	3.283329609	2.271365842	0.943308291	NA
ASV_16s_4657	1.026721283	-0.882729879	3.548161768	1.219995174	NA	NA
ASV_16s_4658	1.12279309	-0.962208311	3.091268867	1.97288145	0.961326042	NA
ASV_16s_4659	1.047593679	0.215282569	3.549756723	1.243861891	NA	NA
ASV_16s_4660	1.104993577	-0.950591911	3.545837972	1.291561013	NA	NA
ASV_16s_4661	0.530985629	0.431013193	3.549394817	2.651928806	NA	NA
ASV_16s_4662	0.611193595	0.219967568	3.550623819	1.343072579	0.987251453	NA
ASV_16s_4663	0.975559023	0.665435427	3.549399851	2.468321289	0.929463552	NA
ASV_16s_4664	1.107033607	1.223198436	3.547921403	1.74585299	NA	NA
ASV_16s_4665	1.008457152	0.741454003	3.548719206	2.8667105	NA	NA
ASV_16s_4666	1.283430034	-0.194558067	3.548786155	2.418600773	0.933100781	NA
ASV_16s_4667	1.119142217	-0.247977128	3.547119147	2.9576455	0.888892517	NA
ASV_16s_4668	1.134037267	-0.63559247	3.549157458	2.235608702	0.945657805	NA
ASV_16s_4669	0.967698252	-0.260662634	3.549214998	2.656438841	0.914863839	NA
ASV_16s_4670	0.884156647	-0.281975924	3.549246388	2.554017636	NA	NA
ASV_16s_4671	0.312100937	-0.680553998	3.55015842	0.838906717	NA	NA
ASV_16s_4672	0.890773967	0.272733795	3.549021956	2.551557969	NA	NA
ASV_16s_4673	0.890164151	0.122144802	3.548896435	2.684117956	NA	NA
ASV_16s_4674	1.028724574	-0.584139887	2.897189095	2.652810639	0.915157654	NA
ASV_16s_4675	0.983575111	1.992780108	3.277673125	1.281886445	0.988917691	NA
ASV_16s_4676	1.037666008	-0.546772386	3.548165587	2.251993701	0.944587542	NA
ASV_16s_4677	0.946229212	1.42780575	3.547220849	2.337368847	NA	NA
ASV_16s_4678	0.933096297	-0.55564753	3.547489396	1.757520343	0.972035479	NA
ASV_16s_4679	0.964304367	1.095271243	3.545765554	1.848783386	NA	NA
ASV_16s_4680	1.143291489	-0.795028395	3.54818534	2.38762611	0.935318616	NA
ASV_16s_4681	1.09928762	-0.794299731	3.548336368	2.383291748	0.935625998	NA
ASV_16s_4682	1.128349504	0.714157111	3.549889304	2.264098808	0.943789932	NA
ASV_16s_4683	1.127065159	-0.915539397	3.277902276	2.207668045	0.947457979	NA
ASV_16s_4684	0.974242283	-0.104155189	3.549518806	2.198268278	0.948056512	NA
ASV_16s_4685	0.608315259	0.219967779	3.550624044	1.340670304	0.987319582	NA
ASV_16s_4686	0.677164098	0.596363344	3.550590482	1.325615356	NA	NA
ASV_16s_4687	1.115954627	0.181513132	3.547954597	3.283897081	0.857556792	NA
ASV_16s_4688	0.923532181	1.589468127	3.548950001	1.44115197	0.984277345	NA
ASV_16s_4689	1.111108882	0.312248278	3.549347936	3.230912498	0.862851022	NA
ASV_16s_4690	0.402251597	0.596288877	3.550618224	1.061156821	NA	NA
ASV_16s_4691	0.950878439	0.04694393	3.5489857	2.652980546	0.915143905	NA
ASV_16s_4692	0.962294911	0.049158807	3.548119235	3.056021717	0.879771829	NA
ASV_16s_4693	0.996676134	0.672837394	3.547770028	2.720109434	0.90963171	NA
ASV_16s_4694	1.173304695	-0.795668079	3.548070629	2.338065227	0.938789617	NA
ASV_16s_4695	1.145321243	-0.795356048	3.548118071	2.349656147	0.937986456	NA
ASV_16s_4696	1.137780815	-0.795256564	3.548143881	2.359702632	0.937286049	NA
ASV_16s_4697	1.131370908	-0.795118329	3.548170311	2.367797026	0.936718864	NA
ASV_16s_4698	1.064318567	0.189970211	3.548195089	3.273100952	0.85864161	NA
ASV_16s_4699	1.106674017	-0.794079141	3.548378902	2.393996531	0.934865518	NA
ASV_16s_4700	1.17862609	1.786871277	3.111581644	2.140424444	0.951660838	NA
ASV_16s_4701	0.378046923	-0.268116163	3.550119489	0.935580467	NA	NA
ASV_16s_4702	1.017378218	2.271906273	2.891433737	2.464338883	0.929758358	NA
ASV_16s_4703	1.431872629	2.309776871	3.548169491	0.803054236	NA	NA
ASV_16s_4704	1.426789727	2.846448033	3.544888715	1.546928424	NA	NA
ASV_16s_4705	0.793277394	0.763749631	3.5500451	1.173687285	NA	NA
ASV_16s_4706	1.592315674	1.206533318	3.549117317	1.310860349	NA	NA
ASV_16s_4707	1.185059445	0.919363076	3.550404704	1.951133422	0.962495395	NA
ASV_16s_4708	1.267308847	0.698683492	3.54939362	2.603363778	0.919114672	NA

ASV_16s_4709	1.11733176	0.361635158	3.55023106	1.451916779	0.983927773	NA
ASV_16s_4710	1.385884124	1.124853427	3.549835311	2.374397307	NA	NA
ASV_16s_4711	1.276951479	1.183543591	3.54988939	1.45160438	0.983937983	NA
ASV_16s_4712	0.865541445	-0.238551409	3.549262426	2.555990958	NA	NA
ASV_16s_4713	0.932243426	-0.290502624	3.549223665	2.599567445	0.919414815	NA
ASV_16s_4714	0.309184105	-0.680495224	3.550171723	0.835890934	NA	NA
ASV_16s_4715	0.1662589	-0.16991077	3.550666351	0.714805517	NA	NA
ASV_16s_4716	0.879166738	-0.441579789	3.549309347	2.02691414	NA	NA
ASV_16s_4717	1.008284849	-0.036698968	3.5481868	2.884818091	0.895449066	NA
ASV_16s_4718	0.251885962	-0.54730814	3.550003242	0.676374772	NA	NA
ASV_16s_4719	1.096675677	-0.793222824	3.548559031	2.389910972	0.935156286	NA
ASV_16s_4720	0.993995032	-0.022196334	3.549282407	1.515201811	0.981778702	NA
ASV_16s_4721	1.16664186	-0.412900173	3.547522585	3.015610169	0.883554416	NA
ASV_16s_4722	1.154352214	0.106996982	3.550594977	1.871864924	0.966590588	NA
ASV_16s_4723	0.62135309	-0.516783016	3.550170094	1.149862053	0.992035872	NA
ASV_16s_4724	0.62436068	0.219966784	3.550622818	1.353904059	0.986941493	NA
ASV_16s_4725	0.979348535	-0.11796709	3.548903468	2.29068789	NA	NA
ASV_16s_4726	1.471297859	0.323364414	3.549579357	1.360379937	NA	NA
ASV_16s_4727	1.342693232	0.607718926	3.549332725	2.809914441	0.902012266	NA
ASV_16s_4728	0.30772569	-0.680465453	3.550178468	0.834372468	NA	NA
ASV_16s_4729	0.364020455	0.031537286	3.550606901	2.015808619	0.958960214	NA
ASV_16s_4730	0.881675575	0.021966253	3.548713704	2.62210971	0.917624915	NA
ASV_16s_4731	0.880208737	-0.158510712	3.548242467	2.442132669	NA	NA
ASV_16s_4732	1.000357414	0.217998821	3.547724233	2.544329629	0.923722172	NA
ASV_16s_4733	1.046151786	0.413951412	3.546882983	2.719793873	NA	NA
ASV_16s_4734	1.154379908	-0.795037688	3.548181973	2.397546029	0.934612376	NA
ASV_16s_4735	1.124218962	-0.9309493	3.087937636	2.305970426	0.940985935	NA
ASV_16s_4736	1.099847653	-1.667463247	3.546621025	1.861192655	0.967121901	NA
ASV_16s_4737	0.934071263	0.403608836	3.549581306	1.175155556	NA	NA
ASV_16s_4738	0.94287491	-0.208505221	3.547832801	1.984699394	0.960682361	NA
ASV_16s_4739	0.94939634	0.136292487	3.549101553	3.536217613	0.831374599	NA
ASV_16s_4740	1.363821888	-0.891944529	3.548643737	2.274389997	NA	NA
ASV_16s_4741	0.878732184	2.751151842	3.548295942	0.608035891	NA	NA
ASV_16s_4742	1.142725015	-0.096929459	3.549899834	1.490727736	0.98262893	NA
ASV_16s_4743	1.340337052	0.675871478	3.550649869	2.939337371	NA	NA
ASV_16s_4744	1.166365703	0.693961796	3.550615949	1.770566725	0.971441836	NA
ASV_16s_4745	0.873032418	-0.068769247	3.549312531	2.480585927	NA	NA
ASV_16s_4746	0.91541019	-0.249782631	3.549237866	2.606910964	0.918833753	NA
ASV_16s_4747	0.922115636	0.204858503	3.54911419	2.677054118	0.913185482	NA
ASV_16s_4748	0.957123872	-7.05E-04	3.54812854	2.718416829	0.909772646	NA
ASV_16s_4749	0.914957899	-0.77163653	3.547918387	1.807974566	NA	NA
ASV_16s_4750	1.078459965	-0.793712502	3.548458466	2.375473292	0.936178615	NA
ASV_16s_4751	1.176854602	1.412307271	2.659951061	3.140745099	0.871683981	NA
ASV_16s_4752	1.163939378	0.166121746	3.54884635	3.164397703	0.869388962	NA
ASV_16s_4753	1.703272239	0.271442955	3.549257877	1.366064047	NA	NA
ASV_16s_4754	0.889853082	0.968913127	3.547297384	2.432412926	0.932099874	NA
ASV_16s_4755	1.011220948	-0.996104781	3.547693618	1.582785955	NA	NA
ASV_16s_4756	1.237498472	0.585126223	3.545876425	2.132638452	NA	NA
ASV_16s_4757	1.301814536	1.121777683	3.549185374	1.788174362	0.970629379	NA
ASV_16s_4758	1.083565731	-0.681364447	3.548382545	1.761363821	0.971861329	NA
ASV_16s_4759	1.207034996	0.356383613	3.549993425	2.244167879	0.945100066	NA
ASV_16s_4760	1.229205335	0.693972421	3.550612694	1.797295642	0.97020341	NA
ASV_16s_4761	1.153811581	0.693963763	3.550616642	1.765050201	0.971693719	NA
ASV_16s_4762	1.182434681	0.693968749	3.550615084	1.777528551	0.971122149	NA
ASV_16s_4763	0.857752069	-0.181752663	3.54927891	2.549897996	NA	NA
ASV_16s_4764	0.889169526	-0.211291449	3.549256698	2.58770331	0.920349415	NA
ASV_16s_4765	0.604164016	0.596350426	3.550595389	1.267627234	NA	NA
ASV_16s_4766	0.925243961	0.081585378	3.548227693	2.969602617	0.88779992	NA
ASV_16s_4767	0.900862791	-0.157918381	3.54871749	2.195542418	NA	NA
ASV_16s_4768	0.911093763	0.761685832	3.548033668	2.818074153	NA	NA
ASV_16s_4769	0.90683074	1.406889562	3.547533785	2.472870876	NA	NA
ASV_16s_4770	0.994737431	-0.101685808	3.54944818	2.184409407	0.948932457	NA
ASV_16s_4771	0.819927618	0.636843755	3.363705376	2.117671016	0.953041259	NA
ASV_16s_4772	1.298683397	-1.095203343	3.547180861	2.028275034	NA	NA
ASV_16s_4773	1.861596561	-2.833169555	3.545263369	0.645081649	NA	NA
ASV_16s_4774	0.721422315	0.69867152	3.550600005	1.57348045	NA	NA
ASV_16s_4775	0.258048464	-0.588727103	3.550915867	0.765040109	NA	NA

ASV_16s_4776	1.428707091	0.892895179	3.549396203	3.398467606	0.845859776	NA
ASV_16s_4777	1.255451834	1.143839824	3.550127732	1.500654111	0.982287006	NA
ASV_16s_4778	0.886455113	-0.306726707	3.549240546	2.535425	0.924405987	NA
ASV_16s_4779	0.910617808	0.034176045	3.549157232	2.604387383	0.919033655	NA
ASV_16s_4780	0.892978684	-0.010030115	3.548644074	2.607498655	0.918787167	NA
ASV_16s_4781	1.026641068	-0.293867368	3.547918005	2.32561705	0.939646297	NA
ASV_16s_4782	1.123897467	-0.796093642	3.548111309	1.967735158	0.961604527	NA
ASV_16s_4783	1.01194404	0.344017294	3.317561821	2.436068521	0.931833742	NA
ASV_16s_4784	0.910575359	0.790565773	3.548000895	2.612827288	0.918364193	NA
ASV_16s_4785	0.915515204	1.109273651	3.547684851	2.508326416	NA	NA
ASV_16s_4786	1.171191661	-0.838971337	3.282816492	2.30428474	0.941100165	NA
ASV_16s_4787	1.113526226	-0.794967623	3.5482079	2.365458933	0.93688296	NA
ASV_16s_4788	1.09749569	-1.337841435	3.127398816	2.680684856	0.912888326	NA
ASV_16s_4789	1.068806226	-0.793954757	3.548410942	2.364662849	0.936938784	NA
ASV_16s_4790	1.100602697	-0.922801449	3.547540841	1.899547476	0.965190247	NA
ASV_16s_4791	1.128329863	0.156676622	3.548482357	3.043209505	0.88097642	NA
ASV_16s_4792	1.095633005	-0.793506063	3.548499176	2.389618226	0.935177096	NA
ASV_16s_4793	1.113766687	-1.103586025	3.545600151	1.712972365	NA	NA
ASV_16s_4794	0.769834442	0.119483906	3.548857536	2.290735638	0.942014225	NA
ASV_16s_4795	1.23293847	0.403666024	3.549418701	1.264685428	NA	NA
ASV_16s_4796	0.846969399	0.744258319	3.549973194	1.500151674	NA	NA
ASV_16s_4797	1.808244964	-3.01430594	3.545180359	0.613029261	NA	NA
ASV_16s_4798	1.263755412	0.193816092	3.549016239	4.006977998	NA	NA
ASV_16s_4799	0.592448888	0.219968703	3.550625321	1.327219812	0.987696929	NA
ASV_16s_4800	0.604245342	0.219968028	3.550624365	1.337243593	0.987416379	NA
ASV_16s_4801	0.730627084	-1.408167324	3.549225704	1.589727738	0.979039735	NA
ASV_16s_4802	1.312121611	1.831759999	3.547073878	2.564289098	NA	NA
ASV_16s_4803	0.874905572	1.26888445	3.547451428	1.200634456	NA	NA
ASV_16s_4804	1.485319041	1.468948471	3.54877522	3.353208751	0.850520104	NA
ASV_16s_4805	1.100882057	0.315939089	3.55065254	1.806709705	0.969760124	NA
ASV_16s_4806	1.249200391	0.958800526	3.550507386	1.626987726	0.977585042	NA
ASV_16s_4807	0.859687193	-0.169132457	3.549281493	2.548529258	NA	NA
ASV_16s_4808	0.855858215	-0.300649111	3.549256135	2.506556029	NA	NA
ASV_16s_4809	0.898999022	0.027981349	3.548497325	2.730283445	0.908782458	NA
ASV_16s_4810	0.864236096	0.010758967	3.548434385	2.700204786	NA	NA
ASV_16s_4811	0.912515274	-0.317200272	3.549338924	1.471296287	NA	NA
ASV_16s_4812	1.148239167	-0.795379505	3.548120018	2.355044406	0.937611296	NA
ASV_16s_4813	1.094592769	-0.795487342	3.548218372	2.34945826	0.938000212	NA
ASV_16s_4814	1.046318758	-0.827367252	3.069300931	2.69847271	0.911425754	NA
ASV_16s_4815	0.952928624	-0.702193051	3.54815629	1.781054922	NA	NA
ASV_16s_4816	1.155778258	1.630803857	2.50182693	4.175220525	0.759388781	NA
ASV_16s_4817	1.17674799	0.355442258	3.550578301	3.318080451	0.854101858	NA
ASV_16s_4818	2.065523774	1.253903192	3.549222667	1.390167747	NA	NA
ASV_16s_4819	0.958653307	0.317719398	3.549053117	2.955499774	0.88908811	NA
ASV_16s_4820	1.160527972	0.348818704	3.55052579	1.272614777	0.989157749	NA
ASV_16s_4821	1.512811349	0.768294272	3.549318438	3.233148695	NA	NA
ASV_16s_4822	1.288939254	1.076795675	3.549433993	1.891954761	0.965577515	NA
ASV_16s_4823	0.300433612	-0.68031469	3.550213162	0.826671949	NA	NA
ASV_16s_4824	0.268131839	-0.170156666	3.550605526	0.950893431	NA	NA
ASV_16s_4825	0.883862715	0.020186944	3.548108957	2.982665714	0.886601149	NA
ASV_16s_4826	0.944967341	1.191201031	3.547251301	2.56923117	0.921794296	NA
ASV_16s_4827	0.912643042	-0.24989546	3.547695282	2.38873976	NA	NA
ASV_16s_4828	0.974424225	-0.547697153	3.547219987	2.212727834	0.947134318	NA
ASV_16s_4829	1.026118144	-0.911435053	2.726850524	3.454411957	0.84003027	NA
ASV_16s_4830	0.869061578	-1.400468406	3.545490322	1.314658025	NA	NA
ASV_16s_4831	0.91233396	-2.331793776	3.169530728	0.881969492	0.996514693	NA
ASV_16s_4832	0.974970138	-0.075142231	3.549876571	1.453538514	NA	NA
ASV_16s_4833	1.178377321	-0.043499699	3.54718867	2.913033011	0.892929103	NA
ASV_16s_4834	0.634574784	0.305247208	3.550391242	1.266689396	0.989309471	NA
ASV_16s_4835	0.632537218	-0.601638461	3.550076317	1.308853476	0.988200958	NA
ASV_16s_4836	2.055545399	1.253897231	3.549224398	1.388602877	NA	NA
ASV_16s_4837	0.505276916	0.311322726	3.550608616	1.012727017	NA	NA
ASV_16s_4838	1.249378363	-0.19942771	3.550081985	2.588657608	0.92027443	NA
ASV_16s_4839	1.183626069	0.988157803	3.549443173	1.500899384	0.982278507	NA
ASV_16s_4840	1.159784413	0.121787712	3.273717112	2.307411077	0.94088822	NA
ASV_16s_4841	1.248438101	0.272787268	3.549745752	2.847929792	NA	NA
ASV_16s_4842	1.329462609	0.429340017	3.54834666	3.225848113	0.863353103	NA

ASV_16s_4843	1.176359169	0.693967829	3.550615409	1.77489589	0.971243279	NA
ASV_16s_4844	1.038277527	-0.394872154	3.549170361	2.564625745	0.922152575	NA
ASV_16s_4845	0.298975196	-0.680282894	3.550220301	0.825109557	NA	NA
ASV_16s_4846	0.916858497	-0.397204595	3.548951543	2.281122047	0.942658361	NA
ASV_16s_4847	0.875707526	1.132724417	3.548337286	2.579960908	NA	NA
ASV_16s_4848	0.891182214	0.900039933	3.547793412	2.759441581	NA	NA
ASV_16s_4849	1.171976707	0.19311165	3.548029343	3.595697906	0.824987781	NA
ASV_16s_4850	1.333228286	-2.013079823	3.546292321	2.772311185	NA	NA
ASV_16s_4851	0.918486647	-1.244719772	3.548301522	2.052827825	NA	NA
ASV_16s_4852	0.997351825	-0.076156803	3.549905874	1.629570951	0.977482068	NA
ASV_16s_4853	0.922267395	-0.412950812	3.546933482	1.831006698	NA	NA
ASV_16s_4854	1.406156248	0.810490828	3.544751923	2.063023279	0.956269896	NA
ASV_16s_4855	1.200185214	2.758652576	3.547357021	1.224908543	NA	NA
ASV_16s_4856	0.692652334	1.260761811	3.549542218	1.666054256	NA	NA
ASV_16s_4857	0.753620251	0.455731583	3.549952758	1.795534584	NA	NA
ASV_16s_4858	1.186048769	0.550634155	3.419922726	3.407024967	0.844972956	NA
ASV_16s_4859	0.854823545	-0.351548271	3.549249293	2.445832274	NA	NA
ASV_16s_4860	0.833206788	-0.462556724	3.549258113	2.19327094	NA	NA
ASV_16s_4861	0.376300369	0.099513329	3.550619674	1.944788522	0.962832837	NA
ASV_16s_4862	0.859206195	0.562218112	3.548641814	2.598630511	NA	NA
ASV_16s_4863	0.950309827	0.199047695	3.548704414	2.101040825	0.954036778	NA
ASV_16s_4864	1.019966198	0.133203613	3.54814946	3.315538535	0.854359814	NA
ASV_16s_4865	0.806251537	0.646741768	3.548212376	2.54483463	NA	NA
ASV_16s_4866	0.963465304	-0.015561411	3.548062417	3.075000336	NA	NA
ASV_16s_4867	1.069739019	-0.795830878	3.548103568	2.078184807	0.955386453	NA
ASV_16s_4868	1.059117262	0.14391915	3.3057537	3.026582867	0.882532252	NA
ASV_16s_4869	1.066340569	-0.795055499	3.548196741	2.304419276	0.941091052	NA
ASV_16s_4870	1.071664858	-0.79465599	3.548269571	2.342925321	0.938453492	NA
ASV_16s_4871	1.102147979	-0.925155992	3.547946841	1.54644103	NA	NA
ASV_16s_4872	1.189505898	0.110924492	3.550435577	1.943462714	0.962903136	NA
ASV_16s_4873	1.394635274	2.321467439	3.54654727	1.768030747	0.971557786	NA
ASV_16s_4874	0.985356187	1.341140527	3.549382083	1.167058891	NA	NA
ASV_16s_4875	0.650484646	-0.18800839	3.55026985	1.268197015	0.989270993	NA
ASV_16s_4876	0.80173566	0.447787932	3.549278301	2.941088519	0.890397995	NA
ASV_16s_4877	1.243202536	1.095896788	3.547005397	1.992804282	0.96023756	NA
ASV_16s_4878	1.244303636	1.153636856	3.550088128	1.485747699	0.982798975	NA
ASV_16s_4879	0.296058365	-0.680218393	3.550234788	0.821963507	NA	NA
ASV_16s_4880	0.278604578	0.22001069	3.550680434	0.944460603	NA	NA
ASV_16s_4881	0.890185736	0.189809322	3.548600073	2.789727598	0.903749019	NA
ASV_16s_4882	0.889807073	0.081349972	3.548475578	2.797653673	0.903068751	NA
ASV_16s_4883	0.969689774	0.440052652	3.547389556	2.88323432	0.895589752	NA
ASV_16s_4884	1.041418929	2.227423494	3.547391472	0.763625378	0.997797692	NA
ASV_16s_4885	1.060268324	-2.277986406	2.918626237	2.005247121	0.959549381	NA
ASV_16s_4886	1.083924698	-0.804014348	3.269592391	2.087240434	0.954854277	NA
ASV_16s_4887	0.944350747	0.492136381	3.547022506	2.680276569	NA	NA
ASV_16s_4888	0.702325805	0.652886518	3.549721733	1.774143421	0.971277847	NA
ASV_16s_4889	1.033108184	0.700847837	3.550200228	0.616756002	0.998896829	NA
ASV_16s_4890	0.912240982	0.372585904	3.549607926	3.077280281	0.877762285	NA
ASV_16s_4891	1.270711905	0.735324656	3.550660313	1.854985181	NA	NA
ASV_16s_4892	1.173121784	0.69396382	3.550615583	1.773492855	0.971307715	NA
ASV_16s_4893	0.872166909	-0.414357689	3.549237012	2.353864384	0.937693553	NA
ASV_16s_4894	0.534167847	0.219972898	3.550630665	1.274573716	NA	NA
ASV_16s_4895	0.837145352	-0.187109235	3.548283627	2.272021108	NA	NA
ASV_16s_4896	0.916990736	0.475067532	3.547376739	2.705715196	0.910827059	NA
ASV_16s_4897	1.11249053	-0.79527289	3.548148737	2.331143019	0.939266755	NA
ASV_16s_4898	1.265290371	-2.023766722	3.545622482	2.350675363	NA	NA
ASV_16s_4899	1.178756063	-0.235628092	3.313553814	2.793232734	0.903448448	NA
ASV_16s_4900	0.903320335	-0.086709059	3.549592171	2.220880623	0.946610633	NA
ASV_16s_4901	0.57991089	0.115498195	3.550599656	1.322576226	0.987825585	NA
ASV_16s_4902	0.873531086	0.507705999	3.548877068	2.557316466	0.922719593	NA
ASV_16s_4903	1.173620985	1.334396774	3.548997759	2.536467053	NA	NA
ASV_16s_4904	1.07940253	0.170499463	3.550085167	1.138161004	NA	NA
ASV_16s_4905	0.868671206	-0.478826549	3.549512434	1.926384017	0.963802156	NA
ASV_16s_4906	1.336975027	1.986394682	2.41642452	4.222964896	0.753767921	NA
ASV_16s_4907	0.76345998	0.893559856	2.948359658	3.082635121	0.877253982	NA
ASV_16s_4908	1.206519882	1.355953153	3.548601928	2.287225001	0.942249873	NA
ASV_16s_4909	2.015631896	1.253874241	3.549231496	1.382266635	NA	NA

ASV_16s_4910	0.408044313	0.300955738	3.550887813	0.941420635	NA	NA
ASV_16s_4911	1.190424811	0.35496271	3.55033577	1.504784943	0.982143543	NA
ASV_16s_4912	1.264452241	0.735323292	3.550660734	1.852466979	NA	NA
ASV_16s_4913	0.896614857	-0.34471928	3.549230354	2.502347596	0.926920185	NA
ASV_16s_4914	0.866606661	0.119314133	3.548472574	2.855661812	0.898025857	NA
ASV_16s_4915	0.834089292	0.065089508	3.548402763	2.762599463	NA	NA
ASV_16s_4916	0.864103144	0.456159942	3.54864665	2.534730306	NA	NA
ASV_16s_4917	0.902701806	0.257465151	3.548175763	3.167667347	0.86907046	NA
ASV_16s_4918	1.073180825	-0.795063669	3.548191292	2.309742815	0.940729892	NA
ASV_16s_4919	0.975594171	-1.150154495	3.547792791	1.86760222	0.966803375	NA
ASV_16s_4920	0.95169997	1.59955282	3.546094629	1.216991226	0.990530402	NA
ASV_16s_4921	1.086431882	-0.843939608	3.548702723	2.457459218	0.930266217	NA
ASV_16s_4922	0.571817387	0.219970018	3.550627089	1.309184237	0.988191995	NA
ASV_16s_4923	2.00565352	1.253867555	3.549233315	1.380662838	NA	NA
ASV_16s_4924	1.134557468	0.823237079	3.549729651	1.216249639	0.990547928	NA
ASV_16s_4925	1.126990009	0.264405496	3.54942193	3.794302707	NA	NA
ASV_16s_4926	0.823158162	-0.257229143	3.549280825	2.498782168	NA	NA
ASV_16s_4927	0.291683118	-0.680119301	3.550257055	0.817187519	NA	NA
ASV_16s_4928	0.291683118	-0.680119301	3.550257055	0.817187519	NA	NA
ASV_16s_4929	0.876251549	-0.017894975	3.548295527	2.742819454	0.907731097	NA
ASV_16s_4930	0.930913837	-3.00E-04	3.547144485	2.607727199	NA	NA
ASV_16s_4931	1.034712683	-0.794637808	3.548278169	2.305525795	0.941016076	NA
ASV_16s_4932	1.041937734	-0.794594105	3.548285033	2.315970841	0.940305946	NA
ASV_16s_4933	1.103759519	-0.889822768	3.548012566	2.053848798	0.956799876	NA
ASV_16s_4934	1.016052713	-0.950474615	2.924115131	2.696790093	0.911564582	NA
ASV_16s_4935	0.910572921	-0.830551819	3.548879226	2.091641585	0.954594415	NA
ASV_16s_4936	1.627347723	0.360456419	3.550656087	2.04490778	NA	NA
ASV_16s_4937	0.600769866	0.219968282	3.550624644	1.334292329	NA	NA
ASV_16s_4938	0.98110793	1.821258113	3.547178221	1.853372976	0.967508178	NA
ASV_16s_4939	1.064176732	0.120743091	3.548313027	3.351956159	0.850648359	NA
ASV_16s_4940	0.307964104	-0.603866581	3.550713747	0.898826984	NA	NA
ASV_16s_4941	0.974018489	0.343071448	3.549384937	2.697574823	0.911499848	NA
ASV_16s_4942	0.878173986	-0.357895599	3.549237396	2.462995479	0.92985767	NA
ASV_16s_4943	1.067459486	-0.685051257	3.549152257	1.234912374	0.990100707	NA
ASV_16s_4944	0.906058261	-0.445439991	3.548981151	2.206520225	0.947531258	NA
ASV_16s_4945	0.851145739	0.093035773	3.548401197	2.810344578	0.90197511	NA
ASV_16s_4946	0.942572211	-0.84081297	3.548284513	1.954999815	0.962288945	NA
ASV_16s_4947	1.05348907	2.247501056	3.547372905	0.661223531	0.998616261	NA
ASV_16s_4948	1.054724236	-0.794935895	3.548204849	2.294292309	0.941774987	NA
ASV_16s_4949	0.631752711	0.037069686	3.54902941	1.8810527	NA	NA
ASV_16s_4950	0.718467896	0.054685067	3.547005981	1.136665294	NA	NA
ASV_16s_4951	0.91197025	0.209775244	3.548904306	2.531831981	0.924681071	NA
ASV_16s_4952	1.613336599	0.271446162	3.549277546	1.348539135	NA	NA
ASV_16s_4953	0.871197483	1.142442073	3.546721636	1.84417116	0.967959457	NA
ASV_16s_4954	0.945845078	-0.295485421	3.55110178	0.811503937	NA	NA
ASV_16s_4955	1.086698997	0.290055755	3.549416423	2.464583665	0.929740255	NA
ASV_16s_4956	0.259937328	-0.590074965	3.550909134	0.767495281	NA	NA
ASV_16s_4957	1.275472051	0.612409681	2.630474588	7.225402734	0.405794544	NA
ASV_16s_4958	1.382817577	0.590534912	3.548260303	3.563139299	NA	NA
ASV_16s_4959	1.036656931	0.351507542	3.550653942	2.605648105	0.918933818	NA
ASV_16s_4960	1.044832868	0.510867984	3.550647254	3.290666022	0.856875069	NA
ASV_16s_4961	0.843746493	-0.359903865	3.549254046	2.420615746	0.932955222	NA
ASV_16s_4962	0.228598602	-0.170088027	3.55062271	0.870869313	NA	NA
ASV_16s_4963	0.271742396	0.220012726	3.55068306	0.931842511	NA	NA
ASV_16s_4964	0.870008184	-0.128803039	3.548164058	2.591521594	0.92004919	NA
ASV_16s_4965	0.965693932	1.669885196	3.547175614	2.292204854	0.94191546	NA
ASV_16s_4966	0.86846512	-0.001562788	3.547695752	2.384035259	0.935573322	NA
ASV_16s_4967	0.961490375	1.773554558	3.339524372	2.403521925	0.934185087	NA
ASV_16s_4968	1.014777425	0.490446731	3.547155443	2.842422806	0.899186662	NA
ASV_16s_4969	1.041877036	-0.794353205	3.548332634	2.328603255	0.939441344	NA
ASV_16s_4970	1.021513994	-0.993162682	3.548541435	2.243624742	0.945135545	NA
ASV_16s_4971	0.830998661	-0.98818907	2.972680606	2.467361464	0.92953466	NA
ASV_16s_4972	0.725160131	-0.926702389	3.182946901	1.46459487	0.983510134	NA
ASV_16s_4973	1.061136604	1.205873293	3.54927566	1.17991556	NA	NA
ASV_16s_4974	1.033208997	-1.019733045	3.548437337	1.533683363	NA	NA
ASV_16s_4975	0.854409791	-0.069628164	3.549425	2.609070596	NA	NA
ASV_16s_4976	0.964518596	0.639721806	3.170573225	1.906012808	0.964858583	NA

ASV_16s_4977	1.316172437	0.349349028	3.206608893	2.523492199	0.925317714	NA
ASV_16s_4978	1.044631847	-0.07723692	3.550033413	1.05928156	0.993814729	NA
ASV_16s_4979	0.772397296	0.287032584	3.550616395	2.943547389	NA	NA
ASV_16s_4980	1.438128744	0.796623529	3.186116145	3.7706689	0.805780507	NA
ASV_16s_4981	1.089184534	0.416103127	3.549384012	2.732615241	0.90858731	NA
ASV_16s_4982	1.230191066	1.181348404	3.54994378	1.443277872	0.984208676	NA
ASV_16s_4983	1.125485358	0.693954121	3.550618263	1.752361823	0.972268245	NA
ASV_16s_4984	0.833978954	-0.282551193	3.549270749	2.495911278	NA	NA
ASV_16s_4985	0.833147596	-0.315211749	3.549265107	2.465632364	0.929662671	NA
ASV_16s_4986	0.804444091	-0.332846426	3.549277967	2.41091159	NA	NA
ASV_16s_4987	0.287307871	-0.680012912	3.550279987	0.812342848	NA	NA
ASV_16s_4988	0.287307871	-0.680012912	3.550279987	0.812342848	NA	NA
ASV_16s_4989	0.804809151	-0.684436611	3.549289022	1.14383057	NA	NA
ASV_16s_4991	0.842084016	0.160100756	3.548740324	2.681518775	0.912820009	NA
ASV_16s_4992	0.093063035	-0.25985681	3.551549473	0.310773281	0.999887285	NA
ASV_16s_4993	0.858213411	0.367060902	3.549053962	2.583615289	0.920670257	NA
ASV_16s_4994	0.960581581	-0.122305439	3.548199417	3.076012798	0.877882476	NA
ASV_16s_4995	1.070987499	-0.794776672	3.548245088	2.333789292	0.939084574	NA
ASV_16s_4996	0.925630154	0.603134059	3.545840289	1.551015188	0.980490776	NA
ASV_16s_4997	1.174885768	-0.870620953	3.548851199	1.297753454	0.988499323	NA
ASV_16s_4998	0.986838916	1.761987645	3.547157523	0.890859715	NA	NA
ASV_16s_4999	0.917587591	-1.504349708	3.548007135	1.452099259	0.983921808	NA
ASV_16s_5000	1.079984536	-0.21322566	3.547055053	2.616738566	0.918053069	NA
ASV_16s_5001	1.365186743	0.684897865	3.548979287	2.780047401	NA	NA
ASV_16s_5002	0.96300759	-2.334775785	3.546885872	0.5806102	NA	NA
ASV_16s_5003	1.35579596	0.99434557	3.549370134	2.565302424	0.922099982	NA
ASV_16s_5004	0.994553063	-0.684913574	3.549183019	1.212082681	0.990646031	NA
ASV_16s_5005	0.864538184	-0.500353803	3.549166691	2.307585675	0.940876372	NA
ASV_16s_5006	0.843558887	0.394305812	3.548973365	2.555046349	0.922895296	NA
ASV_16s_5007	0.821661698	0.659070799	3.548422066	2.60132425	NA	NA
ASV_16s_5008	0.932297375	0.1096951	3.548104998	3.203411657	0.865569007	NA
ASV_16s_5009	1.044581509	-0.793830714	3.548441503	2.345909226	0.938246665	NA
ASV_16s_5010	0.903034905	-1.04281156	3.547915635	1.848136216	NA	NA
ASV_16s_5011	0.980382998	-1.46527534	3.095549027	2.206677986	0.94752119	NA
ASV_16s_5012	1.166444023	0.523284762	3.268738251	3.31570345	0.854343083	NA
ASV_16s_5013	1.010375258	-1.02624649	3.373508666	0.574697047	0.999124416	NA
ASV_16s_5014	1.207700593	1.060635571	3.549200732	2.137198701	0.951857828	NA
ASV_16s_5015	0.984363194	-2.24088058	3.546836989	0.815272355	NA	NA
ASV_16s_5016	1.167386122	1.011999169	3.551393653	0.604286445	NA	NA
ASV_16s_5017	1.032358842	1.226276166	3.327287122	1.240640053	0.98996087	NA
ASV_16s_5018	0.560800123	0.219970839	3.550628086	1.299282415	0.988458504	NA
ASV_16s_5019	0.574429839	0.219969814	3.550626858	1.311488656	0.988129434	NA
ASV_16s_5020	1.052141182	-0.094593996	3.549698359	1.541320345	0.980844574	NA
ASV_16s_5021	1.093702404	-0.44494935	3.549255156	1.503432373	0.982190593	NA
ASV_16s_5022	1.142863168	0.315939133	3.550650143	1.825735854	0.96885292	NA
ASV_16s_5023	0.846876396	-0.272750186	3.549266349	2.517766758	0.925753279	NA
ASV_16s_5024	0.808088973	-0.277002751	3.549285759	2.468787766	NA	NA
ASV_16s_5025	0.798974897	-0.011894011	3.549182694	2.552062176	NA	NA
ASV_16s_5026	0.843432751	0.043635233	3.548367493	2.763331102	0.905999126	NA
ASV_16s_5027	0.963506685	-0.639107911	3.548351703	2.231762436	NA	NA
ASV_16s_5028	0.840787307	0.260718961	3.548313683	2.962032593	0.888492162	NA
ASV_16s_5029	0.863645385	0.740105005	3.548213635	2.569669749	0.921760136	NA
ASV_16s_5030	0.852398493	1.179230362	3.547951533	2.393237285	NA	NA
ASV_16s_5031	0.941814882	-0.086775578	3.548107497	3.169494753	0.868892318	NA
ASV_16s_5032	0.88983285	0.063093668	3.548099689	3.081876662	0.87732603	NA
ASV_16s_5033	0.949480173	0.495947844	3.54851238	2.873052567	NA	NA
ASV_16s_5034	1.05922533	-0.795380014	3.548150291	2.251675488	0.94460843	NA
ASV_16s_5035	1.019565072	-0.846822702	3.548106822	2.009024415	0.959339199	NA
ASV_16s_5036	1.034043023	-0.794302732	3.548342999	2.322887364	0.939833338	NA
ASV_16s_5037	0.777271063	1.280443377	3.547290902	2.136233525	0.951916687	NA
ASV_16s_5038	1.068229198	-0.280755032	3.548997529	3.321788062	0.853725308	NA
ASV_16s_5039	1.065349796	-0.438764869	2.868717517	4.320294892	0.742234592	NA
ASV_16s_5040	0.919084487	0.338782465	3.548206009	3.21258447	0.864664737	NA
ASV_16s_5041	0.915649262	0.27053247	3.548145207	2.782024201	NA	NA
ASV_16s_5042	0.989383018	-0.111895161	3.550046265	2.097923727	0.954222111	NA
ASV_16s_5043	0.813972525	-0.179159196	3.549302651	2.497593219	NA	NA
ASV_16s_5044	0.822971349	-0.250063812	3.549282054	2.501831456	0.926959091	NA

ASV_16s_5045	0.866667688	-0.104461129	3.548144025	2.549882419	NA	NA
ASV_16s_5046	0.847837222	0.132149544	3.548265554	2.921480165	NA	NA
ASV_16s_5047	1.096484453	-0.796011839	3.548121221	1.969251966	0.961522562	NA
ASV_16s_5048	0.933028869	0.065100039	3.548071833	3.126935909	NA	NA
ASV_16s_5049	0.935541024	-0.342392973	3.548647504	2.293396915	0.941835263	NA
ASV_16s_5050	0.765674532	-0.002293917	3.54800931	2.080759361	NA	NA
ASV_16s_5051	0.953240357	-0.14049043	3.549380343	1.968168496	NA	NA
ASV_16s_5052	0.911455543	-0.070059334	3.549912376	1.638103388	0.977139986	NA
ASV_16s_5053	0.573095554	0.581736322	3.550550168	1.440299625	0.984304827	NA
ASV_16s_5054	0.892741558	0.420663805	3.55101535	0.652910939	NA	NA
ASV_16s_5055	0.613877734	0.596352411	3.550594671	1.275693754	NA	NA
ASV_16s_5056	1.178685636	0.646339371	3.550618807	2.876088548	0.896223492	NA
ASV_16s_5057	1.126209596	0.356079788	3.550326629	1.474765403	0.983170443	NA
ASV_16s_5058	1.114100541	0.693951909	3.550618938	1.747174358	0.972501198	NA
ASV_16s_5059	1.086416194	0.693948697	3.550620636	1.734353895	0.973072111	NA
ASV_16s_5060	0.251210898	-0.170129813	3.550612222	0.918086194	NA	NA
ASV_16s_5061	0.89478004	-0.741585059	3.548826912	2.175144181	0.949513719	NA
ASV_16s_5062	0.879675352	0.01201324	3.548502399	2.684827882	0.912548673	NA
ASV_16s_5063	0.843503571	-0.090642245	3.548423182	2.481174702	0.928507945	NA
ASV_16s_5064	0.811730284	0.449245605	3.54888762	2.548889968	NA	NA
ASV_16s_5065	0.891984363	-0.021631467	3.548786444	2.087201681	0.954856562	NA
ASV_16s_5066	0.809729991	0.584564265	3.548602694	2.610673965	NA	NA
ASV_16s_5067	0.752370084	0.915531685	3.548004331	2.281841794	NA	NA
ASV_16s_5068	1.02724569	-0.795319356	3.548155092	2.193238875	0.948375293	NA
ASV_16s_5069	1.076060003	2.265929408	3.546651872	0.48278604	0.999507103	NA
ASV_16s_5070	1.004627769	-0.794367195	3.548342662	2.291940088	0.941933264	NA
ASV_16s_5071	1.008873036	-0.435216439	3.547718845	2.427461964	0.932459493	NA
ASV_16s_5072	1.105087528	1.45168851	2.7982314	2.524064306	0.925274124	NA
ASV_16s_5073	0.645642979	0.230055961	3.550079311	0.91269291	0.99611672	NA
ASV_16s_5074	1.334904645	0.271458857	3.549355123	1.287412041	NA	NA
ASV_16s_5075	0.8212688	-1.124065245	3.54798908	1.5190135	NA	NA
ASV_16s_5076	1.044525288	1.848073672	3.547608763	1.413107924	NA	NA
ASV_16s_5077	1.157539863	0.343706648	3.549494071	2.531957028	0.924671506	NA
ASV_16s_5078	0.918325986	-1.794418691	3.5476217	1.550317824	NA	NA
ASV_16s_5079	0.999522835	-0.794209847	3.548375501	2.294130585	0.941785876	NA
ASV_16s_5080	0.969864368	-0.137574093	3.548002162	1.982469297	0.960804271	NA
ASV_16s_5081	0.640667971	-0.296420777	3.549284771	1.391526649	0.985829382	NA
ASV_16s_5082	0.999474046	0.143917064	3.550586362	3.069181809	0.87852941	NA
ASV_16s_5083	0.540241807	0.219972295	3.550630055	1.280290268	0.98895925	NA
ASV_16s_5084	0.55570433	0.219971219	3.550628561	1.294632161	0.988582378	NA
ASV_16s_5085	0.55829249	0.219970858	3.550628318	1.296993769	0.988519572	NA
ASV_16s_5086	0.869946319	0.728914368	3.548874743	2.699318948	0.911355895	NA
ASV_16s_5087	1.219805827	-0.017984906	3.548904543	2.180789514	0.949159969	NA
ASV_16s_5088	1.164194741	0.181943005	3.549536422	3.589761753	0.825628598	NA
ASV_16s_5089	1.195881609	0.408522162	3.550532791	2.397785359	0.93459529	NA
ASV_16s_5090	1.098405286	0.551775178	3.550637228	3.271341965	0.858818066	NA
ASV_16s_5091	0.793847541	-0.321619661	3.549285514	2.411034914	NA	NA
ASV_16s_5092	0.244048429	-0.170116191	3.550615335	0.903563697	0.996237899	NA
ASV_16s_5094	0.827048467	0.008725865	3.548951742	2.489818518	0.927861787	NA
ASV_16s_5095	0.92810077	-0.445635727	3.547522766	2.269655393	0.943421845	NA
ASV_16s_5096	0.971423645	-0.795576859	3.548185977	1.980065663	NA	NA
ASV_16s_5097	0.82198503	-0.339671396	3.548196198	1.96137078	0.961947403	NA
ASV_16s_5098	0.880244074	0.070460918	3.548132449	3.08942522	0.87660822	NA
ASV_16s_5099	0.96863556	-1.390363239	3.54815512	0.867754531	NA	NA
ASV_16s_5100	0.942571758	-0.102448508	3.549077512	2.648647223	NA	NA
ASV_16s_5101	1.040428829	-0.061293414	3.549663245	1.646919592	0.976783367	NA
ASV_16s_5102	0.677072054	-0.117854435	3.548121913	1.350394555	0.987042421	NA
ASV_16s_5103	0.901298462	0.34864861	3.5491874	3.351196354	0.850726137	NA
ASV_16s_5104	0.367988033	0.418920689	3.550283751	0.805400284	NA	NA
ASV_16s_5105	0.281065122	0.418468005	3.550699332	0.719158779	NA	NA
ASV_16s_5106	0.368130491	0.062209404	3.549685536	1.557465776	NA	NA
ASV_16s_5107	0.948715191	1.625575763	3.548279255	1.914039802	NA	NA
ASV_16s_5108	1.226000538	0.344314741	3.549629412	2.394051238	0.93486162	NA
ASV_16s_5109	1.07310574	0.348502818	3.550451385	1.476145925	0.983124015	NA
ASV_16s_5110	1.06252497	0.315939189	3.5506549	1.788595805	0.970609774	NA
ASV_16s_5111	1.183219135	0.315939232	3.550647999	1.843386625	0.967997769	NA
ASV_16s_5112	0.787295049	-0.195600245	3.549313408	2.467551362	NA	NA

ASV_16s_5113	0.277098962	-0.679303973	3.550335349	0.800673495	NA	NA
ASV_16s_5114	0.171898004	-0.169932796	3.550661111	0.73080103	NA	NA
ASV_16s_5115	0.239170468	-0.170108195	3.550617563	0.893432677	0.996369471	NA
ASV_16s_5116	0.799988658	0.088623263	3.548922826	2.550985193	0.923209146	NA
ASV_16s_5117	0.841051178	0.084027859	3.548707915	2.63935706	0.916243018	NA
ASV_16s_5118	0.786052856	-0.097908669	3.548400345	2.424687418	NA	NA
ASV_16s_5119	0.894418998	0.019158528	3.548234599	2.726768196	0.909076293	NA
ASV_16s_5120	0.875985193	1.896181878	3.546922692	2.221268366	0.94658566	NA
ASV_16s_5121	0.852360843	-0.09673098	3.549567103	2.094808171	NA	NA
ASV_16s_5122	0.680390252	0.419186893	3.549655372	1.003346591	0.994774371	NA
ASV_16s_5123	0.535659928	0.219972678	3.550630515	1.275956318	0.989071606	NA
ASV_16s_5124	0.884414249	-0.097926336	3.547166912	1.793046842	NA	NA
ASV_16s_5125	1.034159473	1.952836852	3.548189843	1.571024402	0.979748441	NA
ASV_16s_5126	0.926694438	2.20468085	3.548130027	1.327666478	0.98768451	NA
ASV_16s_5127	1.24374792	2.307263903	3.547290235	2.253121735	0.944513463	NA
ASV_16s_5128	1.895891387	1.253787194	3.549254577	1.362474081	NA	NA
ASV_16s_5129	1.001162053	-1.837238751	3.547912528	0.803592638	NA	NA
ASV_16s_5130	0.589144456	0.73498038	3.550758403	1.463760502	NA	NA
ASV_16s_5131	0.817513995	-0.216080285	3.549291456	2.504382425	0.926766703	NA
ASV_16s_5132	0.787341016	-0.247036884	3.54930237	2.459488913	NA	NA
ASV_16s_5133	0.772126546	-0.684336749	3.549312536	1.130447946	NA	NA
ASV_16s_5134	0.818893386	-0.003244483	3.54831282	2.702338	0.911106462	NA
ASV_16s_5135	0.916101913	-1.493451918	3.548010745	1.243384501	NA	NA
ASV_16s_5136	0.996458236	-0.794110849	3.548387853	2.29336468	0.941837432	NA
ASV_16s_5137	0.966565985	-0.793812056	3.548452676	2.27331203	0.943178945	NA
ASV_16s_5138	0.933810702	-0.199577756	3.549256523	1.922252236	NA	NA
ASV_16s_5139	0.723566361	0.484813889	3.548149754	1.780088168	0.971004102	NA
ASV_16s_5140	0.824130851	0.419243009	3.549525375	1.06523293	NA	NA
ASV_16s_5141	0.893996995	-1.159023475	3.548288821	1.994392264	NA	NA
ASV_16s_5142	1.012568049	0.175755733	3.548433801	3.378768973	NA	NA
ASV_16s_5143	1.096215016	0.671060176	3.548809703	2.445221845	0.931165122	NA
ASV_16s_5144	1.07937268	0.315939257	3.550653845	1.796599908	0.970236024	NA
ASV_16s_5145	0.586043696	0.734977144	3.55075937	1.461075415	NA	NA
ASV_16s_5146	1.117711325	0.69395789	3.550618723	1.748818611	0.972427481	NA
ASV_16s_5147	0.217053218	-0.170062692	3.550628909	0.845064356	NA	NA
ASV_16s_5148	0.785027583	-0.307086039	3.549274034	2.630397919	NA	NA
ASV_16s_5149	0.783042267	-0.340934977	3.548259897	1.671094542	NA	NA
ASV_16s_5150	1.060896163	-0.795930612	3.548152528	1.932883796	0.963461442	NA
ASV_16s_5151	0.822563297	0.677803126	3.548269184	2.707082579	NA	NA
ASV_16s_5152	1.012286592	-0.795185742	3.5481828	2.206304847	0.947545003	NA
ASV_16s_5153	1.002921818	-0.794673463	3.548280884	2.2684368	0.943502674	NA
ASV_16s_5154	0.98810562	-0.794597017	3.548300563	2.258400502	0.944166126	NA
ASV_16s_5155	0.985963186	-0.794371305	3.548338645	2.269724984	0.943417227	NA
ASV_16s_5156	0.975518844	-0.794265303	3.548360508	2.264110799	0.943789139	NA
ASV_16s_5157	0.912950641	-1.104817349	3.548052734	1.868150029	NA	NA
ASV_16s_5158	0.795608812	-1.364256661	3.546777519	1.65530653	0.976441128	NA
ASV_16s_5159	0.861586484	-0.069752851	3.550104646	1.619064429	0.977899168	NA
ASV_16s_5160	1.079159474	-0.363381095	3.285225224	3.100529955	0.8755492	NA
ASV_16s_5161	0.752232646	0.686934926	3.548373838	2.437235173	NA	NA
ASV_16s_5162	0.964824499	0.214581673	3.547338139	2.746367688	0.907432527	NA
ASV_16s_5163	0.977973855	-1.491025725	3.546715914	1.653130791	NA	NA
ASV_16s_5164	0.582538582	0.394212299	3.550745011	0.940590297	NA	NA
ASV_16s_5165	1.875934636	1.25377285	3.54925871	1.359054285	NA	NA
ASV_16s_5166	1.087082471	0.379100315	3.550450102	0.886559482	0.996457007	NA
ASV_16s_5167	1.203021629	0.082139073	3.549546137	3.030868257	0.88213205	NA
ASV_16s_5168	1.009837102	0.315939172	3.55065843	1.762690048	0.971801094	NA
ASV_16s_5169	1.003497188	0.383957041	3.550654308	2.912376202	0.892988057	NA
ASV_16s_5170	0.582942935	0.734973861	3.550760346	1.458376126	NA	NA
ASV_16s_5171	1.039954013	0.693937573	3.550623691	1.712084062	0.97404754	NA
ASV_16s_5172	0.783313067	-0.306313764	3.549294061	2.413828091	NA	NA
ASV_16s_5173	0.815296804	-0.332315102	3.549272094	2.424668573	0.932661978	NA
ASV_16s_5174	0.775790776	-0.356337226	3.549292003	2.343413135	NA	NA
ASV_16s_5175	0.276730402	0.001292075	3.550614383	1.861837192	NA	NA
ASV_16s_5176	0.805276011	0.025838624	3.548971241	2.483412824	0.928340908	NA
ASV_16s_5177	0.840427714	0.069326508	3.548439575	2.732390683	0.908606111	NA
ASV_16s_5178	1.014288296	-0.795064647	3.548212491	2.241753416	0.945257697	NA
ASV_16s_5179	0.970000355	-0.794052317	3.548403481	2.268377769	0.943506588	NA

ASV_16s_5180	0.861538455	-0.718637681	3.548171752	1.72833558	0.973337753	NA
ASV_16s_5181	0.982371373	-0.794053057	3.548401705	2.281229572	0.942651177	NA
ASV_16s_5182	0.838885586	-0.084474743	3.549633575	2.16484777	0.950155584	NA
ASV_16s_5183	0.87374376	-0.083612316	3.549657128	1.883697501	NA	NA
ASV_16s_5184	1.070034769	0.689994414	3.550622056	1.737467255	0.972934099	NA
ASV_16s_5185	0.436158003	-0.051729201	3.550933867	0.799166146	NA	NA
ASV_16s_5186	0.481494931	0.720505078	3.550431283	1.692586008	NA	NA
ASV_16s_5187	1.129926678	0.065760659	3.549669351	2.260625933	0.944019361	NA
ASV_16s_5188	0.889978089	-0.117808474	3.549511335	2.385822254	0.93544663	NA
ASV_16s_5189	0.972290356	0.481483964	3.550654229	3.214593967	0.864466328	NA
ASV_16s_5190	1.177119707	0.693968679	3.55061537	1.775176673	0.971230374	NA
ASV_16s_5191	0.770521469	-0.236410243	3.549314277	2.441951831	NA	NA
ASV_16s_5192	0.820287844	-0.263261613	3.549281511	2.491999223	0.927698325	NA
ASV_16s_5193	0.805715181	-0.349006	3.549275392	2.391082565	0.93507297	NA
ASV_16s_5194	0.852390214	-0.3935634	3.549247433	2.373177633	0.936340422	NA
ASV_16s_5195	0.813240378	-0.387713118	3.549268344	2.338031188	0.938791968	NA
ASV_16s_5196	0.264153447	-0.047761249	3.550618383	1.583222667	NA	NA
ASV_16s_5197	0.315217827	0.318174919	3.550658863	1.637565673	0.977161633	NA
ASV_16s_5198	0.861891269	0.631592037	3.548293942	2.681977976	NA	NA
ASV_16s_5199	0.817868828	0.185059788	3.548272504	2.914846173	0.892766285	NA
ASV_16s_5200	0.809147702	0.190195478	3.548263908	2.915860501	0.892675154	NA
ASV_16s_5201	0.930327581	0.746529382	3.547580627	2.932514125	0.891174224	NA
ASV_16s_5202	0.967877761	-0.794934222	3.548234673	2.183839227	0.948968328	NA
ASV_16s_5203	0.819813027	1.847095578	3.54692015	2.081083584	0.955216467	NA
ASV_16s_5204	0.913888032	-0.220776865	3.548049731	1.73767919	0.972924689	NA
ASV_16s_5205	0.693435601	2.486875819	3.548027103	0.537064125	NA	NA
ASV_16s_5206	1.019461055	0.042004539	3.549452747	3.492299513	0.836040436	NA
ASV_16s_5207	0.873698831	-0.138773314	3.547592608	1.355993914	NA	NA
ASV_16s_5208	0.460944948	0.370945983	3.551675232	0.54124495	NA	NA
ASV_16s_5209	0.888580866	0.142864034	3.54923203	2.289149927	NA	NA
ASV_16s_5210	0.587168235	-0.512363823	3.549662429	1.7677147	NA	NA
ASV_16s_5211	1.158603145	0.793343724	3.548365483	2.051587583	NA	NA
ASV_16s_5212	1.001779637	0.335612239	3.550624357	1.534270273	0.981099451	NA
ASV_16s_5213	1.116007803	1.187285156	3.549642743	1.32919535	0.987641943	NA
ASV_16s_5214	0.798124215	-0.28461717	3.549289695	2.450966691	0.930743842	NA
ASV_16s_5215	0.770809244	-0.282241532	3.54930614	2.418470059	NA	NA
ASV_16s_5216	0.145084417	-0.16981764	3.550689688	0.650382117	NA	NA
ASV_16s_5217	0.256270128	-0.17013919	3.55061013	0.928081556	NA	NA
ASV_16s_5218	0.848251723	-0.170655282	3.548901325	2.291111187	0.941988987	NA
ASV_16s_5219	0.865218053	0.051917298	3.548287369	2.828258887	0.900422121	NA
ASV_16s_5220	0.353582745	1.627125435	3.550310058	0.648284854	NA	NA
ASV_16s_5221	0.880401683	-0.082447324	3.547691458	1.671065313	0.975790192	NA
ASV_16s_5222	0.938816063	-0.864334439	3.054435526	3.064777592	0.878945779	NA
ASV_16s_5223	1.010857493	1.014866804	3.547388379	2.298347848	0.941501581	NA
ASV_16s_5224	1.053327177	-0.127232981	3.287032513	3.561631961	0.828655038	NA
ASV_16s_5225	1.00149433	0.318247855	3.546324187	2.07762468	0.95541926	NA
ASV_16s_5226	1.018760839	-0.460995562	3.549500596	1.587438117	0.979127264	NA
ASV_16s_5227	0.614793367	-0.086349989	3.550663358	0.586796583	0.999062548	NA
ASV_16s_5228	1.059441048	0.946065627	3.547119979	2.440856856	NA	NA
ASV_16s_5229	0.941172714	0.320772025	3.549764383	1.442412215	0.984236659	NA
ASV_16s_5230	0.942523727	1.057362636	3.550146445	1.899186301	0.965208723	NA
ASV_16s_5231	0.626444556	-0.079346729	3.550601707	1.515188555	NA	NA
ASV_16s_5232	1.169972862	0.149704124	3.548144416	2.417874617	NA	NA
ASV_16s_5233	1.035871056	0.363474495	3.549728088	1.318847761	0.987928288	NA
ASV_16s_5234	0.966262383	1.17873909	3.548862536	2.284209378	0.942451902	NA
ASV_16s_5235	0.22245837	-0.051904159	3.550635082	1.404162983	NA	NA
ASV_16s_5236	0.771661191	0.124704585	3.548325534	2.799067954	NA	NA
ASV_16s_5237	0.798019969	0.131330763	3.548428727	2.532533586	0.924627394	NA
ASV_16s_5238	0.827533676	1.265531239	3.547960079	2.488166439	0.927985505	NA
ASV_16s_5239	0.900170346	-1.171486059	3.548197799	1.807558109	0.969719993	NA
ASV_16s_5240	1.019801231	-0.886525486	3.548331291	1.561624165	0.980099224	NA
ASV_16s_5241	0.908563604	1.801187163	3.546666152	2.0284054	NA	NA
ASV_16s_5242	0.902769835	-1.047407606	3.547635758	1.659298792	NA	NA
ASV_16s_5243	0.876453978	-1.478428319	3.547915001	1.813944545	NA	NA
ASV_16s_5244	0.813859444	-0.309832661	3.548574286	1.667398266	NA	NA
ASV_16s_5245	0.957375312	0.393669802	3.548189727	2.660546199	0.914530654	NA
ASV_16s_5246	0.86761228	0.62054137	3.548538559	1.780595099	NA	NA

ASV_16s_5247	0.859379648	-0.094090094	3.54955485	2.114927852		0.953206252	NA
ASV_16s_5248	0.43573776	-0.12039286	3.549829622	1.345071695	NA		NA
ASV_16s_5249	0.618929329	0.027437582	3.548089527	1.742917154	NA		NA
ASV_16s_5250	0.805627027	0.663096694	3.549750708	1.534783557		0.981080963	NA
ASV_16s_5251	1.193346343	0.44375112	3.550853685	0.650206331	NA		NA
ASV_16s_5252	0.887804664	-0.33672534	3.547050637	1.872671939	NA		NA
ASV_16s_5253	0.311810898	-0.357672821	3.550338918	0.897748706	NA		NA
ASV_16s_5254	0.380969195	0.075503822	3.54966933	1.59639516	NA		NA
ASV_16s_5255	0.270764306	0.417459473	3.550757287	0.70697349	NA		NA
ASV_16s_5256	0.401362359	-0.027705228	3.549548806	1.720014061	NA		NA
ASV_16s_5257	0.524388479	0.219973504	3.550631679	1.265132027		0.989349128	NA
ASV_16s_5258	0.932266926	2.469005235	3.548481735	0.650999642	NA		NA
ASV_16s_5259	1.836021133	1.253744532	3.549267244	1.352104257	NA		NA
ASV_16s_5260	1.027771107	0.315939237	3.550657189	1.771641731		0.971392604	NA
ASV_16s_5261	1.069565031	1.071394961	3.55033092	1.475428764		0.983148143	NA
ASV_16s_5262	0.758863187	-0.293598611	3.549311171	2.394099751	NA		NA
ASV_16s_5263	0.803038049	-0.385005832	3.549274183	2.330531715		0.9393088	NA
ASV_16s_5264	0.836695798	-0.401940465	3.549255219	2.338773746		0.938740674	NA
ASV_16s_5265	0.338023018	0.270723743	3.550615977	1.8219159	NA		NA
ASV_16s_5266	0.761449972	-0.030012634	3.548427451	2.529163482	NA		NA
ASV_16s_5267	0.758018356	-0.167512538	3.548393572	2.204810993	NA		NA
ASV_16s_5268	0.843859548	0.39652612	3.547687922	2.546895155		0.92352461	NA
ASV_16s_5269	0.825602067	0.837238672	3.547089339	2.119565669		0.952927121	NA
ASV_16s_5270	0.795287913	0.897833941	3.547426135	2.540062302	NA		NA
ASV_16s_5271	0.936196314	-0.906748913	3.548261675	2.014184672		0.959051106	NA
ASV_16s_5272	0.961591863	-0.79451302	3.548295575	2.220444855		0.946638692	NA
ASV_16s_5273	0.913009607	0.162710626	3.54826512	2.256769205		0.944273583	NA
ASV_16s_5274	0.813519325	-1.226334884	3.548499393	1.966240229		0.961685217	NA
ASV_16s_5275	0.788240787	-0.967767137	3.548643599	2.271519261		0.9432981	NA
ASV_16s_5276	1.153553086	0.006320804	3.548685532	2.237251768		0.945550966	NA
ASV_16s_5277	0.881735867	2.22565338	3.54864487	1.376482113		0.986280729	NA
ASV_16s_5278	1.51222783	-2.504382691	3.545929853	0.630575557	NA		NA
ASV_16s_5279	0.915260728	-1.51746	3.548060705	0.744650805	NA		NA
ASV_16s_5280	0.687423948	-0.390059434	3.547083022	1.805523999		0.96981616	NA
ASV_16s_5281	0.846793842	-0.069686412	3.549429832	2.6177546	NA		NA
ASV_16s_5282	0.369663908	0.294275534	3.551016797	0.907963297	NA		NA
ASV_16s_5283	0.941816214	-0.10241637	3.550184828	2.010523574		0.959255616	NA
ASV_16s_5284	1.107733115	0.519144079	3.549363314	2.862404388		0.897432438	NA
ASV_16s_5285	0.997573303	0.807478069	3.549579198	2.665601112		0.914119777	NA
ASV_16s_5286	1.158023207	1.151733013	3.549949477	1.893020718	NA		NA
ASV_16s_5287	0.998676277	0.315939189	3.550659226	1.757007943		0.97205865	NA
ASV_16s_5288	0.755876282	-0.354432143	3.549304477	2.320643419	NA		NA
ASV_16s_5289	0.330237277	0.219998321	3.550664183	1.030469795	NA		NA
ASV_16s_5290	0.814309821	-0.473301085	3.54924026	2.262414186		0.943901283	NA
ASV_16s_5291	0.791616853	0.092093933	3.548805479	2.558799664		0.922604694	NA
ASV_16s_5292	0.816296234	0.488127403	3.548134105	2.66446453		0.914212241	NA
ASV_16s_5293	0.927144198	-0.177896265	3.54742005	2.779668738		0.904609266	NA
ASV_16s_5294	0.666166966	1.071517371	3.547266313	1.157587127		0.991870885	NA
ASV_16s_5295	0.912507675	0.352263127	3.548716447	2.766962433		0.905690994	NA
ASV_16s_5296	0.917338314	-0.145507224	3.547563325	2.780195326		0.904564317	NA
ASV_16s_5297	0.944383722	-0.790230015	3.54906157	2.25112359		0.944644648	NA
ASV_16s_5298	0.87939288	-0.109132875	3.549526034	2.0221369		0.958604978	NA
ASV_16s_5299	0.895611389	3.448450213	3.546064212	0.26997473	NA		NA
ASV_16s_5300	0.879137761	-0.065600097	3.550174979	1.272828395	NA		NA
ASV_16s_5301	0.638642741	0.647295614	3.547868386	1.736056918		0.972996668	NA
ASV_16s_5302	1.066499171	0.426947542	3.549992595	0.860616822	NA		NA
ASV_16s_5303	0.943261611	-0.243736233	3.548033953	1.485787223		0.98279763	NA
ASV_16s_5304	0.798021841	-0.069631829	3.549485083	2.535807531		0.924376672	NA
ASV_16s_5305	1.047082281	0.431513426	3.550645843	2.295915705		0.941665623	NA
ASV_16s_5306	1.022713487	0.363481594	3.549713395	1.309188522		0.988191879	NA
ASV_16s_5307	0.3061064	0.348844882	3.550659684	1.679212967	NA		NA
ASV_16s_5308	0.863671635	-0.650905281	3.548716274	2.196219331		0.948186505	NA
ASV_16s_5309	0.942377906	-0.325543624	3.547973891	2.238264139	NA		NA
ASV_16s_5310	0.833004211	0.61730031	3.548188241	2.827979287		0.900446442	NA
ASV_16s_5311	0.936395592	-0.793430845	3.548535922	2.252267293		0.94456958	NA
ASV_16s_5312	0.854075476	-0.093991104	3.549531839	2.073721019		0.955647536	NA
ASV_16s_5313	0.807553359	-0.088874732	3.549731712	2.091897053		0.954579306	NA

ASV_16s_5314	1.17947161	-1.270831096	3.548531461	1.563879255	NA	NA
ASV_16s_5315	1.068909911	0.768409285	3.449804765	1.810812584	0.96956577	NA
ASV_16s_5316	0.937688994	-0.098284899	3.547586287	2.131587015	0.952199509	NA
ASV_16s_5317	1.495866705	0.271450948	3.549306797	1.324106617	NA	NA
ASV_16s_5318	1.135882628	-1.669031767	3.546960669	1.022902153	0.994450584	NA
ASV_16s_5319	1.185160435	0.403658778	3.549439278	1.251877962	NA	NA
ASV_16s_5320	0.568190265	-0.178768014	3.54928347	2.014205398	NA	NA
ASV_16s_5321	0.766502167	-0.069646384	3.549590064	2.418479016	0.933109572	NA
ASV_16s_5322	0.52871426	-0.2645803	3.550485524	0.697406842	0.998355582	NA
ASV_16s_5323	0.671048407	1.674937016	3.549139547	1.303614218	NA	NA
ASV_16s_5324	1.015112411	0.386034203	3.550598602	1.802828694	0.96994332	NA
ASV_16s_5325	1.059569028	0.35175174	3.549880884	2.249057534	0.944780124	NA
ASV_16s_5326	0.931729915	0.416508748	3.550658055	3.019686336	0.883175129	NA
ASV_16s_5327	1.070990521	0.693947667	3.550621622	1.727050732	0.973394269	NA
ASV_16s_5328	0.770772485	-0.232740421	3.549314849	2.443227545	0.931311072	NA
ASV_16s_5329	0.769862957	-0.415909004	3.549293286	2.229426495	0.946058823	NA
ASV_16s_5330	0.262514806	-0.676769893	3.55041863	0.783000644	NA	NA
ASV_16s_5331	0.475991151	0.219977934	3.550637304	1.215930615	NA	NA
ASV_16s_5333	0.786369969	0.100322046	3.549170186	2.495168315	0.927460456	NA
ASV_16s_5334	0.863604783	-0.67655598	3.548909176	2.208897462	0.947379432	NA
ASV_16s_5335	0.768800231	0.063871946	3.548765428	2.546630931	NA	NA
ASV_16s_5336	0.768680185	0.024503825	3.548306071	2.700879207	NA	NA
ASV_16s_5337	0.751359732	-0.340648454	3.548277872	1.679043038	NA	NA
ASV_16s_5338	0.972211019	-1.860590875	3.547264378	1.471540008	0.983278619	NA
ASV_16s_5339	0.971881906	-0.794633532	3.548292089	2.232916941	0.945832599	NA
ASV_16s_5340	0.922569917	-0.793803187	3.548468514	2.22853383	0.9461166	NA
ASV_16s_5341	0.845599014	-0.88900481	3.547964919	1.777648518	0.971116622	NA
ASV_16s_5342	0.913489569	-0.897547973	3.54875643	2.283489865	0.942500052	NA
ASV_16s_5343	0.835715368	1.933881191	3.122542314	2.035439318	0.957852845	NA
ASV_16s_5344	0.919267015	0.296852199	3.549905116	1.790603007	0.970516299	NA
ASV_16s_5345	0.834710131	-0.018547575	3.548206861	1.987632572	NA	NA
ASV_16s_5346	1.017540919	0.531725789	3.550058231	0.970891876	0.995284523	NA
ASV_16s_5347	0.942227131	-1.579933085	3.547772044	1.225018679	0.990339393	NA
ASV_16s_5348	0.898495729	0.597885354	3.547433997	1.922726121	NA	NA
ASV_16s_5349	1.287462522	-3.270270516	3.545489493	0.426567697	NA	NA
ASV_16s_5350	1.023633324	-0.37022418	3.535590006	3.55122423	0.829770465	NA
ASV_16s_5351	1.79610763	1.253707646	3.549276156	1.345000316	NA	NA
ASV_16s_5352	0.721792005	1.251520114	3.549882368	1.050887198	NA	NA
ASV_16s_5353	1.023706577	0.634865471	3.550385309	1.230483571	0.990208	NA
ASV_16s_5354	0.961683962	0.574876015	3.550642207	3.033490717	0.881886869	NA
ASV_16s_5355	0.951227645	0.315939081	3.550662816	1.73222078	0.973166437	NA
ASV_16s_5356	0.769219965	-0.337699455	3.549297934	2.360615063	0.937222242	NA
ASV_16s_5357	0.740604544	-0.367111791	3.549313393	2.281443001	NA	NA
ASV_16s_5358	0.261056391	-0.676301945	3.550426594	0.781156079	NA	NA
ASV_16s_5359	0.261056391	-0.676301945	3.550426594	0.781156079	NA	NA
ASV_16s_5360	0.488674588	0.596319242	3.550606147	1.15978872	0.991823475	NA
ASV_16s_5361	0.813966939	0.059386052	3.54870331	2.603788445	0.919081065	NA
ASV_16s_5362	0.801668066	0.008818118	3.548305803	2.74170012	0.907825194	NA
ASV_16s_5363	0.798078765	1.3169213	3.548065108	2.416642514	0.933242094	NA
ASV_16s_5364	0.941339011	-0.795233755	3.548212839	2.072087145	0.955742895	NA
ASV_16s_5365	0.998865914	-0.987335755	3.547838546	1.867855358	0.96679076	NA
ASV_16s_5366	0.842098031	0.407793843	3.548483416	2.829193187	0.900340832	NA
ASV_16s_5367	0.929214318	-0.794241332	3.548377469	2.212983221	0.947117954	NA
ASV_16s_5368	0.869932803	0.103257824	2.197174655	3.201738305	0.865733719	NA
ASV_16s_5369	0.857840933	1.425786153	3.547659667	1.913037077	0.964496269	NA
ASV_16s_5370	0.841179002	-0.922567821	3.548913225	2.081561526	NA	NA
ASV_16s_5371	0.615248143	-0.301874118	3.550353005	0.863929469	0.996735485	NA
ASV_16s_5372	0.99961059	0.372916263	3.545259599	1.944931715	NA	NA
ASV_16s_5373	0.81891362	2.423294162	3.548869289	0.694119533	NA	NA
ASV_16s_5374	0.717689564	0.153523397	3.548187545	2.466121988	NA	NA
ASV_16s_5375	0.533196769	0.223822329	3.55066002	1.098522901	0.993078913	NA
ASV_16s_5376	0.754128512	0.929045075	3.11981654	2.135204332	NA	NA
ASV_16s_5377	1.026671516	0.314436818	3.550656204	1.652795311	0.976543906	NA
ASV_16s_5378	1.001477807	0.334884148	3.550673877	1.263318568	0.989395193	NA
ASV_16s_5379	1.017832258	0.315939094	3.550657872	1.766669517	0.971619913	NA
ASV_16s_5380	1.129008542	1.187184586	3.549666359	1.340783756	0.98731637	NA
ASV_16s_5381	0.784187147	-0.245747049	3.549304463	2.455898234	0.930381198	NA

ASV_16s_5382	0.808597831	-0.924087741	3.54899514	1.921675096	NA	NA
ASV_16s_5383	0.741419826	0.411890101	3.549107953	2.464998998	NA	NA
ASV_16s_5384	0.715242951	0.378076831	3.54822504	2.161572177	NA	NA
ASV_16s_5385	0.95006755	-0.795318169	3.548200963	2.059488086	0.956474524	NA
ASV_16s_5386	0.845718382	0.310189376	3.548384935	2.950985799	0.889499106	NA
ASV_16s_5387	0.895451661	-0.792176672	3.548800586	2.221823189	0.946549915	NA
ASV_16s_5388	0.903448373	-0.581107579	3.289525037	2.766152055	0.905759797	NA
ASV_16s_5389	1.122282661	2.609227514	3.547680736	0.658624019	0.998633846	NA
ASV_16s_5390	0.92619213	2.416125768	2.861064605	1.564375695	0.979996921	NA
ASV_16s_5391	0.979260304	1.223348346	3.548835088	1.367534218	0.986544972	NA
ASV_16s_5392	0.850413694	0.354401863	3.551441014	0.472770102	NA	NA
ASV_16s_5393	0.806216389	-1.061732671	3.547418914	1.485748026	0.982798964	NA
ASV_16s_5394	0.774287728	-0.069556477	3.549472891	2.523068796	0.925349967	NA
ASV_16s_5395	1.776150878	1.253691076	3.549280762	1.341389211	NA	NA
ASV_16s_5396	1.027417071	-0.024985565	3.550388974	1.953988648	0.962342997	NA
ASV_16s_5397	0.933629141	0.693909967	3.550631821	1.657154333	0.976365334	NA
ASV_16s_5398	0.751899138	-0.270971648	3.549320344	2.401618484	0.934321337	NA
ASV_16s_5399	0.728774804	-0.277911204	3.549333972	2.366296068	NA	NA
ASV_16s_5400	0.750391477	-0.314878316	3.549312815	2.362811097	0.937068538	NA
ASV_16s_5401	0.762724916	-0.356547308	3.549300008	2.326346291	0.93959628	NA
ASV_16s_5402	0.25813956	-0.675054487	3.550442026	0.777405979	NA	NA
ASV_16s_5403	0.813651685	0.260077218	3.549171351	2.488899688	0.927930607	NA
ASV_16s_5404	0.791815799	0.209334493	3.549104328	2.157947743	0.950583309	NA
ASV_16s_5405	0.782091211	0.051366968	3.549047592	2.63378674	0.916690498	NA
ASV_16s_5406	0.758472778	0.275631899	3.549141246	2.061592806	0.956352758	NA
ASV_16s_5407	0.759890563	0.593304796	3.548164058	2.296726783	NA	NA
ASV_16s_5408	0.958873786	-0.795504706	3.548193881	1.989188158	0.960436352	NA
ASV_16s_5409	0.838555655	-0.487487923	2.741827522	3.325646859	0.85333303	NA
ASV_16s_5410	0.868507614	-0.12039309	3.549581671	2.009680175	0.959302649	NA
ASV_16s_5411	0.928324179	0.079312801	3.548263233	2.096409934	0.954311972	NA
ASV_16s_5412	1.035869991	2.429407567	2.935935237	1.275938745	0.98907206	NA
ASV_16s_5413	0.970427623	1.864693711	3.54701764	1.320752949	NA	NA
ASV_16s_5414	1.059617104	0.255775392	3.55014293	2.050692993	0.956981374	NA
ASV_16s_5415	0.914001544	0.593752277	3.147580389	2.810707483	0.901943756	NA
ASV_16s_5416	1.269847067	-0.551676629	3.54894804	1.197423443	NA	NA
ASV_16s_5417	0.531681331	0.219973045	3.550630922	1.272129794	NA	NA
ASV_16s_5418	0.339060767	1.292678108	3.550221362	0.970083875	NA	NA
ASV_16s_5419	0.667922699	0.591498987	3.549659837	1.276033646	0.989069607	NA
ASV_16s_5420	1.067811179	0.668470099	3.550670185	2.919816744	NA	NA
ASV_16s_5421	0.984886908	0.771548828	3.54958834	2.633773021	0.916691598	NA
ASV_16s_5422	1.045220904	0.361635594	3.5502391	1.419401504	0.98496959	NA
ASV_16s_5423	1.045159308	0.59443445	3.550634002	3.036284261	0.881625465	NA
ASV_16s_5424	1.105290944	1.184403716	3.549884814	1.37833542	0.986225607	NA
ASV_16s_5425	0.997280387	0.693924118	3.550626747	1.690722105	0.97496381	NA
ASV_16s_5426	0.779807683	-0.328917277	3.549292693	2.384869372	0.935514201	NA
ASV_16s_5428	0.766191494	0.120988219	3.548697421	2.588701791	0.920270958	NA
ASV_16s_5429	0.776577017	0.218561673	3.548574754	2.68038611	0.912912795	NA
ASV_16s_5430	0.803796982	-0.580826485	3.548050414	1.897210604	0.965309696	NA
ASV_16s_5431	0.754594637	0.629284999	3.548442745	2.48708329	0.928066561	NA
ASV_16s_5432	0.986217065	-0.029599337	3.547279735	2.352493613	NA	NA
ASV_16s_5433	0.935906424	-0.794781404	3.548282022	2.170883626	0.949779839	NA
ASV_16s_5434	0.982613017	-0.9034396	3.547986743	1.881254418	0.966119201	NA
ASV_16s_5435	0.941578876	-0.048308574	3.548282642	2.19388317	NA	NA
ASV_16s_5436	1.063629281	-0.845556365	3.548406285	1.772145284	0.971369526	NA
ASV_16s_5437	1.025283136	0.416575642	3.293761507	2.247966099	0.944851622	NA
ASV_16s_5438	0.797820153	-0.088784246	3.549630549	2.083821212	0.955055613	NA
ASV_16s_5439	0.973363077	-0.212162691	3.549930058	2.355954747	0.937547801	NA
ASV_16s_5440	0.777791295	-0.459016107	3.54730652	1.75125595	0.972318001	NA
ASV_16s_5441	0.33845065	-0.159720915	3.549622212	1.538513489	NA	NA
ASV_16s_5442	0.368742149	1.336149495	3.550181617	0.786847097	NA	NA
ASV_16s_5443	0.806090479	3.022585655	3.547271788	0.374434366	NA	NA
ASV_16s_5444	0.872809351	0.209740174	3.548248425	2.570687846	0.921680811	NA
ASV_16s_5445	0.969149767	0.38998927	3.550372042	0.890343159	0.99640899	NA
ASV_16s_5446	0.932187606	0.364048882	3.550417214	1.045788988	0.994055694	NA
ASV_16s_5447	1.035420199	0.372785803	3.550208633	2.02922889	0.958204901	NA
ASV_16s_5448	1.203327711	0.7957078	3.548466861	1.778320596	0.97108565	NA
ASV_16s_5449	0.949322864	0.279848041	3.549511891	3.255439847	NA	NA

ASV_16s_5450	1.062580569	1.187518052	3.549588316	1.291752758	0.988658668	NA
ASV_16s_5451	0.740738436	-0.231182687	3.549334163	2.404484333	0.934116144	NA
ASV_16s_5452	0.734081577	-0.321661421	3.549322455	2.333843876	NA	NA
ASV_16s_5453	0.724229017	-0.335416146	3.549327335	2.304301784	NA	NA
ASV_16s_5454	0.773897489	-0.355745102	3.549293232	2.341767484	0.938533653	NA
ASV_16s_5455	0.255222728	-0.673946004	3.550457852	0.773631598	NA	NA
ASV_16s_5456	0.154455848	-0.169858544	3.550678583	0.679644246	0.998487299	NA
ASV_16s_5457	0.803287869	0.083205279	3.548464182	2.695921026	0.911636247	NA
ASV_16s_5458	0.781895789	9.91E-04	3.54818675	2.805660943	NA	NA
ASV_16s_5459	0.478959879	0.710033196	3.548793685	1.645959115	0.976822375	NA
ASV_16s_5460	0.759840397	0.088881616	3.547622358	2.188833662	NA	NA
ASV_16s_5461	0.803123582	-0.197857704	3.548343125	2.701966174	0.911137199	NA
ASV_16s_5462	0.868973249	0.471085825	3.547516395	2.407350175	NA	NA
ASV_16s_5463	0.968855505	-0.794607311	3.548297567	2.231522336	0.945923046	NA
ASV_16s_5464	0.790828568	-1.404240774	3.548358629	1.432509932	NA	NA
ASV_16s_5465	0.933270046	-0.793971554	3.548426927	2.232050697	0.945888788	NA
ASV_16s_5466	0.940998832	0.160786383	3.549318936	2.485562225	0.928180314	NA
ASV_16s_5467	0.798574139	0.853902423	3.547776822	1.437563728	NA	NA
ASV_16s_5468	0.653077821	1.786689262	3.548863204	1.5283904	NA	NA
ASV_16s_5469	0.839180777	0.603390788	3.548751051	1.464663642	NA	NA
ASV_16s_5470	0.516525323	0.219974218	3.550632523	1.25742746	0.989543984	NA
ASV_16s_5471	0.525673633	0.219973542	3.550631546	1.266348811	NA	NA
ASV_16s_5472	0.79781248	2.158462291	3.548189263	0.886306696	0.9964602	NA
ASV_16s_5473	0.887949624	1.126573937	3.548888968	1.530532664	0.981233755	NA
ASV_16s_5474	0.97005159	1.205693629	3.549320205	1.150921028	NA	NA
ASV_16s_5475	1.065910478	0.270533056	3.549608241	3.381380788	NA	NA
ASV_16s_5476	1.101443516	1.18722657	3.549656421	1.326233059	0.987724338	NA
ASV_16s_5477	1.095441298	0.735277046	3.550673904	1.779305669	NA	NA
ASV_16s_5478	0.721301388	-0.254497814	3.549343027	2.369853287	NA	NA
ASV_16s_5479	0.723964729	-0.352946073	3.549325726	2.280451827	NA	NA
ASV_16s_5480	0.460124779	0.219979516	3.550639406	1.198694254	NA	NA
ASV_16s_5481	0.246318845	0.220020958	3.550694061	0.882201326	0.996511794	NA
ASV_16s_5482	0.724813925	-0.170753477	3.548467226	2.117065345	NA	NA
ASV_16s_5483	0.782645391	0.009050161	3.548247504	2.532943868	0.924595996	NA
ASV_16s_5484	0.775912255	0.814579473	3.547444435	2.482945442	NA	NA
ASV_16s_5485	0.885263046	-0.150760337	3.54812033	2.787713571	0.903921537	NA
ASV_16s_5486	0.913813569	0.680734995	3.548504422	2.516562834	0.925844711	NA
ASV_16s_5487	0.855521862	-0.861477544	3.548192898	1.801455935	0.970007968	NA
ASV_16s_5488	0.853467034	-0.299385941	3.549000728	2.901903246	0.893926213	NA
ASV_16s_5489	0.999276949	-1.609210011	2.943572997	1.77404048	0.971282574	NA
ASV_16s_5490	0.758143354	-0.086495257	3.550361244	1.93027513	0.963598399	NA
ASV_16s_5491	0.930140876	0.21527731	3.550542721	2.561502334	0.922395116	NA
ASV_16s_5492	1.042202939	0.009160397	3.347745514	2.415171637	NA	NA
ASV_16s_5493	0.800684706	-0.074007396	3.549508547	2.193573294	0.948354128	NA
ASV_16s_5494	0.719094053	2.370927962	3.548481094	1.144862988	NA	NA
ASV_16s_5495	0.720702186	-0.111389874	3.550427304	0.657778197	0.998639535	NA
ASV_16s_5496	0.314566969	0.530478572	3.54999765	1.491230953	NA	NA
ASV_16s_5497	0.498363964	0.219975675	3.550634569	1.239238501	0.9899952	NA
ASV_16s_5498	0.502090471	0.219975362	3.550634137	1.243023112	0.98990233	NA
ASV_16s_5499	0.757109866	1.165014964	3.548548549	2.098420693	0.954192589	NA
ASV_16s_5500	0.782785986	-0.069575951	3.54946528	2.536003895	0.924361622	NA
ASV_16s_5501	0.865840624	0.646337493	3.54960598	1.713817799	NA	NA
ASV_16s_5502	1.040012859	1.54518408	3.548899602	1.247175405	0.989799825	NA
ASV_16s_5503	1.143418352	-0.366698277	3.548600928	2.56004564	0.922508108	NA
ASV_16s_5504	0.942628435	1.492598659	3.548854458	1.727653975	0.973367743	NA
ASV_16s_5505	1.170021259	1.463552359	3.548567209	2.131094678	0.952229424	NA
ASV_16s_5506	0.919992855	-0.116343955	3.549940702	2.105710647	0.953758378	NA
ASV_16s_5507	1.142960558	1.03006884	3.549949947	2.875845923	NA	NA
ASV_16s_5508	1.160216446	0.712673463	3.549409138	2.536488332	0.924324486	NA
ASV_16s_5509	0.78829414	0.360435826	3.550722771	1.675607557	NA	NA
ASV_16s_5510	1.002238325	0.751200975	3.550189432	2.251609066	0.94461279	NA
ASV_16s_5511	0.748605573	-0.405721468	3.549306855	2.223192914	0.946461618	NA
ASV_16s_5512	0.348072578	0.199160293	3.550616805	2.485227777	0.928205314	NA
ASV_16s_5513	0.457480384	0.219979802	3.550639771	1.195764081	NA	NA
ASV_16s_5514	0.758220002	0.349019637	3.548714021	2.261737966	0.943945949	NA
ASV_16s_5515	0.77237522	1.37844787	3.547499959	2.160367064	0.950433558	NA
ASV_16s_5516	1.030739298	0.988378758	3.545570033	1.214836705	0.990581264	NA

ASV_16s_5517	0.8553445	0.755698266	3.547548985	2.448640014	NA	NA
ASV_16s_5518	0.864570039	-0.077062003	3.547775928	2.645011214	0.915787662	NA
ASV_16s_5519	0.86296346	-0.147114224	3.549491257	1.925798891	NA	NA
ASV_16s_5520	0.959056477	-0.827993541	3.070720651	2.379358652	0.935904291	NA
ASV_16s_5521	0.988945447	1.884470322	3.317510948	1.007835826	0.994701141	NA
ASV_16s_5522	0.784982972	-0.561859578	3.548026932	1.946792097	0.962726462	NA
ASV_16s_5523	0.370001068	0.750443204	3.549591415	1.130946943	NA	NA
ASV_16s_5524	0.932405239	-1.925058001	2.859360552	1.068260474	0.993650983	NA
ASV_16s_5525	0.856021482	2.390557814	3.548652245	1.266184889	0.989322328	NA
ASV_16s_5526	0.816962001	1.38529263	3.388523312	1.653454403	0.976516956	NA
ASV_16s_5527	1.104501654	0.789474009	3.549239077	2.683997792	NA	NA
ASV_16s_5528	0.773543432	1.014525202	3.54794471	1.805144991	NA	NA
ASV_16s_5529	1.726259	1.253648663	3.549292741	1.332180845	NA	NA
ASV_16s_5530	1.058121757	0.210380672	3.549345795	2.953206591	0.889296984	NA
ASV_16s_5531	1.028401329	0.363469976	3.549740602	1.318252933	0.987944623	NA
ASV_16s_5532	0.88489379	0.315939233	3.55066848	1.695405526	0.974764549	NA
ASV_16s_5533	1.000490935	0.693925106	3.550626509	1.692335903	0.974895252	NA
ASV_16s_5534	1.029462777	0.69393782	3.550624421	1.706867012	0.974273064	NA
ASV_16s_5535	0.715830538	-0.191535956	3.549360803	2.373332766	NA	NA
ASV_16s_5536	0.726292201	-0.252597629	3.549339915	2.377499232	NA	NA
ASV_16s_5537	0.742449116	-0.408754091	3.549310935	2.209136829	0.947364131	NA
ASV_16s_5538	0.250847482	-0.672131842	3.550482164	0.767876878	NA	NA
ASV_16s_5539	0.732234688	0.066550039	3.54856089	2.571300697	NA	NA
ASV_16s_5540	0.759659172	0.026828891	3.548397343	2.269122018	0.943457231	NA
ASV_16s_5541	0.940825147	-0.794757404	3.548285952	2.182262474	0.949067457	NA
ASV_16s_5542	0.907413954	-0.794193898	3.548389647	2.188406495	0.948680621	NA
ASV_16s_5543	0.901757314	0.453825508	3.548017125	2.203131528	0.947747289	NA
ASV_16s_5544	0.505418068	0.930811971	3.548330918	1.956679651	NA	NA
ASV_16s_5545	0.885900183	-0.921927053	3.547915318	1.7535323	0.972215527	NA
ASV_16s_5546	0.904408708	-24.05424224	3.551021145	-0.221646992	NA	NA
ASV_16s_5547	0.744566926	-0.783988559	3.548920061	1.760339486	0.971907803	NA
ASV_16s_5548	0.838191808	-0.324850872	3.549312128	1.156931211	0.991884976	NA
ASV_16s_5549	0.793511376	-0.104141104	3.54975649	2.009262307	0.959325942	NA
ASV_16s_5550	0.819812205	0.164128934	3.548248549	2.777937448	NA	NA
ASV_16s_5551	0.697758242	-0.637761996	3.5485439	1.385087039	NA	NA
ASV_16s_5552	0.360361645	1.336014634	3.550211841	0.779443611	NA	NA
ASV_16s_5553	0.48923515	0.219976476	3.550635655	1.229840328	0.990223523	NA
ASV_16s_5554	0.888045818	-0.279696299	3.548594323	3.23774264	0.862172783	NA
ASV_16s_5555	1.716280624	1.253639981	3.54929522	1.330307254	NA	NA
ASV_16s_5556	0.913483809	0.095560842	3.549627818	3.580114299	0.826668461	NA
ASV_16s_5557	1.05336545	0.670049054	3.550667274	2.430453402	NA	NA
ASV_16s_5558	0.824930009	0.343352726	3.549418124	2.553614672	0.923006007	NA
ASV_16s_5559	0.967669207	0.563243499	3.550643477	3.08563749	0.876968614	NA
ASV_16s_5560	0.948605575	0.315939222	3.550663027	1.730780061	0.973230038	NA
ASV_16s_5561	0.97861522	0.693919209	3.550628168	1.681079054	0.975371213	NA
ASV_16s_5562	0.288371895	0.006261515	3.550619496	1.75255855	NA	NA
ASV_16s_5563	0.285146777	0.122271001	3.550647121	1.582166346	NA	NA
ASV_16s_5564	0.769537029	-0.418114009	3.54869313	2.288357439	NA	NA
ASV_16s_5565	0.740915198	0.128494663	3.548314133	2.77883039	NA	NA
ASV_16s_5566	0.748088532	0.232771853	3.548449996	2.488702604	NA	NA
ASV_16s_5567	0.961509514	2.115617916	3.548783107	0.787052864	NA	NA
ASV_16s_5568	0.951694206	-0.79463616	3.548297089	2.206726112	0.947518118	NA
ASV_16s_5569	0.895639652	0.604544258	3.303310767	3.11615263	0.874053214	NA
ASV_16s_5570	0.803138403	0.684099065	3.547331158	2.438055507	0.931688872	NA
ASV_16s_5571	0.884272818	-2.387466311	3.546856035	1.369809462	0.986478078	NA
ASV_16s_5572	0.832359092	-0.32453381	3.549135514	1.389310061	0.985896439	NA
ASV_16s_5573	0.839742203	-0.517510414	3.548043552	2.294090688	0.941788563	NA
ASV_16s_5574	0.844139207	-0.238564746	3.328363082	2.478988995	0.928670887	NA
ASV_16s_5575	0.659124903	-0.067746958	3.549964409	1.024106247	0.994430239	NA
ASV_16s_5576	0.736244555	-0.105520337	3.549155988	2.676011928	0.913270693	NA
ASV_16s_5577	0.483032205	0.219977043	3.550636417	1.22335554	0.990379162	NA
ASV_16s_5578	0.482942228	0.219977199	3.550636428	1.223261063	0.990381418	NA
ASV_16s_5579	0.51356226	0.219974469	3.550632847	1.254491646	0.989617648	NA
ASV_16s_5580	0.770596068	2.528177632	3.547487021	1.078852099	NA	NA
ASV_16s_5581	0.988362432	-0.241048975	3.548075641	2.219174069	NA	NA
ASV_16s_5582	1.016713737	0.670453616	3.550103298	2.525816958	0.925140508	NA
ASV_16s_5583	0.974642441	0.368843295	3.550608105	0.944144482	0.995679952	NA

ASV_16s_5584	1.282177034	0.303544202	3.550612499	3.599409422	0.82458674	NA
ASV_16s_5585	0.994168316	0.693925012	3.550626981	1.689108431	0.975032254	NA
ASV_16s_5586	0.773719595	-0.326911552	3.549296619	2.379467427	0.935896602	NA
ASV_16s_5587	0.731140176	-0.346468415	3.549321444	2.299099917	0.941450808	NA
ASV_16s_5588	0.364067009	0.219992065	3.550656033	1.079836954	0.993435838	NA
ASV_16s_5590	0.759507538	0.632239582	3.54808601	2.453639009	NA	NA
ASV_16s_5591	0.792692294	0.197531304	3.105758106	3.114001216	0.874259649	NA
ASV_16s_5592	0.795538999	-0.050717408	3.548390228	2.119779328	0.952914241	NA
ASV_16s_5593	0.619555419	0.819652218	3.548445121	2.284860083	NA	NA
ASV_16s_5594	0.943952488	-0.794938792	3.548231823	2.128437569	0.952390706	NA
ASV_16s_5595	0.751058281	1.7686315	3.548574701	0.680747758	NA	NA
ASV_16s_5596	0.988751354	-0.372247166	3.550176404	1.059886168	0.993803788	NA
ASV_16s_5597	0.74761068	0.440551772	3.550419416	1.696401279	0.974722066	NA
ASV_16s_5598	0.749852287	0.063652569	3.548329181	1.943841601	0.962883054	NA
ASV_16s_5599	0.450688822	1.33717601	3.549944973	0.851387121	NA	NA
ASV_16s_5600	0.714121752	0.360304421	3.549208876	3.195315966	0.866365164	NA
ASV_16s_5601	0.689007347	0.037930305	3.549168192	2.09738665	NA	NA
ASV_16s_5602	0.980828248	-0.001005556	3.546568781	1.975939417	NA	NA
ASV_16s_5603	1.212918977	0.889121516	3.549445444	2.651629187	0.915253227	NA
ASV_16s_5604	0.986583119	0.363334432	3.550121723	1.371216835	0.986436598	NA
ASV_16s_5605	0.948041588	1.084839424	3.550307431	1.406040393	0.985385534	NA
ASV_16s_5606	1.064142975	0.735266485	3.550676802	1.764526127	NA	NA
ASV_16s_5607	1.064142975	0.735266485	3.550676802	1.764526127	NA	NA
ASV_16s_5608	0.969771987	0.693920804	3.55062886	1.67646064	0.975564966	NA
ASV_16s_5609	0.945240256	0.693909907	3.550630847	1.663409391	0.976107715	NA
ASV_16s_5610	0.708349181	-0.347728267	3.549337217	2.266360104	NA	NA
ASV_16s_5611	0.759860827	-0.395026558	3.54929974	2.258256105	0.944175642	NA
ASV_16s_5612	0.700838299	-0.416973838	3.54934055	2.136934749	NA	NA
ASV_16s_5613	0.917815988	-0.684742661	3.549220699	1.186127578	0.991242807	NA
ASV_16s_5614	0.259405475	0.040838223	3.550626627	1.865374665	NA	NA
ASV_16s_5615	0.3066489	0.22000351	3.550670935	0.992892236	NA	NA
ASV_16s_5616	0.709346162	0.261860809	3.549113345	2.310808374	NA	NA
ASV_16s_5617	0.784817478	0.517501857	3.54811506	2.63110513	0.91690552	NA
ASV_16s_5618	0.931265511	-0.795530509	3.548250666	1.863243321	0.967020179	NA
ASV_16s_5619	0.756063444	-0.336972938	3.548104433	2.365116341	0.936906987	NA
ASV_16s_5620	0.738387323	0.377362268	3.547699235	1.444341771	NA	NA
ASV_16s_5621	0.795520797	0.211226128	3.548175744	2.525112088	0.925194258	NA
ASV_16s_5622	0.763913943	-0.671420151	3.548173552	2.331480103	0.939243564	NA
ASV_16s_5623	0.733390637	-0.683778363	3.548467432	1.88908939	NA	NA
ASV_16s_5624	0.904624989	-0.794665383	3.548293545	2.122448702	0.95275316	NA
ASV_16s_5625	0.904111074	-0.872515826	3.548384769	2.133997206	0.952052916	NA
ASV_16s_5626	0.893711982	-0.793194651	3.548592794	2.213340231	0.947095074	NA
ASV_16s_5627	0.861667767	-0.791981261	3.548847327	2.189919645	0.948585116	NA
ASV_16s_5628	0.907118705	2.769171934	3.547948631	1.002548532	0.99478732	NA
ASV_16s_5629	0.789185137	-0.12404187	3.549661432	1.90361636	NA	NA
ASV_16s_5630	0.787774752	-0.111986908	3.549718611	1.965411437	0.961729911	NA
ASV_16s_5631	0.988715969	1.187071118	3.549987074	1.025076035	NA	NA
ASV_16s_5632	0.248691129	0.41239015	3.550869895	0.678683869	NA	NA
ASV_16s_5633	1.012974038	1.315298922	2.936000881	2.628393794	0.917122663	NA
ASV_16s_5634	1.044108469	-2.182147904	3.547345349	0.586251747	NA	NA
ASV_16s_5635	0.706313926	-0.128094149	3.54829205	1.904373005	NA	NA
ASV_16s_5636	1.130831984	1.205993403	3.549246508	1.200360617	NA	NA
ASV_16s_5637	1.09885333	0.997390181	3.447477709	2.039441617	0.957625117	NA
ASV_16s_5638	0.903945824	0.315939258	3.55066677	1.706221603	0.974300885	NA
ASV_16s_5639	0.908388904	0.707393228	3.550598463	2.951604848	0.889442779	NA
ASV_16s_5640	0.941861675	0.454380755	3.550656457	3.146897909	0.871088504	NA
ASV_16s_5641	0.899956173	-0.684699343	3.549230375	1.179787967	0.991384853	NA
ASV_16s_5642	0.738327244	0.095055011	3.549322307	2.406687666	0.93395817	NA
ASV_16s_5643	0.730382742	-0.055533191	3.548899616	2.268865981	0.943474213	NA
ASV_16s_5644	0.76613993	0.134511903	3.548703217	2.595028393	0.919772987	NA
ASV_16s_5645	0.734058403	0.256163701	3.548989788	2.398069233	0.934575022	NA
ASV_16s_5646	0.752497833	0.03041253	3.548843001	2.30901939	0.940779037	NA
ASV_16s_5647	0.742942253	0.600156317	3.548289189	2.597789184	NA	NA
ASV_16s_5648	0.699845682	-0.715834442	3.548503699	1.872008814	NA	NA
ASV_16s_5649	0.923211231	-0.795102773	3.548234433	2.070387616	0.955841969	NA
ASV_16s_5650	0.810273084	0.08347851	3.548220812	2.96608384	0.888121919	NA
ASV_16s_5651	0.642054057	1.072345733	3.548029097	2.072945319	0.955692823	NA

ASV_16s_5652	0.727490594	-0.250902695	3.548815306	1.897169201	NA	NA
ASV_16s_5653	0.882259921	-0.793101061	3.548610649	2.202798134	0.947768518	NA
ASV_16s_5654	0.82326694	0.174073447	2.634449964	3.038829508	0.881387089	NA
ASV_16s_5655	0.78740025	-0.989890391	3.548388244	1.755627373	NA	NA
ASV_16s_5656	0.945058871	-1.245068379	3.164191485	1.463408622	0.983549484	NA
ASV_16s_5657	0.801424019	-0.106245053	3.549595389	1.948409928	0.962640444	NA
ASV_16s_5658	0.791281935	2.06850938	3.547940616	0.566176712	NA	NA
ASV_16s_5659	0.839505082	-0.221332922	3.328359231	1.451691478	0.983935137	NA
ASV_16s_5660	0.850758042	1.750289512	3.357847229	1.85478317	0.967438706	NA
ASV_16s_5661	0.838651824	2.488267945	3.548265783	0.626333794	0.998839969	NA
ASV_16s_5662	0.854991018	1.340752179	3.549471892	1.121202608	NA	NA
ASV_16s_5663	0.247219584	0.41197611	3.550877425	0.676699773	NA	NA
ASV_16s_5664	0.340928111	-0.161764418	3.549615738	1.543713439	NA	NA
ASV_16s_5665	0.477129776	0.219977792	3.550637161	1.2171003	0.990527823	NA
ASV_16s_5666	0.571740611	2.405136329	3.54905531	0.489406985	0.999484352	NA
ASV_16s_5667	0.691131727	0.737094455	2.509441772	2.521855063	0.925442385	NA
ASV_16s_5668	1.02105617	1.131753091	2.918252367	3.824110723	0.799798972	NA
ASV_16s_5669	1.142057222	0.34649394	3.549162645	2.58398376	0.920641363	NA
ASV_16s_5670	0.970930937	-0.382571913	3.548376569	3.089137756	0.876635587	NA
ASV_16s_5671	1.046690349	0.800871995	3.548712922	1.733420866	NA	NA
ASV_16s_5672	1.405963819	0.360453326	3.550665965	1.970289938	NA	NA
ASV_16s_5673	0.951161829	0.69391619	3.550630358	1.666591571	0.975976032	NA
ASV_16s_5674	0.922140793	0.693902697	3.550632814	1.650806382	0.976625123	NA
ASV_16s_5675	0.715859705	-0.239963182	3.549349394	2.368191684	0.936691144	NA
ASV_16s_5676	0.722300974	-0.267054392	3.549340481	2.364393214	0.936957686	NA
ASV_16s_5677	0.697888189	-0.3350354	3.549346185	2.268366066	0.943507364	NA
ASV_16s_5678	0.645050806	0.243125593	3.549031532	2.164139856	NA	NA
ASV_16s_5679	0.750297901	-1.267943095	3.54819798	1.392766111	0.985791802	NA
ASV_16s_5680	0.739918418	0.532545654	3.547981659	2.498990959	0.927173028	NA
ASV_16s_5681	0.626691268	0.66314636	3.548525358	2.284251437	NA	NA
ASV_16s_5682	0.734439038	1.445704896	3.547469643	2.108299955	0.953603626	NA
ASV_16s_5683	0.87113004	-0.291894322	2.77734119	3.551994516	0.829687992	NA
ASV_16s_5684	0.847507997	-0.793876306	3.548465696	2.13202009	0.952173186	NA
ASV_16s_5685	0.450179165	0.133596351	3.548878965	1.078825983	NA	NA
ASV_16s_5686	0.925111202	-0.922961336	3.547701862	1.797793025	0.970180083	NA
ASV_16s_5687	0.873051058	-0.79308161	3.548618738	2.193550967	0.948355541	NA
ASV_16s_5688	0.752653456	-0.975090717	3.548775736	1.855165641	0.96741985	NA
ASV_16s_5689	0.988291401	1.097426272	3.54928685	1.806951398	0.969748694	NA
ASV_16s_5690	0.752611068	0.201854356	3.549179037	1.840648398	0.968131285	NA
ASV_16s_5691	0.809006588	-0.111579913	3.549685832	1.67422295	NA	NA
ASV_16s_5692	1.004434825	-0.265688216	3.547870848	2.09647795	NA	NA
ASV_16s_5693	0.69159447	1.930821956	3.549147718	0.91077491	NA	NA
ASV_16s_5694	0.930578986	-0.321596623	3.54819265	2.565786949	0.922062313	NA
ASV_16s_5695	1.133375922	0.014062965	3.549496463	3.63501363	0.820724992	NA
ASV_16s_5696	1.052922514	0.724529078	3.5490592	2.512779042	0.92613172	NA
ASV_16s_5697	0.886866663	0.343219901	3.549453381	2.485448164	NA	NA
ASV_16s_5698	0.953536575	0.315939263	3.550662636	1.733401065	0.973114269	NA
ASV_16s_5699	0.885219439	0.315939192	3.550668452	1.695550429	0.97475837	NA
ASV_16s_5700	0.695859338	-0.168496822	3.549382634	2.340010504	NA	NA
ASV_16s_5701	0.697412851	-0.194228947	3.549373513	2.347944492	NA	NA
ASV_16s_5702	0.725161497	-0.260147357	3.549339523	2.372146428	0.936413039	NA
ASV_16s_5703	0.690667317	-0.257712756	3.549364649	2.325493696	NA	NA
ASV_16s_5704	0.685165041	-0.30775348	3.54935958	2.28055603	NA	NA
ASV_16s_5705	0.242096988	-0.667126121	3.550527593	0.755963024	NA	NA
ASV_16s_5706	0.242096988	-0.667126121	3.550527593	0.755963024	NA	NA
ASV_16s_5707	0.760622648	-0.279309348	3.548507003	2.13340387	0.952089026	NA
ASV_16s_5708	0.708620196	0.107542164	3.548590987	2.56886165	0.921823072	NA
ASV_16s_5709	0.727961932	0.345103471	3.548799544	2.182905405	NA	NA
ASV_16s_5710	0.688664789	-0.341203666	3.548462169	1.494017169	NA	NA
ASV_16s_5711	0.745584326	0.110746562	3.548311334	2.816602674	0.90143382	NA
ASV_16s_5712	0.764244891	-0.441532151	3.548330153	2.323472974	0.939793236	NA
ASV_16s_5713	0.70064901	0.159410878	3.548354372	2.726877321	NA	NA
ASV_16s_5714	0.827949253	-0.373655871	3.547614807	2.22805964	0.946147279	NA
ASV_16s_5715	0.713551609	0.079277794	3.548324581	2.745583944	NA	NA
ASV_16s_5716	0.745153288	0.243213698	3.548164654	2.444194842	0.931240301	NA
ASV_16s_5717	0.733560626	-0.553891881	3.548814972	1.639119795	NA	NA
ASV_16s_5718	0.899441161	-0.851673825	3.548308287	1.538537984	NA	NA

ASV_16s_5719	0.855996909	0.232209656	3.548586068	2.819465728	0.901185743	NA
ASV_16s_5720	0.864336373	-0.096789103	3.548572589	2.615374662	0.918161624	NA
ASV_16s_5721	0.815249523	0.070590133	3.548925443	2.966908187	0.888046519	NA
ASV_16s_5722	0.736589201	-0.116179562	3.55005275	1.353013139	NA	NA
ASV_16s_5723	1.17304142	0.107252819	3.548949288	2.365291365	0.936894713	NA
ASV_16s_5724	1.006671449	0.717947878	3.55034258	1.515326589	0.981774305	NA
ASV_16s_5725	0.599779518	-1.006014197	3.549446785	0.84652604	NA	NA
ASV_16s_5726	0.488738325	0.216887797	3.550636079	1.006602917	0.994721318	NA
ASV_16s_5727	0.580444885	0.18089207	3.548643929	1.559324446	0.980184492	NA
ASV_16s_5728	1.110758044	0.419312336	3.549366516	1.16138158	NA	NA
ASV_16s_5729	0.920923869	1.6442954	3.548917206	1.375082184	NA	NA
ASV_16s_5730	0.815640177	1.692348887	3.549107468	0.802847684	NA	NA
ASV_16s_5731	0.712904784	-0.069550949	3.549488606	2.437432088	NA	NA
ASV_16s_5732	0.531646203	-0.114087718	3.549369466	1.758401781	NA	NA
ASV_16s_5733	0.615979232	-0.028920913	3.549852912	1.940135863	NA	NA
ASV_16s_5734	1.65641037	1.253586922	3.549310721	1.318831675	NA	NA
ASV_16s_5735	0.665652626	1.248591194	3.549957822	1.02445715	NA	NA
ASV_16s_5736	0.930440869	1.272293141	3.550230205	1.158143201	0.991858927	NA
ASV_16s_5737	1.071192164	-0.511994738	3.548656506	2.474701133	0.928990016	NA
ASV_16s_5738	0.876690444	0.315939295	3.550669242	1.690625994	0.974967889	NA
ASV_16s_5739	0.673599589	-0.198459882	3.549390784	2.314154802	NA	NA
ASV_16s_5740	0.683594751	-0.293733269	3.549363289	2.291046507	NA	NA
ASV_16s_5742	0.740979325	0.09792827	3.548643285	2.559384282	0.922559382	NA
ASV_16s_5743	0.830502917	2.134402648	3.548407828	0.625875432	0.998842734	NA
ASV_16s_5744	0.687891474	-0.020713808	3.548357088	2.514488945	NA	NA
ASV_16s_5745	0.790062782	0.503067353	3.325376701	2.603958212	0.919067628	NA
ASV_16s_5746	0.590666103	0.714742792	3.54828444	2.190358583	NA	NA
ASV_16s_5747	0.679616744	1.493168169	3.5475491	1.978238058	NA	NA
ASV_16s_5748	0.910213467	-0.794472604	3.548340759	2.169275491	0.949880094	NA
ASV_16s_5749	0.764727361	1.852646063	3.547203308	1.914967831	NA	NA
ASV_16s_5750	0.808843596	-0.937319797	3.548556287	1.982077502	0.960825667	NA
ASV_16s_5751	0.871789741	0.276245581	3.165721223	2.004289945	0.959602548	NA
ASV_16s_5752	0.737818559	-1.366267024	2.79151759	2.150942042	0.9510156	NA
ASV_16s_5753	0.789534654	-0.21896511	3.549720682	1.642200575	0.976974653	NA
ASV_16s_5754	0.738778514	-1.148808444	3.548543864	1.836386906	NA	NA
ASV_16s_5755	1.10302852	1.16843881	3.549249804	1.432256851	0.984562719	NA
ASV_16s_5756	0.801363119	-1.061445733	3.547507949	1.448017053	0.984054945	NA
ASV_16s_5757	0.489128581	0.701611851	3.550501859	1.249699564	0.989737198	NA
ASV_16s_5758	0.491677232	1.742071909	3.550005941	0.632717045	NA	NA
ASV_16s_5759	0.71085195	-0.069512204	3.549490359	2.42714187	NA	NA
ASV_16s_5760	0.957493468	0.344315984	3.549675855	2.26280613	NA	NA
ASV_16s_5761	0.959814551	0.315939219	3.550662144	1.736722268	0.97296716	NA
ASV_16s_5762	0.956449382	0.565391243	3.550643953	3.063927855	0.879026046	NA
ASV_16s_5763	0.988423749	1.132940776	3.550184837	1.392101425	0.985811962	NA
ASV_16s_5764	0.862183596	0.693888488	3.550638412	1.616586725	0.977996867	NA
ASV_16s_5765	0.676564349	-0.170162225	3.549397155	2.312942388	NA	NA
ASV_16s_5766	0.680809723	-0.319623269	3.549361118	2.262024696	0.943927012	NA
ASV_16s_5767	0.748973854	0.064735286	3.549054735	2.522337382	0.925405666	NA
ASV_16s_5768	0.702806695	0.766356799	3.548577595	2.357616197	NA	NA
ASV_16s_5769	0.796286665	-0.765133875	3.548317101	1.910858385	0.964608867	NA
ASV_16s_5770	0.723033466	0.633089004	3.548164714	2.108927043	NA	NA
ASV_16s_5771	0.882505149	-0.794605645	3.548313512	2.097083122	0.954272022	NA
ASV_16s_5772	0.882104662	-0.178560637	3.54896769	2.442127031	0.931391547	NA
ASV_16s_5773	0.860707949	-0.314883745	3.548117746	2.422704152	0.932804194	NA
ASV_16s_5774	0.68296489	-0.599870301	3.550320624	1.204603903	0.990820507	NA
ASV_16s_5775	0.944758286	-0.105727831	3.548135246	1.456099807	NA	NA
ASV_16s_5776	1.117092431	0.420680519	3.54984853	0.917673624	0.996049554	NA
ASV_16s_5777	0.706815293	-1.042738774	3.547877448	1.514341102	NA	NA
ASV_16s_5778	0.513408842	0.004189352	3.549315019	2.407050728	NA	NA
ASV_16s_5779	0.241333403	0.409948552	3.550905312	0.668592769	NA	NA
ASV_16s_5780	1.240084229	1.081885621	3.549299266	1.997744672	NA	NA
ASV_16s_5781	1.227148773	0.313511733	3.546858966	2.012099503	NA	NA
ASV_16s_5782	1.071491495	0.051124188	3.547266716	1.953403594	0.962374252	NA
ASV_16s_5783	0.823193753	-0.638644048	3.548542662	2.081108311	0.955215016	NA
ASV_16s_5784	0.886807566	0.250970133	3.549470126	3.394064813	0.846315353	NA
ASV_16s_5785	0.665485978	-0.237466939	3.549387995	2.297228639	NA	NA
ASV_16s_5786	0.212162572	-0.170051618	3.550631744	0.833683551	NA	NA

ASV_16s_5787	0.188189758	-0.16998665	3.550647725	0.774696958	NA	NA
ASV_16s_5788	0.779197242	-0.818641175	3.548733744	1.919248295	0.964174177	NA
ASV_16s_5789	0.716996006	0.149955536	3.548741533	2.548531446	0.923398477	NA
ASV_16s_5790	0.807096796	0.455845663	3.548242777	2.295270014	0.941709134	NA
ASV_16s_5791	0.859984764	-0.795100499	3.548291282	1.923681276	0.963943314	NA
ASV_16s_5792	0.688231416	0.824240066	3.548271339	2.45798101	NA	NA
ASV_16s_5793	0.730274807	1.40765895	3.547047134	1.78937405	NA	NA
ASV_16s_5794	0.87055678	-0.51018691	3.125081454	3.00828543	0.884234704	NA
ASV_16s_5795	0.854494677	-0.084668742	3.547855442	2.607866382	0.918758011	NA
ASV_16s_5796	0.827456255	-1.192410294	3.139714703	2.311950164	0.940579812	NA
ASV_16s_5797	0.938301934	3.500590475	3.545937634	0.351429248	0.999829375	NA
ASV_16s_5798	0.701372371	1.289553031	3.548382746	1.826782694	NA	NA
ASV_16s_5799	0.766985216	0.278985099	3.146731063	2.214268125	0.947035584	NA
ASV_16s_5800	1.202659846	1.341601588	3.549275718	1.231246749	NA	NA
ASV_16s_5801	0.474402293	29.11901067	3.550710075	-0.365040731	NA	NA
ASV_16s_5802	0.563736047	0.730336084	3.55068496	1.693611619	NA	NA
ASV_16s_5803	0.954393167	1.68135002	3.548720478	1.907155811	0.964799767	NA
ASV_16s_5804	1.012656391	0.132448187	3.526778665	1.991003745	0.96033661	NA
ASV_16s_5805	0.762092793	-0.109270966	3.548886279	1.658116911	0.976325795	NA
ASV_16s_5806	0.785864045	0.342355636	3.549501162	2.406475051	NA	NA
ASV_16s_5807	0.911840034	0.363424849	3.549880727	1.289676891	0.988713472	NA
ASV_16s_5808	1.040756622	0.693159989	3.550677102	2.547011283	NA	NA
ASV_16s_5809	0.857164172	-0.684589307	3.549255203	1.164061476	NA	NA
ASV_16s_5810	0.304322919	0.398980417	3.550658958	1.711006789	NA	NA
ASV_16s_5811	0.836123181	-0.881591286	3.548560253	1.849130935	0.967716657	NA
ASV_16s_5812	0.749244152	0.315562077	3.548423207	2.934771865	NA	NA
ASV_16s_5813	0.708924515	1.27807762	3.547858387	2.115822181	0.953152494	NA
ASV_16s_5814	0.974420024	1.205699146	3.54931793	1.152324621	NA	NA
ASV_16s_5815	0.926064929	3.406347717	3.546104651	0.276680867	0.999923953	NA
ASV_16s_5816	0.861290552	-0.834635251	3.274420557	2.500601616	0.927051756	NA
ASV_16s_5817	0.791520145	0.097329399	3.548533498	2.742752681	0.907736711	NA
ASV_16s_5818	0.848037253	-0.792943664	3.548650598	2.168824778	0.949908173	NA
ASV_16s_5819	0.810639481	-0.716974059	3.549249532	1.635902024	0.977228531	NA
ASV_16s_5820	0.800860696	0.631512592	3.549825715	1.700497024	0.974546892	NA
ASV_16s_5821	0.475847052	-0.473620019	3.550397111	1.4543548	0.98384796	NA
ASV_16s_5822	0.709765084	-1.397727393	3.548527909	0.71023967	0.998255817	NA
ASV_16s_5823	0.664320416	-0.199929656	3.547375011	2.065461551	NA	NA
ASV_16s_5824	0.448039904	1.91603044	3.549891355	0.537505741	NA	NA
ASV_16s_5825	0.661983641	1.901774438	3.549765984	0.867536539	0.99669209	NA
ASV_16s_5826	1.616496867	1.253542647	3.54932169	1.310947325	NA	NA
ASV_16s_5827	0.913259532	0.338240209	3.55066375	1.207724136	0.990747963	NA
ASV_16s_5828	0.950242544	0.325829586	3.550682974	1.343870213	0.987228782	NA
ASV_16s_5829	0.934380021	0.342809089	3.549388483	2.648496233	NA	NA
ASV_16s_5830	0.979207898	1.122107409	3.550210178	1.395131956	0.985719901	NA
ASV_16s_5831	0.73597015	-0.374618698	3.549316107	2.263024531	0.943860954	NA
ASV_16s_5832	0.20955935	-0.170044178	3.550633304	0.827567498	NA	NA
ASV_16s_5833	0.699442205	0.035376087	3.549116715	2.338148155	0.93878389	NA
ASV_16s_5834	0.706135468	0.20734089	3.548545347	2.613446673	0.918314961	NA
ASV_16s_5835	0.685792393	-0.075576758	3.548479635	2.331003358	0.939276362	NA
ASV_16s_5836	0.716026041	0.168859815	3.548420735	2.695549171	0.911666903	NA
ASV_16s_5837	0.702213891	0.598135786	3.548173889	2.302103771	NA	NA
ASV_16s_5838	0.690654517	-0.317116991	3.548871717	1.973223662	NA	NA
ASV_16s_5839	0.831897032	-1.321670917	3.547124805	1.592357753	0.978938928	NA
ASV_16s_5840	0.725291336	-0.008578704	3.548411549	2.474938501	0.928972368	NA
ASV_16s_5841	0.833541258	-0.412944742	3.396680445	1.522557513	0.981518428	NA
ASV_16s_5842	0.804749083	0.612739986	3.547479079	2.53770323	0.924231315	NA
ASV_16s_5843	0.785871018	-0.895408695	3.548677229	2.059570702	0.956469748	NA
ASV_16s_5844	0.819328863	0.141559218	3.547608823	2.225924125	0.946285328	NA
ASV_16s_5845	0.797796807	-0.798490014	3.549154807	1.613287047	0.978126585	NA
ASV_16s_5846	0.722081707	-0.698456498	3.54952955	1.692426869	NA	NA
ASV_16s_5847	0.784458397	0.789102574	3.547014227	1.228343938	NA	NA
ASV_16s_5848	0.844020231	-0.806794384	3.548646971	1.633005113	0.977344748	NA
ASV_16s_5849	0.770327333	-0.138733806	3.550021574	1.915234523	0.964382501	NA
ASV_16s_5850	0.838784914	0.297396429	3.549684704	2.672069192	0.913592707	NA
ASV_16s_5851	0.700450005	-0.76631317	3.547657568	1.72502315	0.973483316	NA
ASV_16s_5852	0.641917762	0.166346209	3.550266728	0.671128801	0.998547868	NA
ASV_16s_5853	0.712727362	0.403535848	3.54978923	1.087839391	NA	NA

ASV_16s_5854	0.747161272	0.082287379	3.547399794	2.221158132	0.94659276	NA
ASV_16s_5855	0.910386692	-0.263501006	3.548945226	1.922992967	NA	NA
ASV_16s_5856	0.352328177	0.654553858	3.550008518	1.362830406	NA	NA
ASV_16s_5857	0.691904179	0.235799942	3.548573555	1.881749996	0.966094219	NA
ASV_16s_5858	0.461411961	1.17932981	3.549437391	1.296815225	0.988524328	NA
ASV_16s_5859	0.591248301	0.815462459	3.549254214	1.49628184	NA	NA
ASV_16s_5860	1.003542603	0.818727556	3.54958162	2.346784648	NA	NA
ASV_16s_5861	0.849158974	0.315939149	3.5506719	1.674364118	0.975652628	NA
ASV_16s_5862	1.007805994	0.735246615	3.55068247	1.736794406	NA	NA
ASV_16s_5863	0.668474225	-0.278370733	3.549378354	2.28083988	NA	NA
ASV_16s_5864	0.677790462	-0.336933354	3.549361314	2.237287899	0.945548616	NA
ASV_16s_5865	0.208257739	-0.170041143	3.550634098	0.824482985	NA	NA
ASV_16s_5866	0.216830999	-0.170059897	3.550629044	0.844456954	0.996963342	NA
ASV_16s_5867	0.665189877	0.053926419	3.548744777	2.399269082	NA	NA
ASV_16s_5868	0.672661236	0.558774992	3.548805023	2.398523676	NA	NA
ASV_16s_5869	0.704797644	-0.102697764	3.548283375	2.441497153	NA	NA
ASV_16s_5870	0.577669469	1.852401109	3.548216426	0.663962559	0.998597569	NA
ASV_16s_5871	0.848405123	-0.794492687	3.548354889	2.066107997	0.956090921	NA
ASV_16s_5872	0.882823581	-0.794109764	3.548412075	2.162626519	0.950293488	NA
ASV_16s_5873	0.797641958	-0.889699434	3.54856206	2.029606421	0.958183545	NA
ASV_16s_5874	0.841870482	-0.793801745	3.548483915	2.129948555	0.952299027	NA
ASV_16s_5875	0.838560324	-0.793750014	3.548495543	2.128934587	0.95236056	NA
ASV_16s_5876	0.843640181	-0.793520974	3.548535272	2.146299729	0.951300955	NA
ASV_16s_5877	0.830221842	-0.381730365	3.548859146	2.301781277	0.941269604	NA
ASV_16s_5878	0.739687433	-0.737386603	3.549097602	1.763302508	0.971773253	NA
ASV_16s_5879	0.969031407	0.487405655	3.549490099	1.864950071	0.966935383	NA
ASV_16s_5880	0.787481833	1.117160389	3.549325912	1.895425413	0.965400792	NA
ASV_16s_5881	0.880946252	0.798069626	3.547520604	2.008486445	NA	NA
ASV_16s_5882	0.562422622	-0.147365902	3.54929558	2.029633909	0.95818199	NA
ASV_16s_5883	0.467669094	0.219978546	3.550638391	1.20689869	0.990767189	NA
ASV_16s_5884	0.477380806	0.219977769	3.55063713	1.217338271	0.990522193	NA
ASV_16s_5885	0.633952028	-0.948864008	3.548971101	1.581757077	0.979343513	NA
ASV_16s_5886	0.824411466	2.511294303	3.548358273	0.929801999	NA	NA
ASV_16s_5887	0.698506609	-0.069539556	3.549496852	2.412149426	0.933565766	NA
ASV_16s_5888	0.644819317	0.521231912	3.550963327	1.440520242	NA	NA
ASV_16s_5889	1.596540115	1.253522208	3.54932738	1.306932116	NA	NA
ASV_16s_5890	0.893337246	-0.540226462	3.548866597	2.04221729	0.957466794	NA
ASV_16s_5891	0.874478147	0.61712537	3.549356991	2.380845625	0.935799148	NA
ASV_16s_5892	0.768389479	0.315939326	3.550680792	1.623502898	0.97772352	NA
ASV_16s_5893	0.496121647	0.734861108	3.550792632	1.376342659	NA	NA
ASV_16s_5894	0.896395768	0.693899451	3.550635128	1.636364182	0.977209958	NA
ASV_16s_5895	0.657788203	-0.260201367	3.54939029	2.276157844	NA	NA
ASV_16s_5896	0.720951291	-0.360912087	3.54932724	2.264498005	0.943763529	NA
ASV_16s_5897	0.206956128	-0.170038047	3.550634902	0.821380554	NA	NA
ASV_16s_5898	0.732507122	-0.529115341	3.549251599	2.112237799	0.95336775	NA
ASV_16s_5899	0.703977701	0.120472588	3.548678001	2.517946404	0.925739631	NA
ASV_16s_5900	0.660982018	-0.160259705	3.548443506	2.109601068	NA	NA
ASV_16s_5901	0.714917567	0.480520067	3.548548407	2.52659482	0.925081169	NA
ASV_16s_5902	0.76386155	0.279163263	3.548443181	2.83728461	0.899635617	NA
ASV_16s_5903	0.821848444	-0.793935114	3.548464953	2.091908651	0.95457862	NA
ASV_16s_5904	0.852280032	-0.793840153	3.548497279	2.148351615	0.951174938	NA
ASV_16s_5905	0.675895958	1.457398712	3.547502018	1.888261695	NA	NA
ASV_16s_5906	0.834209028	-0.921922993	3.548339955	1.791651629	0.970467399	NA
ASV_16s_5907	0.828097258	-0.142301353	3.548222567	2.565634908	0.922074134	NA
ASV_16s_5908	0.861871355	0.782900592	3.3186975	2.341466506	0.938554482	NA
ASV_16s_5909	0.911509018	2.281953771	3.548083004	1.099081613	0.993068056	NA
ASV_16s_5910	0.874692516	-0.584308466	3.549876501	1.7144421	0.973945234	NA
ASV_16s_5911	0.699563868	1.431054476	3.549612383	0.992091177	0.99495511	NA
ASV_16s_5912	1.046144591	2.456587392	3.547922717	1.178341919	NA	NA
ASV_16s_5913	0.806999195	0.173204044	3.329455034	0.81207581	0.997318754	NA
ASV_16s_5914	0.780982384	-1.030155802	3.546849169	1.44802458	NA	NA
ASV_16s_5915	0.675132872	-0.077147452	3.547606645	2.334181765	NA	NA
ASV_16s_5916	0.978677083	3.211279476	3.546718294	0.385574369	NA	NA
ASV_16s_5917	0.919554828	1.256610376	3.544961616	1.150189418	0.992028923	NA
ASV_16s_5918	0.682532311	0.309795316	3.549978357	1.880685889	0.966147848	NA
ASV_16s_5919	0.538078113	0.123601621	3.550535131	1.602469842	NA	NA
ASV_16s_5920	1.031050183	0.544964311	3.549110164	2.421427857	0.932896512	NA

ASV_16s_5921	0.873335447	0.693892011	3.550637314	1.623095799	0.977739664	NA
ASV_16s_5922	0.891790572	0.693898215	3.550635556	1.633729948	0.977315702	NA
ASV_16s_5923	0.692083485	-0.292767954	3.549357068	2.303866228	0.941128508	NA
ASV_16s_5924	0.417814455	0.219984373	3.550645791	1.14966453	NA	NA
ASV_16s_5925	0.298383814	0.596224857	3.550642001	0.909770542	NA	NA
ASV_16s_5926	0.810807297	-0.746281247	3.548576194	2.015554039	NA	NA
ASV_16s_5927	0.71478257	-0.449719639	3.54875623	1.997246732	0.959992598	NA
ASV_16s_5928	0.862327862	-0.795158075	3.548291806	1.900708606	0.965130811	NA
ASV_16s_5929	0.879847007	-0.79512379	3.548268867	1.968960391	0.961538325	NA
ASV_16s_5930	0.737728803	0.019902497	3.548265315	2.772684155	NA	NA
ASV_16s_5931	0.726755571	-0.788314807	3.548491271	1.883775093	0.965992027	NA
ASV_16s_5932	0.7167761	-0.706851397	3.54822311	2.077117953	0.955448927	NA
ASV_16s_5933	0.804767685	-0.808595228	3.548291687	1.509928673	NA	NA
ASV_16s_5934	0.836150384	-0.794585589	3.548348785	2.027508543	NA	NA
ASV_16s_5935	0.843761685	-1.548019845	3.548418683	1.211606286	NA	NA
ASV_16s_5936	0.873378887	-0.790397245	3.548752752	2.012887038	0.959123656	NA
ASV_16s_5937	0.789494291	-0.761728558	3.548252902	2.640465973	0.916153802	NA
ASV_16s_5938	0.953467161	-0.451629178	3.549387346	3.264427861	0.859510868	NA
ASV_16s_5939	0.956714563	-0.121893137	3.158954712	1.371545943	0.986426887	NA
ASV_16s_5940	0.900932763	-0.065628163	3.549051572	2.777279111	0.904813124	NA
ASV_16s_5941	0.76374429	1.317804874	3.547974724	1.883723208	NA	NA
ASV_16s_5942	0.418875494	1.336829234	3.550026072	0.827797507	NA	NA
ASV_16s_5943	0.445821424	0.219980848	3.55064143	1.182596498	0.991322104	NA
ASV_16s_5944	0.474608194	0.219978067	3.550637485	1.214372199	NA	NA
ASV_16s_5945	0.622831576	0.664986036	3.54957145	2.082027983	0.955161012	NA
ASV_16s_5946	0.66602454	2.055400586	3.549102046	1.346611795	NA	NA
ASV_16s_5947	0.707681573	-0.456905642	3.548970744	2.126402204	0.952514054	NA
ASV_16s_5948	0.562012818	0.318207799	3.550799269	0.892546616	NA	NA
ASV_16s_5949	0.192838557	0.279455986	3.551558569	0.676666126	NA	NA
ASV_16s_5950	0.319163374	0.284364491	3.551229439	0.858192881	NA	NA
ASV_16s_5951	1.002631363	0.376214502	3.550504789	0.841802949	0.996993568	NA
ASV_16s_5952	0.91406367	0.315939264	3.550665895	1.711813875	0.974059247	NA
ASV_16s_5953	0.204352906	-0.170031683	3.550636542	0.815120995	NA	NA
ASV_16s_5954	0.284875013	0.220008969	3.550678158	0.955568048	NA	NA
ASV_16s_5955	0.665798079	0.129022753	3.548578877	2.517324263	NA	NA
ASV_16s_5956	0.659260125	0.033473357	3.548521463	2.464323554	NA	NA
ASV_16s_5957	0.684706254	0.18861879	3.548378158	2.704589151	NA	NA
ASV_16s_5958	0.668402369	0.695711415	3.54799306	2.272675789	NA	NA
ASV_16s_5959	0.696540645	0.775246608	3.54782092	2.358391042	0.937377713	NA
ASV_16s_5960	0.660478008	0.369302253	3.548434442	1.561555358	NA	NA
ASV_16s_5961	0.741671668	1.579576133	3.547378661	2.109118268	0.953554662	NA
ASV_16s_5962	0.689209343	1.686793392	3.547223447	1.833398302	NA	NA
ASV_16s_5963	0.815219956	-0.794234519	3.548414813	2.049263627	0.957063444	NA
ASV_16s_5964	0.741411316	-0.811592239	2.908489455	2.510814023	0.926280559	NA
ASV_16s_5965	0.838115835	-0.793864772	3.548472244	2.120329791	0.952881048	NA
ASV_16s_5966	0.834962915	-0.793577372	3.548529864	2.133447719	0.952086358	NA
ASV_16s_5967	0.809777678	0.350101992	3.548926363	2.425825248	0.93257817	NA
ASV_16s_5968	0.696063832	-1.043632559	3.548186646	1.453828391	0.983865213	NA
ASV_16s_5969	0.725480908	1.33780242	3.546873277	1.759701278	0.971936736	NA
ASV_16s_5970	0.952700717	1.463339185	3.136853891	2.066773995	0.956052228	NA
ASV_16s_5971	0.765819157	0.270503618	3.548554275	2.161306772	NA	NA
ASV_16s_5972	0.663255648	-0.96629254	3.336628492	1.442475444	0.984234616	NA
ASV_16s_5973	0.719079514	0.847360179	3.547747	1.581851483	NA	NA
ASV_16s_5974	0.689914905	-0.075100084	3.54986776	2.010768448	0.959241954	NA
ASV_16s_5975	0.860189061	0.395196564	3.550167809	0.617727126	NA	NA
ASV_16s_5976	0.231032587	0.406096209	3.550955217	0.653910293	NA	NA
ASV_16s_5977	0.925332697	2.689135454	3.548153903	1.398524083	NA	NA
ASV_16s_5978	0.657413205	0.612553789	3.54814599	1.50392279	NA	NA
ASV_16s_5979	0.731066479	-0.495741943	3.548461563	1.593173529	NA	NA
ASV_16s_5980	0.808878183	0.765082675	3.550250286	1.825821299	0.968848812	NA
ASV_16s_5981	0.446520489	-0.452290034	3.549682216	1.382047535	NA	NA
ASV_16s_5982	1.566604988	1.253490887	3.549336186	1.300813344	NA	NA
ASV_16s_5983	0.894226721	0.346050243	3.550511006	1.11682839	0.992717578	NA
ASV_16s_5984	0.701413018	-0.029644582	3.550635194	1.631859933	NA	NA
ASV_16s_5985	0.804058623	0.343431422	3.549514654	2.423274059	0.93276295	NA
ASV_16s_5986	0.921837101	0.562073278	3.550664596	2.847520924	NA	NA
ASV_16s_5987	0.95919529	0.924792862	3.549567357	1.892047912	0.965572778	NA

ASV_16s_5988	0.763888951	0.809194792	3.548908838	1.842216307	0.968054872	NA
ASV_16s_5989	0.947065645	0.693910213	3.550630698	1.664337295	0.97606936	NA
ASV_16s_5990	0.700479719	-0.21637727	3.549365534	2.351928885	0.937828354	NA
ASV_16s_5991	0.412525664	0.219985066	3.550646681	1.143190969	NA	NA
ASV_16s_5992	0.412525664	0.219985066	3.550646681	1.143190969	NA	NA
ASV_16s_5993	0.412525664	0.219985066	3.550646681	1.143190969	NA	NA
ASV_16s_5994	0.649264429	0.149715295	3.548852215	2.422413052	NA	NA
ASV_16s_5995	0.703663662	0.974411597	3.548210421	2.264200916	NA	NA
ASV_16s_5996	0.712711359	0.293513898	3.547787965	2.162385637	0.950308431	NA
ASV_16s_5997	0.829213166	-0.794128069	3.548427608	2.084979263	0.954987477	NA
ASV_16s_5998	0.843953452	-0.794060524	3.548434773	2.113671624	0.953281707	NA
ASV_16s_5999	0.794146664	0.928686568	3.548027575	2.406283101	0.93398719	NA
ASV_16s_6000	0.837993889	-0.793812744	3.548482674	2.123840047	0.952669085	NA
ASV_16s_6001	0.827743362	-0.502766889	3.164744857	1.525358557	0.981418739	NA
ASV_16s_6002	0.864131949	-2.433717834	3.546829348	0.819179309	0.99724326	NA
ASV_16s_6003	0.822404409	0.056055647	3.548945608	2.376195719	0.936127652	NA
ASV_16s_6004	0.801855121	-0.526522663	3.548504882	2.186189944	NA	NA
ASV_16s_6005	0.665226586	-0.882829456	3.548294196	1.501340634	0.982263211	NA
ASV_16s_6006	0.985190578	0.901410512	3.549504945	2.04632515	0.957231899	NA
ASV_16s_6007	0.292492446	0.370433457	3.551580689	0.479237439	NA	NA
ASV_16s_6008	0.446596717	0.25677247	3.550638276	1.640601952	0.977039244	NA
ASV_16s_6009	0.468600495	0.219978677	3.550638269	1.207894186	NA	NA
ASV_16s_6010	0.732736456	1.069707147	3.545932289	0.871175607	NA	NA
ASV_16s_6011	0.624918035	1.200606925	3.547959178	1.769843346	0.971474938	NA
ASV_16s_6012	0.631821392	2.035548036	3.549196322	0.886844191	0.996453409	NA
ASV_16s_6013	1.042124462	1.901742541	2.879316338	3.958105051	0.784590065	NA
ASV_16s_6014	0.6390644	0.710722447	3.550323717	1.684815042	NA	NA
ASV_16s_6015	0.382636888	0.296595124	3.550970778	0.919553401	NA	NA
ASV_16s_6016	0.646533215	0.241849983	3.549543867	3.187209748	0.867160534	NA
ASV_16s_6017	0.870955653	0.363327047	3.550160701	1.314955472	0.988034934	NA
ASV_16s_6018	0.894905875	0.684251859	3.549627465	2.559008986	0.922588472	NA
ASV_16s_6019	0.840947734	0.315939273	3.550672728	1.669388691	0.975859937	NA
ASV_16s_6020	0.685246238	-0.321292866	3.549357501	2.26643354	0.943635422	NA
ASV_16s_6021	0.633225474	-0.683768142	3.549439407	1.066427121	NA	NA
ASV_16s_6022	0.409881269	0.219985417	3.550647135	1.139923177	NA	NA
ASV_16s_6024	0.71380466	-0.082384475	3.549055125	2.202432652	0.947791785	NA
ASV_16s_6025	0.677985137	-0.152940044	3.549080554	2.083388445	NA	NA
ASV_16s_6026	0.706054794	0.555554144	3.548660562	2.49087845	NA	NA
ASV_16s_6027	0.67479784	1.28683549	3.547670003	2.046291013	NA	NA
ASV_16s_6028	0.846161819	-0.79401802	3.548439532	2.119333683	NA	NA
ASV_16s_6029	0.785954987	-1.096976438	3.548314731	1.661297777	0.976194864	NA
ASV_16s_6030	0.776881719	-1.125841745	3.549371143	1.192587665	0.991096565	NA
ASV_16s_6031	0.713517037	-0.055623735	3.550680175	1.211721434	0.990654505	NA
ASV_16s_6032	0.734540816	-0.067709285	3.550217403	1.619495237	0.977882155	NA
ASV_16s_6033	0.853121061	0.629193703	3.550403818	1.908239009	0.964743978	NA
ASV_16s_6034	0.890496183	0.857061586	3.549303723	2.440855329	NA	NA
ASV_16s_6035	0.474049749	0.242318394	3.550558028	0.74502536	0.99796536	NA
ASV_16s_6036	0.538333087	-0.761802617	3.548499685	1.74655839	0.972528785	NA
ASV_16s_6037	0.506149124	0.579900673	3.549889649	1.467467221	NA	NA
ASV_16s_6038	0.78160344	0.047270363	3.548647189	2.497112188	0.927314363	NA
ASV_16s_6039	1.137992586	0.326129446	3.550622105	3.459164145	0.839531658	NA
ASV_16s_6040	0.718375061	0.343263144	3.549508931	2.368844052	NA	NA
ASV_16s_6041	0.83868065	0.349027407	3.550669815	2.338274237	0.938775181	NA
ASV_16s_6042	0.628826425	-0.307974224	3.549406541	2.19607594	NA	NA
ASV_16s_6043	0.639501374	-0.346841672	3.549392231	2.167644539	NA	NA
ASV_16s_6044	0.177798913	-0.169952928	3.550655991	0.747005897	NA	NA
ASV_16s_6045	0.279431542	0.220010505	3.550680139	0.945796978	NA	NA
ASV_16s_6046	0.5889284	0.344730161	3.549078722	2.148216803	0.951183223	NA
ASV_16s_6047	0.683531366	-0.045997257	3.548235372	2.115794314	0.95315417	NA
ASV_16s_6048	0.820904667	-0.794797304	3.548339439	1.939742279	0.963100015	NA
ASV_16s_6049	0.75712482	-0.527184264	3.547988222	1.882282688	NA	NA
ASV_16s_6050	0.885261466	-0.393983951	3.550294898	1.924894785	0.963879973	NA
ASV_16s_6051	0.884337296	1.092645973	3.550304141	1.474020565	0.983195461	NA
ASV_16s_6052	0.754061036	-1.51079275	3.548159213	1.26604374	NA	NA
ASV_16s_6053	0.756801933	1.119330495	3.54862349	2.462870894	NA	NA
ASV_16s_6054	0.912848675	0.403603298	3.549597086	1.167601609	NA	NA
ASV_16s_6055	0.442786379	0.219981318	3.550641878	1.179111629	0.991399922	NA

ASV_16s_6056	0.447189805	0.219980819	3.550641233	1.184136006	0.991287587	NA
ASV_16s_6057	0.657956081	-0.06965216	3.549823051	2.169878106	0.949842538	NA
ASV_16s_6058	0.696375921	0.3249864	3.551525209	0.428824385	0.99966763	NA
ASV_16s_6059	0.765241859	2.606479363	3.548073349	0.842539658	NA	NA
ASV_16s_6060	0.832995553	0.919354906	3.550429185	1.758711737	0.971981562	NA
ASV_16s_6061	0.956863259	0.119270745	3.549451939	2.738551712	0.908089633	NA
ASV_16s_6062	0.671215418	0.342132651	3.549709298	2.218501032	0.94676376	NA
ASV_16s_6063	0.872877979	0.350591736	3.550434716	1.364885527	0.986622592	NA
ASV_16s_6064	0.638163718	-0.1092858	3.549455575	2.218251286	NA	NA
ASV_16s_6065	0.664855943	-0.224070586	3.549391421	2.299464153	0.94142621	NA
ASV_16s_6066	0.66278647	-0.227384116	3.549392376	2.295719342	0.941678857	NA
ASV_16s_6067	0.64120006	-0.313186316	3.549394709	2.209948095	NA	NA
ASV_16s_6068	0.209982761	0.220036496	3.550714396	0.802198247	NA	NA
ASV_16s_6070	0.076230704	0.686180329	3.551420757	0.413145421	0.999706486	NA
ASV_16s_6071	0.635083156	0.060832691	3.549180367	2.260825855	NA	NA
ASV_16s_6072	0.66820864	0.142756885	3.548664155	2.490600631	0.927803182	NA
ASV_16s_6073	0.754905891	0.041470677	3.548559057	2.582246612	0.920777539	NA
ASV_16s_6074	0.69361046	0.608124453	3.548809632	2.33464937	0.939025303	NA
ASV_16s_6075	0.723305782	0.113536372	3.548326234	2.785673578	0.904096139	NA
ASV_16s_6076	0.703709195	0.203034164	3.548339144	2.814930856	NA	NA
ASV_16s_6077	0.690179318	-0.170841021	3.547993081	2.480252495	0.928576716	NA
ASV_16s_6078	0.769222685	-0.024155308	3.341734794	2.133103839	0.95210728	NA
ASV_16s_6079	0.764692804	0.16652444	3.548571341	2.732263978	0.908616719	NA
ASV_16s_6080	0.77639586	-0.79233597	3.548789307	2.096872924	0.954284498	NA
ASV_16s_6081	0.79714532	-0.786338759	3.549147285	2.120338552	0.952880519	NA
ASV_16s_6082	0.694882185	-0.119907323	3.550446636	1.83787955	0.968265975	NA
ASV_16s_6083	0.703021436	-0.809243168	3.549369638	1.482727932	0.9829016	NA
ASV_16s_6084	0.580420955	-1.277434416	3.548060864	0.7560238	NA	NA
ASV_16s_6085	0.943032094	2.975607353	3.050706617	0.665212778	0.998588981	NA
ASV_16s_6086	0.797386455	-1.394482953	3.548383258	1.082922072	NA	NA
ASV_16s_6087	0.801300993	-0.304051319	3.547564641	2.13382726	NA	NA
ASV_16s_6088	0.695375469	1.33885807	3.549568138	0.990989095	NA	NA
ASV_16s_6089	0.425446357	0.219983385	3.550644547	1.158827747	0.99184419	NA
ASV_16s_6090	1.070681242	1.08634085	3.544730082	0.832617432	NA	NA
ASV_16s_6091	0.672325212	-0.268539869	3.549640732	1.576572265	NA	NA
ASV_16s_6092	0.52598609	-0.607763132	3.549925504	0.995864549	NA	NA
ASV_16s_6093	0.874381887	1.168358824	3.549946623	2.255844553	0.944334444	NA
ASV_16s_6094	0.895459113	0.078363354	3.549568578	2.708383827	0.910605994	NA
ASV_16s_6095	0.832904306	0.315939103	3.550673553	1.664483466	0.976063315	NA
ASV_16s_6096	0.891487746	1.178313374	3.550006308	1.294198941	0.988593876	NA
ASV_16s_6097	0.932135026	1.141900926	3.550165011	1.356283259	0.986872799	NA
ASV_16s_6098	0.206320566	1.140093179	3.550992662	0.658439888	0.998635086	NA
ASV_16s_6099	0.652545436	-0.313072208	3.549384998	2.227214204	0.946201954	NA
ASV_16s_6100	0.631842856	-0.348980466	3.549398907	2.153036301	0.950886582	NA
ASV_16s_6101	0.22167917	-0.652959312	3.550638469	0.726403667	NA	NA
ASV_16s_6102	0.208087863	-0.073953611	3.550636164	1.224841148	NA	NA
ASV_16s_6103	0.654100258	0.035958186	3.549226207	2.261176421	0.943983026	NA
ASV_16s_6104	0.731074864	0.037233141	3.548467189	2.559924807	0.922517477	NA
ASV_16s_6105	0.681908916	0.428006345	3.548388943	2.183487876	0.948990426	NA
ASV_16s_6106	0.694615748	0.606415087	3.548134696	2.484820738	0.928235735	NA
ASV_16s_6107	0.662150937	0.852871363	3.548425254	2.149124621	0.951127419	NA
ASV_16s_6108	0.668712393	0.040314845	3.548320584	2.634390502	NA	NA
ASV_16s_6109	0.660370875	1.424605005	3.547790147	1.949783844	0.96256731	NA
ASV_16s_6110	0.777303928	-0.79354738	3.548550069	2.053624107	0.956812812	NA
ASV_16s_6111	0.83155877	-0.859307019	3.548033121	1.75538743	0.972131856	NA
ASV_16s_6112	0.667032218	-0.294136296	3.549160186	1.796926479	NA	NA
ASV_16s_6113	0.806176935	-1.06563133	3.549507036	0.92543083	0.995943454	NA
ASV_16s_6114	0.679749596	-0.76858815	3.549263609	1.787424205	0.970664257	NA
ASV_16s_6115	0.896136662	0.014883063	3.549111236	1.027877169	NA	NA
ASV_16s_6116	0.799428104	0.834580653	3.548849181	1.995430838	0.960092828	NA
ASV_16s_6117	0.48901675	0.612739354	3.548219723	1.268094453	NA	NA
ASV_16s_6118	0.761514033	2.125059353	3.549236257	0.765278777	NA	NA
ASV_16s_6119	1.146711348	0.43970061	3.550626603	0.687154485	NA	NA
ASV_16s_6120	0.952150455	1.339687396	3.54937989	1.092273179	NA	NA
ASV_16s_6121	0.69343811	1.283095105	3.549390767	1.483670694	NA	NA
ASV_16s_6122	0.827021059	0.222309424	3.549557819	3.338511392	0.852022494	NA
ASV_16s_6123	0.828771698	0.503556775	3.548394645	2.071519176	0.955776018	NA

ASV_16s_6124	0.430547975	0.303637244	3.550813953	0.959215506	NA	NA
ASV_16s_6125	0.887341702	1.212995991	3.549863547	2.325514477	0.939653331	NA
ASV_16s_6126	0.724412301	0.212027241	3.549544136	2.657835805	0.914750586	NA
ASV_16s_6127	0.752307895	-0.116425048	3.549924571	2.008021592	0.959395057	NA
ASV_16s_6128	0.819242426	0.343013702	3.54944868	2.470141806	0.929328584	NA
ASV_16s_6129	0.688624766	0.360430052	3.550741473	1.606731192	NA	NA
ASV_16s_6130	0.688624766	0.360430052	3.550741473	1.606731192	NA	NA
ASV_16s_6131	0.779828302	0.315939164	3.550679424	1.630962331	0.97742649	NA
ASV_16s_6132	0.951469013	0.735224408	3.550688809	1.707468975	NA	NA
ASV_16s_6133	0.647914191	-0.22868094	3.549404594	2.273120687	0.943191668	NA
ASV_16s_6134	0.174335298	-0.169941273	3.550658965	0.737464569	NA	NA
ASV_16s_6135	0.399303688	0.219986812	3.55064901	1.12663891	NA	NA
ASV_16s_6136	0.405529002	0.596288401	3.550617683	1.065061207	0.993709639	NA
ASV_16s_6137	0.630514416	0.440750306	3.548885356	2.196808314	NA	NA
ASV_16s_6138	0.665931434	0.125813603	3.548615343	2.498172699	0.9272346	NA
ASV_16s_6139	0.625384765	-0.341351434	3.548631787	1.377661854	NA	NA
ASV_16s_6140	0.829631594	-0.794275607	3.548402874	2.069104147	0.95591671	NA
ASV_16s_6141	0.822393764	-0.793736901	3.548499281	2.106371354	0.953718916	NA
ASV_16s_6142	0.908955863	1.007424302	3.548174863	1.885106784	0.965924733	NA
ASV_16s_6143	0.846032538	-0.652558497	3.547040228	1.537611231	NA	NA
ASV_16s_6144	0.704042418	-0.093172789	3.54976808	1.659308549	NA	NA
ASV_16s_6145	0.72217877	0.307787833	3.550717815	1.210515024	0.990682773	NA
ASV_16s_6146	0.916953322	1.166680939	3.550056233	1.410190066	0.985257106	NA
ASV_16s_6147	0.76700794	0.281916254	3.54946366	2.495460533	0.927438503	NA
ASV_16s_6148	0.855199344	0.798317709	3.549668367	1.587129854	NA	NA
ASV_16s_6149	1.09217746	-1.323025707	3.547425774	1.971812787	NA	NA
ASV_16s_6150	0.803367472	0.108412566	3.54792661	2.550635201	0.923236165	NA
ASV_16s_6151	0.467530193	0.239573609	3.550629804	0.803108232	0.997412103	NA
ASV_16s_6152	0.741151935	-0.387709261	3.549139662	1.841622538	NA	NA
ASV_16s_6153	0.430491757	0.219982696	3.550643749	1.164802772	0.991714855	NA
ASV_16s_6154	0.436321002	0.219981908	3.550642848	1.171630656	0.991565494	NA
ASV_16s_6155	0.442330201	0.219981389	3.550641946	1.178575455	0.991411855	NA
ASV_16s_6156	0.764195745	0.318038555	3.550641826	1.325677585	0.98773975	NA
ASV_16s_6157	0.427715422	0.303322431	3.550823036	0.957009137	NA	NA
ASV_16s_6158	0.851486368	1.294193861	3.549612019	2.021647834	0.958632491	NA
ASV_16s_6159	0.966625825	0.142235833	3.548311155	2.279577229	0.942761525	NA
ASV_16s_6160	0.945209348	0.735221875	3.55068956	1.704104114	NA	NA
ASV_16s_6161	0.468214804	0.734816448	3.550805544	1.346904816	NA	NA
ASV_16s_6162	0.886948537	0.693896729	3.550636011	1.630946916	0.977427107	NA
ASV_16s_6163	0.662361396	-0.331146082	3.549374394	2.221736551	0.946555498	NA
ASV_16s_6164	0.63636932	-0.335660774	3.54939609	2.17716494	0.949387242	NA
ASV_16s_6165	0.630762219	-0.348562883	3.549399926	2.151911657	NA	NA
ASV_16s_6166	0.624184339	-0.351173668	3.54940576	2.138181323	0.951797866	NA
ASV_16s_6167	0.679195632	-0.421248888	3.549357585	2.097920643	0.954222294	NA
ASV_16s_6168	0.19524163	-0.170006505	3.550642623	0.792651058	NA	NA
ASV_16s_6169	0.19524163	-0.170006505	3.550642623	0.792651058	NA	NA
ASV_16s_6170	0.64407431	0.030650688	3.549372907	2.261601917	0.943954933	NA
ASV_16s_6171	0.172275583	-0.167146887	3.550751212	0.742222555	NA	NA
ASV_16s_6172	0.724973471	2.228431312	3.548222018	0.522148359	NA	NA
ASV_16s_6173	0.643657564	0.695171115	3.548511335	2.370981493	NA	NA
ASV_16s_6174	0.768761468	-0.970927299	3.548441217	1.619493463	0.977882225	NA
ASV_16s_6175	0.712502098	0.4222393	3.546677202	2.015018087	NA	NA
ASV_16s_6176	0.781128723	-0.793487772	3.548559371	2.063090943	0.956265974	NA
ASV_16s_6177	0.811628831	-0.793561441	3.548535892	2.102171551	0.95396945	NA
ASV_16s_6178	0.758211559	-0.791850429	3.548893493	2.081102894	0.955215334	NA
ASV_16s_6179	0.675210014	0.287961146	3.550679993	0.917930947	NA	NA
ASV_16s_6180	0.723338293	-9.141817028	3.548224462	0.218476558	0.999965971	NA
ASV_16s_6181	0.524996205	-0.754941574	3.549307041	1.251440808	0.989693858	NA
ASV_16s_6182	0.715446118	-0.067073662	3.550330951	1.603419268	0.978511835	NA
ASV_16s_6183	0.842008531	-0.13862142	3.550370129	3.157107459	0.870098031	NA
ASV_16s_6184	0.999965036	2.07105841	3.272522762	1.361865092	0.986710773	NA
ASV_16s_6185	0.669880824	0.461577948	3.547469872	2.228317156	0.94613062	NA
ASV_16s_6186	0.427493906	0.219983069	3.550644221	1.16125097	0.991791891	NA
ASV_16s_6187	0.512176178	-8.783108885	3.547901664	-0.697891826	1	NA
ASV_16s_6188	0.56627833	0.433062618	3.550921937	0.820441118	NA	NA
ASV_16s_6189	0.799093699	0.347939992	3.550739562	0.936980506	0.995782093	NA
ASV_16s_6190	1.496756358	1.253415476	3.549358098	1.286071767	NA	NA

ASV_16s_6191	0.875433265	0.885024654	3.550068453	1.345863904	0.987172009	NA
ASV_16s_6192	0.96040981	0.578819565	3.550669224	3.564258232	0.828373202	NA
ASV_16s_6193	0.756927657	0.31593922	3.55068221	1.615806274	0.978027589	NA
ASV_16s_6194	0.980467032	1.187537282	3.549582922	1.262003857	0.989428511	NA
ASV_16s_6195	0.465114044	0.734811269	3.550807074	1.343526639	NA	NA
ASV_16s_6196	0.875411803	0.693886782	3.550637115	1.624267374	0.977693185	NA
ASV_16s_6197	0.997792981	1.205748179	3.549305884	1.159931425	NA	NA
ASV_16s_6198	0.617068376	-0.141681834	3.549460811	2.209431865	NA	NA
ASV_16s_6199	0.6458615	-0.245572623	3.549402932	2.264716479	0.943749076	NA
ASV_16s_6200	0.619282328	-0.298879275	3.549416905	2.189464748	0.948613837	NA
ASV_16s_6201	0.188204193	-0.169984685	3.550647721	0.774676537	0.99769388	NA
ASV_16s_6202	0.209355611	0.22003681	3.550714811	0.800686911	0.997436937	NA
ASV_16s_6203	0.645591599	0.246627879	3.548747515	2.503942675	0.926799885	NA
ASV_16s_6204	0.722505267	-0.011313153	3.548401318	2.555963722	0.922824316	NA
ASV_16s_6205	0.615646748	0.04282284	3.548529401	1.295389837	NA	NA
ASV_16s_6206	0.769002187	-0.303332073	3.548595428	1.536904588	0.981004451	NA
ASV_16s_6207	0.70877135	0.212423456	3.549736305	1.662074142	0.976162844	NA
ASV_16s_6208	0.803179561	-0.748632266	3.548767323	1.550177726	0.980521489	NA
ASV_16s_6209	0.850863901	1.731170006	3.547426113	1.134071754	NA	NA
ASV_16s_6210	0.796789539	7.596357908	3.546399995	-0.488140796	1	NA
ASV_16s_6211	0.481384525	1.716753619	3.548868977	0.565952632	0.999167371	NA
ASV_16s_6212	0.79539127	1.84171848	3.548960381	0.976143832	NA	NA
ASV_16s_6213	0.695329713	1.402630539	3.549936893	0.948173415	0.995621813	NA
ASV_16s_6214	0.518225652	-0.147843123	3.548909303	1.749997728	NA	NA
ASV_16s_6215	0.781382646	-0.544404568	3.549149905	1.05937595	0.993813022	NA
ASV_16s_6216	0.643681788	-0.561892347	3.547785244	2.088008647	NA	NA
ASV_16s_6217	0.700310313	-0.374057956	3.548692147	2.920331286	0.89227309	NA
ASV_16s_6218	0.647032977	1.872578673	3.549084495	1.040159998	NA	NA
ASV_16s_6219	0.183967565	-0.511469316	3.551193371	0.649760976	NA	NA
ASV_16s_6220	0.885052905	0.846620659	3.549558722	1.575678002	0.979573447	NA
ASV_16s_6221	0.741686023	-0.116893349	3.549792088	2.065677066	0.956115947	NA
ASV_16s_6222	0.914165042	0.49064908	3.549450332	3.324603868	NA	NA
ASV_16s_6223	0.871554406	0.315939239	3.550669731	1.687541182	0.975098625	NA
ASV_16s_6224	0.610892351	-0.295082629	3.54942556	2.179377499	NA	NA
ASV_16s_6225	0.612693375	-0.320991333	3.549419689	2.157178343	NA	NA
ASV_16s_6226	0.203120579	0.220040085	3.550719052	0.785688893	NA	NA
ASV_16s_6228	0.668120311	0.370905	3.548595061	2.363741986	0.937003326	NA
ASV_16s_6229	0.620366924	0.110154785	3.548423976	2.559474221	NA	NA
ASV_16s_6230	0.902149879	2.430687937	3.547767856	0.566077176	0.99916677	NA
ASV_16s_6231	0.801571699	-0.794172885	3.548428252	2.030862766	0.958112435	NA
ASV_16s_6232	0.785161692	-0.253766095	3.322676094	2.60082353	0.919315565	NA
ASV_16s_6233	0.733232792	1.306725493	3.547225639	1.718695745	0.9737601	NA
ASV_16s_6234	0.792960696	-0.486334882	3.314804759	2.497241015	0.927304676	NA
ASV_16s_6235	0.749135398	-1.033261747	3.548292269	1.602135109	NA	NA
ASV_16s_6236	0.670379219	-0.391719812	3.549264318	1.575036157	NA	NA
ASV_16s_6237	0.392364387	1.336490211	3.550103593	0.806729949	NA	NA
ASV_16s_6238	0.593002281	0.126146399	3.548546335	2.327528059	0.939515179	NA
ASV_16s_6239	0.339411712	0.287945809	3.551532048	0.619110144	NA	NA
ASV_16s_6240	1.476799607	1.253391247	3.549364738	1.281732798	NA	NA
ASV_16s_6241	1.046272171	0.314032017	3.547046273	1.886946549	NA	NA
ASV_16s_6242	0.836659018	0.341699597	3.550647091	1.146580561	0.992105314	NA
ASV_16s_6243	1.153229893	0.596993154	3.549348306	2.757809656	NA	NA
ASV_16s_6244	0.670377119	-0.07438017	3.55060351	1.573748074	0.979646127	NA
ASV_16s_6245	0.751270506	0.360834139	3.550313264	1.264794752	0.989357705	NA
ASV_16s_6246	0.8398804	0.693878369	3.550640702	1.603180178	0.978521119	NA
ASV_16s_6247	0.632338003	-0.225081208	3.549419153	2.249899995	0.944724902	NA
ASV_16s_6248	0.653786844	-0.258312188	3.549393994	2.270916049	0.943338163	NA
ASV_16s_6249	0.607256414	-0.244640166	3.549438521	2.204546752	NA	NA
ASV_16s_6250	0.608113028	-0.329293521	3.549423085	2.140521534	NA	NA
ASV_16s_6251	0.605350482	-0.349074352	3.549423926	2.111310791	NA	NA
ASV_16s_6252	0.266730108	0.220014253	3.550685077	0.922255128	NA	NA
ASV_16s_6253	0.622418407	-0.139239788	3.549402854	2.195474149	NA	NA
ASV_16s_6254	0.615143623	0.018268047	3.549384459	2.147439274	0.951230991	NA
ASV_16s_6255	0.613086238	0.090479128	3.549217488	2.244872677	NA	NA
ASV_16s_6256	0.662264141	0.057729382	3.548639902	2.415957477	0.933291493	NA
ASV_16s_6257	0.615929511	0.01653659	3.548730089	2.290796471	NA	NA
ASV_16s_6258	0.636436084	-0.043664052	3.54853115	2.311725496	0.940595096	NA

ASV_16s_6259	0.632619025	0.308260563	3.548571245	2.262179455	0.94391679	NA
ASV_16s_6260	0.611543637	-0.340578049	3.548542178	1.465573151	NA	NA
ASV_16s_6261	0.643253667	0.051527254	3.548425483	2.514700909	NA	NA
ASV_16s_6262	0.623531346	0.849169104	3.548735424	2.076902538	0.955461536	NA
ASV_16s_6263	0.654194617	1.056656274	3.548161569	1.950725029	NA	NA
ASV_16s_6264	0.715032531	-1.025021889	3.548698006	1.682819034	NA	NA
ASV_16s_6265	0.789068044	-0.793471965	3.548562299	2.0763245	0.955495361	NA
ASV_16s_6266	0.795303189	-0.792979739	3.548654145	2.105410241	NA	NA
ASV_16s_6267	0.789411102	-0.793076094	3.548636678	2.094579079	0.954420529	NA
ASV_16s_6268	0.745002366	-0.848393064	3.54857815	1.717887693	0.973795328	NA
ASV_16s_6269	0.746846281	-0.791073465	3.549057913	2.071133954	0.955798476	NA
ASV_16s_6270	0.694462689	-0.152324902	3.549753146	1.43085893	NA	NA
ASV_16s_6271	0.960722387	0.929132523	3.549605593	1.656659312	NA	NA
ASV_16s_6272	0.697870187	1.443363455	3.54814012	1.505726429	0.982110749	NA
ASV_16s_6273	0.841448125	2.956045058	3.302771423	0.930372003	0.995874919	NA
ASV_16s_6274	0.582819105	0.201028393	3.549199762	2.210770968	NA	NA
ASV_16s_6275	0.440066318	-1.467557434	3.549000271	0.525509592	NA	NA
ASV_16s_6276	0.410286897	0.219985271	3.550647067	1.140385739	0.992235366	NA
ASV_16s_6277	0.622367023	-0.069497788	3.549576672	2.294855304	NA	NA
ASV_16s_6278	0.639214646	-0.06930265	3.549553218	2.279558401	NA	NA
ASV_16s_6279	0.192014006	-0.52170071	3.551156935	0.664157391	NA	NA
ASV_16s_6280	0.86542113	0.700124109	3.549523088	2.228790983	0.94609996	NA
ASV_16s_6281	0.746154044	0.315939288	3.550683582	1.608497148	0.978314089	NA
ASV_16s_6282	0.832718918	0.501741965	3.550663718	3.030805616	0.882137903	NA
ASV_16s_6283	0.820349201	0.693871471	3.550642806	1.59120101	0.978983301	NA
ASV_16s_6284	0.595944177	-0.121551656	3.549491543	2.161665596	NA	NA
ASV_16s_6285	0.619530858	-0.152808151	3.549453841	2.219192754	0.946719271	NA
ASV_16s_6286	0.596121003	-0.273022047	3.549444768	2.171781183	NA	NA
ASV_16s_6287	0.132090045	-0.169745133	3.550707716	0.607055822	NA	NA
ASV_16s_6289	0.677233083	0.070678688	3.548811568	2.435557111	0.931871004	NA
ASV_16s_6290	0.665672504	-0.018613018	3.548894847	2.246611956	0.944940264	NA
ASV_16s_6291	0.617118314	0.044223846	3.548566009	2.398948908	0.934512192	NA
ASV_16s_6292	0.686479229	0.243773564	3.548514989	2.799145318	NA	NA
ASV_16s_6293	0.703537046	-0.014061673	3.548310162	2.616035849	0.918109007	NA
ASV_16s_6294	0.631492581	1.327466064	3.547948027	2.02122626	NA	NA
ASV_16s_6295	0.769949743	-0.794395114	3.548416655	1.922170534	0.964022083	NA
ASV_16s_6296	0.754982901	-0.793627957	3.548546984	2.012794926	0.959128803	NA
ASV_16s_6297	0.977354467	1.205705397	3.549316433	1.153246473	NA	NA
ASV_16s_6298	0.802199473	-0.793662138	3.548520245	2.082631304	0.955125566	NA
ASV_16s_6299	0.75966079	-0.793088458	3.548645567	2.054110072	0.956784831	NA
ASV_16s_6300	0.775966237	-0.852673038	3.302650348	1.886152891	0.965871818	NA
ASV_16s_6301	0.783278596	-0.182825651	3.546720986	1.1810873	NA	NA
ASV_16s_6302	0.571337348	0.899719378	3.5488847	1.642413684	0.976966034	NA
ASV_16s_6303	0.869126782	0.851165967	3.069392916	2.587183766	0.920390225	NA
ASV_16s_6304	0.870967628	-0.444273678	3.54994679	3.37129339	0.848664012	NA
ASV_16s_6305	0.83931605	-0.206764323	3.549196179	2.334542411	0.939032675	NA
ASV_16s_6306	1.005333529	0.097224674	3.547882753	2.263066992	0.943858147	NA
ASV_16s_6307	0.647347687	0.150384307	3.131234287	2.497499015	0.927285274	NA
ASV_16s_6308	0.646324191	0.40350431	3.549879192	1.056266795	NA	NA
ASV_16s_6309	0.563132479	0.412139583	3.549342702	2.808736552	0.902113982	NA
ASV_16s_6310	0.754815419	1.61632893	3.548283723	2.122016085	NA	NA
ASV_16s_6311	0.902681864	1.39632888	3.548765289	1.176699534	0.991453527	NA
ASV_16s_6312	0.667887979	-0.068919055	3.549624301	2.205489344	NA	NA
ASV_16s_6313	0.775805556	-0.072505229	3.549164419	2.143207383	0.951490549	NA
ASV_16s_6314	0.799792964	0.746013885	3.549523176	2.740222234	0.907949366	NA
ASV_16s_6315	0.848511005	0.343915601	3.549618751	2.356284329	0.937524805	NA
ASV_16s_6316	0.749270548	0.315939307	3.550683182	1.610612423	0.978231401	NA
ASV_16s_6317	0.885904009	1.187172689	3.549668264	1.224815111	0.990344266	NA
ASV_16s_6318	0.788498698	0.693857803	3.550646461	1.571033828	0.979748087	NA
ASV_16s_6319	0.617925233	-0.12310366	3.549468348	2.198065947	NA	NA
ASV_16s_6320	0.613686507	-0.238490003	3.549433554	2.217004305	0.946859958	NA
ASV_16s_6321	0.591990045	-0.231590465	3.549456553	2.183696952	NA	NA
ASV_16s_6322	0.611550176	-0.362097522	3.549417096	2.102770468	0.953933766	NA
ASV_16s_6323	0.688529993	-0.344136769	3.548459732	2.01866098	0.958800303	NA
ASV_16s_6324	0.63646196	0.051906602	3.54854358	2.437792765	0.931708037	NA
ASV_16s_6325	0.783379208	-0.63153478	3.547505798	2.269711138	0.943418146	NA
ASV_16s_6326	0.732320179	-0.792849576	3.54869818	2.024529138	0.958470259	NA

ASV_16s_6327	0.685491353	-0.559559849	3.548809044	1.952189107	0.962439087	NA
ASV_16s_6328	0.786832701	-0.995200345	2.919518042	2.010671597	0.959247358	NA
ASV_16s_6329	0.776638427	-0.791951992	3.548868177	2.101350469	0.954018345	NA
ASV_16s_6330	0.612265099	-0.488183591	3.550401378	1.253863738	NA	NA
ASV_16s_6331	0.805950492	0.678683674	3.550656735	1.749870117	0.97238028	NA
ASV_16s_6332	0.762902019	-0.047674935	3.549198711	2.203794869	0.947705037	NA
ASV_16s_6333	0.684634065	1.438625025	3.549114903	1.45646472	NA	NA
ASV_16s_6334	0.664699367	0.629669416	3.548686416	1.905257359	NA	NA
ASV_16s_6335	0.564270875	0.1620735	3.549406152	2.226514228	NA	NA
ASV_16s_6336	0.649204679	-0.231007041	3.549207449	1.385736108	NA	NA
ASV_16s_6337	0.502006212	-1.379502119	3.54883502	0.849523815	NA	NA
ASV_16s_6338	0.303793247	1.326267354	3.550420987	0.723440301	NA	NA
ASV_16s_6339	0.61093872	-0.079337777	3.550603935	1.502284586	0.982230462	NA
ASV_16s_6340	0.410719378	0.219985217	3.550646993	1.140910462	0.992224403	NA
ASV_16s_6341	0.793514501	0.861333527	3.54964446	1.535793406	0.981044558	NA
ASV_16s_6342	1.02498371	0.739144564	3.549513556	2.745059879	0.907542625	NA
ASV_16s_6343	0.927763098	0.625409668	3.550687598	3.002382624	0.884781732	NA
ASV_16s_6344	0.785948211	0.386702827	3.55067253	2.648194203	0.915530815	NA
ASV_16s_6345	0.701860176	0.315939319	3.550689657	1.577353074	0.97951024	NA
ASV_16s_6346	0.632019854	-0.234061755	3.549417463	2.247197472	0.944901945	NA
ASV_16s_6347	0.596812869	-0.273362302	3.549444001	2.172667693	NA	NA
ASV_16s_6348	0.826562623	-0.684506738	3.549274584	1.152278326	NA	NA
ASV_16s_6349	0.187431965	-0.169984386	3.550648306	0.772647406	NA	NA
ASV_16s_6350	0.172577645	-0.169932367	3.550660522	0.732541485	0.998073018	NA
ASV_16s_6351	0.166253529	-0.169910701	3.550666384	0.714579815	NA	NA
ASV_16s_6352	0.631380307	-0.260536617	3.548904039	1.773816448	0.971292861	NA
ASV_16s_6353	0.663107035	-0.978167756	3.548600514	1.670672937	0.975806525	NA
ASV_16s_6354	0.765404398	-0.793597736	3.548553827	2.034720127	0.957893696	NA
ASV_16s_6355	0.729437239	-0.792613335	3.54874528	2.029303855	0.958200661	NA
ASV_16s_6356	0.643952572	1.538650128	3.546744092	0.94231003	NA	NA
ASV_16s_6357	0.687700073	-0.647275997	3.549181487	1.5749241	0.979601857	NA
ASV_16s_6358	0.676373449	0.219272786	3.548570393	2.295248994	NA	NA
ASV_16s_6359	0.68875579	-1.95955466	3.547873652	0.566725026	NA	NA
ASV_16s_6360	0.409231325	0.219985372	3.55064725	1.13906366	0.992262946	NA
ASV_16s_6361	0.75905778	-2.678431138	3.546378031	0.308257338	0.99989034	NA
ASV_16s_6362	0.608775745	-0.06949709	3.549594203	2.268408682	NA	NA
ASV_16s_6363	0.615217483	-0.069460515	3.549555749	2.293230626	NA	NA
ASV_16s_6364	0.628077369	0.179311606	3.547408658	1.406514369	NA	NA
ASV_16s_6365	0.746665848	0.54471378	3.549251829	3.051356762	0.880210994	NA
ASV_16s_6366	0.91301588	0.76198375	3.549732276	2.794188133	0.903366449	NA
ASV_16s_6367	0.407887555	0.300907646	3.550888708	0.941123571	NA	NA
ASV_16s_6368	1.036757733	0.796718844	3.549573773	2.745062893	NA	NA
ASV_16s_6369	0.787318081	0.143986207	3.549487769	3.561362682	NA	NA
ASV_16s_6370	0.80415462	0.693866239	3.550644628	1.581040103	0.97937071	NA
ASV_16s_6371	0.817779835	0.693870463	3.550643092	1.58957727	0.979045494	NA
ASV_16s_6372	0.656460726	-0.246222215	3.549393857	2.280305318	NA	NA
ASV_16s_6373	0.63651812	-0.296770567	3.54940164	2.218079915	0.946790835	NA
ASV_16s_6374	0.619988655	-0.29426969	3.54941713	2.194450359	0.948298598	NA
ASV_16s_6375	0.591666816	-0.47458725	3.549443879	1.848563571	NA	NA
ASV_16s_6376	0.16509899	-0.169906349	3.550667503	0.711234797	NA	NA
ASV_16s_6377	0.198089883	-0.170015528	3.550640667	0.799730977	NA	NA
ASV_16s_6378	0.183680583	0.687752946	3.550915792	0.810252848	NA	NA
ASV_16s_6379	0.66333293	-0.345630532	3.548788265	1.957317404	0.962164897	NA
ASV_16s_6380	0.672355635	0.171517313	3.548540425	2.727512255	0.909014134	NA
ASV_16s_6381	0.668758794	0.435775706	3.548337878	2.383569772	0.935606303	NA
ASV_16s_6382	0.790649683	-0.183351294	3.548468113	2.063887879	0.956219771	NA
ASV_16s_6383	0.608976592	-0.403419712	3.548896309	1.863653793	NA	NA
ASV_16s_6384	0.624666235	1.269531638	3.548398471	2.143235001	NA	NA
ASV_16s_6385	0.741974434	0.236204418	3.549016217	2.856557462	0.897947117	NA
ASV_16s_6386	0.719900725	-0.299796855	3.302280327	2.238350809	0.945479442	NA
ASV_16s_6387	0.869605174	-2.735887314	3.545175536	0.512211375	0.999400528	NA
ASV_16s_6388	0.727866606	-0.77587407	3.549262826	2.04473856	0.957322706	NA
ASV_16s_6389	0.665811898	-0.098991524	3.549723016	1.795403006	0.970292083	NA
ASV_16s_6390	0.908059875	1.804467762	2.610875084	2.428549309	0.932380593	NA
ASV_16s_6391	0.729853679	-0.380610476	3.549151388	1.663340166	NA	NA
ASV_16s_6392	0.654456693	-0.057984368	3.547572637	1.246695656	NA	NA
ASV_16s_6393	0.638783933	1.250283072	3.548840574	0.674497979	NA	NA

ASV_16s_6394	0.749645051	0.90013787	3.550423355	1.405618122	0.985398565	NA	
ASV_16s_6395	0.737001787	0.315939201	3.550684778	1.602195313	0.97855934	NA	
ASV_16s_6396	0.713330343	0.315939196	3.550688012	1.585586489	0.979197892	NA	
ASV_16s_6397	0.69889818	0.315939351	3.550690091	1.575191254	0.979591792	NA	
ASV_16s_6398	0.768870204	0.69385163	3.550648864	1.558196389	0.980226238	NA	
ASV_16s_6399	0.78805699	0.693854565	3.550646514	1.570738219	0.979759173	NA	
ASV_16s_6400	0.601709084	-0.188900013	3.54945837	2.201962523	0.947821706	NA	
ASV_16s_6401	0.60459993	-0.370276556	3.549423611	2.079349765	NA	NA	
ASV_16s_6402	0.270371606	0.11625577	3.550650365	1.548701756	0.980575549	NA	
ASV_16s_6403	0.647953975	-0.004763591	3.549335697	2.174048427	0.949582232	NA	
ASV_16s_6404	0.651915761	0.140061358	3.548453209	2.58453311	NA	NA	
ASV_16s_6405	0.602758541	0.591255927	3.54851453	2.18248104	NA	NA	
ASV_16s_6406	0.698635661	-0.473932576	3.548616795	1.933623713	0.963422545	NA	
ASV_16s_6407	0.620702899	-0.342472711	3.548137941	1.996778668	0.960018447	NA	
ASV_16s_6408	0.617707315	1.151502364	3.548369897	2.009116988	NA	NA	
ASV_16s_6409	0.786891766	-0.794696466	3.548380885	1.882178948	0.966072587	NA	
ASV_16s_6410	0.757582954	-0.793896385	3.548518375	2.002140957	0.959721777	NA	
ASV_16s_6411	0.577203575	0.316121582	3.547692842	1.363491799	0.986663326	NA	
ASV_16s_6412	0.66227367	-0.923530213	3.549238848	1.296273451	NA	NA	
ASV_16s_6413	0.76866492	-0.840312594	3.549145961	1.219633863	0.990467784	NA	
ASV_16s_6414	0.726748002	-0.780809148	3.549222006	2.045655232	0.957270254	NA	
ASV_16s_6415	0.684247588	-0.440752314	3.55001784	1.075216327	0.993522257	NA	
ASV_16s_6416	0.630323627	1.250045427	3.549703887	1.308827957	0.988201649	NA	
ASV_16s_6417	0.776996037	0.682546127	3.550585544	1.844498138	0.967943482	NA	
ASV_16s_6418	0.633184029	-0.192372205	3.549371944	1.63171348	0.977396453	NA	
ASV_16s_6419	0.785399637	0.85977211	3.550485266	1.522035695	0.981536964	NA	
ASV_16s_6420	0.628616679	0.403494796	3.54990637	1.047298769	NA	NA	
ASV_16s_6421	0.401797808	0.219986385	3.550648561	1.129751576	0.992455457	NA	
ASV_16s_6422	0.605661113	-0.069455223	3.549566123	2.27736362	NA	NA	
ASV_16s_6423	0.52616478	-0.781367469	3.54909134	1.404402388	NA	NA	
ASV_16s_6424	0.622508736	-0.069058195	3.549609835	2.192995045	NA	NA	
ASV_16s_6425	1.416929353	1.253310795	3.549385776	1.268354216	NA	NA	
ASV_16s_6426	0.679848318	0.339291287	3.550187444	0.893395632	0.996369946	NA	
ASV_16s_6427	0.55458764	-0.06960122	3.549924939	2.015105633	NA	NA	
ASV_16s_6428	1.025181912	-0.126292015	3.548650458	2.363584852	NA	NA	
ASV_16s_6429	0.879000457	0.344030263	3.549628856	2.24322909	0.945161383	NA	
ASV_16s_6430	0.860782371	1.153512934	3.55013038	1.306281666	0.988270506	NA	
ASV_16s_6431	0.583837177	-0.302958365	3.549451398	2.128035909	0.952415061	NA	
ASV_16s_6432	0.183527133	-0.16997249	3.550651328	0.762362301	NA	NA	
ASV_16s_6433	0.587467909	6.65E-05	3.549459758	2.146797732	NA	NA	
ASV_16s_6434	0.653919155	-0.515259684	3.549327518	2.009743148	0.959299138	NA	
ASV_16s_6435	0.050077316	0.618664388	3.551840832	0.299597691	0.999900416	NA	
ASV_16s_6436	0.647155524	-0.044172647	3.548901938	2.174896358	0.949529219	NA	
ASV_16s_6437	0.639612806	0.357792658	3.549244974	2.274155426	0.943122844	NA	
ASV_16s_6438	0.654759516	-0.373704603	3.548607356	1.879653183	0.966199849	NA	
ASV_16s_6439	0.602415657	-0.290792331	3.549209976	1.868898259	NA	NA	
ASV_16s_6440	0.782601984	-0.794930653	3.548418943	1.740445797	0.972801685	NA	
ASV_16s_6441	0.763752469	-0.79464377	3.548412941	1.832894208	0.968507679	NA	
ASV_16s_6442	0.650404628	-0.532079965	3.548644789	1.881496428	0.966107003	NA	
ASV_16s_6443	0.720648737	-0.794333685	3.548478011	1.812137944	0.969502837	NA	
ASV_16s_6444	0.73969837	-0.793649383	3.548514377	1.959322864	0.962057374	NA	
ASV_16s_6445	0.771632772	-0.793992635	3.54846681	1.995638736	0.96008136	NA	
ASV_16s_6446	0.768839953	-0.793087186	3.548640473	2.066385128	0.956074823	NA	
ASV_16s_6447	0.721469242	-0.792725165	3.548726669	2.013756882	0.959075031	NA	
ASV_16s_6448	0.719279641	0.809777984	3.54812574	2.317422108	0.940206937	NA	
ASV_16s_6449	0.706749991	-0.081099514	3.550754211	0.990326617	NA	NA	
ASV_16s_6450	0.717348466	-0.730068489	3.548774229	2.064265953	0.956197843	NA	
ASV_16s_6451	0.781034126	0.71397052	3.549940452	2.018425525	0.958813516	NA	
ASV_16s_6452	0.589425036	-0.160324227	3.549889458	2.235589561	NA	NA	
ASV_16s_6453	0.833804173	-0.967055584	2.956607971	1.349077752	0.987080167	NA	
ASV_16s_6454	0.723076028	-0.321658163	3.549784448	1.464150487	NA	NA	
ASV_16s_6455	0.828669133	-0.318772757	3.5469978	1.445526353	0.984135851	NA	
ASV_16s_6456	0.730883834	0.76752849	3.547929902	2.472583107	NA	NA	
ASV_16s_6457	0.510870859	-0.132746371	3.548579985	1.628994253	NA	NA	
ASV_16s_6458	1.117189162	0.314517678	3.550622582	3.437993242	0.841748874	NA	
ASV_16s_6459	0.881083355	0.344088857	3.54964295	2.228541186	NA	NA	
ASV_16s_6460	0.696694868	0.459972447	3.550651663	2.545927822	0.923599129	NA	

ASV_16s_6461	0.711275949	0.31593925	3.550688304	1.584108706	0.979254159	NA
ASV_16s_6462	0.713039202	0.581972145	3.550665225	2.655193013	0.91496478	NA
ASV_16s_6463	0.437207201	0.734760255	3.550821817	1.312072414	NA	NA
ASV_16s_6464	0.80146061	0.693864656	3.55064494	1.579312981	0.97943614	NA
ASV_16s_6465	0.582952378	-0.221524653	3.549468419	2.170635024	NA	NA
ASV_16s_6466	0.580494383	-0.285689675	3.549458187	2.136829359	0.951880356	NA
ASV_16s_6467	0.59748496	-0.314373846	3.549435574	2.139506178	0.951716958	NA
ASV_16s_6468	0.586378832	-0.683511821	3.549495696	1.041601573	NA	NA
ASV_16s_6469	0.192141088	0.220046254	3.550727191	0.758236928	NA	NA
ASV_16s_6470	0.646047159	-0.275301961	3.549290212	1.900814073	0.965125409	NA
ASV_16s_6471	0.6134361	0.016298634	3.548909926	2.208460435	0.94740736	NA
ASV_16s_6472	0.589266685	0.129748386	3.548793802	2.343943382	NA	NA
ASV_16s_6473	0.681552733	-0.310533374	3.548632438	1.967587946	0.961612477	NA
ASV_16s_6474	0.585469649	-0.340119535	3.548554274	1.480996869	0.982960264	NA
ASV_16s_6475	0.674653833	0.226145121	3.548501226	2.784681997	0.904180956	NA
ASV_16s_6476	0.67050579	-0.66710162	3.548691071	1.868723385	NA	NA
ASV_16s_6477	0.654861292	0.089142055	3.548458965	2.63765244	0.916380073	NA
ASV_16s_6478	0.7586745	-0.921701622	3.548243635	1.687609377	0.975095739	NA
ASV_16s_6479	0.7316914	-0.792745821	3.54871911	2.027784055	0.958286577	NA
ASV_16s_6480	0.725266816	-0.792466184	3.547694895	2.029694886	0.95817854	NA
ASV_16s_6481	0.584945879	-0.893584232	3.548847266	1.658169923	0.976323617	NA
ASV_16s_6482	0.629012145	0.23794949	3.548783486	2.495694131	NA	NA
ASV_16s_6483	0.722985618	-0.791849153	3.548901974	2.037956407	0.957709701	NA
ASV_16s_6484	0.75659568	-0.463855868	3.548207107	2.133837214	0.952062655	NA
ASV_16s_6485	0.76724103	-0.71775083	3.549259231	1.604092626	0.978485674	NA
ASV_16s_6486	0.753325214	0.327186635	3.550744161	0.924736946	0.995953019	NA
ASV_16s_6487	0.690926745	1.083138203	3.064759292	2.316648242	0.940259743	NA
ASV_16s_6488	0.664027261	2.344876853	3.549093332	0.622669279	NA	NA
ASV_16s_6489	1.032959377	1.341256907	3.549355164	1.182094905	NA	NA
ASV_16s_6490	0.991363968	-2.278301098	3.546871823	0.600090731	NA	NA
ASV_16s_6491	0.20601632	0.395982945	3.551094977	0.615600154	NA	NA
ASV_16s_6492	0.293317618	1.320607187	3.550453633	0.711435132	NA	NA
ASV_16s_6493	0.403224673	0.219986175	3.550648306	1.131540118	0.992418719	NA
ASV_16s_6494	0.420538906	0.219984058	3.550645346	1.152878389	NA	NA
ASV_16s_6495	0.614330478	-0.062307428	3.549998011	1.805651033	NA	NA
ASV_16s_6496	0.770353295	0.718152704	3.549679204	2.156937123	0.950645793	NA
ASV_16s_6497	0.28280299	0.279948019	3.551367939	0.815940693	NA	NA
ASV_16s_6498	0.804486806	0.377561061	3.548414279	2.116280924	0.953124907	NA
ASV_16s_6499	0.967313706	0.195517453	3.549648725	2.669274371	0.913820634	NA
ASV_16s_6500	0.718836051	0.790981883	3.548603887	2.004747922	NA	NA
ASV_16s_6501	0.763892871	0.709677362	3.549527872	2.21212589	NA	NA
ASV_16s_6502	0.718296686	0.345354512	3.550548069	1.296259908	0.988539111	NA
ASV_16s_6503	1.04310075	0.959338214	3.549487322	2.593353463	NA	NA
ASV_16s_6504	0.634259653	0.360426109	3.550754146	1.564836266	NA	NA
ASV_16s_6505	0.896784338	0.68698775	3.55069257	2.460345205	NA	NA
ASV_16s_6506	0.779503704	0.31593922	3.550679467	1.630695159	0.977437169	NA
ASV_16s_6507	0.814884546	0.693868332	3.550643415	1.587753096	0.979115236	NA
ASV_16s_6508	0.570547425	-0.011218283	3.549591888	1.978874684	NA	NA
ASV_16s_6509	0.567455275	-0.197061758	3.549492302	2.145395609	NA	NA
ASV_16s_6510	0.574061676	-0.221322132	3.54947807	2.155577632	NA	NA
ASV_16s_6511	0.574118716	-0.272037665	3.549468088	2.135465425	NA	NA
ASV_16s_6512	0.577248559	-0.359692683	3.549452399	2.050587835	NA	NA
ASV_16s_6513	0.608563449	0.29795926	3.549352131	2.232456472	NA	NA
ASV_16s_6514	0.614488379	-0.438615046	3.54933634	1.872147109	0.966576475	NA
ASV_16s_6515	0.598918079	0.088201183	3.548552326	2.41641218	0.933258705	NA
ASV_16s_6516	0.632221773	-1.098119864	3.549023478	1.629955557	0.977466714	NA
ASV_16s_6517	0.655537508	0.034303555	3.548540584	2.490813081	0.927787259	NA
ASV_16s_6518	0.580684345	0.009823614	3.548508421	2.347398049	NA	NA
ASV_16s_6519	0.606174177	1.108666243	3.548029082	2.038446621	0.957681793	NA
ASV_16s_6520	0.730634057	-0.793694544	3.548535728	1.954309374	0.962325857	NA
ASV_16s_6521	0.74518227	-0.793865849	3.548499141	1.959660471	0.962039257	NA
ASV_16s_6522	0.702204869	0.009477815	3.548464497	2.621414214	0.917680415	NA
ASV_16s_6523	0.666271955	0.082592783	3.548675349	2.530494756	0.924783327	NA
ASV_16s_6524	0.64627525	-0.598658851	3.547941004	1.644030702	0.976900578	NA
ASV_16s_6525	0.765333353	-0.883159076	3.347710991	1.653646615	0.976509094	NA
ASV_16s_6526	0.700381729	-0.619001548	3.547654735	1.842939441	0.968019595	NA
ASV_16s_6527	0.745948248	-0.636361386	3.549059478	2.497253003	0.927303775	NA

ASV_16s_6528	0.774030042	-0.20108047	3.298629615	2.891921251	0.89481709	NA
ASV_16s_6529	0.873549715	1.777702724	3.548638494	1.808215197	NA	NA
ASV_16s_6530	0.652854056	-0.08642388	3.549717923	1.88420632	0.965970244	NA
ASV_16s_6531	0.870367655	0.431813686	3.548929681	2.058850295	0.956511386	NA
ASV_16s_6532	0.719310503	-0.10589752	3.55056817	1.690665947	0.974966193	NA
ASV_16s_6533	0.799849663	1.28970092	3.548012908	1.93907138	0.963135457	NA
ASV_16s_6534	0.689348385	2.187547945	3.549292489	0.712701401	NA	NA
ASV_16s_6535	1.025581096	1.341239358	3.549359212	1.179778365	NA	NA
ASV_16s_6536	0.449359834	0.244522673	3.550799857	0.481871836	0.999510189	NA
ASV_16s_6537	0.594410455	-0.069555999	3.549700007	2.183815635	0.948969812	NA
ASV_16s_6538	0.709673748	2.68636517	3.548114875	0.436827281	NA	NA
ASV_16s_6539	0.630786357	-0.069295565	3.549557122	2.269703255	NA	NA
ASV_16s_6540	0.956537855	0.061915163	3.548623412	2.317288957	0.940216025	NA
ASV_16s_6541	0.800578766	1.001959014	3.549775979	1.965533848	0.961723312	NA
ASV_16s_6542	0.819701464	1.055723128	3.550390956	1.353175973	0.986962471	NA
ASV_16s_6543	0.870093374	0.735186804	3.550699412	1.661893215	NA	NA
ASV_16s_6544	0.831857167	0.693878016	3.550641557	1.598255318	NA	NA
ASV_16s_6545	0.611485814	-0.237302831	3.549435959	2.213683679	0.947073059	NA
ASV_16s_6546	0.563404174	-0.225185425	3.549489006	2.136490568	NA	NA
ASV_16s_6547	0.569905665	-0.391637919	3.549460226	1.988103935	NA	NA
ASV_16s_6549	0.604979339	0.078079676	3.548757529	2.338969777	0.938727129	NA
ASV_16s_6550	0.615083191	0.699162115	3.548363055	2.252775862	0.944536183	NA
ASV_16s_6551	0.721694776	-0.794194988	3.54848435	1.854524003	0.96745148	NA
ASV_16s_6552	0.710997172	-0.794178198	3.548496964	1.824513067	0.96891168	NA
ASV_16s_6553	0.670704713	1.862994311	3.547358747	1.818407948	0.96920412	NA
ASV_16s_6554	0.790817998	-0.793890953	3.548492606	2.051418829	0.956939666	NA
ASV_16s_6555	0.661639181	0.104043447	3.548538533	2.612694695	0.91837473	NA
ASV_16s_6556	0.700263202	-0.792954155	3.548691609	1.969242957	0.961523049	NA
ASV_16s_6557	0.7262735	-0.07220924	3.54790799	1.428352134	0.984686996	NA
ASV_16s_6558	0.615673755	-0.598821852	3.549323049	1.533402941	NA	NA
ASV_16s_6559	0.822532319	0.364498127	3.549008496	1.513656125	0.981833116	NA
ASV_16s_6560	0.776234799	1.518048567	3.328694298	1.853508433	0.967501508	NA
ASV_16s_6561	0.657888524	-0.238806205	3.549301291	2.176848227	NA	NA
ASV_16s_6562	0.65765785	-1.952270354	3.547975156	0.554010943	NA	NA
ASV_16s_6563	0.203073229	0.39471523	3.551113446	0.610839774	NA	NA
ASV_16s_6564	0.393556451	0.219987577	3.550650074	1.11921585	0.992669593	NA
ASV_16s_6565	0.436675914	0.596302093	3.550612852	1.102534323	NA	NA
ASV_16s_6566	0.8703143	0.392249167	3.551606787	0.56003129	0.999195636	NA
ASV_16s_6567	0.700238807	0.649293567	3.548439149	2.813808401	0.901675671	NA
ASV_16s_6568	0.72647203	0.397740841	3.548280268	1.633399941	NA	NA
ASV_16s_6569	0.785208773	0.234030528	3.549114782	1.267685336	0.989284062	NA
ASV_16s_6570	0.751885411	1.079099481	3.550119148	1.78620097	0.97072108	NA
ASV_16s_6571	0.745687695	0.359771363	3.550340787	1.265527769	0.98933906	NA
ASV_16s_6572	0.749657	0.315939098	3.550683136	1.610806419	0.978223809	NA
ASV_16s_6573	0.693083464	0.507152786	3.550678772	2.812357913	0.901801111	NA
ASV_16s_6574	0.427904921	0.73474207	3.550827157	1.301141716	NA	NA
ASV_16s_6575	0.564716216	-0.299106723	3.549472878	2.098925993	NA	NA
ASV_16s_6576	0.180481349	-0.086885608	3.550656968	1.123515025	0.992582683	NA
ASV_16s_6577	0.61162603	0.521259967	3.548422511	2.101352198	0.954018242	NA
ASV_16s_6578	0.643385113	-0.534583537	3.548671321	1.841590114	0.968085402	NA
ASV_16s_6579	0.631245919	0.538794113	3.54809743	2.303094737	0.941180738	NA
ASV_16s_6580	0.633279728	-1.391123204	3.548663284	1.315703699	NA	NA
ASV_16s_6581	0.712249003	-0.793488632	3.54858216	1.943686661	0.962891267	NA
ASV_16s_6582	0.705421744	-0.793438996	3.548610453	1.945246375	0.962808543	NA
ASV_16s_6583	0.740966805	1.892060342	3.547505074	0.91080939	0.996141926	NA
ASV_16s_6584	0.714096749	0.884025633	3.546793856	1.860074042	0.967177315	NA
ASV_16s_6585	0.727977947	-0.792604284	3.548748254	2.027474938	0.95830404	NA
ASV_16s_6586	0.666878337	-1.904638251	3.54627619	0.72372	NA	NA
ASV_16s_6587	0.918544407	0.550454493	3.54975419	1.702326738	NA	NA
ASV_16s_6588	0.916709952	0.419269924	3.549463419	1.099382781	NA	NA
ASV_16s_6589	0.257826801	-0.068320907	3.549923567	1.321274117	NA	NA
ASV_16s_6590	0.582316457	-2.360925084	3.547269929	0.507209713	NA	NA
ASV_16s_6591	0.393861218	1.287275582	3.549595378	1.260300576	NA	NA
ASV_16s_6592	0.580531391	-0.069449524	3.549607453	2.226325821	NA	NA
ASV_16s_6593	0.69032163	-0.297403707	3.548145159	1.1562789	NA	NA
ASV_16s_6594	0.338008932	1.056992551	3.550720031	1.100682387	NA	NA
ASV_16s_6595	0.746377078	0.342892662	3.549620598	2.203113679	0.947748426	NA

ASV_16s_6596	1.032916885	-0.684986746	3.549166456	1.224118465	NA	NA
ASV_16s_6597	0.82472547	0.366401697	3.5506797	0.903903389	0.996233434	NA
ASV_16s_6598	0.810323229	1.788084821	3.548852467	1.849843126	0.967681709	NA
ASV_16s_6599	1.043502699	0.691447645	3.549407225	2.695194872	0.911696107	NA
ASV_16s_6600	0.819472602	0.343547459	3.549526144	2.418720321	0.93309215	NA
ASV_16s_6601	0.85417525	0.343611467	3.549530909	2.437636094	0.931719464	NA
ASV_16s_6602	0.564625286	-0.246428502	3.549482936	2.132261376	NA	NA
ASV_16s_6603	0.567758596	-0.683401543	3.549520622	1.031186971	NA	NA
ASV_16s_6604	0.555604287	-0.683328124	3.549537769	1.02421561	NA	NA
ASV_16s_6605	0.585756848	0.031961085	3.549362172	2.151417682	0.950986314	NA
ASV_16s_6606	0.603855479	-0.020642496	3.549328955	2.136757102	0.951884762	NA
ASV_16s_6607	0.657632465	0.158951601	3.548921565	2.517529335	0.925771314	NA
ASV_16s_6608	0.627880651	-0.845842493	3.548978409	1.771355984	0.971405695	NA
ASV_16s_6609	0.624462792	-0.343022654	3.548844325	1.840878633	0.968120071	NA
ASV_16s_6610	0.610270931	0.154537172	3.548470828	2.542371364	0.923872806	NA
ASV_16s_6611	0.668394546	0.191044081	3.548474996	2.772417702	NA	NA
ASV_16s_6612	0.69685515	-0.791907718	3.548897836	2.002320044	0.959711848	NA
ASV_16s_6613	0.692292699	-0.268411589	3.550042245	1.33029619	0.987611238	NA
ASV_16s_6614	0.605163636	-1.203676955	3.54911727	2.217196434	NA	NA
ASV_16s_6615	0.525264608	2.125179991	3.549617803	0.629926377	NA	NA
ASV_16s_6616	0.504582074	-1.502175363	3.548765546	1.083264361	NA	NA
ASV_16s_6617	0.961021462	0.419281054	3.54943789	1.114593633	NA	NA
ASV_16s_6618	0.700028062	2.181720714	3.549306818	0.670270235	NA	NA
ASV_16s_6619	0.506197966	0.664177982	3.549795359	1.840762856	NA	NA
ASV_16s_6620	0.800478847	2.974462828	3.547530976	0.445171584	0.99962351	NA
ASV_16s_6621	0.630859221	-0.061270365	3.549751076	1.95740284	0.962160319	NA
ASV_16s_6622	0.677149764	0.309362076	3.549768082	1.100642472	0.993037668	NA
ASV_16s_6623	1.357059098	1.253229898	3.549408663	1.25439828	NA	NA
ASV_16s_6624	1.028554713	0.308528433	3.547087478	1.891020348	0.965625009	NA
ASV_16s_6625	1.003748743	0.64475936	3.549581478	2.836263776	0.899724709	NA
ASV_16s_6626	1.06651884	0.337427001	3.549341337	2.778066791	NA	NA
ASV_16s_6627	0.695992582	0.342893968	3.549493657	2.345682561	0.938262389	NA
ASV_16s_6628	0.767389047	0.693848312	3.550649053	1.557176915	0.980263921	NA
ASV_16s_6629	0.560938012	-0.14630102	3.549519155	2.118600281	NA	NA
ASV_16s_6630	0.590868498	-0.26539703	3.549451436	2.167534137	0.949988535	NA
ASV_16s_6631	0.560343786	-0.296946686	3.54947823	2.093180451	0.954503366	NA
ASV_16s_6632	0.577177679	-0.321126509	3.549455829	2.098695453	0.954176263	NA
ASV_16s_6633	0.66336672	-0.385386043	3.549370017	2.143816092	0.951453259	NA
ASV_16s_6634	0.593343564	-0.411759349	3.54943572	1.989379862	0.960425827	NA
ASV_16s_6635	0.561459318	-0.400662482	3.549470237	1.958213722	NA	NA
ASV_16s_6636	0.561394338	-0.683362054	3.549529515	1.027550599	NA	NA
ASV_16s_6637	0.175717467	-0.169945966	3.550657774	0.741209173	NA	NA
ASV_16s_6638	0.356993363	0.21999336	3.550657619	1.069752875	NA	NA
ASV_16s_6639	0.60407748	0.17107948	3.549172442	2.263310419	0.943842058	NA
ASV_16s_6640	0.623890313	-0.108561313	3.549468524	1.756028493	NA	NA
ASV_16s_6641	0.597885719	0.325487549	3.548862428	2.099095399	0.954152494	NA
ASV_16s_6642	0.586667346	0.230623346	3.548532704	2.551458861	NA	NA
ASV_16s_6643	0.645047363	0.667065916	3.548033833	2.402400815	NA	NA
ASV_16s_6644	0.650295721	-0.149028666	3.548065951	1.95340873	0.962373978	NA
ASV_16s_6645	0.696090896	-0.793420047	3.548618008	1.92875027	0.963678323	NA
ASV_16s_6646	0.717535566	-0.793337411	3.548636459	1.981356484	0.960865026	NA
ASV_16s_6647	0.744884552	-0.53269837	3.548755832	1.995573637	0.960084951	NA
ASV_16s_6648	0.703619449	-0.857618347	3.548431902	1.637411364	0.977167843	NA
ASV_16s_6649	0.657324102	-0.745876286	3.549307334	1.395160264	0.98571904	NA
ASV_16s_6650	0.694677349	-29.99491627	3.548022842	-0.415276284	1	NA
ASV_16s_6651	0.671646089	0.222614651	3.549329397	1.68983806	NA	NA
ASV_16s_6652	0.638809728	-0.088614026	3.549782941	1.861609612	0.967101232	NA
ASV_16s_6653	0.419405366	0.69095162	3.550738108	1.088426328	NA	NA
ASV_16s_6654	0.460845916	2.135214927	3.54959229	0.549563169	NA	NA
ASV_16s_6655	0.597628533	0.403476798	3.549957779	1.030979287	NA	NA
ASV_16s_6656	0.443551024	1.337109995	3.549962437	0.846127778	NA	NA
ASV_16s_6657	0.72350223	-0.550827203	3.549146175	1.016002446	NA	NA
ASV_16s_6658	0.392093892	0.219987749	3.550650349	1.117310738	0.9927079	NA
ASV_16s_6659	0.83089805	1.723630966	3.548230893	1.468968449	NA	NA
ASV_16s_6660	0.622333307	-0.069614371	3.549712992	2.203258046	0.947739232	NA
ASV_16s_6661	0.646412586	1.818466878	3.547564323	1.015190959	NA	NA
ASV_16s_6662	0.615622257	0.385220108	3.55054446	0.809083306	NA	NA

ASV_16s_6663	0.460830766	0.013696437	3.550296778	1.542765445	NA	NA
ASV_16s_6664	0.769898826	0.693852197	3.550648737	1.558845895	0.980202208	NA
ASV_16s_6665	0.765252429	0.693846547	3.550649323	1.555748311	0.980316656	NA
ASV_16s_6666	0.551822616	-0.171093846	3.549519679	2.113072238	NA	NA
ASV_16s_6667	0.583476682	-0.256612586	3.549460539	2.159864258	0.9504647	NA
ASV_16s_6668	0.568936311	-0.317375049	3.549465336	2.088695727	NA	NA
ASV_16s_6669	0.195427689	-0.627891291	3.55079274	0.683975651	NA	NA
ASV_16s_6670	0.221279592	0.130443351	3.550640535	1.58544963	NA	NA
ASV_16s_6671	0.584375009	0.163126121	3.549413791	2.137535098	0.951837305	NA
ASV_16s_6672	0.252497661	0.79771526	3.550530476	0.836423705	0.997054225	NA
ASV_16s_6673	0.575304961	0.271165588	3.549430454	2.153114527	NA	NA
ASV_16s_6674	0.581725793	0.070027805	3.549077303	2.200545034	0.947911867	NA
ASV_16s_6675	0.613382681	1.357692663	3.548171737	1.943312533	0.962911095	NA
ASV_16s_6676	0.71336117	-0.836727573	3.548333154	2.079924358	0.955284486	NA
ASV_16s_6677	0.612577118	0.471933347	3.548095738	2.037390446	0.95774191	NA
ASV_16s_6678	0.725513469	-0.793156919	3.54865305	2.001316298	0.959767479	NA
ASV_16s_6679	0.677801744	-0.805529004	3.54907003	1.344936697	0.987198432	NA
ASV_16s_6680	0.700647641	-0.791879325	3.548902712	2.007982632	0.959397226	NA
ASV_16s_6681	0.676942055	-0.790583842	3.549106255	1.987793896	0.960512855	NA
ASV_16s_6682	0.650400669	-0.471581436	3.54951398	1.045225016	0.994065634	NA
ASV_16s_6683	0.638949846	0.224418351	3.549336815	1.549982299	0.980528652	NA
ASV_16s_6684	0.78423194	0.403563794	3.549709548	1.118556237	0.992682871	NA
ASV_16s_6685	0.307608164	1.052483562	3.550518441	1.034642671	NA	NA
ASV_16s_6686	0.635683336	-0.349018837	3.549147662	1.104474745	NA	NA
ASV_16s_6687	0.467253833	-23.02563721	3.54918776	-0.111475771	NA	NA
ASV_16s_6688	0.777694689	1.897318433	3.54978812	0.565929451	NA	NA
ASV_16s_6689	0.455039583	-0.216819719	3.549660636	1.72006203	NA	NA
ASV_16s_6690	1.025629462	0.341331252	3.55067685	0.736800525	NA	NA
ASV_16s_6691	0.523851879	0.003421569	3.548746051	1.953397588	NA	NA
ASV_16s_6692	0.495242417	0.187254426	3.551023043	0.879114783	NA	NA
ASV_16s_6693	0.460667556	-0.60726364	3.550074934	0.953096451	NA	NA
ASV_16s_6694	0.872094919	0.376444553	3.547977501	1.673806926	0.975675896	NA
ASV_16s_6695	0.614753007	-0.116518325	3.549917188	1.905893837	0.964864702	NA
ASV_16s_6696	0.740060569	-0.014219143	3.550535449	1.476281826	0.98311944	NA
ASV_16s_6697	0.990214141	0.714320708	3.549437605	2.656456155	0.914862436	NA
ASV_16s_6698	0.857163389	0.808248588	3.549558031	1.639748742	0.977073675	NA
ASV_16s_6699	0.709408224	0.898569525	3.549068347	1.99743544	0.959982175	NA
ASV_16s_6700	0.664925483	0.336197283	3.550667002	1.317890528	0.987954569	NA
ASV_16s_6701	0.814456341	0.549958981	3.549836862	1.959844069	0.962029403	NA
ASV_16s_6702	0.607077097	0.36042395	3.550761333	1.542524511	NA	NA
ASV_16s_6703	0.759725669	0.315939246	3.550681868	1.617588736	0.977957387	NA
ASV_16s_6704	0.683851294	0.315939232	3.550692355	1.564064583	0.980008504	NA
ASV_16s_6705	0.7898802	0.69386096	3.550646299	1.571880535	0.979716313	NA
ASV_16s_6706	0.570031514	-0.37983212	3.549459958	2.007864194	0.959403821	NA
ASV_16s_6707	0.56544868	-0.408263601	3.549466071	1.951003068	NA	NA
ASV_16s_6708	0.560060485	-0.683355667	3.54953142	1.026775256	NA	NA
ASV_16s_6709	0.173114246	-0.169937009	3.550660052	0.733986339	NA	NA
ASV_16s_6710	0.351704573	0.219994307	3.550658841	1.062175253	NA	NA
ASV_16s_6711	0.351704573	0.219994307	3.550658841	1.062175253	NA	NA
ASV_16s_6712	0.182534034	0.220052327	3.550735115	0.733072093	NA	NA
ASV_16s_6713	0.366301078	0.596269262	3.550624943	1.01339982	0.994609473	NA
ASV_16s_6714	0.600637777	0.033606347	3.549094866	2.174744769	0.949538698	NA
ASV_16s_6715	0.589122035	0.16296125	3.548671252	2.429707111	NA	NA
ASV_16s_6716	0.584322581	-0.085965061	3.54870465	2.083589479	0.955069241	NA
ASV_16s_6717	0.597536692	0.492342919	3.548634948	2.100814365	0.954050256	NA
ASV_16s_6718	0.573594058	0.440111514	3.548903345	2.182091025	NA	NA
ASV_16s_6719	0.713452215	-0.794514689	3.548505034	1.710143608	0.974131554	NA
ASV_16s_6720	0.638665089	-0.169775342	3.548150666	2.280293282	0.942713718	NA
ASV_16s_6721	0.661498439	0.080637401	3.548378241	2.677441138	0.913153829	NA
ASV_16s_6722	0.555588076	0.749036959	3.548008214	2.009218111	NA	NA
ASV_16s_6723	0.628183779	-0.321281633	3.548969241	1.740892671	0.972781787	NA
ASV_16s_6724	0.683316803	-0.964142015	3.548764951	1.777981552	NA	NA
ASV_16s_6725	0.741340656	-0.793239914	3.548621138	2.017937168	0.958840914	NA
ASV_16s_6726	0.688035276	-0.791590689	3.548964979	1.995659276	0.960080227	NA
ASV_16s_6727	0.714455204	0.300527448	3.549541476	1.91810669	NA	NA
ASV_16s_6728	0.582917978	-0.960216461	3.548953609	1.678928105	NA	NA
ASV_16s_6729	0.621165813	-0.101668957	3.54999662	1.787266533	0.970671585	NA

ASV_16s_6730	0.573127839	0.363100989	3.548557967	1.374121451	NA	NA
ASV_16s_6731	0.65316585	-0.129682992	3.550460586	1.32257163	0.987825712	NA
ASV_16s_6732	0.768669569	0.403558133	3.549725685	1.112088768	NA	NA
ASV_16s_6733	0.378962641	0.219989639	3.550652912	1.100020115	0.993049795	NA
ASV_16s_6734	0.75889101	3.082812711	3.546933878	0.322528151	NA	NA
ASV_16s_6735	0.563019793	0.062907458	3.550142544	2.173959982	NA	NA
ASV_16s_6736	0.440993826	1.185919986	3.550836741	0.966035264	NA	NA
ASV_16s_6737	0.240672047	-0.256632228	3.550860524	1.283026394	NA	NA
ASV_16s_6738	0.505755503	-0.146736643	3.549963625	1.710935609	NA	NA
ASV_16s_6739	0.762181903	0.884730147	3.55000226	1.182934782	0.991314527	NA
ASV_16s_6740	0.712783678	-0.550798756	3.549153094	1.011186917	NA	NA
ASV_16s_6741	0.9335752	0.422792056	3.550646603	3.161609099	0.869660367	NA
ASV_16s_6742	0.412401119	0.734709055	3.550836591	1.282386944	NA	NA
ASV_16s_6743	0.548801821	-0.247928392	3.549501017	2.103752357	NA	NA
ASV_16s_6744	0.569089236	-0.295861205	3.549468627	2.109129076	0.953554015	NA
ASV_16s_6745	0.539262984	-0.683218625	3.549562062	1.014579848	NA	NA
ASV_16s_6746	0.627564129	-0.710457904	3.549125123	1.86261355	0.967051437	NA
ASV_16s_6747	0.593072221	-0.869385682	3.549134926	1.575113725	NA	NA
ASV_16s_6748	0.647634215	0.81867334	3.548639943	2.425647125	NA	NA
ASV_16s_6749	0.705728544	-0.79439334	3.548509334	1.728103011	0.973347988	NA
ASV_16s_6750	0.565984847	1.75E-04	3.548485319	2.347679205	NA	NA
ASV_16s_6751	0.622072801	0.583973613	3.54785992	2.262838513	NA	NA
ASV_16s_6752	0.667116496	-0.832386514	3.549049235	1.35967704	0.986774431	NA
ASV_16s_6753	0.670788929	0.094446613	3.548535178	2.618522134	0.91791101	NA
ASV_16s_6754	0.5614977	0.742434874	3.55085048	0.983151378	NA	NA
ASV_16s_6755	0.69303975	-0.79203062	3.548873856	1.994188219	0.960161336	NA
ASV_16s_6756	0.571698348	-1.624586564	3.548109379	1.047847239	0.994019329	NA
ASV_16s_6757	0.703737395	0.236277372	3.549682174	2.198257429	0.948057201	NA
ASV_16s_6758	0.630544112	1.397746532	3.547518634	1.060163006	NA	NA
ASV_16s_6759	0.688462664	0.68417174	3.550577293	1.798791621	0.970133216	NA
ASV_16s_6760	0.452467959	-0.55768157	3.550167677	0.833434952	0.997087578	NA
ASV_16s_6761	0.629344281	-0.088089421	3.549770222	1.683880275	NA	NA
ASV_16s_6762	0.48120636	0.028161506	3.550075266	1.301287527	NA	NA
ASV_16s_6763	0.571728644	-0.180094524	3.548353748	1.775816466	NA	NA
ASV_16s_6764	0.71098544	-1.349867421	3.549022221	1.053238659	NA	NA
ASV_16s_6765	0.391686506	-0.201727112	3.550262478	1.395492706	0.985708919	NA
ASV_16s_6766	0.415766954	0.247398289	3.550445066	0.853518606	0.996858647	NA
ASV_16s_6767	0.396394384	-0.04917296	3.550467464	1.005952095	NA	NA
ASV_16s_6768	0.451287886	0.67361592	3.549926477	1.343655975	NA	NA
ASV_16s_6769	0.544220248	-0.143868104	3.548800568	1.756465081	NA	NA
ASV_16s_6770	0.819978439	0.799693575	3.549742711	1.28763681	0.988767173	NA
ASV_16s_6771	0.860651128	-0.561241368	3.549369048	1.845029424	NA	NA
ASV_16s_6772	0.323769674	0.285314864	3.551207866	0.862936921	NA	NA
ASV_16s_6773	0.707168566	0.049175653	3.550630736	2.69986059	0.911311168	NA
ASV_16s_6774	0.783608822	0.34492274	3.550564328	1.085204758	0.993334531	NA
ASV_16s_6775	0.882041661	0.880911905	3.549709702	2.457970807	0.930228513	NA
ASV_16s_6776	0.696203252	0.534980423	3.549512101	2.332669071	0.939161728	NA
ASV_16s_6777	0.80083892	1.186206733	3.549894877	1.22047053	0.990447905	NA
ASV_16s_6778	0.624605044	0.693774539	3.550671147	1.452480417	NA	NA
ASV_16s_6779	0.546858531	-0.119182838	3.549549556	2.075950409	NA	NA
ASV_16s_6780	0.546858531	-0.119182838	3.549549556	2.075950409	NA	NA
ASV_16s_6781	0.607445251	-0.298453915	3.549428381	2.170605586	0.94979718	NA
ASV_16s_6782	0.593449223	-0.371584369	3.549434916	2.059298097	0.956485506	NA
ASV_16s_6783	0.547132326	-0.382920085	3.549486702	1.964013736	0.961805221	NA
ASV_16s_6784	0.191052442	-0.621585214	3.550815447	0.676149927	NA	NA
ASV_16s_6786	0.621092398	-0.643177656	3.549136342	1.910148883	0.964645492	NA
ASV_16s_6787	0.615065166	-0.499156396	3.549274449	1.82888764	0.968701176	NA
ASV_16s_6788	0.595214996	0.035855969	3.548793957	2.280042517	0.942730463	NA
ASV_16s_6789	0.541178078	-0.349903746	3.548943491	1.210908318	NA	NA
ASV_16s_6790	0.555194745	0.003945242	3.548790891	2.14589089	NA	NA
ASV_16s_6791	0.684263401	-0.720299969	3.548675013	1.842651696	0.968033635	NA
ASV_16s_6792	0.587211005	0.037370537	3.548494152	2.43500006	NA	NA
ASV_16s_6793	0.781544267	-0.79404034	3.548410087	1.954260914	NA	NA
ASV_16s_6794	0.706943678	-0.059028373	3.547956331	1.847673538	0.967788109	NA
ASV_16s_6795	0.68043328	0.495997317	3.548305295	2.212079437	NA	NA
ASV_16s_6796	0.686808054	-0.888586879	3.548728294	1.609132648	0.978289266	NA
ASV_16s_6797	0.600398491	-0.072062025	3.54966075	1.929883579	0.963618931	NA

ASV_16s_6798	0.649612328	1.282901562	3.549297252	1.408403541	0.985312481	NA
ASV_16s_6799	0.83617117	-0.567416968	3.549166552	1.133333761	NA	NA
ASV_16s_6800	0.192772413	0.390131407	3.551182053	0.593715421	NA	NA
ASV_16s_6801	0.393408287	0.219987595	3.550650103	1.118987305	0.992674195	NA
ASV_16s_6802	0.619838749	-0.987925137	3.549217451	1.007701336	0.994703345	NA
ASV_16s_6803	0.554339871	-0.069447014	3.549638169	2.176207742	NA	NA
ASV_16s_6804	0.545033196	-1.073348618	3.549159232	1.576473361	NA	NA
ASV_16s_6805	0.517569281	1.779889015	3.550078674	0.824580533	NA	NA
ASV_16s_6806	0.60406804	2.474522256	3.548478099	0.838320681	NA	NA
ASV_16s_6807	0.685661508	1.837213348	3.548812058	1.368405285	0.986519386	NA
ASV_16s_6808	0.739138273	0.233660839	3.549808172	2.985470025	NA	NA
ASV_16s_6809	0.698641155	0.512732175	3.550605275	2.511399836	0.926236202	NA
ASV_16s_6810	0.686348218	0.319174686	3.550725958	1.387216505	0.985959596	NA
ASV_16s_6811	0.445617747	0.305276414	3.550767664	0.970599282	NA	NA
ASV_16s_6812	1.001529537	0.546181046	3.549527433	2.814727248	0.901596171	NA
ASV_16s_6813	0.81431605	0.622430351	3.550687384	2.431788527	NA	NA
ASV_16s_6814	0.682979296	0.348957969	3.550509487	1.253512294	0.98964215	NA
ASV_16s_6815	0.406199599	0.734695507	3.550840565	1.27468776	NA	NA
ASV_16s_6816	0.785743805	0.69385785	3.550646797	1.569165756	0.979818086	NA
ASV_16s_6817	0.530459182	0.076597852	3.549734961	1.712538301	NA	NA
ASV_16s_6818	0.559573093	-0.160911615	3.549514548	2.122915296	NA	NA
ASV_16s_6819	0.554342147	-0.157122551	3.549522348	2.112150898	NA	NA
ASV_16s_6820	0.530704457	-0.188297001	3.549539695	2.078768809	NA	NA
ASV_16s_6822	0.571213877	0.203339779	3.549039435	2.245869735	NA	NA
ASV_16s_6823	0.556406349	0.322184901	3.548953748	2.209686252	NA	NA
ASV_16s_6824	0.597761891	-0.190448357	3.549421507	1.824940307	NA	NA
ASV_16s_6825	0.57455284	-0.014726344	3.548818347	2.144899025	0.951386882	NA
ASV_16s_6826	0.630531827	0.065479935	3.548629139	2.39236818	0.934981485	NA
ASV_16s_6827	0.575315454	0.383528766	3.548948263	2.116210542	0.95312914	NA
ASV_16s_6828	0.549479184	0.066880075	3.548634897	2.306224228	0.940968726	NA
ASV_16s_6829	0.795027865	2.055328787	3.54822779	0.670490149	NA	NA
ASV_16s_6830	0.651432489	-0.543061706	3.290979416	2.159721496	0.950473541	NA
ASV_16s_6831	0.685130143	-0.793856326	3.548554996	1.832461999	0.968528585	NA
ASV_16s_6832	0.507755453	1.201290295	3.548599333	1.805685376	NA	NA
ASV_16s_6833	0.689839307	-0.793554852	3.548589442	1.892393365	NA	NA
ASV_16s_6834	0.644983404	0.036826116	3.548414568	2.595613915	0.919726826	NA
ASV_16s_6835	0.614106702	-0.07684863	3.550467524	1.36775788	0.986538405	NA
ASV_16s_6836	0.701548325	-0.616063717	3.549858983	1.924449963	0.963903198	NA
ASV_16s_6837	0.610621231	-0.101034803	3.550229394	1.779906726	0.971012479	NA
ASV_16s_6838	0.720464883	-0.661001563	3.548803637	2.091444595	0.954606063	NA
ASV_16s_6839	0.725150144	0.313641654	3.550620976	2.851243027	0.898413944	NA
ASV_16s_6840	0.765212153	1.331535769	3.336931653	1.97075507	0.961441242	NA
ASV_16s_6841	0.5944535	0.281068496	3.548681582	1.573134574	NA	NA
ASV_16s_6842	0.643555492	0.344463452	3.549882499	1.518064659	NA	NA
ASV_16s_6843	0.34177198	-0.062132519	3.550612337	1.056447466	NA	NA
ASV_16s_6844	0.423553543	0.068045409	3.549579287	1.702051477	NA	NA
ASV_16s_6845	0.191300868	0.389457074	3.551192394	0.59121005	NA	NA
ASV_16s_6846	0.374767959	0.219990333	3.550653769	1.094357435	0.993159517	NA
ASV_16s_6847	0.975128936	0.550385567	3.549357628	1.861967682	NA	NA
ASV_16s_6848	0.624049086	2.067687445	3.548346066	0.708482037	0.998269713	NA
ASV_16s_6849	0.754758891	1.715801241	3.5495121	0.929447821	0.995887794	NA
ASV_16s_6850	0.688087649	0.677314187	3.550633776	1.542775489	0.980791715	NA
ASV_16s_6851	0.781978686	0.363070119	3.550627228	0.808766017	0.997353461	NA
ASV_16s_6852	0.6603319	0.546551453	3.550661863	2.392950387	0.934940034	NA
ASV_16s_6853	0.759002796	0.460874555	3.549887097	2.210857051	0.947254107	NA
ASV_16s_6854	0.75109118	0.343565263	3.54957979	2.240942598	0.945310579	NA
ASV_16s_6855	0.754833734	0.343755865	3.549613114	2.207607885	0.947461821	NA
ASV_16s_6856	0.801651723	0.713337493	3.55070797	2.013365392	NA	NA
ASV_16s_6857	0.615141331	0.31593941	3.550704084	1.510188066	NA	NA
ASV_16s_6858	0.779347713	1.051260039	3.550406598	1.330895928	0.98759449	NA
ASV_16s_6859	0.718127992	0.693822731	3.550655687	1.523394218	0.981488683	NA
ASV_16s_6860	0.585292502	-0.308842816	3.549448995	2.124697394	0.952617238	NA
ASV_16s_6861	0.148935453	-0.169836216	3.550684986	0.662381614	NA	NA
ASV_16s_6862	0.177044288	0.220056019	3.550740028	0.718174288	NA	NA
ASV_16s_6864	0.599657597	0.167156841	3.549222311	2.15638339	0.950680011	NA
ASV_16s_6865	0.589882984	-0.3629267	3.549323411	1.883297623	0.966016137	NA
ASV_16s_6866	0.694781179	-0.794521954	3.548593798	1.581046878	0.979370453	NA

ASV_16s_6867	0.719605536	1.925198462	3.548703913	0.613438734	0.998916078	NA
ASV_16s_6868	0.574663667	0.231290569	3.548531799	2.495438808	NA	NA
ASV_16s_6869	0.661456875	-0.844639039	3.548551371	1.570518349	NA	NA
ASV_16s_6870	0.589727057	1.240236745	3.548000659	1.717030361	NA	NA
ASV_16s_6871	0.669007804	-0.872175265	3.549013861	1.97438446	0.961244501	NA
ASV_16s_6872	0.658112551	-0.768727777	3.549332779	1.963218148	0.961848051	NA
ASV_16s_6873	0.613659902	-0.603477885	3.378725089	1.106193759	0.992928911	NA
ASV_16s_6874	0.601296569	-0.981272752	3.547797607	1.626644315	0.977598711	NA
ASV_16s_6875	0.755446015	0.004340471	3.549054189	1.671805726	NA	NA
ASV_16s_6876	0.733564728	0.651997616	3.550181373	2.332261226	0.939189806	NA
ASV_16s_6877	0.544023164	0.647041698	3.549052097	2.020808557	NA	NA
ASV_16s_6878	0.49210004	0.15024658	3.549034828	1.338007206	NA	NA
ASV_16s_6879	1.235704764	-0.568042207	3.549009487	1.259241754	NA	NA
ASV_16s_6880	0.588985597	-0.890449522	3.548204306	1.363073046	NA	NA
ASV_16s_6881	0.341993283	1.335702189	3.550283599	0.762468166	NA	NA
ASV_16s_6882	0.522465814	0.135989993	3.549494401	1.799555269	NA	NA
ASV_16s_6883	0.622356376	0.959481438	3.549351236	1.764950797	NA	NA
ASV_16s_6884	0.820092805	0.748434909	3.549459601	1.275042834	NA	NA
ASV_16s_6885	0.51728427	1.222875279	3.550164808	0.940331096	NA	NA
ASV_16s_6886	0.790791517	0.020419036	3.55031465	1.901539793	0.965088229	NA
ASV_16s_6887	0.750446144	0.334636434	3.550621442	1.66798424	0.975918269	NA
ASV_16s_6888	0.678493391	0.631959993	3.550235369	1.938766911	0.963151535	NA
ASV_16s_6889	0.85774682	0.189678233	3.548369211	2.132099996	0.952168328	NA
ASV_16s_6890	0.804286677	0.174162023	3.549707315	3.371158464	0.84867789	NA
ASV_16s_6891	0.749419587	0.166905387	3.549483551	2.735789305	0.908321368	NA
ASV_16s_6892	0.734917003	0.758461777	3.54964113	1.949421317	0.962586615	NA
ASV_16s_6893	0.860449825	0.793612578	3.54850878	1.741688032	0.972746351	NA
ASV_16s_6894	0.58839774	0.552996433	3.550662649	2.216511644	0.946891602	NA
ASV_16s_6895	0.584424966	0.36042193	3.55076783	1.523156108	NA	NA
ASV_16s_6896	0.665526018	0.315939333	3.55069525	1.550215609	0.9805201	NA
ASV_16s_6897	0.628203647	0.31593933	3.550701658	1.520866812	0.981578446	NA
ASV_16s_6898	0.728007809	0.515057233	3.550673214	2.86186731	0.897479762	NA
ASV_16s_6899	0.757257909	0.981369029	3.550535845	1.346860724	0.987143566	NA
ASV_16s_6900	0.399998078	0.734680539	3.550844661	1.266870556	NA	NA
ASV_16s_6901	0.399998078	0.734680539	3.550844661	1.266870556	NA	NA
ASV_16s_6902	0.734322407	0.69383474	3.550653409	1.53473694	0.981082643	NA
ASV_16s_6903	0.717080111	0.693826853	3.550655839	1.522640676	0.981515473	NA
ASV_16s_6904	0.536173179	-0.159684314	3.549543816	2.0807391	NA	NA
ASV_16s_6905	0.554597552	-0.230264189	3.549498014	2.119678527	0.952920318	NA
ASV_16s_6906	0.602184876	-0.296028325	3.549434	2.164138371	0.950199648	NA
ASV_16s_6907	0.555712864	-0.278299237	3.549487397	2.099255057	0.954143003	NA
ASV_16s_6908	0.527205753	-0.296766448	3.549518506	2.033937192	NA	NA
ASV_16s_6909	0.166606191	-0.169911995	3.550666056	0.715515299	NA	NA
ASV_16s_6910	0.175671852	0.220056998	3.550741304	0.714390568	NA	NA
ASV_16s_6911	0.175671852	0.220056998	3.550741304	0.714390568	NA	NA
ASV_16s_6913	0.534167694	0.176925447	3.549046529	2.197594509	NA	NA
ASV_16s_6914	0.68087725	-0.849244307	3.548821439	1.730657342	0.973235452	NA
ASV_16s_6915	0.531888086	-0.238353665	3.548726708	1.61484998	NA	NA
ASV_16s_6916	0.579812251	0.073300952	3.548567218	2.400063694	0.934432527	NA
ASV_16s_6917	0.666798852	-0.827322636	3.548586699	1.575518579	0.979579456	NA
ASV_16s_6918	0.698066642	-0.793401603	3.548623831	1.936226283	0.963285546	NA
ASV_16s_6919	0.67313745	0.700870646	3.547972171	2.32676445	0.939567589	NA
ASV_16s_6920	0.650292731	-0.792236619	3.548852245	1.922042223	0.964028769	NA
ASV_16s_6921	0.713480291	-0.792817498	3.548711752	1.997317633	0.959988682	NA
ASV_16s_6922	0.722862682	-0.41568968	3.547562265	2.310809758	0.940657374	NA
ASV_16s_6923	0.702070856	-0.854729021	3.549107265	1.506437316	0.982085963	NA
ASV_16s_6924	0.590112108	-0.816321031	3.54964567	1.981822998	NA	NA
ASV_16s_6925	0.788912453	-1.288755515	3.547199384	2.177525522	0.949364656	NA
ASV_16s_6926	0.5975808	-0.07892318	3.549729038	1.858768333	0.967241931	NA
ASV_16s_6927	0.505407218	0.180894405	3.550517862	0.616726392	0.998897002	NA
ASV_16s_6928	0.607706677	2.307291597	3.549205028	0.601947318	0.99898101	NA
ASV_16s_6929	0.38482169	-0.28638467	3.54950247	1.468523848	NA	NA
ASV_16s_6930	0.377722895	0.100073006	3.54949719	1.563423599	NA	NA
ASV_16s_6931	0.745522121	1.177746331	3.29446244	1.878097407	0.966278105	NA
ASV_16s_6932	0.542235148	-0.069440514	3.549644898	2.156571326	NA	NA
ASV_16s_6933	0.71112432	0.532095652	3.550135649	2.460188617	0.930064947	NA
ASV_16s_6934	0.557036742	-0.109979731	3.549200202	1.695835548	NA	NA

ASV_16s_6935	0.716812801	0.31593928	3.550687532	1.587947745	0.9791078	NA
ASV_16s_6936	0.715918124	0.315939196	3.550687657	1.587302116	0.979132456	NA
ASV_16s_6937	0.761214721	1.084582689	3.550335414	1.297819841	0.988497553	NA
ASV_16s_6938	0.735892492	0.693833746	3.550653193	1.535819846	0.981043604	NA
ASV_16s_6939	0.694180522	0.693815221	3.550659252	1.506123598	0.982096904	NA
ASV_16s_6940	0.526656262	-0.035719148	3.549631547	1.943825704	NA	NA
ASV_16s_6941	0.518380031	-0.111487129	3.549591742	2.01796634	NA	NA
ASV_16s_6942	0.528346672	-0.208442693	3.549536192	2.075411771	NA	NA
ASV_16s_6943	0.518477	-0.207336931	3.549549569	2.057036634	NA	NA
ASV_16s_6944	0.54533725	-0.240595274	3.549506716	2.100161058	0.954089126	NA
ASV_16s_6945	0.553559537	-0.38542211	3.549479057	1.970878323	0.96143457	NA
ASV_16s_6946	0.525479217	-0.380394501	3.549514079	1.929920654	NA	NA
ASV_16s_6947	0.191974323	0.020881413	3.550664387	1.338630972	NA	NA
ASV_16s_6948	0.367123588	0.596270767	3.550624777	1.014510892	NA	NA
ASV_16s_6949	0.548482576	0.344096678	3.549217537	2.049160387	NA	NA
ASV_16s_6950	0.589363547	0.1304551	3.54883626	2.345587409	0.938268989	NA
ASV_16s_6951	0.628151774	-0.356688484	3.548512924	1.951400813	0.962481137	NA
ASV_16s_6952	0.547043512	0.203695882	3.548717992	2.321108047	NA	NA
ASV_16s_6953	0.577360518	0.026845408	3.548614996	2.283679636	0.942487355	NA
ASV_16s_6954	0.551928657	-0.024988245	3.548548989	2.240953715	0.945309854	NA
ASV_16s_6955	0.642894121	-0.741112714	3.54831544	1.77528059	0.971225597	NA
ASV_16s_6956	0.693229526	-0.794277609	3.548526713	1.731259923	0.973208864	NA
ASV_16s_6957	0.59050051	0.719947167	3.549275057	1.722665029	NA	NA
ASV_16s_6958	0.55013615	0.183694641	3.548584753	2.464734656	NA	NA
ASV_16s_6959	0.699543373	-0.793765523	3.548545182	1.874619913	0.966452659	NA
ASV_16s_6960	0.653016266	-0.793111932	3.548687956	1.866843356	0.966841177	NA
ASV_16s_6961	0.705686695	-0.793296455	3.548627407	1.954113569	0.962336322	NA
ASV_16s_6962	0.654321109	-0.792615931	3.548777505	1.909795581	0.964663722	NA
ASV_16s_6963	0.663893726	-0.792240471	3.548845338	1.944144701	0.962866984	NA
ASV_16s_6964	0.663635399	-0.792190871	3.548852749	1.94510627	0.962815978	NA
ASV_16s_6965	0.68703724	-0.791548356	3.548973972	1.994743271	0.960130743	NA
ASV_16s_6966	0.581653448	-0.655812356	3.54938562	1.78685966	0.97069049	NA
ASV_16s_6967	0.696418573	-0.785043984	3.54918346	2.012245441	0.959159501	NA
ASV_16s_6968	0.598984536	-0.951719238	3.54864036	1.359568076	0.986777596	NA
ASV_16s_6969	0.681621746	-0.38173445	3.550367397	1.250227701	0.989724064	NA
ASV_16s_6970	0.667370302	1.112767046	3.550296941	1.277578705	0.989029629	NA
ASV_16s_6971	0.730094377	2.341295861	3.547829757	0.53482892	0.999308695	NA
ASV_16s_6972	0.478113502	1.222619676	3.550055435	1.195672619	NA	NA
ASV_16s_6973	0.62666239	0.278602731	3.549311066	2.168895856	NA	NA
ASV_16s_6974	0.487543539	-0.438491032	3.549501858	1.380125392	NA	NA
ASV_16s_6975	0.345523609	-0.548760085	3.549646735	0.778344961	NA	NA
ASV_16s_6976	0.365738416	0.219991817	3.55065568	1.081971307	0.993395674	NA
ASV_16s_6977	0.37479075	0.219990231	3.550653765	1.09437051	0.993159264	NA
ASV_16s_6978	0.401868413	0.59628807	3.550618306	1.060342826	NA	NA
ASV_16s_6979	0.620922123	0.416138547	3.549443147	1.670150623	0.975828256	NA
ASV_16s_6980	0.576927785	-0.06947298	3.549609312	2.220534074	NA	NA
ASV_16s_6981	0.5123874	0.424021572	3.549484785	1.787581541	NA	NA
ASV_16s_6982	0.682419947	1.120428855	3.550026129	1.757792876	0.972023151	NA
ASV_16s_6983	0.63548355	0.227668679	3.550667293	2.731057749	NA	NA
ASV_16s_6984	0.74136784	0.225259144	3.549656524	3.202253463	NA	NA
ASV_16s_6985	0.666960906	0.315939363	3.550695019	1.551295617	0.980480485	NA
ASV_16s_6986	0.6293111	0.315939207	3.550701457	1.521746205	0.981547243	NA
ASV_16s_6987	0.71692182	0.693826054	3.550655861	1.522527848	0.981519482	NA
ASV_16s_6988	0.642274699	0.693785345	3.550667884	1.466622701	0.983442736	NA
ASV_16s_6989	0.715098021	0.693826429	3.550656125	1.521237501	0.981565297	NA
ASV_16s_6990	0.642285771	0.693785351	3.550667882	1.466629739	0.983442502	NA
ASV_16s_6991	0.52930853	-0.192750143	3.549539989	2.076653214	NA	NA
ASV_16s_6992	0.546432728	-0.257202142	3.549502203	2.095187828	0.95438445	NA
ASV_16s_6993	0.567748784	-0.289833813	3.549471309	2.111622339	0.953404658	NA
ASV_16s_6994	0.536563371	-0.3304657	3.54950194	2.01757896	0.958861004	NA
ASV_16s_6995	0.545084218	-0.346074581	3.549490152	2.013777063	0.959073902	NA
ASV_16s_6996	0.183760364	-0.610550596	3.550854611	0.662702896	NA	NA
ASV_16s_6997	0.16400297	-0.169902118	3.550668591	0.707957391	NA	NA
ASV_16s_6998	0.537776455	-0.052804633	3.549295019	1.959072808	0.96207079	NA
ASV_16s_6999	0.581415559	-0.011417255	3.549095095	2.086197814	0.954915721	NA
ASV_16s_7000	0.548613666	0.129288905	3.548864245	2.088963183	0.954752654	NA
ASV_16s_7001	0.614787955	-0.590281319	3.548698284	1.77691688	0.971150318	NA

ASV_16s_7002	0.652253982	-0.793349695	3.548652089	1.837284596	0.968294875	NA
ASV_16s_7003	0.578936618	0.581334897	3.548508286	1.773467312	NA	NA
ASV_16s_7004	0.644856559	-0.792866874	3.548737893	1.874177048	0.966474852	NA
ASV_16s_7005	0.68644708	-0.792774341	3.548730549	1.955938875	NA	NA
ASV_16s_7006	0.658328885	-0.792266142	3.548842981	1.934122938	0.963396287	NA
ASV_16s_7007	0.646048811	-0.791615281	3.548976362	1.934864577	0.963357261	NA
ASV_16s_7008	0.713487502	0.121518865	3.315199071	2.59682344	0.919631431	NA
ASV_16s_7009	0.599390271	-0.872238249	3.549343246	1.916498943	0.964316947	NA
ASV_16s_7010	0.678192252	-0.886432965	3.548258949	1.964804727	0.961762611	NA
ASV_16s_7011	0.708611778	-0.666273609	3.548038022	1.677804621	0.975508675	NA
ASV_16s_7012	0.703177431	0.241176547	3.550217644	1.912202491	0.964539425	NA
ASV_16s_7013	0.61967942	0.219599805	3.548563677	2.33554012	NA	NA
ASV_16s_7014	0.332611238	1.88735909	3.550028228	0.487514386	NA	NA
ASV_16s_7015	0.769862857	1.392519578	3.548909739	1.543138432	0.980778518	NA
ASV_16s_7016	0.391464411	0.377653918	3.551713663	0.550494412	NA	NA
ASV_16s_7017	0.36121877	0.219992537	3.550656672	1.075660113	0.993513988	NA
ASV_16s_7018	0.736101681	2.464962147	3.548531644	1.384179466	NA	NA
ASV_16s_7019	0.54818897	-1.22106806	3.549025491	1.130649105	0.992437035	NA
ASV_16s_7020	0.535156369	-0.06939348	3.549641022	2.148208539	NA	NA
ASV_16s_7021	0.468439431	1.329032169	3.549970116	1.427422959	NA	NA
ASV_16s_7022	0.489247067	0.363722074	3.550225119	1.751924954	NA	NA
ASV_16s_7023	0.68825231	0.315939113	3.550691689	1.567252486	0.979889629	NA
ASV_16s_7024	0.688440944	0.315939143	3.550691661	1.567381507	0.97988481	NA
ASV_16s_7025	0.686675219	0.449435446	3.550681861	2.766772419	0.905707129	NA
ASV_16s_7026	0.828225485	1.180969069	3.549799462	1.234993786	NA	NA
ASV_16s_7027	0.801127536	1.186777018	3.549757985	1.216531693	0.990541265	NA
ASV_16s_7028	0.746461875	0.693841895	3.550651766	1.543070636	0.980780983	NA
ASV_16s_7029	0.709896247	0.693820836	3.550656887	1.517496679	0.981697736	NA
ASV_16s_7030	0.754057576	0.6938454	3.550650765	1.548220723	NA	NA
ASV_16s_7031	0.535780081	-0.131995206	3.549557207	2.065057496	0.956151915	NA
ASV_16s_7032	0.544140103	-0.17087664	3.549529234	2.098991592	NA	NA
ASV_16s_7033	0.521436391	-0.198275709	3.549548504	2.06243557	NA	NA
ASV_16s_7034	0.526005665	-0.212364501	3.549538102	2.070770013	NA	NA
ASV_16s_7035	0.516474768	-0.296869018	3.549532628	2.013862074	0.959069149	NA
ASV_16s_7036	0.540600773	-0.326551958	3.549497358	2.029193783	0.958206887	NA
ASV_16s_7037	0.518563231	-0.364747174	3.549523266	1.940846678	NA	NA
ASV_16s_7038	0.182301949	-0.608256049	3.550862679	0.659945332	NA	NA
ASV_16s_7041	0.584389497	-0.765875731	3.549221214	1.710992706	0.974094811	NA
ASV_16s_7042	0.540130799	-0.017701415	3.549164975	2.017038503	0.958891305	NA
ASV_16s_7043	0.579862784	0.043626024	3.548679355	2.272948614	0.943203109	NA
ASV_16s_7044	0.522166508	-0.33963867	3.548688921	1.417579851	NA	NA
ASV_16s_7045	0.574202427	-0.881476036	3.549137486	1.290657431	0.988687606	NA
ASV_16s_7046	0.681562378	-0.794093117	3.548543971	1.760297111	0.971909724	NA
ASV_16s_7047	0.528666961	-0.338321353	3.54865683	1.56947564	NA	NA
ASV_16s_7048	0.650189465	-0.620103101	3.076701179	2.468909822	0.929419934	NA
ASV_16s_7049	0.609142543	0.082388254	3.548481836	2.554263013	0.922955881	NA
ASV_16s_7050	0.605245411	-0.752271192	3.549282136	1.467374719	NA	NA
ASV_16s_7051	0.609591699	-0.728327982	3.549166812	1.947717178	NA	NA
ASV_16s_7052	0.662331438	0.092074738	3.548810936	2.464067655	0.929778414	NA
ASV_16s_7053	0.373607794	1.817715105	3.549704195	0.806526765	NA	NA
ASV_16s_7054	0.672116765	0.23590986	3.548741217	2.060107038	0.956438734	NA
ASV_16s_7055	0.775110637	0.820447744	3.550164207	0.96720519	0.995340357	NA
ASV_16s_7056	0.569187553	-0.655105522	3.548682879	1.319930216	NA	NA
ASV_16s_7057	0.630404506	0.343677068	3.549273663	2.997780758	0.885207454	NA
ASV_16s_7058	0.352463656	0.21999406	3.550658666	1.063214729	0.993743336	NA
ASV_16s_7059	0.381948162	0.225280073	3.55070505	0.872555296	0.996631088	NA
ASV_16s_7060	0.372845018	0.219990672	3.55065417	1.091718756	0.993210267	NA
ASV_16s_7061	0.604900413	0.310927787	3.550696221	1.478756147	0.98303602	NA
ASV_16s_7062	0.395539777	0.596285434	3.550619401	1.052282367	NA	NA
ASV_16s_7063	0.61754632	2.143637513	3.548643993	0.715996159	0.998209788	NA
ASV_16s_7064	0.349374074	2.049170059	3.549770157	0.42847717	NA	NA
ASV_16s_7065	0.5103778	-0.04571217	3.550672522	1.237080893	0.990047907	NA
ASV_16s_7066	0.914906714	0.157495132	3.547154262	1.808540991	NA	NA
ASV_16s_7067	0.847371516	0.409091713	3.54891273	2.776635464	0.904868	NA
ASV_16s_7068	0.734327865	0.349584379	3.549868253	2.072036779	0.955745833	NA
ASV_16s_7069	0.685077624	0.345782273	3.550688597	2.086062682	0.954923682	NA
ASV_16s_7070	0.695874471	0.693813966	3.550658991	1.50736468	0.982053599	NA

ASV_16s_7071	0.715474698	0.693823978	3.550656071	1.521489743	0.981556346	NA
ASV_16s_7072	0.721220059	0.693825281	3.550655246	1.525552312	0.981411831	NA
ASV_16s_7073	0.736460163	0.693832054	3.550653117	1.53618607	0.981030391	NA
ASV_16s_7074	0.591482049	0.693751307	3.550677797	1.424741288	NA	NA
ASV_16s_7075	0.511936586	-0.093724896	3.549611702	1.989549879	NA	NA
ASV_16s_7076	0.512062075	-0.219837386	3.549554704	2.04364199	NA	NA
ASV_16s_7077	0.535217891	-0.267173487	3.549514354	2.069522734	0.955892341	NA
ASV_16s_7078	0.562923441	-0.348051932	3.549469077	2.042126967	0.957471951	NA
ASV_16s_7079	0.180843533	-0.605945127	3.550870849	0.657172511	NA	NA
ASV_16s_7080	0.180843533	-0.605945127	3.550870849	0.657172511	NA	NA
ASV_16s_7081	0.222786555	0.220030521	3.550706494	0.831580754	0.997108145	NA
ASV_16s_7083	0.544324933	-0.385820316	3.549514833	1.64004519	0.977061715	NA
ASV_16s_7084	0.54165353	0.168132139	3.54861169	2.367277039	NA	NA
ASV_16s_7085	0.552630378	0.711869722	3.548499725	2.038447954	0.957681717	NA
ASV_16s_7086	0.683508371	-0.549803242	3.547819267	1.676455472	0.975565183	NA
ASV_16s_7087	0.671107597	-0.920857448	3.548349821	1.577744219	0.979495464	NA
ASV_16s_7088	0.546420229	-0.513024407	3.549487501	1.78233388	NA	NA
ASV_16s_7089	0.646338409	-0.829097224	3.548821154	1.522626552	0.981515975	NA
ASV_16s_7090	0.800501301	0.612548735	3.549385574	1.260520799	NA	NA
ASV_16s_7091	0.294385322	-0.301608165	3.550581885	0.919973212	NA	NA
ASV_16s_7092	0.586640264	1.574040674	3.54832377	1.331560416	NA	NA
ASV_16s_7093	1.137948876	0.27147158	3.549433078	1.235683928	NA	NA
ASV_16s_7094	0.708256943	0.598934111	3.549603613	1.953684493	NA	NA
ASV_16s_7095	0.40740983	1.336685799	3.550058687	0.818727962	NA	NA
ASV_16s_7096	0.617052472	-0.139616634	3.549253277	2.125429765	NA	NA
ASV_16s_7097	0.372477317	0.219990827	3.550654248	1.091212363	NA	NA
ASV_16s_7098	0.541074259	-0.592436006	3.549251402	1.011262604	NA	NA
ASV_16s_7099	0.561994056	2.258063016	3.549335547	0.543803525	NA	NA
ASV_16s_7100	0.512009253	0.815675243	3.549399679	2.237934409	NA	NA
ASV_16s_7101	0.742167205	-0.324738005	3.548865154	2.472430873	0.9291587	NA
ASV_16s_7102	0.702478123	0.349521944	3.550753231	0.946647388	0.995643893	NA
ASV_16s_7103	0.691326406	1.1911066	3.550289667	1.724300564	0.973515009	NA
ASV_16s_7104	0.838415179	0.511895133	3.550667656	2.938503978	NA	NA
ASV_16s_7105	0.685238547	0.34955991	3.54987704	2.030775405	0.958117381	NA
ASV_16s_7106	0.711964794	0.374158269	3.550256385	1.855313124	0.967412577	NA
ASV_16s_7107	0.714863392	0.315939222	3.550687808	1.586519813	0.979162309	NA
ASV_16s_7108	0.605570952	0.31593928	3.550705931	1.502174266	0.982234291	NA
ASV_16s_7109	0.602148996	0.31593928	3.550706605	1.499292728	0.982334135	NA
ASV_16s_7110	0.934170236	1.114352819	3.550417201	1.441953774	0.984251467	NA
ASV_16s_7111	0.672959701	0.693801092	3.550662622	1.490318901	0.982642927	NA
ASV_16s_7112	0.502118585	-0.178665432	3.549582487	2.022757997	NA	NA
ASV_16s_7113	0.508724986	-0.206254118	3.549563323	2.038426412	NA	NA
ASV_16s_7114	0.553641777	-0.240132576	3.549496964	2.114969367	NA	NA
ASV_16s_7115	0.52043561	-0.275747942	3.549531709	2.036914149	NA	NA
ASV_16s_7116	0.179385118	-0.603598087	3.550879121	0.654379475	NA	NA
ASV_16s_7117	0.096461531	-0.169447232	3.550781873	0.470794417	NA	NA
ASV_16s_7118	0.513258264	-0.196052102	3.549531974	2.190435863	NA	NA
ASV_16s_7119	0.510765515	0.06902983	3.54940029	1.955035729	0.962287024	NA
ASV_16s_7120	0.521180934	0.377536655	3.549316079	2.066177796	0.956086867	NA
ASV_16s_7121	0.587375188	-0.138590268	3.548991051	1.641147813	0.977017201	NA
ASV_16s_7122	0.551499997	0.596765307	3.548768099	1.861088253	0.967127075	NA
ASV_16s_7123	0.520370641	0.052183332	3.548597011	2.274388843	NA	NA
ASV_16s_7124	0.605128353	0.28590677	3.548171787	2.028310056	0.958256852	NA
ASV_16s_7125	0.563481417	0.081568441	3.548541957	2.449561273	0.93084702	NA
ASV_16s_7126	0.127131172	0.728021251	3.551402819	0.707323352	NA	NA
ASV_16s_7127	0.542212905	-0.839463536	3.549523564	1.174518089	0.991501826	NA
ASV_16s_7128	0.641447775	-0.792910862	3.548730787	1.862411105	0.967061482	NA
ASV_16s_7129	0.64495224	-0.79256408	3.548791372	1.895440768	0.965400009	NA
ASV_16s_7130	0.637541994	-0.789114072	3.549150273	1.935710984	0.963312693	NA
ASV_16s_7131	0.628483353	-1.328363736	3.549138128	1.218486489	0.990495003	NA
ASV_16s_7132	0.595994993	-0.167691479	3.550141874	1.857411371	0.967309008	NA
ASV_16s_7133	0.57782134	0.620762286	3.547375969	1.401963544	NA	NA
ASV_16s_7134	0.621027993	-0.132759248	3.548306311	1.954783616	NA	NA
ASV_16s_7135	0.531644141	-0.414606158	3.548778696	1.250451924	0.989718485	NA
ASV_16s_7136	0.795029517	0.406883957	3.551249252	0.613076415	NA	NA
ASV_16s_7137	0.563336883	0.330448452	3.548686274	1.829862902	0.968654138	NA
ASV_16s_7138	0.830987608	0.419244983	3.549520597	1.067668441	NA	NA

ASV_16s_7139	0.37462876	-0.033327599	3.550620696	1.135068944	NA	NA
ASV_16s_7140	0.558976431	-1.945179092	3.548245317	0.725761202	0.998129875	NA
ASV_16s_7141	0.447208652	-0.217339571	3.549521082	1.789147257	NA	NA
ASV_16s_7142	0.48521067	0.094691488	3.549424483	1.25749496	NA	NA
ASV_16s_7143	0.672562892	0.318052334	3.55111977	0.612009595	0.9989243	NA
ASV_16s_7144	0.480162158	1.881732655	3.550059336	0.630871226	NA	NA
ASV_16s_7145	0.41840445	0.302255726	3.550854085	0.949546523	NA	NA
ASV_16s_7146	0.745384284	-0.223162917	3.548034822	2.653811	0.915076691	NA
ASV_16s_7147	0.778457774	0.37807299	3.549618062	2.540313385	0.924030957	NA
ASV_16s_7148	0.677897124	0.908936884	3.550538286	2.399364133	0.934482524	NA
ASV_16s_7149	0.837334277	0.456245995	3.55066242	3.365794445	NA	NA
ASV_16s_7150	0.651396403	0.188835804	3.55061006	2.486148137	0.928136507	NA
ASV_16s_7151	0.705543423	0.296140127	3.549820058	2.147655059	NA	NA
ASV_16s_7152	0.701647833	0.363410391	3.549979073	1.179513222	0.991390976	NA
ASV_16s_7153	0.55724241	0.360419297	3.550776323	1.498900098	NA	NA
ASV_16s_7154	0.6534231	0.315939305	3.550697254	1.540800717	0.980863429	NA
ASV_16s_7155	0.81316844	0.735154559	3.550708093	1.627356195	0.977570371	NA
ASV_16s_7156	0.704114068	0.693819531	3.550657745	1.513340022	0.981844232	NA
ASV_16s_7157	0.497713832	0.271581475	3.550106722	0.969122887	NA	NA
ASV_16s_7158	0.498027556	-0.169720003	3.54959213	2.012359356	NA	NA
ASV_16s_7159	0.53696955	-0.284846433	3.549508531	2.060983319	0.956388038	NA
ASV_16s_7160	0.545190097	-0.357923116	3.549489428	1.998038588	0.959948848	NA
ASV_16s_7161	0.177926702	-0.601270165	3.550887509	0.651566762	NA	NA
ASV_16s_7162	0.177926702	-0.601270165	3.550887509	0.651566762	NA	NA
ASV_16s_7163	0.548458026	0.051198778	3.548927681	2.151361368	0.950989782	NA
ASV_16s_7164	0.525581809	0.251357617	3.548939731	2.198436774	NA	NA
ASV_16s_7165	0.53394865	0.127428034	3.548911608	2.258033333	0.944190321	NA
ASV_16s_7166	0.519448517	0.046137707	3.548860498	2.179241847	0.949257078	NA
ASV_16s_7167	0.640988029	-0.248201392	3.547819341	2.10147875	0.954010708	NA
ASV_16s_7168	0.646923245	-0.801316079	3.549092039	1.433019689	0.984538369	NA
ASV_16s_7169	0.649898731	-0.792178822	3.548861156	1.923054045	0.963976029	NA
ASV_16s_7170	0.582386372	1.861088978	3.35161279	1.675479841	0.975605999	NA
ASV_16s_7171	0.623432961	-0.791152505	3.549078572	1.909570937	0.964675311	NA
ASV_16s_7172	0.609706982	-1.00470121	3.548581608	1.434182413	NA	NA
ASV_16s_7173	0.737003731	-0.315909564	3.547700922	2.219536975	NA	NA
ASV_16s_7174	0.494256574	-0.443326871	3.548110649	0.75268564	NA	NA
ASV_16s_7175	0.612164283	-0.361706171	3.548881035	2.004455736	0.959593342	NA
ASV_16s_7176	0.571524959	-0.083590696	3.549839256	1.620509194	0.977842082	NA
ASV_16s_7177	0.436628422	0.341778285	3.550406221	0.969294064	0.995308774	NA
ASV_16s_7178	0.787733519	0.79296532	3.31781912	2.124341909	0.952638739	NA
ASV_16s_7179	0.588582532	0.838998282	3.550627509	1.488838313	NA	NA
ASV_16s_7180	0.596093927	-1.874199562	3.548208399	0.536940086	NA	NA
ASV_16s_7181	0.270528192	1.305033507	3.550521249	0.683458962	NA	NA
ASV_16s_7182	0.231693487	0.406290221	3.550952753	0.654489666	NA	NA
ASV_16s_7183	0.179528507	0.383165952	3.551273098	0.570400021	NA	NA
ASV_16s_7184	0.369442798	0.3575327	3.550645323	1.896175166	0.965362549	NA
ASV_16s_7185	0.378580957	0.555203196	3.550517022	1.275326683	0.989087869	NA
ASV_16s_7186	0.645437052	0.48895768	3.547565169	1.920800338	NA	NA
ASV_16s_7187	0.531569883	-0.069423586	3.549671617	2.128423636	0.952391551	NA
ASV_16s_7188	0.545382623	-0.06941687	3.549616864	2.171004284	0.949772313	NA
ASV_16s_7189	0.499515242	0.055861664	3.549612204	2.703122124	0.911041625	NA
ASV_16s_7190	0.543106537	-0.399099192	3.548748356	2.38553254	0.935467178	NA
ASV_16s_7191	0.607834472	0.366200675	3.550853999	0.997221347	NA	NA
ASV_16s_7192	0.551553054	-0.107576051	3.5499154	1.846119051	NA	NA
ASV_16s_7193	0.828774357	0.580287087	3.548561301	2.054139562	0.956783133	NA
ASV_16s_7194	0.552711984	0.360418847	3.55077782	1.494742954	NA	NA
ASV_16s_7195	0.507874747	-0.182968092	3.549572601	2.034708206	NA	NA
ASV_16s_7196	0.517789461	-0.201276931	3.549552463	2.055561113	0.956701225	NA
ASV_16s_7197	0.562884531	-0.30358057	3.54947431	2.091424487	0.954607252	NA
ASV_16s_7198	0.53706994	-0.307561369	3.54950428	2.042170037	0.957469492	NA
ASV_16s_7199	0.618117229	-0.413941995	3.549411073	2.023323532	0.958538183	NA
ASV_16s_7200	0.566273456	-0.601489686	3.549496315	1.36693871	0.986562447	NA
ASV_16s_7201	0.176468287	-0.598903742	3.550896006	0.648732546	NA	NA
ASV_16s_7202	0.494324402	-0.682883769	3.549637096	0.986499059	NA	NA
ASV_16s_7203	0.248107741	0.596176265	3.550660657	0.81681065	0.997268586	NA
ASV_16s_7204	0.53523053	-0.091834242	3.54947946	2.512711622	0.926136829	NA
ASV_16s_7205	0.642769856	-0.794233808	3.548819736	1.406659393	0.98536642	NA

ASV_16s_7206	0.584934998	0.086950468	3.548805129	2.393589975	0.934894482	NA
ASV_16s_7207	0.309288022	1.533010185	3.550363897	0.603288202	NA	NA
ASV_16s_7208	0.636757512	-0.222380434	3.548267696	1.415846701	0.985080944	NA
ASV_16s_7209	0.634860753	-0.881063763	3.548513957	2.078362582	0.955376039	NA
ASV_16s_7210	0.565373806	0.457457279	3.547734733	1.359868204	NA	NA
ASV_16s_7211	0.631151935	-0.791089472	3.54908476	1.92205968	0.964027859	NA
ASV_16s_7212	0.637522027	-0.02004905	3.547977358	1.907191396	0.964797935	NA
ASV_16s_7213	0.587954939	1.790041416	3.54774624	1.217942928	0.990507881	NA
ASV_16s_7214	0.62797247	-0.721412761	3.549568812	1.910448427	0.964630032	NA
ASV_16s_7215	0.605484844	-0.757471385	3.548225614	1.665823018	0.976007874	NA
ASV_16s_7216	0.7779974	1.871962408	3.548249715	1.10309504	0.99298975	NA
ASV_16s_7217	0.548823937	-0.124092457	3.550461804	1.710128988	0.974132187	NA
ASV_16s_7218	0.655361152	-0.817939258	3.55000296	1.05959018	NA	NA
ASV_16s_7219	0.659771499	-0.271670961	3.549655103	1.223601995	0.990373275	NA
ASV_16s_7220	0.674107724	0.229350564	3.549709567	2.677287058	NA	NA
ASV_16s_7221	0.649925691	0.322021842	3.550589105	1.532547668	0.98116142	NA
ASV_16s_7222	0.545394776	-0.375737585	3.549282856	1.577728445	0.97949606	NA
ASV_16s_7223	0.583413588	0.522055549	3.547957166	1.485812658	NA	NA
ASV_16s_7224	0.529212489	-0.069482344	3.549768082	2.071469089	0.955778938	NA
ASV_16s_7225	0.437002646	2.115463037	3.549717775	0.487718755	0.999490219	NA
ASV_16s_7226	0.473117651	-0.564713075	3.548824241	1.426307744	0.984751823	NA
ASV_16s_7227	0.712728451	0.374248528	3.550356159	0.86986217	0.996663913	NA
ASV_16s_7228	0.648472369	-0.550602622	3.549199394	0.980679674	NA	NA
ASV_16s_7229	0.81956571	0.647527991	3.549011968	1.958110796	0.962122379	NA
ASV_16s_7230	0.852631748	0.706948182	3.549473833	2.463082949	0.929851206	NA
ASV_16s_7231	0.649416724	0.355220188	3.550024498	1.942350497	0.962962053	NA
ASV_16s_7232	0.727467795	0.343438585	3.549561854	2.249069532	0.944779337	NA
ASV_16s_7233	0.783499224	0.363509527	3.549795278	1.209482069	NA	NA
ASV_16s_7234	0.633834398	0.315939339	3.55070065	1.525318438	0.981420169	NA
ASV_16s_7235	0.750971624	0.315939189	3.550682978	1.611573368	0.978193777	NA
ASV_16s_7236	0.519314473	-0.168969887	3.54956228	2.052698129	0.956866101	NA
ASV_16s_7237	0.489913945	-0.222166072	3.549585942	2.000069022	NA	NA
ASV_16s_7238	0.518175658	-0.26305824	3.549537061	2.040165518	0.957583857	NA
ASV_16s_7239	0.499598851	-0.293553834	3.549556698	1.98431434	0.960703425	NA
ASV_16s_7240	0.495812038	-0.303695153	3.54956041	1.968421767	NA	NA
ASV_16s_7241	0.581277682	-0.389851552	3.549447805	2.009527932	0.959311136	NA
ASV_16s_7242	0.499408381	-0.362521859	3.549550093	1.908320925	0.964739757	NA
ASV_16s_7243	0.565404344	-0.420733708	3.549467197	1.926890966	0.963775646	NA
ASV_16s_7244	0.175009871	-0.596520766	3.550904613	0.645881095	NA	NA
ASV_16s_7245	0.156193304	-0.169870159	3.550676702	0.684697122	NA	NA
ASV_16s_7246	0.217738864	0.220032776	3.550709507	0.820101131	NA	NA
ASV_16s_7247	0.520885851	-0.329762171	3.549489228	1.774771991	0.971248972	NA
ASV_16s_7248	0.614133609	-0.023192968	3.549142566	1.190723521	NA	NA
ASV_16s_7249	0.499239705	-0.340095896	3.548815169	1.313256835	NA	NA
ASV_16s_7250	0.522644983	0.00219021	3.548629445	2.187791592	0.948719405	NA
ASV_16s_7251	0.603730249	-0.589080732	3.129245002	1.595450475	0.978820022	NA
ASV_16s_7252	0.437832675	-0.619610437	3.549725279	1.103659739	NA	NA
ASV_16s_7253	0.528994817	0.675448805	3.548181909	2.10778947	NA	NA
ASV_16s_7254	0.579336382	0.599267133	3.548061306	2.223981819	0.946410728	NA
ASV_16s_7255	0.614914866	-0.792178804	3.548880308	1.861211563	0.967120964	NA
ASV_16s_7256	0.618315634	-0.792056966	3.54890001	1.873191867	0.966524194	NA
ASV_16s_7257	0.581623566	1.601609584	3.115793541	1.091458293	0.993215264	NA
ASV_16s_7258	0.589444101	-0.849388744	3.548963509	1.605898736	0.97841541	NA
ASV_16s_7259	0.799186378	2.8350973	3.547791196	0.501463724	0.999441104	NA
ASV_16s_7260	0.539385564	-0.073961694	3.549742456	1.847230679	0.967809804	NA
ASV_16s_7261	0.358597762	0.01104944	3.550609409	1.089063094	0.993261103	NA
ASV_16s_7262	0.545731626	-0.792027569	3.549152992	0.846958032	NA	NA
ASV_16s_7263	0.448532787	-0.265706983	3.549916533	1.187186479	NA	NA
ASV_16s_7264	0.376165119	-0.293258447	3.5505623	1.202191679	0.990876347	NA
ASV_16s_7265	0.318133286	1.331825609	3.55037378	0.738773454	NA	NA
ASV_16s_7266	0.176585417	0.381557605	3.551293111	0.564996469	NA	NA
ASV_16s_7267	0.343926579	0.219995665	3.55066071	1.050752677	0.993967756	NA
ASV_16s_7268	0.680570836	1.218030145	3.547953357	1.82728142	NA	NA
ASV_16s_7269	0.590457498	2.455275863	3.50693117	0.53415852	0.999311545	NA
ASV_16s_7270	0.550555926	2.148354544	3.549189675	1.082624052	NA	NA
ASV_16s_7271	0.52036177	-0.069161823	3.549647373	2.087733688	NA	NA
ASV_16s_7272	0.52036177	-0.069161823	3.549647373	2.087733688	NA	NA

ASV_16s_7273	0.692502627	1.767346899	3.549380205	0.736199337	0.998041875	NA
ASV_16s_7274	0.294336067	0.281453646	3.551333797	0.830075383	NA	NA
ASV_16s_7275	0.704428733	0.409535186	3.550433711	1.727224525	0.973386629	NA
ASV_16s_7276	0.661686852	0.262251307	3.549583673	2.878633693	NA	NA
ASV_16s_7277	0.681181042	0.36130848	3.550687624	2.275782394	0.943014543	NA
ASV_16s_7278	0.776745579	0.825744872	3.549656381	2.486151815	0.928136232	NA
ASV_16s_7279	0.689136109	0.315939253	3.550691561	1.567844496	0.979867508	NA
ASV_16s_7280	0.568732017	1.034138389	3.549558714	0.909349311	NA	NA
ASV_16s_7281	0.708985872	0.98535066	3.550539929	1.326077391	0.987728658	NA
ASV_16s_7282	0.718150073	0.693829095	3.550655688	1.523362532	NA	NA
ASV_16s_7283	0.541294814	-0.114424829	3.549559331	2.061628964	NA	NA
ASV_16s_7284	0.493470695	-0.271769032	3.54957029	1.987627205	0.960521996	NA
ASV_16s_7285	0.546875726	-0.329464126	3.549489477	2.036908544	0.957769324	NA
ASV_16s_7286	0.628494759	-0.683742417	3.549444944	1.063850387	NA	NA
ASV_16s_7287	0.486153751	-0.682816437	3.549652218	0.981120857	NA	NA
ASV_16s_7288	0.154891693	-0.169864497	3.550678133	0.68074076	NA	NA
ASV_16s_7289	0.149709152	-0.092355107	3.550684802	0.958665063	0.995468053	NA
ASV_16s_7290	0.224732113	0.596147314	3.550672159	0.76756004	NA	NA
ASV_16s_7291	0.504518828	-0.112451821	3.549369256	1.861807671	NA	NA
ASV_16s_7292	0.550175993	-0.216883572	3.549069622	1.857622651	0.967298569	NA
ASV_16s_7293	0.497983287	0.09218196	3.548861048	2.150096709	0.951067627	NA
ASV_16s_7294	0.55717373	0.055394914	3.548917434	1.796376029	0.970246514	NA
ASV_16s_7295	0.536220339	0.080047912	3.548616418	2.333377753	0.939112924	NA
ASV_16s_7296	0.554060017	0.329576254	3.549030773	2.088530689	0.954778178	NA
ASV_16s_7297	0.574208342	-0.273045773	3.548301585	2.066363782	0.956076063	NA
ASV_16s_7298	0.597627448	-0.793304601	3.548723003	1.696228509	NA	NA
ASV_16s_7299	0.522055511	0.197246806	3.548669472	2.387795961	NA	NA
ASV_16s_7300	0.662751167	-0.818133372	3.3057284	1.832130091	0.968544634	NA
ASV_16s_7301	0.637939239	-0.79246209	3.548823091	1.891125953	0.965619643	NA
ASV_16s_7302	0.586066317	1.117553522	3.548355931	1.888479931	NA	NA
ASV_16s_7303	0.76671146	-0.785041704	3.333376544	2.584348095	0.920612789	NA
ASV_16s_7304	0.606052688	-0.819601935	3.549184781	1.325264184	NA	NA
ASV_16s_7305	0.614581674	-0.791101747	3.549089031	1.896310703	0.965355633	NA
ASV_16s_7306	0.626519197	-0.35809141	3.548698978	2.131458436	0.952207322	NA
ASV_16s_7307	0.54241979	0.055636585	3.54949612	1.773517544	0.971306582	NA
ASV_16s_7308	0.599945008	0.002188806	3.549121391	2.182231062	0.949069431	NA
ASV_16s_7309	0.578634989	-0.815804272	3.549400668	1.648289756	0.976727655	NA
ASV_16s_7310	0.614287523	0.443374669	3.550282148	1.913701445	0.964461894	NA
ASV_16s_7311	0.548461255	-0.086370979	3.549904734	1.727161945	0.97338938	NA
ASV_16s_7312	0.538219817	1.339033571	3.549869644	0.971795787	NA	NA
ASV_16s_7313	0.475128157	1.182328118	3.550030403	1.093318877	NA	NA
ASV_16s_7314	0.662948676	-0.009453605	3.547659669	2.083901186	NA	NA
ASV_16s_7315	0.315482176	1.330956836	3.550382832	0.735982917	NA	NA
ASV_16s_7316	0.33987436	0.219996294	3.550661714	1.044738802	0.994074194	NA
ASV_16s_7317	0.343594868	0.219995634	3.550660791	1.050256499	0.993976583	NA
ASV_16s_7318	0.346392521	0.219995093	3.550660109	1.054366778	0.993903209	NA
ASV_16s_7319	0.66623221	-0.627443978	3.549217465	1.202405665	NA	NA
ASV_16s_7320	0.67813372	1.507902731	3.548720861	1.798907108	0.970127793	NA
ASV_16s_7321	0.491398564	1.741239174	3.550147261	0.818875738	NA	NA
ASV_16s_7322	0.63614849	0.402244306	3.550905706	0.923049434	NA	NA
ASV_16s_7323	0.641430136	0.723058866	3.550632446	1.698042626	0.97465195	NA
ASV_16s_7324	0.682867733	0.247986421	3.55024939	2.691080357	NA	NA
ASV_16s_7325	0.809668558	2.272622779	3.548812665	0.658555706	NA	NA
ASV_16s_7326	0.291883267	0.281052416	3.551342215	0.827118506	NA	NA
ASV_16s_7327	0.250783956	0.279705212	3.551441207	0.772183323	NA	NA
ASV_16s_7328	0.601095177	0.323705149	3.550740331	1.157752925	0.991867321	NA
ASV_16s_7329	0.77427533	1.082703876	3.550330819	1.715005499	NA	NA
ASV_16s_7330	0.616990644	0.315939184	3.550703742	1.511614853	0.981904828	NA
ASV_16s_7331	0.585333626	0.315939368	3.550710033	1.484855934	0.98282932	NA
ASV_16s_7332	0.703579044	0.693820589	3.550657827	1.512929905	0.981858648	NA
ASV_16s_7333	0.694058934	0.693811727	3.550659274	1.505985342	0.982101724	NA
ASV_16s_7334	0.493046694	-0.130106598	3.549618717	1.983275885	NA	NA
ASV_16s_7335	0.509141235	-0.257730687	3.549550253	2.025736402	0.958402182	NA
ASV_16s_7336	0.487607162	-0.26222935	3.549580564	1.981435653	0.960860705	NA
ASV_16s_7337	0.513454401	-0.375279376	3.54953027	1.915861888	0.964349984	NA
ASV_16s_7338	0.520524189	-0.411182695	3.549522499	1.868821533	NA	NA
ASV_16s_7339	0.153590083	-0.169857328	3.550679588	0.676756283	NA	NA

ASV_16s_7340	0.106757708	-0.169553719	3.550755396	0.512977797	NA	NA
ASV_16s_7341	0.214109883	0.220034518	3.550711761	0.811699271	NA	NA
ASV_16s_7342	0.547714699	-0.532578515	3.549448438	1.843385508	0.967997823	NA
ASV_16s_7343	0.518824693	0.1240695	3.549485626	2.063952353	0.956216032	NA
ASV_16s_7344	0.51989144	0.122072507	3.549234839	2.090459585	0.954664283	NA
ASV_16s_7345	0.519557238	0.111047937	3.549094728	2.127952078	0.952420143	NA
ASV_16s_7346	0.607969955	-0.03094082	3.548534938	2.306710538	0.940935746	NA
ASV_16s_7347	0.52962744	0.564349313	3.54861966	2.005211498	0.959551361	NA
ASV_16s_7348	0.509707322	-0.332823013	3.548724563	1.539987036	NA	NA
ASV_16s_7349	0.61330512	0.153805397	3.548563438	2.621967452	0.917636269	NA
ASV_16s_7350	0.438315096	0.58088501	3.549039515	1.861790149	NA	NA
ASV_16s_7351	0.620856378	-0.793548096	3.548666504	1.711528241	0.974071621	NA
ASV_16s_7352	0.574102195	-0.359533602	3.547924371	1.837399524	0.968289293	NA
ASV_16s_7353	0.5860111	-0.386523575	3.548913413	1.758827819	0.971976306	NA
ASV_16s_7354	0.554311339	0.723155797	3.548187944	1.852775504	0.967537587	NA
ASV_16s_7355	0.614419303	-0.792158338	3.548862056	1.854623324	0.967446585	NA
ASV_16s_7356	0.62435294	-0.792357372	3.548846669	1.870680922	0.966649767	NA
ASV_16s_7357	0.572636664	0.809536459	3.547997654	1.899224773	NA	NA
ASV_16s_7358	0.599950023	-0.532580776	3.549198575	1.810687953	0.969571684	NA
ASV_16s_7359	0.716715695	0.682934683	3.548077659	1.736136147	0.972993155	NA
ASV_16s_7360	0.575717947	-1.862480805	3.548348984	0.549674268	NA	NA
ASV_16s_7361	0.603575332	1.217527094	3.548180318	0.935611365	NA	NA
ASV_16s_7362	0.831207494	-1.488629026	3.547525082	1.918931636	NA	NA
ASV_16s_7363	0.706967566	-1.07112967	3.548144368	2.233270384	NA	NA
ASV_16s_7364	0.521601647	-0.773064433	3.549330823	1.627129784	0.977579387	NA
ASV_16s_7365	0.501047147	-0.217723521	3.548733832	0.969905171	NA	NA
ASV_16s_7366	0.525333974	-0.074940686	3.549998815	1.78157916	0.970935213	NA
ASV_16s_7367	0.688477485	2.074344458	3.549390926	0.748846087	0.997931633	NA
ASV_16s_7368	0.426152353	1.317778289	3.549880853	1.339955686	0.987339806	NA
ASV_16s_7369	0.272997954	0.617630453	3.550368513	1.082852727	NA	NA
ASV_16s_7370	0.173642327	0.380218265	3.551311616	0.55946496	NA	NA
ASV_16s_7371	0.173642327	0.380218265	3.551311616	0.55946496	NA	NA
ASV_16s_7372	0.173642327	0.380218265	3.551311616	0.55946496	NA	NA
ASV_16s_7373	0.338721767	0.219996578	3.550662005	1.043010177	0.994104566	NA
ASV_16s_7374	0.340195574	0.219996297	3.550661634	1.045210425	0.994065891	NA
ASV_16s_7375	0.341956854	0.219995949	3.550661196	1.047827524	0.994019678	NA
ASV_16s_7376	0.354454221	0.219993826	3.550658207	1.06602332	NA	NA
ASV_16s_7377	0.354454221	0.219993826	3.550658207	1.06602332	NA	NA
ASV_16s_7378	0.353746401	0.21999395	3.550658371	1.065010042	NA	NA
ASV_16s_7379	0.493792611	-0.069622962	3.550185811	1.840349406	0.968145845	NA
ASV_16s_7380	0.493164952	-0.069567335	3.550009259	1.916266279	0.964329015	NA
ASV_16s_7381	0.524441715	-0.059607996	3.550014562	1.704282373	0.974384374	NA
ASV_16s_7382	0.510408999	-0.069470977	3.549734463	2.066901866	0.956044797	NA
ASV_16s_7383	0.437065901	0.616308429	3.549591282	1.684037311	0.975246642	NA
ASV_16s_7384	0.494466538	0.165801997	3.548803731	2.052182247	0.956895775	NA
ASV_16s_7385	0.65380054	0.523696018	3.548791691	2.710173213	0.910457625	NA
ASV_16s_7386	0.402638485	0.765125858	3.550857487	1.418579527	NA	NA
ASV_16s_7387	0.645907003	-0.077402099	3.550116393	1.726097527	0.973436153	NA
ASV_16s_7388	0.715850104	0.669813496	3.549245716	1.883390894	0.966011428	NA
ASV_16s_7389	0.681528126	0.343876488	3.549678264	2.102119511	0.953972549	NA
ASV_16s_7390	0.634402097	0.315939249	3.550700548	1.525777123	0.981403815	NA
ASV_16s_7391	0.626956605	0.315939248	3.550701894	1.519743813	0.981618247	NA
ASV_16s_7392	0.491297342	-0.187883808	3.549595004	2.003094946	NA	NA
ASV_16s_7393	0.509744201	-0.263814775	3.54954842	2.023657963	NA	NA
ASV_16s_7394	0.483009703	-0.253325044	3.54958917	1.976543009	NA	NA
ASV_16s_7395	0.483780755	-0.265892764	3.549585788	1.971783931	0.961385526	NA
ASV_16s_7396	0.526564959	-0.319726985	3.54951594	2.010943024	0.959232213	NA
ASV_16s_7397	0.486847853	-0.298668382	3.549574572	1.955091836	0.962284024	NA
ASV_16s_7398	0.523005335	-0.392241165	3.549517843	1.90619079	0.964849428	NA
ASV_16s_7399	0.170634624	-0.588800317	3.550928958	0.637175489	NA	NA
ASV_16s_7400	0.23965973	0.145818675	3.550671317	1.329308993	0.987638775	NA
ASV_16s_7401	0.160575052	0.220068766	3.550756777	0.671055642	NA	NA
ASV_16s_7402	0.160575052	0.220068766	3.550756777	0.671055642	NA	NA
ASV_16s_7404	0.487537799	0.337128149	3.549380917	1.919584362	NA	NA
ASV_16s_7405	0.521934603	0.740467453	3.548798891	1.745583526	0.972572412	NA
ASV_16s_7406	0.659862799	-0.793824304	3.548585581	1.749934378	0.972377394	NA
ASV_16s_7407	0.635396406	-0.67652161	3.548653061	1.826861056	0.968798795	NA

ASV_16s_7408	0.618182526	-0.792880921	3.548752452	1.813775736	0.969424968	NA
ASV_16s_7409	0.538612588	0.315428138	3.548840856	1.941374625	NA	NA
ASV_16s_7410	0.552865207	0.148400565	3.548765935	2.382538086	0.935679371	NA
ASV_16s_7411	0.614657953	-0.79154059	3.549002628	1.885984097	0.965880359	NA
ASV_16s_7412	0.640711753	-0.791771976	3.548945372	1.921915099	0.964035392	NA
ASV_16s_7413	0.612012947	-0.66501741	3.54975029	1.860466847	0.967157862	NA
ASV_16s_7414	0.528124816	-0.866892162	3.549594573	1.101888884	NA	NA
ASV_16s_7415	0.756688183	0.750720403	3.549850352	1.597468552	0.978742221	NA
ASV_16s_7416	0.510512637	-0.384208838	3.547826483	1.377655392	NA	NA
ASV_16s_7417	0.563268318	-0.035518536	3.549056571	1.401135524	NA	NA
ASV_16s_7418	0.655616628	1.589681819	3.54758332	0.705967662	0.998289463	NA
ASV_16s_7419	0.487181225	1.14473853	3.548966718	1.190355052	NA	NA
ASV_16s_7420	0.558366075	-1.192851044	3.548651467	1.930117769	0.963606651	NA
ASV_16s_7421	0.223895855	0.803376145	3.55077558	0.866162085	NA	NA
ASV_16s_7422	0.476708905	0.357328108	3.548511485	1.220417293	NA	NA
ASV_16s_7423	0.583478711	1.41320243	3.549718113	1.256941309	NA	NA
ASV_16s_7424	0.400799725	0.787134008	3.549675238	0.978808924	NA	NA
ASV_16s_7425	0.436831063	0.899246873	3.548583116	1.053555649	NA	NA
ASV_16s_7426	0.172170781	0.379569046	3.551320868	0.556665718	NA	NA
ASV_16s_7427	0.330248127	0.219998271	3.550664201	1.030165699	0.994327133	NA
ASV_16s_7428	0.332015405	0.219997916	3.550663734	1.032869747	0.994280732	NA
ASV_16s_7429	0.33713174	0.219996887	3.550662409	1.040618517	0.994146423	NA
ASV_16s_7430	0.68975457	0.905512897	3.548746136	1.269541263	NA	NA
ASV_16s_7431	0.474448137	1.86956576	3.549685067	0.887583905	NA	NA
ASV_16s_7432	0.530830677	-0.068983732	3.549672792	2.062106802	NA	NA
ASV_16s_7433	0.517176377	-0.068398134	3.549825649	1.926048069	NA	NA
ASV_16s_7434	0.670370428	0.244022428	3.550178631	2.687181497	NA	NA
ASV_16s_7435	0.142798172	0.281001268	3.55165187	0.569627681	NA	NA
ASV_16s_7436	0.152827474	-0.463272979	3.551326143	0.587544318	NA	NA
ASV_16s_7437	0.469164803	1.212915403	3.550258225	0.907754639	NA	NA
ASV_16s_7438	0.667586419	0.471235403	3.550556075	1.587894093	0.97910985	NA
ASV_16s_7439	0.530059853	0.360416391	3.550785685	1.473433627	NA	NA
ASV_16s_7440	0.590049347	0.315939267	3.550709054	1.488912772	0.982691018	NA
ASV_16s_7441	0.646463333	0.315939274	3.55069844	1.535312854	0.981061887	NA
ASV_16s_7442	0.603149947	0.513786482	3.550691657	2.643098414	0.915941837	NA
ASV_16s_7443	0.866192317	1.187010562	3.54970397	1.221332076	0.990427409	NA
ASV_16s_7444	0.808107788	1.187051144	3.549694449	1.187699806	0.991207355	NA
ASV_16s_7445	0.703783137	1.186662444	3.549787161	1.140071178	0.992241934	NA
ASV_16s_7446	0.610125394	0.693761598	3.550673973	1.440445355	0.98430013	NA
ASV_16s_7447	0.700196321	0.693819185	3.550658338	1.510464106	NA	NA
ASV_16s_7448	0.48721772	-0.190692734	3.549600175	1.99532368	NA	NA
ASV_16s_7449	0.481209014	-0.258224063	3.549591048	1.970605427	NA	NA
ASV_16s_7450	0.49859815	-0.431067938	3.549556108	1.79230926	0.970436708	NA
ASV_16s_7451	0.515665307	-0.437216031	3.549532436	1.810141486	0.969597608	NA
ASV_16s_7452	0.503002293	-0.46267858	3.5495555	1.732830394	0.9731395	NA
ASV_16s_7453	0.169176209	-0.586157054	3.550937158	0.634228073	NA	NA
ASV_16s_7454	0.186216087	0.086973555	3.550670489	1.482588829	0.982906319	NA
ASV_16s_7456	0.503349034	0.386526613	3.54904434	1.908753909	0.964717441	NA
ASV_16s_7457	0.541319181	-0.322325076	3.548973106	1.767871555	NA	NA
ASV_16s_7458	0.575937617	-0.299062097	3.549197501	1.55998811	0.980159907	NA
ASV_16s_7459	0.521148503	0.552955752	3.548935238	1.854747262	0.967440476	NA
ASV_16s_7460	0.621171236	-0.793762776	3.548663536	1.659279358	0.976277995	NA
ASV_16s_7461	0.591533837	-0.793282278	3.548731033	1.664081565	NA	NA
ASV_16s_7462	0.558478775	-0.774690063	3.548081309	1.653964236	NA	NA
ASV_16s_7463	0.651327731	-0.833034748	3.547873246	1.366639892	0.986571211	NA
ASV_16s_7464	0.610565446	0.126097469	3.548861344	2.403907597	0.934157463	NA
ASV_16s_7465	0.545935428	-0.80486613	3.549111552	1.415496343	NA	NA
ASV_16s_7466	0.595410865	-0.762281944	3.54940185	1.88092331	0.966135887	NA
ASV_16s_7467	0.603470731	0.202148128	3.549214254	1.914740421	NA	NA
ASV_16s_7468	0.580009179	0.220189339	3.548404227	2.424190861	NA	NA
ASV_16s_7469	0.590972748	-0.08602124	3.548802046	1.793332207	0.970388932	NA
ASV_16s_7470	0.442952305	-0.217467385	3.549705992	1.503113709	0.982201668	NA
ASV_16s_7471	0.559629854	-0.09106171	3.549908015	1.710255382	NA	NA
ASV_16s_7472	0.884588233	1.043987866	3.549484718	1.297014251	NA	NA
ASV_16s_7473	0.572489446	-0.384561686	3.548363764	1.276573073	NA	NA
ASV_16s_7474	0.742988295	-0.41003498	3.549533785	1.27041659	NA	NA
ASV_16s_7475	0.631499231	0.666373302	3.547244654	1.682683222	NA	NA

ASV_16s_7476	0.575016188	0.396425208	3.548308913	1.996563814	NA	NA
ASV_16s_7477	0.491457647	-0.59970324	3.549278555	1.09385142	NA	NA
ASV_16s_7478	0.573034437	-1.21544471	3.548921451	0.773143055	NA	NA
ASV_16s_7479	0.381125324	1.336330363	3.550140035	0.79723148	NA	NA
ASV_16s_7480	0.615798732	0.324744871	3.550397929	0.992469688	NA	NA
ASV_16s_7481	0.170699236	0.379073244	3.551329126	0.553823747	NA	NA
ASV_16s_7482	0.243034598	1.283549123	3.550615024	0.646530563	NA	NA
ASV_16s_7483	0.327250276	0.219998901	3.550665006	1.025538249	0.994405982	NA
ASV_16s_7484	0.348446522	0.219994912	3.550659618	1.057343278	NA	NA
ASV_16s_7485	0.367060913	0.59627124	3.550624794	1.014346816	NA	NA
ASV_16s_7486	0.360275174	0.224084376	3.550701889	0.847358901	0.996930067	NA
ASV_16s_7487	0.395265898	0.645411748	3.55041165	0.948928935	NA	NA
ASV_16s_7488	0.4396577	0.92017192	3.54859785	1.013575883	NA	NA
ASV_16s_7489	0.328576086	0.286295227	3.551185885	0.867835143	NA	NA
ASV_16s_7490	0.744539037	0.316156578	3.547571655	1.658946176	NA	NA
ASV_16s_7491	0.622684599	0.354752432	3.550760902	0.848553547	0.996916299	NA
ASV_16s_7492	0.885245294	0.643546521	3.549511445	2.63986596	0.91620208	NA
ASV_16s_7493	0.754790504	0.901515362	3.549795485	2.211326405	0.947224067	NA
ASV_16s_7494	0.750257578	0.633224548	3.549504894	2.339870158	0.938664896	NA
ASV_16s_7495	0.624022795	0.334247484	3.550701382	1.244547239	0.989864779	NA
ASV_16s_7496	0.731671978	1.186241848	3.54988454	1.174594152	0.991500145	NA
ASV_16s_7497	0.46943598	-0.151998085	3.549645312	1.948072426	NA	NA
ASV_16s_7498	0.475886135	-0.285945369	3.549593938	1.943007974	0.962927231	NA
ASV_16s_7499	0.517123736	-0.314971356	3.549529014	1.99827827	0.959935601	NA
ASV_16s_7500	0.477203545	-0.345154981	3.5495843	1.887953137	0.96578065	NA
ASV_16s_7501	0.513366732	-0.450978528	3.549538043	1.777426937	0.97112683	NA
ASV_16s_7502	0.167717793	-0.58349212	3.550945472	0.631258113	NA	NA
ASV_16s_7503	0.090201535	0.774134364	3.551897969	0.318768555	0.999877185	NA
ASV_16s_7504	0.159303053	-0.169883275	3.550673384	0.694012709	NA	NA
ASV_16s_7505	0.15783018	0.22007116	3.550759907	0.662834919	NA	NA
ASV_16s_7506	0.51639649	-0.045485684	3.549414827	1.942889259	0.96293352	NA
ASV_16s_7507	0.584605867	-0.485506533	3.549352362	1.775973625	0.971193727	NA
ASV_16s_7508	0.501104301	0.438525234	3.54927506	2.016619547	NA	NA
ASV_16s_7509	0.513710536	-0.54091495	3.549456339	1.623239679	0.977733959	NA
ASV_16s_7510	0.586860839	-0.793287217	3.548742507	1.659230777	0.976279994	NA
ASV_16s_7511	0.538192167	-0.019683414	3.548569889	2.276049028	0.942996784	NA
ASV_16s_7512	0.594563148	-0.793203115	3.548730079	1.691440757	0.974933293	NA
ASV_16s_7513	0.644136427	-0.793620402	3.548627863	1.770502184	0.971444791	NA
ASV_16s_7514	0.631732075	-0.792878984	3.548668397	1.780711982	0.970975291	NA
ASV_16s_7515	0.601427018	-0.793205518	3.548727045	1.72782106	NA	NA
ASV_16s_7516	0.570105107	-0.484775522	3.550246734	1.193761217	0.991069836	NA
ASV_16s_7517	0.594721066	-0.626273551	3.52669596	2.219129711	0.946723327	NA
ASV_16s_7518	0.588802492	-0.429381546	3.548029782	1.710432931	0.974119038	NA
ASV_16s_7519	0.592928098	-0.313974507	3.548628611	2.177885097	0.949342128	NA
ASV_16s_7520	0.60603863	-0.788692986	3.549172126	1.889677096	0.965693218	NA
ASV_16s_7521	0.593324479	-0.66831013	3.54896397	1.433122821	0.984535075	NA
ASV_16s_7522	0.544612326	-0.487934169	3.549819479	1.693815941	0.974832282	NA
ASV_16s_7523	0.583050512	-0.953134198	3.548906504	1.573610126	NA	NA
ASV_16s_7524	0.593963954	-0.725687311	3.549565224	1.868140125	0.966776565	NA
ASV_16s_7525	0.594303917	0.683024213	3.550616898	1.695046863	0.974779841	NA
ASV_16s_7526	0.507374557	-0.081602018	3.550202272	1.692708326	NA	NA
ASV_16s_7527	0.471390749	-1.60455469	3.548201267	0.549225062	NA	NA
ASV_16s_7528	0.606403159	1.371532547	3.548367977	1.238363626	0.990016593	NA
ASV_16s_7529	0.53022801	0.413031497	3.550892467	0.715203081	NA	NA
ASV_16s_7530	0.394515096	0.40329028	3.550491829	0.897123583	NA	NA
ASV_16s_7531	0.80431735	0.425465244	3.549972711	0.790361131	NA	NA
ASV_16s_7532	0.869654068	-0.299285101	3.548932793	2.080623822	NA	NA
ASV_16s_7533	0.328266405	0.219998679	3.550664732	1.027101146	0.99437943	NA
ASV_16s_7534	0.363896595	0.596269638	3.550625445	1.009952407	NA	NA
ASV_16s_7535	0.469479029	-0.764421895	3.549569467	0.993440845	0.994933652	NA
ASV_16s_7536	0.27422085	0.299318636	3.55163266	0.489833192	NA	NA
ASV_16s_7537	0.595210995	0.73177375	3.550582507	1.482130139	0.982921872	NA
ASV_16s_7538	0.625522535	0.250373959	3.549690399	2.148105674	NA	NA
ASV_16s_7539	0.554736112	-0.114375854	3.549792117	1.734227175	0.97307772	NA
ASV_16s_7540	0.663710861	1.335988915	3.549543458	1.675454141	0.975607074	NA
ASV_16s_7541	0.150215039	-0.458340106	3.55133658	0.581817312	NA	NA
ASV_16s_7542	0.701130216	0.259025769	3.549443658	1.698947006	0.974613268	NA

ASV_16s_7543	0.760787018	0.495890259	3.549216787	2.170389285	NA	NA
ASV_16s_7544	0.733307574	0.726393743	3.549582915	2.054812955	0.956744343	NA
ASV_16s_7545	0.69254651	0.970241929	3.549689139	1.830227737	0.968636531	NA
ASV_16s_7546	0.561712601	0.342510492	3.549652139	2.139222942	0.951734261	NA
ASV_16s_7547	0.709801703	0.312266185	3.550076564	2.365725662	NA	NA
ASV_16s_7548	0.63748152	0.363419122	3.549990439	1.135538336	0.992336185	NA
ASV_16s_7549	0.57186024	0.629422899	3.550271359	2.234675191	0.945718457	NA
ASV_16s_7550	0.520999001	0.36041539	3.550789022	1.46465441	NA	NA
ASV_16s_7551	0.585169964	0.905074239	3.55031443	2.264983858	0.943731386	NA
ASV_16s_7552	0.644551596	0.654134853	3.550669078	1.995414878	0.960093708	NA
ASV_16s_7553	0.719861424	0.735096193	3.550725272	1.56529441	NA	NA
ASV_16s_7554	0.677993266	0.693805414	3.550661809	1.494048858	0.982514973	NA
ASV_16s_7555	0.473177496	-0.130416164	3.549650418	1.943732544	NA	NA
ASV_16s_7556	0.476577666	-0.249151207	3.54960005	1.965240266	NA	NA
ASV_16s_7557	0.465453328	-0.260454347	3.549615877	1.937220092	NA	NA
ASV_16s_7558	0.470327153	-0.334880301	3.549595968	1.886278778	NA	NA
ASV_16s_7559	0.499004427	-0.375504868	3.549550785	1.888650831	0.965745281	NA
ASV_16s_7560	0.166259377	-0.580804219	3.550953902	0.628265122	NA	NA
ASV_16s_7561	0.539381027	0.532240506	3.54902982	2.129549054	NA	NA
ASV_16s_7562	0.533835014	0.038718151	3.549223866	1.803558606	0.969908914	NA
ASV_16s_7563	0.478902713	0.816647964	3.548224305	0.986509285	0.995043234	NA
ASV_16s_7564	0.640454588	-0.374822279	3.54825135	2.057403896	NA	NA
ASV_16s_7565	0.563968608	0.158047765	3.548159446	2.14929822	0.951116744	NA
ASV_16s_7566	0.624850806	0.118845942	3.548999994	2.352597205	0.937781824	NA
ASV_16s_7567	0.538609197	-0.264693997	3.548365799	2.0438086	0.957375883	NA
ASV_16s_7568	0.554025631	-0.421453587	3.332860309	1.793274707	0.970391619	NA
ASV_16s_7569	0.589783647	-1.518209687	3.548281529	0.80819083	0.997359462	NA
ASV_16s_7570	0.647507505	-0.196140821	3.548863632	2.424879795	0.932646677	NA
ASV_16s_7571	0.545183785	0.28817437	3.548852281	1.810117683	0.969598737	NA
ASV_16s_7572	0.59690963	0.633308816	3.549673086	1.237468452	0.990038453	NA
ASV_16s_7573	0.529974915	-0.093611919	3.549926824	1.594255722	NA	NA
ASV_16s_7574	0.418957663	-0.587225006	3.549698668	0.961636677	NA	NA
ASV_16s_7575	0.610897934	-0.283482659	3.550470664	1.662098376	0.976161844	NA
ASV_16s_7576	0.540008006	0.352005562	3.550881414	1.08556645	NA	NA
ASV_16s_7577	0.644130157	0.376346619	3.550605163	1.511009223	0.981926072	NA
ASV_16s_7578	0.659784083	0.159239272	3.550653183	1.558980405	0.980197229	NA
ASV_16s_7579	0.515605539	1.338834239	3.549916582	0.95793439	NA	NA
ASV_16s_7580	0.656980943	1.907087037	3.549062353	1.279590443	NA	NA
ASV_16s_7581	0.504664095	0.403409714	3.55014956	0.976399188	NA	NA
ASV_16s_7582	0.449204043	-0.647760745	3.549207197	1.201707007	NA	NA
ASV_16s_7583	0.445294442	0.41902038	3.550046957	0.866744542	NA	NA
ASV_16s_7584	0.328132189	0.219998719	3.550664769	1.026886308	0.994383085	NA
ASV_16s_7585	0.342156895	0.219996006	3.550661148	1.048095772	0.994014929	NA
ASV_16s_7586	0.512094877	0.777057452	3.54928379	1.554469109	0.980363805	NA
ASV_16s_7587	0.598209137	2.421367887	3.54889076	0.533504903	0.999314317	NA
ASV_16s_7588	0.509476531	2.278639621	3.549240409	0.514862239	NA	NA
ASV_16s_7589	0.536355268	1.813008077	3.5494315	1.134820498	0.992351045	NA
ASV_16s_7590	0.498707009	-0.069361315	3.54967803	2.077863665	0.955405264	NA
ASV_16s_7591	0.487728512	-0.069302143	3.549673409	2.057552322	NA	NA
ASV_16s_7592	0.48822403	-0.069296465	3.549671432	2.057801344	NA	NA
ASV_16s_7593	0.585345059	2.110342135	3.54939408	0.594949222	NA	NA
ASV_16s_7594	0.646644432	1.326802674	3.550313171	1.066994078	0.993674243	NA
ASV_16s_7595	0.230282434	0.279704528	3.551478175	0.740974754	NA	NA
ASV_16s_7596	0.391911204	-0.606555449	3.550285514	0.90098242	NA	NA
ASV_16s_7597	0.555169006	1.009871794	3.550305423	1.56093281	NA	NA
ASV_16s_7598	0.586866435	0.919344273	3.550465898	1.561832898	0.980091474	NA
ASV_16s_7599	0.709984586	0.378475227	3.548529714	1.994319502	0.960154101	NA
ASV_16s_7600	0.726409038	0.200220538	3.549620714	2.394662003	NA	NA
ASV_16s_7601	0.584734147	0.343483187	3.549633256	2.137545651	0.951836661	NA
ASV_16s_7602	0.718970972	0.829644003	3.549211748	1.401258993	NA	NA
ASV_16s_7603	0.604053771	0.363395428	3.550063745	1.12051438	0.992643411	NA
ASV_16s_7604	0.650178706	0.315939093	3.550697807	1.538191318	0.980957946	NA
ASV_16s_7605	0.650408211	0.315939332	3.550697771	1.538362343	0.980951759	NA
ASV_16s_7606	0.513720717	0.688482538	3.550683824	1.328349991	NA	NA
ASV_16s_7607	0.805786511	1.181506108	3.549653804	1.159307618	NA	NA
ASV_16s_7608	0.481280333	-0.198109139	3.549606755	1.983920672	0.960724953	NA
ASV_16s_7609	0.545871102	-0.328980976	3.549490787	2.035563888	0.957845767	NA

ASV_16s_7610	0.480416151	-0.333558193	3.549580039	1.907933395	NA	NA
ASV_16s_7611	0.48243009	-0.347772058	3.549576026	1.89496129	0.965424454	NA
ASV_16s_7612	0.501738127	-0.357077911	3.549546924	1.920006582	0.964134744	NA
ASV_16s_7613	0.508186283	-0.420294851	3.549540684	1.830016946	0.968646704	NA
ASV_16s_7614	0.4731813	-0.468913978	3.54960362	1.658442528	0.976312411	NA
ASV_16s_7615	0.199960998	0.204770804	3.55066781	1.675771859	0.975593787	NA
ASV_16s_7616	0.477851045	-0.122013282	3.549350747	1.733715531	NA	NA
ASV_16s_7617	0.471691695	0.104987898	3.549495819	1.908024903	NA	NA
ASV_16s_7618	0.465931061	0.066456985	3.549310273	1.95366787	NA	NA
ASV_16s_7619	0.609766642	-0.793663957	3.548688491	1.645323016	NA	NA
ASV_16s_7620	0.57261826	-0.615775746	3.548771517	1.72035989	0.973687466	NA
ASV_16s_7621	0.298916943	-0.222104846	3.549771387	1.352284284	0.986988135	NA
ASV_16s_7622	0.532890781	-0.102861357	3.548590607	2.100620221	0.954061809	NA
ASV_16s_7623	0.601015137	0.336501832	3.547563126	1.834156898	0.968446558	NA
ASV_16s_7624	0.609911026	-0.792519516	3.548845756	1.838840821	0.968219251	NA
ASV_16s_7625	0.495353276	-0.675434297	3.549459874	1.362732576	NA	NA
ASV_16s_7626	0.629593344	-0.792077117	3.548894321	1.893085175	0.96552001	NA
ASV_16s_7627	0.563210525	-0.791144957	3.549105165	1.806052558	NA	NA
ASV_16s_7628	0.741771875	-0.439324921	3.548362053	2.086316486	NA	NA
ASV_16s_7629	0.556957791	-0.865576048	3.549214516	1.574057535	0.979634483	NA
ASV_16s_7630	0.604171823	-0.186485559	3.550548407	0.830583689	0.997119165	NA
ASV_16s_7631	0.527568871	0.399733453	3.550758001	1.625161028	0.977657693	NA
ASV_16s_7632	0.492546851	-0.066274826	3.550926065	1.038261675	NA	NA
ASV_16s_7633	0.665034713	0.26950575	3.549807975	1.511474993	0.981909735	NA
ASV_16s_7634	0.58976241	-0.562233826	3.548189385	2.120472805	0.952872422	NA
ASV_16s_7635	0.674548873	0.202270537	3.549217128	1.625485922	0.977644781	NA
ASV_16s_7636	0.523463471	-0.676254909	3.550161187	1.757957076	0.972015721	NA
ASV_16s_7637	0.602116953	-0.207270844	3.550113202	1.234589565	0.990108552	NA
ASV_16s_7638	0.695988834	0.822258838	3.549684758	1.802302707	0.9699681	NA
ASV_16s_7639	0.605598163	1.204403631	3.549631604	0.998806667	NA	NA
ASV_16s_7640	0.575667136	1.33759645	3.549528802	1.315833792	NA	NA
ASV_16s_7641	0.61115785	1.320380168	3.548466133	2.072978777	NA	NA
ASV_16s_7642	0.58861529	1.060333817	3.548268073	1.834937537	0.968408738	NA
ASV_16s_7643	0.570288319	0.755233398	3.550451421	1.066517933	NA	NA
ASV_16s_7644	0.538154209	0.003753837	3.550943202	0.75300339	NA	NA
ASV_16s_7645	0.299575511	1.323966769	3.550434176	0.71850043	NA	NA
ASV_16s_7646	0.268316802	-0.547641928	3.549920762	0.697422475	NA	NA
ASV_16s_7647	0.656589417	1.655319038	3.548771693	1.633237836	NA	NA
ASV_16s_7648	0.495995927	-0.070881989	3.549764821	1.935636273	0.963316628	NA
ASV_16s_7649	0.480015144	0.452126174	3.550147719	1.423015189	NA	NA
ASV_16s_7650	0.61106628	0.533503535	3.550704748	2.520463074	0.925548309	NA
ASV_16s_7651	0.228262413	0.279692024	3.551482009	0.737741948	NA	NA
ASV_16s_7652	0.228262413	0.279692024	3.551482009	0.737741948	NA	NA
ASV_16s_7653	0.66549027	-0.538488912	3.549358552	2.010273847	NA	NA
ASV_16s_7654	0.992030458	0.98130621	2.889862439	4.673957346	0.699681201	NA
ASV_16s_7655	0.602491552	1.433448905	3.549187777	1.769287014	NA	NA
ASV_16s_7656	0.651062809	1.120431261	3.550031804	1.729158447	0.973301522	NA
ASV_16s_7657	0.869105706	0.27015192	3.55063874	3.131337884	NA	NA
ASV_16s_7658	0.86614119	0.61765002	3.549527627	2.637142351	0.916421065	NA
ASV_16s_7659	0.651855886	0.496534078	3.549709348	2.029142704	0.958209775	NA
ASV_16s_7660	0.580673769	0.343741575	3.549687996	2.091039366	0.954630019	NA
ASV_16s_7661	0.650848033	0.343547891	3.549623089	2.129574	0.952321762	NA
ASV_16s_7662	0.57742626	0.343042048	3.549597945	2.122236981	NA	NA
ASV_16s_7663	0.626794278	0.343916985	3.549715652	2.033874459	NA	NA
ASV_16s_7664	0.756716053	0.34260001	3.549470921	2.462976312	NA	NA
ASV_16s_7665	0.575531714	0.346417943	3.550578174	1.182649033	0.991320928	NA
ASV_16s_7666	0.681403518	0.683507658	3.550728041	2.233472384	NA	NA
ASV_16s_7667	0.620265738	0.315939284	3.550703132	1.514257962	0.981811941	NA
ASV_16s_7668	0.689307873	0.315939271	3.550691539	1.567925086	0.979864496	NA
ASV_16s_7669	0.551467358	0.315939344	3.550717566	1.454509984	0.983842871	NA
ASV_16s_7670	0.674219475	0.61705443	3.54941357	2.102898626	0.953926129	NA
ASV_16s_7671	0.350385913	0.734549139	3.550882635	1.199609226	NA	NA
ASV_16s_7672	0.648692678	0.693787985	3.550666752	1.4715602	0.983277943	NA
ASV_16s_7673	0.583528569	0.693744448	3.550679516	1.417745857	0.985021515	NA
ASV_16s_7674	0.467792208	-0.125343945	3.549662426	1.92924346	NA	NA
ASV_16s_7675	0.480918075	-0.221822713	3.549599897	1.981805518	0.960840517	NA
ASV_16s_7676	0.510193482	-0.293393446	3.54954194	2.004504413	0.959590639	NA

ASV_16s_7677	0.463169361	-0.331024692	3.549608095	1.875979771	NA	NA
ASV_16s_7678	0.492757781	-0.386272262	3.54956038	1.860438039	0.967159289	NA
ASV_16s_7679	0.503734552	-0.397748128	3.549544935	1.862096234	0.967077101	NA
ASV_16s_7680	0.47021721	-0.682671457	3.549683254	0.970345364	NA	NA
ASV_16s_7681	0.467938277	-0.682650918	3.549687851	0.968781275	NA	NA
ASV_16s_7682	0.153712871	0.220074904	3.550764812	0.650290001	NA	NA
ASV_16s_7683	0.505546176	0.010947501	3.549296087	1.978623651	0.96101401	NA
ASV_16s_7684	0.467450685	0.014898578	3.549283093	1.909947961	NA	NA
ASV_16s_7685	0.508844902	0.071157953	3.548927848	2.107867787	0.953629474	NA
ASV_16s_7686	0.49684776	0.086373725	3.548950621	2.113383487	0.953299005	NA
ASV_16s_7687	0.464431471	-0.037102681	3.548979115	1.903818376	NA	NA
ASV_16s_7688	0.530625823	0.051902136	3.549218376	1.820579189	0.969100295	NA
ASV_16s_7689	0.465357388	0.083986888	3.548917409	2.068625516	NA	NA
ASV_16s_7690	0.471116671	0.767605283	3.548652386	1.9366104	NA	NA
ASV_16s_7691	0.496301266	-0.45931054	3.549189788	1.697819864	0.974661473	NA
ASV_16s_7692	0.568295835	-0.585789465	3.549038535	1.522625578	0.98151601	NA
ASV_16s_7693	0.606025566	-1.58085441	3.548462207	1.328473655	0.987662048	NA
ASV_16s_7694	0.55570086	-0.792376434	3.54889226	1.713514769	NA	NA
ASV_16s_7695	0.571635716	-0.791683771	3.549000321	1.801453648	0.970008076	NA
ASV_16s_7696	0.568642721	-0.785472427	3.549229017	1.833045319	0.968500368	NA
ASV_16s_7697	0.633814567	-0.731168832	3.548192294	1.984504713	0.960693011	NA
ASV_16s_7698	0.566060543	-0.756052385	3.549457363	1.8386984	0.968226176	NA
ASV_16s_7699	0.591275936	-0.755916481	3.549458645	1.698612068	0.974627598	NA
ASV_16s_7700	0.575757733	-1.494424579	3.548812604	0.858421655	0.996801028	NA
ASV_16s_7701	0.58439772	-0.84868727	3.548980251	1.485723914	0.982799785	NA
ASV_16s_7702	0.569748388	-0.735344807	3.549545282	1.840772428	0.968125244	NA
ASV_16s_7703	0.529855828	-0.508036562	3.550580741	0.961163826	0.995430927	NA
ASV_16s_7704	0.572139534	0.606015846	3.549761906	1.767128252	NA	NA
ASV_16s_7705	0.673010589	-0.888256568	3.548775189	2.537187106	NA	NA
ASV_16s_7706	0.576184829	0.458845578	3.548903596	1.757032925	0.97205752	NA
ASV_16s_7707	0.554588094	0.354601101	3.551670258	0.547181993	NA	NA
ASV_16s_7708	0.560092635	-0.752348517	3.549834913	0.824742308	0.997183174	NA
ASV_16s_7709	0.530914024	1.338967803	3.549884454	0.967346316	NA	NA
ASV_16s_7710	0.437482259	0.419011861	3.550067144	0.861038672	NA	NA
ASV_16s_7711	0.234654095	1.276123521	3.550646772	0.634391853	NA	NA
ASV_16s_7712	0.45190967	-0.079229712	3.550635468	1.348860903	NA	NA
ASV_16s_7713	0.403947629	1.80670048	3.549636767	0.997420696	0.994870037	NA
ASV_16s_7714	0.597296028	1.085828134	3.549583677	1.397773952	NA	NA
ASV_16s_7715	0.797241138	-0.472926309	3.548371485	1.872247854	0.966571435	NA
ASV_16s_7716	0.317245876	0.283930496	3.551239377	0.85593573	NA	NA
ASV_16s_7717	0.683491856	-0.096655933	3.549833946	1.879658232	0.966199595	NA
ASV_16s_7718	0.702551472	0.128029007	3.550523704	1.761466766	0.971856656	NA
ASV_16s_7719	0.633136147	0.343715062	3.549645897	2.177996071	0.949335175	NA
ASV_16s_7720	0.761837746	1.065660299	3.549678837	1.72124879	0.973648621	NA
ASV_16s_7721	0.702486436	0.565459309	3.550721306	2.850882761	0.898445556	NA
ASV_16s_7722	0.659677624	0.31593931	3.55069622	1.545553974	0.980690546	NA
ASV_16s_7723	0.571768349	0.315939192	3.550712946	1.472873702	0.983233939	NA
ASV_16s_7724	0.658937296	0.315939362	3.550696343	1.544976588	0.980711595	NA
ASV_16s_7725	0.70108243	0.735082508	3.550729283	1.551825063	NA	NA
ASV_16s_7726	0.70108243	0.735082508	3.550729283	1.551825063	NA	NA
ASV_16s_7727	0.627698189	0.693775956	3.550670575	1.454842869	0.983831953	NA
ASV_16s_7728	0.606674876	0.693761687	3.550674668	1.437522832	0.984394156	NA
ASV_16s_7729	0.647261307	0.693788404	3.550667006	1.47041834	0.983316141	NA
ASV_16s_7730	0.453117494	-0.162910882	3.54966834	1.918228658	NA	NA
ASV_16s_7731	0.457720992	-0.21734935	3.549639189	1.934445965	NA	NA
ASV_16s_7732	0.484382435	-0.248160899	3.549588171	1.981290915	0.960868604	NA
ASV_16s_7733	0.480885255	-0.251932837	3.549592795	1.972684435	0.961336724	NA
ASV_16s_7734	0.463590565	-0.271877426	3.549617099	1.926954524	0.963772321	NA
ASV_16s_7735	0.457012685	-0.274135035	3.549627661	1.911807145	NA	NA
ASV_16s_7736	0.489972314	-0.306653934	3.549568657	1.954120026	0.962335977	NA
ASV_16s_7737	0.47938604	-0.300695765	3.549585746	1.938344403	0.963173839	NA
ASV_16s_7738	0.461616183	-0.330172664	3.549610786	1.873700458	0.966498727	NA
ASV_16s_7739	0.529534693	-0.369464419	3.549508864	1.953559875	NA	NA
ASV_16s_7740	0.512163125	-0.368242018	3.5495321	1.923759871	0.963939213	NA
ASV_16s_7741	0.505834305	-0.471527513	3.549552471	1.713642315	0.973979959	NA
ASV_16s_7742	0.462754532	-0.50123055	3.549627841	1.548760294	NA	NA
ASV_16s_7743	0.161884131	-0.572595712	3.550979885	0.619161185	NA	NA

ASV_16s_7744	0.161884131	-0.572595712	3.550979885	0.619161185	NA	NA
ASV_16s_7745	0.593240552	-0.683549287	3.549487154	1.045197671	NA	NA
ASV_16s_7746	0.293527876	0.220006718	3.550675181	0.970434349	NA	NA
ASV_16s_7748	0.502042012	-0.116755662	3.549469985	1.897475877	0.965296148	NA
ASV_16s_7749	0.491498976	0.004827707	3.549395803	2.037497553	0.957735815	NA
ASV_16s_7750	0.529915988	-0.654419277	3.549328644	1.751807028	0.972293213	NA
ASV_16s_7751	0.465319599	0.361138466	3.549243203	1.86109102	NA	NA
ASV_16s_7752	0.478347938	0.763542539	3.549055294	1.885667889	NA	NA
ASV_16s_7753	0.484780609	-0.429792225	3.550065523	1.230955699	NA	NA
ASV_16s_7754	0.597893435	-0.792769809	3.548786768	1.776555203	0.971166967	NA
ASV_16s_7755	0.619444727	-0.79279466	3.548762222	1.823087904	0.968980086	NA
ASV_16s_7756	0.619863994	0.62978051	3.547795595	1.998825347	0.959905354	NA
ASV_16s_7757	0.60422673	-0.792683087	3.532400326	1.872388638	0.966564392	NA
ASV_16s_7758	0.44830882	0.879172654	3.548252257	0.956533751	NA	NA
ASV_16s_7759	0.535787713	0.349541777	3.549318449	2.132777482	0.952127132	NA
ASV_16s_7760	0.590524388	-0.762083466	3.549372865	1.897569767	NA	NA
ASV_16s_7761	0.589939406	0.132980436	3.550149697	1.953108259	0.962390024	NA
ASV_16s_7762	0.587850649	-0.70377223	3.549637846	1.855174148	0.96741943	NA
ASV_16s_7763	0.597533837	0.178227335	3.548219196	1.904111627	0.964956292	NA
ASV_16s_7764	0.681697652	-0.254011854	3.549472688	1.905025796	0.964909328	NA
ASV_16s_7765	0.558240919	-0.67455595	3.550616199	0.766594863	0.997770107	NA
ASV_16s_7766	0.630720393	0.249661112	3.550156526	1.905096897	0.964905674	NA
ASV_16s_7767	0.546465944	0.21990293	3.548509508	2.286900367	0.942271639	NA
ASV_16s_7768	0.566010876	0.872248558	3.548745651	1.698099448	NA	NA
ASV_16s_7769	0.562875371	-0.535561846	3.549431896	1.246151541	0.98982516	NA
ASV_16s_7770	0.536046666	-0.087871989	3.54992284	1.526795375	0.981367479	NA
ASV_16s_7771	0.48353516	-0.677783154	3.549565426	1.670825961	NA	NA
ASV_16s_7772	0.458645204	0.550097561	3.548331006	1.353832816	NA	NA
ASV_16s_7773	0.544753512	1.756131459	3.549906872	0.783760191	NA	NA
ASV_16s_7774	0.396662061	1.969975849	3.549977133	0.563440018	NA	NA
ASV_16s_7775	0.605263631	-0.38638972	3.547751589	1.402951058	NA	NA
ASV_16s_7776	0.373952447	0.671921498	3.549517052	1.283622568	NA	NA
ASV_16s_7777	0.50847609	-0.024907418	3.54935539	2.142069313	0.951560226	NA
ASV_16s_7778	0.522611478	-0.534040672	3.549321745	1.704284824	NA	NA
ASV_16s_7779	0.364697509	1.336084107	3.550196769	0.783031424	NA	NA
ASV_16s_7780	0.163341511	0.376224924	3.551369521	0.539282444	NA	NA
ASV_16s_7781	0.322847148	0.219999823	3.550666216	1.018636685	0.994522272	NA
ASV_16s_7782	0.338866844	0.446400215	3.550649951	1.778537807	0.971075636	NA
ASV_16s_7783	0.456529922	1.741050519	3.550142616	0.773439194	NA	NA
ASV_16s_7784	0.476614825	-0.069255204	3.549683418	2.03030942	NA	NA
ASV_16s_7785	0.492406292	-0.059441419	3.550078278	1.49003693	NA	NA
ASV_16s_7786	0.676267623	0.23320194	3.550138011	2.750029648	0.907123932	NA
ASV_16s_7787	0.658362571	0.606266605	3.550670399	2.421131997	0.932917904	NA
ASV_16s_7788	0.666945198	0.315939171	3.550695034	1.551107634	0.980487384	NA
ASV_16s_7789	0.60470501	0.315939231	3.550706113	1.501294596	0.982264807	NA
ASV_16s_7790	0.52523606	0.315939376	3.550724065	1.429724004	NA	NA
ASV_16s_7791	0.646750632	0.64005857	3.550669551	2.144941136	NA	NA
ASV_16s_7792	0.485569398	-0.242932087	3.549587484	1.985606155	0.960632733	NA
ASV_16s_7793	0.483232533	-0.25343331	3.549588894	1.976714851	0.961117887	NA
ASV_16s_7794	0.449134841	-0.275605819	3.549641052	1.894135823	NA	NA
ASV_16s_7795	0.525268034	-0.380253769	3.54951451	1.929425341	0.963642952	NA
ASV_16s_7796	0.51492355	-0.382374611	3.549528469	1.907435429	0.96478537	NA
ASV_16s_7797	0.160425715	-0.569761418	3.550988693	0.616067614	NA	NA
ASV_16s_7798	0.47926984	0.132327594	3.549575122	1.928323216	0.963700689	NA
ASV_16s_7799	0.474913197	0.138288543	3.549144149	2.046920115	0.95719782	NA
ASV_16s_7800	0.485569655	0.42194981	3.54870351	2.089099696	NA	NA
ASV_16s_7801	0.500046178	-0.170041695	3.548772993	1.64879542	0.976707074	NA
ASV_16s_7802	0.476513831	-0.380390254	3.549329	1.657004439	NA	NA
ASV_16s_7803	0.488937093	-0.065948588	3.549919709	2.028770233	NA	NA
ASV_16s_7804	0.505211563	-0.419995612	3.549049959	1.847476824	0.967797747	NA
ASV_16s_7805	0.561720678	-1.274840037	3.548722831	1.367595478	0.986543174	NA
ASV_16s_7806	0.609241361	-0.664215521	3.549753039	1.861330512	0.967115068	NA
ASV_16s_7807	0.564692724	0.526742626	3.549025336	1.463876993	0.983533954	NA
ASV_16s_7808	0.544038547	1.962716726	3.549756391	0.696605077	NA	NA
ASV_16s_7809	0.503800344	-0.086149885	3.549938507	1.611397106	NA	NA
ASV_16s_7810	0.49018919	-0.07649466	3.550032176	1.703500392	0.974417996	NA
ASV_16s_7811	0.643713878	0.350469567	3.549733201	1.383487739	NA	NA

ASV_16s_7812	0.675713957	1.167056579	3.549787214	1.451616978	0.983937571	NA
ASV_16s_7813	0.489956136	18.34598788	3.551930948	-0.800445982	NA	NA
ASV_16s_7814	0.497514117	1.338654789	3.549957173	0.946399258	NA	NA
ASV_16s_7815	0.517459295	1.050439271	3.550037286	0.983312377	NA	NA
ASV_16s_7816	0.161869965	0.37556759	3.551377324	0.536303127	NA	NA
ASV_16s_7817	0.161869965	0.37556759	3.551377324	0.536303127	NA	NA
ASV_16s_7818	0.313845724	-0.224869467	3.549653444	1.119777981	NA	NA
ASV_16s_7819	0.540875214	0.974296858	3.54976613	1.129510338	NA	NA
ASV_16s_7820	0.546002106	0.125453791	3.548827339	1.470380456	NA	NA
ASV_16s_7821	0.286327753	0.306110738	3.551584061	0.45777068	NA	NA
ASV_16s_7822	0.442662413	0.738915125	3.551212249	0.787966409	NA	NA
ASV_16s_7823	0.475277745	-0.069300555	3.549691801	2.032119857	0.958041217	NA
ASV_16s_7824	0.521810928	1.554897332	3.54913234	0.711006529	NA	NA
ASV_16s_7825	0.370115469	0.174536026	3.550702924	1.333435605	NA	NA
ASV_16s_7826	0.821470155	0.302824909	3.547358125	1.742197574	NA	NA
ASV_16s_7827	0.553450633	0.342955582	3.549607118	2.099387988	NA	NA
ASV_16s_7828	0.63504789	0.577116853	3.550734897	2.713340876	NA	NA
ASV_16s_7829	0.599330182	0.315939213	3.550707179	1.496749213	0.982421988	NA
ASV_16s_7830	0.520504204	0.53829457	3.550704735	2.432625999	NA	NA
ASV_16s_7831	0.688563101	0.735071984	3.550732077	1.542643788	NA	NA
ASV_16s_7832	0.588151491	0.693748796	3.550678519	1.421744632	0.984895919	NA
ASV_16s_7833	0.444844169	-0.031041498	3.549775729	1.779944496	NA	NA
ASV_16s_7834	0.444924026	-0.129144257	3.549701351	1.883661382	NA	NA
ASV_16s_7835	0.455875006	-0.209622483	3.549644825	1.931169552	NA	NA
ASV_16s_7836	0.450810711	-0.205698093	3.549655037	1.920508019	NA	NA
ASV_16s_7837	0.469087217	-0.23795903	3.54961462	1.953700426	0.962358396	NA
ASV_16s_7838	0.464028845	-0.29816496	3.549610909	1.90911821	0.964698659	NA
ASV_16s_7839	0.530223861	-0.435202298	3.549512428	1.839087068	0.968207275	NA
ASV_16s_7840	0.158967299	-0.566901851	3.550997629	0.612957944	NA	NA
ASV_16s_7841	0.158967299	-0.566901851	3.550997629	0.612957944	NA	NA
ASV_16s_7842	0.158967299	-0.566901851	3.550997629	0.612957944	NA	NA
ASV_16s_7843	0.158967299	-0.566901851	3.550997629	0.612957944	NA	NA
ASV_16s_7844	0.61747795	-0.683682493	3.54945769	1.058089414	NA	NA
ASV_16s_7845	0.47154815	0.018240725	3.54950746	1.922074026	0.964027112	NA
ASV_16s_7846	0.48580937	-0.059689286	3.549454005	1.863651615	0.966999905	NA
ASV_16s_7847	0.484812031	0.388842099	3.549008161	1.737747087	0.972921674	NA
ASV_16s_7848	0.452318393	0.288034059	3.551540148	0.643669092	NA	NA
ASV_16s_7849	0.500464943	-0.360584053	3.549664302	1.450112269	NA	NA
ASV_16s_7850	0.575011451	-0.792854863	3.548779221	1.667993392	NA	NA
ASV_16s_7851	0.61270988	-0.792934888	3.548751955	1.796784592	0.970227369	NA
ASV_16s_7852	0.57703808	-0.792093891	3.548923266	1.789890941	0.970549479	NA
ASV_16s_7853	0.575459753	-0.783097164	3.549249429	1.846359639	0.967852449	NA
ASV_16s_7854	0.555175333	-0.749454033	3.549490757	1.822162492	0.969024459	NA
ASV_16s_7855	0.579924647	0.291440191	3.550114207	1.563508423	0.9800292	NA
ASV_16s_7856	0.479779184	-0.392134914	3.550331284	1.937913264	NA	NA
ASV_16s_7857	0.509178001	-0.853074851	3.549673745	1.209173328	NA	NA
ASV_16s_7858	0.652482767	0.684247095	3.550320293	1.23692088	NA	NA
ASV_16s_7859	0.468458998	0.878248513	3.548733814	1.041185768	NA	NA
ASV_16s_7860	0.532340808	0.79097827	3.550684533	0.860296751	0.996778812	NA
ASV_16s_7861	0.288971068	1.31784948	3.550466734	0.706138887	NA	NA
ASV_16s_7862	0.634904681	-0.550554991	3.549210422	0.973798471	NA	NA
ASV_16s_7863	0.307679142	0.220003169	3.550670642	0.994248487	0.994920784	NA
ASV_16s_7864	0.316156532	0.220001282	3.550668117	1.008012702	0.994698243	NA
ASV_16s_7865	0.344910686	0.596258373	3.550629604	0.982760458	NA	NA
ASV_16s_7866	0.625814742	-0.091787214	3.109067362	2.571746264	0.921598304	NA
ASV_16s_7867	0.560946543	-1.204361855	3.548589983	0.673807229	NA	NA
ASV_16s_7868	0.694467533	1.180436716	3.549939325	1.13942681	0.992255376	NA
ASV_16s_7869	0.133034195	0.281707307	3.551669491	0.545311093	NA	NA
ASV_16s_7870	0.619447748	1.351387259	3.549491014	1.826936055	0.968795185	NA
ASV_16s_7871	0.486968274	-0.029488267	3.550686263	1.446029281	NA	NA
ASV_16s_7872	0.610613672	0.343308465	3.549580919	2.206614396	0.947525248	NA
ASV_16s_7873	0.542887887	0.315939274	3.550719625	1.446501917	0.984104191	NA
ASV_16s_7874	0.599088539	1.08950057	3.550362422	1.177546734	0.991434723	NA
ASV_16s_7875	0.610503465	0.693763791	3.550673906	1.440677182	0.984292657	NA
ASV_16s_7876	0.60870841	0.693760934	3.550674263	1.439189164	0.984340587	NA
ASV_16s_7877	0.465177223	-0.229019523	3.549623316	1.947932158	0.962665858	NA
ASV_16s_7878	0.46581124	-0.299220172	3.549607793	1.911891159	0.964555516	NA

ASV_16s_7879	0.487047	-0.317668118	3.549571575	1.937751868	0.963205108	NA
ASV_16s_7880	0.46375611	-0.344984855	3.549606261	1.861000938	0.967131402	NA
ASV_16s_7881	0.47473502	-0.429449847	3.549593271	1.751611843	0.972301994	NA
ASV_16s_7882	0.441101088	-0.448628784	3.549656849	1.645000369	NA	NA
ASV_16s_7883	0.475885202	-0.032862081	3.54951811	1.849851175	0.967681314	NA
ASV_16s_7884	0.454194399	0.318440285	3.549603558	1.897796212	NA	NA
ASV_16s_7885	0.449207777	0.314582347	3.54960779	1.887127458	NA	NA
ASV_16s_7886	0.479898242	0.281557073	3.549163784	2.042952941	0.957424779	NA
ASV_16s_7887	0.519846551	-0.793196878	3.549199863	1.576214315	0.979553222	NA
ASV_16s_7888	0.450715832	-0.33928753	3.54891727	1.292372468	NA	NA
ASV_16s_7889	0.592139215	-0.793004149	3.548742114	1.702265766	0.974471028	NA
ASV_16s_7890	0.461190261	0.678714031	3.548665251	1.652631159	0.976550616	NA
ASV_16s_7891	0.489492536	0.839709902	3.549007383	1.271835168	NA	NA
ASV_16s_7892	0.587339962	-0.792083085	3.548914151	1.811144574	0.969550013	NA
ASV_16s_7893	0.584471515	-0.791916495	3.548950407	1.81583718	0.969326796	NA
ASV_16s_7894	0.485924897	0.289454542	3.550890996	0.793585344	0.997508867	NA
ASV_16s_7895	0.548727492	0.014768682	3.54961655	1.968943565	0.961539235	NA
ASV_16s_7896	0.700836944	0.290383835	3.548435277	1.686310367	0.975150678	NA
ASV_16s_7897	0.513221407	0.221784436	3.550235527	2.030538336	NA	NA
ASV_16s_7898	0.599049803	-0.118948412	3.550229026	2.354158186	0.937673078	NA
ASV_16s_7899	0.533119149	0.182306716	3.549453099	1.629729385	0.977475744	NA
ASV_16s_7900	0.7251121	2.994345809	3.547389405	0.723293866	NA	NA
ASV_16s_7901	0.262363526	0.354555073	3.551898566	0.437198691	NA	NA
ASV_16s_7902	0.549787344	0.39237163	3.551480246	0.59080493	NA	NA
ASV_16s_7903	0.494827192	0.967184319	3.549212091	2.045649162	0.957270602	NA
ASV_16s_7904	0.421857892	0.418993893	3.550109741	0.849314477	NA	NA
ASV_16s_7905	0.324415728	0.219999601	3.550665784	1.021068574	NA	NA
ASV_16s_7906	0.361042087	0.610293335	3.5505051	1.051291896	0.993958153	NA
ASV_16s_7907	0.519855876	1.105253445	3.112972228	2.207645396	0.947459426	NA
ASV_16s_7908	0.466927266	-0.06933152	3.549726289	2.008605243	0.959362552	NA
ASV_16s_7909	0.483580835	0.975062718	3.549161976	0.965943749	NA	NA
ASV_16s_7910	0.760934116	0.572943091	3.548428931	1.960742453	0.961981162	NA
ASV_16s_7911	0.664673576	0.746577898	3.549716517	1.806027878	0.969792354	NA
ASV_16s_7912	0.615731748	0.261574586	3.549929844	2.630995934	NA	NA
ASV_16s_7913	0.535683574	0.363314022	3.550308581	1.094477996	0.993157192	NA
ASV_16s_7914	0.62190066	0.334247572	3.550695302	1.306062326	0.988276425	NA
ASV_16s_7915	0.629304543	0.315939257	3.550701477	1.521534179	0.981554769	NA
ASV_16s_7916	0.556872089	0.406143431	3.55070579	2.372226758	0.936407383	NA
ASV_16s_7917	0.52438612	0.315939295	3.550724289	1.428873638	0.984670433	NA
ASV_16s_7918	0.65128079	0.771212532	3.550087506	1.932820734	0.963464756	NA
ASV_16s_7919	0.511040491	0.315939415	3.550727862	1.415774076	NA	NA
ASV_16s_7920	0.800210989	1.180962792	3.549778547	1.176126231	NA	NA
ASV_16s_7921	0.747533988	1.181373287	3.549689214	1.137474631	NA	NA
ASV_16s_7922	0.605541859	0.693760176	3.550674901	1.436528045	0.984426083	NA
ASV_16s_7923	0.603932072	0.693759108	3.550675228	1.435169795	0.984469612	NA
ASV_16s_7924	0.622015028	0.693773004	3.55067166	1.450156689	0.983985246	NA
ASV_16s_7925	0.45282953	-0.097121376	3.549707034	1.874517244	0.966457805	NA
ASV_16s_7926	0.436827528	-0.207597959	3.549680089	1.88992978	NA	NA
ASV_16s_7927	0.466173072	-0.248376123	3.549617104	1.943905747	0.962879653	NA
ASV_16s_7928	0.44140821	-0.237436787	3.549662773	1.894958429	NA	NA
ASV_16s_7929	0.447024568	-0.294441379	3.549640912	1.876289818	0.966368901	NA
ASV_16s_7930	0.445280585	-0.327927388	3.549639544	1.84189749	0.968070418	NA
ASV_16s_7931	0.446615107	-0.417880533	3.549640342	1.718545517	0.973766652	NA
ASV_16s_7932	0.525100575	-0.518606987	3.549532467	1.605907559	NA	NA
ASV_16s_7933	0.197587331	0.155505584	3.550697968	1.125017644	0.992552154	NA
ASV_16s_7934	0.201331883	0.181997752	3.549813472	0.947496377	0.995631618	NA
ASV_16s_7935	0.446866004	-0.237811777	3.549613383	1.681519274	NA	NA
ASV_16s_7936	0.124283875	0.178733241	3.550768545	0.517769126	NA	NA
ASV_16s_7937	0.464143241	-0.070795894	3.549325863	1.791296555	0.970483962	NA
ASV_16s_7938	0.56673119	-0.795564015	3.549452544	1.599301296	NA	NA
ASV_16s_7939	0.447130541	-0.339065284	3.548913649	1.307476041	NA	NA
ASV_16s_7940	0.451718623	-0.76668128	3.549436133	1.413928216	0.985140832	NA
ASV_16s_7941	0.584455613	-0.816789605	3.548897243	1.371799514	0.986419402	NA
ASV_16s_7942	0.534484597	0.055844896	3.548582405	2.348223958	0.938085981	NA
ASV_16s_7943	0.594362148	-0.799877105	3.548892095	1.551299041	0.98048036	NA
ASV_16s_7944	0.533426843	-0.850815812	3.549057124	1.446661457	NA	NA
ASV_16s_7945	0.553047363	-0.79117522	3.549138841	1.791031179	0.970496338	NA

ASV_16s_7946	0.505960818	-1.048054939	3.549122571	1.302334089	0.988376766	NA
ASV_16s_7947	0.52727382	-0.849954262	3.54918673	1.642425549	NA	NA
ASV_16s_7948	0.48795646	-0.297774625	3.550001179	1.523492072	NA	NA
ASV_16s_7949	0.503589815	-1.013795298	3.549309503	1.651627986	NA	NA
ASV_16s_7950	0.559478367	-0.653832187	3.549701322	1.425828822	0.984766985	NA
ASV_16s_7951	0.603472912	0.249706631	3.550692331	1.009895511	0.994667325	NA
ASV_16s_7952	0.471620773	-0.653698267	3.55029433	0.991769035	0.994960223	NA
ASV_16s_7953	0.214703487	-0.403074042	3.551156138	0.730034049	NA	NA
ASV_16s_7954	0.605900738	1.787202177	3.549059897	1.415926149	NA	NA
ASV_16s_7955	0.506308634	-0.079275498	3.550622466	1.406546279	NA	NA
ASV_16s_7956	0.302036896	0.220004544	3.550672403	0.984860263	0.995069078	NA
ASV_16s_7957	0.303510703	0.220004198	3.550671937	0.987324572	0.995030425	NA
ASV_16s_7958	0.410549903	-0.352928214	3.550270219	0.686206346	0.99843949	NA
ASV_16s_7959	0.394575905	0.665604837	3.54934697	1.546933253	NA	NA
ASV_16s_7960	0.582250861	0.717739759	3.549619512	2.116521113	0.95311046	NA
ASV_16s_7961	0.48339313	-0.069333154	3.549676512	2.046961767	0.957195434	NA
ASV_16s_7962	0.430248124	-0.122268111	3.549363382	1.582442827	NA	NA
ASV_16s_7963	0.578621281	0.665387208	3.549661739	1.70704014	NA	NA
ASV_16s_7964	0.579857218	1.717745632	3.549336129	1.107038231	NA	NA
ASV_16s_7965	0.216142285	0.279614871	3.551506145	0.717753225	NA	NA
ASV_16s_7966	0.216142285	0.279614871	3.551506145	0.717753225	NA	NA
ASV_16s_7967	0.573442508	-0.55032122	3.54926649	0.941012969	NA	NA
ASV_16s_7968	0.567615583	-0.116332781	3.549993895	1.832394857	0.968531832	NA
ASV_16s_7969	0.652491723	0.69802397	3.550736069	2.02981412	NA	NA
ASV_16s_7970	0.539122515	0.315939317	3.55072055	1.442946183	0.984219402	NA
ASV_16s_7971	0.519654264	0.315939316	3.550725536	1.424257955	0.984816654	NA
ASV_16s_7972	0.598310309	0.693754716	3.55067638	1.430425007	0.984621097	NA
ASV_16s_7973	0.574722914	0.693739083	3.550681468	1.409960176	0.985264239	NA
ASV_16s_7974	0.35547562	0.794285373	3.550081725	1.00099058	0.994812541	NA
ASV_16s_7975	0.439752359	-0.117152168	3.549718649	1.864005348	NA	NA
ASV_16s_7976	0.44279664	-0.127005891	3.549706701	1.877532518	NA	NA
ASV_16s_7977	0.443055559	-0.133945502	3.549702131	1.882493973	NA	NA
ASV_16s_7978	0.441903567	-0.264138144	3.549656331	1.885038396	0.96592819	NA
ASV_16s_7979	0.480840488	-0.301506737	3.549583414	1.940424503	0.963063956	NA
ASV_16s_7980	0.444256653	-0.355966453	3.549640068	1.807170047	0.969738352	NA
ASV_16s_7981	0.477844801	-0.379606324	3.549583195	1.842085439	0.968061254	NA
ASV_16s_7982	0.451474307	-0.461131108	3.549640564	1.635064107	NA	NA
ASV_16s_7983	0.154592053	-0.558171535	3.551025227	0.603504888	NA	NA
ASV_16s_7984	0.205737921	0.114368167	3.550664094	1.594729184	0.978847789	NA
ASV_16s_7986	0.294502798	0.596221156	3.550643237	0.902829258	0.996247539	NA
ASV_16s_7987	0.474945106	0.151973788	3.549067672	2.121976289	0.952781689	NA
ASV_16s_7988	0.470329734	0.49430964	3.549246585	1.941052286	0.963030758	NA
ASV_16s_7989	0.473344699	0.4765967	3.548699155	1.775850631	NA	NA
ASV_16s_7990	0.462856282	0.713161299	3.54961387	1.40119931	NA	NA
ASV_16s_7991	0.590832282	-1.447967315	3.54880329	1.126179703	0.99252849	NA
ASV_16s_7992	0.520858332	-0.810374196	3.549380944	1.367339305	NA	NA
ASV_16s_7993	0.514966247	0.645791961	3.547992608	1.922459567	NA	NA
ASV_16s_7994	0.57691387	-0.916504071	3.548789971	1.48232461	0.982915279	NA
ASV_16s_7995	0.535557589	-0.786302484	3.549241797	1.772750058	0.971341795	NA
ASV_16s_7996	0.544458523	-0.78031144	3.549295591	1.796690556	0.970231776	NA
ASV_16s_7997	0.550077199	-0.766278947	3.549392719	1.813094152	0.969457388	NA
ASV_16s_7998	0.572169925	-0.768689485	3.549364342	1.846806385	0.967830581	NA
ASV_16s_7999	0.556809383	1.293843233	3.547898439	1.389161079	0.985900939	NA
ASV_16s_8000	0.467592683	-0.116135807	3.550516675	1.575129395	NA	NA
ASV_16s_8001	0.488326401	0.102737872	3.549646632	1.964995135	0.96175235	NA
ASV_16s_8002	0.673402139	0.547414612	3.550703815	2.932715712	NA	NA
ASV_16s_8003	0.408862911	-0.569901244	3.549598447	0.979863533	NA	NA
ASV_16s_8004	0.509458505	0.132287539	3.549675558	1.818013347	0.969222968	NA
ASV_16s_8005	0.305451283	-0.365162387	3.549703349	1.124381937	NA	NA
ASV_16s_8006	0.429722808	0.579603521	3.549875304	1.359121466	NA	NA
ASV_16s_8007	0.19454969	0.3908764	3.551170703	0.596381068	NA	NA
ASV_16s_8008	0.155983785	0.372883363	3.551409734	0.524191282	NA	NA
ASV_16s_8009	0.260684028	0.124250021	3.549815068	1.608859437	NA	NA
ASV_16s_8010	0.353206841	0.942059525	3.550192221	1.133709908	NA	NA
ASV_16s_8011	0.299039045	0.220005291	3.550673365	0.979801568	0.995147817	NA
ASV_16s_8012	0.504340153	1.418751341	3.549454316	1.261725919	NA	NA
ASV_16s_8013	0.624774005	0.795397038	3.550579997	1.798443146	NA	NA

ASV_16s_8014	0.468332726	-0.068252561	3.549891932	1.839732668	NA	NA
ASV_16s_8015	0.479329665	-0.158323457	3.549405867	1.082175494	NA	NA
ASV_16s_8016	0.377153766	0.295585917	3.550990634	0.914416903	NA	NA
ASV_16s_8017	0.385113285	-0.256755858	3.549697314	1.18303012	NA	NA
ASV_16s_8018	0.617662493	0.599517912	3.548664183	1.828684314	NA	NA
ASV_16s_8019	0.592601169	0.277545951	3.550703712	2.723901336	NA	NA
ASV_16s_8020	0.354411355	0.342436982	3.549873664	1.698519121	NA	NA
ASV_16s_8021	0.480225166	0.360410304	3.550805594	1.423164677	NA	NA
ASV_16s_8022	0.599030429	0.315939201	3.550707243	1.496446712	0.982432419	NA
ASV_16s_8023	0.56818758	0.857572815	3.550653098	1.254213899	0.989624601	NA
ASV_16s_8024	0.437422425	-0.093954382	3.549738946	1.839412237	NA	NA
ASV_16s_8025	0.434728327	-0.199271731	3.549687086	1.88521289	NA	NA
ASV_16s_8026	0.463264507	-0.233881658	3.549625303	1.942596846	0.962949008	NA
ASV_16s_8027	0.428668285	-0.214368455	3.549693599	1.871194631	NA	NA
ASV_16s_8028	0.460520637	-0.316017197	3.549614195	1.885787503	NA	NA
ASV_16s_8029	0.45111327	-0.427781882	3.549633809	1.709573612	0.974156203	NA
ASV_16s_8030	0.153133637	-0.555210816	3.551034698	0.600311115	NA	NA
ASV_16s_8031	0.576834333	-0.683457559	3.549508577	1.036119334	0.994224649	NA
ASV_16s_8032	0.440353857	-0.682386927	3.549747339	0.949175343	NA	NA
ASV_16s_8033	0.136669141	-0.169771567	3.550701022	0.622413428	NA	NA
ASV_16s_8035	0.457832271	0.071857505	3.549354538	1.960150769	0.962012938	NA
ASV_16s_8036	0.571608631	-0.793687112	3.548965613	1.342869844	0.987257211	NA
ASV_16s_8037	0.539940616	-0.793130606	3.548872711	1.519595863	0.981623487	NA
ASV_16s_8038	0.447811122	-0.326957858	3.548878738	1.374396263	NA	NA
ASV_16s_8039	0.462920895	0.753329422	3.548753516	1.930080168	NA	NA
ASV_16s_8040	0.476730608	0.15102761	3.548798703	2.216562673	NA	NA
ASV_16s_8041	0.514645847	0.023362253	3.535951756	2.286896836	0.942271876	NA
ASV_16s_8042	0.535555413	-0.79156071	3.549048493	1.729199669	0.973299706	NA
ASV_16s_8043	0.677552589	-0.181496596	3.548250954	1.875254427	NA	NA
ASV_16s_8044	0.531177108	-0.872614801	3.549224221	1.722629252	0.97358823	NA
ASV_16s_8045	0.521643094	-0.344870898	3.549069162	1.829012908	NA	NA
ASV_16s_8046	0.498920405	-0.270772863	3.549358981	2.364350353	NA	NA
ASV_16s_8047	0.586554449	0.893993245	3.549037498	1.747662156	0.97247934	NA
ASV_16s_8048	0.528017765	-0.690646082	3.54970312	1.769480767	0.971491521	NA
ASV_16s_8049	0.464117498	-0.938756569	3.549428465	1.553174433	NA	NA
ASV_16s_8050	0.518836539	-0.101135818	3.550003697	1.517631656	NA	NA
ASV_16s_8051	0.552899157	0.262730933	3.549139581	1.726618056	0.973413286	NA
ASV_16s_8052	0.601030683	0.068758264	3.549375997	2.20535354	0.947605687	NA
ASV_16s_8053	0.483818055	-0.081370186	3.549980436	1.643451212	0.976924048	NA
ASV_16s_8054	0.564650889	-0.051206565	3.550617885	1.536494177	0.98101927	NA
ASV_16s_8055	0.591661809	0.343677023	3.550605151	1.163375979	0.991745856	NA
ASV_16s_8056	0.403117698	-0.078702908	3.550588784	1.533980507	NA	NA
ASV_16s_8057	0.624734774	-0.961113729	3.548890466	1.476396242	0.983115588	NA
ASV_16s_8058	0.423665316	-0.079203461	3.550643541	1.316038247	NA	NA
ASV_16s_8059	0.298946819	0.220005193	3.550673395	0.979636086	0.995150379	NA
ASV_16s_8060	0.656774202	0.072623593	3.548257835	1.807509719	NA	NA
ASV_16s_8061	0.442813828	2.053495986	3.549890001	0.584324877	NA	NA
ASV_16s_8062	0.362490056	1.363507723	3.550765764	0.785299346	NA	NA
ASV_16s_8063	0.347843324	-0.072839172	3.550271061	1.315839717	NA	NA
ASV_16s_8064	0.733989108	0.400255955	3.550055675	1.492394242	NA	NA
ASV_16s_8065	0.421045336	1.20058129	3.550366653	0.871500532	NA	NA
ASV_16s_8066	0.421045336	1.20058129	3.550366653	0.871500532	NA	NA
ASV_16s_8067	0.658052603	1.585996705	3.548905234	1.533544253	NA	NA
ASV_16s_8068	0.659650407	1.470762027	3.548995236	1.485759232	NA	NA
ASV_16s_8069	0.519726069	0.183107673	3.549438891	1.799377679	NA	NA
ASV_16s_8070	0.574654055	0.363422194	3.550030282	1.09289945	0.993187588	NA
ASV_16s_8071	0.506768595	0.315939353	3.550729048	1.411479861	0.98521705	NA
ASV_16s_8072	0.615328696	1.006267237	3.550428707	1.721510496	0.973637178	NA
ASV_16s_8073	0.661106696	1.18619734	3.549896434	1.12791873	0.992492989	NA
ASV_16s_8074	0.585781451	0.693741729	3.550679033	1.419632294	0.984962344	NA
ASV_16s_8075	0.651393097	0.693790782	3.550666284	1.473606148	0.983209371	NA
ASV_16s_8076	0.592132082	0.693751037	3.550677678	1.425103242	0.984789939	NA
ASV_16s_8077	0.437744423	-0.214912445	3.549675992	1.891301236	NA	NA
ASV_16s_8078	0.432950455	-0.231121257	3.549680342	1.877892357	NA	NA
ASV_16s_8079	0.434486856	-0.252317233	3.549672389	1.874302641	0.966468559	NA
ASV_16s_8080	0.444546846	-0.265902525	3.549651254	1.88974992	0.965689522	NA
ASV_16s_8081	0.451206448	-0.283590122	3.549635695	1.893085788	0.965519979	NA

ASV_16s_8082	0.466178121	-0.305971397	3.549606162	1.906698255	0.964823318	NA
ASV_16s_8083	0.453887121	-0.325844615	3.549624509	1.86209757	0.967077035	NA
ASV_16s_8084	0.461947171	-0.401395401	3.549610994	1.776699768	0.971160313	NA
ASV_16s_8085	0.151675221	-0.552222764	3.551044312	0.597094941	NA	NA
ASV_16s_8086	0.08156097	-0.169247099	3.550832006	0.404339037	NA	NA
ASV_16s_8087	0.14273338	0.22008588	3.550779271	0.615505183	NA	NA
ASV_16s_8088	0.425950107	-0.305343668	3.549754133	1.7160238	NA	NA
ASV_16s_8089	0.460259054	-0.079709656	3.549543538	1.859867035	0.967187564	NA
ASV_16s_8090	0.472487078	0.046496028	3.549518335	1.943548367	0.962898597	NA
ASV_16s_8091	0.444585726	0.153067904	3.549374871	1.935067899	0.963346558	NA
ASV_16s_8092	0.491082578	0.018036337	3.549280306	1.578538061	0.979465457	NA
ASV_16s_8093	0.474611259	0.317619942	3.5491333	1.800450299	0.970055277	NA
ASV_16s_8094	0.431322984	-0.174964514	3.54910038	1.522070094	NA	NA
ASV_16s_8095	0.483350051	0.538694255	3.549168797	1.811646772	0.969526168	NA
ASV_16s_8096	0.43437453	-0.339036643	3.548966174	1.277562475	NA	NA
ASV_16s_8097	0.469686954	0.240966672	3.548820855	2.215224767	NA	NA
ASV_16s_8098	0.463476541	0.577130582	3.548660809	1.795454515	NA	NA
ASV_16s_8099	0.462673995	-0.328656584	3.548659483	1.803439348	NA	NA
ASV_16s_8100	0.541205761	-0.812785763	3.549254112	1.174491942	0.991502404	NA
ASV_16s_8101	0.443375144	0.502064864	3.548451323	1.073930048	NA	NA
ASV_16s_8102	0.543088719	-0.792301201	3.548918493	1.687098045	NA	NA
ASV_16s_8103	0.555907447	0.593823149	3.548011975	2.033727835	0.957950024	NA
ASV_16s_8104	0.563664	-0.791831871	3.54897722	1.77597645	0.971193597	NA
ASV_16s_8105	0.42885261	1.367736893	3.548437745	1.36564799	0.986600276	NA
ASV_16s_8106	0.559174242	-0.90948361	3.548758308	1.448864607	0.984027358	NA
ASV_16s_8107	0.528437654	-1.126195688	3.54968386	0.914790888	NA	NA
ASV_16s_8108	0.555899192	2.765087831	3.547892248	0.287542738	NA	NA
ASV_16s_8109	0.462194503	0.911313449	3.549619272	1.329909149	NA	NA
ASV_16s_8110	0.547971767	-0.644814218	3.548093635	1.355880611	0.98688444	NA
ASV_16s_8111	0.51256144	0.219646706	3.548771108	2.083825327	0.955055371	NA
ASV_16s_8112	0.493892964	-0.281040271	3.550763896	1.063508757	NA	NA
ASV_16s_8113	0.454914226	-0.559579625	3.549594688	1.673696348	0.975680512	NA
ASV_16s_8114	0.674514402	0.866192784	3.549731008	1.778612503	NA	NA
ASV_16s_8115	0.468415053	2.125225497	3.549580001	0.554585571	NA	NA
ASV_16s_8116	0.460395314	0.403368393	3.550267851	0.946763827	NA	NA
ASV_16s_8117	0.807527303	0.40357178	3.549686847	1.127818989	NA	NA
ASV_16s_8118	0.695482195	-1.996826644	3.547837844	0.561387712	NA	NA
ASV_16s_8119	0.274861781	-0.547754396	3.549891793	0.705024687	NA	NA
ASV_16s_8120	0.217893088	1.259791537	3.55071501	0.608699453	NA	NA
ASV_16s_8121	0.31240033	0.220002185	3.550669222	1.001917217	NA	NA
ASV_16s_8122	0.572954196	0.693349653	3.551661886	0.647114401	0.998709912	NA
ASV_16s_8123	0.498753019	0.973329035	3.548845002	1.521233344	0.981565445	NA
ASV_16s_8124	0.56348994	0.686417955	3.548388318	1.332166816	NA	NA
ASV_16s_8125	0.427470307	-0.059022994	3.550308338	1.262406334	NA	NA
ASV_16s_8126	0.611176603	2.210448526	3.548547701	1.038869577	NA	NA
ASV_16s_8127	0.318071991	-0.563707889	3.550210468	1.227842892	0.990271628	NA
ASV_16s_8128	0.463620577	-0.05915777	3.549922063	1.483607412	NA	NA
ASV_16s_8129	0.135846644	-0.429205952	3.55139876	0.548684831	NA	NA
ASV_16s_8130	0.614187783	0.17805004	3.550589062	1.648202115	0.97673122	NA
ASV_16s_8131	0.521906228	1.057319921	3.550211882	1.553388057	0.980403598	NA
ASV_16s_8132	0.844367217	0.528431172	3.55066913	2.852457119	0.898307379	NA
ASV_16s_8133	0.696234161	0.893301806	3.549661881	1.7143596	0.973948817	NA
ASV_16s_8134	0.55226908	0.343433654	3.549675823	2.01509842	NA	NA
ASV_16s_8135	0.590385013	0.315939257	3.550708999	1.489039704	0.98268668	NA
ASV_16s_8136	0.659833012	1.184865413	3.54996474	1.137728254	0.992290741	NA
ASV_16s_8137	0.675385327	1.180923933	3.550039967	1.160953829	0.991798316	NA
ASV_16s_8138	0.651005114	0.735041208	3.550741106	1.51406574	NA	NA
ASV_16s_8139	0.616452268	0.693768562	3.550672735	1.445588562	0.984133833	NA
ASV_16s_8140	0.602755927	0.693757192	3.550675469	1.434157603	0.984502003	NA
ASV_16s_8141	0.431334532	-0.156203267	3.549712761	1.867957057	NA	NA
ASV_16s_8142	0.432370208	-0.183978014	3.549697737	1.878302941	NA	NA
ASV_16s_8143	0.446593645	-0.221716406	3.549657627	1.90965721	NA	NA
ASV_16s_8144	0.45582343	-0.286510566	3.549627071	1.900762995	0.965128025	NA
ASV_16s_8145	0.451952783	-0.420149425	3.549631028	1.724964973	0.973485868	NA
ASV_16s_8146	0.150216806	-0.549206371	3.551054075	0.593857267	NA	NA
ASV_16s_8147	0.130686969	-0.081431756	3.550706789	0.747563671	0.997942994	NA
ASV_16s_8149	0.443438273	-0.65328037	3.549532685	1.608299318	0.978321813	NA

ASV_16s_8150	0.217324891	0.585501893	3.550720308	1.011224437	0.994645433	NA
ASV_16s_8151	0.471302005	0.068652606	3.549196588	2.010897957	0.959234728	NA
ASV_16s_8152	0.478510344	0.330750412	3.549110877	2.22106274	NA	NA
ASV_16s_8153	0.430140405	0.68040628	3.549366306	1.88758119	NA	NA
ASV_16s_8154	0.526275248	-0.792618211	3.548902846	1.546774202	0.980646016	NA
ASV_16s_8155	0.456154604	1.004556164	3.54884873	1.774370669	0.97126741	NA
ASV_16s_8156	0.569382304	-0.792940301	3.548800681	1.674226696	0.975658368	NA
ASV_16s_8157	0.571835556	-0.791838678	3.548989618	1.798618212	0.970141357	NA
ASV_16s_8158	0.457072772	0.205246134	3.549270692	1.815102199	NA	NA
ASV_16s_8159	0.55460331	-0.780255274	3.549290179	1.814088623	0.969410079	NA
ASV_16s_8160	0.545538575	0.249106545	3.549372106	2.129775984	0.952309503	NA
ASV_16s_8161	0.551316126	0.182783408	3.549340593	1.914511173	NA	NA
ASV_16s_8162	0.51561977	-0.68471369	3.549728516	1.751317317	0.972315241	NA
ASV_16s_8163	0.610420943	2.040096631	3.549294724	0.613327348	0.99891672	NA
ASV_16s_8164	0.477971252	-0.10831038	3.550578372	1.307186634	NA	NA
ASV_16s_8165	0.560610446	0.940674448	3.549832834	1.444201037	0.9841788	NA
ASV_16s_8166	0.550239677	2.202716246	3.503705534	0.609593266	0.998938107	NA
ASV_16s_8167	0.651696474	1.435875762	3.549116324	1.449237875	0.984015199	NA
ASV_16s_8168	0.455968436	0.403363827	3.550280932	0.94364507	NA	NA
ASV_16s_8169	0.44395328	0.388169856	3.551371057	0.551255854	NA	NA
ASV_16s_8170	0.389843045	-0.29331877	3.549484563	1.179109575	NA	NA
ASV_16s_8171	0.151569149	0.370805637	3.551435401	0.514882416	NA	NA
ASV_16s_8172	0.151569149	0.370805637	3.551435401	0.514882416	NA	NA
ASV_16s_8173	0.299864723	0.22000501	3.5506731	0.981169706	0.995126602	NA
ASV_16s_8174	0.546879257	0.665446166	3.549564077	1.904465243	NA	NA
ASV_16s_8175	0.447308673	-0.068964229	3.549743321	1.930506688	NA	NA
ASV_16s_8176	0.450281783	-0.068784781	3.549780052	1.898060091	NA	NA
ASV_16s_8177	0.354268573	0.848019682	3.55043435	1.365413457	NA	NA
ASV_16s_8178	0.469941648	-0.068735426	3.549771136	1.922331672	0.964013686	NA
ASV_16s_8179	0.368253312	0.69376292	3.550099423	1.172001729	NA	NA
ASV_16s_8180	0.569898538	0.365770144	3.550796159	0.792741561	0.997517324	NA
ASV_16s_8181	0.461112481	0.107120093	3.550765477	1.419578739	NA	NA
ASV_16s_8182	0.786660848	-0.145556031	3.548286812	2.036617298	NA	NA
ASV_16s_8183	0.770361112	0.069069113	3.550616639	2.433656241	0.932009417	NA
ASV_16s_8184	0.541544216	0.328437893	3.550770484	1.028171712	0.994361196	NA
ASV_16s_8185	0.573450535	0.885771753	3.550140974	1.150700163	0.992018075	NA
ASV_16s_8186	0.763662561	-0.005540624	3.550080905	2.657035128	0.914815506	NA
ASV_16s_8187	0.638504794	0.343758012	3.549661075	2.169480594	0.949867313	NA
ASV_16s_8188	0.588006288	0.353716139	3.550000724	1.872675937	0.966550017	NA
ASV_16s_8189	0.581462333	0.344267428	3.549822705	1.925505517	NA	NA
ASV_16s_8190	0.637681493	0.792798187	3.548721845	1.601702397	0.978578454	NA
ASV_16s_8191	0.553710888	0.418793668	3.550669555	1.870271644	0.96667021	NA
ASV_16s_8192	0.629182303	0.700302387	3.55074217	1.9684866	NA	NA
ASV_16s_8193	0.535661219	0.315939233	3.550721414	1.43963079	0.984326371	NA
ASV_16s_8194	0.512108759	0.315939229	3.550727573	1.416783526	0.985051646	NA
ASV_16s_8195	0.503806599	0.315939385	3.550729882	1.408481658	0.985310062	NA
ASV_16s_8196	0.584926546	0.985251235	3.550568701	1.230355733	0.990211086	NA
ASV_16s_8197	0.679730476	1.72590638	3.549372161	0.816325085	NA	NA
ASV_16s_8198	0.423957415	-0.149111801	3.549731408	1.8482851	NA	NA
ASV_16s_8199	0.42751383	-0.166175098	3.549715414	1.863030227	NA	NA
ASV_16s_8200	0.434583618	-0.205747666	3.549685075	1.884808337	0.965939821	NA
ASV_16s_8201	0.4452953	-0.273017181	3.549648656	1.887197227	0.965818947	NA
ASV_16s_8202	0.445039343	-0.299998626	3.549643593	1.867407693	0.966813067	NA
ASV_16s_8203	0.440760484	-0.297328522	3.549651842	1.860372827	0.967162519	NA
ASV_16s_8204	0.439044801	-0.303190044	3.549654051	1.85182788	0.9675842	NA
ASV_16s_8205	0.428393259	-0.310711391	3.549673217	1.821989465	NA	NA
ASV_16s_8206	0.424134241	-0.367793805	3.549678823	1.748942194	0.972421936	NA
ASV_16s_8207	0.488081902	-0.405236097	3.549568972	1.820164768	0.969120127	NA
ASV_16s_8208	0.14875839	-0.546163425	3.551063985	0.590598442	NA	NA
ASV_16s_8209	0.14875839	-0.546163425	3.551063985	0.590598442	NA	NA
ASV_16s_8210	0.416703215	-0.682136297	3.549804513	0.931388623	NA	NA
ASV_16s_8211	0.416703215	-0.682136297	3.549804513	0.931388623	NA	NA
ASV_16s_8212	0.416703215	-0.682136297	3.549804513	0.931388623	NA	NA
ASV_16s_8213	0.430698729	0.198059583	3.548949649	2.104811083	NA	NA
ASV_16s_8214	0.432029676	0.692617663	3.548730295	1.802105212	0.969977402	NA
ASV_16s_8215	0.482840698	-0.515262867	3.548122858	1.399384438	0.985590111	NA
ASV_16s_8216	0.465574329	-1.295764775	3.54936809	1.18620125	0.991241148	NA

ASV_16s_8217	0.477153749	0.465416995	3.548938865	1.639563258	0.977081156	NA
ASV_16s_8218	0.531821871	-0.791847129	3.548993771	1.695639984	0.97475455	NA
ASV_16s_8219	0.539873511	-0.615115611	3.548987148	1.72904341	0.973306588	NA
ASV_16s_8220	0.567486654	1.109569191	3.547497832	1.034911231	NA	NA
ASV_16s_8221	0.505810999	-0.475904539	3.549391544	1.713242517	NA	NA
ASV_16s_8222	0.395397845	0.733094519	3.551332278	1.108490249	NA	NA
ASV_16s_8223	0.547270848	-0.773788842	3.549340955	1.805525024	NA	NA
ASV_16s_8224	0.52794273	-0.763667129	3.549423406	1.777315861	0.971131946	NA
ASV_16s_8225	0.565991032	-1.608347206	3.548491297	1.036611515	NA	NA
ASV_16s_8226	0.511761291	-1.11477551	2.941962881	0.886501956	0.996457734	NA
ASV_16s_8227	0.445874499	0.339530731	3.551131023	0.850240452	NA	NA
ASV_16s_8228	0.528659488	0.144599388	3.549522635	2.041823349	0.957489284	NA
ASV_16s_8229	0.519408874	0.283511074	3.549629772	1.72889075	0.973313312	NA
ASV_16s_8230	0.60105651	0.76484955	3.548789482	1.627846725	0.977550831	NA
ASV_16s_8231	0.489139193	-0.690951091	3.549501365	1.423326412	0.984846062	NA
ASV_16s_8232	0.517321067	-0.737867803	3.549601485	1.564928401	0.979976334	NA
ASV_16s_8233	0.461210333	-0.116127493	3.550519298	1.566283187	0.979925818	NA
ASV_16s_8234	0.560891265	0.063898819	3.550446059	1.566214231	0.979928391	NA
ASV_16s_8235	0.459998308	-0.254603707	3.548872286	1.787388075	NA	NA
ASV_16s_8236	0.488261118	-0.073471205	3.550395485	1.220429585	NA	NA
ASV_16s_8237	0.555584854	-0.219956876	3.55053339	1.583833543	0.979264626	NA
ASV_16s_8238	0.622965957	-0.586657086	3.548865892	2.060551333	0.956413034	NA
ASV_16s_8239	0.529393551	0.002493013	3.550645136	1.485765075	0.982798384	NA
ASV_16s_8240	0.644054579	1.339787973	3.549693935	1.029532299	0.994337968	NA
ASV_16s_8241	0.455162658	-0.774869544	3.549182179	1.259437532	NA	NA
ASV_16s_8242	0.358827489	0.686735645	3.550632629	0.830187587	NA	NA
ASV_16s_8243	0.499685679	0.97469423	3.550576677	0.829021971	NA	NA
ASV_16s_8244	0.527650129	-1.771276475	3.548515074	0.522583642	NA	NA
ASV_16s_8245	0.711428185	0.41920047	3.549623257	1.017484372	NA	NA
ASV_16s_8246	0.324993259	-0.107049016	3.549675512	1.523504403	NA	NA
ASV_16s_8247	0.150097604	0.370100041	3.551444233	0.511736973	NA	NA
ASV_16s_8248	0.150097604	0.370100041	3.551444233	0.511736973	NA	NA
ASV_16s_8249	0.258400876	0.177173281	3.549877663	1.564217197	NA	NA
ASV_16s_8250	0.29381174	0.220006536	3.550675092	0.970837445	0.995285351	NA
ASV_16s_8251	0.318931709	0.495144622	3.550647843	1.582226143	NA	NA
ASV_16s_8252	0.567654603	2.230657788	3.549149168	0.936756286	NA	NA
ASV_16s_8253	0.419984404	-0.069724696	3.550335968	1.684187213	NA	NA
ASV_16s_8254	0.673045178	-1.203962495	3.548470095	1.55681129	NA	NA
ASV_16s_8255	0.415317618	-0.916785344	3.549496679	1.348192068	0.987105517	NA
ASV_16s_8256	0.346325151	1.085298366	3.549542306	1.186476642	NA	NA
ASV_16s_8257	0.431876844	-0.06929538	3.549787925	1.926906605	NA	NA
ASV_16s_8258	0.495550514	-0.583824716	3.549908408	1.124465369	NA	NA
ASV_16s_8259	0.443516024	-0.069215856	3.54972787	1.959787097	0.962032461	NA
ASV_16s_8260	0.457269419	-0.069097157	3.54971908	1.964626783	0.961772199	NA
ASV_16s_8261	0.288476082	0.716057218	3.551018685	1.111617235	NA	NA
ASV_16s_8262	0.534845947	1.203958542	3.549740903	0.958709597	NA	NA
ASV_16s_8263	0.538671576	1.174508577	3.549934376	1.643557173	0.976919757	NA
ASV_16s_8264	0.646211696	0.287087695	3.548652243	1.858024305	0.967278719	NA
ASV_16s_8265	0.544944336	0.342525976	3.549602772	2.130791824	0.952247822	NA
ASV_16s_8266	0.589117768	0.351148896	3.54998628	1.845152661	NA	NA
ASV_16s_8267	0.609145111	0.522030272	3.550369809	1.540776651	0.980864302	NA
ASV_16s_8268	0.49770596	0.315939399	3.550731629	1.402281418	0.985501284	NA
ASV_16s_8269	0.494968395	0.3159394	3.550732426	1.399479096	0.985587214	NA
ASV_16s_8270	0.547824946	0.315939262	3.550718445	1.450994385	0.983957908	NA
ASV_16s_8271	0.748519898	1.186920229	3.549725551	1.158928976	0.99184201	NA
ASV_16s_8272	0.421476939	-0.249655399	3.549698439	1.846085189	0.96786588	NA
ASV_16s_8273	0.449226709	-0.275613536	3.549641023	1.894010108	0.965472919	NA
ASV_16s_8274	0.434131602	-0.279179953	3.549667868	1.858988357	0.967231048	NA
ASV_16s_8275	0.477535233	-0.305984049	3.549587927	1.929699489	0.963628582	NA
ASV_16s_8276	0.147299975	-0.543092378	3.551074048	0.587316719	NA	NA
ASV_16s_8277	0.419454689	-0.682164312	3.549797556	0.933497502	NA	NA
ASV_16s_8278	0.41261789	-0.682089404	3.549815055	0.928210727	NA	NA
ASV_16s_8279	0.116608378	-0.169638081	3.550734441	0.551071553	NA	NA
ASV_16s_8280	0.267083923	0.220014172	3.550684959	0.922604397	NA	NA
ASV_16s_8282	0.450486072	-0.249764306	3.549588911	1.524606943	0.98144552	NA
ASV_16s_8283	0.430472276	-0.018861927	3.549622419	1.851033247	0.967623259	NA
ASV_16s_8284	0.03751493	0.633443729	3.551962276	0.14007731	0.999992597	NA

ASV_16s_8285	0.425642197	0.05723161	3.549452953	1.844071203	0.96796434	NA
ASV_16s_8286	0.423632177	0.225653763	3.549339673	1.7018758	0.974487766	NA
ASV_16s_8287	0.421618518	-0.33901659	3.54902294	1.246839608	NA	NA
ASV_16s_8288	0.498918046	-0.792690863	3.549004423	1.430157874	0.984629599	NA
ASV_16s_8289	0.504947496	-0.595833551	3.548994806	1.607817803	0.978340606	NA
ASV_16s_8290	0.459734572	0.632293276	3.548853706	1.981865793	NA	NA
ASV_16s_8291	0.5198445	-0.792628619	3.54892316	1.553157546	0.980412077	NA
ASV_16s_8292	0.556294508	-0.792903802	3.548824135	1.630369108	NA	NA
ASV_16s_8293	0.560244055	-0.792438083	3.548877027	1.717554367	0.973809851	NA
ASV_16s_8294	0.527037027	-0.737863021	3.531357372	1.930082556	0.963608498	NA
ASV_16s_8295	0.546993728	-0.791735591	3.549006469	1.744148161	NA	NA
ASV_16s_8296	0.503878514	-0.277046862	3.548300887	1.890924552	NA	NA
ASV_16s_8297	0.462454368	0.191002545	3.548917319	1.800501269	NA	NA
ASV_16s_8298	0.538227137	-0.788822417	3.549211179	1.772829288	0.971338161	NA
ASV_16s_8299	0.500404055	1.595926339	3.342697083	1.421396357	0.984906883	NA
ASV_16s_8300	0.481358465	-0.90712595	3.549847679	1.053315156	0.993922036	NA
ASV_16s_8301	0.469178773	-0.89134173	3.549327831	1.398341768	NA	NA
ASV_16s_8302	0.854507379	-0.886440729	3.549522331	1.36290694	0.986680397	NA
ASV_16s_8303	0.452263648	-1.199294342	3.549409217	0.996914648	NA	NA
ASV_16s_8304	0.484563659	-0.917121036	3.549159437	1.450799866	NA	NA
ASV_16s_8305	0.334837227	-0.022710062	3.550387265	1.37356994	NA	NA
ASV_16s_8306	0.550485185	-0.601067189	3.549963546	1.159008652	0.991840293	NA
ASV_16s_8307	0.54515466	0.679200653	3.550712648	1.516746072	0.981724242	NA
ASV_16s_8308	0.461933622	1.269002674	3.55099728	0.485150211	0.999499059	NA
ASV_16s_8309	0.398406201	0.933492116	3.548975818	1.60138057	NA	NA
ASV_16s_8310	0.403613891	1.962107317	3.549842149	0.54702343	0.999255436	NA
ASV_16s_8311	0.474792881	1.519201677	3.549588112	1.236291384	NA	NA
ASV_16s_8312	0.583757504	0.481051926	3.548563562	2.13478698	NA	NA
ASV_16s_8313	0.306316291	-0.580971212	3.550409726	0.96181653	NA	NA
ASV_16s_8314	0.267762183	1.302996883	3.550530179	0.67983164	NA	NA
ASV_16s_8315	0.199893312	-0.072229599	3.550753873	0.886598741	NA	NA
ASV_16s_8316	0.239822982	-0.547045771	3.550066098	0.661754252	NA	NA
ASV_16s_8317	0.28970508	0.220007619	3.55067649	0.963703896	0.995392986	NA
ASV_16s_8318	0.421361635	0.418113644	3.551046123	0.703587803	NA	NA
ASV_16s_8319	0.464824526	-0.069056963	3.549712493	1.976064163	NA	NA
ASV_16s_8320	0.529574232	0.342797143	3.5507284	0.984926744	0.995068038	NA
ASV_16s_8321	0.660624942	0.360267368	3.548287998	1.561844807	0.980091032	NA
ASV_16s_8322	0.556067136	0.919341941	3.550473094	1.530714415	0.981227237	NA
ASV_16s_8323	0.569228372	0.919343016	3.55046992	1.544146551	0.980741832	NA
ASV_16s_8324	0.612513397	0.643052048	3.550730984	2.047515394	NA	NA
ASV_16s_8325	0.506454329	0.400828644	3.550693096	3.101645285	0.875442634	NA
ASV_16s_8326	0.457573036	0.360407047	3.550816073	1.398568877	NA	NA
ASV_16s_8327	0.527947262	0.693699206	3.550692896	1.366795754	0.98656664	NA
ASV_16s_8328	0.598828764	0.693756865	3.550676278	1.430816298	0.984608638	NA
ASV_16s_8329	0.665412847	0.693797674	3.550663887	1.484405986	0.982844618	NA
ASV_16s_8330	0.962658152	1.205674642	3.549324569	1.148104766	NA	NA
ASV_16s_8331	0.417542155	-0.121472685	3.549761401	1.817589896	NA	NA
ASV_16s_8332	0.411775255	-0.18623524	3.549738909	1.831658155	NA	NA
ASV_16s_8333	0.420131343	-0.193634654	3.549718304	1.851786135	NA	NA
ASV_16s_8334	0.421167018	-0.221913945	3.549706278	1.853051831	NA	NA
ASV_16s_8335	0.42747442	-0.288718009	3.549678683	1.83767996	0.968275672	NA
ASV_16s_8336	0.414495198	-0.294421453	3.549703789	1.804182869	NA	NA
ASV_16s_8337	0.454332666	-0.36115604	3.54962213	1.821101242	0.969075301	NA
ASV_16s_8338	0.408350034	-0.335569643	3.549711967	1.752550963	NA	NA
ASV_16s_8339	0.145841559	-0.539993554	3.551084267	0.584008651	NA	NA
ASV_16s_8340	0.408532564	-0.682040474	3.549825806	0.925001167	NA	NA
ASV_16s_8341	0.090472634	-0.169375252	3.550800078	0.444801218	NA	NA
ASV_16s_8342	0.264439528	0.220015004	3.550686045	0.917567943	NA	NA
ASV_16s_8343	0.181449053	0.220053042	3.550736093	0.729919181	NA	NA
ASV_16s_8344	0.429665577	-3.37E-06	3.549657347	1.843177305	0.968007986	NA
ASV_16s_8345	0.444872974	-0.190876656	3.54957858	1.804152123	0.969880921	NA
ASV_16s_8346	0.4246352	-0.410650636	3.549635439	1.577660193	0.979498639	NA
ASV_16s_8347	0.427709559	-0.220226284	3.549840726	1.516437333	NA	NA
ASV_16s_8348	0.534236047	-0.792843323	3.54887958	1.55949006	0.980178358	NA
ASV_16s_8349	0.522259194	-0.79254689	3.548923656	1.586437889	0.979165434	NA
ASV_16s_8350	0.543168708	-0.791371154	3.549075763	1.755901611	0.97210864	NA
ASV_16s_8351	0.540199182	-0.291408196	3.548433323	1.60224206	0.978557527	NA

ASV_16s_8352	0.458435392	-0.188548167	3.550033659	1.654863078	NA	NA
ASV_16s_8353	0.454670563	-0.331796002	3.548779066	1.915937118	NA	NA
ASV_16s_8354	0.591742902	-0.821701414	3.548848517	1.450674877	0.983968338	NA
ASV_16s_8355	0.539396807	0.030828537	3.549578759	1.97668399	0.961119565	NA
ASV_16s_8356	0.495115809	0.355661823	3.549724068	1.698536569	0.974630828	NA
ASV_16s_8357	0.441168567	-0.669347633	3.549698393	1.502234303	NA	NA
ASV_16s_8358	0.533751394	0.546165454	3.550714531	2.126481632	0.952509243	NA
ASV_16s_8359	0.452565224	-0.693559861	3.549544776	1.53562787	0.981050528	NA
ASV_16s_8360	0.522342336	-0.423553973	3.549421067	2.244067537	0.945106621	NA
ASV_16s_8361	0.531563612	-0.310931483	3.55047799	1.434149735	0.984502255	NA
ASV_16s_8362	0.454140457	1.8728422	3.549858266	0.62684177	NA	NA
ASV_16s_8363	0.370451394	-0.583267608	3.549882999	1.068117574	NA	NA
ASV_16s_8364	0.255624706	-0.566764787	3.550645137	0.843218304	NA	NA
ASV_16s_8365	0.199319494	0.392981017	3.551139061	0.604184808	NA	NA
ASV_16s_8366	0.300267911	-0.111802389	3.549982419	1.030280544	NA	NA
ASV_16s_8367	0.282465763	0.220009584	3.550679054	0.950891584	0.995582306	NA
ASV_16s_8368	0.282819218	0.220009487	3.550678926	0.951524131	0.995573079	NA
ASV_16s_8369	0.295238605	0.220006244	3.550674617	0.973270852	0.995248267	NA
ASV_16s_8370	0.522850618	-0.178893358	3.549317514	1.733000019	NA	NA
ASV_16s_8371	0.426332965	-0.069421458	3.550039934	1.817989808	0.969224093	NA
ASV_16s_8372	0.426177594	0.664709126	3.549709158	1.674454328	0.97564886	NA
ASV_16s_8373	0.433056711	-0.069260414	3.549767652	1.935716132	0.963312422	NA
ASV_16s_8374	0.483696764	1.265402452	3.549523467	1.551634911	NA	NA
ASV_16s_8375	0.495998501	-1.171390255	3.548457985	1.180074686	NA	NA
ASV_16s_8376	0.391531455	-0.131719359	3.549313579	1.476482369	NA	NA
ASV_16s_8377	0.378409932	0.518748609	3.550592048	1.441433939	NA	NA
ASV_16s_8378	0.513251299	0.328438186	3.550782368	1.001879901	0.994798154	NA
ASV_16s_8379	0.564583392	0.112682294	3.549615743	2.18941682	0.948616863	NA
ASV_16s_8380	0.729499765	0.670940234	3.549509024	2.281876119	0.942607969	NA
ASV_16s_8381	0.602016294	0.273949406	3.549584844	2.193148644	0.948381003	NA
ASV_16s_8382	0.502313921	0.235494445	3.550701253	2.466468198	NA	NA
ASV_16s_8383	0.506562959	0.359778521	3.550442635	1.085377022	0.993331264	NA
ASV_16s_8384	0.574033709	0.363384655	3.550107993	1.102118524	0.993008854	NA
ASV_16s_8385	0.563141597	0.361638197	3.550363372	1.126746343	0.992516934	NA
ASV_16s_8386	0.546798996	0.315939134	3.550718691	1.450026609	0.983989489	NA
ASV_16s_8387	0.493375181	0.315939305	3.550732895	1.397820453	0.985637928	NA
ASV_16s_8388	0.47318564	0.315939426	3.550739098	1.376595313	NA	NA
ASV_16s_8389	0.450632208	0.688424713	3.550701924	1.261702944	NA	NA
ASV_16s_8390	0.438400395	-0.208796674	3.549676856	1.892954934	NA	NA
ASV_16s_8391	0.409271963	-0.261692565	3.549721408	1.812542894	0.969483594	NA
ASV_16s_8392	0.490490538	-0.319467255	3.549566302	1.942423628	0.962958181	NA
ASV_16s_8393	0.431119034	-0.298165693	3.549669893	1.838542961	0.968233733	NA
ASV_16s_8394	0.408304736	-0.320203036	3.549713263	1.767911433	NA	NA
ASV_16s_8395	0.413138633	-0.353929531	3.549701279	1.742407148	0.972714289	NA
ASV_16s_8396	0.43489725	-0.404610703	3.549660702	1.717065648	0.973831137	NA
ASV_16s_8397	0.413172858	-0.402423807	3.549704605	1.675052091	0.975623882	NA
ASV_16s_8398	0.404447238	-0.681991454	3.549836773	0.9217595	NA	NA
ASV_16s_8399	0.114299301	-0.169619838	3.55073904	0.542293993	NA	NA
ASV_16s_8401	0.43209751	0.121694238	3.549486949	1.881747093	0.966094365	NA
ASV_16s_8402	0.414066802	0.075829149	3.54955648	1.820064413	0.969124928	NA
ASV_16s_8403	0.436943455	-0.641793435	3.549550381	1.605071185	0.978447621	NA
ASV_16s_8404	0.497875316	0.154793478	3.549047612	2.223165144	0.946463409	NA
ASV_16s_8405	0.512166054	-0.792984838	3.549102425	1.31136287	NA	NA
ASV_16s_8406	0.353476775	-0.266413861	3.550093303	1.365189081	NA	NA
ASV_16s_8407	0.426701651	0.603302944	3.549201272	1.875688523	0.966399074	NA
ASV_16s_8408	0.469051216	-0.006974443	3.548788207	2.096918771	NA	NA
ASV_16s_8409	0.421099233	1.334257595	3.549732406	1.132392501	NA	NA
ASV_16s_8410	0.547905662	-0.792179242	3.548930916	1.711770147	0.974061142	NA
ASV_16s_8411	0.522433511	-0.795400207	3.5491762	1.407856835	0.985329401	NA
ASV_16s_8412	0.530944338	-0.791385909	3.549082002	1.727745603	NA	NA
ASV_16s_8413	0.469567384	-1.258308237	3.549324888	0.735941908	0.998044077	NA
ASV_16s_8414	0.528910649	-0.791079678	3.549156055	1.741773423	0.972742545	NA
ASV_16s_8415	0.510715364	-0.787808814	3.549252659	1.721168559	0.973652129	NA
ASV_16s_8416	0.517486727	-0.879216095	3.549381244	1.725367388	NA	NA
ASV_16s_8417	0.52588315	-0.765444941	3.549411424	1.772917604	0.97133411	NA
ASV_16s_8418	0.531633761	-0.395017578	2.745100278	3.497618976	0.835477614	NA
ASV_16s_8419	0.498527249	-0.729443098	3.549594226	1.73166795	0.973190852	NA

ASV_16s_8420	0.468535155	-0.63796764	3.550614068	0.930791697	NA	NA
ASV_16s_8421	0.487202657	0.567523041	3.548613691	1.94836415	NA	NA
ASV_16s_8422	0.593247036	0.788669482	3.55046855	1.154547792	NA	NA
ASV_16s_8423	0.299174103	0.115537871	3.550648406	0.977378859	0.995185239	NA
ASV_16s_8424	0.9085237	0.271493329	3.54956613	1.162905377	NA	NA
ASV_16s_8425	0.507679488	0.690368401	3.550439052	0.733878367	NA	NA
ASV_16s_8426	0.470269911	1.144936988	3.550084541	1.046762962	0.994038504	NA
ASV_16s_8427	0.634162552	0.387729078	3.551466114	0.594060408	NA	NA
ASV_16s_8428	0.145682969	0.367941598	3.551471619	0.502174495	NA	NA
ASV_16s_8429	0.145682969	0.367941598	3.551471619	0.502174495	NA	NA
ASV_16s_8430	0.586995009	0.865405218	3.549186617	1.524974394	NA	NA
ASV_16s_8431	0.377773363	1.075670587	3.549951592	1.338206928	NA	NA
ASV_16s_8432	0.328766543	1.762918869	3.550105042	0.691591514	0.998399513	NA
ASV_16s_8433	0.424716303	-0.069417971	3.54996685	1.842714144	0.968030588	NA
ASV_16s_8434	0.431144536	2.084053793	3.54938372	0.736682511	0.998037736	NA
ASV_16s_8435	0.70742581	-1.994898829	3.548165344	0.63848915	NA	NA
ASV_16s_8436	0.406259144	1.322932906	3.550102063	1.005370319	NA	NA
ASV_16s_8437	0.427909145	0.394020591	3.550955227	0.801455157	NA	NA
ASV_16s_8438	0.533432218	-0.174394194	3.549276743	1.649690957	NA	NA
ASV_16s_8439	0.446948924	-0.315603656	3.549237904	2.225403102	NA	NA
ASV_16s_8440	0.575458221	-0.635569446	3.549240431	1.780942061	0.970964661	NA
ASV_16s_8441	0.335232492	-0.07908697	3.550677576	1.197272386	NA	NA
ASV_16s_8442	0.523516455	0.343352358	3.549687343	1.983372138	NA	NA
ASV_16s_8443	0.448512183	0.360405693	3.55082056	1.388388827	NA	NA
ASV_16s_8444	0.523034338	0.315939326	3.550724656	1.427435765	0.984716074	NA
ASV_16s_8445	0.306975269	0.734400485	3.550925879	1.132507055	NA	NA
ASV_16s_8446	0.615427418	0.693767281	3.550672941	1.444679359	0.984163307	NA
ASV_16s_8447	0.514133134	0.693686482	3.55069667	1.353307756	0.986958675	NA
ASV_16s_8448	0.410677506	-0.164098133	3.549751673	1.823690828	NA	NA
ASV_16s_8449	0.400048189	-0.16081261	3.549777121	1.797539695	NA	NA
ASV_16s_8450	0.426140114	-0.225826849	3.549695202	1.863495744	0.967007646	NA
ASV_16s_8451	0.400167974	-0.31492026	3.54973167	1.753962936	NA	NA
ASV_16s_8452	0.415417449	-0.332197258	3.549697265	1.771956787	0.971378166	NA
ASV_16s_8453	0.408310776	-0.375138473	3.549712302	1.704374719	0.974380402	NA
ASV_16s_8454	0.405277902	-0.381707156	3.549719439	1.688573936	NA	NA
ASV_16s_8455	0.142924728	-0.533704663	3.551105197	0.577311333	NA	NA
ASV_16s_8456	0.127557865	-0.169716658	3.550714911	0.590950315	NA	NA
ASV_16s_8457	0.418801994	0.186595968	3.549452137	1.875619094	NA	NA
ASV_16s_8458	0.430996153	0.156497156	3.549442202	1.777862522	0.971106762	NA
ASV_16s_8459	0.410264772	0.023142959	3.549155692	1.836404684	NA	NA
ASV_16s_8460	0.398715409	0.030431663	3.548780468	1.755546739	0.972124664	NA
ASV_16s_8461	0.428007004	0.730707018	3.549334527	1.662670784	0.976138219	NA
ASV_16s_8462	0.456319926	0.098205759	3.548839692	2.142417662	0.951538904	NA
ASV_16s_8463	0.483969058	-0.389200587	3.549882129	1.270584587	0.989209882	NA
ASV_16s_8464	0.485561498	-0.333125053	3.548795955	1.461601925	NA	NA
ASV_16s_8465	0.435818477	0.909811426	3.548968995	1.723784333	0.973537638	NA
ASV_16s_8466	0.504920603	-0.791992286	3.5490163	1.610698909	NA	NA
ASV_16s_8467	0.525947497	-0.79135344	3.549092257	1.718013622	NA	NA
ASV_16s_8468	0.457413052	-0.731507336	3.549552876	1.366982194	0.986561172	NA
ASV_16s_8469	0.50047605	-0.755651766	3.549493399	1.732173225	NA	NA
ASV_16s_8470	0.447642821	0.29804548	3.551571617	0.57622976	NA	NA
ASV_16s_8471	0.508917359	-0.760218039	3.549459863	1.745481009	0.972576998	NA
ASV_16s_8472	0.544817165	2.136146545	3.549194513	0.938503504	NA	NA
ASV_16s_8473	0.496566158	-0.199692127	3.549768673	1.65777326	0.976339916	NA
ASV_16s_8474	0.487796142	0.135445901	3.548725196	1.364462292	NA	NA
ASV_16s_8475	0.674889121	0.803008049	3.549690459	1.838935155	0.968214663	NA
ASV_16s_8476	0.450116745	1.596277403	3.539351753	0.583928849	0.999077469	NA
ASV_16s_8477	0.28717434	-0.093449722	3.550610551	0.982489072	0.995106087	NA
ASV_16s_8478	0.555830909	0.159241303	3.550674549	1.47155509	0.983278114	NA
ASV_16s_8479	0.719482549	0.243099562	3.54949762	2.857860918	NA	NA
ASV_16s_8480	0.42481873	-0.074682216	3.549647564	1.339147818	NA	NA
ASV_16s_8481	0.477464171	-0.080890854	3.550061537	1.240812972	NA	NA
ASV_16s_8482	0.503392301	0.325758873	3.550488611	0.687233274	NA	NA
ASV_16s_8483	0.516263987	-0.211095702	3.548189508	1.927875559	0.963724127	NA
ASV_16s_8484	0.40438266	-0.273879513	3.549450888	1.438418923	0.984365362	NA
ASV_16s_8485	0.799476446	0.419234477	3.549544863	1.055049191	NA	NA
ASV_16s_8486	0.205322333	1.245163595	3.550765029	0.588126536	NA	NA

ASV_16s_8487	0.294251266	0.220006502	3.550674947	0.971556087	0.995274419	NA
ASV_16s_8488	0.474757047	2.22551586	3.549490583	0.511724797	0.999402407	NA
ASV_16s_8489	0.328647288	0.651000061	3.550394203	1.00326287	0.99477573	NA
ASV_16s_8490	0.486703496	0.325464962	3.547770582	0.786519774	NA	NA
ASV_16s_8491	0.571438765	0.274941808	3.550440416	2.410474721	0.933686209	NA
ASV_16s_8492	0.51719764	0.452473011	3.550659746	2.151114294	NA	NA
ASV_16s_8493	0.32574901	0.97914132	3.550959259	0.928516132	NA	NA
ASV_16s_8494	0.506920778	1.220791073	3.550183878	0.933391491	NA	NA
ASV_16s_8495	0.197962093	0.279491651	3.551546469	0.685783643	NA	NA
ASV_16s_8496	0.569107179	0.338231695	3.550752546	0.976018566	0.995206169	NA
ASV_16s_8497	0.525209026	-0.550108817	3.549319707	0.912674816	NA	NA
ASV_16s_8498	0.484301574	1.100247431	3.550126476	1.531940258	NA	NA
ASV_16s_8499	0.392401438	-0.117711995	3.549696494	1.802292503	NA	NA
ASV_16s_8500	0.639996435	0.411059063	3.548595705	1.855936277	0.967381837	NA
ASV_16s_8501	0.595849918	0.924890692	3.549909156	1.462257668	0.983587609	NA
ASV_16s_8502	0.528300962	0.315939344	3.550723286	1.432550594	0.984553345	NA
ASV_16s_8503	0.550549689	0.315939077	3.5507178	1.453462114	0.983877211	NA
ASV_16s_8504	0.567526549	0.378574376	3.550705837	2.239026515	0.945435444	NA
ASV_16s_8505	0.531369214	0.525072587	3.550704185	2.480203578	NA	NA
ASV_16s_8506	0.441619564	0.688415469	3.550704931	1.251429703	NA	NA
ASV_16s_8507	0.613447127	0.73500564	3.550751237	1.48379211	NA	NA
ASV_16s_8508	0.613447127	0.73500564	3.550751237	1.48379211	NA	NA
ASV_16s_8509	0.553864017	1.184758965	3.550022698	1.062440334	0.993757433	NA
ASV_16s_8510	0.303874509	0.7343883	3.550929438	1.127363959	NA	NA
ASV_16s_8511	0.558238693	0.693724346	3.550685284	1.395087903	0.985721242	NA
ASV_16s_8512	0.526056652	0.693696087	3.550693404	1.364939525	0.986621013	NA
ASV_16s_8513	0.534745631	0.69370404	3.550691118	1.373235717	0.986376961	NA
ASV_16s_8514	0.402455183	-0.042149633	3.549859858	1.703563133	NA	NA
ASV_16s_8515	0.413394603	-0.215550098	3.549724421	1.835878665	0.968363109	NA
ASV_16s_8516	0.417451743	-0.225808982	3.549712777	1.843717542	NA	NA
ASV_16s_8517	0.412629474	-0.271242693	3.549712732	1.815152409	0.969359427	NA
ASV_16s_8518	0.413977503	-0.294052076	3.549704983	1.803172463	0.969927119	NA
ASV_16s_8519	0.396059833	-0.281441865	3.549746545	1.77009804	NA	NA
ASV_16s_8520	0.417625388	-0.348739978	3.549692008	1.758481298	0.971991995	NA
ASV_16s_8521	0.402947968	-0.355961695	3.549723371	1.716986249	0.973834594	NA
ASV_16s_8522	0.405739035	-0.365610161	3.549717445	1.711225439	0.974084734	NA
ASV_16s_8523	0.141466312	-0.530317738	3.551114918	0.573901763	NA	NA
ASV_16s_8524	0.51578174	-0.683023399	3.549599999	1.000012646	NA	NA
ASV_16s_8525	0.40969697	-0.682047797	3.549822782	0.925890299	NA	NA
ASV_16s_8526	0.076071289	-0.169154453	3.550855398	0.378149139	NA	NA
ASV_16s_8527	0.076071289	-0.169154453	3.550855398	0.378149139	NA	NA
ASV_16s_8528	0.133126325	0.22009695	3.550793872	0.583444013	NA	NA
ASV_16s_8529	0.474560704	0.077252494	3.548715734	2.161743291	NA	NA
ASV_16s_8530	0.407589156	0.148694569	3.548944859	2.010022325	NA	NA
ASV_16s_8531	0.566831117	-0.767033644	3.548953693	1.591803415	NA	NA
ASV_16s_8532	0.436288432	-0.540632937	3.549261003	1.380530375	0.98616015	NA
ASV_16s_8533	0.444917187	0.365471681	3.549262555	1.787439992	NA	NA
ASV_16s_8534	0.457291053	0.016821854	3.548804282	2.099392239	0.954134847	NA
ASV_16s_8535	0.508239611	-0.551144078	3.549105642	1.69992389	NA	NA
ASV_16s_8536	0.488835417	-0.791267509	3.549146483	1.630169702	0.977458161	NA
ASV_16s_8537	0.545614709	0.264751401	3.548973998	2.369004335	0.936634047	NA
ASV_16s_8538	0.496727783	-0.780081741	3.549353175	1.512518637	0.981873098	NA
ASV_16s_8539	0.562338712	-0.023799242	3.328410835	1.838775457	0.968222429	NA
ASV_16s_8540	0.536576837	0.17260816	3.336003525	2.053537635	0.95681779	NA
ASV_16s_8541	0.522451561	-0.829446719	3.549161849	1.532139848	0.981176073	NA
ASV_16s_8542	0.352265633	1.126438832	3.549087991	1.298793096	NA	NA
ASV_16s_8543	0.523516934	-0.76922142	3.549386325	1.766783329	0.971614722	NA
ASV_16s_8544	0.442864115	-0.269538532	3.549489948	1.59021453	NA	NA
ASV_16s_8545	0.516808413	0.51274657	3.548814344	1.865758975	0.966895152	NA
ASV_16s_8546	0.48538072	-0.746519355	3.549539662	1.70684044	0.974274209	NA
ASV_16s_8547	0.508334502	-0.803745543	3.549349531	1.48839577	0.98270868	NA
ASV_16s_8548	0.491977378	-0.764599021	3.549616844	1.764832746	0.971703622	NA
ASV_16s_8549	0.482108017	0.168816439	3.549853409	1.885814013	0.965888964	NA
ASV_16s_8550	0.501697092	0.220146574	3.548716065	2.188664601	NA	NA
ASV_16s_8551	0.485157101	-0.384019295	3.549019432	1.484064606	0.98285622	NA
ASV_16s_8552	0.495945965	0.338203839	3.551053639	0.875375224	NA	NA
ASV_16s_8553	0.544539183	0.183750208	3.550558324	1.479232014	0.983019949	NA

ASV_16s_8554	0.48845535	0.183782484	3.550573726	1.419325858	0.984971965	NA
ASV_16s_8555	0.424490107	-0.616560275	3.549629091	1.553787961	NA	NA
ASV_16s_8556	0.527120318	0.682949811	3.550707039	1.420272696	0.984942224	NA
ASV_16s_8557	0.421489947	-0.073091576	3.550041764	1.640864137	NA	NA
ASV_16s_8558	0.265396083	0.982099496	3.550496232	0.880240126	0.996536268	NA
ASV_16s_8559	0.438461587	0.12404372	3.55016761	1.483337888	0.982880901	NA
ASV_16s_8560	0.579225435	-0.56652595	3.549381708	1.014832881	NA	NA
ASV_16s_8561	0.373985766	-0.565021367	3.549763751	0.874064641	NA	NA
ASV_16s_8562	0.468209205	-0.567707269	3.548712703	1.167624572	NA	NA
ASV_16s_8563	0.142739879	0.366512731	3.551490342	0.495668219	NA	NA
ASV_16s_8564	0.142739879	0.366512731	3.551490342	0.495668219	NA	NA
ASV_16s_8565	0.256361469	-0.547408811	3.549978075	0.682831569	NA	NA
ASV_16s_8566	0.279295456	0.220010348	3.55068022	0.945151633	0.995665465	NA
ASV_16s_8567	0.313347154	0.5690264	3.550417038	1.110241217	NA	NA
ASV_16s_8568	0.422603708	-0.068792417	3.54979878	1.858273146	NA	NA
ASV_16s_8569	0.406710903	-0.512513319	3.549780249	1.211658713	NA	NA
ASV_16s_8570	0.5816158	1.112869636	3.550261031	1.293936132	0.988600848	NA
ASV_16s_8571	0.566315187	0.054406559	3.549619134	1.887614941	NA	NA
ASV_16s_8572	0.315883489	0.588411767	3.550871033	1.089878036	NA	NA
ASV_16s_8573	0.118388228	0.282859541	3.551699611	0.5066206	NA	NA
ASV_16s_8574	0.195942071	0.279477358	3.551551296	0.682077725	NA	NA
ASV_16s_8575	0.388965691	1.188547908	3.550442151	0.844500507	NA	NA
ASV_16s_8576	0.43904023	-0.498113409	3.549831946	1.015113888	NA	NA
ASV_16s_8577	0.681717428	0.985147441	3.549645968	1.643872761	0.976906976	NA
ASV_16s_8578	0.537798538	0.358185927	3.550138724	1.789326725	0.970575755	NA
ASV_16s_8579	0.407367884	-0.063275742	3.549831454	1.869564055	NA	NA
ASV_16s_8580	0.562047255	0.591711683	3.550024551	1.848210655	NA	NA
ASV_16s_8581	0.439451331	0.360404277	3.550825232	1.378001597	NA	NA
ASV_16s_8582	0.498826795	0.315939362	3.550731311	1.403360676	0.985468108	NA
ASV_16s_8583	0.485071644	0.315939363	3.550735388	1.389158769	0.985901009	NA
ASV_16s_8584	0.530610048	0.79241226	3.550211803	2.031724452	0.958063625	NA
ASV_16s_8585	0.593458003	1.141569384	3.550242932	1.139663629	0.992250438	NA
ASV_16s_8586	0.529023077	1.069212547	3.550437433	1.130339242	0.992443398	NA
ASV_16s_8587	0.593812071	1.021802043	3.550511831	1.214475673	0.99058977	NA
ASV_16s_8588	0.300773749	0.734375863	3.550933071	1.122170156	NA	NA
ASV_16s_8589	0.573074065	0.693736692	3.550681845	1.408433142	0.985311564	NA
ASV_16s_8590	0.064769869	0.678839466	3.551955164	0.225395065	0.999962149	NA
ASV_16s_8591	0.39192317	-0.211471144	3.54977359	1.785254868	NA	NA
ASV_16s_8592	0.391951691	-0.248403088	3.549762803	1.77703181	NA	NA
ASV_16s_8593	0.405682329	-0.333912813	3.549717897	1.748116074	NA	NA
ASV_16s_8594	0.140007897	-0.526730947	3.551123964	0.570453258	NA	NA
ASV_16s_8595	0.140007897	-0.526730947	3.551123964	0.570453258	NA	NA
ASV_16s_8596	0.526168563	-0.683100548	3.549582997	1.006424745	NA	NA
ASV_16s_8597	0.140726007	0.123065485	3.550739523	0.94677976	0.995641981	NA
ASV_16s_8598	0.131753889	0.220098675	3.550796132	0.578746183	NA	NA
ASV_16s_8599	0.4394963	-0.53384172	3.549640353	1.627155979	NA	NA
ASV_16s_8600	0.427298601	-0.028697788	3.54962101	2.282077316	0.94259452	NA
ASV_16s_8601	0.440998193	0.053834679	3.54914211	1.92638089	0.96380232	NA
ASV_16s_8602	0.433535867	0.307953493	3.549085195	2.00958378	NA	NA
ASV_16s_8603	0.43497087	0.104954462	3.549022003	1.975727527	0.961171557	NA
ASV_16s_8604	0.41055363	0.067624645	3.54921512	1.906960983	0.964809796	NA
ASV_16s_8605	0.400430743	0.06635347	3.549240352	1.879328169	NA	NA
ASV_16s_8606	0.468471693	0.279432198	3.549048529	1.651413743	0.976600339	NA
ASV_16s_8607	0.536700294	-0.793299687	3.548953854	1.395909867	0.985696212	NA
ASV_16s_8608	0.489390626	-0.209091061	3.54874783	1.703555216	0.97441564	NA
ASV_16s_8609	0.452625557	0.621129235	3.548789172	1.862360727	0.967063981	NA
ASV_16s_8610	0.13934117	-0.492539186	3.55087848	0.48598003	NA	NA
ASV_16s_8611	0.479626487	0.913168456	3.548355392	1.606338708	0.978398274	NA
ASV_16s_8612	0.41951587	0.300860112	3.549067664	2.051312246	NA	NA
ASV_16s_8613	0.501914087	-0.79142049	3.549105161	1.654084339	0.976491182	NA
ASV_16s_8614	0.50067046	-0.839516121	3.549176724	1.459413822	0.983681584	NA
ASV_16s_8615	0.519632087	-0.791604196	3.549054643	1.686618776	0.975137641	NA
ASV_16s_8616	0.492276138	-0.790820096	3.54921573	1.664812191	0.976049717	NA
ASV_16s_8617	0.508493031	-0.781877232	3.549305267	1.726574008	0.973415221	NA
ASV_16s_8618	0.540948276	-0.790554437	3.549174662	1.771695659	0.971390133	NA
ASV_16s_8619	0.490306856	-0.770455822	3.549401818	1.703239886	0.974429191	NA
ASV_16s_8620	0.464530989	0.161626956	3.549363559	2.033213607	0.957979199	NA

ASV_16s_8621	0.559459806	0.344769685	3.55011062	1.944606292	NA	NA
ASV_16s_8622	0.474368407	-0.095673118	3.550104622	1.37606977	0.986292975	NA
ASV_16s_8623	0.430864769	-0.374604052	3.54931607	1.425385139	0.984781024	NA
ASV_16s_8624	0.559625278	0.084295143	3.549604941	2.105495781	0.953771207	NA
ASV_16s_8625	0.435319177	-0.076385507	3.550137416	1.592574332	0.978930614	NA
ASV_16s_8626	0.4484799	-0.532411935	3.549565467	1.084858339	0.993341099	NA
ASV_16s_8627	0.438780276	-0.542035569	3.549457332	1.173623008	NA	NA
ASV_16s_8628	0.584294576	1.10926714	3.548724465	1.82452583	0.968911067	NA
ASV_16s_8629	0.593200568	1.339440121	3.549770805	1.002976055	NA	NA
ASV_16s_8630	0.408288475	0.260228761	3.549457992	1.678812807	0.975466398	NA
ASV_16s_8631	0.408658434	-0.069201632	3.549799417	1.880030815	NA	NA
ASV_16s_8632	0.421296167	-0.069222921	3.549771349	1.911197881	0.964591334	NA
ASV_16s_8633	0.413118099	-0.069096711	3.549773756	1.883464295	NA	NA
ASV_16s_8634	0.382578139	0.224335486	3.549794279	1.003903743	NA	NA
ASV_16s_8635	0.387648521	-0.255272627	3.549585052	1.46464915	0.983508332	NA
ASV_16s_8636	0.565373682	1.209141196	3.549909644	1.381554266	0.986129552	NA
ASV_16s_8637	0.341573222	0.28888833	3.55112905	0.880809885	NA	NA
ASV_16s_8638	0.235468854	0.279735794	3.551468684	0.74898881	NA	NA
ASV_16s_8639	0.543051227	0.249835503	3.550449024	1.543158688	0.980777781	NA
ASV_16s_8640	0.530773247	0.344055772	3.549815849	1.887216303	NA	NA
ASV_16s_8641	0.516993643	0.360837858	3.550411674	1.090974142	0.993224546	NA
ASV_16s_8642	0.434920905	0.360403545	3.550827641	1.372727621	NA	NA
ASV_16s_8643	0.504732421	0.315939211	3.550729632	1.409298645	0.985284752	NA
ASV_16s_8644	0.454258214	0.464594924	3.550729273	2.312267071	NA	NA
ASV_16s_8645	0.454258214	0.577180085	3.5507185	2.164433802	NA	NA
ASV_16s_8646	0.297672988	0.734362257	3.550936777	1.116923942	NA	NA
ASV_16s_8647	0.297672988	0.734362257	3.550936777	1.116923942	NA	NA
ASV_16s_8648	0.568575596	0.693733428	3.55068287	1.404417529	0.985435576	NA
ASV_16s_8649	0.523779057	0.693694709	3.550694019	1.36271415	0.986686021	NA
ASV_16s_8650	0.624681019	0.693774173	3.55067116	1.452225428	0.983917682	NA
ASV_16s_8651	0.409483518	-0.17705831	3.549748063	1.824328701	NA	NA
ASV_16s_8652	0.397966436	-0.21673942	3.549757842	1.799314295	NA	NA
ASV_16s_8653	0.416124334	-0.238632919	3.549712083	1.837375034	0.968290483	NA
ASV_16s_8654	0.393926407	-0.227893625	3.54976361	1.78778308	0.970647574	NA
ASV_16s_8655	0.39893373	-0.32185247	3.549733774	1.744539686	0.972619083	NA
ASV_16s_8656	0.391980546	-0.333199818	3.549748856	1.717093071	NA	NA
ASV_16s_8657	0.393258028	-0.350193313	3.549745395	1.701521888	0.97450295	NA
ASV_16s_8658	0.391997658	-0.357653382	3.549748409	1.689786942	NA	NA
ASV_16s_8659	0.407351122	-0.408033882	3.549718198	1.653500302	0.976515079	NA
ASV_16s_8660	0.395588298	-0.429026504	3.549749169	1.587814092	0.979112906	NA
ASV_16s_8661	0.470736532	-0.563507484	3.549591592	1.656078076	0.976409497	NA
ASV_16s_8662	0.422304684	-0.348666324	3.550180063	1.366400059	NA	NA
ASV_16s_8663	0.397606599	-0.33825417	3.549094833	1.241537248	NA	NA
ASV_16s_8664	0.398867133	0.247793802	3.54904691	1.974972279	NA	NA
ASV_16s_8665	0.446369262	-0.446419281	3.54925682	1.560060509	0.980157224	NA
ASV_16s_8666	0.558939573	-0.793306144	3.54882794	1.514892312	NA	NA
ASV_16s_8667	0.496469835	-0.792444063	3.548997544	1.509708613	0.981971644	NA
ASV_16s_8668	0.457153242	0.712442932	3.548596746	1.886349549	0.965861865	NA
ASV_16s_8669	0.330617554	0.595888772	3.549238194	1.492457486	NA	NA
ASV_16s_8670	0.415402939	1.376576685	3.548627635	1.422481135	NA	NA
ASV_16s_8671	0.473935749	-0.790849224	3.549235357	1.617533339	NA	NA
ASV_16s_8672	0.457783716	-1.265100872	3.549353267	0.727802564	0.998112875	NA
ASV_16s_8673	0.500405736	-0.78362557	3.549295477	1.706617522	0.97428382	NA
ASV_16s_8674	0.476565847	-0.75651368	3.549506138	1.686705237	0.975133985	NA
ASV_16s_8675	0.50701074	-0.756316225	3.549485903	1.743433762	0.97266848	NA
ASV_16s_8676	0.511847112	0.031986591	3.548940604	1.540196061	0.980885355	NA
ASV_16s_8677	0.571277755	-0.331908731	3.54891565	2.265337449	NA	NA
ASV_16s_8678	0.442667595	0.517700448	3.549128872	1.13211641	NA	NA
ASV_16s_8679	0.487876028	-0.068213912	3.550188987	1.578424356	0.979469757	NA
ASV_16s_8680	0.524689091	0.250575404	3.549024324	1.600908658	0.978609213	NA
ASV_16s_8681	0.483938463	-0.669658734	3.549157311	1.200844746	0.990907435	NA
ASV_16s_8682	0.503316912	0.065823906	3.55054022	2.187474691	0.948739387	NA
ASV_16s_8683	0.526212928	0.223142334	3.550655722	2.40488619	0.934087345	NA
ASV_16s_8684	0.391363255	0.304514241	3.550675776	1.958627788	NA	NA
ASV_16s_8685	0.338615568	1.070972197	3.54944655	1.187623246	NA	NA
ASV_16s_8686	0.477101077	1.904835274	3.549610111	0.876856329	NA	NA
ASV_16s_8687	0.252224974	1.655827345	3.550449841	0.450920386	NA	NA

ASV_16s_8688	0.634088708	2.339685305	3.549143383	0.608521138	NA	NA
ASV_16s_8689	0.622875571	-0.270165979	3.547510563	1.310625076	NA	NA
ASV_16s_8690	0.606383673	-0.566672799	3.549350455	1.029573936	NA	NA
ASV_16s_8691	0.139796788	0.365411638	3.551507206	0.489032505	NA	NA
ASV_16s_8692	0.162392187	0.0891728	3.550804896	0.894997036	NA	NA
ASV_16s_8693	0.369717557	-0.011572029	3.549505106	1.467387019	0.983417291	NA
ASV_16s_8694	0.251075665	-0.547298074	3.550005039	0.676206677	NA	NA
ASV_16s_8695	0.307607189	0.120077408	3.549661726	1.379081059	NA	NA
ASV_16s_8696	0.199036955	1.237495826	3.550792028	0.577437669	NA	NA
ASV_16s_8697	0.199036955	1.237495826	3.550792028	0.577437669	NA	NA
ASV_16s_8698	0.268890331	0.220013597	3.550684236	0.925940819	0.995936415	NA
ASV_16s_8699	0.270657608	0.220013063	3.550683533	0.929250825	0.995890535	NA
ASV_16s_8700	0.285365686	0.220008882	3.550678016	0.956007723	NA	NA
ASV_16s_8701	0.285365686	0.220008882	3.550678016	0.956007723	NA	NA
ASV_16s_8702	0.298103178	0.224857915	3.550731486	0.766632423	NA	NA
ASV_16s_8703	0.355723607	0.448431403	3.549124245	1.293948705	NA	NA
ASV_16s_8704	0.465333771	0.630690044	3.550662089	1.022681339	0.99445431	NA
ASV_16s_8705	0.41489244	-0.069274946	3.549837654	1.879018401	0.966231791	NA
ASV_16s_8706	0.409083191	-0.069075742	3.549780865	1.872297288	NA	NA
ASV_16s_8707	0.396294818	0.370766333	3.551229481	0.858515224	NA	NA
ASV_16s_8708	0.233016053	0.279720894	3.551473191	0.745143622	NA	NA
ASV_16s_8709	0.38094578	1.185202123	3.550462564	0.837353622	NA	NA
ASV_16s_8710	0.44760549	-0.927705567	3.549748133	0.796771957	NA	NA
ASV_16s_8711	0.635881712	0.450171291	3.54848441	1.263859784	NA	NA
ASV_16s_8712	0.523550338	2.116090149	3.548714394	0.400805243	NA	NA
ASV_16s_8713	0.618964969	0.497199175	3.550703249	2.565548717	NA	NA
ASV_16s_8714	0.535491767	0.31593928	3.550721472	1.439315102	0.984336534	NA
ASV_16s_8715	0.463556378	0.315939397	3.550742252	1.366095674	0.986587163	NA
ASV_16s_8716	0.473061358	0.717009434	3.54987618	1.805993146	0.969793995	NA
ASV_16s_8717	0.428100597	0.688400806	3.550709679	1.235621413	NA	NA
ASV_16s_8718	0.497380916	0.693668968	3.550701528	1.336440065	0.987439011	NA
ASV_16s_8719	0.543044945	0.693711819	3.550689002	1.381041419	0.986144883	NA
ASV_16s_8720	0.550435138	0.693717015	3.55068717	1.387902711	0.985938914	NA
ASV_16s_8721	0.38771269	-0.086213407	3.54985722	1.71895093	NA	NA
ASV_16s_8722	0.388535079	-0.171329458	3.549798904	1.772839796	NA	NA
ASV_16s_8723	0.412296817	-0.263869969	3.549714716	1.818255074	0.969211423	NA
ASV_16s_8724	0.406114686	-0.288621473	3.549722779	1.788856075	0.970597663	NA
ASV_16s_8725	0.387883812	-0.314498264	3.549760092	1.725145494	0.973477948	NA
ASV_16s_8726	0.38383808	-0.31977434	3.549769265	1.710702727	NA	NA
ASV_16s_8727	0.408737879	-0.34353109	3.549710911	1.74452199	0.972619874	NA
ASV_16s_8728	0.417728189	-0.348784757	3.549691852	1.758559338	0.971988462	NA
ASV_16s_8729	0.396934564	-0.68189245	3.549857591	0.915682362	NA	NA
ASV_16s_8730	0.122351422	-0.169680282	3.550723773	0.572264826	NA	NA
ASV_16s_8731	0.122351422	-0.169680282	3.550723773	0.572264826	NA	NA
ASV_16s_8732	0.418499027	-0.133834061	3.549651656	1.743826605	0.972650939	NA
ASV_16s_8733	0.419832424	0.190728704	3.549253998	1.940589532	NA	NA
ASV_16s_8734	0.437590053	0.146377905	3.549238471	1.982807768	0.960785781	NA
ASV_16s_8735	0.048301774	0.751970852	3.551940456	0.194036867	0.99997732	NA
ASV_16s_8736	0.391404111	0.029807215	3.549306419	1.76490219	NA	NA
ASV_16s_8737	0.428847794	0.02401076	3.549157415	1.915917742	0.964347088	NA
ASV_16s_8738	0.477818339	-0.141850574	3.548798493	1.757137939	0.972052772	NA
ASV_16s_8739	0.532199354	-0.793232288	3.54894596	1.408353395	0.985314033	NA
ASV_16s_8740	0.42006484	-0.03304141	3.548892059	1.878269025	NA	NA
ASV_16s_8741	0.479876333	-0.791525819	3.549120298	1.582209455	NA	NA
ASV_16s_8742	0.476768506	-0.790757546	3.549236287	1.626476621	0.977605384	NA
ASV_16s_8743	0.497087873	-0.622905662	3.549081008	1.655112013	0.976449098	NA
ASV_16s_8744	0.494024517	-0.791232142	3.549145806	1.646282709	0.976809237	NA
ASV_16s_8745	0.401208406	-0.23590004	3.549997868	1.409361535	NA	NA
ASV_16s_8746	0.542710835	-0.791720175	3.549013456	1.73501382	NA	NA
ASV_16s_8747	0.455830432	-1.265329179	3.549359037	0.726617191	NA	NA
ASV_16s_8748	0.483032617	-0.782999368	3.549317871	1.669862798	0.975840226	NA
ASV_16s_8749	0.495310378	-0.783825629	3.549298563	1.695307574	0.974768726	NA
ASV_16s_8750	0.435999039	-0.655513551	3.549329224	1.673732511	0.975679002	NA
ASV_16s_8751	0.500117082	-0.877179573	3.549083228	1.378905764	0.986208617	NA
ASV_16s_8752	0.474801875	-0.750212338	3.29986205	1.750591017	0.972347893	NA
ASV_16s_8753	0.507228511	-0.756622485	3.549484329	1.743650751	0.972658792	NA
ASV_16s_8754	0.410218498	0.422975789	3.548544483	1.114441938	NA	NA

ASV_16s_8755	0.477517169	-0.695282863	3.549711742	1.693578715	0.974842381	NA
ASV_16s_8756	0.497817767	2.161023917	3.549503659	0.568122896	0.999156846	NA
ASV_16s_8757	0.463752716	1.517005186	3.549628405	1.220145765	NA	NA
ASV_16s_8758	0.311183546	0.31048543	3.549994221	1.298120301	NA	NA
ASV_16s_8759	0.425858262	-0.633526615	3.549408769	1.164742235	NA	NA
ASV_16s_8760	0.635420768	1.339721851	3.549706223	1.025120331	NA	NA
ASV_16s_8761	0.330368584	0.063609032	3.54978427	1.479355575	NA	NA
ASV_16s_8762	0.413415439	-0.071373648	3.550109356	1.502750438	NA	NA
ASV_16s_8763	0.264125202	0.220014963	3.55068618	0.916896786	0.996060079	NA
ASV_16s_8764	0.266952846	0.220014082	3.550685018	0.922275851	0.995986827	NA
ASV_16s_8765	0.268366669	0.220013648	3.550684447	0.924944475	0.99595016	NA
ASV_16s_8766	0.271362271	0.220012704	3.550683255	0.930553374	0.995872389	NA
ASV_16s_8767	0.282361837	0.220009712	3.550679097	0.950643371	NA	NA
ASV_16s_8768	0.282361837	0.220009712	3.550679097	0.950643371	NA	NA
ASV_16s_8769	0.282361837	0.220009712	3.550679097	0.950643371	NA	NA
ASV_16s_8770	0.419907013	0.277361895	3.54893352	1.026006062	NA	NA
ASV_16s_8771	0.451413446	1.069943752	3.549713511	1.378765432	NA	NA
ASV_16s_8772	0.323970242	-0.387110132	3.550605184	0.962034056	NA	NA
ASV_16s_8773	0.531119182	1.957581916	3.549434255	0.788012527	NA	NA
ASV_16s_8774	0.451008982	0.370796703	3.551136009	0.917155175	NA	NA
ASV_16s_8775	0.426080288	-0.049549489	3.550430997	1.534251184	NA	NA
ASV_16s_8776	0.511170663	1.203758664	3.549784257	0.944056965	NA	NA
ASV_16s_8777	0.591248189	1.717526277	3.549877084	0.858059554	0.996805307	NA
ASV_16s_8778	0.114726737	0.283167644	3.55170796	0.49646112	NA	NA
ASV_16s_8779	0.376935825	1.183510528	3.550473066	0.833724346	NA	NA
ASV_16s_8780	0.524777718	0.17008175	3.550672873	1.529882196	0.98125707	NA
ASV_16s_8781	0.701123733	0.277264292	3.54892445	2.096594966	0.954300993	NA
ASV_16s_8782	0.545210488	0.344021183	3.549779296	1.990890468	0.960342837	NA
ASV_16s_8783	0.53182009	0.337366971	3.550709265	1.151661767	0.991997625	NA
ASV_16s_8784	0.425860053	0.360402031	3.550832611	1.362013315	NA	NA
ASV_16s_8785	0.51501296	0.315939219	3.550726799	1.419511671	0.984966131	NA
ASV_16s_8786	0.521460052	0.315939271	3.550725077	1.425829883	0.984766952	NA
ASV_16s_8787	0.520226753	0.315939256	3.550725405	1.424603231	0.984805745	NA
ASV_16s_8788	0.605384516	1.068939929	3.55065409	1.230464304	0.990208465	NA
ASV_16s_8789	0.550008105	1.047969745	3.550469789	1.161238162	0.991792168	NA
ASV_16s_8790	0.502215176	0.693674171	3.550700096	1.341338519	0.987300654	NA
ASV_16s_8791	0.322308696	1.273271435	3.550584512	0.705707989	0.998291494	NA
ASV_16s_8792	0.388851374	-0.29936612	3.5497599	1.739984001	0.972822239	NA
ASV_16s_8793	0.399270886	-0.329883164	3.54973242	1.73737861	0.972938034	NA
ASV_16s_8794	0.386088828	-0.32937272	3.549763075	1.706937918	0.974270006	NA
ASV_16s_8795	0.409287314	-0.417602953	3.549715633	1.6419463	0.976984934	NA
ASV_16s_8796	0.13563265	-0.515788664	3.551151793	0.559929573	NA	NA
ASV_16s_8797	0.13563265	-0.515788664	3.551151793	0.559929573	NA	NA
ASV_16s_8798	0.556662342	-0.683313434	3.549536753	1.024523674	0.994423175	NA
ASV_16s_8799	0.121049811	-0.169671076	3.550726107	0.567506406	NA	NA
ASV_16s_8800	0.13385681	0.005806127	3.550715994	1.008320199	0.994693201	NA
ASV_16s_8801	0.12627401	0.010382507	3.550727685	0.961121894	0.995431551	NA
ASV_16s_8802	0.408212203	0.042284937	3.549714611	1.763257263	0.97177531	NA
ASV_16s_8803	0.400445441	0.125122239	3.549557521	1.803681506	NA	NA
ASV_16s_8804	0.411049576	0.314599012	3.54958539	1.770460592	0.971446694	NA
ASV_16s_8805	0.390309182	-0.23041541	3.549270762	1.17758355	NA	NA
ASV_16s_8806	0.487282718	-0.792380054	3.54904989	1.400116196	NA	NA
ASV_16s_8807	0.509636214	-0.447860149	3.549039847	1.94489712	0.962827076	NA
ASV_16s_8808	0.419459105	-0.508435627	3.550097906	1.161567264	0.991785049	NA
ASV_16s_8809	0.424560885	1.018805516	3.54895981	1.625110093	0.977659717	NA
ASV_16s_8810	0.49553565	-0.79200328	3.549031544	1.578596998	0.979463228	NA
ASV_16s_8811	0.515701711	-0.792048458	3.548992455	1.636843159	0.9771907	NA
ASV_16s_8812	0.475388125	-0.787651152	3.549135484	1.574102931	0.979632775	NA
ASV_16s_8813	0.502771908	-0.790920918	3.549192494	1.686310674	0.975150665	NA
ASV_16s_8814	0.454544178	-0.773452832	3.549417283	1.621340397	NA	NA
ASV_16s_8815	0.502527426	-0.767295348	3.549413757	1.729461051	0.973288191	NA
ASV_16s_8816	0.466915538	1.28543766	3.548045465	0.831815911	NA	NA
ASV_16s_8817	0.435799873	-0.977406127	3.549323177	1.284668014	NA	NA
ASV_16s_8818	0.484450358	-0.600948628	3.550435009	0.860885325	NA	NA
ASV_16s_8819	0.431557562	-1.109566888	3.548327681	1.178209107	NA	NA
ASV_16s_8820	0.478352826	-0.116062376	3.550283291	1.311028728	NA	NA
ASV_16s_8821	0.589218893	-0.450784259	3.54868254	1.389448184	0.985892266	NA

ASV_16s_8822	0.45267511	-0.488600046	3.550267629	1.535988817	0.981037508	NA
ASV_16s_8823	0.619848179	0.727069072	3.550700698	1.708480792	0.974203424	NA
ASV_16s_8824	0.483425672	-0.376797985	3.55035535	1.273367871	0.989138372	NA
ASV_16s_8825	0.431986419	-1.713575456	3.548642018	0.527411443	NA	NA
ASV_16s_8826	0.504909216	-1.983986886	3.548028225	0.409856602	NA	NA
ASV_16s_8827	0.487240256	0.255493558	3.549103366	1.355869548	0.98688476	NA
ASV_16s_8828	0.533847604	1.209139418	3.54991755	1.427257946	0.984721713	NA
ASV_16s_8829	0.348284702	-0.165921858	3.550162136	0.023130275	NA	NA
ASV_16s_8830	0.489678899	0.666551494	3.549854346	1.783005507	NA	NA
ASV_16s_8831	0.61921657	0.414158378	3.551016982	0.612614719	NA	NA
ASV_16s_8832	0.363266518	0.418913041	3.550301788	0.801125758	NA	NA
ASV_16s_8833	0.136853698	0.36426108	3.551524421	0.482297369	NA	NA
ASV_16s_8834	0.291519076	-0.191247807	3.550518667	0.962686841	0.995408202	NA
ASV_16s_8835	0.261834262	0.220015714	3.550687141	0.912485506	0.996119501	NA
ASV_16s_8836	0.265368818	0.220014596	3.550685667	0.919257825	0.996028034	NA
ASV_16s_8837	0.294281594	0.596222258	3.550643316	0.902328606	NA	NA
ASV_16s_8838	0.384383298	-0.068568579	3.550527479	1.477634297	0.983073873	NA
ASV_16s_8839	0.349239853	-0.570511067	3.550177138	1.286667612	0.98879263	NA
ASV_16s_8840	0.415567123	1.924699687	3.54998416	0.809948906	NA	NA
ASV_16s_8841	0.314900919	-0.306144462	3.550690284	0.587199049	0.999060441	NA
ASV_16s_8842	0.397049229	-0.06915336	3.549809967	1.852541489	NA	NA
ASV_16s_8843	0.362146363	0.292817765	3.551046412	0.900487587	NA	NA
ASV_16s_8844	0.121478249	-0.397148449	3.551469967	0.512738887	NA	NA
ASV_16s_8845	0.372925869	1.181795118	3.550483772	0.830055176	NA	NA
ASV_16s_8846	0.411309228	-0.276924875	3.548938929	1.346194511	NA	NA
ASV_16s_8847	0.464804641	0.332214559	3.550745695	1.191720228	0.99111629	NA
ASV_16s_8848	0.497430359	0.320423607	3.550739323	1.287858652	0.988761341	NA
ASV_16s_8849	0.471968924	0.315939293	3.550739498	1.375203306	0.986318685	NA
ASV_16s_8850	0.440062645	0.315939428	3.550750513	1.339659085	NA	NA
ASV_16s_8851	0.56319137	0.693727078	3.55068412	1.399543845	0.985585232	NA
ASV_16s_8852	0.465077528	0.693633653	3.550711874	1.302324727	0.988377018	NA
ASV_16s_8853	0.375576164	-0.188692953	3.549822995	1.743965122	NA	NA
ASV_16s_8854	0.385756091	-0.266021119	3.549774018	1.753897939	0.972199048	NA
ASV_16s_8855	0.403847516	-0.294510922	3.549726659	1.779185032	0.971045785	NA
ASV_16s_8856	0.399449172	-0.299047055	3.549735607	1.765427106	0.97167655	NA
ASV_16s_8857	0.432690441	-0.342264292	3.549662319	1.79868657	0.970138148	NA
ASV_16s_8858	0.388362392	-0.37229498	3.549757741	1.662904784	NA	NA
ASV_16s_8859	0.135939049	0.099945057	3.550737798	0.962883866	0.995405257	NA
ASV_16s_8860	0.106217532	-0.169548559	3.550756705	0.510644398	NA	NA
ASV_16s_8862	0.394714209	0.091214358	3.549739227	1.766954068	0.971606932	NA
ASV_16s_8863	0.42049747	-0.060638791	3.549639021	1.761624439	0.971849498	NA
ASV_16s_8864	0.389823924	0.112775982	3.549687264	1.618164902	0.977934667	NA
ASV_16s_8865	0.415268378	0.400420347	3.549481636	1.941307383	NA	NA
ASV_16s_8866	0.394550041	0.384603871	3.54945427	1.603109077	0.97852388	NA
ASV_16s_8867	0.436431851	-0.29362152	3.549031336	1.279328277	0.98898425	NA
ASV_16s_8868	0.409398068	0.152226228	3.549582966	1.973296651	0.961303526	NA
ASV_16s_8869	0.389434032	-0.339326823	3.549235983	1.135199522	NA	NA
ASV_16s_8870	0.47202832	-0.79229148	3.549095064	1.404416401	NA	NA
ASV_16s_8871	0.387594114	0.316649891	3.549146259	1.910119031	NA	NA
ASV_16s_8872	0.491528129	-0.79200736	3.54903138	1.55346444	0.980400788	NA
ASV_16s_8873	0.511962767	-0.817482454	3.549470627	1.114765746	0.992758875	NA
ASV_16s_8874	0.459098436	-0.729005117	3.54928777	1.567907867	NA	NA
ASV_16s_8875	0.461702879	-0.79153961	3.549154374	1.521427785	NA	NA
ASV_16s_8876	0.424034404	-0.092797349	3.549285748	2.060556137	0.956412756	NA
ASV_16s_8877	0.459581671	-0.541740894	3.54940644	1.584552115	0.979237285	NA
ASV_16s_8878	0.39627562	0.669507777	3.548658367	0.941815368	NA	NA
ASV_16s_8879	0.47802216	-0.44166727	3.549024577	1.225468041	0.990328631	NA
ASV_16s_8880	0.445799579	-0.55502166	3.550087119	1.587679707	0.979118039	NA
ASV_16s_8881	0.425262314	-0.208097376	3.548864501	1.283029144	NA	NA
ASV_16s_8882	0.507899279	-0.64506562	3.549843606	0.42463568	0.999678336	NA
ASV_16s_8883	0.658325875	0.305694527	3.548496759	1.451959509	NA	NA
ASV_16s_8884	0.47764243	0.983590867	3.549390934	0.826717361	0.997161637	NA
ASV_16s_8885	0.416102716	1.33768256	3.550183072	0.888694454	NA	NA
ASV_16s_8886	0.481710486	2.162048391	3.549550753	0.561762424	0.999187441	NA
ASV_16s_8887	0.407272778	0.403307161	3.550443386	0.907190136	NA	NA
ASV_16s_8888	0.473838969	0.286181701	3.548466388	1.728266362	0.973340799	NA
ASV_16s_8889	0.315612077	0.400100392	3.550852404	0.824564637	NA	NA

ASV_16s_8890	0.31324562	0.217119492	3.549895007	1.385323265	NA	NA
ASV_16s_8891	0.285610978	0.224105151	3.550734791	0.679817867	0.998486046	NA
ASV_16s_8892	0.478966841	-0.954668784	3.548871672	1.119308481	NA	NA
ASV_16s_8893	0.243902186	1.284209717	3.55061216	0.647572319	NA	NA
ASV_16s_8894	0.435330788	0.419009233	3.5500732	0.859295074	NA	NA
ASV_16s_8895	0.277709183	-0.083903246	3.549835668	1.384708362	NA	NA
ASV_16s_8896	0.251725531	-0.547304571	3.550001961	0.676921272	0.998506847	NA
ASV_16s_8897	0.218452617	-0.544058213	3.550195319	0.6319253	NA	NA
ASV_16s_8898	0.425573508	-0.079202817	3.550642977	1.318175311	0.987946754	NA
ASV_16s_8899	0.262724423	0.220015451	3.550686767	0.914187404	0.996096645	NA
ASV_16s_8900	0.276354138	0.220011423	3.550681329	0.93974514	NA	NA
ASV_16s_8901	0.276354138	0.220011423	3.550681329	0.93974514	NA	NA
ASV_16s_8902	0.385594152	-0.39541393	3.550754814	0.43308179	0.999656501	NA
ASV_16s_8903	0.262397877	0.534143274	3.550588585	1.104081496	0.992970419	NA
ASV_16s_8904	0.49541009	-0.580548574	3.549998251	1.06682713	0.993677305	NA
ASV_16s_8905	0.391032248	-0.069183446	3.549837874	1.834952699	NA	NA
ASV_16s_8906	0.393014321	-0.069138217	3.549815689	1.842384364	NA	NA
ASV_16s_8907	0.39350984	-0.06912503	3.549811923	1.84321017	NA	NA
ASV_16s_8908	0.357913452	0.347672488	3.549518549	1.372049334	NA	NA
ASV_16s_8909	0.341453615	1.778166095	3.550335836	0.560847893	NA	NA
ASV_16s_8910	0.603414142	0.30682899	3.550749814	3.167958807	0.869042054	NA
ASV_16s_8911	0.498250924	0.36604623	3.550287851	1.656323978	0.976399411	NA
ASV_16s_8912	0.185841965	0.279403856	3.551576606	0.663030802	NA	NA
ASV_16s_8913	0.580442961	-0.525275924	3.549077888	2.577400885	0.921156811	NA
ASV_16s_8914	0.493053372	-0.549936211	3.549360946	0.892300997	NA	NA
ASV_16s_8915	0.41014724	-0.104475281	3.549918066	1.454984076	0.98382732	NA
ASV_16s_8916	0.684030365	0.864691124	3.549824114	2.194793599	0.948276858	NA
ASV_16s_8917	0.534460952	0.353652433	3.550016569	1.8148289	0.969374835	NA
ASV_16s_8918	0.557364364	0.238385095	3.550178185	2.622257256	0.917613139	NA
ASV_16s_8919	0.46315364	-0.060690609	3.549790568	1.974295514	NA	NA
ASV_16s_8920	0.469573504	0.343020989	3.549705471	1.929260659	NA	NA
ASV_16s_8921	0.508332905	0.344081488	3.549845315	1.849095624	NA	NA
ASV_16s_8922	0.565314747	0.363347376	3.550204384	1.107360428	0.992905918	NA
ASV_16s_8923	0.439703996	0.33912416	3.550733945	1.095535687	NA	NA
ASV_16s_8924	0.555138278	0.684249041	3.550761862	2.011349084	NA	NA
ASV_16s_8925	0.467452898	0.315939316	3.550740967	1.370305163	0.986463477	NA
ASV_16s_8926	0.524104854	0.315939316	3.550724385	1.428377904	0.984686178	NA
ASV_16s_8927	0.435330788	0.31593944	3.550752285	1.334157329	NA	NA
ASV_16s_8928	0.372268338	1.180991019	3.550310379	0.911306262	NA	NA
ASV_16s_8929	0.533312956	0.693703239	3.550691495	1.371828519	0.986418546	NA
ASV_16s_8930	0.516291303	0.693685984	3.550696075	1.355355576	0.98689961	NA
ASV_16s_8931	0.391250776	-0.188744121	3.549784302	1.782670385	0.970884736	NA
ASV_16s_8932	0.395858649	-0.214882465	3.549763381	1.794216939	0.970347576	NA
ASV_16s_8933	0.381670766	-0.26283276	3.549784516	1.745410112	0.972580169	NA
ASV_16s_8934	0.395935544	-0.28130851	3.549746983	1.769634921	0.971484471	NA
ASV_16s_8935	0.410210881	-0.344387998	3.549707795	1.746759396	0.972519784	NA
ASV_16s_8936	0.397882159	-0.336898649	3.549735205	1.726823393	0.973404262	NA
ASV_16s_8937	0.371599215	-0.336381957	3.549798954	1.66475558	NA	NA
ASV_16s_8938	0.403489384	-0.441815917	3.549734108	1.577540147	0.979503174	NA
ASV_16s_8939	0.371656256	-0.426819813	3.549808779	1.53420671	NA	NA
ASV_16s_8940	0.379252699	-0.430586915	3.549789851	1.545001917	0.980710672	NA
ASV_16s_8941	0.377733411	-0.42984344	3.549793578	1.542866049	NA	NA
ASV_16s_8943	0.3828594	-0.337442281	3.549823568	1.620983226	0.977823333	NA
ASV_16s_8944	0.396534919	-0.395664877	3.549513663	0.97562508	NA	NA
ASV_16s_8945	0.449643214	0.164805868	3.549374381	1.948455832	NA	NA
ASV_16s_8946	0.394085384	-0.008806829	3.549399924	1.699612584	NA	NA
ASV_16s_8947	0.436527335	-0.644403847	3.549460714	1.560349661	0.980146506	NA
ASV_16s_8948	0.495172825	-0.792668786	3.549019036	1.421804143	0.984894045	NA
ASV_16s_8949	0.318811212	1.333219167	3.550070923	0.520331711	NA	NA
ASV_16s_8950	0.540616532	-0.792374071	3.548862861	1.54740026	0.980623145	NA
ASV_16s_8951	0.466789602	-0.791874557	3.549110984	1.494384355	0.982503436	NA
ASV_16s_8952	0.429059554	-0.189833337	3.549781672	1.281937486	NA	NA
ASV_16s_8953	0.475096253	-0.791361695	3.549152634	1.583341987	0.979283316	NA
ASV_16s_8954	0.40817398	-0.196689966	3.550034401	1.271319088	0.989191039	NA
ASV_16s_8955	0.458234745	-0.745371687	3.549564105	1.654426909	0.976477158	NA
ASV_16s_8956	0.442500549	0.124121406	3.549562776	1.904893268	0.964916139	NA
ASV_16s_8957	0.359471855	-0.927657463	3.548968105	1.302284599	0.988378095	NA

ASV_16s_8958	0.458092388	-0.098170778	3.550504836	1.53457944	0.981088316	NA
ASV_16s_8959	0.545384108	-1.348776141	3.548777537	1.477982729	NA	NA
ASV_16s_8960	0.20658799	1.220832015	3.550805593	0.468525785	NA	NA
ASV_16s_8961	0.465230486	2.162069343	3.549172159	0.771314435	NA	NA
ASV_16s_8962	0.645233817	0.271537111	3.549834324	1.052516065	NA	NA
ASV_16s_8963	0.610869593	0.271545561	3.549886215	1.034887107	NA	NA
ASV_16s_8964	0.242767066	0.35327652	3.551931123	0.427136651	NA	NA
ASV_16s_8965	0.70658639	0.403532932	3.549797561	1.084661164	NA	NA
ASV_16s_8966	0.393698665	1.384957124	3.54853142	1.355609523	NA	NA
ASV_16s_8967	0.443240003	-1.62620742	3.54891325	0.497246262	NA	NA
ASV_16s_8968	0.133910608	0.362819815	3.551539931	0.475426991	NA	NA
ASV_16s_8969	0.133910608	0.362819815	3.551539931	0.475426991	NA	NA
ASV_16s_8970	0.367176607	-0.079135375	3.550663408	1.243297157	NA	NA
ASV_16s_8971	0.256994903	0.220017292	3.550689225	0.903046086	0.996244695	NA
ASV_16s_8972	0.273350289	0.220012231	3.550682482	0.934209303	NA	NA
ASV_16s_8973	0.272804428	0.220012392	3.550682694	0.933199167	NA	NA
ASV_16s_8974	0.287952958	0.596217131	3.550645368	0.891344919	NA	NA
ASV_16s_8975	0.585154149	-1.918065574	3.548098678	0.510994065	NA	NA
ASV_16s_8976	0.494104646	1.370389552	3.549225143	1.271622043	0.989183261	NA
ASV_16s_8977	0.473697433	0.428915915	3.549247584	1.502555681	NA	NA
ASV_16s_8978	0.604333255	2.439788191	3.096649087	0.595249118	0.999017623	NA
ASV_16s_8979	0.394430115	-0.068865908	3.549821425	1.815956943	NA	NA
ASV_16s_8980	0.342506117	0.691003152	3.550111739	1.542865871	NA	NA
ASV_16s_8981	0.303533221	0.662287057	3.551123034	1.11191711	NA	NA
ASV_16s_8982	0.487694096	-0.54989859	3.549368346	0.888776139	NA	NA
ASV_16s_8983	0.384761979	-0.127944208	3.54988523	1.733737953	0.973099368	NA
ASV_16s_8984	0.437730499	-0.102388495	3.550277791	1.585744254	0.979191879	NA
ASV_16s_8985	0.461183938	0.217301502	3.548525094	1.265394847	NA	NA
ASV_16s_8986	0.456641342	0.35413409	3.550542879	1.049398791	0.993991823	NA
ASV_16s_8987	0.485444658	0.31593933	3.550735286	1.389449999	0.985892211	NA
ASV_16s_8988	0.458517848	0.315939337	3.550743958	1.360492367	0.986750732	NA
ASV_16s_8989	0.549106496	1.183929123	3.55003995	1.060611524	0.993790646	NA
ASV_16s_8990	0.569629475	0.734958462	3.550764742	1.446042271	NA	NA
ASV_16s_8991	0.57193519	1.186187031	3.549898455	1.064010734	0.993728824	NA
ASV_16s_8992	0.499498947	0.693671919	3.550700902	1.33854718	0.987379609	NA
ASV_16s_8993	0.373471259	-0.171130255	3.549837017	1.735129752	NA	NA
ASV_16s_8994	0.373494075	-0.202031397	3.549822709	1.739533889	NA	NA
ASV_16s_8995	0.375019068	-0.211165986	3.549815277	1.743133271	NA	NA
ASV_16s_8996	0.367439737	-0.227567175	3.549829516	1.721613646	NA	NA
ASV_16s_8997	0.367439737	-0.227567175	3.549829516	1.721613646	NA	NA
ASV_16s_8998	0.389930068	-0.2845557	3.549760185	1.753077502	NA	NA
ASV_16s_8999	0.417831106	-0.326102565	3.549692884	1.783382022	0.970851791	NA
ASV_16s_9000	0.387799139	-0.314415362	3.549760395	1.724854099	0.973490733	NA
ASV_16s_9001	0.407756965	-0.327446324	3.549713966	1.759248408	0.971957256	NA
ASV_16s_9002	0.419322227	-0.349676227	3.549688639	1.760889627	0.971882849	NA
ASV_16s_9003	0.376376407	-0.339532874	3.54978662	1.673099523	0.975705417	NA
ASV_16s_9004	0.375528966	-0.681592445	3.549921205	0.897811815	NA	NA
ASV_16s_9005	0.06536521	-0.266995894	3.551724706	0.321706005	0.999873322	NA
ASV_16s_9006	0.142575544	0.231791765	3.550719888	1.251219399	0.989699375	NA
ASV_16s_9007	0.237995575	0.220024202	3.550698221	0.864376112	NA	NA
ASV_16s_9008	0.163304148	0.220066525	3.550753806	0.678870417	NA	NA
ASV_16s_9009	0.378842606	-0.113132723	3.549771122	1.685353743	0.975191091	NA
ASV_16s_9010	0.385738391	0.091924358	3.549806496	1.710395972	0.974120637	NA
ASV_16s_9011	0.396585299	-0.018866645	3.549702263	1.73872672	0.972878153	NA
ASV_16s_9012	0.38864268	0.234473789	3.549389017	1.821953312	NA	NA
ASV_16s_9013	0.415714473	0.203820345	3.548968338	2.057163896	0.956608774	NA
ASV_16s_9014	0.51665533	-0.576711008	3.549162764	1.657561279	NA	NA
ASV_16s_9015	0.419584346	0.570358888	3.549093537	1.793004164	NA	NA
ASV_16s_9016	0.556984011	2.183106358	3.548743795	0.483142428	0.999505896	NA
ASV_16s_9017	0.481466544	-0.792272052	3.549049471	1.470853274	0.983301598	NA
ASV_16s_9018	0.44955626	-0.782467595	3.54939604	1.263829634	NA	NA
ASV_16s_9019	0.386936542	-1.027519976	3.549436574	0.949477879	0.995602882	NA
ASV_16s_9020	0.375166116	-0.536394351	3.549451255	1.28911754	NA	NA
ASV_16s_9021	0.470314407	-0.790925573	3.549227925	1.602719865	0.978538988	NA
ASV_16s_9022	0.462451702	-0.787098732	3.54972896	1.180907062	0.991359884	NA
ASV_16s_9023	0.54244144	-0.87924516	3.549483898	1.251756774	0.989685981	NA
ASV_16s_9024	0.476089209	-0.766031682	3.549442788	1.678074782	0.97549735	NA

ASV_16s_9025	0.46705748	-0.822149751	3.549336262	1.33600581	0.987451232	NA
ASV_16s_9026	0.517726268	-0.825567896	3.549157418	1.399662237	0.985581608	NA
ASV_16s_9027	0.46464113	-0.569129518	3.550234675	1.214236079	0.990595413	NA
ASV_16s_9028	0.477035864	0.619254673	3.550855803	0.848466732	0.996917301	NA
ASV_16s_9029	0.418325412	1.578532603	3.549468591	0.543052897	NA	NA
ASV_16s_9030	0.409445822	-0.158531485	3.549243343	1.268781082	0.989256063	NA
ASV_16s_9031	0.311500272	0.109784742	3.332030507	0.954518342	0.995529235	NA
ASV_16s_9032	0.492853858	-0.626061563	3.549573341	0.96317118	NA	NA
ASV_16s_9033	0.489185462	0.371637651	3.551648652	0.547798694	NA	NA
ASV_16s_9034	0.390720664	1.490417226	3.549902035	1.089032466	NA	NA
ASV_16s_9035	0.510005272	0.378211225	3.551614346	0.564772287	NA	NA
ASV_16s_9036	0.351548244	0.418893751	3.550347805	0.790562574	NA	NA
ASV_16s_9037	0.188561326	1.223186731	3.550837024	0.558793402	NA	NA
ASV_16s_9038	0.436505504	0.31093059	3.550738008	1.312643404	0.988098009	NA
ASV_16s_9039	0.455167162	0.295041535	3.550727866	1.478021365	0.983060819	NA
ASV_16s_9040	0.400992475	0.295989056	3.549631966	2.016020383	0.958948353	NA
ASV_16s_9041	0.456701674	-0.073432249	3.550546164	1.403317243	NA	NA
ASV_16s_9042	0.354828436	-0.107390919	3.550985646	0.937518361	0.99577448	NA
ASV_16s_9043	0.414184118	-0.068930551	3.549788197	1.861919032	NA	NA
ASV_16s_9044	0.350930544	0.289162267	3.551762379	0.553163246	0.999227599	NA
ASV_16s_9045	0.422480475	-0.065128665	3.550143046	1.468393244	0.983383755	NA
ASV_16s_9046	0.46935104	0.313946858	3.550522312	1.050015119	0.993980875	NA
ASV_16s_9047	0.220752051	0.279644241	3.551496861	0.725306366	NA	NA
ASV_16s_9048	0.181801922	0.279373465	3.551587318	0.655152281	NA	NA
ASV_16s_9049	0.478414608	1.301722059	3.549784805	1.6898053	NA	NA
ASV_16s_9050	0.493977888	1.009871308	3.550323319	1.490954003	0.98262118	NA
ASV_16s_9051	0.507463641	-0.109099565	3.549393195	1.443053933	0.984215918	NA
ASV_16s_9052	0.6936587	0.21425043	3.550655381	2.815749541	0.901507688	NA
ASV_16s_9053	0.528180365	0.365540428	3.550218475	1.696833994	0.974703592	NA
ASV_16s_9054	0.335550592	-0.074063842	3.550693246	1.221869938	NA	NA
ASV_16s_9055	0.479836097	0.343488246	3.549758854	1.884443272	NA	NA
ASV_16s_9056	0.44627538	0.363433003	3.550272233	1.013999335	NA	NA
ASV_16s_9057	0.468163719	0.36083747	3.550449688	1.046539492	0.994042451	NA
ASV_16s_9058	0.407738349	0.360398742	3.550843213	1.339885619	NA	NA
ASV_16s_9059	0.407738349	0.360398742	3.550843213	1.339885619	NA	NA
ASV_16s_9060	0.438870509	0.315939359	3.550750959	1.338242782	0.987388201	NA
ASV_16s_9061	0.59862313	1.186286677	3.549875239	1.079899287	0.993434667	NA
ASV_16s_9062	0.56336981	0.734951264	3.550766842	1.440414101	NA	NA
ASV_16s_9063	0.485073246	0.693654934	3.550705315	1.323640419	0.987796174	NA
ASV_16s_9064	0.538612554	0.69370935	3.550690129	1.376840036	NA	NA
ASV_16s_9065	0.425867076	0.693583599	3.550726535	1.257598992	NA	NA
ASV_16s_9066	0.379187005	-0.176968029	3.54981938	1.750805735	0.972338242	NA
ASV_16s_9067	0.363320187	-0.176193696	3.549861726	1.710334169	NA	NA
ASV_16s_9068	0.394894835	-0.33501953	3.549742135	1.721822813	NA	NA
ASV_16s_9069	0.436825564	-0.359305791	3.549654165	1.78675763	0.970695229	NA
ASV_16s_9070	0.392237622	-0.37448372	3.549748771	1.668800442	0.975884379	NA
ASV_16s_9071	0.36905633	-0.425493847	3.549815472	1.530403334	NA	NA
ASV_16s_9072	0.129798988	-0.50079521	3.55119035	0.545439074	NA	NA
ASV_16s_9073	0.363593982	-0.681437287	3.549959871	0.887420851	NA	NA
ASV_16s_9074	0.122146835	0.220111733	3.550813363	0.544882729	NA	NA
ASV_16s_9075	0.386646819	-0.071247367	3.549724857	1.6459173	0.976824072	NA
ASV_16s_9076	0.383886936	-0.060694315	3.549752147	1.572053187	0.979709831	NA
ASV_16s_9077	0.39114872	0.038329328	3.54956659	1.736191528	0.9729907	NA
ASV_16s_9078	0.394943021	-0.26530354	3.549467073	1.281538659	NA	NA
ASV_16s_9079	0.400202258	0.113657376	3.54938705	1.834862672	0.968412366	NA
ASV_16s_9080	0.411432129	-0.078554737	3.549602526	0.997782958	0.994864221	NA
ASV_16s_9081	0.377645028	0.303712467	3.549554431	1.802825127	NA	NA
ASV_16s_9082	0.417011926	-0.440649199	3.549498802	0.780777117	NA	NA
ASV_16s_9083	0.466658809	-0.792308349	3.549132189	1.356410849	NA	NA
ASV_16s_9084	0.448542597	0.636278901	3.548801304	1.91482311	0.964403816	NA
ASV_16s_9085	0.490563817	-0.792243758	3.549022428	1.524112407	NA	NA
ASV_16s_9086	0.422748426	1.234893557	3.548871242	1.488135981	0.982717551	NA
ASV_16s_9087	0.483559698	-0.791691237	3.549092089	1.576692288	NA	NA
ASV_16s_9088	0.475235806	-0.79149723	3.549132196	1.570116029	0.979782496	NA
ASV_16s_9089	0.461128092	-0.781919553	3.549352478	1.620659754	0.977836128	NA
ASV_16s_9090	0.4589899	0.211143706	3.54988647	1.856392809	0.967359307	NA
ASV_16s_9091	0.442174775	0.858725067	3.550487572	1.364603074	NA	NA

ASV_16s_9092	0.444711917	-0.587027859	3.550005951	1.611470869	0.978197792	NA
ASV_16s_9093	0.427884283	-0.960101219	3.549402618	1.465457569	NA	NA
ASV_16s_9094	0.310753661	-0.492373033	3.550214554	0.818425012	0.997251342	NA
ASV_16s_9095	0.539027893	1.10981667	3.549631202	1.596177324	NA	NA
ASV_16s_9096	0.40275311	0.522368934	3.550229234	1.243699665	NA	NA
ASV_16s_9097	0.258162597	1.554523806	3.550591964	0.514272067	NA	NA
ASV_16s_9098	0.526366264	1.338926814	3.549894307	0.964361989	NA	NA
ASV_16s_9099	0.418824154	-0.21333431	3.5502174	1.107043545	NA	NA
ASV_16s_9100	0.438950884	-0.565654966	3.549604558	0.925519235	NA	NA
ASV_16s_9101	0.130967517	0.361471131	3.551555166	0.468474776	NA	NA
ASV_16s_9102	0.17198132	-0.070878967	3.550966285	0.758614545	NA	NA
ASV_16s_9103	0.211329162	-0.542936395	3.550243931	0.621413211	NA	NA
ASV_16s_9104	0.259674086	0.220016384	3.550688065	0.908254871	0.996175941	NA
ASV_16s_9105	0.26734259	0.22001407	3.550684865	0.922955277	NA	NA
ASV_16s_9106	0.377936488	-0.069167967	3.549868257	1.800282709	NA	NA
ASV_16s_9107	0.384378226	-0.068943457	3.549829288	1.803248957	NA	NA
ASV_16s_9108	0.410393724	-0.12500653	3.548922625	1.426997794	NA	NA
ASV_16s_9109	0.260349059	0.396393687	3.550834879	0.489425018	NA	NA
ASV_16s_9110	0.51498394	0.762014272	3.550845524	1.443539489	0.984200213	NA
ASV_16s_9111	0.305965765	-0.602331789	3.550673568	0.820674872	NA	NA
ASV_16s_9112	0.476975544	-0.549843498	3.549383643	0.881611893	NA	NA
ASV_16s_9113	0.458835521	0.744889776	3.550635544	1.376433503	0.986282173	NA
ASV_16s_9114	0.35723034	-0.554927268	3.549857402	0.891661143	NA	NA
ASV_16s_9115	0.543356808	0.869638012	3.549754401	1.690741662	0.974962979	NA
ASV_16s_9116	0.441658849	0.337719223	3.550740772	1.106458946	0.992923689	NA
ASV_16s_9117	0.486264741	0.649066555	3.550704876	1.76852487	0.971535215	NA
ASV_16s_9118	0.557110146	0.7349439	3.550768989	1.434723178	NA	NA
ASV_16s_9119	0.275967666	0.734265508	3.55096505	1.078666059	NA	NA
ASV_16s_9120	0.453763327	0.693618861	3.55071585	1.289771287	0.988710984	NA
ASV_16s_9121	0.453763327	0.693618861	3.55071585	1.289771287	0.988710984	NA
ASV_16s_9122	0.359006699	0.271656032	3.550565972	0.863716466	NA	NA
ASV_16s_9123	0.367292101	-0.116525312	3.549888014	1.693310117	NA	NA
ASV_16s_9124	0.366077364	-0.186946403	3.549848959	1.719326409	NA	NA
ASV_16s_9125	0.373499996	-0.23292599	3.549812129	1.735732541	0.973011047	NA
ASV_16s_9126	0.365943935	-0.358717569	3.549813999	1.62632675	0.977611346	NA
ASV_16s_9127	0.364182839	-0.375613237	3.549819933	1.601345864	NA	NA
ASV_16s_9128	0.377927038	-0.41099058	3.549788873	1.582157043	NA	NA
ASV_16s_9129	0.365742057	-0.443844772	3.549829805	1.483027203	NA	NA
ASV_16s_9130	0.128340572	-0.496949407	3.551200413	0.541755042	NA	NA
ASV_16s_9131	0.374954758	-0.681586795	3.549923059	0.897299654	NA	NA
ASV_16s_9132	0.367864744	-0.681485062	3.549945782	0.891161384	NA	NA
ASV_16s_9133	0.101599379	-0.169503558	3.550768056	0.491948263	NA	NA
ASV_16s_9134	0.121901466	-0.169677086	3.550724586	0.570567749	NA	NA
ASV_16s_9136	0.463110183	-0.609114118	3.549315057	1.479366888	0.983015392	NA
ASV_16s_9137	0.366343727	0.105726852	3.549258299	1.792227095	NA	NA
ASV_16s_9138	0.428227812	-0.892236712	3.549464661	1.347168576	0.987134774	NA
ASV_16s_9139	0.380791216	0.11955262	3.549058093	1.879784009	NA	NA
ASV_16s_9140	0.485537677	-0.792632489	3.549089437	1.349922566	0.987055958	NA
ASV_16s_9141	0.398031755	-0.338088872	3.549084029	1.259485589	NA	NA
ASV_16s_9142	0.410855032	0.433915491	3.550079047	1.20721665	0.990759786	NA
ASV_16s_9143	0.518556032	-0.433577978	3.548293427	1.797128018	0.97021127	NA
ASV_16s_9144	0.511692076	-0.365371342	3.548339076	1.721202982	0.973650624	NA
ASV_16s_9145	0.516839222	-0.339891945	3.548419244	0.944074923	NA	NA
ASV_16s_9146	0.4787711	-0.791031542	3.549198176	1.618424228	0.977924436	NA
ASV_16s_9147	0.422060647	-1.250994623	3.549475691	0.708075093	0.998272919	NA
ASV_16s_9148	0.42914448	-0.307983984	3.549792491	1.363545812	0.986661749	NA
ASV_16s_9149	0.38395577	1.345020269	3.550300023	1.133576606	NA	NA
ASV_16s_9150	0.432214675	1.245298003	3.548724933	1.326471392	0.987717721	NA
ASV_16s_9151	0.493273238	-0.739161155	3.549562199	1.721635183	0.973631726	NA
ASV_16s_9152	0.449506166	-0.671893093	3.549797476	1.642198211	0.976974748	NA
ASV_16s_9153	0.412182678	-0.918483819	3.549375016	1.327003012	NA	NA
ASV_16s_9154	0.399125969	-0.905686733	3.549930006	0.793460958	0.997510115	NA
ASV_16s_9155	0.43198703	1.384153076	3.548819466	0.682993366	0.998463022	NA
ASV_16s_9156	0.384735539	-0.874345998	3.54966857	1.462844642	0.983568172	NA
ASV_16s_9157	0.605824487	0.271546782	3.549894364	1.032180759	NA	NA
ASV_16s_9158	0.41090404	1.337607881	3.550200592	0.884605643	NA	NA
ASV_16s_9159	0.378590099	-0.074744705	3.549949376	1.349794226	NA	NA

ASV_16s_9160	0.322455345	0.230127396	3.549921932	1.380549612	NA	NA
ASV_16s_9161	0.233297743	1.274765665	3.550652366	0.632177736	NA	NA
ASV_16s_9162	0.129495972	0.360921204	3.55156208	0.464952228	NA	NA
ASV_16s_9163	0.129495972	0.360921204	3.55156208	0.464952228	NA	NA
ASV_16s_9164	0.184371074	1.216770936	3.550854306	0.551030291	NA	NA
ASV_16s_9165	0.250870984	0.22001943	3.55069198	0.890846296	0.996402574	NA
ASV_16s_9166	0.254669018	0.220018001	3.550690257	0.898421957	0.996305055	NA
ASV_16s_9167	0.390918907	-0.717902772	3.549945005	0.998728802	0.994849017	NA
ASV_16s_9168	0.506860609	1.425872086	3.548866666	1.306826483	0.988255793	NA
ASV_16s_9169	0.44158479	1.641226881	3.549165263	1.269927241	0.989226728	NA
ASV_16s_9170	0.379660611	-0.06921817	3.549893325	1.796248835	0.970252473	NA
ASV_16s_9171	0.378856763	-0.068987095	3.549835237	1.79717084	NA	NA
ASV_16s_9172	0.293953139	-0.415047501	3.551459127	0.588872949	NA	NA
ASV_16s_9173	0.295620431	1.291743698	3.550867911	0.773275461	NA	NA
ASV_16s_9174	0.302527947	-0.601961558	3.550693175	0.816979357	NA	NA
ASV_16s_9175	0.512379639	0.348982323	3.55086375	0.798121723	0.997463075	NA
ASV_16s_9176	0.531137758	0.630388767	3.55075293	1.964383751	NA	NA
ASV_16s_9177	0.446666033	0.343027267	3.549699841	1.932721642	0.963469964	NA
ASV_16s_9178	0.421868497	0.150996917	3.54984503	2.09152579	0.954601262	NA
ASV_16s_9179	0.567151048	0.819665157	3.54914015	1.304408857	NA	NA
ASV_16s_9180	0.553735501	0.978268333	3.549305423	2.218595254	0.946757701	NA
ASV_16s_9181	0.398677496	0.360397021	3.550848874	1.328451733	NA	NA
ASV_16s_9182	0.426394602	1.033915693	3.549612068	0.766577686	NA	NA
ASV_16s_9183	0.566777213	0.734954279	3.550765698	1.443447057	0.984203203	NA
ASV_16s_9184	0.550850481	0.734936364	3.550771186	1.428968087	NA	NA
ASV_16s_9185	0.516304307	0.693685543	3.550696078	1.35530972	0.986900934	NA
ASV_16s_9186	0.354927077	0.271659092	3.550584852	0.860030372	NA	NA
ASV_16s_9187	0.36321248	-0.119613813	3.549897233	1.684970949	NA	NA
ASV_16s_9188	0.379060965	-0.161527847	3.549827794	1.74511648	NA	NA
ASV_16s_9189	0.365283831	-0.186131514	3.549851518	1.71711439	NA	NA
ASV_16s_9190	0.381417643	-0.201838558	3.549802786	1.759196294	NA	NA
ASV_16s_9191	0.390552146	-0.232363402	3.549770392	1.778133343	0.971094281	NA
ASV_16s_9192	0.364564115	-0.240802249	3.549833475	1.710729552	0.974106202	NA
ASV_16s_9193	0.400678707	-0.269757226	3.549739032	1.787603405	0.970655927	NA
ASV_16s_9194	0.371830296	-0.27865522	3.549806121	1.712026273	0.974050044	NA
ASV_16s_9195	0.35952834	-0.277009493	3.549839335	1.681614994	0.975348672	NA
ASV_16s_9196	0.362820132	-0.279676717	3.549829711	1.688526793	0.975056898	NA
ASV_16s_9197	0.382684059	-0.31086338	3.54977322	1.71544886	0.973901484	NA
ASV_16s_9198	0.397384295	-0.336566582	3.549736432	1.725882032	0.973445617	NA
ASV_16s_9199	0.359107471	-0.410426115	3.54983954	1.535048843	NA	NA
ASV_16s_9200	0.39862721	-0.43040635	3.549742559	1.591703689	0.978964025	NA
ASV_16s_9201	0.126882156	-0.493036445	3.551210597	0.538034749	NA	NA
ASV_16s_9202	0.355423331	-0.681313332	3.549987855	0.88009561	NA	NA
ASV_16s_9203	0.113240146	-0.169611131	3.550741236	0.538135843	NA	NA
ASV_16s_9204	0.119401962	0.220115877	3.550818794	0.534858006	NA	NA
ASV_16s_9205	0.379197434	0.202894317	3.549275066	1.830966841	0.968600845	NA
ASV_16s_9206	0.361423749	-0.347527139	3.549402079	1.062180054	NA	NA
ASV_16s_9207	0.362423819	-0.337924283	3.549339946	1.107169758	NA	NA
ASV_16s_9208	0.393356973	-0.021796381	3.549106509	1.75886472	0.971974635	NA
ASV_16s_9209	0.369240251	0.274399325	3.549580123	1.515507124	0.981767943	NA
ASV_16s_9210	0.356619664	-0.633038573	3.549757033	1.41976974	0.984958027	NA
ASV_16s_9211	0.377477092	-0.917601484	3.54958753	1.16937418	0.99161504	NA
ASV_16s_9212	0.461076643	-0.797216193	3.549351859	1.27882906	0.98899721	NA
ASV_16s_9213	0.369558567	-1.227327368	3.54970793	0.681395194	NA	NA
ASV_16s_9214	0.421460587	-0.768839635	3.549157622	1.184991449	NA	NA
ASV_16s_9215	0.476963312	-0.791603136	3.549115552	1.564917465	NA	NA
ASV_16s_9216	0.422112709	1.336126048	3.550061906	1.207487944	NA	NA
ASV_16s_9217	0.472173228	-0.791011506	3.549331522	1.416531924	0.985059518	NA
ASV_16s_9218	0.464830313	-0.023614902	3.54915406	1.960159273	0.962012481	NA
ASV_16s_9219	0.453157714	-0.768193194	3.549450948	1.625339354	0.977650607	NA
ASV_16s_9220	0.388813661	0.146898852	3.549755331	1.913352145	0.964479969	NA
ASV_16s_9221	0.42864432	-0.716191934	3.549682047	1.601443324	0.978588497	NA
ASV_16s_9222	0.477783482	0.688610954	3.550555033	1.604314884	0.978477034	NA
ASV_16s_9223	0.603058471	-0.706312895	3.549022777	1.406672671	NA	NA
ASV_16s_9224	0.431840005	-1.232316466	3.549188599	1.07531187	0.993520477	NA
ASV_16s_9225	0.438533791	0.563167899	3.549503452	1.51191129	0.981894424	NA
ASV_16s_9226	0.452344083	-0.674681885	3.549789318	1.647479564	0.976760607	NA

ASV_16s_9227	0.539077241	0.713062497	3.54933633	1.440828643	NA	NA
ASV_16s_9228	0.42089826	0.757623291	3.55004538	1.273783763	NA	NA
ASV_16s_9229	0.564398921	0.246757162	3.549573316	1.655287694	0.976441899	NA
ASV_16s_9230	0.527243326	0.649646847	3.548410396	1.83466179	0.9684221	NA
ASV_16s_9231	0.443010211	-0.72054687	3.549471843	1.429104824	NA	NA
ASV_16s_9232	0.456118036	-0.076852514	3.549485859	2.081072627	0.95521711	NA
ASV_16s_9233	0.465243722	2.081901544	3.549732578	0.589381469	0.999048959	NA
ASV_16s_9234	0.406234676	1.337539286	3.550216628	0.88091473	NA	NA
ASV_16s_9235	0.39852024	1.959144424	3.54985447	0.542859974	NA	NA
ASV_16s_9236	0.473520508	-0.417384954	3.549502454	1.231340765	NA	NA
ASV_16s_9237	0.510133767	0.654483963	3.551035632	0.796220791	0.99748233	NA
ASV_16s_9238	0.298459246	0.397321993	3.550935244	0.806000557	NA	NA
ASV_16s_9239	0.330791577	0.104780981	3.550074619	1.436140223	NA	NA
ASV_16s_9240	0.480422974	0.403387841	3.550212164	0.960323768	NA	NA
ASV_16s_9241	0.548253165	-0.970169167	3.549326888	0.721308294	NA	NA
ASV_16s_9242	0.438722482	-0.565654579	3.549605112	0.925312308	0.995945089	NA
ASV_16s_9243	0.298074597	-0.563924309	3.550036936	0.801239807	NA	NA
ASV_16s_9244	0.339769816	-1.355718161	3.549615109	0.490119613	0.999481861	NA
ASV_16s_9245	0.250886906	-0.547271359	3.550006231	0.675888773	NA	NA
ASV_16s_9246	0.243847157	0.220022015	3.550695306	0.876537503	0.996582167	NA
ASV_16s_9247	0.258871158	0.224064866	3.550742034	0.715458082	0.998214124	NA
ASV_16s_9248	0.248550052	0.220020252	3.550693059	0.88615131	0.996462162	NA
ASV_16s_9249	0.253222955	0.220018601	3.550690909	0.895537776	0.996342382	NA
ASV_16s_9250	0.261190928	0.220016023	3.55068742	0.91117122	0.996137092	NA
ASV_16s_9251	0.277613968	0.486513737	3.550630361	1.013846893	NA	NA
ASV_16s_9252	0.525336639	0.833547255	3.548067801	1.156070728	0.991903439	NA
ASV_16s_9253	0.370857709	-0.069112489	3.549865407	1.785038664	NA	NA
ASV_16s_9254	0.371848746	-0.069081101	3.549855219	1.787737496	NA	NA
ASV_16s_9255	0.442437954	-0.237664176	3.550675066	1.225146781	NA	NA
ASV_16s_9256	0.376803929	-0.068850654	3.549852322	1.7745182	NA	NA
ASV_16s_9257	0.113640942	-0.379130866	3.551500106	0.491830097	NA	NA
ASV_16s_9258	0.418661672	-0.105744903	3.550237331	1.579286295	0.97943715	NA
ASV_16s_9259	0.516153157	-0.002615218	3.549629239	2.058665127	0.956522085	NA
ASV_16s_9260	0.667077126	0.638673491	3.549536558	2.204012747	0.947691156	NA
ASV_16s_9261	0.510406906	0.34272477	3.549623516	2.064511388	NA	NA
ASV_16s_9262	0.534515863	0.562330165	3.549670628	1.983729919	0.960735383	NA
ASV_16s_9263	0.513588683	0.76606547	3.549708772	1.449276972	NA	NA
ASV_16s_9264	0.509869855	0.363398604	3.550136078	1.050290609	NA	NA
ASV_16s_9265	0.497696414	0.342721062	3.55066442	1.133723242	0.992373723	NA
ASV_16s_9266	0.39414707	0.360396129	3.550851802	1.322637393	NA	NA
ASV_16s_9267	0.39414707	0.360396129	3.550851802	1.322637393	NA	NA
ASV_16s_9268	0.474874379	0.315939402	3.550738577	1.378242624	0.98622837	NA
ASV_16s_9269	0.519531567	0.315939224	3.550725599	1.423838058	0.984829914	NA
ASV_16s_9270	0.43274651	0.31593925	3.550753276	1.331062085	0.987589847	NA
ASV_16s_9271	0.411671506	0.430510896	3.550745267	2.136266462	NA	NA
ASV_16s_9272	0.544590817	0.734928652	3.550773432	1.423147363	NA	NA
ASV_16s_9273	0.460500303	0.693627784	3.550713469	1.297197016	0.988514157	NA
ASV_16s_9274	0.350847455	0.271662222	3.550604165	0.856301789	NA	NA
ASV_16s_9275	0.37075755	-0.136739643	3.549864504	1.713875831	NA	NA
ASV_16s_9276	0.378766264	-0.153521009	3.549833084	1.742146913	NA	NA
ASV_16s_9277	0.351041394	-0.129367246	3.549925845	1.659382806	NA	NA
ASV_16s_9278	0.373413337	-0.24836301	3.549808371	1.730692054	0.973233921	NA
ASV_16s_9279	0.369056458	-0.268382475	3.549815662	1.710743598	0.974105594	NA
ASV_16s_9280	0.351132659	-0.261670639	3.549866234	1.667555898	NA	NA
ASV_16s_9281	0.351144067	-0.278535303	3.549862691	1.658770654	NA	NA
ASV_16s_9282	0.390945679	-0.316517886	3.549752843	1.730309488	0.973250793	NA
ASV_16s_9283	0.356575658	-0.471742323	3.549865355	1.398868747	NA	NA
ASV_16s_9284	0.125423741	-0.489058288	3.551220917	0.534276248	NA	NA
ASV_16s_9285	0.351338005	-0.681249294	3.55000233	0.876369912	NA	NA
ASV_16s_9289	0.362569019	0.161206963	3.549822832	1.657319416	NA	NA
ASV_16s_9290	0.391546638	0.016765753	3.549508851	1.726942632	0.973399021	NA
ASV_16s_9291	0.41861465	-0.480485477	3.54966681	1.590857954	NA	NA
ASV_16s_9292	0.363042258	0.515826882	3.549464836	1.608230089	NA	NA
ASV_16s_9293	0.37792485	0.067609888	3.549167142	1.814418802	0.969394362	NA
ASV_16s_9294	0.372644539	-0.631225584	3.292755972	1.534155322	0.98110359	NA
ASV_16s_9295	0.382640275	-0.017344561	3.549052114	1.810892583	0.969561974	NA
ASV_16s_9296	0.376349882	-0.367405359	3.549704938	1.413147211	NA	NA

ASV_16s_9297	0.468969541	-0.771205951	3.549820493	1.104695478	0.992958369	NA
ASV_16s_9298	0.41820755	-0.722223488	3.549548065	1.566835708	NA	NA
ASV_16s_9299	0.483097664	-1.316286587	3.549127763	1.300284192	0.988431711	NA
ASV_16s_9300	0.444023552	-0.755732492	3.549538535	1.618623487	0.977916573	NA
ASV_16s_9301	0.428340445	-0.834332214	3.549374843	1.352049746	NA	NA
ASV_16s_9302	0.533319679	0.421464816	3.550416412	1.750498763	NA	NA
ASV_16s_9303	0.441366144	0.182572334	3.54942517	1.610184306	NA	NA
ASV_16s_9304	0.410271197	-0.282431314	3.549480694	0.849987014	NA	NA
ASV_16s_9305	0.487581034	-0.298093442	3.549618171	1.973719842	0.961280569	NA
ASV_16s_9306	0.418727407	0.217429606	3.549538322	1.452283339	NA	NA
ASV_16s_9307	0.364634421	-0.159550475	3.551586306	0.500117273	NA	NA
ASV_16s_9308	0.404479056	-0.348918757	3.550283334	1.44916076	0.984017711	NA
ASV_16s_9309	0.391903514	-0.070685752	3.550578466	1.31954862	0.987909023	NA
ASV_16s_9310	0.475062667	0.519801969	3.550728843	2.092483153	0.954544634	NA
ASV_16s_9311	0.499499398	-0.303555619	3.550250325	2.40997874	0.933721858	NA
ASV_16s_9312	0.532719648	-0.008642684	3.549729865	1.731424123	NA	NA
ASV_16s_9313	0.498732425	2.121793394	3.549641622	0.605065323	NA	NA
ASV_16s_9314	0.396971884	1.876458766	3.550125151	0.609331786	NA	NA
ASV_16s_9315	0.47490087	0.403382587	3.5502272	0.956589864	NA	NA
ASV_16s_9316	0.513540076	-0.566194405	3.549471124	0.975978955	NA	NA
ASV_16s_9317	0.189550797	-0.384920255	3.551292164	0.642399096	NA	NA
ASV_16s_9318	0.589575504	-0.566602331	3.549369606	1.020432393	NA	NA
ASV_16s_9319	0.6126098	-1.889084037	3.548161574	0.545109805	NA	NA
ASV_16s_9320	0.823803176	-0.567388909	3.549174173	1.128206045	NA	NA
ASV_16s_9321	0.346487899	-0.951213795	3.549758028	0.929462361	NA	NA
ASV_16s_9322	0.126552882	0.35961486	3.551574743	0.457790046	NA	NA
ASV_16s_9323	0.204205707	-0.541711211	3.550295723	0.61057949	NA	NA
ASV_16s_9324	0.204205707	-0.541711211	3.550295723	0.61057949	NA	NA
ASV_16s_9325	0.36149324	-0.079126868	3.550665756	1.235320055	0.990090794	NA
ASV_16s_9326	0.243323495	0.220022097	3.550695562	0.875442723	0.996595662	NA
ASV_16s_9327	0.257815174	0.220017146	3.550688873	0.904592458	NA	NA
ASV_16s_9328	0.408415902	0.040145914	3.55013728	1.919162088	NA	NA
ASV_16s_9329	0.360381063	-0.069284182	3.550038024	1.713402573	NA	NA
ASV_16s_9330	0.41923337	1.414021794	3.54999457	1.512760922	NA	NA
ASV_16s_9331	0.267008948	-0.928020584	3.550082343	0.57280104	NA	NA
ASV_16s_9332	0.581339826	0.66970072	3.549588656	2.073638294	0.955652367	NA
ASV_16s_9333	0.383109311	1.378492468	3.550451947	1.005164814	NA	NA
ASV_16s_9334	0.374751094	-0.06862316	3.549889747	1.741746101	NA	NA
ASV_16s_9335	0.447938214	-0.564066642	3.548961491	1.594594791	0.97885296	NA
ASV_16s_9336	0.293361265	-1.07856129	3.549791374	0.476809554	NA	NA
ASV_16s_9337	0.173721836	0.279026767	3.551605841	0.638863265	NA	NA
ASV_16s_9338	0.173721836	0.279026767	3.551605841	0.638863265	NA	NA
ASV_16s_9339	0.173721836	0.279026767	3.551605841	0.638863265	NA	NA
ASV_16s_9340	0.112334724	-0.376034536	3.551505226	0.488259566	NA	NA
ASV_16s_9341	0.606124073	0.362118285	3.55082031	0.809200368	0.997348923	NA
ASV_16s_9342	0.460897717	-0.549743835	3.549407916	0.870557376	NA	NA
ASV_16s_9343	0.330684748	-0.078787743	3.550477804	1.439026143	NA	NA
ASV_16s_9344	0.336349778	-0.11767686	3.549809577	1.649712305	0.97666973	NA
ASV_16s_9345	0.426603534	0.342858631	3.549751096	1.855470437	NA	NA
ASV_16s_9346	0.460381651	0.343664431	3.549813311	1.825533547	NA	NA
ASV_16s_9347	0.500561381	0.343964939	3.549827123	1.845657693	NA	NA
ASV_16s_9348	0.468771905	0.315939216	3.550740547	1.371631705	0.986424356	NA
ASV_16s_9349	0.421996257	0.315939426	3.550757498	1.318281584	0.987943837	NA
ASV_16s_9350	0.419258692	0.315939427	3.550758607	1.314977522	0.988034331	NA
ASV_16s_9351	0.41497354	0.716223454	3.550135249	1.619367208	0.977887211	NA
ASV_16s_9352	0.417547714	0.315939427	3.550759308	1.312901624	0.988090974	NA
ASV_16s_9353	0.511661483	1.153074554	3.550242625	1.061557368	0.993773483	NA
ASV_16s_9354	0.538525607	1.071033012	3.550430405	1.137695075	0.992291431	NA
ASV_16s_9355	0.266665385	0.734218356	3.550978567	1.061379946	NA	NA
ASV_16s_9356	0.454118771	0.693620225	3.55071573	1.290110787	0.988702031	NA
ASV_16s_9357	0.565318501	0.693726146	3.550683634	1.401357726	0.985529642	NA
ASV_16s_9358	0.346767834	0.271665423	3.550623927	0.852529728	NA	NA
ASV_16s_9359	0.363937979	-0.128645852	3.549888805	1.69064119	NA	NA
ASV_16s_9360	0.355571074	-0.143214268	3.549902959	1.678447155	NA	NA
ASV_16s_9361	0.347007405	-0.199725463	3.549897617	1.669972097	NA	NA
ASV_16s_9362	0.374857038	-0.234083013	3.549808403	1.738706743	0.972879041	NA
ASV_16s_9363	0.380165965	-0.293016477	3.549782165	1.723209444	0.973562825	NA

ASV_16s_9364	0.408625359	-0.367152934	3.549711484	1.715231071	0.973910952	NA
ASV_16s_9365	0.383597081	-0.352496323	3.549768682	1.675839105	0.975590974	NA
ASV_16s_9366	0.123965325	-0.485036707	3.551231424	0.530484153	NA	NA
ASV_16s_9367	0.398483482	-0.681916988	3.549853504	0.916831434	NA	NA
ASV_16s_9368	0.155132453	0.200450741	3.550690968	1.073405361	0.993555929	NA
ASV_16s_9369	0.066660408	-0.16895859	3.550904401	0.330699176	NA	NA
ASV_16s_9370	0.154231695	0.220074412	3.550764221	0.651621704	NA	NA
ASV_16s_9372	0.363748776	0.00880317	3.54979619	1.668286794	NA	NA
ASV_16s_9373	0.39011858	0.184971149	3.549673164	1.599690869	0.978656353	NA
ASV_16s_9374	0.379914389	0.003995376	3.549364043	1.684507955	0.97522679	NA
ASV_16s_9375	0.45127727	-0.813855189	3.54890395	0.988760065	0.99500782	NA
ASV_16s_9376	0.488632176	-0.20826384	3.54889879	2.111360683	0.953420344	NA
ASV_16s_9377	0.396296855	0.717188645	3.550132916	1.403128174	NA	NA
ASV_16s_9378	0.378211988	0.172786352	3.549690408	1.466775183	0.983437662	NA
ASV_16s_9379	0.429383909	-0.79126791	3.549267654	1.43407827	NA	NA
ASV_16s_9380	0.471144353	2.21596646	3.549018131	0.417066901	0.999697077	NA
ASV_16s_9381	0.38806517	0.588289958	3.551442355	0.750067412	NA	NA
ASV_16s_9382	0.489771751	-0.730501419	3.548734901	1.324884291	NA	NA
ASV_16s_9383	0.450131838	-0.764973219	3.549474728	1.622417213	0.97776656	NA
ASV_16s_9384	0.405622094	-0.875204353	3.54994241	1.044736639	NA	NA
ASV_16s_9385	0.374118448	0.362172057	3.548944696	1.132046531	NA	NA
ASV_16s_9386	0.398278183	-0.641357669	3.549953656	1.277342772	0.98903574	NA
ASV_16s_9387	0.359008986	-0.673009675	3.549933439	1.192392393	NA	NA
ASV_16s_9388	0.41493307	-0.684178658	3.549790677	1.577017338	NA	NA
ASV_16s_9389	0.418746103	0.148816382	3.549195393	1.242399252	0.989917675	NA
ASV_16s_9390	0.434197087	25.07102199	3.549670244	-0.537576775	1	NA
ASV_16s_9391	0.261401537	0.461355643	3.550676044	0.901900157	0.996259712	NA
ASV_16s_9392	0.49990028	-0.583414693	3.548964551	1.266308928	0.989319167	NA
ASV_16s_9393	0.296900558	-0.969720305	3.549945877	0.541499835	NA	NA
ASV_16s_9394	0.279272867	1.311122148	3.550494984	0.694153958	NA	NA
ASV_16s_9395	0.60616341	1.338399721	3.549671308	0.94633247	NA	NA
ASV_16s_9396	0.125081337	0.358901384	3.551580843	0.454157495	NA	NA
ASV_16s_9397	0.248000147	0.220020443	3.550693319	0.885009325	0.996476558	NA
ASV_16s_9398	0.255327193	0.220017987	3.550689969	0.899686183	NA	NA
ASV_16s_9399	0.254913298	0.220018014	3.550690152	0.898867858	0.996299262	NA
ASV_16s_9400	0.465518884	-0.129998179	3.548785196	1.307692699	0.988232379	NA
ASV_16s_9401	0.481893502	0.76497573	3.54949536	1.585735595	0.979192209	NA
ASV_16s_9402	0.321607299	-0.033486414	3.550571793	0.449549606	0.999611048	NA
ASV_16s_9403	0.342529799	0.343640966	3.550233742	1.312230099	NA	NA
ASV_16s_9404	0.369984782	-0.235563258	3.549936703	1.515698988	NA	NA
ASV_16s_9405	0.385200367	-0.069227464	3.549887354	1.808438522	0.969678315	NA
ASV_16s_9406	0.42379263	1.553324705	3.549275467	0.839316829	NA	NA
ASV_16s_9407	0.3853505	-0.059075837	3.550317505	1.524698321	NA	NA
ASV_16s_9408	0.437147548	0.677119009	3.550710023	1.309263565	0.988189845	NA
ASV_16s_9409	0.713531454	-0.199332645	3.547556515	2.38279193	NA	NA
ASV_16s_9410	0.445475414	1.234558682	3.549852348	1.539148744	0.980923298	NA
ASV_16s_9411	0.325504626	-0.117728389	3.549854993	1.609333692	NA	NA
ASV_16s_9412	0.544642646	0.2490977	3.55033598	2.01996098	0.95872731	NA
ASV_16s_9413	0.485577116	0.586148503	3.549095655	1.850412961	0.96765373	NA
ASV_16s_9414	0.379745902	-0.029344873	3.55073337	1.319510582	NA	NA
ASV_16s_9415	0.468120593	0.356516067	3.550119879	1.725970769	0.97344172	NA
ASV_16s_9416	0.295153668	0.734350273	3.55093991	1.11238863	0.992806284	NA
ASV_16s_9417	0.513868353	0.981135233	3.550596336	1.161320716	0.991790383	NA
ASV_16s_9418	0.487969572	0.693656785	3.550704414	1.32661504	0.987713732	NA
ASV_16s_9419	0.502084677	0.69367429	3.550700145	1.341102214	0.987307349	NA
ASV_16s_9420	0.437011109	0.69359841	3.550722113	1.270624836	0.98920885	NA
ASV_16s_9421	0.499350109	0.693665123	3.550700955	1.338309661	0.987386314	NA
ASV_16s_9422	0.493960411	0.693660782	3.550702576	1.332777357	0.98754186	NA
ASV_16s_9423	0.448558713	0.693613459	3.550717751	1.283856217	0.988866273	NA
ASV_16s_9424	0.353303885	-0.206093887	3.549876441	1.686802169	NA	NA
ASV_16s_9425	0.34905057	-0.276768442	3.549869248	1.654167018	0.976487798	NA
ASV_16s_9426	0.342973415	-0.271569504	3.549888644	1.64075884	NA	NA
ASV_16s_9427	0.352865904	-0.305740718	3.549852798	1.64474964	0.97687144	NA
ASV_16s_9428	0.365275799	-0.314982156	3.549817315	1.668889915	0.975880662	NA
ASV_16s_9429	0.373339702	-0.346007559	3.549794436	1.65853164	0.976308748	NA
ASV_16s_9430	0.399811539	-0.362168401	3.549730788	1.70177587	0.974492054	NA
ASV_16s_9431	0.377853787	-0.34890245	3.549782914	1.666249087	0.975990224	NA

ASV_16s_9432	0.3488601	-0.375408363	3.549863621	1.563708538	0.980021755	NA
ASV_16s_9433	0.361105674	-0.561549618	3.549890626	1.189856847	NA	NA
ASV_16s_9434	0.343167354	-0.681116809	3.550032305	0.868787421	NA	NA
ASV_16s_9435	0.394648035	-0.026871357	3.549629428	1.773139358	0.971323937	NA
ASV_16s_9436	0.384198217	0.041620484	3.549443724	1.722276084	0.973603688	NA
ASV_16s_9437	0.364749961	0.070379231	3.549426986	1.702597237	NA	NA
ASV_16s_9438	0.34728597	0.414673298	3.549882406	1.600759059	NA	NA
ASV_16s_9439	0.357922939	-0.818393397	3.549702759	1.288465792	0.988745371	NA
ASV_16s_9440	0.356124451	-0.249960708	3.550144672	1.399301463	0.98559265	NA
ASV_16s_9441	0.354394361	0.427170914	3.549473293	1.662887124	NA	NA
ASV_16s_9442	0.43689657	0.048186141	3.549023094	2.038329323	0.957688472	NA
ASV_16s_9443	0.352020203	-0.320751137	3.550826255	0.96583546	0.995360992	NA
ASV_16s_9444	0.426507603	-0.77697716	3.549436361	1.542741036	0.980792968	NA
ASV_16s_9445	0.463656031	-0.775416851	3.549395583	1.639102734	0.977099724	NA
ASV_16s_9446	0.43530243	-0.761443619	3.54951414	1.592201792	0.978944914	NA
ASV_16s_9447	0.448014888	-0.757658644	3.54952534	1.625848267	0.977630377	NA
ASV_16s_9448	0.45829645	-1.882835139	3.547676659	0.493651748	0.999469396	NA
ASV_16s_9449	0.446185344	-0.733006983	3.549618153	1.633616792	0.977320237	NA
ASV_16s_9450	0.470940821	-0.276796856	3.549434708	1.64108794	NA	NA
ASV_16s_9451	0.445176834	-0.722772051	3.549649524	1.633830494	0.977311671	NA
ASV_16s_9452	0.303786169	-0.185233858	3.549968396	1.30127504	NA	NA
ASV_16s_9453	0.429019752	-0.673315382	3.549807941	1.604792194	NA	NA
ASV_16s_9454	0.420666085	-0.651521727	3.549864236	1.58708991	0.979140557	NA
ASV_16s_9455	0.378471421	1.520484134	3.54884553	0.907798947	NA	NA
ASV_16s_9456	0.419281763	-0.06831356	3.550219179	1.482027166	0.982925363	NA
ASV_16s_9457	0.472534739	0.187926201	3.550394837	1.595639059	0.978812759	NA
ASV_16s_9458	0.401219702	-0.417130193	3.550361524	1.452176896	0.983919269	NA
ASV_16s_9459	0.419857575	-1.009878885	3.349038322	0.602636415	0.998977192	NA
ASV_16s_9460	0.479601207	0.913736509	3.548378758	1.269789468	0.989230257	NA
ASV_16s_9461	0.405034487	0.220559563	3.54883165	1.744227644	NA	NA
ASV_16s_9462	0.261733026	0.117203548	3.550686301	0.746995592	0.997948014	NA
ASV_16s_9463	0.427495509	1.337845065	3.550146642	0.897310635	NA	NA
ASV_16s_9464	0.48015567	0.4033874	3.550213118	0.960051697	0.995447475	NA
ASV_16s_9465	0.251990842	0.22904166	3.550854188	0.524363908	0.99935229	NA
ASV_16s_9466	0.353835328	-1.347501695	3.549442242	0.687542089	0.998429637	NA
ASV_16s_9467	0.487947209	-0.273818794	3.547950974	1.558166958	NA	NA
ASV_16s_9468	0.230932919	-0.540649481	3.550563892	0.682389993	NA	NA
ASV_16s_9469	0.251471015	-1.106223175	3.550177893	0.430312661	NA	NA
ASV_16s_9470	0.396774855	0.703759525	3.549854796	1.556126051	0.98030272	NA
ASV_16s_9471	0.259055399	0.189933481	3.549830018	1.246596047	NA	NA
ASV_16s_9472	0.222003746	-0.544593354	3.550172507	0.636956827	NA	NA
ASV_16s_9473	0.244211407	0.220021847	3.550695132	0.877247021	0.996573403	NA
ASV_16s_9474	0.252323344	0.220019022	3.55069132	0.893704567	NA	NA
ASV_16s_9475	0.273552497	0.21930647	3.550705314	0.67438743	NA	NA
ASV_16s_9476	0.417229801	0.310930937	3.550744942	1.289633331	0.988714621	NA
ASV_16s_9477	0.58047978	0.666153167	3.064637702	3.628643977	0.82141779	NA
ASV_16s_9478	0.403787262	1.065626409	3.55069588	0.950101201	NA	NA
ASV_16s_9479	0.356770912	-0.069123536	3.54991285	1.744194753	NA	NA
ASV_16s_9480	0.362419826	-0.06910489	3.549889795	1.761555092	0.971852647	NA
ASV_16s_9481	0.369037752	-0.069060875	3.549857173	1.779134433	0.971048119	NA
ASV_16s_9482	0.363708169	-0.068804562	3.549878453	1.741354792	NA	NA
ASV_16s_9483	0.364699206	-0.068733139	3.549888294	1.733328446	NA	NA
ASV_16s_9484	0.208717573	0.205441316	3.551283515	0.434645454	NA	NA
ASV_16s_9485	0.206035247	0.279547107	3.551528043	0.700100658	NA	NA
ASV_16s_9486	0.389106032	-0.457215194	3.550196824	1.417585292	0.985026545	NA
ASV_16s_9487	0.419451766	-0.423643664	3.550351597	1.601316311	NA	NA
ASV_16s_9488	0.465900341	0.343776823	3.549793799	1.899466901	0.965194369	NA
ASV_16s_9489	0.488210731	0.351378742	3.5499804	1.790520011	0.970520168	NA
ASV_16s_9490	0.4281029	0.315939329	3.550755079	1.325536873	0.987743653	NA
ASV_16s_9491	0.421511717	0.31593933	3.550757697	1.317663165	0.987960806	NA
ASV_16s_9492	0.451372101	0.315939328	3.55074645	1.352374568	0.986985538	NA
ASV_16s_9493	0.436431665	0.39226795	3.550739623	2.00669055	0.959469133	NA
ASV_16s_9494	0.345599399	-0.087402646	3.549976766	1.614105185	NA	NA
ASV_16s_9495	0.346893994	-0.132608661	3.549936296	1.649999253	NA	NA
ASV_16s_9496	0.338808233	-0.148292539	3.549951058	1.635856157	NA	NA
ASV_16s_9497	0.363778154	-0.208287026	3.549846253	1.714083909	NA	NA
ASV_16s_9498	0.348706425	-0.193200103	3.549895513	1.6740507	NA	NA

ASV_16s_9499	0.35519585	-0.207954472	3.549870307	1.691671988	0.97492347	NA
ASV_16s_9500	0.38474818	-0.242444186	3.54978165	1.760837722	0.971885204	NA
ASV_16s_9501	0.341881035	-0.202752386	3.549911959	1.656003757	NA	NA
ASV_16s_9502	0.346703524	-0.224343808	3.549889144	1.667296966	0.975946785	NA
ASV_16s_9503	0.344430295	-0.230523832	3.549893972	1.659971531	0.976249506	NA
ASV_16s_9504	0.33885957	-0.22512424	3.549912842	1.645814128	NA	NA
ASV_16s_9505	0.371329882	-0.294491388	3.54980431	1.70010289	0.97456378	NA
ASV_16s_9506	0.343445956	-0.271972407	3.549887136	1.641775159	0.976991852	NA
ASV_16s_9507	0.366649502	-0.315958463	3.549813578	1.67143717	0.975774708	NA
ASV_16s_9508	0.344988061	-0.308431238	3.549875576	1.621984777	0.97778369	NA
ASV_16s_9509	0.377225699	-0.357035914	3.549784661	1.655344007	0.976439592	NA
ASV_16s_9510	0.400498254	-0.68193482	3.549847964	0.918430698	0.996039279	NA
ASV_16s_9511	0.339082028	-0.681051479	3.550047831	0.86492889	NA	NA
ASV_16s_9512	0.219484808	0.220032079	3.550708489	0.823712947	NA	NA
ASV_16s_9513	0.353138754	-0.120120351	3.549840331	1.557227191	0.980262064	NA
ASV_16s_9514	0.392985257	0.088657007	3.549709629	1.837870679	NA	NA
ASV_16s_9515	0.358765234	0.043713245	3.549749975	1.661098757	0.976203069	NA
ASV_16s_9516	0.36749274	0.305183992	3.549429728	1.582234756	0.979325381	NA
ASV_16s_9517	0.346459824	0.079049084	3.549455917	1.679265005	0.975447422	NA
ASV_16s_9518	0.373727306	-0.363978444	3.549815035	1.447642376	0.984067132	NA
ASV_16s_9519	0.345257101	0.942771232	3.550580964	0.953761222	NA	NA
ASV_16s_9520	0.34687724	0.218037343	3.549324895	1.770931837	0.971425121	NA
ASV_16s_9521	0.29319784	-0.067460851	3.550658475	1.107920994	NA	NA
ASV_16s_9522	0.394790237	0.792265318	3.549491276	1.658846236	NA	NA
ASV_16s_9523	0.375338722	0.852073999	3.549115517	1.605246721	NA	NA
ASV_16s_9524	0.350582831	-0.324690065	3.549303413	1.244912021	NA	NA
ASV_16s_9525	0.289133095	0.504572904	3.549822001	1.403542168	0.985462524	NA
ASV_16s_9526	0.431084863	-0.820005653	3.548701466	0.849467506	NA	NA
ASV_16s_9527	0.44778046	-0.840777062	3.549416745	1.319878611	0.987899946	NA
ASV_16s_9528	0.546555492	-1.307935708	3.547680612	0.539440474	NA	NA
ASV_16s_9529	0.400235106	-0.755076181	3.549769694	1.56532633	0.979961504	NA
ASV_16s_9530	0.438029641	0.267119061	3.550355689	1.637648292	0.977158308	NA
ASV_16s_9531	0.416482869	-0.097904586	3.550345389	1.221900069	NA	NA
ASV_16s_9532	0.409641557	0.449604059	3.550784185	1.713101744	0.974003415	NA
ASV_16s_9533	0.456379938	-0.718089923	3.548581391	0.864976487	NA	NA
ASV_16s_9534	0.322569252	-1.415941724	3.549201639	0.743622462	0.997977652	NA
ASV_16s_9535	0.436144559	-0.110827152	3.550626831	1.404774698	0.985424571	NA
ASV_16s_9536	0.531956063	-0.538819208	3.549545755	1.549286529	0.980554141	NA
ASV_16s_9537	0.260522836	-0.037082598	3.550686967	0.713313878	0.998231335	NA
ASV_16s_9538	0.472687744	0.429108771	3.550654381	1.334844016	0.987483892	NA
ASV_16s_9539	0.503898226	1.248004995	3.549560511	1.302260482	NA	NA
ASV_16s_9540	0.495482355	0.667919842	3.531542082	1.305564116	0.988289865	NA
ASV_16s_9541	0.405322987	0.131257383	3.550704029	0.998203422	NA	NA
ASV_16s_9542	0.453348369	2.095184412	3.549708542	0.567716091	NA	NA
ASV_16s_9543	0.375397015	1.333140899	3.55031609	0.855001885	NA	NA
ASV_16s_9544	0.400904723	1.337460324	3.550235483	0.876617176	NA	NA
ASV_16s_9545	0.367430876	0.403249843	3.550607961	0.873959974	NA	NA
ASV_16s_9546	0.366197333	0.097689266	3.548994812	1.434302846	0.984497358	NA
ASV_16s_9547	0.555778692	0.424324622	3.550821793	0.634425642	NA	NA
ASV_16s_9548	0.50415339	-0.241350397	3.549865285	1.176789535	NA	NA
ASV_16s_9549	0.433134007	0.366911112	3.551707669	0.527199876	NA	NA
ASV_16s_9550	0.40246541	-1.554673436	3.549112522	0.482981083	NA	NA
ASV_16s_9551	0.302735568	-0.884608957	3.54997755	0.692819012	NA	NA
ASV_16s_9552	0.403072167	0.951032066	3.54978891	1.621299405	0.977810822	NA
ASV_16s_9553	0.122138247	0.357450401	3.551593383	0.446805608	NA	NA
ASV_16s_9554	0.219360844	-0.544148011	3.550189626	0.633142663	NA	NA
ASV_16s_9555	0.219360844	-0.544148011	3.550189626	0.633142663	NA	NA
ASV_16s_9556	0.219360844	-0.544148011	3.550189626	0.633142663	NA	NA
ASV_16s_9557	0.416178856	0.19127758	3.550696414	2.175190373	0.94951083	NA
ASV_16s_9558	0.235390309	0.220025207	3.550699577	0.858750157	0.996797144	NA
ASV_16s_9559	0.236594639	0.22002463	3.55069895	0.861311446	0.996766748	NA
ASV_16s_9560	0.238385911	0.220023923	3.550698031	0.865098487	0.996721462	NA
ASV_16s_9561	0.352121778	-0.321825264	3.549714694	0.945832068	0.99565566	NA
ASV_16s_9562	0.573871049	-1.948271364	3.548492321	0.428202595	NA	NA
ASV_16s_9563	0.345493673	0.330669791	3.550874537	0.931917093	NA	NA
ASV_16s_9564	0.366610518	-0.067761999	3.550104878	1.612172238	NA	NA
ASV_16s_9565	0.467384036	1.909972516	3.549729603	0.542526139	NA	NA

ASV_16s_9566	0.408726647	0.312475857	3.550520975	0.977326693	0.995186043	NA
ASV_16s_9567	0.531035157	0.771508343	3.549847849	1.70617722	NA	NA
ASV_16s_9568	0.522336306	0.316758376	3.550734767	1.366352097	0.986579648	NA
ASV_16s_9569	0.203582447	0.279530328	3.55153357	0.695749437	NA	NA
ASV_16s_9570	0.285338859	-0.599926728	3.550794147	0.79785722	NA	NA
ASV_16s_9571	0.108416071	-0.366547271	3.551521119	0.477359448	NA	NA
ASV_16s_9572	0.439327899	0.341698689	3.550814304	0.836289595	0.997055727	NA
ASV_16s_9573	0.422446513	0.700038539	3.550663655	1.181301795	0.991351066	NA
ASV_16s_9574	0.409684404	-0.595755223	3.54969478	1.507586092	NA	NA
ASV_16s_9575	0.446645606	0.167870677	3.548797619	1.900009349	NA	NA
ASV_16s_9576	0.620406444	0.381313147	3.55068748	2.760265679	0.906258886	NA
ASV_16s_9577	0.480745349	0.343814123	3.549784987	1.922127855	0.964024307	NA
ASV_16s_9578	0.467566755	0.301648051	3.549996688	2.095930753	0.954340398	NA
ASV_16s_9579	0.498081972	0.183594379	3.550511154	2.11503209	NA	NA
ASV_16s_9580	0.451654815	0.35209283	3.55057147	1.048382652	0.994009846	NA
ASV_16s_9581	0.454827022	0.357197907	3.550510756	1.04143226	0.994132202	NA
ASV_16s_9582	0.376025366	0.36039228	3.550864215	1.298687521	NA	NA
ASV_16s_9583	0.376025366	0.36039228	3.550864215	1.298687521	NA	NA
ASV_16s_9584	0.43761149	0.315939349	3.550751447	1.336639731	0.98743339	NA
ASV_16s_9585	0.398561406	0.315939355	3.550767489	1.289229691	0.988725258	NA
ASV_16s_9586	0.422229651	0.983141173	3.550636192	1.070668558	0.993606604	NA
ASV_16s_9587	0.434073018	0.693594797	3.550723263	1.26717168	0.989297171	NA
ASV_16s_9588	0.491931873	0.69366407	3.550703194	1.330699169	0.987599986	NA
ASV_16s_9589	0.417758964	0.693571847	3.550729923	1.247737373	0.989785902	NA
ASV_16s_9590	0.494475879	0.693664731	3.550702424	1.333212976	0.987529655	NA
ASV_16s_9591	0.33646689	-0.101169119	3.549994498	1.601184875	NA	NA
ASV_16s_9592	0.382047206	-0.225031833	3.549793178	1.758451984	0.971993323	NA
ASV_16s_9593	0.358708489	-0.243616443	3.549848927	1.694326455	0.97481054	NA
ASV_16s_9594	0.337829933	-0.249812044	3.549908953	1.636363899	0.97720997	NA
ASV_16s_9595	0.340131683	-0.286718627	3.549893895	1.624318815	0.977691143	NA
ASV_16s_9596	0.342163105	-0.29728038	3.54988574	1.622761411	0.97775292	NA
ASV_16s_9597	0.365657258	-0.340938599	3.549814741	1.645043119	0.97685954	NA
ASV_16s_9598	0.338894129	-0.331018255	3.54989238	1.586394517	0.979167088	NA
ASV_16s_9599	0.340672337	-0.341614795	3.549886639	1.580785406	0.979380367	NA
ASV_16s_9600	0.387442804	-0.380285361	3.549760873	1.649744631	0.976668413	NA
ASV_16s_9601	0.341972635	-0.400562155	3.549888557	1.507617234	0.982044779	NA
ASV_16s_9602	0.119590078	-0.472707515	3.551264119	0.518931185	NA	NA
ASV_16s_9603	0.119590078	-0.472707515	3.551264119	0.518931185	NA	NA
ASV_16s_9604	0.184599268	0.377144935	3.550680996	1.111253465	NA	NA
ASV_16s_9605	0.064307688	-0.168901249	3.550918878	0.318328915	NA	NA
ASV_16s_9606	0.216840413	0.220033233	3.550710098	0.817645454	NA	NA
ASV_16s_9607	0.11253978	0.220127099	3.550833527	0.50913761	NA	NA
ASV_16s_9608	0.129337839	0.220101686	3.550800256	0.570251418	0.999146437	NA
ASV_16s_9609	0.378988759	0.276203881	3.549622694	1.836967952	NA	NA
ASV_16s_9610	0.340340925	0.395769314	3.549512881	1.540420202	NA	NA
ASV_16s_9611	0.406624092	-0.984147391	3.549755153	1.267231488	NA	NA
ASV_16s_9612	0.364391458	0.329133656	3.549500132	1.633664866	0.97731831	NA
ASV_16s_9613	0.400115903	-0.410958821	3.549759578	1.259175574	0.989499968	NA
ASV_16s_9614	0.345616252	0.684942206	3.549499646	1.502651049	NA	NA
ASV_16s_9615	0.419997112	-0.791242983	3.5493031	1.390661317	NA	NA
ASV_16s_9616	0.43973887	-0.791478139	3.549219776	1.442047261	0.984248448	NA
ASV_16s_9617	0.411910855	-0.001968499	3.549217395	1.871218349	NA	NA
ASV_16s_9618	0.414189495	-0.771378361	3.549485353	1.52065174	0.981586073	NA
ASV_16s_9619	0.43649939	-0.768130593	3.549470861	1.585368798	0.979206186	NA
ASV_16s_9620	0.387017081	-0.264928836	3.54914093	1.187187583	NA	NA
ASV_16s_9621	0.455890757	-0.667417205	3.549679218	1.633622045	0.977320027	NA
ASV_16s_9622	0.423488061	-0.660972609	3.549842561	1.591604508	0.978967829	NA
ASV_16s_9623	0.434034423	-0.638186068	3.549887049	1.604284741	0.978478206	NA
ASV_16s_9624	0.402756906	-0.088023164	3.550253497	1.371809376	NA	NA
ASV_16s_9625	0.345990381	-0.195204165	3.549697051	1.407495527	NA	NA
ASV_16s_9626	0.362221703	-0.074054129	3.55053853	1.409428694	0.985280721	NA
ASV_16s_9627	0.374490797	0.158570742	3.549685799	1.509979602	0.981962154	NA
ASV_16s_9628	0.334190829	0.283571355	3.549667613	1.283998987	0.98886254	NA
ASV_16s_9629	0.257974216	1.716798129	3.550350526	0.433773497	NA	NA
ASV_16s_9630	0.288797812	0.533484896	3.549535708	0.878988989	NA	NA
ASV_16s_9631	0.363003998	0.403242711	3.550628451	0.870056147	NA	NA
ASV_16s_9632	0.296071483	0.129790349	3.550812089	0.628717436	0.998825519	NA

ASV_16s_9633	0.579439411	0.419131353	3.549785544	0.951208954	NA	NA
ASV_16s_9634	0.384918179	0.02577353	3.549618236	1.639039322	NA	NA
ASV_16s_9635	0.120666701	0.356712529	3.55159983	0.44308532	NA	NA
ASV_16s_9636	0.120666701	0.356712529	3.55159983	0.44308532	NA	NA
ASV_16s_9637	0.120666701	0.356712529	3.55159983	0.44308532	NA	NA
ASV_16s_9638	0.236468118	-0.546680523	3.550085533	0.657073256	NA	NA
ASV_16s_9639	0.236468118	-0.546680523	3.550085533	0.657073256	NA	NA
ASV_16s_9640	0.171800319	1.19665311	3.550910353	0.526904039	NA	NA
ASV_16s_9641	0.234687148	0.220025398	3.550699946	0.85723394	0.996815049	NA
ASV_16s_9642	0.237185331	0.220024393	3.550698647	0.862548791	0.996751997	NA
ASV_16s_9643	0.237460806	0.22002427	3.550698505	0.86313165	0.996745033	NA
ASV_16s_9644	0.364040971	-0.023010611	3.550849352	0.54914151	0.999245911	NA
ASV_16s_9645	0.442796764	0.34118977	3.549435039	1.832910548	0.968506889	NA
ASV_16s_9646	0.416249345	1.129431157	3.550092439	1.098589812	0.993077613	NA
ASV_16s_9647	0.347092226	-0.273347615	3.549291605	1.390484035	NA	NA
ASV_16s_9648	0.352434008	-0.06919177	3.549974616	1.718085689	0.973786698	NA
ASV_16s_9649	0.348205579	-0.069106835	3.549934062	1.720342078	NA	NA
ASV_16s_9650	0.385892591	1.013324382	3.549260993	1.48833782	NA	NA
ASV_16s_9651	0.343641879	0.073466211	3.551127652	0.674823619	NA	NA
ASV_16s_9652	0.351674207	-0.06897574	3.549892574	1.730742861	NA	NA
ASV_16s_9653	0.353160762	-0.068890297	3.549889259	1.727960183	NA	NA
ASV_16s_9654	0.434469281	0.804604477	3.550162527	1.479595085	NA	NA
ASV_16s_9655	0.346198159	0.369799921	3.551233008	0.84975716	NA	NA
ASV_16s_9656	0.304628144	0.235207151	3.550623749	1.140084056	NA	NA
ASV_16s_9657	0.355396338	-0.11210333	3.55058272	1.375768925	NA	NA
ASV_16s_9658	0.232269302	0.279715971	3.551474687	0.743822592	NA	NA
ASV_16s_9659	0.584344485	-0.144658589	3.549512235	1.81683902	0.969279022	NA
ASV_16s_9660	0.499889978	0.87477625	3.550466414	1.275923823	0.989072445	NA
ASV_16s_9661	0.439460614	-0.549598739	3.54944303	0.855204307	NA	NA
ASV_16s_9662	0.436681919	-0.022572332	3.550641346	1.434701642	0.984484598	NA
ASV_16s_9663	0.405684964	0.027207733	3.550705002	1.351441765	0.987012355	NA
ASV_16s_9664	0.54054071	0.12289969	3.550092216	2.202884417	NA	NA
ASV_16s_9665	0.638778036	0.67226793	3.549616454	2.289266632	0.94211289	NA
ASV_16s_9666	0.616042786	0.148767613	3.549090526	1.917082849	0.964286652	NA
ASV_16s_9667	0.408050782	0.363300227	3.550461441	0.979678302	0.995149726	NA
ASV_16s_9668	0.37149494	0.36039128	3.550867507	1.292529773	NA	NA
ASV_16s_9669	0.37149494	0.36039128	3.550867507	1.292529773	NA	NA
ASV_16s_9670	0.445209967	0.547791811	3.550679742	1.173049301	0.99153425	NA
ASV_16s_9671	0.513292494	0.7348864	3.550785484	1.39300429	NA	NA
ASV_16s_9672	0.484130925	0.693654092	3.550705622	1.322567428	0.987825828	NA
ASV_16s_9673	0.46964994	0.69363569	3.550710344	1.307118118	0.988247914	NA
ASV_16s_9674	0.490735883	0.693662943	3.550703563	1.329448084	NA	NA
ASV_16s_9675	0.440626781	0.693601743	3.550720728	1.274756671	0.98910258	NA
ASV_16s_9676	0.388012224	0.693525261	3.550743504	1.2102691	NA	NA
ASV_16s_9677	0.337440156	-0.093518125	3.549998008	1.597567901	NA	NA
ASV_16s_9678	0.338475832	-0.130486014	3.549964541	1.626048051	NA	NA
ASV_16s_9679	0.342197006	-0.16935395	3.54992759	1.652123566	0.976571355	NA
ASV_16s_9680	0.339511507	-0.166273904	3.549937778	1.644007176	NA	NA
ASV_16s_9681	0.330654694	-0.164489342	3.549967671	1.619060477	NA	NA
ASV_16s_9682	0.340029345	-0.183873641	3.549926596	1.649277407	NA	NA
ASV_16s_9683	0.358143585	-0.251217851	3.549848689	1.690109433	0.974989809	NA
ASV_16s_9684	0.350180381	-0.260805582	3.549869314	1.6651523	0.976035643	NA
ASV_16s_9685	0.367222075	-0.307960627	3.549813045	1.679456979	0.975439364	NA
ASV_16s_9686	0.35767138	-0.300788165	3.549840036	1.660722069	0.976218593	NA
ASV_16s_9687	0.354699086	-0.307115646	3.549847488	1.648259275	NA	NA
ASV_16s_9688	0.335332345	-0.356806484	3.549903848	1.550790366	0.980499024	NA
ASV_16s_9689	0.340154834	-0.379510024	3.54989073	1.536447503	0.981020955	NA
ASV_16s_9690	0.094775647	-0.169427302	3.550786863	0.463273952	0.999570156	NA
ASV_16s_9691	0.125313078	0.220107158	3.550807422	0.556117063	0.99921396	NA
ASV_16s_9692	0.34125456	0.2644133	3.550037391	1.463172805	NA	NA
ASV_16s_9693	0.356091751	0.021242975	3.549761162	1.640232372	0.977054162	NA
ASV_16s_9694	0.334911656	-0.383375593	3.549681947	0.939771519	NA	NA
ASV_16s_9695	0.517844238	2.114036159	3.549039672	0.500990339	0.999442847	NA
ASV_16s_9696	0.339240848	0.165903355	3.549294767	1.755859582	0.972110538	NA
ASV_16s_9697	0.398018964	-0.459559269	3.549433283	1.357429708	NA	NA
ASV_16s_9698	0.428707198	-0.791366683	3.549264336	1.403190872	NA	NA
ASV_16s_9699	0.453677692	-0.791234086	3.549209296	1.526632011	0.981373311	NA

ASV_16s_9700	0.444704107	-0.788509652	3.549320855	1.553812	NA	NA
ASV_16s_9701	0.430620091	-0.493338146	3.550205719	1.10778715	0.992897497	NA
ASV_16s_9702	0.439761965	-0.839278781	3.54938566	1.317802879	0.987956974	NA
ASV_16s_9703	0.406656802	-0.755120773	3.549586413	1.528841845	0.981294324	NA
ASV_16s_9704	0.401403896	-0.92888378	3.54958046	1.187569109	0.991210305	NA
ASV_16s_9705	0.43020408	-0.729847367	3.549642931	1.600865506	0.978610884	NA
ASV_16s_9706	0.424735556	-0.705663008	3.550447631	0.831239897	NA	NA
ASV_16s_9707	0.434867992	-0.790762496	3.549544518	1.325420578	0.987746877	NA
ASV_16s_9708	0.448688486	-0.740192856	3.549590791	1.6353452	0.977250896	NA
ASV_16s_9709	0.435036078	-0.72281588	3.549657902	1.612983197	0.978138507	NA
ASV_16s_9710	0.435308525	-0.707520552	3.549701812	1.614477114	0.978079852	NA
ASV_16s_9711	0.377116027	1.007808859	3.548690666	0.785900202	NA	NA
ASV_16s_9712	0.413737898	-0.601354934	3.549989016	1.565816382	NA	NA
ASV_16s_9713	0.410903188	-0.662610989	3.549665642	1.227654756	NA	NA
ASV_16s_9714	0.467542626	-0.178185074	3.550181176	1.728254513	0.973341321	NA
ASV_16s_9715	0.386182155	0.955370259	3.549708185	1.189989007	NA	NA
ASV_16s_9716	0.410933351	-1.030113769	3.549337536	1.270368707	NA	NA
ASV_16s_9717	0.364955176	1.555457126	3.549413164	0.781825697	NA	NA
ASV_16s_9718	0.354891915	-0.054051043	3.550712639	1.447176449	NA	NA
ASV_16s_9719	0.669465857	-0.61657304	3.549180089	1.207357136	NA	NA
ASV_16s_9720	0.316818863	-0.558397611	3.550051986	1.086268777	NA	NA
ASV_16s_9721	0.394222469	0.35740111	3.551151113	0.754740035	NA	NA
ASV_16s_9722	0.255847757	-0.139340867	3.550327109	1.048761505	0.994003131	NA
ASV_16s_9723	0.301663542	0.042631584	3.550100892	1.47054886	0.983311777	NA
ASV_16s_9724	0.343168459	-0.795172274	3.54931738	0.992868932	0.994942752	NA
ASV_16s_9725	0.404742221	0.292701903	3.551217783	0.345918215	0.999838214	NA
ASV_16s_9726	0.376778593	0.397201436	3.551217643	0.556906991	NA	NA
ASV_16s_9727	0.399494625	-0.56529643	3.549695353	0.895148578	NA	NA
ASV_16s_9728	0.364142786	-0.940418019	3.549649797	0.883707125	NA	NA
ASV_16s_9729	0.408254191	-0.56538287	3.549673735	0.902120338	NA	NA
ASV_16s_9730	0.266130615	1.301729679	3.55053578	0.67752655	NA	NA
ASV_16s_9731	0.174289306	0.380414875	3.551308887	0.560091013	NA	NA
ASV_16s_9732	0.231868797	0.220026661	3.550701448	0.851158299	0.996886141	NA
ASV_16s_9733	0.243311796	0.220022213	3.550695575	0.875349544	NA	NA
ASV_16s_9734	0.41080343	1.000077398	3.549831026	1.603424678	0.978511625	NA
ASV_16s_9735	0.423719356	1.880939972	3.55008334	0.623941111	0.998854354	NA
ASV_16s_9736	0.497128514	1.399335395	3.55051139	0.712480037	0.998237998	NA
ASV_16s_9737	0.341197561	-0.069177407	3.550019895	1.68240473	NA	NA
ASV_16s_9738	0.373564448	-0.069119055	3.549852481	1.791979301	0.970452109	NA
ASV_16s_9739	0.350116891	-0.06879299	3.549903074	1.710298969	NA	NA
ASV_16s_9740	0.350116891	-0.06879299	3.549903074	1.710298969	NA	NA
ASV_16s_9741	0.402752113	-6.179317782	3.547817687	-0.844235243	1	NA
ASV_16s_9742	0.330987694	1.339265794	3.550576001	0.650615923	NA	NA
ASV_16s_9743	0.274487305	0.509024216	3.550914298	1.021631109	NA	NA
ASV_16s_9744	0.499851597	1.219294364	3.550197644	0.92840079	NA	NA
ASV_16s_9745	0.458653831	0.359905287	3.550699543	0.730180178	NA	NA
ASV_16s_9746	0.494226033	0.36405198	3.550624454	0.797982964	0.997464483	NA
ASV_16s_9747	0.434101338	-0.549560311	3.549452348	0.851249384	NA	NA
ASV_16s_9748	0.341820511	-0.081928502	3.550422819	1.460371829	NA	NA
ASV_16s_9749	0.653566841	0.530425303	3.549529146	2.296469266	0.941628307	NA
ASV_16s_9750	0.420935217	0.36265404	3.550461089	0.99480435	0.994911915	NA
ASV_16s_9751	0.403642367	0.363416135	3.550399508	0.98075137	0.995133096	NA
ASV_16s_9752	0.477888973	0.338370427	3.550716502	1.140232846	0.992238559	NA
ASV_16s_9753	0.41628794	0.315939389	3.550759838	1.311279716	0.988135115	NA
ASV_16s_9754	0.421205044	0.315939388	3.550757827	1.317245449	0.98797226	NA
ASV_16s_9755	0.401758242	0.715071482	3.550475273	1.493887886	0.982520507	NA
ASV_16s_9756	0.460783216	0.315939382	3.550743218	1.362749308	0.986684996	NA
ASV_16s_9757	0.251161584	0.734133609	3.551003307	1.031240509	NA	NA
ASV_16s_9758	0.463713317	0.693631126	3.550712365	1.300645302	0.988422044	NA
ASV_16s_9759	0.562040261	0.693729391	3.550684406	1.398342171	NA	NA
ASV_16s_9760	0.168852944	1.091682043	3.55127829	0.447575047	0.999616703	NA
ASV_16s_9761	0.326460991	0.025862748	3.550180462	1.398642545	NA	NA
ASV_16s_9762	0.33206594	-0.047527588	3.550062887	1.534687571	NA	NA
ASV_16s_9763	0.326552256	-0.132598386	3.550003101	1.594280944	NA	NA
ASV_16s_9764	0.332905442	-0.175842262	3.54995373	1.628105379	NA	NA
ASV_16s_9765	0.326580776	-0.177272712	3.549974113	1.610686588	NA	NA
ASV_16s_9766	0.332444645	-0.244757033	3.54992742	1.623100424	0.977739481	NA

ASV_16s_9767	0.331690704	-0.252798869	3.549927871	1.618197106	0.977933396	NA
ASV_16s_9768	0.354934742	-0.307283241	3.549846826	1.648683037	0.976711649	NA
ASV_16s_9769	0.339986857	-0.322608465	3.549889521	1.596784965	0.978768594	NA
ASV_16s_9770	0.332237062	-0.316608714	3.549914498	1.581171852	0.979365714	NA
ASV_16s_9771	0.331742041	-0.335043195	3.549915079	1.563476912	0.980030372	NA
ASV_16s_9772	0.354115396	-0.369309358	3.549847788	1.584212731	0.979250201	NA
ASV_16s_9773	0.366861243	-0.434234487	3.549824054	1.506190978	0.982094554	NA
ASV_16s_9774	0.116673247	-0.464256799	3.551286955	0.511056422	NA	NA
ASV_16s_9775	0.47000973	-0.682672749	3.549684299	0.969890743	NA	NA
ASV_16s_9776	0.326826051	-0.680832486	3.550096708	0.853069925	NA	NA
ASV_16s_9777	0.054120921	0.588012567	3.551969716	0.177302855	0.999983352	NA
ASV_16s_9778	0.062739207	-0.168860611	3.550929128	0.309949481	NA	NA
ASV_16s_9779	0.110819515	-0.169589347	3.55074637	0.528700794	NA	NA
ASV_16s_9781	0.355502755	0.007332286	3.549819372	1.6445215	0.976880689	NA
ASV_16s_9782	0.299060455	-0.209458571	3.549384521	1.11443993	0.992765384	NA
ASV_16s_9783	0.35959496	-0.017903938	3.549303497	1.622093617	0.977779379	NA
ASV_16s_9784	0.393086922	-0.502031056	3.549599644	1.449305017	0.984013011	NA
ASV_16s_9785	0.363579401	-0.062251804	3.549320742	1.571062126	0.979747026	NA
ASV_16s_9786	0.342817657	0.177091631	3.54936333	1.783111736	0.970864306	NA
ASV_16s_9787	0.340433461	0.840151251	3.549606942	1.380675248	0.986155823	NA
ASV_16s_9788	0.37943489	-0.526119997	3.549236855	1.11073424	0.992839163	NA
ASV_16s_9789	0.365068864	-0.122973893	3.549687658	1.32383264	NA	NA
ASV_16s_9790	0.353186498	0.142277931	3.549264598	1.848371742	NA	NA
ASV_16s_9791	0.433865453	-0.789265197	3.549331123	1.515923265	0.981753271	NA
ASV_16s_9792	0.404539642	-0.782810289	3.549445214	1.457219312	NA	NA
ASV_16s_9793	0.437737582	-0.781417008	3.549389609	1.561195103	NA	NA
ASV_16s_9794	0.438209688	-0.7784226	3.54941104	1.570828951	0.979755771	NA
ASV_16s_9795	0.272086313	1.132572855	3.550729195	0.854690012	NA	NA
ASV_16s_9796	0.413574204	-0.741379148	3.549622155	1.557345643	0.980257687	NA
ASV_16s_9797	0.416268732	-0.753947671	3.549578207	1.554135906	0.980376075	NA
ASV_16s_9798	0.416266667	-0.727298466	3.549663583	1.570847003	0.979755094	NA
ASV_16s_9799	0.392681996	-1.261527288	3.549504922	0.691929064	0.998396984	NA
ASV_16s_9800	0.376114756	-0.822761417	3.549924777	1.27102088	0.989198692	NA
ASV_16s_9801	0.39302605	-0.760928896	3.549000497	1.120269	NA	NA
ASV_16s_9802	0.42007889	0.678367906	3.550761422	1.381326361	0.986136366	NA
ASV_16s_9803	0.411040517	0.218975333	3.549421693	1.44701181	NA	NA
ASV_16s_9804	0.376400487	0.074818925	3.550710168	1.517989048	NA	NA
ASV_16s_9805	0.355622328	-0.074579136	3.550345705	1.426108502	NA	NA
ASV_16s_9806	0.296849628	1.835857544	3.550430164	0.443423854	0.999628408	NA
ASV_16s_9807	0.272574446	0.48592002	3.549639403	0.855000003	NA	NA
ASV_16s_9808	0.274445284	0.393143338	3.551067556	0.778152681	NA	NA
ASV_16s_9809	0.274445284	0.393143338	3.551067556	0.778152681	NA	NA
ASV_16s_9810	0.477711699	-0.565958359	3.549530208	0.952635888	NA	NA
ASV_16s_9811	0.459201457	-0.565822009	3.549564251	0.939922912	NA	NA
ASV_16s_9812	0.329470851	0.630059545	3.550108265	1.256931275	NA	NA
ASV_16s_9813	0.117723611	0.355235682	3.551612875	0.435579775	NA	NA
ASV_16s_9814	0.117723611	0.355235682	3.551612875	0.435579775	NA	NA
ASV_16s_9815	0.117723611	0.355235682	3.551612875	0.435579775	NA	NA
ASV_16s_9816	0.189958798	-0.537317605	3.550406626	0.587585029	NA	NA
ASV_16s_9817	0.292999033	-0.822989584	3.549888038	0.911542085	NA	NA
ASV_16s_9818	0.22533639	0.220029427	3.550705069	0.836821112	0.997049771	NA
ASV_16s_9819	0.22863746	0.220027914	3.550703214	0.844106895	0.99696734	NA
ASV_16s_9820	0.233112413	0.220026172	3.550700783	0.853826453	0.996855049	NA
ASV_16s_9821	0.345266662	-0.446819231	3.549744952	1.009656763	0.994671252	NA
ASV_16s_9822	0.315191697	0.663630378	3.550027648	1.393754072	NA	NA
ASV_16s_9823	0.319269222	0.695902111	3.551073763	0.709081223	NA	NA
ASV_16s_9824	0.343162009	1.259927947	3.550476323	1.047711073	NA	NA
ASV_16s_9825	0.348823506	0.700450031	3.549630767	1.283261829	NA	NA
ASV_16s_9826	0.342613355	-0.068968149	3.54991549	1.706747427	NA	NA
ASV_16s_9827	0.624474094	-0.723291136	3.549079663	1.449458661	NA	NA
ASV_16s_9828	0.315296932	-0.09415571	3.549870833	1.317570903	NA	NA
ASV_16s_9829	0.310459799	-0.05415394	3.550488317	1.330860828	NA	NA
ASV_16s_9830	0.491732032	0.713121268	3.549824948	1.847594648	NA	NA
ASV_16s_9831	0.097639776	0.284733396	3.551752412	0.446443534	NA	NA
ASV_16s_9832	0.439010171	1.387844308	3.549790799	1.567717635	NA	NA
ASV_16s_9833	0.270894943	-0.078956102	3.550716287	1.089338608	NA	NA
ASV_16s_9834	0.358729195	-0.030220611	3.550747669	1.345610154	NA	NA

ASV_16s_9835	0.405793682	0.255171884	3.550741391	2.235504797	NA	NA
ASV_16s_9836	0.4543018	0.663863747	3.549779962	1.836144109	NA	NA
ASV_16s_9837	0.405083886	0.315939409	3.550764602	1.297419462	0.988508228	NA
ASV_16s_9838	0.43168326	0.315939351	3.550753702	1.329671539	0.987628667	NA
ASV_16s_9839	0.390183163	0.315939413	3.550771356	1.278408443	0.989008123	NA
ASV_16s_9840	0.512061093	1.068762704	3.55069947	1.14768298	NA	NA
ASV_16s_9841	0.500773165	0.734868401	3.550790725	1.380430343	NA	NA
ASV_16s_9842	0.442329684	0.693604771	3.550720085	1.276687129	0.989052709	NA
ASV_16s_9843	0.454594976	0.693620385	3.550715567	1.290571468	0.988689875	NA
ASV_16s_9844	0.422672625	0.69357787	3.550727872	1.253599971	0.989639958	NA
ASV_16s_9845	0.375862226	-0.142455492	3.549847527	1.721568641	NA	NA
ASV_16s_9846	0.346423992	-0.190741032	3.549903654	1.666489736	NA	NA
ASV_16s_9847	0.322495451	-0.172423243	3.549990991	1.598008069	NA	NA
ASV_16s_9848	0.322518267	-0.20818442	3.549973061	1.601548996	NA	NA
ASV_16s_9849	0.332646858	-0.218852566	3.549934941	1.629310903	NA	NA
ASV_16s_9850	0.387540792	-0.274930813	3.549768313	1.752665882	0.972254556	NA
ASV_16s_9851	0.332905777	-0.227791876	3.549930991	1.628645262	NA	NA
ASV_16s_9852	0.346481033	-0.274538578	3.549877538	1.64827675	0.976728184	NA
ASV_16s_9853	0.322552491	-0.26210991	3.549956376	1.588669394	NA	NA
ASV_16s_9854	0.328658166	-0.313740635	3.549926496	1.573721178	0.979647139	NA
ASV_16s_9855	0.329941352	-0.34327442	3.549920954	1.550605716	0.980505797	NA
ASV_16s_9856	0.342863381	-0.361945609	3.549880672	1.564445111	0.979994336	NA
ASV_16s_9857	0.115214832	-0.459958099	3.551298706	0.507063987	NA	NA
ASV_16s_9858	0.115214832	-0.459958099	3.551298706	0.507063987	NA	NA
ASV_16s_9859	0.115214832	-0.459958099	3.551298706	0.507063987	NA	NA
ASV_16s_9860	0.322740725	-0.680749957	3.550113814	0.849019195	NA	NA
ASV_16s_9862	0.336228306	-0.210558495	3.550126364	1.379694594	0.986185097	NA
ASV_16s_9863	0.359113971	0.05407624	3.549438755	1.674177178	0.975660436	NA
ASV_16s_9864	0.328241109	-0.349688135	3.549574931	1.002067936	NA	NA
ASV_16s_9865	0.3319396	-0.057842272	3.549327726	1.521429209	NA	NA
ASV_16s_9866	0.390925305	-0.583465752	3.549566148	1.422151711	NA	NA
ASV_16s_9867	0.337759274	-0.24810644	3.549670422	1.075020646	NA	NA
ASV_16s_9868	0.445982159	-0.791669069	3.549188851	1.432267895	0.984562366	NA
ASV_16s_9869	0.411576873	-0.791079919	3.549344015	1.378204438	0.986229507	NA
ASV_16s_9870	0.383083086	0.395495829	3.548988843	1.478590137	0.983041625	NA
ASV_16s_9871	0.440879988	-0.790810703	3.549292915	1.522513803	0.981519981	NA
ASV_16s_9872	0.426898756	-0.78771362	3.549364658	1.505933932	0.982103516	NA
ASV_16s_9873	0.399244063	0.491738993	3.549082958	1.784705452	0.970790467	NA
ASV_16s_9874	0.340269391	-0.232656333	3.550347951	1.289387191	NA	NA
ASV_16s_9875	0.456359779	-0.788896998	3.549295775	1.583553126	0.979275289	NA
ASV_16s_9876	0.402501194	-0.767988428	3.549523608	1.494530613	0.982498406	NA
ASV_16s_9877	0.395233745	-0.777277185	3.549643943	1.506381389	0.982087914	NA
ASV_16s_9878	0.424071801	-0.743976363	3.549601574	1.580407934	0.979394673	NA
ASV_16s_9879	0.384657123	1.903855211	3.549625739	0.801479802	NA	NA
ASV_16s_9880	0.237304208	0.216153961	3.549973336	0.788768757	NA	NA
ASV_16s_9881	0.347593514	-0.318865728	3.550362847	1.35474533	NA	NA
ASV_16s_9882	0.386584131	-0.040672247	3.550290071	1.374125904	0.986350614	NA
ASV_16s_9883	0.436775649	-0.677990239	3.549792091	1.613303382	0.978125944	NA
ASV_16s_9884	0.31850525	-1.364667675	3.549331121	0.726457498	0.998124088	NA
ASV_16s_9885	0.41316122	0.182696631	3.549430672	1.592589407	NA	NA
ASV_16s_9886	0.436164465	-0.649057087	3.549859759	1.611251513	0.978206383	NA
ASV_16s_9887	0.427413153	-0.578002015	3.550034271	1.592005818	0.978952434	NA
ASV_16s_9888	0.397920694	0.34397227	3.55115112	0.83981597	NA	NA
ASV_16s_9889	0.394792779	0.207666542	3.54931271	1.63387248	0.977309987	NA
ASV_16s_9890	0.360631323	-0.08106978	3.550436605	1.374296056	NA	NA
ASV_16s_9891	0.429650079	1.353798157	3.549269366	0.72061997	NA	NA
ASV_16s_9892	0.494089398	0.345848954	3.550124617	1.630412614	0.977448458	NA
ASV_16s_9893	0.363954165	0.772783463	3.54961645	0.87794459	NA	NA
ASV_16s_9894	0.521209757	1.522937789	3.549450893	1.302912976	NA	NA
ASV_16s_9895	0.271014718	0.392515687	3.551088262	0.773975918	NA	NA
ASV_16s_9896	0.5415868	-0.566357983	3.549430512	0.99301206	NA	NA
ASV_16s_9897	0.38963056	-0.564838136	3.549720852	0.887045454	NA	NA
ASV_16s_9898	0.22022778	-0.726593476	3.550677514	0.746494563	NA	NA
ASV_16s_9899	0.209437747	1.24994003	3.550748447	0.594757537	NA	NA
ASV_16s_9900	0.434575537	0.65209671	3.549516561	1.616556287	0.977998066	NA
ASV_16s_9901	0.354717141	0.448161186	3.549056866	1.316280037	0.987998707	NA
ASV_16s_9902	0.382101619	-0.02012784	3.549591042	1.664921308	NA	NA

ASV_16s_9903	0.116252066	0.354491121	3.551619528	0.431782802	NA	NA
ASV_16s_9904	0.373816655	-0.079143741	3.550660783	1.252209743	NA	NA
ASV_16s_9905	0.227101419	0.220028577	3.550704072	0.840713396	0.997005919	NA
ASV_16s_9906	0.36194137	0.721578611	3.551222961	0.628732517	NA	NA
ASV_16s_9907	0.411826289	-1.328390621	3.549315147	0.820400217	NA	NA
ASV_16s_9908	0.321730824	-0.066774213	3.55062613	1.275736295	NA	NA
ASV_16s_9909	0.407607745	0.189934362	3.549560979	1.306471616	0.988265377	NA
ASV_16s_9910	0.334118782	-0.069122539	3.549998837	1.67345508	NA	NA
ASV_16s_9911	0.336100855	-0.069037956	3.549953591	1.688086098	NA	NA
ASV_16s_9912	0.472119122	2.245377908	3.549121065	0.402964306	0.999729973	NA
ASV_16s_9913	0.434189822	0.060434094	3.549630737	2.514196962	0.926024231	NA
ASV_16s_9914	0.507038554	0.068096983	3.549028049	1.976925853	NA	NA
ASV_16s_9915	0.347289975	0.388108104	3.551062411	0.83520592	NA	NA
ASV_16s_9916	0.558255967	0.299136359	3.547905001	1.588120237	0.97910121	NA
ASV_16s_9917	0.193771244	0.279461386	3.551556725	0.677868547	NA	NA
ASV_16s_9918	0.223771645	0.279663084	3.551490965	0.73013931	NA	NA
ASV_16s_9919	0.628952632	0.21696583	3.548069704	1.538694185	0.980939753	NA
ASV_16s_9920	0.410369168	0.321581589	3.55080949	1.016889749	0.994551461	NA
ASV_16s_9921	0.483667984	2.085355873	3.549376648	0.514226279	NA	NA
ASV_16s_9922	0.446511946	1.28384061	3.54975892	1.553084577	0.980414761	NA
ASV_16s_9923	0.413745569	1.100228	3.550159911	1.428399787	0.984685483	NA
ASV_16s_9924	0.352940309	-0.029296561	3.550749606	1.282302737	NA	NA
ASV_16s_9925	0.357903661	0.360388121	3.550877882	1.273575094	NA	NA
ASV_16s_9926	0.432771776	0.315939365	3.550753289	1.330917517	0.987593887	NA
ASV_16s_9927	0.418233833	0.315939355	3.550759044	1.313587475	0.988072278	NA
ASV_16s_9928	0.418010622	0.315939341	3.550759136	1.313317389	0.988079643	NA
ASV_16s_9929	0.384082524	0.31593943	3.550774275	1.270396107	0.989214714	NA
ASV_16s_9930	0.458917996	0.315939267	3.550743854	1.360654991	0.986746002	NA
ASV_16s_9931	0.468488909	0.693637361	3.550710737	1.305845473	0.988282276	NA
ASV_16s_9932	0.438738088	0.693600315	3.55072146	1.272513489	0.989160354	NA
ASV_16s_9933	0.49702092	0.693666598	3.550701662	1.335836106	0.987456006	NA
ASV_16s_9934	0.355423714	-0.117947797	3.549921406	1.652399799	NA	NA
ASV_16s_9935	0.32266344	-0.089976261	3.550053047	1.554109963	NA	NA
ASV_16s_9936	0.318421533	-0.185600725	3.549998181	1.588624389	NA	NA
ASV_16s_9937	0.324532912	-0.245982308	3.549953364	1.600372799	0.978629963	NA
ASV_16s_9938	0.318495686	-0.305317767	3.549962116	1.55193603	NA	NA
ASV_16s_9939	0.336430468	-0.347967484	3.549900109	1.562967271	0.980049326	NA
ASV_16s_9940	0.365547888	-0.394835086	3.549819	1.578191888	0.979478546	NA
ASV_16s_9941	0.322834562	-0.367873114	3.54994639	1.505114867	0.982132055	NA
ASV_16s_9942	0.413530968	-0.437085672	3.549710854	1.610916045	0.978219518	NA
ASV_16s_9943	0.3186554	-0.680671422	3.550131358	0.844917978	NA	NA
ASV_16s_9944	0.090053995	-0.169369616	3.550801503	0.442759914	NA	NA
ASV_16s_9945	0.206262832	0.22003849	3.550716949	0.792688176	NA	NA
ASV_16s_9946	0.125269642	0.321788999	3.550781873	0.825457782	NA	NA
ASV_16s_9947	0.354438669	0.226125974	3.549655747	1.68916307	NA	NA
ASV_16s_9948	0.363360286	0.049414182	3.549302115	1.712609859	0.974024747	NA
ASV_16s_9949	0.404518831	-0.695683088	3.549967753	1.027801117	0.994367512	NA
ASV_16s_9950	0.326558989	0.010390404	3.549325896	1.604435567	NA	NA
ASV_16s_9951	0.333287865	0.834189683	3.549651614	1.397875935	NA	NA
ASV_16s_9952	0.397559364	-0.791208768	3.549426015	1.276245535	0.98906413	NA
ASV_16s_9953	0.410632204	-0.790995164	3.549352409	1.379460089	NA	NA
ASV_16s_9954	0.338908098	0.264574336	3.549369673	1.796875452	NA	NA
ASV_16s_9955	0.371707287	-0.314339466	3.549291712	1.355577477	NA	NA
ASV_16s_9956	0.3630446	-0.070665875	3.549178613	1.667613673	NA	NA
ASV_16s_9957	0.377606151	0.043920586	3.549164061	1.850343145	0.967657159	NA
ASV_16s_9958	0.46091655	2.067315077	3.549235809	0.473559647	0.999537652	NA
ASV_16s_9959	0.420847428	-0.782876627	3.549408639	1.507638569	0.982044034	NA
ASV_16s_9960	0.417904739	-0.772082957	3.549475572	1.529209104	0.981281178	NA
ASV_16s_9961	0.246257119	0.001138364	3.549128836	1.112583964	NA	NA
ASV_16s_9962	0.411528159	-0.774588683	3.549620029	1.552246072	0.980445583	NA
ASV_16s_9963	0.404775213	-0.737843366	3.549910477	1.161082323	0.991795538	NA
ASV_16s_9964	0.409207448	0.19011616	3.54954259	1.611437773	NA	NA
ASV_16s_9965	0.353551789	-0.623489752	3.550837289	0.823404869	NA	NA
ASV_16s_9966	0.415172666	-0.009133363	3.549750504	1.759954439	0.971925261	NA
ASV_16s_9967	0.417604089	-0.663406411	3.549839067	1.578618386	0.979462419	NA
ASV_16s_9968	0.363219596	0.683252112	3.550738767	1.384685663	0.986035716	NA
ASV_16s_9969	0.460213284	-0.477581543	3.32543305	1.640340567	0.977049795	NA

ASV_16s_9970	0.336128726	-0.771968938	3.550031534	1.125244224	0.992547544	NA
ASV_16s_9971	0.396483024	-0.473159806	3.550274166	1.46717835	0.98342424	NA
ASV_16s_9972	0.407181254	0.242725062	3.549474598	1.567716794	NA	NA
ASV_16s_9973	0.383888403	0.182165256	3.54959359	1.331883336	0.987566886	NA
ASV_16s_9974	0.377746653	1.075090839	3.550084315	0.863126752	0.996745092	NA
ASV_16s_9975	0.351594342	0.219093197	3.549322803	1.50159686	0.982254325	NA
ASV_16s_9976	0.370159166	-0.072453753	3.550379614	1.204619537	NA	NA
ASV_16s_9977	0.451125716	0.054325727	3.549862643	1.613561949	0.978115795	NA
ASV_16s_9978	0.391709455	0.496884112	3.549349476	1.658018376	NA	NA
ASV_16s_9979	0.345296486	0.403212399	3.550715597	0.853920167	NA	NA
ASV_16s_9980	0.379275886	0.020445187	3.549820075	1.680562884	NA	NA
ASV_16s_9981	0.301057406	0.249537009	3.550290102	1.08922413	NA	NA
ASV_16s_9982	0.373883987	0.959296511	3.550591405	1.000808501	0.994815484	NA
ASV_16s_9983	0.282400747	-0.018607244	3.54970486	0.919222144	NA	NA
ASV_16s_9984	0.262694288	1.744903156	3.550306312	0.431664735	NA	NA
ASV_16s_9985	0.354505141	1.335915602	3.550235405	0.773609264	NA	NA
ASV_16s_9986	0.329647706	0.418854117	3.550442743	0.769767055	NA	NA
ASV_16s_9987	0.224933088	-0.545019741	3.550154225	0.641046871	NA	NA
ASV_16s_9988	0.321968392	-0.079063663	3.550684324	1.176548972	0.991456866	NA
ASV_16s_9989	0.222168333	0.220030846	3.550706904	0.829704106	0.997128864	NA
ASV_16s_9990	0.234300248	0.220025727	3.550700157	0.856346829	NA	NA
ASV_16s_9991	0.325596749	0.218365277	3.549314982	1.536935713	NA	NA
ASV_16s_9992	0.367827408	0.619548309	3.549864529	1.439006894	NA	NA
ASV_16s_9993	0.346435979	-0.060855956	3.550329612	1.485826294	NA	NA
ASV_16s_9994	0.265131766	0.492214413	3.550914749	0.99154052	NA	NA
ASV_16s_9995	0.60899021	-0.202239734	3.548411792	1.711434245	NA	NA
ASV_16s_9996	0.362412596	-0.081909366	3.550463703	1.216948566	NA	NA
ASV_16s_9997	0.551418396	0.859435825	3.550537397	1.509878935	0.98196568	NA
ASV_16s_9998	0.420794925	0.770573971	3.549968735	1.851226673	0.967613754	NA
ASV_16s_9999	0.4282184	0.34466526	3.55068373	1.051351128	0.993957098	NA
ASV_16s_10000	0.395052661	0.770636584	3.550037041	1.788225523	0.970626999	NA
ASV_16s_10001	0.411197908	0.315939435	3.550761976	1.304984145	0.988305498	NA
ASV_16s_10002	0.378666276	0.315939447	3.550776946	1.263173939	0.989398861	NA
ASV_16s_10003	0.378666276	0.315939447	3.550776946	1.263173939	0.989398861	NA
ASV_16s_10004	0.395144475	0.315939295	3.550769059	1.284752754	0.988842821	NA
ASV_16s_10005	0.531524229	1.180354214	3.549940844	1.026293552	NA	NA
ASV_16s_10006	0.241859303	0.734076717	3.551019663	1.012311498	NA	NA
ASV_16s_10007	0.241859303	0.734076717	3.551019663	1.012311498	NA	NA
ASV_16s_10008	0.460687961	0.693628327	3.550713416	1.297304959	0.98851128	NA
ASV_16s_10009	0.369084799	0.693492192	3.550753291	1.184849674	NA	NA
ASV_16s_10010	0.319309238	-0.034886034	3.55012541	1.477157953	NA	NA
ASV_16s_10011	0.321380589	-0.116270168	3.550034426	1.56992309	NA	NA
ASV_16s_10012	0.314330503	-0.171552206	3.55002091	1.574156794	NA	NA
ASV_16s_10013	0.339109954	-0.331132967	3.549891882	1.586592957	0.979159519	NA
ASV_16s_10014	0.314427473	-0.331335623	3.549974637	1.519364181	NA	NA
ASV_16s_10015	0.317719265	-0.333945561	3.549962795	1.526166726	0.981389917	NA
ASV_16s_10016	0.332998261	-0.219160144	3.549613439	1.199404501	0.990940603	NA
ASV_16s_10017	0.321073801	-0.454321598	3.549975605	1.346884337	0.987142892	NA
ASV_16s_10018	0.112298001	-0.451208914	3.551322909	0.499013691	NA	NA
ASV_16s_10019	0.314570074	-0.680599554	3.550149355	0.840765404	NA	NA
ASV_16s_10020	0.088899456	-0.169354457	3.550805323	0.437621176	NA	NA
ASV_16s_10021	0.34121684	-0.014563805	3.549870997	1.611130746	0.978211112	NA
ASV_16s_10022	0.335567063	-0.084621469	3.549858354	1.519937317	0.981611393	NA
ASV_16s_10023	0.329269487	0.019520058	3.549917175	1.50854446	0.982012376	NA
ASV_16s_10024	0.331680486	-0.022618233	3.549795792	1.565027416	0.979972644	NA
ASV_16s_10025	0.342635127	0.091534075	3.549590814	1.639307071	0.977091486	NA
ASV_16s_10026	0.336635338	0.046564468	3.549612867	1.607265484	0.978362151	NA
ASV_16s_10027	0.436552127	-0.787863245	3.549370701	1.182873276	0.991315905	NA
ASV_16s_10028	0.345977141	-0.715969718	3.549794143	1.347830997	0.987115843	NA
ASV_16s_10029	0.334129878	0.526665853	3.549378691	1.513805768	0.981827852	NA
ASV_16s_10030	0.422648776	-0.791676305	3.549307654	1.297060718	0.988517788	NA
ASV_16s_10031	0.433826863	-0.771275863	3.548986626	0.985856014	NA	NA
ASV_16s_10032	0.353903653	-0.137786972	3.549203524	1.543558264	0.980763245	NA
ASV_16s_10033	0.381873817	-0.611630875	3.549506341	1.425709533	NA	NA
ASV_16s_10034	0.421204002	-0.789983634	3.549352262	1.468890927	0.983367154	NA
ASV_16s_10035	0.371059147	0.172584372	3.549273516	1.898015822	NA	NA
ASV_16s_10036	0.356983303	1.033679797	3.549670719	0.683642387	NA	NA

ASV_16s_10037	0.414260299	-0.75888474	3.54955746	1.54333738	0.980771281	NA
ASV_16s_10038	0.394225116	-0.748695094	3.549625673	1.502186288	0.982233874	NA
ASV_16s_10039	0.404004783	-0.744153146	3.549625315	1.531616316	NA	NA
ASV_16s_10040	0.376307312	-0.232839615	3.550269909	1.2475724	0.98978999	NA
ASV_16s_10041	0.401918356	-0.717111188	3.549706334	1.540725195	0.980866168	NA
ASV_16s_10042	0.404646828	-0.701664021	3.549746641	1.550371298	0.980514393	NA
ASV_16s_10043	0.405031506	-0.727369037	3.548535514	1.206772435	0.990770127	NA
ASV_16s_10044	0.443476958	0.315972405	3.549763941	1.92407666	NA	NA
ASV_16s_10045	0.389178642	-0.673230922	3.549842048	1.522730289	0.981512288	NA
ASV_16s_10046	0.372322462	-0.159579926	3.550448199	1.257947511	NA	NA
ASV_16s_10047	0.409710713	-0.283587478	3.549546464	1.690880735	0.974957076	NA
ASV_16s_10048	0.3738056	0.26335375	3.549749211	1.698316959	0.97464022	NA
ASV_16s_10049	0.285884227	-0.595369894	3.550443662	0.763092168	NA	NA
ASV_16s_10050	0.450198563	-0.416702899	3.549869488	1.150074875	NA	NA
ASV_16s_10051	0.337397043	0.374177242	3.551363623	0.65103122	NA	NA
ASV_16s_10052	0.506932326	1.183239768	3.550025285	1.075758205	0.99351216	NA
ASV_16s_10053	0.355143254	-0.076164855	3.550364526	1.400887329	0.985544071	NA
ASV_16s_10054	0.421218515	0.161844877	3.549928912	1.54097819	0.98085699	NA
ASV_16s_10055	0.469652037	1.536256265	3.549614076	1.226465661	NA	NA
ASV_16s_10056	0.401191685	0.611022745	3.549520466	1.975899972	0.961162186	NA
ASV_16s_10057	0.340869608	0.403071627	3.550737532	0.849730092	NA	NA
ASV_16s_10058	0.316446791	-0.429416438	3.55041375	1.101390344	NA	NA
ASV_16s_10059	0.303781383	-0.070662863	3.550294999	0.822645675	0.99720592	NA
ASV_16s_10060	0.279449935	-0.122981436	3.550639865	1.001750884	NA	NA
ASV_16s_10061	0.434519161	-0.579339781	3.549504346	1.219138322	NA	NA
ASV_16s_10062	0.241431489	-0.488481076	3.550475046	1.007777561	NA	NA
ASV_16s_10063	0.113308976	0.35297509	3.551633224	0.424138096	NA	NA
ASV_16s_10064	0.113308976	0.35297509	3.551633224	0.424138096	NA	NA
ASV_16s_10065	0.113308976	0.35297509	3.551633224	0.424138096	NA	NA
ASV_16s_10066	0.182835343	-0.534282154	3.550466691	0.575401837	NA	NA
ASV_16s_10067	0.16132469	1.178525558	3.550962001	0.505728856	NA	NA
ASV_16s_10068	0.364352942	-0.079131179	3.550664585	1.239176318	NA	NA
ASV_16s_10069	0.310687898	-0.079042918	3.550690485	1.158489225	NA	NA
ASV_16s_10070	0.220584304	0.220031455	3.550707841	0.826109347	0.997168279	NA
ASV_16s_10071	0.231296398	0.220026956	3.550701763	0.849857897	NA	NA
ASV_16s_10072	0.345455034	-0.051793186	3.549832568	1.634728769	0.977275641	NA
ASV_16s_10073	0.316744236	-0.066833621	3.550618134	1.292214286	0.988646461	NA
ASV_16s_10074	0.483123102	0.690796343	3.550413202	1.277667258	0.989027335	NA
ASV_16s_10075	0.269136681	-0.764819352	3.550777835	0.834154199	NA	NA
ASV_16s_10076	0.32803104	-0.068998591	3.549968147	1.665532517	NA	NA
ASV_16s_10077	0.328526558	-0.068979313	3.549960708	1.66741864	NA	NA
ASV_16s_10078	0.33100415	-0.068861236	3.549941092	1.669086815	NA	NA
ASV_16s_10079	0.367639113	0.365771278	3.549820435	1.619427212	NA	NA
ASV_16s_10080	0.285192685	0.78043802	3.55102381	1.225399324	NA	NA
ASV_16s_10081	0.290061721	0.750810019	3.551108653	1.204178397	NA	NA
ASV_16s_10082	0.273970189	0.279453537	3.551390427	0.803994776	NA	NA
ASV_16s_10083	0.412664235	-0.549396984	3.549492029	0.834927336	NA	NA
ASV_16s_10084	0.407965012	1.180799822	3.550108917	1.179184506	NA	NA
ASV_16s_10085	0.321106932	-0.33088744	3.550590253	0.967043744	NA	NA
ASV_16s_10086	0.451135343	0.559520253	3.550697742	1.867363665	NA	NA
ASV_16s_10087	0.380741185	0.342740469	3.549824075	1.754275601	NA	NA
ASV_16s_10088	0.345221275	0.340028112	3.549952827	1.665186458	0.976034229	NA
ASV_16s_10089	0.416852259	0.721726993	3.549317723	1.522735789	NA	NA
ASV_16s_10090	0.41995874	0.348515652	3.550653652	1.025328136	0.994409545	NA
ASV_16s_10091	0.412787391	0.348516173	3.55066054	1.017430199	0.994542441	NA
ASV_16s_10092	0.348842809	0.360385797	3.550885245	1.26053631	NA	NA
ASV_16s_10093	0.451279266	0.315939317	3.550746501	1.352116224	0.986992968	NA
ASV_16s_10094	0.39772485	0.315939321	3.550767881	1.288063963	0.988755942	NA
ASV_16s_10095	0.388046526	0.315939322	3.550772375	1.275564272	0.989081734	NA
ASV_16s_10096	0.407520323	0.315939376	3.550763555	1.300379438	0.988429162	NA
ASV_16s_10097	0.404108547	0.464737098	3.550746749	2.174856241	0.949531728	NA
ASV_16s_10098	0.364352942	0.315939466	3.55078438	1.243612987	NA	NA
ASV_16s_10099	0.364352942	0.410084791	3.550767463	1.934584944	NA	NA
ASV_16s_10100	0.3469868	0.688285284	3.55074592	1.128862167	NA	NA
ASV_16s_10101	0.524565021	1.180092191	3.550004705	1.036101151	0.994224964	NA
ASV_16s_10102	0.508159515	0.734878648	3.550787614	1.387808048	0.985941768	NA
ASV_16s_10103	0.481994171	0.734838696	3.550799096	1.360968738	NA	NA

ASV_16s_10104	0.481994171	0.734838696	3.550799096	1.360968738	NA	NA
ASV_16s_10105	0.453877758	0.693620184	3.55071583	1.289728377	0.988712115	NA
ASV_16s_10106	0.454538254	0.693621002	3.550715593	1.290466438	0.988692647	NA
ASV_16s_10107	0.450590337	0.693613349	3.550717024	1.286004798	0.988810019	NA
ASV_16s_10108	0.424090515	0.693577438	3.550727295	1.255232722	0.989599084	NA
ASV_16s_10109	0.310199545	-0.089832233	3.55010071	1.518788415	NA	NA
ASV_16s_10110	0.318077724	-0.148715454	3.550021849	1.57757507	NA	NA
ASV_16s_10111	0.319372318	-0.195700125	3.549989794	1.592188435	0.978945427	NA
ASV_16s_10112	0.313076173	-0.24372148	3.549994318	1.568007485	0.979861415	NA
ASV_16s_10113	0.31029081	-0.240880498	3.550005365	1.560674562	NA	NA
ASV_16s_10114	0.332463961	-0.280079557	3.54991971	1.607059183	0.978370195	NA
ASV_16s_10115	0.336188111	-0.283310251	3.54990711	1.615377274	0.978044465	NA
ASV_16s_10116	0.310313627	-0.278687575	3.549996477	1.545356881	NA	NA
ASV_16s_10117	0.317409344	-0.294767516	3.549967581	1.556083936	0.980304274	NA
ASV_16s_10118	0.338795399	-0.330894373	3.549892892	1.585953772	0.979183893	NA
ASV_16s_10119	0.403070865	-0.681972894	3.549841093	0.92041282	NA	NA
ASV_16s_10120	0.310484749	-0.680517279	3.550167818	0.836557892	NA	NA
ASV_16s_10121	0.059602247	-0.168772912	3.550951234	0.292914396	NA	NA
ASV_16s_10122	0.342095674	0.138959977	3.549410905	1.697180006	0.974688814	NA
ASV_16s_10123	0.333746455	0.101121604	3.549340036	1.699331439	0.974596815	NA
ASV_16s_10124	0.420467229	-0.791409035	3.549299029	1.353139009	0.986963535	NA
ASV_16s_10125	0.385283975	0.084285563	3.549438874	1.478017286	0.983060956	NA
ASV_16s_10126	0.389573497	-0.82819307	3.549736851	1.453089972	0.983889396	NA
ASV_16s_10127	0.382261299	-0.461490353	3.55021175	1.071514474	NA	NA
ASV_16s_10128	0.400949879	-0.031841148	3.549234792	1.395241586	0.985716564	NA
ASV_16s_10129	0.40614246	-0.684469716	3.549797501	1.558323034	0.980221554	NA
ASV_16s_10130	0.330806402	0.329478162	3.549737844	0.914831704	NA	NA
ASV_16s_10131	0.424663186	0.685201424	3.550658235	1.50340345	0.982191599	NA
ASV_16s_10132	0.438072507	-0.712779079	3.549684185	1.617925153	NA	NA
ASV_16s_10133	0.404613073	-0.583053231	3.550039259	1.529602872	0.981267077	NA
ASV_16s_10134	0.379222308	-0.446289058	3.549377265	1.193519159	0.991075353	NA
ASV_16s_10135	0.340473733	-0.074449755	3.550318622	1.382990224	NA	NA
ASV_16s_10136	0.449823577	0.658583003	3.550729657	1.53684993	0.981006425	NA
ASV_16s_10137	0.252377476	1.706482035	3.550374054	0.42737435	NA	NA
ASV_16s_10138	0.399860935	1.337445889	3.550239502	0.875682021	NA	NA
ASV_16s_10139	0.438831675	1.30318831	3.549630373	1.299594943	NA	NA
ASV_16s_10140	0.450496749	0.40335707	3.550298104	0.939466014	NA	NA
ASV_16s_10141	0.219535858	-0.463654561	3.551053885	0.76538434	NA	NA
ASV_16s_10142	0.688327294	-0.566992348	3.549271254	1.070166813	NA	NA
ASV_16s_10143	0.351495405	-1.186039246	3.549591661	0.525165585	NA	NA
ASV_16s_10144	0.248535671	-0.257040652	3.549905553	1.13019312	NA	NA
ASV_16s_10145	0.159229564	1.174659935	3.550972758	0.501337045	NA	NA
ASV_16s_10146	0.214051898	0.220034517	3.550711847	0.811085435	0.99732917	NA
ASV_16s_10147	0.21546572	0.220033844	3.55071096	0.814368823	0.997294535	NA
ASV_16s_10148	0.216244613	0.220033472	3.550710476	0.816169472	0.997275416	NA
ASV_16s_10149	0.219707187	0.220031875	3.550708367	0.824101857	0.997190135	NA
ASV_16s_10150	0.221660417	0.220030974	3.550707206	0.828526382	0.997141816	NA
ASV_16s_10151	0.236476462	0.456599843	3.55068096	1.385388561	NA	NA
ASV_16s_10152	0.469424422	1.941074023	3.549629241	0.959428851	0.995456726	NA
ASV_16s_10153	0.384751858	0.72656231	3.548753008	0.975507328	NA	NA
ASV_16s_10154	0.372022397	1.122029347	3.550186136	1.416949428	0.985046455	NA
ASV_16s_10155	0.520819174	0.92003973	3.550648956	1.026706932	NA	NA
ASV_16s_10156	0.286180323	0.205341593	3.550635849	1.110313483	NA	NA
ASV_16s_10157	0.33341098	-0.068076761	3.550064894	1.592478644	NA	NA
ASV_16s_10158	0.365922927	1.422476561	3.550250025	0.728573244	NA	NA
ASV_16s_10159	0.186412843	0.279407624	3.551575285	0.663938078	NA	NA
ASV_16s_10160	0.092757787	0.285225721	3.551767114	0.431251511	NA	NA
ASV_16s_10161	0.153521623	0.280281946	3.551634542	0.594607666	NA	NA
ASV_16s_10162	0.304756624	1.148231763	3.550699181	0.760661229	NA	NA
ASV_16s_10163	0.464250432	0.149506411	3.550661184	2.031725773	0.95806355	NA
ASV_16s_10164	0.428972857	0.343732808	3.550794561	0.8119644	0.997319927	NA
ASV_16s_10165	0.407304959	-0.549353557	3.549502598	0.830714801	NA	NA
ASV_16s_10166	0.407304959	-0.549353557	3.549502598	0.830714801	NA	NA
ASV_16s_10167	0.367690296	1.442069353	3.549681741	1.220914292	NA	NA
ASV_16s_10168	0.257350196	-0.078919477	3.550726897	1.063464235	NA	NA
ASV_16s_10169	0.387578466	0.27326137	3.549983964	1.81945253	0.969154194	NA
ASV_16s_10170	0.307848802	0.289723903	3.550414857	1.409203315	0.985287707	NA

ASV_16s_10171	0.493581817	0.791590561	3.549386865	1.390359074	NA	NA
ASV_16s_10172	0.446123689	0.315939447	3.55074833	1.346354461	NA	NA
ASV_16s_10173	0.38569528	0.380276066	3.550761132	1.837683342	0.968275508	NA
ASV_16s_10174	0.411858659	0.315939231	3.550761706	1.305712795	0.988285855	NA
ASV_16s_10175	0.416659034	0.315939251	3.550759699	1.311609914	0.988126137	NA
ASV_16s_10176	0.423931038	0.315939254	3.550756749	1.32038611	0.987885978	NA
ASV_16s_10177	0.371003007	0.433677944	3.550762243	2.030174579	0.958151395	NA
ASV_16s_10178	0.375019889	0.315939473	3.550778791	1.258230042	0.98952379	NA
ASV_16s_10179	0.466307967	0.315939337	3.550741379	1.368755805	0.986509081	NA
ASV_16s_10180	0.43338206	0.315939261	3.550753065	1.331550216	0.987576203	NA
ASV_16s_10181	0.418163295	0.315939342	3.550759083	1.31342221	0.988076785	NA
ASV_16s_10182	0.394090959	0.315939345	3.550769551	1.283347683	0.988879561	NA
ASV_16s_10183	0.485973119	0.73484519	3.550797274	1.365121277	0.986615695	NA
ASV_16s_10184	0.475734506	0.734828643	3.550802033	1.354313106	NA	NA
ASV_16s_10185	0.235657782	0.734036927	3.551031281	0.999332349	NA	NA
ASV_16s_10186	0.446060305	0.693609286	3.550718695	1.280864703	0.988944305	NA
ASV_16s_10187	0.415993826	0.693569119	3.550730691	1.245468146	0.989842048	NA
ASV_16s_10188	0.306119924	-0.093229709	3.550113771	1.509706376	NA	NA
ASV_16s_10189	0.313739183	-0.14295901	3.550041971	1.562519507	NA	NA
ASV_16s_10190	0.314774859	-0.181250799	3.550013819	1.577199156	NA	NA
ASV_16s_10191	0.309497277	-0.240052439	3.550008595	1.558507792	0.980214719	NA
ASV_16s_10192	0.318569967	-0.258309704	3.549971139	1.578659788	0.979460853	NA
ASV_16s_10193	0.332940312	-0.289549621	3.549916219	1.60262932	0.978542502	NA
ASV_16s_10194	0.312046537	-0.270697658	3.549992136	1.55442369	0.980365477	NA
ASV_16s_10195	0.322106527	-0.289410585	3.549952234	1.572700036	0.979685532	NA
ASV_16s_10196	0.306262525	-0.334867977	3.55000496	1.493072642	NA	NA
ASV_16s_10197	0.315125043	-0.341905579	3.549972123	1.511419364	0.981911687	NA
ASV_16s_10198	0.306302454	-0.410637297	3.550015517	1.388402309	NA	NA
ASV_16s_10199	0.306330974	-0.471595535	3.550038886	1.273140405	NA	NA
ASV_16s_10200	0.306399423	-0.680432861	3.550186768	0.832295118	NA	NA
ASV_16s_10201	0.067854475	-0.168985941	3.550897489	0.336791857	NA	NA
ASV_16s_10203	0.0631402	0.685629144	3.551599303	0.32914441	0.999863164	NA
ASV_16s_10204	0.216805269	0.596135437	3.55067667	0.749314363	NA	NA
ASV_16s_10205	0.313067528	-0.141915467	3.549961156	1.491520433	0.982601771	NA
ASV_16s_10206	0.431250301	1.655276674	3.549930661	0.593016379	0.999029628	NA
ASV_16s_10207	0.314354367	-0.24412091	3.549865811	0.800820968	NA	NA
ASV_16s_10208	0.324368575	0.247351531	3.549691216	1.611148321	NA	NA
ASV_16s_10209	0.325857134	-0.390519741	3.550073994	1.364652173	0.986629418	NA
ASV_16s_10210	0.310899737	-0.366903042	3.549738813	0.935345246	NA	NA
ASV_16s_10211	0.351746702	-0.556033495	3.549778167	1.32136562	0.987858991	NA
ASV_16s_10212	0.387859641	-0.784795696	3.549585504	1.111080768	NA	NA
ASV_16s_10213	0.335430072	0.023674525	3.549429746	1.610988936	0.978216664	NA
ASV_16s_10214	0.390853915	-0.79074519	3.5494484	1.324771806	0.987764857	NA
ASV_16s_10215	0.318400261	-0.307688037	3.549519707	1.207852432	NA	NA
ASV_16s_10216	0.386315734	-0.476355672	3.5495592	1.42827016	NA	NA
ASV_16s_10217	0.454933418	-0.777865897	3.549490812	1.305103561	NA	NA
ASV_16s_10218	0.323342057	-0.161454474	3.551357601	0.850643261	0.996892119	NA
ASV_16s_10219	0.33722483	0.306870523	3.548958456	1.006463786	NA	NA
ASV_16s_10220	0.387813408	-0.735632445	3.549674653	1.496364329	0.98243526	NA
ASV_16s_10221	0.385788125	-0.708239739	3.549749195	1.506161681	0.982095576	NA
ASV_16s_10222	0.386287786	-0.725510733	3.549704428	1.49861256	0.982357654	NA
ASV_16s_10223	0.3680149	-0.683105633	3.549842685	1.471308099	0.983286381	NA
ASV_16s_10224	0.368276683	0.860654369	3.548819274	0.836125974	0.997057559	NA
ASV_16s_10225	0.380992699	-0.674170091	3.5498486	1.504056411	0.982168895	NA
ASV_16s_10226	0.344462766	0.765699417	3.549733406	0.979938739	0.995145693	NA
ASV_16s_10227	0.239039279	-0.661577157	3.550141196	0.720705104	NA	NA
ASV_16s_10228	0.433450001	0.860388089	3.287010876	1.330460507	0.987606651	NA
ASV_16s_10229	0.36973019	-0.283776321	3.550600559	1.027670201	0.994369742	NA
ASV_16s_10230	0.403738169	-0.420506001	3.551232867	0.561315464	NA	NA
ASV_16s_10231	0.451493858	1.381673462	3.550037826	1.209488354	NA	NA
ASV_16s_10232	0.387732735	-0.379148586	3.550427265	1.401681048	0.98551972	NA
ASV_16s_10233	0.483960809	1.865898654	2.315950573	2.649359473	0.915436695	NA
ASV_16s_10234	0.382888345	1.698940801	3.549361275	0.358406678	0.999817713	NA
ASV_16s_10235	0.279520963	-0.677381551	3.54942234	0.483585482	NA	NA
ASV_16s_10236	0.331861693	0.279298305	3.549601844	0.890083666	0.996412297	NA
ASV_16s_10237	0.374035753	0.403259783	3.550578734	0.879568201	NA	NA
ASV_16s_10238	0.514164684	-0.566197604	3.549470398	0.976229037	NA	NA

ASV_16s_10239	0.198833304	1.237119591	3.550793297	0.576857685	NA	NA
ASV_16s_10240	0.256152844	-0.005505842	3.54998217	1.300592122	NA	NA
ASV_16s_10241	0.110365886	0.351421905	3.55164747	0.416385325	NA	NA
ASV_16s_10242	0.19821763	-0.54001643	3.550341067	0.600997864	NA	NA
ASV_16s_10243	0.208226402	0.220037411	3.550715632	0.797331734	0.997471089	NA
ASV_16s_10244	0.221634175	0.220031034	3.550707223	0.82845067	0.997142648	NA
ASV_16s_10245	0.456417507	1.98763046	3.549891238	0.62495978	NA	NA
ASV_16s_10246	0.429972789	1.745006605	3.550004863	0.722092752	NA	NA
ASV_16s_10247	0.390379757	1.357234148	3.550144238	0.868280632	NA	NA
ASV_16s_10248	0.303023884	-0.217147997	3.549652467	1.397683213	NA	NA
ASV_16s_10249	0.322438816	-0.068824997	3.549963491	1.643836682	NA	NA
ASV_16s_10250	0.282669368	1.277930494	3.550698449	0.971292581	NA	NA
ASV_16s_10251	0.340376593	-0.068648562	3.549940516	1.670545064	NA	NA
ASV_16s_10252	0.340266475	-0.068339205	3.550002688	1.624417059	NA	NA
ASV_16s_10253	0.349843395	0.364599535	3.551336271	0.616520406	0.998898204	NA
ASV_16s_10254	0.475209616	1.214110269	3.550246636	0.911465693	NA	NA
ASV_16s_10255	0.097966329	-0.339867746	3.551562811	0.447181967	NA	NA
ASV_16s_10256	0.404311296	-0.081386696	3.550080565	1.241737893	0.989933928	NA
ASV_16s_10257	0.529958477	0.825515937	3.550266352	2.087778084	NA	NA
ASV_16s_10258	0.506746986	0.693066702	3.549729975	1.942872963	0.962934383	NA
ASV_16s_10259	0.428799549	0.198042967	3.549729759	1.894645509	0.965440548	NA
ASV_16s_10260	0.341676352	0.342348845	3.549957569	1.63784693	NA	NA
ASV_16s_10261	0.471929628	0.788581701	3.5490768	1.39506509	0.985721936	NA
ASV_16s_10262	0.36764442	0.363338816	3.550467802	0.93295991	NA	NA
ASV_16s_10263	0.44565176	0.770869735	3.550096804	1.839288611	0.968197472	NA
ASV_16s_10264	0.438642374	0.340549466	3.550727844	1.085175251	0.993335091	NA
ASV_16s_10265	0.339781957	0.360383443	3.550893001	1.247156064	NA	NA
ASV_16s_10266	0.339781957	0.360383443	3.550893001	1.247156064	NA	NA
ASV_16s_10267	0.3965414	0.36172431	3.55075922	1.731936515	0.973178993	NA
ASV_16s_10268	0.423894809	0.315939361	3.550756768	1.320310118	0.98788807	NA
ASV_16s_10269	0.487631293	0.962063873	3.550547857	1.58274874	0.97930586	NA
ASV_16s_10270	0.337974156	0.688269528	3.550751018	1.115496032	NA	NA
ASV_16s_10271	0.337974156	0.688269528	3.550751018	1.115496032	NA	NA
ASV_16s_10272	0.459502858	1.104417792	3.550391263	1.041521737	0.994130637	NA
ASV_16s_10273	0.232557022	0.734016234	3.55103732	0.99272294	NA	NA
ASV_16s_10274	0.42762868	0.693584947	3.550725853	1.259430103	0.989493549	NA
ASV_16s_10275	0.35488923	0.69346397	3.550761314	1.164918353	NA	NA
ASV_16s_10276	0.308106049	-0.086426163	3.550112527	1.509816391	NA	NA
ASV_16s_10277	0.305360615	-0.150562804	3.550068797	1.541063103	NA	NA
ASV_16s_10278	0.310177399	-0.166318018	3.550039735	1.560470927	NA	NA
ASV_16s_10279	0.329275979	-0.197800123	3.549954922	1.61850096	0.977921408	NA
ASV_16s_10280	0.331677782	-0.217789841	3.549938714	1.625780457	0.977633073	NA
ASV_16s_10281	0.315133385	-0.199933969	3.550003084	1.579927695	0.979412866	NA
ASV_16s_10282	0.302097343	-0.194040398	3.55005535	1.541161849	0.980850326	NA
ASV_16s_10283	0.307949803	-0.247852085	3.55001226	1.551575069	0.980470227	NA
ASV_16s_10284	0.305170145	-0.254526774	3.550021151	1.541081654	0.980853236	NA
ASV_16s_10285	0.354447722	-0.324316151	3.549846702	1.632637706	0.977359462	NA
ASV_16s_10286	0.307471894	-0.295597968	3.550003781	1.526945062	0.981362134	NA
ASV_16s_10287	0.31723896	-0.333542603	3.549964623	1.525050562	0.981429716	NA
ASV_16s_10288	0.334668286	-0.385894255	3.549909367	1.512737838	0.981865397	NA
ASV_16s_10289	0.302194312	-0.363281903	3.550022204	1.452149038	NA	NA
ASV_16s_10290	0.307045321	-0.446615715	3.550025421	1.324231045	NA	NA
ASV_16s_10291	0.107922754	-0.437686615	3.551361079	0.486663292	NA	NA
ASV_16s_10292	0.107922754	-0.437686615	3.551361079	0.486663292	NA	NA
ASV_16s_10293	0.30509946	-0.680405345	3.550192935	0.830916382	NA	NA
ASV_16s_10294	0.302314097	-0.680346215	3.550206224	0.827975588	NA	NA
ASV_16s_10295	0.302314097	-0.680346215	3.550206224	0.827975588	NA	NA
ASV_16s_10296	0.085435841	-0.1693065	3.5508174	0.421962376	NA	NA
ASV_16s_10297	0.195685251	0.22004429	3.55072454	0.766598589	NA	NA
ASV_16s_10299	0.114800364	0.687070086	3.551131525	0.57949303	0.999100233	NA
ASV_16s_10300	0.316569337	-0.299934566	3.550123829	1.394104416	0.985751156	NA
ASV_16s_10301	0.315828657	-0.046300484	3.549934638	1.496716263	0.982423125	NA
ASV_16s_10302	0.308711283	-0.173733851	3.550802407	0.843424593	NA	NA
ASV_16s_10303	0.389866793	-0.458817317	3.54965613	1.464333965	0.983518793	NA
ASV_16s_10304	0.312588405	-0.344973658	3.55020068	1.349967976	0.987054656	NA
ASV_16s_10305	0.328123477	-0.712409171	3.550153035	0.915460836	NA	NA
ASV_16s_10306	0.344406876	-0.533094873	3.549733542	1.33566252	0.987460888	NA

ASV_16s_10307	0.329200215	1.741027858	3.549681666	0.394863691	NA	NA
ASV_16s_10308	0.323291423	0.491529937	3.549373502	1.458016836	0.983727629	NA
ASV_16s_10309	0.372955493	-0.516359911	3.549551563	1.40621767	0.985380062	NA
ASV_16s_10310	0.408358463	-0.79114516	3.549357052	1.340329091	0.987329241	NA
ASV_16s_10311	0.330669436	0.147829265	3.549287476	1.658435972	0.976312681	NA
ASV_16s_10312	0.389702294	-0.782148767	3.5494895	1.408171925	0.985319651	NA
ASV_16s_10313	0.382865712	-1.050958307	3.55028087	0.753235024	0.997892436	NA
ASV_16s_10314	0.396663809	-0.786158732	3.549447245	1.408443804	0.985311234	NA
ASV_16s_10315	0.358593828	0.02674466	3.549211841	1.781044127	0.970959944	NA
ASV_16s_10316	0.426142309	0.517347108	3.549635908	2.181439562	0.949119152	NA
ASV_16s_10317	0.322780832	-1.081384502	3.54977595	1.075500839	0.993516957	NA
ASV_16s_10318	0.338492074	0.336194282	3.551489787	0.728570972	NA	NA
ASV_16s_10319	0.403171829	-0.7550086	3.549592187	1.519293704	0.981634186	NA
ASV_16s_10320	0.363288219	-0.899563063	3.54931956	1.036711748	0.994214387	NA
ASV_16s_10321	0.395707231	-0.701468454	3.549756566	1.531918461	0.981184025	NA
ASV_16s_10322	0.413585425	-0.724412057	3.549736027	1.387090566	0.98596339	NA
ASV_16s_10323	0.353042542	0.190723352	3.550636911	0.730990085	NA	NA
ASV_16s_10324	0.332981049	0.927162817	3.551212968	0.878987371	NA	NA
ASV_16s_10325	0.381346913	-0.015913686	3.549395573	1.080298301	0.99342717	NA
ASV_16s_10326	0.394226164	-0.250828923	3.549366819	1.740479187	0.972800198	NA
ASV_16s_10327	0.363285281	-0.081089002	3.550345893	1.367016893	0.986560154	NA
ASV_16s_10328	0.398190988	0.063110825	3.550780361	0.906249294	0.99620251	NA
ASV_16s_10329	0.480092402	1.338475606	3.55000001	0.93454278	NA	NA
ASV_16s_10330	0.376603186	1.333453085	3.550312839	0.855949593	NA	NA
ASV_16s_10331	0.384599444	0.830333545	3.550960424	0.772490937	NA	NA
ASV_16s_10332	0.253861888	0.388135199	3.551188016	0.752050933	NA	NA
ASV_16s_10333	0.235238567	0.229124474	3.550881031	0.491576375	0.999476744	NA
ASV_16s_10334	0.361920088	0.911808148	3.550410814	1.176113867	0.991466511	NA
ASV_16s_10335	0.361884017	0.35184371	3.549803498	1.704188282	0.974388421	NA
ASV_16s_10336	0.50022486	1.338685044	3.54995179	0.947791788	NA	NA
ASV_16s_10337	0.213398058	-0.54326467	3.550230094	0.624311837	NA	NA
ASV_16s_10338	0.155039312	1.166762062	3.550994902	0.492433245	NA	NA
ASV_16s_10339	0.22228485	0.220030767	3.550706842	0.829900699	NA	NA
ASV_16s_10340	0.222122892	0.220030824	3.550706937	0.82953616	0.997130713	NA
ASV_16s_10341	0.42406461	0.33958801	3.55074081	0.773614744	NA	NA
ASV_16s_10342	0.348009692	-1.007703105	3.549696649	1.185006804	NA	NA
ASV_16s_10343	0.325913777	-0.069131167	3.550057499	1.640375634	0.97704838	NA
ASV_16s_10344	0.359161473	-0.06072572	3.550482154	1.296862928	0.988523057	NA
ASV_16s_10345	0.316421835	-0.068904468	3.549986597	1.631162761	NA	NA
ASV_16s_10346	0.329363354	0.616256099	3.549700378	1.368876132	NA	NA
ASV_16s_10347	0.318899426	-0.068755999	3.549975014	1.628818728	NA	NA
ASV_16s_10348	0.322805892	-0.694473847	3.549784337	1.022840711	NA	NA
ASV_16s_10349	0.447104993	0.693801084	3.549392871	1.312639904	NA	NA
ASV_16s_10350	0.417971516	0.625245375	3.549384807	1.163637575	NA	NA
ASV_16s_10351	0.513566234	0.312033546	3.550591072	1.017432412	NA	NA
ASV_16s_10352	0.251723002	0.279664674	3.551439627	0.773173952	NA	NA
ASV_16s_10353	0.090316793	0.285479897	3.551774845	0.423502762	NA	NA
ASV_16s_10354	0.330602314	-0.029249032	3.550765142	1.249086796	NA	NA
ASV_16s_10355	0.368562722	0.289566428	3.54967962	1.681411248	NA	NA
ASV_16s_10356	0.408828194	0.750386397	3.549618024	1.442734437	NA	NA
ASV_16s_10357	0.335251531	0.360382216	3.550897035	1.240332191	NA	NA
ASV_16s_10358	0.386579522	0.315939388	3.550773084	1.273579946	0.989132911	NA
ASV_16s_10359	0.361107633	0.315939396	3.550786154	1.239018931	0.990000572	NA
ASV_16s_10360	0.395393322	0.402945511	3.550754152	1.972194442	0.961363283	NA
ASV_16s_10361	0.463215177	0.73480767	3.550808145	1.340735676	NA	NA
ASV_16s_10362	0.463215177	0.73480767	3.550808145	1.340735676	NA	NA
ASV_16s_10363	0.463215177	0.73480767	3.550808145	1.340735676	NA	NA
ASV_16s_10364	0.29793216	-0.047758503	3.550198605	1.441983711	NA	NA
ASV_16s_10365	0.30557994	-0.150844723	3.550067777	1.541736208	NA	NA
ASV_16s_10366	0.305838859	-0.160713517	3.550060067	1.545943485	NA	NA
ASV_16s_10367	0.305838859	-0.160713517	3.550060067	1.545943485	NA	NA
ASV_16s_10368	0.306615616	-0.189998401	3.550039844	1.554247272	0.980371974	NA
ASV_16s_10369	0.337017991	-0.231754361	3.549916857	1.638728115	0.977114821	NA
ASV_16s_10370	0.314177683	-0.263334317	3.549985824	1.563823063	0.980017493	NA
ASV_16s_10371	0.298046242	-0.24718816	3.550050974	1.522185183	NA	NA
ASV_16s_10372	0.313657103	-0.281774184	3.549983669	1.553129161	0.980413121	NA
ASV_16s_10373	0.315739642	-0.293259526	3.549973907	1.552133578	0.980449716	NA

ASV_16s_10374	0.322183654	-0.317952666	3.549948168	1.55218398	0.980447864	NA
ASV_16s_10375	0.301872984	-0.300494948	3.550024542	1.507325927	0.982054952	NA
ASV_16s_10376	0.304669754	-0.323269673	3.550011472	1.498404137	0.982364857	NA
ASV_16s_10377	0.298097578	-0.338608696	3.550037028	1.466108918	NA	NA
ASV_16s_10378	0.325993832	-0.37002508	3.549936005	1.510778022	0.981934178	NA
ASV_16s_10379	0.106464338	-0.433066732	3.551374336	0.482489023	NA	NA
ASV_16s_10380	0.387300487	-0.681773094	3.549885928	0.907530308	NA	NA
ASV_16s_10381	0.351587527	-0.681255847	3.550001937	0.876412374	NA	NA
ASV_16s_10382	0.326642315	-0.68082884	3.550097789	0.85277757	0.996867296	NA
ASV_16s_10383	0.33124846	-0.680912331	3.550079042	0.857262869	0.996814708	NA
ASV_16s_10384	0.298228772	-0.680257253	3.550226207	0.823598321	NA	NA
ASV_16s_10385	0.095017593	-0.169430357	3.550786165	0.464265091	NA	NA
ASV_16s_10386	0.101122807	-0.169498502	3.550769333	0.489823275	NA	NA
ASV_16s_10387	0.317121914	-0.122470554	3.549968085	1.682574773	0.975308274	NA
ASV_16s_10388	0.304945965	-0.108934119	3.549755841	1.318873915	NA	NA
ASV_16s_10389	0.321067445	0.236650059	3.549650351	1.644676086	0.976874422	NA
ASV_16s_10390	0.342154215	-0.330925423	3.549372613	1.109296292	NA	NA
ASV_16s_10391	0.350262294	0.486796113	3.549401492	1.589761363	0.979038448	NA
ASV_16s_10392	0.386995521	0.118348964	3.549056682	1.941494894	0.963007342	NA
ASV_16s_10393	0.372343496	-0.790056553	3.549550773	1.267845166	0.98927998	NA
ASV_16s_10394	0.347902603	0.131944099	3.54937116	1.057918525	NA	NA
ASV_16s_10395	0.39047704	-0.784272166	3.549477877	1.401708763	NA	NA
ASV_16s_10396	0.337018757	-0.714658684	3.550392051	1.084911414	NA	NA
ASV_16s_10397	0.361336677	-0.704621267	3.549798136	1.439993164	NA	NA
ASV_16s_10398	0.377302176	0.783909647	3.548838542	1.38547433	0.986012023	NA
ASV_16s_10399	0.367059187	-0.81895093	3.549712166	1.37821659	0.986229145	NA
ASV_16s_10400	0.387299109	-0.744865752	3.549647759	1.486757218	0.982764586	NA
ASV_16s_10401	0.365542583	-0.722392756	3.549743677	1.445245467	0.984144959	NA
ASV_16s_10402	0.242100452	1.115410849	3.550918101	0.793522776	NA	NA
ASV_16s_10403	0.374464797	-0.716155948	3.550198732	1.042122262	0.994120127	NA
ASV_16s_10404	0.385206451	-0.700759391	3.549770557	1.507361571	0.982053708	NA
ASV_16s_10405	0.379725757	0.687310702	3.550660825	1.433057088	NA	NA
ASV_16s_10406	0.333523044	-1.034938712	3.549868232	0.672052383	NA	NA
ASV_16s_10407	0.396810811	-0.669084504	3.549843784	1.533611125	0.981123178	NA
ASV_16s_10408	0.354692496	-0.191409234	3.549898471	1.374726313	0.986332827	NA
ASV_16s_10409	0.395390763	0.320369436	3.55038567	1.588567844	NA	NA
ASV_16s_10410	0.37086271	-0.096285551	3.5508365	0.995889374	NA	NA
ASV_16s_10411	0.371655227	0.182500041	3.549506603	1.413415549	NA	NA
ASV_16s_10412	0.468505753	0.371851203	3.549657531	1.265848316	0.9893309	NA
ASV_16s_10413	0.468743077	-0.234241867	3.54967103	1.596968251	0.978761525	NA
ASV_16s_10414	0.401867492	0.614901291	3.550765746	1.648862162	0.976704357	NA
ASV_16s_10415	0.515121314	-0.490657996	3.551096232	0.683967524	NA	NA
ASV_16s_10416	0.3236499	0.116962296	3.549936991	1.632992135	0.977345267	NA
ASV_16s_10417	0.319729426	0.268157248	3.549075602	1.016923539	NA	NA
ASV_16s_10418	0.23773391	-0.146256365	3.550682721	0.65884638	0.998632347	NA
ASV_16s_10419	0.411048046	0.810412528	3.549562072	1.416062943	0.985074184	NA
ASV_16s_10420	0.351336589	0.539310346	3.549857957	1.191800017	NA	NA
ASV_16s_10421	0.323162096	0.40125179	3.550819287	0.832163958	NA	NA
ASV_16s_10422	0.327979232	0.552544246	3.549911423	1.413697431	0.985148027	NA
ASV_16s_10423	0.250431322	0.387181412	3.551209006	0.747486879	NA	NA
ASV_16s_10424	0.250431322	0.387181412	3.551209006	0.747486879	NA	NA
ASV_16s_10425	0.3942621	1.188583188	3.550202927	1.089686807	NA	NA
ASV_16s_10426	0.330175568	-0.539282859	3.550473855	1.509245876	0.981987841	NA
ASV_16s_10427	0.493465065	1.338617033	3.549967572	0.943393147	NA	NA
ASV_16s_10428	0.192931827	-0.538450405	3.55038298	0.5924292	NA	NA
ASV_16s_10429	0.345425517	-0.07910222	3.550672812	1.212070452	NA	NA
ASV_16s_10430	0.294548267	-0.079010876	3.550700126	1.131440913	NA	NA
ASV_16s_10431	0.207658502	0.220037735	3.550716016	0.795939051	0.997485176	NA
ASV_16s_10432	0.23099523	0.596154774	3.550668899	0.780669109	NA	NA
ASV_16s_10433	0.307431744	-0.069075706	3.550123588	1.583753014	NA	NA
ASV_16s_10434	0.286937415	0.296690451	3.549943122	1.337924583	NA	NA
ASV_16s_10435	0.319114971	-0.068929642	3.54998052	1.639295036	0.977091971	NA
ASV_16s_10436	0.350319874	1.28607489	3.550102904	1.393050657	NA	NA
ASV_16s_10437	0.340043377	-0.354823419	3.549463059	1.80158603	0.970001845	NA
ASV_16s_10438	0.326437628	-0.067638543	3.550186173	1.511382792	0.98191297	NA
ASV_16s_10439	0.331051458	-0.06748552	3.550222705	1.494556122	NA	NA
ASV_16s_10440	0.403524102	1.069209399	3.550397157	1.016467759	0.994558496	NA

ASV_16s_10441	0.424949357	0.32767122	3.549768228	1.838087614		0.968255865	NA
ASV_16s_10442	0.179054441	0.279347967	3.55159483	0.64951021	NA		NA
ASV_16s_10443	0.416358809	-0.986518447	3.54986185	0.786038981	NA		NA
ASV_16s_10444	0.423978975	1.10872299	3.550184293	1.09375704		0.993171085	NA
ASV_16s_10445	0.413767632	0.189239993	3.550265773	2.360919398		0.937200952	NA
ASV_16s_10446	0.482726374	0.176638784	3.550333396	2.035390351		0.957855627	NA
ASV_16s_10447	0.586601573	0.418802215	3.550699854	2.625886107		0.91732326	NA
ASV_16s_10448	0.431548498	0.343516012	3.549829081	1.772726809	NA		NA
ASV_16s_10449	0.402389838	0.342956327	3.54980124	1.786202778		0.970720996	NA
ASV_16s_10450	0.409954287	0.784410357	3.549397497	1.41652531	NA		NA
ASV_16s_10451	0.420148273	0.699160805	3.549928042	1.390449897	NA		NA
ASV_16s_10452	0.433570662	0.401625254	3.550596096	1.453532119	NA		NA
ASV_16s_10453	0.454460262	0.342568018	3.549701385	1.937367543	NA		NA
ASV_16s_10454	0.404341401	0.335337011	3.550782649	1.094980784		0.993147492	NA
ASV_16s_10455	0.492283597	0.489102293	3.550371804	1.812499602		0.969485652	NA
ASV_16s_10456	0.387538626	0.315939405	3.550772629	1.274817931		0.989101	NA
ASV_16s_10457	0.430075456	0.315939395	3.550754346	1.327596335		0.987686461	NA
ASV_16s_10458	0.353980407	0.315939417	3.55079015	1.228891013		0.990246404	NA
ASV_16s_10459	0.345425517	0.315939516	3.550795161	1.21648881	NA		NA
ASV_16s_10460	0.345425517	0.315939516	3.550795161	1.21648881	NA		NA
ASV_16s_10461	0.328961512	0.688252852	3.550756396	1.101772598	NA		NA
ASV_16s_10462	0.472173791	1.157073137	3.55025653	1.022176676		0.994462819	NA
ASV_16s_10463	0.489450668	1.176916448	3.55016787	1.022677007		0.994454383	NA
ASV_16s_10464	0.345425517	0.693444673	3.550767028	1.151177848	NA		NA
ASV_16s_10465	0.293732753	0.271715015	3.550930109	0.799011781	NA		NA
ASV_16s_10466	0.293875355	-0.093557681	3.550165256	1.473872518	NA		NA
ASV_16s_10467	0.300464643	-0.114243931	3.550117526	1.507840298	NA		NA
ASV_16s_10468	0.31338393	-0.179529009	3.55002004	1.571701389	NA		NA
ASV_16s_10469	0.30331275	-0.223977038	3.550037594	1.543316553		0.980772039	NA
ASV_16s_10470	0.304089507	-0.253416285	3.550025643	1.538163161	NA		NA
ASV_16s_10471	0.298012352	-0.247142126	3.550051163	1.52203532		0.981536978	NA
ASV_16s_10472	0.318300426	-0.31475781	3.54996199	1.543900736		0.980750781	NA
ASV_16s_10473	0.299053732	-0.297897602	3.550036087	1.500684375		0.982285957	NA
ASV_16s_10474	0.297793362	-0.306980674	3.5500399	1.490772839		0.982627385	NA
ASV_16s_10475	0.300578725	-0.309484715	3.550028535	1.497168051		0.98240754	NA
ASV_16s_10476	0.297545851	-0.317109651	3.550039932	1.482546875		0.982907742	NA
ASV_16s_10477	0.328377528	-0.36166007	3.549927211	1.526632424		0.981373297	NA
ASV_16s_10478	0.294017957	-0.345979477	3.550053947	1.447339915	NA		NA
ASV_16s_10479	0.320291768	-0.386810038	3.549958231	1.469825838		0.98333594	NA
ASV_16s_10480	0.323805731	-0.480406694	3.549975968	1.299616875		0.988449563	NA
ASV_16s_10481	0.105005923	-0.428389704	3.551387872	0.478298733	NA		NA
ASV_16s_10482	0.402317493	-0.681965642	3.549843285	0.919750267		0.996021329	NA
ASV_16s_10483	0.32587434	-0.680814496	3.550101015	0.852006688		0.996876276	NA
ASV_16s_10484	0.351666759	-0.681256857	3.550001724	0.876458612		0.996583141	NA
ASV_16s_10485	0.294143446	-0.680165878	3.550246739	0.819163623	NA		NA
ASV_16s_10486	0.100383418	-0.06955343	3.550766857	0.715122389	NA		NA
ASV_16s_10487	0.178745798	0.687720398	3.550925717	0.797234225		0.997472077	NA
ASV_16s_10488	0.310222317	-0.187877987	3.550306126	1.314210299		0.988055285	NA
ASV_16s_10489	0.355344029	-0.504120974	3.5498767	1.424167617	NA		NA
ASV_16s_10490	0.398823208	-0.68119305	3.549614359	1.402389109		0.985497976	NA
ASV_16s_10491	0.30164397	-0.321772443	3.5496206	1.050982284	NA		NA
ASV_16s_10492	0.328556911	0.691581345	3.549814638	1.398719312		0.985610458	NA
ASV_16s_10493	0.330955762	0.458369161	3.549610364	1.553255688		0.980408467	NA
ASV_16s_10494	0.332479729	0.230305184	3.549763949	1.43932519	NA		NA
ASV_16s_10495	0.408512134	-0.786277608	3.549416243	1.452957623		0.983893728	NA
ASV_16s_10496	0.360840622	-0.369674701	3.549532933	1.3198114		0.987901795	NA
ASV_16s_10497	0.406452965	0.030457731	3.5493595	1.360995302		0.986736101	NA
ASV_16s_10498	0.34781709	-0.294105664	3.549486473	1.3448099	NA		NA
ASV_16s_10499	0.334878786	1.784217838	3.549904411	0.727217984	NA		NA
ASV_16s_10500	0.382871601	0.456024846	3.549358496	1.477882331	NA		NA
ASV_16s_10501	0.363795863	-0.288592091	3.550810328	0.81651075		0.997271782	NA
ASV_16s_10502	0.378705243	-0.661139755	3.549878809	1.500029214		0.982308648	NA
ASV_16s_10503	0.380612637	-0.124033546	3.550558352	1.297199738		0.988514084	NA
ASV_16s_10504	0.34353899	-0.298574102	3.550396687	1.2197448		0.99046515	NA
ASV_16s_10505	0.322990391	-0.974836737	3.549769606	1.01323066	NA		NA
ASV_16s_10506	0.37326954	0.340896667	3.551292443	0.785177361	NA		NA
ASV_16s_10507	0.341670263	-0.780681256	3.549780965	1.1734088	NA		NA

ASV_16s_10508	0.327082083	-0.608665511	3.549950376	1.233066232	NA	NA
ASV_16s_10509	0.37711857	-0.163530265	3.550505315	2.108427668	0.953595986	NA
ASV_16s_10510	0.338723991	0.220799866	3.548949099	1.615555448	NA	NA
ASV_16s_10511	0.428994821	-0.214362431	3.548979232	1.15918136	NA	NA
ASV_16s_10512	0.352482247	0.219984804	3.548897645	1.663307153	NA	NA
ASV_16s_10513	0.346189075	0.083118344	3.550244994	0.971122519	NA	NA
ASV_16s_10514	0.468950852	0.770237884	3.549024862	1.284393027	0.988852234	NA
ASV_16s_10515	0.435435547	0.727665458	3.550830557	1.345836167	NA	NA
ASV_16s_10516	0.325293457	-0.068203273	3.550251932	1.435251194	NA	NA
ASV_16s_10517	0.168524296	0.888189425	3.550958886	0.489918511	NA	NA
ASV_16s_10518	0.339790336	-0.026792357	3.550858764	0.906008952	NA	NA
ASV_16s_10519	0.346263855	0.693313287	3.549344319	1.579763104	0.979419099	NA
ASV_16s_10520	0.514939462	0.271575164	3.550068057	0.979593973	0.995151031	NA
ASV_16s_10521	0.318735218	0.40056403	3.550838982	0.82757843	NA	NA
ASV_16s_10522	0.453637941	0.627045615	3.549635866	2.368149793	0.936694087	NA
ASV_16s_10523	0.247000756	0.386214533	3.551230527	0.742866031	NA	NA
ASV_16s_10524	0.576663528	0.066596702	3.549880526	0.962942158	NA	NA
ASV_16s_10525	0.265249698	-0.558868303	3.550038691	0.94897423	0.995610197	NA
ASV_16s_10526	0.23938723	-0.083939023	3.55067449	0.880963251	NA	NA
ASV_16s_10527	0.365737939	1.336096372	3.55019413	0.783552534	NA	NA
ASV_16s_10528	0.190879972	1.226516106	3.550828026	0.562784293	NA	NA
ASV_16s_10529	0.128397078	0.590472989	3.551284195	0.620509219	NA	NA
ASV_16s_10530	0.259388803	0.133001255	3.549825215	1.227280437	0.990285147	NA
ASV_16s_10531	0.190288925	-0.53743625	3.550404313	0.588031071	NA	NA
ASV_16s_10532	0.179333407	-0.082734429	3.550285887	0.885795143	NA	NA
ASV_16s_10533	0.29051336	-0.079002283	3.550702703	1.1244529	NA	NA
ASV_16s_10534	0.20705686	0.220038004	3.550716423	0.794481296	0.997499866	NA
ASV_16s_10535	0.321862039	0.310934154	3.550791402	1.157819217	NA	NA
ASV_16s_10536	0.227830912	0.59615067	3.550670551	0.77380246	NA	NA
ASV_16s_10537	0.391800539	1.45483354	3.550172173	1.193835153	0.99106815	NA
ASV_16s_10538	0.22932903	-0.050032311	3.550554854	0.827862187	NA	NA
ASV_16s_10539	0.43917902	0.397841314	3.548713337	1.049708844	0.993986317	NA
ASV_16s_10540	0.306852439	0.676312397	3.550544092	1.321532717	0.987854383	NA
ASV_16s_10541	0.411744096	1.331996428	3.315897209	1.617129311	0.977975494	NA
ASV_16s_10542	0.297719996	-0.214931711	3.550169915	0.797374643	NA	NA
ASV_16s_10543	0.303892354	-0.069045083	3.550109069	1.579123582	NA	NA
ASV_16s_10544	0.35024556	-0.055775152	3.550560513	1.1218557	NA	NA
ASV_16s_10545	0.307856501	-0.068873448	3.550011406	1.605091991	NA	NA
ASV_16s_10546	0.427305916	0.047733681	3.549271754	0.768245104	NA	NA
ASV_16s_10547	0.290112884	0.758266009	3.550390146	1.469170239	NA	NA
ASV_16s_10548	0.310334092	-0.068712077	3.549999467	1.602646728	NA	NA
ASV_16s_10549	0.299687928	-0.361853614	3.550688989	1.347507112	NA	NA
ASV_16s_10550	0.404597698	0.342314221	3.550884209	0.878133511	0.996562431	NA
ASV_16s_10551	0.256179917	0.279433151	3.551430671	0.779547952	NA	NA
ASV_16s_10552	0.421181722	0.343732656	3.550803039	0.803387189	0.997409232	NA
ASV_16s_10553	0.452622583	0.582372412	3.550668218	1.721819073	0.973623683	NA
ASV_16s_10554	0.287490725	0.342619916	3.550736685	1.78953667	0.970565979	NA
ASV_16s_10555	0.438751455	0.470077336	3.549609268	1.636832376	0.977191134	NA
ASV_16s_10556	0.527254369	0.739848775	3.549977973	2.116492985	0.953112152	NA
ASV_16s_10557	0.443650271	0.353516114	3.550054385	1.698596629	0.974628259	NA
ASV_16s_10558	0.430937608	0.353490322	3.550061275	1.680041473	0.975414819	NA
ASV_16s_10559	0.35714956	0.409208731	3.550546274	1.347221045	NA	NA
ASV_16s_10560	0.523811397	0.473831965	3.548866636	1.607536065	NA	NA
ASV_16s_10561	0.326190679	0.360379657	3.550905438	1.226404757	NA	NA
ASV_16s_10562	0.381125311	0.315939424	3.550775742	1.266338091	0.989318424	NA
ASV_16s_10563	0.400066447	0.315939418	3.550766847	1.290897691	0.988681262	NA
ASV_16s_10564	0.416519279	0.315939414	3.550759768	1.31136538	0.988132786	NA
ASV_16s_10565	0.348221964	0.315939437	3.550793499	1.22055526	0.990445891	NA
ASV_16s_10566	0.373452555	0.315939427	3.550779611	1.255974638	0.989580478	NA
ASV_16s_10567	0.340693661	0.315939526	3.550798043	1.20947663	NA	NA
ASV_16s_10568	0.340693661	0.315939526	3.550798043	1.20947663	NA	NA
ASV_16s_10569	0.46574642	1.149935913	3.550572277	1.074407053	NA	NA
ASV_16s_10570	0.450695848	0.734785505	3.550814594	1.326787178	NA	NA
ASV_16s_10571	0.450695848	0.734785505	3.550814594	1.326787178	NA	NA
ASV_16s_10572	0.398598255	0.693538578	3.550738467	1.223717616	0.990370513	NA
ASV_16s_10573	0.370759122	0.693493449	3.550752405	1.187013707	0.991222837	NA
ASV_16s_10574	0.374335874	0.693500008	3.5507505	1.191866009	0.991112977	NA

ASV_16s_10575	0.340693661	0.693434608	3.550770004	1.144178041	NA	NA
ASV_16s_10576	0.340693661	0.693434608	3.550770004	1.144178041	NA	NA
ASV_16s_10577	0.311238288	-0.008260371	3.550193199	1.381973111	NA	NA
ASV_16s_10578	0.294313671	-0.029527376	3.550238503	1.392758535	NA	NA
ASV_16s_10579	0.289772917	-0.054100913	3.550227929	1.42474012	NA	NA
ASV_16s_10580	0.289807142	-0.117991989	3.55016002	1.478050268	NA	NA
ASV_16s_10581	0.289824254	-0.148564383	3.550134887	1.493122888	NA	NA
ASV_16s_10582	0.297420697	-0.15912567	3.550095069	1.520019323	NA	NA
ASV_16s_10583	0.296925676	-0.177963428	3.550085172	1.52327781	0.981492823	NA
ASV_16s_10584	0.317413199	-0.229725446	3.549983232	1.582882956	0.979300761	NA
ASV_16s_10585	0.289852774	-0.198503399	3.550103652	1.503786238	0.982178291	NA
ASV_16s_10586	0.289858478	-0.208439405	3.550098882	1.503772494	0.982178769	NA
ASV_16s_10587	0.296447766	-0.225952466	3.550064254	1.522184488	0.98153168	NA
ASV_16s_10588	0.308843511	-0.25814503	3.55000658	1.550312879	0.980516534	NA
ASV_16s_10589	0.33427464	-0.308891441	3.549909103	1.592339016	0.978939647	NA
ASV_16s_10590	0.310385616	-0.298184297	3.549992633	1.533462642	0.98112852	NA
ASV_16s_10591	0.31154338	-0.328947909	3.549985486	1.513029044	0.981855164	NA
ASV_16s_10592	0.298018392	-0.317517341	3.550038042	1.483570159	0.982873015	NA
ASV_16s_10593	0.306092592	-0.410473643	3.550016462	1.387883483	0.985939494	NA
ASV_16s_10594	0.303876185	-0.432591297	3.550032549	1.341782856	NA	NA
ASV_16s_10595	0.390545901	-0.681813555	3.549876505	0.91018059	0.996150317	NA
ASV_16s_10596	0.318016351	-0.680665448	3.550134497	0.844153549	0.996966808	NA
ASV_16s_10597	0.29005812	-0.680073754	3.550267842	0.814669421	NA	NA
ASV_16s_10598	0.29005812	-0.680073754	3.550267842	0.814669421	NA	NA
ASV_16s_10599	0.29005812	-0.680073754	3.550267842	0.814669421	NA	NA
ASV_16s_10600	0.29005812	-0.680073754	3.550267842	0.814669421	NA	NA
ASV_16s_10601	0.090649502	-0.169376513	3.550799601	0.445299659	0.99962315	NA
ASV_16s_10602	0.128828828	0.220102532	3.550801167	0.568344634	NA	NA
ASV_16s_10604	0.305936926	-0.062804069	3.550012408	1.499677627	0.982320818	NA
ASV_16s_10605	0.308466187	-0.36259503	3.550156434	1.342908166	NA	NA
ASV_16s_10606	0.304255019	0.211017549	3.549937162	1.524441178	0.981451424	NA
ASV_16s_10607	0.327488552	-0.836948554	3.550102063	1.146893013	0.992098718	NA
ASV_16s_10608	0.314568278	0.044308364	3.54962663	1.544519505	0.980728249	NA
ASV_16s_10609	0.30125056	0.085979281	3.549594303	1.550707427	0.980502066	NA
ASV_16s_10610	0.318238786	-0.009728295	3.549460611	1.511833019	0.981897172	NA
ASV_16s_10611	0.299350331	-0.333761413	3.549698659	0.987591049	NA	NA
ASV_16s_10612	0.30513251	0.338007739	3.549811316	1.469831518	0.983335751	NA
ASV_16s_10613	0.303983955	0.628180382	3.549585389	1.428200501	0.98469181	NA
ASV_16s_10614	0.288002479	0.726659293	3.549954912	1.295463055	0.988560305	NA
ASV_16s_10615	0.30410386	-0.307496167	3.549586484	1.152750076	NA	NA
ASV_16s_10616	0.318436067	0.45459411	3.549346125	0.883288858	0.996498175	NA
ASV_16s_10617	0.350777317	-1.136861888	3.549757036	0.667937352	NA	NA
ASV_16s_10618	0.360233529	-0.72308396	3.54975154	1.429490944	0.984650813	NA
ASV_16s_10619	0.364112436	-0.718910168	3.549754009	1.443523064	0.984200744	NA
ASV_16s_10620	0.337008186	-1.021656281	3.550048691	0.716438437	0.998206219	NA
ASV_16s_10621	0.382244165	-0.284525745	3.549479297	1.45195841	0.983926412	NA
ASV_16s_10622	0.376090511	0.44729733	3.549402264	1.454953299	NA	NA
ASV_16s_10623	0.372072904	-0.80705096	3.549727949	1.177819102	0.991428672	NA
ASV_16s_10624	0.336490142	0.685961307	3.550740112	1.339586093	0.987350258	NA
ASV_16s_10625	0.374533448	-0.644886622	3.549918698	1.489337524	0.9826765	NA
ASV_16s_10626	0.379082144	-0.610617487	3.549993331	1.489334752	0.982676594	NA
ASV_16s_10627	0.385065518	-0.534682783	3.549687289	1.674037858	0.975666254	NA
ASV_16s_10628	0.36003233	-0.572059115	3.55010087	1.44821728	0.984048431	NA
ASV_16s_10629	0.37582108	-0.677155604	3.549661021	1.129631986	NA	NA
ASV_16s_10630	0.34756615	0.335717111	3.550518665	1.444491159	0.984169404	NA
ASV_16s_10631	0.389029947	-0.518247703	3.550203148	1.500417252	0.982295211	NA
ASV_16s_10632	0.362592397	-0.684193137	3.5505116	0.726170374	0.998126476	NA
ASV_16s_10633	0.423203181	-1.438613104	3.548878545	1.120357921	NA	NA
ASV_16s_10634	0.355524935	24.4631986	3.548522069	-0.993621816	NA	NA
ASV_16s_10635	0.405830018	-0.456277277	3.550301445	1.501569137	0.982255287	NA
ASV_16s_10636	0.373415647	4.50E-04	3.550397807	1.310657193	0.98815203	NA
ASV_16s_10637	0.470382351	-0.331308877	3.548908437	0.898436669	0.996304864	NA
ASV_16s_10638	0.335783911	0.355557777	3.55100418	0.953121653	NA	NA
ASV_16s_10639	0.350883423	0.499676177	3.549476953	1.034369257	0.994254896	NA
ASV_16s_10640	0.401030874	1.4763665	3.550307855	0.762119258	NA	NA
ASV_16s_10641	0.352926355	1.949587587	3.549962378	0.4989175	0.999450435	NA
ASV_16s_10642	0.332913732	2.041121607	3.549789341	0.399663729	NA	NA

ASV_16s_10643	0.297445894	1.143780996	3.550530603	0.862052845	NA	NA
ASV_16s_10644	0.555965008	1.532163897	3.54976559	1.224764403	0.99034548	NA
ASV_16s_10645	0.378993767	-1.002485125	3.549462115	1.02095032	NA	NA
ASV_16s_10646	0.201189887	-1.038461333	3.550601165	0.362464212	NA	NA
ASV_16s_10647	0.311797831	-0.214336261	3.549768446	0.751336922	0.997909447	NA
ASV_16s_10648	0.422455158	1.336867542	3.550017959	0.82986973	NA	NA
ASV_16s_10649	0.407889292	0.418976202	3.550151508	0.838095153	NA	NA
ASV_16s_10650	0.277332503	0.418066192	3.55072218	0.714078852	NA	NA
ASV_16s_10651	0.204746785	-0.541874156	3.550292412	0.611234911	NA	NA
ASV_16s_10652	0.200844112	-0.279897109	3.550182224	0.769535502	NA	NA
ASV_16s_10653	0.148753935	1.153756594	3.551026025	0.478667785	NA	NA
ASV_16s_10654	0.308083859	-0.079037446	3.550691987	1.154108495	0.991945442	NA
ASV_16s_10655	0.202657184	0.220040349	3.55071946	0.783820661	0.997605584	NA
ASV_16s_10656	0.214242412	0.487388612	3.550665976	0.936948403	NA	NA
ASV_16s_10657	0.298370892	-0.069076594	3.550184806	1.549180604	NA	NA
ASV_16s_10658	0.37070923	0.376830328	3.551743709	0.523988536	NA	NA
ASV_16s_10659	0.31075885	-0.068090438	3.550101361	1.543528281	NA	NA
ASV_16s_10660	0.393563788	1.202583404	3.550075959	0.85961534	NA	NA
ASV_16s_10661	0.405552386	0.345300295	3.550795771	0.759848624	0.997832449	NA
ASV_16s_10662	0.38050858	-0.549111679	3.549559887	0.808786438	NA	NA
ASV_16s_10663	0.359702084	0.91186589	3.549683917	1.360222075	0.986758592	NA
ASV_16s_10664	0.320248416	-0.105727888	3.550301717	1.419528105	0.984965615	NA
ASV_16s_10665	0.541375468	0.400104046	3.549891726	2.003369215	0.959653655	NA
ASV_16s_10666	0.492940234	0.235804074	3.54898117	1.718923641	0.97375016	NA
ASV_16s_10667	0.456879538	0.641137954	3.55078563	1.720365763	NA	NA
ASV_16s_10668	0.558682098	0.337746631	3.549591653	2.104457686	NA	NA
ASV_16s_10669	0.391278366	0.346208674	3.550028754	1.630952152	NA	NA
ASV_16s_10670	0.332401461	0.329882177	3.550163212	1.554405582	0.980366144	NA
ASV_16s_10671	0.369085	0.34339687	3.549916071	1.665094197	NA	NA
ASV_16s_10672	0.374281633	0.343601989	3.549935935	1.658618823	NA	NA
ASV_16s_10673	0.345437088	0.359794591	3.550639525	0.918680825	NA	NA
ASV_16s_10674	0.469247904	0.363340428	3.550297357	1.028864887	NA	NA
ASV_16s_10675	0.450899288	0.31593942	3.550746642	1.351638384	0.987006705	NA
ASV_16s_10676	0.400124284	0.31593928	3.550766831	1.29088678	0.98868155	NA
ASV_16s_10677	0.388831381	0.315939281	3.550772025	1.276419573	0.98905963	NA
ASV_16s_10678	0.446140982	0.537720777	3.55072553	2.253835822	0.944466542	NA
ASV_16s_10679	0.458639994	0.315939418	3.550743964	1.360223752	0.986758543	NA
ASV_16s_10680	0.442579078	1.184949945	3.550186534	0.991498206	NA	NA
ASV_16s_10681	0.421950021	1.069008891	3.5504957	1.021683968	0.994471118	NA
ASV_16s_10682	0.445816294	1.184984356	3.550179485	0.994227811	0.994921113	NA
ASV_16s_10683	0.444436184	0.734773951	3.550817955	1.31966733	NA	NA
ASV_16s_10684	0.444436184	0.734773951	3.550817955	1.31966733	NA	NA
ASV_16s_10685	0.375086938	0.693501708	3.550750105	1.192877902	0.991089959	NA
ASV_16s_10686	0.387697876	0.693521335	3.550743691	1.209629105	0.990703497	NA
ASV_16s_10687	0.391871714	0.693527816	3.550741659	1.215050403	0.990576227	NA
ASV_16s_10688	0.285681887	-0.034661044	3.550272489	1.382951646	NA	NA
ASV_16s_10689	0.285710408	-0.090221481	3.550205728	1.446655229	NA	NA
ASV_16s_10690	0.292046481	-0.111216502	3.550156334	1.480519439	NA	NA
ASV_16s_10691	0.292046481	-0.111216502	3.550156334	1.480519439	NA	NA
ASV_16s_10692	0.302379541	-0.126945279	3.550098939	1.515202737	NA	NA
ASV_16s_10693	0.289266823	-0.117125561	3.550163259	1.47577803	NA	NA
ASV_16s_10694	0.304385953	-0.168276889	3.550061113	1.539909661	NA	NA
ASV_16s_10695	0.293341075	-0.163425503	3.550109318	1.508810161	NA	NA
ASV_16s_10696	0.291884531	-0.270493001	3.550071195	1.494272495	0.982507283	NA
ASV_16s_10697	0.333810996	-0.308509019	3.54991067	1.591321543	0.97897868	NA
ASV_16s_10698	0.30898579	-0.296916956	3.549998093	1.530182336	0.981246314	NA
ASV_16s_10699	0.312394323	-0.329629421	3.549982384	1.514772227	0.981793835	NA
ASV_16s_10700	0.333559537	-0.345836203	3.549909561	1.557160176	0.980264539	NA
ASV_16s_10701	0.306348665	-0.334889341	3.55000485	1.493017815	0.982550398	NA
ASV_16s_10702	0.30790492	-0.356917624	3.549999731	1.47592572	0.983131426	NA
ASV_16s_10703	0.317278131	-0.363851249	3.549965858	1.494244369	0.982508251	NA
ASV_16s_10704	0.294715527	-0.35745154	3.550051945	1.436744481	0.98441914	NA
ASV_16s_10705	0.290169069	-0.376369626	3.550073619	1.394959998	0.985725135	NA
ASV_16s_10706	0.321834324	-0.466797919	3.549978236	1.322911345	0.987816328	NA
ASV_16s_10707	0.102089091	-0.418855216	3.551415825	0.469839172	NA	NA
ASV_16s_10708	0.39282097	-0.681843116	3.549870017	0.912016529	0.996125784	NA
ASV_16s_10709	0.357157095	-0.681113273	3.549982331	0.881405425	NA	NA

ASV_16s_10710	0.303697829	-0.680376624	3.550199781	0.829378006	NA	NA
ASV_16s_10711	0.288251728	-0.679749481	3.550277249	0.812618129	NA	NA
ASV_16s_10712	0.285972795	-0.679694947	3.550289395	0.810079155	NA	NA
ASV_16s_10713	0.091112761	-0.169383036	3.550798121	0.447323884	NA	NA
ASV_16s_10714	0.089026639	-0.100752851	3.550797875	0.58960604	0.999047773	NA
ASV_16s_10715	0.31093354	0.478540215	3.549904964	1.485642399	0.98280256	NA
ASV_16s_10716	0.344212102	-0.053749344	3.549557037	1.445353205	NA	NA
ASV_16s_10717	0.327959992	-0.327838786	3.549450859	1.105044496	0.992951514	NA
ASV_16s_10718	0.414377257	1.504168954	3.549949585	0.80584378	0.997383857	NA
ASV_16s_10719	0.360463191	0.282520569	3.549740412	2.130383346	0.952272629	NA
ASV_16s_10720	0.384289802	-0.782106192	3.549506905	1.382893481	0.986089468	NA
ASV_16s_10721	0.36653768	-0.771161683	3.549598144	1.366680721	0.986570014	NA
ASV_16s_10722	0.384807139	-0.771357595	3.549546009	1.43112499	0.984598805	NA
ASV_16s_10723	0.306213527	0.772275272	3.549400951	0.741964026	NA	NA
ASV_16s_10724	0.34132228	0.19074762	3.549912871	1.643030712	0.97694107	NA
ASV_16s_10725	0.306974261	-0.150257997	3.550080026	1.263830289	NA	NA
ASV_16s_10726	0.362095587	-0.629928924	3.54996548	1.462393608	0.983583109	NA
ASV_16s_10727	0.369008543	-0.641700735	3.549931504	1.476769617	0.983103014	NA
ASV_16s_10728	0.288995037	0.484150087	3.550400025	0.768922972	NA	NA
ASV_16s_10729	0.306530754	-0.070919256	3.551046212	0.881470702	NA	NA
ASV_16s_10730	0.352637943	0.198162104	3.550204254	1.555676454	0.980319306	NA
ASV_16s_10731	0.370725551	-0.561313854	3.550118181	1.45737719	0.983748686	NA
ASV_16s_10732	0.35526517	-0.495625412	3.550264733	1.402215026	0.985503324	NA
ASV_16s_10733	0.386594786	0.168258808	3.550480502	1.420862617	0.984923676	NA
ASV_16s_10734	0.351957157	-0.11227119	3.550257311	1.470818243	0.983302769	NA
ASV_16s_10735	0.377582655	-0.89745743	3.54985395	1.025559289	NA	NA
ASV_16s_10736	0.33420895	-0.087632951	3.550642802	1.283581384	0.988873456	NA
ASV_16s_10737	0.346638294	-0.336182447	3.550521557	1.343431165	0.987241264	NA
ASV_16s_10738	0.27542739	0.711807312	3.550250101	0.840474504	NA	NA
ASV_16s_10739	0.292680121	-0.497387392	3.550519211	0.872883595	0.996627073	NA
ASV_16s_10740	0.477343043	0.672721751	3.550059385	0.92955319	NA	NA
ASV_16s_10741	0.373032034	1.668146081	3.549997458	0.845744656	NA	NA
ASV_16s_10742	0.312280541	1.659060523	3.550092657	0.602645935	NA	NA
ASV_16s_10743	0.341520601	1.926105035	3.550015041	0.493828148	NA	NA
ASV_16s_10744	0.457005993	0.872010618	3.550467647	1.117671627	0.992700653	NA
ASV_16s_10745	0.335157382	1.377395652	3.550160094	0.998022167	NA	NA
ASV_16s_10746	0.240139624	0.384906506	3.551269899	0.733346769	NA	NA
ASV_16s_10747	0.369984931	-0.617439433	3.549755169	1.065515379	NA	NA
ASV_16s_10748	0.367584122	0.969036893	3.550527943	1.092692962	0.993191558	NA
ASV_16s_10749	0.18557775	1.218497531	3.550849627	0.55304209	NA	NA
ASV_16s_10750	0.291814692	0.418771154	3.550639373	0.730486597	NA	NA
ASV_16s_10751	0.321376687	0.418837385	3.550482197	0.761461125	NA	NA
ASV_16s_10752	0.10300816	0.347844458	3.551682577	0.396441753	NA	NA
ASV_16s_10753	0.10300816	0.347844458	3.551682577	0.396441753	NA	NA
ASV_16s_10754	0.166213948	-0.525800147	3.55062148	0.545361626	NA	NA
ASV_16s_10755	0.394878712	-0.018561936	3.549458352	1.522681102	NA	NA
ASV_16s_10756	0.201863028	-0.54106115	3.550313678	0.60670452	NA	NA
ASV_16s_10757	0.185003122	-0.535200543	3.550448344	0.579041303	NA	NA
ASV_16s_10758	0.282443544	-0.078982992	3.550708077	1.110192595	NA	NA
ASV_16s_10759	0.200659715	0.220041475	3.550720883	0.778900384	0.997653366	NA
ASV_16s_10760	0.293926962	-0.58111001	3.550209721	0.667265652	0.998574804	NA
ASV_16s_10761	0.287004536	-0.483449504	3.550180351	0.796175778	NA	NA
ASV_16s_10762	0.28098993	-1.113771416	3.549872694	0.862139487	0.996756881	NA
ASV_16s_10763	0.296989764	-0.069189566	3.550278208	1.514415284	0.981806403	NA
ASV_16s_10764	0.293840465	-0.06907745	3.550219366	1.528405488	NA	NA
ASV_16s_10765	0.298397813	-0.270746095	3.549477857	1.13042291	NA	NA
ASV_16s_10766	0.318142909	-0.069179524	3.550157775	1.597894825	NA	NA
ASV_16s_10767	0.320957002	-0.068124369	3.550078589	1.546618732	NA	NA
ASV_16s_10768	0.313582646	0.505121949	3.550503802	1.231326193	NA	NA
ASV_16s_10769	0.291473997	0.36413714	3.551567728	0.59698267	NA	NA
ASV_16s_10770	0.417276082	1.19847184	3.55037594	0.867838219	NA	NA
ASV_16s_10771	0.54022451	-0.104091484	3.549484013	1.807679209	0.969714262	NA
ASV_16s_10772	0.345501788	-0.112394914	3.550212771	1.26472244	0.989359543	NA
ASV_16s_10773	0.3372344	-0.275016051	3.550621339	1.026953508	0.994381942	NA
ASV_16s_10774	0.553922245	0.559600086	3.549708712	1.879549877	NA	NA
ASV_16s_10775	0.384963726	0.358729967	3.550220449	1.543292891	0.980772899	NA
ASV_16s_10776	0.385934048	0.354841612	3.550126193	1.622449812	0.977765268	NA

ASV_16s_10777	0.421504647	0.346706275	3.550011145	1.646058839	0.976818326	NA
ASV_16s_10778	0.356740328	0.360387836	3.550878842	1.27175431	NA	NA
ASV_16s_10779	0.33778976	0.346422314	3.55076841	0.933780158	NA	NA
ASV_16s_10780	0.317129827	0.360376946	3.55091432	1.212086956	NA	NA
ASV_16s_10781	0.317129827	0.360376946	3.55091432	1.212086956	NA	NA
ASV_16s_10782	0.372442835	0.315939312	3.55078013	1.254602032	0.989614885	NA
ASV_16s_10783	0.366186468	0.315939313	3.550783416	1.246010126	0.989828656	NA
ASV_16s_10784	0.446639568	0.315939306	3.550748172	1.346701949	0.987148099	NA
ASV_16s_10785	0.385180755	0.31593931	3.550773775	1.271614033	0.989183467	NA
ASV_16s_10786	0.344233381	0.31593947	3.550795888	1.214673231	0.990585116	NA
ASV_16s_10787	0.398186168	0.315939309	3.550767704	1.288417253	0.988746648	NA
ASV_16s_10788	0.394694563	0.315939309	3.550769293	1.283975183	0.988863163	NA
ASV_16s_10789	0.410584153	0.315939442	3.550762274	1.303939668	0.988333619	NA
ASV_16s_10790	0.400980382	0.917421411	3.550356539	1.810643101	0.969573812	NA
ASV_16s_10791	0.503893768	1.179933752	3.550046027	1.023058551	0.994447944	NA
ASV_16s_10792	0.438176519	0.734762065	3.550821412	1.31244676	NA	NA
ASV_16s_10793	0.404304181	0.69355106	3.55073585	1.2309052	0.990197817	NA
ASV_16s_10794	0.444299385	0.693607891	3.550719366	1.278756715	0.988999088	NA
ASV_16s_10795	0.356244831	0.693466101	3.550760539	1.166736285	0.991672729	NA
ASV_16s_10796	0.374048717	0.693497308	3.550750657	1.191440652	0.991122641	NA
ASV_16s_10797	0.423983585	0.693578285	3.550727354	1.254985966	0.989605268	NA
ASV_16s_10798	0.346262678	0.693445533	3.550766523	1.152337142	0.991983242	NA
ASV_16s_10799	0.408635397	0.693557722	3.550733916	1.236261504	0.990067879	NA
ASV_16s_10800	0.286413347	-0.047530131	3.550252124	1.393791789	NA	NA
ASV_16s_10801	0.287190103	-0.082037975	3.550207665	1.441210177	NA	NA
ASV_16s_10802	0.28163649	-0.104705931	3.550210169	1.444439459	NA	NA
ASV_16s_10803	0.288743616	-0.14698043	3.550140919	1.488990265	NA	NA
ASV_16s_10804	0.289261454	-0.167838124	3.550123998	1.497344525	NA	NA
ASV_16s_10805	0.298345552	-0.208686045	3.550063619	1.5294111	0.981273945	NA
ASV_16s_10806	0.29003821	-0.198706646	3.550102847	1.504217174	0.982163302	NA
ASV_16s_10807	0.294755132	-0.21421534	3.550075903	1.518212299	0.981672444	NA
ASV_16s_10808	0.300681306	-0.220997242	3.550049213	1.535550007	0.981053336	NA
ASV_16s_10809	0.317190491	-0.256914624	3.54997659	1.574810831	0.979606123	NA
ASV_16s_10810	0.285497457	-0.223277178	3.55011166	1.488654781	0.982699833	NA
ASV_16s_10811	0.286769235	-0.234841551	3.550101952	1.49056585	0.982634473	NA
ASV_16s_10812	0.310192416	-0.297990809	3.549993468	1.532909336	0.981148419	NA
ASV_16s_10813	0.313996108	-0.311103524	3.549977859	1.5343894	0.981095161	NA
ASV_16s_10814	0.30536148	-0.303594037	3.550010835	1.515114785	0.981781769	NA
ASV_16s_10815	0.303802483	-0.322492721	3.550015052	1.496291897	0.982437756	NA
ASV_16s_10816	0.288334156	-0.319442571	3.550078179	1.453226626	0.983884922	NA
ASV_16s_10817	0.312249945	-0.33960057	3.549982842	1.505246172	0.982127482	NA
ASV_16s_10818	0.318800922	-0.364939868	3.549960576	1.497124752	0.982409034	NA
ASV_16s_10819	0.33085058	-0.373301635	3.549920291	1.51949006	0.981627234	NA
ASV_16s_10820	0.289870557	-0.353552858	3.550071997	1.426521812	0.984745043	NA
ASV_16s_10821	0.366427864	-0.68148142	3.549951154	0.889650904	0.996417807	NA
ASV_16s_10822	0.364573282	-0.681454017	3.549957292	0.888020045	NA	NA
ASV_16s_10823	0.293788564	-0.680157249	3.550248703	0.818727228	NA	NA
ASV_16s_10824	0.08981115	-0.169366318	3.550802336	0.441571206	NA	NA
ASV_16s_10825	0.149434741	0.1745256	3.550749112	0.842157224	0.996989545	NA
ASV_16s_10826	0.28572627	0.001157902	3.550096017	1.429678981	0.984644834	NA
ASV_16s_10827	0.31079327	0.109405498	3.54981766	1.538999372	0.980928706	NA
ASV_16s_10828	0.291868624	-0.020295992	3.54989105	1.395897794	0.98569658	NA
ASV_16s_10829	0.373574375	-0.778955553	3.549721496	1.043801388	0.994090677	NA
ASV_16s_10830	0.141080427	1.193008692	3.551468852	0.381451239	0.999775294	NA
ASV_16s_10831	0.366163895	-0.784196704	3.54968615	1.096650401	0.99311522	NA
ASV_16s_10832	0.367540381	-0.790644519	3.549602466	1.191928628	0.991111553	NA
ASV_16s_10833	0.375542765	-0.790638908	3.54953629	1.246913965	0.989806297	NA
ASV_16s_10834	0.375090795	-0.7565177	3.549636276	1.436659621	0.984421862	NA
ASV_16s_10835	0.373984128	-0.716961077	3.549743044	1.471399923	NA	NA
ASV_16s_10836	0.350331501	-0.681405685	3.549872397	1.426071232	0.984759312	NA
ASV_16s_10837	0.325750514	-0.508884805	3.549374094	0.774578103	0.997694819	NA
ASV_16s_10838	0.363211651	-0.599410016	3.550033673	1.455043441	0.983825372	NA
ASV_16s_10839	0.396629797	-0.658396469	3.549671895	1.161980433	0.991776107	NA
ASV_16s_10840	0.191817172	-0.521669393	3.55100415	0.679380508	NA	NA
ASV_16s_10841	0.356006515	0.18250445	3.549500542	1.412584742	NA	NA
ASV_16s_10842	0.329574369	-0.093826171	3.550658648	1.237069428	NA	NA
ASV_16s_10843	0.345554604	0.347133701	3.551204926	0.810897038	NA	NA

ASV_16s_10844	0.320991668	0.063543441	3.55055594	1.234925239	NA	NA
ASV_16s_10845	0.368183091	0.230293962	3.54943084	1.282713942	NA	NA
ASV_16s_10846	0.38124909	1.76843388	3.550053712	0.801815728	NA	NA
ASV_16s_10847	0.330618221	1.714418336	3.550331033	0.573841625	NA	NA
ASV_16s_10848	0.448651739	0.489039399	3.549930781	0.961476351	NA	NA
ASV_16s_10849	0.417319014	0.539940393	3.550644522	1.129980272	0.992450766	NA
ASV_16s_10850	0.321309696	0.183132252	3.54970562	1.410311703	0.985253332	NA
ASV_16s_10851	0.404058095	-0.363365083	3.326093523	0.910772981	0.996142412	NA
ASV_16s_10852	0.279472569	0.541989676	3.549611288	0.931565593	NA	NA
ASV_16s_10853	0.301872206	1.303139821	3.550316892	1.039961248	NA	NA
ASV_16s_10854	0.257055721	0.635042317	3.551021731	0.987280608	NA	NA
ASV_16s_10855	0.248836184	-0.171617471	3.550112959	1.090748631	NA	NA
ASV_16s_10856	0.399821847	0.719893667	3.549631055	1.535151799	NA	NA
ASV_16s_10857	0.236709058	0.384257546	3.551290192	0.728490372	NA	NA
ASV_16s_10858	0.273301087	0.217807629	3.550785668	0.386843922	0.999764468	NA
ASV_16s_10859	0.346153317	-0.104043547	3.549151862	1.455353188	NA	NA
ASV_16s_10860	0.245492612	-0.8049869	3.550269538	0.749736262	0.997923722	NA
ASV_16s_10861	0.341367999	0.418875633	3.550391097	0.780847864	0.997634529	NA
ASV_16s_10862	0.181189777	-0.368530154	3.550359343	0.732960184	NA	NA
ASV_16s_10863	0.163839463	-0.523539904	3.550642081	0.540773871	NA	NA
ASV_16s_10864	0.19897927	-0.54022848	3.550335513	0.602123334	NA	NA
ASV_16s_10865	0.18236022	-0.534057592	3.550471248	0.574463131	NA	NA
ASV_16s_10866	0.18236022	-0.534057592	3.550471248	0.574463131	NA	NA
ASV_16s_10867	0.18236022	-0.534057592	3.550471248	0.574463131	NA	NA
ASV_16s_10868	0.278408636	-0.07897371	3.550710881	1.102914872	NA	NA
ASV_16s_10869	0.196739462	0.220043517	3.550723758	0.769142861	0.997746256	NA
ASV_16s_10870	0.207265604	0.220038002	3.550716289	0.794925237	NA	NA
ASV_16s_10871	0.228266036	0.276433022	3.550646044	0.756030129	NA	NA
ASV_16s_10872	0.385208628	0.338282151	3.551991018	0.331209722	0.999860247	NA
ASV_16s_10873	0.296126178	-0.069336827	3.550396418	1.473009854	0.983229374	NA
ASV_16s_10874	0.288814521	-0.069152717	3.550289567	1.494071831	NA	NA
ASV_16s_10875	0.288814521	-0.069152717	3.550289567	1.494071831	NA	NA
ASV_16s_10876	0.299220406	-0.068467819	3.550054191	1.55139684	NA	NA
ASV_16s_10877	0.300211442	-0.068346624	3.55007305	1.541818078	NA	NA
ASV_16s_10878	0.343749013	0.135069657	3.549902217	2.214448564	0.947024012	NA
ASV_16s_10879	0.352854042	0.309386735	3.550320305	0.871677851	0.996641806	NA
ASV_16s_10880	0.401617904	0.42997209	3.549415999	1.277895669	0.989021416	NA
ASV_16s_10881	0.312995824	-0.067930569	3.550136847	1.50850817	NA	NA
ASV_16s_10882	0.307148699	-0.048270462	3.550556195	1.352573537	NA	NA
ASV_16s_10883	0.139381473	0.281250927	3.551658031	0.560818833	NA	NA
ASV_16s_10884	0.27451476	-0.596420088	3.550846759	0.784879667	NA	NA
ASV_16s_10885	0.388597849	0.385590513	3.55072846	1.264482544	0.98936564	NA
ASV_16s_10886	0.359183413	1.260531251	3.549888794	1.368029196	NA	NA
ASV_16s_10887	0.348288306	1.120355294	3.55016207	1.316246837	NA	NA
ASV_16s_10888	0.372883594	0.635831094	3.55011446	2.244381237	0.945086125	NA
ASV_16s_10889	0.312944596	0.337811988	3.550033515	1.568853371	NA	NA
ASV_16s_10890	0.413629208	0.784035302	3.549368233	1.432860947	0.984543438	NA
ASV_16s_10891	0.405129671	0.719387895	3.55069034	1.354398249	0.986927243	NA
ASV_16s_10892	0.443275933	0.363326648	3.550357333	1.008177214	0.994695546	NA
ASV_16s_10893	0.360726833	0.340551074	3.550793789	0.991967048	0.99495708	NA
ASV_16s_10894	0.312599401	0.360375531	3.550918953	1.204774703	NA	NA
ASV_16s_10895	0.352371885	0.502635805	3.550854859	2.044931614	NA	NA
ASV_16s_10896	0.35792105	0.315939341	3.550787937	1.2344089	0.990112941	NA
ASV_16s_10897	0.339159329	0.315939486	3.550799004	1.207123912	0.990761945	NA
ASV_16s_10898	0.337106156	0.315939487	3.550800289	1.204046058	0.99083344	NA
ASV_16s_10899	0.365495408	0.31593934	3.550783799	1.244965329	0.989854463	NA
ASV_16s_10900	0.439613715	0.596303447	3.550612467	1.105180606	NA	NA
ASV_16s_10901	0.423839255	1.185018837	3.55017204	0.957523702	0.995484947	NA
ASV_16s_10902	0.434192102	0.734753407	3.550823665	1.307787529	0.988229814	NA
ASV_16s_10903	0.431916854	0.734748883	3.550824968	1.305122481	NA	NA
ASV_16s_10904	0.431916854	0.734748883	3.550824968	1.305122481	NA	NA
ASV_16s_10905	0.431916854	0.734748883	3.550824968	1.305122481	NA	NA
ASV_16s_10906	0.356042059	0.898260416	3.55073013	1.033858225	0.994263709	NA
ASV_16s_10907	0.406661582	0.693554913	3.550734791	1.23383695	0.990126827	NA
ASV_16s_10908	0.409964061	0.693559963	3.550733324	1.23793956	0.990026952	NA
ASV_16s_10909	0.384123558	0.69351815	3.550745471	1.204905593	0.990813509	NA
ASV_16s_10910	0.3678381	0.69348882	3.550753999	1.182939751	0.991314416	NA

ASV_16s_10911	0.365332645	0.693484079	3.550755378	1.17947539	0.991391819	NA
ASV_16s_10912	0.277539756	-0.075511256	3.550261437	1.409680818	NA	NA
ASV_16s_10913	0.280589742	-0.102920961	3.550216989	1.439905228	NA	NA
ASV_16s_10914	0.284146157	-0.129841567	3.550175415	1.466879785	NA	NA
ASV_16s_10915	0.301455217	-0.174044455	3.550069305	1.532415796	NA	NA
ASV_16s_10916	0.277579685	-0.151279394	3.550188874	1.455537088	NA	NA
ASV_16s_10917	0.28569967	-0.193249389	3.550124604	1.490319608	0.982642903	NA
ASV_16s_10918	0.296842833	-0.226338338	3.550062716	1.521035909	0.981572449	NA
ASV_16s_10919	0.297113818	-0.236380574	3.550058156	1.521870558	0.981542828	NA
ASV_16s_10920	0.281929969	-0.239521456	3.550121854	1.47427427	0.983186942	NA
ASV_16s_10921	0.283207451	-0.261502223	3.550110365	1.471215323	0.983289486	NA
ASV_16s_10922	0.277659542	-0.298450622	3.550127852	1.435038077	NA	NA
ASV_16s_10923	0.311006803	-0.348854889	3.549987715	1.492712061	0.982560896	NA
ASV_16s_10924	0.295600453	-0.336478027	3.550047372	1.460476647	0.983646501	NA
ASV_16s_10925	0.28298846	-0.32549333	3.550101309	1.432251648	0.984562885	NA
ASV_16s_10926	0.281745203	-0.369795009	3.55010974	1.37753145	0.986249536	NA
ASV_16s_10927	0.369444644	-0.681511687	3.549941339	0.892266673	0.996384419	NA
ASV_16s_10928	0.284132513	-0.679930854	3.55029961	0.808015235	NA	NA
ASV_16s_10929	0.179818879	0.220054204	3.550737596	0.725062514	NA	NA
ASV_16s_10930	0.093325671	0.220167061	3.550886252	0.431575818	NA	NA
ASV_16s_10931	0.093325671	0.220167061	3.550886252	0.431575818	NA	NA
ASV_16s_10933	0.19657011	0.596101515	3.550689709	0.701212135	NA	NA
ASV_16s_10934	0.19657011	0.596101515	3.550689709	0.701212135	NA	NA
ASV_16s_10935	0.314549937	-0.176587471	3.550017644	1.569699444	0.979798102	NA
ASV_16s_10936	0.296485944	-0.0124501	3.549986625	1.482713602	0.982902086	NA
ASV_16s_10937	0.304404072	0.074645037	3.549949645	1.533611436	0.981123166	NA
ASV_16s_10938	0.280802353	-0.398082447	3.55004507	0.829458398	NA	NA
ASV_16s_10939	0.305238906	0.203196066	3.54958881	1.619923371	0.977865239	NA
ASV_16s_10940	0.288230068	-0.016157193	3.55004122	0.885147581	NA	NA
ASV_16s_10941	0.315033787	0.07927237	3.549716767	0.951220779	0.995577505	NA
ASV_16s_10942	0.366369	-0.788000612	3.549579526	1.255505682	0.989592241	NA
ASV_16s_10943	0.303021968	-0.327970611	3.54995595	0.954044837	NA	NA
ASV_16s_10944	0.290458963	0.2898816	3.551715014	0.625289448	NA	NA
ASV_16s_10945	0.396561949	-0.790856369	3.54941867	1.328434364	0.987663142	NA
ASV_16s_10946	0.370907255	0.715186112	3.548922189	1.559126535	NA	NA
ASV_16s_10947	0.291285723	-0.626459578	3.550253234	1.204921089	NA	NA
ASV_16s_10948	0.29981789	-0.662413543	3.550124812	1.296080057	NA	NA
ASV_16s_10949	0.3152996	0.57766175	3.549885721	1.171725941	0.991563397	NA
ASV_16s_10950	0.236581936	0.388809944	3.549974933	1.035263107	NA	NA
ASV_16s_10951	0.454110476	1.401081485	3.550010187	1.213430265	NA	NA
ASV_16s_10952	0.403802434	-1.493540435	3.548857563	0.955209791	0.99551907	NA
ASV_16s_10953	0.355001967	-0.69546889	3.549826629	1.432106806	0.984567505	NA
ASV_16s_10954	0.358401634	-0.692673916	3.549828901	1.442568135	0.984231621	NA
ASV_16s_10955	0.30350366	0.685788039	3.550797823	1.260911612	NA	NA
ASV_16s_10956	0.347698306	-0.614971797	3.550015095	1.430617985	0.984614953	NA
ASV_16s_10957	0.36243088	0.371086083	3.550371529	1.318715088	0.987931932	NA
ASV_16s_10958	0.428059933	-0.51930445	3.549308963	1.701729953	0.974494024	NA
ASV_16s_10959	0.34202613	0.182367827	3.549554179	1.320961477	0.98787013	NA
ASV_16s_10960	0.343937879	-0.008226948	3.550500714	1.266715713	0.9893088	NA
ASV_16s_10961	0.403108033	0.414808216	3.550800235	1.347214587	NA	NA
ASV_16s_10962	0.339591483	-0.271449397	3.550216037	0.933363122	0.99583307	NA
ASV_16s_10963	0.350203717	-0.160920229	3.550659966	1.302101328	0.988383013	NA
ASV_16s_10964	0.381812168	-0.161742042	3.551703488	0.468081993	NA	NA
ASV_16s_10965	0.413284579	1.485303694	2.588685906	1.250907207	0.989707151	NA
ASV_16s_10966	0.321729308	0.28051743	3.550143368	1.137197093	0.992301779	NA
ASV_16s_10967	0.281103837	-0.111206968	3.550085677	0.694823428	0.998375196	NA
ASV_16s_10968	0.384911184	1.721781239	3.549971185	0.966741387	0.99534735	NA
ASV_16s_10969	0.328452062	1.318981916	3.550472698	0.810263435	NA	NA
ASV_16s_10970	0.301027706	0.397714913	3.550923182	0.808577172	NA	NA
ASV_16s_10971	0.426158334	0.396966001	3.551439661	0.612776203	NA	NA
ASV_16s_10972	0.335378203	-0.564524964	3.549888114	0.838774663	NA	NA
ASV_16s_10973	0.331897364	-0.19817952	3.549357274	1.027251422	NA	NA
ASV_16s_10974	0.304041722	-0.760774696	3.549503964	0.932725835	NA	NA
ASV_16s_10975	0.273266303	-0.286042532	3.549398481	0.908850933	NA	NA
ASV_16s_10976	0.273982012	1.307259762	3.550511571	0.687203172	NA	NA
ASV_16s_10977	0.225836992	0.856223472	3.55041981	0.824444078	NA	NA
ASV_16s_10978	0.265614229	0.416511911	3.550786766	0.699882524	NA	NA

ASV_16s_10979	0.10006507	0.347003787	3.551693314	0.388192845	NA	NA
ASV_16s_10980	0.368017897	0.373710651	3.549550568	1.581544503	0.979351579	NA
ASV_16s_10981	0.161464978	-0.520979285	3.550662178	0.53609936	NA	NA
ASV_16s_10982	0.161464978	-0.520979285	3.550662178	0.53609936	NA	NA
ASV_16s_10983	0.404276822	-0.149944428	3.549444241	1.411294316	NA	NA
ASV_16s_10984	0.179717318	-0.532897193	3.550494777	0.56983596	NA	NA
ASV_16s_10985	0.142468557	1.139996793	3.551058179	0.464495385	NA	NA
ASV_16s_10986	0.300857776	-0.079020946	3.550696253	1.142040283	0.992200764	NA
ASV_16s_10987	0.194035082	0.220045081	3.55072581	0.76230585	0.997809877	NA
ASV_16s_10988	0.19851049	0.220042581	3.550722449	0.77354138	0.997704689	NA
ASV_16s_10989	0.322138751	-0.954214318	3.55031066	0.767813175	0.997758724	NA
ASV_16s_10990	0.319990926	0.358233121	3.551818093	0.548546443	0.999248595	NA
ASV_16s_10991	0.284284095	-0.069154915	3.550331866	1.470887937	NA	NA
ASV_16s_10992	0.297156571	-0.069108809	3.55021445	1.536784547	0.981008786	NA
ASV_16s_10993	0.28874376	-0.068918989	3.55010616	1.54211473	NA	NA
ASV_16s_10994	0.372607919	1.901308139	3.549986068	0.541345623	NA	NA
ASV_16s_10995	0.362975751	0.663264447	3.551145712	1.360887128	0.986739248	NA
ASV_16s_10996	0.303532238	-0.068689142	3.550019405	1.582001765	0.979334226	NA
ASV_16s_10997	0.360723708	0.717443227	3.549917694	1.608541905	0.978312341	NA
ASV_16s_10998	0.239383899	0.297073783	3.551668526	0.47344277	NA	NA
ASV_16s_10999	0.241947699	0.279773473	3.551457414	0.758560801	NA	NA
ASV_16s_11000	0.137361452	0.281396576	3.55166166	0.555791939	NA	NA
ASV_16s_11001	0.137361452	0.281396576	3.55166166	0.555791939	NA	NA
ASV_16s_11002	0.192613568	0.279452635	3.551559712	0.675543262	NA	NA
ASV_16s_11003	0.233771596	-0.56810146	3.551002163	0.730483888	NA	NA
ASV_16s_11004	0.364430753	-0.54896012	3.549598286	0.794881435	NA	NA
ASV_16s_11005	0.374012356	1.10021157	3.550186354	1.359200222	0.986788278	NA
ASV_16s_11006	0.397596239	0.744884083	3.550662297	1.294072523	0.98859723	NA
ASV_16s_11007	0.496011864	0.869498372	3.550030727	1.817216355	0.969261017	NA
ASV_16s_11008	0.394372122	0.343680075	3.549871494	1.758809964	0.971977114	NA
ASV_16s_11009	0.394585166	0.409228311	3.550523479	1.404407632	0.98543588	NA
ASV_16s_11010	0.403714768	0.343754797	3.549912687	1.679983106	NA	NA
ASV_16s_11011	0.332117789	0.342697191	3.549930546	1.625138258	NA	NA
ASV_16s_11012	0.350306006	0.343307191	3.549942719	1.627771495	NA	NA
ASV_16s_11013	0.37909658	0.771039931	3.54949371	1.414559204	0.985121151	NA
ASV_16s_11014	0.399637571	0.317876612	3.550250076	1.786728828	0.970696567	NA
ASV_16s_11015	0.375869765	0.34294678	3.549849459	1.722621817	NA	NA
ASV_16s_11016	0.406772305	0.36754143	3.550310367	1.492517111	NA	NA
ASV_16s_11017	0.308068974	0.360374004	3.550923721	1.197356227	NA	NA
ASV_16s_11018	0.344872273	0.31593937	3.550795511	1.215555359	0.990564318	NA
ASV_16s_11019	0.345564044	0.315939369	3.550795095	1.216573878	0.990540268	NA
ASV_16s_11020	0.378694982	0.553035555	3.550749544	2.024052789	0.958497104	NA
ASV_16s_11021	0.332009964	0.315939503	3.550803554	1.196295559	0.991011942	NA
ASV_16s_11022	0.357570068	0.315939387	3.550788142	1.233854098	0.990126411	NA
ASV_16s_11023	0.329636734	0.315939504	3.550805105	1.192667765	0.991094742	NA
ASV_16s_11024	0.415909938	0.315939229	3.550760038	1.310468785	0.988157147	NA
ASV_16s_11025	0.397809569	1.032081923	3.550055402	0.752640225	NA	NA
ASV_16s_11026	0.459357472	1.152205381	3.550282324	1.013222547	NA	NA
ASV_16s_11027	0.42565719	0.734736344	3.550828628	1.297691674	NA	NA
ASV_16s_11028	0.395729582	1.153220166	3.550343381	0.947096691	0.9956374	NA
ASV_16s_11029	0.2108517	0.733853624	3.551084532	0.944097017	NA	NA
ASV_16s_11030	0.367445586	0.693488269	3.550754216	1.182382527	0.991326895	NA
ASV_16s_11031	0.386731871	0.693520046	3.550744173	1.208322901	0.990734001	NA
ASV_16s_11032	0.401832866	0.693547548	3.550736981	1.227756235	0.990273712	NA
ASV_16s_11033	0.352219136	0.693458373	3.550762919	1.160932391	0.991798779	NA
ASV_16s_11034	0.277517275	-0.097624863	3.550237337	1.42629816	NA	NA
ASV_16s_11035	0.276515824	-0.117676322	3.550222464	1.436279131	NA	NA
ASV_16s_11036	0.28136113	-0.187619859	3.55014725	1.476031302	0.983127873	NA
ASV_16s_11037	0.281878967	-0.208715604	3.550134026	1.478461483	0.983045968	NA
ASV_16s_11038	0.279352524	-0.215878206	3.550142364	1.469978682	0.983330834	NA
ASV_16s_11039	0.273534288	-0.219139455	3.550168259	1.451039232	0.983956443	NA
ASV_16s_11040	0.280641414	-0.258740619	3.550122512	1.464152426	0.983524817	NA
ASV_16s_11041	0.283691399	-0.282941049	3.550103494	1.462711274	NA	NA
ASV_16s_11042	0.284209237	-0.304827348	3.550097516	1.451123682	NA	NA
ASV_16s_11043	0.2779245	-0.390724678	3.550131713	1.334057462	0.987505974	NA
ASV_16s_11044	0.279708411	-0.416943514	3.550131393	1.297825756	0.988497395	NA
ASV_16s_11045	0.097713845	-0.404306636	3.551456761	0.456888864	NA	NA

ASV_16s_11046	0.304613763	-0.680391997	3.550195672	0.830262908	NA		NA
ASV_16s_11047	0.273716818	-0.67882746	3.550356747	0.795909762	NA		NA
ASV_16s_11048	0.273716818	-0.67882746	3.550356747	0.795909762	NA		NA
ASV_16s_11049	0.273716818	-0.67882746	3.550356747	0.795909762	NA		NA
ASV_16s_11050	0.127000226	0.153572916	3.550737258	1.101440504		0.993022099	NA
ASV_16s_11051	0.121570866	0.220112667	3.55081455	0.542493866	NA		NA
ASV_16s_11053	0.374001134	-0.705847244	3.549899526	0.916827715		0.996061014	NA
ASV_16s_11054	0.292622574	0.13094967	3.550015881	1.407473811		0.985341248	NA
ASV_16s_11055	0.283770403	0.29237825	3.551747834	0.621635479	NA		NA
ASV_16s_11056	0.337489396	-0.782041015	3.549754178	1.164819569	NA		NA
ASV_16s_11057	0.347777403	-1.021936264	3.54987919	0.951024923		0.995580362	NA
ASV_16s_11058	0.317425217	1.169653692	3.550679997	0.994944629	NA		NA
ASV_16s_11059	0.348465744	-0.764830034	3.54967828	1.319316193		0.987915414	NA
ASV_16s_11060	0.306380826	-0.382794298	3.550114031	1.213119613	NA		NA
ASV_16s_11061	0.374144696	-0.755671036	3.549641454	1.434932228		0.984477218	NA
ASV_16s_11062	0.336760354	-0.716681364	3.549813075	1.363635492	NA		NA
ASV_16s_11063	0.353134173	-0.681705138	3.549867366	1.433249068		0.984531042	NA
ASV_16s_11064	0.358148773	-0.689450672	3.549838159	1.443239936		0.984209903	NA
ASV_16s_11065	0.335500398	-0.628888743	3.550000198	1.399436808		0.985588508	NA
ASV_16s_11066	0.310208416	0.887758227	3.549566599	0.918168301		0.996042842	NA
ASV_16s_11067	0.348053995	-0.772380849	3.549639577	1.11799845		0.992694086	NA
ASV_16s_11068	0.34673207	-0.61205996	3.550022595	1.421254776		0.984911338	NA
ASV_16s_11069	0.362247963	-0.651541309	3.549917805	1.454462367		0.983844433	NA
ASV_16s_11070	0.301789113	-0.090664879	3.550257797	1.241308314	NA		NA
ASV_16s_11071	0.332741141	-0.399680269	3.550298431	1.351288396	NA		NA
ASV_16s_11072	0.284214195	-0.681442939	3.550400901	0.737128366	NA		NA
ASV_16s_11073	0.63030354	-0.409342731	3.550510901	1.294728163		0.988579829	NA
ASV_16s_11074	0.322231883	-0.061949784	3.550597901	1.064346924	NA		NA
ASV_16s_11075	0.207346632	0.215506907	3.550754504	0.5867179	NA		NA
ASV_16s_11076	0.366020542	0.020938185	3.550869158	0.849326487		0.99690737	NA
ASV_16s_11077	0.291433908	-0.04803226	3.550542913	0.68653904	NA		NA
ASV_16s_11078	0.381366563	0.315557037	3.549639166	1.110055192		0.99285263	NA
ASV_16s_11079	0.340978561	1.322936518	3.55042714	0.822713297	NA		NA
ASV_16s_11080	0.320244263	1.755761211	3.550427054	0.415775642		0.999700198	NA
ASV_16s_11081	0.334109893	-0.338906942	3.550545878	0.826005369		0.997169414	NA
ASV_16s_11082	0.250494373	0.387180919	3.551209012	0.747445422	NA		NA
ASV_16s_11083	0.38259015	0.359191071	3.551748312	0.499619281	NA		NA
ASV_16s_11084	0.262744893	-0.117091896	3.550915225	0.375972826		0.999785936	NA
ASV_16s_11085	0.400083548	-0.565300666	3.549694316	0.895412797	NA		NA
ASV_16s_11086	0.325847696	-0.006402434	3.54996089	1.162210482	NA		NA
ASV_16s_11087	0.261708137	0.415769398	3.55080753	0.694968995	NA		NA
ASV_16s_11088	0.098593524	0.346757797	3.551697646	0.383994232	NA		NA
ASV_16s_11089	0.177074416	-0.531718807	3.550518959	0.565150116	NA		NA
ASV_16s_11090	0.140373432	1.135104891	3.551068485	0.459657212	NA		NA
ASV_16s_11091	0.140373432	1.135104891	3.551068485	0.459657212	NA		NA
ASV_16s_11092	0.191420679	0.220046658	3.550727849	0.755619407		0.997870937	NA
ASV_16s_11093	0.201257905	0.220041187	3.550720462	0.780313744	NA		NA
ASV_16s_11094	0.201257905	0.220041187	3.550720462	0.780313744	NA		NA
ASV_16s_11095	0.233101635	0.209836549	3.55050315	0.565043086	NA		NA
ASV_16s_11096	0.347127931	0.31093308	3.550776637	1.196010187		0.991018472	NA
ASV_16s_11097	0.21200932	0.596128028	3.550679549	0.738148086	NA		NA
ASV_16s_11098	0.307272997	1.342753365	3.550573063	0.771174246	NA		NA
ASV_16s_11099	0.228655877	-0.605241916	3.550444856	0.505196943	NA		NA
ASV_16s_11100	0.332726586	0.342564708	3.550737459	0.899185607		0.996295131	NA
ASV_16s_11101	0.322605329	0.759778991	3.551514224	0.852643712	NA		NA
ASV_16s_11102	0.287681962	-0.068697204	3.550068139	1.537950186	NA		NA
ASV_16s_11103	0.304199254	-0.068783675	3.550015656	1.590051256	NA		NA
ASV_16s_11104	0.308812084	0.7832412	3.55121289	0.893012121	NA		NA
ASV_16s_11105	0.290159554	-0.068460182	3.550077111	1.527401262	NA		NA
ASV_16s_11106	0.379042777	0.336488043	3.551780232	0.379930076		0.999778285	NA
ASV_16s_11107	0.358733899	0.320004802	3.550874253	0.957986104		0.995478107	NA
ASV_16s_11108	0.36894401	0.77440355	3.550590324	1.247921214	NA		NA
ASV_16s_11109	0.448318006	-0.607147561	3.550109577	0.943744655	NA		NA
ASV_16s_11110	0.517306575	-0.201480515	3.548630616	1.601607833	NA		NA
ASV_16s_11111	0.435169921	-0.039610163	3.550241166	1.544603946		0.980725173	NA
ASV_16s_11112	0.392449448	1.160240678	3.55002084	1.122979681		0.992593541	NA
ASV_16s_11113	0.226874515	-0.078823319	3.550755389	1.000137973	NA		NA

ASV_16s_11114	0.279944196	-0.103534928	3.550155695	1.248980772	NA	NA
ASV_16s_11115	0.421894457	0.201033889	3.55006302	1.842937424	NA	NA
ASV_16s_11116	0.440615087	0.550487927	3.550662538	1.344682796	0.987205661	NA
ASV_16s_11117	0.416476397	0.269940947	3.550092825	1.728954214	NA	NA
ASV_16s_11118	0.33849058	0.235173929	3.549978201	1.64828135	NA	NA
ASV_16s_11119	0.299329122	-0.029171226	3.550790775	1.198650923	NA	NA
ASV_16s_11120	0.402074561	0.34690934	3.550032093	1.601389098	NA	NA
ASV_16s_11121	0.24878662	-0.062330206	3.550731659	1.111601139	NA	NA
ASV_16s_11122	0.267725949	0.232886977	3.550596115	1.219634136	NA	NA
ASV_16s_11123	0.325858124	0.342648038	3.549945174	1.609606565	NA	NA
ASV_16s_11124	0.322500889	0.343051482	3.550817791	0.927601932	NA	NA
ASV_16s_11125	0.404901079	0.62954394	3.549639216	1.35805475	0.986821509	NA
ASV_16s_11126	0.303538548	0.360372511	3.550928631	1.18982846	NA	NA
ASV_16s_11127	0.346270138	0.315939391	3.550794689	1.217504625	0.990518257	NA
ASV_16s_11128	0.385712026	0.802705048	3.550730282	1.096931331	0.993109781	NA
ASV_16s_11129	0.419397525	0.734723408	3.550832398	1.290151083	NA	NA
ASV_16s_11130	0.419397525	0.734723408	3.550832398	1.290151083	NA	NA
ASV_16s_11131	0.20775094	0.733827776	3.551092076	0.936799155	NA	NA
ASV_16s_11132	0.396343654	0.693538919	3.550739535	1.220757662	0.990441077	NA
ASV_16s_11133	0.377621004	0.693504709	3.550748794	1.196208421	0.991013936	NA
ASV_16s_11134	0.361996042	0.693475509	3.550757251	1.174782317	0.991495985	NA
ASV_16s_11135	0.269255024	0.272356845	3.551105205	0.770868285	NA	NA
ASV_16s_11136	0.292500577	-0.036966672	3.550237402	1.370141103	NA	NA
ASV_16s_11137	0.274174482	-0.057547043	3.550300458	1.366762049	NA	NA
ASV_16s_11138	0.274433401	-0.069588393	3.550284241	1.382846297	NA	NA
ASV_16s_11139	0.274951238	-0.093085072	3.550254918	1.411991975	NA	NA
ASV_16s_11140	0.27676367	-0.171023386	3.550178884	1.458488346	0.983712096	NA
ASV_16s_11141	0.277281508	-0.192543789	3.550163429	1.463407542	0.983549519	NA
ASV_16s_11142	0.274755064	-0.199653066	3.550171569	1.45566682	0.98380491	NA
ASV_16s_11143	0.284257508	-0.221766541	3.550117885	1.484511324	0.982841037	NA
ASV_16s_11144	0.275785036	-0.232367436	3.550152549	1.456210984	0.983787034	NA
ASV_16s_11145	0.275278606	-0.231766282	3.550155144	1.454706186	0.983836437	NA
ASV_16s_11146	0.27909394	-0.26751626	3.550127778	1.455687078	NA	NA
ASV_16s_11147	0.273787838	-0.283485757	3.550148758	1.431278066	0.984593928	NA
ASV_16s_11148	0.275307126	-0.285076848	3.550141258	1.435255656	0.984466863	NA
ASV_16s_11149	0.274553186	-0.295306787	3.550142923	1.427088572	0.984727083	NA
ASV_16s_11150	0.280129615	-0.311812215	3.550115072	1.433722599	NA	NA
ASV_16s_11151	0.269494595	-0.301357746	3.550166341	1.407361105	NA	NA
ASV_16s_11152	0.29421237	-0.426561807	3.550070347	1.324618772	0.987769096	NA
ASV_16s_11153	0.35019384	-0.681226977	3.550007202	0.875034195	0.996600689	NA
ASV_16s_11154	0.33273713	-0.680924593	3.550073433	0.858573932	0.996799228	NA
ASV_16s_11155	0.269631492	-0.678272742	3.550380131	0.791010961	NA	NA
ASV_16s_11156	0.076199534	-0.169156267	3.550854944	0.378482043	NA	NA
ASV_16s_11157	0.079032207	-0.072813725	3.55083253	0.560537321	NA	NA
ASV_16s_11158	0.051759846	-0.166969065	3.551012284	0.248886398	NA	NA
ASV_16s_11159	0.090580799	0.220174172	3.550895602	0.419743043	NA	NA
ASV_16s_11161	0.307484645	-0.058621043	3.549979095	1.436693745	0.984420768	NA
ASV_16s_11162	0.015821591	0.526744295	3.551888789	-0.023542502	1	NA
ASV_16s_11163	0.264638979	0.182362404	3.550139763	1.329040787	0.987646251	NA
ASV_16s_11164	0.273631771	-0.369123491	3.549997475	0.86413655	NA	NA
ASV_16s_11165	0.296159006	0.210359762	3.549780269	1.517940287	0.98168206	NA
ASV_16s_11166	0.278074865	0.043435726	3.549864703	1.40911506	0.985290442	NA
ASV_16s_11167	0.359833616	-0.781823939	3.549750122	1.055550901	0.993881965	NA
ASV_16s_11168	0.284620772	0.089732665	3.549620257	1.509297854	0.981986022	NA
ASV_16s_11169	0.30031912	0.535601886	3.549808416	1.403976498	0.985449157	NA
ASV_16s_11170	0.309776274	0.630312271	3.549374754	1.396808074	0.985668829	NA
ASV_16s_11171	0.359042397	-0.782011625	3.549611408	1.27032532	0.989216528	NA
ASV_16s_11172	0.33330041	-0.085320454	3.549102652	1.507180357	0.982060035	NA
ASV_16s_11173	0.331296911	-0.766717859	3.549752896	1.232319546	NA	NA
ASV_16s_11174	0.352874772	-0.773448969	3.549644849	1.293844011	0.988603291	NA
ASV_16s_11175	0.350415989	-0.769856638	3.549660055	1.306988955	NA	NA
ASV_16s_11176	0.331014631	-0.705540033	3.549953534	1.322496149	NA	NA
ASV_16s_11177	0.292501955	0.228447466	3.54939664	0.906082387	NA	NA
ASV_16s_11178	0.351467321	0.744820906	3.550644246	1.352051302	NA	NA
ASV_16s_11179	0.350616629	-0.753610889	3.549705356	1.361520589	0.986720808	NA
ASV_16s_11180	0.347524722	-0.732880283	3.549757002	1.381515346	0.986130715	NA
ASV_16s_11181	0.318752544	1.183128082	3.550549664	1.020560298	NA	NA

ASV_16s_11182	0.328571307	-0.708170374	3.549848795	1.345137391	NA	NA
ASV_16s_11183	0.353352404	0.192712378	3.549863501	1.547408191	0.980622855	NA
ASV_16s_11184	0.301080996	-0.241954903	3.550458378	1.129999814	0.992450365	NA
ASV_16s_11185	0.354737078	0.383257329	3.549160361	1.571101792	0.979745538	NA
ASV_16s_11186	0.356935335	-0.703653314	3.549802589	1.432779615	0.984546034	NA
ASV_16s_11187	0.310306651	-0.280608631	3.550373808	1.152907899	NA	NA
ASV_16s_11188	0.369576408	-0.716612528	3.549750753	1.457526097	0.983743785	NA
ASV_16s_11189	0.332140356	-0.772943354	3.549898483	1.317766535	0.987957971	NA
ASV_16s_11190	0.332382454	-0.930864815	3.550048278	0.836158708	NA	NA
ASV_16s_11191	0.348853832	-0.688354386	3.549855629	1.414871711	0.985111398	NA
ASV_16s_11192	0.352446573	-0.636929319	3.549960797	1.435597139	0.984455924	NA
ASV_16s_11193	0.322375245	0.183250528	3.549531713	1.380184332	NA	NA
ASV_16s_11194	0.360308764	-0.624020864	3.549981253	1.448266784	0.98404682	NA
ASV_16s_11195	0.350435324	0.794260018	3.5497086	1.127879608	0.992493789	NA
ASV_16s_11196	0.346086984	0.340861614	3.551391338	0.753927713	NA	NA
ASV_16s_11197	0.348560634	-0.536599007	3.550193418	1.403892426	0.985451745	NA
ASV_16s_11198	0.221290821	0.723503316	3.551234874	1.049302265	NA	NA
ASV_16s_11199	0.338572696	-0.147622936	3.54941459	1.336258375	NA	NA
ASV_16s_11200	0.264045909	-0.48409091	3.550479245	0.475809201	0.999530326	NA
ASV_16s_11201	0.273394673	-0.321802327	3.550270788	1.117938662	NA	NA
ASV_16s_11202	0.294115806	-0.696441325	3.550931733	0.609973717	NA	NA
ASV_16s_11203	0.311628109	0.25183438	3.549138905	1.072992931	NA	NA
ASV_16s_11204	0.305127869	-0.077898366	3.550685579	1.2252011	0.990335025	NA
ASV_16s_11205	0.323432081	-0.078007668	3.550524079	1.291816313	0.988656987	NA
ASV_16s_11206	0.330614017	0.02750941	3.549445576	0.857588744	0.996810865	NA
ASV_16s_11207	0.244061243	0.684464318	3.551212716	0.54053783	NA	NA
ASV_16s_11208	0.303925909	0.126647822	3.550020331	1.246963715	0.989805066	NA
ASV_16s_11209	0.30817803	1.311692562	3.550551664	0.788961821	NA	NA
ASV_16s_11210	0.254865915	0.895110451	3.550987099	0.751771164	NA	NA
ASV_16s_11211	0.362507149	-0.267899428	3.549743825	1.235514893	NA	NA
ASV_16s_11212	0.226417359	0.381900734	3.551350906	0.713440705	NA	NA
ASV_16s_11213	0.317608974	-1.306764026	3.549773568	0.478545492	NA	NA
ASV_16s_11214	0.226125556	-0.560465802	3.550458928	0.712948516	NA	NA
ASV_16s_11215	0.226125556	-0.560465802	3.550458928	0.712948516	NA	NA
ASV_16s_11216	0.308425149	-1.043602025	3.549882518	0.527317131	NA	NA
ASV_16s_11217	0.216847167	1.258387063	3.550719718	0.606573076	NA	NA
ASV_16s_11218	0.257802045	0.41480732	3.550826904	0.689941181	NA	NA
ASV_16s_11219	0.257802045	0.41480732	3.550826904	0.689941181	NA	NA
ASV_16s_11220	0.278932674	0.418216674	3.55071359	0.715866907	NA	NA
ASV_16s_11221	0.097121979	0.346514224	3.551701968	0.379762932	NA	NA
ASV_16s_11222	0.097121979	0.346514224	3.551701968	0.379762932	NA	NA
ASV_16s_11223	0.097121979	0.346514224	3.551701968	0.379762932	NA	NA
ASV_16s_11224	0.156716008	-0.515593918	3.550703648	0.526586945	NA	NA
ASV_16s_11225	0.156716008	-0.515593918	3.550703648	0.526586945	NA	NA
ASV_16s_11226	0.174431515	-0.530521907	3.55054382	0.56041202	NA	NA
ASV_16s_11227	0.302545241	-0.079025472	3.550695247	1.144825798	0.992142291	NA
ASV_16s_11228	0.266303913	-0.078944026	3.550719802	1.080462009	NA	NA
ASV_16s_11229	0.192909779	0.220045836	3.550726686	0.759404913	0.997836508	NA
ASV_16s_11230	0.208845002	0.596123062	3.550681512	0.730742142	NA	NA
ASV_16s_11231	0.233471571	0.610065667	3.550452443	1.040574419	0.994147193	NA
ASV_16s_11232	0.356510201	0.414947333	3.549741991	1.735956028	0.973001141	NA
ASV_16s_11233	0.278196353	-0.068989456	3.550218878	1.494371035	NA	NA
ASV_16s_11234	0.280673944	-0.068852718	3.550124802	1.517240487	NA	NA
ASV_16s_11235	0.274647263	-0.014203588	3.550558832	1.212498723	NA	NA
ASV_16s_11236	0.268403388	0.049943771	3.550067975	1.830572368	0.968619894	NA
ASV_16s_11237	0.379346103	1.939184005	3.549894751	0.461032782	NA	NA
ASV_16s_11238	0.290088792	-0.067638868	3.550244499	1.442409526	NA	NA
ASV_16s_11239	0.30141241	0.460233637	3.55125844	1.145703022	NA	NA
ASV_16s_11240	0.356578167	0.321227534	3.550894823	1.002360676	0.994790366	NA
ASV_16s_11241	0.161884837	0.279750254	3.551622127	0.613266097	NA	NA
ASV_16s_11242	0.080552815	0.286533206	3.551807929	0.3912345	NA	NA
ASV_16s_11243	0.22689596	-0.560978332	3.551024574	0.7201946	NA	NA
ASV_16s_11244	0.346563187	0.338243245	3.550932825	0.741321182	NA	NA
ASV_16s_11245	0.29294152	-0.116345183	3.550121521	1.432780415	0.984546009	NA
ASV_16s_11246	0.4121702	0.184104321	3.549961751	1.840930296	NA	NA
ASV_16s_11247	0.444330059	-0.129839728	3.548987975	1.580386483	0.979395486	NA
ASV_16s_11248	0.416854773	0.343579013	3.549865621	1.722086341	0.97361199	NA

ASV_16s_11249	0.241648959	-0.073816203	3.550762932	1.055863276	NA	NA
ASV_16s_11250	0.32739341	0.342847072	3.549952103	1.601032435	NA	NA
ASV_16s_11251	0.3476165	0.342713203	3.549895444	1.660406299	0.976231602	NA
ASV_16s_11252	0.396916635	0.789564345	3.549315808	1.277870827	NA	NA
ASV_16s_11253	0.335800546	0.360842075	3.550646396	0.90533284	0.99621461	NA
ASV_16s_11254	0.299008122	0.360370971	3.55093369	1.182188096	NA	NA
ASV_16s_11255	0.299008122	0.360370971	3.55093369	1.182188096	NA	NA
ASV_16s_11256	0.387423203	0.315939489	3.550772703	1.274531668	NA	NA
ASV_16s_11257	0.364339304	0.315939405	3.550784421	1.243360365	0.989894028	NA
ASV_16s_11258	0.345548064	0.315939412	3.550795112	1.216502263	0.99054196	NA
ASV_16s_11259	0.337911966	0.315939415	3.550799798	1.205158261	0.990807645	NA
ASV_16s_11260	0.323468613	0.315939422	3.550809254	1.183044732	0.991312063	NA
ASV_16s_11261	0.321199608	0.315939424	3.550810816	1.179482541	0.99139166	NA
ASV_16s_11262	0.385286637	0.315939398	3.550773748	1.271585507	0.989184199	NA
ASV_16s_11263	0.460170897	1.179937454	3.550036624	0.963053621	NA	NA
ASV_16s_11264	0.461396829	1.179794423	3.550077872	0.979840247	0.995147218	NA
ASV_16s_11265	0.344006163	1.032681428	3.550626206	0.943110926	NA	NA
ASV_16s_11266	0.421823922	1.160518783	3.550293682	0.970352607	NA	NA
ASV_16s_11267	0.383145707	1.10976323	3.550443737	0.954340048	0.995531853	NA
ASV_16s_11268	0.413327879	0.80276361	3.550712889	1.150825713	0.992015407	NA
ASV_16s_11269	0.413137861	0.734710066	3.550836281	1.282497417	NA	NA
ASV_16s_11270	0.413137861	0.734710066	3.550836281	1.282497417	NA	NA
ASV_16s_11271	0.413137861	0.734710066	3.550836281	1.282497417	NA	NA
ASV_16s_11272	0.204650179	0.733801149	3.551099847	0.929405371	NA	NA
ASV_16s_11273	0.204650179	0.733801149	3.551099847	0.929405371	NA	NA
ASV_16s_11274	0.204650179	0.733801149	3.551099847	0.929405371	NA	NA
ASV_16s_11275	0.204650179	0.733801149	3.551099847	0.929405371	NA	NA
ASV_16s_11276	0.204650179	0.733801149	3.551099847	0.929405371	NA	NA
ASV_16s_11277	0.353631459	0.693461562	3.550762087	1.162914274	0.991755872	NA
ASV_16s_11278	0.370340535	0.69349253	3.550752651	1.186304931	0.991238812	NA
ASV_16s_11279	0.39498254	0.693536845	3.550740182	1.218989842	NA	NA
ASV_16s_11280	0.407532121	0.693555466	3.550734416	1.234819392	0.990102967	NA
ASV_16s_11281	0.265175402	0.272744858	3.551134606	0.765881513	NA	NA
ASV_16s_11282	0.265175402	0.272744858	3.551134606	0.765881513	NA	NA
ASV_16s_11283	0.265175402	0.272744858	3.551134606	0.765881513	NA	NA
ASV_16s_11284	0.273832503	-0.068384371	3.550288903	1.372379627	NA	NA
ASV_16s_11285	0.270117677	-0.106822071	3.550265226	1.409285869	NA	NA
ASV_16s_11286	0.265352228	-0.186973631	3.5502252	1.423942241	0.984826625	NA
ASV_16s_11287	0.275273237	-0.285030588	3.550141477	1.435110792	NA	NA
ASV_16s_11288	0.27172823	-0.281291985	3.550159129	1.425776216	0.98476865	NA
ASV_16s_11289	0.305239703	-0.323668316	3.55000965	1.499216181	0.982336783	NA
ASV_16s_11290	0.271486424	-0.303359716	3.550156459	1.412354878	0.985189838	NA
ASV_16s_11291	0.285950557	-0.373079128	3.550091658	1.386127357	0.985992385	NA
ASV_16s_11292	0.281518113	-0.369585551	3.550110903	1.37691186	0.986267959	NA
ASV_16s_11293	0.270007063	-0.384556941	3.550168469	1.317571701	0.987963315	NA
ASV_16s_11294	0.094797013	-0.394225481	3.551483334	0.448045435	NA	NA
ASV_16s_11295	0.449850653	-0.682484247	3.54972707	0.955568606	NA	NA
ASV_16s_11296	0.265546167	-0.677377992	3.550403508	0.785975376	NA	NA
ASV_16s_11297	0.265546167	-0.677377992	3.550403508	0.785975376	NA	NA
ASV_16s_11298	0.117941885	0.220118233	3.550821858	0.529162802	NA	NA
ASV_16s_11299	0.117941885	0.220118233	3.550821858	0.529162802	NA	NA
ASV_16s_11300	0.117941885	0.220118233	3.550821858	0.529162802	NA	NA
ASV_16s_11304	0.301687476	-0.127313736	3.549991316	1.443155363	0.984212638	NA
ASV_16s_11305	0.274161315	-0.186938789	3.550398277	1.301052657	0.988411132	NA
ASV_16s_11306	0.285271824	0.198367376	3.550126584	1.42297293	NA	NA
ASV_16s_11307	0.304257899	-0.704362364	3.550169732	1.141160521	NA	NA
ASV_16s_11308	0.330471191	-0.776780632	3.549780038	1.178835197	0.991406076	NA
ASV_16s_11309	0.345723314	-0.776915914	3.549679987	1.244007752	0.989878081	NA
ASV_16s_11310	0.326467227	-0.766697947	3.549780555	1.208134588	NA	NA
ASV_16s_11311	0.285143426	-0.86914147	3.550076909	0.935660484	0.995800742	NA
ASV_16s_11312	0.301385485	0.176000301	3.549625936	1.637700348	0.977156212	NA
ASV_16s_11313	0.355652358	-0.76672331	3.549647591	1.337691634	0.987403749	NA
ASV_16s_11314	0.315422993	0.200101293	3.54949224	1.018333921	NA	NA
ASV_16s_11315	0.365373428	-0.74989256	3.549676345	1.415793548	NA	NA
ASV_16s_11316	0.287569812	-0.610278943	3.550221822	1.303150744	NA	NA
ASV_16s_11317	0.366024365	-0.748140918	3.549679006	1.420592245	NA	NA
ASV_16s_11318	0.34406458	-0.720852992	3.549788642	1.382531192	0.986100318	NA

ASV_16s_11319	0.353477069	-0.716936135	3.549777583	1.414060113	NA		NA
ASV_16s_11320	0.379105655	0.032844428	3.549896485	1.683313427	NA		NA
ASV_16s_11321	0.290320078	-0.601561497	3.549856897	1.066876833		0.993676394	NA
ASV_16s_11322	0.351503393	-0.704472574	3.549809815	1.417001419		0.985044827	NA
ASV_16s_11323	0.342303992	-0.670879562	3.549072793	0.812612164	NA		NA
ASV_16s_11324	0.33784778	-0.636737917	3.549824823	1.544433146		0.980731395	NA
ASV_16s_11325	0.33384649	-0.667618895	3.54992544	1.385678399		0.986005888	NA
ASV_16s_11326	0.332277264	-0.627782076	3.550007149	1.391272099		0.985837093	NA
ASV_16s_11327	0.367288464	-0.09149699	3.549369079	1.305287216		0.98829733	NA
ASV_16s_11328	0.347602153	-0.624504765	3.549994474	1.421759844		0.98489544	NA
ASV_16s_11329	0.293346989	-0.064135119	3.550336806	1.246410298	NA		NA
ASV_16s_11330	0.593424075	2.236577872	3.51784641	0.496156513		0.999460433	NA
ASV_16s_11331	0.370896562	0.407813322	3.550354269	1.326929939		0.987704985	NA
ASV_16s_11332	0.375784033	-0.175908088	3.550205671	1.384710245		0.986034978	NA
ASV_16s_11333	0.265678636	0.364215889	3.551290417	1.058175888	NA		NA
ASV_16s_11334	0.326064837	0.677982714	3.550828557	1.24072195		0.989958861	NA
ASV_16s_11335	0.339238829	-0.41448565	3.550505629	1.101576983		0.993019434	NA
ASV_16s_11336	0.327224446	-0.051182523	3.550728782	1.243732127	NA		NA
ASV_16s_11337	0.318156539	0.149505294	3.550810864	0.888311402		0.996434827	NA
ASV_16s_11338	0.330800096	1.319722649	3.550464046	0.812579404	NA		NA
ASV_16s_11339	0.369864644	1.323120794	3.550159906	1.124150577	NA		NA
ASV_16s_11340	0.287747071	0.395466985	3.550992686	0.79357938	NA		NA
ASV_16s_11341	0.29029504	0.385490775	3.551353412	0.524476294	NA		NA
ASV_16s_11342	0.425695951	0.402914051	3.551140689	0.573541335		0.999130177	NA
ASV_16s_11343	0.350762003	-0.076815527	3.550729051	1.391784915	NA		NA
ASV_16s_11344	0.373101184	-0.56501009	3.549767135	0.872938165	NA		NA
ASV_16s_11345	0.299480893	-0.436665288	3.550465505	0.892378038	NA		NA
ASV_16s_11346	0.172322197	1.197373138	3.550908297	0.527680689	NA		NA
ASV_16s_11347	0.095650434	0.346267621	3.551706379	0.375494909	NA		NA
ASV_16s_11348	0.189775916	-0.150266404	3.550219671	0.826775795	NA		NA
ASV_16s_11349	0.269238491	-0.078949888	3.550717573	1.085948168		0.993320423	NA
ASV_16s_11350	0.262269005	-0.078933493	3.550722958	1.072756649	NA		NA
ASV_16s_11351	0.19403257	0.385920966	3.550705464	1.250738731		0.989711346	NA
ASV_16s_11352	0.319958505	0.310934146	3.550792634	1.154650934		0.991933845	NA
ASV_16s_11353	0.307806307	1.077294076	3.550767413	1.056198773	NA		NA
ASV_16s_11354	0.186198463	0.512368226	3.550669237	0.878687674		0.996555561	NA
ASV_16s_11355	0.271188335	-0.069230381	3.550429719	1.412026915	NA		NA
ASV_16s_11356	0.497572481	1.527417826	3.550019861	0.854496673	NA		NA
ASV_16s_11357	0.276143518	-0.068850542	3.550148386	1.501416551	NA		NA
ASV_16s_11358	0.277134555	-0.068796163	3.55012353	1.506760318	NA		NA
ASV_16s_11359	0.333290037	1.33552979	3.549964655	0.809979309	NA		NA
ASV_16s_11360	0.279839613	-0.120317914	3.550676632	1.234481293	NA		NA
ASV_16s_11361	0.294808049	-0.067489219	3.550275985	1.419592064	NA		NA
ASV_16s_11362	0.221917119	0.093225763	3.550549114	0.967977844	NA		NA
ASV_16s_11363	0.325955006	0.666593	3.550822684	1.209782707	NA		NA
ASV_16s_11364	0.261583687	0.738109894	3.551092777	1.070833109		0.993603565	NA
ASV_16s_11365	0.447363309	1.207896625	3.550308279	0.89115435	NA		NA
ASV_16s_11366	0.374133069	1.283793438	3.549831121	1.397366724		0.985651783	NA
ASV_16s_11367	0.364109173	0.919318923	3.550551214	1.278169879		0.989014309	NA
ASV_16s_11368	0.393046252	0.919323949	3.550533621	1.324951346		0.987759883	NA
ASV_16s_11369	0.378050291	1.330739812	3.550220602	1.215605212	NA		NA
ASV_16s_11370	0.364270576	-0.087845053	3.550371566	1.208238645	NA		NA
ASV_16s_11371	0.433006258	0.744067617	3.549871206	1.758839406		0.971975781	NA
ASV_16s_11372	0.40623495	0.287449425	3.549769037	1.828670974		0.968711621	NA
ASV_16s_11373	0.341063755	0.343478804	3.549988596	1.589675658	NA		NA
ASV_16s_11374	0.294477696	0.360369383	3.550938903	1.174431715	NA		NA
ASV_16s_11375	0.347292216	0.315939431	3.550794078	1.21901405		0.990482494	NA
ASV_16s_11376	0.330914674	0.315939438	3.550804279	1.194546352		0.991051925	NA
ASV_16s_11377	0.324643548	0.315939441	3.550808458	1.184849374		0.991271564	NA
ASV_16s_11378	0.322605134	0.315939442	3.550809851	1.181662708		0.991342999	NA
ASV_16s_11379	0.316809947	0.315939446	3.550813908	1.172486466		0.991546654	NA
ASV_16s_11380	0.315783361	0.315939446	3.550814642	1.170842258		0.991582825	NA
ASV_16s_11381	0.361256302	0.315939424	3.550786114	1.238949892		0.99000226	NA
ASV_16s_11382	0.307570666	0.31593955	3.550820694	1.157495867	NA		NA
ASV_16s_11383	0.460431357	1.179821339	3.550065028	0.975130258		0.995219805	NA
ASV_16s_11384	0.485756969	0.73484378	3.550797425	1.364578889		0.986631561	NA
ASV_16s_11385	0.387533621	1.184625802	3.550264705	0.929131624	NA		NA

ASV_16s_11386	0.201549419	0.733773709	3.551107855	0.921913696	NA	NA
ASV_16s_11387	0.201549419	0.733773709	3.551107855	0.921913696	NA	NA
ASV_16s_11388	0.385200105	0.693518981	3.550744943	1.206248293	0.99078232	NA
ASV_16s_11389	0.352190111	0.69345733	3.550762949	1.160808682	0.991801453	NA
ASV_16s_11390	0.388997956	0.693527063	3.550743068	1.211223488	NA	NA
ASV_16s_11391	0.354097253	0.693460827	3.55076182	1.163531158	0.991742487	NA
ASV_16s_11392	0.261095781	0.273413063	3.551162049	0.760766015	NA	NA
ASV_16s_11393	0.26578484	-0.110477135	3.550284242	1.397486951	NA	NA
ASV_16s_11394	0.278613629	-0.142215074	3.550191315	1.447324325	NA	NA
ASV_16s_11395	0.267827671	-0.147125733	3.550240484	1.422196824	NA	NA
ASV_16s_11396	0.268604427	-0.180720201	3.550212589	1.433717083	0.984516087	NA
ASV_16s_11397	0.266342606	-0.209937716	3.550207884	1.428033988	0.984697095	NA
ASV_16s_11398	0.269899021	-0.23592657	3.55017965	1.436333046	0.984432337	NA
ASV_16s_11399	0.263067926	-0.238521939	3.55021308	1.413335165	0.985159316	NA
ASV_16s_11400	0.265853289	-0.241887426	3.550197797	1.421700143	0.984897319	NA
ASV_16s_11401	0.264846134	-0.251634367	3.550200054	1.415609912	0.985088343	NA
ASV_16s_11402	0.265105053	-0.26293057	3.550196031	1.412491786	0.985185577	NA
ASV_16s_11403	0.293206557	-0.33440345	3.550057466	1.455012105	0.983826401	NA
ASV_16s_11404	0.267406802	-0.310645307	3.550175727	1.394488973	0.985739464	NA
ASV_16s_11405	0.26817215	-0.322950596	3.550171182	1.388072344	0.985933799	NA
ASV_16s_11406	0.27625754	-0.34195158	3.55013241	1.396347669	0.985682869	NA
ASV_16s_11407	0.261352464	-0.352423773	3.550207016	1.332275774	NA	NA
ASV_16s_11408	0.263884611	-0.354698956	3.550194122	1.33813496	0.987391244	NA
ASV_16s_11409	0.292750606	-0.401465694	3.550068576	1.363008596	0.986677431	NA
ASV_16s_11410	0.267204924	-0.421176372	3.550194416	1.251897481	0.989682472	NA
ASV_16s_11411	0.093338598	-0.388940256	3.551496332	0.443536992	NA	NA
ASV_16s_11412	0.33813477	-0.681031725	3.550052284	0.863738448	NA	NA
ASV_16s_11413	0.345034895	-0.68114797	3.550026172	0.870227159	0.996659477	NA
ASV_16s_11414	0.34555728	-0.68115658	3.550024238	0.870713034	0.996653565	NA
ASV_16s_11415	0.346717439	-0.681175611	3.550019961	0.871789727	0.996640441	NA
ASV_16s_11416	0.32654363	-0.680825624	3.550098599	0.852540168	NA	NA
ASV_16s_11417	0.261460841	-0.676285145	3.550426819	0.780846973	NA	NA
ASV_16s_11418	0.261460841	-0.676285145	3.550426819	0.780846973	NA	NA
ASV_16s_11419	0.083303096	-0.169274813	3.550825378	0.412044671	NA	NA
ASV_16s_11420	0.073890457	-0.169113226	3.550865789	0.367141183	NA	NA
ASV_16s_11421	0.088655612	-0.169350974	3.550806203	0.436354706	NA	NA
ASV_16s_11422	0.087835926	0.22018172	3.550905534	0.407693209	NA	NA
ASV_16s_11425	0.278178093	0.05222083	3.55013578	1.414960544	0.985108625	NA
ASV_16s_11426	0.349923123	-1.002362184	3.550082343	0.732810096	0.998070742	NA
ASV_16s_11427	0.283931076	0.208044454	3.550030608	1.450327565	0.983979672	NA
ASV_16s_11428	0.287808236	0.165123838	3.549946093	1.551547179	0.980471251	NA
ASV_16s_11429	0.301712669	0.381653976	3.549890301	1.381066786	NA	NA
ASV_16s_11430	0.265961155	-0.353641197	3.550002291	0.8753377	NA	NA
ASV_16s_11431	0.270868042	-0.114129003	3.549962734	0.757441206	NA	NA
ASV_16s_11432	0.287016567	-0.307750014	3.550650198	1.06236901	0.993758731	NA
ASV_16s_11433	0.273103696	0.330139969	3.549987485	1.445903597	NA	NA
ASV_16s_11434	0.275703217	0.444635012	3.549962884	1.130922964	0.992431409	NA
ASV_16s_11435	0.328098483	-0.779710866	3.549819626	1.126264164	NA	NA
ASV_16s_11436	0.352127516	-0.788210518	3.549684552	1.174168993	NA	NA
ASV_16s_11437	0.284928121	-0.354176696	3.550289335	1.186413833	0.991236359	NA
ASV_16s_11438	0.420628869	-0.741579687	3.549529733	1.204236339	0.99082903	NA
ASV_16s_11439	0.342252912	-0.748723234	3.549738568	1.337129575	0.987419592	NA
ASV_16s_11440	0.352029826	-0.759114823	3.549685323	1.350493192	NA	NA
ASV_16s_11441	0.28745974	0.703611377	3.549865045	1.330268927	NA	NA
ASV_16s_11442	0.32173786	0.0222918	3.549360799	1.615809075	0.978027479	NA
ASV_16s_11443	0.319166991	-1.00867874	3.549711851	1.058939552	NA	NA
ASV_16s_11444	0.355308362	-0.706293337	3.550494071	0.932058475	0.995851357	NA
ASV_16s_11445	0.350803319	-0.747806736	3.549716027	1.37230846	NA	NA
ASV_16s_11446	0.29490677	1.395840127	3.551016681	0.573810299	NA	NA
ASV_16s_11447	0.333499498	-0.118297805	3.549504318	1.432635007	NA	NA
ASV_16s_11448	0.338126596	-0.569900583	3.550371169	1.101752312	NA	NA
ASV_16s_11449	0.339775255	-0.69531355	3.549853465	1.388970193	0.985906704	NA
ASV_16s_11450	0.297533311	-0.663806643	3.550632807	1.013331072	NA	NA
ASV_16s_11451	0.342048061	-0.637324905	3.549973197	1.409731552	0.98527133	NA
ASV_16s_11452	0.334529578	-0.71746348	3.54992321	1.089787581	0.993247259	NA
ASV_16s_11453	0.251932525	0.261221458	3.550801321	1.158694865	NA	NA
ASV_16s_11454	0.27851349	-0.827119917	3.550219718	0.667524737	NA	NA

ASV_16s_11455	0.314295416	-0.430784123	3.550403054	1.289429908		0.988719982	NA
ASV_16s_11456	0.298119018	-0.258410821	3.550627453	1.228222951	NA		NA
ASV_16s_11457	0.392695769	0.114879729	3.550139262	1.672267312	NA		NA
ASV_16s_11458	0.373349996	0.831048945	3.550465865	0.799221043		0.997451895	NA
ASV_16s_11459	0.315598988	0.101554026	3.550845859	0.902337171		0.99625399	NA
ASV_16s_11460	0.295583517	0.98913531	3.550615696	0.788454212		0.997560004	NA
ASV_16s_11461	0.267794078	0.195202213	3.55091229	0.448099437		0.999615207	NA
ASV_16s_11462	0.289462759	1.302235665	3.550624074	0.767763389	NA		NA
ASV_16s_11463	0.314306821	1.093812168	3.550323389	1.200109251	NA		NA
ASV_16s_11464	0.294297539	1.304908694	3.550604929	0.773394315		0.997706087	NA
ASV_16s_11465	0.352771493	1.890590475	3.550006145	0.51333254		0.999396184	NA
ASV_16s_11466	0.325405627	1.898035627	3.550037985	0.475616111	NA		NA
ASV_16s_11467	0.283320193	0.394694754	3.551017208	0.788427623	NA		NA
ASV_16s_11468	0.298697485	-0.227881319	3.550721676	0.804967279	NA		NA
ASV_16s_11469	0.221595374	-0.51454359	3.55100594	0.772375146	NA		NA
ASV_16s_11470	0.299147567	-0.852416174	3.549901671	0.983741719		0.995086558	NA
ASV_16s_11471	0.320159304	-0.552532639	3.550103779	0.884926029	NA		NA
ASV_16s_11472	0.219556227	0.379955895	3.551391049	0.702989785	NA		NA
ASV_16s_11473	0.219556227	0.379955895	3.551391049	0.702989785	NA		NA
ASV_16s_11474	0.257864188	-0.480147464	3.55081723	0.809645384	NA		NA
ASV_16s_11475	0.486936488	-0.133346754	3.547949817	1.721784058		0.973625214	NA
ASV_16s_11476	0.222465403	0.228893565	3.550892192	0.474751824	NA		NA
ASV_16s_11477	0.28934909	-0.52891377	3.55054688	0.920566031	NA		NA
ASV_16s_11478	0.221303864	-0.646063356	3.550445993	0.775955819		0.997681659	NA
ASV_16s_11479	0.371828069	-1.481618409	3.549295426	0.47099722	NA		NA
ASV_16s_11480	0.169671086	1.192961551	3.550920949	0.522445537	NA		NA
ASV_16s_11481	0.151967038	-0.509793525	3.550746814	0.516834577	NA		NA
ASV_16s_11482	0.18931074	0.220047878	3.550729541	0.750115558		0.997920346	NA
ASV_16s_11483	0.192246357	0.220046275	3.550727209	0.757669502	NA		NA
ASV_16s_11484	0.202516366	0.596111547	3.550685622	0.715633142	NA		NA
ASV_16s_11485	0.234700698	0.449209587	3.550888371	0.964154918	NA		NA
ASV_16s_11486	0.277327866	-0.320532461	3.550038102	0.86357709	NA		NA
ASV_16s_11487	0.301748061	0.554018615	3.549757929	1.696009712		0.974738777	NA
ASV_16s_11488	0.269807207	-0.068347358	3.550504026	1.363974654		0.986649223	NA
ASV_16s_11489	0.356125168	0.79381703	3.550218959	1.428294165	NA		NA
ASV_16s_11490	0.347410829	0.166012505	3.549565428	1.509487384		0.981979388	NA
ASV_16s_11491	0.393652552	1.06022702	3.549514659	0.870983879	NA		NA
ASV_16s_11492	0.273099647	-0.068763074	3.550134819	1.493349342	NA		NA
ASV_16s_11493	0.299138177	0.100964226	3.549661717	1.445561089	NA		NA
ASV_16s_11494	0.285539798	0.791172246	3.549844136	0.958589139	NA		NA
ASV_16s_11495	0.361626872	0.258715129	3.550147313	0.821168772	NA		NA
ASV_16s_11496	0.359126569	1.806432091	3.550085198	0.560819788		0.999191911	NA
ASV_16s_11497	0.279541385	-0.068005048	3.550185201	1.459290401	NA		NA
ASV_16s_11498	0.290365717	-0.068059442	3.55015259	1.470825836	NA		NA
ASV_16s_11499	0.229232782	1.227925584	3.551121163	0.680110756	NA		NA
ASV_16s_11500	0.364502736	0.265430407	3.55045196	1.875028337	NA		NA
ASV_16s_11501	0.129281367	0.281999266	3.551676962	0.535247499	NA		NA
ASV_16s_11502	0.220020325	-0.553643725	3.551048145	0.709632498	NA		NA
ASV_16s_11503	0.364556413	-0.193775723	3.549915936	1.343806307	NA		NA
ASV_16s_11504	0.083597934	-0.298824587	3.551621687	0.402050663	NA		NA
ASV_16s_11505	0.448240405	0.322488967	3.548742339	1.351450691		0.987012098	NA
ASV_16s_11506	0.368374027	0.590842099	3.550747821	1.177683286		0.99143169	NA
ASV_16s_11507	0.396419609	0.502700064	3.550673616	1.36564706		0.986600304	NA
ASV_16s_11508	0.539906512	-0.029538148	3.550669984	1.497741063		0.982387761	NA
ASV_16s_11509	0.478368408	-0.029478845	3.550689347	1.436304023	NA		NA
ASV_16s_11510	0.498514591	0.783495683	3.550316651	2.054198795		0.956779722	NA
ASV_16s_11511	0.285926326	-0.029133283	3.550803471	1.175431667	NA		NA
ASV_16s_11512	0.35492116	0.778302595	3.549440474	1.316853182	NA		NA
ASV_16s_11513	0.353848831	0.353221449	3.550163638	1.521327011	NA		NA
ASV_16s_11514	0.313749737	0.324661531	3.550843147	1.01937109	NA		NA
ASV_16s_11515	0.360955793	0.337366475	3.55083111	0.968186577		0.995325536	NA
ASV_16s_11516	0.28994727	0.360367744	3.550944279	1.166555783	NA		NA
ASV_16s_11517	0.28994727	0.360367744	3.550944279	1.166555783	NA		NA
ASV_16s_11518	0.342675534	0.455962534	3.550775381	1.983560432		0.960744649	NA
ASV_16s_11519	0.323326268	0.315939459	3.550809361	1.18276224		0.991318392	NA
ASV_16s_11520	0.325498427	0.315939458	3.550807884	1.186157736		0.991242128	NA
ASV_16s_11521	0.318237612	0.315939462	3.550812899	1.174739689		0.991496928	NA

ASV_16s_11522	0.356471678	0.315939291	3.55078878	1.232151311	0.990167682	NA
ASV_16s_11523	0.288404613	0.688163682	3.550784741	1.035083467	NA	NA
ASV_16s_11524	0.434482318	1.179567458	3.550134528	0.958139948	NA	NA
ASV_16s_11525	0.37210444	1.136480948	3.550415757	0.929060329	0.995893185	NA
ASV_16s_11526	0.400618532	0.734682117	3.550844411	1.26683693	NA	NA
ASV_16s_11527	0.198448659	0.733745417	3.551116111	0.914331023	NA	NA
ASV_16s_11528	0.376903786	0.693504762	3.550749176	1.1951843	0.991037356	NA
ASV_16s_11529	0.378224778	0.693507123	3.55074849	1.196958047	0.990996769	NA
ASV_16s_11530	0.362502956	0.693477907	3.550756975	1.175424702	0.991481774	NA
ASV_16s_11531	0.354990308	0.693463036	3.550761296	1.164794741	0.99171503	NA
ASV_16s_11532	0.302838809	0.693341642	3.55079715	1.08440112	NA	NA
ASV_16s_11533	0.262194536	-0.08075881	3.550336369	1.353313076	NA	NA
ASV_16s_11534	0.25713024	-0.070683644	3.550377517	1.334212826	NA	NA
ASV_16s_11535	0.27209621	-0.110153071	3.550252009	1.402590852	NA	NA
ASV_16s_11536	0.262453455	-0.092974462	3.550320871	1.367450988	NA	NA
ASV_16s_11537	0.262971293	-0.116889176	3.55029299	1.392046824	NA	NA
ASV_16s_11538	0.257164465	-0.141212389	3.550302382	1.384527038	NA	NA
ASV_16s_11539	0.260461961	-0.157753352	3.550270885	1.401413912	0.985527918	NA
ASV_16s_11540	0.264006968	-0.163165084	3.550248053	1.414742387	0.985115434	NA
ASV_16s_11541	0.262251577	-0.193531261	3.550237555	1.414078333	0.985136151	NA
ASV_16s_11542	0.257192985	-0.197563999	3.55026228	1.397386505	0.985651179	NA
ASV_16s_11543	0.259230112	-0.222617595	3.550239184	1.403284868	0.98547044	NA
ASV_16s_11544	0.272120878	-0.238540063	3.550168089	1.44238172	0.984237645	NA
ASV_16s_11545	0.268732555	-0.245277073	3.55018249	1.429930876	0.984636822	NA
ASV_16s_11546	0.263287252	-0.238775821	3.550211926	1.413925532	0.985140916	NA
ASV_16s_11547	0.263045445	-0.260572966	3.550207104	1.406556003	0.985369614	NA
ASV_16s_11548	0.272971705	-0.271585278	3.550155724	1.433460434	0.984524289	NA
ASV_16s_11549	0.25722721	-0.265103578	3.550236693	1.38554439	NA	NA
ASV_16s_11550	0.263056853	-0.283140612	3.550201928	1.396673769	0.985672926	NA
ASV_16s_11551	0.276206538	-0.30799334	3.550133683	1.42396005	0.984826062	NA
ASV_16s_11552	0.261318574	-0.352383321	3.550207247	1.332150365	0.987559414	NA
ASV_16s_11553	0.091880182	-0.383580016	3.551509635	0.438984053	NA	NA
ASV_16s_11554	0.329787006	-0.680884043	3.550085377	0.855690388	NA	NA
ASV_16s_11555	0.263199455	-0.676851105	3.550417344	0.783018647	NA	NA
ASV_16s_11556	0.283898291	0.688152331	3.550788389	1.027121701	NA	NA
ASV_16s_11557	0.27840916	-0.007345972	3.550107535	1.32834036	0.987665759	NA
ASV_16s_11558	0.268524006	0.171626422	3.550111746	1.404028057	0.985447569	NA
ASV_16s_11559	0.269131487	0.898518213	3.551118398	0.818570758	NA	NA
ASV_16s_11560	0.286742321	0.143941244	3.549701251	1.512063778	0.981889071	NA
ASV_16s_11561	0.334254061	-0.78350638	3.54982588	1.080993175	0.9934141	NA
ASV_16s_11562	0.263375934	-0.316665155	3.549933677	0.934632357	NA	NA
ASV_16s_11563	0.311917407	0.316207355	3.551655492	0.673296555	NA	NA
ASV_16s_11564	0.272743744	0.283472579	3.549992655	1.293845356	NA	NA
ASV_16s_11565	0.285450876	0.696694495	3.549881233	1.335759527	0.98745816	NA
ASV_16s_11566	0.371686537	-0.78748694	3.549549765	1.285812281	0.988815067	NA
ASV_16s_11567	0.332756457	-0.772400299	3.54975027	1.217958911	0.990507502	NA
ASV_16s_11568	0.286799595	0.986302374	3.549892305	1.143701636	0.992165922	NA
ASV_16s_11569	0.364651367	-0.734528208	3.549908904	1.080697425	NA	NA
ASV_16s_11570	0.282563442	0.055524256	3.549622454	1.012049622	0.99463181	NA
ASV_16s_11571	0.359232433	-0.756641107	3.549673737	1.385192585	NA	NA
ASV_16s_11572	0.35529706	-0.73758047	3.549901248	1.17415028	0.991509953	NA
ASV_16s_11573	0.310756797	-0.717940192	3.549885215	1.273679782	NA	NA
ASV_16s_11574	0.323540291	-0.021071995	3.549885212	1.539243062	NA	NA
ASV_16s_11575	0.307471473	0.689792158	3.550745677	1.264324723	NA	NA
ASV_16s_11576	0.307155153	-0.683029282	3.549954768	1.296262535	0.988539041	NA
ASV_16s_11577	0.231783115	0.254403989	3.550037866	0.776613614	NA	NA
ASV_16s_11578	0.314037879	-0.147280644	3.550486121	1.335074612	NA	NA
ASV_16s_11579	0.323336648	-0.52214613	3.550249244	1.349968991	0.987054627	NA
ASV_16s_11580	0.355629755	0.500789408	3.549506224	1.006065257	NA	NA
ASV_16s_11581	0.466630245	-0.023235921	3.549394776	1.560451424	0.980142733	NA
ASV_16s_11582	0.322936096	-0.393025901	3.550452021	1.299041544	0.988464941	NA
ASV_16s_11583	0.296416608	-0.515861413	3.55051674	1.179180609	NA	NA
ASV_16s_11584	0.309396745	-0.080338724	3.550534501	1.138271568	NA	NA
ASV_16s_11585	0.36997772	-0.241496458	3.550581849	1.051601767	0.99395263	NA
ASV_16s_11586	0.329079284	-0.097864078	3.549576309	0.86573954	0.996713756	NA
ASV_16s_11587	0.266014712	0.212512521	3.549851633	0.999977087	NA	NA
ASV_16s_11588	0.329747392	-0.110811403	3.550678437	1.227682454	0.990275485	NA

ASV_16s_11589	0.306978401	-0.063131457	3.550829643	0.910408569	0.996147276	NA
ASV_16s_11590	0.293169079	-0.07182473	3.550281787	1.338260977	0.987387688	NA
ASV_16s_11591	0.357304025	-0.134209323	3.550628782	1.307802369	0.988229413	NA
ASV_16s_11592	0.319678809	0.154244102	3.550753093	1.152990785	0.991969306	NA
ASV_16s_11593	0.334496135	-0.41367537	3.550264968	-0.098189605	1	NA
ASV_16s_11594	0.320621632	1.316404571	3.550503156	0.802132066	NA	NA
ASV_16s_11595	0.333700916	1.320634494	3.55045346	0.815438406	NA	NA
ASV_16s_11596	0.294169938	1.304838585	3.550605488	0.773228055	NA	NA
ASV_16s_11597	0.288957602	0.353751644	3.551812769	0.44564403	NA	NA
ASV_16s_11598	0.295392882	0.353712733	3.551837422	0.451612161	NA	NA
ASV_16s_11599	0.216125661	0.378961135	3.551411952	0.697653376	NA	NA
ASV_16s_11600	0.246083771	0.411523385	3.550885427	0.67434807	NA	NA
ASV_16s_11601	0.246083771	0.411523385	3.550885427	0.67434807	NA	NA
ASV_16s_11602	0.195006013	0.224900271	3.550781693	0.527552329	0.999339211	NA
ASV_16s_11603	0.240860367	0.113581413	3.549903309	1.14827212	0.992069565	NA
ASV_16s_11604	0.276598239	-0.078964497	3.550712187	1.099476	0.993060386	NA
ASV_16s_11605	0.260409692	-0.078927259	3.550724452	1.069124184	0.993635088	NA
ASV_16s_11606	0.265585111	-0.07894112	3.550720369	1.079022136	0.99345113	NA
ASV_16s_11607	0.174726381	0.220057741	3.550742299	0.710990753	0.998249856	NA
ASV_16s_11608	0.178614392	0.220055004	3.550738696	0.72167274	0.998163615	NA
ASV_16s_11609	0.181184557	0.220053201	3.550736399	0.728629732	0.998105958	NA
ASV_16s_11610	0.281629284	0.310935973	3.550820425	1.090001855	NA	NA
ASV_16s_11611	0.461153191	0.394678108	3.549631844	1.971762811	0.96138667	NA
ASV_16s_11612	0.296061263	-1.240163933	3.54990217	0.522308119	0.999360629	NA
ASV_16s_11613	0.304766812	-0.203451339	3.550060924	0.951001759	0.995580699	NA
ASV_16s_11614	0.275429406	-0.068983928	3.550250198	1.478456193	0.983046146	NA
ASV_16s_11615	0.270551294	-0.068612595	3.550129348	1.480975067	NA	NA
ASV_16s_11616	0.293356045	-0.185022892	3.550205053	1.541056726	0.980854141	NA
ASV_16s_11617	0.351609404	0.312396997	3.550581998	0.906598216	0.996197896	NA
ASV_16s_11618	0.310316947	1.566139036	3.550455049	0.52569598	NA	NA
ASV_16s_11619	0.324380837	0.319362113	3.550898826	0.919190208	0.996028954	NA
ASV_16s_11620	0.154526435	0.28022016	3.551633082	0.59669654	NA	NA
ASV_16s_11621	0.214304718	0.279601464	3.551510424	0.714074417	NA	NA
ASV_16s_11622	0.328624813	0.716225704	3.550778423	1.337433379	NA	NA
ASV_16s_11623	0.076891323	0.286956961	3.551821729	0.378619787	NA	NA
ASV_16s_11624	0.127261345	0.282155268	3.551680997	0.529988112	NA	NA
ASV_16s_11625	0.426479504	0.074717427	3.550423919	1.521389589	NA	NA
ASV_16s_11626	0.364198332	1.139507725	3.550105863	1.354736453	0.986917485	NA
ASV_16s_11627	0.316265465	1.057258024	3.550331845	1.226249702	NA	NA
ASV_16s_11628	0.281458727	-0.02911981	3.550807971	1.167458153	NA	NA
ASV_16s_11629	0.281458727	-0.02911981	3.550807971	1.167458153	NA	NA
ASV_16s_11630	0.314676302	0.340187094	3.550001875	1.581842975	0.979340253	NA
ASV_16s_11631	0.328744189	-0.07404966	3.550697032	1.210958295	0.990672393	NA
ASV_16s_11632	0.398994516	0.613761071	3.550279431	1.868876608	0.966739839	NA
ASV_16s_11633	0.321399968	0.348964568	3.550782079	0.903978188	0.996232451	NA
ASV_16s_11634	0.285416844	0.360366052	3.550949825	1.158558202	NA	NA
ASV_16s_11635	0.285416844	0.360366052	3.550949825	1.158558202	NA	NA
ASV_16s_11636	0.285416844	0.360366052	3.550949825	1.158558202	NA	NA
ASV_16s_11637	0.523076054	0.36041559	3.550788357	1.465926424	NA	NA
ASV_16s_11638	0.433618302	0.31593943	3.550753009	1.331562149	0.987575869	NA
ASV_16s_11639	0.324753933	0.315939475	3.550808392	1.184971612	0.991268816	NA
ASV_16s_11640	0.316786588	0.31593948	3.550813935	1.172389054	0.9915488	NA
ASV_16s_11641	0.314279613	0.315939481	3.550815737	1.168364227	0.991637156	NA
ASV_16s_11642	0.308372822	0.315939486	3.550820097	1.158762284	0.9918456	NA
ASV_16s_11643	0.306319648	0.315939487	3.550821653	1.155378859	0.991918265	NA
ASV_16s_11644	0.357877065	0.3159393	3.550788001	1.234078201	0.990120971	NA
ASV_16s_11645	0.35373655	0.616761224	3.549646321	1.4375864	0.984392114	NA
ASV_16s_11646	0.283898291	0.688152331	3.550788389	1.027121701	NA	NA
ASV_16s_11647	0.283898291	0.688152331	3.550788389	1.027121701	NA	NA
ASV_16s_11648	0.395006875	1.171787941	3.550301332	0.936737199	0.995785535	NA
ASV_16s_11649	0.394358867	0.734667473	3.550848669	1.258822735	NA	NA
ASV_16s_11650	0.394358867	0.734667473	3.550848669	1.258822735	NA	NA
ASV_16s_11651	0.397177592	1.047670054	3.550551009	1.003739314	0.99476799	NA
ASV_16s_11652	0.371084326	0.693494456	3.550752258	1.18727022	0.99121705	NA
ASV_16s_11653	0.383396721	0.693516492	3.550745856	1.203811319	0.990838878	NA
ASV_16s_11654	0.252936538	0.274918167	3.551217972	0.750277297	NA	NA
ASV_16s_11655	0.256359523	-0.11676878	3.550329759	1.36957745	NA	NA

ASV_16s_11656	0.258891671	-0.121218525	3.550311168	1.380905695	NA	NA
ASV_16s_11657	0.260445184	-0.190993166	3.550248617	1.407756311	0.985332511	NA
ASV_16s_11658	0.265919008	-0.252850367	3.550194457	1.418468131	0.984998876	NA
ASV_16s_11659	0.261998697	-0.259358112	3.550212844	1.403481661	0.985464386	NA
ASV_16s_11660	0.285765919	-0.306237273	3.550090836	1.45453517	0.983842045	NA
ASV_16s_11661	0.259230447	-0.290503729	3.550220455	1.380199568	0.986170027	NA
ASV_16s_11662	0.260001499	-0.3148585	3.550213406	1.367632809	0.986542078	NA
ASV_16s_11663	0.25772827	-0.324559674	3.550225075	1.352050131	0.986994869	NA
ASV_16s_11664	0.257987189	-0.336984365	3.550223824	1.338988786	0.987367138	NA
ASV_16s_11665	0.263811129	-0.342469752	3.550193543	1.352963695	NA	NA
ASV_16s_11666	0.253193221	-0.370411433	3.55025414	1.28040877	NA	NA
ASV_16s_11667	0.253221741	-0.439207538	3.550278813	1.178612341	NA	NA
ASV_16s_11668	0.283299697	-0.679883152	3.55030459	0.806937597	0.997372507	NA
ASV_16s_11669	0.25329019	-0.672960009	3.550470973	0.770251318	NA	NA
ASV_16s_11670	0.080699874	-0.169232812	3.550835629	0.39990141	NA	NA
ASV_16s_11671	0.048622886	-0.164038258	3.55103376	0.230518159	NA	NA
ASV_16s_11672	0.085091053	0.220189748	3.550916103	0.395428746	NA	NA
ASV_16s_11673	0.26428401	-0.015744194	3.550180925	1.348119129	0.987107603	NA
ASV_16s_11674	0.274798903	0.105423456	3.549982133	1.41519037	0.985101449	NA
ASV_16s_11675	0.2887599	-0.423068968	3.550329609	1.249142788	0.989751033	NA
ASV_16s_11676	0.337114341	-0.766221509	3.549941094	0.965926611	0.99535962	NA
ASV_16s_11677	0.259611939	0.268114252	3.550171724	1.277339236	NA	NA
ASV_16s_11678	0.262776777	-0.084850068	3.551054056	0.974643893	NA	NA
ASV_16s_11679	0.348202628	-0.79024477	3.549925562	1.203245994	0.990851967	NA
ASV_16s_11680	0.316488416	-0.759539087	3.549836843	1.188608979	NA	NA
ASV_16s_11681	0.357883043	-0.779885613	3.549618685	1.282291554	0.988907128	NA
ASV_16s_11682	0.33496285	-0.757290024	3.549750366	1.284330549	0.988853869	NA
ASV_16s_11683	0.305266901	0.093522844	3.549700433	1.589168868	0.97906112	NA
ASV_16s_11684	0.322565567	-0.732453164	3.549827933	1.290236187	NA	NA
ASV_16s_11685	0.321240993	-0.72858507	3.549837788	1.29559884	0.988556695	NA
ASV_16s_11686	0.321004015	-0.744025939	3.549819157	1.272369253	NA	NA
ASV_16s_11687	0.341549797	0.986935409	3.549725332	1.351294486	NA	NA
ASV_16s_11688	0.270582959	-0.219314429	3.549958284	1.144695839	NA	NA
ASV_16s_11689	0.33395763	-0.711032812	3.54983067	1.359351182	0.986783895	NA
ASV_16s_11690	0.334951851	-0.702237094	3.549846985	1.369594198	0.986484415	NA
ASV_16s_11691	0.333051973	-0.694310856	3.549869151	1.369514031	0.986486775	NA
ASV_16s_11692	0.27664093	-0.202728063	3.549988872	1.171796652	NA	NA
ASV_16s_11693	0.334510493	-0.684989584	3.549889473	1.379665936	0.986185951	NA
ASV_16s_11694	0.329826421	-0.635637995	3.550335134	0.96882297	NA	NA
ASV_16s_11695	0.331818538	-0.676371319	3.549913723	1.376216629	0.986288614	NA
ASV_16s_11696	0.332947336	-0.629068239	3.550003549	1.384269669	0.986048204	NA
ASV_16s_11697	0.286473106	-0.72512359	3.550116059	0.908071123	NA	NA
ASV_16s_11698	0.323113616	-0.588071296	3.550104831	1.357662117	0.986832887	NA
ASV_16s_11699	0.260962634	0.327797546	3.550810367	1.637675358	0.977157218	NA
ASV_16s_11700	0.316591276	-0.562081505	3.550172732	1.338880898	0.987370185	NA
ASV_16s_11701	0.305808893	-0.988277211	3.550051043	0.835115761	0.997068852	NA
ASV_16s_11702	0.282287105	0.678559474	3.550875349	1.168573109	NA	NA
ASV_16s_11703	0.362054258	-0.495176132	3.549676548	1.050249758	NA	NA
ASV_16s_11704	0.283206918	-0.753472438	3.550201549	0.908518653	NA	NA
ASV_16s_11705	0.33438984	0.678180631	3.550824131	1.244598961	0.989863503	NA
ASV_16s_11706	0.321157957	0.110363542	3.55060735	1.210198384	0.990690183	NA
ASV_16s_11707	0.243779323	0.333760238	3.551879489	0.50458514	NA	NA
ASV_16s_11708	0.333092742	-0.051185263	3.550717693	1.246804266	0.989809013	NA
ASV_16s_11709	0.352590371	1.326452521	3.550387857	0.833760884	NA	NA
ASV_16s_11710	0.212695095	0.377950911	3.551433445	0.692240308	NA	NA
ASV_16s_11711	0.172610797	-0.312587313	3.551522392	0.59738898	NA	NA
ASV_16s_11712	0.281333958	-0.127214912	3.550711222	0.396940898	0.999743243	NA
ASV_16s_11713	0.280513354	0.008814662	3.549751715	0.724311828	0.998141882	NA
ASV_16s_11714	0.305962692	0.418804757	3.550560917	0.745560849	NA	NA
ASV_16s_11715	0.239713863	-0.113022801	3.549982838	1.222654773	NA	NA
ASV_16s_11716	0.091235799	0.345506706	3.551720209	0.36248365	NA	NA
ASV_16s_11717	0.147218068	-0.503813626	3.550792375	0.506876757	NA	NA
ASV_16s_11718	0.240555145	-0.54705965	3.550063885	0.662062775	0.998610551	NA
ASV_16s_11719	0.178792968	-0.532468981	3.550503529	0.568101557	NA	NA
ASV_16s_11720	0.163859908	-0.520316687	3.550641445	0.54038488	NA	NA
ASV_16s_11721	0.163859908	-0.520316687	3.550641445	0.54038488	NA	NA
ASV_16s_11722	0.250164282	-0.078898433	3.550733035	1.048927898	NA	NA

ASV_16s_11723	0.25792741	-0.07892069	3.550726472	1.064281025	0.993723891	NA
ASV_16s_11724	0.172788897	0.220059166	3.550744158	0.70556517	0.99829261	NA
ASV_16s_11725	0.186238658	0.220049991	3.550732069	0.742054084	NA	NA
ASV_16s_11726	0.186238658	0.220049991	3.550732069	0.742054084	NA	NA
ASV_16s_11727	0.186238658	0.220049991	3.550732069	0.742054084	NA	NA
ASV_16s_11728	0.196589672	0.224085006	3.550795568	0.600543537	NA	NA
ASV_16s_11729	0.255887548	-0.89586339	3.550499626	0.569896601	0.999148178	NA
ASV_16s_11730	0.381167697	1.092476757	3.550307468	1.234234584	NA	NA
ASV_16s_11731	0.305421669	-0.287535996	3.550864148	0.562875647	NA	NA
ASV_16s_11732	0.260768378	-0.068809562	3.550585571	1.289032067	0.988730463	NA
ASV_16s_11733	0.268136069	4.30E-04	3.550371186	1.489928658	NA	NA
ASV_16s_11734	0.288339339	-0.069032147	3.550176867	1.527267994	NA	NA
ASV_16s_11735	0.270218675	-0.068907333	3.550212624	1.47495301	0.983164138	NA
ASV_16s_11736	0.26255224	-0.068845577	3.550230344	1.451433347	NA	NA
ASV_16s_11737	0.189859151	-0.251664202	3.550691595	0.743744401	NA	NA
ASV_16s_11738	0.290563155	-0.045596555	3.550890236	1.206101687	NA	NA
ASV_16s_11739	0.313791141	0.71489148	3.550792249	1.317437692	NA	NA
ASV_16s_11740	0.075670826	0.287098327	3.551826397	0.374341252	NA	NA
ASV_16s_11741	0.353248201	0.29109028	3.551082233	0.891702061	NA	NA
ASV_16s_11742	0.080985499	-0.290725471	3.551633868	0.393406427	NA	NA
ASV_16s_11743	0.439874805	-0.033096746	3.548875791	1.357445705	0.986839156	NA
ASV_16s_11744	0.390504013	-0.039696189	3.550273381	1.465009655	0.983496361	NA
ASV_16s_11745	0.333133279	0.801952342	3.550672877	1.20120921	0.990899029	NA
ASV_16s_11746	0.310771921	0.801947173	3.550692007	1.159170942	0.991836796	NA
ASV_16s_11747	0.209943581	-0.078757067	3.550774781	0.961515843	NA	NA
ASV_16s_11748	0.263770365	-0.116317886	3.550166162	1.361612591	NA	NA
ASV_16s_11749	0.4145578	0.0836717	3.550493715	1.990754076	0.960350334	NA
ASV_16s_11750	0.357827401	0.353296018	3.550113566	1.557972652	0.980234512	NA
ASV_16s_11751	0.374064214	0.725143233	3.550263277	1.271449437	0.989187693	NA
ASV_16s_11752	0.326007162	0.19938695	3.550237566	1.625332488	NA	NA
ASV_16s_11753	0.382723005	0.692214053	3.55084299	1.419642019	NA	NA
ASV_16s_11754	0.302538309	-0.073993312	3.550712903	1.168825506	0.991627059	NA
ASV_16s_11755	0.264471366	0.160522825	3.550778114	1.793029531	0.970403073	NA
ASV_16s_11756	0.280886418	0.360364305	3.550955548	1.150433679	NA	NA
ASV_16s_11757	0.280886418	0.360364305	3.550955548	1.150433679	NA	NA
ASV_16s_11758	0.280886418	0.360364305	3.550955548	1.150433679	NA	NA
ASV_16s_11759	0.280886418	0.360364305	3.550955548	1.150433679	NA	NA
ASV_16s_11760	0.280886418	0.360364305	3.550955548	1.150433679	NA	NA
ASV_16s_11761	0.299876813	0.315939508	3.550826676	1.144587092	0.992147312	NA
ASV_16s_11762	0.293375097	0.315939513	3.550831962	1.133490191	NA	NA
ASV_16s_11763	0.293375097	0.482878181	3.55080615	1.83421098	0.968443939	NA
ASV_16s_11764	0.388099203	0.734652358	3.550853063	1.250680696	NA	NA
ASV_16s_11765	0.311864358	0.693364734	3.550790102	1.099176823	0.993066205	NA
ASV_16s_11766	0.341535024	0.693431878	3.550769512	1.145163765	0.992135177	NA
ASV_16s_11767	0.373276763	0.693498693	3.550751099	1.190174656	0.991151367	NA
ASV_16s_11768	0.248856916	0.275688147	3.551247206	0.744913344	NA	NA
ASV_16s_11769	0.249633673	0.188149415	3.550987759	0.881576079	NA	NA
ASV_16s_11770	0.253517455	-0.063113355	3.550408874	1.302001169	NA	NA
ASV_16s_11771	0.24898811	-0.115258941	3.550374454	1.344521056	NA	NA
ASV_16s_11772	0.255847725	-0.173118038	3.550285044	1.389575416	0.985888421	NA
ASV_16s_11773	0.251289859	-0.166117598	3.550315798	1.372491739	0.986398956	NA
ASV_16s_11774	0.271178372	-0.205443647	3.550186278	1.438657929	0.984357677	NA
ASV_16s_11775	0.256365562	-0.1963681	3.550267593	1.394348765	0.985743728	NA
ASV_16s_11776	0.249033742	-0.208851347	3.550302149	1.369468946	0.986488102	NA
ASV_16s_11777	0.268446115	-0.244904836	3.550184171	1.427129214	0.984725794	NA
ASV_16s_11778	0.270317562	-0.257855892	3.55017152	1.431043991	0.984601386	NA
ASV_16s_11779	0.257660157	-0.254253221	3.55023701	1.390754236	0.985852771	NA
ASV_16s_11780	0.265713858	-0.285993531	3.55018799	1.403137	0.985474987	NA
ASV_16s_11781	0.24907367	-0.291124471	3.550276253	1.345917974	NA	NA
ASV_16s_11782	0.271947751	-0.326490111	3.550152864	1.396959152	0.985664221	NA
ASV_16s_11783	0.269620663	-0.38420677	3.550170573	1.316546325	0.987991416	NA
ASV_16s_11784	0.350141111	-0.681232482	3.550007659	0.874888476	NA	NA
ASV_16s_11785	0.249204864	-0.671054784	3.550493144	0.764809731	NA	NA
ASV_16s_11786	0.249204864	-0.671054784	3.550493144	0.764809731	NA	NA
ASV_16s_11787	0.079398263	-0.16921153	3.550841006	0.393748152	NA	NA
ASV_16s_11788	0.110683922	0.220130375	3.550837904	0.501711555	NA	NA
ASV_16s_11789	0.273654221	-0.157920221	3.5501402	1.349823045	0.987058811	NA

ASV_16s_11790	0.271640696	-0.143901041	3.550145891	1.205007535	0.990811143	NA
ASV_16s_11791	0.260033527	-0.368339129	3.550333278	0.766882981	NA	NA
ASV_16s_11792	0.338833436	-0.777383695	3.549895283	1.004746064	0.994751612	NA
ASV_16s_11793	0.270538085	0.170684668	3.549758899	1.463389757	0.983550109	NA
ASV_16s_11794	0.269330327	0.004243797	3.549685833	1.358376321	0.986812185	NA
ASV_16s_11795	0.337278002	-0.776844413	3.549731738	1.199027572	NA	NA
ASV_16s_11796	0.286409065	-0.734819569	3.550058198	1.130405524	NA	NA
ASV_16s_11797	0.340891616	-0.77404171	3.54992778	1.072843122	0.99356636	NA
ASV_16s_11798	0.322872306	-0.760432914	3.549800653	1.215868916	0.990556918	NA
ASV_16s_11799	0.297165201	0.373516253	3.55008532	1.497654067	NA	NA
ASV_16s_11800	0.334383708	-0.726206205	3.549802613	1.345055752	0.987195041	NA
ASV_16s_11801	0.268423961	0.68716489	3.550877271	1.156550908	NA	NA
ASV_16s_11802	0.329052563	-0.68616111	3.549897118	1.362771444	0.98668435	NA
ASV_16s_11803	0.344620341	0.322546888	3.551577606	0.70717735	NA	NA
ASV_16s_11804	0.30717748	1.231404706	3.549535119	0.939810015	0.99574194	NA
ASV_16s_11805	0.328495893	0.320020351	3.549048796	1.297358836	0.988509844	NA
ASV_16s_11806	0.270471585	-0.210347101	3.549839439	1.10963964	NA	NA
ASV_16s_11807	0.265525226	0.342700691	3.551785929	0.547527869	NA	NA
ASV_16s_11808	0.248047982	-0.149745838	3.550715387	1.61417162	0.978091854	NA
ASV_16s_11809	0.344471711	0.029984332	3.550489404	1.282882667	0.988891704	NA
ASV_16s_11810	0.263158874	0.250056311	3.551584482	0.201411119	0.999974231	NA
ASV_16s_11811	0.344020886	-0.350290656	3.551183923	0.473673299	0.999537283	NA
ASV_16s_11812	0.277461322	-0.325054551	3.549836285	1.138561347	0.992273408	NA
ASV_16s_11813	0.343165863	0.165977304	3.550628825	1.221201441	0.990430518	NA
ASV_16s_11814	0.326915822	1.200851334	3.550331928	0.799611683	NA	NA
ASV_16s_11815	0.413005994	1.0535909	3.550922188	0.93455396	0.995816333	NA
ASV_16s_11816	0.28483121	1.299440336	3.550642761	0.762175686	NA	NA
ASV_16s_11817	0.270039559	0.392301889	3.551095309	0.77247754	NA	NA
ASV_16s_11818	0.270039559	0.392301889	3.551095309	0.77247754	NA	NA
ASV_16s_11819	0.283348841	0.39469333	3.551017254	0.788397673	NA	NA
ASV_16s_11820	0.285692264	0.354291683	3.551857304	0.447932714	NA	NA
ASV_16s_11821	0.375277908	0.364966962	3.551664944	0.500009709	NA	NA
ASV_16s_11822	0.29520743	-1.196496663	3.550038019	0.49724421	NA	NA
ASV_16s_11823	0.286940786	-1.130486767	3.550137301	0.516623162	0.999383309	NA
ASV_16s_11824	0.208994832	-0.550827743	3.550575499	0.686939054	NA	NA
ASV_16s_11825	0.208994832	-0.550827743	3.550575499	0.686939054	NA	NA
ASV_16s_11826	0.253389027	-0.792042387	3.550465614	0.626585611	NA	NA
ASV_16s_11827	0.344576208	1.89959046	3.54999931	0.498411734	NA	NA
ASV_16s_11828	0.205831541	-0.177004447	3.550115301	0.876751877	NA	NA
ASV_16s_11829	0.257801411	0.414792228	3.550827169	0.68983746	NA	NA
ASV_16s_11830	0.089764254	0.345251304	3.551724927	0.358081422	NA	NA
ASV_16s_11831	0.207395365	-0.542278495	3.550273986	0.614934565	NA	NA
ASV_16s_11832	0.144843583	-0.500750233	3.550816126	0.501810535	NA	NA
ASV_16s_11833	0.17590921	-0.53117386	3.550530217	0.562963238	NA	NA
ASV_16s_11834	0.161217006	-0.520660819	3.550664692	0.535484548	NA	NA
ASV_16s_11835	0.254492758	-0.078911239	3.550729332	1.057512554	0.993846669	NA
ASV_16s_11836	0.171911779	0.220059706	3.550745014	0.703077229	0.998311981	NA
ASV_16s_11837	0.17509288	0.220057386	3.550741958	0.711956471	0.998242173	NA
ASV_16s_11838	0.183234809	0.220051937	3.550734619	0.734077453	NA	NA
ASV_16s_11839	0.277838788	-0.929366795	3.550143148	0.634588474	0.998789414	NA
ASV_16s_11840	0.31764812	0.832459777	3.550213748	1.202545022	NA	NA
ASV_16s_11841	0.278634577	-0.068387876	3.550123724	1.477797681	0.983068363	NA
ASV_16s_11842	0.289779023	-0.059308795	3.550528284	1.072168176	NA	NA
ASV_16s_11843	0.236323211	0.505683649	3.550596727	0.953617952	NA	NA
ASV_16s_11844	0.310591533	-0.327235284	3.549615077	1.430388955	NA	NA
ASV_16s_11845	0.276620274	-0.059083352	3.550447253	1.322774844	NA	NA
ASV_16s_11846	0.315532321	1.154892895	3.550664434	0.772399143	NA	NA
ASV_16s_11847	0.123221303	0.282474067	3.551689342	0.51928436	NA	NA
ASV_16s_11848	0.54511944	-0.607885337	3.549890232	1.00657201	NA	NA
ASV_16s_11849	0.485319516	-0.14625962	3.54882216	1.557370842	NA	NA
ASV_16s_11850	0.357742665	0.436848206	3.550749387	1.196409123	0.991009342	NA
ASV_16s_11851	0.326915822	-0.548533311	3.549702477	0.75991478	NA	NA
ASV_16s_11852	0.320387683	1.034293503	3.550372085	1.226742095	0.990298076	NA
ASV_16s_11853	0.642530791	-0.029612566	3.55064592	1.586203481	NA	NA
ASV_16s_11854	0.449814936	0.426750231	3.550744949	2.344755167	0.938326699	NA
ASV_16s_11855	0.407652474	0.12337479	3.550503959	1.947927881	0.962666085	NA
ASV_16s_11856	0.367171527	0.312524593	3.54933171	1.444814876	0.984158916	NA

ASV_16s_11857	0.312012554	-0.264404065	3.55002471	1.22428642	0.990356915	NA
ASV_16s_11858	0.480604636	0.337913492	3.549684111	1.956016256	NA	NA
ASV_16s_11859	0.413918789	0.348963064	3.549912115	2.532048558	NA	NA
ASV_16s_11860	0.272523529	-0.029090195	3.550817411	1.151142924	NA	NA
ASV_16s_11861	0.324094966	0.089104455	3.550345807	1.717816602	0.973798426	NA
ASV_16s_11862	0.228538853	0.030724047	3.550763271	1.037577468	NA	NA
ASV_16s_11863	0.290898454	0.342368992	3.550046182	1.507479548	NA	NA
ASV_16s_11864	0.348874612	0.329407672	3.550818858	1.042671947	0.994110497	NA
ASV_16s_11865	0.276355992	0.3603625	3.550961459	1.1421805	NA	NA
ASV_16s_11866	0.276355992	0.3603625	3.550961459	1.1421805	NA	NA
ASV_16s_11867	0.276355992	0.3603625	3.550961459	1.1421805	NA	NA
ASV_16s_11868	0.323026974	0.315939367	3.550809579	1.182213708	0.991330673	NA
ASV_16s_11869	0.359191231	0.315939355	3.550787269	1.235936662	0.990075789	NA
ASV_16s_11870	0.306750089	0.379529404	3.55080578	1.604153178	0.97848332	NA
ASV_16s_11871	0.308922248	0.315939373	3.550819694	1.159601646	0.991827511	NA
ASV_16s_11872	0.303677708	0.315939375	3.550823695	1.150928795	0.992013216	NA
ASV_16s_11873	0.2995935	0.315939522	3.550826907	1.144080694	0.992157959	NA
ASV_16s_11874	0.381839538	0.734635771	3.550857601	1.24240657	NA	NA
ASV_16s_11875	0.381839538	0.734635771	3.550857601	1.24240657	NA	NA
ASV_16s_11876	0.189146378	0.733654209	3.551142491	0.890975011	NA	NA
ASV_16s_11877	0.189146378	0.733654209	3.551142491	0.890975011	NA	NA
ASV_16s_11878	0.333274656	0.693417212	3.550774881	1.132748316	0.992393838	NA
ASV_16s_11879	0.36505962	0.693483579	3.550755557	1.178919401	NA	NA
ASV_16s_11880	0.319540012	0.693384481	3.550784421	1.111409158	0.992825761	NA
ASV_16s_11881	0.244777294	0.27609809	3.551273547	0.739391822	NA	NA
ASV_16s_11882	0.244777294	0.27609809	3.551273547	0.739391822	NA	NA
ASV_16s_11883	0.244777294	0.27609809	3.551273547	0.739391822	NA	NA
ASV_16s_11884	0.25021459	-0.105485649	3.550377563	1.335073925	NA	NA
ASV_16s_11885	0.244902784	-0.10757979	3.550407775	1.326124282	NA	NA
ASV_16s_11886	0.250991347	-0.142420537	3.550336721	1.363857651	NA	NA
ASV_16s_11887	0.250991347	-0.142420537	3.550336721	1.363857651	NA	NA
ASV_16s_11888	0.251250265	-0.154479221	3.550325092	1.368964472	0.986502945	NA
ASV_16s_11889	0.252027022	-0.19014213	3.550295488	1.379034242	0.986204788	NA
ASV_16s_11890	0.244942712	-0.191272814	3.550336133	1.354679294	0.986919134	NA
ASV_16s_11891	0.244948416	-0.203013025	3.550329274	1.355280914	0.986901766	NA
ASV_16s_11892	0.258806628	-0.233171248	3.550237556	1.398609384	0.98561382	NA
ASV_16s_11893	0.244959825	-0.22656812	3.550317981	1.353691632	0.986947616	NA
ASV_16s_11894	0.264946308	-0.262688842	3.550197172	1.411712162	0.985209829	NA
ASV_16s_11895	0.249534803	-0.267602526	3.550278944	1.35842694	0.986810717	NA
ASV_16s_11896	0.250041232	-0.268201481	3.550275947	1.359907659	0.98676773	NA
ASV_16s_11897	0.25982574	-0.279504708	3.550219794	1.386313907	0.985986773	NA
ASV_16s_11898	0.247773707	-0.289629716	3.55028406	1.342238009	0.987275147	NA
ASV_16s_11899	0.244988345	-0.286418048	3.550300956	1.33440594	NA	NA
ASV_16s_11900	0.265318445	-0.320119785	3.550185874	1.380733518	0.986154082	NA
ASV_16s_11901	0.244999753	-0.311293131	3.550297495	1.318900398	NA	NA
ASV_16s_11902	0.24803833	-0.314558291	3.550279508	1.326991206	0.987703282	NA
ASV_16s_11903	0.250334375	-0.354824534	3.550267831	1.29023244	0.988698821	NA
ASV_16s_11904	0.264676749	-0.36766294	3.550192217	1.323355896	0.987804041	NA
ASV_16s_11905	0.245016865	-0.349740218	3.550298205	1.277295322	NA	NA
ASV_16s_11906	0.245033977	-0.390116183	3.550306709	1.225383515	NA	NA
ASV_16s_11907	0.306134653	-0.68042403	3.550188832	0.831748944	NA	NA
ASV_16s_11908	0.290019972	-0.680069882	3.550268784	0.814397177	NA	NA
ASV_16s_11909	0.245119538	-0.668658183	3.550514066	0.759243935	NA	NA
ASV_16s_11910	0.078096652	-0.169189519	3.550846562	0.38753422	NA	NA
ASV_16s_11911	0.076550026	0.024921926	3.550854738	0.597829991	0.999003622	NA
ASV_16s_11912	0.071887079	-0.071464653	3.550863265	0.508257918	NA	NA
ASV_16s_11913	0.108869432	0.220133685	3.550842249	0.494673412	NA	NA
ASV_16s_11914	0.108869432	0.220133685	3.550842249	0.494673412	NA	NA
ASV_16s_11915	0.344401093	0.688280949	3.550747409	1.124690401	NA	NA
ASV_16s_11916	0.27445817	0.561287384	3.551225695	0.901475834	NA	NA
ASV_16s_11917	0.265330833	0.142515851	3.5501937	1.398251986	0.985624744	NA
ASV_16s_11918	0.262487677	-0.009605042	3.550245328	1.181377716	0.99134937	NA
ASV_16s_11919	0.260259287	-0.29925037	3.549924957	0.948192734	NA	NA
ASV_16s_11920	0.321737662	-0.773537753	3.549836016	1.135580316	0.992335316	NA
ASV_16s_11921	0.316368309	-0.761797031	3.549839237	1.174176876	0.991509366	NA
ASV_16s_11922	0.278511899	0.202869322	3.549690622	1.50801394	0.98203092	NA
ASV_16s_11923	0.232361236	-0.406258292	3.551184047	0.837969781	NA	NA

ASV_16s_11924	0.278509069	0.246003324	3.549837032	1.521406227	0.98155931	NA
ASV_16s_11925	0.321304489	-0.439665822	3.549475904	1.192812713	0.991091443	NA
ASV_16s_11926	0.255180502	-0.712165734	3.550447323	0.921208775	0.996001428	NA
ASV_16s_11927	0.314723727	-0.697254139	3.549905601	1.307581221	0.988235394	NA
ASV_16s_11928	0.314496552	-0.745641505	3.549814663	1.084933372	0.993339677	NA
ASV_16s_11929	0.313253826	0.678748843	3.549450206	0.807864421	0.997362864	NA
ASV_16s_11930	0.269771275	-0.110699626	3.550053797	1.147061848	NA	NA
ASV_16s_11931	0.342985883	-0.744389934	3.550072652	1.141693232	0.99220803	NA
ASV_16s_11932	0.312088236	-0.620839198	3.550053547	1.334292546	0.987499377	NA
ASV_16s_11933	0.260252954	-0.472775954	3.550519242	1.256239276	NA	NA
ASV_16s_11934	0.261407499	-0.618969701	3.550844838	0.823077799	0.997201242	NA
ASV_16s_11935	0.327244385	-0.588823599	3.550918718	0.862043579	NA	NA
ASV_16s_11936	0.305082782	-0.560249532	3.550199899	1.329895081	NA	NA
ASV_16s_11937	0.318050988	-0.548106553	3.550201686	1.336167237	0.98744669	NA
ASV_16s_11938	0.326949587	0.625803816	3.550828112	0.668469907	0.998566444	NA
ASV_16s_11939	0.26326028	0.350427129	3.551393392	0.726137957	NA	NA
ASV_16s_11940	0.311494296	-0.339299154	3.550062322	1.191233253	0.991127351	NA
ASV_16s_11941	0.315059164	0.182874565	3.549538626	1.329904092	NA	NA
ASV_16s_11942	0.313266242	-1.191712972	3.549683479	0.733766528	0.998062625	NA
ASV_16s_11943	0.399662213	0.576484909	3.549948782	1.621459877	NA	NA
ASV_16s_11944	0.318537742	0.273701857	3.550581023	1.313080401	0.988086103	NA
ASV_16s_11945	0.350922061	0.323556042	3.550192614	1.61614444	NA	NA
ASV_16s_11946	0.313396628	-0.399334182	3.55045213	1.275007516	0.989096108	NA
ASV_16s_11947	0.143654867	0.661645615	3.551125307	0.450990001	NA	NA
ASV_16s_11948	0.251758563	-0.1421092	3.550182115	0.856876742	0.996819257	NA
ASV_16s_11949	0.222248397	0.286988555	3.551711621	0.406747971	NA	NA
ASV_16s_11950	0.31275764	0.179295938	3.551210645	0.659561142	0.998627524	NA
ASV_16s_11951	0.297987524	1.226827876	3.550787522	0.981169872	NA	NA
ASV_16s_11952	0.418258421	0.05702754	3.549195059	0.96874068	NA	NA
ASV_16s_11953	0.335231124	1.642293588	3.549997921	0.883951295	NA	NA
ASV_16s_11954	0.321556547	0.400978742	3.550827077	0.830268473	NA	NA
ASV_16s_11955	0.299569419	1.302912516	3.550389343	0.924850087	NA	NA
ASV_16s_11956	0.390937568	0.403284534	3.550508338	0.893534519	NA	NA
ASV_16s_11957	0.205833963	0.375589236	3.551475371	0.681134286	NA	NA
ASV_16s_11958	0.23185977	0.351922355	3.551938984	0.413330501	NA	NA
ASV_16s_11959	0.278703778	0.393869963	3.551043769	0.782905851	NA	NA
ASV_16s_11960	0.34114095	0.384999931	3.551387904	0.525429997	0.999347937	NA
ASV_16s_11961	0.314052372	-0.839108329	3.549959762	0.896968775	0.996323893	NA
ASV_16s_11962	0.269076362	-0.563350574	3.550182736	0.768155263	NA	NA
ASV_16s_11963	0.208306145	1.24840083	3.550753762	0.592464584	0.999032579	NA
ASV_16s_11964	0.088292708	0.344992899	3.551729739	0.353639648	NA	NA
ASV_16s_11965	0.088292708	0.344992899	3.551729739	0.353639648	NA	NA
ASV_16s_11966	0.088292708	0.344992899	3.551729739	0.353639648	NA	NA
ASV_16s_11967	0.088292708	0.344992899	3.551729739	0.353639648	NA	NA
ASV_16s_11968	0.142469098	-0.497638192	3.550840557	0.49668089	NA	NA
ASV_16s_11969	0.125707551	1.098558559	3.551147715	0.424316362	NA	NA
ASV_16s_11970	0.242094466	-0.078874255	3.550740312	1.03242598	NA	NA
ASV_16s_11971	0.18023096	0.220053947	3.550737253	0.725986738	NA	NA
ASV_16s_11972	0.18023096	0.220053947	3.550737253	0.725986738	NA	NA
ASV_16s_11973	0.189859093	0.596088765	3.550694663	0.684205658	NA	NA
ASV_16s_11974	0.250022759	-0.068429594	3.550515014	1.328105343	NA	NA
ASV_16s_11975	0.26134261	-0.069060244	3.550407931	1.398044934	0.985631071	NA
ASV_16s_11976	0.252004833	-0.068969013	3.550387981	1.38225266	NA	NA
ASV_16s_11977	0.339350988	1.66071925	3.55010208	1.084250732	NA	NA
ASV_16s_11978	0.253491388	-0.068843894	3.550296734	1.413155184	NA	NA
ASV_16s_11979	0.266314349	0.064007828	3.550372625	1.448622633	NA	NA
ASV_16s_11980	0.293120191	1.787300513	3.550185231	0.818375412	0.997251873	NA
ASV_16s_11981	0.201130877	0.688961075	3.550494434	0.826253808	NA	NA
ASV_16s_11982	0.351358799	0.449781485	3.551536112	0.823223148	0.997199667	NA
ASV_16s_11983	0.276458463	-0.068452463	3.550116853	1.482282636	NA	NA
ASV_16s_11984	0.311463601	-0.720944903	3.549945816	0.765511818	0.997780194	NA
ASV_16s_11985	0.272266036	-0.068140553	3.550174868	1.437088449	NA	NA
ASV_16s_11986	0.384582229	1.484435975	3.550479199	0.810140788	NA	NA
ASV_16s_11987	0.299246567	1.336627052	3.550487403	1.007510456	NA	NA
ASV_16s_11988	0.285378795	0.31535651	3.550782983	0.823752739	NA	NA
ASV_16s_11989	0.203285148	0.751102097	3.551317045	0.966958275	NA	NA
ASV_16s_11990	0.22009338	1.00505654	3.551274542	0.828269983	NA	NA

ASV_16s_11991	0.274242729	0.111717744	3.550607264	1.246675873	NA	NA
ASV_16s_11992	0.307430025	1.347233447	3.550599901	0.925596954	NA	NA
ASV_16s_11993	0.204099731	0.279532906	3.551532718	0.696274655	NA	NA
ASV_16s_11994	0.078373064	-0.282404763	3.551646585	0.384611953	NA	NA
ASV_16s_11995	0.321556547	-0.548464334	3.549719334	0.754598841	NA	NA
ASV_16s_11996	0.321556547	-0.548464334	3.549719334	0.754598841	NA	NA
ASV_16s_11997	0.222010582	-0.117392761	3.550396661	1.188029563	NA	NA
ASV_16s_11998	0.253798153	-0.117326961	3.55004007	1.366206453	0.986583917	NA
ASV_16s_11999	0.277350699	-0.084699824	3.550694177	1.022946607	NA	NA
ASV_16s_12000	0.361693305	0.34354693	3.54990758	1.691338832	0.974937623	NA
ASV_16s_12001	0.395168632	0.720939849	3.54991082	1.571024538	0.979748436	NA
ASV_16s_12002	0.268055931	-0.029075464	3.550822367	1.14279092	NA	NA
ASV_16s_12003	0.268055931	-0.029075464	3.550822367	1.14279092	NA	NA
ASV_16s_12004	0.276247915	0.343062111	3.550083907	1.469990455	NA	NA
ASV_16s_12005	0.306288015	-0.074002479	3.55071046	1.175110926	NA	NA
ASV_16s_12006	0.374050526	0.343183477	3.549879227	1.674040451	NA	NA
ASV_16s_12007	0.321244513	0.356106639	3.550718226	0.892512198	0.996381274	NA
ASV_16s_12008	0.271825566	0.360360561	3.550967564	1.13379367	NA	NA
ASV_16s_12009	0.271825566	0.360360561	3.550967564	1.13379367	NA	NA
ASV_16s_12010	0.271825566	0.360360561	3.550967564	1.13379367	NA	NA
ASV_16s_12011	0.264677237	0.383472952	3.550967274	1.307169049	NA	NA
ASV_16s_12012	0.315780764	0.315939521	3.550814671	1.17068647	0.991586248	NA
ASV_16s_12013	0.343884398	0.315939383	3.55079615	1.213827915	0.99060502	NA
ASV_16s_12014	0.294177252	0.315939539	3.550831308	1.13481464	0.992351166	NA
ASV_16s_12015	0.283911384	0.315939549	3.5508401	1.116838281	NA	NA
ASV_16s_12016	0.283911384	0.466509614	3.550813589	1.791415157	0.97047843	NA
ASV_16s_12017	0.392511767	1.166502287	3.550592438	0.98041351	NA	NA
ASV_16s_12018	0.318861773	0.802559559	3.550771164	1.029060239	0.994346034	NA
ASV_16s_12019	0.375579873	0.734619703	3.55086229	1.23399621	NA	NA
ASV_16s_12020	0.375579873	0.734619703	3.55086229	1.23399621	NA	NA
ASV_16s_12021	0.375579873	0.734619703	3.55086229	1.23399621	NA	NA
ASV_16s_12022	0.376441383	1.181128549	3.550300208	0.914683577	NA	NA
ASV_16s_12023	0.354451566	0.693462914	3.550761626	1.163945826	0.991733483	NA
ASV_16s_12024	0.325018005	0.693394941	3.550780521	1.120015532	0.992653476	NA
ASV_16s_12025	0.283911384	0.693285314	3.55081343	1.051732583	NA	NA
ASV_16s_12026	0.378808611	0.69350901	3.550748211	1.197588942	0.990982306	NA
ASV_16s_12027	0.158665335	0.884727993	3.551085926	0.543011357	NA	NA
ASV_16s_12028	0.240697673	0.276793428	3.551297639	0.73372988	NA	NA
ASV_16s_12029	0.240697673	0.276793428	3.551297639	0.73372988	NA	NA
ASV_16s_12030	0.24587605	-0.097112572	3.550413586	1.310828962	NA	NA
ASV_16s_12031	0.246134969	-0.109894921	3.550397687	1.323798811	NA	NA
ASV_16s_12032	0.246911725	-0.147293078	3.550356745	1.351582634	0.987008307	NA
ASV_16s_12033	0.251090954	-0.154188844	3.550326365	1.363252092	0.986670324	NA
ASV_16s_12034	0.247429563	-0.171639805	3.550334467	1.36024485	0.986757929	NA
ASV_16s_12035	0.240857387	-0.18504815	3.550365118	1.339574871	0.987350575	NA
ASV_16s_12036	0.240857387	-0.18504815	3.550365118	1.339574871	0.987350575	NA
ASV_16s_12037	0.243654157	-0.212937092	3.550332008	1.350397395	0.987042339	NA
ASV_16s_12038	0.244672721	-0.22616209	3.550319932	1.352659364	0.986977343	NA
ASV_16s_12039	0.240885907	-0.244939511	3.550335921	1.335786548	0.9874574	NA
ASV_16s_12040	0.240885907	-0.244939511	3.550335921	1.335786548	0.9874574	NA
ASV_16s_12041	0.24367127	-0.248543983	3.550317944	1.344580534	0.987208572	NA
ASV_16s_12042	0.2890642	-0.309276869	3.550076521	1.462132938	0.983591737	NA
ASV_16s_12043	0.262474059	-0.29394918	3.550203179	1.388621443	0.985917232	NA
ASV_16s_12044	0.240897315	-0.269144963	3.550329711	1.328012004	NA	NA
ASV_16s_12045	0.260599764	-0.303620887	3.550211531	1.376731946	0.986273306	NA
ASV_16s_12046	0.256357196	-0.311063592	3.550233377	1.358026496	0.986822328	NA
ASV_16s_12047	0.244217628	-0.335927861	3.550301906	1.290256452	0.988698188	NA
ASV_16s_12048	0.258040394	-0.387577607	3.550231776	1.273149415	0.989143995	NA
ASV_16s_12049	0.24423474	-0.375671776	3.550307603	1.242009864	0.989927246	NA
ASV_16s_12050	0.08604652	-0.361277206	3.551560335	0.420247114	NA	NA
ASV_16s_12051	0.328387498	-0.680859839	3.550091319	0.854240595	NA	NA
ASV_16s_12052	0.314478131	-0.680593435	3.550150725	0.84034898	0.997010043	NA
ASV_16s_12053	0.313901187	-0.680584503	3.5501533	0.839759414	0.997016706	NA
ASV_16s_12054	0.3321802	-0.680926913	3.550075977	0.857926939	NA	NA
ASV_16s_12055	0.076795041	-0.169166747	3.550852304	0.381258419	NA	NA
ASV_16s_12056	0.107054941	0.220137105	3.550846741	0.487554877	NA	NA
ASV_16s_12057	0.107054941	0.220137105	3.550846741	0.487554877	NA	NA

ASV_16s_12058	0.107054941	0.220137105	3.550846741	0.487554877	NA		NA
ASV_16s_12060	0.25503188	-0.021111025	3.550308661	1.339584809		0.987350294	NA
ASV_16s_12061	0.254575416	0.047039022	3.549987372	1.304564852		0.988316792	NA
ASV_16s_12062	0.245068048	0.205392633	3.550070004	1.176998622	NA		NA
ASV_16s_12063	0.253887002	-0.170886525	3.550379532	0.777674798		0.99766517	NA
ASV_16s_12064	0.30708973	0.784094904	3.551188325	0.93300236	NA		NA
ASV_16s_12065	0.249884881	-0.448657772	3.550535548	1.174749119		0.991496719	NA
ASV_16s_12066	0.285092029	-0.014402595	3.549576024	1.399416364		0.985589134	NA
ASV_16s_12067	0.248534736	-0.280983301	3.54999085	0.96356532	NA		NA
ASV_16s_12068	0.294143536	-0.694256675	3.55031915	0.872602432	NA		NA
ASV_16s_12069	0.31074	-0.759024625	3.549873277	1.161162431		0.991793806	NA
ASV_16s_12070	0.259189302	0.898114158	3.550045855	1.058004523		0.993837797	NA
ASV_16s_12071	0.298977131	-0.695387725	3.550175881	1.209972247		0.990695474	NA
ASV_16s_12072	0.324931073	-0.73527553	3.549815934	1.294068527		0.988597336	NA
ASV_16s_12073	0.303919992	-0.683022429	3.54996366	1.282934858	NA		NA
ASV_16s_12074	0.321759111	-0.673887936	3.55046356	0.838769453		0.997027874	NA
ASV_16s_12075	0.261997234	0.628904325	3.549929669	1.152218023		0.99198578	NA
ASV_16s_12076	0.292918849	0.811635229	3.55005634	1.428288182		0.984689027	NA
ASV_16s_12077	0.348562942	-0.323439691	3.549617076	1.331913321		0.987566047	NA
ASV_16s_12078	0.313988233	-0.650843585	3.549992204	1.33277028		0.987542059	NA
ASV_16s_12079	0.300841887	-0.566950054	3.550232711	1.363196996	NA		NA
ASV_16s_12080	0.303709844	-0.617254157	3.550075512	1.311637285		0.988125392	NA
ASV_16s_12081	0.309296094	-0.166761219	3.550421433	1.297625412	NA		NA
ASV_16s_12082	0.302005712	-0.536559712	3.550247078	1.294842457		0.988576794	NA
ASV_16s_12083	0.259631463	0.3514733	3.551356261	0.737988274	NA		NA
ASV_16s_12084	0.210129344	-1.060223419	3.550319877	0.383059647	NA		NA
ASV_16s_12085	0.308769096	-0.397514402	3.550492409	0.973461091		0.99524536	NA
ASV_16s_12086	0.330230904	0.679357103	3.550824721	1.212640773	NA		NA
ASV_16s_12087	0.364978495	-0.631056662	3.550115524	1.197864799	NA		NA
ASV_16s_12088	0.334111573	0.621995393	3.550804716	1.469952167		0.98333172	NA
ASV_16s_12089	0.300461087	-0.535290845	3.549990007	0.868162222	NA		NA
ASV_16s_12090	0.283159163	-0.072954225	3.550406393	1.196718637		0.991002254	NA
ASV_16s_12091	0.293155789	-0.002493182	3.550740572	1.130621968		0.992437593	NA
ASV_16s_12092	0.338235679	0.359134244	3.550427081	1.03461907		0.994250585	NA
ASV_16s_12093	0.289235797	1.155782892	3.551137862	0.44152932		0.999633668	NA
ASV_16s_12094	0.28429068	0.102017889	3.550776689	1.095329858		0.993140753	NA
ASV_16s_12095	0.174680561	0.063367563	3.550715602	0.693772355		0.998383131	NA
ASV_16s_12096	0.276712678	0.001863789	3.549822484	0.816290439		0.997274128	NA
ASV_16s_12097	0.266848481	1.286962031	3.550717375	0.739534007	NA		NA
ASV_16s_12098	0.300264703	1.307824811	3.550581774	0.780043559	NA		NA
ASV_16s_12099	0.296905757	1.306172379	3.550594851	0.776253401	NA		NA
ASV_16s_12100	0.201459191	1.575413766	3.550774679	0.388445501		0.999761185	NA
ASV_16s_12101	0.260403418	0.941618827	3.550132842	1.086250407	NA		NA
ASV_16s_12102	0.277016153	-0.025682823	3.549724024	1.183272314	NA		NA
ASV_16s_12103	0.300161577	1.364564487	3.550377208	0.797473715	NA		NA
ASV_16s_12104	0.291405244	-0.07633298	3.551000367	0.870631452	NA		NA
ASV_16s_12105	0.338661075	-0.564571173	3.54987697	0.841719042	NA		NA
ASV_16s_12106	0.17316255	-0.933121361	3.550808988	0.332883956	NA		NA
ASV_16s_12107	0.297370337	-0.563915711	3.550041375	0.800044872	NA		NA
ASV_16s_12108	0.320110562	-0.476645593	3.550300286	0.931904593	NA		NA
ASV_16s_12109	0.258180905	1.295610823	3.550563215	0.6665477		0.998579773	NA
ASV_16s_12110	0.230459404	0.40571725	3.550960201	0.652206098	NA		NA
ASV_16s_12111	0.086821163	0.344730632	3.551734664	0.349157471	NA		NA
ASV_16s_12112	0.140094613	-0.494317118	3.550865316	0.491456764	NA		NA
ASV_16s_12113	0.155931203	-0.514592759	3.550711024	0.524863963	NA		NA
ASV_16s_12114	0.155931203	-0.514592759	3.550711024	0.524863963	NA		NA
ASV_16s_12115	0.240924789	1.363072505	3.549896031	0.509934068	NA		NA
ASV_16s_12116	0.123612425	1.092990912	3.551160195	0.419093198	NA		NA
ASV_16s_12117	0.123612425	1.092990912	3.551160195	0.419093198	NA		NA
ASV_16s_12118	0.279179527	-0.078975924	3.550710377	1.104055423	NA		NA
ASV_16s_12119	0.171313887	0.220060236	3.550745609	0.701340629		0.998325415	NA
ASV_16s_12120	0.284951345	0.310935848	3.550817742	1.095823454	NA		NA
ASV_16s_12121	0.186694775	0.596082562	3.550697114	0.676094729	NA		NA
ASV_16s_12122	0.270536272	0.688115494	3.550799925	1.002753645	NA		NA
ASV_16s_12123	0.181229303	0.217806749	3.5507647	0.574968955		0.999123057	NA
ASV_16s_12124	0.302604244	-0.575496675	3.550380742	0.812335377	NA		NA
ASV_16s_12125	0.251934072	-0.068643363	3.550218653	1.419934568	NA		NA

ASV_16s_12126	0.31502951	0.372392394	3.551694645	0.690993572	NA	NA
ASV_16s_12127	0.254907182	-0.068333443	3.550201632	1.416304313	NA	NA
ASV_16s_12128	0.263077734	-0.068030741	3.550219077	1.407869362	0.985329014	NA
ASV_16s_12129	0.327045965	1.200861329	3.550331486	0.799696327	NA	NA
ASV_16s_12130	0.249704784	0.301100624	3.550496188	1.247852783	NA	NA
ASV_16s_12131	0.072846217	-0.264283841	3.551671668	0.365436078	NA	NA
ASV_16s_12132	0.072846217	-0.264283841	3.551671668	0.365436078	NA	NA
ASV_16s_12133	0.513743786	1.299214906	3.549919501	1.394676428	NA	NA
ASV_16s_12134	0.289457727	0.31945913	3.550895011	0.900185141	NA	NA
ASV_16s_12135	0.33099699	1.220657467	3.549990601	1.294426377	0.988587841	NA
ASV_16s_12136	0.233426761	-0.1170655	3.55010578	1.316534399	NA	NA
ASV_16s_12137	0.479176165	0.278810143	3.550714749	2.473286935	0.929095116	NA
ASV_16s_12138	0.440837404	0.698894567	3.550513662	2.031131461	0.958097218	NA
ASV_16s_12139	0.317576627	0.348839339	3.55007136	1.523209538	NA	NA
ASV_16s_12140	0.263588332	-0.029060192	3.550827489	1.134302096	NA	NA
ASV_16s_12141	0.35084238	0.344900069	3.550051692	1.533734603	NA	NA
ASV_16s_12142	0.309558925	0.343777095	3.550098994	1.493353314	NA	NA
ASV_16s_12143	0.312157242	0.345042957	3.55011402	1.489857844	NA	NA
ASV_16s_12144	0.352152131	0.373666038	3.550431911	1.383974283	NA	NA
ASV_16s_12145	0.286832641	0.350045709	3.550853635	0.852626074	NA	NA
ASV_16s_12146	0.292129864	0.340434248	3.55088899	0.89410703	0.996360807	NA
ASV_16s_12147	0.26729514	0.36035864	3.550973877	1.125267541	NA	NA
ASV_16s_12148	0.307634331	0.315939422	3.550820676	1.157417465	0.991874531	NA
ASV_16s_12149	0.299666986	0.315939427	3.55082686	1.144130411	0.992156914	NA
ASV_16s_12150	0.297970767	0.315939428	3.550828219	1.141261567	0.992217062	NA
ASV_16s_12151	0.29410239	0.315939431	3.550831376	1.134648181	0.992354609	NA
ASV_16s_12152	0.322743988	0.315939414	3.550809795	1.181639594	0.991343516	NA
ASV_16s_12153	0.279179527	0.315939566	3.550844375	1.108305937	NA	NA
ASV_16s_12154	0.265873003	0.688101991	3.550804215	0.994017524	NA	NA
ASV_16s_12155	0.265873003	0.688101991	3.550804215	0.994017524	NA	NA
ASV_16s_12156	0.379560816	1.081290014	3.550504803	0.964644562	0.995378885	NA
ASV_16s_12157	0.369320209	0.734603064	3.550867138	1.22544494	NA	NA
ASV_16s_12158	0.369320209	0.734603064	3.550867138	1.22544494	NA	NA
ASV_16s_12159	0.360417821	1.070591364	3.550546309	0.946186861	0.995650542	NA
ASV_16s_12160	0.182944857	0.733588908	3.551161555	0.874897736	NA	NA
ASV_16s_12161	0.347476238	0.693448683	3.550765819	1.153845772	0.991951056	NA
ASV_16s_12162	0.331492432	0.693410927	3.550776082	1.129978714	0.992450798	NA
ASV_16s_12163	0.353090452	0.693460303	3.550762435	1.161967178	NA	NA
ASV_16s_12164	0.311750443	0.69336505	3.550790205	1.098898338	0.993071618	NA
ASV_16s_12165	0.302981351	0.693341548	3.550797065	1.084478985	0.993348286	NA
ASV_16s_12166	0.40938735	0.693559389	3.550733614	1.236969665	NA	NA
ASV_16s_12167	0.23671502	-0.051529855	3.550534116	1.236393843	NA	NA
ASV_16s_12168	0.236732133	-0.091245793	3.550480027	1.280869617	NA	NA
ASV_16s_12169	0.241312815	-0.149734476	3.550389149	1.332887751	0.987538768	NA
ASV_16s_12170	0.253165163	-0.168955984	3.550303292	1.372505757	0.986398542	NA
ASV_16s_12171	0.236766357	-0.166390093	3.550404522	1.321575035	0.987853216	NA
ASV_16s_12172	0.253683001	-0.19248718	3.550284952	1.380042339	0.98617472	NA
ASV_16s_12173	0.259045266	-0.211135201	3.55024574	1.396557185	0.985676481	NA
ASV_16s_12174	0.24360886	-0.18923232	3.550345605	1.349618003	0.987064689	NA
ASV_16s_12175	0.243867779	-0.201409901	3.550336805	1.351284882	0.987016862	NA
ASV_16s_12176	0.24159455	-0.209978274	3.550346079	1.34321226	0.987247484	NA
ASV_16s_12177	0.263388428	-0.238810798	3.550211776	1.412702293	0.985179025	NA
ASV_16s_12178	0.240339884	-0.232166718	3.550343863	1.336457846	0.987438511	NA
ASV_16s_12179	0.2568893	-0.276150677	3.550236364	1.379255939	0.986198179	NA
ASV_16s_12180	0.241369855	-0.269706581	3.550326798	1.329352929	0.987637551	NA
ASV_16s_12181	0.239349841	-0.279673825	3.550336658	1.318029625	0.987950752	NA
ASV_16s_12182	0.236829102	-0.302114234	3.550348497	1.296362714	NA	NA
ASV_16s_12183	0.251953388	-0.331016635	3.550257052	1.323801368	0.987791722	NA
ASV_16s_12184	0.23684051	-0.328214158	3.550347134	1.270992193	NA	NA
ASV_16s_12185	0.241910509	-0.346659674	3.55031673	1.269449333	0.989238966	NA
ASV_16s_12186	0.238382615	-0.384232201	3.55034618	1.209937692	NA	NA
ASV_16s_12187	0.357717039	-0.681351002	3.549981137	0.88168421	NA	NA
ASV_16s_12188	0.310766823	-0.680518508	3.550167531	0.83651686	0.997053181	NA
ASV_16s_12189	0.308912241	-0.680480632	3.550176044	0.834596768	0.997074642	NA
ASV_16s_12190	0.307057659	-0.680442308	3.550184657	0.832665343	0.997096126	NA
ASV_16s_12191	0.332921056	-0.680939733	3.550073087	0.858618808	NA	NA
ASV_16s_12192	0.304859001	-0.680396263	3.550195006	0.830359379	NA	NA

ASV_16s_12193	0.251635344	-0.672267559	3.550480482	0.767983876	NA	NA
ASV_16s_12194	0.242772827	-0.667254466	3.55052646	0.75597415	NA	NA
ASV_16s_12195	0.236948887	-0.663741764	3.550557848	0.74784944	NA	NA
ASV_16s_12196	0.153374926	0.220075311	3.550765348	0.648484282	NA	NA
ASV_16s_12198	0.245444938	-0.030176794	3.550206874	1.141464299	0.992212821	NA
ASV_16s_12199	0.244694937	0.705363213	3.551336134	0.74007936	NA	NA
ASV_16s_12200	0.259759385	0.14546221	3.550268514	1.217554308	0.990517081	NA
ASV_16s_12201	0.305407423	1.286573544	3.550189956	0.504720765	0.999429012	NA
ASV_16s_12202	0.256716033	0.178911055	3.549920997	1.400363001	0.985560144	NA
ASV_16s_12203	0.253093599	0.696304193	3.551059132	0.935424197	NA	NA
ASV_16s_12204	0.245846308	0.283492552	3.550148311	1.36779629	0.986537277	NA
ASV_16s_12205	0.316338933	-1.255568596	3.550006872	0.561007499	0.999191022	NA
ASV_16s_12206	0.303115359	-0.760827709	3.549935781	1.115975159	0.992734679	NA
ASV_16s_12207	0.258525492	-0.24563217	3.550622613	1.045423651	0.994062134	NA
ASV_16s_12208	0.295565784	-0.762639024	3.550245265	0.936971556	0.99578222	NA
ASV_16s_12209	0.298500825	-0.743960338	3.549933312	1.154163502	0.991944267	NA
ASV_16s_12210	0.30721369	-0.750730697	3.54988663	1.175197207	0.991486809	NA
ASV_16s_12211	0.331081513	-0.76548287	3.549755184	1.229787129	0.990224806	NA
ASV_16s_12212	0.281593245	-0.683332422	3.550621295	0.858429901	NA	NA
ASV_16s_12213	0.308484796	-0.709313372	3.549903968	1.267454285	0.98928996	NA
ASV_16s_12214	0.31542949	-0.239373148	3.549421643	0.940815904	0.995727606	NA
ASV_16s_12215	0.305661371	-0.694636917	3.549936116	1.275335023	NA	NA
ASV_16s_12216	0.299650889	-0.681928157	3.54997816	1.270435881	0.989213694	NA
ASV_16s_12217	0.269605113	-0.08573806	3.549923267	1.029764847	0.994333992	NA
ASV_16s_12218	0.282871053	0.210671229	3.550227011	1.437239273	0.98440326	NA
ASV_16s_12219	0.31986555	0.110559136	3.549596762	1.088226326	0.993277071	NA
ASV_16s_12220	0.28873839	-0.556563221	3.550227786	1.269600599	0.989235093	NA
ASV_16s_12221	0.297884591	0.81566644	3.55000853	0.652306278	NA	NA
ASV_16s_12222	0.306043066	-0.128855159	3.549887029	1.325207605	NA	NA
ASV_16s_12223	0.267994267	-0.381568066	3.549494506	0.835941681	0.997059621	NA
ASV_16s_12224	0.289655813	-0.894475611	3.550328906	0.762153807	NA	NA
ASV_16s_12225	0.212879713	-0.710632388	3.550712561	0.400555745	NA	NA
ASV_16s_12226	0.398790325	0.236472233	3.549779722	1.739185724	0.972857748	NA
ASV_16s_12227	0.299306507	-0.299274353	3.550660944	0.965390121	0.995367688	NA
ASV_16s_12228	0.31578105	0.237630547	3.549198787	1.152856142	NA	NA
ASV_16s_12229	0.29768573	-0.14121317	3.550224226	1.095279259	NA	NA
ASV_16s_12230	0.198848781	0.605986788	3.550519176	0.837334091	NA	NA
ASV_16s_12231	0.276073705	0.310383498	3.550946367	0.847554255	0.996927818	NA
ASV_16s_12232	0.249721465	-0.292167265	3.550714081	0.856066797	0.996828787	NA
ASV_16s_12233	0.23014579	-0.346748837	3.550165833	1.137775366	NA	NA
ASV_16s_12234	0.237547025	1.098769402	3.551178934	0.827904105	NA	NA
ASV_16s_12235	0.304674105	1.734380476	3.550369573	0.48230905	NA	NA
ASV_16s_12236	0.295101285	1.39146404	3.55036111	0.967007141	0.995343344	NA
ASV_16s_12237	0.281988996	0.756090101	3.550980729	0.709770668	NA	NA
ASV_16s_12238	0.35314057	1.827786532	3.549945034	0.682360063	0.998467633	NA
ASV_16s_12239	0.277004332	1.063153234	3.550370817	1.010763277	NA	NA
ASV_16s_12240	0.333605765	-0.564499948	3.549895143	0.83681107	NA	NA
ASV_16s_12241	0.22993443	0.095939164	3.550258899	0.692810057	NA	NA
ASV_16s_12242	0.35839201	1.328157046	3.550369188	0.839119587	NA	NA
ASV_16s_12243	0.181578114	1.212005297	3.550867327	0.543565598	NA	NA
ASV_16s_12244	0.302867462	0.418797644	3.550577881	0.742212536	NA	NA
ASV_16s_12245	0.333634623	0.028216318	3.549742456	1.500758471	0.98228339	NA
ASV_16s_12246	0.203680473	0.24229224	3.550807317	0.371281079	0.99979477	NA
ASV_16s_12247	0.29040123	0.451800802	3.549915136	1.603612788	0.978504318	NA
ASV_16s_12248	0.226553313	0.404205301	3.550980344	0.646449824	NA	NA
ASV_16s_12249	0.435791808	-0.549571307	3.549450029	0.852101188	NA	NA
ASV_16s_12250	0.167257937	-0.526506945	3.550612605	0.547108792	NA	NA
ASV_16s_12251	0.153288301	-0.511371151	3.550734979	0.519441447	NA	NA
ASV_16s_12252	0.153288301	-0.511371151	3.550734979	0.519441447	NA	NA
ASV_16s_12253	0.234024651	-0.078848313	3.55074809	1.015394336	NA	NA
ASV_16s_12254	0.234024651	-0.078848313	3.55074809	1.015394336	NA	NA
ASV_16s_12255	0.242822862	-0.078874648	3.550739647	1.033867051	0.994263557	NA
ASV_16s_12256	0.277698925	0.195604646	3.550764329	1.721117805	NA	NA
ASV_16s_12257	0.166926208	0.220063482	3.550750049	0.688802232	0.998420303	NA
ASV_16s_12258	0.169789843	0.220061391	3.550747128	0.696995974	0.998358712	NA
ASV_16s_12259	0.26603941	-0.057638827	3.550703726	1.058163288	NA	NA
ASV_16s_12260	0.369728913	0.001335153	3.536353984	0.556909996	0.999210271	NA

ASV_16s_12261	0.170723139	0.571734594	3.550685698	0.677804945	NA	NA
ASV_16s_12262	0.297406664	-0.24778542	3.549983543	1.450488312	0.983974427	NA
ASV_16s_12263	0.2488902	-0.068504209	3.550219423	1.406109339	NA	NA
ASV_16s_12264	0.293679752	1.318253128	3.549918557	0.957895318	NA	NA
ASV_16s_12265	0.259219014	-0.068590339	3.550175521	1.443022161	0.984216945	NA
ASV_16s_12266	0.252358829	-0.067983366	3.550257795	1.385699252	NA	NA
ASV_16s_12267	0.316773797	1.201452503	3.55046696	0.941850485	0.995712831	NA
ASV_16s_12268	0.332618408	0.700884988	3.550860126	1.241022959	0.989951478	NA
ASV_16s_12269	0.255442086	-0.099577661	3.549959446	1.095329457	NA	NA
ASV_16s_12270	0.270471532	0.834755397	3.550833795	1.178489076	NA	NA
ASV_16s_12271	0.117161239	0.282970391	3.5517026	0.502768909	NA	NA
ASV_16s_12272	0.117161239	0.282970391	3.5517026	0.502768909	NA	NA
ASV_16s_12273	0.117161239	0.282970391	3.5517026	0.502768909	NA	NA
ASV_16s_12274	0.418802257	0.302228191	3.550854897	0.94910579	NA	NA
ASV_16s_12275	0.164288043	0.279604152	3.551618778	0.618359828	NA	NA
ASV_16s_12276	0.164288043	0.279604152	3.551618778	0.618359828	NA	NA
ASV_16s_12277	0.534541651	-0.566316187	3.549440945	0.988424109	NA	NA
ASV_16s_12278	0.304563801	-0.629630976	3.549640207	0.909796031	NA	NA
ASV_16s_12279	0.358399057	0.03457294	3.550242623	1.05172323	NA	NA
ASV_16s_12280	0.28414458	0.082623359	3.550827894	0.797075082	NA	NA
ASV_16s_12281	0.35090506	0.227756948	3.5506047	1.6764051	NA	NA
ASV_16s_12282	0.354100216	0.660878676	3.550854554	1.47804478	NA	NA
ASV_16s_12283	0.259120733	-0.029044365	3.550832788	1.125671858	NA	NA
ASV_16s_12284	0.216243715	-0.073712667	3.550792161	1.000250486	NA	NA
ASV_16s_12285	0.215305826	-0.073708383	3.55079337	0.998093948	0.994859225	NA
ASV_16s_12286	0.262764713	0.360356651	3.550980406	1.116597425	NA	NA
ASV_16s_12287	0.308147014	0.315939443	3.550820294	1.158233977	0.991856973	NA
ASV_16s_12288	0.296192307	0.315939451	3.550829667	1.138195897	0.992281014	NA
ASV_16s_12289	0.347559598	0.315939422	3.550793958	1.219156007	0.990479126	NA
ASV_16s_12290	0.281291583	0.315939462	3.550842456	1.112085834	0.992812309	NA
ASV_16s_12291	0.321842118	0.315939435	3.550810423	1.180187269	0.991375949	NA
ASV_16s_12292	0.280949388	0.315939463	3.550842766	1.111470134	0.992824549	NA
ASV_16s_12293	0.312063138	0.315939441	3.550817391	1.164566052	0.991720004	NA
ASV_16s_12294	0.382299153	1.115806085	3.550434578	0.950111069	0.995593673	NA
ASV_16s_12295	0.363060544	0.734585837	3.550872151	1.216747964	NA	NA
ASV_16s_12296	0.311426193	0.693364355	3.550790456	1.098348412	0.993082301	NA
ASV_16s_12297	0.343142894	0.693439598	3.550768512	1.14745764	0.992086791	NA
ASV_16s_12298	0.344133638	0.693441727	3.550767891	1.148918256	0.992055883	NA
ASV_16s_12299	0.321749666	0.693388254	3.550782841	1.114844089	0.992757309	NA
ASV_16s_12300	0.323002393	0.693391321	3.55078195	1.116811467	0.992717918	NA
ASV_16s_12301	0.325838096	0.693398175	3.550779958	1.121237055	0.992628814	NA
ASV_16s_12302	0.315816276	0.693373404	3.550787157	1.105424291	0.992944049	NA
ASV_16s_12303	0.314534952	0.693372388	3.550788112	1.1033652	0.992984459	NA
ASV_16s_12304	0.421484164	1.687272669	3.549962772	0.682292934	NA	NA
ASV_16s_12305	0.23253843	0.278219454	3.551348068	0.722133433	NA	NA
ASV_16s_12306	0.23253843	0.278219454	3.551348068	0.722133433	NA	NA
ASV_16s_12307	0.23253843	0.278219454	3.551348068	0.722133433	NA	NA
ASV_16s_12308	0.23253843	0.278219454	3.551348068	0.722133433	NA	NA
ASV_16s_12309	0.257347251	-0.094928263	3.550347878	1.322672307	NA	NA
ASV_16s_12310	0.236681131	-0.051414595	3.550534629	1.227439536	NA	NA
ASV_16s_12311	0.232641103	-0.069147188	3.550537452	1.241464432	NA	NA
ASV_16s_12312	0.237716807	-0.106007065	3.550456045	1.288127634	NA	NA
ASV_16s_12313	0.237716807	-0.106007065	3.550456045	1.288127634	NA	NA
ASV_16s_12314	0.237975725	-0.119106446	3.550439982	1.300437309	NA	NA
ASV_16s_12315	0.238234644	-0.132038856	3.550425159	1.311932961	NA	NA
ASV_16s_12316	0.238493563	-0.144827265	3.550411499	1.321194279	0.987863714	NA
ASV_16s_12317	0.238493563	-0.144827265	3.550411499	1.321194279	0.987863714	NA
ASV_16s_12318	0.232675327	-0.146934213	3.550448021	1.301301615	0.98840446	NA
ASV_16s_12319	0.239011401	-0.170057013	3.550387418	1.330239896	0.987612809	NA
ASV_16s_12320	0.239529238	-0.194955068	3.550367318	1.335613278	0.987462273	NA
ASV_16s_12321	0.232698144	-0.196684875	3.550410296	1.311267836	0.988135438	NA
ASV_16s_12322	0.239788157	-0.207339063	3.550358687	1.336819533	0.987428326	NA
ASV_16s_12323	0.239788157	-0.207339063	3.550358687	1.336819533	0.987428326	NA
ASV_16s_12324	0.239788157	-0.207339063	3.550358687	1.336819533	0.987428326	NA
ASV_16s_12325	0.232726664	-0.258811147	3.550383825	1.302731839	0.988366087	NA
ASV_16s_12326	0.236018456	-0.263041138	3.550361567	1.313013797	0.988087918	NA
ASV_16s_12327	0.267717195	-0.31084339	3.550174714	1.394200438	0.985748237	NA

ASV_16s_12328	0.24134167	-0.282063377	3.550323962	1.323759022	NA	NA
ASV_16s_12329	0.232743776	-0.297309402	3.550375471	1.284707142	NA	NA
ASV_16s_12330	0.236305895	-0.340954701	3.550351249	1.255091672	0.989602619	NA
ASV_16s_12331	0.238343021	-0.370214093	3.550342849	1.227886783	0.990270572	NA
ASV_16s_12332	0.232789409	-0.408427408	3.550391493	1.157188087	NA	NA
ASV_16s_12333	0.267675813	-0.448929087	3.550205353	1.206234642	0.990782637	NA
ASV_16s_12334	0.270535013	-0.450755752	3.55019168	1.211502277	0.990659644	NA
ASV_16s_12335	0.234342922	-0.509873166	3.550443144	1.006473868	NA	NA
ASV_16s_12336	0.247689	-0.517346133	3.550360478	1.030479678	0.994321758	NA
ASV_16s_12337	0.083129689	-0.349524369	3.551582574	0.410561455	NA	NA
ASV_16s_12338	0.083129689	-0.349524369	3.551582574	0.410561455	NA	NA
ASV_16s_12339	0.083129689	-0.349524369	3.551582574	0.410561455	NA	NA
ASV_16s_12340	0.303346351	-0.680363773	3.550202282	0.828740768	0.997139461	NA
ASV_16s_12341	0.307410044	-0.680449144	3.550183094	0.833006468	0.997092339	NA
ASV_16s_12342	0.261581437	-0.676293024	3.550426708	0.780817683	0.997634822	NA
ASV_16s_12343	0.282121422	-0.679846312	3.550311453	0.805473172	0.997387696	NA
ASV_16s_12344	0.247296804	-0.669915421	3.550503058	0.762135993	NA	NA
ASV_16s_12345	0.239193931	-0.66509755	3.550545656	0.750977995	0.997912654	NA
ASV_16s_12346	0.232863561	-0.661218619	3.550580772	0.74201594	NA	NA
ASV_16s_12347	0.07419182	-0.169118783	3.55086439	0.368516362	NA	NA
ASV_16s_12348	0.067279413	-0.168972759	3.550900941	0.333595144	0.999856824	NA
ASV_16s_12349	0.150730531	0.220077755	3.550768657	0.640258213	NA	NA
ASV_16s_12350	0.117417163	0.336080534	3.550793387	0.795270545	NA	NA
ASV_16s_12351	0.263472423	-0.423104233	3.550319446	1.241985807	0.989927837	NA
ASV_16s_12352	0.243998017	0.064997648	3.550407335	1.287182754	NA	NA
ASV_16s_12353	0.263730982	-0.043295833	3.550167467	1.348199934	0.987105292	NA
ASV_16s_12354	0.262355848	0.074097911	3.550215782	1.283525986	0.988874903	NA
ASV_16s_12355	0.247337767	0.280354329	3.550247445	1.293932276	0.98860095	NA
ASV_16s_12356	0.266044005	-0.222037867	3.550460158	0.797851305	0.99746582	NA
ASV_16s_12357	0.268011851	-0.4921761	3.55044303	1.203619215	0.990843327	NA
ASV_16s_12358	0.24368785	-0.324674993	3.550124725	0.861089355	0.996769391	NA
ASV_16s_12359	0.302755813	0.301653281	3.549850975	1.649548691	0.976676396	NA
ASV_16s_12360	0.257549186	0.201030152	3.549920639	1.433323612	0.984528661	NA
ASV_16s_12361	0.273029225	0.209884044	3.54977948	1.500355391	0.982297354	NA
ASV_16s_12362	0.249302885	-0.470856535	3.550516809	1.121612897	NA	NA
ASV_16s_12363	0.264517473	-0.283682536	3.549903479	1.037351373	0.994203294	NA
ASV_16s_12364	0.256010391	-0.047828697	3.550273255	0.840457968	0.99700881	NA
ASV_16s_12365	0.34691136	-0.563289296	3.550032977	1.333057867	0.987534002	NA
ASV_16s_12366	0.302393627	-0.743190643	3.549909399	1.171406065	0.991570433	NA
ASV_16s_12367	0.33427261	1.26607906	3.550557539	1.074167779	NA	NA
ASV_16s_12368	0.285104152	-0.708060499	3.549999267	1.173697394	NA	NA
ASV_16s_12369	0.316525854	-0.735855614	3.549845704	1.255777165	0.989585432	NA
ASV_16s_12370	0.261279735	-0.629739525	3.550554382	0.869175091	NA	NA
ASV_16s_12371	0.310185052	-0.68933973	3.549933714	1.300444833	NA	NA
ASV_16s_12372	0.283981517	-0.614897752	3.550121504	1.254867123	0.989608245	NA
ASV_16s_12373	0.312634879	-0.616644282	3.550207838	1.063691432	NA	NA
ASV_16s_12374	0.315330633	-0.269395102	3.549369591	1.384198487	0.98605034	NA
ASV_16s_12375	0.30694367	-0.61466969	3.550074649	1.313356138	NA	NA
ASV_16s_12376	0.27452881	0.250890381	3.550485393	1.324422593	0.987774529	NA
ASV_16s_12377	0.301886338	-0.571787019	3.550172092	1.299915621	NA	NA
ASV_16s_12378	0.326922841	0.22593356	3.549285136	1.030201536	NA	NA
ASV_16s_12379	0.256562588	1.642078203	3.550497576	0.541988389	0.999277751	NA
ASV_16s_12380	0.263029443	0.002674115	3.550519435	1.134748968	NA	NA
ASV_16s_12381	0.260646188	-0.659353307	3.551149192	0.661632643	0.998613479	NA
ASV_16s_12382	0.300670942	0.373919872	3.550687724	1.233246263	0.990141156	NA
ASV_16s_12383	0.292977442	1.791955715	3.54976288	0.369995439	0.999797145	NA
ASV_16s_12384	0.281281481	0.11023852	3.550646385	1.129798749	0.99245449	NA
ASV_16s_12385	0.333926962	0.207048663	3.549452323	1.220839747	NA	NA
ASV_16s_12386	0.319712327	0.137105693	3.550627451	1.193936838	0.991065831	NA
ASV_16s_12387	0.283289359	0.213607129	3.54944945	1.316710486	NA	NA
ASV_16s_12388	0.254448619	-0.49098797	3.55053745	1.131477647	NA	NA
ASV_16s_12389	0.289821905	0.12934424	3.550258492	1.489501845	0.982670881	NA
ASV_16s_12390	0.261952061	-0.072626145	3.550537287	1.120600027	NA	NA
ASV_16s_12391	0.37399688	1.620682958	3.550405414	0.701647054	0.99832305	NA
ASV_16s_12392	0.311115797	-0.002493867	3.550737758	1.17289849	NA	NA
ASV_16s_12393	0.26761173	-0.438906675	3.550689333	0.928910577	0.995895267	NA
ASV_16s_12394	0.209653842	0.108168375	3.550618161	0.84584705	0.996947432	NA

ASV_16s_12395	0.29284614	0.159253318	3.550792411	1.129352153	0.992463646	NA
ASV_16s_12396	0.316724347	0.36518229	2.586597626	1.859796915	0.967191035	NA
ASV_16s_12397	0.282711634	1.719259923	3.550418542	0.483252743	NA	NA
ASV_16s_12398	0.336591044	1.715046841	3.550420397	0.605628349	0.998960503	NA
ASV_16s_12399	0.252332047	0.387664786	3.551198334	0.749696266	NA	NA
ASV_16s_12400	0.195542265	0.37152268	3.551539139	0.663815226	NA	NA
ASV_16s_12401	0.295029707	-0.058841696	3.549620878	1.324118813	0.987782938	NA
ASV_16s_12402	0.264768589	0.391031122	3.55112575	0.765800622	NA	NA
ASV_16s_12403	0.318717283	0.888608301	3.550770857	0.955410661	0.995516114	NA
ASV_16s_12404	0.451168686	-0.858585267	3.549483374	0.627103287	NA	NA
ASV_16s_12405	0.283063232	-0.581874286	3.550472254	0.883314396	NA	NA
ASV_16s_12406	0.289542535	1.317956742	3.550466288	0.706113023	NA	NA
ASV_16s_12407	0.129221421	1.107580277	3.551127796	0.43283373	NA	NA
ASV_16s_12408	0.240896401	0.409617026	3.550909535	0.667035268	NA	NA
ASV_16s_12409	0.083878073	0.344194099	3.551744869	0.340101472	NA	NA
ASV_16s_12410	0.280488782	-0.073120578	3.550644032	1.095235318	NA	NA
ASV_16s_12411	0.135345643	-0.486729887	3.550913919	0.480717828	NA	NA
ASV_16s_12412	0.135345643	-0.486729887	3.550913919	0.480717828	NA	NA
ASV_16s_12413	0.152373832	-0.123934754	3.55055055	0.701246203	NA	NA
ASV_16s_12414	0.150645399	-0.508093489	3.550759662	0.513954617	NA	NA
ASV_16s_12415	0.295471948	1.321514524	3.550448418	0.713061632	NA	NA
ASV_16s_12416	0.269715815	-0.078952952	3.550717247	1.086623483	NA	NA
ASV_16s_12417	0.229989743	-0.078834639	3.550752183	1.006678143	NA	NA
ASV_16s_12418	0.241375664	-0.078870561	3.550741	1.030840321	0.994315579	NA
ASV_16s_12419	0.160273832	0.220069071	3.550757242	0.669312394	0.998560575	NA
ASV_16s_12420	0.162748021	0.220066989	3.5507545	0.676626624	0.998508952	NA
ASV_16s_12421	0.180366138	0.596069484	3.550702275	0.659526262	NA	NA
ASV_16s_12422	0.180366138	0.596069484	3.550702275	0.659526262	NA	NA
ASV_16s_12423	0.180366138	0.596069484	3.550702275	0.659526262	NA	NA
ASV_16s_12424	0.253686071	0.951625913	3.550428636	0.912547753	0.996118667	NA
ASV_16s_12425	0.17725324	-1.031083631	3.550642091	0.287236222	NA	NA
ASV_16s_12426	0.273695621	0.126539317	3.550054254	1.677550398	NA	NA
ASV_16s_12427	0.461655326	-0.725562479	3.54807653	0.794417879	NA	NA
ASV_16s_12428	0.297123524	-0.263742181	3.550652014	0.98500866	NA	NA
ASV_16s_12429	0.244855293	-0.068448196	3.550237164	1.390324902	NA	NA
ASV_16s_12430	0.229139176	0.908216605	3.550796058	0.828298012	NA	NA
ASV_16s_12431	0.248819439	-0.067764806	3.550311093	1.351291348	NA	NA
ASV_16s_12432	0.272410387	-0.672013907	3.550361963	1.131956732	NA	NA
ASV_16s_12433	0.268180484	0.368796975	3.551371851	0.767218168	NA	NA
ASV_16s_12434	0.260450021	-0.05654932	3.550510152	1.269601114	NA	NA
ASV_16s_12435	0.183030174	0.622159593	3.551706203	0.459811904	NA	NA
ASV_16s_12436	0.258454523	-0.049558228	3.550612341	1.240066262	NA	NA
ASV_16s_12437	0.290458472	0.716944786	3.55067777	1.183353561	0.991305141	NA
ASV_16s_12438	0.276741245	0.009236622	3.550878539	1.098243351	NA	NA
ASV_16s_12439	0.193894745	0.279461264	3.551556766	0.677686451	NA	NA
ASV_16s_12440	0.195955602	-0.526103061	3.551141327	0.670137457	NA	NA
ASV_16s_12441	0.193012647	-0.078681339	3.550797565	0.919972248	NA	NA
ASV_16s_12442	0.193012647	-0.078681339	3.550797565	0.919972248	NA	NA
ASV_16s_12443	0.252291448	-0.105706642	3.550383409	1.271239541	0.989193081	NA
ASV_16s_12444	0.453967738	0.687697126	3.550071634	2.040018684	0.957592227	NA
ASV_16s_12445	0.337649644	0.116477346	3.550246723	1.704272157	0.974384813	NA
ASV_16s_12446	0.272039481	-0.411743304	3.550524629	0.883579059	NA	NA
ASV_16s_12447	0.338612663	0.433967567	3.550767524	1.271040077	NA	NA
ASV_16s_12448	0.285386477	0.360952156	3.551111308	0.892940409	NA	NA
ASV_16s_12449	0.258234287	0.360354592	3.550987163	1.107781683	NA	NA
ASV_16s_12450	0.258234287	0.360354592	3.550987163	1.107781683	NA	NA
ASV_16s_12451	0.310527932	0.315939467	3.550818531	1.162035822	0.991774907	NA
ASV_16s_12452	0.279981683	0.315939484	3.550843652	1.109693219	0.992859803	NA
ASV_16s_12453	0.278270705	0.315939485	3.550845223	1.106592769	0.992921052	NA
ASV_16s_12454	0.276217531	0.315939487	3.550847133	1.102848209	0.992994582	NA
ASV_16s_12455	0.332679933	0.315939284	3.550803187	1.196891501	0.990998294	NA
ASV_16s_12456	0.274506553	0.315939489	3.550848746	1.099707015	0.99305589	NA
ASV_16s_12457	0.315657723	0.315939457	3.550814798	1.170256954	0.991595678	NA
ASV_16s_12458	0.332465717	0.621545657	3.55077083	1.600316862	0.978632129	NA
ASV_16s_12459	0.450062029	1.185249902	3.550118459	0.978454459	0.995168648	NA
ASV_16s_12460	0.363916406	1.169422604	3.5504912	0.936400077	0.9957903	NA
ASV_16s_12461	0.373865235	0.734613802	3.550863622	1.231573892	0.990181653	NA

ASV_16s_12462	0.35680088	0.734567997	3.550877341	1.207900349	NA	NA
ASV_16s_12463	0.35680088	0.734567997	3.550877341	1.207900349	NA	NA
ASV_16s_12464	0.35680088	0.734567997	3.550877341	1.207900349	NA	NA
ASV_16s_12465	0.269715815	0.69323899	3.550827134	1.02583201	NA	NA
ASV_16s_12466	0.269715815	0.69323899	3.550827134	1.02583201	NA	NA
ASV_16s_12467	0.228458808	0.278950938	3.55137448	0.71618951	NA	NA
ASV_16s_12468	0.228458808	0.278950938	3.55137448	0.71618951	NA	NA
ASV_16s_12469	0.228458808	0.278950938	3.55137448	0.71618951	NA	NA
ASV_16s_12470	0.233119347	-0.08352935	3.550514822	1.248926772	NA	NA
ASV_16s_12471	0.228572889	-0.100340171	3.550525947	1.257790512	NA	NA
ASV_16s_12472	0.228572889	-0.100340171	3.550525947	1.257790512	NA	NA
ASV_16s_12473	0.23467286	-0.162837851	3.550421194	1.313034775	0.988087346	NA
ASV_16s_12474	0.228595706	-0.15226119	3.550471391	1.288265055	0.988750653	NA
ASV_16s_12475	0.234931779	-0.175586164	3.550409742	1.316727078	0.987986465	NA
ASV_16s_12476	0.228607114	-0.177626304	3.550450824	1.294244411	0.98859267	NA
ASV_16s_12477	0.228612818	-0.190230153	3.550442053	1.295867222	0.988549558	NA
ASV_16s_12478	0.232681367	-0.233797355	3.550392156	1.308717263	0.988204648	NA
ASV_16s_12479	0.236744211	-0.263943699	3.550356839	1.315172901	0.988028992	NA
ASV_16s_12480	0.247595108	-0.301630277	3.55028359	1.334542692	0.987492354	NA
ASV_16s_12481	0.234476686	-0.286417792	3.550366142	1.297596196	0.988503517	NA
ASV_16s_12482	0.256441801	-0.323169991	3.55023258	1.347373521	0.987128919	NA
ASV_16s_12483	0.24173146	-0.320399498	3.550316983	1.297220258	0.988513537	NA
ASV_16s_12484	0.233987369	-0.325094489	3.550365564	1.263239075	0.989397209	NA
ASV_16s_12485	0.233233429	-0.337724545	3.550370949	1.247009136	0.989803941	NA
ASV_16s_12486	0.251017655	-0.368392034	3.550266512	1.275019333	0.989095803	NA
ASV_16s_12487	0.24446746	-0.375843709	3.550306502	1.242371498	NA	NA
ASV_16s_12488	0.315615938	-0.597457498	3.550075321	1.020465801	0.994491604	NA
ASV_16s_12489	0.081671273	-0.343378448	3.551593384	0.405644384	NA	NA
ASV_16s_12490	0.303868684	-0.680374429	3.550199866	0.829266601	0.99713368	NA
ASV_16s_12491	0.293607651	-0.680149204	3.550250495	0.818257395	NA	NA
ASV_16s_12492	0.278213348	-0.679369019	3.550332626	0.800922798	NA	NA
ASV_16s_12493	0.232576457	-0.661031992	3.550582478	0.741577936	NA	NA
ASV_16s_12494	0.072890209	-0.169093512	3.550870755	0.362047443	NA	NA
ASV_16s_12495	0.043917445	-0.159256175	3.551071394	0.202113595	NA	NA
ASV_16s_12496	0.10161147	0.220148086	3.550861176	0.465779868	NA	NA
ASV_16s_12499	0.280433524	-0.585502801	3.550344483	1.162406776	0.991766872	NA
ASV_16s_12500	0.323066019	-0.775799526	3.549981218	0.986783603	0.995038927	NA
ASV_16s_12501	0.244294578	-0.340310857	3.550158316	0.841355456	0.996998645	NA
ASV_16s_12502	0.245275008	0.296920466	3.550167131	1.290116375	0.988701883	NA
ASV_16s_12503	0.271869263	-0.313891878	3.550487859	1.138138064	0.992282218	NA
ASV_16s_12504	0.257538408	0.158287352	3.54987577	1.433699027	0.984516664	NA
ASV_16s_12505	0.305078187	-0.742907692	3.550314288	0.833737188	0.997084216	NA
ASV_16s_12506	0.297967778	-0.757006794	3.549965864	1.101777669	0.993015515	NA
ASV_16s_12507	0.299575908	-0.119272093	3.549905685	1.265578161	0.989337777	NA
ASV_16s_12508	0.237778864	-0.243740736	3.550059221	0.987133402	NA	NA
ASV_16s_12509	0.313355979	-0.745984811	3.549852491	1.217808621	0.990511061	NA
ASV_16s_12510	0.307432354	-0.738851546	3.549882888	1.20814692	0.990738105	NA
ASV_16s_12511	0.291529011	-0.719452623	3.549964986	1.177801872	0.991429055	NA
ASV_16s_12512	0.30491348	-0.729150085	3.549899848	1.219359706	0.990474292	NA
ASV_16s_12513	0.298756753	-0.719700291	3.549931063	1.209054407	0.990716926	NA
ASV_16s_12514	0.246950676	0.212089038	3.55014449	1.386639438	NA	NA
ASV_16s_12515	0.289961573	-0.708086627	3.549976605	1.196304335	0.991011741	NA
ASV_16s_12516	0.256868338	0.075460654	3.550905208	0.980316999	0.995139832	NA
ASV_16s_12517	0.268613667	0.859338654	3.549745994	0.925937939	0.995936455	NA
ASV_16s_12518	0.301235262	-0.697412771	3.549945466	1.254221643	0.989624407	NA
ASV_16s_12519	0.287967467	-0.668404973	3.550509522	0.89588848	NA	NA
ASV_16s_12520	0.238225921	0.294385179	3.551522463	0.561997491	NA	NA
ASV_16s_12521	0.309559933	-0.710633273	3.549898931	1.27222422	0.98916779	NA
ASV_16s_12522	0.294046136	-0.682904525	3.549993526	1.247538247	0.989790837	NA
ASV_16s_12523	0.304671533	-0.306547665	3.550109954	1.12255546	0.992602137	NA
ASV_16s_12524	0.302984814	-0.696136004	3.549942029	1.263903472	0.989380349	NA
ASV_16s_12525	0.290016159	-0.670192429	3.550022193	1.243661134	0.989886621	NA
ASV_16s_12526	0.246069148	0.502239519	3.551135775	0.968518747	NA	NA
ASV_16s_12527	0.312061219	-0.691956862	3.549923483	1.302104762	0.988382921	NA
ASV_16s_12528	0.250044631	0.529098678	3.550273975	0.878522246	NA	NA
ASV_16s_12529	0.29797394	-0.508312312	3.549930825	0.879644805	0.996543675	NA
ASV_16s_12530	0.279270798	-0.474912515	3.550395568	1.261978125	0.989429162	NA

ASV_16s_12531	0.292339112	-1.24269841	3.549917228	0.515767608	NA	NA
ASV_16s_12532	0.320697009	-0.244578247	3.550145435	1.203456421	NA	NA
ASV_16s_12533	0.234218153	0.324266772	3.55013293	1.112446509	NA	NA
ASV_16s_12534	0.265194186	1.269802805	3.550643669	0.748543111	NA	NA
ASV_16s_12535	0.278358901	-0.071288297	3.551121375	0.877677636	NA	NA
ASV_16s_12536	0.284503333	-0.05934364	3.551171336	0.834675481	NA	NA
ASV_16s_12537	0.318595799	2.08192749	3.549747923	0.267619628	0.999932082	NA
ASV_16s_12538	0.291902891	0.085165394	3.550691704	1.778243366	0.97108921	NA
ASV_16s_12539	0.258793415	0.161088267	3.55013189	1.047363079	0.994027896	NA
ASV_16s_12540	0.266046557	-0.07175751	3.550419377	1.250090827	0.989727469	NA
ASV_16s_12541	0.271010915	0.986201724	3.551472373	0.837111174	NA	NA
ASV_16s_12542	0.260728587	1.715164419	3.550468468	0.402287746	NA	NA
ASV_16s_12543	0.336386049	0.205691234	3.551124442	0.572432649	NA	NA
ASV_16s_12544	0.284997006	1.299500752	3.550642378	0.762252532	NA	NA
ASV_16s_12545	0.284997006	1.299500752	3.550642378	0.762252532	NA	NA
ASV_16s_12546	0.247905169	0.386423388	3.551225856	0.743758514	NA	NA
ASV_16s_12547	0.192111699	0.370120894	3.55156169	0.657876099	NA	NA
ASV_16s_12548	0.262206568	0.353389603	3.551891153	0.433252128	NA	NA
ASV_16s_12549	0.304971721	-0.713880126	3.55016587	0.943806163	0.995684811	NA
ASV_16s_12550	0.191864108	-0.54016584	3.550710416	0.658978498	NA	NA
ASV_16s_12551	0.264983958	-0.072461559	3.550470926	1.142815996	NA	NA
ASV_16s_12552	0.236670148	0.408050042	3.550929674	0.661059297	NA	NA
ASV_16s_12553	0.256395425	0.776703897	3.550314975	1.223763557	NA	NA
ASV_16s_12554	0.082406528	0.343919488	3.551750158	0.335515209	NA	NA
ASV_16s_12555	0.111749329	-0.04850358	3.55141847	0.480960811	NA	NA
ASV_16s_12556	0.308376658	0.882745209	3.550054121	1.398258149	0.985624556	NA
ASV_16s_12557	0.132971158	-0.482503791	3.550937991	0.475193639	NA	NA
ASV_16s_12558	0.132971158	-0.482503791	3.550937991	0.475193639	NA	NA
ASV_16s_12559	0.132971158	-0.482503791	3.550937991	0.475193639	NA	NA
ASV_16s_12560	0.132971158	-0.482503791	3.550937991	0.475193639	NA	NA
ASV_16s_12561	0.172218486	0.130152045	3.550341294	0.872533753	NA	NA
ASV_16s_12562	0.276062825	-0.547776237	3.549888406	0.70570568	NA	NA
ASV_16s_12563	0.161490422	-0.520913331	3.550662691	0.535913573	NA	NA
ASV_16s_12564	0.161490422	-0.520913331	3.550662691	0.535913573	NA	NA
ASV_16s_12565	0.161490422	-0.520913331	3.550662691	0.535913573	NA	NA
ASV_16s_12566	0.148002497	-0.504757204	3.550785109	0.508406048	NA	NA
ASV_16s_12567	0.117327047	1.075752023	3.551199766	0.403173024	NA	NA
ASV_16s_12568	0.263728111	-0.07893598	3.550721847	1.075293091	0.993520827	NA
ASV_16s_12569	0.156922525	0.220072016	3.550761098	0.659241366	0.998629683	NA
ASV_16s_12570	0.168101591	0.22006271	3.550748846	0.692123188	0.998395529	NA
ASV_16s_12571	0.250337141	0.310937928	3.550849431	1.030304451	NA	NA
ASV_16s_12572	0.209964093	0.604907941	3.551890291	0.507553135	NA	NA
ASV_16s_12573	0.236492235	1.483845356	3.550686031	0.533040081	0.999316284	NA
ASV_16s_12574	0.239829348	-0.068499563	3.550265037	1.374813593	NA	NA
ASV_16s_12575	0.212857671	1.378276842	3.550983527	0.566786965	NA	NA
ASV_16s_12576	0.239624024	-0.096807396	3.550285573	1.068299044	NA	NA
ASV_16s_12577	0.254056238	-0.067648898	3.550323061	1.335674612	NA	NA
ASV_16s_12578	0.324026312	1.382639519	3.549988441	0.964702258	NA	NA
ASV_16s_12579	0.253924097	-0.049558267	3.55062127	1.22931279	NA	NA
ASV_16s_12580	0.266886011	-0.094920482	3.550551775	1.078436262	0.993462112	NA
ASV_16s_12581	0.420929343	0.737441675	3.550349192	1.530485468	0.981235447	NA
ASV_16s_12582	0.332775737	1.334690064	3.550321297	0.752769863	NA	NA
ASV_16s_12583	0.296458713	1.247688195	3.550026786	1.190011785	NA	NA
ASV_16s_12584	0.284617937	0.859393639	3.550683494	1.113623945	0.992781671	NA
ASV_16s_12585	0.328939844	0.469128628	3.550800021	1.159280808	NA	NA
ASV_16s_12586	0.18962646	-0.078664501	3.550802608	0.911281387	NA	NA
ASV_16s_12587	0.304374098	0.358445454	3.550294594	1.391795685	0.98582123	NA
ASV_16s_12588	0.250185535	-0.029010961	3.550843952	1.107967557	NA	NA
ASV_16s_12589	0.250185535	-0.029010961	3.550843952	1.107967557	NA	NA
ASV_16s_12590	0.328598793	0.343400619	3.550009041	1.538292285	NA	NA
ASV_16s_12591	0.234100281	0.278256047	3.550550711	1.209879518	NA	NA
ASV_16s_12592	0.307508696	0.35489854	3.550247411	1.417861632	NA	NA
ASV_16s_12593	0.240872586	0.287728346	3.550260097	1.161208714	NA	NA
ASV_16s_12594	0.285392263	0.360366023	3.55094991	1.158308507	NA	NA
ASV_16s_12595	0.253703861	0.360352458	3.55099416	1.098816735	NA	NA
ASV_16s_12596	0.253703861	0.360352458	3.55099416	1.098816735	NA	NA
ASV_16s_12597	0.287898039	0.315939495	3.550836644	1.123718032	0.992578563	NA

ASV_16s_12598	0.270801284	0.315939511	3.550852317	1.092804448	0.993189415	NA
ASV_16s_12599	0.324644722	0.315939469	3.550808523	1.184461093	0.991280287	NA
ASV_16s_12600	0.264983958	0.315939611	3.550858113	1.081831665	NA	NA
ASV_16s_12601	0.264983958	0.315939611	3.550858113	1.081831665	NA	NA
ASV_16s_12602	0.350541215	0.734549518	3.550882714	1.198896838	NA	NA
ASV_16s_12603	0.350541215	0.734549518	3.550882714	1.198896838	NA	NA
ASV_16s_12604	0.350541215	0.734549518	3.550882714	1.198896838	NA	NA
ASV_16s_12605	0.173642576	0.73348228	3.551192683	0.849939547	NA	NA
ASV_16s_12606	0.294349255	0.69331575	3.550804232	1.069793465	0.993622754	NA
ASV_16s_12607	0.329590751	0.693407939	3.550777383	1.126985054	0.992512063	NA
ASV_16s_12608	0.326392566	0.693400569	3.550779586	1.122018179	0.992613016	NA
ASV_16s_12609	0.321356698	0.693388186	3.550783132	1.114166222	0.99277085	NA
ASV_16s_12610	0.298807602	0.693328597	3.550800489	1.077376179	0.993481952	NA
ASV_16s_12611	0.295049419	0.69331779	3.550803638	1.070986586	0.993600729	NA
ASV_16s_12612	0.264983958	0.693222417	3.550832027	1.016906947	NA	NA
ASV_16s_12613	0.264983958	0.693222417	3.550832027	1.016906947	NA	NA
ASV_16s_12614	0.307154282	0.693351633	3.550793777	1.091252073	0.993219218	NA
ASV_16s_12615	0.224379187	0.279695353	3.551401741	0.710145666	NA	NA
ASV_16s_12616	0.258665345	-0.120587363	3.550313794	1.345877145	NA	NA
ASV_16s_12617	0.238977394	-0.133312514	3.550419386	1.299503911	0.988452583	NA
ASV_16s_12618	0.241959012	-0.174728816	3.550365772	1.332773428	0.98754197	NA
ASV_16s_12619	0.22832001	-0.177125506	3.550453274	1.293002528	0.988625594	NA
ASV_16s_12620	0.247902298	-0.218760321	3.550304518	1.358311309	0.98681407	NA
ASV_16s_12621	0.231369995	-0.207004623	3.550413369	1.306464671	0.988265565	NA
ASV_16s_12622	0.2245389	-0.20916853	3.550459233	1.28143286	0.988929511	NA
ASV_16s_12623	0.224544604	-0.222013242	3.550452729	1.280626435	0.988950506	NA
ASV_16s_12624	0.231887833	-0.232666829	3.550397919	1.305960837	0.988279164	NA
ASV_16s_12625	0.230121034	-0.242731879	3.550405997	1.297581261	0.988503915	NA
ASV_16s_12626	0.230886382	-0.256364436	3.550396772	1.296717305	0.988526935	NA
ASV_16s_12627	0.224584533	-0.314416517	3.550429403	1.237185037	NA	NA
ASV_16s_12628	0.239734377	-0.3444278	3.550330189	1.263725606	0.989384864	NA
ASV_16s_12629	0.242804217	-0.360786147	3.55031353	1.255441785	0.989593843	NA
ASV_16s_12630	0.240778499	-0.358845188	3.55032562	1.250492812	0.989717468	NA
ASV_16s_12631	0.229660237	-0.347695105	3.550395793	1.222384209	0.990402341	NA
ASV_16s_12632	0.227387008	-0.359453862	3.550413211	1.200480662	0.990915826	NA
ASV_16s_12633	0.244340618	-0.403504047	3.550315921	1.203720666	0.990840978	NA
ASV_16s_12634	0.231230862	-0.489312183	3.5504499	1.03024747	0.994325734	NA
ASV_16s_12635	0.080212858	-0.33713582	3.551604481	0.400686692	NA	NA
ASV_16s_12636	0.080212858	-0.33713582	3.551604481	0.400686692	NA	NA
ASV_16s_12637	0.290011386	0.503213907	3.550740503	1.05122457	NA	NA
ASV_16s_12638	0.337188544	-0.681012327	3.550056559	0.862618356	0.996751166	NA
ASV_16s_12639	0.272604098	-0.678666774	3.550363966	0.794311835	0.99750157	NA
ASV_16s_12640	0.300681852	-0.680305649	3.55021531	0.82585878	0.997171013	NA
ASV_16s_12641	0.280623432	-0.679663711	3.550319518	0.803730488	NA	NA
ASV_16s_12642	0.280623432	-0.679663711	3.550319518	0.803730488	NA	NA
ASV_16s_12643	0.238619723	-0.664734983	3.550548909	0.75012278	NA	NA
ASV_16s_12644	0.071588598	-0.169067323	3.550877351	0.355512619	NA	NA
ASV_16s_12645	0.071588598	-0.169067323	3.550877351	0.355512619	NA	NA
ASV_16s_12646	0.071588598	-0.169067323	3.550877351	0.355512619	NA	NA
ASV_16s_12647	0.071801074	-0.141739694	3.550870329	0.407517526	NA	NA
ASV_16s_12648	0.024311443	-0.127259151	3.551363569	0.073888617	NA	NA
ASV_16s_12649	0.075483999	0.220222323	3.550959117	0.350708079	NA	NA
ASV_16s_12651	0.240121785	-0.326250293	3.55059893	0.734863979	NA	NA
ASV_16s_12652	0.294727007	-0.764077309	3.550127994	0.95089739	0.995582221	NA
ASV_16s_12653	0.29314415	-0.736900844	3.550244995	1.08972579	0.99324844	NA
ASV_16s_12654	0.254802208	0.189843439	3.549924427	1.372972501	0.986384745	NA
ASV_16s_12655	0.230562135	0.216663357	3.550182782	1.29583634	0.98855038	NA
ASV_16s_12656	0.230562135	0.216663357	3.550182782	1.29583634	0.98855038	NA
ASV_16s_12657	0.238161138	0.145324996	3.550128858	1.351018946	0.987024499	NA
ASV_16s_12658	0.260482176	-0.788304791	3.5503156	0.995422314	0.994902043	NA
ASV_16s_12659	0.310327401	-0.772270087	3.549947467	1.049826739	0.993984222	NA
ASV_16s_12660	0.295460478	-0.76046092	3.550005047	1.057672688	0.993843782	NA
ASV_16s_12661	0.279125814	-0.74554992	3.550093381	1.039869721	NA	NA
ASV_16s_12662	0.308900535	-0.760697594	3.549889808	1.14082204	0.992226251	NA
ASV_16s_12663	0.234193573	-0.229137494	3.550097049	0.992502048	NA	NA
ASV_16s_12664	0.23511949	0.107351651	3.550113155	1.306357417	0.988268461	NA
ASV_16s_12665	0.31011043	-0.73602752	3.54987228	1.227828176	0.990271982	NA

ASV_16s_12666	0.294622682	-0.71188174	3.549953699	1.203481809	0.990846509	NA
ASV_16s_12667	0.302822676	-0.725501952	3.549910614	1.216358061	NA	NA
ASV_16s_12668	0.337247649	-0.677204286	3.550433576	0.864867026	0.996724242	NA
ASV_16s_12669	0.300982916	-0.698779581	3.549944301	1.252136234	NA	NA
ASV_16s_12670	0.230981235	-0.469334212	3.550461117	1.012812794	0.994619192	NA
ASV_16s_12671	0.297844471	-0.673127314	3.549995723	1.269557023	0.989236209	NA
ASV_16s_12672	0.284839271	-0.656450018	3.550054753	1.236306662	0.990066779	NA
ASV_16s_12673	0.30855549	-0.675511205	3.549964598	1.30072632	0.988419874	NA
ASV_16s_12674	0.33112611	-0.701755696	3.549856822	1.355242584	NA	NA
ASV_16s_12675	0.329656391	0.852867705	3.550067612	1.46040799	NA	NA
ASV_16s_12676	0.285119716	-0.601148482	3.550273618	1.060935059	0.993784779	NA
ASV_16s_12677	0.250787137	0.710177594	3.551874684	0.596918923	0.99900858	NA
ASV_16s_12678	0.289587009	0.206504895	3.549495707	1.349016775	NA	NA
ASV_16s_12679	0.266299267	-0.065699612	3.551543236	0.533007213	0.999316422	NA
ASV_16s_12680	0.253959046	-0.3581335	3.550691901	0.781273132	NA	NA
ASV_16s_12681	0.264786621	-0.303730509	3.551089581	0.846211978	NA	NA
ASV_16s_12682	0.281238017	0.679191798	3.550879858	1.116573339	0.992722693	NA
ASV_16s_12683	0.275066809	0.350860642	3.551333767	0.749688127	NA	NA
ASV_16s_12684	0.133092383	1.027248726	3.551407253	0.427540743	NA	NA
ASV_16s_12685	0.271597302	-0.092238642	3.550828055	0.906862084	NA	NA
ASV_16s_12686	0.291447543	-0.092708619	3.550766798	0.930163137	0.995877831	NA
ASV_16s_12687	0.394266981	0.886910763	3.550057976	0.905798696	0.996208462	NA
ASV_16s_12688	0.296350236	-0.088923365	3.540671485	1.214137811	0.990597726	NA
ASV_16s_12689	0.259376899	0.20918309	3.551264105	0.355869926	NA	NA
ASV_16s_12690	0.279907774	1.296233774	3.550663093	0.756020101	NA	NA
ASV_16s_12691	0.279907774	1.296233774	3.550663093	0.756020101	NA	NA
ASV_16s_12692	0.256815025	1.27832631	3.550757473	0.726016547	NA	NA
ASV_16s_12693	0.256815025	1.27832631	3.550757473	0.726016547	NA	NA
ASV_16s_12694	0.243478291	0.385498449	3.55125159	0.737672854	NA	NA
ASV_16s_12695	0.289426582	1.187445646	3.550648723	0.84851625	0.996916729	NA
ASV_16s_12696	0.319075955	0.367298608	3.55162645	0.480998302	NA	NA
ASV_16s_12697	0.188681133	0.368553618	3.551583507	0.651818867	NA	NA
ASV_16s_12698	0.188681133	0.368553618	3.551583507	0.651818867	NA	NA
ASV_16s_12699	0.278176604	1.346891914	3.550454919	0.78997787	NA	NA
ASV_16s_12700	0.125364232	0.345787035	3.551986451	0.284600532	NA	NA
ASV_16s_12701	0.346806695	0.28705467	3.551051127	0.555858694	NA	NA
ASV_16s_12702	0.17633076	-0.912780829	3.550776149	0.350865781	NA	NA
ASV_16s_12703	0.271261782	-0.563398091	3.550171155	0.770609126	NA	NA
ASV_16s_12704	0.344528125	1.335738942	3.550275798	0.763998139	NA	NA
ASV_16s_12705	0.257769805	0.727071356	3.550016594	0.715908912	NA	NA
ASV_16s_12706	0.214835038	0.399510855	3.551044719	0.628625434	NA	NA
ASV_16s_12707	0.232443895	0.406455312	3.550950473	0.654981436	NA	NA
ASV_16s_12708	0.080934983	0.343640541	3.551755578	0.33088701	NA	NA
ASV_16s_12709	0.130596673	-0.478204088	3.55096279	0.469595964	NA	NA
ASV_16s_12710	0.158606665	-0.517752899	3.550687821	0.530179871	NA	NA
ASV_16s_12711	0.145359596	-0.501362807	3.550811352	0.502784979	NA	NA
ASV_16s_12712	0.286688406	1.316008644	3.550474384	0.702642904	NA	NA
ASV_16s_12713	0.115231921	1.069816887	3.55121372	0.397777461	NA	NA
ASV_16s_12714	0.227612888	-0.078825001	3.550754671	1.001424411	0.994805526	NA
ASV_16s_12715	0.145441741	0.220083086	3.550775637	0.623480956	NA	NA
ASV_16s_12716	0.145441741	0.220083086	3.550775637	0.623480956	NA	NA
ASV_16s_12717	0.159035011	0.220070054	3.550758655	0.665559328	0.998586595	NA
ASV_16s_12718	0.159627951	0.22006953	3.55075798	0.667331001	0.998574351	NA
ASV_16s_12719	0.161257719	0.220068274	3.55075615	0.672173068	0.998540529	NA
ASV_16s_12720	0.162252102	0.220067287	3.55075505	0.675110546	0.998519752	NA
ASV_16s_12721	0.271871801	0.310936523	3.550828789	1.071923567	0.993583398	NA
ASV_16s_12722	0.218394101	0.43378666	3.550672002	0.697452672	0.998355233	NA
ASV_16s_12723	0.314003006	1.5529096	3.550401743	0.74731379	NA	NA
ASV_16s_12724	0.233316849	-0.068677576	3.550353475	1.346013322	NA	NA
ASV_16s_12725	0.234803404	-0.06854752	3.550301077	1.357298586	NA	NA
ASV_16s_12726	0.229012137	-0.495261842	3.550381019	1.001077203	0.994811141	NA
ASV_16s_12727	0.210318409	1.364944624	3.551006625	0.566645725	NA	NA
ASV_16s_12728	0.208322783	1.333746593	3.551048885	0.57766322	NA	NA
ASV_16s_12729	0.209727893	0.609746111	3.551034467	0.720656582	NA	NA
ASV_16s_12730	0.237896678	0.78408401	3.551243406	1.090361693	NA	NA
ASV_16s_12731	0.198246882	1.304040119	3.551076793	0.550780702	NA	NA
ASV_16s_12732	0.266306595	-0.019957622	3.550139339	1.032348995	NA	NA

ASV_16s_12733	0.251993459	-0.067407414	3.550385089	1.303922228	NA	NA
ASV_16s_12734	0.195692992	0.279473954	3.551552447	0.680955037	NA	NA
ASV_16s_12735	0.195692992	0.279473954	3.551552447	0.680955037	NA	NA
ASV_16s_12736	0.38044701	0.445685526	3.549556189	1.171736836	0.991563158	NA
ASV_16s_12737	0.316863998	0.026119403	3.550910839	0.728429396	0.998107635	NA
ASV_16s_12738	0.337429204	0.325832079	3.550893975	0.832363807	0.997099471	NA
ASV_16s_12739	0.309307661	1.344105472	3.549954229	1.106443526	0.992923992	NA
ASV_16s_12740	0.347834524	0.59416146	3.550808562	0.976147021	0.995204195	NA
ASV_16s_12741	0.462212073	0.454821575	3.549685924	2.015365902	NA	NA
ASV_16s_12742	0.332734684	0.372137961	3.550382876	1.381119606	0.986142546	NA
ASV_16s_12743	0.309609127	0.527387439	3.550223531	1.362137405	0.986702838	NA
ASV_16s_12744	0.360603537	0.522028816	3.550811456	1.873730282	0.966497233	NA
ASV_16s_12745	0.245717936	-0.028993333	3.550849836	1.098888294	NA	NA
ASV_16s_12746	0.245717936	-0.028993333	3.550849836	1.098888294	NA	NA
ASV_16s_12747	0.287012218	0.384178679	3.550529671	1.28119078	0.988935816	NA
ASV_16s_12748	0.205058695	-0.073659569	3.550807315	0.973871158	NA	NA
ASV_16s_12749	0.201374133	-0.073640627	3.550812667	0.964928726	NA	NA
ASV_16s_12750	0.201374133	-0.073640627	3.550812667	0.964928726	NA	NA
ASV_16s_12751	0.284520267	0.343589008	3.550149456	1.435097477	NA	NA
ASV_16s_12752	0.325731672	0.788339352	3.549628732	1.157270907	0.99187768	NA
ASV_16s_12753	0.3053068	0.36188586	3.550343622	1.377005536	NA	NA
ASV_16s_12754	0.352808824	0.132245887	3.549873735	2.253517694	NA	NA
ASV_16s_12755	0.280295972	0.360364052	3.550956365	1.149155907	NA	NA
ASV_16s_12756	0.249173435	0.360350246	3.551001411	1.089697733	NA	NA
ASV_16s_12757	0.249173435	0.360350246	3.551001411	1.089697733	NA	NA
ASV_16s_12758	0.284992083	0.745749387	3.550624654	1.326152675	0.987726569	NA
ASV_16s_12759	0.295660588	0.315939505	3.550830131	1.137125234	0.992303271	NA
ASV_16s_12760	0.294048532	0.315939335	3.550831452	1.134376525	0.992360226	NA
ASV_16s_12761	0.269833579	0.31593953	3.55085327	1.090961025	0.993224797	NA
ASV_16s_12762	0.269833579	0.31593953	3.55085327	1.090961025	0.993224797	NA
ASV_16s_12763	0.269833579	0.31593953	3.55085327	1.090961025	0.993224797	NA
ASV_16s_12764	0.295822567	0.502458443	3.550802966	1.838524876	0.968234612	NA
ASV_16s_12765	0.260252102	0.315939624	3.550863024	1.072695703	NA	NA
ASV_16s_12766	0.373727304	1.166407285	3.55061804	0.957173857	0.995490116	NA
ASV_16s_12767	0.365896401	0.734592831	3.550869886	1.220581932	0.990445256	NA
ASV_16s_12768	0.344281551	0.734530367	3.550888283	1.189731836	NA	NA
ASV_16s_12769	0.344281551	0.734530367	3.550888283	1.189731836	NA	NA
ASV_16s_12770	0.344281551	0.734530367	3.550888283	1.189731836	NA	NA
ASV_16s_12771	0.170541816	0.733443277	3.551203805	0.841399993	NA	NA
ASV_16s_12772	0.314119386	0.693370319	3.550788438	1.102607029	0.992999302	NA
ASV_16s_12773	0.324416078	0.693395925	3.550780978	1.118876061	0.992676435	NA
ASV_16s_12774	0.382324093	0.693515851	3.550746431	1.202157451	NA	NA
ASV_16s_12775	0.382324093	0.693515851	3.550746431	1.202157451	NA	NA
ASV_16s_12776	0.371755935	0.271647252	3.550511677	0.874361581	NA	NA
ASV_16s_12777	0.220368014	0.007069859	3.550762009	1.101139343	NA	NA
ASV_16s_12778	0.224183348	-0.048902557	3.550630629	1.178094865	NA	NA
ASV_16s_12779	0.235351096	-0.08818214	3.55049378	1.244262547	NA	NA
ASV_16s_12780	0.23297178	-0.184689126	3.55041651	1.30624188	0.988271579	NA
ASV_16s_12781	0.227290374	-0.213367826	3.550437852	1.291285944	0.988671006	NA
ASV_16s_12782	0.24287994	-0.259402385	3.550320283	1.333799255	0.987513217	NA
ASV_16s_12783	0.243307801	-0.259941907	3.550317547	1.336943099	0.987424846	NA
ASV_16s_12784	0.224268909	-0.247362984	3.550444832	1.275066716	0.98909458	NA
ASV_16s_12785	0.23323366	-0.259413517	3.550380651	1.3029995	0.988358896	NA
ASV_16s_12786	0.225793901	-0.262371677	3.550430015	1.276213501	0.989064958	NA
ASV_16s_12787	0.220482095	-0.268399135	3.550466514	1.254579684	0.989615444	NA
ASV_16s_12788	0.220487799	-0.281956848	3.550463102	1.247920097	0.989781373	NA
ASV_16s_12789	0.226058524	-0.289097932	3.550422081	1.264885437	0.989355399	NA
ASV_16s_12790	0.22253063	-0.32589712	3.550443939	1.217905922	0.990508757	NA
ASV_16s_12791	0.226328851	-0.330194655	3.550417262	1.228380555	0.990258694	NA
ASV_16s_12792	0.078754442	-0.330795603	3.551615873	0.39567627	NA	NA
ASV_16s_12793	0.078754442	-0.330795603	3.551615873	0.39567627	NA	NA
ASV_16s_12794	0.295869125	-0.680199271	3.550239212	0.820660784	0.997227342	NA
ASV_16s_12795	0.275521188	-0.679032436	3.550347568	0.797749177	NA	NA
ASV_16s_12796	0.234281182	-0.662072819	3.550572976	0.743960091	NA	NA
ASV_16s_12797	0.234281182	-0.662072819	3.550572976	0.743960091	NA	NA
ASV_16s_12798	0.226684739	-0.656629518	3.550614016	0.732912432	0.998069875	NA
ASV_16s_12799	0.164002393	1.240870161	3.551323441	0.44391155	0.999627046	NA

ASV_16s_12800	0.08274272	-0.005004415	3.550825148	0.642291885	0.998740921	NA
ASV_16s_12801	0.062345073	-0.168849312	3.550931979	0.307525953	NA	NA
ASV_16s_12802	0.142797345	0.220085896	3.550779321	0.614919571	NA	NA
ASV_16s_12803	0.097982489	0.220155995	3.550871686	0.450886505	NA	NA
ASV_16s_12804	0.156099794	0.596007851	3.550725912	0.591680649	NA	NA
ASV_16s_12805	0.253425824	-0.358573136	3.550320163	1.271222499	0.989193518	NA
ASV_16s_12806	0.225140566	0.120500083	3.550496115	1.197000708	0.990995792	NA
ASV_16s_12807	0.235543856	0.042773395	3.550348095	1.280709778	0.988948337	NA
ASV_16s_12808	0.278858366	-0.699255904	3.550381438	0.820647807	NA	NA
ASV_16s_12809	0.304814833	-0.757851412	3.550134235	0.907843979	0.996181394	NA
ASV_16s_12810	0.22555494	0.379640035	3.550167095	1.114142675	0.99277132	NA
ASV_16s_12811	0.302826607	-0.768610923	3.550011519	1.01449349	0.994591336	NA
ASV_16s_12812	0.284114163	-0.756833814	3.550129417	0.978658113	0.995165503	NA
ASV_16s_12813	0.251639868	0.316886987	3.551865382	0.447206343	NA	NA
ASV_16s_12814	0.199094361	-0.08400883	3.5504932	0.664197202	NA	NA
ASV_16s_12815	0.272545541	-0.439120265	3.550265692	1.1523428	0.991983121	NA
ASV_16s_12816	0.248475323	-0.078096716	3.549937963	1.238417088	0.990015286	NA
ASV_16s_12817	0.29677351	-0.743729221	3.549944774	1.141626005	0.992209437	NA
ASV_16s_12818	0.229522652	0.129118812	3.550120639	1.292388263	0.988641857	NA
ASV_16s_12819	0.286804713	-0.724566534	3.549992176	1.141616322	0.99220964	NA
ASV_16s_12820	0.288386718	-0.670901912	3.550000366	1.185903559	0.991247851	NA
ASV_16s_12821	0.27006998	-0.691532117	3.550083278	1.131185105	NA	NA
ASV_16s_12822	0.286316255	-0.710709079	3.549992874	1.173951621	0.991514341	NA
ASV_16s_12823	0.272916426	-0.700550605	3.550065787	1.138483129	0.992275037	NA
ASV_16s_12824	0.296469405	-0.707933818	3.54994944	1.21888866	NA	NA
ASV_16s_12825	0.293418353	-0.694803519	3.549976542	1.225923312	NA	NA
ASV_16s_12826	0.209485991	-0.995513293	3.550700618	0.549262728	0.999245364	NA
ASV_16s_12827	0.24570525	0.288536652	3.550212741	0.870831679	NA	NA
ASV_16s_12828	0.230164415	-0.83536021	3.550593363	0.619350147	NA	NA
ASV_16s_12829	0.24127585	-0.19133969	3.550791515	0.969402475	NA	NA
ASV_16s_12830	0.278519832	-0.614885742	3.550135163	1.236899161	NA	NA
ASV_16s_12831	0.288262544	-0.631965593	3.550081803	1.257386135	NA	NA
ASV_16s_12832	0.290685678	-0.631969513	3.550075906	1.265168578	NA	NA
ASV_16s_12833	0.280665714	-0.613588589	3.550131936	1.242103249	0.989924951	NA
ASV_16s_12834	0.286429755	-0.603558336	3.55013638	1.255176718	0.989600488	NA
ASV_16s_12835	0.282615157	0.275923717	3.550522482	1.327118409	0.987699747	NA
ASV_16s_12836	0.283552056	-0.554909684	3.550240975	1.246887439	0.989806954	NA
ASV_16s_12837	0.279234925	0.984661575	3.551173323	1.077658582	0.99347667	NA
ASV_16s_12838	0.271219064	-0.427155706	3.550268219	1.526270056	0.98138623	NA
ASV_16s_12839	0.279408319	-0.482877258	3.550370311	1.229356208	NA	NA
ASV_16s_12840	0.298014827	-0.487450608	3.550319534	0.896024403	NA	NA
ASV_16s_12841	0.281684691	-0.452027933	3.550474372	0.922812038	0.995979477	NA
ASV_16s_12842	0.248345662	-0.09180027	3.550737172	1.091818007	NA	NA
ASV_16s_12843	0.240192005	-0.146888493	3.550866638	1.107784106	NA	NA
ASV_16s_12844	0.241036573	0.35474037	3.551199334	0.821987761	NA	NA
ASV_16s_12845	0.222250056	0.260971682	3.551586879	0.659819467	0.998625778	NA
ASV_16s_12846	0.295559086	1.836429018	3.550162482	0.454783095	0.999595784	NA
ASV_16s_12847	0.25728438	1.715121023	3.550354168	0.432883581	0.999657025	NA
ASV_16s_12848	0.260829579	1.281788618	3.550741255	0.731414029	NA	NA
ASV_16s_12849	0.252145661	1.274203382	3.550777025	0.719572994	NA	NA
ASV_16s_12850	0.252895569	0.85982619	3.550085988	0.90911928	NA	NA
ASV_16s_12851	0.253520492	0.77872444	3.550093673	1.084046506	NA	NA
ASV_16s_12852	0.302474649	0.626442675	3.550482395	1.281933013	NA	NA
ASV_16s_12853	0.239051413	0.384670206	3.551277261	0.731468554	NA	NA
ASV_16s_12854	0.239051413	0.384670206	3.551277261	0.731468554	NA	NA
ASV_16s_12855	0.289400892	0.395725188	3.55098456	0.795217835	NA	NA
ASV_16s_12856	0.354813911	-0.153392623	3.54976754	1.667320608	0.975945804	NA
ASV_16s_12857	0.185250567	0.366833725	3.551604721	0.645639426	NA	NA
ASV_16s_12858	0.185250567	0.366833725	3.551604721	0.645639426	NA	NA
ASV_16s_12859	0.244755782	1.263193861	3.550636009	0.787160689	NA	NA
ASV_16s_12860	0.2508334	0.387236786	3.551207779	0.747624485	NA	NA
ASV_16s_12861	0.292066761	-0.074125723	3.550789929	1.226359086	NA	NA
ASV_16s_12862	0.185011819	-0.535627369	3.55077069	0.647202866	NA	NA
ASV_16s_12863	0.307857005	0.297206121	3.550235453	0.968822984	0.995315909	NA
ASV_16s_12864	0.210928946	0.397890005	3.551067605	0.622491899	NA	NA
ASV_16s_12865	0.263507646	0.080433194	3.550047326	1.205726235	NA	NA
ASV_16s_12866	0.155722907	-0.514292304	3.550713244	0.524325133	NA	NA

ASV_16s_12867	0.155722907	-0.514292304	3.550713244	0.524325133	NA	NA
ASV_16s_12868	0.155722907	-0.514292304	3.550713244	0.524325133	NA	NA
ASV_16s_12869	0.142716694	-0.497908294	3.550838432	0.497085746	NA	NA
ASV_16s_12870	0.142716694	-0.497908294	3.550838432	0.497085746	NA	NA
ASV_16s_12871	0.260816181	0.012453292	3.550728707	1.566291251	NA	NA
ASV_16s_12872	0.21788502	-0.078789105	3.550765369	0.979675942	NA	NA
ASV_16s_12873	0.21788502	-0.078789105	3.550765369	0.979675942	NA	NA
ASV_16s_12874	0.15667209	0.220072161	3.550761401	0.658424089	0.998635192	NA
ASV_16s_12875	0.244441137	1.266419673	3.550939162	0.621348609	0.998869804	NA
ASV_16s_12876	0.238647415	0.743978777	3.550913258	0.859366328	0.996789848	NA
ASV_16s_12877	0.226804349	-0.068946172	3.550517221	1.283947318	NA	NA
ASV_16s_12878	0.227795386	-0.06877176	3.550447331	1.305930669	NA	NA
ASV_16s_12879	0.25158242	1.648054849	3.550536366	0.43825159	NA	NA
ASV_16s_12880	0.229777459	-0.068592811	3.550346883	1.337772497	NA	NA
ASV_16s_12881	0.231759533	-0.06838272	3.550301879	1.34290121	NA	NA
ASV_16s_12882	0.282302265	-0.825610779	3.550254916	0.823420412	0.997197529	NA
ASV_16s_12883	0.232750569	-0.068248929	3.550299211	1.340854	NA	NA
ASV_16s_12884	0.281013372	0.299841985	3.550047769	1.208794427	0.990722996	NA
ASV_16s_12885	0.236219198	-0.067488194	3.550407397	1.289243106	NA	NA
ASV_16s_12886	0.214815169	0.073023661	3.551117106	0.938572782	NA	NA
ASV_16s_12887	0.244863244	-0.049562574	3.550640338	1.207194688	NA	NA
ASV_16s_12888	0.36329272	0.082423156	3.549176261	1.406047024	0.98538533	NA
ASV_16s_12889	0.247045528	0.364136746	3.551088356	0.92018521	NA	NA
ASV_16s_12890	0.25193866	0.866670779	3.550863401	1.147406486	NA	NA
ASV_16s_12891	0.066672809	-0.242874361	3.551698954	0.343047765	NA	NA
ASV_16s_12892	0.306213603	1.078994474	3.550302562	1.208829227	0.990722184	NA
ASV_16s_12893	0.315858545	0.34270532	3.549953536	1.602179221	NA	NA
ASV_16s_12894	0.360924666	0.380032349	3.550433445	1.39189828	0.985818121	NA
ASV_16s_12895	0.30064021	0.288071871	3.550355728	1.536239027	0.98102848	NA
ASV_16s_12896	0.336210889	0.606886776	3.550855773	1.823310066	0.968969428	NA
ASV_16s_12897	0.241250338	-0.028975048	3.550855938	1.089655305	NA	NA
ASV_16s_12898	0.241250338	-0.028975048	3.550855938	1.089655305	NA	NA
ASV_16s_12899	0.241250338	-0.028975048	3.550855938	1.089655305	NA	NA
ASV_16s_12900	0.253406185	0.416670378	3.550662769	1.139818426	NA	NA
ASV_16s_12901	0.275659213	-0.073924362	3.550732311	1.121711687	NA	NA
ASV_16s_12902	0.245346713	-0.073828542	3.550759259	1.063085886	0.993745683	NA
ASV_16s_12903	0.249689265	0.18787346	3.550682732	1.152312369	0.99198377	NA
ASV_16s_12904	0.201330355	-0.073640538	3.55081274	0.964788275	NA	NA
ASV_16s_12905	0.201330355	-0.073640538	3.55081274	0.964788275	NA	NA
ASV_16s_12906	0.198650673	-0.073624517	3.550816757	0.958197443	0.995474979	NA
ASV_16s_12907	0.305114666	0.369091568	3.550434998	1.341087088	NA	NA
ASV_16s_12908	0.30390003	0.35286446	3.550229239	1.425307365	0.984783484	NA
ASV_16s_12909	0.299047136	0.36185127	3.550353023	1.364276859	NA	NA
ASV_16s_12910	0.33367532	-0.074059616	3.550694358	1.218261281	NA	NA
ASV_16s_12911	0.245314267	-0.073828457	3.550759283	1.063064827	NA	NA
ASV_16s_12912	0.259893453	0.339129465	3.550952451	0.846597745	NA	NA
ASV_16s_12913	0.346184986	0.344667148	3.550769489	0.951964699	0.995566645	NA
ASV_16s_12914	0.244643009	0.360347952	3.551008928	1.080421629	NA	NA
ASV_16s_12915	0.424126281	0.994134427	3.549989484	1.584326128	0.979245886	NA
ASV_16s_12916	0.29535697	0.315939521	3.550830375	1.136631572	0.99231352	NA
ASV_16s_12917	0.271454935	0.315939544	3.550851698	1.093937633	0.993167607	NA
ASV_16s_12918	0.270316743	0.315939545	3.550852807	1.091814573	0.993208429	NA
ASV_16s_12919	0.305712024	0.315939374	3.550822182	1.153995349	0.99194786	NA
ASV_16s_12920	0.338718003	0.315939352	3.550799363	1.205923459	0.990789871	NA
ASV_16s_12921	0.26373294	0.315939552	3.550859406	1.079369681	0.993444611	NA
ASV_16s_12922	0.262706353	0.315939554	3.550860465	1.07739942	0.993481518	NA
ASV_16s_12923	0.296281307	0.315939368	3.550829631	1.138137968	0.99228222	NA
ASV_16s_12924	0.286844523	0.315939372	3.550837573	1.121767704	0.992618084	NA
ASV_16s_12925	0.261337571	0.315939555	3.55086189	1.074761244	0.993530729	NA
ASV_16s_12926	0.283495907	0.450045014	3.550814663	1.742572351	0.97270692	NA
ASV_16s_12927	0.344045516	0.596256922	3.550629863	0.980516985	NA	NA
ASV_16s_12928	0.344045516	0.596256922	3.550629863	0.980516985	NA	NA
ASV_16s_12929	0.366241708	1.17879286	3.55032546	0.898537696	NA	NA
ASV_16s_12930	0.338021886	0.734510508	3.550894057	1.18039995	NA	NA
ASV_16s_12931	0.167441056	0.733403836	3.551215337	0.83274322	NA	NA
ASV_16s_12932	0.318213814	0.693380981	3.550785414	1.109137457	0.992870807	NA
ASV_16s_12933	0.255520245	0.693187416	3.550842356	0.998587935	NA	NA

ASV_16s_12934	0.216219943	0.280429714	3.551452311	0.697622905	NA	NA
ASV_16s_12935	0.216219943	0.280429714	3.551452311	0.697622905	NA	NA
ASV_16s_12936	0.216219943	0.280429714	3.551452311	0.697622905	NA	NA
ASV_16s_12937	0.216219943	0.280429714	3.551452311	0.697622905	NA	NA
ASV_16s_12938	0.231220138	0.03762477	3.55073782	1.053269482	NA	NA
ASV_16s_12939	0.228624354	0.029699944	3.55074141	1.064090264	NA	NA
ASV_16s_12940	0.219361194	-0.0939536	3.550603449	1.207966131	NA	NA
ASV_16s_12941	0.21860155	-0.092258123	3.550611641	1.204922283	NA	NA
ASV_16s_12942	0.221139401	-0.11150788	3.550567991	1.227318698	NA	NA
ASV_16s_12943	0.216334025	-0.115093054	3.550601182	1.221108468	0.990432731	NA
ASV_16s_12944	0.216339729	-0.128838084	3.550585645	1.232109443	0.990168696	NA
ASV_16s_12945	0.221657239	-0.139323479	3.55053397	1.250372942	0.989720451	NA
ASV_16s_12946	0.22718218	-0.213167505	3.55043887	1.285572628	0.988821348	NA
ASV_16s_12947	0.235868158	-0.225868025	3.550375045	1.315741981	0.988013431	NA
ASV_16s_12948	0.221185034	-0.216869437	3.550479532	1.268390791	0.989266041	NA
ASV_16s_12949	0.22372859	-0.246589028	3.550448999	1.273176063	0.989143309	NA
ASV_16s_12950	0.216385361	-0.23596933	3.550506667	1.248075266	0.989777525	NA
ASV_16s_12951	0.221196442	-0.243002033	3.550468368	1.264656274	0.989361225	NA
ASV_16s_12952	0.220948931	-0.255788388	3.5504662	1.260409503	0.989468829	NA
ASV_16s_12953	0.224246427	-0.273398252	3.550438384	1.266416366	0.98931643	NA
ASV_16s_12954	0.219446755	-0.294323317	3.550468502	1.233974882	0.990123479	NA
ASV_16s_12955	0.216413882	-0.304467114	3.550489864	1.213252727	NA	NA
ASV_16s_12956	0.219452459	-0.308231244	3.55046688	1.222233522	0.990405933	NA
ASV_16s_12957	0.216419586	-0.318695845	3.550489052	1.200482151	NA	NA
ASV_16s_12958	0.229725955	-0.361786202	3.550397859	1.206300834	0.990781098	NA
ASV_16s_12959	0.297851448	-0.680242782	3.550229429	0.822762335	0.997204657	NA
ASV_16s_12960	0.304220965	-0.680381617	3.550198428	0.829560135	NA	NA
ASV_16s_12961	0.216522259	-0.648337974	3.55066981	0.717536842	NA	NA
ASV_16s_12962	0.216522259	-0.648337974	3.55066981	0.717536842	NA	NA
ASV_16s_12964	0.238228855	-0.22051058	3.55034208	1.217819603	0.990510801	NA
ASV_16s_12965	0.225281503	-0.126802291	3.550446055	1.138099299	0.992283024	NA
ASV_16s_12966	0.224542512	-0.213166503	3.550450946	1.041417227	0.994132465	NA
ASV_16s_12967	0.209729319	0.272816516	3.551680414	0.486485781	NA	NA
ASV_16s_12968	0.230788883	0.188151707	3.550263787	1.265264747	0.989345752	NA
ASV_16s_12969	0.315771438	-0.532756417	3.55001112	1.220815617	0.990439699	NA
ASV_16s_12970	0.235742429	0.058538691	3.550304685	1.080160019	0.993429769	NA
ASV_16s_12971	0.22406115	-0.317475642	3.550301351	0.805909002	NA	NA
ASV_16s_12972	0.22135719	0.325868936	3.550513962	1.177960839	0.991425523	NA
ASV_16s_12973	0.194792281	0.818974241	3.551457889	0.648689094	NA	NA
ASV_16s_12974	0.292269844	-0.490532944	3.550308383	1.05389231	0.993911708	NA
ASV_16s_12975	0.229199162	0.3224824	3.550341602	1.239981284	0.989977015	NA
ASV_16s_12976	0.231585034	0.265374118	3.550298859	1.309708882	0.98817777	NA
ASV_16s_12977	0.289434836	-0.720762273	3.549976226	1.16269242	0.991760682	NA
ASV_16s_12978	0.24156694	0.682369398	3.550953451	1.016128711	NA	NA
ASV_16s_12979	0.289518096	-0.713074258	3.549976534	1.178268362	0.991418686	NA
ASV_16s_12980	0.290466761	-0.705178204	3.549976783	1.195951062	0.991019825	NA
ASV_16s_12981	0.283555806	-0.686857247	3.550476935	0.899410182	0.996292209	NA
ASV_16s_12982	0.276288858	0.331917492	3.551705488	0.432595671	NA	NA
ASV_16s_12983	0.296991309	-0.701119695	3.549955269	1.229553247	0.990230446	NA
ASV_16s_12984	0.27210749	-0.669192086	3.55008735	1.174186183	0.99150916	NA
ASV_16s_12985	0.275255726	-0.827580056	3.549893164	0.854705814	NA	NA
ASV_16s_12986	0.279016139	-0.665407726	3.550063897	1.203757007	0.990840136	NA
ASV_16s_12987	0.250340487	-0.459254201	3.55003513	0.865182407	0.996720454	NA
ASV_16s_12988	0.267261643	-0.627606204	3.55014847	1.188521699	0.991188786	NA
ASV_16s_12989	0.287685359	-0.603364405	3.550134112	1.256478268	0.989567836	NA
ASV_16s_12990	0.274928888	-0.661864643	3.550341674	0.963224488	0.995400163	NA
ASV_16s_12991	0.28002013	-0.631954789	3.550268682	1.090393618	0.993235664	NA
ASV_16s_12992	0.277200629	-0.531408002	3.550310655	1.246856808	NA	NA
ASV_16s_12993	0.280296812	0.18341446	3.549593549	1.226751563	NA	NA
ASV_16s_12994	0.214775447	-0.572828521	3.550588317	0.918843965	0.996033663	NA
ASV_16s_12995	0.269710779	0.220828249	3.549299176	1.339152141	0.987362523	NA
ASV_16s_12996	0.306546669	0.75301344	3.550060719	0.769695415	NA	NA
ASV_16s_12997	0.199245646	-0.148214144	3.550205495	0.805395928	NA	NA
ASV_16s_12998	0.330296307	-0.585655645	3.549998411	0.861686115	0.996762286	NA
ASV_16s_12999	0.310278341	0.654687032	3.550614074	0.915421281	0.99608002	NA
ASV_16s_13000	0.269636809	0.297951542	3.550548421	1.28529918	NA	NA
ASV_16s_13001	0.256590185	0.354635177	3.551201984	0.795782291	NA	NA

ASV_16s_13002	0.238131434	-0.071283014	3.550536379	1.136226033	NA	NA
ASV_16s_13003	0.285135644	1.51416653	3.550279845	0.441839663	0.99963281	NA
ASV_16s_13004	0.366580032	0.271650901	3.550534172	0.869823297	NA	NA
ASV_16s_13005	0.366580032	0.271650901	3.550534172	0.869823297	NA	NA
ASV_16s_13006	0.31064407	-0.592304525	3.550693739	1.01977364	NA	NA
ASV_16s_13007	0.22517978	-0.557734957	3.550848336	0.7188807	NA	NA
ASV_16s_13008	0.295270536	1.305308909	3.550601715	0.774226931	0.997698166	NA
ASV_16s_13009	0.153611082	1.346198606	3.551063567	0.353085799	NA	NA
ASV_16s_13010	0.255999402	1.277587226	3.550760955	0.724845824	NA	NA
ASV_16s_13011	0.277915347	1.748419268	3.550422521	0.481668449	0.999510874	NA
ASV_16s_13012	0.205563427	1.597616048	3.550719044	0.38654997	0.999765068	NA
ASV_16s_13013	0.247476297	1.270004637	3.550797214	0.713015632	NA	NA
ASV_16s_13014	0.234624535	0.38382673	3.551303794	0.725156	NA	NA
ASV_16s_13015	0.234624535	0.38382673	3.551303794	0.725156	NA	NA
ASV_16s_13016	0.416678105	0.403318402	3.550411186	0.913889902	NA	NA
ASV_16s_13017	0.152798707	-0.456670909	3.55118501	0.475518741	NA	NA
ASV_16s_13018	0.181820001	0.365082507	3.551626607	0.639360416	NA	NA
ASV_16s_13019	0.209147098	-0.077943864	3.550975454	0.999153531	NA	NA
ASV_16s_13020	0.285715328	-0.019317263	3.551328111	0.767403023	0.99776256	NA
ASV_16s_13021	0.213072103	-1.052011584	3.550457886	0.378445725	0.999781177	NA
ASV_16s_13022	0.140508868	1.13522914	3.551068221	0.459692737	NA	NA
ASV_16s_13023	0.244059316	0.836436127	3.549834352	0.929660155	NA	NA
ASV_16s_13024	0.265773621	0.416505634	3.550786937	0.699732971	0.998337788	NA
ASV_16s_13025	0.18055288	0.383447719	3.551269706	0.570847872	0.999143504	NA
ASV_16s_13026	0.354639616	0.418898287	3.550337287	0.792747447	NA	NA
ASV_16s_13027	0.22399139	0.403178532	3.550994188	0.642507303	NA	NA
ASV_16s_13028	0.15283915	-0.510767923	3.550739492	0.518396914	NA	NA
ASV_16s_13029	0.140073792	-0.494223813	3.550865999	0.491275381	NA	NA
ASV_16s_13030	0.140073792	-0.494223813	3.550865999	0.491275381	NA	NA
ASV_16s_13031	0.11104167	1.057643782	3.551242883	0.386812906	NA	NA
ASV_16s_13032	0.213850112	-0.078773397	3.550770095	0.970368724	NA	NA
ASV_16s_13033	0.146868606	0.220081643	3.550773719	0.627969479	0.998830066	NA
ASV_16s_13034	0.158886095	0.220070345	3.550758836	0.665055692	NA	NA
ASV_16s_13035	0.272811063	0.310936497	3.550827974	1.073605305	0.993552217	NA
ASV_16s_13036	0.270858474	0.310936609	3.550829699	1.069974874	NA	NA
ASV_16s_13037	0.167708865	0.596039345	3.550713764	0.624985925	NA	NA
ASV_16s_13038	0.219300813	-0.067840992	3.550668855	1.162177497	NA	NA
ASV_16s_13039	0.219300813	-0.067840992	3.550668855	1.162177497	NA	NA
ASV_16s_13040	0.197193714	-0.58949544	3.550545462	0.878028575	0.996563731	NA
ASV_16s_13041	0.223760478	-0.068734	3.55045931	1.292196161	NA	NA
ASV_16s_13042	0.226693874	0.685634605	3.551759893	0.753570164	0.997889423	NA
ASV_16s_13043	0.24415543	-0.062502185	3.550618174	1.145029494	0.992138004	NA
ASV_16s_13044	0.228220143	-0.068246487	3.55032124	1.325382125	NA	NA
ASV_16s_13045	0.25600975	0.905168342	3.551493378	0.70804529	NA	NA
ASV_16s_13046	0.233239166	0.974047071	3.550769373	0.560491867	NA	NA
ASV_16s_13047	0.218882379	-0.047985034	3.550558765	1.018563598	NA	NA
ASV_16s_13048	0.230697735	-0.067780001	3.550370605	1.297815799	NA	NA
ASV_16s_13049	0.157014559	0.193474825	3.550932689	0.788161449	NA	NA
ASV_16s_13050	0.202300945	1.319877161	3.551046863	0.551572475	NA	NA
ASV_16s_13051	0.313414878	0.695442271	3.550883411	1.146266844	0.992111933	NA
ASV_16s_13052	0.195911107	-0.253172106	3.55123299	0.637550494	NA	NA
ASV_16s_13053	0.12999843	0.281948796	3.551675662	0.536859588	NA	NA
ASV_16s_13054	0.12999843	0.281948796	3.551675662	0.536859588	NA	NA
ASV_16s_13055	0.064686351	0.288840567	3.551869198	0.333862886	NA	NA
ASV_16s_13056	0.488460474	-0.566030579	3.54951252	0.959278002	NA	NA
ASV_16s_13057	0.458894766	-0.193279173	3.549228148	1.1888971	NA	NA
ASV_16s_13058	0.39976335	-0.13145025	3.549299065	1.554392537	NA	NA
ASV_16s_13059	0.370184725	0.402926438	3.551196032	0.68598397	0.998441127	NA
ASV_16s_13060	0.281961073	0.332165616	3.551013171	0.6868092	0.998435048	NA
ASV_16s_13061	0.284041616	-0.547910498	3.54985502	0.714751449	NA	NA
ASV_16s_13062	0.314948465	1.330597255	3.550386612	0.734554368	NA	NA
ASV_16s_13063	0.3353668	1.520912694	3.550134589	1.004701891	0.994752331	NA
ASV_16s_13064	0.1794679	-0.078608645	3.550818876	0.88434844	NA	NA
ASV_16s_13065	0.394047302	-0.101149083	3.549340284	1.503324849	NA	NA
ASV_16s_13066	0.322097288	0.342188495	3.549965481	1.605501371	NA	NA
ASV_16s_13067	0.331215809	0.738212646	3.550090868	1.346101978	0.987165219	NA
ASV_16s_13068	0.200501445	-0.339248948	3.550737865	0.78426361	NA	NA

ASV_16s_13069	0.305200342	0.342677535	3.550008803	1.530027465	NA	NA
ASV_16s_13070	0.197602015	-0.073619193	3.550818367	0.955561961	NA	NA
ASV_16s_13071	0.27101043	0.34315623	3.550160898	1.411157345	NA	NA
ASV_16s_13072	0.306448975	0.387988113	3.550627074	1.282465506	NA	NA
ASV_16s_13073	0.233026187	0.326386155	3.550383874	1.27655451	NA	NA
ASV_16s_13074	0.33047435	0.343362055	3.549999472	1.536578485	NA	NA
ASV_16s_13075	0.24077141	-0.073812311	3.55076391	1.053661234	NA	NA
ASV_16s_13076	0.319744567	0.406257162	3.550704066	1.253206431	NA	NA
ASV_16s_13077	0.270103391	0.360359885	3.550970001	1.130352065	NA	NA
ASV_16s_13078	0.270058915	0.361984091	3.551020818	1.037098209	NA	NA
ASV_16s_13079	0.308879638	0.334247999	3.550870425	0.961506073	0.995425827	NA
ASV_16s_13080	0.240112583	0.36034557	3.551016728	1.07098406	NA	NA
ASV_16s_13081	0.240112583	0.36034557	3.551016728	1.07098406	NA	NA
ASV_16s_13082	0.240112583	0.36034557	3.551016728	1.07098406	NA	NA
ASV_16s_13083	0.240112583	0.36034557	3.551016728	1.07098406	NA	NA
ASV_16s_13084	0.240112583	0.36034557	3.551016728	1.07098406	NA	NA
ASV_16s_13085	0.274244003	0.31593941	3.550849025	1.099069606	0.993068289	NA
ASV_16s_13086	0.259001084	0.315939573	3.550864365	1.07019068	0.993615427	NA
ASV_16s_13087	0.259001084	0.315939573	3.550864365	1.07019068	0.993615427	NA
ASV_16s_13088	0.258658888	0.315939573	3.55086473	1.069523335	0.993627734	NA
ASV_16s_13089	0.25694791	0.315939576	3.55086657	1.066173696	0.993689282	NA
ASV_16s_13090	0.256263519	0.315939577	3.550867313	1.064827773	0.993713906	NA
ASV_16s_13091	0.408896402	1.067179059	3.550476261	0.979558858	NA	NA
ASV_16s_13092	0.32294378	1.008562503	3.550689083	0.925567775	0.995941565	NA
ASV_16s_13093	0.331762222	0.734489901	3.550900048	1.170895231	NA	NA
ASV_16s_13094	0.331762222	0.734489901	3.550900048	1.170895231	NA	NA
ASV_16s_13095	0.331762222	0.734489901	3.550900048	1.170895231	NA	NA
ASV_16s_13096	0.331762222	0.734489901	3.550900048	1.170895231	NA	NA
ASV_16s_13097	0.164340296	0.733362897	3.5512273	0.823958	NA	NA
ASV_16s_13098	0.265185716	1.011891855	3.55076825	0.831026782	0.997114271	NA
ASV_16s_13099	0.306364105	0.693350388	3.550794406	1.089903748	0.993245037	NA
ASV_16s_13100	0.314375842	0.693371467	3.550788256	1.102962841	0.992992339	NA
ASV_16s_13101	0.285728232	0.693290719	3.550811825	1.054697454	0.993897281	NA
ASV_16s_13102	0.289330091	0.693301711	3.550808614	1.060971865	0.993784111	NA
ASV_16s_13103	0.294674499	0.693317577	3.550803982	1.070210631	0.993615059	NA
ASV_16s_13104	0.277271336	0.693263706	3.550819715	1.039529984	0.99416541	NA
ASV_16s_13105	0.279641769	0.693271431	3.550817458	1.04381127	0.994090503	NA
ASV_16s_13106	0.314788844	0.693372484	3.55078796	1.103560375	0.992980635	NA
ASV_16s_13107	0.348223054	1.201906473	3.550241316	0.819823926	NA	NA
ASV_16s_13108	0.212140322	0.280877685	3.551477841	0.691182803	NA	NA
ASV_16s_13109	0.212140322	0.280877685	3.551477841	0.691182803	NA	NA
ASV_16s_13110	0.212140322	0.280877685	3.551477841	0.691182803	NA	NA
ASV_16s_13111	0.212140322	0.280877685	3.551477841	0.691182803	NA	NA
ASV_16s_13112	0.216283023	-0.072637136	3.550657657	1.17268243	NA	NA
ASV_16s_13113	0.216283023	-0.072637136	3.550657657	1.17268243	NA	NA
ASV_16s_13114	0.216541942	-0.087550403	3.550634505	1.187849973	NA	NA
ASV_16s_13115	0.21705978	-0.116588293	3.550593816	1.214799226	0.990582147	NA
ASV_16s_13116	0.21705978	-0.116588293	3.550593816	1.214799226	0.990582147	NA
ASV_16s_13117	0.217318699	-0.130782259	3.550575999	1.226592397	0.990301669	NA
ASV_16s_13118	0.217836536	-0.15867317	3.550544873	1.246764642	0.989809993	NA
ASV_16s_13119	0.212271515	-0.16182123	3.550585821	1.229872162	0.990222755	NA
ASV_16s_13120	0.218095455	-0.172425185	3.550531389	1.254086792	0.989627782	NA
ASV_16s_13121	0.218354374	-0.186082476	3.550519197	1.257037083	0.989553798	NA
ASV_16s_13122	0.218613293	-0.19966938	3.55050824	1.258985218	0.989504766	NA
ASV_16s_13123	0.212288628	-0.202568645	3.550555228	1.235114382	0.990095796	NA
ASV_16s_13124	0.214314346	-0.205921098	3.550537312	1.24283088	0.98990706	NA
ASV_16s_13125	0.219131131	-0.226811185	3.550490027	1.25965253	0.989487938	NA
ASV_16s_13126	0.215332909	-0.220967165	3.550521411	1.245986079	0.98982925	NA
ASV_16s_13127	0.238210828	-0.29080571	3.550342134	1.303253963	0.988352058	NA
ASV_16s_13128	0.238628063	-0.304065299	3.550337587	1.299162768	0.988461702	NA
ASV_16s_13129	0.214872112	-0.331302855	3.550501242	1.182063501	0.991334034	NA
ASV_16s_13130	0.216391401	-0.333070498	3.55048974	1.186343055	0.991237954	NA
ASV_16s_13131	0.232883995	-0.364895591	3.550377475	1.214145342	0.990597549	NA
ASV_16s_13132	0.239687528	-0.429074065	3.550356592	1.150174778	0.992029234	NA
ASV_16s_13133	0.240628428	-0.59673027	3.550479754	0.87835787	0.996559651	NA
ASV_16s_13134	0.075837611	-0.317804825	3.551639605	0.38549271	NA	NA
ASV_16s_13135	0.075837611	-0.317804825	3.551639605	0.38549271	NA	NA

ASV_16s_13136	0.285664637	-0.679962156	3.550292519	0.809368304	0.997347167	NA
ASV_16s_13137	0.222059095	-0.65288623	3.550638968	0.725946812	NA	NA
ASV_16s_13138	0.212436933	-0.644685432	3.550693194	0.711105869	NA	NA
ASV_16s_13139	0.212436933	-0.644685432	3.550693194	0.711105869	NA	NA
ASV_16s_13140	0.212436933	-0.644685432	3.550693194	0.711105869	NA	NA
ASV_16s_13141	0.212436933	-0.644685432	3.550693194	0.711105869	NA	NA
ASV_16s_13142	0.212436933	-0.644685432	3.550693194	0.711105869	NA	NA
ASV_16s_13143	0.067683765	-0.16898138	3.550898648	0.335592528	NA	NA
ASV_16s_13144	0.067683765	-0.16898138	3.550898648	0.335592528	NA	NA
ASV_16s_13145	0.060035997	-0.168784756	3.550948252	0.294992218	NA	NA
ASV_16s_13146	0.072032685	-0.169076235	3.550875107	0.357682309	NA	NA
ASV_16s_13147	0.094353508	0.220164606	3.550883002	0.435637123	NA	NA
ASV_16s_13149	0.226171216	-0.040024007	3.550351538	1.145112817	0.99213625	NA
ASV_16s_13150	0.229563265	-0.009870802	3.550492969	1.05534195	0.993885717	NA
ASV_16s_13151	0.275478098	-0.74893004	3.550251725	0.89976544	0.996287584	NA
ASV_16s_13152	0.251427496	-0.129233597	3.549954273	1.107655412	0.992900097	NA
ASV_16s_13153	0.289539483	-0.026145099	3.549684806	1.407233822	NA	NA
ASV_16s_13154	0.284530437	-0.689167651	3.550328304	1.022589543	0.994455858	NA
ASV_16s_13155	0.283411667	-0.750305882	3.550072197	1.039577942	0.994164574	NA
ASV_16s_13156	0.287283471	-0.751208126	3.550040784	1.064542014	0.993719126	NA
ASV_16s_13157	0.26013127	-1.046856643	3.550247092	0.59581937	NA	NA
ASV_16s_13158	0.233819302	-0.68982507	3.550402873	0.972933549	0.995253419	NA
ASV_16s_13159	0.271153677	-0.708451566	3.550353776	0.905736425	0.996209285	NA
ASV_16s_13160	0.307428176	-0.756223817	3.549893021	1.155857111	0.991908018	NA
ASV_16s_13161	0.276471877	-0.717077175	3.550051751	1.105352188	0.992945467	NA
ASV_16s_13162	0.239354239	0.181941655	3.55011773	1.311479522	0.988129683	NA
ASV_16s_13163	0.240389914	0.093867116	3.550043305	1.300507395	0.988425736	NA
ASV_16s_13164	0.401164469	-1.090286355	3.549797488	0.760737588	NA	NA
ASV_16s_13165	0.267747466	-0.687571563	3.550097786	1.126604394	0.99251983	NA
ASV_16s_13166	0.273372828	-0.678716293	3.550077063	1.16131864	0.991790428	NA
ASV_16s_13167	0.259592931	-0.662438257	3.550147208	1.124004667	NA	NA
ASV_16s_13168	0.239651325	-0.259830493	3.55034472	1.014923792	NA	NA
ASV_16s_13169	0.2678711	-0.639717434	3.550130731	1.176642852	NA	NA
ASV_16s_13170	0.280964237	-0.65737152	3.550065957	1.214996296	NA	NA
ASV_16s_13171	0.256647839	-1.074597907	3.550073592	0.731069452	0.998085458	NA
ASV_16s_13172	0.27890564	-0.632767856	3.550105385	1.224130729	0.990360638	NA
ASV_16s_13173	0.299869352	-0.16665851	3.55045203	1.264603822	NA	NA
ASV_16s_13174	0.265050365	-0.580232376	3.513070425	1.215917886	0.990555762	NA
ASV_16s_13175	0.343836143	-0.601680245	3.550159285	1.181036753	NA	NA
ASV_16s_13176	0.245622523	-0.917299842	3.550263517	0.590694907	NA	NA
ASV_16s_13177	0.268759587	-0.522091977	3.550330947	1.206866265	NA	NA
ASV_16s_13178	0.278332326	-0.526971412	3.550857572	0.948664841	NA	NA
ASV_16s_13179	0.234784726	-0.847704341	3.550338766	0.591441709	0.999038034	NA
ASV_16s_13180	0.259687434	-0.906338945	3.55016723	0.816976529	NA	NA
ASV_16s_13181	0.275564956	0.183361688	3.549602273	1.212825596	NA	NA
ASV_16s_13182	0.187574652	-0.536101797	3.550430473	0.582326991	NA	NA
ASV_16s_13183	0.339386886	0.51302351	3.550196694	1.35542289	NA	NA
ASV_16s_13184	0.26166896	0.189867078	3.549816438	1.031560351	NA	NA
ASV_16s_13185	0.292306878	-0.357474247	3.550110215	1.196565396	0.991005764	NA
ASV_16s_13186	0.251169224	1.273307105	3.550781308	0.718156629	NA	NA
ASV_16s_13187	0.295033488	1.689393005	3.550520392	0.549253855	0.999245404	NA
ASV_16s_13188	0.245572248	0.353777216	3.551931511	0.432302823	NA	NA
ASV_16s_13189	0.213734907	-0.07336129	3.550847893	0.988651589	NA	NA
ASV_16s_13190	0.249544765	0.174381524	3.5507871	1.549157173	0.980558878	NA
ASV_16s_13191	0.178389435	0.363315211	3.551647706	0.63296038	NA	NA
ASV_16s_13192	0.178389435	0.363315211	3.551647706	0.63296038	NA	NA
ASV_16s_13193	0.178389435	0.363315211	3.551647706	0.63296038	NA	NA
ASV_16s_13194	0.263958536	-28.64611717	3.55053027	-0.368463894	NA	NA
ASV_16s_13195	0.180721812	-0.469704189	3.55117547	0.564369385	NA	NA
ASV_16s_13196	0.335872986	-0.825091126	3.549802134	0.913017497	0.996112366	NA
ASV_16s_13197	0.26877575	-0.168443859	3.549979623	0.889106973	0.996424724	NA
ASV_16s_13198	0.266980945	-0.5593315	3.550665934	0.864003813	NA	NA
ASV_16s_13199	0.275798373	0.835998266	3.550307519	0.716759686	NA	NA
ASV_16s_13200	0.233199514	-0.562364717	3.550411308	0.722577005	NA	NA
ASV_16s_13201	0.14668455	-0.808302176	3.551038311	0.313040012	NA	NA
ASV_16s_13202	0.333191014	0.545527419	3.549266483	1.013843744	0.994602116	NA
ASV_16s_13203	0.137857757	1.128945063	3.551081617	0.453504342	NA	NA

ASV_16s_13204	0.158689854	1.173328207	3.55097648	0.499658585	NA	NA
ASV_16s_13205	0.300413593	-0.006960651	3.549811806	1.421799176	0.984894201	NA
ASV_16s_13206	0.246056533	0.411463053	3.550886181	0.674031887	NA	NA
ASV_16s_13207	0.347948303	0.418886858	3.550364544	0.786599343	NA	NA
ASV_16s_13208	0.232586567	0.69475566	3.550522099	1.065455239	NA	NA
ASV_16s_13209	0.343374215	-0.548731112	3.549654608	0.775412233	0.997686857	NA
ASV_16s_13210	0.337750722	-0.017311546	3.549575446	1.377804851	NA	NA
ASV_16s_13211	0.13743089	-0.490283437	3.550893719	0.485364823	NA	NA
ASV_16s_13212	0.13743089	-0.490283437	3.550893719	0.485364823	NA	NA
ASV_16s_13213	0.246056533	-0.078886527	3.550736729	1.040300186	NA	NA
ASV_16s_13214	0.209815204	-0.078757049	3.550775003	0.960903796	NA	NA
ASV_16s_13215	0.209815204	-0.078757049	3.550775003	0.960903796	NA	NA
ASV_16s_13216	0.23736854	0.248427147	3.550763693	1.59789798	0.978725645	NA
ASV_16s_13217	0.241479053	0.150952212	3.550776442	1.679052704	0.975456332	NA
ASV_16s_13218	0.145991488	0.220082439	3.550774906	0.625139335	0.998847165	NA
ASV_16s_13219	0.151826732	0.220076669	3.550767299	0.643509429	0.99873314	NA
ASV_16s_13220	0.156200165	0.220072735	3.550761969	0.65693388	NA	NA
ASV_16s_13221	0.156050203	0.220072752	3.550762146	0.656478852	0.998648244	NA
ASV_16s_13222	0.157542411	0.289031332	3.550744913	0.922568691	0.995982814	NA
ASV_16s_13223	0.251143559	0.310937847	3.550848622	1.031782462	NA	NA
ASV_16s_13224	0.249544895	0.310937955	3.550850288	1.028553315	0.994354687	NA
ASV_16s_13225	0.232455917	0.3109392	3.550869501	0.992909505	NA	NA
ASV_16s_13226	0.212441828	-0.187610464	3.550516789	0.888896061	NA	NA
ASV_16s_13227	0.221212126	-0.068544345	3.55039688	1.301951008	NA	NA
ASV_16s_13228	0.221707644	-0.068493331	3.550378838	1.307327205	NA	NA
ASV_16s_13229	0.222203162	-0.068438379	3.550364751	1.30904581	NA	NA
ASV_16s_13230	0.240617914	-0.068580874	3.550269005	1.378316752	NA	NA
ASV_16s_13231	0.226662827	-0.067651064	3.550409642	1.273473916	NA	NA
ASV_16s_13232	0.235978581	-0.067931812	3.550325609	1.309098115	NA	NA
ASV_16s_13233	0.227653864	-0.067323135	3.550470831	1.250985842	NA	NA
ASV_16s_13234	0.127103423	0.30263072	3.551861106	0.316603867	NA	NA
ASV_16s_13235	0.268978372	1.120103401	3.550818366	0.716678764	NA	NA
ASV_16s_13236	0.185018829	0.27939603	3.551579351	0.660745366	NA	NA
ASV_16s_13237	0.176886434	0.279164115	3.551599893	0.644697525	NA	NA
ASV_16s_13238	0.247000099	-0.188995778	3.551188258	1.4838765	0.98286261	NA
ASV_16s_13239	0.147292728	0.280705942	3.551644681	0.579588227	NA	NA
ASV_16s_13240	0.178766514	-0.503924199	3.551219692	0.639093635	NA	NA
ASV_16s_13241	0.340474016	-0.690987443	3.549488704	0.864281896	NA	NA
ASV_16s_13242	0.27868234	-0.547819497	3.549877365	0.708651076	NA	NA
ASV_16s_13243	0.26663618	1.100132243	3.550320818	1.110494548	NA	NA
ASV_16s_13244	0.35674702	0.092864337	3.550118108	1.752634967	0.972255948	NA
ASV_16s_13245	0.33722073	0.743903415	3.550888533	1.373088348	NA	NA
ASV_16s_13246	0.23231514	-0.028935106	3.550868843	1.070692056	NA	NA
ASV_16s_13247	0.23231514	-0.028935106	3.550868843	1.070692056	NA	NA
ASV_16s_13248	0.321865558	0.352812288	3.550200501	1.417752069	0.985021321	NA
ASV_16s_13249	0.324053701	0.342996927	3.549976502	1.553170324	NA	NA
ASV_16s_13250	0.324536653	0.347504233	3.550137342	1.44063714	NA	NA
ASV_16s_13251	0.324939113	0.376972351	3.550504291	1.29787498	NA	NA
ASV_16s_13252	0.265007101	0.360357612	3.55097721	1.120685771	NA	NA
ASV_16s_13253	0.265007101	0.360357612	3.55097721	1.120685771	NA	NA
ASV_16s_13254	0.27807403	0.339128363	3.55091412	0.877612414	0.996568883	NA
ASV_16s_13255	0.235582157	0.360343097	3.551024826	1.061377073	NA	NA
ASV_16s_13256	0.235582157	0.360343097	3.551024826	1.061377073	NA	NA
ASV_16s_13257	0.273373144	0.315939569	3.550849866	1.097418968	0.993100333	NA
ASV_16s_13258	0.258576646	0.315939587	3.550864829	1.069310323	0.99363166	NA
ASV_16s_13259	0.262801979	0.315939447	3.550860382	1.077498951	0.993479656	NA
ASV_16s_13260	0.262920964	0.315939447	3.550860258	1.077730344	0.993475328	NA
ASV_16s_13261	0.252558249	0.315939595	3.550871412	1.057443111	0.993847921	NA
ASV_16s_13262	0.280526405	0.315939433	3.550843206	1.110413749	0.992845521	NA
ASV_16s_13263	0.246056533	0.315939604	3.550878887	1.044305472	NA	NA
ASV_16s_13264	0.246056533	0.315939604	3.550878887	1.044305472	NA	NA
ASV_16s_13265	0.246056533	0.488138635	3.55084754	1.649117572	0.976693957	NA
ASV_16s_13266	0.290798462	0.626902245	3.550800501	1.445653549	0.984131725	NA
ASV_16s_13267	0.426555122	0.734738019	3.550828194	1.298245567	0.988486195	NA
ASV_16s_13268	0.325502557	0.734467572	3.550906267	1.161211629	NA	NA
ASV_16s_13269	0.161239535	0.733320385	3.551239718	0.815039058	NA	NA
ASV_16s_13270	0.29220266	0.693307332	3.550806102	1.06596274	0.993693145	NA

ASV_16s_13271	0.26345819	0.693216193	3.550833674	1.013846894	0.994602063	NA
ASV_16s_13272	0.291348444	0.69330478	3.550806843	1.064483415	0.993720196	NA
ASV_16s_13273	0.294127893	0.693313018	3.550804449	1.069264287	0.993632508	NA
ASV_16s_13274	0.334400682	0.693419928	3.550774182	1.134168781	0.99236452	NA
ASV_16s_13275	0.277129403	0.69326344	3.550819861	1.039228029	0.99417067	NA
ASV_16s_13276	0.279160216	0.693270072	3.550817923	1.042901355	0.994106474	NA
ASV_16s_13277	0.283787875	0.693284833	3.550813609	1.051176193	0.993960214	NA
ASV_16s_13278	0.2080607	0.281670201	3.551501106	0.684580048	NA	NA
ASV_16s_13279	0.2080607	0.281670201	3.551501106	0.684580048	NA	NA
ASV_16s_13280	0.2080607	0.281670201	3.551501106	0.684580048	NA	NA
ASV_16s_13281	0.211685564	-0.046083392	3.550738342	1.126940732	NA	NA
ASV_16s_13282	0.225445285	-0.120223999	3.550526248	1.231291302	NA	NA
ASV_16s_13283	0.212980158	-0.121839404	3.550620626	1.202020149	0.99088031	NA
ASV_16s_13284	0.212980158	-0.121839404	3.550620626	1.202020149	0.99088031	NA
ASV_16s_13285	0.213756915	-0.164556929	3.550571691	1.232721785	0.990153867	NA
ASV_16s_13286	0.220215077	-0.176099136	3.550512747	1.25386004	0.989633454	NA
ASV_16s_13287	0.21274976	-0.176275674	3.550570086	1.234396293	0.990113247	NA
ASV_16s_13288	0.214015834	-0.17853204	3.550558306	1.239614497	0.989985997	NA
ASV_16s_13289	0.214274752	-0.192420161	3.550546262	1.242115549	0.989924649	NA
ASV_16s_13290	0.214274752	-0.192420161	3.550546262	1.242115549	0.989924649	NA
ASV_16s_13291	0.214533671	-0.206257334	3.550535524	1.243548957	0.989889384	NA
ASV_16s_13292	0.210493643	-0.226896886	3.550556718	1.226811414	0.990296412	NA
ASV_16s_13293	0.20821471	-0.223272306	3.550576917	1.218437851	0.990496156	NA
ASV_16s_13294	0.208220414	-0.237146686	3.550570757	1.216497357	0.990542076	NA
ASV_16s_13295	0.215310428	-0.247763633	3.550510829	1.241318544	0.989944224	NA
ASV_16s_13296	0.215310428	-0.247763633	3.550510829	1.241318544	0.989944224	NA
ASV_16s_13297	0.211264695	-0.255525872	3.550539997	1.223927871	0.990365488	NA
ASV_16s_13298	0.208231822	-0.265070625	3.55056198	1.208169532	0.990737578	NA
ASV_16s_13299	0.212789688	-0.271481542	3.550524119	1.223222604	0.990382336	NA
ASV_16s_13300	0.226871374	-0.344673881	3.550415033	1.212926991	0.990626204	NA
ASV_16s_13301	0.074379195	-0.311194617	3.551651399	0.380316	NA	NA
ASV_16s_13302	0.074379195	-0.311194617	3.551651399	0.380316	NA	NA
ASV_16s_13303	0.074379195	-0.311194617	3.551651399	0.380316	NA	NA
ASV_16s_13304	0.278040864	-0.679338888	3.550334031	0.800578507	0.997438045	NA
ASV_16s_13305	0.208351608	-0.640889846	3.550717225	0.70454963	NA	NA
ASV_16s_13306	0.208351608	-0.640889846	3.550717225	0.70454963	NA	NA
ASV_16s_13307	0.066382154	-0.168951093	3.550906301	0.328832285	NA	NA
ASV_16s_13308	0.066382154	-0.168951093	3.550906301	0.328832285	NA	NA
ASV_16s_13309	0.068081966	0.018778734	3.55089233	0.526069298	0.999345317	NA
ASV_16s_13310	0.073411649	-0.01177595	3.550860857	0.56461904	0.999173795	NA
ASV_16s_13311	0.058881458	-0.168750586	3.550956863	0.2886545	NA	NA
ASV_16s_13312	0.069994254	0.220244958	3.550988968	0.323779256	NA	NA
ASV_16s_13313	0.092539017	0.220169161	3.550888991	0.427878903	NA	NA
ASV_16s_13316	0.224079759	-0.260167584	3.550654571	1.156488082	0.991894487	NA
ASV_16s_13317	0.240488765	-0.519992002	3.55063531	1.070973381	0.993600973	NA
ASV_16s_13318	0.216220233	-0.019500223	3.550542145	1.117873872	0.99269659	NA
ASV_16s_13319	0.212371071	-0.388394787	3.550605394	0.708768859	NA	NA
ASV_16s_13320	0.213184999	-0.496888791	3.550673724	1.040129847	0.994154951	NA
ASV_16s_13321	0.227863774	-0.243786561	3.550946015	1.015578968	0.994573296	NA
ASV_16s_13322	0.245535627	-0.603367639	3.550553303	1.057082278	0.993854422	NA
ASV_16s_13323	0.30998314	-0.771988929	3.549949619	1.049179288	0.993995719	NA
ASV_16s_13324	0.301797327	-0.979598106	3.550208474	0.685747875	NA	NA
ASV_16s_13325	0.214852061	-0.24479314	3.550283175	0.862752392	NA	NA
ASV_16s_13326	0.254799844	-0.715691243	3.550229745	0.991524848	0.994964096	NA
ASV_16s_13327	0.251765203	-0.479330546	3.55035529	1.09953771	0.993059185	NA
ASV_16s_13328	0.28196838	-0.72449701	3.550022945	1.115689913	0.99274039	NA
ASV_16s_13329	0.220453162	0.686810664	3.551160835	0.967346346	NA	NA
ASV_16s_13330	0.274012767	-0.696582595	3.550060719	1.134634752	0.992354887	NA
ASV_16s_13331	0.297414532	0.849854581	3.550231994	1.239993644	0.989976712	NA
ASV_16s_13332	0.257984304	0.221932609	3.550378675	1.406656463	0.985366511	NA
ASV_16s_13333	0.276456101	-0.680426174	3.55006263	1.170976478	NA	NA
ASV_16s_13334	0.262262598	-0.670361324	3.550131989	1.127638261	0.992498722	NA
ASV_16s_13335	0.256103368	-0.670815515	3.550165123	1.105732678	0.992937985	NA
ASV_16s_13336	0.26662745	-0.651278384	3.550123472	1.161339338	0.99178998	NA
ASV_16s_13337	0.224846876	0.131725544	3.550380773	1.194696569	NA	NA
ASV_16s_13338	0.276796336	-0.628994989	3.550117339	1.215393334	NA	NA
ASV_16s_13339	0.21490838	-0.575678577	3.550514391	0.908643955	NA	NA

ASV_16s_13340	0.269709543	-0.597963997	3.550187043	1.203258321	0.990851682	NA
ASV_16s_13341	0.269646089	-0.596472055	3.550189794	1.200586378	0.99091339	NA
ASV_16s_13342	0.313788632	0.105015802	3.550797882	1.094329472	0.993160056	NA
ASV_16s_13343	0.231167278	0.91692849	3.550272664	0.405950982	0.999723223	NA
ASV_16s_13344	0.271817563	-0.12870736	3.550666395	1.21199999	NA	NA
ASV_16s_13345	0.291411514	-0.55666272	3.550223412	1.267870346	NA	NA
ASV_16s_13346	0.23800371	0.911675072	3.55024469	0.504572725	0.999429565	NA
ASV_16s_13347	0.264407501	-0.436880075	3.550459014	1.168316525	0.9916382	NA
ASV_16s_13348	0.2706638	-0.436885492	3.550448929	1.185136325	NA	NA
ASV_16s_13349	0.331400722	0.498369093	3.550246009	1.306445599	NA	NA
ASV_16s_13350	0.235454563	-0.089477394	3.550751497	1.039722496	NA	NA
ASV_16s_13351	0.316606023	0.248831296	3.550093628	1.498214586	NA	NA
ASV_16s_13352	0.289902169	0.131171489	3.55072228	1.744951226	0.972600688	NA
ASV_16s_13353	0.237957879	1.64036291	3.550577981	0.386180575	0.999765819	NA
ASV_16s_13354	0.304120046	0.423177151	3.551026808	0.908855659	NA	NA
ASV_16s_13355	0.316187222	0.496445474	3.549604624	1.406742235	0.985363861	NA
ASV_16s_13356	0.258596134	1.750119018	3.55032379	0.42405414	0.999679804	NA
ASV_16s_13357	0.130804269	1.305942798	3.55124826	0.290410838	NA	NA
ASV_16s_13358	0.232604389	1.201219351	3.55085245	0.850649028	NA	NA
ASV_16s_13359	0.238137569	1.261011995	3.550837953	0.699540112	NA	NA
ASV_16s_13360	0.235227736	1.025740881	3.550678176	1.085453548	NA	NA
ASV_16s_13361	0.254704331	0.388288538	3.551184664	0.752608592	NA	NA
ASV_16s_13362	0.273323065	0.392876074	3.55107628	0.77621301	NA	NA
ASV_16s_13363	0.236898211	0.384254948	3.551290274	0.72833503	NA	NA
ASV_16s_13364	0.265212226	-0.564857165	3.550639075	0.862382647	NA	NA
ASV_16s_13365	0.251533653	-0.562936329	3.550286986	0.746493785	NA	NA
ASV_16s_13366	0.251533653	-0.562936329	3.550286986	0.746493785	NA	NA
ASV_16s_13367	0.257048935	-0.563072027	3.550252943	0.753381114	NA	NA
ASV_16s_13368	0.207309874	-18.84198888	3.551644044	-0.606274032	NA	NA
ASV_16s_13369	0.315137802	1.314509223	3.550525965	0.796081401	NA	NA
ASV_16s_13370	0.315137802	1.314509223	3.550525965	0.796081401	NA	NA
ASV_16s_13371	0.195357578	1.513581902	3.550761582	0.385635476	NA	NA
ASV_16s_13372	0.273418531	0.906100317	3.550201594	1.275251741	0.989089804	NA
ASV_16s_13373	0.260991328	0.415592043	3.550812529	0.693675835	NA	NA
ASV_16s_13374	0.215538885	0.399777959	3.551040983	0.629599074	NA	NA
ASV_16s_13375	0.215538885	0.399777959	3.551040983	0.629599074	NA	NA
ASV_16s_13376	0.189984291	0.18272724	3.550341434	1.154015351	0.991947433	NA
ASV_16s_13377	0.330555624	-0.225982423	3.549630505	1.418809811	0.98498816	NA
ASV_16s_13378	0.121098733	-0.459199553	3.551059377	0.446539359	NA	NA
ASV_16s_13379	0.121098733	-0.459199553	3.551059377	0.446539359	NA	NA
ASV_16s_13380	0.147071635	-0.503517938	3.55079465	0.506317591	NA	NA
ASV_16s_13381	0.134787989	-0.485666721	3.550919946	0.479287327	NA	NA
ASV_16s_13382	0.205780296	-0.078740035	3.550780102	0.951268954	NA	NA
ASV_16s_13383	0.215794458	0.102789149	3.550786948	1.585127965	0.97921536	NA
ASV_16s_13384	0.156719143	0.383131906	3.550736001	1.057064353	0.993854745	NA
ASV_16s_13385	0.161380229	0.596023093	3.550720183	0.606962681	NA	NA
ASV_16s_13386	0.20659183	0.369299688	3.551534095	0.494218363	NA	NA
ASV_16s_13387	0.309932939	2.026905897	3.549924377	0.344109925	NA	NA
ASV_16s_13388	0.212222035	-0.068362721	3.550644989	1.174023239	NA	NA
ASV_16s_13389	0.22142767	-0.06889861	3.550531627	1.266261664	0.989320372	NA
ASV_16s_13390	0.309099412	0.157261579	3.549603092	1.004549513	NA	NA
ASV_16s_13391	0.233711153	-0.068439226	3.550292657	1.345480917	0.987182927	NA
ASV_16s_13392	0.24535493	0.37801089	3.551521991	0.59236956	NA	NA
ASV_16s_13393	0.257457316	0.344965071	3.551946263	0.520932762	0.999366167	NA
ASV_16s_13394	0.23136006	-0.067097876	3.55046704	1.242762494	NA	NA
ASV_16s_13395	0.231338037	-0.063249536	3.55050331	1.226723104	NA	NA
ASV_16s_13396	0.253038833	0.320826688	3.551045149	0.82298016	0.997202299	NA
ASV_16s_13397	0.289903153	0.69460307	3.550867337	1.092514793	0.993194982	NA
ASV_16s_13398	0.410896587	-0.153200577	3.549091756	1.385182601	NA	NA
ASV_16s_13399	0.273323065	-0.547726255	3.549900579	0.702442156	NA	NA
ASV_16s_13400	0.279637126	1.120298183	3.550255013	1.149576182	0.992041937	NA
ASV_16s_13401	0.477895665	-0.029479155	3.550689568	1.435385394	NA	NA
ASV_16s_13402	0.401905739	0.407224497	3.550226932	1.713795366	0.973973316	NA
ASV_16s_13403	0.295035961	0.379817032	3.550491522	1.270983382	0.989199654	NA
ASV_16s_13404	0.288021746	0.13449394	3.550084813	1.510284585	0.981951471	NA
ASV_16s_13405	0.267328528	0.326117583	3.550310711	1.379852067	0.986180398	NA
ASV_16s_13406	0.186728741	-0.073557197	3.550836051	0.927776136	NA	NA

ASV_16s_13407	0.233498442	0.332955429	3.550299803	1.297025161	NA	NA
ASV_16s_13408	0.286623545	0.343413787	3.550135315	1.424753648	NA	NA
ASV_16s_13409	0.259910811	0.36035533	3.550984701	1.110836362	NA	NA
ASV_16s_13410	0.259910811	0.36035533	3.550984701	1.110836362	NA	NA
ASV_16s_13411	0.383648693	0.363403793	3.550453895	0.961163264	0.995430935	NA
ASV_16s_13412	0.239713234	0.334257576	3.550971386	0.851922791	NA	NA
ASV_16s_13413	0.231051731	0.360340459	3.551033238	1.051591681	NA	NA
ASV_16s_13414	0.231051731	0.360340459	3.551033238	1.051591681	NA	NA
ASV_16s_13415	0.279470408	0.575442564	3.550920329	1.787478824	0.970661718	NA
ASV_16s_13416	0.299372475	0.315939552	3.550827143	1.143374723	NA	NA
ASV_16s_13417	0.270917672	0.315939583	3.550852244	1.092818844	0.993189138	NA
ASV_16s_13418	0.26977948	0.315939584	3.550853358	1.090690688	0.993229976	NA
ASV_16s_13419	0.273781602	0.315939462	3.55084948	1.098144633	0.993086257	NA
ASV_16s_13420	0.265226711	0.31593959	3.550857907	1.082089425	0.993393447	NA
ASV_16s_13421	0.348290412	0.769717739	3.550186106	1.682562618	0.975308786	NA
ASV_16s_13422	0.258776653	0.315939598	3.55086462	1.069678894	NA	NA
ASV_16s_13423	0.241324676	0.315939624	3.550884589	1.034498177	NA	NA
ASV_16s_13424	0.267631955	0.478189387	3.550827089	1.71788906	0.973795268	NA
ASV_16s_13425	0.343675144	1.564067174	3.550354152	0.69080665	NA	NA
ASV_16s_13426	0.311287909	1.153912636	3.550500383	0.847122631	0.996932785	NA
ASV_16s_13427	0.319242892	0.734445389	3.550912731	1.151342072	NA	NA
ASV_16s_13428	0.158138775	0.733276216	3.551252619	0.806000947	NA	NA
ASV_16s_13429	0.302406674	0.693339914	3.550797575	1.083266784	0.99337122	NA
ASV_16s_13430	0.273759064	0.693249509	3.550823139	1.033077247	0.994277161	NA
ASV_16s_13431	0.274089312	0.693250625	3.550822814	1.033683175	0.994266726	NA
ASV_16s_13432	0.254113936	0.693181172	3.550843982	0.995690721	0.994897752	NA
ASV_16s_13433	0.305213781	0.693347612	3.550795327	1.087935281	NA	NA
ASV_16s_13434	0.271390137	0.693244365	3.550825495	1.028703058	0.994352132	NA
ASV_16s_13435	0.241324676	0.69312864	3.550859362	0.969880807	NA	NA
ASV_16s_13436	0.241324676	0.69312864	3.550859362	0.969880807	NA	NA
ASV_16s_13437	0.241324676	0.69312864	3.550859362	0.969880807	NA	NA
ASV_16s_13438	0.203981079	0.282704689	3.551523034	0.677840307	NA	NA
ASV_16s_13439	0.203981079	0.282704689	3.551523034	0.677840307	NA	NA
ASV_16s_13440	0.203981079	0.282704689	3.551523034	0.677840307	NA	NA
ASV_16s_13441	0.207088105	-0.017215122	3.550832116	1.075820726	NA	NA
ASV_16s_13442	0.224397165	-0.091156673	3.550568973	1.19702149	NA	NA
ASV_16s_13443	0.207864861	-0.06647163	3.550739004	1.133108619	NA	NA
ASV_16s_13444	0.20812378	-0.082099133	3.550713216	1.148954759	NA	NA
ASV_16s_13445	0.20812378	-0.082099133	3.550713216	1.148954759	NA	NA
ASV_16s_13446	0.208382699	-0.097414158	3.550689652	1.163404154	NA	NA
ASV_16s_13447	0.208382699	-0.097414158	3.550689652	1.163404154	NA	NA
ASV_16s_13448	0.214917112	-0.139524821	3.55058615	1.211891494	0.990650516	NA
ASV_16s_13449	0.204089456	-0.116848102	3.55070246	1.17085469	0.991582552	NA
ASV_16s_13450	0.208900537	-0.127271422	3.550648486	1.188927761	0.991179603	NA
ASV_16s_13451	0.207133737	-0.137756829	3.55065204	1.192622201	0.991095779	NA
ASV_16s_13452	0.209159456	-0.141886855	3.550630594	1.199892179	0.99092938	NA
ASV_16s_13453	0.209418374	-0.156337546	3.550614338	1.209554309	0.990705246	NA
ASV_16s_13454	0.209418374	-0.156337546	3.550614338	1.209554309	0.990705246	NA
ASV_16s_13455	0.204100864	-0.145780449	3.550669946	1.191575076	0.991119588	NA
ASV_16s_13456	0.204106568	-0.160053471	3.550656047	1.19775897	0.990978405	NA
ASV_16s_13457	0.206897635	-0.179396405	3.550615695	1.211751318	0.990653805	NA
ASV_16s_13458	0.210195131	-0.198996609	3.550574503	1.226776109	0.990297259	NA
ASV_16s_13459	0.219614961	-0.214342755	3.55049278	1.255775741	0.989585468	NA
ASV_16s_13460	0.20412368	-0.202435448	3.550622912	1.203388393	0.990848671	NA
ASV_16s_13461	0.223629293	-0.246372916	3.550450166	1.267226004	0.989295785	NA
ASV_16s_13462	0.208940465	-0.224411853	3.550570556	1.221009636	0.990435083	NA
ASV_16s_13463	0.210712969	-0.227219354	3.550554928	1.227502241	0.990279817	NA
ASV_16s_13464	0.204135088	-0.230689589	3.550607707	1.201555732	0.990891033	NA
ASV_16s_13465	0.211489725	-0.269639537	3.550534955	1.218890641	0.990485421	NA
ASV_16s_13466	0.20642543	-0.262449142	3.550577578	1.2019803	0.99088123	NA
ASV_16s_13467	0.211748644	-0.284156954	3.550529282	1.209860225	0.990698093	NA
ASV_16s_13468	0.207443993	-0.278209029	3.550565291	1.196033648	0.991017936	NA
ASV_16s_13469	0.224700304	-0.342326673	3.550430037	1.207135355	0.990761679	NA
ASV_16s_13470	0.244766754	-0.362559043	3.550302534	1.259540241	0.989490771	NA
ASV_16s_13471	0.209492527	-0.339837362	3.55054467	1.151987167	0.991990697	NA
ASV_16s_13472	0.229120229	-0.361126944	3.550402203	1.204521481	0.990822419	NA
ASV_16s_13473	0.209503935	-0.370693329	3.550550782	1.118906598	0.99267582	NA

ASV_16s_13474	0.209256425	-0.386368564	3.550558016	1.099672729	0.993056558	NA
ASV_16s_13475	0.07292078	-0.304594841	3.551662066	0.375078246	NA	NA
ASV_16s_13476	0.295848338	-0.680197238	3.550239719	0.820510963	0.997228954	NA
ASV_16s_13477	0.266239705	-0.677484606	3.550400723	0.786452685	0.997579762	NA
ASV_16s_13478	0.27528602	-0.678993296	3.550349285	0.79734853	0.997470918	NA
ASV_16s_13479	0.274822375	-0.678935369	3.550351881	0.796801859	0.997476455	NA
ASV_16s_13480	0.216927021	-0.648640735	3.550667738	0.718068608	NA	NA
ASV_16s_13481	0.204266282	-0.637019655	3.550742085	0.697874219	NA	NA
ASV_16s_13482	0.204266282	-0.637019655	3.550742085	0.697874219	NA	NA
ASV_16s_13483	0.204266282	-0.637019655	3.550742085	0.697874219	NA	NA
ASV_16s_13484	0.204266282	-0.637019655	3.550742085	0.697874219	NA	NA
ASV_16s_13485	0.227775271	-0.657426849	3.550608744	0.734343535	NA	NA
ASV_16s_13486	0.065080543	-0.168919575	3.550914259	0.322000971	NA	NA
ASV_16s_13487	0.068621817	0.220251175	3.550997172	0.316872481	NA	NA
ASV_16s_13488	0.08895631	0.220178588	3.550901523	0.412330381	0.999708416	NA
ASV_16s_13489	0.090724527	0.220173895	3.550895219	0.420040996	NA	NA
ASV_16s_13490	0.216829364	-0.305142782	3.550740459	0.694765897	NA	NA
ASV_16s_13491	0.244355832	0.219067641	3.550624793	1.174653021	0.991498844	NA
ASV_16s_13492	0.216726747	-0.297554044	3.550881192	1.018687985	0.994521414	NA
ASV_16s_13493	0.236593234	0.231156073	3.550208709	1.291497924	0.988665404	NA
ASV_16s_13494	0.219939214	0.238708476	3.550188995	0.899483911	0.996291249	NA
ASV_16s_13495	0.251296365	-0.706978498	3.550240859	0.99244398	NA	NA
ASV_16s_13496	0.209800738	-0.076695713	3.551323571	0.968636526	NA	NA
ASV_16s_13497	0.194217617	0.117498112	3.550762046	1.18814012	0.99119741	NA
ASV_16s_13498	0.22837445	0.361170271	3.550375006	0.978953999	0.99516093	NA
ASV_16s_13499	0.263477612	-0.628149914	3.550440268	1.045153088	0.9940669	NA
ASV_16s_13500	0.222183771	0.312732716	3.550510634	1.07038484	0.993611843	NA
ASV_16s_13501	0.262978007	-0.712950673	3.550148876	1.056368841	0.993867264	NA
ASV_16s_13502	0.26928422	-0.705873604	3.55009066	1.092306361	0.993198986	NA
ASV_16s_13503	0.255672139	0.687486927	3.550932377	1.086693886	NA	NA
ASV_16s_13504	0.221488376	-0.596354205	3.550451779	0.722105502	NA	NA
ASV_16s_13505	0.263933291	-0.642560984	3.550142357	1.1558704	NA	NA
ASV_16s_13506	0.241209572	-0.627614596	3.550256775	1.086912507	NA	NA
ASV_16s_13507	0.274408769	-0.163992111	3.550110655	1.185984034	NA	NA
ASV_16s_13508	0.264631642	-0.650081552	3.550132731	1.155468447	0.991916346	NA
ASV_16s_13509	0.263199282	-0.617178983	3.550176507	1.172559911	0.991545036	NA
ASV_16s_13510	0.262949314	-0.590715862	3.55021791	1.182093205	0.99133337	NA
ASV_16s_13511	0.292289439	1.236957405	3.550054327	0.96647163	NA	NA
ASV_16s_13512	0.26555986	-0.554882953	3.550278662	1.19232664	NA	NA
ASV_16s_13513	0.274461639	-0.562486635	3.550243284	1.217320473	0.990522614	NA
ASV_16s_13514	0.263733254	-0.543490253	3.550303083	1.185540971	0.991256012	NA
ASV_16s_13515	0.259139964	0.373683685	3.550525765	0.997746177	0.994864812	NA
ASV_16s_13516	0.263793896	-0.477788342	3.550706874	1.047502928	NA	NA
ASV_16s_13517	0.255945905	-0.417048015	3.550500894	1.147751486	NA	NA
ASV_16s_13518	0.264261487	-0.431265381	3.550467005	1.167083556	NA	NA
ASV_16s_13519	0.329451546	0.362099803	3.550941968	1.223331302	0.99037974	NA
ASV_16s_13520	0.272232106	-0.942987595	3.550111478	0.950007455	NA	NA
ASV_16s_13521	0.235412432	-0.485114509	3.550519975	1.186251635	0.991240013	NA
ASV_16s_13522	0.270261241	0.037430786	3.550575551	1.120151589	0.992650732	NA
ASV_16s_13523	0.248925725	-0.243291665	3.551245939	0.751811969	NA	NA
ASV_16s_13524	0.261984512	1.062627034	3.550715623	0.617424955	0.99889292	NA
ASV_16s_13525	0.40944563	-0.591048814	3.549703198	0.952173362	NA	NA
ASV_16s_13526	0.24788611	0.165672127	3.550724317	1.029875729	0.994332095	NA
ASV_16s_13527	0.247706792	0.35284287	3.551331684	0.735755873	NA	NA
ASV_16s_13528	0.283651669	-0.105852798	3.550749787	1.168135647	0.991642157	NA
ASV_16s_13529	0.23479533	-0.07329775	3.550790101	1.051602695	0.993952614	NA
ASV_16s_13530	0.233953533	1.145905799	3.550647616	0.614844871	NA	NA
ASV_16s_13531	0.23829102	0.356359882	3.551173295	0.819659522	NA	NA
ASV_16s_13532	0.262667512	0.218824447	3.550284315	0.647403534	NA	NA
ASV_16s_13533	0.252180778	1.456601927	3.550430065	0.401210331	0.999733885	NA
ASV_16s_13534	0.266709385	0.160135168	3.551273522	0.24640115	0.999948715	NA
ASV_16s_13535	0.272084435	0.35195638	3.550367356	1.287808784	0.988762653	NA
ASV_16s_13536	0.156047805	-0.087016303	3.550739466	0.61483101	0.998908027	NA
ASV_16s_13537	0.272619167	0.402968948	3.55129505	-0.238633323	NA	NA
ASV_16s_13538	0.345631088	0.271666765	3.550631921	0.85080697	NA	NA
ASV_16s_13539	0.327869251	0.969627209	3.549578809	0.994302944	0.994919915	NA
ASV_16s_13540	0.233468205	1.256018395	3.550857613	0.692580313	NA	NA

ASV_16s_13541	0.233468205	1.256018395	3.550857613	0.692580313	NA	NA
ASV_16s_13542	0.233468205	1.256018395	3.550857613	0.692580313	NA	NA
ASV_16s_13543	0.260395245	0.354125298	3.551790195	0.427206916	NA	NA
ASV_16s_13544	0.267963789	0.391841703	3.551108706	0.769617258	NA	NA
ASV_16s_13545	0.288133675	0.472051832	3.550407853	1.101049698	NA	NA
ASV_16s_13546	0.232253148	0.383361373	3.551318603	0.721650207	NA	NA
ASV_16s_13547	0.310546302	0.067497746	3.551445898	0.702380166	NA	NA
ASV_16s_13548	0.24660162	-0.562809485	3.550318811	0.740183102	NA	NA
ASV_16s_13549	0.24660162	-0.562809485	3.550318811	0.740183102	NA	NA
ASV_16s_13550	0.17130724	-0.525149393	3.550902086	0.622366324	NA	NA
ASV_16s_13551	0.235134174	-0.553539298	3.550772414	0.748406587	NA	NA
ASV_16s_13552	0.132555536	1.115975281	3.551109591	0.440854362	NA	NA
ASV_16s_13553	0.28724598	0.418505993	3.550644012	0.722866587	NA	NA
ASV_16s_13554	0.196721558	-0.049196406	3.551008202	0.726001953	NA	NA
ASV_16s_13555	0.196498491	0.267905935	3.550874006	0.828746422	NA	NA
ASV_16s_13556	0.252875217	-0.145525949	3.549908478	1.277707704	0.989026287	NA
ASV_16s_13557	0.073577257	0.342971959	3.55177926	0.307038116	NA	NA
ASV_16s_13558	0.227437667	0.121785361	3.550304277	1.109742216	NA	NA
ASV_16s_13559	0.118724248	-0.454118546	3.551082957	0.440581636	NA	NA
ASV_16s_13560	0.144187877	-0.49978818	3.550823654	0.500149261	NA	NA
ASV_16s_13561	0.215201477	-0.078777051	3.550768519	0.973371389	0.995246731	NA
ASV_16s_13562	0.140702698	0.220088137	3.550782356	0.607946755	0.998947447	NA
ASV_16s_13563	0.144674688	0.220083868	3.550776719	0.620846945	0.998872777	NA
ASV_16s_13564	0.150192466	0.22007838	3.550769382	0.638353074	NA	NA
ASV_16s_13565	0.149892543	0.220078673	3.550769767	0.637414172	NA	NA
ASV_16s_13566	0.149892543	0.220078673	3.550769767	0.637414172	NA	NA
ASV_16s_13567	0.255754045	0.310937464	3.550843952	1.040877526	0.994141899	NA
ASV_16s_13568	0.158215911	0.596014472	3.550723585	0.597757872	NA	NA
ASV_16s_13569	0.320012551	0.100815873	3.550409632	0.721976332	NA	NA
ASV_16s_13570	0.210664718	-0.068793618	3.550563897	1.229634479	NA	NA
ASV_16s_13571	0.210664718	-0.068793618	3.550563897	1.229634479	NA	NA
ASV_16s_13572	0.261292258	1.569956388	3.550765056	0.407111801	0.999720568	NA
ASV_16s_13573	0.205267768	-0.071485512	3.551121953	0.980659375	NA	NA
ASV_16s_13574	0.251275285	1.770111608	3.550535739	0.302684451	0.999896901	NA
ASV_16s_13575	0.231154202	-0.068465979	3.550308815	1.335640502	NA	NA
ASV_16s_13576	0.168593262	0.319788607	3.551714479	0.352523437	0.999827581	NA
ASV_16s_13577	0.229389594	-0.546716415	3.550650298	0.759160657	NA	NA
ASV_16s_13578	0.204070974	-0.040548185	3.550381224	0.902240489	NA	NA
ASV_16s_13579	0.226917728	-0.067924826	3.550360906	1.280483174	NA	NA
ASV_16s_13580	0.191987626	0.040297677	3.550912231	0.925028478	NA	NA
ASV_16s_13581	0.16852313	0.289878883	3.551838087	0.413476349	NA	NA
ASV_16s_13582	0.197261786	0.604550387	3.551139792	0.709625869	0.998260678	NA
ASV_16s_13583	0.320300129	0.342446086	3.550699519	1.136479102	NA	NA
ASV_16s_13584	0.227528456	0.107083643	3.55100862	1.061144546	NA	NA
ASV_16s_13585	0.101001068	0.284416303	3.551743131	0.45615558	NA	NA
ASV_16s_13586	0.385622173	0.971027004	3.549028115	0.899030457	0.996297148	NA
ASV_16s_13587	0.261702532	0.180516842	3.550933563	0.861380923	0.996765921	NA
ASV_16s_13588	0.302665314	0.707653752	3.550810513	1.034806201	NA	NA
ASV_16s_13589	0.16930934	-0.07854802	3.550837089	0.856083523	NA	NA
ASV_16s_13590	0.16930934	-0.07854802	3.550837089	0.856083523	NA	NA
ASV_16s_13591	0.194627318	-0.117010421	3.55038662	1.112847501	NA	NA
ASV_16s_13592	0.220444785	-0.086271588	3.550645277	1.124595084	NA	NA
ASV_16s_13593	0.364354645	0.535744648	3.549992887	1.770004458	0.971467567	NA
ASV_16s_13594	0.223379942	-0.028893503	3.550882776	1.051054646	NA	NA
ASV_16s_13595	0.296815128	0.349040381	3.550200111	1.385991889	NA	NA
ASV_16s_13596	0.177082454	0.352696189	3.55070603	0.999829619	NA	NA
ASV_16s_13597	0.374233533	0.363377665	3.550435506	0.947409902	NA	NA
ASV_16s_13598	0.226521305	0.360337794	3.551041986	1.041621007	NA	NA
ASV_16s_13599	0.226521305	0.360337794	3.551041986	1.041621007	NA	NA
ASV_16s_13600	0.226521305	0.360337794	3.551041986	1.041621007	NA	NA
ASV_16s_13601	0.226521305	0.360337794	3.551041986	1.041621007	NA	NA
ASV_16s_13602	0.278541414	0.315939479	3.550845035	1.106761659	0.992917725	NA
ASV_16s_13603	0.250251125	0.315939508	3.55087404	1.052720583	0.993932664	NA
ASV_16s_13604	0.252884465	0.315939505	3.550871065	1.058004769	0.993837793	NA
ASV_16s_13605	0.250719686	0.315939507	3.550873505	1.05367075	0.993915674	NA
ASV_16s_13606	0.27927805	0.315939475	3.550844368	1.108036696	0.992892569	NA
ASV_16s_13607	0.243436732	0.315939516	3.550882029	1.038838171	0.994177457	NA

ASV_16s_13608	0.24206795	0.315939518	3.550883688	1.036002623	0.994226669	NA
ASV_16s_13609	0.296130626	0.315939468	3.550829796	1.137656474	0.992292233	NA
ASV_16s_13610	0.23659282	0.315939643	3.550890517	1.024510657	NA	NA
ASV_16s_13611	0.23659282	0.315939643	3.550890517	1.024510657	NA	NA
ASV_16s_13612	0.255250843	0.818832561	3.550737221	1.402012699	0.985509538	NA
ASV_16s_13613	0.225316104	0.687961372	3.550849054	0.91105823	NA	NA
ASV_16s_13614	0.312983228	0.734422302	3.550919452	1.141282353	NA	NA
ASV_16s_13615	0.312983228	0.734422302	3.550919452	1.141282353	NA	NA
ASV_16s_13616	0.155038015	0.733230299	3.551266031	0.796829006	NA	NA
ASV_16s_13617	0.155038015	0.733230299	3.551266031	0.796829006	NA	NA
ASV_16s_13618	0.155038015	0.733230299	3.551266031	0.796829006	NA	NA
ASV_16s_13619	0.155038015	0.733230299	3.551266031	0.796829006	NA	NA
ASV_16s_13620	0.294275479	0.693316935	3.550804334	1.069464752	0.993628814	NA
ASV_16s_13621	0.286420522	0.69329108	3.550811225	1.055787639	0.993877713	NA
ASV_16s_13622	0.264152826	0.693219032	3.550832956	1.015081126	0.994581574	NA
ASV_16s_13623	0.23659282	0.693107941	3.550865483	0.95997707	NA	NA
ASV_16s_13624	0.23659282	0.693107941	3.550865483	0.95997707	NA	NA
ASV_16s_13625	0.23659282	0.693107941	3.550865483	0.95997707	NA	NA
ASV_16s_13626	0.199901457	0.283759403	3.551545705	0.670979097	NA	NA
ASV_16s_13627	0.205079834	-0.147737571	3.550659568	1.18600529	0.991245561	NA
ASV_16s_13628	0.204832323	-0.161439319	3.550648617	1.1935169	0.991075404	NA
ASV_16s_13629	0.23838637	-0.217158063	3.550363485	1.31315417	0.988084092	NA
ASV_16s_13630	0.204078383	-0.174143185	3.550644118	1.197605138	0.990981934	NA
ASV_16s_13631	0.200032651	-0.180800179	3.550674423	1.185607672	0.991254511	NA
ASV_16s_13632	0.20585659	-0.191437372	3.550615658	1.209506094	0.990706372	NA
ASV_16s_13633	0.203071228	-0.186428013	3.550643206	1.197605004	0.990981938	NA
ASV_16s_13634	0.205355865	-0.204537291	3.550611232	1.208070746	0.990739882	NA
ASV_16s_13635	0.203076932	-0.200582337	3.550633254	1.199138115	0.990946729	NA
ASV_16s_13636	0.203082636	-0.214771394	3.550624753	1.198890218	0.990952428	NA
ASV_16s_13637	0.206374428	-0.22024114	3.550593901	1.211342394	0.990663393	NA
ASV_16s_13638	0.219278686	-0.240110721	3.550484045	1.247989734	0.989779646	NA
ASV_16s_13639	0.214383142	-0.246358267	3.550518706	1.234157612	0.990119043	NA
ASV_16s_13640	0.200055467	-0.238420243	3.55064001	1.184162382	0.991286995	NA
ASV_16s_13641	0.205125466	-0.26053009	3.550589038	1.197447692	0.990985545	NA
ASV_16s_13642	0.204112607	-0.259035608	3.550597986	1.193962856	0.991065238	NA
ASV_16s_13643	0.207151185	-0.263478822	3.550571445	1.204342716	0.990826564	NA
ASV_16s_13644	0.200066875	-0.267502034	3.550630989	1.171316739	0.991572397	NA
ASV_16s_13645	0.205643304	-0.290265211	3.550577822	1.179644463	0.991388052	NA
ASV_16s_13646	0.212886669	-0.299893934	3.550517946	1.20058768	0.99091336	NA
ASV_16s_13647	0.20412972	-0.303047607	3.550588865	1.16320257	0.991749618	NA
ASV_16s_13648	0.221157238	-0.3242063	3.550454461	1.210846973	0.990675	NA
ASV_16s_13649	0.204641853	-0.318764518	3.550583761	1.151954436	0.991991394	NA
ASV_16s_13650	0.213999021	-0.360036017	3.55051241	1.148152164	0.992072104	NA
ASV_16s_13651	0.210590844	-0.387703546	3.550547774	1.103012569	0.992991365	NA
ASV_16s_13652	0.200112508	-0.39287035	3.550636526	1.056208237	NA	NA
ASV_16s_13653	0.071462364	-0.297881962	3.551673039	0.369784567	NA	NA
ASV_16s_13654	0.071462364	-0.297881962	3.551673039	0.369784567	NA	NA
ASV_16s_13655	0.283073116	-0.679897884	3.550307033	0.806327832	0.997378838	NA
ASV_16s_13656	0.262064752	-0.676439402	3.55042479	0.781217718	0.997630941	NA
ASV_16s_13657	0.261601106	-0.67625505	3.550427212	0.78062881	0.997636653	NA
ASV_16s_13658	0.237544881	-0.66402381	3.550555317	0.748411708	0.997935486	NA
ASV_16s_13659	0.235196073	-0.662575983	3.550568399	0.745059494	NA	NA
ASV_16s_13660	0.203219534	-0.636006715	3.550748659	0.696120062	NA	NA
ASV_16s_13661	0.200180956	-0.633071836	3.550767822	0.691087615	NA	NA
ASV_16s_13662	0.200180956	-0.633071836	3.550767822	0.691087615	NA	NA
ASV_16s_13663	0.200180956	-0.633071836	3.550767822	0.691087615	NA	NA
ASV_16s_13664	0.056572381	-0.168678088	3.550975132	0.275903447	NA	NA
ASV_16s_13665	0.080953061	0.220202816	3.550933489	0.376288568	0.999785333	NA
ASV_16s_13666	0.088910036	0.220178819	3.5509017	0.412106566	NA	NA
ASV_16s_13667	0.088910036	0.220178819	3.5509017	0.412106566	NA	NA
ASV_16s_13668	0.288234568	-0.894328727	3.550410399	0.684498442	0.998452029	NA
ASV_16s_13669	0.207652586	0.006235182	3.550605841	1.126000477	0.992532143	NA
ASV_16s_13670	0.203181166	-0.342297231	3.55057609	0.707689499	NA	NA
ASV_16s_13671	0.21876602	0.285948449	3.550574228	1.174125588	0.991510499	NA
ASV_16s_13672	0.256697168	-0.734311576	3.550363936	0.869969938	0.996662603	NA
ASV_16s_13673	0.260837731	-0.7143425	3.550171969	1.023899587	0.994433734	NA
ASV_16s_13674	0.304979301	-0.550663197	3.550209494	1.256209039	0.989574595	NA

ASV_16s_13675	0.260758219	-0.715007434	3.550174556	1.029132931	NA	NA
ASV_16s_13676	0.24537644	-0.673031307	3.550236938	1.035939306	NA	NA
ASV_16s_13677	0.268128597	-0.694150895	3.550094336	1.109125049	0.992871052	NA
ASV_16s_13678	0.21364435	0.493177069	3.550554487	1.021810394	0.99446899	NA
ASV_16s_13679	0.274945631	-0.717462602	3.550063411	1.109038219	0.99287277	NA
ASV_16s_13680	0.261686734	-0.671670867	3.550134938	1.113795052	0.992778258	NA
ASV_16s_13681	0.256414176	-0.653311142	3.550166801	1.113505939	0.992784025	NA
ASV_16s_13682	0.252703273	-0.652590613	3.550185712	1.10460705	0.992960105	NA
ASV_16s_13683	0.217150067	-0.362575651	3.550685651	1.014572417	NA	NA
ASV_16s_13684	0.320280833	-0.157158064	3.549297054	1.206461228	NA	NA
ASV_16s_13685	0.262628721	-0.63191405	3.550159191	1.158175286	NA	NA
ASV_16s_13686	0.266318735	0.206688235	3.549662719	0.844827045	NA	NA
ASV_16s_13687	0.233850518	0.663053351	3.550874382	0.677316607	0.998504019	NA
ASV_16s_13688	0.258276265	-0.593968114	3.550226624	1.1615718	NA	NA
ASV_16s_13689	0.24409546	-0.508207765	3.550859785	0.949714641	0.99559944	NA
ASV_16s_13690	0.25895529	0.679112786	3.55091514	1.118471561	NA	NA
ASV_16s_13691	0.261122292	0.244961187	3.55012696	0.840648392	NA	NA
ASV_16s_13692	0.273669261	0.209376384	3.549781063	0.976401406	0.995200284	NA
ASV_16s_13693	0.264514995	0.386837461	3.550796136	1.195613383	0.991027548	NA
ASV_16s_13694	0.353523711	0.72980425	3.551005549	1.355220749	NA	NA
ASV_16s_13695	0.25922037	-0.43192192	3.550638751	0.901356159	0.996266828	NA
ASV_16s_13696	0.245487615	-0.504757523	3.550537079	1.00898129	0.994682352	NA
ASV_16s_13697	0.27036031	-0.429264364	3.550468931	1.182660799	0.991320664	NA
ASV_16s_13698	0.253627141	0.308798166	3.551047811	0.743163695	0.997981661	NA
ASV_16s_13699	0.243958905	-0.694257913	3.550461217	0.66194165	NA	NA
ASV_16s_13700	0.209150191	-0.154108294	3.551373502	0.576321022	0.999116277	NA
ASV_16s_13701	0.2179125	-0.083399404	3.550809231	0.975647895	0.995211862	NA
ASV_16s_13702	0.225672453	0.459994703	3.551064787	0.873366929	NA	NA
ASV_16s_13703	0.3358533	0.073509287	3.549415318	0.893648529	NA	NA
ASV_16s_13704	0.279661207	1.796154474	3.550224918	0.441993082	0.999632385	NA
ASV_16s_13705	0.234120448	1.552605146	3.550482651	0.559268746	0.999199228	NA
ASV_16s_13706	0.24937238	1.271655754	3.550789238	0.71554867	NA	NA
ASV_16s_13707	0.24937238	1.271655754	3.550789238	0.71554867	NA	NA
ASV_16s_13708	0.231298533	0.364398747	3.550745432	1.019012453	NA	NA
ASV_16s_13709	0.205315472	0.57406251	3.551108544	0.902846043	NA	NA
ASV_16s_13710	0.251031595	-0.537824108	3.550391777	0.892508495	0.996381322	NA
ASV_16s_13711	0.279765185	0.286616726	3.550526563	0.980916222	0.995130538	NA
ASV_16s_13712	0.255802652	0.400165728	3.55108407	0.587232534	0.999060265	NA
ASV_16s_13713	0.290451851	0.395889304	3.550979415	0.79626174	NA	NA
ASV_16s_13714	0.168097736	0.358247195	3.551702837	0.61290479	NA	NA
ASV_16s_13715	0.168097736	0.358247195	3.551702837	0.61290479	NA	NA
ASV_16s_13716	0.168097736	0.358247195	3.551702837	0.61290479	NA	NA
ASV_16s_13717	0.305526026	0.398403905	3.550902428	0.813018648	NA	NA
ASV_16s_13718	0.284454547	-0.565048993	3.550588599	0.899557349	0.996290293	NA
ASV_16s_13719	0.256621893	-0.568304235	3.550602793	0.799032627	NA	NA
ASV_16s_13720	0.167881095	-0.521999348	3.550936507	0.615854822	NA	NA
ASV_16s_13721	0.215854471	-1.080394174	3.550437022	0.373879881	NA	NA
ASV_16s_13722	0.246968585	-0.562818573	3.550316521	0.740621789	NA	NA
ASV_16s_13723	0.184432473	-0.982185152	3.550743037	0.347127199	NA	NA
ASV_16s_13724	0.242312766	1.657600932	3.550457603	0.428039008	NA	NA
ASV_16s_13725	0.248904987	1.288090767	3.550595495	0.653790555	NA	NA
ASV_16s_13726	0.222701948	1.264274383	3.550696519	0.615339642	NA	NA
ASV_16s_13727	0.263112781	0.415986836	3.550801423	0.696253954	NA	NA
ASV_16s_13728	0.231860963	0.406200557	3.550953824	0.653954903	NA	NA
ASV_16s_13729	0.191398488	0.389316535	3.551194557	0.59037314	NA	NA
ASV_16s_13730	0.191398488	0.389316535	3.551194557	0.59037314	NA	NA
ASV_16s_13731	0.191398488	0.389316535	3.551194557	0.59037314	NA	NA
ASV_16s_13732	0.191398488	0.389316535	3.551194557	0.59037314	NA	NA
ASV_16s_13733	0.182316081	0.296049653	3.551378719	0.335291721	NA	NA
ASV_16s_13734	0.34056252	0.333990843	3.549641861	2.020575332	0.958692791	NA
ASV_16s_13735	0.116349764	-0.449001695	3.55110596	0.434542837	NA	NA
ASV_16s_13736	0.116349764	-0.449001695	3.55110596	0.434542837	NA	NA
ASV_16s_13737	0.116349764	-0.449001695	3.55110596	0.434542837	NA	NA
ASV_16s_13738	0.270108438	0.27468248	3.549800192	1.291731864	0.98865922	NA
ASV_16s_13739	0.368168941	-0.548995687	3.549589962	0.79769411	NA	NA
ASV_16s_13740	0.183878691	-0.534562079	3.55046111	0.573509409	0.999130336	NA
ASV_16s_13741	0.14130412	-0.495917975	3.550853531	0.493872108	NA	NA

ASV_16s_13742	0.197710481	-0.078703873	3.550790924	0.931487262	NA	NA
ASV_16s_13743	0.203920983	-0.078730115	3.550782534	0.946702273	0.9956431	NA
ASV_16s_13744	0.208061318	-0.078748119	3.550777217	0.956628563	0.995498167	NA
ASV_16s_13745	0.210649027	-0.078759016	3.550774	0.96274077	0.995407396	NA
ASV_16s_13746	0.13664448	0.220092789	3.550788459	0.594455078	0.999021904	NA
ASV_16s_13747	0.139825581	0.220089139	3.550783651	0.605025458	0.998963881	NA
ASV_16s_13748	0.140885947	0.220087958	3.550782096	0.608512419	0.998944244	NA
ASV_16s_13749	0.141652843	0.220087123	3.550780986	0.611021423	0.998929961	NA
ASV_16s_13750	0.147188617	0.220081304	3.550773314	0.628853417	NA	NA
ASV_16s_13751	0.147188617	0.220081304	3.550773314	0.628853417	NA	NA
ASV_16s_13752	0.219044999	0.310940347	3.550886698	0.963033211	NA	NA
ASV_16s_13753	0.155051593	0.596005493	3.550727125	0.588414646	NA	NA
ASV_16s_13754	0.155051593	0.596005493	3.550727125	0.588414646	NA	NA
ASV_16s_13755	0.155051593	0.596005493	3.550727125	0.588414646	NA	NA
ASV_16s_13756	0.279478082	1.044885184	3.550213736	1.075111607	0.993524207	NA
ASV_16s_13757	0.202665664	-0.067979232	3.550712588	1.095418858	NA	NA
ASV_16s_13758	0.210428792	-0.06803008	3.550696408	1.118348044	0.992687058	NA
ASV_16s_13759	0.238336434	0.657904963	3.551722227	0.677435982	NA	NA
ASV_16s_13760	0.208116366	-0.068484364	3.550489681	1.241874416	NA	NA
ASV_16s_13761	0.183623288	1.399389226	3.55135946	0.346822382	0.999836786	NA
ASV_16s_13762	0.188749938	0.320710457	3.551023902	0.957407844	NA	NA
ASV_16s_13763	0.224335535	-0.06854516	3.550368105	1.316964544	0.987979959	NA
ASV_16s_13764	0.254014406	0.385125293	3.550270177	0.775933504	NA	NA
ASV_16s_13765	0.273200972	0.290440905	3.550023487	1.291766565	NA	NA
ASV_16s_13766	0.21554914	-0.05701477	3.550619686	1.171587466	NA	NA
ASV_16s_13767	0.203480411	-0.232403455	3.551295604	0.590943608	NA	NA
ASV_16s_13768	0.255698121	0.323841545	3.551135794	0.729813412	0.99809603	NA
ASV_16s_13769	0.222145043	-0.00941931	3.550915877	1.087361181	NA	NA
ASV_16s_13770	0.098981046	0.284611392	3.551748824	0.450050946	NA	NA
ASV_16s_13771	0.143354604	0.618797709	3.551586855	0.74116591	NA	NA
ASV_16s_13772	0.196487823	1.040383546	3.551124321	0.607757508	NA	NA
ASV_16s_13773	0.27180969	1.320380685	3.550173444	0.978678225	NA	NA
ASV_16s_13774	0.250019476	1.057195866	3.550435561	1.056250981	NA	NA
ASV_16s_13775	0.243705361	0.469121961	3.55088255	0.997442664	0.994869684	NA
ASV_16s_13776	0.165923153	-0.078526109	3.550843654	0.846340457	NA	NA
ASV_16s_13777	0.165923153	-0.078526109	3.550843654	0.846340457	NA	NA
ASV_16s_13778	0.196873554	-0.116574194	3.550299241	1.16257239	NA	NA
ASV_16s_13779	0.516131291	-0.029516834	3.550676974	1.474407289	NA	NA
ASV_16s_13780	0.314012614	0.581718301	3.550865151	1.567406163	NA	NA
ASV_16s_13781	0.310368089	0.651086011	3.550891116	1.371708674	NA	NA
ASV_16s_13782	0.343830506	0.801139349	3.550194138	1.492443338	NA	NA
ASV_16s_13783	0.283626277	0.342867166	3.550090923	1.443498201	NA	NA
ASV_16s_13784	0.182688656	-0.073532484	3.550843169	0.917031286	NA	NA
ASV_16s_13785	0.275542748	0.342663008	3.550107076	1.428905572	0.984669418	NA
ASV_16s_13786	0.275543763	0.375401014	3.550557528	1.259365562	NA	NA
ASV_16s_13787	0.24971823	0.360350483	3.551000596	1.090568581	NA	NA
ASV_16s_13788	0.24971823	0.360350483	3.551000596	1.090568581	NA	NA
ASV_16s_13789	0.24971823	0.360350483	3.551000596	1.090568581	NA	NA
ASV_16s_13790	0.266730996	0.327759197	3.550891581	0.95091948	0.995581899	NA
ASV_16s_13791	0.317886409	0.359868854	3.550708794	0.891176495	NA	NA
ASV_16s_13792	0.294098237	0.727368722	3.550981914	1.259199722	NA	NA
ASV_16s_13793	0.221990879	0.360335019	3.55105109	1.031462734	NA	NA
ASV_16s_13794	0.221990879	0.360335019	3.55105109	1.031462734	NA	NA
ASV_16s_13795	0.221990879	0.360335019	3.55105109	1.031462734	NA	NA
ASV_16s_13796	0.221990879	0.360335019	3.55105109	1.031462734	NA	NA
ASV_16s_13797	0.298077371	0.694242742	3.550921847	1.371370862	NA	NA
ASV_16s_13798	0.287632378	0.315939581	3.550836914	1.123038766	NA	NA
ASV_16s_13799	0.25690119	0.315939521	3.550866658	1.06590021	0.99369429	NA
ASV_16s_13800	0.245519269	0.315939535	3.550879553	1.043063951	0.994103622	NA
ASV_16s_13801	0.296005702	0.551245351	3.550797622	1.745619187	0.972570817	NA
ASV_16s_13802	0.239731463	0.315939543	3.550886574	1.03108571	0.994311373	NA
ASV_16s_13803	0.236993898	0.315939546	3.550890015	1.025317139	0.994409732	NA
ASV_16s_13804	0.236651702	0.315939547	3.550890451	1.024591647	0.994422024	NA
ASV_16s_13805	0.236309506	0.315939547	3.550890888	1.023865165	0.994434316	NA
ASV_16s_13806	0.248549689	0.315939531	3.550876013	1.049202388	0.993995309	NA
ASV_16s_13807	0.279044883	0.315939498	3.550844593	1.107566135	0.992901859	NA
ASV_16s_13808	0.231860963	0.315939661	3.550896687	1.014329489	NA	NA

ASV_16s_13809	0.231860963	0.315939661	3.550896687	1.014329489	NA	NA
ASV_16s_13810	0.228477949	0.715477868	3.55054219	1.166015917	NA	NA
ASV_16s_13811	0.263702279	1.032614615	3.549931388	0.549475897	NA	NA
ASV_16s_13812	0.398908315	0.688363457	3.550721146	1.198768034	NA	NA
ASV_16s_13813	0.384778057	1.155408941	3.55030409	0.905401301	NA	NA
ASV_16s_13814	0.261926381	0.856916898	3.550835551	0.893341894	0.996370636	NA
ASV_16s_13815	0.306723563	0.734398264	3.550926445	1.131024113	NA	NA
ASV_16s_13816	0.306723563	0.734398264	3.550926445	1.131024113	NA	NA
ASV_16s_13817	0.306723563	0.734398264	3.550926445	1.131024113	NA	NA
ASV_16s_13818	0.306723563	0.734398264	3.550926445	1.131024113	NA	NA
ASV_16s_13819	0.306723563	0.734398264	3.550926445	1.131024113	NA	NA
ASV_16s_13820	0.306723563	0.734398264	3.550926445	1.131024113	NA	NA
ASV_16s_13821	0.306723563	0.734398264	3.550926445	1.131024113	NA	NA
ASV_16s_13822	0.151937254	0.733011098	3.551279629	0.787503688	NA	NA
ASV_16s_13823	0.269374526	0.693235638	3.550827546	1.024877495	0.994417183	NA
ASV_16s_13824	0.293244613	0.693314041	3.550805225	1.067652691	NA	NA
ASV_16s_13825	0.268012968	0.693230851	3.550828942	1.022313026	0.994460521	NA
ASV_16s_13826	0.250651877	0.693167754	3.550848013	0.988725998	0.995008357	NA
ASV_16s_13827	0.246893694	0.693152456	3.550852492	0.981166642	0.99512665	NA
ASV_16s_13828	0.231860963	0.693086379	3.550871852	0.949886837	NA	NA
ASV_16s_13829	0.34061601	0.693434182	3.550770164	1.143384328	NA	NA
ASV_16s_13830	0.462075913	1.203349054	3.549889767	0.910720229	NA	NA
ASV_16s_13831	0.195821836	0.284835156	3.55156916	0.66400687	NA	NA
ASV_16s_13832	0.195821836	0.284835156	3.55156916	0.66400687	NA	NA
ASV_16s_13833	0.195924509	-0.113055367	3.550783311	1.132609583	0.992396698	NA
ASV_16s_13834	0.200741294	-0.13872372	3.550707648	1.161676214	0.991782692	NA
ASV_16s_13835	0.202115306	-0.156035272	3.55067769	1.175702615	0.991475621	NA
ASV_16s_13836	0.195947325	-0.172917186	3.550718628	1.168024231	0.991644593	NA
ASV_16s_13837	0.195947325	-0.172917186	3.550718628	1.168024231	0.991644593	NA
ASV_16s_13838	0.213080765	-0.217282025	3.550541338	1.228733139	0.990250206	NA
ASV_16s_13839	0.201776969	-0.198267945	3.550646272	1.193838553	0.991068072	NA
ASV_16s_13840	0.201776969	-0.198267945	3.550646272	1.193838553	0.991068072	NA
ASV_16s_13841	0.200004465	-0.195091802	3.550664155	1.186373824	0.99123726	NA
ASV_16s_13842	0.217535477	-0.237545445	3.550498033	1.242744382	0.989909188	NA
ASV_16s_13843	0.202294807	-0.227661443	3.550625168	1.194495589	0.991053084	NA
ASV_16s_13844	0.200021578	-0.238341992	3.550640475	1.183927165	0.991292275	NA
ASV_16s_13845	0.210348508	-0.267989787	3.550544694	1.211308515	0.990664187	NA
ASV_16s_13846	0.195981549	-0.261334237	3.550669391	1.157007383	0.99188334	NA
ASV_16s_13847	0.207757222	-0.278594994	3.55056292	1.194532521	0.991052241	NA
ASV_16s_13848	0.204618498	-0.303672214	3.550584803	1.164625169	0.991718718	NA
ASV_16s_13849	0.200550823	-0.298177242	3.550620434	1.151827415	0.991994098	NA
ASV_16s_13850	0.219523494	-0.351082183	3.550468828	1.17884178	0.991405929	NA
ASV_16s_13851	0.202610766	-0.395892829	3.550617891	1.062694808	0.993752803	NA
ASV_16s_13852	0.200095731	-0.444535255	3.550659225	0.989330425	0.99499882	NA
ASV_16s_13853	0.070003948	-0.291052633	3.551684332	0.364454204	NA	NA
ASV_16s_13854	0.070003948	-0.291052633	3.551684332	0.364454204	NA	NA
ASV_16s_13855	0.270993535	-0.678448381	3.550373852	0.792190617	0.997522836	NA
ASV_16s_13856	0.211544103	-0.643798879	3.550698776	0.709525138	NA	NA
ASV_16s_13857	0.20824994	-0.640754934	3.550718085	0.704286363	NA	NA
ASV_16s_13858	0.196095631	-0.628205708	3.550791419	0.684057343	NA	NA
ASV_16s_13859	0.196095631	-0.628205708	3.550791419	0.684057343	NA	NA
ASV_16s_13860	0.196095631	-0.628205708	3.550791419	0.684057343	NA	NA
ASV_16s_13861	0.062477322	-0.168852562	3.550931161	0.308119519	NA	NA
ASV_16s_13862	0.062477322	-0.168852562	3.550931161	0.308119519	NA	NA
ASV_16s_13863	0.055417843	-0.168639592	3.550984833	0.269460499	NA	NA
ASV_16s_13864	0.126930974	0.220105101	3.550804635	0.561144214	NA	NA
ASV_16s_13865	0.220096683	-0.156168841	3.55104687	0.93630536	0.995791638	NA
ASV_16s_13866	0.201381168	-0.373192732	3.550683398	0.688566122	NA	NA
ASV_16s_13867	0.309727652	1.525853257	3.550401873	0.579947963	0.999097916	NA
ASV_16s_13868	0.224487459	0.321449689	3.550447393	1.210086561	0.9906928	NA
ASV_16s_13869	0.261527542	-0.10407105	3.549841789	1.176141234	0.991465905	NA
ASV_16s_13870	0.203369874	-0.024338455	3.551366901	0.859941554	0.996783028	NA
ASV_16s_13871	0.249838423	-0.711915632	3.550268667	0.957623063	0.995483477	NA
ASV_16s_13872	0.251915261	-0.707701432	3.550237434	0.986580457	0.995042117	NA
ASV_16s_13873	0.203111582	-0.460339633	3.550896303	0.987175692	0.995032766	NA
ASV_16s_13874	0.260379402	0.117094736	3.549809173	0.784053009	0.997603312	NA
ASV_16s_13875	0.276395381	-0.724103743	3.550062255	1.086358523	0.993312627	NA

ASV_16s_13876	0.268706246	-0.688145603	3.550093054	1.119730708	0.992659219	NA
ASV_16s_13877	0.263433392	-1.041240047	3.550174417	0.594201282	0.999023269	NA
ASV_16s_13878	0.268971051	-0.679901743	3.550097501	1.136612219	0.992313921	NA
ASV_16s_13879	0.246005696	-0.640643758	3.550225241	1.082908641	0.993377986	NA
ASV_16s_13880	0.276752293	-0.454056065	3.550518086	1.108260726	NA	NA
ASV_16s_13881	0.236388803	-0.938361619	3.550309692	0.572890174	NA	NA
ASV_16s_13882	0.267741308	-0.632046369	3.550141302	1.173647987	0.991521044	NA
ASV_16s_13883	0.229741208	-0.571868932	3.550364817	1.072240581	NA	NA
ASV_16s_13884	0.253933435	-0.604489302	3.550225769	1.138914849	0.992266046	NA
ASV_16s_13885	0.246645759	-0.556495171	3.550326316	1.132131231	0.992406552	NA
ASV_16s_13886	0.250603333	-0.579437968	3.550510811	0.891319252	0.996396535	NA
ASV_16s_13887	0.129453113	-0.824089635	3.550590968	0.321881875	0.999873088	NA
ASV_16s_13888	0.255662106	-0.436870835	3.550474409	1.143730479	0.992165316	NA
ASV_16s_13889	0.245424507	0.063234039	3.550649587	1.064768008	0.993714998	NA
ASV_16s_13890	0.246655788	0.221065123	3.549494027	1.214037701	0.990600083	NA
ASV_16s_13891	0.243471217	0.678507086	3.550949517	1.038341422	0.994186098	NA
ASV_16s_13892	0.251335376	1.213505583	3.550903122	0.690325785	NA	NA
ASV_16s_13893	0.22786911	-0.103189105	3.550796744	1.05437688	NA	NA
ASV_16s_13894	0.238518158	-0.426588418	3.550664811	0.986237277	0.995047503	NA
ASV_16s_13895	0.222409315	-0.168377267	3.550703072	1.349504981	0.987067928	NA
ASV_16s_13896	0.199695515	-0.306033249	3.550556414	0.823282088	0.997199028	NA
ASV_16s_13897	0.238910223	-0.068414104	3.551051439	0.867074045	NA	NA
ASV_16s_13898	0.263396544	-0.145296897	3.5511232	0.71067121	0.998252394	NA
ASV_16s_13899	0.175527897	0.868886379	3.551000562	0.390351087	0.999757238	NA
ASV_16s_13900	0.301840111	0.758940593	3.549802148	0.738677198	0.998020589	NA
ASV_16s_13901	0.246753944	-0.110787545	3.550755005	1.050784876	0.993967183	NA
ASV_16s_13902	0.218205116	-0.07215778	3.550820244	1.012046	NA	NA
ASV_16s_13903	0.277020141	-0.133251563	3.550360335	0.840518059	0.99700813	NA
ASV_16s_13904	0.228743405	1.576975464	3.550698348	0.411635968	NA	NA
ASV_16s_13905	0.239521747	0.292376458	3.551249757	0.648463766	NA	NA
ASV_16s_13906	0.217097069	1.237543058	3.550931482	0.667148903	NA	NA
ASV_16s_13907	0.212490145	0.3778412	3.551435794	0.691529889	NA	NA
ASV_16s_13908	0.26520162	0.391105907	3.551124167	0.766071365	NA	NA
ASV_16s_13909	0.323611434	0.401253868	3.550819228	0.831973007	0.997103802	NA
ASV_16s_13910	0.16466717	0.356483468	3.551722491	0.605995672	NA	NA
ASV_16s_13911	0.192330803	-0.995106257	3.550730162	0.374249801	NA	NA
ASV_16s_13912	0.243825293	1.283802246	3.550613928	0.646631061	NA	NA
ASV_16s_13913	0.155443116	1.056156082	3.55062164	0.311467817	NA	NA
ASV_16s_13914	0.25015348	0.412622588	3.550865693	0.679409531	NA	NA
ASV_16s_13915	0.227129107	0.404375633	3.550978061	0.647004745	NA	NA
ASV_16s_13916	0.236261649	0.785316933	3.550429097	1.155776668	0.991909742	NA
ASV_16s_13917	0.187492397	0.387456325	3.551222345	0.583620226	NA	NA
ASV_16s_13918	0.261389772	-0.07370117	3.550675365	1.057414684	NA	NA
ASV_16s_13919	0.070634167	0.342797287	3.551788638	0.297167064	NA	NA
ASV_16s_13920	0.229235544	-0.072024256	3.550760686	0.956440985	0.995500934	NA
ASV_16s_13921	0.113975279	-0.443724304	3.551128012	0.428405986	NA	NA
ASV_16s_13922	0.113975279	-0.443724304	3.551128012	0.428405986	NA	NA
ASV_16s_13923	0.138420362	-0.49179157	3.550884035	0.4874729	NA	NA
ASV_16s_13924	0.126859283	-0.471144464	3.551003592	0.46054357	NA	NA
ASV_16s_13925	0.126859283	-0.471144464	3.551003592	0.46054357	NA	NA
ASV_16s_13926	0.100566041	1.025059717	3.551323499	0.358354432	NA	NA
ASV_16s_13927	0.199251162	-0.078709377	3.550788804	0.93525182	0.995806504	NA
ASV_16s_13928	0.136474274	0.220093012	3.55078873	0.593848028	0.999025168	NA
ASV_16s_13929	0.137888096	0.220091365	3.55078656	0.598566095	0.998999605	NA
ASV_16s_13930	0.144184768	0.22008443	3.55077741	0.619200035	NA	NA
ASV_16s_13931	0.146726786	0.351374505	3.55074885	0.983646467	0.995088045	NA
ASV_16s_13932	0.151887274	0.595994978	3.550730813	0.578932176	NA	NA
ASV_16s_13933	0.151887274	0.595994978	3.550730813	0.578932176	NA	NA
ASV_16s_13934	0.171266331	0.351169908	3.551888937	0.526336129	NA	NA
ASV_16s_13935	0.229932909	0.09823776	3.550859101	1.254998645	0.98960495	NA
ASV_16s_13936	0.271743654	0.331104238	3.550106731	1.503085545	0.982202646	NA
ASV_16s_13937	0.198560051	-0.343056578	3.5506519	0.989575968	NA	NA
ASV_16s_13938	0.300506483	1.760477417	3.550209793	0.471842981	NA	NA
ASV_16s_13939	0.205072494	-0.068309737	3.550470876	1.238182331	NA	NA
ASV_16s_13940	0.229684168	-0.061822686	3.550877078	0.964826354	NA	NA
ASV_16s_13941	0.205568013	-0.068241384	3.550458634	1.242071913	NA	NA
ASV_16s_13942	0.206063531	-0.068166954	3.550451061	1.242239027	NA	NA

ASV_16s_13943	0.207054568	-0.067996482	3.550449811	1.239057056	NA	NA
ASV_16s_13944	0.185398241	-0.545639114	3.550474625	0.648683259	NA	NA
ASV_16s_13945	0.300335171	-0.503097877	3.55016714	0.87238899	NA	NA
ASV_16s_13946	0.193748946	0.036269702	3.550447725	1.397211728	0.985656513	NA
ASV_16s_13947	0.233945362	0.309633752	3.550818859	0.71917379	NA	NA
ASV_16s_13948	0.268569089	0.341108385	3.551035954	0.685614898	0.99844384	NA
ASV_16s_13949	0.335140702	0.2649961	3.550943258	0.963827869	0.995391129	NA
ASV_16s_13950	0.228736703	-0.177747292	3.550422496	0.952379978	NA	NA
ASV_16s_13951	0.217658664	-0.041669229	3.550783228	1.115976967	NA	NA
ASV_16s_13952	0.257245237	-0.547419208	3.549975984	0.683086849	NA	NA
ASV_16s_13953	0.257245237	-0.547419208	3.549975984	0.683086849	NA	NA
ASV_16s_13954	0.256467808	1.073233445	3.55065855	1.114644142	NA	NA
ASV_16s_13955	0.162536966	-0.078503273	3.550850491	0.836433184	NA	NA
ASV_16s_13956	0.188104688	-0.116752506	3.550356184	1.114481697	NA	NA
ASV_16s_13957	0.335322075	0.737709063	3.55020461	1.863047645	0.967029893	NA
ASV_16s_13958	0.342922186	0.525471072	3.550838323	1.374305906	0.986345283	NA
ASV_16s_13959	0.276121419	0.550098955	3.550777637	1.104056748	0.992970904	NA
ASV_16s_13960	0.298027348	0.36501347	3.550360832	1.346258152	0.987160764	NA
ASV_16s_13961	0.214444744	-0.028848342	3.550897864	1.030696331	NA	NA
ASV_16s_13962	0.214444744	-0.028848342	3.550897864	1.030696331	NA	NA
ASV_16s_13963	0.199659566	-0.471001118	3.550652814	0.735275114	NA	NA
ASV_16s_13964	0.178960316	-0.073508493	3.550850012	0.906968541	NA	NA
ASV_16s_13965	0.227711031	0.336056758	3.550302034	1.276813355	NA	NA
ASV_16s_13966	0.248497565	0.347349612	3.550299433	1.317496437	NA	NA
ASV_16s_13967	0.27642859	0.354575408	3.550300795	1.321821724	NA	NA
ASV_16s_13968	0.244621939	0.360347907	3.551009038	1.080139968	NA	NA
ASV_16s_13969	0.235511089	0.354172578	3.551073229	0.773899039	NA	NA
ASV_16s_13970	0.232595617	0.343056891	3.551035887	0.781750449	NA	NA
ASV_16s_13971	0.401703948	0.360397502	3.550847143	1.331301944	NA	NA
ASV_16s_13972	0.217460453	0.360332129	3.55106057	1.021111655	NA	NA
ASV_16s_13973	0.217460453	0.360332129	3.55106057	1.021111655	NA	NA
ASV_16s_13974	0.217460453	0.360332129	3.55106057	1.021111655	NA	NA
ASV_16s_13975	0.268008292	0.764312244	3.550370486	1.365942351	NA	NA
ASV_16s_13976	0.243399029	0.495109814	3.550971379	1.618333362	0.977928021	NA
ASV_16s_13977	0.257860295	0.315939539	3.550865633	1.067737777	0.99366059	NA
ASV_16s_13978	0.24774293	0.315939551	3.55087695	1.047555528	0.994024492	NA
ASV_16s_13979	0.241925605	0.315939559	3.550883886	1.035601951	0.994233602	NA
ASV_16s_13980	0.23658422	0.315939567	3.550890549	1.024396219	0.994425333	NA
ASV_16s_13981	0.236026193	0.315939568	3.550891261	1.023218753	0.994445239	NA
ASV_16s_13982	0.319083613	0.315939485	3.550812415	1.175417289	0.991481938	NA
ASV_16s_13983	0.233630824	0.315939571	3.550894364	1.018098699	0.994531272	NA
ASV_16s_13984	0.233288628	0.315939572	3.550894812	1.01736322	0.994543559	NA
ASV_16s_13985	0.262182192	0.315939534	3.550861088	1.075903962	0.993509442	NA
ASV_16s_13986	0.260572393	0.315939536	3.550862764	1.072940628	0.993564552	NA
ASV_16s_13987	0.227129107	0.315939679	3.550903113	1.003947589	NA	NA
ASV_16s_13988	0.277247552	0.315939518	3.550846278	1.104129587	0.992969475	NA
ASV_16s_13989	0.301423675	1.120814475	3.550554262	0.843204824	NA	NA
ASV_16s_13990	0.315253007	0.734429431	3.550917012	1.144838036	0.992142033	NA
ASV_16s_13991	0.16778992	0.733407395	3.551214225	0.833025695	0.997092125	NA
ASV_16s_13992	0.300463899	0.734373222	3.550933729	1.12055997	NA	NA
ASV_16s_13993	0.300463899	0.734373222	3.550933729	1.12055997	NA	NA
ASV_16s_13994	0.300463899	0.734373222	3.550933729	1.12055997	NA	NA
ASV_16s_13995	0.300463899	0.734373222	3.550933729	1.12055997	NA	NA
ASV_16s_13996	0.300463899	0.734373222	3.550933729	1.12055997	NA	NA
ASV_16s_13997	0.300463899	0.734373222	3.550933729	1.12055997	NA	NA
ASV_16s_13998	0.300463899	0.734373222	3.550933729	1.12055997	NA	NA
ASV_16s_13999	0.260844819	0.693205399	3.550836516	1.00867788	0.994687333	NA
ASV_16s_14000	0.283131931	0.693281759	3.55081423	1.049917216	0.993982615	NA
ASV_16s_14001	0.273256097	0.6932482	3.550823669	1.031992295	0.994295815	NA
ASV_16s_14002	0.287260029	0.693296198	3.550810482	1.057211881	NA	NA
ASV_16s_14003	0.287260029	0.693296198	3.550810482	1.057211881	NA	NA
ASV_16s_14004	0.309495583	0.693358	3.550792002	1.09483737	0.99315026	NA
ASV_16s_14005	0.452449331	1.203254188	3.549912819	0.903906561	NA	NA
ASV_16s_14006	0.206742408	0.018513536	3.550905141	0.996875395	NA	NA
ASV_16s_14007	0.19484924	-0.028354817	3.550917425	1.042438597	NA	NA
ASV_16s_14008	0.195367078	-0.063789601	3.550855499	1.080157259	NA	NA
ASV_16s_14009	0.191844887	-0.118161831	3.550812301	1.118949495	0.992674956	NA

ASV_16s_14010	0.196402753	-0.129258182	3.550758889	1.136686358	0.992312383	NA
ASV_16s_14011	0.191850591	-0.133766712	3.550794522	1.12971654	0.992456176	NA
ASV_16s_14012	0.196661672	-0.14476948	3.550739113	1.147527422	0.992085316	NA
ASV_16s_14013	0.196661672	-0.14476948	3.550739113	1.147527422	0.992085316	NA
ASV_16s_14014	0.191861999	-0.164443279	3.55076407	1.147775381	0.992080074	NA
ASV_16s_14015	0.191861999	-0.164443279	3.55076407	1.147775381	0.992080074	NA
ASV_16s_14016	0.200573536	-0.181740562	3.550669168	1.176884556	0.991449423	NA
ASV_16s_14017	0.197438428	-0.190371042	3.550690967	1.172792394	0.991539914	NA
ASV_16s_14018	0.208289326	-0.237141648	3.550570771	1.209575581	0.990704748	NA
ASV_16s_14019	0.198215185	-0.235396456	3.550657977	1.177139124	0.991443774	NA
ASV_16s_14020	0.210098796	-0.267614236	3.550546893	1.209001475	0.990718162	NA
ASV_16s_14021	0.198733023	-0.265476925	3.550643558	1.166516252	0.99167753	NA
ASV_16s_14022	0.198991942	-0.280819574	3.550637416	1.157786965	0.991866589	NA
ASV_16s_14023	0.195199424	-0.29061019	3.550669964	1.13437104	0.992360339	NA
ASV_16s_14024	0.19925086	-0.296356959	3.550632297	1.147574331	0.992084324	NA
ASV_16s_14025	0.195452639	-0.290975529	3.550667559	1.135206217	0.992343062	NA
ASV_16s_14026	0.195959068	-0.291703697	3.55066277	1.136872174	0.992308526	NA
ASV_16s_14027	0.196971927	-0.293150093	3.550653274	1.140186643	0.992239523	NA
ASV_16s_14028	0.211153667	-0.341691618	3.550532077	1.152544295	0.991978827	NA
ASV_16s_14029	0.227026738	-0.388143664	3.550424349	1.16172618	0.99178161	NA
ASV_16s_14030	0.270651522	-0.678402992	3.550376023	0.79172108	0.997527527	NA
ASV_16s_14031	0.249542035	-0.671130779	3.550492497	0.764857229	0.997786276	NA
ASV_16s_14032	0.256675029	-0.674259121	3.550453656	0.77423607	0.997698079	NA
ASV_16s_14033	0.233342433	-0.661415931	3.55057898	0.742362006	0.997988654	NA
ASV_16s_14034	0.221994254	-0.652761817	3.550639805	0.725656561	NA	NA
ASV_16s_14035	0.197074601	-0.629458782	3.550786047	0.685730326	0.998442992	NA
ASV_16s_14036	0.192010305	-0.622359867	3.55081269	0.676762194	NA	NA
ASV_16s_14037	0.192010305	-0.622359867	3.55081269	0.676762194	NA	NA
ASV_16s_14038	0.192010305	-0.622359867	3.55081269	0.676762194	NA	NA
ASV_16s_14039	0.192010305	-0.622359867	3.55081269	0.676762194	NA	NA
ASV_16s_14040	0.061175711	-0.168816911	3.550940148	0.301083315	NA	NA
ASV_16s_14042	0.217838475	-0.041335387	3.550510793	1.164857819	0.991713658	NA
ASV_16s_14043	0.258470082	-0.664360384	3.550595282	0.7548763	0.997877653	NA
ASV_16s_14044	0.205030245	-0.413056021	3.550733385	0.622821296	0.998861045	NA
ASV_16s_14045	0.085254437	-0.147026425	3.551226172	0.244743617	0.99994988	NA
ASV_16s_14046	0.208638663	-0.00920376	3.550470634	1.097092182	0.993106665	NA
ASV_16s_14047	0.223199851	-0.661668779	3.550657808	0.685607597	0.998443894	NA
ASV_16s_14048	0.217530244	0.185812899	3.550584116	1.009961532	0.994666239	NA
ASV_16s_14049	0.212585173	0.755203648	3.551578376	0.702520796	NA	NA
ASV_16s_14050	0.198229382	0.816415799	3.551618873	0.678036392	NA	NA
ASV_16s_14051	0.19744224	0.064035078	3.550462937	1.083074738	0.993374849	NA
ASV_16s_14052	0.259548125	-0.731147735	3.550243659	0.950255706	0.995591568	NA
ASV_16s_14053	0.197163696	0.384445377	3.550805532	1.021293123	0.994477696	NA
ASV_16s_14054	0.2152608	0.157941551	3.550349259	1.210034169	0.990694025	NA
ASV_16s_14055	0.270779648	-0.774719686	3.550381449	0.866776562	0.996701264	NA
ASV_16s_14056	0.242617822	-0.670017954	3.550355005	0.701763583	NA	NA
ASV_16s_14057	0.180217657	0.050393247	3.550884535	1.089471505	0.993253301	NA
ASV_16s_14058	0.216421431	0.15385712	3.550312295	1.185521133	0.991256458	NA
ASV_16s_14059	0.202354797	-0.190112333	3.550412702	0.905575139	NA	NA
ASV_16s_14060	0.230543076	0.313029232	3.551996197	0.44934405	NA	NA
ASV_16s_14061	0.230982988	0.723831239	3.551312678	1.051434858	NA	NA
ASV_16s_14062	0.229554705	0.335316896	3.550449534	1.188883195	0.991180611	NA
ASV_16s_14063	0.237157135	-0.355792617	3.550565924	1.010427479	0.994658568	NA
ASV_16s_14064	0.199959369	-0.240931908	3.551169663	0.966535165	0.995350458	NA
ASV_16s_14065	0.227905607	0.384988791	3.550681939	1.194970421	0.991042242	NA
ASV_16s_14066	0.240769539	0.253871266	3.550190148	1.261340163	0.989445306	NA
ASV_16s_14067	0.171186127	0.66047429	3.551661616	0.846123191	NA	NA
ASV_16s_14068	0.255676024	-0.614575175	3.550206289	1.135913463	NA	NA
ASV_16s_14069	0.203879949	0.676313884	3.551073107	0.938968116	NA	NA
ASV_16s_14070	0.249889269	-0.578620952	3.550278014	1.131007772	NA	NA
ASV_16s_14071	0.254449516	-0.58289522	3.550256514	1.146477725	NA	NA
ASV_16s_14072	0.232833068	0.021203321	3.55004796	0.83815047	0.997034842	NA
ASV_16s_14073	0.250080163	-0.516670577	3.550381892	1.14167807	0.992208347	NA
ASV_16s_14074	0.248503455	-0.082399955	3.55122177	0.816744478	0.997269292	NA
ASV_16s_14075	0.266181354	-0.50329911	3.550383509	1.209575083	NA	NA
ASV_16s_14076	0.244648865	-0.413871285	3.550526526	1.109970007	0.992854319	NA
ASV_16s_14077	0.18793321	-0.617364212	3.5507453	0.603821011	NA	NA

ASV_16s_14078	0.235908875	0.017700168	3.550463524	1.159653557	0.991826391	NA
ASV_16s_14079	0.231981806	-0.056432846	3.551100173	0.829741814	NA	NA
ASV_16s_14080	0.227656324	0.664340249	3.550220971	0.976355405	NA	NA
ASV_16s_14081	0.240413065	-0.056106869	3.550782576	1.026052696	0.994397251	NA
ASV_16s_14082	0.180398031	-0.2866584	3.550866845	0.723026209	NA	NA
ASV_16s_14083	0.242662631	1.38543733	3.550962287	0.63053182	0.998814439	NA
ASV_16s_14084	0.212574214	1.231540551	3.550950876	0.659716782	NA	NA
ASV_16s_14085	0.265389812	1.614132371	3.550680369	0.513247192	0.999396515	NA
ASV_16s_14086	0.239193916	1.262056069	3.550833878	0.700949101	NA	NA
ASV_16s_14087	0.235835458	1.057885598	3.550487481	0.775214914	0.997688742	NA
ASV_16s_14088	0.198955306	0.364892503	3.551136971	0.839057201	0.99702463	NA
ASV_16s_14089	0.251885962	0.387497118	3.55120203	0.748795396	NA	NA
ASV_16s_14090	0.288083928	-0.709279916	3.550304712	0.90335693	0.996240615	NA
ASV_16s_14091	0.187200136	-0.363927708	3.551211013	0.549808383	0.999242895	NA
ASV_16s_14092	0.161236604	0.354684331	3.551742824	0.59897325	NA	NA
ASV_16s_14093	0.269780856	-0.563361266	3.55018003	0.76861905	NA	NA
ASV_16s_14094	0.284420578	0.351994781	3.549437761	0.879155556	NA	NA
ASV_16s_14095	0.163570034	-0.889839808	3.550894249	0.32439049	NA	NA
ASV_16s_14096	0.219078717	-1.017338021	3.550564699	0.439361027	0.999639625	NA
ASV_16s_14097	0.213554329	-0.553403473	3.550544247	0.693626337	NA	NA
ASV_16s_14098	0.23339804	1.274500066	3.550653463	0.631496043	NA	NA
ASV_16s_14099	0.124602204	1.095421213	3.551154731	0.421275968	NA	NA
ASV_16s_14100	0.124602204	1.095421213	3.551154731	0.421275968	NA	NA
ASV_16s_14101	0.213612072	1.254446412	3.550733029	0.600865614	NA	NA
ASV_16s_14102	0.294414943	1.320836844	3.550452384	0.711525163	NA	NA
ASV_16s_14103	0.279477583	0.002775912	3.549743162	1.205665301	NA	NA
ASV_16s_14104	0.222397251	0.402513075	3.551003227	0.639920157	NA	NA
ASV_16s_14105	0.161105594	0.011474417	3.550686318	0.914142368	0.996097251	NA
ASV_16s_14106	0.183586305	0.385262046	3.551248094	0.576679687	NA	NA
ASV_16s_14107	0.195972777	-0.071873287	3.550816034	0.819228216	NA	NA
ASV_16s_14108	0.069162622	0.342708574	3.551793465	0.292155621	NA	NA
ASV_16s_14109	0.122583322	0.227872929	3.550913466	0.361161579	NA	NA
ASV_16s_14110	0.111600794	-0.438370467	3.551149586	0.422198453	NA	NA
ASV_16s_14111	0.111600794	-0.438370467	3.551149586	0.422198453	NA	NA
ASV_16s_14112	0.111600794	-0.438370467	3.551149586	0.422198453	NA	NA
ASV_16s_14113	0.111600794	-0.438370467	3.551149586	0.422198453	NA	NA
ASV_16s_14114	0.265462041	0.747105031	3.550026732	1.064970675	NA	NA
ASV_16s_14115	0.135536604	-0.486922007	3.550912835	0.480894671	NA	NA
ASV_16s_14116	0.135536604	-0.486922007	3.550912835	0.480894671	NA	NA
ASV_16s_14117	0.135536604	-0.486922007	3.550912835	0.480894671	NA	NA
ASV_16s_14118	0.124216382	-0.465640572	3.551029924	0.454097524	NA	NA
ASV_16s_14119	0.124216382	-0.465640572	3.551029924	0.454097524	NA	NA
ASV_16s_14120	0.124216382	-0.465640572	3.551029924	0.454097524	NA	NA
ASV_16s_14121	0.222397251	-0.07880604	3.550760346	0.989579203	NA	NA
ASV_16s_14122	0.222397251	-0.07880604	3.550760346	0.989579203	NA	NA
ASV_16s_14123	0.189640665	-0.078664599	3.550802663	0.910983779	NA	NA
ASV_16s_14124	0.189640665	-0.078664599	3.550802663	0.910983779	NA	NA
ASV_16s_14125	0.124286578	0.220108795	3.550809481	0.551743452	NA	NA
ASV_16s_14126	0.130295323	0.220100652	3.550798772	0.572808334	0.999133817	NA
ASV_16s_14127	0.133829879	0.220096208	3.550792919	0.584886658	0.999072503	NA
ASV_16s_14128	0.135315683	0.22009427	3.550790548	0.589915935	0.999046133	NA
ASV_16s_14129	0.141180918	0.220087687	3.550781681	0.609407699	NA	NA
ASV_16s_14130	0.141180918	0.220087687	3.550781681	0.609407699	NA	NA
ASV_16s_14131	0.141180918	0.220087687	3.550781681	0.609407699	NA	NA
ASV_16s_14132	0.232893061	0.310939138	3.55086902	0.993640767	0.994930468	NA
ASV_16s_14133	0.217032795	1.490999362	3.550795907	0.480093058	NA	NA
ASV_16s_14134	0.19410033	-0.068185623	3.550743587	1.051267814	NA	NA
ASV_16s_14135	0.282985708	-0.11807429	3.550667958	0.974720757	0.995226083	NA
ASV_16s_14136	0.181321294	0.632711311	3.550459088	0.879480152	NA	NA
ASV_16s_14137	0.228969569	0.243065055	3.550383504	0.719793095	NA	NA
ASV_16s_14138	0.219174366	-0.068226134	3.550579461	1.236437667	NA	NA
ASV_16s_14139	0.198064477	-0.068750449	3.55062646	1.175136124	NA	NA
ASV_16s_14140	0.242884709	-0.117303973	3.550093021	1.319604655	NA	NA
ASV_16s_14141	0.199055513	-0.068493963	3.550578556	1.194849907	NA	NA
ASV_16s_14142	0.207692616	0.739926412	3.55144297	0.928039801	0.99590736	NA
ASV_16s_14143	0.235252949	0.658747517	3.551109346	0.789430734	0.997550326	NA
ASV_16s_14144	0.199551032	-0.068437665	3.550549742	1.202905983	NA	NA

ASV_16s_14145	0.214467813	0.32401577	3.551830976	0.557858742	NA	NA
ASV_16s_14146	0.200542068	-0.06830779	3.550508521	1.216097882	NA	NA
ASV_16s_14147	0.231035941	1.532004393	3.550835757	0.416020896	0.999699607	NA
ASV_16s_14148	0.325945357	0.084984954	3.550910651	0.845046207	NA	NA
ASV_16s_14149	0.213635788	0.202612463	3.5502965	0.879441888	0.996546197	NA
ASV_16s_14150	0.337536343	0.300605531	3.551845653	0.55517737	0.999218317	NA
ASV_16s_14151	0.218008234	1.022751743	3.55073356	1.042027218	NA	NA
ASV_16s_14152	0.204010697	-0.067635894	3.550506951	1.199437256	NA	NA
ASV_16s_14153	0.21332645	-0.067916315	3.550422905	1.235061507	NA	NA
ASV_16s_14154	0.28284412	0.386266898	3.549974549	1.322607136	0.987824732	NA
ASV_16s_14155	0.214066101	0.150292517	3.550478963	1.179805643	NA	NA
ASV_16s_14156	0.247887766	0.317392224	3.550941721	0.767752176	0.997759295	NA
ASV_16s_14157	0.178321062	-0.413704438	3.550739344	0.697293865	NA	NA
ASV_16s_14158	0.231445464	0.320807851	3.551054473	0.80573301	0.997385005	NA
ASV_16s_14159	0.24307212	0.556853008	3.550876012	1.353851886	0.986942997	NA
ASV_16s_14160	0.167228557	0.279429497	3.551614825	0.624367519	NA	NA
ASV_16s_14161	0.159878123	0.279884856	3.551625239	0.608327116	NA	NA
ASV_16s_14162	0.133129966	0.281716157	3.551669716	0.544665208	NA	NA
ASV_16s_14163	0.161577426	-0.478501094	3.551294101	0.605135972	NA	NA
ASV_16s_14164	0.284738508	0.346065359	3.551131338	0.598029834	NA	NA
ASV_16s_14165	0.217503021	-0.116022404	3.550328056	1.20750433	0.990753085	NA
ASV_16s_14166	0.318813724	0.208865701	3.549724164	1.335735791	0.987458828	NA
ASV_16s_14167	0.272847236	0.352871882	3.550226628	1.369116381	0.986498476	NA
ASV_16s_14168	0.289725657	0.512679867	3.550869898	1.656832159	NA	NA
ASV_16s_14169	0.232958785	0.340265441	3.550275889	1.289379716	NA	NA
ASV_16s_14170	0.195482573	0.249681193	3.550684271	1.057642905	NA	NA
ASV_16s_14171	0.3133927	0.724779963	3.550100401	1.02592718	NA	NA
ASV_16s_14172	0.237226107	0.337327333	3.550255019	1.31311584	0.988085137	NA
ASV_16s_14173	0.247850425	0.335749361	3.550220128	1.354174527	NA	NA
ASV_16s_14174	0.239525649	0.360345221	3.551017837	1.069506663	NA	NA
ASV_16s_14175	0.234788007	0.361027907	3.551303957	0.810447249	NA	NA
ASV_16s_14176	0.236298839	0.35801493	3.551094816	0.77677524	0.997673808	NA
ASV_16s_14177	0.277149196	0.342727029	3.550906091	0.859436726	0.996789014	NA
ASV_16s_14178	0.212930026	0.360329115	3.551070451	1.010559531	NA	NA
ASV_16s_14179	0.212930026	0.360329115	3.551070451	1.010559531	NA	NA
ASV_16s_14180	0.212930026	0.360329115	3.551070451	1.010559531	NA	NA
ASV_16s_14181	0.212930026	0.360329115	3.551070451	1.010559531	NA	NA
ASV_16s_14182	0.212930026	0.360329115	3.551070451	1.010559531	NA	NA
ASV_16s_14183	0.212930026	0.360329115	3.551070451	1.010559531	NA	NA
ASV_16s_14184	0.212930026	0.360329115	3.551070451	1.010559531	NA	NA
ASV_16s_14185	0.212930026	0.360329115	3.551070451	1.010559531	NA	NA
ASV_16s_14186	0.306275731	0.315939509	3.550821799	1.154665702	0.991933529	NA
ASV_16s_14187	0.23810873	0.315939583	3.550888629	1.027567366	0.994371494	NA
ASV_16s_14188	0.238480445	0.315939665	3.550888158	1.028370719	NA	NA
ASV_16s_14189	0.228898967	0.315939597	3.550900693	1.007790306	0.994701887	NA
ASV_16s_14190	0.228214576	0.315939598	3.550901628	1.006289326	0.994726442	NA
ASV_16s_14191	0.226845794	0.3159396	3.550903516	1.003274584	0.99477554	NA
ASV_16s_14192	0.253853197	0.315939561	3.55087004	1.059689118	0.993807355	NA
ASV_16s_14193	0.222397251	0.315939696	3.550909812	0.993361068	NA	NA
ASV_16s_14194	0.222397251	0.315939696	3.550909812	0.993361068	NA	NA
ASV_16s_14195	0.222397251	0.315939696	3.550909812	0.993361068	NA	NA
ASV_16s_14196	0.222397251	0.315939696	3.550909812	0.993361068	NA	NA
ASV_16s_14197	0.294204234	0.734347119	3.550941322	1.10988305	NA	NA
ASV_16s_14198	0.294204234	0.734347119	3.550941322	1.10988305	NA	NA
ASV_16s_14199	0.294204234	0.734347119	3.550941322	1.10988305	NA	NA
ASV_16s_14200	0.145735734	0.731990469	3.551307269	0.768383061	NA	NA
ASV_16s_14201	0.145735734	0.731990469	3.551307269	0.768383061	NA	NA
ASV_16s_14202	0.278798586	0.693268206	3.550818294	1.042106155	0.994120409	NA
ASV_16s_14203	0.281275445	0.693277594	3.550815962	1.046559015	NA	NA
ASV_16s_14204	0.281275445	0.693277594	3.550815962	1.046559015	NA	NA
ASV_16s_14205	0.222397251	0.693040479	3.550885401	0.929120005	NA	NA
ASV_16s_14206	0.222397251	0.693040479	3.550885401	0.929120005	NA	NA
ASV_16s_14207	0.187662592	0.286560613	3.551612323	0.649564879	NA	NA
ASV_16s_14208	0.187662592	0.286560613	3.551612323	0.649564879	NA	NA
ASV_16s_14209	0.187662592	0.286560613	3.551612323	0.649564879	NA	NA
ASV_16s_14210	0.203168105	-0.037926703	3.550831091	1.057945527	NA	NA
ASV_16s_14211	0.190769619	-0.031697141	3.550943816	1.030744273	NA	NA

ASV_16s_14212	0.187742449	-0.056641962	3.550929422	1.051470447	NA	NA
ASV_16s_14213	0.189284554	-0.127312856	3.550824229	1.110792259	0.992838011	NA
ASV_16s_14214	0.192840969	-0.166610843	3.550754005	1.142159518	0.992198267	NA
ASV_16s_14215	0.193099888	-0.182109943	3.550738334	1.149958414	0.992033827	NA
ASV_16s_14216	0.190579148	-0.206984311	3.550742749	1.148283461	0.992069325	NA
ASV_16s_14217	0.197695164	-0.234512941	3.550663223	1.172468218	0.991547056	NA
ASV_16s_14218	0.193876645	-0.228141876	3.550701434	1.160731148	0.991803128	NA
ASV_16s_14219	0.190354454	-0.282677569	3.550713478	1.117955092	0.992694958	NA
ASV_16s_14220	0.218420623	-0.335279689	3.550475423	1.190064958	0.991153854	NA
ASV_16s_14221	0.195171239	-0.306131043	3.550668494	1.122284888	0.992607617	NA
ASV_16s_14222	0.191890855	-0.332669783	3.550694129	1.084758453	0.993342992	NA
ASV_16s_14223	0.213389805	-0.390448055	3.550526789	1.109907098	0.992855565	NA
ASV_16s_14224	0.187856531	-0.375908406	3.550730314	1.022390317	NA	NA
ASV_16s_14225	0.192667611	-0.383149003	3.550694341	1.035907699	0.994228312	NA
ASV_16s_14226	0.196724752	-0.405622637	3.550669143	1.026607471	NA	NA
ASV_16s_14227	0.206360693	-0.451239032	3.550615037	1.003953355	0.994764511	NA
ASV_16s_14228	0.067087117	-0.277027571	3.551707938	0.353644979	NA	NA
ASV_16s_14229	0.269470709	-0.678170294	3.550382809	0.790257593	0.997542111	NA
ASV_16s_14230	0.256030931	-0.673992238	3.550457248	0.773367963	NA	NA
ASV_16s_14231	0.258816985	-0.675117677	3.550442232	0.776964506	0.997671992	NA
ASV_16s_14232	0.243976146	-0.667858212	3.550521122	0.757257457	0.997856084	NA
ASV_16s_14233	0.249372036	-0.671019824	3.550493458	0.764587195	0.997788782	NA
ASV_16s_14234	0.187924979	-0.616314324	3.550834348	0.669320249	NA	NA
ASV_16s_14235	0.187924979	-0.616314324	3.550834348	0.669320249	NA	NA
ASV_16s_14236	0.187924979	-0.616314324	3.550834348	0.669320249	NA	NA
ASV_16s_14237	0.187924979	-0.616314324	3.550834348	0.669320249	NA	NA
ASV_16s_14238	0.187924979	-0.616314324	3.550834348	0.669320249	NA	NA
ASV_16s_14239	0.232805777	-0.661058211	3.550582251	0.741512372	0.997996048	NA
ASV_16s_14240	0.121642183	0.220112646	3.550814537	0.542194881	NA	NA
ASV_16s_14241	0.086871237	0.595651987	3.550865541	0.349155498	NA	NA
ASV_16s_14242	0.197987607	-0.078168007	3.550536658	1.2723553	0.989164421	NA
ASV_16s_14243	0.259980997	-0.726942055	3.550376249	0.835313767	0.99706664	NA
ASV_16s_14244	0.264939615	-0.736162934	3.550347519	0.854753444	0.9968442	NA
ASV_16s_14245	0.194825219	0.10862884	3.550560958	0.932161348	0.995849917	NA
ASV_16s_14246	0.250044117	-0.724832619	3.550433523	0.844866483	0.996958661	NA
ASV_16s_14247	0.23942262	-0.707588606	3.550386548	0.891658962	0.996392194	NA
ASV_16s_14248	0.249003986	-0.716321908	3.550299101	0.932918565	0.995839307	NA
ASV_16s_14249	0.205449287	0.003520659	3.550781177	0.706909588	NA	NA
ASV_16s_14250	0.226007082	0.234137425	3.550324171	1.247177886	0.989799763	NA
ASV_16s_14251	0.219454242	-0.483041946	3.550586388	0.985509519	0.995058913	NA
ASV_16s_14252	0.199766509	0.241708458	3.550656187	1.140501451	0.992232949	NA
ASV_16s_14253	0.196571589	0.362202433	3.55078905	0.996748675	0.994880814	NA
ASV_16s_14254	0.255751455	-0.244910025	3.550621899	0.791700506	NA	NA
ASV_16s_14255	0.230342268	-0.651207453	3.550333481	0.988039386	NA	NA
ASV_16s_14256	0.239230314	1.304084961	3.550246711	0.380793306	NA	NA
ASV_16s_14257	0.250857554	-0.674056591	3.550199611	1.057117179	0.993853794	NA
ASV_16s_14258	0.199402819	0.684381104	3.551271074	0.904022741	NA	NA
ASV_16s_14259	0.246681182	-0.654108869	3.550219208	1.063966347	0.993729633	NA
ASV_16s_14260	0.215145931	-0.061616481	3.550308859	0.674939313	0.998520968	NA
ASV_16s_14261	0.263673938	1.701758309	3.550312711	0.377542101	0.999782924	NA
ASV_16s_14262	0.249331822	-0.644819548	3.550206345	1.087373936	NA	NA
ASV_16s_14263	0.243725693	-0.014077641	3.550230596	0.791294636	0.997531783	NA
ASV_16s_14264	0.248809764	-0.643383378	3.550209746	1.086051202	0.993318466	NA
ASV_16s_14265	0.262753574	-0.217089248	3.550007407	1.11496539	0.992754884	NA
ASV_16s_14266	0.205211113	0.010460634	3.551021008	0.925531762	NA	NA
ASV_16s_14267	0.249052371	-0.624354249	3.550222187	1.101566407	0.993019641	NA
ASV_16s_14268	0.270856752	-0.922678917	3.550099632	0.773026335	NA	NA
ASV_16s_14269	0.230972672	-0.568572898	3.551054354	0.850725346	NA	NA
ASV_16s_14270	0.238074551	-0.581219986	3.550317216	1.085512386	NA	NA
ASV_16s_14271	0.249936005	-0.599164487	3.550247517	1.120008052	NA	NA
ASV_16s_14272	0.247568898	-0.57169814	3.55029715	1.122184649	NA	NA
ASV_16s_14273	0.249322752	-0.582883238	3.550273257	1.125215033	NA	NA
ASV_16s_14274	0.217744534	0.375258152	3.550781145	1.118306176	0.9926879	NA
ASV_16s_14275	0.241615801	-0.587403267	3.550047459	0.47851543	NA	NA
ASV_16s_14276	0.218648312	-0.576313203	3.550749035	0.865110632	NA	NA
ASV_16s_14277	0.248813175	0.183282093	3.549669812	1.13142034	NA	NA
ASV_16s_14278	0.246869737	-0.507597718	3.550402816	1.129147439	NA	NA

ASV_16s_14279	0.251998015	-0.508014442	3.550389878	1.143622993	0.992167574	NA
ASV_16s_14280	0.214442248	-0.409764175	3.551313069	0.778411871	NA	NA
ASV_16s_14281	0.22516362	-0.285356871	3.550692353	1.020400881	NA	NA
ASV_16s_14282	0.237142151	-0.360334552	3.54978733	0.673117425	NA	NA
ASV_16s_14283	0.277282044	-0.610045029	3.55020899	1.111903895	0.992815927	NA
ASV_16s_14284	0.266980184	0.210512038	3.550739139	1.074274825	NA	NA
ASV_16s_14285	0.226206097	-0.497359362	3.550713029	0.794538589	0.99749929	NA
ASV_16s_14286	0.261297535	0.104589679	3.550791501	1.188189337	0.991196298	NA
ASV_16s_14287	0.162404452	-0.118224081	3.550804776	0.50456323	0.999429601	NA
ASV_16s_14288	0.186356972	-0.50328734	3.551224052	0.622946965	NA	NA
ASV_16s_14289	0.208051358	1.225412583	3.550970976	0.652146404	NA	NA
ASV_16s_14290	0.270266544	0.079613151	3.549896599	0.87010357	0.996660979	NA
ASV_16s_14291	0.207040163	1.596917238	3.550722664	0.389196496	0.999759635	NA
ASV_16s_14292	0.203636389	0.37467409	3.55148951	0.677099402	NA	NA
ASV_16s_14293	0.246526686	0.386031568	3.551235264	0.741570299	NA	NA
ASV_16s_14294	0.261946289	0.837458318	3.551138146	0.725071716	NA	NA
ASV_16s_14295	0.2914732	0.353142315	3.55183532	0.447698559	NA	NA
ASV_16s_14296	0.183102998	-0.154113587	3.550668054	1.096357157	0.993120895	NA
ASV_16s_14297	0.268992304	0.392062511	3.551103294	0.770744336	0.99773118	NA
ASV_16s_14298	0.21890503	0.124517884	3.550872463	1.273324461	0.989139489	NA
ASV_16s_14299	0.254016745	0.38808072	3.551189205	0.75157379	NA	NA
ASV_16s_14300	0.254016745	0.38808072	3.551189205	0.75157379	NA	NA
ASV_16s_14301	0.225813042	-0.239047459	3.550686197	0.512947586	0.999397678	NA
ASV_16s_14302	0.249270789	0.494072026	3.549857474	0.777306467	NA	NA
ASV_16s_14303	0.157985723	-0.240196201	3.550746103	0.648538994	0.998700654	NA
ASV_16s_14304	0.219692386	-1.09468072	3.550471245	0.383895147	NA	NA
ASV_16s_14305	0.210989634	-1.06747543	3.550553064	0.375862337	NA	NA
ASV_16s_14306	0.121951093	1.088299807	3.551170824	0.414625714	NA	NA
ASV_16s_14307	0.209067134	1.249143307	3.550751196	0.593404652	NA	NA
ASV_16s_14308	0.209067134	1.249143307	3.550751196	0.593404652	NA	NA
ASV_16s_14309	0.213366264	0.695973212	3.550146573	0.939267017	NA	NA
ASV_16s_14310	0.269969868	0.119199169	3.549793695	1.597847376	0.978727598	NA
ASV_16s_14311	0.217665394	0.400611239	3.551029379	0.632699273	NA	NA
ASV_16s_14312	0.225836616	0.766458329	3.550510293	1.105150148	0.992949438	NA
ASV_16s_14313	0.109226309	-0.43298364	3.551170267	0.415902881	NA	NA
ASV_16s_14314	0.109226309	-0.43298364	3.551170267	0.415902881	NA	NA
ASV_16s_14315	0.109226309	-0.43298364	3.551170267	0.415902881	NA	NA
ASV_16s_14316	0.109226309	-0.43298364	3.551170267	0.415902881	NA	NA
ASV_16s_14317	0.132652847	-0.481784636	3.550942121	0.474179953	NA	NA
ASV_16s_14318	0.132652847	-0.481784636	3.550942121	0.474179953	NA	NA
ASV_16s_14319	0.12157348	-0.460114181	3.551055164	0.447574491	NA	NA
ASV_16s_14320	0.12157348	-0.460114181	3.551055164	0.447574491	NA	NA
ASV_16s_14321	0.185605758	-0.07864219	3.550808914	0.900436747	NA	NA
ASV_16s_14322	0.185605758	-0.07864219	3.550808914	0.900436747	NA	NA
ASV_16s_14323	0.130161108	0.220100718	3.550799007	0.572305892	0.999136307	NA
ASV_16s_14324	0.135229455	0.220094384	3.550790692	0.589587241	0.999047872	NA
ASV_16s_14325	0.138177069	0.220091082	3.550786136	0.599453053	NA	NA
ASV_16s_14326	0.235587762	0.310938947	3.550865826	0.99936805	0.994838724	NA
ASV_16s_14327	0.191551977	-0.068380723	3.550718287	1.092687148	NA	NA
ASV_16s_14328	0.243267196	0.104490896	3.550454207	0.779334704	NA	NA
ASV_16s_14329	0.195020606	-0.068438101	3.550596307	1.179065429	NA	NA
ASV_16s_14330	0.187599818	1.339672153	3.55109518	0.51882262	NA	NA
ASV_16s_14331	0.196011642	-0.068304485	3.550547152	1.193504673	NA	NA
ASV_16s_14332	0.249314775	0.36145846	3.551830743	0.620544854	NA	NA
ASV_16s_14333	0.314093723	0.808603228	3.550679694	1.312025218	NA	NA
ASV_16s_14334	0.211553196	-0.068288621	3.550418803	1.251950885	0.98968114	NA
ASV_16s_14335	0.208862095	-0.068235901	3.550434242	1.242442764	NA	NA
ASV_16s_14336	0.175597871	0.002188167	3.551003239	0.871590085	NA	NA
ASV_16s_14337	0.200966826	-0.064453788	3.550601597	1.154525868	NA	NA
ASV_16s_14338	0.245421718	1.062915333	3.550858566	1.131792427	NA	NA
ASV_16s_14339	0.261915411	0.732926695	3.551378628	0.791880677	NA	NA
ASV_16s_14340	0.288640996	0.766741143	3.550622948	1.028670562	NA	NA
ASV_16s_14341	0.319941438	-0.239368849	3.549758408	1.296815893	NA	NA
ASV_16s_14342	0.213846167	0.665557468	3.550707283	0.852936033	NA	NA
ASV_16s_14343	0.112828826	0.283344303	3.551712805	0.490400994	NA	NA
ASV_16s_14344	0.130297413	0.281929806	3.551675175	0.537419332	NA	NA
ASV_16s_14345	0.307800422	-0.602470167	3.550666374	0.821767384	NA	NA

ASV_16s_14346	0.28960457	0.350885148	3.551278306	0.577185014	0.999111926	NA
ASV_16s_14347	0.246526686	-0.547191532	3.550031677	0.669527134	NA	NA
ASV_16s_14348	0.241876744	1.157395993	3.550293966	1.027815092	NA	NA
ASV_16s_14349	0.235823906	1.057175673	3.550469923	1.012401937	NA	NA
ASV_16s_14350	0.22800856	0.630186793	3.550876805	0.954535299	0.995528986	NA
ASV_16s_14351	0.166529949	-0.09859756	3.550806645	0.913715588	NA	NA
ASV_16s_14352	0.223350487	-0.075274972	3.550690236	1.115255078	0.992749091	NA
ASV_16s_14353	0.225191251	0.02724655	3.550853635	1.031509496	0.994304104	NA
ASV_16s_14354	0.322133586	0.481280185	3.550831434	1.819435321	NA	NA
ASV_16s_14355	0.449355641	0.774923785	3.550808598	1.647106631	NA	NA
ASV_16s_14356	0.258805144	0.346443131	3.550166745	1.382723556	NA	NA
ASV_16s_14357	0.205509547	-0.028799223	3.550914259	1.009546646	NA	NA
ASV_16s_14358	0.219846072	0.13208887	3.550891052	1.403366381	NA	NA
ASV_16s_14359	0.22091371	-0.445559369	3.550664732	0.799690499	NA	NA
ASV_16s_14360	0.255570055	0.37329432	3.550489586	1.249687028	NA	NA
ASV_16s_14361	0.257918098	0.335617615	3.550188461	1.389519138	NA	NA
ASV_16s_14362	0.232255427	0.342112237	3.550313606	1.278249584	NA	NA
ASV_16s_14363	0.2499747	0.360607041	3.550435908	1.254324249	NA	NA
ASV_16s_14364	0.168422002	-0.073434793	3.550870976	0.877571322	NA	NA
ASV_16s_14365	0.168422002	-0.073434793	3.550870976	0.877571322	NA	NA
ASV_16s_14366	0.240323627	0.290400439	3.55046695	1.442591563	0.984230864	NA
ASV_16s_14367	0.234429359	0.360342418	3.551027017	1.058656859	NA	NA
ASV_16s_14368	0.256103047	0.665034519	3.550028685	1.182852709	NA	NA
ASV_16s_14369	0.232213298	0.329408813	3.550973007	0.844635946	NA	NA
ASV_16s_14370	0.2083996	0.360325971	3.55108076	0.999804015	NA	NA
ASV_16s_14371	0.2083996	0.360325971	3.55108076	0.999804015	NA	NA
ASV_16s_14372	0.239075214	0.315939598	3.550887427	1.029556405	0.994337556	NA
ASV_16s_14373	0.225982315	0.315939619	3.550904732	1.001308694	0.994807398	NA
ASV_16s_14374	0.24762089	0.315939585	3.550877132	1.047135446	0.994031921	NA
ASV_16s_14375	0.29001407	0.315939537	3.550834922	1.126933661	0.992513112	NA
ASV_16s_14376	0.217665394	0.315939713	3.5509168	0.982564991	NA	NA
ASV_16s_14377	0.217665394	0.315939713	3.5509168	0.982564991	NA	NA
ASV_16s_14378	0.207290816	0.687881876	3.550874595	0.869786671	NA	NA
ASV_16s_14379	0.304876463	1.165915187	3.550746892	0.862394979	NA	NA
ASV_16s_14380	0.310955509	1.085407337	3.550836265	0.919893136	NA	NA
ASV_16s_14381	0.303871301	0.734386026	3.550929762	1.126148699	0.992529122	NA
ASV_16s_14382	0.28794457	0.734319884	3.550949243	1.098986863	NA	NA
ASV_16s_14383	0.142634974	0.731462533	3.551321946	0.758602905	NA	NA
ASV_16s_14384	0.142634974	0.731462533	3.551321946	0.758602905	NA	NA
ASV_16s_14385	0.26769516	0.693232161	3.55082929	1.021622217	NA	NA
ASV_16s_14386	0.24050507	0.693124285	3.55086046	0.967945892	0.995329173	NA
ASV_16s_14387	0.272153506	0.693246338	3.550824771	1.02992341	0.994331279	NA
ASV_16s_14388	0.258014659	0.693195465	3.55083964	1.00310908	0.994778227	NA
ASV_16s_14389	0.259005403	0.693199207	3.550838546	1.005031194	0.994746968	NA
ASV_16s_14390	0.232698125	0.693089132	3.550870747	0.951518502	0.995573161	NA
ASV_16s_14391	0.217665394	0.693016029	3.550892616	0.91844534	NA	NA
ASV_16s_14392	0.217665394	0.693016029	3.550892616	0.91844534	NA	NA
ASV_16s_14393	0.217665394	0.693016029	3.550892616	0.91844534	NA	NA
ASV_16s_14394	0.25297599	0.693175935	3.550845351	0.993160801	0.994938109	NA
ASV_16s_14395	0.183582971	0.287585454	3.551632138	0.642084245	NA	NA
ASV_16s_14396	0.183582971	0.287585454	3.551632138	0.642084245	NA	NA
ASV_16s_14397	0.183582971	0.287585454	3.551632138	0.642084245	NA	NA
ASV_16s_14398	0.183582971	0.287585454	3.551632138	0.642084245	NA	NA
ASV_16s_14399	0.183582971	0.287585454	3.551632138	0.642084245	NA	NA
ASV_16s_14400	0.183582971	0.287585454	3.551632138	0.642084245	NA	NA
ASV_16s_14401	0.183657124	-0.042563959	3.55098461	1.022092337	NA	NA
ASV_16s_14402	0.183668532	-0.078001543	3.550932273	1.053921441	0.993911186	NA
ASV_16s_14403	0.183668532	-0.078001543	3.550932273	1.053921441	0.993911186	NA
ASV_16s_14404	0.187725672	-0.107175744	3.550862375	1.084518967	0.993347529	NA
ASV_16s_14405	0.187725672	-0.107175744	3.550862375	1.084518967	0.993347529	NA
ASV_16s_14406	0.187984591	-0.123946405	3.550839845	1.096924747	0.993109908	NA
ASV_16s_14407	0.186471007	-0.136000609	3.550839913	1.101271146	0.993025405	NA
ASV_16s_14408	0.201431272	-0.168925617	3.550672571	1.160486588	0.991808411	NA
ASV_16s_14409	0.188502429	-0.156621408	3.55080068	1.118043197	0.992693187	NA
ASV_16s_14410	0.188761348	-0.172648225	3.55078397	1.12670931	0.99251769	NA
ASV_16s_14411	0.18370846	-0.192336785	3.550812822	1.12013796	0.992651007	NA
ASV_16s_14412	0.191666057	-0.224161602	3.550723928	1.146854288	0.992099536	NA

ASV_16s_14413	0.189538104	-0.220008827	3.550744221	1.143828826	0.992163251	NA
ASV_16s_14414	0.20825828	-0.264945938	3.55056271	1.197680867	0.990980197	NA
ASV_16s_14415	0.188277734	-0.232780754	3.550748685	1.135885619	0.99232899	NA
ASV_16s_14416	0.183725572	-0.239309249	3.550785429	1.111617486	0.992821621	NA
ASV_16s_14417	0.190314861	-0.267038677	3.550718644	1.127762785	0.992496177	NA
ASV_16s_14418	0.183731277	-0.254945533	3.55077903	1.103838179	0.99297519	NA
ASV_16s_14419	0.186781262	-0.29240843	3.550742375	1.094529267	0.993156203	NA
ASV_16s_14420	0.065628702	-0.269822201	3.551720287	0.348159436	NA	NA
ASV_16s_14421	0.262387065	-0.67653248	3.55042356	0.781468163	0.997628509	NA
ASV_16s_14422	0.23794661	-0.664226012	3.55055348	0.748842413	0.997931666	NA
ASV_16s_14423	0.237482965	-0.663943963	3.55055602	0.748190089	0.99793745	NA
ASV_16s_14424	0.24334041	-0.667466602	3.550524568	0.756341739	0.997864396	NA
ASV_16s_14425	0.22960099	-0.658823605	3.550599566	0.736908785	NA	NA
ASV_16s_14426	0.235274925	-0.66258689	3.550568298	0.745046353	NA	NA
ASV_16s_14427	0.195234319	-0.626983405	3.550796331	0.682428325	NA	NA
ASV_16s_14428	0.190170023	-0.619617905	3.550822477	0.67336619	0.998532114	NA
ASV_16s_14429	0.189916808	-0.61924472	3.550823814	0.672907448	0.998535353	NA
ASV_16s_14430	0.183839654	-0.61000597	3.550856522	0.661692499	NA	NA
ASV_16s_14431	0.183839654	-0.61000597	3.550856522	0.661692499	NA	NA
ASV_16s_14432	0.183839654	-0.61000597	3.550856522	0.661692499	NA	NA
ASV_16s_14433	0.183839654	-0.61000597	3.550856522	0.661692499	NA	NA
ASV_16s_14434	0.053719097	-0.168578712	3.550999889	0.259838333	0.999938561	NA
ASV_16s_14435	0.061759636	0.220286293	3.551043627	0.28125761	NA	NA
ASV_16s_14436	0.061759636	0.220286293	3.551043627	0.28125761	NA	NA
ASV_16s_14438	0.130083161	0.595917845	3.550761059	0.509689814	NA	NA
ASV_16s_14439	0.187442556	0.082672734	3.551006677	0.96304032	NA	NA
ASV_16s_14440	0.205724816	-0.010291097	3.550694171	1.12892349	0.992472429	NA
ASV_16s_14441	0.186754302	0.038294045	3.550755982	1.030196922	0.994326599	NA
ASV_16s_14442	0.230918898	-0.428260082	3.550703369	1.090699272	0.993229812	NA
ASV_16s_14443	0.190194738	0.246620798	3.550862506	1.038622733	0.994181206	NA
ASV_16s_14444	0.255050268	-0.655817989	3.550684985	0.719320575	0.998182842	NA
ASV_16s_14445	0.208169397	0.312934645	3.550595291	0.989280944	0.994999601	NA
ASV_16s_14446	0.188248703	0.143526201	3.550696116	1.0630597	0.99374616	NA
ASV_16s_14447	0.247632292	-0.714407371	3.550307484	0.929571376	0.995886075	NA
ASV_16s_14448	0.189206693	0.680458066	3.551026544	0.81334266	0.997305391	NA
ASV_16s_14449	0.198848209	0.352698219	3.550768477	1.061063228	0.993782453	NA
ASV_16s_14450	0.242636317	-0.694485865	3.550294157	0.965549201	0.995365297	NA
ASV_16s_14451	0.280787464	1.76955922	3.550019122	0.3482556	0.999834505	NA
ASV_16s_14452	0.242724047	-0.657253262	3.550245056	1.040058424	NA	NA
ASV_16s_14453	0.224967463	-0.627548693	3.550359353	0.992294159	NA	NA
ASV_16s_14454	0.246187485	0.274121103	3.549824551	0.739441208	0.998013996	NA
ASV_16s_14455	0.24351469	-0.635121832	3.550241298	1.067404528	0.99366671	NA
ASV_16s_14456	0.207995177	0.773907616	3.550658411	0.862805867	0.996748927	NA
ASV_16s_14457	0.252887448	-0.645259991	3.550188629	1.097152195	0.993105502	NA
ASV_16s_14458	0.246035748	-0.628918928	3.550232275	1.082613157	NA	NA
ASV_16s_14459	0.231529675	-0.415232867	3.550500674	1.022817207	NA	NA
ASV_16s_14460	0.22928397	-0.539369751	3.550438407	1.087917073	0.993282966	NA
ASV_16s_14461	0.235474007	-0.052291485	3.549935618	0.994712551	NA	NA
ASV_16s_14462	0.242441961	0.183066754	3.549681435	1.105392213	NA	NA
ASV_16s_14463	0.229743979	-0.115315633	3.550783116	1.069518105	0.993627831	NA
ASV_16s_14464	0.202885743	0.057304576	3.550707914	0.917816145	NA	NA
ASV_16s_14465	0.254026599	-0.429254123	3.550498308	1.136484969	NA	NA
ASV_16s_14466	0.23015846	-0.341555321	3.55064356	1.052275549	0.993940611	NA
ASV_16s_14467	0.25011097	-0.073747286	3.55050997	0.712625271	0.998236838	NA
ASV_16s_14468	0.199493247	-0.547454388	3.5508921	0.670770308	NA	NA
ASV_16s_14469	0.25920555	0.309465903	3.550773941	1.101498004	NA	NA
ASV_16s_14470	0.238340063	-0.247760095	3.551224	0.772153084	NA	NA
ASV_16s_14471	0.218964017	0.202134584	3.549866523	0.907541548	0.996185404	NA
ASV_16s_14472	0.228330361	0.14561849	3.550626289	0.749485823	NA	NA
ASV_16s_14473	0.29504191	-0.970654231	3.549885927	0.546020074	NA	NA
ASV_16s_14474	0.253826194	0.266084831	3.550756996	1.024155812	0.994429401	NA
ASV_16s_14475	0.189154951	0.354979463	3.551556755	0.436095074	NA	NA
ASV_16s_14476	0.237771839	0.012193553	3.550593901	1.012536264	NA	NA
ASV_16s_14477	0.215155333	-0.418020668	3.550556687	0.994012506	0.994924546	NA
ASV_16s_14478	0.259392011	0.714428059	3.550777732	1.037008721	NA	NA
ASV_16s_14479	0.217974347	0.054788766	3.550871727	0.977485657	NA	NA
ASV_16s_14480	0.243554986	1.711823051	3.550448923	0.417718655	0.999695494	NA

ASV_16s_14481	0.229916913	1.523973212	3.550632776	0.55143173	NA	NA
ASV_16s_14482	0.261444089	0.355412525	3.55175082	0.433286733	NA	NA
ASV_16s_14483	0.197753459	1.138219899	3.55104899	0.675926138	0.998513948	NA
ASV_16s_14484	0.260050435	0.360887834	3.551675688	0.445269638	NA	NA
ASV_16s_14485	0.154375472	0.351322671	3.551783209	0.584558449	NA	NA
ASV_16s_14486	0.154375472	0.351322671	3.551783209	0.584558449	NA	NA
ASV_16s_14487	0.220357191	0.352502556	3.551914763	0.394434522	0.999748629	NA
ASV_16s_14488	0.277044313	-0.405430393	3.550044705	1.038148789	NA	NA
ASV_16s_14489	0.356031982	0.40323014	3.550664653	0.863004813	NA	NA
ASV_16s_14490	0.284749228	0.360453966	3.551884217	0.47015553	NA	NA
ASV_16s_14491	0.229755386	-0.883351876	3.550478425	0.628501507	NA	NA
ASV_16s_14492	0.154176516	-0.505527308	3.551074369	0.588373699	NA	NA
ASV_16s_14493	0.119299982	1.081035378	3.551187488	0.407912346	NA	NA
ASV_16s_14494	0.256685161	0.132019323	3.54991287	1.465601388	0.983476702	NA
ASV_16s_14495	0.225175693	0.336465203	3.55113245	0.575566775	NA	NA
ASV_16s_14496	0.175774122	0.381009535	3.551300655	0.562396262	NA	NA
ASV_16s_14497	0.124068389	0.358256745	3.55158639	0.450569039	0.999608106	NA
ASV_16s_14498	0.155156424	-0.513342577	3.55072028	0.521376828	0.999364382	NA
ASV_16s_14499	0.262116612	-0.547513316	3.549952552	0.688929934	NA	NA
ASV_16s_14500	0.212933538	-0.688507144	3.550471123	0.56109097	NA	NA
ASV_16s_14501	0.094280663	1.003897185	3.551378235	0.340634314	NA	NA
ASV_16s_14502	0.094280663	1.003897185	3.551378235	0.340634314	NA	NA
ASV_16s_14503	0.094280663	1.003897185	3.551378235	0.340634314	NA	NA
ASV_16s_14504	0.18157085	-0.078620463	3.550815443	0.889684364	NA	NA
ASV_16s_14505	0.18157085	-0.078620463	3.550815443	0.889684364	NA	NA
ASV_16s_14506	0.13517322	0.220094627	3.550790789	0.589359592	NA	NA
ASV_16s_14507	0.13517322	0.220094627	3.550790789	0.589359592	NA	NA
ASV_16s_14508	0.13517322	0.220094627	3.550790789	0.589359592	NA	NA
ASV_16s_14509	0.137674739	0.351734708	3.550760077	0.931974911	0.995852527	NA
ASV_16s_14510	0.230331178	0.310939371	3.550872155	0.987997776	0.995019832	NA
ASV_16s_14511	0.210572188	0.491247627	3.550681582	1.04113986	NA	NA
ASV_16s_14512	0.212933538	0.253580101	3.550492031	1.086924596	NA	NA
ASV_16s_14513	0.187021551	-0.06760152	3.550744161	1.062372438	NA	NA
ASV_16s_14514	0.335259966	1.236244998	3.550464276	1.118740003	0.992679173	NA
ASV_16s_14515	0.192967771	-0.068078508	3.550543935	1.186121549	NA	NA
ASV_16s_14516	0.186420135	0.510712169	3.550994925	0.572414113	0.999135771	NA
ASV_16s_14517	0.245427685	0.455184381	3.550577846	0.825154826	0.997178685	NA
ASV_16s_14518	0.183584431	0.385808906	3.551481581	0.718392154	NA	NA
ASV_16s_14519	0.181602206	-0.385220134	3.551498636	0.69836285	NA	NA
ASV_16s_14520	0.233195964	1.321101058	3.550734028	0.59998419	NA	NA
ASV_16s_14521	0.19125494	0.873985213	3.551347677	0.493854134	NA	NA
ASV_16s_14522	0.197427436	-0.05701629	3.55068643	1.11564847	NA	NA
ASV_16s_14523	0.20415548	-0.063255859	3.550598165	1.144632529	NA	NA
ASV_16s_14524	0.414730591	-0.565438882	3.549659982	0.906378414	NA	NA
ASV_16s_14525	0.2894568	0.352848749	3.551413744	0.559251331	NA	NA
ASV_16s_14526	0.269919591	0.323708143	3.550962606	0.764365075	0.997790841	NA
ASV_16s_14527	0.24116741	-0.547070878	3.550061364	0.662547153	NA	NA
ASV_16s_14528	0.223740686	-0.800230503	3.550593584	0.620288181	NA	NA
ASV_16s_14529	0.152378406	-0.078428636	3.550872819	0.805739711	NA	NA
ASV_16s_14530	0.187823575	-0.116394463	3.55036377	1.112417808	NA	NA
ASV_16s_14531	0.394623267	0.337262802	3.549749915	1.848902568	NA	NA
ASV_16s_14532	0.201041948	-0.028773025	3.550923	0.998658345	NA	NA
ASV_16s_14533	0.201041948	-0.028773025	3.550923	0.998658345	NA	NA
ASV_16s_14534	0.229716011	-0.073769673	3.550775984	1.02981157	NA	NA
ASV_16s_14535	0.241126791	-0.024447955	3.550763491	1.294088365	NA	NA
ASV_16s_14536	0.27253446	0.252370508	3.550205036	1.603312519	0.978515981	NA
ASV_16s_14537	0.167775296	-0.073430074	3.55087236	0.875676116	NA	NA
ASV_16s_14538	0.167775296	-0.073430074	3.55087236	0.875676116	NA	NA
ASV_16s_14539	0.209604262	0.548120606	3.550713779	1.265076418	0.989350543	NA
ASV_16s_14540	0.221923621	0.33913086	3.550349549	1.244483851	NA	NA
ASV_16s_14541	0.229333068	0.36033949	3.551036602	1.047578884	NA	NA
ASV_16s_14542	0.229333068	0.36033949	3.551036602	1.047578884	NA	NA
ASV_16s_14543	0.222230915	0.346430069	3.551109183	0.754982106	0.997876698	NA
ASV_16s_14544	0.263389758	0.349272184	3.550937501	0.818590498	0.99724957	NA
ASV_16s_14545	0.218770299	0.328877342	3.550977827	0.845910735	0.996946702	NA
ASV_16s_14546	0.203869174	0.360322686	3.551091523	0.988853479	NA	NA
ASV_16s_14547	0.203869174	0.360322686	3.551091523	0.988853479	NA	NA

ASV_16s_14548	0.203869174	0.360322686	3.551091523	0.988853479	NA	NA
ASV_16s_14549	0.203869174	0.360322686	3.551091523	0.988853479	NA	NA
ASV_16s_14550	0.203869174	0.360322686	3.551091523	0.988853479	NA	NA
ASV_16s_14551	0.203869174	0.360322686	3.551091523	0.988853479	NA	NA
ASV_16s_14552	0.207092059	0.765316835	3.55085382	1.172755661	NA	NA
ASV_16s_14553	0.272306324	0.315939436	3.550850964	1.095064489	0.993145877	NA
ASV_16s_14554	0.229448394	0.315939476	3.550899971	1.008881159	0.994683996	NA
ASV_16s_14555	0.228332341	0.315939631	3.55090149	1.00645361	NA	NA
ASV_16s_14556	0.218750863	0.315939648	3.550915186	0.984997872	0.995066925	NA
ASV_16s_14557	0.245933449	0.315939458	3.550879136	1.043382245	0.994098037	NA
ASV_16s_14558	0.212933538	0.31593966	3.550924098	0.971550129	NA	NA
ASV_16s_14559	0.212933538	0.31593966	3.550924098	0.971550129	NA	NA
ASV_16s_14560	0.212933538	0.31593966	3.550924098	0.971550129	NA	NA
ASV_16s_14561	0.212933538	0.31593966	3.550924098	0.971550129	NA	NA
ASV_16s_14562	0.286704597	0.596215045	3.550645868	0.887997177	NA	NA
ASV_16s_14563	0.202784494	0.687859786	3.550881687	0.858981478	NA	NA
ASV_16s_14564	0.202784494	0.687859786	3.550881687	0.858981478	NA	NA
ASV_16s_14565	0.349998768	1.183081929	3.550396655	0.895475261	NA	NA
ASV_16s_14566	0.289548912	1.021031056	3.550716428	0.866959573	NA	NA
ASV_16s_14567	0.139534213	0.730922449	3.551337244	0.748674693	NA	NA
ASV_16s_14568	0.2618757	0.693211314	3.550835429	1.010534896	NA	NA
ASV_16s_14569	0.274188095	0.693253439	3.550822771	1.033597931	0.994268195	NA
ASV_16s_14570	0.246125282	0.693148483	3.550853467	0.979423971	0.995153662	NA
ASV_16s_14571	0.260112658	0.693203572	3.55083735	1.007093214	0.9947133	NA
ASV_16s_14572	0.269306277	0.693236747	3.55082765	1.024587613	NA	NA
ASV_16s_14573	0.269306277	0.693236747	3.55082765	1.024587613	NA	NA
ASV_16s_14574	0.269306277	0.693236747	3.55082765	1.024587613	NA	NA
ASV_16s_14575	0.255526274	0.693186183	3.550842437	0.998200357	0.994857515	NA
ASV_16s_14576	0.212933538	0.692990494	3.55090015	0.90757135	NA	NA
ASV_16s_14577	0.212933538	0.692990494	3.55090015	0.90757135	NA	NA
ASV_16s_14578	0.077012652	0.678839466	3.551955164	0.260843459	0.999937749	NA
ASV_16s_14579	0.190022683	1.212096542	3.551063501	0.494115839	0.999467743	NA
ASV_16s_14580	0.179503349	0.288631881	3.551652683	0.634454198	NA	NA
ASV_16s_14581	0.179503349	0.288631881	3.551652683	0.634454198	NA	NA
ASV_16s_14582	0.183128213	-0.076208128	3.550939371	1.042009884	0.994122095	NA
ASV_16s_14583	0.183387132	-0.094180654	3.550913328	1.057123889	0.993853673	NA
ASV_16s_14584	0.183387132	-0.094180654	3.550913328	1.057123889	0.993853673	NA
ASV_16s_14585	0.183646051	-0.111742953	3.550889676	1.070570243	0.99360842	NA
ASV_16s_14586	0.184163888	-0.145856495	3.550848757	1.093259957	0.993180654	NA
ASV_16s_14587	0.184422807	-0.162505034	3.550831173	1.102681489	0.992997845	NA
ASV_16s_14588	0.179617431	-0.166234573	3.550867081	1.093280652	0.993180256	NA
ASV_16s_14589	0.184681726	-0.178939931	3.550815332	1.110830318	0.992837256	NA
ASV_16s_14590	0.182160986	-0.204409366	3.550817181	1.111808068	0.992817832	NA
ASV_16s_14591	0.185717401	-0.243255907	3.550766882	1.119059467	0.992672742	NA
ASV_16s_14592	0.183449877	-0.270063531	3.550776342	1.093531202	0.993175434	NA
ASV_16s_14593	0.179651655	-0.262411614	3.550809935	1.079674331	0.993438892	NA
ASV_16s_14594	0.186494158	-0.291895245	3.550745171	1.093453609	0.993176927	NA
ASV_16s_14595	0.183708795	-0.286611626	3.550768711	1.083873318	0.993359751	NA
ASV_16s_14596	0.182949151	-0.285145831	3.550775251	1.081234622	0.993409555	NA
ASV_16s_14597	0.191976428	-0.332738194	3.550693745	1.082623804	0.993383364	NA
ASV_16s_14598	0.179674471	-0.328540723	3.550794657	1.031255401	NA	NA
ASV_16s_14599	0.035559681	-0.021875155	3.551645458	0.046747009	0.999999835	NA
ASV_16s_14600	0.064170286	-0.2626403	3.551731139	0.34260275	NA	NA
ASV_16s_14601	0.064170286	-0.2626403	3.551731139	0.34260275	NA	NA
ASV_16s_14602	0.2671524	-0.677642998	3.550396291	0.787320784	0.997571206	NA
ASV_16s_14603	0.237017176	-0.663649657	3.550558691	0.747492371	0.997943625	NA
ASV_16s_14604	0.233308012	-0.661368361	3.550579416	0.742224817	0.997989849	NA
ASV_16s_14605	0.232844366	-0.661080643	3.550582048	0.741561658	0.997995619	NA
ASV_16s_14606	0.190895778	-0.620661182	3.550818743	0.674638111	NA	NA
ASV_16s_14607	0.186591127	-0.614250344	3.550841682	0.666782942	0.998578146	NA
ASV_16s_14608	0.18355255	-0.609532807	3.550858186	0.661110352	0.998617029	NA
ASV_16s_14609	0.183299335	-0.60913622	3.550859581	0.660633813	0.998620263	NA
ASV_16s_14610	0.179754328	-0.603527702	3.550879441	0.653910955	NA	NA
ASV_16s_14611	0.179754328	-0.603527702	3.550879441	0.653910955	NA	NA
ASV_16s_14612	0.179754328	-0.603527702	3.550879441	0.653910955	NA	NA
ASV_16s_14613	0.179754328	-0.603527702	3.550879441	0.653910955	NA	NA
ASV_16s_14614	0.057270878	-0.168700269	3.550969543	0.279655767	NA	NA

ASV_16s_14615	0.057270878	-0.168700269	3.550969543	0.279655767	NA	NA
ASV_16s_14616	0.057270878	-0.168700269	3.550969543	0.279655767	NA	NA
ASV_16s_14617	0.050799689	-0.16606484	3.551018783	0.243008901	NA	NA
ASV_16s_14618	0.039807959	-0.154620465	3.55111121	0.17635197	NA	NA
ASV_16s_14619	0.060950733	-0.168810413	3.550941786	0.299794187	NA	NA
ASV_16s_14620	0.087098337	0.220183988	3.550908549	0.403809978	0.999728074	NA
ASV_16s_14621	0.079837583	0.220206685	3.550938504	0.370992587	NA	NA
ASV_16s_14622	0.192176234	-0.050182695	3.550737561	1.077031506	0.993488395	NA
ASV_16s_14623	0.202688521	-0.404028416	3.55077904	0.608286883	NA	NA
ASV_16s_14624	0.204520867	-0.058246828	3.550619756	1.05864887	0.993826165	NA
ASV_16s_14625	0.213209971	-0.559387501	3.550774349	0.955514233	0.99551459	NA
ASV_16s_14626	0.23625601	-0.707195187	3.550444107	0.861850653	0.996760325	NA
ASV_16s_14627	0.191932555	-0.914545331	3.550820436	0.468701746	0.999553205	NA
ASV_16s_14628	0.271597821	-0.246403086	3.550051248	1.078773193	0.993455798	NA
ASV_16s_14629	0.265633173	-0.738468395	3.550212249	0.959874579	0.995450107	NA
ASV_16s_14630	0.185254712	-0.215897167	3.550564809	0.76207371	NA	NA
ASV_16s_14631	0.227719059	-0.680661524	3.550412833	0.906594351	0.996197947	NA
ASV_16s_14632	0.242059839	-0.685884973	3.550285369	0.985503846	0.995059002	NA
ASV_16s_14633	0.234663939	-0.66134824	3.55030978	0.989594847	0.994994644	NA
ASV_16s_14634	0.24213549	-0.655302141	3.550248418	1.037179119	NA	NA
ASV_16s_14635	0.196658584	0.225204029	3.550491822	0.663077616	NA	NA
ASV_16s_14636	0.191895811	-0.117852084	3.550971704	0.931894889	NA	NA
ASV_16s_14637	0.234609271	-0.597934692	3.550313425	1.054959474	0.993892582	NA
ASV_16s_14638	0.241938866	-0.56834902	3.551014039	0.865224795	0.996719945	NA
ASV_16s_14639	0.211859983	-0.571802284	3.55046074	0.984998402	NA	NA
ASV_16s_14640	0.208910854	0.238218885	3.550390467	1.148614426	0.992062319	NA
ASV_16s_14641	0.206899719	0.844222924	3.550591009	0.65977189	0.998626099	NA
ASV_16s_14642	0.233696723	-0.58126704	3.550335855	1.060867487	0.993786005	NA
ASV_16s_14643	0.266949857	-0.648876144	3.550125445	1.159574645	NA	NA
ASV_16s_14644	0.22826413	-0.536442296	3.550419177	1.05982498	0.993804896	NA
ASV_16s_14645	0.222692427	0.004195769	3.5504794	1.026401434	0.994391327	NA
ASV_16s_14646	0.22749739	-0.440004761	3.550532921	1.066157651	NA	NA
ASV_16s_14647	0.224933774	0.15603259	3.55087131	1.237046257	0.990048752	NA
ASV_16s_14648	0.209459515	-0.615869072	3.550977034	0.814654651	0.997291506	NA
ASV_16s_14649	0.244384287	-0.490453008	3.550598439	0.840208637	0.99701163	NA
ASV_16s_14650	0.236902291	-0.350708213	3.550639287	1.072220402	0.993577901	NA
ASV_16s_14651	0.234840577	-0.35071151	3.550626656	1.060371047	0.993795005	NA
ASV_16s_14652	0.222902376	-0.315021115	3.550675984	1.022642666	0.994454962	NA
ASV_16s_14653	0.298724133	0.032064821	3.550735724	1.507314653	0.982055346	NA
ASV_16s_14654	0.214100867	0.305121226	3.550662038	0.844004749	0.996968506	NA
ASV_16s_14655	0.203636038	0.310856382	3.551084186	0.720547176	NA	NA
ASV_16s_14656	0.18290322	0.206725639	3.551022801	0.354402426	0.999824471	NA
ASV_16s_14657	0.25667656	1.096868835	3.551318632	0.559880367	0.999196348	NA
ASV_16s_14658	0.211995865	0.92954363	3.550793511	1.076392607	0.993500326	NA
ASV_16s_14659	0.194782633	0.371148095	3.551545132	0.662094849	NA	NA
ASV_16s_14660	0.252472939	0.703086128	3.550467451	0.982296176	0.99510909	NA
ASV_16s_14661	0.299795982	0.561463872	3.550056402	1.311408583	0.988131611	NA
ASV_16s_14662	0.147598944	-0.386262164	3.551408067	0.480991398	NA	NA
ASV_16s_14663	0.150944906	0.350126724	3.55180068	0.577095181	NA	NA
ASV_16s_14664	0.237889335	0.384409661	3.551285415	0.729376286	NA	NA
ASV_16s_14665	0.235937248	-0.225364548	3.551351718	0.705863888	NA	NA
ASV_16s_14666	0.2340789	0.354649993	3.551752912	0.415234056	NA	NA
ASV_16s_14667	0.264820384	0.355360855	3.551754865	0.43455535	NA	NA
ASV_16s_14668	0.206469206	-1.032115046	3.550515791	0.371794094	NA	NA
ASV_16s_14669	0.150750371	-0.50102422	3.551111265	0.581183197	NA	NA
ASV_16s_14670	0.172186831	0.204070477	3.550721596	0.567948437	NA	NA
ASV_16s_14671	0.271883594	1.290461722	3.550697124	0.745536106	NA	NA
ASV_16s_14672	0.22352556	0.402934892	3.550997492	0.641477927	NA	NA
ASV_16s_14673	0.252776463	0.413332879	3.550852972	0.682734193	NA	NA
ASV_16s_14674	0.229307356	0.405189072	3.550967205	0.650047633	NA	NA
ASV_16s_14675	0.208201681	0.396682306	3.551084883	0.617832542	NA	NA
ASV_16s_14676	0.17186803	0.379333604	3.551324783	0.554974629	NA	NA
ASV_16s_14677	0.17186803	0.379333604	3.551324783	0.554974629	NA	NA
ASV_16s_14678	0.064747986	0.342432489	3.551808782	0.276813141	NA	NA
ASV_16s_14679	0.104477339	-0.421942151	3.551212446	0.403118866	NA	NA
ASV_16s_14680	0.104477339	-0.421942151	3.551212446	0.403118866	NA	NA
ASV_16s_14681	0.358042973	-0.548890129	3.549615722	0.788559815	NA	NA

ASV_16s_14682	0.116287676	-0.448776735	3.551106925	0.434234582	NA	NA
ASV_16s_14683	0.177535942	-0.078597711	3.550822267	0.878723598	NA	NA
ASV_16s_14684	0.177535942	-0.078597711	3.550822267	0.878723598	NA	NA
ASV_16s_14685	0.177535942	-0.078597711	3.550822267	0.878723598	NA	NA
ASV_16s_14686	0.177535942	-0.078597711	3.550822267	0.878723598	NA	NA
ASV_16s_14687	0.184781528	-0.07863825	3.550810247	0.898162696	0.99630842	NA
ASV_16s_14688	0.218409862	0.25688168	3.550851361	1.234596792	NA	NA
ASV_16s_14689	0.132169371	0.22009833	3.550795654	0.579117693	NA	NA
ASV_16s_14690	0.131905438	0.220098662	3.550796091	0.578215981	NA	NA
ASV_16s_14691	0.130408424	-0.41457402	3.55106393	0.593605019	NA	NA
ASV_16s_14692	0.271492303	1.188252948	3.550578533	0.845369373	NA	NA
ASV_16s_14693	0.184968717	-0.067722231	3.550698845	1.106831754	NA	NA
ASV_16s_14694	0.286892702	1.723427015	3.550373192	0.486482843	0.999494486	NA
ASV_16s_14695	0.186455272	-0.068478587	3.5506607	1.138260749	NA	NA
ASV_16s_14696	0.18695079	-0.068296737	3.550636056	1.146480086	NA	NA
ASV_16s_14697	0.202941074	0.237665595	3.550709546	0.777668153	0.997665234	NA
ASV_16s_14698	0.181005548	0.691082803	3.550872244	1.052786361	NA	NA
ASV_16s_14699	0.189428382	-0.067885425	3.550570161	1.16442387	NA	NA
ASV_16s_14700	0.197474883	0.356467557	3.551804329	0.600113571	NA	NA
ASV_16s_14701	0.202763063	-0.067898337	3.550484224	1.189795205	NA	NA
ASV_16s_14702	0.318490618	-0.763995505	3.549795746	0.81634769	0.997273519	NA
ASV_16s_14703	0.259584525	0.900426621	3.551680147	0.550909173	0.999237899	NA
ASV_16s_14704	0.218395859	0.523438932	3.550958116	0.646204161	0.998715803	NA
ASV_16s_14705	0.263038479	-0.017776361	3.55143851	0.707679183	NA	NA
ASV_16s_14706	0.216677939	0.319958175	3.551053841	0.792554051	0.997519201	NA
ASV_16s_14707	0.301609902	1.145442584	3.550710482	0.757305633	0.997855646	NA
ASV_16s_14708	0.215727245	0.929626248	3.550873512	0.646876273	NA	NA
ASV_16s_14709	0.053701877	0.292678295	3.551898003	0.289335021	NA	NA
ASV_16s_14710	0.08888094	0.285638104	3.551779705	0.418377146	NA	NA
ASV_16s_14711	0.124632308	0.282370038	3.551686605	0.522590243	NA	NA
ASV_16s_14712	0.263672332	-0.570223744	3.550864087	0.679612626	0.998487527	NA
ASV_16s_14713	0.303262317	0.817162924	3.49047564	0.892904637	0.996376245	NA
ASV_16s_14714	0.245373051	0.323708774	3.551007473	0.719025815	0.998185242	NA
ASV_16s_14715	0.235808134	-0.546531381	3.550091707	0.655337774	NA	NA
ASV_16s_14716	0.285847075	0.343029782	3.550051148	1.49339799	NA	NA
ASV_16s_14717	0.196574349	-0.028744321	3.550932136	0.987559645	NA	NA
ASV_16s_14718	0.196574349	-0.028744321	3.550932136	0.987559645	NA	NA
ASV_16s_14719	0.246553633	0.334331745	3.550238902	1.343315644	NA	NA
ASV_16s_14720	0.263876597	0.365529263	3.550468531	1.227084715	NA	NA
ASV_16s_14721	0.271960648	0.581256789	3.550950319	1.081019323	NA	NA
ASV_16s_14722	0.164046956	-0.073400049	3.550880483	0.864854664	NA	NA
ASV_16s_14723	0.164046956	-0.073400049	3.550880483	0.864854664	NA	NA
ASV_16s_14724	0.271101375	0.414191189	3.550794734	1.169861682	0.991604351	NA
ASV_16s_14725	0.161099306	-0.073376465	3.550887158	0.856192464	NA	NA
ASV_16s_14726	0.247059041	0.579159605	3.550823781	1.28383959	NA	NA
ASV_16s_14727	0.244522701	0.344020291	3.550271661	1.294794374	NA	NA
ASV_16s_14728	0.224236778	0.360336357	3.551046619	1.036264817	NA	NA
ASV_16s_14729	0.224236778	0.360336357	3.551046619	1.036264817	NA	NA
ASV_16s_14730	0.224236778	0.360336357	3.551046619	1.036264817	NA	NA
ASV_16s_14731	0.216948098	0.356145095	3.551250153	0.748229885	NA	NA
ASV_16s_14732	0.214032626	0.344246758	3.551125865	0.743865725	NA	NA
ASV_16s_14733	0.246067398	0.342729118	3.550989869	0.806532545	0.997376714	NA
ASV_16s_14734	0.228224715	0.329408763	3.550981552	0.83625139	0.997056155	NA
ASV_16s_14735	0.199338748	0.360319253	3.551102773	0.977681625	NA	NA
ASV_16s_14736	0.199338748	0.360319253	3.551102773	0.977681625	NA	NA
ASV_16s_14737	0.199338748	0.360319253	3.551102773	0.977681625	NA	NA
ASV_16s_14738	0.199338748	0.360319253	3.551102773	0.977681625	NA	NA
ASV_16s_14739	0.199338748	0.360319253	3.551102773	0.977681625	NA	NA
ASV_16s_14740	0.234380101	0.315939634	3.550893438	1.019487604	0.994508019	NA
ASV_16s_14741	0.223258289	0.315939654	3.55090861	0.995146534	NA	NA
ASV_16s_14742	0.212650224	0.315939675	3.550924558	0.970833006	0.995285418	NA
ASV_16s_14743	0.211623638	0.315939678	3.550926186	0.968422073	0.995321975	NA
ASV_16s_14744	0.239657628	0.315939626	3.550886751	1.030430339	0.994322603	NA
ASV_16s_14745	0.208201681	0.315939685	3.550931726	0.960314179	NA	NA
ASV_16s_14746	0.322152659	1.167001608	3.550409903	0.832717662	0.997095545	NA
ASV_16s_14747	0.240186863	1.096342859	3.550744796	0.754797838	NA	NA
ASV_16s_14748	0.275425241	0.734261721	3.550966161	1.076494135	NA	NA

ASV_16s_14749	0.275425241	0.734261721	3.550966161	1.076494135	NA	NA
ASV_16s_14750	0.275425241	0.734261721	3.550966161	1.076494135	NA	NA
ASV_16s_14751	0.136433453	0.730366393	3.551353203	0.738584326	NA	NA
ASV_16s_14752	0.136433453	0.730366393	3.551353203	0.738584326	NA	NA
ASV_16s_14753	0.255317392	0.693185549	3.55084269	0.997715298	0.994865308	NA
ASV_16s_14754	0.23470268	0.693098875	3.550868065	0.955683449	0.995512099	NA
ASV_16s_14755	0.270536447	0.69323814	3.550826416	1.02681485	0.9943843	NA
ASV_16s_14756	0.263321693	0.693215621	3.550833892	1.013239917	NA	NA
ASV_16s_14757	0.263321693	0.693215621	3.550833892	1.013239917	NA	NA
ASV_16s_14758	0.263321693	0.693215621	3.550833892	1.013239917	NA	NA
ASV_16s_14759	0.228245322	0.693068534	3.550876954	0.941815236	0.995713335	NA
ASV_16s_14760	0.252373756	0.693174017	3.550846065	0.991887257	0.994958347	NA
ASV_16s_14761	0.208201681	0.692963798	3.550908025	0.896483308	NA	NA
ASV_16s_14762	0.208201681	0.692963798	3.550908025	0.896483308	NA	NA
ASV_16s_14763	0.175423728	0.28970025	3.551674019	0.62667103	NA	NA
ASV_16s_14764	0.175423728	0.28970025	3.551674019	0.62667103	NA	NA
ASV_16s_14765	0.175423728	0.28970025	3.551674019	0.62667103	NA	NA
ASV_16s_14766	0.175423728	0.28970025	3.551674019	0.62667103	NA	NA
ASV_16s_14767	0.175423728	0.28970025	3.551674019	0.62667103	NA	NA
ASV_16s_14768	0.177753997	0.020670057	3.551138508	0.928778244	NA	NA
ASV_16s_14769	0.178789673	-0.061757937	3.550997232	1.012197284	0.99462937	NA
ASV_16s_14770	0.179566429	-0.116498537	3.550918148	1.056267705	0.993869083	NA
ASV_16s_14771	0.175514993	-0.10431948	3.550967926	1.039354624	0.994168465	NA
ASV_16s_14772	0.179566429	-0.116498537	3.550918148	1.056267705	0.993869083	NA
ASV_16s_14773	0.179566429	-0.116498537	3.550918148	1.056267705	0.993869083	NA
ASV_16s_14774	0.179825348	-0.133964562	3.5508966	1.067726699	0.993660794	NA
ASV_16s_14775	0.179825348	-0.133964562	3.5508966	1.067726699	0.993660794	NA
ASV_16s_14776	0.175526401	-0.138629176	3.550928627	1.060570118	0.993791397	NA
ASV_16s_14777	0.180602105	-0.184768605	3.550843906	1.094406224	0.993158576	NA
ASV_16s_14778	0.180602105	-0.184768605	3.550843906	1.094406224	0.993158576	NA
ASV_16s_14779	0.179094224	-0.213192891	3.550837149	1.096528507	0.99311758	NA
ASV_16s_14780	0.177574935	-0.209662414	3.550852092	1.091190075	0.993220407	NA
ASV_16s_14781	0.178593498	-0.228093334	3.550833126	1.090720423	0.993229406	NA
ASV_16s_14782	0.181378861	-0.234210901	3.550807007	1.102294829	0.993005408	NA
ASV_16s_14783	0.175554921	-0.221228262	3.550862743	1.079091007	0.993449839	NA
ASV_16s_14784	0.18163778	-0.250564863	3.550797842	1.09578181	0.993132021	NA
ASV_16s_14785	0.178092773	-0.24305884	3.550830352	1.082301659	0.993389444	NA
ASV_16s_14786	0.193200495	-0.287366403	3.550688872	1.120705644	0.992639549	NA
ASV_16s_14787	0.175566329	-0.253883413	3.550847805	1.064311126	0.993723341	NA
ASV_16s_14788	0.175572033	-0.27034997	3.550842129	1.054762944	0.993896107	NA
ASV_16s_14789	0.180129899	-0.27958128	3.550800185	1.071204651	0.993596698	NA
ASV_16s_14790	0.17962347	-0.27857363	3.55080473	1.069399894	0.993630009	NA
ASV_16s_14791	0.175572033	-0.27034997	3.550842129	1.054762944	0.993896107	NA
ASV_16s_14792	0.179370255	-0.27806809	3.550807013	1.068494975	0.99364667	NA
ASV_16s_14793	0.182414536	-0.300320483	3.550775826	1.067630147	0.993662567	NA
ASV_16s_14794	0.179375959	-0.294546355	3.550802272	1.057246142	0.993851471	NA
ASV_16s_14795	0.203857412	-0.380610576	3.550602476	1.084300184	0.993351672	NA
ASV_16s_14796	0.17559485	-0.338168261	3.550828846	1.002571337	NA	NA
ASV_16s_14797	0.17559485	-0.338168261	3.550828846	1.002571337	NA	NA
ASV_16s_14798	0.17965199	-0.362827362	3.550796388	0.998133748	0.994858586	NA
ASV_16s_14799	0.213593668	-0.406714916	3.550532737	1.087282479	0.993295053	NA
ASV_16s_14800	0.175606258	-0.37318296	3.550829368	0.967491741	NA	NA
ASV_16s_14801	0.175606258	-0.37318296	3.550829368	0.967491741	NA	NA
ASV_16s_14802	0.06271187	-0.255368064	3.55174113	0.336938524	NA	NA
ASV_16s_14803	0.269353096	-0.678129401	3.550383869	0.789989548	0.997544776	NA
ASV_16s_14804	0.259656603	-0.675423299	3.550438182	0.77789507	0.997663051	NA
ASV_16s_14805	0.242989958	-0.667236374	3.55052662	0.755779363	0.99786949	NA
ASV_16s_14806	0.229133059	-0.658422008	3.550602197	0.736133618	0.998042437	NA
ASV_16s_14807	0.22588754	-0.655848684	3.550619188	0.731340476	0.998083172	NA
ASV_16s_14808	0.219396502	-0.650585582	3.550654492	0.721585905	NA	NA
ASV_16s_14809	0.219396502	-0.650585582	3.550654492	0.721585905	NA	NA
ASV_16s_14810	0.195902997	-0.627838227	3.550792996	0.683493186	0.998459377	NA
ASV_16s_14811	0.186557238	-0.614174256	3.550841947	0.666678623	NA	NA
ASV_16s_14812	0.211048796	-0.6432536	3.550702219	0.708515903	0.998269446	NA
ASV_16s_14813	0.180226868	-0.604256896	3.550876847	0.654769823	0.998659643	NA
ASV_16s_14814	0.175669002	-0.596908305	3.550903208	0.645975195	NA	NA
ASV_16s_14815	0.175669002	-0.596908305	3.550903208	0.645975195	NA	NA

ASV_16s_14816	0.175669002	-0.596908305	3.550903208	0.645975195	NA	NA
ASV_16s_14817	0.175669002	-0.596908305	3.550903208	0.645975195	NA	NA
ASV_16s_14818	0.236162747	1.439097075	3.550809176	0.531371093	0.999323313	NA
ASV_16s_14819	0.068278379	0.072038078	3.55090814	0.512758204	0.999398412	NA
ASV_16s_14820	0.059014763	0.220302634	3.551065214	0.266518778	NA	NA
ASV_16s_14821	0.059014763	0.220302634	3.551065214	0.266518778	NA	NA
ASV_16s_14822	0.078023093	0.22021305	3.550946887	0.362463818	NA	NA
ASV_16s_14823	0.224875086	-0.39847174	3.550818287	1.062930279	0.993748516	NA
ASV_16s_14824	0.208171311	-0.056456108	3.550642088	1.089480148	0.993253136	NA
ASV_16s_14825	0.180902512	-0.096017579	3.550784515	0.897417485	0.996318083	NA
ASV_16s_14826	0.220073036	-0.682671464	3.550606027	0.771516111	0.997723891	NA
ASV_16s_14827	0.248106532	1.161656142	3.550813932	0.639083313	NA	NA
ASV_16s_14828	0.236588053	-0.697295611	3.550368068	0.909962421	0.996153225	NA
ASV_16s_14829	0.202188584	-0.250221336	3.55041435	0.799795187	0.997446043	NA
ASV_16s_14830	0.192391095	-0.006848378	3.550910471	0.588348204	NA	NA
ASV_16s_14831	0.098006597	-0.205038628	3.550627452	0.433327894	0.99965585	NA
ASV_16s_14832	0.223544106	-0.673429088	3.550439798	0.898361648	0.996305838	NA
ASV_16s_14833	0.182078191	0.232719313	3.550777162	1.026761802	0.994385202	NA
ASV_16s_14834	0.197087755	-0.89153024	3.550602227	0.524974239	NA	NA
ASV_16s_14835	0.188631768	-0.201714669	3.550552676	0.827703061	0.997150848	NA
ASV_16s_14836	0.193166625	0.679408401	3.551280354	0.823604536	NA	NA
ASV_16s_14837	0.206488898	0.398296183	3.550807262	1.084779746	0.993342588	NA
ASV_16s_14838	0.234397076	-0.668971003	3.550324923	0.97326005	NA	NA
ASV_16s_14839	0.202752024	0.231781863	3.550765125	0.735886081	NA	NA
ASV_16s_14840	0.126319453	-0.20996043	3.550429494	0.681438009	NA	NA
ASV_16s_14841	0.203033883	1.031923918	3.550152299	0.496983066	NA	NA
ASV_16s_14842	0.216369326	-0.609634517	3.550420346	0.970050069	0.99529731	NA
ASV_16s_14843	0.215411552	-0.974212391	3.550517749	0.601900505	NA	NA
ASV_16s_14844	0.209386962	0.064144465	3.550348147	1.104541234	0.992961397	NA
ASV_16s_14845	0.228479827	-0.619443463	3.550514861	0.910038948	0.996152205	NA
ASV_16s_14846	0.232163384	0.278336959	3.551352287	0.720991654	0.998169196	NA
ASV_16s_14847	0.212497705	-0.573580496	3.550455743	0.981422262	0.99512268	NA
ASV_16s_14848	0.236650347	-0.199489111	3.55026583	0.985893044	0.995052902	NA
ASV_16s_14849	0.204861925	-0.502945715	3.550555334	0.984060415	NA	NA
ASV_16s_14850	0.170540744	0.314611037	3.550834928	0.593918224	0.999024791	NA
ASV_16s_14851	0.185906313	-0.37261191	3.550958935	0.997795476	NA	NA
ASV_16s_14852	0.230871229	-0.516635461	3.550437487	1.068333524	NA	NA
ASV_16s_14853	0.214694634	-0.493817544	3.550518686	1.017473159	0.994541724	NA
ASV_16s_14854	0.215960117	-0.491144149	3.551025816	0.842478001	0.996985899	NA
ASV_16s_14855	0.204229987	-0.482750554	3.550572067	0.985028376	NA	NA
ASV_16s_14856	0.243859559	-0.548730475	3.550347888	1.11236264	0.992806801	NA
ASV_16s_14857	0.229566699	-0.495497459	3.550464925	1.066970319	0.993674679	NA
ASV_16s_14858	0.222810798	-0.464320999	3.550520935	1.047308572	NA	NA
ASV_16s_14859	0.222689584	-0.475865339	3.550508214	1.043534559	0.994095363	NA
ASV_16s_14860	0.184002419	-0.452984295	3.55091842	0.726172893	0.998126455	NA
ASV_16s_14861	0.265478083	-0.048834167	3.550690128	1.28472914	0.988843439	NA
ASV_16s_14862	0.259438627	-0.335350082	3.551069783	0.639516861	0.998758537	NA
ASV_16s_14863	0.221424399	-0.472650572	3.551043557	0.712535074	0.998237558	NA
ASV_16s_14864	0.242056039	0.240448908	3.55070894	0.926050235	NA	NA
ASV_16s_14865	0.216237648	0.22104493	3.54963972	1.052168378	0.993942524	NA
ASV_16s_14866	0.210801439	-0.080638175	3.550980249	0.688147245	NA	NA
ASV_16s_14867	0.147261279	0.330340866	3.550750484	0.88619254	0.996461641	NA
ASV_16s_14868	0.243707746	0.733361492	3.550461864	0.939768378	0.995742532	NA
ASV_16s_14869	0.219477676	-0.326006038	3.550416386	0.414369408	NA	NA
ASV_16s_14870	0.189759701	1.002819031	3.551196172	0.625975964	NA	NA
ASV_16s_14871	0.24163253	1.034158757	3.550871363	0.733722918	NA	NA
ASV_16s_14872	0.167065498	0.20931795	3.551022427	0.33663789	0.999852375	NA
ASV_16s_14873	0.339554112	0.271671876	3.550663451	0.844817897	NA	NA
ASV_16s_14874	0.200782656	1.214824715	3.551003098	0.639553507	NA	NA
ASV_16s_14875	0.207412498	0.980979884	3.551221877	0.659852046	NA	NA
ASV_16s_14876	0.230448858	0.382928547	3.551330072	0.718751941	NA	NA
ASV_16s_14877	0.254886318	0.388304307	3.551184353	0.752589653	NA	NA
ASV_16s_14878	0.254886318	0.388304307	3.551184353	0.752589653	NA	NA
ASV_16s_14879	0.14751434	0.349013441	3.551818006	0.569504011	NA	NA
ASV_16s_14880	0.14751434	0.349013441	3.551818006	0.569504011	NA	NA
ASV_16s_14881	0.15383858	0.162984626	3.551415886	0.782777005	NA	NA
ASV_16s_14882	0.268114676	0.391842586	3.55110869	0.769534799	NA	NA

ASV_16s_14883	0.340669695	-0.787202	3.54998658	0.872104982	NA	NA
ASV_16s_14884	0.128085369	0.339553308	3.552037087	0.309628741	0.999888682	NA
ASV_16s_14885	0.245278938	0.116503111	3.551393029	0.571454615	NA	NA
ASV_16s_14886	0.212077393	-0.552501638	3.550555139	0.691192352	NA	NA
ASV_16s_14887	0.208313029	-0.976180438	3.550653246	0.437574115	NA	NA
ASV_16s_14888	0.149095603	-0.410538006	3.55070893	0.623133313	0.998859183	NA
ASV_16s_14889	0.07364696	0.438602256	3.551823937	0.268928412	NA	NA
ASV_16s_14890	0.113997761	1.066051375	3.551222663	0.394278468	NA	NA
ASV_16s_14891	0.113997761	1.066051375	3.551222663	0.394278468	NA	NA
ASV_16s_14892	0.141279215	1.136815135	3.551064869	0.461158565	NA	NA
ASV_16s_14893	0.195432321	1.232325033	3.550809607	0.570122197	NA	NA
ASV_16s_14894	0.217691934	1.003412019	3.550544518	1.009070312	0.99468089	NA
ASV_16s_14895	0.234786271	0.848890798	3.550416012	1.15302471	0.991968583	NA
ASV_16s_14896	0.185962161	0.386554832	3.551232869	0.580699677	0.999094079	NA
ASV_16s_14897	0.167961939	0.378003569	3.551347115	0.547362629	NA	NA
ASV_16s_14898	0.167961939	0.378003569	3.551347115	0.547362629	NA	NA
ASV_16s_14899	0.167961939	0.378003569	3.551347115	0.547362629	NA	NA
ASV_16s_14900	0.063276441	0.342337905	3.551814133	0.271589927	NA	NA
ASV_16s_14901	0.102102854	-0.416348622	3.551232456	0.396658085	NA	NA
ASV_16s_14902	0.102102854	-0.416348622	3.551232456	0.396658085	NA	NA
ASV_16s_14903	0.102102854	-0.416348622	3.551232456	0.396658085	NA	NA
ASV_16s_14904	0.250466984	-0.547276562	3.550011071	0.67442679	NA	NA
ASV_16s_14905	0.330576663	-0.548574015	3.549692483	0.762926922	NA	NA
ASV_16s_14906	0.242547292	0.686440282	3.550095531	1.034237804	NA	NA
ASV_16s_14907	0.124001574	-0.465115053	3.551032306	0.453439287	NA	NA
ASV_16s_14908	0.124001574	-0.465115053	3.551032306	0.453439287	NA	NA
ASV_16s_14909	0.124001574	-0.465115053	3.551032306	0.453439287	NA	NA
ASV_16s_14910	0.124001574	-0.465115053	3.551032306	0.453439287	NA	NA
ASV_16s_14911	0.124001574	-0.465115053	3.551032306	0.453439287	NA	NA
ASV_16s_14912	0.124001574	-0.465115053	3.551032306	0.453439287	NA	NA
ASV_16s_14913	0.173501034	-0.078573872	3.550829408	0.867555095	NA	NA
ASV_16s_14914	0.113708997	0.220125261	3.550831113	0.512711094	NA	NA
ASV_16s_14915	0.122335894	0.220111565	3.550813221	0.544572269	0.999266357	NA
ASV_16s_14916	0.129165521	0.220102204	3.550800744	0.568741728	NA	NA
ASV_16s_14917	0.220450879	0.310940218	3.550884866	0.965940058	NA	NA
ASV_16s_14918	0.210055139	0.310941168	3.550899502	0.941831785	0.995713098	NA
ASV_16s_14919	0.192223162	0.310943033	3.550928265	0.898000289	NA	NA
ASV_16s_14920	0.219753101	0.310940279	3.550885805	0.96435509	NA	NA
ASV_16s_14921	0.136065683	0.59594188	3.550751821	0.529321272	NA	NA
ASV_16s_14922	0.135583146	1.301487811	3.551240736	0.265466015	NA	NA
ASV_16s_14923	0.208375916	1.34848891	3.551003966	0.505999569	NA	NA
ASV_16s_14924	0.237523589	0.331576422	3.551950414	0.415674686	NA	NA
ASV_16s_14925	0.185118191	-0.068098904	3.550720979	1.083941871	0.993358454	NA
ASV_16s_14926	0.180438291	-0.067844114	3.550725165	1.078929276	NA	NA
ASV_16s_14927	0.214431547	0.533401309	3.550867628	0.952223591	0.995562862	NA
ASV_16s_14928	0.163184632	-0.948347753	3.550513241	0.297184767	0.999903105	NA
ASV_16s_14929	0.184402437	-0.067999499	3.550610529	1.144957156	NA	NA
ASV_16s_14930	0.209634563	0.146481825	3.550297024	1.428963358	0.984667582	NA
ASV_16s_14931	0.159957083	0.313406477	3.551798233	0.33721626	0.999851518	NA
ASV_16s_14932	0.185393474	-0.067771781	3.550605331	1.140468787	NA	NA
ASV_16s_14933	0.18264918	0.323833018	3.55142463	0.580475818	NA	NA
ASV_16s_14934	0.203844813	0.004824488	3.550681854	0.887174573	NA	NA
ASV_16s_14935	0.197628346	-0.067401193	3.550579004	1.139246184	NA	NA
ASV_16s_14936	0.195028557	-0.049570249	3.550786217	1.067536517	NA	NA
ASV_16s_14937	0.228909548	0.322551438	3.551200039	0.654447807	NA	NA
ASV_16s_14938	0.19637588	0.714960586	3.55132544	0.845763274	0.996948393	NA
ASV_16s_14939	0.344944066	0.289407231	3.551117897	0.883055464	NA	NA
ASV_16s_14940	0.146271474	0.28078112	3.5516465	0.576848613	NA	NA
ASV_16s_14941	0.146271474	0.28078112	3.5516465	0.576848613	NA	NA
ASV_16s_14942	0.112864454	0.283354587	3.551713089	0.480281312	0.999515528	NA
ASV_16s_14943	0.24499472	0.279789413	3.551452705	0.762400676	NA	NA
ASV_16s_14944	0.171074416	-0.493136582	3.551255222	0.624055607	NA	NA
ASV_16s_14945	0.39629812	-0.565257086	3.549705382	0.891681442	NA	NA
ASV_16s_14946	0.361890142	0.122048465	3.550639218	1.31007375	NA	NA
ASV_16s_14947	0.322023561	0.359294176	3.551543087	0.586111844	0.999066125	NA
ASV_16s_14948	0.224360199	0.321584815	3.55102231	0.721598157	0.998164227	NA
ASV_16s_14949	0.230448858	-0.545768024	3.550123076	0.647946466	NA	NA

ASV_16s_14950	0.234683302	1.139359132	3.550343087	0.998359954	NA	NA
ASV_16s_14951	0.238701219	1.057181704	3.550462564	1.017820887	0.994535915	NA
ASV_16s_14952	0.219207202	1.009721473	3.550592566	0.954076791	NA	NA
ASV_16s_14953	0.211005071	1.307413629	3.550708969	0.513135615	NA	NA
ASV_16s_14954	0.2155755	0.859359669	3.550802297	0.933739803	NA	NA
ASV_16s_14955	0.145606032	-0.078371795	3.550889427	0.784407324	NA	NA
ASV_16s_14956	0.145606032	-0.078371795	3.550889427	0.784407324	NA	NA
ASV_16s_14957	0.202734852	-0.115991772	3.550374227	1.153870344	NA	NA
ASV_16s_14958	0.33846086	0.897456901	3.549898101	1.387047878	0.985964676	NA
ASV_16s_14959	0.324697619	0.417232182	3.550815837	1.9447304	0.962835921	NA
ASV_16s_14960	0.321403774	-0.029227451	3.550772349	1.233785773	NA	NA
ASV_16s_14961	0.262651282	0.409656393	3.550875382	1.699940875	0.97457072	NA
ASV_16s_14962	0.19210675	-0.028715789	3.550941695	0.976236039	NA	NA
ASV_16s_14963	0.256400198	0.416686413	3.550658781	1.14585864	0.99212054	NA
ASV_16s_14964	0.219506411	-0.073726947	3.550788165	1.007079988	NA	NA
ASV_16s_14965	0.243208651	0.260006154	3.550602351	1.502957203	0.982207105	NA
ASV_16s_14966	0.190694512	0.301828602	3.550596653	1.096707544	NA	NA
ASV_16s_14967	0.217865751	-0.07371674	3.550790255	1.003213397	0.994776533	NA
ASV_16s_14968	0.261370626	0.58122335	3.55096009	1.057893828	NA	NA
ASV_16s_14969	0.268280164	0.351343171	3.55028386	1.291844336	NA	NA
ASV_16s_14970	0.257147922	0.405940184	3.550794214	1.129868318	NA	NA
ASV_16s_14971	0.219140487	0.360333163	3.551057101	1.02471049	NA	NA
ASV_16s_14972	0.346914649	0.363396958	3.550742279	0.948390932	0.99561866	NA
ASV_16s_14973	0.282699152	0.334248261	3.550905739	0.918430393	0.996039283	NA
ASV_16s_14974	0.332507993	0.36335899	3.550607475	0.924066858	NA	NA
ASV_16s_14975	0.194808322	0.360315661	3.551114542	0.966291627	NA	NA
ASV_16s_14976	0.201725276	0.407544366	3.55107149	1.183142261	NA	NA
ASV_16s_14977	0.208602759	0.315939698	3.550931083	0.961214136	0.995430177	NA
ASV_16s_14978	0.203469825	0.31593971	3.550939709	0.948863768	NA	NA
ASV_16s_14979	0.203469825	0.31593971	3.550939709	0.948863768	NA	NA
ASV_16s_14980	0.203469825	0.31593971	3.550939709	0.948863768	NA	NA
ASV_16s_14981	0.203469825	0.31593971	3.550939709	0.948863768	NA	NA
ASV_16s_14982	0.203469825	0.31593971	3.550939709	0.948863768	NA	NA
ASV_16s_14983	0.203469825	0.499664958	3.550900903	1.441231343	0.984274785	NA
ASV_16s_14984	0.193771849	0.687811438	3.550896857	0.836762583	NA	NA
ASV_16s_14985	0.193771849	0.687811438	3.550896857	0.836762583	NA	NA
ASV_16s_14986	0.297385398	1.173275184	3.55052453	0.810642345	NA	NA
ASV_16s_14987	0.289860112	1.176136314	3.55067749	0.82185986	NA	NA
ASV_16s_14988	0.222543912	0.733939678	3.551058416	0.969589532	0.995304296	NA
ASV_16s_14989	0.269165576	0.734229725	3.550975206	1.064877278	NA	NA
ASV_16s_14990	0.269165576	0.734229725	3.550975206	1.064877278	NA	NA
ASV_16s_14991	0.269165576	0.734229725	3.550975206	1.064877278	NA	NA
ASV_16s_14992	0.269165576	0.734229725	3.550975206	1.064877278	NA	NA
ASV_16s_14993	0.269165576	0.734229725	3.550975206	1.064877278	NA	NA
ASV_16s_14994	0.287252333	1.104767969	3.550600953	0.827375814	0.997154433	NA
ASV_16s_14995	0.133332693	0.729799488	3.551369869	0.728324888	NA	NA
ASV_16s_14996	0.133332693	0.729799488	3.551369869	0.728324888	NA	NA
ASV_16s_14997	0.25023678	0.693165689	3.550848561	0.987631096	NA	NA
ASV_16s_14998	0.269361698	0.693234733	3.550827619	1.024569034	0.994422407	NA
ASV_16s_14999	0.218696275	0.693020621	3.550891066	0.920602873	0.996009703	NA
ASV_16s_15000	0.255520746	0.693186523	3.550842462	0.998103748	0.994859068	NA
ASV_16s_15001	0.257337109	0.69319349	3.550840423	1.001656382	NA	NA
ASV_16s_15002	0.257337109	0.69319349	3.550840423	1.001656382	NA	NA
ASV_16s_15003	0.203469825	0.692934722	3.550916264	0.885172248	NA	NA
ASV_16s_15004	0.203469825	0.692934722	3.550916264	0.885172248	NA	NA
ASV_16s_15005	0.203469825	0.692934722	3.550916264	0.885172248	NA	NA
ASV_16s_15006	0.171344106	0.290772962	3.551696023	0.618728996	NA	NA
ASV_16s_15007	0.171344106	0.290772962	3.551696023	0.618728996	NA	NA
ASV_16s_15008	0.171344106	0.290772962	3.551696023	0.618728996	NA	NA
ASV_16s_15009	0.171401147	0.007160425	3.551166372	0.927239918	NA	NA
ASV_16s_15010	0.171418259	-0.053217808	3.55106854	0.983456129	0.995091015	NA
ASV_16s_15011	0.17496897	-0.084742196	3.550997416	1.014873463	0.994585025	NA
ASV_16s_15012	0.175227889	-0.103369958	3.550971822	1.029090006	0.994345525	NA
ASV_16s_15013	0.181503383	-0.13851762	3.550878156	1.06699356	0.993674253	NA
ASV_16s_15014	0.190558536	-0.161209479	3.550779138	1.106465492	0.99292356	NA
ASV_16s_15015	0.205177365	-0.218021204	3.550606386	1.179426737	0.991392903	NA
ASV_16s_15016	0.171446779	-0.143905932	3.550958639	1.044955577	0.994070378	NA

ASV_16s_15017	0.171452483	-0.161087373	3.550942688	1.053189094	0.99392429	NA
ASV_16s_15018	0.176263564	-0.173845506	3.5508894	1.070537576	0.993609023	NA
ASV_16s_15019	0.176781402	-0.207763483	3.550860204	1.081647608	0.993401775	NA
ASV_16s_15020	0.171463891	-0.194875683	3.550915725	1.064539861	0.993719165	NA
ASV_16s_15021	0.177040321	-0.2245716	3.55084828	1.081679318	0.993401178	NA
ASV_16s_15022	0.18428939	-0.255956631	3.550774683	1.099154681	0.993066635	NA
ASV_16s_15023	0.171481004	-0.244955081	3.550887404	1.048492752	0.994007895	NA
ASV_16s_15024	0.174519581	-0.251613361	3.55085795	1.060213927	0.993797852	NA
ASV_16s_15025	0.190863087	-0.299075461	3.550706262	1.099352005	0.993062798	NA
ASV_16s_15026	0.184832786	-0.288673074	3.55075951	1.081970362	0.993395692	NA
ASV_16s_15027	0.190733402	-0.298880467	3.550707312	1.10090533	0.993032542	NA
ASV_16s_15028	0.177817077	-0.274914639	3.550821329	1.062794695	0.993750985	NA
ASV_16s_15029	0.188409056	-0.311058908	3.55072569	1.082762233	0.993380751	NA
ASV_16s_15030	0.175037419	-0.286007363	3.550841897	1.041950362	0.994123137	NA
ASV_16s_15031	0.171498116	-0.295592992	3.550868735	1.01738691	0.994543164	NA
ASV_16s_15032	0.17732776	-0.324268695	3.550815291	1.023476648	0.994440883	NA
ASV_16s_15033	0.191781596	-0.34864096	3.550696755	1.063863514	0.993731509	NA
ASV_16s_15034	0.171509524	-0.330121855	3.550862232	0.989256953	0.99499998	NA
ASV_16s_15035	0.175572369	-0.373091348	3.55082975	0.967308188	0.995338803	NA
ASV_16s_15036	0.177896934	-0.533343587	3.550867301	0.762119681	0.997811592	NA
ASV_16s_15037	0.061253455	-0.24815747	3.551749083	0.33120878	NA	NA
ASV_16s_15038	0.214294257	-0.646232342	3.550683499	0.713662743	NA	NA
ASV_16s_15039	0.200301552	-0.633068012	3.550767853	0.690984592	0.998404052	NA
ASV_16s_15040	0.195439354	-0.6271851	3.550795622	0.68262594	0.998465698	NA
ASV_16s_15041	0.182218697	-0.607386782	3.550865747	0.658509522	NA	NA
ASV_16s_15042	0.182218697	-0.607386782	3.550865747	0.658509522	NA	NA
ASV_16s_15043	0.182218697	-0.607386782	3.550865747	0.658509522	NA	NA
ASV_16s_15044	0.197401888	-0.629737686	3.550784792	0.686036174	0.998440743	NA
ASV_16s_15045	0.174369039	-0.594706365	3.550910795	0.643385563	NA	NA
ASV_16s_15046	0.171583677	-0.589753068	3.550926011	0.637861707	NA	NA
ASV_16s_15047	0.171583677	-0.589753068	3.550926011	0.637861707	NA	NA
ASV_16s_15048	0.171583677	-0.589753068	3.550926011	0.637861707	NA	NA
ASV_16s_15049	0.171583677	-0.589753068	3.550926011	0.637861707	NA	NA
ASV_16s_15050	0.171583677	-0.589753068	3.550926011	0.637861707	NA	NA
ASV_16s_15051	0.171583677	-0.589753068	3.550926011	0.637861707	NA	NA
ASV_16s_15052	0.054667657	-0.168613317	3.550991456	0.265124076	NA	NA
ASV_16s_15053	0.054667657	-0.168613317	3.550991456	0.265124076	NA	NA
ASV_16s_15054	0.048490613	-0.163882813	3.551034933	0.229437236	NA	NA
ASV_16s_15055	0.076208602	0.220219713	3.550955667	0.353842469	NA	NA
ASV_16s_15056	0.176255098	0.032848398	3.550943231	0.996382966	0.994886673	NA
ASV_16s_15057	0.183205978	-0.390295894	3.550874857	0.640790259	NA	NA
ASV_16s_15058	0.193839294	0.303440676	3.551048479	1.036388163	0.994219994	NA
ASV_16s_15059	0.182580049	0.222283569	3.550921196	1.0069771	0.9947152	NA
ASV_16s_15060	0.193778086	0.171824201	3.55071297	1.054938044	0.993892966	NA
ASV_16s_15061	0.175083921	-0.272621395	3.550765501	0.667629323	NA	NA
ASV_16s_15062	0.235993385	-0.708383166	3.550476952	0.835328086	0.99706648	NA
ASV_16s_15063	0.208917818	-0.475408827	3.550865212	0.978405948	0.995169397	NA
ASV_16s_15064	0.228629289	-0.68809976	3.550433384	0.886571945	0.99645685	NA
ASV_16s_15065	0.215673785	1.346363607	3.550430769	0.355813689	0.999822109	NA
ASV_16s_15066	0.190346608	0.202545328	3.551503613	0.653851125	0.998665743	NA
ASV_16s_15067	0.212162581	-0.48265632	3.550822297	0.966626228	0.995349086	NA
ASV_16s_15068	0.196635687	0.437308155	3.550923494	0.938743332	0.995757106	NA
ASV_16s_15069	0.234428568	-0.691404905	3.550371159	0.925702154	0.99593971	NA
ASV_16s_15070	0.207373567	-0.927597833	3.550535624	0.534896973	0.999308405	NA
ASV_16s_15071	0.214402067	-0.638484128	3.550462895	0.908168991	NA	NA
ASV_16s_15072	0.201766817	0.133269104	3.550430788	0.655316904	0.998656001	NA
ASV_16s_15073	0.228571272	-0.636675915	3.550336829	0.988338594	0.995014464	NA
ASV_16s_15074	0.230655454	-0.628861084	3.550320003	1.006081058	NA	NA
ASV_16s_15075	0.270017206	-1.218839915	3.550061046	0.487750599	0.999490109	NA
ASV_16s_15076	0.214119649	-0.538098244	3.55047829	0.998838864	0.994847245	NA
ASV_16s_15077	0.226590159	-0.582466636	3.550627803	0.851893122	0.996877598	NA
ASV_16s_15078	0.241022843	-0.561859206	3.550760997	0.697516386	0.998354747	NA
ASV_16s_15079	0.210645326	0.608980092	3.550519168	0.521299	0.999364695	NA
ASV_16s_15080	0.2418861	-0.033776901	3.550616386	1.009626	0.994671758	NA
ASV_16s_15081	0.188954446	0.677094283	3.551125577	0.892864955	NA	NA
ASV_16s_15082	0.188954446	-0.098683706	3.550866546	0.948104713	NA	NA
ASV_16s_15083	0.253126273	1.082472609	3.550929709	0.851590662	0.996881116	NA

ASV_16s_15084	0.223895	-0.399251388	3.550591683	1.040595837	NA	NA
ASV_16s_15085	0.209827749	-0.66302575	3.550586608	0.609205969	NA	NA
ASV_16s_15086	0.158950876	1.237341726	3.551389128	0.39939305	0.999737896	NA
ASV_16s_15087	0.234146953	0.680390128	3.550941679	0.983680124	0.99508752	NA
ASV_16s_15088	0.248832156	0.834711299	3.072887267	0.53123473	0.999323885	NA
ASV_16s_15089	0.213774565	-0.222178801	3.551384537	0.319433994	0.999876317	NA
ASV_16s_15090	0.177649522	-0.53922484	3.550824645	0.749500274	NA	NA
ASV_16s_15091	0.219617815	-0.106763209	3.550947587	0.778714312	0.997655161	NA
ASV_16s_15092	0.155630777	-0.778364616	3.551150678	0.41782138	NA	NA
ASV_16s_15093	0.218605197	0.208295275	3.550253903	0.704281673	0.998302622	NA
ASV_16s_15094	0.21399342	-0.867109903	3.550803509	0.511716723	NA	NA
ASV_16s_15095	0.222976372	0.033730147	3.550986591	0.697862793	NA	NA
ASV_16s_15096	0.238987007	0.061940965	3.550303238	1.112525221	NA	NA
ASV_16s_15097	0.210400117	-0.079205249	3.526351317	0.522216921	0.999360997	NA
ASV_16s_15098	0.225089583	1.160415339	3.550876445	0.673704099	NA	NA
ASV_16s_15099	0.294689821	0.345254928	3.551584128	0.459219879	NA	NA
ASV_16s_15100	0.168656027	1.487371895	3.550936535	0.329932769	0.999862056	NA
ASV_16s_15101	0.123924562	1.305239439	3.551364384	0.267007798	0.999932608	NA
ASV_16s_15102	0.185928877	0.367094802	3.551601481	0.64643988	NA	NA
ASV_16s_15103	0.185928877	0.367094802	3.551601481	0.64643988	NA	NA
ASV_16s_15104	0.185928877	0.367094802	3.551601481	0.64643988	NA	NA
ASV_16s_15105	0.229143711	-0.390340467	3.551237287	0.746243285	NA	NA
ASV_16s_15106	0.240453109	0.355117947	3.551921459	0.461345894	NA	NA
ASV_16s_15107	0.251520762	0.84279103	3.550907576	0.847092158	0.996933135	NA
ASV_16s_15108	0.195092645	0.371259828	3.551543344	0.662539338	NA	NA
ASV_16s_15109	0.287001784	-0.570692913	3.550537693	0.921523944	NA	NA
ASV_16s_15110	0.266080814	0.437491059	3.550300596	1.107453595	0.99290408	NA
ASV_16s_15111	0.281124206	-1.27824441	3.549901084	0.423297247	NA	NA
ASV_16s_15112	0.143898081	-0.490573949	3.551176762	0.566347769	NA	NA
ASV_16s_15113	0.122042461	-0.033164533	3.550805554	0.52141304	0.999364237	NA
ASV_16s_15114	0.188353453	-0.537742896	3.550742363	0.652555029	NA	NA
ASV_16s_15115	0.137993652	1.129046831	3.551081398	0.453503138	NA	NA
ASV_16s_15116	0.19425192	0.872471812	3.550658354	0.998904808	0.994846184	NA
ASV_16s_15117	0.279879279	-0.216009371	3.549786046	1.280250387	0.988960287	NA
ASV_16s_15118	0.211653837	-0.09699393	3.550148635	1.116886075	0.992716421	NA
ASV_16s_15119	0.198737969	0.392574125	3.551145129	0.602409576	NA	NA
ASV_16s_15120	0.164055847	0.376365741	3.551367845	0.539576929	NA	NA
ASV_16s_15121	0.135292344	-0.222365412	3.550841281	0.687693246	NA	NA
ASV_16s_15122	0.293392495	-0.342697938	3.549739915	1.116016268	0.992733856	NA
ASV_16s_15123	0.123606695	-0.464121272	3.551036824	0.449454971	NA	NA
ASV_16s_15124	0.241249938	-0.992158664	3.550137466	0.499536584	0.999448176	NA
ASV_16s_15125	0.121117817	-0.459068945	3.551059979	0.44630924	NA	NA
ASV_16s_15126	0.198737969	-0.078708431	3.550789572	0.933698348	NA	NA
ASV_16s_15127	0.198737969	-0.078708431	3.550789572	0.933698348	NA	NA
ASV_16s_15128	0.169466126	-0.07854888	3.550836887	0.85616689	NA	NA
ASV_16s_15129	0.111064602	0.220129862	3.550837163	0.502601729	NA	NA
ASV_16s_15130	0.119972973	0.220115125	3.550817877	0.535944721	0.999303932	NA
ASV_16s_15131	0.120254447	0.220114693	3.550817314	0.536972408	0.999299527	NA
ASV_16s_15132	0.126161672	0.22010626	3.550806075	0.558186934	NA	NA
ASV_16s_15133	0.126161672	0.22010626	3.550806075	0.558186934	NA	NA
ASV_16s_15134	0.214642564	0.310940748	3.550892881	0.952552616	NA	NA
ASV_16s_15135	0.132901365	0.595929716	3.550756622	0.518913069	NA	NA
ASV_16s_15136	0.224572798	0.6879582	3.550850112	0.909037394	NA	NA
ASV_16s_15137	0.167646666	0.438632104	3.551289087	0.400547814	NA	NA
ASV_16s_15138	0.230898408	0.235648309	3.551049934	0.606878139	NA	NA
ASV_16s_15139	0.22015608	1.378958815	3.551001927	0.544925637	NA	NA
ASV_16s_15140	0.311730504	-0.698945385	3.550193101	0.892844825	0.996377012	NA
ASV_16s_15141	0.169068953	0.253229253	3.550804276	0.849933668	0.996900344	NA
ASV_16s_15142	0.176403383	-0.067807096	3.550741688	1.063530302	NA	NA
ASV_16s_15143	0.167782591	0.674654156	3.55167387	0.788983582	0.997554761	NA
ASV_16s_15144	0.230532415	0.664697774	3.5501165	1.118850619	NA	NA
ASV_16s_15145	0.270076292	-1.158523717	3.550511569	0.474598909	0.999534277	NA
ASV_16s_15146	0.179872011	-0.067982587	3.550649195	1.122363994	0.992606015	NA
ASV_16s_15147	0.19074039	-0.068229451	3.550576941	1.159486629	0.991829991	NA
ASV_16s_15148	0.177581471	1.452653299	3.551234962	0.276935493	0.999923716	NA
ASV_16s_15149	0.257846803	0.70580137	3.550417497	1.103787749	0.992976179	NA
ASV_16s_15150	0.19067432	-0.067929074	3.550560312	1.151751191	NA	NA

ASV_16s_15151	0.308422855	1.492852608	3.550658799	0.67714998	0.998505211	NA
ASV_16s_15152	0.214450436	0.052794775	3.551121779	0.986846177	NA	NA
ASV_16s_15153	0.186313749	-0.011795628	3.55101769	1.00027694	NA	NA
ASV_16s_15154	0.246168548	1.097926241	3.550900699	0.684572983	NA	NA
ASV_16s_15155	0.217251762	1.06676043	3.551022464	0.64207267	NA	NA
ASV_16s_15156	0.217251762	1.06676043	3.551022464	0.64207267	NA	NA
ASV_16s_15157	0.158352159	0.361614515	3.551500985	0.841058844	NA	NA
ASV_16s_15158	0.051260882	0.293376415	3.551903385	0.27891199	NA	NA
ASV_16s_15159	0.118967203	0.282828725	3.551698782	0.507277719	NA	NA
ASV_16s_15160	0.200962714	1.235366227	3.551299245	0.405247479	NA	NA
ASV_16s_15161	0.387081885	-0.565159769	3.549729696	0.884074612	NA	NA
ASV_16s_15162	0.274961642	0.35058777	3.551370775	0.524421631	0.999352055	NA
ASV_16s_15163	0.214491298	0.319461102	3.551013821	0.753281277	0.99789202	NA
ASV_16s_15164	0.225089583	-0.544985835	3.55015589	0.640402968	NA	NA
ASV_16s_15165	0.249581803	1.288536227	3.550593598	0.654444058	NA	NA
ASV_16s_15166	0.197786902	0.736934442	3.550226998	0.818228044	0.99725345	NA
ASV_16s_15167	0.262016695	1.244648987	3.550211501	0.443890152	NA	NA
ASV_16s_15168	0.16014201	0.233410095	3.550866667	1.218367345	0.990497826	NA
ASV_16s_15169	0.278460105	0.558795232	3.550896076	1.492865	NA	NA
ASV_16s_15170	0.265595061	0.355280678	3.550292633	1.326159971	0.987726366	NA
ASV_16s_15171	0.187639151	-0.028685864	3.550951707	0.964674526	NA	NA
ASV_16s_15172	0.191867272	0.331332496	3.550531697	1.133942364	NA	NA
ASV_16s_15173	0.203926888	0.278485917	3.550616217	1.117569381	0.992702707	NA
ASV_16s_15174	0.163880777	-0.07339914	3.550880897	0.864225607	0.996731937	NA
ASV_16s_15175	0.156590276	-0.073339162	3.550897885	0.842616649	NA	NA
ASV_16s_15176	0.171295272	-0.073454945	3.550865098	0.885203214	0.996474117	NA
ASV_16s_15177	0.15377661	-0.073314493	3.550904875	0.834063112	NA	NA
ASV_16s_15178	0.213537894	0.346771941	3.550441075	1.195217383	NA	NA
ASV_16s_15179	0.190799985	-0.073581487	3.550829305	0.937905686	NA	NA
ASV_16s_15180	0.190799985	-0.073581487	3.550829305	0.937905686	NA	NA
ASV_16s_15181	0.214044197	0.360329817	3.55106808	1.012905224	NA	NA
ASV_16s_15182	0.214044197	0.360329817	3.55106808	1.012905224	NA	NA
ASV_16s_15183	0.207484385	0.355420915	3.551354455	0.735147826	NA	NA
ASV_16s_15184	0.208586307	0.339132872	3.551111923	0.748008302	0.99793906	NA
ASV_16s_15185	0.278567301	0.636883473	3.550583503	1.029818094	NA	NA
ASV_16s_15186	0.237410306	0.334248775	3.550988602	0.835316338	0.997066612	NA
ASV_16s_15187	0.250801242	0.670615912	3.550977621	1.350737241	NA	NA
ASV_16s_15188	0.190277896	0.360311835	3.551126867	0.954670266	NA	NA
ASV_16s_15189	0.190277896	0.360311835	3.551126867	0.954670266	NA	NA
ASV_16s_15190	0.190277896	0.360311835	3.551126867	0.954670266	NA	NA
ASV_16s_15191	0.190277896	0.360311835	3.551126867	0.954670266	NA	NA
ASV_16s_15192	0.246542039	0.315939636	3.550878419	1.044825937	NA	NA
ASV_16s_15193	0.237436501	0.315939545	3.550889536	1.025892036	0.994399978	NA
ASV_16s_15194	0.244928148	0.315939535	3.550880343	1.041441765	0.994132036	NA
ASV_16s_15195	0.216725832	0.315939577	3.550918279	0.980170467	0.995142103	NA
ASV_16s_15196	0.21559502	0.315939579	3.550920005	0.977565487	0.995182363	NA
ASV_16s_15197	0.214568433	0.31593958	3.550921589	0.975183584	0.995218987	NA
ASV_16s_15198	0.211496052	0.315939586	3.55092642	0.967997785	0.995328389	NA
ASV_16s_15199	0.205581881	0.315939717	3.550936123	0.953921946	0.99553799	NA
ASV_16s_15200	0.2332362	0.548200803	3.550856707	1.501832577	0.982246148	NA
ASV_16s_15201	0.226298095	0.315939561	3.550904369	1.001317078	0.994807262	NA
ASV_16s_15202	0.233339509	0.315939551	3.550894851	1.016722818	0.994554244	NA
ASV_16s_15203	0.198737969	0.315939734	3.550948069	0.937172976	NA	NA
ASV_16s_15204	0.198737969	0.315939734	3.550948069	0.937172976	NA	NA
ASV_16s_15205	0.198737969	0.315939734	3.550948069	0.937172976	NA	NA
ASV_16s_15206	0.310686106	0.734413316	3.550922084	1.137161339	NA	NA
ASV_16s_15207	0.275419772	0.734260945	3.550966205	1.076358471	0.993500963	NA
ASV_16s_15208	0.262905911	0.734197195	3.55098468	1.052997582	NA	NA
ASV_16s_15209	0.262905911	0.734197195	3.55098468	1.052997582	NA	NA
ASV_16s_15210	0.262905911	0.734197195	3.55098468	1.052997582	NA	NA
ASV_16s_15211	0.262905911	0.734197195	3.55098468	1.052997582	NA	NA
ASV_16s_15212	0.262905911	0.734197195	3.55098468	1.052997582	NA	NA
ASV_16s_15213	0.262905911	0.734197195	3.55098468	1.052997582	NA	NA
ASV_16s_15214	0.262905911	0.734197195	3.55098468	1.052997582	NA	NA
ASV_16s_15215	0.262905911	0.734197195	3.55098468	1.052997582	NA	NA
ASV_16s_15216	0.130231932	0.729218277	3.55138729	0.717926556	NA	NA
ASV_16s_15217	0.24441732	0.693141847	3.550855594	0.975805573	NA	NA

ASV_16s_15218	0.24441732	0.693141847	3.550855594	0.975805573	NA	NA
ASV_16s_15219	0.247884923	0.693156266	3.550851371	0.982847014	0.995100512	NA
ASV_16s_15220	0.248875666	0.693160313	3.550850186	0.984843164	0.995069345	NA
ASV_16s_15221	0.23381434	0.693095111	3.550869286	0.953688059	0.99554142	NA
ASV_16s_15222	0.198737969	0.692905539	3.550924895	0.873643539	NA	NA
ASV_16s_15223	0.198737969	0.692905539	3.550924895	0.873643539	NA	NA
ASV_16s_15224	0.254225237	0.693181566	3.550843964	0.995419668	0.994902086	NA
ASV_16s_15225	0.167264485	0.291459705	3.551715849	0.610573012	NA	NA
ASV_16s_15226	0.167264485	0.291459705	3.551715849	0.610573012	NA	NA
ASV_16s_15227	0.167264485	0.291459705	3.551715849	0.610573012	NA	NA
ASV_16s_15228	0.170371511	-0.049170307	3.551083371	0.968032652	0.995327862	NA
ASV_16s_15229	0.170371511	-0.049170307	3.551083371	0.968032652	0.995327862	NA
ASV_16s_15230	0.170630429	-0.06920916	3.551052616	0.9852389	0.995063152	NA
ASV_16s_15231	0.170889348	-0.088744576	3.551024881	1.000757662	0.994816305	NA
ASV_16s_15232	0.169876489	-0.085201379	3.551037657	0.99618582	0.99488983	NA
ASV_16s_15233	0.167350046	-0.094878596	3.551046405	0.998114298	0.994858898	NA
ASV_16s_15234	0.171407186	-0.126311622	3.550977003	1.026473021	0.994390111	NA
ASV_16s_15235	0.171407186	-0.126311622	3.550977003	1.026473021	0.994390111	NA
ASV_16s_15236	0.171666105	-0.144511331	3.550956461	1.036918806	0.994210798	NA
ASV_16s_15237	0.169133957	-0.136775301	3.550984474	1.026848953	0.99438372	NA
ASV_16s_15238	0.171666105	-0.144511331	3.550956461	1.036918806	0.994210798	NA
ASV_16s_15239	0.167367158	-0.148935987	3.550987081	1.028821453	0.994350111	NA
ASV_16s_15240	0.178383186	-0.179154158	3.550867185	1.070897595	0.993602373	NA
ASV_16s_15241	0.181270783	-0.186272005	3.550837684	1.082206537	0.993391238	NA
ASV_16s_15242	0.167367158	-0.148935987	3.550987081	1.028821453	0.994350111	NA
ASV_16s_15243	0.167372862	-0.166411981	3.550971264	1.036465431	0.994218655	NA
ASV_16s_15244	0.174539551	-0.18588005	3.550895047	1.062264213	0.993760637	NA
ASV_16s_15245	0.172442861	-0.19725535	3.550905371	1.058808474	0.993823281	NA
ASV_16s_15246	0.180710494	-0.248530619	3.550806602	1.082522896	0.993385269	NA
ASV_16s_15247	0.167395678	-0.23498722	3.550925344	1.032153013	0.994293054	NA
ASV_16s_15248	0.171959248	-0.26263744	3.55087685	1.041183493	0.994136552	NA
ASV_16s_15249	0.173737456	-0.28335942	3.5508543	1.037191112	0.994206075	NA
ASV_16s_15250	0.173737456	-0.28335942	3.5508543	1.037191112	0.994206075	NA
ASV_16s_15251	0.167407086	-0.269154215	3.550911242	1.014061053	0.994598512	NA
ASV_16s_15252	0.167407086	-0.269154215	3.550911242	1.014061053	0.994598512	NA
ASV_16s_15253	0.181310711	-0.298146679	3.550785741	1.056423886	0.993866274	NA
ASV_16s_15254	0.18440611	-0.320184398	3.550756876	1.054017617	0.993909464	NA
ASV_16s_15255	0.173996374	-0.300892506	3.550847666	1.026008974	0.994397993	NA
ASV_16s_15256	0.198321143	-0.373931928	3.550646868	1.066503957	0.99368323	NA
ASV_16s_15257	0.212230472	-0.405408556	3.550543227	1.086448328	NA	NA
ASV_16s_15258	0.169455621	-0.343736774	3.550878442	0.96632174	0.995353673	NA
ASV_16s_15259	0.190306126	-0.396493552	3.55071627	1.009111395	0.994680215	NA
ASV_16s_15260	0.167435606	-0.357650999	3.550894936	0.942358544	NA	NA
ASV_16s_15261	0.172246687	-0.366882729	3.550855752	0.957210672	0.995489573	NA
ASV_16s_15262	0.17503205	-0.372068858	3.550834014	0.965611944	NA	NA
ASV_16s_15263	0.167441311	-0.3758041	3.550895625	0.923193202	NA	NA
ASV_16s_15264	0.252517398	-0.672499256	3.550477349	0.768529117	0.997752017	NA
ASV_16s_15265	0.228201482	-0.657654845	3.550607248	0.734673555	NA	NA
ASV_16s_15266	0.217073988	-0.648628344	3.55066782	0.71793443	0.99819411	NA
ASV_16s_15267	0.209192013	-0.641519832	3.550713218	0.705513433	NA	NA
ASV_16s_15268	0.209192013	-0.641519832	3.550713218	0.705513433	NA	NA
ASV_16s_15269	0.177880157	-0.600443844	3.550890472	0.650174465	NA	NA
ASV_16s_15270	0.175935612	-0.59727339	3.550901891	0.646350413	0.998714858	NA
ASV_16s_15271	0.167498351	-0.582321133	3.550949139	0.629567223	NA	NA
ASV_16s_15272	0.167498351	-0.582321133	3.550949139	0.629567223	NA	NA
ASV_16s_15273	0.167498351	-0.582321133	3.550949139	0.629567223	NA	NA
ASV_16s_15274	0.167498351	-0.582321133	3.550949139	0.629567223	NA	NA
ASV_16s_15275	0.167498351	-0.582321133	3.550949139	0.629567223	NA	NA
ASV_16s_15276	0.053366046	-0.168364656	3.55100246	0.257737251	NA	NA
ASV_16s_15277	0.053230162	0.056594582	3.551002766	0.386556972	0.999765053	NA
ASV_16s_15278	0.108420207	0.220134684	3.550843507	0.4923362	NA	NA
ASV_16s_15279	0.195905617	0.096243203	3.550825132	1.020167563	0.994496612	NA
ASV_16s_15280	0.182149383	-0.240646601	3.550630373	0.709492217	0.998261735	NA
ASV_16s_15281	0.213900726	-0.670731923	3.5505724	0.822331289	0.99720932	NA
ASV_16s_15282	0.215866369	-0.667312968	3.550519374	0.849892437	0.996900821	NA
ASV_16s_15283	0.1724987	-0.20359809	3.550700032	0.71417518	NA	NA
ASV_16s_15284	0.219027114	-0.292394049	3.550339782	0.859494418	NA	NA

ASV_16s_15285	0.226152787	-0.680137825	3.550431416	0.890133119	0.996411667	NA
ASV_16s_15286	0.225349177	-0.67139561	3.550414392	0.910512454	0.99614589	NA
ASV_16s_15287	0.181617743	0.957601468	3.551453499	0.599341961	NA	NA
ASV_16s_15288	0.220840189	-0.650400614	3.550420609	0.923959734	0.995963715	NA
ASV_16s_15289	0.225094636	-0.321678653	3.550691697	1.003694868	0.994768713	NA
ASV_16s_15290	0.217123677	-0.633447845	3.550432761	0.93114462	0.995864136	NA
ASV_16s_15291	0.201648209	-0.603959044	3.55054905	0.889379967	0.996421254	NA
ASV_16s_15292	0.218962529	-0.624311357	3.55040641	0.952773782	0.995554814	NA
ASV_16s_15293	0.19906152	-0.544031594	3.551163667	0.739882015	NA	NA
ASV_16s_15294	0.197743882	0.682293068	3.55121987	0.895845891	NA	NA
ASV_16s_15295	0.154547645	-0.272985547	3.550194481	0.784396868	0.997599947	NA
ASV_16s_15296	0.224200291	-0.616217643	3.550364041	0.986212094	0.995047898	NA
ASV_16s_15297	0.219232119	-0.599303273	3.550400187	0.977617549	0.995181561	NA
ASV_16s_15298	0.22987887	-0.076189786	3.550533075	1.073426261	NA	NA
ASV_16s_15299	0.225859911	-0.599085449	3.550359074	1.007390352	NA	NA
ASV_16s_15300	0.219691718	-0.586541493	3.550403128	0.989651607	0.994993747	NA
ASV_16s_15301	0.193059206	-0.458425362	3.551390294	0.739810411	0.998010804	NA
ASV_16s_15302	0.234892974	-0.511117571	3.550456725	1.099998182	NA	NA
ASV_16s_15303	0.207408506	-0.159859264	3.550698645	0.929834292	0.995882414	NA
ASV_16s_15304	0.175251383	0.31762204	3.552012862	0.396385861	0.999744442	NA
ASV_16s_15305	0.221875179	0.183208776	3.549742342	1.02377455	NA	NA
ASV_16s_15306	0.2307653	-0.3874651	3.550323932	0.866179045	0.996708466	NA
ASV_16s_15307	0.227702307	-0.483910661	3.550734675	0.789403483	0.997550596	NA
ASV_16s_15308	0.210725982	0.468712963	3.550767234	0.590552047	0.999042762	NA
ASV_16s_15309	0.215802443	-0.203324736	3.551365242	0.508497621	0.999414768	NA
ASV_16s_15310	0.217715192	-0.150599676	3.551125841	0.783234315	0.997611311	NA
ASV_16s_15311	0.214903168	-0.160874872	3.55082945	1.000109159	0.994826775	NA
ASV_16s_15312	0.228572062	0.210377885	3.550778543	0.972018025	0.995267383	NA
ASV_16s_15313	0.214795058	-0.110768708	3.550816152	0.968280099	0.995324122	NA
ASV_16s_15314	0.214097729	-0.060872194	3.550996831	1.023609378	NA	NA
ASV_16s_15315	0.186361065	-0.006747523	3.550650193	0.882236686	0.996511352	NA
ASV_16s_15316	0.195243917	0.049792029	3.550884294	0.892320234	0.996383733	NA
ASV_16s_15317	0.276810625	-0.042997661	3.550752702	1.009707614	0.994670415	NA
ASV_16s_15318	0.198565023	0.51085015	3.550864183	1.257615718	0.989539249	NA
ASV_16s_15319	0.219730307	1.155429135	3.550909281	0.665278725	NA	NA
ASV_16s_15320	0.242130837	0.234863028	3.549962429	0.924238649	0.995959879	NA
ASV_16s_15321	0.22661426	0.790009137	3.55032164	0.805823625	0.997384066	NA
ASV_16s_15322	0.216995388	0.327114097	3.551936101	0.464823263	0.999565363	NA
ASV_16s_15323	0.18543708	1.189986313	3.551071722	0.611681727	NA	NA
ASV_16s_15324	0.205056012	0.575662648	3.550517049	1.033454852	0.99427066	NA
ASV_16s_15325	0.208658522	1.226118713	3.550968642	0.652946091	NA	NA
ASV_16s_15326	0.232046406	1.254285681	3.550864506	0.69003756	0.998411118	NA
ASV_16s_15327	0.243031141	0.385368705	3.551255583	0.736538424	NA	NA
ASV_16s_15328	0.204622694	0.348553106	3.551985055	0.397074086	0.999742954	NA
ASV_16s_15329	0.140653208	0.347127047	3.551846882	0.5537604	NA	NA
ASV_16s_15330	0.140653208	0.347127047	3.551846882	0.5537604	NA	NA
ASV_16s_15331	0.190447582	0.369330421	3.551574021	0.654448728	NA	NA
ASV_16s_15332	0.252232179	0.107438873	3.551911676	0.478100684	NA	NA
ASV_16s_15333	0.186342446	1.219212504	3.550847696	0.553651236	NA	NA
ASV_16s_15334	0.235541704	0.407530538	3.550936415	0.658913285	NA	NA
ASV_16s_15335	0.200735353	0.739337431	3.550684721	0.992342821	0.994951113	NA
ASV_16s_15336	0.160149755	0.374614826	3.551388731	0.53163379	NA	NA
ASV_16s_15337	0.16668188	0.553488484	3.551065804	0.709264244	NA	NA
ASV_16s_15338	0.222245282	-0.155606365	3.55005842	1.16441378	0.991723314	NA
ASV_16s_15339	0.173276358	0.379842834	3.551316844	0.557533259	NA	NA
ASV_16s_15340	0.060333351	0.342147566	3.551825057	0.261024903	NA	NA
ASV_16s_15341	0.205976076	-0.073487305	3.55074373	0.906174465	NA	NA
ASV_16s_15342	0.097353884	-0.403705651	3.551269543	0.383360591	NA	NA
ASV_16s_15343	0.097353884	-0.403705651	3.551269543	0.383360591	NA	NA
ASV_16s_15344	0.14835455	-0.246252097	3.550630485	0.549377086	NA	NA
ASV_16s_15345	0.118234059	-0.452882536	3.551088738	0.439060109	NA	NA
ASV_16s_15346	0.118234059	-0.452882536	3.551088738	0.439060109	NA	NA
ASV_16s_15347	0.108358971	-0.430894893	3.551178361	0.413426636	NA	NA
ASV_16s_15348	0.108358971	-0.430894893	3.551178361	0.413426636	NA	NA
ASV_16s_15349	0.108358971	-0.430894893	3.551178361	0.413426636	NA	NA
ASV_16s_15350	0.194006112	-0.078686006	3.55079627	0.921814889	NA	NA
ASV_16s_15351	0.165431219	-0.078522658	3.55084473	0.844542527	NA	NA

ASV_16s_15352	0.165431219	-0.078522658	3.55084473	0.844542527	NA	NA
ASV_16s_15353	0.165431219	-0.078522658	3.55084473	0.844542527	NA	NA
ASV_16s_15354	0.165431219	-0.078522658	3.55084473	0.844542527	NA	NA
ASV_16s_15355	0.170606637	-0.078554052	3.550834757	0.859332974	0.996790244	NA
ASV_16s_15356	0.115842774	0.220121655	3.550826463	0.520627205	0.999367393	NA
ASV_16s_15357	0.116549685	0.220120509	3.550824953	0.523260821	0.999356774	NA
ASV_16s_15358	0.134641362	0.223119114	3.550983945	0.380377021	0.999777409	NA
ASV_16s_15359	0.120929117	0.220113693	3.550815986	0.539384618	0.999289111	NA
ASV_16s_15360	0.123157823	0.220110442	3.550811666	0.547445616	NA	NA
ASV_16s_15361	0.122989865	0.220110669	3.550811986	0.546842117	0.999256248	NA
ASV_16s_15362	0.18328255	0.310944128	3.550944814	0.874555082	NA	NA
ASV_16s_15363	0.188784511	-0.068143365	3.550756407	1.034303116	0.994256037	NA
ASV_16s_15364	0.189952264	-0.068163187	3.550705251	1.100676315	0.993037008	NA
ASV_16s_15365	0.171872957	-0.067953867	3.55077123	1.03485618	NA	NA
ASV_16s_15366	0.171872957	-0.067953867	3.55077123	1.03485618	NA	NA
ASV_16s_15367	0.138559361	-0.319426521	3.551148548	0.71825861	NA	NA
ASV_16s_15368	0.176332622	-0.067840432	3.550677483	1.097608638	NA	NA
ASV_16s_15369	0.177819177	-0.067343492	3.550705161	1.083817731	NA	NA
ASV_16s_15370	0.185060169	-0.293922732	3.551090559	0.971385566	NA	NA
ASV_16s_15371	0.156491873	0.723687344	3.551619449	0.786919668	NA	NA
ASV_16s_15372	0.154571189	0.747867396	3.551417111	0.724239668	NA	NA
ASV_16s_15373	0.213811488	0.337536277	3.551426669	0.565182772	0.999171084	NA
ASV_16s_15374	0.159696201	-0.481555529	3.550746061	0.621717029	0.998867617	NA
ASV_16s_15375	0.185945682	-0.041677666	3.55089116	1.021229818	NA	NA
ASV_16s_15376	0.15773937	-0.385111623	3.550971228	0.618346139	NA	NA
ASV_16s_15377	0.157915295	0.700757848	3.551564348	0.782368253	NA	NA
ASV_16s_15378	0.225150769	1.413816521	3.551034556	0.418864804	NA	NA
ASV_16s_15379	0.212079101	1.060488286	3.551047002	0.633690118	NA	NA
ASV_16s_15380	0.223910041	0.324476202	3.551516858	0.656418878	0.998648645	NA
ASV_16s_15381	0.050040385	0.293731033	3.551906155	0.273609193	NA	NA
ASV_16s_15382	0.050040385	0.293731033	3.551906155	0.273609193	NA	NA
ASV_16s_15383	0.233599617	0.279721396	3.551473039	0.744849639	NA	NA
ASV_16s_15384	0.162718485	0.691015857	3.55134521	0.585689096	NA	NA
ASV_16s_15385	0.37786565	-0.565057757	3.54975519	0.876284497	NA	NA
ASV_16s_15386	0.300994809	-0.044381373	3.550236464	1.300978567	NA	NA
ASV_16s_15387	0.287998887	0.486793288	3.550474472	0.953564347	0.995543234	NA
ASV_16s_15388	0.32036402	-0.498190752	3.550057381	0.901340111	0.996267037	NA
ASV_16s_15389	0.307325763	0.381432974	3.550823802	1.923284414	0.963964015	NA
ASV_16s_15390	0.312882566	0.199629649	3.549801733	1.332341996	0.987554051	NA
ASV_16s_15391	0.311674428	0.337673528	3.549932007	1.551356811	0.980478239	NA
ASV_16s_15392	0.183171553	-0.028654458	3.550962205	0.952860009	NA	NA
ASV_16s_15393	0.183171553	-0.028654458	3.550962205	0.952860009	NA	NA
ASV_16s_15394	0.20929681	-0.073679997	3.550801531	0.983361816	NA	NA
ASV_16s_15395	0.233548961	0.337347025	3.550272841	1.277478299	0.98903223	NA
ASV_16s_15396	0.152861936	-0.07330645	3.55090722	0.831201363	NA	NA
ASV_16s_15397	0.152861936	-0.07330645	3.55090722	0.831201363	NA	NA
ASV_16s_15398	0.152125024	-0.073299763	3.550909116	0.828931759	0.997137362	NA
ASV_16s_15399	0.190432006	0.288637259	3.550310696	1.017498775	NA	NA
ASV_16s_15400	0.228064763	0.381301059	3.550718516	1.128659352	NA	NA
ASV_16s_15401	0.208947906	0.360326307	3.55107959	1.00084245	NA	NA
ASV_16s_15402	0.208947906	0.360326307	3.55107959	1.00084245	NA	NA
ASV_16s_15403	0.208947906	0.360326307	3.55107959	1.00084245	NA	NA
ASV_16s_15404	0.24461146	0.7558246	3.550644413	1.251301432	0.989697331	NA
ASV_16s_15405	0.18574747	0.360307898	3.55113979	0.942799657	NA	NA
ASV_16s_15406	0.18574747	0.360307898	3.55113979	0.942799657	NA	NA
ASV_16s_15407	0.18574747	0.360307898	3.55113979	0.942799657	NA	NA
ASV_16s_15408	0.18574747	0.360307898	3.55113979	0.942799657	NA	NA
ASV_16s_15409	0.18574747	0.360307898	3.55113979	0.942799657	NA	NA
ASV_16s_15410	0.18574747	0.360307898	3.55113979	0.942799657	NA	NA
ASV_16s_15411	0.18574747	0.360307898	3.55113979	0.942799657	NA	NA
ASV_16s_15412	0.18574747	0.360307898	3.55113979	0.942799657	NA	NA
ASV_16s_15413	0.24067199	0.315939654	3.550885503	1.032646613	NA	NA
ASV_16s_15414	0.214493571	0.315939603	3.550921723	0.974940679	0.995222712	NA
ASV_16s_15415	0.211197979	0.315939609	3.550926914	0.96722915	0.995339995	NA
ASV_16s_15416	0.202218807	0.315939627	3.550941908	0.945693733	0.995657655	NA
ASV_16s_15417	0.20119222	0.315939629	3.550943708	0.943174992	0.995693867	NA
ASV_16s_15418	0.209944613	0.315939612	3.55092894	0.963886752	0.995390247	NA

ASV_16s_15419	0.233056196	0.315939573	3.550895234	1.016059037	0.994565305	NA
ASV_16s_15420	0.194006112	0.315939757	3.550956836	0.925241554	NA	NA
ASV_16s_15421	0.194006112	0.315939757	3.550956836	0.925241554	NA	NA
ASV_16s_15422	0.194006112	0.315939757	3.550956836	0.925241554	NA	NA
ASV_16s_15423	0.194006112	0.315939757	3.550956836	0.925241554	NA	NA
ASV_16s_15424	0.194006112	0.371279157	3.550934497	1.164346851	NA	NA
ASV_16s_15425	0.184759205	0.687759681	3.550913503	0.813707999	NA	NA
ASV_16s_15426	0.288244449	1.094384953	3.550610975	0.832579377	0.99709708	NA
ASV_16s_15427	0.256646247	0.734163072	3.550994615	1.040842614	NA	NA
ASV_16s_15428	0.256646247	0.734163072	3.550994615	1.040842614	NA	NA
ASV_16s_15429	0.256646247	0.734163072	3.550994615	1.040842614	NA	NA
ASV_16s_15430	0.127131172	0.728021251	3.551402819	0.707323352	NA	NA
ASV_16s_15431	0.127131172	0.728021251	3.551402819	0.707323352	NA	NA
ASV_16s_15432	0.243056206	0.693136194	3.550857298	0.972960242	0.995253011	NA
ASV_16s_15433	0.245367941	0.693145958	3.550854438	0.977685545	NA	NA
ASV_16s_15434	0.194006112	0.692874913	3.550933944	0.861876716	NA	NA
ASV_16s_15435	0.224902884	0.69305255	3.550881812	0.934270315	0.995820324	NA
ASV_16s_15436	0.227109796	0.693063414	3.550878626	0.939113382	0.995751849	NA
ASV_16s_15437	0.284489515	0.693287386	3.550813058	1.051960656	NA	NA
ASV_16s_15438	0.280126051	1.391323508	3.550546996	0.598079025	NA	NA
ASV_16s_15439	0.163184863	0.291855548	3.551734177	0.602229328	NA	NA
ASV_16s_15440	0.163184863	0.291855548	3.551734177	0.602229328	NA	NA
ASV_16s_15441	0.163184863	0.291855548	3.551734177	0.602229328	NA	NA
ASV_16s_15442	0.163184863	0.291855548	3.551734177	0.602229328	NA	NA
ASV_16s_15443	0.164738376	0.088782884	3.551375299	0.814319091	NA	NA
ASV_16s_15444	0.178185057	-0.001486262	3.55109668	0.920367726	NA	NA
ASV_16s_15445	0.175589273	-0.010765734	3.551101551	0.927623197	NA	NA
ASV_16s_15446	0.184613712	-0.080575519	3.550922111	1.017851015	0.994535412	NA
ASV_16s_15447	0.167068646	-0.112204329	3.551028241	0.999472562	0.99483704	NA
ASV_16s_15448	0.169461221	-0.155127564	3.550964361	1.028198862	0.994360733	NA
ASV_16s_15449	0.167845402	-0.167727615	3.550966394	1.029297437	0.994341982	NA
ASV_16s_15450	0.167845402	-0.167727615	3.550966394	1.029297437	0.994341982	NA
ASV_16s_15451	0.166326113	-0.163293515	3.550982825	1.023558672	0.994439497	NA
ASV_16s_15452	0.181292083	-0.218024979	3.550816783	1.083607051	0.993364788	NA
ASV_16s_15453	0.168104321	-0.185662374	3.550949913	1.035153549	0.994241353	NA
ASV_16s_15454	0.166090011	-0.214538745	3.550947364	1.028203143	0.99436066	NA
ASV_16s_15455	0.168622159	-0.221069497	3.550922604	1.03630003	0.99422152	NA
ASV_16s_15456	0.163304648	-0.207157135	3.550975656	1.019414461	0.994509245	NA
ASV_16s_15457	0.169139996	-0.256071269	3.550902092	1.030359316	0.994323819	NA
ASV_16s_15458	0.172534023	-0.263863115	3.550871911	1.038289968	0.994186992	NA
ASV_16s_15459	0.163344577	-0.3311631	3.550931072	0.946197796	0.995650385	NA
ASV_16s_15460	0.166895288	-0.356559726	3.550899612	0.940571728	0.995731088	NA
ASV_16s_15461	0.163350281	-0.349399965	3.550929797	0.929278338	NA	NA
ASV_16s_15462	0.167913851	-0.376678098	3.550891923	0.924534523	0.995955807	NA
ASV_16s_15463	0.174884147	-0.407554925	3.550840389	0.922460452	0.995984297	NA
ASV_16s_15464	0.058336624	-0.233500606	3.551763599	0.319600425	NA	NA
ASV_16s_15465	0.058336624	-0.233500606	3.551763599	0.319600425	NA	NA
ASV_16s_15466	0.058336624	-0.233500606	3.551763599	0.319600425	NA	NA
ASV_16s_15467	0.058336624	-0.233500606	3.551763599	0.319600425	NA	NA
ASV_16s_15468	0.058336624	-0.233500606	3.551763599	0.319600425	NA	NA
ASV_16s_15469	0.242662107	-0.666988252	3.550528822	0.755148909	0.997875191	NA
ASV_16s_15470	0.222635592	-0.653178079	3.550637001	0.726344895	NA	NA
ASV_16s_15471	0.211743057	-0.643859216	3.550698403	0.709530416	0.998261433	NA
ASV_16s_15472	0.204089769	-0.636689308	3.550744224	0.697180057	NA	NA
ASV_16s_15473	0.202034207	-0.634700085	3.550757159	0.693750464	0.998383296	NA
ASV_16s_15474	0.170756254	-0.588208602	3.55093079	0.636109142	NA	NA
ASV_16s_15475	0.168224106	-0.583618108	3.550945078	0.630995192	0.998811598	NA
ASV_16s_15476	0.168224106	-0.583618108	3.550945078	0.630995192	0.998811598	NA
ASV_16s_15477	0.163413026	-0.574714661	3.550973181	0.621107157	NA	NA
ASV_16s_15478	0.163413026	-0.574714661	3.550973181	0.621107157	NA	NA
ASV_16s_15479	0.163413026	-0.574714661	3.550973181	0.621107157	NA	NA
ASV_16s_15480	0.163413026	-0.574714661	3.550973181	0.621107157	NA	NA
ASV_16s_15481	0.163413026	-0.574714661	3.550973181	0.621107157	NA	NA
ASV_16s_15482	0.163413026	-0.574714661	3.550973181	0.621107157	NA	NA
ASV_16s_15483	0.052064435	-0.167207886	3.551010586	0.250235393	NA	NA
ASV_16s_15484	0.046181536	-0.161588794	3.551052608	0.215615813	NA	NA
ASV_16s_15485	0.046181536	-0.161588794	3.551052608	0.215615813	NA	NA

ASV_16s_15486	0.072579621	0.220234025	3.550974538	0.33625507	NA	NA
ASV_16s_15490	0.188781923	0.110120009	3.551059678	1.048888046	0.994000887	NA
ASV_16s_15491	0.1922855	-0.459897199	3.550965844	0.906737178	0.996196058	NA
ASV_16s_15492	0.236624066	-0.678522794	3.550588577	0.778610852	0.997656158	NA
ASV_16s_15493	0.216060435	-0.64924188	3.550730104	0.722056034	0.998160469	NA
ASV_16s_15494	0.177934405	-0.789932745	3.550884466	0.458561082	0.999584513	NA
ASV_16s_15495	0.169282264	0.142277933	3.551043328	0.86448931	0.996728775	NA
ASV_16s_15496	0.220964718	-0.685312471	3.550598927	0.778682869	0.997655464	NA
ASV_16s_15497	0.16691327	-0.253597238	3.550836309	0.648659505	NA	NA
ASV_16s_15498	0.171401996	0.052561091	3.550859272	0.933610684	0.995829594	NA
ASV_16s_15499	0.221904455	-0.686739667	3.550551484	0.812600845	0.997313221	NA
ASV_16s_15500	0.20157847	-0.872108613	3.550614717	0.545947874	0.999260242	NA
ASV_16s_15501	0.178965387	0.269195858	3.550880415	0.977618625	0.995181544	NA
ASV_16s_15502	0.180030859	0.041879614	3.550635508	0.950376274	0.995589813	NA
ASV_16s_15503	0.196842155	-0.212604531	3.550446627	0.797748159	0.997466866	NA
ASV_16s_15504	0.178112391	0.308020891	3.550944886	0.721539805	0.998164705	NA
ASV_16s_15505	0.188539189	0.118974543	3.550953986	0.673415254	NA	NA
ASV_16s_15506	0.219142726	-0.595302457	3.550687134	0.97192226	0.995268842	NA
ASV_16s_15507	0.180391246	0.135153359	3.55068445	0.601338376	0.998984376	NA
ASV_16s_15508	0.18092776	0.676895101	3.551325462	0.791848639	NA	NA
ASV_16s_15509	0.198749409	0.958299975	3.551522256	0.744876732	NA	NA
ASV_16s_15510	0.147523082	0.623557012	3.551761958	0.743058295	NA	NA
ASV_16s_15511	0.210856296	-0.603327959	3.55046381	0.933489206	NA	NA
ASV_16s_15512	0.201992956	-0.583895863	3.550533752	0.910228711	0.996149675	NA
ASV_16s_15513	0.219788671	-0.614439396	3.550395791	0.963188786	NA	NA
ASV_16s_15514	0.204222457	-0.571763883	3.550514801	0.92846006	NA	NA
ASV_16s_15515	0.188066763	-0.698608434	3.55028271	0.697034628	0.998358418	NA
ASV_16s_15516	0.218899552	-0.599052966	3.550402725	0.973444755	NA	NA
ASV_16s_15517	0.250372723	-1.081142017	3.550380678	0.525571723	0.999347356	NA
ASV_16s_15518	0.226625385	-0.203742582	3.550180428	0.957436144	0.995486241	NA
ASV_16s_15519	0.202534644	-0.637639905	3.550588516	0.882637755	0.996506333	NA
ASV_16s_15520	0.227829387	0.372468276	3.550286901	1.197705862	0.990979624	NA
ASV_16s_15521	0.196361578	-0.04903872	3.550612459	0.931864811	0.995854067	NA
ASV_16s_15522	0.205175883	-0.103335021	3.550887359	1.026560233	0.994388629	NA
ASV_16s_15523	0.237833599	1.41677916	3.550195762	0.398180904	0.999740549	NA
ASV_16s_15524	0.218094632	-0.300386925	3.551382511	0.703889251	0.998305675	NA
ASV_16s_15525	0.198358968	1.081921923	3.551348975	0.705735808	NA	NA
ASV_16s_15526	0.215452756	-0.502880376	3.550508488	1.005917262	0.994732517	NA
ASV_16s_15527	0.19018713	-0.461624097	3.550656684	0.924254951	NA	NA
ASV_16s_15528	0.204653738	-0.439960992	3.550605863	0.976136976	NA	NA
ASV_16s_15529	0.216062519	1.098340797	3.551133116	0.731523266	0.998081629	NA
ASV_16s_15530	0.231980805	-0.508779227	3.550444238	1.07145398	NA	NA
ASV_16s_15531	0.195245999	-0.425175339	3.550656842	0.94393701	NA	NA
ASV_16s_15532	0.205262166	-0.479683611	3.551071207	0.448884694	0.999612958	NA
ASV_16s_15533	0.220575794	-0.344170057	3.550695083	1.022046661	0.99446501	NA
ASV_16s_15534	0.218398072	-0.344305372	3.55076085	0.827997415	0.997147621	NA
ASV_16s_15535	0.189716718	-0.285324982	3.550778462	0.909524632	0.996159058	NA
ASV_16s_15536	0.207307181	0.183217919	3.549795103	0.92459973	NA	NA
ASV_16s_15537	0.179002666	-0.238517643	3.550959122	0.806519481	0.997376849	NA
ASV_16s_15538	0.238443808	1.297643626	3.550853843	0.598378824	0.999000628	NA
ASV_16s_15539	0.210038031	0.681022764	3.550984523	0.909372138	0.996161088	NA
ASV_16s_15540	0.120616804	1.247807604	3.551461762	0.250133549	NA	NA
ASV_16s_15541	0.259274705	0.567995922	3.550516432	1.203789828	NA	NA
ASV_16s_15542	0.225438333	0.054786365	3.55085729	0.994872308	0.99491083	NA
ASV_16s_15543	0.175395397	-0.069619868	3.550887473	0.911423752	NA	NA
ASV_16s_15544	0.239538951	0.846565578	3.550638194	1.220594212	0.990444964	NA
ASV_16s_15545	0.186774564	1.192218616	3.551065526	0.614131759	NA	NA
ASV_16s_15546	0.177075121	0.362607044	3.551655279	0.630007075	NA	NA
ASV_16s_15547	0.177075121	0.362607044	3.551655279	0.630007075	NA	NA
ASV_16s_15548	0.189629566	0.350791725	3.551966774	0.367991789	0.99980081	NA
ASV_16s_15549	0.239925304	0.354745003	3.55175468	0.418586703	0.999693376	NA
ASV_16s_15550	0.214122574	0.352190126	3.551933425	0.389270278	0.999759482	NA
ASV_16s_15551	0.183203838	0.363582118	3.551585174	0.427953116	NA	NA
ASV_16s_15552	0.137222642	0.345916466	3.551860064	0.545642508	NA	NA
ASV_16s_15553	0.145030384	0.337058405	3.552005569	0.38832143	NA	NA
ASV_16s_15554	0.173136355	-0.421318252	3.551318464	0.602544607	NA	NA
ASV_16s_15555	0.172046219	0.484135603	3.550860208	0.901659614	0.99626286	NA

ASV_16s_15556	0.198131826	-0.543978389	3.550661094	0.668708264	0.998564785	NA
ASV_16s_15557	0.137045792	-0.478059761	3.551232147	0.550799029	NA	NA
ASV_16s_15558	0.192105368	-18.5717247	3.551624149	-0.625298598	1	NA
ASV_16s_15559	0.201607008	-0.546174387	3.550633253	0.674486442	NA	NA
ASV_16s_15560	0.195440632	-1.01750483	3.550646939	0.355695497	0.999822308	NA
ASV_16s_15561	0.179952161	-0.881558778	3.55087928	0.416624935	NA	NA
ASV_16s_15562	0.134560484	-0.777134006	3.551130743	0.288425653	NA	NA
ASV_16s_15563	0.106044429	1.042311587	3.551280574	0.373142545	NA	NA
ASV_16s_15564	0.106044429	1.042311587	3.551280574	0.373142545	NA	NA
ASV_16s_15565	0.106044429	1.042311587	3.551280574	0.373142545	NA	NA
ASV_16s_15566	0.106044429	1.042311587	3.551280574	0.373142545	NA	NA
ASV_16s_15567	0.192271302	-0.072274723	3.550832195	0.856627122	NA	NA
ASV_16s_15568	0.282653371	0.418528572	3.550695915	0.719382535	NA	NA
ASV_16s_15569	0.219623566	1.067028794	3.550563206	0.889850238	NA	NA
ASV_16s_15570	0.235338827	0.02016379	3.550114201	1.165804747	0.991693041	NA
ASV_16s_15571	0.156243664	0.372822315	3.55141048	0.523556896	NA	NA
ASV_16s_15572	0.156243664	0.372822315	3.55141048	0.523556896	NA	NA
ASV_16s_15573	0.177713549	-0.072314257	3.550747497	0.848388308	NA	NA
ASV_16s_15574	0.232161412	0.868729724	3.550430976	1.137137614	0.992303014	NA
ASV_16s_15575	0.094979399	-0.396699874	3.551286879	0.376534028	NA	NA
ASV_16s_15576	0.112007588	-0.002621639	3.551025685	0.565644387	NA	NA
ASV_16s_15577	0.115350302	-0.446631554	3.551115925	0.431681261	NA	NA
ASV_16s_15578	0.115350302	-0.446631554	3.551115925	0.431681261	NA	NA
ASV_16s_15579	0.105716069	-0.424724221	3.551202502	0.406320079	NA	NA
ASV_16s_15580	0.189274256	-0.078662457	3.550803303	0.909674489	NA	NA
ASV_16s_15581	0.189274256	-0.078662457	3.550803303	0.909674489	NA	NA
ASV_16s_15582	0.173244437	-0.078570919	3.550829919	0.866659567	0.996702675	NA
ASV_16s_15583	0.112605438	0.220127116	3.550833635	0.508381547	0.999415209	NA
ASV_16s_15584	0.114708176	0.220123551	3.550828939	0.516331942	0.999384456	NA
ASV_16s_15585	0.120153973	0.220114914	3.550817536	0.53651616	NA	NA
ASV_16s_15586	0.123409849	0.407308205	3.550777961	0.857292738	0.996814356	NA
ASV_16s_15587	0.126572729	0.595903552	3.550766942	0.497635388	NA	NA
ASV_16s_15588	0.12200183	0.215309669	3.550907493	0.339430865	0.999848207	NA
ASV_16s_15589	0.168829086	-0.067463748	3.550772816	1.043062524	NA	NA
ASV_16s_15590	0.169324604	-0.067586696	3.550769386	1.053251434	NA	NA
ASV_16s_15591	0.177605274	-0.06773517	3.550725529	1.085747229	0.993324238	NA
ASV_16s_15592	0.113243382	0.308907078	3.551987176	0.329787142	0.999862261	NA
ASV_16s_15593	0.197860614	-0.350387202	3.550742337	0.86001881	0.996782112	NA
ASV_16s_15594	0.174704163	0.566812158	3.550662196	0.63203703	0.998805194	NA
ASV_16s_15595	0.171306677	-0.06791511	3.550723316	1.07578643	0.993511634	NA
ASV_16s_15596	0.181657515	-0.068122505	3.550647567	1.112592642	0.992802223	NA
ASV_16s_15597	0.138526375	0.716666959	3.551530878	0.687944811	0.998426658	NA
ASV_16s_15598	0.225941652	0.334858348	3.550302552	1.189012192	NA	NA
ASV_16s_15599	0.184238498	-0.067596547	3.550630355	1.101905385	NA	NA
ASV_16s_15600	0.170294812	0.909268099	3.551380197	0.503768623	NA	NA
ASV_16s_15601	0.259801889	1.128024377	3.551010496	0.806762328	NA	NA
ASV_16s_15602	0.182627056	-0.012606693	3.551031869	0.976037245	NA	NA
ASV_16s_15603	0.231406566	0.193452865	3.550469671	1.096903295	NA	NA
ASV_16s_15604	0.20206176	1.04793463	3.551097483	0.616998243	NA	NA
ASV_16s_15605	0.27760337	0.27957758	3.551382976	0.807701947	NA	NA
ASV_16s_15606	0.080800854	0.286515285	3.551807353	0.391570629	NA	NA
ASV_16s_15607	0.080800854	0.286515285	3.551807353	0.391570629	NA	NA
ASV_16s_15608	0.159138992	-0.474091846	3.551303476	0.599777191	NA	NA
ASV_16s_15609	0.135447472	-0.078278291	3.550917439	0.750883334	NA	NA
ASV_16s_15610	0.135447472	-0.078278291	3.550917439	0.750883334	NA	NA
ASV_16s_15611	0.135447472	-0.078278291	3.550917439	0.750883334	NA	NA
ASV_16s_15612	0.135447472	-0.078278291	3.550917439	0.750883334	NA	NA
ASV_16s_15613	0.147558498	-0.103441724	3.550865992	0.856943617	NA	NA
ASV_16s_15614	0.374820129	-0.02933595	3.550736401	1.311508262	NA	NA
ASV_16s_15615	0.269571894	0.836357751	3.550458793	1.349094348	0.987079692	NA
ASV_16s_15616	0.205730156	0.408482364	3.55074689	1.019799922	NA	NA
ASV_16s_15617	0.236528211	0.356077315	3.550419775	1.199933592	NA	NA
ASV_16s_15618	0.254850874	0.48107268	3.550953524	1.085560609	0.99332778	NA
ASV_16s_15619	0.149133596	-0.073272096	3.55091702	0.819574794	NA	NA
ASV_16s_15620	0.199760089	0.339873196	3.55048486	1.150955498	0.992012648	NA
ASV_16s_15621	0.146453915	-0.073246227	3.550924355	0.811119661	NA	NA
ASV_16s_15622	0.146453915	-0.073246227	3.550924355	0.811119661	NA	NA

ASV_16s_15623	0.146453915	-0.073246227	3.550924355	0.811119661	NA	NA
ASV_16s_15624	0.198420248	0.340469342	3.550496499	1.144303928	0.992153267	NA
ASV_16s_15625	0.202892454	0.312124545	3.550529939	1.142209983	0.99219721	NA
ASV_16s_15626	0.220436735	0.301488555	3.550504954	1.191829063	0.991113816	NA
ASV_16s_15627	0.181714272	-0.073526226	3.550845045	0.913985298	NA	NA
ASV_16s_15628	0.219484043	0.339860443	3.550367212	1.209024083	NA	NA
ASV_16s_15629	0.211197607	0.345368874	3.551156876	0.735743008	0.998045778	NA
ASV_16s_15630	0.197962577	0.332220121	3.551057399	0.774866969	0.997692064	NA
ASV_16s_15631	0.230159393	0.332218256	3.55097594	0.846004508	NA	NA
ASV_16s_15632	0.215034235	0.320427747	3.550987261	0.827637914	0.997151562	NA
ASV_16s_15633	0.181217044	0.360303765	3.551153355	0.930669498	NA	NA
ASV_16s_15634	0.181217044	0.360303765	3.551153355	0.930669498	NA	NA
ASV_16s_15635	0.181217044	0.360303765	3.551153355	0.930669498	NA	NA
ASV_16s_15636	0.181217044	0.360303765	3.551153355	0.930669498	NA	NA
ASV_16s_15637	0.181217044	0.360303765	3.551153355	0.930669498	NA	NA
ASV_16s_15638	0.181217044	0.360303765	3.551153355	0.930669498	NA	NA
ASV_16s_15639	0.234801941	0.315939672	3.550892939	1.020183423	NA	NA
ASV_16s_15640	0.202939941	0.315939647	3.550940676	0.947377958	0.995633332	NA
ASV_16s_15641	0.202962081	0.315939742	3.550940634	0.94745178	NA	NA
ASV_16s_15642	0.202962081	0.315939742	3.550940634	0.94745178	NA	NA
ASV_16s_15643	0.202962081	0.315939742	3.550940634	0.94745178	NA	NA
ASV_16s_15644	0.196460364	0.315939661	3.550952262	0.931367755	0.995861018	NA
ASV_16s_15645	0.195433777	0.315939664	3.550954169	0.928786621	0.99589699	NA
ASV_16s_15646	0.194064995	0.315939667	3.550956742	0.925327591	0.995944878	NA
ASV_16s_15647	0.193380603	0.315939669	3.550958042	0.923589919	0.995968798	NA
ASV_16s_15648	0.201856634	0.485538885	3.550904473	1.423929438	0.984827029	NA
ASV_16s_15649	0.189274256	0.315939781	3.55096604	0.913055141	NA	NA
ASV_16s_15650	0.189274256	0.315939781	3.55096604	0.913055141	NA	NA
ASV_16s_15651	0.189274256	0.315939781	3.55096604	0.913055141	NA	NA
ASV_16s_15652	0.25484853	0.596184504	3.550657872	0.828541515	NA	NA
ASV_16s_15653	0.180252883	0.687731851	3.550922447	0.801839597	NA	NA
ASV_16s_15654	0.295538297	1.111206392	3.550526197	0.810695065	NA	NA
ASV_16s_15655	0.260625195	0.734185045	3.550988281	1.048487215	0.994007993	NA
ASV_16s_15656	0.250386582	0.734127244	3.551005043	1.028399145	NA	NA
ASV_16s_15657	0.250386582	0.734127244	3.551005043	1.028399145	NA	NA
ASV_16s_15658	0.250386582	0.734127244	3.551005043	1.028399145	NA	NA
ASV_16s_15659	0.124030412	0.726412987	3.551417345	0.696567378	NA	NA
ASV_16s_15660	0.236741374	0.693106973	3.550865408	0.959783683	0.995451457	NA
ASV_16s_15661	0.20558831	0.6929478	3.550912591	0.890044707	0.996412793	NA
ASV_16s_15662	0.214852774	0.692998512	3.550897141	0.911682824	0.996130251	NA
ASV_16s_15663	0.250093362	0.69316386	3.550848778	0.987129123	0.995033498	NA
ASV_16s_15664	0.239383358	0.693120406	3.55086197	0.965310213	NA	NA
ASV_16s_15665	0.239383358	0.693120406	3.55086197	0.965310213	NA	NA
ASV_16s_15666	0.250984516	0.693167464	3.550847725	0.988934954	0.995005061	NA
ASV_16s_15667	0.189274256	0.692842747	3.550943444	0.849888522	NA	NA
ASV_16s_15668	0.189274256	0.692842747	3.550943444	0.849888522	NA	NA
ASV_16s_15669	0.189274256	0.692842747	3.550943444	0.849888522	NA	NA
ASV_16s_15670	0.159105241	0.291796768	3.551749774	0.593650335	NA	NA
ASV_16s_15671	0.159105241	0.291796768	3.551749774	0.593650335	NA	NA
ASV_16s_15672	0.159105241	0.291796768	3.551749774	0.593650335	NA	NA
ASV_16s_15673	0.159105241	0.291796768	3.551749774	0.593650335	NA	NA
ASV_16s_15674	0.159105241	0.291796768	3.551749774	0.593650335	NA	NA
ASV_16s_15675	0.161953349	-0.035291097	3.551178274	0.922570644	0.995982787	NA
ASV_16s_15676	0.161953349	-0.035291097	3.551178274	0.922570644	0.995982787	NA
ASV_16s_15677	0.160692979	-0.050558328	3.551166078	0.93338113	0.995832817	NA
ASV_16s_15678	0.169962062	-0.085289647	3.551037215	0.978652258	0.995165593	NA
ASV_16s_15679	0.162989024	-0.116875573	3.551058071	0.984061646	0.995081563	NA
ASV_16s_15680	0.163247943	-0.136024369	3.551035164	0.994920288	0.994910064	NA
ASV_16s_15681	0.163247943	-0.136024369	3.551035164	0.994920288	0.994910064	NA
ASV_16s_15682	0.163247943	-0.136024369	3.551035164	0.994920288	0.994910064	NA
ASV_16s_15683	0.163247943	-0.136024369	3.551035164	0.994920288	0.994910064	NA
ASV_16s_15684	0.163247943	-0.136024369	3.551035164	0.994920288	0.994910064	NA
ASV_16s_15685	0.163506862	-0.154818851	3.551014622	1.004163057	0.994761101	NA
ASV_16s_15686	0.163506862	-0.154818851	3.551014622	1.004163057	0.994761101	NA
ASV_16s_15687	0.163506862	-0.154818851	3.551014622	1.004163057	0.994761101	NA
ASV_16s_15688	0.163765781	-0.173319562	3.550996274	1.010830421	0.99465193	NA
ASV_16s_15689	0.163765781	-0.173319562	3.550996274	1.010830421	0.99465193	NA

ASV_16s_15690	0.159207915	-0.159749666	3.551048276	0.993874817	0.99492674	NA
ASV_16s_15691	0.160727203	-0.16434724	3.551030512	0.999482115	0.994836886	NA
ASV_16s_15692	0.163765781	-0.173319562	3.550996274	1.010830421	0.99465193	NA
ASV_16s_15693	0.159213619	-0.177874988	3.551032874	0.996055895	0.99489191	NA
ASV_16s_15694	0.164283618	-0.20972724	3.55096577	1.013641917	0.994605461	NA
ASV_16s_15695	0.164542537	-0.227723392	3.55095337	1.012480937	0.994624681	NA
ASV_16s_15696	0.162263604	-0.221841816	3.550976296	1.005253757	0.99474334	NA
ASV_16s_15697	0.164542537	-0.227723392	3.55095337	1.012480937	0.994624681	NA
ASV_16s_15698	0.177458862	-0.257678819	3.550830875	1.05470008	0.993897234	NA
ASV_16s_15699	0.174101802	-0.284003153	3.550851289	1.031197443	0.994309457	NA
ASV_16s_15700	0.168686555	-0.272008636	3.550899907	1.013145296	0.994613688	NA
ASV_16s_15701	0.162528227	-0.257499504	3.550957961	0.994715178	0.994913338	NA
ASV_16s_15702	0.17104337	-0.294475709	3.550873205	1.0096881	0.994670736	NA
ASV_16s_15703	0.160761428	-0.271121508	3.550968356	0.977973708	0.995176068	NA
ASV_16s_15704	0.165837131	-0.318407028	3.550911604	0.969990581	0.995298213	NA
ASV_16s_15705	0.159276363	-0.378586919	3.550967158	0.877777205	NA	NA
ASV_16s_15706	0.162073134	-0.422755521	3.55095073	0.840535718	0.99700793	NA
ASV_16s_15707	0.056878208	-0.225955712	3.551771205	0.313746347	NA	NA
ASV_16s_15708	0.056878208	-0.225955712	3.551771205	0.313746347	NA	NA
ASV_16s_15709	0.056878208	-0.225955712	3.551771205	0.313746347	NA	NA
ASV_16s_15710	0.056878208	-0.225955712	3.551771205	0.313746347	NA	NA
ASV_16s_15711	0.056878208	-0.225955712	3.551771205	0.313746347	NA	NA
ASV_16s_15712	0.229029864	-0.658264738	3.55060323	0.735777009	0.998045487	NA
ASV_16s_15713	0.189453414	-0.618387913	3.550826887	0.671760406	0.998543432	NA
ASV_16s_15714	0.219576742	-0.650661331	3.550653977	0.721666178	NA	NA
ASV_16s_15715	0.169203076	-0.585368873	3.550939611	0.632930814	NA	NA
ASV_16s_15716	0.169203076	-0.585368873	3.550939611	0.632930814	NA	NA
ASV_16s_15717	0.169203076	-0.585368873	3.550939611	0.632930814	NA	NA
ASV_16s_15718	0.167430573	-0.582135363	3.550949723	0.629328706	NA	NA
ASV_16s_15719	0.16515164	-0.5779287	3.550962977	0.624655707	0.99885007	NA
ASV_16s_15720	0.1593277	-0.566798929	3.550997951	0.612445696	NA	NA
ASV_16s_15721	0.1593277	-0.566798929	3.550997951	0.612445696	NA	NA
ASV_16s_15722	0.1593277	-0.566798929	3.550997951	0.612445696	NA	NA
ASV_16s_15723	0.1593277	-0.566798929	3.550997951	0.612445696	NA	NA
ASV_16s_15724	0.296023686	1.553128993	3.550497118	0.591474313	NA	NA
ASV_16s_15725	0.049602854	0.052504152	3.551034386	0.35384616	0.999825396	NA
ASV_16s_15726	0.030585364	-0.142163541	3.551236554	0.11638502	NA	NA
ASV_16s_15727	0.061481993	0.220287905	3.551045861	0.279595502	0.999921201	NA
ASV_16s_15730	0.167343136	-0.131579976	3.55097885	0.917635166	0.996050075	NA
ASV_16s_15731	0.180161729	-0.067897742	3.551388358	0.799045243	0.997453685	NA
ASV_16s_15732	0.166424524	0.036705322	3.550981281	0.936411668	0.995790136	NA
ASV_16s_15733	0.168590343	0.063026219	3.550966288	0.947980134	0.995624614	NA
ASV_16s_15734	0.169501497	-0.308524003	3.551195939	0.84428775	0.996965275	NA
ASV_16s_15735	0.192129634	0.290967501	3.550778572	0.743008226	NA	NA
ASV_16s_15736	0.173494648	0.734257449	3.551794281	0.60778241	NA	NA
ASV_16s_15737	0.163731045	0.256313141	3.551134949	0.915101155	0.996084338	NA
ASV_16s_15738	0.225436443	-0.692424477	3.550538906	0.823964142	0.99719163	NA
ASV_16s_15739	0.163828014	-0.201847771	3.550808126	0.675516358	NA	NA
ASV_16s_15740	0.171954069	0.455962186	3.551071337	0.795017299	0.997494471	NA
ASV_16s_15741	0.22634437	-0.680337278	3.550430671	0.887422617	0.996446091	NA
ASV_16s_15742	0.207602869	-0.64210346	3.550540395	0.852561337	0.996869817	NA
ASV_16s_15743	0.203238744	-0.067878986	3.551205366	1.017521545	NA	NA
ASV_16s_15744	0.191984992	-0.035764703	3.551227233	0.814457998	0.99729359	NA
ASV_16s_15745	0.207386	-0.618813469	3.550508389	0.892394581	0.996382781	NA
ASV_16s_15746	0.198204421	-0.595881423	3.550575782	0.877750571	0.996567173	NA
ASV_16s_15747	0.214048653	-0.599029965	3.550437543	0.94728303	NA	NA
ASV_16s_15748	0.300505646	0.763155277	3.550183097	1.428512281	0.984681911	NA
ASV_16s_15749	0.227282609	-0.583065793	3.550808653	0.675683697	0.998515675	NA
ASV_16s_15750	0.209487842	-0.695896408	3.550441377	0.840238739	0.99701129	NA
ASV_16s_15751	0.206708433	-0.93928117	3.550827389	0.445114622	0.999623671	NA
ASV_16s_15752	0.204531084	-0.539621832	3.550529665	0.945525425	0.995660081	NA
ASV_16s_15753	0.173038278	1.190027349	3.551375376	0.483084362	0.999506093	NA
ASV_16s_15754	0.214209691	-0.477859708	3.550535941	1.002444903	0.994789	NA
ASV_16s_15755	0.220380436	-0.541438897	3.550446407	1.034395194	NA	NA
ASV_16s_15756	0.16161226	-0.649507945	3.550822595	0.507442186	NA	NA
ASV_16s_15757	0.142303951	-0.2471499	3.551742111	0.57435507	NA	NA
ASV_16s_15758	0.193991272	-0.406918738	3.550707954	1.023779049	0.994435772	NA

ASV_16s_15759	0.200935968	0.182630395	3.549831208	0.889890442	NA	NA
ASV_16s_15760	0.197657254	0.18214643	3.549907328	0.8494584	NA	NA
ASV_16s_15761	0.179774885	-0.461806985	3.550796967	0.77281836	NA	NA
ASV_16s_15762	0.192757337	-0.002447721	3.55091605	0.882509928	NA	NA
ASV_16s_15763	0.174333599	-0.069118032	3.550934326	0.915431871	0.996079877	NA
ASV_16s_15764	0.209846457	0.334727123	3.550868946	1.370606735	0.986454589	NA
ASV_16s_15765	0.221459337	1.242529811	3.550912175	0.673692185	NA	NA
ASV_16s_15766	0.176391369	1.174101435	3.551116173	0.594268742	NA	NA
ASV_16s_15767	0.185321602	1.391773152	3.550904905	0.42991413	0.999664806	NA
ASV_16s_15768	0.1821052	1.184182889	3.551087787	0.605281955	NA	NA
ASV_16s_15769	0.20628116	0.982918642	3.551030394	0.859261285	0.996791093	NA
ASV_16s_15770	0.227375369	-0.311999259	3.550288125	1.07326768	NA	NA
ASV_16s_15771	0.225330549	0.368650287	3.551570843	0.458925815	NA	NA
ASV_16s_15772	0.241020323	0.384985429	3.551267448	0.733636625	NA	NA
ASV_16s_15773	0.206357888	0.352331746	3.551927254	0.379822923	0.999778495	NA
ASV_16s_15774	0.181157456	0.364639497	3.551632194	0.637603717	NA	NA
ASV_16s_15775	0.181157456	0.364639497	3.551632194	0.637603717	NA	NA
ASV_16s_15776	0.265935569	0.098472694	3.550316715	1.089722625	0.993248501	NA
ASV_16s_15777	0.22386071	-0.559163558	3.550476233	0.708805657	NA	NA
ASV_16s_15778	0.214174832	-1.014530297	3.550476593	0.396213516	0.999744814	NA
ASV_16s_15779	0.133619647	-0.471148163	3.551258907	0.542737511	NA	NA
ASV_16s_15780	0.133619647	-0.471148163	3.551258907	0.542737511	NA	NA
ASV_16s_15781	0.118432804	-0.689459973	3.551239201	0.276330172	NA	NA
ASV_16s_15782	0.191633732	-1.00222855	3.550718076	0.362536275	0.999810557	NA
ASV_16s_15783	0.174899635	-0.527949114	3.550867875	0.628577522	NA	NA
ASV_16s_15784	0.174899635	-0.527949114	3.550867875	0.628577522	NA	NA
ASV_16s_15785	0.142350612	1.37491145	3.551218766	0.295749081	0.99990468	NA
ASV_16s_15786	0.103393318	1.033949951	3.551301193	0.365882022	NA	NA
ASV_16s_15787	0.103393318	1.033949951	3.551301193	0.365882022	NA	NA
ASV_16s_15788	0.177252571	1.204929732	3.550886949	0.536441142	NA	NA
ASV_16s_15789	0.224051865	0.403101595	3.55099523	0.642040734	NA	NA
ASV_16s_15790	0.203249702	0.394521429	3.551116287	0.609648575	NA	NA
ASV_16s_15791	0.184542399	0.38572635	3.551242608	0.5780371	NA	NA
ASV_16s_15792	0.184542399	0.38572635	3.551242608	0.5780371	NA	NA
ASV_16s_15793	0.152337572	0.370986212	3.55143315	0.515322957	NA	NA
ASV_16s_15794	0.152337572	0.370986212	3.55143315	0.515322957	NA	NA
ASV_16s_15795	0.239298729	-0.152790194	3.549969738	1.18478093	0.991273102	NA
ASV_16s_15796	0.05739026	0.341952344	3.551836488	0.250230199	NA	NA
ASV_16s_15797	0.11533526	0.353792081	3.551625815	0.414532697	0.999703181	NA
ASV_16s_15798	0.092604914	-0.389204005	3.551303297	0.369567036	NA	NA
ASV_16s_15799	0.092604914	-0.389204005	3.551303297	0.369567036	NA	NA
ASV_16s_15800	0.092604914	-0.389204005	3.551303297	0.369567036	NA	NA
ASV_16s_15801	0.092604914	-0.389204005	3.551303297	0.369567036	NA	NA
ASV_16s_15802	0.092604914	-0.389204005	3.551303297	0.369567036	NA	NA
ASV_16s_15803	0.146527513	0.548835532	3.550713751	0.782370672	0.997619731	NA
ASV_16s_15804	0.112466544	-0.440131264	3.551142883	0.424184684	NA	NA
ASV_16s_15805	0.103073168	-0.418553033	3.55122454	0.399154192	NA	NA
ASV_16s_15806	0.157361403	-0.078466164	3.550861619	0.820595302	NA	NA
ASV_16s_15807	0.157361403	-0.078466164	3.550861619	0.820595302	NA	NA
ASV_16s_15808	0.157361403	-0.078466164	3.550861619	0.820595302	NA	NA
ASV_16s_15809	0.157361403	-0.078466164	3.550861619	0.820595302	NA	NA
ASV_16s_15810	0.157361403	-0.078466164	3.550861619	0.820595302	NA	NA
ASV_16s_15811	0.165642073	-0.078522677	3.550844346	0.845025981	0.996956836	NA
ASV_16s_15812	0.177545536	-0.078596983	3.55082232	0.878468856	NA	NA
ASV_16s_15813	0.108523227	0.220134453	3.550843282	0.492634518	0.999473006	NA
ASV_16s_15814	0.110553983	0.220130749	3.550838401	0.500492557	0.999444676	NA
ASV_16s_15815	0.117150124	0.220119612	3.550823707	0.525403506	NA	NA
ASV_16s_15816	0.116916183	0.220119986	3.5508242	0.524533607	NA	NA
ASV_16s_15817	0.194536444	0.310942736	3.550924299	0.903642116	0.996236869	NA
ASV_16s_15818	0.12340841	0.595888366	3.550772498	0.486781426	NA	NA
ASV_16s_15819	0.222695478	0.519268592	3.551338165	0.721123396	NA	NA
ASV_16s_15820	0.18181358	0.313389302	3.551032771	0.753727129	0.997888011	NA
ASV_16s_15821	0.165983426	-0.06737794	3.550827442	0.941081448	0.995723817	NA
ASV_16s_15822	0.217660005	0.371332746	3.515920257	0.94855	0.995616353	NA
ASV_16s_15823	0.153979204	0.612416552	3.550599776	0.757149093	0.997857069	NA
ASV_16s_15824	0.181923806	-0.068078861	3.550732267	1.072079584	NA	NA
ASV_16s_15825	0.188602258	1.42454609	3.55105859	0.377987136	0.999782065	NA

ASV_16s_15826	0.198514484	-0.147883296	3.550688688	0.872399889	0.996632988	NA
ASV_16s_15827	0.210332663	1.671245449	3.550763378	0.279035193	0.999921736	NA
ASV_16s_15828	0.166280733	-0.068081277	3.550783363	1.050662579	0.993969359	NA
ASV_16s_15829	0.192840948	0.63367626	3.55157977	0.570819545	0.999143643	NA
ASV_16s_15830	0.16727177	-0.067766591	3.550758553	1.051831354	0.993948537	NA
ASV_16s_15831	0.167767288	-0.067623148	3.550755288	1.050673639	0.993969162	NA
ASV_16s_15832	0.361738313	1.547952002	3.550412762	0.605266389	NA	NA
ASV_16s_15833	0.286816288	0.532266709	3.550757422	1.464426527	NA	NA
ASV_16s_15834	0.188718207	0.35788705	3.55077866	1.041994262	0.994122369	NA
ASV_16s_15835	0.206471051	0.436355769	3.550894665	0.907178747	0.996190212	NA
ASV_16s_15836	0.187617974	0.956419009	3.551005776	0.679156011	NA	NA
ASV_16s_15837	0.19279235	0.33416564	3.551264631	0.635505772	0.998783706	NA
ASV_16s_15838	0.095659222	0.2849452	3.551758694	0.439579337	NA	NA
ASV_16s_15839	0.078780833	0.28674648	3.551814839	0.384646687	NA	NA
ASV_16s_15840	0.110469546	0.283554981	3.55171864	0.483467692	NA	NA
ASV_16s_15841	0.260961227	-0.590145228	3.550908656	0.767110823	NA	NA
ASV_16s_15842	0.050942491	-0.175285292	3.551761104	0.280872156	NA	NA
ASV_16s_15843	0.359433179	-0.564839631	3.54981008	0.860122194	NA	NA
ASV_16s_15844	0.232507331	0.345296046	3.551420608	0.530001942	NA	NA
ASV_16s_15845	0.132061285	-0.078243907	3.55092773	0.739263351	NA	NA
ASV_16s_15846	0.132061285	-0.078243907	3.55092773	0.739263351	NA	NA
ASV_16s_15847	0.296548122	0.416303699	3.550840327	1.832077496	0.968547177	NA
ASV_16s_15848	0.291505748	-0.029148341	3.550798268	1.184154197	NA	NA
ASV_16s_15849	0.241286281	0.204851965	3.550394681	1.30532858	0.988296215	NA
ASV_16s_15850	0.235166589	0.627353219	3.55098345	1.244788553	NA	NA
ASV_16s_15851	0.174236355	-0.028586787	3.550984807	0.928473619	NA	NA
ASV_16s_15852	0.145405256	-0.073235976	3.550927319	0.807718821	NA	NA
ASV_16s_15853	0.18209474	0.299790043	3.55063719	1.049768479	NA	NA
ASV_16s_15854	0.193500425	0.338536436	3.550520021	1.125960797	0.992532952	NA
ASV_16s_15855	0.142792567	-0.073209459	3.55093484	0.799302194	NA	NA
ASV_16s_15856	0.142792567	-0.073209459	3.55093484	0.799302194	NA	NA
ASV_16s_15857	0.142792567	-0.073209459	3.55093484	0.799302194	NA	NA
ASV_16s_15858	0.19869921	0.582316276	3.550349947	0.950520688	NA	NA
ASV_16s_15859	0.198755326	0.360318749	3.551104371	0.975944385	NA	NA
ASV_16s_15860	0.207266665	0.359830794	3.551440175	0.751583324	NA	NA
ASV_16s_15861	0.189280042	0.340441714	3.551228636	0.70274738	NA	NA
ASV_16s_15862	0.25686767	0.335335915	3.550947217	0.874241738	0.996610427	NA
ASV_16s_15863	0.176686618	0.360299421	3.55116761	0.91828673	NA	NA
ASV_16s_15864	0.176686618	0.360299421	3.55116761	0.91828673	NA	NA
ASV_16s_15865	0.176686618	0.360299421	3.55116761	0.91828673	NA	NA
ASV_16s_15866	0.176686618	0.360299421	3.55116761	0.91828673	NA	NA
ASV_16s_15867	0.20093827	0.315939671	3.550944197	0.942404514	0.995704905	NA
ASV_16s_15868	0.214797889	0.315939642	3.550921305	0.974908938	0.995223199	NA
ASV_16s_15869	0.191371552	0.315939694	3.550961935	0.918381707	0.996039945	NA
ASV_16s_15870	0.228725418	0.315939617	3.550901065	1.006434681	0.994724067	NA
ASV_16s_15871	0.190701921	0.315939696	3.550963239	0.916675532	0.996063073	NA
ASV_16s_15872	0.190017529	0.315939697	3.550964585	0.91491137	0.996086896	NA
ASV_16s_15873	0.190017529	0.315939697	3.550964585	0.91491137	0.996086896	NA
ASV_16s_15874	0.190017529	0.315939697	3.550964585	0.91491137	0.996086896	NA
ASV_16s_15875	0.189675334	0.315939698	3.550965262	0.914027195	0.9960988	NA
ASV_16s_15876	0.237135348	0.552256799	3.550851975	1.50240348	0.982226335	NA
ASV_16s_15877	0.188306551	0.559131589	3.550922653	1.263348142	0.989394442	NA
ASV_16s_15878	0.188306551	0.315939702	3.550967994	0.910476386	0.996146371	NA
ASV_16s_15879	0.21685792	0.315939638	3.550918173	0.979409249	0.99515389	NA
ASV_16s_15880	0.184542399	0.315939804	3.550975713	0.900593647	NA	NA
ASV_16s_15881	0.184542399	0.315939804	3.550975713	0.900593647	NA	NA
ASV_16s_15882	0.184542399	0.315939804	3.550975713	0.900593647	NA	NA
ASV_16s_15883	0.184542399	0.315939804	3.550975713	0.900593647	NA	NA
ASV_16s_15884	0.184542399	0.315939804	3.550975713	0.900593647	NA	NA
ASV_16s_15885	0.184542399	0.315939804	3.550975713	0.900593647	NA	NA
ASV_16s_15886	0.222131233	0.315939629	3.550910366	0.991748042	0.994960556	NA
ASV_16s_15887	0.20901706	0.746802342	3.550845419	1.238978185	0.990001568	NA
ASV_16s_15888	0.248477317	0.596176203	3.550660642	0.815799001	NA	NA
ASV_16s_15889	0.175746561	0.687702594	3.550931848	0.789731537	NA	NA
ASV_16s_15890	0.175746561	0.687702594	3.550931848	0.789731537	NA	NA
ASV_16s_15891	0.240803616	1.096350491	3.550742805	0.755583141	0.997871265	NA
ASV_16s_15892	0.244126918	0.734089589	3.551016003	1.015667979	NA	NA

ASV_16s_15893	0.244126918	0.734089589	3.551016003	1.015667979	NA	NA
ASV_16s_15894	0.244126918	0.734089589	3.551016003	1.015667979	NA	NA
ASV_16s_15895	0.244126918	0.734089589	3.551016003	1.015667979	NA	NA
ASV_16s_15896	0.244126918	0.734089589	3.551016003	1.015667979	NA	NA
ASV_16s_15897	0.364975897	0.734590148	3.550870794	1.218539591	NA	NA
ASV_16s_15898	0.120929651	0.724763162	3.551432553	0.685650965	NA	NA
ASV_16s_15899	0.120929651	0.724763162	3.551432553	0.685650965	NA	NA
ASV_16s_15900	0.120929651	0.724763162	3.551432553	0.685650965	NA	NA
ASV_16s_15901	0.120929651	0.724763162	3.551432553	0.685650965	NA	NA
ASV_16s_15902	0.120929651	0.724763162	3.551432553	0.685650965	NA	NA
ASV_16s_15903	0.210644886	0.692975551	3.550904007	0.901896131	0.996259765	NA
ASV_16s_15904	0.229766047	0.693075016	3.550874882	0.944849563	0.995669813	NA
ASV_16s_15905	0.230261419	0.693077375	3.550874191	0.945919279	0.995654403	NA
ASV_16s_15906	0.213548853	0.692991787	3.550899252	0.908625797	0.996171014	NA
ASV_16s_15907	0.194330832	0.692877253	3.550933354	0.862546059	0.99675203	NA
ASV_16s_15908	0.233398774	0.693093545	3.550869886	0.952645569	NA	NA
ASV_16s_15909	0.233398774	0.693093545	3.550869886	0.952645569	NA	NA
ASV_16s_15910	0.233398774	0.693093545	3.550869886	0.952645569	NA	NA
ASV_16s_15911	0.215860588	0.6930044	3.550895557	0.913922683	0.996100206	NA
ASV_16s_15912	0.217834761	0.693014905	3.55089248	0.918345826	0.996040432	NA
ASV_16s_15913	0.270611977	0.693242057	3.5508264	1.026667198	NA	NA
ASV_16s_15914	0.270611977	0.693242057	3.5508264	1.026667198	NA	NA
ASV_16s_15915	0.15502562	0.292235328	3.551762147	0.584838954	NA	NA
ASV_16s_15916	0.15502562	0.292235328	3.551762147	0.584838954	NA	NA
ASV_16s_15917	0.15502562	0.292235328	3.551762147	0.584838954	NA	NA
ASV_16s_15918	0.15502562	0.292235328	3.551762147	0.584838954	NA	NA
ASV_16s_15919	0.15502562	0.292235328	3.551762147	0.584838954	NA	NA
ASV_16s_15920	0.15502562	0.292235328	3.551762147	0.584838954	NA	NA
ASV_16s_15921	0.15502562	0.292235328	3.551762147	0.584838954	NA	NA
ASV_16s_15922	0.15502562	0.292235328	3.551762147	0.584838954	NA	NA
ASV_16s_15923	0.15502562	0.292235328	3.551762147	0.584838954	NA	NA
ASV_16s_15924	0.15502562	0.292235328	3.551762147	0.584838954	NA	NA
ASV_16s_15925	0.15502562	0.292235328	3.551762147	0.584838954	NA	NA
ASV_16s_15926	0.15502562	0.292235328	3.551762147	0.584838954	NA	NA
ASV_16s_15927	0.164328928	0.041620115	3.551294071	0.837519568	NA	NA
ASV_16s_15928	0.157096971	0.032749774	3.551332548	0.844687307	NA	NA
ASV_16s_15929	0.157355889	0.007555174	3.551288873	0.868057781	NA	NA
ASV_16s_15930	0.157614808	-0.016324831	3.551248638	0.889393962	0.996421076	NA
ASV_16s_15931	0.157873727	-0.038966306	3.551210754	0.908860804	0.99616789	NA
ASV_16s_15932	0.155116885	-0.127955058	3.551118103	0.962040863	0.99541785	NA
ASV_16s_15933	0.172356084	-0.18014363	3.550919668	1.033841197	0.994264003	NA
ASV_16s_15934	0.15942724	-0.160356057	3.55104591	0.98582135	0.995054026	NA
ASV_16s_15935	0.15942724	-0.160356057	3.55104591	0.98582135	0.995054026	NA
ASV_16s_15936	0.170278138	-0.225124536	3.550907359	1.02754412	0.99437189	NA
ASV_16s_15937	0.160203997	-0.216357112	3.550997868	0.989947647	0.994989069	NA
ASV_16s_15938	0.160203997	-0.216357112	3.550997868	0.989947647	0.994989069	NA
ASV_16s_15939	0.158431493	-0.211583397	3.551016795	0.98404489	0.995081824	NA
ASV_16s_15940	0.160462915	-0.234744777	3.550985911	0.987963771	0.995020368	NA
ASV_16s_15941	0.160721834	-0.253073108	3.55097584	0.984808574	0.995069887	NA
ASV_16s_15942	0.158955035	-0.266732248	3.550986637	0.970566628	0.995289466	NA
ASV_16s_15943	0.158448605	-0.265499951	3.550991792	0.968498491	0.995320819	NA
ASV_16s_15944	0.172605445	-0.297794282	3.550859971	1.012235764	0.994628734	NA
ASV_16s_15945	0.155162517	-0.27581532	3.551018118	0.945081157	0.99566648	NA
ASV_16s_15946	0.161239672	-0.29026865	3.550958019	0.967550639	0.995335143	NA
ASV_16s_15947	0.158206799	-0.301511402	3.550982144	0.943221625	0.995693198	NA
ASV_16s_15948	0.15517963	-0.331798469	3.55100396	0.902020123	0.996258142	NA
ASV_16s_15949	0.155185334	-0.350599615	3.551001474	0.884314809	0.996485295	NA
ASV_16s_15950	0.155191038	-0.369558084	3.551000054	0.864933204	NA	NA
ASV_16s_15951	0.155191038	-0.369558084	3.551000054	0.864933204	NA	NA
ASV_16s_15952	0.174238596	-0.505765944	3.55088221	0.78653734	0.997578929	NA
ASV_16s_15953	0.055419792	-0.218258262	3.55177906	0.307846848	NA	NA
ASV_16s_15954	0.055419792	-0.218258262	3.55177906	0.307846848	NA	NA
ASV_16s_15955	0.055419792	-0.218258262	3.55177906	0.307846848	NA	NA
ASV_16s_15956	0.211503812	-0.643598227	3.550700048	0.709049415	NA	NA
ASV_16s_15957	0.202694546	-0.635310595	3.550753186	0.694775557	0.998375558	NA
ASV_16s_15958	0.199912673	-0.632617983	3.550770803	0.690162328	0.998410188	NA
ASV_16s_15959	0.218120692	-0.649441281	3.550662279	0.719388396	NA	NA

ASV_16s_15960	0.205365212	-0.637858573	3.550736673	0.699154653	0.998342224	NA
ASV_16s_15961	0.18811392	-0.61636673	3.550834162	0.669257832	0.998560956	NA
ASV_16s_15962	0.17778086	-0.600176468	3.550891432	0.649796504	0.998692446	NA
ASV_16s_15963	0.16030667	-0.568677097	3.550992073	0.614469741	0.99891012	NA
ASV_16s_15964	0.159547025	-0.567194348	3.550996712	0.612857939	0.998919424	NA
ASV_16s_15965	0.159040596	-0.566201297	3.550999826	0.611779689	0.998925619	NA
ASV_16s_15966	0.155242374	-0.55865697	3.551023679	0.603622209	NA	NA
ASV_16s_15967	0.155242374	-0.55865697	3.551023679	0.603622209	NA	NA
ASV_16s_15968	0.155242374	-0.55865697	3.551023679	0.603622209	NA	NA
ASV_16s_15969	0.155242374	-0.55865697	3.551023679	0.603622209	NA	NA
ASV_16s_15970	0.049461213	-0.164801889	3.55102805	0.235054196	NA	NA
ASV_16s_15971	0.045784401	-0.161178215	3.551055853	0.213169973	0.999968713	NA
ASV_16s_15972	0.052152581	0.221301565	3.551121257	0.22844402	NA	NA
ASV_16s_15974	0.109848003	0.595818484	3.550799879	0.438553309	NA	NA
ASV_16s_15975	0.201797481	-0.625759051	3.550830321	0.698080677	0.998350441	NA
ASV_16s_15976	0.213989666	-0.674030833	3.550640322	0.769914937	0.997738996	NA
ASV_16s_15977	0.205590633	0.657624848	3.55170382	0.688500295	NA	NA
ASV_16s_15978	0.159242653	-0.211599112	3.550882162	0.646968764	NA	NA
ASV_16s_15979	0.159213465	0.760962766	3.551679487	0.531153385	NA	NA
ASV_16s_15980	0.197818745	-0.137287821	3.550493925	0.902687343	0.9962494	NA
ASV_16s_15981	0.201835437	-0.646651537	3.550646678	0.796285653	0.997481675	NA
ASV_16s_15982	0.164191686	-0.047211233	3.550933085	0.544336184	NA	NA
ASV_16s_15983	0.174163842	0.675357787	3.551536416	0.757452311	NA	NA
ASV_16s_15984	0.170414108	0.459031104	3.551344612	0.75606687	0.997866886	NA
ASV_16s_15985	0.177643519	0.681704959	3.551422265	0.798850173	NA	NA
ASV_16s_15986	0.209307958	-0.614375898	3.550486754	0.904482083	NA	NA
ASV_16s_15987	0.162611704	0.675347299	3.551429717	0.744810572	NA	NA
ASV_16s_15988	0.196655034	-0.568857105	3.550578419	0.886292123	0.996460384	NA
ASV_16s_15989	0.210172478	-0.586551132	3.550467665	0.936504475	0.995788825	NA
ASV_16s_15990	0.207695381	-0.565751249	3.550493042	0.941037708	NA	NA
ASV_16s_15991	0.193707668	1.421805182	3.550706454	0.341025791	0.999845791	NA
ASV_16s_15992	0.228757288	-0.597135933	3.550345109	1.026459679	0.994390337	NA
ASV_16s_15993	0.213809954	-0.551151176	3.550468134	0.978944902	0.995161071	NA
ASV_16s_15994	0.164413435	0.157664412	3.551792591	0.63900111	0.998761793	NA
ASV_16s_15995	0.192967304	-0.500128303	3.550622979	0.914481445	0.996092687	NA
ASV_16s_15996	0.194051141	-0.46163628	3.550636705	0.925813279	0.995938176	NA
ASV_16s_15997	0.159288632	0.674645094	3.551211883	0.603069283	0.998974789	NA
ASV_16s_15998	0.214250747	-0.122803508	3.550869464	0.99995229	NA	NA
ASV_16s_15999	0.16777683	-0.088068276	3.551024598	0.820012575	0.997234314	NA
ASV_16s_16000	0.213162903	-0.427304018	3.551003846	0.604887731	0.998964651	NA
ASV_16s_16001	0.173910803	-0.330075388	3.550859755	0.895979618	NA	NA
ASV_16s_16002	0.230482251	0.22904777	3.550856261	0.922927288	0.995977896	NA
ASV_16s_16003	0.190461472	-0.399187417	3.550700347	0.920376414	0.996012793	NA
ASV_16s_16004	0.175660207	-0.740662807	3.550710177	0.510217764	NA	NA
ASV_16s_16005	0.219795396	-0.266514513	3.550504119	0.845956856	0.996946173	NA
ASV_16s_16006	0.204623851	0.678217089	3.551048412	0.920833655	0.996006552	NA
ASV_16s_16007	0.175740965	-0.378004942	3.550951666	0.417955321	0.999694917	NA
ASV_16s_16008	0.245175656	0.949824797	3.550770468	0.778922565	0.997653152	NA
ASV_16s_16009	0.180970241	0.14742022	3.551699365	0.725226513	0.99813431	NA
ASV_16s_16010	0.210269528	0.681024468	3.550983992	0.913111159	NA	NA
ASV_16s_16011	0.189672765	0.381112159	3.551112261	0.821408743	0.997219282	NA
ASV_16s_16012	0.210752232	-0.165756957	3.550835488	0.965248939	0.99536981	NA
ASV_16s_16013	0.212599798	0.210276372	3.550808796	0.929686738	NA	NA
ASV_16s_16014	0.223606338	0.12350202	3.550765601	1.145769278	0.992122423	NA
ASV_16s_16015	0.095318684	0.552871116	3.551520216	0.313297446	NA	NA
ASV_16s_16016	0.199263545	1.513803784	3.550845261	0.370976725	NA	NA
ASV_16s_16017	0.28870662	0.334045721	3.551859697	0.527313213	NA	NA
ASV_16s_16018	0.209441252	1.362961754	3.550956445	0.559315789	0.999199007	NA
ASV_16s_16019	0.234277567	1.035820847	3.551325309	0.596173457	0.999012624	NA
ASV_16s_16020	0.255487796	0.70166087	3.550655551	1.2550682	0.989603207	NA
ASV_16s_16021	0.177435836	1.175933308	3.551110968	0.596248168	NA	NA
ASV_16s_16022	0.177435836	1.175933308	3.551110968	0.596248168	NA	NA
ASV_16s_16023	0.234060798	0.596202935	3.551584473	0.656250176	0.998649773	NA
ASV_16s_16024	0.270534187	0.498264126	3.550862272	0.936199622	NA	NA
ASV_16s_16025	0.168221365	0.358229449	3.551703033	0.612687063	NA	NA
ASV_16s_16026	0.168221365	0.358229449	3.551703033	0.612687063	NA	NA
ASV_16s_16027	0.168221365	0.358229449	3.551703033	0.612687063	NA	NA

ASV_16s_16028	0.20365248	0.37462075	3.551490338	0.676752369	NA	NA
ASV_16s_16029	0.20365248	0.37462075	3.551490338	0.676752369	NA	NA
ASV_16s_16030	0.215221795	0.364570721	3.551614138	0.44136853	NA	NA
ASV_16s_16031	0.218812723	0.880778113	3.551257787	0.661634367	NA	NA
ASV_16s_16032	0.13036151	0.343507039	3.55188174	0.528938224	NA	NA
ASV_16s_16033	0.13036151	0.343507039	3.55188174	0.528938224	NA	NA
ASV_16s_16034	0.13036151	0.343507039	3.55188174	0.528938224	NA	NA
ASV_16s_16035	0.176512393	0.362318522	3.55165838	0.628830006	NA	NA
ASV_16s_16036	0.176512393	0.362318522	3.55165838	0.628830006	NA	NA
ASV_16s_16037	0.223262066	0.68219292	3.550685084	0.652567667	NA	NA
ASV_16s_16038	0.146390616	-0.494567155	3.551155216	0.570497709	NA	NA
ASV_16s_16039	0.224545148	0.555180595	3.550520732	0.94596076	0.995653804	NA
ASV_16s_16040	0.130193502	-0.463978653	3.551286572	0.534497345	NA	NA
ASV_16s_16041	0.155202706	0.309000527	3.550952995	0.530032766	NA	NA
ASV_16s_16042	0.100742207	1.025391277	3.55132266	0.35854406	NA	NA
ASV_16s_16043	0.124851399	1.0958422	3.551153787	0.421567169	NA	NA
ASV_16s_16044	0.188202273	0.38774979	3.551218864	0.584419546	0.999074927	NA
ASV_16s_16045	0.219904825	-0.095302558	3.550100089	1.132460087	0.992399778	NA
ASV_16s_16046	0.148431481	0.369103905	3.551456802	0.506944582	NA	NA
ASV_16s_16047	0.148431481	0.369103905	3.551456802	0.506944582	NA	NA
ASV_16s_16048	0.15627003	0.584279104	3.551096315	0.707067363	NA	NA
ASV_16s_16049	0.090230429	-0.381440985	3.551320221	0.362456591	NA	NA
ASV_16s_16050	0.090230429	-0.381440985	3.551320221	0.362456591	NA	NA
ASV_16s_16051	0.201623799	-0.08588391	3.550125604	0.903697152	0.996236145	NA
ASV_16s_16052	0.294699612	-0.548075751	3.549814543	0.725912077	NA	NA
ASV_16s_16053	0.109582787	-0.433619452	3.551167812	0.416564788	NA	NA
ASV_16s_16054	0.109582787	-0.433619452	3.551167812	0.416564788	NA	NA
ASV_16s_16055	0.109582787	-0.433619452	3.551167812	0.416564788	NA	NA
ASV_16s_16056	0.109582787	-0.433619452	3.551167812	0.416564788	NA	NA
ASV_16s_16057	0.109582787	-0.433619452	3.551167812	0.416564788	NA	NA
ASV_16s_16058	0.079614782	0.950832963	3.551500083	0.297329525	NA	NA
ASV_16s_16059	0.179810543	-0.07861029	3.550818474	0.884555115	NA	NA
ASV_16s_16060	0.179810543	-0.07861029	3.550818474	0.884555115	NA	NA
ASV_16s_16061	0.179810543	-0.07861029	3.550818474	0.884555115	NA	NA
ASV_16s_16062	0.153326495	-0.078435687	3.550870727	0.808281526	NA	NA
ASV_16s_16063	0.153326495	-0.078435687	3.550870727	0.808281526	NA	NA
ASV_16s_16064	0.153326495	-0.078435687	3.550870727	0.808281526	NA	NA
ASV_16s_16065	0.153326495	-0.078435687	3.550870727	0.808281526	NA	NA
ASV_16s_16066	0.153326495	-0.078435687	3.550870727	0.808281526	NA	NA
ASV_16s_16067	0.153326495	-0.078435687	3.550870727	0.808281526	NA	NA
ASV_16s_16068	0.172993086	-0.078570052	3.550830404	0.865848758	NA	NA
ASV_16s_16069	0.173824787	0.310945336	3.550964184	0.848599999	0.996915763	NA
ASV_16s_16070	0.18783429	1.21250432	3.551028369	0.584578067	0.999074105	NA
ASV_16s_16071	0.209967454	0.608992142	3.550978214	0.713221569	NA	NA
ASV_16s_16072	0.152560305	0.35333206	3.551679724	0.415962888	NA	NA
ASV_16s_16073	0.17799767	-0.068051237	3.550785247	0.999020771	NA	NA
ASV_16s_16074	0.253881635	1.825177003	3.550433937	0.278743757	0.999922013	NA
ASV_16s_16075	0.212898849	1.377949113	3.550984625	0.565461472	0.999169741	NA
ASV_16s_16076	0.159768234	-0.067767059	3.550828693	0.986485906	NA	NA
ASV_16s_16077	0.193887213	1.63776743	3.550881797	0.23284847	0.999957706	NA
ASV_16s_16078	0.195750159	-0.064477061	3.550748614	0.915286573	0.996081837	NA
ASV_16s_16079	0.248235965	0.317188853	3.550934532	0.767199832	NA	NA
ASV_16s_16080	0.169854788	-0.060953773	3.55079745	1.021998605	0.994465819	NA
ASV_16s_16081	0.174573355	-0.061098488	3.550765975	1.03053049	NA	NA
ASV_16s_16082	0.172398451	-0.05456249	3.550834809	1.006818175	NA	NA
ASV_16s_16083	0.165912847	0.373479989	3.551601977	0.586138442	NA	NA
ASV_16s_16084	0.195678928	0.592898677	3.550705707	0.835958839	NA	NA
ASV_16s_16085	0.093206421	0.28519452	3.551766172	0.431903507	NA	NA
ASV_16s_16086	0.093206421	0.28519452	3.551766172	0.431903507	NA	NA
ASV_16s_16087	0.308824952	-0.160616682	3.549665468	1.193395156	NA	NA
ASV_16s_16088	0.209356764	0.32371003	3.55109699	0.643929893	0.998730445	NA
ASV_16s_16089	0.20365248	-0.541451666	3.550303521	0.608573812	NA	NA
ASV_16s_16090	0.225812107	1.267094415	3.550684493	0.619639846	NA	NA
ASV_16s_16091	0.186949236	0.672165826	3.550536681	0.780558988	0.99763733	NA
ASV_16s_16092	0.128675098	-0.078207715	3.55093856	0.727434972	NA	NA
ASV_16s_16093	0.128675098	-0.078207715	3.55093856	0.727434972	NA	NA
ASV_16s_16094	0.180836195	-0.108783729	3.550532478	1.05310858	0.993925729	NA

ASV_16s_16095	0.275267823	0.472796426	3.550875535	1.652688923	0.976548255	NA
ASV_16s_16096	0.271847566	0.475626451	3.550243643	1.463866739	0.983534294	NA
ASV_16s_16097	0.251472484	0.472536986	3.550252084	1.371576858	0.986425975	NA
ASV_16s_16098	0.169768756	-0.028550282	3.550996995	0.915886991	NA	NA
ASV_16s_16099	0.169768756	-0.028550282	3.550996995	0.915886991	NA	NA
ASV_16s_16100	0.169768756	-0.028550282	3.550996995	0.915886991	NA	NA
ASV_16s_16101	0.244405368	0.791461102	3.550293273	0.892120278	0.996386293	NA
ASV_16s_16102	0.176562162	-0.073490863	3.550854705	0.899890783	0.996285951	NA
ASV_16s_16103	0.141676917	-0.073197953	3.550938158	0.795609849	NA	NA
ASV_16s_16104	0.141676917	-0.073197953	3.550938158	0.795609849	NA	NA
ASV_16s_16105	0.139131219	-0.073170757	3.550945875	0.787248826	NA	NA
ASV_16s_16106	0.139131219	-0.073170757	3.550945875	0.787248826	NA	NA
ASV_16s_16107	0.206325791	0.290765503	3.550169006	1.118780965	NA	NA
ASV_16s_16108	0.20888331	0.344479761	3.550453982	1.150260882	0.992027406	NA
ASV_16s_16109	0.200097482	0.330114016	3.550479999	1.152137866	NA	NA
ASV_16s_16110	0.193659035	0.360314672	3.551117733	0.963076832	NA	NA
ASV_16s_16111	0.172156192	0.360294849	3.55118261	0.905627307	NA	NA
ASV_16s_16112	0.172156192	0.360294849	3.55118261	0.905627307	NA	NA
ASV_16s_16113	0.172156192	0.360294849	3.55118261	0.905627307	NA	NA
ASV_16s_16114	0.172156192	0.360294849	3.55118261	0.905627307	NA	NA
ASV_16s_16115	0.172156192	0.360294849	3.55118261	0.905627307	NA	NA
ASV_16s_16116	0.172156192	0.360294849	3.55118261	0.905627307	NA	NA
ASV_16s_16117	0.250126033	0.639964101	3.550969734	1.431031541	NA	NA
ASV_16s_16118	0.213478725	0.613953779	3.550797278	1.100146214	0.993047339	NA
ASV_16s_16119	0.247150674	0.315939606	3.550877774	1.045693899	0.994057371	NA
ASV_16s_16120	0.199159809	0.315939693	3.550947388	0.937939119	0.995768517	NA
ASV_16s_16121	0.192813977	0.315939785	3.550959164	0.922021027	NA	NA
ASV_16s_16122	0.186996651	0.315939724	3.550970665	0.906989627	0.996192716	NA
ASV_16s_16123	0.186654455	0.315939725	3.550971364	0.906092687	0.996204579	NA
ASV_16s_16124	0.184601282	0.315939731	3.55097561	0.900680538	0.996275652	NA
ASV_16s_16125	0.19890335	0.315939524	3.550947871	0.936253392	0.995792372	NA
ASV_16s_16126	0.183574695	0.315939734	3.550977769	0.897954634	0.99631112	NA
ASV_16s_16127	0.183574695	0.315939734	3.550977769	0.897954634	0.99631112	NA
ASV_16s_16128	0.182548108	0.346370282	3.550965616	1.02372202	0.994436736	NA
ASV_16s_16129	0.179810543	0.315939827	3.550985894	0.887849855	NA	NA
ASV_16s_16130	0.179810543	0.315939827	3.550985894	0.887849855	NA	NA
ASV_16s_16131	0.171240239	0.6876718	3.550941742	0.777387864	NA	NA
ASV_16s_16132	0.171240239	0.6876718	3.550941742	0.777387864	NA	NA
ASV_16s_16133	0.171240239	0.6876718	3.550941742	0.777387864	NA	NA
ASV_16s_16134	0.171240239	0.6876718	3.550941742	0.777387864	NA	NA
ASV_16s_16135	0.246386447	0.984343571	3.550826302	0.809545353	0.997345315	NA
ASV_16s_16136	0.248105866	0.734112582	3.551009013	1.023667732	0.994437654	NA
ASV_16s_16137	0.237867253	0.734049965	3.551027537	1.002650338	NA	NA
ASV_16s_16138	0.237867253	0.734049965	3.551027537	1.002650338	NA	NA
ASV_16s_16139	0.237867253	0.734049965	3.551027537	1.002650338	NA	NA
ASV_16s_16140	0.237867253	0.734049965	3.551027537	1.002650338	NA	NA
ASV_16s_16141	0.237867253	0.734049965	3.551027537	1.002650338	NA	NA
ASV_16s_16142	0.237867253	0.734049965	3.551027537	1.002650338	NA	NA
ASV_16s_16143	0.237867253	0.734049965	3.551027537	1.002650338	NA	NA
ASV_16s_16144	0.237867253	0.734049965	3.551027537	1.002650338	NA	NA
ASV_16s_16145	0.237867253	0.734049965	3.551027537	1.002650338	NA	NA
ASV_16s_16146	0.117828891	0.723065989	3.551448492	0.674599298	NA	NA
ASV_16s_16147	0.22113948	0.693033904	3.550887417	0.925808406	NA	NA
ASV_16s_16148	0.19394939	0.692873261	3.55093412	0.861523202	0.996764227	NA
ASV_16s_16149	0.237033861	0.69310891	3.55086506	0.960249411	0.995444536	NA
ASV_16s_16150	0.22741419	0.693065272	3.550878218	0.939674247	NA	NA
ASV_16s_16151	0.22741419	0.693065272	3.550878218	0.939674247	NA	NA
ASV_16s_16152	0.22741419	0.693065272	3.550878218	0.939674247	NA	NA
ASV_16s_16153	0.238048585	0.693113444	3.550863732	0.962379359	0.995412796	NA
ASV_16s_16154	0.201106911	0.692919312	3.550920613	0.879136273	0.996549994	NA
ASV_16s_16155	0.179810543	0.692773342	3.550963937	0.825173746	NA	NA
ASV_16s_16156	0.179810543	0.692773342	3.550963937	0.825173746	NA	NA
ASV_16s_16157	0.179810543	0.692773342	3.550963937	0.825173746	NA	NA
ASV_16s_16158	0.179810543	0.692773342	3.550963937	0.825173746	NA	NA
ASV_16s_16159	0.199672753	0.692910297	3.550923256	0.875431802	0.996595797	NA
ASV_16s_16160	0.208663923	0.692964493	3.550907366	0.897122565	0.996321902	NA
ASV_16s_16161	0.263673209	0.69321645	3.550833597	1.013548864	NA	NA

ASV_16s_16162	0.150945998	0.292847599	3.551773776	0.57581696	NA	NA
ASV_16s_16163	0.150945998	0.292847599	3.551773776	0.57581696	NA	NA
ASV_16s_16164	0.150945998	0.292847599	3.551773776	0.57581696	NA	NA
ASV_16s_16165	0.150945998	0.292847599	3.551773776	0.57581696	NA	NA
ASV_16s_16166	0.150945998	0.292847599	3.551773776	0.57581696	NA	NA
ASV_16s_16167	0.150945998	0.292847599	3.551773776	0.57581696	NA	NA
ASV_16s_16168	0.150945998	0.292847599	3.551773776	0.57581696	NA	NA
ASV_16s_16169	0.150945998	0.292847599	3.551773776	0.57581696	NA	NA
ASV_16s_16170	0.150945998	0.292847599	3.551773776	0.57581696	NA	NA
ASV_16s_16171	0.150945998	0.292847599	3.551773776	0.57581696	NA	NA
ASV_16s_16172	0.150945998	0.292847599	3.551773776	0.57581696	NA	NA
ASV_16s_16173	0.150945998	0.292847599	3.551773776	0.57581696	NA	NA
ASV_16s_16174	0.150974519	0.122704152	3.551521111	0.743780531	NA	NA
ASV_16s_16175	0.150980223	0.094602352	3.551478387	0.771563527	NA	NA
ASV_16s_16176	0.16490096	-0.047002265	3.551134233	0.918032191	0.99604469	NA
ASV_16s_16177	0.154053024	-0.064564877	3.551207536	0.911819187	0.996128426	NA
ASV_16s_16178	0.151014447	-0.051248782	3.551249968	0.897477723	0.996317302	NA
ASV_16s_16179	0.154570862	-0.106691479	3.551147881	0.940350227	0.995734246	NA
ASV_16s_16180	0.1550887	-0.14671765	3.551099236	0.960221653	0.995444948	NA
ASV_16s_16181	0.153822626	-0.142602853	3.551115465	0.95510062	0.995520676	NA
ASV_16s_16182	0.1550887	-0.14671765	3.551099236	0.960221653	0.995444948	NA
ASV_16s_16183	0.1550887	-0.14671765	3.551099236	0.960221653	0.995444948	NA
ASV_16s_16184	0.1550887	-0.14671765	3.551099236	0.960221653	0.995444948	NA
ASV_16s_16185	0.155865456	-0.204456928	3.551045315	0.966686614	0.995348176	NA
ASV_16s_16186	0.15106008	-0.20944526	3.551089085	0.949005298	0.995609746	NA
ASV_16s_16187	0.166457435	-0.249531714	3.550927495	1.000567566	0.994819376	NA
ASV_16s_16188	0.153592227	-0.216494254	3.551059908	0.957226397	0.99548934	NA
ASV_16s_16189	0.156383294	-0.242171013	3.551020419	0.962867961	0.995405495	NA
ASV_16s_16190	0.151065784	-0.228125659	3.55107844	0.946466776	0.995646502	NA
ASV_16s_16191	0.156642213	-0.261008388	3.551010668	0.958673125	0.995467934	NA
ASV_16s_16192	0.15716005	-0.29905774	3.550992512	0.939360601	0.995748334	NA
ASV_16s_16193	0.163618213	-0.313563708	3.550931829	0.957526133	0.995484911	NA
ASV_16s_16194	0.157677888	-0.337298059	3.550981012	0.910346688	0.996148102	NA
ASV_16s_16195	0.152366082	-0.343753552	3.551024572	0.874858507	0.99660285	NA
ASV_16s_16196	0.153885371	-0.347525312	3.551012558	0.879929409	0.996540135	NA
ASV_16s_16197	0.151128528	-0.440277795	3.551038561	0.757900978	NA	NA
ASV_16s_16198	0.176281542	-0.508896242	3.550868207	0.791161085	0.997533114	NA
ASV_16s_16199	0.154679239	-0.469398286	3.55101532	0.73870587	0.998020342	NA
ASV_16s_16200	0.053961377	-0.210401577	3.551787179	0.301874665	NA	NA
ASV_16s_16201	0.208898106	-0.641158522	3.55071551	0.70482632	0.998298378	NA
ASV_16s_16202	0.193883138	-0.624811178	3.550803992	0.679634647	0.998487368	NA
ASV_16s_16203	0.193419492	-0.624140368	3.550806367	0.678809293	0.998493311	NA
ASV_16s_16204	0.212380674	-0.644379995	3.550695116	0.710382158	NA	NA
ASV_16s_16205	0.200262968	-0.632936337	3.55076872	0.690685426	0.998406286	NA
ASV_16s_16206	0.208316397	-0.640617197	3.55071897	0.703890972	NA	NA
ASV_16s_16207	0.174329757	-0.594442512	3.5509116	0.642995917	0.998736426	NA
ASV_16s_16208	0.160525995	-0.569070779	3.550990845	0.614880334	NA	NA
ASV_16s_16209	0.160525995	-0.569070779	3.550990845	0.614880334	NA	NA
ASV_16s_16210	0.160525995	-0.569070779	3.550990845	0.614880334	NA	NA
ASV_16s_16211	0.17118161	-0.588835054	3.550928848	0.636350658	0.998778433	NA
ASV_16s_16212	0.153689196	-0.555487142	3.551033812	0.600187619	0.998990716	NA
ASV_16s_16213	0.151157049	-0.550311491	3.551050491	0.594629242	NA	NA
ASV_16s_16214	0.151157049	-0.550311491	3.551050491	0.594629242	NA	NA
ASV_16s_16215	0.151157049	-0.550311491	3.551050491	0.594629242	NA	NA
ASV_16s_16216	0.151157049	-0.550311491	3.551050491	0.594629242	NA	NA
ASV_16s_16217	0.151157049	-0.550311491	3.551050491	0.594629242	NA	NA
ASV_16s_16218	0.151157049	-0.550311491	3.551050491	0.594629242	NA	NA
ASV_16s_16219	0.048159602	-0.163550264	3.551037452	0.227353496	NA	NA
ASV_16s_16220	0.065639989	0.175494059	3.550895158	0.464161912	0.999567413	NA
ASV_16s_16221	0.049434461	0.024542286	3.551026725	0.356416451	0.999821094	NA
ASV_16s_16222	0.042717921	-0.157922493	3.551082503	0.194410751	NA	NA
ASV_16s_16223	0.042717921	-0.157922493	3.551082503	0.194410751	NA	NA
ASV_16s_16224	0.042717921	-0.157922493	3.551082503	0.194410751	NA	NA
ASV_16s_16225	0.046422186	0.026130914	3.551055591	0.327583004	0.999865341	NA
ASV_16s_16226	0.051254026	-0.166466109	3.551015886	0.245465109	NA	NA
ASV_16s_16227	0.050780145	0.221897571	3.551131089	0.22057358	NA	NA
ASV_16s_16228	0.195830346	-0.51773942	3.550849237	0.91577054	0.996075306	NA

ASV_16s_16229	0.154486171	0.61720886	3.551805615	0.60252909	NA	NA
ASV_16s_16230	0.165014387	0.168915544	3.551209664	0.842629704	0.996984174	NA
ASV_16s_16231	0.15682214	0.380228675	3.551415441	0.785828976	0.997585898	NA
ASV_16s_16232	0.198338559	-0.644428784	3.550697766	0.76629638	0.99777289	NA
ASV_16s_16233	0.194542946	-0.636714908	3.550724525	0.756601589	0.997862039	NA
ASV_16s_16234	0.16114915	0.306082152	3.551966943	0.451499992	NA	NA
ASV_16s_16235	0.163287292	0.121977239	3.550960934	0.697013	0.998358582	NA
ASV_16s_16236	0.195031112	-0.631887076	3.550691508	0.780555307	0.997637366	NA
ASV_16s_16237	0.16956088	0.006558516	3.550875786	0.557854262	0.999205863	NA
ASV_16s_16238	0.196032099	-0.612518747	3.550613127	0.826374168	0.997165387	NA
ASV_16s_16239	0.156657433	-0.137407058	3.550848885	0.688578234	NA	NA
ASV_16s_16240	0.204197421	-0.613366966	3.550532711	0.874173108	NA	NA
ASV_16s_16241	0.186313642	-0.572974866	3.550674657	0.829527549	0.997130808	NA
ASV_16s_16242	0.199107634	-0.571513557	3.55055733	0.89166373	NA	NA
ASV_16s_16243	0.188907431	-0.539670742	3.550643681	0.866791014	NA	NA
ASV_16s_16244	0.182739789	-0.090456262	3.550648951	0.853206145	0.996862296	NA
ASV_16s_16245	0.183724337	0.459154984	3.550988842	0.661815301	0.998612237	NA
ASV_16s_16246	0.195056419	-0.539577737	3.55059392	0.895112527	0.996347865	NA
ASV_16s_16247	0.224074063	-0.586212623	3.551017676	0.813668156	0.99730195	NA
ASV_16s_16248	0.198472795	-0.536336935	3.550570963	0.913321266	0.996108288	NA
ASV_16s_16249	0.199754296	-0.212776223	3.550378921	0.643283024	0.998734589	NA
ASV_16s_16250	0.201679497	-0.547869935	3.550543551	0.921978669	0.995990896	NA
ASV_16s_16251	0.215583456	0.002611661	3.550731542	1.022140222	0.994463433	NA
ASV_16s_16252	0.195079168	-0.502146407	3.550850348	0.76363988	0.997797557	NA
ASV_16s_16253	0.19492398	-0.485104991	3.550617705	0.918811349	0.996034107	NA
ASV_16s_16254	0.198369945	-0.478637803	3.550603721	0.934673807	0.995814646	NA
ASV_16s_16255	0.211263647	-0.152470998	3.550559637	0.927903501	0.995909251	NA
ASV_16s_16256	0.200792986	-0.436779645	3.550624163	0.949689893	NA	NA
ASV_16s_16257	0.211350994	-0.087523028	3.550596143	0.951612699	0.995571786	NA
ASV_16s_16258	0.128432189	0.665266988	3.551726715	0.691277469	0.998401863	NA
ASV_16s_16259	0.214433266	-0.481531969	3.550531949	1.000682876	NA	NA
ASV_16s_16260	0.164234298	-0.703445951	3.550792384	0.495407388	NA	NA
ASV_16s_16261	0.203623598	1.369077044	3.551020922	0.490972642	0.999478869	NA
ASV_16s_16262	0.219718502	0.153323902	3.551213588	0.775002632	0.997690769	NA
ASV_16s_16263	0.197662588	-0.383238855	3.550686196	0.939005632	NA	NA
ASV_16s_16264	0.235789178	-0.577811932	3.550742206	0.732946868	NA	NA
ASV_16s_16265	0.141154331	0.64991041	3.551792227	0.514730491	0.999390737	NA
ASV_16s_16266	0.179967852	-0.39999644	3.551186641	0.421991207	0.999684972	NA
ASV_16s_16267	0.170265815	1.355242063	3.551332895	0.356402044	0.999821118	NA
ASV_16s_16268	0.173786867	-0.209157072	3.550854696	0.852816043	NA	NA
ASV_16s_16269	0.190342821	-0.403938203	3.551054399	0.633009961	0.998799193	NA
ASV_16s_16270	0.186458519	-0.165748113	3.550862278	0.88813083	0.996437117	NA
ASV_16s_16271	0.187003887	0.265504154	3.550929339	0.84753142	NA	NA
ASV_16s_16272	0.166081576	-0.038076942	3.551427005	0.70449684	NA	NA
ASV_16s_16273	0.182555145	0.152965977	3.551493583	0.6397432	NA	NA
ASV_16s_16274	0.192032949	-0.002455036	3.550888133	0.887500092	0.99644511	NA
ASV_16s_16275	0.192425112	0.0469419	3.551087702	0.945880233	NA	NA
ASV_16s_16276	0.174687595	0.357964766	3.551314321	0.748862843	NA	NA
ASV_16s_16277	0.179927966	-0.531611663	3.550958329	0.617589345	NA	NA
ASV_16s_16278	0.213642961	0.733114114	3.550385004	0.800461335	0.997439242	NA
ASV_16s_16279	0.201610765	1.215843243	3.55100036	0.639184755	0.998760634	NA
ASV_16s_16280	0.188301593	1.194652315	3.551058654	0.616791679	NA	NA
ASV_16s_16281	0.188301593	1.194652315	3.551058654	0.616791679	NA	NA
ASV_16s_16282	0.172766472	1.167478099	3.551135159	0.587037291	NA	NA
ASV_16s_16283	0.172766472	1.167478099	3.551135159	0.587037291	NA	NA
ASV_16s_16284	0.168644208	0.826464787	3.55111781	0.953832396	0.995539304	NA
ASV_16s_16285	0.194095196	1.439030454	3.550819253	0.486041004	0.999496005	NA
ASV_16s_16286	0.163794487	0.355946963	3.551728526	0.603748305	NA	NA
ASV_16s_16287	0.128114961	-0.272246479	3.551609267	0.481978955	0.999509828	NA
ASV_16s_16288	0.198293204	0.372503582	3.551523534	0.667728434	NA	NA
ASV_16s_16289	0.193704688	0.370635587	3.551553375	0.658396459	NA	NA
ASV_16s_16290	0.126930944	0.342392824	3.551892329	0.520378234	NA	NA
ASV_16s_16291	0.126930944	0.342392824	3.551892329	0.520378234	NA	NA
ASV_16s_16292	0.126930944	0.342392824	3.551892329	0.520378234	NA	NA
ASV_16s_16293	0.17186733	0.360043124	3.551683064	0.619812447	NA	NA
ASV_16s_16294	0.203297826	-0.106865352	3.55165114	0.59932208	NA	NA
ASV_16s_16295	0.177168391	0.963247935	3.551407382	0.581763233	NA	NA

ASV_16s_16296	0.186415462	-0.178943363	3.551531669	0.666172597	0.998582365	NA
ASV_16s_16297	0.195763282	-0.789049833	3.550771051	0.526742529	NA	NA
ASV_16s_16298	0.212380674	-0.552598273	3.550553958	0.691358941	NA	NA
ASV_16s_16299	0.172041684	-0.525587189	3.550896705	0.623192853	0.998858828	NA
ASV_16s_16300	0.197783075	0.25733389	3.550437036	1.071099324	0.993598645	NA
ASV_16s_16301	0.115091177	1.068893605	3.551215907	0.395955415	0.99974537	NA
ASV_16s_16302	0.098091097	1.016625198	3.551345035	0.351122744	NA	NA
ASV_16s_16303	0.098091097	1.016625198	3.551345035	0.351122744	NA	NA
ASV_16s_16304	0.121565836	1.087007822	3.551173769	0.413319354	NA	NA
ASV_16s_16305	0.121565836	1.087007822	3.551173769	0.413319354	NA	NA
ASV_16s_16306	0.168162695	1.189927027	3.550929734	0.518585837	NA	NA
ASV_16s_16307	0.168162695	1.189927027	3.550929734	0.518585837	NA	NA
ASV_16s_16308	0.261454368	0.415617426	3.550811813	0.693652407	NA	NA
ASV_16s_16309	0.144525389	0.367170232	3.551481536	0.498399613	NA	NA
ASV_16s_16310	0.2475786	0.411821552	3.550880173	0.675447018	NA	NA
ASV_16s_16311	0.156371348	0.372857714	3.551410047	0.523668533	NA	NA
ASV_16s_16312	0.156371348	0.372857714	3.551410047	0.523668533	NA	NA
ASV_16s_16313	0.166668375	0.603310147	3.551000059	0.746081649	NA	NA
ASV_16s_16314	0.05444717	0.341748808	3.551848657	0.239320047	NA	NA
ASV_16s_16315	0.087855944	-0.373465373	3.551337753	0.355219446	NA	NA
ASV_16s_16316	0.087855944	-0.373465373	3.551337753	0.355219446	NA	NA
ASV_16s_16317	0.215518103	-0.543519582	3.55021897	0.626381321	NA	NA
ASV_16s_16318	0.106699029	-0.426949254	3.551193756	0.408830058	NA	NA
ASV_16s_16319	0.106699029	-0.426949254	3.551193756	0.408830058	NA	NA
ASV_16s_16320	0.106699029	-0.426949254	3.551193756	0.408830058	NA	NA
ASV_16s_16321	0.097787364	-0.404836805	3.551266661	0.384437756	NA	NA
ASV_16s_16322	0.097787364	-0.404836805	3.551266661	0.384437756	NA	NA
ASV_16s_16323	0.097787364	-0.404836805	3.551266661	0.384437756	NA	NA
ASV_16s_16324	0.175078687	-0.078582915	3.550826673	0.871563091	NA	NA
ASV_16s_16325	0.149291588	-0.078402177	3.550880326	0.795717129	NA	NA
ASV_16s_16326	0.149291588	-0.078402177	3.550880326	0.795717129	NA	NA
ASV_16s_16327	0.149291588	-0.078402177	3.550880326	0.795717129	NA	NA
ASV_16s_16328	0.149291588	-0.078402177	3.550880326	0.795717129	NA	NA
ASV_16s_16329	0.149291588	-0.078402177	3.550880326	0.795717129	NA	NA
ASV_16s_16330	0.10385137	0.220143573	3.550855263	0.474104719	0.999535884	NA
ASV_16s_16331	0.107188431	0.220137016	3.55084662	0.487311907	0.999491627	NA
ASV_16s_16332	0.111142425	0.220129761	3.550837046	0.502651685	NA	NA
ASV_16s_16333	0.142854188	0.212944523	3.550820548	0.383161427	NA	NA
ASV_16s_16334	0.192892289	0.310942956	3.550927189	0.89933452	NA	NA
ASV_16s_16335	0.189089878	0.310943395	3.550933978	0.889563043	NA	NA
ASV_16s_16336	0.272940372	0.813219952	3.550446813	1.343248067	0.987246467	NA
ASV_16s_16337	0.21793531	-0.140667547	3.550861573	0.874888478	0.996602481	NA
ASV_16s_16338	0.157163898	0.536715746	3.55171805	0.625824257	NA	NA
ASV_16s_16339	0.265588235	0.789590409	3.551058945	0.775610301	NA	NA
ASV_16s_16340	0.155237807	-0.067921408	3.550860388	0.957075394	NA	NA
ASV_16s_16341	0.231020503	-1.055227242	3.550775554	0.44765309	NA	NA
ASV_16s_16342	0.21138156	0.668311852	3.550357263	0.693614506	NA	NA
ASV_16s_16343	0.149522301	-0.051022624	3.551235551	0.744482979	0.997970118	NA
ASV_16s_16344	0.209946599	1.524316043	3.550786388	0.355452146	0.999822716	NA
ASV_16s_16345	0.158210917	-0.06786368	3.550850383	1.003314033	0.994774899	NA
ASV_16s_16346	0.192188441	1.40577927	3.551098361	0.378051857	0.99978194	NA
ASV_16s_16347	0.159697472	-0.067318127	3.550847938	1.000861429	0.994814629	NA
ASV_16s_16348	0.183409751	0.456317153	3.551322998	0.824381855	0.997187093	NA
ASV_16s_16349	0.134637465	1.135829645	3.551497797	0.451546523	0.999605271	NA
ASV_16s_16350	0.137693218	0.309244904	3.551482035	0.698851876	NA	NA
ASV_16s_16351	0.169035778	-0.012614157	3.551090177	0.931616237	NA	NA
ASV_16s_16352	0.181731531	0.320808294	3.551200863	0.692101073	0.998395695	NA
ASV_16s_16353	0.276599436	1.191042762	3.550587056	0.743462153	NA	NA
ASV_16s_16354	0.296812336	0.281658567	3.551328021	0.831409135	NA	NA
ASV_16s_16355	0.125861501	0.282278861	3.551684217	0.525527465	NA	NA
ASV_16s_16356	0.07474079	0.287217345	3.551830353	0.370540771	NA	NA
ASV_16s_16357	0.07474079	0.287217345	3.551830353	0.370540771	NA	NA
ASV_16s_16358	0.07474079	0.287217345	3.551830353	0.370540771	NA	NA
ASV_16s_16359	0.07474079	0.287217345	3.551830353	0.370540771	NA	NA
ASV_16s_16360	0.252501213	0.358510045	3.551568771	0.52958672	NA	NA
ASV_16s_16361	0.297288256	0.356874518	3.551363535	0.531522447	0.999322677	NA
ASV_16s_16362	0.219869683	1.261239976	3.550709591	0.610382362	NA	NA

ASV_16s_16363	0.125288911	-0.07816957	3.550949972	0.715397514	NA	NA
ASV_16s_16364	0.153617312	-0.114506409	3.55065034	0.945810819	0.995655967	NA
ASV_16s_16365	0.278270169	0.307056631	3.550839688	1.848035664	0.967770363	NA
ASV_16s_16366	0.224306956	0.263508121	3.550084664	0.985263255	0.99506277	NA
ASV_16s_16367	0.223464721	0.294437705	3.550908365	1.560209951	0.980151685	NA
ASV_16s_16368	0.227943298	0.505326068	3.550951009	1.424626086	0.984805023	NA
ASV_16s_16369	0.22264726	0.621963398	3.551004392	1.213335341	NA	NA
ASV_16s_16370	0.165301157	-0.028511812	3.551009838	0.903011078	NA	NA
ASV_16s_16371	0.165301157	-0.028511812	3.551009838	0.903011078	NA	NA
ASV_16s_16372	0.165301157	-0.028511812	3.551009838	0.903011078	NA	NA
ASV_16s_16373	0.165301157	-0.028511812	3.551009838	0.903011078	NA	NA
ASV_16s_16374	0.165301157	-0.028511812	3.551009838	0.903011078	NA	NA
ASV_16s_16375	0.137948577	-0.07315788	3.55094958	0.783260099	NA	NA
ASV_16s_16376	0.137948577	-0.07315788	3.55094958	0.783260099	NA	NA
ASV_16s_16377	0.137948577	-0.07315788	3.55094958	0.783260099	NA	NA
ASV_16s_16378	0.15262437	0.137623746	3.550907481	0.849788263	NA	NA
ASV_16s_16379	0.135469871	-0.073129966	3.550957502	0.774942022	NA	NA
ASV_16s_16380	0.135469871	-0.073129966	3.550957502	0.774942022	NA	NA
ASV_16s_16381	0.135469871	-0.073129966	3.550957502	0.774942022	NA	NA
ASV_16s_16382	0.222446905	0.126344443	3.550807995	1.590636849	0.979004922	NA
ASV_16s_16383	0.188562745	0.360310376	3.551131813	0.949910654	NA	NA
ASV_16s_16384	0.188562745	0.360310376	3.551131813	0.949910654	NA	NA
ASV_16s_16385	0.188562745	0.360310376	3.551131813	0.949910654	NA	NA
ASV_16s_16386	0.177265291	0.332221744	3.551128602	0.723923811	NA	NA
ASV_16s_16387	0.167625765	0.360290032	3.551198414	0.892683803	NA	NA
ASV_16s_16388	0.167625765	0.360290032	3.551198414	0.892683803	NA	NA
ASV_16s_16389	0.167625765	0.360290032	3.551198414	0.892683803	NA	NA
ASV_16s_16390	0.167625765	0.360290032	3.551198414	0.892683803	NA	NA
ASV_16s_16391	0.192047343	0.315939728	3.550960666	0.919972162	0.996018305	NA
ASV_16s_16392	0.187739925	0.31593974	3.550969177	0.908865815	NA	NA
ASV_16s_16393	0.22979091	0.315939647	3.550899654	1.008584333	0.994688868	NA
ASV_16s_16394	0.178842838	0.315939766	3.550988059	0.885152635	0.996474754	NA
ASV_16s_16395	0.175078687	0.315939778	3.550996621	0.874827057	NA	NA
ASV_16s_16396	0.175078687	0.315939778	3.550996621	0.874827057	NA	NA
ASV_16s_16397	0.175078687	0.315939778	3.550996621	0.874827057	NA	NA
ASV_16s_16398	0.175078687	0.315939778	3.550996621	0.874827057	NA	NA
ASV_16s_16399	0.235734891	0.596160955	3.550666631	0.789419307	NA	NA
ASV_16s_16400	0.36006543	0.734576503	3.550874837	1.211525173	0.990659108	NA
ASV_16s_16401	0.253953632	1.143788254	3.550713594	0.77033278	0.997735061	NA
ASV_16s_16402	0.246858283	1.146866439	3.550767809	0.762012809	0.997812577	NA
ASV_16s_16403	0.235556649	1.067974645	3.550780172	0.754672633	NA	NA
ASV_16s_16404	0.231607589	0.734008214	3.551039691	0.989308774	NA	NA
ASV_16s_16405	0.231607589	0.734008214	3.551039691	0.989308774	NA	NA
ASV_16s_16406	0.231607589	0.734008214	3.551039691	0.989308774	NA	NA
ASV_16s_16407	0.231607589	0.734008214	3.551039691	0.989308774	NA	NA
ASV_16s_16408	0.231607589	0.734008214	3.551039691	0.989308774	NA	NA
ASV_16s_16409	0.231607589	0.734008214	3.551039691	0.989308774	NA	NA
ASV_16s_16410	0.231607589	0.734008214	3.551039691	0.989308774	NA	NA
ASV_16s_16411	0.155793886	0.733239588	3.551263273	0.795207279	0.997492557	NA
ASV_16s_16412	0.204863876	0.842870426	3.550923569	0.794463643	0.997500044	NA
ASV_16s_16413	0.114728131	0.721330479	3.55146522	0.663364944	NA	NA
ASV_16s_16414	0.114728131	0.721330479	3.55146522	0.663364944	NA	NA
ASV_16s_16415	0.114728131	0.721330479	3.55146522	0.663364944	NA	NA
ASV_16s_16416	0.114728131	0.721330479	3.55146522	0.663364944	NA	NA
ASV_16s_16417	0.114728131	0.721330479	3.55146522	0.663364944	NA	NA
ASV_16s_16418	0.114728131	0.721330479	3.55146522	0.663364944	NA	NA
ASV_16s_16419	0.197918362	0.692899493	3.550926529	0.871293172	0.996646498	NA
ASV_16s_16420	0.218292251	0.69301797	3.550891783	0.919337296	0.996026952	NA
ASV_16s_16421	0.218292251	0.69301797	3.550891783	0.919337296	0.996026952	NA
ASV_16s_16422	0.200315505	0.692914626	3.550922089	0.877014782	0.996576273	NA
ASV_16s_16423	0.221429606	0.693035474	3.550886999	0.926386048	NA	NA
ASV_16s_16424	0.221429606	0.693035474	3.550886999	0.926386048	NA	NA
ASV_16s_16425	0.247356113	0.693153493	3.550852085	0.98144042	0.995122398	NA
ASV_16s_16426	0.175078687	0.692735837	3.550975011	0.812423285	NA	NA
ASV_16s_16427	0.175078687	0.692735837	3.550975011	0.812423285	NA	NA
ASV_16s_16428	0.175078687	0.692735837	3.550975011	0.812423285	NA	NA
ASV_16s_16429	0.175078687	0.692735837	3.550975011	0.812423285	NA	NA

ASV_16s_16430	0.175078687	0.692735837	3.550975011	0.812423285	NA	NA
ASV_16s_16431	0.203768546	0.692935833	3.550915868	0.88542284	0.99647135	NA
ASV_16s_16432	0.219468501	0.69302406	3.550889998	0.921896982	0.995992015	NA
ASV_16s_16433	0.146866377	0.293475527	3.551785878	0.566624114	NA	NA
ASV_16s_16434	0.146866377	0.293475527	3.551785878	0.566624114	NA	NA
ASV_16s_16435	0.146866377	0.293475527	3.551785878	0.566624114	NA	NA
ASV_16s_16436	0.146866377	0.293475527	3.551785878	0.566624114	NA	NA
ASV_16s_16437	0.146866377	0.293475527	3.551785878	0.566624114	NA	NA
ASV_16s_16438	0.146866377	0.293475527	3.551785878	0.566624114	NA	NA
ASV_16s_16439	0.146866377	0.293475527	3.551785878	0.566624114	NA	NA
ASV_16s_16440	0.146866377	0.293475527	3.551785878	0.566624114	NA	NA
ASV_16s_16441	0.146866377	0.293475527	3.551785878	0.566624114	NA	NA
ASV_16s_16442	0.146866377	0.293475527	3.551785878	0.566624114	NA	NA
ASV_16s_16443	0.146866377	0.293475527	3.551785878	0.566624114	NA	NA
ASV_16s_16444	0.146866377	0.293475527	3.551785878	0.566624114	NA	NA
ASV_16s_16445	0.155437916	0.093706983	3.551448007	0.754558826	NA	NA
ASV_16s_16446	0.146917713	0.015216264	3.551381768	0.829670915	0.997129229	NA
ASV_16s_16447	0.146923417	-0.008726755	3.551344746	0.849074719	0.99691028	NA
ASV_16s_16448	0.149714484	-0.045161127	3.551268614	0.880218713	0.996536535	NA
ASV_16s_16449	0.146929121	-0.031934529	3.551309696	0.866698766	0.996702202	NA
ASV_16s_16450	0.149714484	-0.045161127	3.551268614	0.880218713	0.996536535	NA
ASV_16s_16451	0.149973403	-0.067980671	3.551236177	0.896763948	0.996326543	NA
ASV_16s_16452	0.14694053	-0.076039758	3.551250003	0.8964661	0.996330394	NA
ASV_16s_16453	0.15049124	-0.11141325	3.551180203	0.92411446	0.995961588	NA
ASV_16s_16454	0.15746724	-0.172491723	3.551053882	0.96488956	0.995375207	NA
ASV_16s_16455	0.146957642	-0.137925365	3.55118146	0.922325209	0.99598615	NA
ASV_16s_16456	0.151267997	-0.172317804	3.551113615	0.94156167	0.995716959	NA
ASV_16s_16457	0.151267997	-0.172317804	3.551113615	0.94156167	0.995716959	NA
ASV_16s_16458	0.177964417	-0.242431972	3.550833111	1.049907706	0.993982784	NA
ASV_16s_16459	0.167032835	-0.216751093	3.55093894	1.004279151	0.994759212	NA
ASV_16s_16460	0.151526916	-0.191972877	3.551096047	0.942822629	0.995698917	NA
ASV_16s_16461	0.148499747	-0.22047964	3.551105293	0.92905047	0.995893322	NA
ASV_16s_16462	0.152044753	-0.230734568	3.551067783	0.94060584	0.995730602	NA
ASV_16s_16463	0.16334855	-0.259276553	3.550950809	0.978591739	0.995166528	NA
ASV_16s_16464	0.15027225	-0.225743459	3.551086769	0.934805319	0.995812795	NA
ASV_16s_16465	0.146986162	-0.234598472	3.551109216	0.920587859	0.996009908	NA
ASV_16s_16466	0.151620704	-0.248083906	3.551063854	0.934742882	0.995813674	NA
ASV_16s_16467	0.14901188	-0.240756196	3.551089974	0.926755146	0.995925159	NA
ASV_16s_16468	0.15282151	-0.28862291	3.551035856	0.923432476	0.995970961	NA
ASV_16s_16469	0.149276503	-0.279048331	3.551068811	0.909483611	0.996159604	NA
ASV_16s_16470	0.147003274	-0.291539364	3.551080831	0.888121396	0.996437237	NA
ASV_16s_16471	0.150295066	-0.300674809	3.551052123	0.900264407	0.996281081	NA
ASV_16s_16472	0.150047556	-0.318846688	3.551048053	0.883994654	0.996489317	NA
ASV_16s_16473	0.052502961	-0.202397486	3.551795355	0.295826665	NA	NA
ASV_16s_16474	0.192490058	-0.62275715	3.550811278	0.677087028	0.998505662	NA
ASV_16s_16475	0.203506344	-0.636041264	3.550748426	0.695997385	0.998366302	NA
ASV_16s_16476	0.191562767	-0.621404365	3.550816094	0.675423278	0.998517528	NA
ASV_16s_16477	0.203854601	-0.636374217	3.550746264	0.696569336	0.998361958	NA
ASV_16s_16478	0.195970536	-0.627744771	3.550793391	0.683264061	0.998461049	NA
ASV_16s_16479	0.206640656	-0.639018552	3.5507292	0.701119604	NA	NA
ASV_16s_16480	0.206640656	-0.639018552	3.5507292	0.701119604	NA	NA
ASV_16s_16481	0.193885176	-0.624778629	3.55080411	0.679572851	0.998487813	NA
ASV_16s_16482	0.183680792	-0.609496632	3.550858311	0.660942299	NA	NA
ASV_16s_16483	0.183680792	-0.609496632	3.550858311	0.660942299	NA	NA
ASV_16s_16484	0.183680792	-0.609496632	3.550858311	0.660942299	NA	NA
ASV_16s_16485	0.162520195	-0.572862216	3.550979061	0.618974337	0.99888383	NA
ASV_16s_16486	0.156187455	-0.560478673	3.551017886	0.605551794	NA	NA
ASV_16s_16487	0.152895663	-0.553837189	3.551039111	0.598393723	0.999000546	NA
ASV_16s_16488	0.152389233	-0.552802182	3.551042442	0.597283535	0.999006598	NA
ASV_16s_16489	0.147071723	-0.541751931	3.551078462	0.585463134	NA	NA
ASV_16s_16490	0.147071723	-0.541751931	3.551078462	0.585463134	NA	NA
ASV_16s_16491	0.147071723	-0.541751931	3.551078462	0.585463134	NA	NA
ASV_16s_16492	0.147071723	-0.541751931	3.551078462	0.585463134	NA	NA
ASV_16s_16493	0.147071723	-0.541751931	3.551078462	0.585463134	NA	NA
ASV_16s_16494	0.147071723	-0.541751931	3.551078462	0.585463134	NA	NA
ASV_16s_16495	0.147071723	-0.541751931	3.551078462	0.585463134	NA	NA
ASV_16s_16496	0.147071723	-0.541751931	3.551078462	0.585463134	NA	NA

ASV_16s_16497	0.147071723	-0.541751931	3.551078462	0.585463134	NA	NA
ASV_16s_16498	0.147071723	-0.541751931	3.551078462	0.585463134	NA	NA
ASV_16s_16499	0.147071723	-0.541751931	3.551078462	0.585463134	NA	NA
ASV_16s_16500	0.147071723	-0.541751931	3.551078462	0.585463134	NA	NA
ASV_16s_16501	0.176992254	-0.598794175	3.550896403	0.648077208	0.99870366	NA
ASV_16s_16502	0.041563382	-0.156632039	3.551093514	0.187215846	NA	NA
ASV_16s_16503	0.173977022	0.10335	3.551209139	0.967781604	0.995331655	NA
ASV_16s_16504	0.161903043	-0.325077988	3.551087042	0.522555604	0.999359629	NA
ASV_16s_16505	0.156020278	0.166495131	3.551879103	0.536208508	0.999302803	NA
ASV_16s_16506	0.201734627	-0.652120794	3.550681558	0.771478077	NA	NA
ASV_16s_16507	0.151923766	0.660353244	3.551729667	0.645211559	NA	NA
ASV_16s_16508	0.165602777	0.234710354	3.551032742	0.696926339	0.998359242	NA
ASV_16s_16509	0.194952046	-0.622755112	3.550654357	0.7981411	0.997462878	NA
ASV_16s_16510	0.186678377	0.698445526	3.551519916	0.876993249	NA	NA
ASV_16s_16511	0.194542714	-0.900408716	3.550613125	0.514737361	NA	NA
ASV_16s_16512	0.180410067	-0.567506215	3.550732436	0.793835706	NA	NA
ASV_16s_16513	0.205272658	-0.613018813	3.550524017	0.878031406	0.996563696	NA
ASV_16s_16514	0.195965564	-0.576607597	3.550588402	0.86779978	0.996688909	NA
ASV_16s_16515	0.15242248	0.315628822	3.552007176	0.363868353	0.999808208	NA
ASV_16s_16516	0.194730678	-0.53631706	3.550597692	0.891980166	0.996388086	NA
ASV_16s_16517	0.190668929	-0.502731846	3.550637302	0.89004396	0.996412803	NA
ASV_16s_16518	0.190694313	-0.486709189	3.550641541	0.896481627	0.996330194	NA
ASV_16s_16519	0.195309998	-0.507467943	3.550606948	0.909792202	0.996155494	NA
ASV_16s_16520	0.192671135	0.307158027	3.551422356	0.566516017	0.999164648	NA
ASV_16s_16521	0.207721342	-0.529289137	3.550521365	0.95862232	0.995468687	NA
ASV_16s_16522	0.146918348	-0.281406567	3.551022986	0.702546935	NA	NA
ASV_16s_16523	0.162975595	-0.560596951	3.550463075	0.658110311	NA	NA
ASV_16s_16524	0.168822736	-0.344928257	3.551608187	0.646393219	NA	NA
ASV_16s_16525	0.194203141	-0.109013268	3.550919787	0.932427994	NA	NA
ASV_16s_16526	0.154933246	-0.480177895	3.551172717	0.557773343	0.999206241	NA
ASV_16s_16527	0.193417478	0.309541733	3.551192749	0.645524592	NA	NA
ASV_16s_16528	0.189282112	-0.304845887	3.55091569	0.813979554	0.997298656	NA
ASV_16s_16529	0.181503211	0.664554391	3.550362096	0.858869554	NA	NA
ASV_16s_16530	0.202897111	0.680967452	3.551002815	0.889681514	0.996417417	NA
ASV_16s_16531	0.198300361	0.680929498	3.551015343	0.878997746	NA	NA
ASV_16s_16532	0.159488903	0.351744012	3.551569364	0.630039586	NA	NA
ASV_16s_16533	0.138614574	0.821673791	3.551457672	0.316815295	0.999879708	NA
ASV_16s_16534	0.116266302	-0.031587867	3.551788732	0.53028448	NA	NA
ASV_16s_16535	0.183209067	-0.859123265	3.550924341	0.456209527	NA	NA
ASV_16s_16536	0.190897476	0.42363052	3.551358476	0.516198289	0.999384982	NA
ASV_16s_16537	0.194081813	0.604248575	3.551818844	0.228597242	0.999960283	NA
ASV_16s_16538	0.183755361	-0.257787241	3.550532001	0.436069108	0.999648541	NA
ASV_16s_16539	0.242937381	0.246884399	3.550442042	1.273878431	0.989125222	NA
ASV_16s_16540	0.228127057	-0.333859314	3.551433917	0.552878015	0.999228908	NA
ASV_16s_16541	0.114042079	0.929675985	3.551589588	0.219914225	0.9999652	NA
ASV_16s_16542	0.170531569	-0.069250474	3.550951171	0.879431588	0.996546325	NA
ASV_16s_16543	0.188824668	1.439040477	3.550908959	0.391250977	NA	NA
ASV_16s_16544	0.162822802	1.148733592	3.551190185	0.566876821	NA	NA
ASV_16s_16545	0.163922668	0.952328338	3.551137299	0.836612535	0.997052109	NA
ASV_16s_16546	0.183212361	1.185995981	3.551082745	0.607213277	NA	NA
ASV_16s_16547	0.178417003	1.5153017	3.550828394	0.340891908	0.999845995	NA
ASV_16s_16548	0.159367609	0.353604374	3.551755164	0.594623275	NA	NA
ASV_16s_16549	0.159367609	0.353604374	3.551755164	0.594623275	NA	NA
ASV_16s_16550	0.167609806	0.198523175	3.551778883	0.525608668	NA	NA
ASV_16s_16551	0.145077337	0.267915674	3.55169875	0.320565183	NA	NA
ASV_16s_16552	0.19407372	-0.053836811	3.551606621	0.595023247	0.999018842	NA
ASV_16s_16553	0.223062702	-0.10236507	3.551517447	0.690039144	0.998411106	NA
ASV_16s_16554	0.194086244	1.175899328	3.551049808	0.577751197	NA	NA
ASV_16s_16555	0.103772329	0.907337669	3.551696686	0.318233418	NA	NA
ASV_16s_16556	0.123500378	0.341250503	3.55190335	0.511655624	NA	NA
ASV_16s_16557	0.123500378	0.341250503	3.55190335	0.511655624	NA	NA
ASV_16s_16558	0.180420547	0.34683313	3.552002404	0.367525524	0.999801657	NA
ASV_16s_16559	0.205553484	0.350602052	3.551925992	0.464813109	0.999565395	NA
ASV_16s_16560	0.26269806	-0.187970986	3.550519186	1.007753902	0.994702484	NA
ASV_16s_16561	0.249583111	-0.080018597	3.550898373	0.798670454	0.997457498	NA
ASV_16s_16562	0.235722518	0.38396952	3.551299272	0.725987688	NA	NA
ASV_16s_16563	0.204356201	-0.857903084	3.550755005	0.475676409	NA	NA

ASV_16s_16564	0.207867002	-0.791683976	3.550619677	0.602516275	0.998977858	NA
ASV_16s_16565	0.177553166	-0.530024915	3.550842834	0.633255073	NA	NA
ASV_16s_16566	0.177553166	-0.530024915	3.550842834	0.633255073	NA	NA
ASV_16s_16567	0.123341212	-0.4482592	3.551341797	0.517508326	NA	NA
ASV_16s_16568	0.170368224	-0.465220862	3.55131957	0.553417088	0.999226434	NA
ASV_16s_16569	0.17311277	-0.526444329	3.550886182	0.625119147	0.998847287	NA
ASV_16s_16570	0.161445817	-0.514312526	3.55100074	0.602672184	NA	NA
ASV_16s_16571	0.196107739	-0.253631472	3.551234512	0.615853215	NA	NA
ASV_16s_16572	0.18286897	1.213704012	3.550862668	0.546920033	NA	NA
ASV_16s_16573	0.17110434	1.194807184	3.550915637	0.524332661	0.999352417	NA
ASV_16s_16574	0.189550581	0.861197839	3.550685181	0.955054356	0.995521356	NA
ASV_16s_16575	0.206817106	0.396020747	3.551094432	0.615218821	NA	NA
ASV_16s_16576	0.206817106	0.396020747	3.551094432	0.615218821	NA	NA
ASV_16s_16577	0.17034683	0.378773324	3.551334144	0.5516463	NA	NA
ASV_16s_16578	0.17034683	0.378773324	3.551334144	0.5516463	NA	NA
ASV_16s_16579	0.140619297	0.365577027	3.551504658	0.489648629	NA	NA
ASV_16s_16580	0.152145095	0.37087003	3.551434596	0.514755681	NA	NA
ASV_16s_16581	0.152145095	0.37087003	3.551434596	0.514755681	NA	NA
ASV_16s_16582	0.164933583	0.090691075	3.550784363	0.82334318	0.997198366	NA
ASV_16s_16583	0.293685225	0.418774903	3.550632441	0.731480841	NA	NA
ASV_16s_16584	0.052975625	0.34164366	3.551855043	0.233790733	NA	NA
ASV_16s_16585	0.052975625	0.34164366	3.551855043	0.233790733	NA	NA
ASV_16s_16586	0.242877842	-0.047373638	3.549892049	1.0797016	0.99343838	NA
ASV_16s_16587	0.103815272	-0.420213037	3.551218605	0.401032989	NA	NA
ASV_16s_16588	0.103815272	-0.420213037	3.551218605	0.401032989	NA	NA
ASV_16s_16589	0.095144463	-0.397091174	3.551286028	0.376855965	NA	NA
ASV_16s_16590	0.186613862	1.219473743	3.550846991	0.553881408	NA	NA
ASV_16s_16591	0.17034683	-0.078553994	3.550835327	0.858283527	NA	NA
ASV_16s_16592	0.167655729	-0.078536717	3.550840454	0.850626266	0.996892316	NA
ASV_16s_16593	0.14525668	-0.078368383	3.550890456	0.782885761	NA	NA
ASV_16s_16594	0.15353735	-0.078436264	3.550870283	0.808785324	0.997353259	NA
ASV_16s_16595	0.163888187	-0.078511588	3.550847913	0.839738534	NA	NA
ASV_16s_16596	0.101560431	0.220148348	3.55086154	0.464846681	0.99956529	NA
ASV_16s_16597	0.101560431	0.220148348	3.55086154	0.464846681	0.99956529	NA
ASV_16s_16598	0.102974253	0.220145392	3.550857644	0.47053906	0.999547365	NA
ASV_16s_16599	0.103327708	0.220144548	3.550856685	0.471956349	0.999542825	NA
ASV_16s_16600	0.108138576	0.220135254	3.55084427	0.490980325	NA	NA
ASV_16s_16601	0.108138576	0.220135254	3.55084427	0.490980325	NA	NA
ASV_16s_16602	0.108090588	0.220135224	3.550844387	0.490792862	0.999479501	NA
ASV_16s_16603	0.185683133	0.310943695	3.550940314	0.880584835	0.996531975	NA
ASV_16s_16604	0.16093102	0.310947312	3.550994197	0.811409074	NA	NA
ASV_16s_16605	0.171208236	1.276619765	3.551179167	0.503372019	0.999434041	NA
ASV_16s_16606	0.167140571	1.117573395	3.551235603	0.566576117	0.999164357	NA
ASV_16s_16607	0.263349224	0.138003124	3.550022027	1.279707354	0.988974403	NA
ASV_16s_16608	0.157435426	-0.067255199	3.550852776	0.953673303	0.995541637	NA
ASV_16s_16609	0.1512029	-0.067848387	3.550882719	0.940189284	NA	NA
ASV_16s_16610	0.237276318	0.337389782	3.551225385	0.674952595	0.998520874	NA
ASV_16s_16611	0.163730246	-0.593786775	3.550455345	0.651088012	NA	NA
ASV_16s_16612	0.145880389	0.682176089	3.551479055	0.656710106	0.998646697	NA
ASV_16s_16613	0.28249634	1.405228791	3.550806888	0.70185347	0.998321455	NA
ASV_16s_16614	0.15417601	-0.067736295	3.550889438	0.978972156	0.99516065	NA
ASV_16s_16615	0.168453819	0.477820479	3.551246812	0.78307777	NA	NA
ASV_16s_16616	0.165713933	-0.065260019	3.55080593	1.005634148	NA	NA
ASV_16s_16617	0.210215757	0.325620529	3.551667296	0.564730848	NA	NA
ASV_16s_16618	0.230096063	0.777889704	3.551137613	0.964573803	NA	NA
ASV_16s_16619	0.236488025	-0.52687321	3.55116226	0.754719654	0.997879067	NA
ASV_16s_16620	0.163271528	-0.027691079	3.55105883	0.927519581	NA	NA
ASV_16s_16621	0.205365405	0.328448112	3.55114409	0.636154529	0.998779659	NA
ASV_16s_16622	0.19302785	0.550681522	3.550987048	0.870345115	0.996658042	NA
ASV_16s_16623	0.280489224	0.249964426	3.550773262	1.177207779	0.99144225	NA
ASV_16s_16624	0.122459839	0.282549596	3.55169134	0.516447751	NA	NA
ASV_16s_16625	0.043937899	0.295577496	3.551920976	0.246217851	0.999948845	NA
ASV_16s_16626	0.072720769	0.287457698	3.551838411	0.363325991	NA	NA
ASV_16s_16627	0.205111859	0.279537491	3.551531208	0.697116614	NA	NA
ASV_16s_16628	0.123761433	-0.401706179	3.551460134	0.517453936	NA	NA
ASV_16s_16629	0.299020213	-0.161689892	3.550341256	1.251124954	NA	NA
ASV_16s_16630	0.272653057	-0.092959846	3.550077895	1.202382254	0.990871943	NA

ASV_16s_16631	0.191222585	-0.572819019	3.550530882	0.641337205	NA	NA
ASV_16s_16632	0.213927259	1.254548651	3.550732682	0.600839605	NA	NA
ASV_16s_16633	0.186084207	1.009653583	3.550716355	0.829603264	NA	NA
ASV_16s_16634	0.121902724	-0.078127857	3.550962015	0.703122013	NA	NA
ASV_16s_16635	0.207236745	-0.10882045	3.550442481	1.166983201	0.991667339	NA
ASV_16s_16636	0.265976092	0.183505837	3.550834515	1.69405817	0.974821967	NA
ASV_16s_16637	0.283078302	0.34084083	3.550068981	1.429590236	0.984647656	NA
ASV_16s_16638	0.251339907	0.926569828	3.550825871	1.053022233	0.993927273	NA
ASV_16s_16639	0.23481782	0.348112564	3.550265943	1.277998558	0.98901875	NA
ASV_16s_16640	0.20325863	-0.060362154	3.550560593	1.203260526	NA	NA
ASV_16s_16641	0.237455464	0.371656982	3.55051229	1.165413018	0.991701574	NA
ASV_16s_16642	0.160833558	-0.028471217	3.55102339	0.889846195	NA	NA
ASV_16s_16643	0.160833558	-0.028471217	3.55102339	0.889846195	NA	NA
ASV_16s_16644	0.160833558	-0.028471217	3.55102339	0.889846195	NA	NA
ASV_16s_16645	0.160833558	-0.028471217	3.55102339	0.889846195	NA	NA
ASV_16s_16646	0.160833558	-0.028471217	3.55102339	0.889846195	NA	NA
ASV_16s_16647	0.183772809	-0.073538462	3.550841421	0.919197294	NA	NA
ASV_16s_16648	0.134220237	-0.073115587	3.550961633	0.770636615	NA	NA
ASV_16s_16649	0.143994439	-0.073220563	3.550931424	0.802976108	0.997413463	NA
ASV_16s_16650	0.131808523	-0.073086917	3.550969773	0.762362961	NA	NA
ASV_16s_16651	0.131808523	-0.073086917	3.550969773	0.762362961	NA	NA
ASV_16s_16652	0.190833967	0.143594298	3.550856936	1.457108548	0.983757524	NA
ASV_16s_16653	0.170783274	0.329649485	3.550679995	1.026854034	0.994383633	NA
ASV_16s_16654	0.178578224	0.34271875	3.550639713	1.050894156	0.993965237	NA
ASV_16s_16655	0.22478448	0.376291879	3.550685059	1.079591592	NA	NA
ASV_16s_16656	0.183466454	0.360305843	3.551146669	0.936422757	NA	NA
ASV_16s_16657	0.25299547	0.337724313	3.550966285	0.840196442	NA	NA
ASV_16s_16658	0.163095339	0.36028495	3.55121509	0.879469338	NA	NA
ASV_16s_16659	0.163095339	0.36028495	3.55121509	0.879469338	NA	NA
ASV_16s_16660	0.163095339	0.36028495	3.55121509	0.879469338	NA	NA
ASV_16s_16661	0.163095339	0.36028495	3.55121509	0.879469338	NA	NA
ASV_16s_16662	0.163095339	0.36028495	3.55121509	0.879469338	NA	NA
ASV_16s_16663	0.163095339	0.36028495	3.55121509	0.879469338	NA	NA
ASV_16s_16664	0.163095339	0.36028495	3.55121509	0.879469338	NA	NA
ASV_16s_16665	0.190834289	0.315939747	3.550963051	0.916776773	0.996061703	NA
ASV_16s_16666	0.182665873	0.31593977	3.550979744	0.895389408	NA	NA
ASV_16s_16667	0.175479765	0.315939793	3.550995716	0.875860955	0.996590511	NA
ASV_16s_16668	0.175137569	0.315939794	3.550996509	0.874914425	0.996602162	NA
ASV_16s_16669	0.240168469	0.31593953	3.550886232	1.030839638	0.994315591	NA
ASV_16s_16670	0.189154564	0.315939613	3.550966391	0.911036795	0.996138888	NA
ASV_16s_16671	0.226967533	0.315939665	3.55090355	1.002419283	0.994789416	NA
ASV_16s_16672	0.208093966	0.315939704	3.550932088	0.958365416	0.995472492	NA
ASV_16s_16673	0.17034683	0.31593981	3.551007943	0.861493178	NA	NA
ASV_16s_16674	0.17034683	0.31593981	3.551007943	0.861493178	NA	NA
ASV_16s_16675	0.17034683	0.31593981	3.551007943	0.861493178	NA	NA
ASV_16s_16676	0.190209041	0.432380976	3.550927403	1.285746291	0.988816796	NA
ASV_16s_16677	0.23287321	0.935465342	3.551165189	0.799525445	NA	NA
ASV_16s_16678	0.360568677	0.734578036	3.550874438	1.212157941	NA	NA
ASV_16s_16679	0.247694723	1.080450223	3.55073137	0.771319984	NA	NA
ASV_16s_16680	0.224761885	1.114249881	3.55082347	0.723850404	0.998145694	NA
ASV_16s_16681	0.266302376	0.73421452	3.550979618	1.059037838	NA	NA
ASV_16s_16682	0.266302376	0.73421452	3.550979618	1.059037838	NA	NA
ASV_16s_16683	0.22933353	1.082886656	3.550792241	0.73883758	0.998019207	NA
ASV_16s_16684	0.219196056	1.067758775	3.550839695	0.724068161	0.998143896	NA
ASV_16s_16685	0.266302376	0.73421452	3.550979618	1.059037838	NA	NA
ASV_16s_16686	0.23331129	0.734018849	3.551036357	0.992863725	0.994942834	NA
ASV_16s_16687	0.23331129	0.734018849	3.551036357	0.992863725	0.994942834	NA
ASV_16s_16688	0.225347924	0.733964162	3.551052516	0.975626318	NA	NA
ASV_16s_16689	0.225347924	0.733964162	3.551052516	0.975626318	NA	NA
ASV_16s_16690	0.225347924	0.733964162	3.551052516	0.975626318	NA	NA
ASV_16s_16691	0.225347924	0.733964162	3.551052516	0.975626318	NA	NA
ASV_16s_16692	0.225347924	0.733964162	3.551052516	0.975626318	NA	NA
ASV_16s_16693	0.225347924	0.733964162	3.551052516	0.975626318	NA	NA
ASV_16s_16694	0.225347924	0.733964162	3.551052516	0.975626318	NA	NA
ASV_16s_16695	0.111627371	0.719362204	3.551481941	0.651919529	NA	NA
ASV_16s_16696	0.20950056	0.692969844	3.55090597	0.899035887	NA	NA
ASV_16s_16697	0.211977419	0.692983853	3.550901861	0.904807792	0.996221532	NA

ASV_16s_16698	0.215445022	0.693002933	3.550896265	0.912791496	NA	NA
ASV_16s_16699	0.226895238	0.693061824	3.550879	0.938379301	0.995762274	NA
ASV_16s_16700	0.194148654	0.692875089	3.550933777	0.861869775	0.996760097	NA
ASV_16s_16701	0.190390471	0.692849601	3.55094126	0.852419071	0.996871474	NA
ASV_16s_16702	0.185379561	0.692814031	3.550951706	0.839593745	0.997018577	NA
ASV_16s_16703	0.177863196	0.692756973	3.550968469	0.819825758	0.997236321	NA
ASV_16s_16704	0.17034683	0.692696258	3.550986696	0.799414613	NA	NA
ASV_16s_16705	0.17034683	0.692696258	3.550986696	0.799414613	NA	NA
ASV_16s_16706	0.17034683	0.692696258	3.550986696	0.799414613	NA	NA
ASV_16s_16707	0.17034683	0.692696258	3.550986696	0.799414613	NA	NA
ASV_16s_16708	0.205858682	0.692948539	3.55091222	0.890339985	0.996409031	NA
ASV_16s_16709	0.142786755	0.294119952	3.551798487	0.557246589	NA	NA
ASV_16s_16710	0.142786755	0.294119952	3.551798487	0.557246589	NA	NA
ASV_16s_16711	0.142786755	0.294119952	3.551798487	0.557246589	NA	NA
ASV_16s_16712	0.142786755	0.294119952	3.551798487	0.557246589	NA	NA
ASV_16s_16713	0.142786755	0.294119952	3.551798487	0.557246589	NA	NA
ASV_16s_16714	0.142820979	0.091922127	3.551523985	0.74742851	NA	NA
ASV_16s_16715	0.145375944	-0.024187527	3.551334126	0.847413017	0.996929444	NA
ASV_16s_16716	0.142843796	-0.011331025	3.551375233	0.834824379	0.997072104	NA
ASV_16s_16717	0.1428495	-0.034866858	3.551339731	0.85189786	0.996877543	NA
ASV_16s_16718	0.153541108	-0.10277156	3.551163371	0.917767584	0.99604828	NA
ASV_16s_16719	0.1461527	-0.093750372	3.551235145	0.895201021	0.996346724	NA
ASV_16s_16720	0.142860908	-0.079633196	3.551279397	0.880442055	0.996533753	NA
ASV_16s_16721	0.142866612	-0.101077581	3.551253873	0.890829141	0.996402793	NA
ASV_16s_16722	0.146411619	-0.115523787	3.55120873	0.906113989	0.996204298	NA
ASV_16s_16723	0.146670538	-0.136759709	3.551185056	0.912114673	0.99612447	NA
ASV_16s_16724	0.154420333	-0.163156317	3.551090871	0.945049766	0.995666932	NA
ASV_16s_16725	0.142872316	-0.122020974	3.551231056	0.896390248	0.996331375	NA
ASV_16s_16726	0.146929457	-0.157543043	3.551163888	0.916179548	0.99606978	NA
ASV_16s_16727	0.146929457	-0.157543043	3.551163888	0.916179548	0.99606978	NA
ASV_16s_16728	0.146929457	-0.157543043	3.551163888	0.916179548	0.99606978	NA
ASV_16s_16729	0.146929457	-0.157543043	3.551163888	0.916179548	0.99606978	NA
ASV_16s_16730	0.145669087	-0.172670864	3.551161446	0.912448738	0.996119994	NA
ASV_16s_16731	0.157521435	-0.208892351	3.551027519	0.95853602	0.995469965	NA
ASV_16s_16732	0.144408717	-0.187794911	3.551159728	0.907686607	0.996183481	NA
ASV_16s_16733	0.145168361	-0.19039911	3.55115171	0.91041435	0.996147199	NA
ASV_16s_16734	0.142889428	-0.182519381	3.551176067	0.902268096	0.996254895	NA
ASV_16s_16735	0.147706213	-0.217908124	3.551113237	0.91757574	0.996050881	NA
ASV_16s_16736	0.14467334	-0.227260925	3.551131649	0.904043775	0.996231588	NA
ASV_16s_16737	0.144932259	-0.247083532	3.55111888	0.899918022	0.996285596	NA
ASV_16s_16738	0.148224051	-0.257058481	3.551088175	0.909959919	0.996153259	NA
ASV_16s_16739	0.148224051	-0.257058481	3.551088175	0.909959919	0.996153259	NA
ASV_16s_16740	0.148224051	-0.257058481	3.551088175	0.909959919	0.996153259	NA
ASV_16s_16741	0.144431533	-0.264797373	3.551113405	0.889835495	0.996415457	NA
ASV_16s_16742	0.163554698	-0.313299079	3.550932913	0.948309878	0.995619835	NA
ASV_16s_16743	0.148741888	-0.296345611	3.551065676	0.89444686	0.996356436	NA
ASV_16s_16744	0.051044546	-0.19440236	3.551801766	0.289701277	NA	NA
ASV_16s_16745	0.197418069	-0.629591238	3.550785451	0.685731288	0.998442985	NA
ASV_16s_16746	0.186460523	-0.613772632	3.550843345	0.666061525	0.998583131	NA
ASV_16s_16747	0.197940454	-0.630243505	3.550782519	0.686633444	0.998436344	NA
ASV_16s_16748	0.200900637	-0.633506996	3.550764967	0.691628685	NA	NA
ASV_16s_16749	0.200900637	-0.633506996	3.550764967	0.691628685	NA	NA
ASV_16s_16750	0.178578548	-0.60137381	3.550887138	0.651183765	NA	NA
ASV_16s_16751	0.178578548	-0.60137381	3.550887138	0.651183765	NA	NA
ASV_16s_16752	0.178578548	-0.60137381	3.550887138	0.651183765	NA	NA
ASV_16s_16753	0.163473607	-0.574633067	3.550973439	0.620919734	0.998872346	NA
ASV_16s_16754	0.157319776	-0.562674005	3.551010932	0.607893352	0.998947749	NA
ASV_16s_16755	0.151848915	-0.551659492	3.551046131	0.596037653	NA	NA
ASV_16s_16756	0.151848915	-0.551659492	3.551046131	0.596037653	NA	NA
ASV_16s_16757	0.151848915	-0.551659492	3.551046131	0.596037653	NA	NA
ASV_16s_16758	0.151848915	-0.551659492	3.551046131	0.596037653	NA	NA
ASV_16s_16759	0.151848915	-0.551659492	3.551046131	0.596037653	NA	NA
ASV_16s_16760	0.161268745	-0.570408064	3.550986678	0.615472813	NA	NA
ASV_16s_16761	0.146024975	-0.539492979	3.551085924	0.583036226	0.999082081	NA
ASV_16s_16762	0.144758901	-0.536787183	3.551094904	0.580156908	NA	NA
ASV_16s_16763	0.142986397	-0.532964675	3.551107679	0.576092538	NA	NA
ASV_16s_16764	0.142986397	-0.532964675	3.551107679	0.576092538	NA	NA

ASV_16s_16765	0.142986397	-0.532964675	3.551107679	0.576092538	NA	NA
ASV_16s_16766	0.142986397	-0.532964675	3.551107679	0.576092538	NA	NA
ASV_16s_16767	0.142986397	-0.532964675	3.551107679	0.576092538	NA	NA
ASV_16s_16768	0.142986397	-0.532964675	3.551107679	0.576092538	NA	NA
ASV_16s_16769	0.142986397	-0.532964675	3.551107679	0.576092538	NA	NA
ASV_16s_16770	0.142986397	-0.532964675	3.551107679	0.576092538	NA	NA
ASV_16s_16771	0.049378536	0.149133395	3.551090049	0.292312546	0.999908379	NA
ASV_16s_16772	0.046680526	-0.162079779	3.55104877	0.218463636	0.999965978	NA
ASV_16s_16773	0.048483538	-0.163858708	3.551035115	0.229210223	NA	NA
ASV_16s_16774	0.092553835	0.220169316	3.550889159	0.427333001	NA	NA
ASV_16s_16775	0.048035272	0.223139442	3.551152349	0.20457948	NA	NA
ASV_16s_16777	0.101175792	0.595764452	3.550821258	0.405745674	NA	NA
ASV_16s_16778	0.154284978	0.208285343	3.551361103	0.854989539	0.996841433	NA
ASV_16s_16779	0.161037963	-0.536938033	3.551083768	0.665864961	NA	NA
ASV_16s_16780	0.166550949	-0.036041507	3.551591023	0.763431811	NA	NA
ASV_16s_16781	0.185265749	-0.615454785	3.5507856	0.734825318	0.998053612	NA
ASV_16s_16782	0.151773539	-0.076006121	3.551083698	0.509723133	NA	NA
ASV_16s_16783	0.158900598	0.30531452	3.551959583	0.463512652	NA	NA
ASV_16s_16784	0.216519807	0.002809877	3.551142241	0.751477125	NA	NA
ASV_16s_16785	0.192677541	-0.60468838	3.550639674	0.80931655	0.997347708	NA
ASV_16s_16786	0.138288542	-0.457132901	3.551236353	0.660018114	0.998624434	NA
ASV_16s_16787	0.186400079	-0.559487367	3.550673733	0.828736227	0.997139511	NA
ASV_16s_16788	0.155366294	-0.028646278	3.551748936	0.567150329	0.999161574	NA
ASV_16s_16789	0.180244461	-0.502844582	3.550715504	0.838347526	0.997032625	NA
ASV_16s_16790	0.189164788	-0.516497312	3.550645831	0.873202894	0.996623164	NA
ASV_16s_16791	0.189521424	-0.522858364	3.550641522	0.872334202	0.996633791	NA
ASV_16s_16792	0.188812203	-0.495380656	3.550651094	0.881088604	0.996525694	NA
ASV_16s_16793	0.180466583	-0.461588181	3.550715719	0.856684015	0.996821527	NA
ASV_16s_16794	0.196087515	-0.07534929	3.551597809	0.595326214	0.999017207	NA
ASV_16s_16795	0.189330261	-0.476486316	3.550654426	0.889963294	0.99641383	NA
ASV_16s_16796	0.189495432	-0.484693749	3.551177533	0.558330864	0.999203632	NA
ASV_16s_16797	0.135635904	-0.756631133	3.551254103	0.346935423	0.999836607	NA
ASV_16s_16798	0.186154444	-0.44962784	3.550684791	0.884006591	0.996489167	NA
ASV_16s_16799	0.185699092	-0.425147446	3.550701427	0.886484972	0.996457948	NA
ASV_16s_16800	0.178268505	-0.353578016	3.551700034	0.453232473	0.999600348	NA
ASV_16s_16801	0.191392021	-0.048492656	3.550597682	0.855632638	0.996833888	NA
ASV_16s_16802	0.159008583	0.756598762	3.5513664	0.66004665	0.998624241	NA
ASV_16s_16803	0.188022312	-0.410579931	3.550701808	0.896599732	0.996328667	NA
ASV_16s_16804	0.186926613	0.18316993	3.549853331	0.8586925	NA	NA
ASV_16s_16805	0.186926613	0.18316993	3.549853331	0.8586925	NA	NA
ASV_16s_16806	0.188786236	-0.35066719	3.55075179	0.898991271	NA	NA
ASV_16s_16807	0.213891796	0.196714815	3.55097537	0.973053592	NA	NA
ASV_16s_16808	0.181978511	0.829297315	3.551193933	0.381217054	0.999775756	NA
ASV_16s_16809	0.236374813	0.558705736	3.550683272	1.106090106	0.992930951	NA
ASV_16s_16810	0.197530517	-0.159775093	3.551103974	0.629385941	0.998821445	NA
ASV_16s_16811	0.180254564	-0.165744001	3.550875503	0.865956581	0.996711144	NA
ASV_16s_16812	0.179790432	-0.110732834	3.550932888	0.854190542	0.996850791	NA
ASV_16s_16813	0.168381314	0.68954495	3.551230476	0.567506947	0.999159842	NA
ASV_16s_16814	0.160442728	-0.467098676	3.550850876	0.585652536	NA	NA
ASV_16s_16815	0.166743591	-0.085126821	3.550951612	0.816657735	0.997270216	NA
ASV_16s_16816	0.169449222	-0.453735284	3.551002711	0.771984202	0.997719462	NA
ASV_16s_16817	0.182737945	-0.065306971	3.551275414	0.634038773	0.998792825	NA
ASV_16s_16818	0.187545612	-0.03228802	3.550693622	0.857900015	0.996807191	NA
ASV_16s_16819	0.17286476	-0.032603215	3.551463372	0.678154476	0.998498015	NA
ASV_16s_16820	0.230268179	0.215510043	3.550453459	0.597799489	0.999003789	NA
ASV_16s_16821	0.175451115	0.136228795	3.550495022	0.689827397	NA	NA
ASV_16s_16822	0.117884944	-0.04100867	3.550821615	0.485650867	0.999497344	NA
ASV_16s_16823	0.165278358	-0.317879512	3.551386837	0.378747941	0.99978059	NA
ASV_16s_16824	0.166727791	-0.069586835	3.551017485	0.825937322	0.997170156	NA
ASV_16s_16825	0.173064978	0.111961216	3.551236897	0.635041846	0.998786595	NA
ASV_16s_16826	0.107849448	0.3696696	3.550884562	0.438983198	0.999640656	NA
ASV_16s_16827	0.107317621	1.221491321	3.551533269	0.247996045	0.999947576	NA
ASV_16s_16828	0.123978183	1.298891452	3.551365385	0.26000803	0.999938425	NA
ASV_16s_16829	0.178123129	1.177046253	3.551107817	0.59740216	NA	NA
ASV_16s_16830	0.185010465	1.505940702	3.550798003	0.360650571	0.999813848	NA
ASV_16s_16831	0.163427743	1.149881895	3.551186844	0.56807271	NA	NA
ASV_16s_16832	0.163427743	1.149881895	3.551186844	0.56807271	NA	NA

ASV_16s_16833	0.163427743	1.149881895	3.551186844	0.56807271	NA	NA
ASV_16s_16834	0.154940731	0.351497314	3.551780929	0.58527967	NA	NA
ASV_16s_16835	0.159648648	0.340359943	3.551980848	0.409879409	NA	NA
ASV_16s_16836	0.15886806	0.353326392	3.551758357	0.593528358	0.999026884	NA
ASV_16s_16837	0.233241914	0.633877135	3.550848575	1.067207232	0.993670332	NA
ASV_16s_16838	0.206186924	0.350302894	3.5519176	0.464814524	NA	NA
ASV_16s_16839	0.183034645	0.316680338	3.551877795	0.184319809	0.999980981	NA
ASV_16s_16840	0.149999952	-0.286115251	3.551419377	0.526616867	0.999343067	NA
ASV_16s_16841	0.112529539	0.726045818	3.551138845	0.212233257	0.99996918	NA
ASV_16s_16842	0.209228251	0.426303652	3.550642049	0.860773733	0.996773145	NA
ASV_16s_16843	0.218232876	0.379439634	3.551401869	0.699880318	NA	NA
ASV_16s_16844	0.208998868	-0.550573213	3.550578613	0.685972725	NA	NA
ASV_16s_16845	0.198856875	-0.543748577	3.550889514	0.600922907	NA	NA
ASV_16s_16846	0.212637557	-1.058631994	3.550573291	0.386801733	0.999764554	NA
ASV_16s_16847	0.119915068	-0.43989163	3.551370198	0.508759313	NA	NA
ASV_16s_16848	0.156961211	-0.508919474	3.55104696	0.593618856	NA	NA
ASV_16s_16849	0.134549332	-0.770115395	3.551218318	0.318756993	0.9998772	NA
ASV_16s_16850	0.236592839	1.259007627	3.55084581	0.69650821	NA	NA
ASV_16s_16851	0.182032292	1.212341234	3.550866402	0.545250593	0.999263346	NA
ASV_16s_16852	0.092788875	0.998431845	3.551392755	0.336093675	NA	NA
ASV_16s_16853	0.201072186	0.393530991	3.551130891	0.605864064	NA	NA
ASV_16s_16854	0.182403579	0.384465703	3.551257543	0.573990204	NA	NA
ASV_16s_16855	0.182403579	0.384465703	3.551257543	0.573990204	NA	NA
ASV_16s_16856	0.182403579	0.384465703	3.551257543	0.573990204	NA	NA
ASV_16s_16857	0.165614974	0.376994415	3.551360428	0.542320451	NA	NA
ASV_16s_16858	0.165614974	0.376994415	3.551360428	0.542320451	NA	NA
ASV_16s_16859	0.136713206	0.364007444	3.551527136	0.480703569	NA	NA
ASV_16s_16860	0.136713206	0.364007444	3.551527136	0.480703569	NA	NA
ASV_16s_16861	0.147918842	0.368828403	3.5514603	0.505675055	NA	NA
ASV_16s_16862	0.05150408	0.341536645	3.551861614	0.228201285	NA	NA
ASV_16s_16863	0.207509847	0.396303301	3.551090348	0.616259608	NA	NA
ASV_16s_16864	0.083106974	-0.356927828	3.551374922	0.340471306	NA	NA
ASV_16s_16865	0.083106974	-0.356927828	3.551374922	0.340471306	NA	NA
ASV_16s_16866	0.100931514	-0.413361363	3.551243251	0.393147677	NA	NA
ASV_16s_16867	0.092501561	-0.388748195	3.551304303	0.369101582	NA	NA
ASV_16s_16868	0.182438077	1.212996203	3.550864605	0.546049463	NA	NA
ASV_16s_16869	0.073329405	0.925723885	3.551557496	0.277684979	NA	NA
ASV_16s_16870	0.073329405	0.925723885	3.551557496	0.277684979	NA	NA
ASV_16s_16871	0.181430143	1.211420289	3.550868935	0.544154296	NA	NA
ASV_16s_16872	0.165614974	-0.078523406	3.550844473	0.844679729	NA	NA
ASV_16s_16873	0.164538533	-0.078513644	3.550846621	0.841557755	0.99699635	NA
ASV_16s_16874	0.141221772	-0.078332633	3.550901162	0.769760707	NA	NA
ASV_16s_16875	0.141221772	-0.078332633	3.550901162	0.769760707	NA	NA
ASV_16s_16876	0.141221772	-0.078332633	3.550901162	0.769760707	NA	NA
ASV_16s_16877	0.141221772	-0.078332633	3.550901162	0.769760707	NA	NA
ASV_16s_16878	0.141221772	-0.078332633	3.550901162	0.769760707	NA	NA
ASV_16s_16879	0.141221772	-0.078332633	3.550901162	0.769760707	NA	NA
ASV_16s_16880	0.100329858	0.220150908	3.550865037	0.459798367	0.999580776	NA
ASV_16s_16881	0.105134727	0.220141057	3.550851906	0.479105755	NA	NA
ASV_16s_16882	0.10492478	0.220141473	3.550852454	0.478272156	NA	NA
ASV_16s_16883	0.162222794	0.310947119	3.550991009	0.815141129	0.997286345	NA
ASV_16s_16884	0.110751138	0.595824143	3.550797893	0.441712041	NA	NA
ASV_16s_16885	0.154978768	0.753855203	3.551839969	0.341768404	0.999844657	NA
ASV_16s_16886	0.172728338	0.613516536	3.551013773	0.52510838	0.999349252	NA
ASV_16s_16887	0.204465693	-0.230252088	3.551376168	0.78654965	0.997578808	NA
ASV_16s_16888	0.161359562	1.008487998	3.551032583	0.680975945	0.998477677	NA
ASV_16s_16889	0.194501724	1.459513375	3.550950498	0.333088903	0.999857555	NA
ASV_16s_16890	0.148159029	-0.06729702	3.550901136	0.944018479	0.995681762	NA
ASV_16s_16891	0.180305929	0.065511598	3.55169868	0.529873849	0.999329577	NA
ASV_16s_16892	0.152618693	-0.052856758	3.550985013	0.93838531	NA	NA
ASV_16s_16893	0.193454572	0.052261398	3.551178851	0.812972497	0.9973093	NA
ASV_16s_16894	0.15360973	-0.04035144	3.551070219	0.919404557	NA	NA
ASV_16s_16895	0.178305634	-0.144206756	3.550905458	1.137225126	0.992301196	NA
ASV_16s_16896	0.181926965	-0.416508992	3.550725233	0.704480845	0.998301071	NA
ASV_16s_16897	0.095554694	0.299391975	3.551946495	0.323955707	0.999870307	NA
ASV_16s_16898	0.397100389	1.191377747	3.550425146	0.850036508	NA	NA
ASV_16s_16899	0.375158954	1.182280533	3.550480739	0.83059094	NA	NA

ASV_16s_16900	0.181043135	1.016725676	3.551206689	0.579185998	NA	NA
ASV_16s_16901	0.181043135	1.016725676	3.551206689	0.579185998	NA	NA
ASV_16s_16902	0.119058177	0.282827223	3.551698743	0.50719815	NA	NA
ASV_16s_16903	0.119058177	0.282827223	3.551698743	0.50719815	NA	NA
ASV_16s_16904	0.070700747	0.28769917	3.551846601	0.355986871	NA	NA
ASV_16s_16905	0.199414307	0.279498013	3.551544332	0.686862816	NA	NA
ASV_16s_16906	0.120323615	-0.3938318	3.551476245	0.508520171	NA	NA
ASV_16s_16907	0.322568237	-0.564326193	3.549938576	0.825164647	NA	NA
ASV_16s_16908	0.271616607	-0.181033064	3.549751493	1.104766911	NA	NA
ASV_16s_16909	0.272181673	0.140100622	3.549937227	0.972669617	NA	NA
ASV_16s_16910	0.187574652	-0.536101797	3.550430473	0.582326991	NA	NA
ASV_16s_16911	0.192247458	1.189103559	3.550648437	0.748523728	NA	NA
ASV_16s_16912	0.118516538	-0.078085441	3.550974743	0.690594177	NA	NA
ASV_16s_16913	0.118516538	-0.078085441	3.550974743	0.690594177	NA	NA
ASV_16s_16914	0.118516538	-0.078085441	3.550974743	0.690594177	NA	NA
ASV_16s_16915	0.270784079	-0.029082766	3.550819611	1.146327386	0.992110655	NA
ASV_16s_16916	0.18042122	-0.028633471	3.550969117	0.942287064	0.995706586	NA
ASV_16s_16917	0.156365959	-0.028427077	3.551037711	0.876407861	NA	NA
ASV_16s_16918	0.156365959	-0.028427077	3.551037711	0.876407861	NA	NA
ASV_16s_16919	0.161742157	0.056499589	3.551016267	1.034434496	NA	NA
ASV_16s_16920	0.174569781	0.332127087	3.550648166	1.031712858	NA	NA
ASV_16s_16921	0.178668009	-0.073505677	3.550850762	0.905427874	NA	NA
ASV_16s_16922	0.202920159	0.343047617	3.550480832	1.113923414	0.992775697	NA
ASV_16s_16923	0.196681589	0.294283626	3.550243552	1.072455773	0.993573541	NA
ASV_16s_16924	0.218744819	0.308635935	3.55032475	1.106668349	0.992919563	NA
ASV_16s_16925	0.190343713	0.312304775	3.550579151	1.087527409	0.99329039	NA
ASV_16s_16926	0.128147175	-0.073039984	3.550982739	0.749544466	NA	NA
ASV_16s_16927	0.218187848	0.196975593	3.550805492	1.73933304	0.972851197	NA
ASV_16s_16928	0.217961372	0.337034124	3.550363858	1.186440316	0.991235762	NA
ASV_16s_16929	0.201356738	0.333498697	3.550454519	1.133032124	0.992387986	NA
ASV_16s_16930	0.158999988	-0.073359161	3.550892234	0.849411545	NA	NA
ASV_16s_16931	0.178370164	0.360300986	3.551162367	0.922610785	NA	NA
ASV_16s_16932	0.178370164	0.360300986	3.551162367	0.922610785	NA	NA
ASV_16s_16933	0.178370164	0.360300986	3.551162367	0.922610785	NA	NA
ASV_16s_16934	0.178370164	0.360300986	3.551162367	0.922610785	NA	NA
ASV_16s_16935	0.158564913	0.36027958	3.551232711	0.865956995	NA	NA
ASV_16s_16936	0.158564913	0.36027958	3.551232711	0.865956995	NA	NA
ASV_16s_16937	0.158564913	0.36027958	3.551232711	0.865956995	NA	NA
ASV_16s_16938	0.158564913	0.36027958	3.551232711	0.865956995	NA	NA
ASV_16s_16939	0.158564913	0.36027958	3.551232711	0.865956995	NA	NA
ASV_16s_16940	0.158564913	0.36027958	3.551232711	0.865956995	NA	NA
ASV_16s_16941	0.186102432	0.315939774	3.550972574	0.904392315	0.996227003	NA
ASV_16s_16942	0.177569681	0.31593967	3.550990969	0.881508679	0.996520451	NA
ASV_16s_16943	0.177591821	0.315939801	3.550990913	0.881594273	NA	NA
ASV_16s_16944	0.173143277	0.315939816	3.551001219	0.869289054	0.996670871	NA
ASV_16s_16945	0.170063517	0.315939826	3.551008666	0.86060972	0.996775094	NA
ASV_16s_16946	0.16903693	0.31593983	3.551011208	0.857688047	0.996809693	NA
ASV_16s_16947	0.193925325	0.315939752	3.55095716	0.922757623	0.995980223	NA
ASV_16s_16948	0.165614974	0.315939843	3.551019908	0.847862128	NA	NA
ASV_16s_16949	0.165614974	0.315939843	3.551019908	0.847862128	NA	NA
ASV_16s_16950	0.165614974	0.315939843	3.551019908	0.847862128	NA	NA
ASV_16s_16951	0.165614974	0.315939843	3.551019908	0.847862128	NA	NA
ASV_16s_16952	0.165614974	0.315939843	3.551019908	0.847862128	NA	NA
ASV_16s_16953	0.165614974	0.315939843	3.551019908	0.847862128	NA	NA
ASV_16s_16954	0.165614974	0.315939843	3.551019908	0.847862128	NA	NA
ASV_16s_16955	0.236616105	0.315939559	3.550890753	1.027146413	0.99437866	NA
ASV_16s_16956	0.222992464	0.596143919	3.550673306	0.761690873	NA	NA
ASV_16s_16957	0.222992464	0.596143919	3.550673306	0.761690873	NA	NA
ASV_16s_16958	0.157721273	0.687567781	3.550974801	0.738823088	NA	NA
ASV_16s_16959	0.284934511	0.68815432	3.550787772	1.027646449	NA	NA
ASV_16s_16960	0.23225751	1.123314866	3.551039359	0.757511393	NA	NA
ASV_16s_16961	0.237290238	0.734045389	3.551028715	1.001191491	0.994809293	NA
ASV_16s_16962	0.208854354	0.929082364	3.550925295	0.757479936	0.997854061	NA
ASV_16s_16963	0.21908826	0.733916696	3.551066069	0.961602009	NA	NA
ASV_16s_16964	0.21908826	0.733916696	3.551066069	0.961602009	NA	NA
ASV_16s_16965	0.21908826	0.733916696	3.551066069	0.961602009	NA	NA
ASV_16s_16966	0.21908826	0.733916696	3.551066069	0.961602009	NA	NA

ASV_16s_16967	0.21908826	0.733916696	3.551066069	0.961602009	NA		NA
ASV_16s_16968	0.21908826	0.733916696	3.551066069	0.961602009	NA		NA
ASV_16s_16969	0.21908826	0.733916696	3.551066069	0.961602009	NA		NA
ASV_16s_16970	0.21908826	0.733916696	3.551066069	0.961602009	NA		NA
ASV_16s_16971	0.21908826	0.733916696	3.551066069	0.961602009	NA		NA
ASV_16s_16972	0.21908826	0.733916696	3.551066069	0.961602009	NA		NA
ASV_16s_16973	0.21908826	0.733916696	3.551066069	0.961602009	NA		NA
ASV_16s_16974	0.21908826	0.733916696	3.551066069	0.961602009	NA		NA
ASV_16s_16975	0.10852661	0.716574841	3.551495966	0.640224514	NA		NA
ASV_16s_16976	0.10852661	0.716574841	3.551495966	0.640224514	NA		NA
ASV_16s_16977	0.2036811	0.69293575	3.550916039	0.88515871	NA		NA
ASV_16s_16978	0.2036811	0.69293575	3.550916039	0.88515871	NA		NA
ASV_16s_16979	0.2036811	0.69293575	3.550916039	0.88515871	NA		NA
ASV_16s_16980	0.205497463	0.692946642	3.550912842	0.889502598		0.996419694	NA
ASV_16s_16981	0.206488207	0.692952503	3.550911122	0.891857718		0.996389652	NA
ASV_16s_16982	0.199467213	0.69290971	3.550923683	0.874946567		0.996601767	NA
ASV_16s_16983	0.189852238	0.692845996	3.550942391	0.85031813		0.996895889	NA
ASV_16s_16984	0.209460438	0.692969758	3.550906059	0.898863645	NA		NA
ASV_16s_16985	0.209460438	0.692969758	3.550906059	0.898863645	NA		NA
ASV_16s_16986	0.209460438	0.692969758	3.550906059	0.898863645	NA		NA
ASV_16s_16987	0.209460438	0.692969758	3.550906059	0.898863645	NA		NA
ASV_16s_16988	0.209460438	0.692969758	3.550906059	0.898863645	NA		NA
ASV_16s_16989	0.209460438	0.692969758	3.550906059	0.898863645	NA		NA
ASV_16s_16990	0.185658615	0.69281623	3.550951135	0.840237502		0.997011304	NA
ASV_16s_16991	0.165614974	0.69265443	3.550999045	0.78613375	NA		NA
ASV_16s_16992	0.165614974	0.69265443	3.550999045	0.78613375	NA		NA
ASV_16s_16993	0.165614974	0.69265443	3.550999045	0.78613375	NA		NA
ASV_16s_16994	0.165614974	0.69265443	3.550999045	0.78613375	NA		NA
ASV_16s_16995	0.165614974	0.69265443	3.550999045	0.78613375	NA		NA
ASV_16s_16996	0.150933796	-0.280278873	3.550986389	0.718007281	NA		NA
ASV_16s_16997	0.138707134	0.294754771	3.551811453	0.547682225	NA		NA
ASV_16s_16998	0.138707134	0.294754771	3.551811453	0.547682225	NA		NA
ASV_16s_16999	0.138707134	0.294754771	3.551811453	0.547682225	NA		NA
ASV_16s_17000	0.138707134	0.294754771	3.551811453	0.547682225	NA		NA
ASV_16s_17001	0.138707134	0.294754771	3.551811453	0.547682225	NA		NA
ASV_16s_17002	0.138707134	0.294754771	3.551811453	0.547682225	NA		NA
ASV_16s_17003	0.138707134	0.294754771	3.551811453	0.547682225	NA		NA
ASV_16s_17004	0.138707134	0.294754771	3.551811453	0.547682225	NA		NA
ASV_16s_17005	0.138707134	0.294754771	3.551811453	0.547682225	NA		NA
ASV_16s_17006	0.138707134	0.294754771	3.551811453	0.547682225	NA		NA
ASV_16s_17007	0.138707134	0.294754771	3.551811453	0.547682225	NA		NA
ASV_16s_17008	0.140001728	0.110881466	3.551571404	0.715777729	NA		NA
ASV_16s_17009	0.140519565	0.052176978	3.551481372	0.768762393		0.997749828	NA
ASV_16s_17010	0.140778484	0.024557436	3.551438466	0.792064202		0.9975241	NA
ASV_16s_17011	0.13875847	0.010678816	3.551433127	0.801783774		0.997425707	NA
ASV_16s_17012	0.141555241	-0.051411939	3.551327675	0.849940894		0.99690026	NA
ASV_16s_17013	0.14181416	-0.074902474	3.551294433	0.865245972		0.99671969	NA
ASV_16s_17014	0.14181416	-0.074902474	3.551294433	0.865245972		0.99671969	NA
ASV_16s_17015	0.142331997	-0.119777132	3.551238149	0.884629264		0.996481341	NA
ASV_16s_17016	0.161965403	-0.203139346	3.55099119	0.973738104		0.995241125	NA
ASV_16s_17017	0.142590916	-0.141356306	3.551214338	0.889900349		0.996414632	NA
ASV_16s_17018	0.152010746	-0.174403705	3.551105142	0.929803164		0.995882847	NA
ASV_16s_17019	0.142590916	-0.141356306	3.551214338	0.889900349		0.996414632	NA
ASV_16s_17020	0.153894712	-0.18008301	3.551082204	0.937953664		0.995768311	NA
ASV_16s_17021	0.138798399	-0.147274996	3.551240555	0.877110487		0.99657509	NA
ASV_16s_17022	0.143108754	-0.183213484	3.55117391	0.894189364		0.996359748	NA
ASV_16s_17023	0.143108754	-0.183213484	3.55117391	0.894189364		0.996359748	NA
ASV_16s_17024	0.14133625	-0.176955404	3.551193442	0.887503544		0.996445066	NA
ASV_16s_17025	0.144989196	-0.209020485	3.551140554	0.899725873		0.996288099	NA
ASV_16s_17026	0.158197594	-0.282898513	3.550988583	0.935410476		0.995804268	NA
ASV_16s_17027	0.140340503	-0.232633999	3.551163897	0.876556473		0.996581933	NA
ASV_16s_17028	0.141859792	-0.237469847	3.551148718	0.881394402		0.996521877	NA
ASV_16s_17029	0.156801835	-0.27957107	3.551002389	0.930713029		0.995870162	NA
ASV_16s_17030	0.142118711	-0.257721554	3.551135913	0.87598494		0.996588983	NA
ASV_16s_17031	0.162341612	-0.3287211	3.5509414	0.926901802		0.995923129	NA
ASV_16s_17032	0.138832623	-0.267484588	3.551158416	0.856424136		0.996824585	NA
ASV_16s_17033	0.15220426	-0.305561424	3.551035688	0.896136267		0.996334656	NA

ASV_16s_17034	0.144403348	-0.284011134	3.551104798	0.874785895	0.996603742	NA
ASV_16s_17035	0.144403348	-0.284011134	3.551104798	0.874785895	0.996603742	NA
ASV_16s_17036	0.162235853	-0.346791305	3.550940748	0.912784748	0.996115489	NA
ASV_16s_17037	0.14162369	-0.295613784	3.551123086	0.852654785	0.996868727	NA
ASV_16s_17038	0.145180105	-0.344814344	3.551081541	0.831713923	0.997106671	NA
ASV_16s_17039	0.141888312	-0.336265114	3.551110481	0.82049066	0.997229173	NA
ASV_16s_17040	0.141140076	-0.374827141	3.55111133	0.777216069	0.997669578	NA
ASV_16s_17041	0.142659365	-0.378804182	3.551099485	0.78207829	0.997622577	NA
ASV_16s_17042	0.185223673	-0.520976194	3.55080445	0.810815141	NA	NA
ASV_16s_17043	0.04958613	-0.18623189	3.5518084	0.283524455	NA	NA
ASV_16s_17044	0.04958613	-0.18623189	3.5518084	0.283524455	NA	NA
ASV_16s_17045	0.04958613	-0.18623189	3.5518084	0.283524455	NA	NA
ASV_16s_17046	0.04958613	-0.18623189	3.5518084	0.283524455	NA	NA
ASV_16s_17047	0.202723845	-0.635204127	3.550753881	0.692788948	0.998390532	NA
ASV_16s_17048	0.181821924	-0.6064964	3.550868896	0.657301043	0.998642738	NA
ASV_16s_17049	0.192200436	-0.622260551	3.550813041	0.676438063	0.998510298	NA
ASV_16s_17050	0.173257595	-0.592445722	3.550917719	0.640718795	0.998750928	NA
ASV_16s_17051	0.193071078	-0.623526694	3.550808542	0.677995581	0.998499154	NA
ASV_16s_17052	0.17904005	-0.602072916	3.550884637	0.651998707	0.998677988	NA
ASV_16s_17053	0.170620311	-0.587733178	3.550932261	0.635465599	0.998783956	NA
ASV_16s_17054	0.195160619	-0.62654392	3.550797876	0.681708199	NA	NA
ASV_16s_17055	0.195160619	-0.62654392	3.550797876	0.681708199	NA	NA
ASV_16s_17056	0.173476304	-0.592849425	3.55091648	0.641176209	NA	NA
ASV_16s_17057	0.173476304	-0.592849425	3.55091648	0.641176209	NA	NA
ASV_16s_17058	0.173476304	-0.592849425	3.55091648	0.641176209	NA	NA
ASV_16s_17059	0.191425878	-0.621131095	3.550817068	0.675047856	NA	NA
ASV_16s_17060	0.147510374	-0.542599005	3.551075672	0.586327905	NA	NA
ASV_16s_17061	0.147510374	-0.542599005	3.551075672	0.586327905	NA	NA
ASV_16s_17062	0.147510374	-0.542599005	3.551075672	0.586327905	NA	NA
ASV_16s_17063	0.147510374	-0.542599005	3.551075672	0.586327905	NA	NA
ASV_16s_17064	0.153131983	-0.55420343	3.551037933	0.59738837	0.999006027	NA
ASV_16s_17065	0.141433219	-0.529225969	3.551117666	0.572404042	0.999135821	NA
ASV_16s_17066	0.138901072	-0.522992103	3.551133455	0.566426959	NA	NA
ASV_16s_17067	0.138901072	-0.522992103	3.551133455	0.566426959	NA	NA
ASV_16s_17068	0.138901072	-0.522992103	3.551133455	0.566426959	NA	NA
ASV_16s_17069	0.138901072	-0.522992103	3.551133455	0.566426959	NA	NA
ASV_16s_17070	0.138901072	-0.522992103	3.551133455	0.566426959	NA	NA
ASV_16s_17071	0.04425477	-0.159573583	3.55106879	0.203773072	NA	NA
ASV_16s_17072	0.061692678	0.220286876	3.551044391	0.280552351	NA	NA
ASV_16s_17076	0.154504725	-0.375620109	3.551244798	0.719989038	0.998177392	NA
ASV_16s_17077	0.171571836	-0.190165636	3.551049798	0.609386684	NA	NA
ASV_16s_17078	0.154152826	-0.125034662	3.55114436	0.503261814	NA	NA
ASV_16s_17079	0.166014518	-0.716101759	3.551007562	0.509584094	NA	NA
ASV_16s_17080	0.144073318	-0.177838847	3.551104108	0.629093167	NA	NA
ASV_16s_17081	0.154255983	0.121323583	3.551018124	0.818039871	0.997255462	NA
ASV_16s_17082	0.187754006	-0.614727308	3.550741788	0.756209634	0.997865593	NA
ASV_16s_17083	0.189641337	-0.619155312	3.55072798	0.760596213	NA	NA
ASV_16s_17084	0.180150659	-0.581141609	3.550757166	0.760869628	0.997823088	NA
ASV_16s_17085	0.169403619	1.01102186	3.550460685	0.472054185	NA	NA
ASV_16s_17086	0.252468187	-0.891409308	3.550460677	0.640125749	0.998754686	NA
ASV_16s_17087	0.14438107	-0.101388622	3.551351559	0.693072233	0.998388402	NA
ASV_16s_17088	0.184304068	-0.536255965	3.550686371	0.83379766	0.997083543	NA
ASV_16s_17089	0.147952502	-0.049388011	3.550966355	0.720603372	0.998172373	NA
ASV_16s_17090	0.169724168	-0.040045889	3.550779127	0.800435251	0.997439509	NA
ASV_16s_17091	0.178901723	-0.479678613	3.550723747	0.83854286	0.997030426	NA
ASV_16s_17092	0.184296321	-0.480699382	3.550684925	0.863005609	0.99674654	NA
ASV_16s_17093	0.151945556	-0.381177367	3.551333839	0.541685728	0.999279078	NA
ASV_16s_17094	0.180133202	-0.39915792	3.55074842	0.863491031	0.996740735	NA
ASV_16s_17095	0.205745502	-0.414502268	3.551228216	0.525365796	0.999348199	NA
ASV_16s_17096	0.168331195	-0.330921136	3.551103971	0.879797063	0.996541781	NA
ASV_16s_17097	0.160755694	0.429300266	3.551251225	0.582351371	0.999085609	NA
ASV_16s_17098	0.182538034	0.486013829	3.551329643	0.806782456	0.997374119	NA
ASV_16s_17099	0.149839095	0.620948652	3.551191614	0.725170255	NA	NA
ASV_16s_17100	0.181411219	0.267771867	3.550874911	0.730280097	0.998092107	NA
ASV_16s_17101	0.166787631	-0.224662135	3.550914677	0.818882897	0.997246438	NA
ASV_16s_17102	0.208151778	0.254784897	3.551021297	0.947721652	NA	NA
ASV_16s_17103	0.178274514	-0.113970234	3.55188669	0.478627594	0.999521036	NA

ASV_16s_17104	0.172721148	0.205032247	3.550872425	0.785103334	NA	NA
ASV_16s_17105	0.174113834	-0.110740811	3.550906725	0.840549662	NA	NA
ASV_16s_17106	0.163209763	0.685856243	3.551047823	0.496192086	NA	NA
ASV_16s_17107	0.175391888	0.265542249	3.550918228	0.806942964	0.997372451	NA
ASV_16s_17108	0.168760785	0.664215363	3.550472082	0.787433435	NA	NA
ASV_16s_17109	0.166551186	0.311371749	3.551190473	0.647320874	NA	NA
ASV_16s_17110	0.17586248	0.695492214	3.551129832	0.62759113	0.998832362	NA
ASV_16s_17111	0.176454723	0.428147347	3.550939538	0.802599474	0.997417334	NA
ASV_16s_17112	0.200931001	0.789494048	3.550989789	0.766287538	0.997772972	NA
ASV_16s_17113	0.190533337	0.951786706	3.551003327	0.499783626	NA	NA
ASV_16s_17114	0.196962976	0.425212266	3.551029123	1.043376248	NA	NA
ASV_16s_17115	0.180113147	0.471402733	3.551463246	0.742321153	0.99798901	NA
ASV_16s_17116	0.186870476	0.639913296	3.550938284	0.833652976	0.997085153	NA
ASV_16s_17117	0.209562361	0.338975511	3.552017876	0.322463313	0.999872312	NA
ASV_16s_17118	0.138252713	-0.284237381	3.551742639	0.550348509	0.999240447	NA
ASV_16s_17119	0.173033896	1.167850978	3.551134083	0.587379791	NA	NA
ASV_16s_17120	0.158758379	1.140347496	3.551212975	0.558207904	NA	NA
ASV_16s_17121	0.187126191	1.466720266	3.550976743	0.412871056	0.999707137	NA
ASV_16s_17122	0.150513853	0.349938312	3.551803597	0.575647089	NA	NA
ASV_16s_17123	0.150513853	0.349938312	3.551803597	0.575647089	NA	NA
ASV_16s_17124	0.150513853	0.349938312	3.551803597	0.575647089	NA	NA
ASV_16s_17125	0.132705012	-0.295729668	3.55157726	0.4763547	0.999528538	NA
ASV_16s_17126	0.14645744	-0.072026744	3.551034604	0.74816763	NA	NA
ASV_16s_17127	0.177623371	-0.076038203	3.551081865	0.853767119	0.996855743	NA
ASV_16s_17128	0.26399931	0.390686854	3.551133039	0.763837955	NA	NA
ASV_16s_17129	0.157932141	0.352812861	3.551764276	0.591523442	NA	NA
ASV_16s_17130	0.211997651	0.377605741	3.551440847	0.690044518	NA	NA
ASV_16s_17131	0.211997651	0.377605741	3.551440847	0.690044518	NA	NA
ASV_16s_17132	0.19952847	-0.500905683	3.551094705	0.542863637	0.999273905	NA
ASV_16s_17133	0.187751507	0.367921093	3.551591275	0.649259084	NA	NA
ASV_16s_17134	0.195160619	-0.542040895	3.550686004	0.663582878	NA	NA
ASV_16s_17135	0.174960974	-0.527905114	3.550868394	0.62839043	0.998827508	NA
ASV_16s_17136	0.23308799	-0.562287479	3.550414986	0.721614151	NA	NA
ASV_16s_17137	0.167689102	-0.521497385	3.550940651	0.614793567	NA	NA
ASV_16s_17138	0.167689102	-0.521497385	3.550940651	0.614793567	NA	NA
ASV_16s_17139	0.165054047	-0.822403172	3.551009026	0.40415459	NA	NA
ASV_16s_17140	0.116488923	-0.431300975	3.551399866	0.499823103	NA	NA
ASV_16s_17141	0.116488923	-0.431300975	3.551399866	0.499823103	NA	NA
ASV_16s_17142	0.090137764	0.989123232	3.551416241	0.328485979	NA	NA
ASV_16s_17143	0.090137764	0.989123232	3.551416241	0.328485979	NA	NA
ASV_16s_17144	0.090137764	0.989123232	3.551416241	0.328485979	NA	NA
ASV_16s_17145	0.154527882	1.16496505	3.550999148	0.490150152	NA	NA
ASV_16s_17146	0.240255366	0.409203797	3.550914817	0.665155079	NA	NA
ASV_16s_17147	0.177192048	0.381554482	3.551293152	0.564472113	NA	NA
ASV_16s_17148	0.184928176	-0.215612907	3.550298216	0.92824189	0.995904556	NA
ASV_16s_17149	0.132807114	0.362081914	3.551547956	0.471555106	NA	NA
ASV_16s_17150	0.132807114	0.362081914	3.551547956	0.471555106	NA	NA
ASV_16s_17151	0.132807114	0.362081914	3.551547956	0.471555106	NA	NA
ASV_16s_17152	0.132807114	0.362081914	3.551547956	0.471555106	NA	NA
ASV_16s_17153	0.22750466	0.404393449	3.550977822	0.646850837	NA	NA
ASV_16s_17154	0.211237892	0.676465381	3.550678592	0.966500386	0.995350982	NA
ASV_16s_17155	0.14369259	0.366725404	3.551487289	0.496395651	NA	NA
ASV_16s_17156	0.14369259	0.366725404	3.551487289	0.496395651	NA	NA
ASV_16s_17157	0.050032535	0.341429288	3.551868277	0.222538674	NA	NA
ASV_16s_17158	0.080732489	-0.348407318	3.551394245	0.332948415	NA	NA
ASV_16s_17159	0.080732489	-0.348407318	3.551394245	0.332948415	NA	NA
ASV_16s_17160	0.080732489	-0.348407318	3.551394245	0.332948415	NA	NA
ASV_16s_17161	0.080732489	-0.348407318	3.551394245	0.332948415	NA	NA
ASV_16s_17162	0.080732489	-0.348407318	3.551394245	0.332948415	NA	NA
ASV_16s_17163	0.115694093	-0.447087479	3.551114033	0.426612737	0.999673313	NA
ASV_16s_17164	0.165016817	-0.514328418	3.55085115	0.445064874	NA	NA
ASV_16s_17165	0.154890263	-0.512943774	3.550723245	0.521827289	NA	NA
ASV_16s_17166	0.098047756	-0.405480923	3.551265024	0.385037032	NA	NA
ASV_16s_17167	0.098047756	-0.405480923	3.551265024	0.385037032	NA	NA
ASV_16s_17168	0.160883117	-0.078491013	3.550854155	0.830753064	NA	NA
ASV_16s_17169	0.137186864	-0.078294765	3.550912496	0.75634734	NA	NA
ASV_16s_17170	0.137186864	-0.078294765	3.550912496	0.75634734	NA	NA

ASV_16s_17171	0.137186864	-0.078294765	3.550912496	0.75634734	NA	NA
ASV_16s_17172	0.137186864	-0.078294765	3.550912496	0.75634734	NA	NA
ASV_16s_17173	0.137186864	-0.078294765	3.550912496	0.75634734	NA	NA
ASV_16s_17174	0.137186864	-0.078294765	3.550912496	0.75634734	NA	NA
ASV_16s_17175	0.137186864	-0.078294765	3.550912496	0.75634734	NA	NA
ASV_16s_17176	0.137186864	-0.078294765	3.550912496	0.75634734	NA	NA
ASV_16s_17177	0.137186864	-0.078294765	3.550912496	0.75634734	NA	NA
ASV_16s_17178	0.137186864	-0.078294765	3.550912496	0.75634734	NA	NA
ASV_16s_17179	0.137186864	-0.078294765	3.550912496	0.75634734	NA	NA
ASV_16s_17180	0.142362283	-0.078340556	3.550898109	0.773395879	0.997706072	NA
ASV_16s_17181	0.102130877	0.220147122	3.550859988	0.467036363	NA	NA
ASV_16s_17182	0.102130877	0.220147122	3.550859988	0.467036363	NA	NA
ASV_16s_17183	0.102130877	0.220147122	3.550859988	0.467036363	NA	NA
ASV_16s_17184	0.102130877	0.220147122	3.550859988	0.467036363	NA	NA
ASV_16s_17185	0.102130877	0.220147122	3.550859988	0.467036363	NA	NA
ASV_16s_17186	0.101980915	0.220147419	3.550860403	0.466432527	0.999560346	NA
ASV_16s_17187	0.101926929	0.220147627	3.550860554	0.466214916	NA	NA
ASV_16s_17188	0.199830128	-0.134918423	3.55073062	0.776857388	0.99767302	NA
ASV_16s_17189	0.240135573	0.603878189	3.551226413	0.808189221	NA	NA
ASV_16s_17190	0.190260494	-0.570855147	3.550640211	0.486862481	0.999493178	NA
ASV_16s_17191	0.147717636	-0.067389953	3.550897348	0.898995252	0.996297606	NA
ASV_16s_17192	0.142142047	-0.067293396	3.550927629	0.878681244	NA	NA
ASV_16s_17193	0.143628602	-0.067436647	3.550930692	0.915343796	0.996081065	NA
ASV_16s_17194	0.143628602	-0.067436647	3.550930692	0.915343796	0.996081065	NA
ASV_16s_17195	0.229951967	-1.049011701	3.55081904	0.437176874	0.999645558	NA
ASV_16s_17196	0.144619639	-0.066946316	3.550941785	0.924572542	0.995955283	NA
ASV_16s_17197	0.154226661	-0.078066594	3.551174763	0.742667389	0.997985992	NA
ASV_16s_17198	0.149148327	1.381755505	3.551350666	0.298634724	0.999901495	NA
ASV_16s_17199	0.168699151	0.072365787	3.550618137	1.150871865	0.992014426	NA
ASV_16s_17200	0.151237566	-0.064631434	3.550933965	0.94279248	0.995699349	NA
ASV_16s_17201	0.139179347	0.084691351	3.551449928	0.703187584	0.998311125	NA
ASV_16s_17202	0.148583786	-0.048304033	3.551060504	0.910044495	NA	NA
ASV_16s_17203	0.185749055	0.356171566	3.551753523	0.58131619	NA	NA
ASV_16s_17204	0.19195264	0.364693481	3.551559574	0.641214685	NA	NA
ASV_16s_17205	0.150774275	0.352474549	3.551547435	0.707788789	NA	NA
ASV_16s_17206	0.145058436	0.913987116	3.55150671	0.48004909	NA	NA
ASV_16s_17207	0.221215784	0.85392383	3.550958109	0.760308253	NA	NA
ASV_16s_17208	0.148512489	1.158720005	3.551490351	0.45510655	0.999594827	NA
ASV_16s_17209	0.171752496	1.001305729	3.551260617	0.561296115	NA	NA
ASV_16s_17210	0.171752496	1.001305729	3.551260617	0.561296115	NA	NA
ASV_16s_17211	0.272746471	0.279444365	3.551394658	0.800931946	NA	NA
ASV_16s_17212	0.193716755	0.279457537	3.551558036	0.676356545	NA	NA
ASV_16s_17213	0.193716755	0.279457537	3.551558036	0.676356545	NA	NA
ASV_16s_17214	0.135268143	-0.427138083	3.551403286	0.545864842	NA	NA
ASV_16s_17215	0.13633849	0.936678623	3.551462764	0.486197355	NA	NA
ASV_16s_17216	0.265427392	-0.052267414	3.54981882	1.012413955	NA	NA
ASV_16s_17217	0.239486371	-0.293457766	3.550670896	0.897687683	0.996314581	NA
ASV_16s_17218	0.182215376	-0.533794209	3.550476562	0.573073099	NA	NA
ASV_16s_17219	0.202042412	1.240449702	3.55078163	0.581040476	NA	NA
ASV_16s_17220	0.202042412	1.240449702	3.55078163	0.581040476	NA	NA
ASV_16s_17221	0.202042412	1.240449702	3.55078163	0.581040476	NA	NA
ASV_16s_17222	0.189016834	1.139189525	3.550617908	0.776184727	0.997679468	NA
ASV_16s_17223	0.205169198	0.744837938	3.550880929	0.888504257	0.996432379	NA
ASV_16s_17224	0.115130351	-0.078040504	3.550988215	0.67779396	NA	NA
ASV_16s_17225	0.115130351	-0.078040504	3.550988215	0.67779396	NA	NA
ASV_16s_17226	0.154463213	-0.067290573	3.550860693	0.935938697	NA	NA
ASV_16s_17227	0.315393984	0.005853813	3.550777103	1.471417273	NA	NA
ASV_16s_17228	0.26679633	0.34102958	3.550105232	1.37583826	0.986299847	NA
ASV_16s_17229	0.239237799	0.30669032	3.550600227	1.207246117	NA	NA
ASV_16s_17230	0.267548105	0.148393338	3.550844702	1.863677064	0.966998641	NA
ASV_16s_17231	0.231425828	0.429838249	3.550923571	1.533809176	0.981116051	NA
ASV_16s_17232	0.167435781	0.195520561	3.550702834	1.006624893	0.994720959	NA
ASV_16s_17233	0.151898361	-0.028381777	3.55105287	0.86267691	NA	NA
ASV_16s_17234	0.155333709	0.326253845	3.550833419	0.953837679	0.995539226	NA
ASV_16s_17235	0.173563208	-0.073470956	3.550860651	0.89132194	NA	NA
ASV_16s_17236	0.173563208	-0.073470956	3.550860651	0.89132194	NA	NA
ASV_16s_17237	0.126763557	-0.073022271	3.550987853	0.744594459	NA	NA

ASV_16s_17238	0.126763557	-0.073022271	3.550987853	0.744594459	NA	NA
ASV_16s_17239	0.126763557	-0.073022271	3.550987853	0.744594459	NA	NA
ASV_16s_17240	0.124485827	-0.072991943	3.550996465	0.736471826	NA	NA
ASV_16s_17241	0.124485827	-0.072991943	3.550996465	0.736471826	NA	NA
ASV_16s_17242	0.124485827	-0.072991943	3.550996465	0.736471826	NA	NA
ASV_16s_17243	0.124485827	-0.072991943	3.550996465	0.736471826	NA	NA
ASV_16s_17244	0.124485827	-0.072991943	3.550996465	0.736471826	NA	NA
ASV_16s_17245	0.154457131	-0.073320236	3.550903331	0.835629874	NA	NA
ASV_16s_17246	0.173273874	0.360295925	3.551178984	0.908462508	NA	NA
ASV_16s_17247	0.173273874	0.360295925	3.551178984	0.908462508	NA	NA
ASV_16s_17248	0.173273874	0.360295925	3.551178984	0.908462508	NA	NA
ASV_16s_17249	0.173273874	0.360295925	3.551178984	0.908462508	NA	NA
ASV_16s_17250	0.231823813	0.360943406	3.551263748	0.784756258	0.997596426	NA
ASV_16s_17251	0.251519605	0.714349652	3.550793274	1.084144413	NA	NA
ASV_16s_17252	0.154034487	0.36027383	3.551251357	0.852138166	NA	NA
ASV_16s_17253	0.154034487	0.36027383	3.551251357	0.852138166	NA	NA
ASV_16s_17254	0.154034487	0.36027383	3.551251357	0.852138166	NA	NA
ASV_16s_17255	0.154034487	0.36027383	3.551251357	0.852138166	NA	NA
ASV_16s_17256	0.154034487	0.36027383	3.551251357	0.852138166	NA	NA
ASV_16s_17257	0.154034487	0.36027383	3.551251357	0.852138166	NA	NA
ASV_16s_17258	0.154034487	0.36027383	3.551251357	0.852138166	NA	NA
ASV_16s_17259	0.154034487	0.36027383	3.551251357	0.852138166	NA	NA
ASV_16s_17260	0.154034487	0.36027383	3.551251357	0.852138166	NA	NA
ASV_16s_17261	0.154034487	0.36027383	3.551251357	0.852138166	NA	NA
ASV_16s_17262	0.198994687	0.646550924	3.551070814	1.237181315	0.990045458	NA
ASV_16s_17263	0.175049975	0.320428634	3.551042123	0.800038262	0.997443563	NA
ASV_16s_17264	0.218957985	0.676239729	3.551037519	1.176517545	NA	NA
ASV_16s_17265	0.185923345	0.315939676	3.550972965	0.903842069	0.996234241	NA
ASV_16s_17266	0.187641702	0.315939672	3.55096945	0.908359944	0.996174546	NA
ASV_16s_17267	0.183709225	0.315939681	3.550977609	0.896594782	0.996328731	NA
ASV_16s_17268	0.171134227	0.315939716	3.551006078	0.863545798	0.996740079	NA
ASV_16s_17269	0.1773403	0.606768288	3.550979621	0.940976558	0.995725314	NA
ASV_16s_17270	0.170122399	0.31593984	3.551008549	0.860697287	0.996774054	NA
ASV_16s_17271	0.16772703	0.315939849	3.551014526	0.853863873	0.996854612	NA
ASV_16s_17272	0.16772703	0.315939849	3.551014526	0.853863873	0.996854612	NA
ASV_16s_17273	0.167042639	0.315939851	3.551016264	0.851897915	0.996877542	NA
ASV_16s_17274	0.164305074	0.315939862	3.551023364	0.843974648	0.99696885	NA
ASV_16s_17275	0.189127635	0.315939668	3.550966507	0.910178866	0.99615034	NA
ASV_16s_17276	0.160883117	0.315939876	3.551032574	0.833917262	NA	NA
ASV_16s_17277	0.160883117	0.315939876	3.551032574	0.833917262	NA	NA
ASV_16s_17278	0.160883117	0.315939876	3.551032574	0.833917262	NA	NA
ASV_16s_17279	0.160883117	0.315939876	3.551032574	0.833917262	NA	NA
ASV_16s_17280	0.160883117	0.315939876	3.551032574	0.833917262	NA	NA
ASV_16s_17281	0.160883117	0.315939876	3.551032574	0.833917262	NA	NA
ASV_16s_17282	0.160883117	0.315939876	3.551032574	0.833917262	NA	NA
ASV_16s_17283	0.177589266	0.315939813	3.550991006	0.87901335	0.99655152	NA
ASV_16s_17284	0.174124591	0.465003565	3.550955258	1.2505889	0.989715076	NA
ASV_16s_17285	0.160883117	0.507571193	3.550983449	1.190556511	0.991142709	NA
ASV_16s_17286	0.153214951	0.687529527	3.550987111	0.72542204	NA	NA
ASV_16s_17287	0.276793525	0.688132912	3.550794627	1.012968617	NA	NA
ASV_16s_17288	0.233523131	1.156070779	3.550855183	0.726875107	NA	NA
ASV_16s_17289	0.208438579	1.095066484	3.550893378	0.695675527	NA	NA
ASV_16s_17290	0.171289012	0.733448845	3.551202139	0.835135887	0.997068627	NA
ASV_16s_17291	0.212828595	0.733867483	3.551080415	0.947232907	NA	NA
ASV_16s_17292	0.212828595	0.733867483	3.551080415	0.947232907	NA	NA
ASV_16s_17293	0.173105583	1.001473406	3.551066441	0.645089431	NA	NA
ASV_16s_17294	0.201328865	0.856549571	3.550938377	0.775315785	0.997687779	NA
ASV_16s_17295	0.212828595	0.733867483	3.551080415	0.947232907	NA	NA
ASV_16s_17296	0.212828595	0.733867483	3.551080415	0.947232907	NA	NA
ASV_16s_17297	0.212828595	0.733867483	3.551080415	0.947232907	NA	NA
ASV_16s_17298	0.212828595	0.733867483	3.551080415	0.947232907	NA	NA
ASV_16s_17299	0.225110773	1.046678458	3.550827406	0.740280863	0.998006732	NA
ASV_16s_17300	0.10542585	0.713708152	3.551510721	0.628334806	NA	NA
ASV_16s_17301	0.10542585	0.713708152	3.551510721	0.628334806	NA	NA
ASV_16s_17302	0.10542585	0.713708152	3.551510721	0.628334806	NA	NA
ASV_16s_17303	0.10542585	0.713708152	3.551510721	0.628334806	NA	NA
ASV_16s_17304	0.199843127	0.692912207	3.550923008	0.875798014	0.996591287	NA

ASV_16s_17305	0.176109568	0.692743279	3.55097264	0.81495851	0.997288283	NA
ASV_16s_17306	0.184451553	0.692804794	3.550953752	0.837033137	0.997047394	NA
ASV_16s_17307	0.203475854	0.692934621	3.550916428	0.884583222	NA	NA
ASV_16s_17308	0.203475854	0.692934621	3.550916428	0.884583222	NA	NA
ASV_16s_17309	0.203475854	0.692934621	3.550916428	0.884583222	NA	NA
ASV_16s_17310	0.203475854	0.692934621	3.550916428	0.884583222	NA	NA
ASV_16s_17311	0.179674031	0.692771447	3.55096436	0.824482587	0.997185999	NA
ASV_16s_17312	0.160883117	0.692609069	3.551012117	0.772547455	NA	NA
ASV_16s_17313	0.160883117	0.692609069	3.551012117	0.772547455	NA	NA
ASV_16s_17314	0.160883117	0.692609069	3.551012117	0.772547455	NA	NA
ASV_16s_17315	0.189572977	0.692844139	3.550942998	0.848801217	0.99691344	NA
ASV_16s_17316	0.236345803	0.693106442	3.550866038	0.958505859	NA	NA
ASV_16s_17317	0.235918134	0.69310451	3.550866604	0.957604686	NA	NA
ASV_16s_17318	0.134627512	0.295035958	3.551822438	0.537888949	NA	NA
ASV_16s_17319	0.134627512	0.295035958	3.551822438	0.537888949	NA	NA
ASV_16s_17320	0.134627512	0.295035958	3.551822438	0.537888949	NA	NA
ASV_16s_17321	0.134627512	0.295035958	3.551822438	0.537888949	NA	NA
ASV_16s_17322	0.134627512	0.295035958	3.551822438	0.537888949	NA	NA
ASV_16s_17323	0.134627512	0.295035958	3.551822438	0.537888949	NA	NA
ASV_16s_17324	0.134627512	0.295035958	3.551822438	0.537888949	NA	NA
ASV_16s_17325	0.134627512	0.295035958	3.551822438	0.537888949	NA	NA
ASV_16s_17326	0.136957782	-0.004177494	3.551426021	0.798807268	0.997456107	NA
ASV_16s_17327	0.150951808	-0.071821968	3.551224482	0.873507836	0.996619428	NA
ASV_16s_17328	0.14238323	-0.055138412	3.551315741	0.843408692	0.996975304	NA
ASV_16s_17329	0.137475619	-0.054490127	3.551357205	0.834109164	0.997080076	NA
ASV_16s_17330	0.134690257	-0.040547989	3.551396181	0.820894629	0.997224823	NA
ASV_16s_17331	0.137734538	-0.078549669	3.551324964	0.847561972	0.996927729	NA
ASV_16s_17332	0.136721679	-0.073764714	3.551338557	0.842886337	0.996981253	NA
ASV_16s_17333	0.137734538	-0.078549669	3.551324964	0.847561972	0.996927729	NA
ASV_16s_17334	0.137993457	-0.101726552	3.551295381	0.856036205	0.996829147	NA
ASV_16s_17335	0.137993457	-0.101726552	3.551295381	0.856036205	0.996829147	NA
ASV_16s_17336	0.137993457	-0.101726552	3.551295381	0.856036205	0.996829147	NA
ASV_16s_17337	0.138252376	-0.124172863	3.551268389	0.862579943	0.996751625	NA
ASV_16s_17338	0.149556172	-0.166459777	3.551134864	0.912379511	0.996120922	NA
ASV_16s_17339	0.138252376	-0.124172863	3.551268389	0.862579943	0.996751625	NA
ASV_16s_17340	0.134713073	-0.130787838	3.551290682	0.851472043	0.996882495	NA
ASV_16s_17341	0.134713073	-0.130787838	3.551290682	0.851472043	0.996882495	NA
ASV_16s_17342	0.138770213	-0.167564755	3.551222927	0.869151029	0.996672546	NA
ASV_16s_17343	0.138770213	-0.167564755	3.551222927	0.869151029	0.996672546	NA
ASV_16s_17344	0.138770213	-0.167564755	3.551222927	0.869151029	0.996672546	NA
ASV_16s_17345	0.138770213	-0.167564755	3.551222927	0.869151029	0.996672546	NA
ASV_16s_17346	0.134718777	-0.152014614	3.551270307	0.853432545	0.996859652	NA
ASV_16s_17347	0.139029132	-0.188652913	3.551203759	0.86958142	0.996667323	NA
ASV_16s_17348	0.137009118	-0.201643354	3.551211545	0.86018032	0.996780195	NA
ASV_16s_17349	0.137768762	-0.204265503	3.551203222	0.862863682	0.996748236	NA
ASV_16s_17350	0.134730185	-0.193499095	3.551236284	0.852196992	0.996874061	NA
ASV_16s_17351	0.13954697	-0.230010895	3.551172178	0.865139752	0.996720967	NA
ASV_16s_17352	0.14988003	-0.261759458	3.551073165	0.900967434	0.996271907	NA
ASV_16s_17353	0.139805889	-0.250477559	3.551159211	0.860254748	0.996779311	NA
ASV_16s_17354	0.139805889	-0.250477559	3.551159211	0.860254748	0.996779311	NA
ASV_16s_17355	0.151109685	-0.283770826	3.551052666	0.897193163	0.996320988	NA
ASV_16s_17356	0.13753266	-0.263431216	3.551171776	0.845880802	0.996947045	NA
ASV_16s_17357	0.134753001	-0.274783172	3.551188159	0.826823443	0.997160477	NA
ASV_16s_17358	0.145492043	-0.325851865	3.551082622	0.846663384	0.996938063	NA
ASV_16s_17359	0.134770114	-0.336636385	3.551167359	0.776650702	0.997675003	NA
ASV_16s_17360	0.141100483	-0.354293091	3.551114076	0.798190236	0.997462379	NA
ASV_16s_17361	0.048127715	-0.177878214	3.551815271	0.277268578	NA	NA
ASV_16s_17362	0.048127715	-0.177878214	3.551815271	0.277268578	NA	NA
ASV_16s_17363	0.048127715	-0.177878214	3.551815271	0.277268578	NA	NA
ASV_16s_17364	0.194015877	-0.624837378	3.550803904	0.678911516	0.998492575	NA
ASV_16s_17365	0.183674363	-0.609369237	3.55085876	0.660724981	NA	NA
ASV_16s_17366	0.187156932	-0.614766275	3.550839891	0.667212927	0.99857517	NA
ASV_16s_17367	0.157900026	-0.563736504	3.55100758	0.608996832	0.998941497	NA
ASV_16s_17368	0.187679317	-0.615555121	3.55083709	0.668173552	0.998568504	NA
ASV_16s_17369	0.189420601	-0.618141441	3.550827772	0.671350202	NA	NA
ASV_16s_17370	0.189420601	-0.618141441	3.550827772	0.671350202	NA	NA
ASV_16s_17371	0.180491765	-0.604345002	3.550876531	0.654698954	0.998660114	NA

ASV_16s_17372	0.177940669	-0.600262355	3.550891124	0.64980657	0.99869238	NA
ASV_16s_17373	0.168374059	-0.583631501	3.550945035	0.630880374	NA	NA
ASV_16s_17374	0.168374059	-0.583631501	3.550945035	0.630880374	NA	NA
ASV_16s_17375	0.159533302	-0.566933017	3.550997529	0.612459909	0.998921714	NA
ASV_16s_17376	0.143171834	-0.533284205	3.551106607	0.576391294	NA	NA
ASV_16s_17377	0.143171834	-0.533284205	3.551106607	0.576391294	NA	NA
ASV_16s_17378	0.143171834	-0.533284205	3.551106607	0.576391294	NA	NA
ASV_16s_17379	0.143171834	-0.533284205	3.551106607	0.576391294	NA	NA
ASV_16s_17380	0.139880042	-0.525365601	3.551127422	0.56868087	0.999154126	NA
ASV_16s_17381	0.139120397	-0.523486989	3.551132195	0.566880448	0.999162883	NA
ASV_16s_17382	0.137601109	-0.519701321	3.551141862	0.563256213	0.999180324	NA
ASV_16s_17383	0.137601109	-0.519701321	3.551141862	0.563256213	0.999180324	NA
ASV_16s_17384	0.137347894	-0.519066646	3.551143489	0.562649135	0.999183222	NA
ASV_16s_17385	0.134815746	-0.512722794	3.55115959	0.556508707	NA	NA
ASV_16s_17386	0.134815746	-0.512722794	3.55115959	0.556508707	NA	NA
ASV_16s_17387	0.134815746	-0.512722794	3.55115959	0.556508707	NA	NA
ASV_16s_17388	0.134815746	-0.512722794	3.55115959	0.556508707	NA	NA
ASV_16s_17389	0.134815746	-0.512722794	3.55115959	0.556508707	NA	NA
ASV_16s_17390	0.134815746	-0.512722794	3.55115959	0.556508707	NA	NA
ASV_16s_17391	0.134815746	-0.512722794	3.55115959	0.556508707	NA	NA
ASV_16s_17392	0.134815746	-0.512722794	3.55115959	0.556508707	NA	NA
ASV_16s_17393	0.050898789	0.041990041	3.550995105	0.316630773	0.999879944	NA
ASV_16s_17394	0.038099767	-0.152528167	3.551130325	0.165255473	NA	NA
ASV_16s_17395	0.087265044	0.220183659	3.550908053	0.404207217	NA	NA
ASV_16s_17396	0.087265044	0.220183659	3.550908053	0.404207217	NA	NA
ASV_16s_17397	0.045290399	0.224454347	3.551176049	0.188271438	NA	NA
ASV_16s_17398	0.045290399	0.224454347	3.551176049	0.188271438	NA	NA
ASV_16s_17400	0.114800364	0.687070086	3.551131525	0.57949303	0.999100233	NA
ASV_16s_17401	0.143027434	-0.153512664	3.551377272	0.762532426	0.997807787	NA
ASV_16s_17402	0.006328636	0.407894309	3.55200467	-0.030111831	1	NA
ASV_16s_17403	0.157740257	-0.545992073	3.551034228	0.645769485	0.99871861	NA
ASV_16s_17404	0.179614095	-0.593067079	3.550800386	0.735171721	0.998050657	NA
ASV_16s_17405	0.175573947	-0.569959338	3.550799566	0.744242756	0.997972223	NA
ASV_16s_17406	0.179459331	-0.581147942	3.550767693	0.75342078	0.997890766	NA
ASV_16s_17407	0.14835922	-0.222851273	3.551121792	0.685248046	NA	NA
ASV_16s_17408	0.142878197	0.307449425	3.551960754	0.420152277	0.999689531	NA
ASV_16s_17409	0.158689124	0.211871856	3.551047473	0.858898952	0.996795383	NA
ASV_16s_17410	0.17469583	-0.55169572	3.550784625	0.761396682	0.997818246	NA
ASV_16s_17411	0.174976059	-0.518234974	3.551259107	0.632919563	NA	NA
ASV_16s_17412	0.167433497	0.085884711	3.551267023	0.484892451	0.99949994	NA
ASV_16s_17413	0.161278312	-0.102992017	3.550831369	0.737362922	0.998031898	NA
ASV_16s_17414	0.164200899	0.681052893	3.551610545	0.794249516	0.997502197	NA
ASV_16s_17415	0.174806891	-0.537150356	3.550774571	0.777268883	0.99766907	NA
ASV_16s_17416	0.167991301	-0.346850795	3.551022313	0.670119696	0.998554937	NA
ASV_16s_17417	0.147752451	0.866777269	3.551799536	0.556652521	0.99921147	NA
ASV_16s_17418	0.171959944	0.67922129	3.551310816	0.794826339	0.997496394	NA
ASV_16s_17419	0.173513457	0.404740371	3.551060724	0.894086437	0.996361072	NA
ASV_16s_17420	0.1926564	-0.509479481	3.551268164	0.615833303	0.998902206	NA
ASV_16s_17421	0.169932966	-0.519474451	3.550817434	0.768939796	0.997748163	NA
ASV_16s_17422	0.13009751	-0.68841414	3.551085163	0.354716462	0.999823947	NA
ASV_16s_17423	0.228858301	1.163535914	3.550855818	0.677675795	0.998501447	NA
ASV_16s_17424	0.180835051	-0.528396728	3.550718573	0.819295284	0.997242016	NA
ASV_16s_17425	0.19220539	-0.131997171	3.551087575	0.855852125	0.99683131	NA
ASV_16s_17426	0.18407195	-0.503335272	3.550685444	0.850694893	0.99689152	NA
ASV_16s_17427	0.187100418	-0.549749491	3.550665664	0.853384623	0.996860212	NA
ASV_16s_17428	0.123593568	0.316321468	3.552008415	0.323271314	0.999871229	NA
ASV_16s_17429	0.177697515	-0.453987317	3.55073597	0.838444169	0.997031537	NA
ASV_16s_17430	0.152628808	1.024406674	3.551401097	0.504629235	0.999429354	NA
ASV_16s_17431	0.175475347	-0.416669586	3.550763285	0.837977511	0.997036787	NA
ASV_16s_17432	0.147614596	0.035821899	3.550943622	0.706232923	0.998287386	NA
ASV_16s_17433	0.165296071	-0.410500876	3.550833646	0.800719855	0.9974366	NA
ASV_16s_17434	0.176369421	-0.129918478	3.550767244	1.004325051	0.994758465	NA
ASV_16s_17435	0.172795208	0.141519317	3.550894242	0.829372827	0.997132511	NA
ASV_16s_17436	0.184607259	-0.424862128	3.550779972	0.898911459	0.996298695	NA
ASV_16s_17437	0.155787406	0.603701197	3.551330916	0.373022862	0.99979152	NA
ASV_16s_17438	0.197730403	-0.300847235	3.551497461	0.693733102	NA	NA
ASV_16s_17439	0.177580762	-0.350650231	3.55079819	0.850888064	0.996889278	NA

ASV_16s_17440	0.175881953	-0.096670303	3.550991776	0.852714609	NA	NA
ASV_16s_17441	0.184785094	-0.021529046	3.55087662	0.842366782	0.996987163	NA
ASV_16s_17442	0.134941379	-0.032256742	3.551698152	0.56265202	0.999183208	NA
ASV_16s_17443	0.178792683	0.367480746	3.551595433	0.434693181	0.999652223	NA
ASV_16s_17444	0.207603173	-0.006957656	3.256662806	1.521003167	0.98157361	NA
ASV_16s_17445	0.201892113	0.254205983	3.551043782	0.92668619	NA	NA
ASV_16s_17446	0.166157851	-0.033333482	3.551539585	0.63112778	0.998810785	NA
ASV_16s_17447	0.172554434	-0.00244065	3.550943431	0.824894582	0.997181518	NA
ASV_16s_17448	0.163842532	-0.00243682	3.550959047	0.79792718	0.99746505	NA
ASV_16s_17449	0.18482256	0.929527778	3.550979792	0.557382067	0.999208069	NA
ASV_16s_17450	0.160321428	0.054813145	3.551019768	0.805474521	NA	NA
ASV_16s_17451	0.186013491	0.428242503	3.550911371	0.832393463	0.997099142	NA
ASV_16s_17452	0.176363783	0.141147209	3.551941108	0.40288441	0.999730152	NA
ASV_16s_17453	0.194776086	0.534109348	3.550914838	0.846366924	0.996941468	NA
ASV_16s_17454	0.175094989	1.436118725	3.55112493	0.347319681	0.999835997	NA
ASV_16s_17455	0.188334656	1.453744069	3.551058392	0.392266244	0.999753226	NA
ASV_16s_17456	0.160072941	0.936582509	3.551679326	0.583215033	0.999081158	NA
ASV_16s_17457	0.165562552	0.971787598	3.551388305	0.536476465	0.999301655	NA
ASV_16s_17458	0.234964682	0.277882624	3.551336033	0.724247395	0.998142415	NA
ASV_16s_17459	0.240679742	0.451472407	3.551857642	0.568488883	0.999155062	NA
ASV_16s_17460	0.233695411	0.278104266	3.551343945	0.722738724	0.998154857	NA
ASV_16s_17461	0.156015314	1.134433177	3.551228066	0.552269501	0.999231694	NA
ASV_16s_17462	0.154089015	1.130237108	3.551238792	0.548097445	NA	NA
ASV_16s_17463	0.154089015	1.130237108	3.551238792	0.548097445	NA	NA
ASV_16s_17464	0.19502715	0.695443446	3.551024203	0.983712406	0.995087016	NA
ASV_16s_17465	0.146086975	0.348639674	3.551825157	0.565779518	NA	NA
ASV_16s_17466	0.146086975	0.348639674	3.551825157	0.565779518	NA	NA
ASV_16s_17467	0.146086975	0.348639674	3.551825157	0.565779518	NA	NA
ASV_16s_17468	0.164983913	0.315832291	3.551197363	0.613940204	NA	NA
ASV_16s_17469	0.19561043	0.37138516	3.551541337	0.662901687	NA	NA
ASV_16s_17470	0.19561043	0.37138516	3.551541337	0.662901687	NA	NA
ASV_16s_17471	0.192322385	0.351861719	3.551887465	0.363624776	0.999808639	NA
ASV_16s_17472	0.11320868	0.337705124	3.551937645	0.484528111	NA	NA
ASV_16s_17473	0.11320868	0.337705124	3.551937645	0.484528111	NA	NA
ASV_16s_17474	0.11320868	0.337705124	3.551937645	0.484528111	NA	NA
ASV_16s_17475	0.11320868	0.337705124	3.551937645	0.484528111	NA	NA
ASV_16s_17476	0.153287078	0.350813123	3.551790119	0.581609032	NA	NA
ASV_16s_17477	0.214428107	0.352388016	3.551827364	0.387696837	0.999762724	NA
ASV_16s_17478	0.170816936	0.066380043	3.551062225	0.932102053	0.995850747	NA
ASV_16s_17479	0.202946832	-1.028948574	3.550548469	0.362162603	0.999811213	NA
ASV_16s_17480	0.19144274	-0.058660427	3.550488669	0.93558811	0.995801763	NA
ASV_16s_17481	0.189420601	-0.538309816	3.55073484	0.653870216	NA	NA
ASV_16s_17482	0.113062778	-0.422474933	3.551430893	0.490734183	NA	NA
ASV_16s_17483	0.149269818	-0.844930785	3.551013583	0.30406379	0.999895303	NA
ASV_16s_17484	0.167629889	1.188862098	3.550932721	0.517267845	NA	NA
ASV_16s_17485	0.170438558	1.193593572	3.550919127	0.522835176	NA	NA
ASV_16s_17486	0.188943794	0.284327089	3.550232397	0.945083569	0.995666445	NA
ASV_16s_17487	0.149982944	1.155566711	3.551021638	0.480198418	NA	NA
ASV_16s_17488	0.149982944	1.155566711	3.551021638	0.480198418	NA	NA
ASV_16s_17489	0.206716875	1.246027422	3.550762013	0.588814538	NA	NA
ASV_16s_17490	0.171980517	0.379299683	3.551325348	0.554597118	NA	NA
ASV_16s_17491	0.171980517	0.379299683	3.551325348	0.554597118	NA	NA
ASV_16s_17492	0.156151261	0.372717714	3.551411761	0.522964477	NA	NA
ASV_16s_17493	0.156151261	0.372717714	3.551411761	0.522964477	NA	NA
ASV_16s_17494	0.220813346	0.40174909	3.551013676	0.636789942	NA	NA
ASV_16s_17495	0.139466337	0.365122044	3.55151168	0.486861559	NA	NA
ASV_16s_17496	0.139466337	0.365122044	3.55151168	0.486861559	NA	NA
ASV_16s_17497	0.139466337	0.365122044	3.55151168	0.486861559	NA	NA
ASV_16s_17498	0.228156371	0.31164432	3.550113926	1.329287231	0.987639382	NA
ASV_16s_17499	0.04856099	0.341319451	3.551875174	0.21681556	NA	NA
ASV_16s_17500	0.04856099	0.341319451	3.551875174	0.21681556	NA	NA
ASV_16s_17501	0.078358004	-0.339714837	3.551414056	0.325293592	NA	NA
ASV_16s_17502	0.078358004	-0.339714837	3.551414056	0.325293592	NA	NA
ASV_16s_17503	0.08971013	-0.049700642	3.551267768	0.443159086	NA	NA
ASV_16s_17504	0.102510304	-0.416826416	3.551230739	0.386948359	0.999764255	NA
ASV_16s_17505	0.095163999	-0.397041017	3.551286137	0.376770708	NA	NA
ASV_16s_17506	0.095163999	-0.397041017	3.551286137	0.376770708	NA	NA

ASV_16s_17507	0.095163999	-0.397041017	3.551286137	0.376770708	NA	NA
ASV_16s_17508	0.087215757	-0.371150795	3.551342889	0.35308514	NA	NA
ASV_16s_17509	0.087215757	-0.371150795	3.551342889	0.35308514	NA	NA
ASV_16s_17510	0.146439009	-0.089922185	3.550904034	0.729312438	NA	NA
ASV_16s_17511	0.172013044	1.196202961	3.550911639	0.525926233	NA	NA
ASV_16s_17512	0.172013044	1.196202961	3.550911639	0.525926233	NA	NA
ASV_16s_17513	0.069139153	0.908292139	3.551595057	0.264229758	NA	NA
ASV_16s_17514	0.171062707	1.194631358	3.550916144	0.524063086	NA	NA
ASV_16s_17515	0.171062707	1.194631358	3.550916144	0.524063086	NA	NA
ASV_16s_17516	0.156151261	-0.078456657	3.550864423	0.816519572	NA	NA
ASV_16s_17517	0.150575672	-0.078411343	3.550877317	0.799411622	0.997449953	NA
ASV_16s_17518	0.133151956	-0.078254598	3.550924514	0.742610339	NA	NA
ASV_16s_17519	0.133151956	-0.078254598	3.550924514	0.742610339	NA	NA
ASV_16s_17520	0.133151956	-0.078254598	3.550924514	0.742610339	NA	NA
ASV_16s_17521	0.133151956	-0.078254598	3.550924514	0.742610339	NA	NA
ASV_16s_17522	0.133151956	-0.078254598	3.550924514	0.742610339	NA	NA
ASV_16s_17523	0.133151956	-0.078254598	3.550924514	0.742610339	NA	NA
ASV_16s_17524	0.133151956	-0.078254598	3.550924514	0.742610339	NA	NA
ASV_16s_17525	0.133151956	-0.078254598	3.550924514	0.742610339	NA	NA
ASV_16s_17526	0.087265044	0.220183659	3.550908053	0.404207217	NA	NA
ASV_16s_17527	0.091153056	0.2201729	3.550893984	0.42119395	0.999686954	NA
ASV_16s_17528	0.092920334	0.220168346	3.550887976	0.428771398	0.999667767	NA
ASV_16s_17529	0.093273789	0.220167456	3.550886802	0.430276524	0.999663863	NA
ASV_16s_17530	0.096550865	0.220159491	3.550876318	0.444101219	0.999626515	NA
ASV_16s_17531	0.096886325	0.220158707	3.550875285	0.445499912	0.999622585	NA
ASV_16s_17532	0.099127028	0.220153644	3.550868561	0.454765038	NA	NA
ASV_16s_17533	0.099127028	0.220153644	3.550868561	0.454765038	NA	NA
ASV_16s_17534	0.098995062	0.22015392	3.550868948	0.454223438	0.999597435	NA
ASV_16s_17535	0.102091958	0.424027202	3.550819745	0.708394113	0.998270406	NA
ASV_16s_17536	0.159379566	0.310947608	3.550998209	0.806558967	NA	NA
ASV_16s_17537	0.169183233	0.310946075	3.550974568	0.835135023	NA	NA
ASV_16s_17538	0.169183233	0.310946075	3.550974568	0.835135023	NA	NA
ASV_16s_17539	0.147520101	0.310949733	3.551030978	0.770082998	NA	NA
ASV_16s_17540	0.168647729	0.310946154	3.550975789	0.833609082	NA	NA
ASV_16s_17541	0.104422501	0.595786184	3.550812893	0.41801984	NA	NA
ASV_16s_17542	0.104422501	0.595786184	3.550812893	0.41801984	NA	NA
ASV_16s_17543	0.135629548	-0.066248138	3.550935786	0.780479592	NA	NA
ASV_16s_17544	0.162850078	0.311361213	3.551947103	0.418475009	NA	NA
ASV_16s_17545	0.217238365	0.047808345	2.544430514	1.145219031	0.992134014	NA
ASV_16s_17546	0.209282003	-0.11732755	3.550580454	0.879793937	NA	NA
ASV_16s_17547	0.121942187	0.207250779	3.550975059	0.795573865	0.997488862	NA
ASV_16s_17548	0.158357936	-0.049380335	3.551411595	0.663780387	0.998598817	NA
ASV_16s_17549	0.158669947	0.789690799	3.551505744	0.404796132	0.999725846	NA
ASV_16s_17550	0.143040101	-0.04839616	3.551359658	0.706429177	0.998285849	NA
ASV_16s_17551	0.293409429	-1.078611145	3.550641741	0.562063335	0.999186011	NA
ASV_16s_17552	0.152324085	-0.06738928	3.550916305	0.943457817	0.99568981	NA
ASV_16s_17553	0.173892988	-0.517914048	3.550633222	0.642264274	0.998741097	NA
ASV_16s_17554	0.126102786	1.118955091	3.551549518	0.426693848	0.999673106	NA
ASV_16s_17555	0.196168005	1.390367034	3.5510624	0.439635554	0.999638874	NA
ASV_16s_17556	0.150421156	0.357447512	3.551706692	0.588657639	NA	NA
ASV_16s_17557	0.196285008	0.70177108	3.551134724	0.8167238	0.997269512	NA
ASV_16s_17558	0.199674167	0.742549883	3.551214532	0.943276148	NA	NA
ASV_16s_17559	0.162441099	0.363443625	3.551496822	0.658210797	NA	NA
ASV_16s_17560	0.175042393	0.163987285	3.551006186	0.843419366	0.996975183	NA
ASV_16s_17561	0.203912695	0.282811637	3.551525317	0.676883038	NA	NA
ASV_16s_17562	0.117415795	0.282965498	3.551702468	0.502578965	NA	NA
ASV_16s_17563	0.080942419	0.286504802	3.551807014	0.391777142	NA	NA
ASV_16s_17564	0.080942419	0.286504802	3.551807014	0.391777142	NA	NA
ASV_16s_17565	0.112254852	0.283404589	3.551714468	0.488217576	NA	NA
ASV_16s_17566	0.220813346	-0.553926823	3.551047224	0.709649393	NA	NA
ASV_16s_17567	0.152592835	-0.020165364	3.551245806	0.739571844	0.998012867	NA
ASV_16s_17568	0.298094943	-0.60137	3.550724775	0.810620536	NA	NA
ASV_16s_17569	0.043105185	-0.134369153	3.551796335	0.247181358	0.99994816	NA
ASV_16s_17570	0.176856101	-0.531414564	3.550525248	0.563597277	NA	NA
ASV_16s_17571	0.209645178	1.235763054	3.550716467	0.730526867	NA	NA
ASV_16s_17572	0.215450259	1.205799165	3.550433303	0.835191736	NA	NA
ASV_16s_17573	0.111744164	-0.077992831	3.5510025	0.664766192	NA	NA

ASV_16s_17574	0.111744164	-0.077992831	3.5510025	0.664766192	NA	NA
ASV_16s_17575	0.139259689	0.130479935	3.550604179	0.91293102	0.996113526	NA
ASV_16s_17576	0.309226607	-0.029196348	3.550782428	1.213509138	NA	NA
ASV_16s_17577	0.195260197	0.133384789	3.550494469	1.113467767	0.992784786	NA
ASV_16s_17578	0.24665871	-0.028996404	3.550848892	1.099564076	NA	NA
ASV_16s_17579	0.147430762	-0.028333716	3.551068941	0.848636711	NA	NA
ASV_16s_17580	0.147430762	-0.028333716	3.551068941	0.848636711	NA	NA
ASV_16s_17581	0.147430762	-0.028333716	3.551068941	0.848636711	NA	NA
ASV_16s_17582	0.147430762	-0.028333716	3.551068941	0.848636711	NA	NA
ASV_16s_17583	0.168458408	-0.073434127	3.550871137	0.876862231	NA	NA
ASV_16s_17584	0.123035217	-0.072972194	3.551002149	0.731170903	NA	NA
ASV_16s_17585	0.123035217	-0.072972194	3.551002149	0.731170903	NA	NA
ASV_16s_17586	0.12216432	-0.072960028	3.551005605	0.728013194	0.998111116	NA
ASV_16s_17587	0.12082448	-0.072940973	3.551011018	0.723121733	NA	NA
ASV_16s_17588	0.12082448	-0.072940973	3.551011018	0.723121733	NA	NA
ASV_16s_17589	0.12082448	-0.072940973	3.551011018	0.723121733	NA	NA
ASV_16s_17590	0.12082448	-0.072940973	3.551011018	0.723121733	NA	NA
ASV_16s_17591	0.149405963	0.292102814	3.550842894	0.914122362	NA	NA
ASV_16s_17592	0.172790813	0.362999877	3.550816697	0.976372225	0.995200733	NA
ASV_16s_17593	0.17538913	0.369714438	3.55085389	0.973669298	0.995242177	NA
ASV_16s_17594	0.198234652	0.783579165	3.55073989	0.759917161	0.997831821	NA
ASV_16s_17595	0.219495987	0.360722809	3.550529737	1.107113	0.992910799	NA
ASV_16s_17596	0.205844503	0.359003423	3.550578494	1.065497518	NA	NA
ASV_16s_17597	0.168177583	0.360290559	3.5511966	0.893953071	NA	NA
ASV_16s_17598	0.168177583	0.360290559	3.5511966	0.893953071	NA	NA
ASV_16s_17599	0.168177583	0.360290559	3.5511966	0.893953071	NA	NA
ASV_16s_17600	0.168177583	0.360290559	3.5511966	0.893953071	NA	NA
ASV_16s_17601	0.168177583	0.360290559	3.5511966	0.893953071	NA	NA
ASV_16s_17602	0.149504061	0.360267814	3.551271127	0.837990231	NA	NA
ASV_16s_17603	0.149504061	0.360267814	3.551271127	0.837990231	NA	NA
ASV_16s_17604	0.149504061	0.360267814	3.551271127	0.837990231	NA	NA
ASV_16s_17605	0.149504061	0.360267814	3.551271127	0.837990231	NA	NA
ASV_16s_17606	0.149504061	0.360267814	3.551271127	0.837990231	NA	NA
ASV_16s_17607	0.149504061	0.360267814	3.551271127	0.837990231	NA	NA
ASV_16s_17608	0.149504061	0.360267814	3.551271127	0.837990231	NA	NA
ASV_16s_17609	0.149504061	0.360267814	3.551271127	0.837990231	NA	NA
ASV_16s_17610	0.149504061	0.360267814	3.551271127	0.837990231	NA	NA
ASV_16s_17611	0.149504061	0.360267814	3.551271127	0.837990231	NA	NA
ASV_16s_17612	0.139496401	0.418573722	3.551275987	1.009114544	0.994680163	NA
ASV_16s_17613	0.182359592	0.583848216	3.551097261	1.298317023	0.988484288	NA
ASV_16s_17614	0.273992219	0.360361411	3.550965017	1.136391986	NA	NA
ASV_16s_17615	0.193711602	0.315939774	3.550957565	0.923908972	NA	NA
ASV_16s_17616	0.185751636	0.315939701	3.550973342	0.903318335	0.996241121	NA
ASV_16s_17617	0.168671375	0.31593975	3.551012187	0.856451613	0.996824262	NA
ASV_16s_17618	0.167533182	0.315939753	3.551015054	0.853195589	0.996862419	NA
ASV_16s_17619	0.170159142	0.315939745	3.551008493	0.860695107	0.996774079	NA
ASV_16s_17620	0.165256798	0.315939761	3.551020906	0.84651921	0.996939719	NA
ASV_16s_17621	0.202268798	0.898527465	3.550807674	1.013037686	0.99461547	NA
ASV_16s_17622	0.167443717	0.315939861	3.551015272	0.852969248	NA	NA
ASV_16s_17623	0.167443717	0.315939861	3.551015272	0.852969248	NA	NA
ASV_16s_17624	0.259955883	0.315939577	3.550863597	1.070865851	NA	NA
ASV_16s_17625	0.156151261	0.315939909	3.551046004	0.819651121	NA	NA
ASV_16s_17626	0.156151261	0.315939909	3.551046004	0.819651121	NA	NA
ASV_16s_17627	0.181210484	0.315939713	3.550983034	0.888330946	0.996434579	NA
ASV_16s_17628	0.185387021	0.315939701	3.550974176	0.899690073	0.996288565	NA
ASV_16s_17629	0.210250038	0.596119474	3.550680789	0.732536649	NA	NA
ASV_16s_17630	0.210250038	0.596119474	3.550680789	0.732536649	NA	NA
ASV_16s_17631	0.148708629	0.687488948	3.551000164	0.71173801	NA	NA
ASV_16s_17632	0.148708629	0.687488948	3.551000164	0.71173801	NA	NA
ASV_16s_17633	0.261830065	1.11263332	3.55090824	0.835765662	0.99706159	NA
ASV_16s_17634	0.230314118	1.12502983	3.550979188	0.757590175	0.997853059	NA
ASV_16s_17635	0.214081194	0.73387753	3.551077542	0.949126976	0.99560798	NA
ASV_16s_17636	0.215856426	1.105514586	3.550861927	0.709839511	0.998258987	NA
ASV_16s_17637	0.182805579	1.06200959	3.5510241	0.648200006	NA	NA
ASV_16s_17638	0.244110512	0.734088855	3.551016192	1.015160845	NA	NA
ASV_16s_17639	0.20656893	0.73381529	3.551095625	0.932489243	NA	NA
ASV_16s_17640	0.20656893	0.73381529	3.551095625	0.932489243	NA	NA

ASV_16s_17641	0.20656893	0.73381529	3.551095625	0.932489243	NA	NA
ASV_16s_17642	0.20656893	0.73381529	3.551095625	0.932489243	NA	NA
ASV_16s_17643	0.20656893	0.73381529	3.551095625	0.932489243	NA	NA
ASV_16s_17644	0.20656893	0.73381529	3.551095625	0.932489243	NA	NA
ASV_16s_17645	0.201286476	1.106147419	3.551080107	0.699415549	0.998340223	NA
ASV_16s_17646	0.169901535	1.018240421	3.551087234	0.631337174	NA	NA
ASV_16s_17647	0.19204218	0.69286134	3.550938	0.856381326	NA	NA
ASV_16s_17648	0.172465315	0.692713376	3.550981491	0.804977207	0.997392827	NA
ASV_16s_17649	0.195179535	0.692882319	3.550931838	0.864229089	0.996731895	NA
ASV_16s_17650	0.188040521	0.692831361	3.550946182	0.844935469	0.996957872	NA
ASV_16s_17651	0.19749127	0.692897358	3.550927422	0.869950713	NA	NA
ASV_16s_17652	0.185753664	0.692814941	3.550950994	0.839559853	0.99701896	NA
ASV_16s_17653	0.156151261	0.692562196	3.551025977	0.758672677	NA	NA
ASV_16s_17654	0.156151261	0.692562196	3.551025977	0.758672677	NA	NA
ASV_16s_17655	0.156151261	0.692562196	3.551025977	0.758672677	NA	NA
ASV_16s_17656	0.156151261	0.692562196	3.551025977	0.758672677	NA	NA
ASV_16s_17657	0.156151261	0.692562196	3.551025977	0.758672677	NA	NA
ASV_16s_17658	0.156151261	0.692562196	3.551025977	0.758672677	NA	NA
ASV_16s_17659	0.173907187	0.692725318	3.550977979	0.806898563	0.997372912	NA
ASV_16s_17660	0.229394456	0.693074269	3.550875519	0.94357669	NA	NA
ASV_16s_17661	0.261203359	1.473846299	3.550721282	0.571484787	0.999140365	NA
ASV_16s_17662	0.220299813	0.280119618	3.55142969	0.702890342	NA	NA
ASV_16s_17663	0.13054789	0.295318371	3.551833642	0.527957813	NA	NA
ASV_16s_17664	0.13054789	0.295318371	3.551833642	0.527957813	NA	NA
ASV_16s_17665	0.13054789	0.295318371	3.551833642	0.527957813	NA	NA
ASV_16s_17666	0.13054789	0.295318371	3.551833642	0.527957813	NA	NA
ASV_16s_17667	0.13054789	0.295318371	3.551833642	0.527957813	NA	NA
ASV_16s_17668	0.13054789	0.295318371	3.551833642	0.527957813	NA	NA
ASV_16s_17669	0.13054789	0.295318371	3.551833642	0.527957813	NA	NA
ASV_16s_17670	0.13054789	0.295318371	3.551833642	0.527957813	NA	NA
ASV_16s_17671	0.13054789	0.295318371	3.551833642	0.527957813	NA	NA
ASV_16s_17672	0.13054789	0.295318371	3.551833642	0.527957813	NA	NA
ASV_16s_17673	0.13054789	0.295318371	3.551833642	0.527957813	NA	NA
ASV_16s_17674	0.13054789	0.295318371	3.551833642	0.527957813	NA	NA
ASV_16s_17675	0.13054789	0.295318371	3.551833642	0.527957813	NA	NA
ASV_16s_17676	0.13054789	0.295318371	3.551833642	0.527957813	NA	NA
ASV_16s_17677	0.13054789	0.295318371	3.551833642	0.527957813	NA	NA
ASV_16s_17678	0.13054789	0.295318371	3.551833642	0.527957813	NA	NA
ASV_16s_17679	0.130593523	0.031881692	3.551521343	0.753223952	0.997892536	NA
ASV_16s_17680	0.132619241	0.020253713	3.55149206	0.763651118	0.997797453	NA
ASV_16s_17681	0.149833854	-0.087843479	3.551213752	0.87440287	0.996608448	NA
ASV_16s_17682	0.133137079	-0.032573041	3.551417304	0.801783434	0.99742571	NA
ASV_16s_17683	0.133654916	-0.08183718	3.551353047	0.826385327	0.997165265	NA
ASV_16s_17684	0.132642057	-0.077016748	3.55136705	0.821752741	0.99721557	NA
ASV_16s_17685	0.137529814	-0.09960881	3.551302156	0.844349556	0.996964569	NA
ASV_16s_17686	0.133913835	-0.105462313	3.551323803	0.834126501	0.997079883	NA
ASV_16s_17687	0.133913835	-0.105462313	3.551323803	0.834126501	0.997079883	NA
ASV_16s_17688	0.158405394	-0.193041463	3.551030673	0.94909938	0.99560838	NA
ASV_16s_17689	0.132653465	-0.121840475	3.551315995	0.833316618	0.997088893	NA
ASV_16s_17690	0.134172754	-0.128392203	3.551297427	0.839749072	0.997016823	NA
ASV_16s_17691	0.134172754	-0.128392203	3.551297427	0.839749072	0.997016823	NA
ASV_16s_17692	0.130633451	-0.134938445	3.551318826	0.827949014	0.997148152	NA
ASV_16s_17693	0.130633451	-0.134938445	3.551318826	0.827949014	0.997148152	NA
ASV_16s_17694	0.130633451	-0.134938445	3.551318826	0.827949014	0.997148152	NA
ASV_16s_17695	0.134431673	-0.150757225	3.551273832	0.843258919	0.996977011	NA
ASV_16s_17696	0.130639155	-0.156568153	3.551298615	0.8291108	0.997135394	NA
ASV_16s_17697	0.134949511	-0.194233552	3.55123422	0.8441053	0.996967359	NA
ASV_16s_17698	0.134949511	-0.194233552	3.55123422	0.8441053	0.996967359	NA
ASV_16s_17699	0.13520843	-0.215467298	3.551217773	0.841786281	0.996993757	NA
ASV_16s_17700	0.13520843	-0.215467298	3.551217773	0.841786281	0.996993757	NA
ASV_16s_17701	0.130650564	-0.198865266	3.551265135	0.825860448	0.997170995	NA
ASV_16s_17702	0.130650564	-0.198865266	3.551265135	0.825860448	0.997170995	NA
ASV_16s_17703	0.130656268	-0.219670031	3.551251598	0.821750406	0.997215595	NA
ASV_16s_17704	0.14218443	-0.277385668	3.551126128	0.852352706	0.996872247	NA
ASV_16s_17705	0.134206979	-0.252474961	3.551204273	0.826961586	0.997158967	NA
ASV_16s_17706	0.130667676	-0.261332334	3.551228173	0.808272624	0.997358609	NA
ASV_16s_17707	0.130667676	-0.261332334	3.551228173	0.808272624	0.997358609	NA

ASV_16s_17708	0.130679084	-0.303438236	3.551210553	0.780156127	0.997641232	NA
ASV_16s_17709	0.046669299	-0.169333869	3.551822392	0.270931686	0.999929185	NA
ASV_16s_17710	0.046669299	-0.169333869	3.551822392	0.270931686	0.999929185	NA
ASV_16s_17711	0.178108473	-0.600493181	3.550890295	0.650062256	NA	NA
ASV_16s_17712	0.183680583	-0.60933364	3.550858884	0.660659437	NA	NA
ASV_16s_17713	0.163271815	-0.574145388	3.550974996	0.620322058	NA	NA
ASV_16s_17714	0.163271815	-0.574145388	3.550974996	0.620322058	NA	NA
ASV_16s_17715	0.163271815	-0.574145388	3.550974996	0.620322058	NA	NA
ASV_16s_17716	0.159499413	-0.566832017	3.550997846	0.612333184	0.998922442	NA
ASV_16s_17717	0.154332883	-0.556559466	3.551030378	0.60122885	0.99898498	NA
ASV_16s_17718	0.145291456	-0.537785575	3.551091586	0.580645037	NA	NA
ASV_16s_17719	0.138833293	-0.52272634	3.551134132	0.566130258	NA	NA
ASV_16s_17720	0.138833293	-0.52272634	3.551134132	0.566130258	NA	NA
ASV_16s_17721	0.138833293	-0.52272634	3.551134132	0.566130258	NA	NA
ASV_16s_17722	0.138833293	-0.52272634	3.551134132	0.566130258	NA	NA
ASV_16s_17723	0.136047931	-0.515772963	3.551151834	0.559438595	0.999198429	NA
ASV_16s_17724	0.136047931	-0.515772963	3.551151834	0.559438595	0.999198429	NA
ASV_16s_17725	0.15955692	-0.566920983	3.550997567	0.610125652	0.998935075	NA
ASV_16s_17726	0.135288286	-0.513865164	3.551156681	0.557591045	0.999207093	NA
ASV_16s_17727	0.13073042	-0.502207268	3.551186673	0.546338149	NA	NA
ASV_16s_17728	0.13073042	-0.502207268	3.551186673	0.546338149	NA	NA
ASV_16s_17729	0.13073042	-0.502207268	3.551186673	0.546338149	NA	NA
ASV_16s_17730	0.13073042	-0.502207268	3.551186673	0.546338149	NA	NA
ASV_16s_17731	0.13073042	-0.502207268	3.551186673	0.546338149	NA	NA
ASV_16s_17732	0.13073042	-0.502207268	3.551186673	0.546338149	NA	NA
ASV_16s_17733	0.13073042	-0.502207268	3.551186673	0.546338149	NA	NA
ASV_16s_17734	0.13073042	-0.502207268	3.551186673	0.546338149	NA	NA
ASV_16s_17735	0.13073042	-0.502207268	3.551186673	0.546338149	NA	NA
ASV_16s_17736	0.13073042	-0.502207268	3.551186673	0.546338149	NA	NA
ASV_16s_17737	0.13073042	-0.502207268	3.551186673	0.546338149	NA	NA
ASV_16s_17738	0.162788133	-0.573169322	3.55097811	0.616459766	0.998898558	NA
ASV_16s_17739	0.036945229	-0.151074632	3.551144043	0.157812673	NA	NA
ASV_16s_17740	0.084620649	0.220191429	3.550918382	0.392351019	NA	NA
ASV_16s_17741	0.142529012	0.027395163	3.55138147	0.792106847	0.997523673	NA
ASV_16s_17742	0.13799762	-0.086609943	3.551291355	0.756019874	0.997867312	NA
ASV_16s_17743	0.190447384	-0.815752365	3.550737049	0.543021421	0.99927321	NA
ASV_16s_17744	0.141517934	0.151648352	3.551412782	0.762692887	0.997806307	NA
ASV_16s_17745	0.133696405	-0.012078344	3.551306043	0.688566232	0.998422054	NA
ASV_16s_17746	0.13373063	-0.18540627	3.551204502	0.547304957	NA	NA
ASV_16s_17747	0.137971076	-0.156295594	3.551555481	0.707979972	0.998273668	NA
ASV_16s_17748	0.182620033	-0.605396196	3.550793893	0.734729593	0.998054428	NA
ASV_16s_17749	0.162979826	-0.547708819	3.550945513	0.696603303	0.9983617	NA
ASV_16s_17750	0.172283581	-0.560524741	3.550828933	0.732549162	0.998072953	NA
ASV_16s_17751	0.151127151	-0.508444366	3.551053065	0.667401643	0.998573862	NA
ASV_16s_17752	0.156952434	-0.528588041	3.550999307	0.682104671	NA	NA
ASV_16s_17753	0.179273902	-0.552531724	3.550737469	0.786049358	0.997583731	NA
ASV_16s_17754	0.173877457	-0.533891085	3.550783001	0.771518295	0.99772387	NA
ASV_16s_17755	0.139026943	0.668387867	3.551651716	0.662679784	0.998606344	NA
ASV_16s_17756	0.173524873	-0.515947279	3.550785512	0.78439326	0.997599982	NA
ASV_16s_17757	0.171919659	-0.517728164	3.550799136	0.775817878	0.997682979	NA
ASV_16s_17758	0.175708286	-0.52751065	3.550766408	0.788938109	0.997555211	NA
ASV_16s_17759	0.164715607	-0.482159041	3.550853191	0.759531003	0.997835355	NA
ASV_16s_17760	0.184819981	0.225686025	3.55100936	0.643819756	0.998731151	NA
ASV_16s_17761	0.160231859	-0.045161117	3.550849499	0.749476944	0.997926029	NA
ASV_16s_17762	0.116896707	0.059259065	3.551322483	0.437969576	0.999643412	NA
ASV_16s_17763	0.170019203	-0.778580089	3.55093627	0.442814061	0.999630107	NA
ASV_16s_17764	0.164937729	-0.439824737	3.550833629	0.780806893	0.997634927	NA
ASV_16s_17765	0.163310189	-0.445341876	3.550849776	0.774116016	0.997699222	NA
ASV_16s_17766	0.158501729	-0.840469832	3.550887583	0.324064988	0.999870159	NA
ASV_16s_17767	0.158638288	-0.392771681	3.550886553	0.768455651	0.997752706	NA
ASV_16s_17768	0.219091497	-0.040218032	3.550656693	1.003677107	0.994769001	NA
ASV_16s_17769	0.1730693	-0.410532858	3.550781554	0.824538564	0.99718539	NA
ASV_16s_17770	0.146173782	-0.667485688	3.55091224	0.264030998	0.999935126	NA
ASV_16s_17771	0.125434724	-0.447686859	3.55073662	0.503050305	0.999435236	NA
ASV_16s_17772	0.193583557	0.722177655	3.551326504	0.921206455	0.996001459	NA
ASV_16s_17773	0.140845182	0.865308965	3.551421759	0.416506211	0.999698435	NA
ASV_16s_17774	0.158838497	-0.348068553	3.551384348	0.532256239	0.999319591	NA

ASV_16s_17775	0.162199202	-0.342001648	3.550924785	0.812548506	0.997313773	NA
ASV_16s_17776	0.171754164	0.109472174	3.550887124	0.806557181	0.997376458	NA
ASV_16s_17777	0.170005	-0.285260738	3.550951742	0.818748173	0.997247881	NA
ASV_16s_17778	0.155101216	-0.285260738	3.550951742	0.766842748	0.997767794	NA
ASV_16s_17779	0.171294449	-0.285289849	3.550875098	0.824375106	0.997187167	NA
ASV_16s_17780	0.173400207	0.136291505	3.55088776	0.808607664	0.997355114	NA
ASV_16s_17781	0.145335876	-0.666105263	3.551018234	0.472012505	0.999542644	NA
ASV_16s_17782	0.16945233	0.183115455	3.549931202	0.76861783	0.997751185	NA
ASV_16s_17783	0.168513066	0.33702619	3.551766278	0.548828066	0.999247326	NA
ASV_16s_17784	0.175746814	-0.224665565	3.550905212	0.83874601	0.997028138	NA
ASV_16s_17785	0.161435622	-0.224653507	3.550940023	0.790058699	0.997544089	NA
ASV_16s_17786	0.213996443	0.586913955	3.55082828	1.045883228	0.994054032	NA
ASV_16s_17787	0.16957575	-0.1657243	3.55093397	0.817043967	NA	NA
ASV_16s_17788	0.21096051	-0.467024641	3.550871974	0.737430845	NA	NA
ASV_16s_17789	0.164113509	-0.071298006	3.551260702	0.688964759	0.998419097	NA
ASV_16s_17790	0.165537282	0.77049452	3.551406189	0.401699888	0.999732797	NA
ASV_16s_17791	0.162894903	0.167677154	3.550302051	0.692786737	NA	NA
ASV_16s_17792	0.147505996	0.311372021	3.551285801	0.593051598	NA	NA
ASV_16s_17793	0.197254998	1.364001436	3.551000363	0.460775605	0.999577809	NA
ASV_16s_17794	0.168886239	1.09527561	3.551329673	0.657571862	0.998640921	NA
ASV_16s_17795	0.177211882	0.690097482	3.550989308	0.801371443	0.997429932	NA
ASV_16s_17796	0.151379421	0.021432261	3.551268566	0.558728455	NA	NA
ASV_16s_17797	0.22133134	0.280032386	3.551423378	0.704462519	NA	NA
ASV_16s_17798	0.167199804	-0.769438777	3.551268107	0.333134955	0.999857489	NA
ASV_16s_17799	0.17781593	1.482391673	3.550855066	0.351451089	0.999829339	NA
ASV_16s_17800	0.162855432	1.148629476	3.551190488	0.566679943	NA	NA
ASV_16s_17801	0.174737037	0.804261946	3.551071496	0.584329988	0.999075392	NA
ASV_16s_17802	0.149419651	1.119247382	3.551263107	0.537671342	NA	NA
ASV_16s_17803	0.146806697	1.377984128	3.551119477	0.307470146	0.999891284	NA
ASV_16s_17804	0.141660097	0.347399626	3.55184348	0.555591971	NA	NA
ASV_16s_17805	0.150846445	0.596521994	3.55181164	0.494406103	NA	NA
ASV_16s_17806	0.1679455	0.358018793	3.551705366	0.611731383	0.998925896	NA
ASV_16s_17807	0.171496825	0.359802398	3.551685701	0.618754776	NA	NA
ASV_16s_17808	0.201600426	0.86704581	3.551271419	0.662889443	0.998604912	NA
ASV_16s_17809	0.109778114	0.336534418	3.55194548	0.475100278	NA	NA
ASV_16s_17810	0.091824735	-0.189040413	3.551729587	0.373569043	0.999790494	NA
ASV_16s_17811	0.111369652	0.449433719	3.550960512	0.311463413	0.999886437	NA
ASV_16s_17812	0.21140244	0.333295139	3.551916895	0.525213077	0.999348824	NA
ASV_16s_17813	0.195793478	-0.480765224	3.551167413	0.629889962	0.998818367	NA
ASV_16s_17814	0.183680583	-0.534461719	3.550786439	0.643895742	NA	NA
ASV_16s_17815	0.183680583	-0.534461719	3.550786439	0.643895742	NA	NA
ASV_16s_17816	0.183680583	-0.534461719	3.550786439	0.643895742	NA	NA
ASV_16s_17817	0.161673463	-0.463796045	3.551284358	0.540968465	NA	NA
ASV_16s_17818	0.125713979	0.114227124	3.551375171	0.430566784	NA	NA
ASV_16s_17819	0.097161727	-0.592764501	3.551390474	0.241555577	0.999952069	NA
ASV_16s_17820	0.143507393	-0.489599735	3.551181766	0.564944543	NA	NA
ASV_16s_17821	0.143507393	-0.489599735	3.551181766	0.564944543	NA	NA
ASV_16s_17822	0.197733523	1.209658828	3.551017088	0.63351317	NA	NA
ASV_16s_17823	0.084835543	0.970157374	3.551457767	0.312854695	NA	NA
ASV_16s_17824	0.084835543	0.970157374	3.551457767	0.312854695	NA	NA
ASV_16s_17825	0.233175237	1.273933318	3.550655813	0.630334722	NA	NA
ASV_16s_17826	0.145438007	1.145898414	3.551045269	0.470054661	NA	NA
ASV_16s_17827	0.157636923	-0.044522743	3.551296899	0.603419755	NA	NA
ASV_16s_17828	0.122902012	-0.019828184	3.55110752	0.68809975	0.998425511	NA
ASV_16s_17829	0.183837428	0.385232645	3.551248442	0.576340742	NA	NA
ASV_16s_17830	0.166768987	0.377465785	3.551354899	0.544399674	NA	NA
ASV_16s_17831	0.194394439	-0.047149946	3.550308499	1.01564172	0.994572252	NA
ASV_16s_17832	0.176103592	0.759194846	3.550803593	0.881470914	0.996520922	NA
ASV_16s_17833	0.124994931	0.358676083	3.551582777	0.452622169	NA	NA
ASV_16s_17834	0.124994931	0.358676083	3.551582777	0.452622169	NA	NA
ASV_16s_17835	0.124994931	0.358676083	3.551582777	0.452622169	NA	NA
ASV_16s_17836	0.135240085	0.363262855	3.551535141	0.477100571	NA	NA
ASV_16s_17837	0.047089444	0.341202862	3.551882322	0.211003956	NA	NA
ASV_16s_17838	0.047089444	0.341202862	3.551882322	0.211003956	NA	NA
ASV_16s_17839	0.047089444	0.341202862	3.551882322	0.211003956	NA	NA
ASV_16s_17840	0.047089444	0.341202862	3.551882322	0.211003956	NA	NA
ASV_16s_17841	0.189723289	0.388386035	3.551208949	0.586654675	NA	NA

ASV_16s_17842	0.162192372	-0.072301637	3.550904336	0.74137546	NA	NA
ASV_16s_17843	0.075983519	-0.330861586	3.551433025	0.317497347	NA	NA
ASV_16s_17844	0.075983519	-0.330861586	3.551433025	0.317497347	NA	NA
ASV_16s_17845	0.186394035	-0.535564597	3.550441083	0.580114876	NA	NA
ASV_16s_17846	0.092280241	-0.387926199	3.551306118	0.368302564	NA	NA
ASV_16s_17847	0.067044027	0.899294426	3.551614295	0.257408635	NA	NA
ASV_16s_17848	0.14863161	-0.078395864	3.550882064	0.79326104	0.99751212	NA
ASV_16s_17849	0.129117049	-0.078211925	3.550937279	0.728563325	NA	NA
ASV_16s_17850	0.129117049	-0.078211925	3.550937279	0.728563325	NA	NA
ASV_16s_17851	0.129117049	-0.078211925	3.550937279	0.728563325	NA	NA
ASV_16s_17852	0.129117049	-0.078211925	3.550937279	0.728563325	NA	NA
ASV_16s_17853	0.129117049	-0.078211925	3.550937279	0.728563325	NA	NA
ASV_16s_17854	0.129117049	-0.078211925	3.550937279	0.728563325	NA	NA
ASV_16s_17855	0.089215572	0.22017814	3.550900868	0.412718225	0.999707499	NA
ASV_16s_17856	0.090982849	0.220173389	3.550894599	0.420395043	0.999688932	NA
ASV_16s_17857	0.092043216	0.220170625	3.550890953	0.424958271	0.99967752	NA
ASV_16s_17858	0.096123179	0.22016057	3.550877667	0.442253488	NA	NA
ASV_16s_17859	0.096123179	0.22016057	3.550877667	0.442253488	NA	NA
ASV_16s_17860	0.164975942	0.31094664	3.5509844	0.82294987	0.997202627	NA
ASV_16s_17861	0.163537192	0.310946946	3.550987869	0.818756799	NA	NA
ASV_16s_17862	0.101258183	0.595764239	3.550821095	0.405884848	NA	NA
ASV_16s_17863	0.101258183	0.595764239	3.550821095	0.405884848	NA	NA
ASV_16s_17864	0.101258183	0.595764239	3.550821095	0.405884848	NA	NA
ASV_16s_17865	0.156811247	-0.387571816	3.551117427	0.573594599	0.999129912	NA
ASV_16s_17866	0.210615024	0.907776233	3.551031418	0.623728234	0.998855628	NA
ASV_16s_17867	0.133642784	-0.065739015	3.550951303	0.787621129	0.997568241	NA
ASV_16s_17868	0.202221006	0.40308011	3.550767634	1.031849387	NA	NA
ASV_16s_17869	0.162498321	1.09918397	3.551054684	0.687190181	0.998432237	NA
ASV_16s_17870	0.136054305	-0.066957257	3.551007162	0.872327387	0.996633874	NA
ASV_16s_17871	0.136054305	-0.066957257	3.551007162	0.872327387	0.996633874	NA
ASV_16s_17872	0.163307654	0.322607411	3.551850993	0.497328174	0.999456206	NA
ASV_16s_17873	0.137045342	-0.066705972	3.551047755	0.877493395	0.996570356	NA
ASV_16s_17874	0.231689115	-1.115406765	3.550415788	0.4577019	0.999587095	NA
ASV_16s_17875	0.120557893	0.127675468	3.551556714	0.633094982	0.998798668	NA
ASV_16s_17876	0.152181191	0.366960811	3.551729671	0.518526806	NA	NA
ASV_16s_17877	0.153826648	0.319958492	3.551274612	0.632054606	NA	NA
ASV_16s_17878	0.168950605	0.319175413	3.551158156	0.693192771	0.998387495	NA
ASV_16s_17879	0.146339393	0.361867947	3.551400274	0.727662238	NA	NA
ASV_16s_17880	0.182321652	0.279373349	3.551587362	0.65449722	NA	NA
ASV_16s_17881	0.182321652	0.279373349	3.551587362	0.65449722	NA	NA
ASV_16s_17882	0.294919531	-0.563857709	3.550055902	0.796243119	NA	NA
ASV_16s_17883	0.294919531	-0.563857709	3.550055902	0.796243119	NA	NA
ASV_16s_17884	0.235788115	0.116713324	3.551012865	1.040881963	0.994141821	NA
ASV_16s_17885	0.178325678	0.384456824	3.55106659	0.784028408	NA	NA
ASV_16s_17886	0.189939052	0.739883361	3.551383006	0.761483991	0.997817443	NA
ASV_16s_17887	0.171496825	-0.528958254	3.550576762	0.553909316	NA	NA
ASV_16s_17888	0.171496825	-0.528958254	3.550576762	0.553909316	NA	NA
ASV_16s_17889	0.180473024	1.201501037	3.550843471	0.624025644	NA	NA
ASV_16s_17890	0.163133176	0.001197514	3.550973883	0.863845791	0.996736488	NA
ASV_16s_17891	0.299856104	-0.029171661	3.550790644	1.197845567	NA	NA
ASV_16s_17892	0.239184204	-0.028964264	3.550859169	1.084045267	NA	NA
ASV_16s_17893	0.231691871	-0.131103338	3.55087835	0.987100819	NA	NA
ASV_16s_17894	0.142963163	-0.028282651	3.55108601	0.834248198	NA	NA
ASV_16s_17895	0.142963163	-0.028282651	3.55108601	0.834248198	NA	NA
ASV_16s_17896	0.216205398	0.57961204	3.550999642	1.269247535	0.989244131	NA
ASV_16s_17897	0.173747387	0.265992905	3.550734388	0.985937593	NA	NA
ASV_16s_17898	0.179521708	0.320187411	3.55061861	1.036312189	0.99422131	NA
ASV_16s_17899	0.18529603	0.343928722	3.55059709	1.033861702	0.994263649	NA
ASV_16s_17900	0.119306877	-0.072918985	3.551017333	0.717453307	NA	NA
ASV_16s_17901	0.119306877	-0.072918985	3.551017333	0.717453307	NA	NA
ASV_16s_17902	0.117163132	-0.072886815	3.551026475	0.709463243	NA	NA
ASV_16s_17903	0.117163132	-0.072886815	3.551026475	0.709463243	NA	NA
ASV_16s_17904	0.117163132	-0.072886815	3.551026475	0.709463243	NA	NA
ASV_16s_17905	0.117163132	-0.072886815	3.551026475	0.709463243	NA	NA
ASV_16s_17906	0.117163132	-0.072886815	3.551026475	0.709463243	NA	NA
ASV_16s_17907	0.166531149	0.361572669	3.550852456	0.951381032	0.995575167	NA
ASV_16s_17908	0.169129466	0.368360538	3.550886532	0.949659333	0.995600244	NA

ASV_16s_17909	0.211235533	0.330077247	3.550575646	1.023010184	0.994448761	NA
ASV_16s_17910	0.210071207	0.517170373	3.550580018	0.887456318	0.996445664	NA
ASV_16s_17911	0.199584838	0.357852466	3.550603423	1.043881964	NA	NA
ASV_16s_17912	0.145371417	-0.073235088	3.550927595	0.80710834	NA	NA
ASV_16s_17913	0.18135271	0.289031016	3.550309104	0.985222348	NA	NA
ASV_16s_17914	0.163081293	0.360284859	3.551215309	0.879100299	NA	NA
ASV_16s_17915	0.163081293	0.360284859	3.551215309	0.879100299	NA	NA
ASV_16s_17916	0.163081293	0.360284859	3.551215309	0.879100299	NA	NA
ASV_16s_17917	0.163081293	0.360284859	3.551215309	0.879100299	NA	NA
ASV_16s_17918	0.1684976	0.362822749	3.551592945	0.693849357	NA	NA
ASV_16s_17919	0.157250349	0.341585107	3.551622295	0.631008647	0.998811516	NA
ASV_16s_17920	0.144973635	0.360261426	3.551292121	0.823515174	NA	NA
ASV_16s_17921	0.144973635	0.360261426	3.551292121	0.823515174	NA	NA
ASV_16s_17922	0.144973635	0.360261426	3.551292121	0.823515174	NA	NA
ASV_16s_17923	0.144973635	0.360261426	3.551292121	0.823515174	NA	NA
ASV_16s_17924	0.144973635	0.360261426	3.551292121	0.823515174	NA	NA
ASV_16s_17925	0.144973635	0.360261426	3.551292121	0.823515174	NA	NA
ASV_16s_17926	0.187841553	0.315939799	3.550969097	0.90871523	NA	NA
ASV_16s_17927	0.187841553	0.315939799	3.550969097	0.90871523	NA	NA
ASV_16s_17928	0.169630479	0.31593977	3.551009832	0.859090271	0.996793118	NA
ASV_16s_17929	0.163374112	0.315939792	3.551025902	0.840761045	0.99700538	NA
ASV_16s_17930	0.163150901	0.315939792	3.551026496	0.84017648	0.997011994	NA
ASV_16s_17931	0.162369665	0.315939892	3.551028588	0.838134942	NA	NA
ASV_16s_17932	0.198813851	0.315939692	3.550948183	0.935147683	0.995807972	NA
ASV_16s_17933	0.181796404	0.487177346	3.550939182	1.283863852	0.988866073	NA
ASV_16s_17934	0.156210143	0.315939818	3.551045866	0.819738284	0.997237261	NA
ASV_16s_17935	0.154841361	0.315939824	3.55104989	0.815572639	0.997281763	NA
ASV_16s_17936	0.15415697	0.315939826	3.551051929	0.813478524	0.997303955	NA
ASV_16s_17937	0.182875351	0.315939731	3.550979464	0.892804274	0.996377532	NA
ASV_16s_17938	0.151419405	0.315939942	3.551060269	0.805028247	NA	NA
ASV_16s_17939	0.151419405	0.315939942	3.551060269	0.805028247	NA	NA
ASV_16s_17940	0.151419405	0.315939942	3.551060269	0.805028247	NA	NA
ASV_16s_17941	0.151419405	0.467615966	3.551009816	1.134301715	0.992361772	NA
ASV_16s_17942	0.144202306	0.687445838	3.551014029	0.697766108	NA	NA
ASV_16s_17943	0.144202306	0.687445838	3.551014029	0.697766108	NA	NA
ASV_16s_17944	0.260511553	0.688084927	3.550809621	0.982324061	NA	NA
ASV_16s_17945	0.241698338	1.035105881	3.550737595	0.749292856	NA	NA
ASV_16s_17946	0.222885123	1.14113511	3.55084104	0.701823329	0.998321688	NA
ASV_16s_17947	0.236713223	0.734041757	3.551029899	0.999729233	NA	NA
ASV_16s_17948	0.236713223	0.734041757	3.551029899	0.999729233	NA	NA
ASV_16s_17949	0.236713223	0.734041757	3.551029899	0.999729233	NA	NA
ASV_16s_17950	0.215098374	0.733885701	3.551075209	0.951788788	0.995569215	NA
ASV_16s_17951	0.200309266	0.733759852	3.55111178	0.917357576	NA	NA
ASV_16s_17952	0.200309266	0.733759852	3.55111178	0.917357576	NA	NA
ASV_16s_17953	0.200309266	0.733759852	3.55111178	0.917357576	NA	NA
ASV_16s_17954	0.200309266	0.733759852	3.55111178	0.917357576	NA	NA
ASV_16s_17955	0.200309266	0.733759852	3.55111178	0.917357576	NA	NA
ASV_16s_17956	0.200309266	0.733759852	3.55111178	0.917357576	NA	NA
ASV_16s_17957	0.200309266	0.733759852	3.55111178	0.917357576	NA	NA
ASV_16s_17958	0.200309266	0.733759852	3.55111178	0.917357576	NA	NA
ASV_16s_17959	0.200309266	0.733759852	3.55111178	0.917357576	NA	NA
ASV_16s_17960	0.200309266	0.733759852	3.55111178	0.917357576	NA	NA
ASV_16s_17961	0.200309266	0.733759852	3.55111178	0.917357576	NA	NA
ASV_16s_17962	0.196768115	0.801923483	3.550952392	0.769516609	0.997742744	NA
ASV_16s_17963	0.099224329	0.707717792	3.551542675	0.603959546	NA	NA
ASV_16s_17964	0.099224329	0.707717792	3.551542675	0.603959546	NA	NA
ASV_16s_17965	0.099224329	0.707717792	3.551542675	0.603959546	NA	NA
ASV_16s_17966	0.099224329	0.707717792	3.551542675	0.603959546	NA	NA
ASV_16s_17967	0.18622272	0.692820656	3.550950006	0.841476968	NA	NA
ASV_16s_17968	0.178250651	0.692758657	3.550967682	0.820533008	0.997228717	NA
ASV_16s_17969	0.191506686	0.692857773	3.5509391	0.854942091	NA	NA
ASV_16s_17970	0.178979411	0.692766222	3.550966005	0.822469669	0.997207824	NA
ASV_16s_17971	0.176473956	0.692746375	3.55097184	0.815762817	0.997279741	NA
ASV_16s_17972	0.16394668	0.692638184	3.551003666	0.7811263	0.997631828	NA
ASV_16s_17973	0.151419405	0.692512393	3.551040699	0.744482999	NA	NA
ASV_16s_17974	0.151419405	0.692512393	3.551040699	0.744482999	NA	NA
ASV_16s_17975	0.151419405	0.692512393	3.551040699	0.744482999	NA	NA

ASV_16s_17976	0.175833803	0.69274113	3.550973383	0.811397571	0.99732589	NA
ASV_16s_17977	0.178053294	0.692758861	3.550968169	0.817369339	0.997262626	NA
ASV_16s_17978	0.126468269	0.295607535	3.551845298	0.517830767	NA	NA
ASV_16s_17979	0.126468269	0.295607535	3.551845298	0.517830767	NA	NA
ASV_16s_17980	0.126468269	0.295607535	3.551845298	0.517830767	NA	NA
ASV_16s_17981	0.126468269	0.295607535	3.551845298	0.517830767	NA	NA
ASV_16s_17982	0.126468269	0.295607535	3.551845298	0.517830767	NA	NA
ASV_16s_17983	0.126468269	0.295607535	3.551845298	0.517830767	NA	NA
ASV_16s_17984	0.126468269	0.295607535	3.551845298	0.517830767	NA	NA
ASV_16s_17985	0.126468269	0.295607535	3.551845298	0.517830767	NA	NA
ASV_16s_17986	0.126468269	0.295607535	3.551845298	0.517830767	NA	NA
ASV_16s_17987	0.126468269	0.295607535	3.551845298	0.517830767	NA	NA
ASV_16s_17988	0.126468269	0.295607535	3.551845298	0.517830767	NA	NA
ASV_16s_17989	0.126468269	0.295607535	3.551845298	0.517830767	NA	NA
ASV_16s_17990	0.128798538	-0.00943136	3.551479898	0.768322629	0.997753952	NA
ASV_16s_17991	0.129316376	-0.060792243	3.551412042	0.795592804	0.99748867	NA
ASV_16s_17992	0.129316376	-0.060792243	3.551412042	0.795592804	0.99748867	NA
ASV_16s_17993	0.129316376	-0.060792243	3.551412042	0.795592804	0.99748867	NA
ASV_16s_17994	0.129316376	-0.060792243	3.551412042	0.795592804	0.99748867	NA
ASV_16s_17995	0.127549577	-0.075629641	3.5514087	0.795518279	0.997489422	NA
ASV_16s_17996	0.126536718	-0.070784218	3.551421719	0.791002725	0.997534693	NA
ASV_16s_17997	0.129575295	-0.085254617	3.551381933	0.804657472	0.997396131	NA
ASV_16s_17998	0.129575295	-0.085254617	3.551381933	0.804657472	0.997396131	NA
ASV_16s_17999	0.129834214	-0.109222682	3.551352264	0.811460378	0.99732523	NA
ASV_16s_18000	0.12806171	-0.101104751	3.551375958	0.803742559	0.997405572	NA
ASV_16s_18001	0.129834214	-0.109222682	3.551352264	0.811460378	0.99732523	NA
ASV_16s_18002	0.130093133	-0.132526367	3.551325783	0.816249666	0.997274562	NA
ASV_16s_18003	0.130093133	-0.132526367	3.551325783	0.816249666	0.997274562	NA
ASV_16s_18004	0.130093133	-0.132526367	3.551325783	0.816249666	0.997274562	NA
ASV_16s_18005	0.130352051	-0.155302091	3.551302255	0.818950111	0.997245717	NA
ASV_16s_18006	0.130352051	-0.155302091	3.551302255	0.818950111	0.997245717	NA
ASV_16s_18007	0.141807609	-0.198133906	3.551174309	0.866519936	0.996704358	NA
ASV_16s_18008	0.13061097	-0.177636678	3.551281406	0.819361403	0.997241307	NA
ASV_16s_18009	0.126559534	-0.161429062	3.551328635	0.803946933	0.997403465	NA
ASV_16s_18010	0.13061097	-0.177636678	3.551281406	0.819361403	0.997241307	NA
ASV_16s_18011	0.131128808	-0.221281394	3.551246921	0.81445102	0.997293664	NA
ASV_16s_18012	0.131128808	-0.221281394	3.551246921	0.81445102	0.997293664	NA
ASV_16s_18013	0.131128808	-0.221281394	3.551246921	0.81445102	0.997293664	NA
ASV_16s_18014	0.129609519	-0.215818635	3.551262825	0.809156071	0.997349386	NA
ASV_16s_18015	0.138923591	-0.267517576	3.5511583	0.834624436	0.997074334	NA
ASV_16s_18016	0.12658235	-0.247297006	3.551270816	0.786764658	0.99757669	NA
ASV_16s_18017	0.132164483	-0.307930329	3.551196735	0.782234247	0.997621059	NA
ASV_16s_18018	0.128872691	-0.297686268	3.55122801	0.771390627	0.997725077	NA
ASV_16s_18019	0.151011212	-0.398583248	3.551033663	0.800284459	0.997441049	NA
ASV_16s_18020	0.137179608	-0.364009951	3.551143949	0.758041435	0.997848951	NA
ASV_16s_18021	0.045210883	-0.160587314	3.551829781	0.26451224	0.999934723	NA
ASV_16s_18022	0.045210883	-0.160587314	3.551829781	0.26451224	0.999934723	NA
ASV_16s_18023	0.045210883	-0.160587314	3.551829781	0.26451224	0.999934723	NA
ASV_16s_18024	0.045210883	-0.160587314	3.551829781	0.26451224	0.999934723	NA
ASV_16s_18025	0.045210883	-0.160587314	3.551829781	0.26451224	0.999934723	NA
ASV_16s_18026	0.189748175	-0.618523376	3.550826401	0.670635585	0.998551326	NA
ASV_16s_18027	0.172542584	-0.591049971	3.550922013	0.639097621	NA	NA
ASV_16s_18028	0.172542584	-0.591049971	3.550922013	0.639097621	NA	NA
ASV_16s_18029	0.167906128	-0.582679693	3.550948016	0.629769813	0.998819101	NA
ASV_16s_18030	0.176025152	-0.597071645	3.550902617	0.645946401	0.998717468	NA
ASV_16s_18031	0.169821541	-0.586161698	3.550937144	0.633646762	0.998795254	NA
ASV_16s_18032	0.177940565	-0.600175903	3.550891434	0.649657088	NA	NA
ASV_16s_18033	0.177940565	-0.600175903	3.550891434	0.649657088	NA	NA
ASV_16s_18034	0.177940565	-0.600175903	3.550891434	0.649657088	NA	NA
ASV_16s_18035	0.145706575	-0.538611812	3.551088842	0.580686181	0.999094148	NA
ASV_16s_18036	0.160720667	-0.569189386	3.550990476	0.61487908	NA	NA
ASV_16s_18037	0.158169571	-0.564195121	3.551006134	0.609457975	NA	NA
ASV_16s_18038	0.158169571	-0.564195121	3.551006134	0.609457975	NA	NA
ASV_16s_18039	0.158169571	-0.564195121	3.551006134	0.609457975	NA	NA
ASV_16s_18040	0.14054872	-0.526869055	3.551123614	0.568334818	0.999155814	NA
ASV_16s_18041	0.134494753	-0.511810419	3.551161919	0.555580808	NA	NA
ASV_16s_18042	0.134494753	-0.511810419	3.551161919	0.555580808	NA	NA

ASV_16s_18043	0.134494753	-0.511810419	3.551161919	0.555580808	NA	NA
ASV_16s_18044	0.134494753	-0.511810419	3.551161919	0.555580808	NA	NA
ASV_16s_18045	0.134494753	-0.511810419	3.551161919	0.555580808	NA	NA
ASV_16s_18046	0.134494753	-0.511810419	3.551161919	0.555580808	NA	NA
ASV_16s_18047	0.134494753	-0.511810419	3.551161919	0.555580808	NA	NA
ASV_16s_18048	0.132469035	-0.506646417	3.551175176	0.550589237	0.999239354	NA
ASV_16s_18049	0.130443316	-0.501407337	3.551188754	0.545548408	0.999262021	NA
ASV_16s_18050	0.126645095	-0.491334957	3.551215002	0.535963085	NA	NA
ASV_16s_18051	0.126645095	-0.491334957	3.551215002	0.535963085	NA	NA
ASV_16s_18052	0.126645095	-0.491334957	3.551215002	0.535963085	NA	NA
ASV_16s_18053	0.126645095	-0.491334957	3.551215002	0.535963085	NA	NA
ASV_16s_18054	0.040349937	-0.155222436	3.551105846	0.179470478	NA	NA
ASV_16s_18055	0.086070019	0.253651901	3.550842608	0.332583964	0.999858282	NA
ASV_16s_18056	0.081976254	0.220199778	3.550929375	0.38028447	NA	NA
ASV_16s_18057	0.076477844	0.330253871	3.550910137	0.508470358	0.999414871	NA
ASV_16s_18058	0.056249206	0.220320925	3.551089391	0.25104297	NA	NA
ASV_16s_18060	0.171772084	0.687673744	3.550940734	0.778282501	0.997659322	NA
ASV_16s_18061	0.140415977	-0.209185198	3.551383742	0.76125343	0.997819563	NA
ASV_16s_18062	0.136170963	0.107391935	3.551455879	0.734765968	0.998054118	NA
ASV_16s_18063	0.149497482	-0.512676323	3.55126176	0.614809645	0.998908151	NA
ASV_16s_18064	0.163416795	-0.551649029	3.551043675	0.634310033	0.998791143	NA
ASV_16s_18065	0.13265017	0.04370323	3.551336544	0.714379921	0.998222792	NA
ASV_16s_18066	0.149492897	0.195319105	3.551199922	0.784618362	0.997597777	NA
ASV_16s_18067	0.1550487	0.240715392	3.551130621	0.828127916	0.99714619	NA
ASV_16s_18068	0.175797875	-0.501579826	3.551309022	0.623884312	NA	NA
ASV_16s_18069	0.161950992	-0.539764503	3.550946641	0.688265606	0.998424283	NA
ASV_16s_18070	0.179200619	-0.579331772	3.550768567	0.750211939	0.997919487	NA
ASV_16s_18071	0.17413314	-0.499868505	3.551320422	0.624324041	NA	NA
ASV_16s_18072	0.15185019	-0.510984365	3.551046527	0.664743588	0.998592208	NA
ASV_16s_18073	0.140443341	0.409183523	3.551352228	0.719658016	0.998180092	NA
ASV_16s_18074	0.154927416	-0.508864765	3.550994119	0.686658665	0.998436158	NA
ASV_16s_18075	0.159391241	-0.498646708	3.550916493	0.713922395	0.998226462	NA
ASV_16s_18076	0.15088133	0.398695687	3.551259601	0.786491498	0.99757938	NA
ASV_16s_18077	0.166623	-0.494402668	3.550839818	0.758589311	0.997843957	NA
ASV_16s_18078	0.180948479	-0.948188351	3.550808784	0.406406875	0.999722182	NA
ASV_16s_18079	0.143046489	-0.003675188	3.550974128	0.66428092	0.998595385	NA
ASV_16s_18080	0.150826007	0.67691496	3.551429866	0.711237837	0.998247893	NA
ASV_16s_18081	0.138030771	-0.115190519	3.551267556	0.677084275	0.998505681	NA
ASV_16s_18082	0.160458748	-0.036468907	3.551356148	0.50964235	0.999410403	NA
ASV_16s_18083	0.167253814	-0.449547409	3.550816095	0.783320773	0.997610467	NA
ASV_16s_18084	0.125752848	-0.096890575	3.551283329	0.652893158	0.998672085	NA
ASV_16s_18085	0.165256943	-0.425069679	3.550831076	0.782746594	0.997616069	NA
ASV_16s_18086	0.136729365	1.03035781	3.550556094	0.340783238	0.99984616	NA
ASV_16s_18087	0.164974601	-0.1141255	3.551173211	0.779545815	0.997647134	NA
ASV_16s_18088	0.153917661	-0.39905252	3.550927788	0.742812529	0.997984726	NA
ASV_16s_18089	0.155635552	-0.378771963	3.551256426	0.631560473	0.998808127	NA
ASV_16s_18090	0.145100039	0.048997172	3.551146094	0.60403718	0.998969401	NA
ASV_16s_18091	0.166196059	-0.372046204	3.550840718	0.797075733	0.997473682	NA
ASV_16s_18092	0.164346238	-0.372039658	3.550852162	0.789551542	0.997549127	NA
ASV_16s_18093	0.166755954	-0.383154084	3.550832239	0.797803025	0.99746631	NA
ASV_16s_18094	0.167999187	0.183481724	3.549957741	0.761615654	0.997816232	NA
ASV_16s_18095	0.13539335	-0.307762382	3.55136163	0.654147171	0.998663779	NA
ASV_16s_18096	0.164444392	-0.338820279	3.550869599	0.792828102	0.997516458	NA
ASV_16s_18097	0.156009624	-0.314892553	3.550913638	0.763352889	0.997800211	NA
ASV_16s_18098	0.138263119	0.966974982	3.551673557	0.50658149	0.999422024	NA
ASV_16s_18099	0.158864485	0.468067253	3.55110838	0.775498457	0.997686033	NA
ASV_16s_18100	0.155088904	-0.28526155	3.550951932	0.759850135	0.997832435	NA
ASV_16s_18101	0.128118467	-0.540549719	3.551055309	0.45794283	0.999586372	NA
ASV_16s_18102	0.142754388	0.164057803	3.551039784	0.703281323	0.998310397	NA
ASV_16s_18103	0.16388018	-0.165718531	3.550953601	0.793209212	0.997512639	NA
ASV_16s_18104	0.143147704	0.209474398	3.551047062	0.704876475	NA	NA
ASV_16s_18105	0.100156366	-0.006712276	3.551841784	0.46400489	0.999567899	NA
ASV_16s_18106	0.156523689	0.13644261	3.550553317	0.622651938	NA	NA
ASV_16s_18107	0.179822339	1.104411167	3.551158344	0.592815646	0.999030702	NA
ASV_16s_18108	0.154831518	-0.067910508	3.551222799	0.706599449	0.998284514	NA
ASV_16s_18109	0.15560688	0.688531578	3.551067093	0.734525776	0.998056165	NA
ASV_16s_18110	0.166137549	1.080133511	3.551242569	0.568314529	NA	NA

ASV_16s_18111	0.166137549	1.080133511	3.551242569	0.568314529	NA	NA
ASV_16s_18112	0.156889885	1.438744499	3.551076785	0.319387618	0.999876377	NA
ASV_16s_18113	0.140208524	1.096648832	3.551314585	0.5165026	NA	NA
ASV_16s_18114	0.140208524	1.096648832	3.551314585	0.5165026	NA	NA
ASV_16s_18115	0.1448975	0.376503433	3.550961082	0.82789899	0.997148701	NA
ASV_16s_18116	0.176428345	0.746517844	3.551239754	0.493558172	NA	NA
ASV_16s_18117	0.171622885	1.476777398	3.550887389	0.335648536	0.999853832	NA
ASV_16s_18118	0.144750287	1.107927678	3.551288615	0.52703058	NA	NA
ASV_16s_18119	0.144750287	1.107927678	3.551288615	0.52703058	NA	NA
ASV_16s_18120	0.144750287	1.107927678	3.551288615	0.52703058	NA	NA
ASV_16s_18121	0.168444607	0.902110097	3.551102707	0.86596595	0.996711031	NA
ASV_16s_18122	0.152546548	1.404922944	3.551060965	0.313057969	0.99988446	NA
ASV_16s_18123	0.226987172	1.64905401	3.550505865	0.398292208	0.999740306	NA
ASV_16s_18124	0.137233219	0.34585835	3.551860555	0.5451387	NA	NA
ASV_16s_18125	0.157613138	0.342281595	3.552028094	0.336323879	0.999852838	NA
ASV_16s_18126	0.166137549	0.357086089	3.551715747	0.608062851	NA	NA
ASV_16s_18127	0.183755253	0.365868471	3.55161675	0.641828509	NA	NA
ASV_16s_18128	0.240705254	0.384876584	3.551270829	0.732376293	NA	NA
ASV_16s_18129	0.197318379	1.111283615	3.551127446	0.637852098	0.998769025	NA
ASV_16s_18130	0.136523216	0.348370318	3.551746623	0.336757174	NA	NA
ASV_16s_18131	0.154846043	-0.294693771	3.550954588	0.839924144	0.997014846	NA
ASV_16s_18132	0.117284635	0.347568083	3.551821164	0.299862477	0.999900118	NA
ASV_16s_18133	0.143996952	0.348126893	3.551834458	0.560895251	NA	NA
ASV_16s_18134	0.143996952	0.348126893	3.551834458	0.560895251	NA	NA
ASV_16s_18135	0.143996952	0.348126893	3.551834458	0.560895251	NA	NA
ASV_16s_18136	0.134390324	0.343821032	3.552021096	0.309976403	0.999888259	NA
ASV_16s_18137	0.186918005	0.348347666	3.551992916	0.371043459	0.99979521	NA
ASV_16s_18138	0.252273674	0.377451402	3.551442545	0.480778425	NA	NA
ASV_16s_18139	0.245266476	0.385721191	3.55124475	0.738836076	NA	NA
ASV_16s_18140	0.188026691	0.353914956	3.551716276	0.395175181	0.999747046	NA
ASV_16s_18141	0.177940565	-0.530226931	3.550840413	0.633611787	NA	NA
ASV_16s_18142	0.184756165	0.058400825	3.551385359	0.539539753	NA	NA
ASV_16s_18143	0.276737755	-0.570710811	3.550360134	0.794663956	NA	NA
ASV_16s_18144	0.106210489	-0.404141074	3.551489341	0.472022397	NA	NA
ASV_16s_18145	0.106210489	-0.404141074	3.551489341	0.472022397	NA	NA
ASV_16s_18146	0.106210489	-0.404141074	3.551489341	0.472022397	NA	NA
ASV_16s_18147	0.156245431	-0.507879563	3.551055381	0.591885681	NA	NA
ASV_16s_18148	0.139022787	-0.48164389	3.551218438	0.554830977	NA	NA
ASV_16s_18149	0.157470502	1.170519016	3.550984364	0.49621031	NA	NA
ASV_16s_18150	0.157470502	1.170519016	3.550984364	0.49621031	NA	NA
ASV_16s_18151	0.132836586	1.115958801	3.551109627	0.440520378	0.999636448	NA
ASV_16s_18152	0.140893069	1.135474429	3.551067703	0.459635429	NA	NA
ASV_16s_18153	0.140893069	1.135474429	3.551067703	0.459635429	NA	NA
ASV_16s_18154	0.140893069	1.135474429	3.551067703	0.459635429	NA	NA
ASV_16s_18155	0.140893069	1.135474429	3.551067703	0.459635429	NA	NA
ASV_16s_18156	0.178971342	0.030780268	3.550532894	0.911448199	0.996133389	NA
ASV_16s_18157	0.165935749	-0.064685582	3.551052239	0.667697502	0.99857181	NA
ASV_16s_18158	0.161557456	0.375155655	3.551382244	0.53389095	NA	NA
ASV_16s_18159	0.146687548	0.368182965	3.551468531	0.502741005	NA	NA
ASV_16s_18160	0.146687548	0.368182965	3.551468531	0.502741005	NA	NA
ASV_16s_18161	0.146687548	0.368182965	3.551468531	0.502741005	NA	NA
ASV_16s_18162	0.121088839	0.356737717	3.551599607	0.442816429	NA	NA
ASV_16s_18163	0.121088839	0.356737717	3.551599607	0.442816429	NA	NA
ASV_16s_18164	0.121088839	0.356737717	3.551599607	0.442816429	NA	NA
ASV_16s_18165	0.181298087	0.714095429	3.55080609	0.881531682	0.996520163	NA
ASV_16s_18166	0.131013832	0.36132896	3.55155695	0.467128737	NA	NA
ASV_16s_18167	0.131013832	0.36132896	3.55155695	0.467128737	NA	NA
ASV_16s_18168	0.25289561	0.413251611	3.550854422	0.682677165	NA	NA
ASV_16s_18169	0.183032923	0.326920449	3.551315129	0.474335515	0.999535134	NA
ASV_16s_18170	0.183794436	0.385191038	3.551248934	0.576184605	NA	NA
ASV_16s_18171	0.073609034	-0.321635113	3.551450342	0.309512275	NA	NA
ASV_16s_18172	0.073609034	-0.321635113	3.551450342	0.309512275	NA	NA
ASV_16s_18173	0.172609359	0.149732094	3.55034245	0.863627688	0.996739099	NA
ASV_16s_18174	0.141223475	-0.495417367	3.550857213	0.492856133	NA	NA
ASV_16s_18175	0.222402594	-0.544538501	3.550174907	0.635926902	NA	NA
ASV_16s_18176	0.089396484	-0.37841442	3.551326844	0.359620963	NA	NA
ASV_16s_18177	0.089396484	-0.37841442	3.551326844	0.359620963	NA	NA

ASV_16s_18178	0.089396484	-0.37841442	3.551326844	0.359620963	NA		NA
ASV_16s_18179	0.146687548	-0.502635694	3.550801477	0.504597902	NA		NA
ASV_16s_18180	0.161588011	1.178138067	3.550963099	0.504822961	NA		NA
ASV_16s_18181	0.064948901	0.890213214	3.551632302	0.250501729	NA		NA
ASV_16s_18182	0.064948901	0.890213214	3.551632302	0.250501729	NA		NA
ASV_16s_18183	0.064948901	0.890213214	3.551632302	0.250501729	NA		NA
ASV_16s_18184	0.064948901	0.890213214	3.551632302	0.250501729	NA		NA
ASV_16s_18185	0.146687548	-0.078379865	3.550886937	0.787042523	NA		NA
ASV_16s_18186	0.146687548	-0.078379865	3.550886937	0.787042523	NA		NA
ASV_16s_18187	0.146687548	-0.078379865	3.550886937	0.787042523	NA		NA
ASV_16s_18188	0.142740599	-0.078345387	3.55089718	0.77437895		0.997696717	NA
ASV_16s_18189	0.125082141	-0.078166508	3.550950865	0.714220424	NA		NA
ASV_16s_18190	0.125082141	-0.078166508	3.550950865	0.714220424	NA		NA
ASV_16s_18191	0.125082141	-0.078166508	3.550950865	0.714220424	NA		NA
ASV_16s_18192	0.125082141	-0.078166508	3.550950865	0.714220424	NA		NA
ASV_16s_18193	0.125082141	-0.078166508	3.550950865	0.714220424	NA		NA
ASV_16s_18194	0.125082141	-0.078166508	3.550950865	0.714220424	NA		NA
ASV_16s_18195	0.125082141	-0.078166508	3.550950865	0.714220424	NA		NA
ASV_16s_18196	0.125082141	-0.078166508	3.550950865	0.714220424	NA		NA
ASV_16s_18197	0.125082141	-0.078166508	3.550950865	0.714220424	NA		NA
ASV_16s_18198	0.125082141	-0.078166508	3.550950865	0.714220424	NA		NA
ASV_16s_18199	0.125082141	-0.078166508	3.550950865	0.714220424	NA		NA
ASV_16s_18200	0.125082141	-0.078166508	3.550950865	0.714220424	NA		NA
ASV_16s_18201	0.134915436	-0.078271121	3.55091925	0.748441153		0.997935225	NA
ASV_16s_18202	0.141125939	-0.078330733	3.550901534	0.769121479	NA		NA
ASV_16s_18203	0.141125939	-0.078330733	3.550901534	0.769121479	NA		NA
ASV_16s_18204	0.086217721	0.220186683	3.550912112	0.399421389		0.999737834	NA
ASV_16s_18205	0.086924632	0.220184625	3.550909398	0.402562082		0.999730874	NA
ASV_16s_18206	0.088338454	0.220180609	3.550904098	0.408807488		0.999716658	NA
ASV_16s_18207	0.093119329	0.220167937	3.550887359	0.429493756	NA		NA
ASV_16s_18208	0.093119329	0.220167937	3.550887359	0.429493756	NA		NA
ASV_16s_18209	0.093119329	0.220167937	3.550887359	0.429493756	NA		NA
ASV_16s_18210	0.158653838	0.310947679	3.551000136	0.804208321		0.997400769	NA
ASV_16s_18211	0.138579489	0.310951596	3.551059434	0.740919322	NA		NA
ASV_16s_18212	0.138579489	0.310951596	3.551059434	0.740919322	NA		NA
ASV_16s_18213	0.158426654	0.310947717	3.551000724	0.803527959	NA		NA
ASV_16s_18214	0.142146177	0.687425298	3.551020666	0.691236218	NA		NA
ASV_16s_18215	0.112219291	0.07348456	3.550861585	0.513531074	NA		NA
ASV_16s_18216	0.164502393	-0.418939112	3.550798041	0.634654127	NA		NA
ASV_16s_18217	0.202488683	1.396179021	3.551102104	0.389886805		0.999758204	NA
ASV_16s_18218	0.160795652	0.698793113	3.550870568	0.866721983	NA		NA
ASV_16s_18219	0.130037324	-0.067039037	3.551007271	0.824424134		0.997186634	NA
ASV_16s_18220	0.188361006	1.641324536	3.550909689	0.244024909		0.99995038	NA
ASV_16s_18221	0.149006755	0.647400902	3.551452059	0.616131429		0.998900471	NA
ASV_16s_18222	0.19130215	0.309882657	3.550830759	0.719687975		0.998179848	NA
ASV_16s_18223	0.130532842	-0.066724514	3.551019962	0.832223614		0.997101025	NA
ASV_16s_18224	0.130532842	-0.066724514	3.551019962	0.832223614		0.997101025	NA
ASV_16s_18225	0.131523879	-0.067102652	3.551036727	0.843652499		0.996972525	NA
ASV_16s_18226	0.141956558	0.639000593	3.55066656	0.691180056		0.998402591	NA
ASV_16s_18227	0.101004337	0.097808595	3.5516661	0.584120866		0.999076475	NA
ASV_16s_18228	0.140729515	-0.06345777	3.551042709	0.878748081		0.996554812	NA
ASV_16s_18229	0.142860373	-0.061120174	3.551027128	0.883479671		0.996495782	NA
ASV_16s_18230	0.155190355	0.332783651	3.551371875	0.53168482		0.999321995	NA
ASV_16s_18231	0.231745474	1.165964561	3.550839062	0.683115965	NA		NA
ASV_16s_18232	0.135489682	1.081723245	3.551496456	0.405129574		0.999725091	NA
ASV_16s_18233	0.062620662	0.289565306	3.55187586	0.325253177		0.999868545	NA
ASV_16s_18234	0.062620662	0.289565306	3.55187586	0.325253177		0.999868545	NA
ASV_16s_18235	0.207430719	-0.539018428	3.551096688	0.6881988	NA		NA
ASV_16s_18236	0.280028582	-0.598399976	3.55082727	0.789790555	NA		NA
ASV_16s_18237	0.124308623	0.913525512	3.551508553	0.457560953	NA		NA
ASV_16s_18238	0.256137588	-0.17276192	3.550129069	1.015898311	NA		NA
ASV_16s_18239	0.203283314	0.333375281	3.550994935	0.862962263		0.996747058	NA
ASV_16s_18240	0.18421514	1.215589179	3.550857521	0.549076964	NA		NA
ASV_16s_18241	0.104971791	-0.077888267	3.551033817	0.637895304	NA		NA
ASV_16s_18242	0.104971791	-0.077888267	3.551033817	0.637895304	NA		NA
ASV_16s_18243	0.104971791	-0.077888267	3.551033817	0.637895304	NA		NA
ASV_16s_18244	0.134884804	-0.094009538	3.550929589	0.817850073		0.997257491	NA

ASV_16s_18245	0.134576522	-0.043927186	3.55106738	0.786569345	NA	NA
ASV_16s_18246	0.135922191	-0.02245309	3.551072036	0.782123548	NA	NA
ASV_16s_18247	0.326532041	-0.029238798	3.550768562	1.250146998	NA	NA
ASV_16s_18248	0.246702332	0.289348602	3.550873881	1.69539397	0.974765042	NA
ASV_16s_18249	0.229850318	-0.485454667	3.550429352	0.774594278	0.997694665	NA
ASV_16s_18250	0.218936311	0.420119937	3.550942009	1.46612615	0.983459255	NA
ASV_16s_18251	0.241089147	0.661180322	3.550313765	1.144861017	0.99214155	NA
ASV_16s_18252	0.231709697	-0.028931499	3.550870107	1.068064205	NA	NA
ASV_16s_18253	0.231091952	0.150837768	3.550875624	1.475451368	0.983147383	NA
ASV_16s_18254	0.195951559	0.379129224	3.550678978	1.009341759	NA	NA
ASV_16s_18255	0.149536598	-0.075221787	3.551137754	1.015946984	0.994567171	NA
ASV_16s_18256	0.138495564	-0.028228305	3.551104173	0.819506387	NA	NA
ASV_16s_18257	0.130766485	0.212445319	3.550974771	0.786045614	NA	NA
ASV_16s_18258	0.113501784	-0.072829179	3.551042923	0.695511197	NA	NA
ASV_16s_18259	0.113501784	-0.072829179	3.551042923	0.695511197	NA	NA
ASV_16s_18260	0.113501784	-0.072829179	3.551042923	0.695511197	NA	NA
ASV_16s_18261	0.113501784	-0.072829179	3.551042923	0.695511197	NA	NA
ASV_16s_18262	0.165468118	0.374654528	3.550955589	0.922362649	0.995985637	NA
ASV_16s_18263	0.192520254	0.301789039	3.550590378	1.081097601	0.993412134	NA
ASV_16s_18264	0.140828561	-0.073188404	3.550940896	0.792312047	NA	NA
ASV_16s_18265	0.157985002	0.360278795	3.551235215	0.863872914	NA	NA
ASV_16s_18266	0.157985002	0.360278795	3.551235215	0.863872914	NA	NA
ASV_16s_18267	0.2594338	0.360354996	3.550985801	1.110230745	NA	NA
ASV_16s_18268	0.201242186	0.354094891	3.551445463	0.729294022	0.998100391	NA
ASV_16s_18269	0.203168569	0.338379657	3.55112845	0.738282397	0.998023991	NA
ASV_16s_18270	0.140443209	0.360254631	3.551314456	0.808709888	NA	NA
ASV_16s_18271	0.140443209	0.360254631	3.551314456	0.808709888	NA	NA
ASV_16s_18272	0.140443209	0.360254631	3.551314456	0.808709888	NA	NA
ASV_16s_18273	0.140443209	0.360254631	3.551314456	0.808709888	NA	NA
ASV_16s_18274	0.140443209	0.360254631	3.551314456	0.808709888	NA	NA
ASV_16s_18275	0.140443209	0.360254631	3.551314456	0.808709888	NA	NA
ASV_16s_18276	0.140443209	0.360254631	3.551314456	0.808709888	NA	NA
ASV_16s_18277	0.140443209	0.360254631	3.551314456	0.808709888	NA	NA
ASV_16s_18278	0.140443209	0.360254631	3.551314456	0.808709888	NA	NA
ASV_16s_18279	0.140443209	0.360254631	3.551314456	0.808709888	NA	NA
ASV_16s_18280	0.140443209	0.360254631	3.551314456	0.808709888	NA	NA
ASV_16s_18281	0.140443209	0.360254631	3.551314456	0.808709888	NA	NA
ASV_16s_18282	0.181971505	0.315939755	3.55098137	0.893092008	NA	NA
ASV_16s_18283	0.172865967	0.315939782	3.551002004	0.868136215	0.996684839	NA
ASV_16s_18284	0.162622238	0.315939817	3.551027954	0.838089815	0.997035524	NA
ASV_16s_18285	0.157957864	0.315939834	3.551040873	0.82409494	0.99719021	NA
ASV_16s_18286	0.157295612	0.315939923	3.55104276	0.822930295	NA	NA
ASV_16s_18287	0.1909364	0.315939732	3.550963019	0.914568024	0.996091522	NA
ASV_16s_18288	0.152162678	0.315939858	3.551058012	0.807237346	0.997369391	NA
ASV_16s_18289	0.222479418	0.315939667	3.550910025	0.998370293	0.994854783	NA
ASV_16s_18290	0.151136091	0.315939862	3.551061186	0.80405127	0.997402389	NA
ASV_16s_18291	0.150109504	0.315939867	3.551064403	0.800848074	0.997435289	NA
ASV_16s_18292	0.190575795	0.796782978	3.550935318	1.001088749	0.994810954	NA
ASV_16s_18293	0.148398526	0.315939874	3.551069862	0.79547748	0.997489834	NA
ASV_16s_18294	0.170075493	0.315939791	3.551008814	0.856840341	0.996819686	NA
ASV_16s_18295	0.184434684	0.315939749	3.550976202	0.897064947	0.996322648	NA
ASV_16s_18296	0.146687548	0.315939975	3.551075449	0.790057758	NA	NA
ASV_16s_18297	0.146687548	0.315939975	3.551075449	0.790057758	NA	NA
ASV_16s_18298	0.146687548	0.315939975	3.551075449	0.790057758	NA	NA
ASV_16s_18299	0.146687548	0.315939975	3.551075449	0.790057758	NA	NA
ASV_16s_18300	0.146687548	0.315939975	3.551075449	0.790057758	NA	NA
ASV_16s_18301	0.146687548	0.315939975	3.551075449	0.790057758	NA	NA
ASV_16s_18302	0.146687548	0.315939975	3.551075449	0.790057758	NA	NA
ASV_16s_18303	0.146687548	0.315939975	3.551075449	0.790057758	NA	NA
ASV_16s_18304	0.276160199	0.315939597	3.550847485	1.107343784	NA	NA
ASV_16s_18305	0.197507611	0.596102872	3.550689238	0.701821894	NA	NA
ASV_16s_18306	0.139695984	0.687399958	3.551028783	0.683478139	NA	NA
ASV_16s_18307	0.21850678	1.128495799	3.550825549	0.690410219	0.99840834	NA
ASV_16s_18308	0.210981494	1.142675262	3.551037953	0.695732223	0.998368314	NA
ASV_16s_18309	0.20533753	1.059785145	3.551210107	0.709078951	NA	NA
ASV_16s_18310	0.303915292	0.734385638	3.550929998	1.129428634	0.992462079	NA
ASV_16s_18311	0.29931321	0.734366903	3.550935419	1.120598644	0.99264171	NA

ASV_16s_18312	0.229315935	0.733991645	3.551044486	0.983840166	NA	NA
ASV_16s_18313	0.213323344	0.979725669	3.551189892	0.789658655	NA	NA
ASV_16s_18314	0.209538075	1.121272869	3.550994136	0.705370146	0.998294134	NA
ASV_16s_18315	0.194049601	0.733700866	3.551128969	0.901822016	NA	NA
ASV_16s_18316	0.194049601	0.733700866	3.551128969	0.901822016	NA	NA
ASV_16s_18317	0.194049601	0.733700866	3.551128969	0.901822016	NA	NA
ASV_16s_18318	0.194049601	0.733700866	3.551128969	0.901822016	NA	NA
ASV_16s_18319	0.194049601	0.733700866	3.551128969	0.901822016	NA	NA
ASV_16s_18320	0.194049601	0.733700866	3.551128969	0.901822016	NA	NA
ASV_16s_18321	0.194049601	0.733700866	3.551128969	0.901822016	NA	NA
ASV_16s_18322	0.194049601	0.733700866	3.551128969	0.901822016	NA	NA
ASV_16s_18323	0.194049601	0.733700866	3.551128969	0.901822016	NA	NA
ASV_16s_18324	0.194049601	0.733700866	3.551128969	0.901822016	NA	NA
ASV_16s_18325	0.096123569	0.704560554	3.551560016	0.591461848	NA	NA
ASV_16s_18326	0.096123569	0.704560554	3.551560016	0.591461848	NA	NA
ASV_16s_18327	0.18040326	0.69277736	3.550962783	0.826185575	NA	NA
ASV_16s_18328	0.190590244	0.692850141	3.550940985	0.851710512	0.996879722	NA
ASV_16s_18329	0.183375491	0.69279839	3.550956172	0.833993675	0.997081362	NA
ASV_16s_18330	0.183540615	0.69279962	3.550955811	0.834424382	0.997076564	NA
ASV_16s_18331	0.183870863	0.692802073	3.550955091	0.835284831	0.997066964	NA
ASV_16s_18332	0.166497801	0.69265991	3.550996832	0.788251347	0.997562011	NA
ASV_16s_18333	0.185522102	0.692815649	3.550951529	0.839571666	NA	NA
ASV_16s_18334	0.185522102	0.692815649	3.550951529	0.839571666	NA	NA
ASV_16s_18335	0.185522102	0.692815649	3.550951529	0.839571666	NA	NA
ASV_16s_18336	0.185522102	0.692815649	3.550951529	0.839571666	NA	NA
ASV_16s_18337	0.185522102	0.692815649	3.550951529	0.839571666	NA	NA
ASV_16s_18338	0.185522102	0.692815649	3.550951529	0.839571666	NA	NA
ASV_16s_18339	0.162973006	0.692629138	3.55100638	0.778253977	0.997659597	NA
ASV_16s_18340	0.146687548	0.692459391	3.551056365	0.729985312	NA	NA
ASV_16s_18341	0.146687548	0.692459391	3.551056365	0.729985312	NA	NA
ASV_16s_18342	0.146687548	0.692459391	3.551056365	0.729985312	NA	NA
ASV_16s_18343	0.146687548	0.692459391	3.551056365	0.729985312	NA	NA
ASV_16s_18344	0.146687548	0.692459391	3.551056365	0.729985312	NA	NA
ASV_16s_18345	0.164443474	0.69264276	3.551002371	0.779429578	0.997648257	NA
ASV_16s_18346	0.168756671	0.692679934	3.550990951	0.791358885	0.997531142	NA
ASV_16s_18347	0.179791232	0.692771013	3.550964207	0.821446421	0.997218876	NA
ASV_16s_18348	0.181998144	0.692787921	3.550959244	0.827379457	0.997154393	NA
ASV_16s_18349	0.215491761	0.693003721	3.550896311	0.912453425	NA	NA
ASV_16s_18350	0.215101828	0.693001607	3.550896931	0.91156184	NA	NA
ASV_16s_18351	0.215303172	1.384382025	3.550963587	0.513328705	0.999396199	NA
ASV_16s_18352	0.122388647	0.295899691	3.551857264	0.507478219	NA	NA
ASV_16s_18353	0.122388647	0.295899691	3.551857264	0.507478219	NA	NA
ASV_16s_18354	0.122388647	0.295899691	3.551857264	0.507478219	NA	NA
ASV_16s_18355	0.122388647	0.295899691	3.551857264	0.507478219	NA	NA
ASV_16s_18356	0.122388647	0.295899691	3.551857264	0.507478219	NA	NA
ASV_16s_18357	0.122388647	0.295899691	3.551857264	0.507478219	NA	NA
ASV_16s_18358	0.122388647	0.295899691	3.551857264	0.507478219	NA	NA
ASV_16s_18359	0.122388647	0.295899691	3.551857264	0.507478219	NA	NA
ASV_16s_18360	0.122388647	0.295899691	3.551857264	0.507478219	NA	NA
ASV_16s_18361	0.130141404	0.062122248	3.551566433	0.700768907	0.998329822	NA
ASV_16s_18362	0.12394216	0.074189387	3.551623154	0.689743334	0.998413309	NA
ASV_16s_18363	0.134793058	-0.040580409	3.551396096	0.78847493	0.997559799	NA
ASV_16s_18364	0.124459998	0.014957467	3.551542928	0.733594721	0.998064085	NA
ASV_16s_18365	0.124459998	0.014957467	3.551542928	0.733594721	0.998064085	NA
ASV_16s_18366	0.12243428	0.026815818	3.551572827	0.723383417	0.998149547	NA
ASV_16s_18367	0.124718917	-0.012225184	3.551507767	0.751206695	0.997910611	NA
ASV_16s_18368	0.124977836	-0.038777976	3.551471536	0.763667685	0.9977973	NA
ASV_16s_18369	0.124977836	-0.038777976	3.551471536	0.763667685	0.9977973	NA
ASV_16s_18370	0.125495673	-0.089034989	3.551410397	0.782184249	0.997621546	NA
ASV_16s_18371	0.12372317	-0.080693731	3.551433085	0.774260944	0.997697842	NA
ASV_16s_18372	0.130181332	-0.110623417	3.551348205	0.803527341	0.997407789	NA
ASV_16s_18373	0.124235304	-0.106254587	3.551403058	0.781600109	0.997627227	NA
ASV_16s_18374	0.125754592	-0.113231379	3.551382338	0.788151727	0.997562997	NA
ASV_16s_18375	0.125754592	-0.113231379	3.551382338	0.788151727	0.997562997	NA
ASV_16s_18376	0.123728874	-0.103969821	3.551409279	0.779432949	0.997648225	NA
ASV_16s_18377	0.125754592	-0.113231379	3.551382338	0.788151727	0.997562997	NA
ASV_16s_18378	0.1224628	-0.098187068	3.551425119	0.774052935	0.997699823	NA

ASV_16s_18379	0.126013511	-0.136943669	3.551355816	0.79219379	0.997522804	NA
ASV_16s_18380	0.126013511	-0.136943669	3.551355816	0.79219379	0.997522804	NA
ASV_16s_18381	0.12627243	-0.160154283	3.551332399	0.793800033	0.997506712	NA
ASV_16s_18382	0.12627243	-0.160154283	3.551332399	0.793800033	0.997506712	NA
ASV_16s_18383	0.12627243	-0.160154283	3.551332399	0.793800033	0.997506712	NA
ASV_16s_18384	0.12627243	-0.160154283	3.551332399	0.793800033	0.997506712	NA
ASV_16s_18385	0.131697879	-0.202558245	3.551254497	0.813163322	0.997307285	NA
ASV_16s_18386	0.126531349	-0.182924612	3.551311748	0.79320507	0.997512681	NA
ASV_16s_18387	0.122485616	-0.188819815	3.551343411	0.775178572	0.997689089	NA
ASV_16s_18388	0.126790268	-0.205349364	3.551293679	0.790615356	0.997538551	NA
ASV_16s_18389	0.122485616	-0.188819815	3.551343411	0.775178572	0.997689089	NA
ASV_16s_18390	0.127049186	-0.227518952	3.551278057	0.786328444	0.997580985	NA
ASV_16s_18391	0.12249132	-0.210784971	3.551328734	0.770816119	0.997730503	NA
ASV_16s_18392	0.124775957	-0.240849599	3.551290718	0.771881404	0.997720435	NA
ASV_16s_18393	0.127825943	-0.294278557	3.55123843	0.761378472	0.997818414	NA
ASV_16s_18394	0.122519841	-0.320766011	3.55127652	0.713238905	0.998231934	NA
ASV_16s_18395	0.043752468	-0.151630303	3.551837453	0.258008389	0.99994002	NA
ASV_16s_18396	0.167615607	-0.582064272	3.550949946	0.626261509	0.998840406	NA
ASV_16s_18397	0.166976694	-0.580924535	3.550953524	0.627793263	NA	NA
ASV_16s_18398	0.158631074	-0.565048804	3.551003447	0.610356527	0.998933759	NA
ASV_16s_18399	0.158167428	-0.564135145	3.551006323	0.609365167	0.998939404	NA
ASV_16s_18400	0.161996163	-0.571603653	3.550982964	0.617478066	0.998892609	NA
ASV_16s_18401	0.160720615	-0.569133342	3.550990651	0.614790474	0.998908262	NA
ASV_16s_18402	0.160082841	-0.567891339	3.550994531	0.613441327	0.998916063	NA
ASV_16s_18403	0.160082841	-0.567891339	3.550994531	0.613441327	0.998916063	NA
ASV_16s_18404	0.153067327	-0.553917269	3.551038853	0.598347153	NA	NA
ASV_16s_18405	0.153067327	-0.553917269	3.551038853	0.598347153	NA	NA
ASV_16s_18406	0.153067327	-0.553917269	3.551038853	0.598347153	NA	NA
ASV_16s_18407	0.136614375	-0.517062093	3.551148568	0.559415405	0.999198538	NA
ASV_16s_18408	0.130156212	-0.500606048	3.551190843	0.544756914	NA	NA
ASV_16s_18409	0.130156212	-0.500606048	3.551190843	0.544756914	NA	NA
ASV_16s_18410	0.130156212	-0.500606048	3.551190843	0.544756914	NA	NA
ASV_16s_18411	0.130156212	-0.500606048	3.551190843	0.544756914	NA	NA
ASV_16s_18412	0.130156212	-0.500606048	3.551190843	0.544756914	NA	NA
ASV_16s_18413	0.130156212	-0.500606048	3.551190843	0.544756914	NA	NA
ASV_16s_18414	0.126104776	-0.489811503	3.551218957	0.534501061	0.99931009	NA
ASV_16s_18415	0.126104776	-0.489811503	3.551218957	0.534501061	0.99931009	NA
ASV_16s_18416	0.125598346	-0.488431091	3.55122255	0.533199709	0.999315609	NA
ASV_16s_18417	0.136284251	-0.516208841	3.551150728	0.556811368	0.999210731	NA
ASV_16s_18418	0.125345132	-0.487738986	3.551224355	0.532547561	0.999318363	NA
ASV_16s_18419	0.123572628	-0.482857344	3.55123715	0.527957638	NA	NA
ASV_16s_18420	0.122559769	-0.480040825	3.551244585	0.525319329	NA	NA
ASV_16s_18421	0.122559769	-0.480040825	3.551244585	0.525319329	NA	NA
ASV_16s_18422	0.122559769	-0.480040825	3.551244585	0.525319329	NA	NA
ASV_16s_18423	0.122559769	-0.480040825	3.551244585	0.525319329	NA	NA
ASV_16s_18424	0.122559769	-0.480040825	3.551244585	0.525319329	NA	NA
ASV_16s_18425	0.122559769	-0.480040825	3.551244585	0.525319329	NA	NA
ASV_16s_18426	0.122559769	-0.480040825	3.551244585	0.525319329	NA	NA
ASV_16s_18427	0.122559769	-0.480040825	3.551244585	0.525319329	NA	NA
ASV_16s_18428	0.122559769	-0.480040825	3.551244585	0.525319329	NA	NA
ASV_16s_18429	0.122559769	-0.480040825	3.551244585	0.525319329	NA	NA
ASV_16s_18430	0.122559769	-0.480040825	3.551244585	0.525319329	NA	NA
ASV_16s_18431	0.143931577	-0.53471116	3.551101829	0.573136637	0.999132188	NA
ASV_16s_18432	0.079331858	0.220208677	3.550941098	0.367990423	NA	NA
ASV_16s_18433	0.04117309	0.226583918	3.551217195	0.163273418	NA	NA
ASV_16s_18434	0.054434716	0.220358278	3.551106174	0.240998219	NA	NA
ASV_16s_18435	0.054434716	0.220358278	3.551106174	0.240998219	NA	NA
ASV_16s_18436	0.054434716	0.220358278	3.551106174	0.240998219	NA	NA
ASV_16s_18439	0.124059874	-0.306511189	3.551343475	0.496205184	NA	NA
ASV_16s_18440	0.130796747	0.240821328	3.5516287	0.680387523	0.998481933	NA
ASV_16s_18441	0.141043544	0.184541626	3.55152276	0.652278662	0.998676142	NA
ASV_16s_18442	0.163623913	-0.560039219	3.550963412	0.663845906	0.998598368	NA
ASV_16s_18443	0.149673215	-0.655987064	3.551134707	0.481912589	0.999510052	NA
ASV_16s_18444	0.126940298	0.646187792	3.551836056	0.557641762	0.999206856	NA
ASV_16s_18445	0.109952181	0.03186661	3.551805114	0.454686989	0.999596068	NA
ASV_16s_18446	0.158414723	-0.514879939	3.55095182	0.689975938	0.998411577	NA
ASV_16s_18447	0.16983292	-0.538134662	3.550829966	0.738750103	0.998019961	NA

ASV_16s_18448	0.151218199	-0.495107051	3.551024819	0.672465607	0.998538469	NA
ASV_16s_18449	0.1308677	0.666597699	3.55167241	0.62729074	0.998834182	NA
ASV_16s_18450	0.1308677	0.666597699	3.55167241	0.62729074	0.998834182	NA
ASV_16s_18451	0.126902981	0.315918097	3.552007331	0.328756292	0.999863708	NA
ASV_16s_18452	0.16422825	-0.484004913	3.550858408	0.748442226	0.997935215	NA
ASV_16s_18453	0.168209172	-0.477521204	3.550817799	0.77318097	0.997708114	NA
ASV_16s_18454	0.135662636	-0.030412243	3.551514224	0.461822682	0.999574613	NA
ASV_16s_18455	0.170585257	0.626236428	3.550857925	0.729868714	0.998095566	NA
ASV_16s_18456	0.161608715	-0.43050727	3.550861987	0.759514233	0.997835508	NA
ASV_16s_18457	0.14870636	-0.367598299	3.550970547	0.720018486	0.998177151	NA
ASV_16s_18458	0.160894199	0.093050004	3.551138304	0.794731085	0.997497353	NA
ASV_16s_18459	0.139418392	0.756588031	3.551312173	0.486107933	0.999495775	NA
ASV_16s_18460	0.166438148	-0.062919284	3.550930978	0.682106363	0.998469477	NA
ASV_16s_18461	0.160982754	-0.372027013	3.550874337	0.770458718	0.997733874	NA
ASV_16s_18462	0.136842584	-0.283181488	3.551103909	0.71240092	0.998238629	NA
ASV_16s_18463	0.183963513	-0.521333255	3.550698339	0.387414448	0.999763302	NA
ASV_16s_18464	0.153508694	-0.31488281	3.550929232	0.748085124	0.99793838	NA
ASV_16s_18465	0.157185416	0.679635717	3.551192409	0.73530617	0.99804951	NA
ASV_16s_18466	0.15567783	0.164892468	3.550970521	0.738050845	0.998025984	NA
ASV_16s_18467	0.15834926	0.183512437	3.549991849	0.710090711	0.998256997	NA
ASV_16s_18468	0.15834926	0.183512437	3.549991849	0.710090711	0.998256997	NA
ASV_16s_18469	0.164297819	0.681690546	3.551117189	0.754074435	NA	NA
ASV_16s_18470	0.149566674	-0.110711095	3.551003536	0.740284981	0.998006697	NA
ASV_16s_18471	0.142915511	-0.105778276	3.551052994	0.743449623	0.997979163	NA
ASV_16s_18472	0.136866561	-0.163231971	3.551032375	0.360502045	0.999814106	NA
ASV_16s_18473	0.143594703	-0.060822221	3.551381453	0.630476151	0.99881478	NA
ASV_16s_18474	0.14161263	-0.069418955	3.551231151	0.651894075	0.998678677	NA
ASV_16s_18475	0.166689026	0.375118275	3.550967303	0.768683058	0.997750573	NA
ASV_16s_18476	0.216751176	1.216422316	3.550877095	0.5794841	NA	NA
ASV_16s_18477	0.160778273	1.070041906	3.551278917	0.557218811	NA	NA
ASV_16s_18478	0.160778273	1.070041906	3.551278917	0.557218811	NA	NA
ASV_16s_18479	0.160778273	1.070041906	3.551278917	0.557218811	NA	NA
ASV_16s_18480	0.118811365	0.217171388	3.55113807	0.243125589	0.999951	NA
ASV_16s_18481	0.131361571	0.746461627	3.551651023	0.434360612	NA	NA
ASV_16s_18482	0.152676967	1.126807093	3.551246371	0.544763661	NA	NA
ASV_16s_18483	0.149318022	1.118893228	3.551263897	0.537286979	0.999298174	NA
ASV_16s_18484	0.158077838	0.656494716	3.551336293	0.83782328	0.997038521	NA
ASV_16s_18485	0.170039932	1.409880632	3.551085008	0.38932779	0.999759363	NA
ASV_16s_18486	0.140080923	1.096272842	3.55131546	0.516127392	NA	NA
ASV_16s_18487	0.140080923	1.096272842	3.55131546	0.516127392	NA	NA
ASV_16s_18488	0.140080923	1.096272842	3.55131546	0.516127392	NA	NA
ASV_16s_18489	0.140080923	1.096272842	3.55131546	0.516127392	NA	NA
ASV_16s_18490	0.178033142	1.50737745	3.55083018	0.338244074	0.999849988	NA
ASV_16s_18491	0.137243266	1.301608682	3.551281326	0.284177338	0.999916736	NA
ASV_16s_18492	0.132806341	0.344237018	3.551874884	0.534410963	NA	NA
ASV_16s_18493	0.160778273	0.354279298	3.551747438	0.597103264	NA	NA
ASV_16s_18494	0.156949066	-0.00578728	3.551089618	0.895409162	0.996344041	NA
ASV_16s_18495	0.177827664	0.362853251	3.551652641	0.63077046	NA	NA
ASV_16s_18496	0.169165887	0.341949619	3.551982412	0.409941038	0.999714024	NA
ASV_16s_18497	0.173320254	0.34623538	3.551919976	0.47262819	0.999540662	NA
ASV_16s_18498	0.141682008	0.395849816	3.551199266	0.579275048	0.999101342	NA
ASV_16s_18499	0.102916982	0.334193147	3.551961531	0.455747326	NA	NA
ASV_16s_18500	0.102916982	0.334193147	3.551961531	0.455747326	NA	NA
ASV_16s_18501	0.102916982	0.334193147	3.551961531	0.455747326	NA	NA
ASV_16s_18502	0.150801932	-0.471744344	3.55117949	0.492955579	NA	NA
ASV_16s_18503	0.139351889	0.346648609	3.551852881	0.550060947	NA	NA
ASV_16s_18504	0.135080803	-0.001567877	3.551833482	0.56113704	NA	NA
ASV_16s_18505	0.187056751	0.367515192	3.551596283	0.647701088	NA	NA
ASV_16s_18506	0.198013555	0.34792754	3.551991291	0.387979955	0.999762143	NA
ASV_16s_18507	0.172200546	-0.525569731	3.550896905	0.623013073	NA	NA
ASV_16s_18508	0.23812915	-0.714693309	3.550308876	0.508511635	NA	NA
ASV_16s_18509	0.102784344	-0.394194245	3.551518013	0.462428676	NA	NA
ASV_16s_18510	0.102784344	-0.394194245	3.551518013	0.462428676	NA	NA
ASV_16s_18511	0.148430387	-0.820604421	3.551110471	0.341553988	0.999844985	NA
ASV_16s_18512	0.090337278	-0.546297054	3.551432526	0.231013364	0.999958832	NA
ASV_16s_18513	0.079533322	0.950252252	3.55150138	0.296776088	NA	NA
ASV_16s_18514	0.079533322	0.950252252	3.55150138	0.296776088	NA	NA

ASV_16s_18515	0.079533322	0.950252252	3.55150138	0.296776088	NA	NA
ASV_16s_18516	0.218601785	1.259637818	3.550715526	0.607861598	NA	NA
ASV_16s_18517	0.136348131	1.124644326	3.551090888	0.448980454	NA	NA
ASV_16s_18518	0.156345925	0.372772352	3.551411092	0.523139151	NA	NA
ASV_16s_18519	0.156345925	0.372772352	3.551411092	0.523139151	NA	NA
ASV_16s_18520	0.141955692	0.366029249	3.551497718	0.4922463	NA	NA
ASV_16s_18521	0.141955692	0.366029249	3.551497718	0.4922463	NA	NA
ASV_16s_18522	0.117182748	0.354776371	3.551616974	0.432835626	NA	NA
ASV_16s_18523	0.200739405	0.393315368	3.551134089	0.604808964	NA	NA
ASV_16s_18524	0.126787579	0.359522891	3.551575527	0.456880997	NA	NA
ASV_16s_18525	0.126787579	0.359522891	3.551575527	0.456880997	NA	NA
ASV_16s_18526	0.044146354	0.340731822	3.551895835	0.199193597	0.999975189	NA
ASV_16s_18527	0.177865584	0.381804164	3.551289724	0.565408602	NA	NA
ASV_16s_18528	0.177865584	0.381804164	3.551289724	0.565408602	NA	NA
ASV_16s_18529	0.071234549	-0.311850349	3.55146697	0.301361615	NA	NA
ASV_16s_18530	0.071234549	-0.311850349	3.55146697	0.301361615	NA	NA
ASV_16s_18531	0.174744408	-0.53042041	3.550545943	0.559605829	NA	NA
ASV_16s_18532	0.174744408	-0.53042041	3.550545943	0.559605829	NA	NA
ASV_16s_18533	0.183682624	-0.534354108	3.55046528	0.571489973	0.99914034	NA
ASV_16s_18534	0.231691184	-0.545888409	3.550118076	0.647887374	NA	NA
ASV_16s_18535	0.13666788	-0.488432051	3.550904306	0.48263457	NA	NA
ASV_16s_18536	0.079287052	-0.343006937	3.551406515	0.328139009	NA	NA
ASV_16s_18537	0.079287052	-0.343006937	3.551406515	0.328139009	NA	NA
ASV_16s_18538	0.156375494	1.168412856	3.550990309	0.49381297	NA	NA
ASV_16s_18539	0.107652851	0.645404382	3.550846739	0.355647429	NA	NA
ASV_16s_18540	0.062853775	0.880972449	3.551649972	0.24351016	NA	NA
ASV_16s_18541	0.062853775	0.880972449	3.551649972	0.24351016	NA	NA
ASV_16s_18542	0.141955692	-0.078338542	3.550899316	0.771731985	NA	NA
ASV_16s_18543	0.141955692	-0.078338542	3.550899316	0.771731985	NA	NA
ASV_16s_18544	0.141955692	-0.078338542	3.550899316	0.771731985	NA	NA
ASV_16s_18545	0.141955692	-0.078338542	3.550899316	0.771731985	NA	NA
ASV_16s_18546	0.141955692	-0.078338542	3.550899316	0.771731985	NA	NA
ASV_16s_18547	0.141955692	-0.078338542	3.550899316	0.771731985	NA	NA
ASV_16s_18548	0.121047233	-0.078118076	3.550965351	0.699541455	NA	NA
ASV_16s_18549	0.121047233	-0.078118076	3.550965351	0.699541455	NA	NA
ASV_16s_18550	0.121047233	-0.078118076	3.550965351	0.699541455	NA	NA
ASV_16s_18551	0.133954787	0.096619955	3.55093138	1.075720723	0.993512859	NA
ASV_16s_18552	0.136573489	-0.078287828	3.550914395	0.753949196	NA	NA
ASV_16s_18553	0.079331858	0.220208677	3.550941098	0.367990423	NA	NA
ASV_16s_18554	0.084280237	0.220192538	3.550919814	0.390677748	0.999756557	NA
ASV_16s_18555	0.09011548	0.220175789	3.550897695	0.416507833	NA	NA
ASV_16s_18556	0.09011548	0.220175789	3.550897695	0.416507833	NA	NA
ASV_16s_18557	0.09011548	0.220175789	3.550897695	0.416507833	NA	NA
ASV_16s_18558	0.090019504	0.220176033	3.550898036	0.416090855	0.999699438	NA
ASV_16s_18559	0.089935526	0.220176274	3.550898334	0.41572595	NA	NA
ASV_16s_18560	0.153802939	0.310948537	3.551013092	0.789402343	NA	NA
ASV_16s_18561	0.134109183	0.310952618	3.551075077	0.725845006	NA	NA
ASV_16s_18562	0.153316117	0.310948624	3.551014433	0.78790541	NA	NA
ASV_16s_18563	0.094929546	0.595718633	3.550839136	0.381025521	NA	NA
ASV_16s_18564	0.144309975	-0.545010639	3.551304229	0.490411774	NA	NA
ASV_16s_18565	0.181036744	1.247091021	3.551095677	0.546790354	NA	NA
ASV_16s_18566	0.116460244	0.237717396	3.551002071	0.358697872	NA	NA
ASV_16s_18567	0.174001754	0.448564195	3.551453774	0.520751835	0.999366893	NA
ASV_16s_18568	0.190279833	1.262973553	3.55146195	0.431376273	0.99966099	NA
ASV_16s_18569	0.154178157	0.245329325	3.550782168	0.872641443	NA	NA
ASV_16s_18570	0.126002416	-0.066938874	3.551039094	0.801030256	0.997433425	NA
ASV_16s_18571	0.146349496	-0.174024403	3.551041432	0.726284818	0.998125524	NA
ASV_16s_18572	0.153114256	-0.640635032	3.551266089	0.491075767	0.999478507	NA
ASV_16s_18573	0.128975526	-0.060460661	3.551141547	0.825310079	0.997176994	NA
ASV_16s_18574	0.136221112	-0.066528415	3.5510585	0.856060005	0.996828867	NA
ASV_16s_18575	0.136221112	-0.066528415	3.5510585	0.856060005	0.996828867	NA
ASV_16s_18576	0.1478326	0.514690649	3.551568859	0.571526055	0.999140161	NA
ASV_16s_18577	0.206384834	1.032699646	3.551469381	0.740369147	0.998005968	NA
ASV_16s_18578	0.175279831	0.618524907	3.550796976	0.578158387	0.999107007	NA
ASV_16s_18579	0.130370308	0.021768178	3.551236123	0.882563383	0.996507264	NA
ASV_16s_18580	0.137322795	-0.012639437	3.55128476	0.812057296	NA	NA
ASV_16s_18581	0.209125863	-0.015264004	3.551547478	0.693139904	NA	NA

ASV_16s_18582	0.161659033	0.02246907	3.550910118	0.84878833		0.996913588	NA
ASV_16s_18583	0.099547193	0.50159685	3.551560245	0.523405826	NA		NA
ASV_16s_18584	0.224269813	1.159337313	3.550883491	0.671451354	NA		NA
ASV_16s_18585	0.15517983	0.971474438	3.551366128	0.527320787	NA		NA
ASV_16s_18586	0.106741632	0.283897738	3.551728261	0.472244291	NA		NA
ASV_16s_18587	0.170926549	0.279224247	3.551610225	0.631224289	NA		NA
ASV_16s_18588	0.170926549	0.279224247	3.551610225	0.631224289	NA		NA
ASV_16s_18589	0.120298667	0.905381233	3.551522867	0.447619126	NA		NA
ASV_16s_18590	0.276487061	-0.563492757	3.550147073	0.784884019	NA		NA
ASV_16s_18591	0.20514442	-0.861550286	3.550677399	0.548342241		0.999249515	NA
ASV_16s_18592	0.160778273	-0.519687011	3.550672398	0.533354363	NA		NA
ASV_16s_18593	0.160778273	-0.519687011	3.550672398	0.533354363	NA		NA
ASV_16s_18594	0.160778273	-0.519687011	3.550672398	0.533354363	NA		NA
ASV_16s_18595	0.178272716	1.20621666	3.550883354	0.537789426	NA		NA
ASV_16s_18596	0.178272716	1.20621666	3.550883354	0.537789426	NA		NA
ASV_16s_18597	0.178272716	1.20621666	3.550883354	0.537789426	NA		NA
ASV_16s_18598	0.167272981	1.116199621	3.551006894	0.721647576		0.998163821	NA
ASV_16s_18599	0.101585604	-0.077830781	3.551051032	0.624118007	NA		NA
ASV_16s_18600	0.131481477	-0.067215067	3.551058894	0.797629524	NA		NA
ASV_16s_18601	0.26051674	0.411694246	3.55015271	1.406733336		0.985364136	NA
ASV_16s_18602	0.247858258	0.639017925	3.550332718	1.279203317		0.988987495	NA
ASV_16s_18603	0.224235191	-0.028896513	3.550881772	1.050923538	NA		NA
ASV_16s_18604	0.242820901	0.613043126	3.550671913	1.368714794		0.986510287	NA
ASV_16s_18605	0.189966975	0.379058303	3.550698899	0.987871968	NA		NA
ASV_16s_18606	0.160237422	-0.302634181	3.551036936	0.714753928	NA		NA
ASV_16s_18607	0.196639507	0.475949191	3.551002764	1.308166567		0.988219558	NA
ASV_16s_18608	0.134027965	-0.028170359	3.551123538	0.804379707	NA		NA
ASV_16s_18609	0.134027965	-0.028170359	3.551123538	0.804379707	NA		NA
ASV_16s_18610	0.134027965	-0.028170359	3.551123538	0.804379707	NA		NA
ASV_16s_18611	0.134027965	-0.028170359	3.551123538	0.804379707	NA		NA
ASV_16s_18612	0.16986928	0.479598997	3.551073628	1.189169589		0.991174131	NA
ASV_16s_18613	0.153144007	-0.0733075	3.550906772	0.831250983	NA		NA
ASV_16s_18614	0.111850197	-0.072801979	3.551050718	0.689074931	NA		NA
ASV_16s_18615	0.111850197	-0.072801979	3.551050718	0.689074931	NA		NA
ASV_16s_18616	0.111850197	-0.072801979	3.551050718	0.689074931	NA		NA
ASV_16s_18617	0.109840436	-0.072767721	3.551060461	0.681252667	NA		NA
ASV_16s_18618	0.109840436	-0.072767721	3.551060461	0.681252667	NA		NA
ASV_16s_18619	0.109840436	-0.072767721	3.551060461	0.681252667	NA		NA
ASV_16s_18620	0.14621687	0.334851436	3.550910148	0.886773462		0.996454303	NA
ASV_16s_18621	0.186582557	0.327039227	3.550570339	1.05974051		0.993806425	NA
ASV_16s_18622	0.154285178	-0.073317625	3.550903905	0.831755504		0.99710621	NA
ASV_16s_18623	0.187306985	0.424335534	3.551070957	0.936020431	NA		NA
ASV_16s_18624	0.136285704	-0.073138621	3.550955079	0.777142559	NA		NA
ASV_16s_18625	0.136285704	-0.073138621	3.550955079	0.777142559	NA		NA
ASV_16s_18626	0.16203782	0.334510999	3.550758609	0.946760207		0.995642263	NA
ASV_16s_18627	0.152888712	0.360272331	3.551256438	0.848248387	NA		NA
ASV_16s_18628	0.152888712	0.360272331	3.551256438	0.848248387	NA		NA
ASV_16s_18629	0.148382074	0.339142381	3.551535298	0.610691119		0.998931849	NA
ASV_16s_18630	0.181747407	0.344681544	3.551367453	0.6772506		0.998504491	NA
ASV_16s_18631	0.135912783	0.360247387	3.551338267	0.79355626	NA		NA
ASV_16s_18632	0.135912783	0.360247387	3.551338267	0.79355626	NA		NA
ASV_16s_18633	0.135912783	0.360247387	3.551338267	0.79355626	NA		NA
ASV_16s_18634	0.135912783	0.360247387	3.551338267	0.79355626	NA		NA
ASV_16s_18635	0.135912783	0.360247387	3.551338267	0.79355626	NA		NA
ASV_16s_18636	0.135912783	0.360247387	3.551338267	0.79355626	NA		NA
ASV_16s_18637	0.135912783	0.360247387	3.551338267	0.79355626	NA		NA
ASV_16s_18638	0.176101456	0.315939793	3.550994458	0.877042059	NA		NA
ASV_16s_18639	0.184044263	0.315939626	3.550977023	0.896490876		0.996330074	NA
ASV_16s_18640	0.16244315	0.315939839	3.551028473	0.837106271		0.997046573	NA
ASV_16s_18641	0.151061229	0.315939885	3.55106147	0.802337388		0.997420026	NA
ASV_16s_18642	0.15222156	0.315939955	3.551057871	0.807324427	NA		NA
ASV_16s_18643	0.147088626	0.315939902	3.551074169	0.79123212		0.997532406	NA
ASV_16s_18644	0.146746431	0.315939904	3.551075295	0.790144104		0.99754324	NA
ASV_16s_18645	0.156949016	0.610050687	3.551103908	0.812126434		0.997318221	NA
ASV_16s_18646	0.141955692	0.31594001	3.551091636	0.774721529	NA		NA
ASV_16s_18647	0.141955692	0.31594001	3.551091636	0.774721529	NA		NA
ASV_16s_18648	0.141955692	0.31594001	3.551091636	0.774721529	NA		NA

ASV_16s_18649	0.141955692	0.31594001	3.551091636	0.774721529	NA	NA
ASV_16s_18650	0.141955692	0.31594001	3.551091636	0.774721529	NA	NA
ASV_16s_18651	0.141955692	0.31594001	3.551091636	0.774721529	NA	NA
ASV_16s_18652	0.141955692	0.31594001	3.551091636	0.774721529	NA	NA
ASV_16s_18653	0.141955692	0.31594001	3.551091636	0.774721529	NA	NA
ASV_16s_18654	0.141955692	0.31594001	3.551091636	0.774721529	NA	NA
ASV_16s_18655	0.141955692	0.31594001	3.551091636	0.774721529	NA	NA
ASV_16s_18656	0.15866184	0.315939853	3.551038985	0.822817629	0.997204059	NA
ASV_16s_18657	0.171191452	0.315939808	3.551006164	0.859218861	0.996791595	NA
ASV_16s_18658	0.191136398	0.596090564	3.550693885	0.685841917	NA	NA
ASV_16s_18659	0.135189662	0.687351037	3.551044517	0.668854441	NA	NA
ASV_16s_18660	0.216009759	1.100335301	3.550788674	0.683937474	0.998456132	NA
ASV_16s_18661	0.300473898	0.734371749	3.550934065	1.13805397	NA	NA
ASV_16s_18662	0.181937169	1.090268696	3.551151427	0.651689964	0.998680021	NA
ASV_16s_18663	0.187789937	0.733637985	3.551147297	0.88589452	NA	NA
ASV_16s_18664	0.187789937	0.733637985	3.551147297	0.88589452	NA	NA
ASV_16s_18665	0.187789937	0.733637985	3.551147297	0.88589452	NA	NA
ASV_16s_18666	0.187789937	0.733637985	3.551147297	0.88589452	NA	NA
ASV_16s_18667	0.187789937	0.733637985	3.551147297	0.88589452	NA	NA
ASV_16s_18668	0.187789937	0.733637985	3.551147297	0.88589452	NA	NA
ASV_16s_18669	0.174341191	1.041251641	3.551063954	0.630071185	NA	NA
ASV_16s_18670	0.093022809	0.701351127	3.551578382	0.578807747	NA	NA
ASV_16s_18671	0.093022809	0.701351127	3.551578382	0.578807747	NA	NA
ASV_16s_18672	0.1745838	0.692731196	3.550976408	0.810494653	NA	NA
ASV_16s_18673	0.1745838	0.692731196	3.550976408	0.810494653	NA	NA
ASV_16s_18674	0.1745838	0.692731196	3.550976408	0.810494653	NA	NA
ASV_16s_18675	0.156094539	0.692560229	3.551026272	0.758206204	0.99784745	NA
ASV_16s_18676	0.177721155	0.692755194	3.550968971	0.818949052	0.997245729	NA
ASV_16s_18677	0.153919332	0.69253763	3.551032918	0.751639697	0.99790674	NA
ASV_16s_18678	0.177721155	0.692755194	3.550968971	0.818949052	0.997245729	NA
ASV_16s_18679	0.151744124	0.692514393	3.551039752	0.744913204	0.997966345	NA
ASV_16s_18680	0.178546775	0.69276171	3.550967057	0.821146586	0.997222109	NA
ASV_16s_18681	0.200362854	0.692911029	3.550922167	0.87599758	0.996588827	NA
ASV_16s_18682	0.179537518	0.692770732	3.550964784	0.823789769	NA	NA
ASV_16s_18683	0.179537518	0.692770732	3.550964784	0.823789769	NA	NA
ASV_16s_18684	0.165757515	0.692653533	3.550998842	0.786067866	0.997583549	NA
ASV_16s_18685	0.141955692	0.69240288	3.551073069	0.71514795	NA	NA
ASV_16s_18686	0.141955692	0.69240288	3.551073069	0.71514795	NA	NA
ASV_16s_18687	0.141955692	0.69240288	3.551073069	0.71514795	NA	NA
ASV_16s_18688	0.141955692	0.69240288	3.551073069	0.71514795	NA	NA
ASV_16s_18689	0.141955692	0.69240288	3.551073069	0.71514795	NA	NA
ASV_16s_18690	0.141955692	0.69240288	3.551073069	0.71514795	NA	NA
ASV_16s_18691	0.159711618	0.692596368	3.551015645	0.765173744	0.997783336	NA
ASV_16s_18692	0.208540414	0.692963775	3.550907742	0.896235261	NA	NA
ASV_16s_18693	0.208540414	0.692963775	3.550907742	0.896235261	NA	NA
ASV_16s_18694	0.20816306	0.692961591	3.550908383	0.895347475	NA	NA
ASV_16s_18695	0.118309026	0.296200825	3.5518698	0.496900464	NA	NA
ASV_16s_18696	0.118309026	0.296200825	3.5518698	0.496900464	NA	NA
ASV_16s_18697	0.118309026	0.296200825	3.5518698	0.496900464	NA	NA
ASV_16s_18698	0.118309026	0.296200825	3.5518698	0.496900464	NA	NA
ASV_16s_18699	0.118309026	0.296200825	3.5518698	0.496900464	NA	NA
ASV_16s_18700	0.118309026	0.296200825	3.5518698	0.496900464	NA	NA
ASV_16s_18701	0.118309026	0.296200825	3.5518698	0.496900464	NA	NA
ASV_16s_18702	0.118309026	0.296200825	3.5518698	0.496900464	NA	NA
ASV_16s_18703	0.118309026	0.296200825	3.5518698	0.496900464	NA	NA
ASV_16s_18704	0.118309026	0.296200825	3.5518698	0.496900464	NA	NA
ASV_16s_18705	0.118309026	0.296200825	3.5518698	0.496900464	NA	NA
ASV_16s_18706	0.118309026	0.296200825	3.5518698	0.496900464	NA	NA
ASV_16s_18707	0.118309026	0.296200825	3.5518698	0.496900464	NA	NA
ASV_16s_18708	0.126061782	0.060042862	3.551592899	0.686734779	0.998435597	NA
ASV_16s_18709	0.12902345	0.041828701	3.55154638	0.703876258	0.998305776	NA
ASV_16s_18710	0.120121458	0.041909897	3.551603735	0.697633805	0.998353852	NA
ASV_16s_18711	0.120380376	0.012376058	3.551567625	0.716674768	0.998204309	NA
ASV_16s_18712	0.120639295	-0.015505081	3.551533915	0.730640028	0.998089077	NA
ASV_16s_18713	0.120639295	-0.015505081	3.551533915	0.730640028	0.998089077	NA
ASV_16s_18714	0.118360362	-0.002367496	3.551562552	0.719535569	0.99818109	NA
ASV_16s_18715	0.120898214	-0.042140964	3.551500551	0.742351023	0.99798875	NA

ASV_16s_18716	0.118377474	-0.078671462	3.551479564	0.745717982	0.997959273	NA
ASV_16s_18717	0.136915641	-0.160084589	3.551246035	0.831550915	0.997108475	NA
ASV_16s_18718	0.121416052	-0.093209267	3.55143886	0.759078704	0.997839489	NA
ASV_16s_18719	0.118377474	-0.078671462	3.551479564	0.745717982	0.997959273	NA
ASV_16s_18720	0.121416052	-0.093209267	3.55143886	0.759078704	0.997839489	NA
ASV_16s_18721	0.121416052	-0.093209267	3.55143886	0.759078704	0.997839489	NA
ASV_16s_18722	0.121674971	-0.117605312	3.551413161	0.764325137	0.997791212	NA
ASV_16s_18723	0.121933889	-0.141686715	3.551387735	0.767245089	0.997764036	NA
ASV_16s_18724	0.119908171	-0.132797049	3.551414476	0.758998398	0.997840223	NA
ASV_16s_18725	0.128963324	-0.170879242	3.551300958	0.796641089	0.997478081	NA
ASV_16s_18726	0.119908171	-0.132797049	3.551414476	0.758998398	0.997840223	NA
ASV_16s_18727	0.121933889	-0.141686715	3.551387735	0.767245089	0.997764036	NA
ASV_16s_18728	0.137005618	-0.201197926	3.551212949	0.831842847	0.997105243	NA
ASV_16s_18729	0.121933889	-0.141686715	3.551387735	0.767245089	0.997764036	NA
ASV_16s_18730	0.122192808	-0.165350052	3.551364458	0.767844789	0.997758428	NA
ASV_16s_18731	0.122192808	-0.165350052	3.551364458	0.767844789	0.997758428	NA
ASV_16s_18732	0.120932438	-0.182551033	3.551362603	0.76057367	0.997825804	NA
ASV_16s_18733	0.121185653	-0.183566244	3.551359483	0.761517429	0.997817136	NA
ASV_16s_18734	0.154738069	-0.311506764	3.551013848	0.886921607	0.99645243	NA
ASV_16s_18735	0.122969565	-0.234266131	3.551311238	0.757307113	0.997855633	NA
ASV_16s_18736	0.122969565	-0.234266131	3.551311238	0.757307113	0.997855633	NA
ASV_16s_18737	0.122969565	-0.234266131	3.551311238	0.757307113	0.997855633	NA
ASV_16s_18738	0.118417403	-0.239878932	3.551350284	0.734578026	0.99805572	NA
ASV_16s_18739	0.129764922	-0.300264483	3.551220164	0.759390956	0.997836636	NA
ASV_16s_18740	0.121461684	-0.273113051	3.551304409	0.734196335	0.99805897	NA
ASV_16s_18741	0.12070204	-0.270502166	3.551312702	0.731947059	0.998078048	NA
ASV_16s_18742	0.119694885	-0.289336241	3.551313849	0.717314012	0.998199138	NA
ASV_16s_18743	0.136921565	-0.363186623	3.551146458	0.750504191	0.997916882	NA
ASV_16s_18744	0.120465937	-0.335732193	3.551287922	0.684639063	0.998450999	NA
ASV_16s_18745	0.123003789	-0.365877326	3.55126009	0.671345213	0.998546349	NA
ASV_16s_18746	0.042294052	-0.142449717	3.551845428	0.251418783	0.999945073	NA
ASV_16s_18747	0.164593566	-0.576438268	3.550967698	0.62086604	0.998872664	NA
ASV_16s_18748	0.161410804	-0.570420665	3.55098664	0.616164288	NA	NA
ASV_16s_18749	0.161410804	-0.570420665	3.55098664	0.616164288	NA	NA
ASV_16s_18750	0.161410804	-0.570420665	3.55098664	0.616164288	NA	NA
ASV_16s_18751	0.161410804	-0.570420665	3.55098664	0.616164288	NA	NA
ASV_16s_18752	0.163674474	-0.574742831	3.550973092	0.620904221	0.998872438	NA
ASV_16s_18753	0.15445612	-0.556671371	3.551030018	0.601283654	0.998984678	NA
ASV_16s_18754	0.164196859	-0.575719708	3.550969983	0.621987458	0.99886601	NA
ASV_16s_18755	0.153528829	-0.554795016	3.551036031	0.59926258	0.998995793	NA
ASV_16s_18756	0.156891828	-0.561549228	3.551014491	0.606474289	0.998955752	NA
ASV_16s_18757	0.154225343	-0.556204477	3.551031512	0.600618237	0.998988347	NA
ASV_16s_18758	0.159501663	-0.566699916	3.550998262	0.612120014	0.998923666	NA
ASV_16s_18759	0.166460528	-0.579918226	3.550956692	0.626647554	NA	NA
ASV_16s_18760	0.166460528	-0.579918226	3.550956692	0.626647554	NA	NA
ASV_16s_18761	0.166460528	-0.579918226	3.550956692	0.626647554	NA	NA
ASV_16s_18762	0.166460528	-0.579918226	3.550956692	0.626647554	NA	NA
ASV_16s_18763	0.160720562	-0.569077258	3.550990827	0.614701703	0.998908776	NA
ASV_16s_18764	0.147965082	-0.54331081	3.551073331	0.586967499	NA	NA
ASV_16s_18765	0.143191999	-0.53308786	3.551107264	0.573168626	0.999132029	NA
ASV_16s_18766	0.138557458	-0.52182516	3.551136431	0.561828675	0.999187126	NA
ASV_16s_18767	0.132378069	-0.50625596	3.551176183	0.546963756	0.999255703	NA
ASV_16s_18768	0.12718598	-0.492660896	3.551211569	0.535454179	0.999306029	NA
ASV_16s_18769	0.125817672	-0.488971597	3.551221142	0.533684797	NA	NA
ASV_16s_18770	0.125817672	-0.488971597	3.551221142	0.533684797	NA	NA
ASV_16s_18771	0.125817672	-0.488971597	3.551221142	0.533684797	NA	NA
ASV_16s_18772	0.125817672	-0.488971597	3.551221142	0.533684797	NA	NA
ASV_16s_18773	0.125817672	-0.488971597	3.551221142	0.533684797	NA	NA
ASV_16s_18774	0.120753377	-0.474901174	3.551258248	0.520490259	0.999367942	NA
ASV_16s_18775	0.118474444	-0.468394104	3.551275731	0.514426526	NA	NA
ASV_16s_18776	0.118474444	-0.468394104	3.551275731	0.514426526	NA	NA
ASV_16s_18777	0.118474444	-0.468394104	3.551275731	0.514426526	NA	NA
ASV_16s_18778	0.118474444	-0.468394104	3.551275731	0.514426526	NA	NA
ASV_16s_18779	0.118474444	-0.468394104	3.551275731	0.514426526	NA	NA
ASV_16s_18780	0.118474444	-0.468394104	3.551275731	0.514426526	NA	NA
ASV_16s_18781	0.118474444	-0.468394104	3.551275731	0.514426526	NA	NA
ASV_16s_18782	0.118474444	-0.468394104	3.551275731	0.514426526	NA	NA

ASV_16s_18783	0.033481613	-0.146414423	3.551190545	0.135282125	NA	NA
ASV_16s_18785	0.141335025	-0.013133513	3.551358777	0.763923479	0.997794933	NA
ASV_16s_18786	0.144805392	-0.501688739	3.551224064	0.565384023	NA	NA
ASV_16s_18787	0.113869406	-0.250269788	3.551092957	0.37237855	NA	NA
ASV_16s_18788	0.154720722	-0.52680561	3.551117327	0.605989482	0.998958477	NA
ASV_16s_18789	0.168139832	-0.450151875	3.551166	0.772851178	0.997711245	NA
ASV_16s_18790	0.123587928	-0.11236234	3.551754245	0.549993607	0.999242056	NA
ASV_16s_18791	0.122550189	-0.466060848	3.55133151	0.466193997	NA	NA
ASV_16s_18792	0.161292603	-0.21307212	3.550861167	0.623604422	NA	NA
ASV_16s_18793	0.136753138	-0.084161537	3.551646561	0.616646348	0.998897469	NA
ASV_16s_18794	0.16172011	-0.538767443	3.550949271	0.68125267	0.998475672	NA
ASV_16s_18795	0.153141912	-0.511991712	3.55103009	0.655644292	0.998653819	NA
ASV_16s_18796	0.155404559	-0.450498974	3.551335693	0.503989899	0.99943174	NA
ASV_16s_18797	0.124831374	0.639495255	3.551834301	0.57885082	0.999103497	NA
ASV_16s_18798	0.158702379	-0.03931822	3.551848697	0.525273103	0.999348579	NA
ASV_16s_18799	0.181821027	-0.987467004	3.550819521	0.298175523	0.999902007	NA
ASV_16s_18800	0.159101485	-0.482232499	3.550906506	0.717142135	0.998200529	NA
ASV_16s_18801	0.149044546	-0.43363447	3.55098288	0.692146044	0.998395358	NA
ASV_16s_18802	0.148283848	-0.416033775	3.550986688	0.695023666	0.998373681	NA
ASV_16s_18803	0.124974897	0.033647925	3.551183108	0.591542655	0.999037497	NA
ASV_16s_18804	0.123778609	0.311016298	3.551883439	0.398506013	0.999739839	NA
ASV_16s_18805	0.148081185	-0.392716855	3.550982461	0.70330393	0.998310222	NA
ASV_16s_18806	0.133686706	-0.090775579	3.551232467	0.698959517	0.998343719	NA
ASV_16s_18807	0.128092548	0.674069489	3.551580412	0.624463499	0.998851223	NA
ASV_16s_18808	0.156648471	-0.383114156	3.550903743	0.744625138	0.997968872	NA
ASV_16s_18809	0.156648471	-0.383114156	3.550903743	0.744625138	0.997968872	NA
ASV_16s_18810	0.156648471	-0.383114156	3.550903743	0.744625138	0.997968872	NA
ASV_16s_18811	0.158033119	-0.383120372	3.550892981	0.750600043	0.997916027	NA
ASV_16s_18812	0.13816611	0.677707917	3.551384962	0.665238923	0.998588801	NA
ASV_16s_18813	0.144875422	-0.350574425	3.551010903	0.701462366	0.998324475	NA
ASV_16s_18814	0.142220179	-0.314839488	3.551010991	0.696224413	0.998364579	NA
ASV_16s_18815	0.127475072	0.148284409	3.551085552	0.617049257	0.998895117	NA
ASV_16s_18816	0.132560208	0.304649015	3.551513762	0.464243477	0.999567161	NA
ASV_16s_18817	0.153617404	0.18323399	3.550019104	0.685484429	0.998444798	NA
ASV_16s_18818	0.190696868	0.978848669	3.551335874	0.587238996	0.999060231	NA
ASV_16s_18819	0.145178305	0.485224774	3.551189695	0.813782629	0.99730074	NA
ASV_16s_18820	0.177888094	-0.074089698	3.551290506	0.644323431	0.99872792	NA
ASV_16s_18821	0.131259632	-0.081375586	3.551067485	0.678217655	NA	NA
ASV_16s_18822	0.170630057	0.728267628	3.551280013	0.812523278	NA	NA
ASV_16s_18823	0.151590911	0.050641278	3.551054878	0.691553137	0.9983998	NA
ASV_16s_18824	0.145833887	-0.080951355	3.550967544	0.740339471	NA	NA
ASV_16s_18825	0.151914555	0.4807409	3.551041644	0.712623209	0.998236855	NA
ASV_16s_18826	0.155418998	1.059429831	3.551316636	0.545813465	NA	NA
ASV_16s_18827	0.172519799	0.153046348	3.550812758	0.541814124	0.999278515	NA
ASV_16s_18828	0.164674892	1.152008505	3.551180571	0.570190913	NA	NA
ASV_16s_18829	0.139129731	1.394904066	3.551326136	0.263408554	0.999935644	NA
ASV_16s_18830	0.131162813	1.073043229	3.55137075	0.494689016	NA	NA
ASV_16s_18831	0.140075144	1.096199647	3.551315631	0.516032334	NA	NA
ASV_16s_18832	0.147587735	1.114697357	3.551273295	0.533315875	NA	NA
ASV_16s_18833	0.168997107	1.486096732	3.550922902	0.325460381	0.999868263	NA
ASV_16s_18834	0.135411559	1.084255496	3.551343762	0.504970658	NA	NA
ASV_16s_18835	0.135411559	1.084255496	3.551343762	0.504970658	NA	NA
ASV_16s_18836	0.135411559	1.084255496	3.551343762	0.504970658	NA	NA
ASV_16s_18837	0.162490781	0.85423359	3.551156152	0.866161396	0.996708678	NA
ASV_16s_18838	0.128379463	0.342818056	3.551888269	0.523469124	NA	NA
ASV_16s_18839	0.128379463	0.342818056	3.551888269	0.523469124	NA	NA
ASV_16s_18840	0.135450209	0.801720843	3.551750871	0.451350917	0.999605839	NA
ASV_16s_18841	0.155418998	0.351638736	3.551779086	0.585844702	NA	NA
ASV_16s_18842	0.155418998	0.351638736	3.551779086	0.585844702	NA	NA
ASV_16s_18843	0.155418998	0.351638736	3.551779086	0.585844702	NA	NA
ASV_16s_18844	0.120036194	0.042519607	3.551607068	0.696271753	0.998364219	NA
ASV_16s_18845	0.177310835	-0.499158797	3.551116857	0.40235753	0.999731331	NA
ASV_16s_18846	0.160140991	0.353925897	3.551751478	0.595704083	NA	NA
ASV_16s_18847	0.159658761	-0.511972319	3.551020686	0.59682406	0.999009095	NA
ASV_16s_18848	0.166460528	-0.519934426	3.550953582	0.612024564	NA	NA
ASV_16s_18849	0.166460528	-0.519934426	3.550953582	0.612024564	NA	NA
ASV_16s_18850	0.268674825	-0.865986835	3.550375918	0.559964945	NA	NA

ASV_16s_18851	0.19482888	0.562260634	3.551115596	0.575474449		0.999120526	NA
ASV_16s_18852	0.14302894	-0.488641384	3.551186705	0.563650626	NA		NA
ASV_16s_18853	0.175082019	-0.854254749	3.551047585	0.322869841		0.999871768	NA
ASV_16s_18854	0.17680423	1.486329633	3.550972019	0.360955016		0.99981332	NA
ASV_16s_18855	0.147311114	1.149745537	3.551035805	0.473997758	NA		NA
ASV_16s_18856	0.076882211	0.939864293	3.551524819	0.288566467	NA		NA
ASV_16s_18857	0.131803193	1.113385581	3.551115173	0.438057205	NA		NA
ASV_16s_18858	0.131803193	1.113385581	3.551115173	0.438057205	NA		NA
ASV_16s_18859	0.131803193	1.113385581	3.551115173	0.438057205	NA		NA
ASV_16s_18860	0.166602669	0.377355179	3.551356193	0.543819981	NA		NA
ASV_16s_18861	0.166602669	0.377355179	3.551356193	0.543819981	NA		NA
ASV_16s_18862	0.132898322	0.170054238	3.551241081	0.674871324		0.998521451	NA
ASV_16s_18863	0.151134394	0.370311251	3.551441581	0.512113502	NA		NA
ASV_16s_18864	0.137223835	0.36419234	3.551525156	0.481458635	NA		NA
ASV_16s_18865	0.113276656	0.352767087	3.551635118	0.422693592	NA		NA
ASV_16s_18866	0.122561327	0.357450924	3.551593378	0.446361696	NA		NA
ASV_16s_18867	0.122561327	0.357450924	3.551593378	0.446361696	NA		NA
ASV_16s_18868	0.177671636	0.877142864	3.550748508	0.867510542		0.996692404	NA
ASV_16s_18869	0.042674809	0.340327476	3.551901853	0.193175661		0.999977663	NA
ASV_16s_18870	0.068860064	-0.301785679	3.551484369	0.293101316	NA		NA
ASV_16s_18871	0.068860064	-0.301785679	3.551484369	0.293101316	NA		NA
ASV_16s_18872	0.07664415	-0.333208294	3.551428417	0.319523563	NA		NA
ASV_16s_18873	0.07664415	-0.333208294	3.551428417	0.319523563	NA		NA
ASV_16s_18874	0.07664415	-0.333208294	3.551428417	0.319523563	NA		NA
ASV_16s_18875	0.060758649	0.87147281	3.551668464	0.236438493		0.999955442	NA
ASV_16s_18876	0.150327833	1.15608868	3.551020378	0.480650277	NA		NA
ASV_16s_18877	0.137223835	-0.078294336	3.550912546	0.756027061	NA		NA
ASV_16s_18878	0.137223835	-0.078294336	3.550912546	0.756027061	NA		NA
ASV_16s_18879	0.128860452	-0.078208023	3.550938229	0.727373123		0.99811646	NA
ASV_16s_18880	0.117012325	-0.07806494	3.550980832	0.684491914	NA		NA
ASV_16s_18881	0.117012325	-0.07806494	3.550980832	0.684491914	NA		NA
ASV_16s_18882	0.117012325	-0.07806494	3.550980832	0.684491914	NA		NA
ASV_16s_18883	0.117012325	-0.07806494	3.550980832	0.684491914	NA		NA
ASV_16s_18884	0.117012325	-0.07806494	3.550980832	0.684491914	NA		NA
ASV_16s_18885	0.117012325	-0.07806494	3.550980832	0.684491914	NA		NA
ASV_16s_18886	0.087111631	0.220184177	3.550908742	0.403254835	NA		NA
ASV_16s_18887	0.087111631	0.220184177	3.550908742	0.403254835	NA		NA
ASV_16s_18888	0.086937675	0.220184678	3.550909403	0.402483425	NA		NA
ASV_16s_18889	0.086937675	0.220184678	3.550909403	0.402483425	NA		NA
ASV_16s_18890	0.086937675	0.220184678	3.550909403	0.402483425	NA		NA
ASV_16s_18891	0.140060831	0.310951238	3.551054561	0.745620392	NA		NA
ASV_16s_18892	0.135793579	0.310952032	3.55106912	0.731423797		0.998082468	NA
ASV_16s_18893	0.151185848	0.310949032	3.551020444	0.781207062	NA		NA
ASV_16s_18894	0.149952634	0.31094926	3.551023978	0.777359226		0.997668203	NA
ASV_16s_18895	0.129638877	0.310953644	3.551091793	0.710467407	NA		NA
ASV_16s_18896	0.129638877	0.310953644	3.551091793	0.710467407	NA		NA
ASV_16s_18897	0.14820558	0.310949591	3.551029084	0.771871434	NA		NA
ASV_16s_18898	0.091765228	0.59569346	3.550849087	0.368352995	NA		NA
ASV_16s_18899	0.091765228	0.59569346	3.550849087	0.368352995	NA		NA
ASV_16s_18900	0.091765228	0.59569346	3.550849087	0.368352995	NA		NA
ASV_16s_18901	0.15506217	0.687544751	3.550982159	0.730470769	NA		NA
ASV_16s_18902	0.180526106	0.522552505	3.55157413	0.447513624		0.999616878	NA
ASV_16s_18903	0.189875552	1.396046023	3.550984621	0.331224796		0.999860225	NA
ASV_16s_18904	0.175559554	-0.679523491	3.551072324	0.467072932		0.999558339	NA
ASV_16s_18905	0.172641012	0.728309693	3.551417559	0.754030616		0.997885279	NA
ASV_16s_18906	0.124940619	-0.056702768	3.551197452	0.798108585		0.997463208	NA
ASV_16s_18907	0.180086483	1.371877019	3.551181021	0.337330692		0.999851348	NA
ASV_16s_18908	0.125436137	-0.053296706	3.551230077	0.796532545		0.997479179	NA
ASV_16s_18909	0.149443038	0.893445887	3.551522746	0.464556998		0.999566189	NA
ASV_16s_18910	0.139725466	1.040427055	3.551276563	0.496699937		0.999458475	NA
ASV_16s_18911	0.152332826	0.364867341	3.551656798	0.547379639		0.99925384	NA
ASV_16s_18912	0.144725104	-0.382831956	3.550906962	0.616719883	NA		NA
ASV_16s_18913	0.182272556	0.329560523	3.551857104	0.346712369		0.99983696	NA
ASV_16s_18914	0.143010414	0.494733085	3.551116482	0.619181565	NA		NA
ASV_16s_18915	0.194048092	1.125773565	3.551071029	0.621378992	NA		NA
ASV_16s_18916	0.058580619	0.291232135	3.551887309	0.309056917		0.999889375	NA
ASV_16s_18917	0.209401129	0.279565026	3.551522188	0.704482952	NA		NA

ASV_16s_18918	0.037880314	-0.103849711	3.551824302	0.22346046	0.999963247	NA
ASV_16s_18919	0.244158809	1.357969158	3.550808478	0.542140937	0.999277082	NA
ASV_16s_18920	0.267270825	-0.563292349	3.550197342	0.785087568	NA	NA
ASV_16s_18921	0.226265391	0.344974663	3.551478053	0.534036493	0.999312063	NA
ASV_16s_18922	0.161206301	0.328457191	3.551465156	0.462810765	NA	NA
ASV_16s_18923	0.172330292	1.1965397	3.550910675	0.526223771	NA	NA
ASV_16s_18924	0.172330292	1.1965397	3.550910675	0.526223771	NA	NA
ASV_16s_18925	0.195550618	1.099978625	3.550578484	0.819592356	0.997238828	NA
ASV_16s_18926	0.167468209	0.801869055	3.550992625	0.748161231	0.997937705	NA
ASV_16s_18927	0.153226111	0.6870436	3.551082275	0.705674521	NA	NA
ASV_16s_18928	0.098199417	-0.077769356	3.551069427	0.610036122	NA	NA
ASV_16s_18929	0.098199417	-0.077769356	3.551069427	0.610036122	NA	NA
ASV_16s_18930	0.098199417	-0.077769356	3.551069427	0.610036122	NA	NA
ASV_16s_18931	0.098199417	-0.077769356	3.551069427	0.610036122	NA	NA
ASV_16s_18932	0.228975444	0.738203068	3.550984266	1.130421316	0.992441713	NA
ASV_16s_18933	0.210798046	0.348010439	3.550380554	1.184260807	0.991284785	NA
ASV_16s_18934	0.215749498	0.325509427	3.550926704	1.523732017	0.981476666	NA
ASV_16s_18935	0.216760685	-0.028859098	3.550894238	1.033521118	NA	NA
ASV_16s_18936	0.233715334	0.218767945	3.550879899	1.562083832	0.980082155	NA
ASV_16s_18937	0.178363585	0.325837258	3.55065713	1.043862232	0.994089608	NA
ASV_16s_18938	0.18448157	0.371086141	3.550669193	0.990717191	0.994976894	NA
ASV_16s_18939	0.16657438	0.263585019	3.550980431	0.962044152	0.9954178	NA
ASV_16s_18940	0.129560366	-0.028108449	3.551144228	0.788864707	NA	NA
ASV_16s_18941	0.129560366	-0.028108449	3.551144228	0.788864707	NA	NA
ASV_16s_18942	0.129560366	-0.028108449	3.551144228	0.788864707	NA	NA
ASV_16s_18943	0.129560366	-0.028108449	3.551144228	0.788864707	NA	NA
ASV_16s_18944	0.129560366	-0.028108449	3.551144228	0.788864707	NA	NA
ASV_16s_18945	0.148039207	-0.073260134	3.550920282	0.815282359	NA	NA
ASV_16s_18946	0.148039207	-0.073260134	3.550920282	0.815282359	NA	NA
ASV_16s_18947	0.108121857	-0.072737461	3.551069126	0.67439071	NA	NA
ASV_16s_18948	0.108121857	-0.072737461	3.551069126	0.67439071	NA	NA
ASV_16s_18949	0.108121857	-0.072737461	3.551069126	0.67439071	NA	NA
ASV_16s_18950	0.108121857	-0.072737461	3.551069126	0.67439071	NA	NA
ASV_16s_18951	0.106179088	-0.072702054	3.551079199	0.666686828	NA	NA
ASV_16s_18952	0.106179088	-0.072702054	3.551079199	0.666686828	NA	NA
ASV_16s_18953	0.106179088	-0.072702054	3.551079199	0.666686828	NA	NA
ASV_16s_18954	0.106179088	-0.072702054	3.551079199	0.666686828	NA	NA
ASV_16s_18955	0.106179088	-0.072702054	3.551079199	0.666686828	NA	NA
ASV_16s_18956	0.154061348	0.316613847	3.550809933	0.911252645	0.996136004	NA
ASV_16s_18957	0.140503742	0.728682985	3.551200766	0.809732042	0.997343362	NA
ASV_16s_18958	0.138681572	0.327769931	3.551248924	0.642409667	NA	NA
ASV_16s_18959	0.158823291	0.334249846	3.551283751	0.649750019	0.99869275	NA
ASV_16s_18960	0.131382357	0.36023965	3.551363704	0.778079426	NA	NA
ASV_16s_18961	0.169425603	0.644268358	3.551154594	1.099672241	0.993056567	NA
ASV_16s_18962	0.131382357	0.36023965	3.551363704	0.778079426	NA	NA
ASV_16s_18963	0.131382357	0.36023965	3.551363704	0.778079426	NA	NA
ASV_16s_18964	0.131382357	0.36023965	3.551363704	0.778079426	NA	NA
ASV_16s_18965	0.131382357	0.36023965	3.551363704	0.778079426	NA	NA
ASV_16s_18966	0.131382357	0.36023965	3.551363704	0.778079426	NA	NA
ASV_16s_18967	0.131382357	0.36023965	3.551363704	0.778079426	NA	NA
ASV_16s_18968	0.131382357	0.36023965	3.551363704	0.778079426	NA	NA
ASV_16s_18969	0.131382357	0.36023965	3.551363704	0.778079426	NA	NA
ASV_16s_18970	0.131382357	0.36023965	3.551363704	0.778079426	NA	NA
ASV_16s_18971	0.131382357	0.36023965	3.551363704	0.778079426	NA	NA
ASV_16s_18972	0.131382357	0.36023965	3.551363704	0.778079426	NA	NA
ASV_16s_18973	0.131382357	0.36023965	3.551363704	0.778079426	NA	NA
ASV_16s_18974	0.170231408	0.315939831	3.551008445	0.860521907	NA	NA
ASV_16s_18975	0.170231408	0.315939831	3.551008445	0.860521907	NA	NA
ASV_16s_18976	0.15543491	0.315939886	3.551048266	0.8147718	0.997290264	NA
ASV_16s_18977	0.154296718	0.315939891	3.551051642	0.811265828	0.997327275	NA
ASV_16s_18978	0.153158525	0.315939896	3.551055068	0.807762035	0.99736393	NA
ASV_16s_18979	0.142914796	0.315939944	3.551088331	0.776352262	0.997677863	NA
ASV_16s_18980	0.147147508	0.315939923	3.551074018	0.791318591	NA	NA
ASV_16s_18981	0.147147508	0.315939923	3.551074018	0.791318591	NA	NA
ASV_16s_18982	0.140645792	0.315939955	3.551096341	0.770332316	0.997735065	NA
ASV_16s_18983	0.192481605	0.518729443	3.550917586	1.309398843	0.988186177	NA
ASV_16s_18984	0.168679782	0.315939836	3.551012369	0.851744404	0.996879328	NA

ASV_16s_18985	0.152951809	0.315939897	3.551055744	0.805180653	0.997390723	NA
ASV_16s_18986	0.137223835	0.315939973	3.551108931	0.75901077	NA	NA
ASV_16s_18987	0.137223835	0.315939973	3.551108931	0.75901077	NA	NA
ASV_16s_18988	0.137223835	0.315939973	3.551108931	0.75901077	NA	NA
ASV_16s_18989	0.137223835	0.315939973	3.551108931	0.75901077	NA	NA
ASV_16s_18990	0.137223835	0.315939973	3.551108931	0.75901077	NA	NA
ASV_16s_18991	0.137223835	0.315939973	3.551108931	0.75901077	NA	NA
ASV_16s_18992	0.137223835	0.315939973	3.551108931	0.75901077	NA	NA
ASV_16s_18993	0.137223835	0.315939973	3.551108931	0.75901077	NA	NA
ASV_16s_18994	0.137223835	0.315939973	3.551108931	0.75901077	NA	NA
ASV_16s_18995	0.137223835	0.315939973	3.551108931	0.75901077	NA	NA
ASV_16s_18996	0.137223835	0.315939973	3.551108931	0.75901077	NA	NA
ASV_16s_18997	0.137223835	0.315939973	3.551108931	0.75901077	NA	NA
ASV_16s_18998	0.137223835	0.315939973	3.551108931	0.75901077	NA	NA
ASV_16s_18999	0.137223835	0.315939973	3.551108931	0.75901077	NA	NA
ASV_16s_19000	0.167901871	0.533555489	3.550965032	1.175699461	0.991475691	NA
ASV_16s_19001	0.184765184	0.596079578	3.550698852	0.669445908	NA	NA
ASV_16s_19002	0.13068334	0.687298763	3.551061329	0.653909246	NA	NA
ASV_16s_19003	0.13068334	0.687298763	3.551061329	0.653909246	NA	NA
ASV_16s_19004	0.236088595	0.688002655	3.550835983	0.934096461	NA	NA
ASV_16s_19005	0.211631416	1.078371265	3.550803619	0.678552713	0.998495155	NA
ASV_16s_19006	0.214521359	0.733881142	3.55107666	0.948852137	NA	NA
ASV_16s_19007	0.214521359	0.733881142	3.55107666	0.948852137	NA	NA
ASV_16s_19008	0.214521359	0.733881142	3.55107666	0.948852137	NA	NA
ASV_16s_19009	0.181386966	1.027977455	3.551021603	0.653307638	0.998669343	NA
ASV_16s_19010	0.19631938	0.733721561	3.55112273	0.905153301	0.996216978	NA
ASV_16s_19011	0.181530272	0.73357081	3.551166879	0.869530717	NA	NA
ASV_16s_19012	0.181530272	0.73357081	3.551166879	0.869530717	NA	NA
ASV_16s_19013	0.181530272	0.73357081	3.551166879	0.869530717	NA	NA
ASV_16s_19014	0.181530272	0.73357081	3.551166879	0.869530717	NA	NA
ASV_16s_19015	0.181530272	0.73357081	3.551166879	0.869530717	NA	NA
ASV_16s_19016	0.181530272	0.73357081	3.551166879	0.869530717	NA	NA
ASV_16s_19017	0.181530272	0.73357081	3.551166879	0.869530717	NA	NA
ASV_16s_19018	0.181530272	0.73357081	3.551166879	0.869530717	NA	NA
ASV_16s_19019	0.181530272	0.73357081	3.551166879	0.869530717	NA	NA
ASV_16s_19020	0.181530272	0.73357081	3.551166879	0.869530717	NA	NA
ASV_16s_19021	0.181530272	0.73357081	3.551166879	0.869530717	NA	NA
ASV_16s_19022	0.181530272	0.73357081	3.551166879	0.869530717	NA	NA
ASV_16s_19023	0.181530272	0.73357081	3.551166879	0.869530717	NA	NA
ASV_16s_19024	0.089922049	0.698008904	3.551597864	0.565951627	NA	NA
ASV_16s_19025	0.089922049	0.698008904	3.551597864	0.565951627	NA	NA
ASV_16s_19026	0.089922049	0.698008904	3.551597864	0.565951627	NA	NA
ASV_16s_19027	0.089922049	0.698008904	3.551597864	0.565951627	NA	NA
ASV_16s_19028	0.170580703	0.692696443	3.550986329	0.799444388	0.997449619	NA
ASV_16s_19029	0.171571447	0.69270491	3.550983841	0.80217115	0.997421733	NA
ASV_16s_19030	0.171571447	0.69270491	3.550983841	0.80217115	0.997421733	NA
ASV_16s_19031	0.145924664	0.692449154	3.551059077	0.726495332	0.998123773	NA
ASV_16s_19032	0.195740954	0.692885163	3.550930882	0.863332006	0.996742637	NA
ASV_16s_19033	0.173552934	0.692722734	3.550978948	0.80758843	NA	NA
ASV_16s_19034	0.173552934	0.692722734	3.550978948	0.80758843	NA	NA
ASV_16s_19035	0.173552934	0.692722734	3.550978948	0.80758843	NA	NA
ASV_16s_19036	0.173552934	0.692722734	3.550978948	0.80758843	NA	NA
ASV_16s_19037	0.173552934	0.692722734	3.550978948	0.80758843	NA	NA
ASV_16s_19038	0.173552934	0.692722734	3.550978948	0.80758843	NA	NA
ASV_16s_19039	0.173552934	0.692722734	3.550978948	0.80758843	NA	NA
ASV_16s_19040	0.164783841	0.692644854	3.551001498	0.783219416	0.997611457	NA
ASV_16s_19041	0.158520204	0.692584945	3.551019118	0.764319594	0.997791263	NA
ASV_16s_19042	0.137223835	0.692342502	3.551090918	0.699960716	NA	NA
ASV_16s_19043	0.137223835	0.692342502	3.551090918	0.699960716	NA	NA
ASV_16s_19044	0.137223835	0.692342502	3.551090918	0.699960716	NA	NA
ASV_16s_19045	0.137223835	0.692342502	3.551090918	0.699960716	NA	NA
ASV_16s_19046	0.137223835	0.692342502	3.551090918	0.699960716	NA	NA
ASV_16s_19047	0.137223835	0.692342502	3.551090918	0.699960716	NA	NA
ASV_16s_19048	0.152760271	0.692525499	3.551036608	0.744228601	0.997972347	NA
ASV_16s_19049	0.161638234	0.69261523	3.55101021	0.769477219	0.997743114	NA
ASV_16s_19050	0.2108874	1.387641277	3.550973387	0.499249043	0.999449226	NA
ASV_16s_19051	0.114229404	0.29651153	3.551882954	0.486116838	NA	NA

ASV_16s_19052	0.114229404	0.29651153	3.551882954	0.486116838	NA	NA
ASV_16s_19053	0.114229404	0.29651153	3.551882954	0.486116838	NA	NA
ASV_16s_19054	0.114229404	0.29651153	3.551882954	0.486116838	NA	NA
ASV_16s_19055	0.114229404	0.29651153	3.551882954	0.486116838	NA	NA
ASV_16s_19056	0.114229404	0.29651153	3.551882954	0.486116838	NA	NA
ASV_16s_19057	0.114229404	0.29651153	3.551882954	0.486116838	NA	NA
ASV_16s_19058	0.114229404	0.29651153	3.551882954	0.486116838	NA	NA
ASV_16s_19059	0.114229404	0.29651153	3.551882954	0.486116838	NA	NA
ASV_16s_19060	0.114229404	0.29651153	3.551882954	0.486116838	NA	NA
ASV_16s_19061	0.114229404	0.29651153	3.551882954	0.486116838	NA	NA
ASV_16s_19062	0.114229404	0.29651153	3.551882954	0.486116838	NA	NA
ASV_16s_19063	0.114229404	0.29651153	3.551882954	0.486116838	NA	NA
ASV_16s_19064	0.114229404	0.29651153	3.551882954	0.486116838	NA	NA
ASV_16s_19065	0.114229404	0.29651153	3.551882954	0.486116838	NA	NA
ASV_16s_19066	0.114269333	0.052096444	3.551649117	0.671912869	0.99854236	NA
ASV_16s_19067	0.116559674	-0.018171035	3.551556116	0.709481069	0.998261823	NA
ASV_16s_19068	0.114280741	-0.004662136	3.55158395	0.69848386	0.998347359	NA
ASV_16s_19069	0.116818592	-0.045502445	3.551527109	0.720278916	0.998175025	NA
ASV_16s_19070	0.116818592	-0.045502445	3.551527109	0.720278916	0.998175025	NA
ASV_16s_19071	0.129734917	-0.108329192	3.551354858	0.784146721	0.997602395	NA
ASV_16s_19072	0.116818592	-0.045502445	3.551527109	0.720278916	0.998175025	NA
ASV_16s_19073	0.117077511	-0.072109416	3.551498146	0.728909808	0.998103612	NA
ASV_16s_19074	0.114292149	-0.057127843	3.55153082	0.716293761	0.998207387	NA
ASV_16s_19075	0.139944023	-0.191299128	3.551195496	0.841946873	0.996991934	NA
ASV_16s_19076	0.11733643	-0.097662734	3.551469035	0.735468531	0.998048123	NA
ASV_16s_19077	0.11733643	-0.097662734	3.551469035	0.735468531	0.998048123	NA
ASV_16s_19078	0.114303557	-0.106931642	3.551482918	0.725882331	0.998128869	NA
ASV_16s_19079	0.117595349	-0.122519619	3.551443345	0.739646276	0.998012223	NA
ASV_16s_19080	0.117854268	-0.146846219	3.551420852	0.741505653	0.997996106	NA
ASV_16s_19081	0.118113187	-0.170928806	3.551398672	0.741117797	0.997999475	NA
ASV_16s_19082	0.118113187	-0.170928806	3.551398672	0.741117797	0.997999475	NA
ASV_16s_19083	0.118113187	-0.170928806	3.551398672	0.741117797	0.997999475	NA
ASV_16s_19084	0.145039159	-0.266007633	3.551109582	0.853665288	0.996856933	NA
ASV_16s_19085	0.118372106	-0.194695406	3.551378708	0.73856279	0.998021576	NA
ASV_16s_19086	0.118372106	-0.194695406	3.551378708	0.73856279	0.998021576	NA
ASV_16s_19087	0.114320669	-0.177762695	3.551427075	0.723707472	0.998146874	NA
ASV_16s_19088	0.114326373	-0.200760814	3.551410548	0.718854321	0.998186637	NA
ASV_16s_19089	0.118631024	-0.218127611	3.551361367	0.73390348	0.998061461	NA
ASV_16s_19090	0.114326373	-0.200760814	3.551410548	0.718854321	0.998186637	NA
ASV_16s_19091	0.118889943	-0.241534065	3.55134502	0.727205247	0.99811786	NA
ASV_16s_19092	0.118889943	-0.241534065	3.55134502	0.727205247	0.99811786	NA
ASV_16s_19093	0.116357795	-0.231957523	3.551374388	0.718816835	0.998186942	NA
ASV_16s_19094	0.116628122	-0.300059164	3.551331081	0.68627884	0.998438957	NA
ASV_16s_19095	0.040835637	-0.133035523	3.551853726	0.244741319	0.999949882	NA
ASV_16s_19096	0.040835637	-0.133035523	3.551853726	0.244741319	0.999949882	NA
ASV_16s_19097	0.040835637	-0.133035523	3.551853726	0.244741319	0.999949882	NA
ASV_16s_19098	0.177415004	-0.599136365	3.550895168	0.644025813	0.99872983	NA
ASV_16s_19099	0.169673084	-0.5856578	3.550938711	0.62803811	0.998829649	NA
ASV_16s_19100	0.155844914	-0.559405233	3.551021297	0.604201987	NA	NA
ASV_16s_19101	0.150744813	-0.549037066	3.551054625	0.593053406	0.999029429	NA
ASV_16s_19102	0.148890231	-0.545187268	3.551067179	0.588578547	0.999053194	NA
ASV_16s_19103	0.152079101	-0.551781031	3.551045739	0.595655762	0.999015426	NA
ASV_16s_19104	0.148890231	-0.545187268	3.551067179	0.588578547	0.999053194	NA
ASV_16s_19105	0.148426585	-0.544217505	3.551070356	0.587497618	0.999058876	NA
ASV_16s_19106	0.148426585	-0.544217505	3.551070356	0.587497618	0.999058876	NA
ASV_16s_19107	0.16072051	-0.569020671	3.550991003	0.614612813	NA	NA
ASV_16s_19108	0.151791674	-0.551188225	3.551047653	0.594079428	0.999023924	NA
ASV_16s_19109	0.149878352	-0.547240754	3.55106047	0.58990488	0.999046192	NA
ASV_16s_19110	0.142862838	-0.532347248	3.551109684	0.575276856	NA	NA
ASV_16s_19111	0.142862838	-0.532347248	3.551109684	0.575276856	NA	NA
ASV_16s_19112	0.142862838	-0.532347248	3.551109684	0.575276856	NA	NA
ASV_16s_19113	0.142862838	-0.532347248	3.551109684	0.575276856	NA	NA
ASV_16s_19114	0.142862838	-0.532347248	3.551109684	0.575276856	NA	NA
ASV_16s_19115	0.136016979	-0.515419696	3.55115273	0.554519933	0.999221355	NA
ASV_16s_19116	0.138270354	-0.521060397	3.551138385	0.560983143	0.999191137	NA
ASV_16s_19117	0.121479132	-0.47689001	3.551252946	0.522320956	NA	NA
ASV_16s_19118	0.121479132	-0.47689001	3.551252946	0.522320956	NA	NA

ASV_16s_19119	0.121479132	-0.47689001	3.551252946	0.522320956	NA	NA
ASV_16s_19120	0.121479132	-0.47689001	3.551252946	0.522320956	NA	NA
ASV_16s_19121	0.121479132	-0.47689001	3.551252946	0.522320956	NA	NA
ASV_16s_19122	0.14597069	-0.538980496	3.551087621	0.576468743	0.999115534	NA
ASV_16s_19123	0.117934125	-0.466771242	3.551280123	0.51288986	0.999397902	NA
ASV_16s_19124	0.11717448	-0.464567177	3.55128611	0.510842085	0.999405806	NA
ASV_16s_19125	0.11717448	-0.464567177	3.55128611	0.510842085	0.999405806	NA
ASV_16s_19126	0.114389118	-0.456373242	3.551308576	0.50327079	NA	NA
ASV_16s_19127	0.114389118	-0.456373242	3.551308576	0.50327079	NA	NA
ASV_16s_19128	0.114389118	-0.456373242	3.551308576	0.50327079	NA	NA
ASV_16s_19129	0.114389118	-0.456373242	3.551308576	0.50327079	NA	NA
ASV_16s_19130	0.114389118	-0.456373242	3.551308576	0.50327079	NA	NA
ASV_16s_19131	0.114389118	-0.456373242	3.551308576	0.50327079	NA	NA
ASV_16s_19132	0.174230181	-0.593870733	3.55091335	0.640121325	NA	NA
ASV_16s_19133	0.025332337	-0.130544653	3.551340605	0.080800728	NA	NA
ASV_16s_19134	0.021958723	-0.115515495	3.551408282	0.057569594	NA	NA
ASV_16s_19135	0.050805735	0.221901399	3.551131153	0.220372456	NA	NA
ASV_16s_19137	0.178393971	0.596066244	3.550704174	0.652161695	NA	NA
ASV_16s_19138	0.16072051	0.687591628	3.550967235	0.746896394	NA	NA
ASV_16s_19139	0.147060169	-0.531641731	3.551127369	0.560074301	0.999195433	NA
ASV_16s_19140	0.118171006	-0.057769622	3.551632323	0.641098251	0.998748519	NA
ASV_16s_19141	0.120898739	-0.172520576	3.551454933	0.477065338	0.999526201	NA
ASV_16s_19142	0.155028212	-0.527885504	3.551114063	0.603554996	0.998972088	NA
ASV_16s_19143	0.126408273	0.622180564	3.551878669	0.552896413	0.999228824	NA
ASV_16s_19144	0.147582991	-0.194836668	3.551608547	0.5985768	0.998999546	NA
ASV_16s_19145	0.149936403	-0.521648994	3.551107107	0.609165811	0.998940537	NA
ASV_16s_19146	0.147466908	-0.511185227	3.551136799	0.604078807	0.998969169	NA
ASV_16s_19147	0.151635391	-0.516963997	3.551060281	0.632750305	0.998800797	NA
ASV_16s_19148	0.149475707	-0.447488132	3.551361791	0.483190575	0.999505733	NA
ASV_16s_19149	0.129583184	-0.06931701	3.551224337	0.588400412	0.999054132	NA
ASV_16s_19150	0.147725945	-0.50642532	3.55110081	0.627778862	0.998831223	NA
ASV_16s_19151	0.145653444	-0.486972814	3.551097979	0.629186859	0.998822659	NA
ASV_16s_19152	0.150240126	-0.498687977	3.551051084	0.645646553	0.998719404	NA
ASV_16s_19153	0.143862386	-0.47399323	3.551101847	0.627835514	0.998830879	NA
ASV_16s_19154	0.118746438	-0.642112844	3.551186461	0.384529417	0.999769158	NA
ASV_16s_19155	0.123340941	0.225902199	3.551496753	0.664669883	0.998592715	NA
ASV_16s_19156	0.152319068	-0.489031794	3.550996936	0.667138165	0.998575687	NA
ASV_16s_19157	0.147305373	-0.453003077	3.551016529	0.664231577	0.998595724	NA
ASV_16s_19158	0.146549089	-0.453792336	3.551027076	0.660207758	0.99862315	NA
ASV_16s_19159	0.135107465	0.673105841	3.551578019	0.631990382	0.998805482	NA
ASV_16s_19160	0.159743235	-0.480770064	3.550899847	0.71825531	0.998191506	NA
ASV_16s_19161	0.145836513	-0.739975064	3.550949561	0.437597662	0.99964442	NA
ASV_16s_19162	0.139592113	-0.412933315	3.551074231	0.647343997	0.998708423	NA
ASV_16s_19163	0.130704952	0.670778474	3.551499936	0.597807804	0.999003743	NA
ASV_16s_19164	0.156078622	-0.478832077	3.550946638	0.70787662	0.998274482	NA
ASV_16s_19165	0.123973645	-0.282135144	3.551486839	0.663755224	0.99859899	NA
ASV_16s_19166	0.13332266	0.041307032	3.551074945	0.614898581	0.998907635	NA
ASV_16s_19167	0.123360692	0.33276863	3.551890338	0.277095288	0.999923566	NA
ASV_16s_19168	0.154946823	-0.337093772	3.551265347	0.715651782	0.998212564	NA
ASV_16s_19169	0.14566649	-0.376798428	3.551001423	0.690976223	0.998404115	NA
ASV_16s_19170	0.143206124	-0.367568169	3.551023856	0.682513995	0.998466513	NA
ASV_16s_19171	0.14837487	0.0881197	3.551241215	0.734832954	0.998053547	NA
ASV_16s_19172	0.150556144	-0.371980989	3.550954941	0.714363643	0.998222923	NA
ASV_16s_19173	0.145598083	-0.377622243	3.551068117	0.713903628	0.998226612	NA
ASV_16s_19174	0.142987233	-0.341346398	3.551018818	0.688196503	0.998424795	NA
ASV_16s_19175	0.150908453	-0.350592285	3.550959338	0.719518451	0.99818123	NA
ASV_16s_19176	0.150908453	-0.350592285	3.550959338	0.719518451	0.99818123	NA
ASV_16s_19177	0.158079006	-0.623512865	3.551465438	0.300782924	0.999899076	NA
ASV_16s_19178	0.149331732	-0.285246179	3.550990229	0.719621539	0.998180389	NA
ASV_16s_19179	0.143304206	-0.224626148	3.551016864	0.702225715	0.998318577	NA
ASV_16s_19180	0.135202947	-0.224606529	3.551074406	0.669948491	0.998556134	NA
ASV_16s_19181	0.156736455	-0.162047899	3.551481559	0.556876441	0.999210427	NA
ASV_16s_19182	0.144078574	-0.164211107	3.55112759	0.652855905	0.998672331	NA
ASV_16s_19183	0.241960299	0.255124461	3.550922412	1.085019007	0.993338053	NA
ASV_16s_19184	0.180582737	0.725164893	3.551289139	0.848564904	0.996916168	NA
ASV_16s_19185	0.164539739	-0.506980125	3.551230415	0.393573008	0.999750463	NA
ASV_16s_19186	0.149147762	-0.110998279	3.551023048	0.725468407	0.998132305	NA

ASV_16s_19187	0.151097523	0.265227181	3.551012105	0.712454109	0.998238204	NA
ASV_16s_19188	0.143329532	0.341074173	3.551748615	0.531281896	0.999323687	NA
ASV_16s_19189	0.147202226	-0.006232595	3.551325525	0.776975001	0.997671892	NA
ASV_16s_19190	0.12857857	0.311372438	3.551414928	0.534343881	NA	NA
ASV_16s_19191	0.14232812	0.136655638	3.550610891	0.557207748	NA	NA
ASV_16s_19192	0.101079173	0.693078603	3.551460677	0.321527853	0.999873558	NA
ASV_16s_19193	0.16854633	1.084280831	3.551227862	0.568370286	0.999155641	NA
ASV_16s_19194	0.137035104	0.116991507	3.551005583	0.507669269	0.999417912	NA
ASV_16s_19195	0.172011221	0.545424059	3.55102517	0.79578001	NA	NA
ASV_16s_19196	0.090402966	0.063531015	3.550911252	0.376386695	NA	NA
ASV_16s_19197	0.155891203	1.376181262	3.551269085	0.318480426	0.999877559	NA
ASV_16s_19198	0.147271295	1.344803767	3.551177169	0.309440378	0.999888911	NA
ASV_16s_19199	0.193664923	0.285472689	3.551584184	0.658524575	NA	NA
ASV_16s_19200	0.158996447	1.140533287	3.551212503	0.558238198	NA	NA
ASV_16s_19201	0.126639957	1.060733834	3.55140038	0.483451281	NA	NA
ASV_16s_19202	0.138965587	1.248139385	3.551339134	0.391400292	0.999755046	NA
ASV_16s_19203	0.159937548	1.142506333	3.551207511	0.557939407	0.999205465	NA
ASV_16s_19204	0.130742195	1.07185305	3.551373648	0.493577027	NA	NA
ASV_16s_19205	0.130742195	1.07185305	3.551373648	0.493577027	NA	NA
ASV_16s_19206	0.138538455	1.332591141	3.551184522	0.30290992	0.999896641	NA
ASV_16s_19207	0.153663378	0.322752644	3.551014342	0.721786511	0.998162682	NA
ASV_16s_19208	0.146319554	-0.421139417	3.551340585	0.473308019	0.999538466	NA
ASV_16s_19209	0.186198698	-0.799477515	3.550897991	0.485873727	0.99949658	NA
ASV_16s_19210	0.150059722	0.349744851	3.551806595	0.574176627	NA	NA
ASV_16s_19211	0.150059722	0.349744851	3.551806595	0.574176627	NA	NA
ASV_16s_19212	0.172012408	0.359984219	3.551683706	0.615895139	0.998901846	NA
ASV_16s_19213	0.165972486	0.356954425	3.551717219	0.605609034	NA	NA
ASV_16s_19214	0.16505914	0.342023222	3.551957337	0.430424989	NA	NA
ASV_16s_19215	0.149334201	-0.266578098	3.551264164	0.689261839	0.99841689	NA
ASV_16s_19216	0.217411197	0.379127197	3.551408445	0.708060589	NA	NA
ASV_16s_19217	0.096055849	0.331743518	3.551978889	0.43570955	NA	NA
ASV_16s_19218	0.096055849	0.331743518	3.551978889	0.43570955	NA	NA
ASV_16s_19219	0.096055849	0.331743518	3.551978889	0.43570955	NA	NA
ASV_16s_19220	0.130061763	0.343352089	3.551883203	0.527538692	NA	NA
ASV_16s_19221	0.130061763	0.343352089	3.551883203	0.527538692	NA	NA
ASV_16s_19222	0.129598696	0.094052993	3.551909355	0.513134064	NA	NA
ASV_16s_19223	0.174586301	0.361257427	3.551669834	0.621494564	NA	NA
ASV_16s_19224	0.183637055	0.85496334	3.55147423	0.573129166	0.999132225	NA
ASV_16s_19225	0.154618888	0.351286782	3.551783677	0.584049779	NA	NA
ASV_16s_19226	0.148996906	0.343079309	3.55202624	0.33067795	0.999861002	NA
ASV_16s_19227	0.143647838	-0.560085061	3.551078607	0.469387505	0.999551031	NA
ASV_16s_19228	0.170154841	0.604105617	3.550974593	0.500455789	0.999444811	NA
ASV_16s_19229	0.190435461	-0.758404644	3.550814386	0.541206595	0.999281175	NA
ASV_16s_19230	0.119218202	-0.437829754	3.551377272	0.506466816	0.999422456	NA
ASV_16s_19231	0.125568969	-0.453196136	3.551325265	0.522534964	NA	NA
ASV_16s_19232	0.147086338	-0.731773195	3.55128809	0.303789682	0.999895622	NA
ASV_16s_19233	0.173016832	1.167514498	3.551135053	0.586854813	NA	NA
ASV_16s_19234	0.26415346	1.283986494	3.550731062	0.758975076	NA	NA
ASV_16s_19235	0.127258256	1.101704695	3.551140726	0.4268921	NA	NA
ASV_16s_19236	0.136702017	-0.067989212	3.551107762	0.589757708	0.999046971	NA
ASV_16s_19237	0.210956423	0.397644697	3.551071099	0.627533096	0.998832714	NA
ASV_16s_19238	0.160857749	0.374801087	3.551386494	0.53163878	NA	NA
ASV_16s_19239	0.132491979	0.361859702	3.551550383	0.470379266	NA	NA
ASV_16s_19240	0.132491979	0.361859702	3.551550383	0.470379266	NA	NA
ASV_16s_19241	0.132491979	0.361859702	3.551550383	0.470379266	NA	NA
ASV_16s_19242	0.109370565	0.350692401	3.55165424	0.412366339	NA	NA
ASV_16s_19243	0.109370565	0.350692401	3.55165424	0.412366339	NA	NA
ASV_16s_19244	0.109370565	0.350692401	3.55165424	0.412366339	NA	NA
ASV_16s_19245	0.118335074	0.355332781	3.551612013	0.435624266	NA	NA
ASV_16s_19246	0.118335074	0.355332781	3.551612013	0.435624266	NA	NA
ASV_16s_19247	0.18975108	-0.095808417	3.550306248	0.989293403	0.994999405	NA
ASV_16s_19248	0.066485579	-0.291513353	3.551501333	0.284735979	NA	NA
ASV_16s_19249	0.066485579	-0.291513353	3.551501333	0.284735979	NA	NA
ASV_16s_19250	0.163094781	-0.522118851	3.550653199	0.537720256	NA	NA
ASV_16s_19251	0.163094781	-0.522118851	3.550653199	0.537720256	NA	NA
ASV_16s_19252	0.127296664	-0.509977153	3.55102368	0.390586071	0.999756748	NA
ASV_16s_19253	0.127556688	-0.471935293	3.550999514	0.461205563	NA	NA

ASV_16s_19254	0.127556688	-0.471935293	3.550999514	0.461205563	NA	NA
ASV_16s_19255	0.166387868	1.186462166	3.550939453	0.514355034	NA	NA
ASV_16s_19256	0.145950461	1.14678735	3.551043074	0.470880921	NA	NA
ASV_16s_19257	0.058663524	0.861701656	3.551687838	0.229286251	0.999959873	NA
ASV_16s_19258	0.058663524	0.861701656	3.551687838	0.229286251	0.999959873	NA
ASV_16s_19259	0.058663524	0.861701656	3.551687838	0.229286251	0.999959873	NA
ASV_16s_19260	0.132491979	-0.078246958	3.550926717	0.739876841	NA	NA
ASV_16s_19261	0.132491979	-0.078246958	3.550926717	0.739876841	NA	NA
ASV_16s_19262	0.132491979	-0.078246958	3.550926717	0.739876841	NA	NA
ASV_16s_19263	0.112977418	-0.078009602	3.550997413	0.669089588	NA	NA
ASV_16s_19264	0.112977418	-0.078009602	3.550997413	0.669089588	NA	NA
ASV_16s_19265	0.112977418	-0.078009602	3.550997413	0.669089588	NA	NA
ASV_16s_19266	0.112977418	-0.078009602	3.550997413	0.669089588	NA	NA
ASV_16s_19267	0.112977418	-0.078009602	3.550997413	0.669089588	NA	NA
ASV_16s_19268	0.112977418	-0.078009602	3.550997413	0.669089588	NA	NA
ASV_16s_19269	0.12746859	-0.078192725	3.550942861	0.722400979	NA	NA
ASV_16s_19270	0.12746859	-0.078192725	3.550942861	0.722400979	NA	NA
ASV_16s_19271	0.12746859	-0.078192725	3.550942861	0.722400979	NA	NA
ASV_16s_19272	0.074043068	0.220228361	3.550967046	0.342763057	NA	NA
ASV_16s_19273	0.07863799	0.220211174	3.550944368	0.364593853	0.999806921	NA
ASV_16s_19274	0.07863799	0.220211174	3.550944368	0.364593853	0.999806921	NA
ASV_16s_19275	0.084107781	0.220193158	3.550920575	0.389757611	NA	NA
ASV_16s_19276	0.083939824	0.220193677	3.550921259	0.388999485	NA	NA
ASV_16s_19277	0.083939824	0.220193677	3.550921259	0.388999485	NA	NA
ASV_16s_19278	0.138542553	0.310951588	3.551059691	0.740418184	0.998005543	NA
ASV_16s_19279	0.143095043	0.310950623	3.551044777	0.755415098	NA	NA
ASV_16s_19280	0.08860091	0.595666506	3.550859747	0.355456321	NA	NA
ASV_16s_19281	0.128390095	0.68727077	3.551070361	0.646124783	NA	NA
ASV_16s_19282	0.138318439	0.493898022	3.551857095	0.558361339	NA	NA
ASV_16s_19283	0.115950528	-0.065418804	3.551052338	0.712995075	NA	NA
ASV_16s_19284	0.152615915	1.360913836	3.551304328	0.337582622	0.999850974	NA
ASV_16s_19285	0.197345037	-0.24485609	3.550911284	0.653484058	0.998668176	NA
ASV_16s_19286	0.117437083	-0.067059313	3.551096206	0.745233397	0.997963533	NA
ASV_16s_19287	0.132324319	1.283860829	3.551231484	0.267983033	0.999931768	NA
ASV_16s_19288	0.110996909	1.093281348	3.551599767	0.37663651	0.999784666	NA
ASV_16s_19289	0.118428119	-0.066351615	3.551137711	0.759158805	0.997838757	NA
ASV_16s_19290	0.12153337	-0.066458514	3.551103901	0.771146083	0.997727388	NA
ASV_16s_19291	0.139674499	0.298011157	3.550875899	0.820628366	0.997227691	NA
ASV_16s_19292	0.118526634	0.304599362	3.551975536	0.317022469	0.999879442	NA
ASV_16s_19293	0.129470525	-0.064571994	3.55110781	0.810516664	0.99733514	NA
ASV_16s_19294	0.132491979	0.273913671	3.550710574	0.754046978	0.997885131	NA
ASV_16s_19295	0.128394084	-0.063693933	3.551119725	0.80563926	0.997385976	NA
ASV_16s_19296	0.153277169	0.727881708	3.551572617	0.751306195	0.997909722	NA
ASV_16s_19297	0.210473519	0.29240695	3.551885714	0.45118179	0.99960633	NA
ASV_16s_19298	0.120905711	-0.052630443	3.55126215	0.770358778	0.997734816	NA
ASV_16s_19299	0.225619016	0.18039298	3.550755708	0.940743744	0.995728635	NA
ASV_16s_19300	0.115809118	-0.095099158	3.551545448	0.673628727	0.998530258	NA
ASV_16s_19301	0.130763574	0.706172403	3.551534876	0.715452803	0.998214167	NA
ASV_16s_19302	0.138138389	0.318392742	3.551249993	0.62662171	0.99883823	NA
ASV_16s_19303	0.16466728	-0.344911191	3.551118718	0.608235865	0.998945811	NA
ASV_16s_19304	0.087357422	0.190466469	3.551817997	0.373466961	0.999790686	NA
ASV_16s_19305	0.149193424	0.348834234	3.551626504	0.588839426	0.99905182	NA
ASV_16s_19306	0.211107101	-0.642926421	3.550704294	0.709188681	NA	NA
ASV_16s_19307	0.211107101	-0.642926421	3.550704294	0.709188681	NA	NA
ASV_16s_19308	0.187356778	1.116478983	3.551114259	0.606934487	NA	NA
ASV_16s_19309	0.187356778	1.116478983	3.551114259	0.606934487	NA	NA
ASV_16s_19310	0.203783348	1.049505848	3.551091061	0.620368475	0.998875609	NA
ASV_16s_19311	0.141443232	0.945979112	3.55144117	0.497347007	NA	NA
ASV_16s_19312	0.068678416	0.287949971	3.551855213	0.348236065	0.999834536	NA
ASV_16s_19313	0.068678416	0.287949971	3.551855213	0.348236065	0.999834536	NA
ASV_16s_19314	0.079311469	0.286695709	3.551813188	0.385956201	0.999766275	NA
ASV_16s_19315	0.159531446	0.279927248	3.551626224	0.606242211	NA	NA
ASV_16s_19316	0.22566436	-0.558879707	3.551031266	0.724896287	NA	NA
ASV_16s_19317	0.036574096	-0.095758591	3.551831835	0.21736044	0.999966561	NA
ASV_16s_19318	0.299365983	1.14342615	3.550718726	0.808807858	NA	NA
ASV_16s_19319	0.221669394	0.057072134	3.551029478	0.979940785	0.995145661	NA
ASV_16s_19320	0.17285139	-0.143336633	3.551195061	0.803061377	0.997412585	NA

ASV_16s_19321	0.145945116	0.538223973	3.551129527	0.67672281	NA	NA
ASV_16s_19322	0.166387868	1.186462166	3.550939453	0.514355034	NA	NA
ASV_16s_19323	0.166387868	1.186462166	3.550939453	0.514355034	NA	NA
ASV_16s_19324	0.154282194	1.111810735	3.550991067	0.586410958	0.999064564	NA
ASV_16s_19325	0.09481323	-0.077702104	3.551089126	0.595659572	NA	NA
ASV_16s_19326	0.09481323	-0.077702104	3.551089126	0.595659572	NA	NA
ASV_16s_19327	0.09481323	-0.077702104	3.551089126	0.595659572	NA	NA
ASV_16s_19328	0.09481323	-0.077702104	3.551089126	0.595659572	NA	NA
ASV_16s_19329	0.09481323	-0.077702104	3.551089126	0.595659572	NA	NA
ASV_16s_19330	0.154313372	0.111814469	3.550912081	0.923266627	0.995973239	NA
ASV_16s_19331	0.262374091	-0.029055244	3.550829363	1.165744611	NA	NA
ASV_16s_19332	0.209286178	-0.028819011	3.550907592	1.018037055	NA	NA
ASV_16s_19333	0.209286178	-0.028819011	3.550907592	1.018037055	NA	NA
ASV_16s_19334	0.198571424	0.371274594	3.55061606	1.037345163	0.994203402	NA
ASV_16s_19335	0.172875158	0.408108735	3.550853043	0.902779436	NA	NA
ASV_16s_19336	0.183880647	0.595701431	3.551083253	1.084220285	0.993353185	NA
ASV_16s_19337	0.125092768	-0.028042159	3.551166385	0.772987101	NA	NA
ASV_16s_19338	0.125092768	-0.028042159	3.551166385	0.772987101	NA	NA
ASV_16s_19339	0.125092768	-0.028042159	3.551166385	0.772987101	NA	NA
ASV_16s_19340	0.125092768	-0.028042159	3.551166385	0.772987101	NA	NA
ASV_16s_19341	0.131229268	0.303848153	3.551140851	0.805125073	0.997391298	NA
ASV_16s_19342	0.142934407	-0.073209361	3.550934754	0.79886519	NA	NA
ASV_16s_19343	0.136191086	-0.07313633	3.550955469	0.776606208	0.997675429	NA
ASV_16s_19344	0.104393517	-0.07266837	3.55108884	0.659441259	NA	NA
ASV_16s_19345	0.104393517	-0.07266837	3.55108884	0.659441259	NA	NA
ASV_16s_19346	0.10251774	-0.072631732	3.551099267	0.651865926	NA	NA
ASV_16s_19347	0.10251774	-0.072631732	3.551099267	0.651865926	NA	NA
ASV_16s_19348	0.10251774	-0.072631732	3.551099267	0.651865926	NA	NA
ASV_16s_19349	0.10251774	-0.072631732	3.551099267	0.651865926	NA	NA
ASV_16s_19350	0.10251774	-0.072631732	3.551099267	0.651865926	NA	NA
ASV_16s_19351	0.10251774	-0.072631732	3.551099267	0.651865926	NA	NA
ASV_16s_19352	0.10251774	-0.072631732	3.551099267	0.651865926	NA	NA
ASV_16s_19353	0.10251774	-0.072631732	3.551099267	0.651865926	NA	NA
ASV_16s_19354	0.133697541	0.316323034	3.551005004	0.821537453	0.997217894	NA
ASV_16s_19355	0.146689124	0.370076441	3.551075633	0.844297416	0.996965165	NA
ASV_16s_19356	0.189596752	0.688019992	3.550539383	0.778906629	0.997653306	NA
ASV_16s_19357	0.12719999	-0.073027096	3.550986469	0.745606443	NA	NA
ASV_16s_19358	0.12719999	-0.073027096	3.550986469	0.745606443	NA	NA
ASV_16s_19359	0.12719999	-0.073027096	3.550986469	0.745606443	NA	NA
ASV_16s_19360	0.142696131	0.360258031	3.551303391	0.815762157	NA	NA
ASV_16s_19361	0.142696131	0.360258031	3.551303391	0.815762157	NA	NA
ASV_16s_19362	0.142696131	0.360258031	3.551303391	0.815762157	NA	NA
ASV_16s_19363	0.194346764	0.348555502	3.55137051	0.704554498	0.998300497	NA
ASV_16s_19364	0.170082892	0.691583679	3.551177204	0.967166362	0.995340942	NA
ASV_16s_19365	0.126851931	0.360231367	3.55139094	0.762250173	NA	NA
ASV_16s_19366	0.126851931	0.360231367	3.55139094	0.762250173	NA	NA
ASV_16s_19367	0.126851931	0.360231367	3.55139094	0.762250173	NA	NA
ASV_16s_19368	0.126851931	0.360231367	3.55139094	0.762250173	NA	NA
ASV_16s_19369	0.126851931	0.360231367	3.55139094	0.762250173	NA	NA
ASV_16s_19370	0.126851931	0.360231367	3.55139094	0.762250173	NA	NA
ASV_16s_19371	0.126851931	0.360231367	3.55139094	0.762250173	NA	NA
ASV_16s_19372	0.126851931	0.360231367	3.55139094	0.762250173	NA	NA
ASV_16s_19373	0.126851931	0.360231367	3.55139094	0.762250173	NA	NA
ASV_16s_19374	0.126851931	0.360231367	3.55139094	0.762250173	NA	NA
ASV_16s_19375	0.126851931	0.360231367	3.55139094	0.762250173	NA	NA
ASV_16s_19376	0.111125607	0.46915972	3.551421695	0.927211853	0.995918837	NA
ASV_16s_19377	0.164361359	0.315939869	3.551023428	0.84203925	NA	NA
ASV_16s_19378	0.165292834	0.315939739	3.551021022	0.841940042	0.996992011	NA
ASV_16s_19379	0.16214724	0.315939749	3.551029376	0.832679636	0.997095967	NA
ASV_16s_19380	0.152421411	0.315939918	3.551057351	0.805470969	0.997387719	NA
ASV_16s_19381	0.16564241	0.315939738	3.551020108	0.843323115	0.996976279	NA
ASV_16s_19382	0.148553034	0.315939798	3.551069498	0.793751058	0.997507204	NA
ASV_16s_19383	0.142073456	0.315939967	3.551091313	0.774892879	NA	NA
ASV_16s_19384	0.142073456	0.315939967	3.551091313	0.774892879	NA	NA
ASV_16s_19385	0.142073456	0.315939967	3.551091313	0.774892879	NA	NA
ASV_16s_19386	0.135229544	0.356379821	3.551091774	0.863293298	0.9967431	NA
ASV_16s_19387	0.135229544	0.315940005	3.551116613	0.752211417	0.997901621	NA

ASV_16s_19388	0.156906511	0.315939767	3.551044071	0.816346891	0.997273527	NA
ASV_16s_19389	0.132491979	0.315940021	3.551127456	0.742936875	NA	NA
ASV_16s_19390	0.132491979	0.315940021	3.551127456	0.742936875	NA	NA
ASV_16s_19391	0.132491979	0.315940021	3.551127456	0.742936875	NA	NA
ASV_16s_19392	0.132491979	0.315940021	3.551127456	0.742936875	NA	NA
ASV_16s_19393	0.132491979	0.315940021	3.551127456	0.742936875	NA	NA
ASV_16s_19394	0.132491979	0.315940021	3.551127456	0.742936875	NA	NA
ASV_16s_19395	0.132491979	0.315940021	3.551127456	0.742936875	NA	NA
ASV_16s_19396	0.132491979	0.315940021	3.551127456	0.742936875	NA	NA
ASV_16s_19397	0.132491979	0.315940021	3.551127456	0.742936875	NA	NA
ASV_16s_19398	0.14502159	0.315939953	3.55108123	0.779954781	0.99764318	NA
ASV_16s_19399	0.249435018	0.315939664	3.550875324	1.084851333	NA	NA
ASV_16s_19400	0.126177018	0.68724278	3.551079336	0.638636226	NA	NA
ASV_16s_19401	0.126177018	0.68724278	3.551079336	0.638636226	NA	NA
ASV_16s_19402	0.126177018	0.68724278	3.551079336	0.638636226	NA	NA
ASV_16s_19403	0.189939263	1.031406443	3.55097882	0.670077826	0.99855523	NA
ASV_16s_19404	0.207124071	0.733819083	3.55109446	0.931128494	NA	NA
ASV_16s_19405	0.175138888	1.066352899	3.551231137	0.64589456	0.998717803	NA
ASV_16s_19406	0.236540092	0.734039674	3.551030403	1.006139955	0.994728881	NA
ASV_16s_19407	0.170834021	0.954229801	3.551054504	0.654035522	NA	NA
ASV_16s_19408	0.175270608	0.733498889	3.55118785	0.852707463	NA	NA
ASV_16s_19409	0.175270608	0.733498889	3.55118785	0.852707463	NA	NA
ASV_16s_19410	0.175270608	0.733498889	3.55118785	0.852707463	NA	NA
ASV_16s_19411	0.175270608	0.733498889	3.55118785	0.852707463	NA	NA
ASV_16s_19412	0.175270608	0.733498889	3.55118785	0.852707463	NA	NA
ASV_16s_19413	0.175270608	0.733498889	3.55118785	0.852707463	NA	NA
ASV_16s_19414	0.175270608	0.733498889	3.55118785	0.852707463	NA	NA
ASV_16s_19415	0.175270608	0.733498889	3.55118785	0.852707463	NA	NA
ASV_16s_19416	0.175270608	0.733498889	3.55118785	0.852707463	NA	NA
ASV_16s_19417	0.175270608	0.733498889	3.55118785	0.852707463	NA	NA
ASV_16s_19418	0.175270608	0.733498889	3.55118785	0.852707463	NA	NA
ASV_16s_19419	0.175270608	0.733498889	3.55118785	0.852707463	NA	NA
ASV_16s_19420	0.175270608	0.733498889	3.55118785	0.852707463	NA	NA
ASV_16s_19421	0.086821288	0.694458712	3.55161832	0.552833627	NA	NA
ASV_16s_19422	0.086821288	0.694458712	3.55161832	0.552833627	NA	NA
ASV_16s_19423	0.086821288	0.694458712	3.55161832	0.552833627	NA	NA
ASV_16s_19424	0.086821288	0.694458712	3.55161832	0.552833627	NA	NA
ASV_16s_19425	0.086821288	0.694458712	3.55161832	0.552833627	NA	NA
ASV_16s_19426	0.086821288	0.694458712	3.55161832	0.552833627	NA	NA
ASV_16s_19427	0.086821288	0.694458712	3.55161832	0.552833627	NA	NA
ASV_16s_19428	0.086821288	0.694458712	3.55161832	0.552833627	NA	NA
ASV_16s_19429	0.086821288	0.694458712	3.55161832	0.552833627	NA	NA
ASV_16s_19430	0.086821288	0.694458712	3.55161832	0.552833627	NA	NA
ASV_16s_19431	0.148806033	0.69248239	3.551049393	0.734773888	0.998054051	NA
ASV_16s_19432	0.165256615	0.692649353	3.551000255	0.784472781	0.997599203	NA
ASV_16s_19433	0.165256615	0.692649353	3.551000255	0.784472781	0.997599203	NA
ASV_16s_19434	0.16756835	0.692670214	3.550994119	0.790971754	NA	NA
ASV_16s_19435	0.16756835	0.692670214	3.550994119	0.790971754	NA	NA
ASV_16s_19436	0.16756835	0.692670214	3.550994119	0.790971754	NA	NA
ASV_16s_19437	0.16756835	0.692670214	3.550994119	0.790971754	NA	NA
ASV_16s_19438	0.16756835	0.692670214	3.550994119	0.790971754	NA	NA
ASV_16s_19439	0.16756835	0.692670214	3.550994119	0.790971754	NA	NA
ASV_16s_19440	0.16756835	0.692670214	3.550994119	0.790971754	NA	NA
ASV_16s_19441	0.16756835	0.692670214	3.550994119	0.790971754	NA	NA
ASV_16s_19442	0.159156357	0.692591365	3.551017316	0.764245948	0.997791946	NA
ASV_16s_19443	0.181103035	0.692781755	3.550961323	0.825030361	0.99718004	NA
ASV_16s_19444	0.148777437	0.692482052	3.551049492	0.733609207	0.998063962	NA
ASV_16s_19445	0.154204003	0.692540953	3.551032152	0.74772846	0.997941537	NA
ASV_16s_19446	0.132491979	0.692277849	3.551110033	0.684398758	NA	NA
ASV_16s_19447	0.132491979	0.692277849	3.551110033	0.684398758	NA	NA
ASV_16s_19448	0.132491979	0.692277849	3.551110033	0.684398758	NA	NA
ASV_16s_19449	0.132491979	0.692277849	3.551110033	0.684398758	NA	NA
ASV_16s_19450	0.132491979	0.692277849	3.551110033	0.684398758	NA	NA
ASV_16s_19451	0.132491979	0.692277849	3.551110033	0.684398758	NA	NA
ASV_16s_19452	0.132491979	0.692277849	3.551110033	0.684398758	NA	NA
ASV_16s_19453	0.132491979	0.692277849	3.551110033	0.684398758	NA	NA
ASV_16s_19454	0.132491979	0.692277849	3.551110033	0.684398758	NA	NA

ASV_16s_19455	0.132491979	0.692277849	3.551110033	0.684398758	NA	NA
ASV_16s_19456	0.132491979	0.692277849	3.551110033	0.684398758	NA	NA
ASV_16s_19457	0.150147277	0.692497239	3.551045021	0.735261303	0.998049893	NA
ASV_16s_19458	0.154561102	0.692544658	3.551031062	0.748010782	0.997939038	NA
ASV_16s_19459	0.163388751	0.69263192	3.551005383	0.773394389	0.997706087	NA
ASV_16s_19460	0.16578434	0.692654019	3.550998882	0.780212695	0.997640684	NA
ASV_16s_19461	0.19463772	0.692877994	3.550933048	0.859511574	NA	NA
ASV_16s_19462	0.19463772	0.692877994	3.550933048	0.859511574	NA	NA
ASV_16s_19463	0.194285522	0.692875658	3.550933734	0.858645444	NA	NA
ASV_16s_19464	0.110149783	0.29683243	3.551896778	0.475120617	NA	NA
ASV_16s_19465	0.110149783	0.29683243	3.551896778	0.475120617	NA	NA
ASV_16s_19466	0.110149783	0.29683243	3.551896778	0.475120617	NA	NA
ASV_16s_19467	0.110149783	0.29683243	3.551896778	0.475120617	NA	NA
ASV_16s_19468	0.110149783	0.29683243	3.551896778	0.475120617	NA	NA
ASV_16s_19469	0.110149783	0.29683243	3.551896778	0.475120617	NA	NA
ASV_16s_19470	0.110149783	0.29683243	3.551896778	0.475120617	NA	NA
ASV_16s_19471	0.110149783	0.29683243	3.551896778	0.475120617	NA	NA
ASV_16s_19472	0.110149783	0.29683243	3.551896778	0.475120617	NA	NA
ASV_16s_19473	0.110149783	0.29683243	3.551896778	0.475120617	NA	NA
ASV_16s_19474	0.110149783	0.29683243	3.551896778	0.475120617	NA	NA
ASV_16s_19475	0.110149783	0.29683243	3.551896778	0.475120617	NA	NA
ASV_16s_19476	0.110149783	0.29683243	3.551896778	0.475120617	NA	NA
ASV_16s_19477	0.110149783	0.29683243	3.551896778	0.475120617	NA	NA
ASV_16s_19478	0.110149783	0.29683243	3.551896778	0.475120617	NA	NA
ASV_16s_19479	0.110149783	0.29683243	3.551896778	0.475120617	NA	NA
ASV_16s_19480	0.110149783	0.29683243	3.551896778	0.475120617	NA	NA
ASV_16s_19481	0.110149783	0.29683243	3.551896778	0.475120617	NA	NA
ASV_16s_19482	0.110149783	0.29683243	3.551896778	0.475120617	NA	NA
ASV_16s_19483	0.110184007	0.080587774	3.551704145	0.636281806	0.998778864	NA
ASV_16s_19484	0.110184007	0.080587774	3.551704145	0.636281806	0.998778864	NA
ASV_16s_19485	0.111703296	0.069794043	3.55168305	0.644035299	0.998729769	NA
ASV_16s_19486	0.121003642	-0.017064735	3.551529931	0.708583746	0.99826891	NA
ASV_16s_19487	0.111720408	-0.01627452	3.551587227	0.683937422	0.998456133	NA
ASV_16s_19488	0.112738971	-0.048510971	3.551548469	0.697535049	0.998354605	NA
ASV_16s_19489	0.110212527	-0.060432868	3.551552881	0.692975419	0.99838913	NA
ASV_16s_19490	0.11299789	-0.075248611	3.551522333	0.705441567	0.998293576	NA
ASV_16s_19491	0.11299789	-0.075248611	3.551522333	0.705441567	0.998293576	NA
ASV_16s_19492	0.112238245	-0.071268466	3.551530477	0.702009718	0.998320247	NA
ASV_16s_19493	0.110212527	-0.060432868	3.551552881	0.692975419	0.99838913	NA
ASV_16s_19494	0.110218231	-0.085826175	3.551530404	0.697879187	0.998351979	NA
ASV_16s_19495	0.113256809	-0.101512217	3.551495228	0.710992905	0.998249839	NA
ASV_16s_19496	0.110218231	-0.085826175	3.551530404	0.697879187	0.998351979	NA
ASV_16s_19497	0.113256809	-0.101512217	3.551495228	0.710992905	0.998249839	NA
ASV_16s_19498	0.12670349	-0.183189703	3.551310963	0.771490457	0.997724133	NA
ASV_16s_19499	0.113515727	-0.12707247	3.551470951	0.714079775	0.9982252	NA
ASV_16s_19500	0.113774646	-0.152013422	3.551449409	0.714962541	0.998218111	NA
ASV_16s_19501	0.113774646	-0.152013422	3.551449409	0.714962541	0.998218111	NA
ASV_16s_19502	0.127327086	-0.206951976	3.551288923	0.771530455	0.997723755	NA
ASV_16s_19503	0.113774646	-0.152013422	3.551449409	0.714962541	0.998218111	NA
ASV_16s_19504	0.113774646	-0.152013422	3.551449409	0.714962541	0.998218111	NA
ASV_16s_19505	0.110235343	-0.159306511	3.551468534	0.699178446	0.998342041	NA
ASV_16s_19506	0.114033565	-0.176396438	3.551430106	0.713600061	0.998229044	NA
ASV_16s_19507	0.114292484	-0.200509631	3.551411173	0.709942418	0.998258172	NA
ASV_16s_19508	0.114292484	-0.200509631	3.551411173	0.709942418	0.998258172	NA
ASV_16s_19509	0.127338494	-0.249488276	3.5512641	0.759527979	0.997835383	NA
ASV_16s_19510	0.110246752	-0.206211183	3.551437604	0.689172237	0.998417556	NA
ASV_16s_19511	0.110246752	-0.206211183	3.551437604	0.689172237	0.998417556	NA
ASV_16s_19512	0.110246752	-0.206211183	3.551437604	0.689172237	0.998417556	NA
ASV_16s_19513	0.112024959	-0.236810069	3.551403489	0.686962663	0.998433916	NA
ASV_16s_19514	0.112784603	-0.239976831	3.55139563	0.689437551	0.998415584	NA
ASV_16s_19515	0.123077954	-0.32229651	3.551271848	0.697170669	0.998357382	NA
ASV_16s_19516	0.110269568	-0.298694619	3.551378859	0.64525436	0.998721932	NA
ASV_16s_19517	0.039377221	-0.123372733	3.551862372	0.237974818	0.999954447	NA
ASV_16s_19518	0.039377221	-0.123372733	3.551862372	0.237974818	0.999954447	NA
ASV_16s_19519	0.039377221	-0.123372733	3.551862372	0.237974818	0.999954447	NA
ASV_16s_19520	0.180266248	-0.201593085	3.550253901	0.630102093	0.99881707	NA
ASV_16s_19521	0.150279024	-0.548014005	3.551057952	0.591548602	NA	NA

ASV_16s_19522	0.150279024	-0.548014005	3.551057952	0.591548602	NA	NA
ASV_16s_19523	0.154305642	-0.32786229	3.550869061	0.375625916	NA	NA
ASV_16s_19524	0.14877479	-0.544881739	3.551068179	0.586400886	0.999064616	NA
ASV_16s_19525	0.14239705	-0.531164107	3.551112792	0.57282168	0.999133751	NA
ASV_16s_19526	0.141469759	-0.528908552	3.551118465	0.570841028	0.999143538	NA
ASV_16s_19527	0.154980492	-0.557606468	3.55102703	0.600108005	NA	NA
ASV_16s_19528	0.147327204	-0.541840134	3.551078171	0.582751557	0.999083548	NA
ASV_16s_19529	0.146051656	-0.539138507	3.551087098	0.580058195	0.999097354	NA
ASV_16s_19530	0.145413882	-0.537779483	3.551091606	0.578724063	0.99910414	NA
ASV_16s_19531	0.142862786	-0.532275797	3.551109892	0.573462756	0.999130568	NA
ASV_16s_19532	0.137760594	-0.519748864	3.55114174	0.563145492	NA	NA
ASV_16s_19533	0.137760594	-0.519748864	3.55114174	0.563145492	NA	NA
ASV_16s_19534	0.152014668	-0.551587999	3.551046362	0.594679122	NA	NA
ASV_16s_19535	0.117140591	-0.464405307	3.551286551	0.510663006	NA	NA
ASV_16s_19536	0.117140591	-0.464405307	3.551286551	0.510663006	NA	NA
ASV_16s_19537	0.117140591	-0.464405307	3.551286551	0.510663006	NA	NA
ASV_16s_19538	0.117140591	-0.464405307	3.551286551	0.510663006	NA	NA
ASV_16s_19539	0.117140591	-0.464405307	3.551286551	0.510663006	NA	NA
ASV_16s_19540	0.114355229	-0.456206858	3.551309036	0.503088442	0.999435094	NA
ASV_16s_19541	0.114102014	-0.45545255	3.551311122	0.502395953	0.999437661	NA
ASV_16s_19542	0.114102014	-0.45545255	3.551311122	0.502395953	0.999437661	NA
ASV_16s_19543	0.14163215	-0.52924499	3.551117617	0.565247348	0.999170773	NA
ASV_16s_19544	0.11283594	-0.45165814	3.551321657	0.498917162	0.999450437	NA
ASV_16s_19545	0.11232951	-0.450129583	3.551325921	0.497518042	0.999455519	NA
ASV_16s_19546	0.110303792	-0.443952393	3.551343276	0.491877187	NA	NA
ASV_16s_19547	0.110303792	-0.443952393	3.551343276	0.491877187	NA	NA
ASV_16s_19548	0.110303792	-0.443952393	3.551343276	0.491877187	NA	NA
ASV_16s_19549	0.110303792	-0.443952393	3.551343276	0.491877187	NA	NA
ASV_16s_19550	0.110303792	-0.443952393	3.551343276	0.491877187	NA	NA
ASV_16s_19551	0.110303792	-0.443952393	3.551343276	0.491877187	NA	NA
ASV_16s_19552	0.110303792	-0.443952393	3.551343276	0.491877187	NA	NA
ASV_16s_19553	0.110303792	-0.443952393	3.551343276	0.491877187	NA	NA
ASV_16s_19554	0.110303792	-0.443952393	3.551343276	0.491877187	NA	NA
ASV_16s_19555	0.110303792	-0.443952393	3.551343276	0.491877187	NA	NA
ASV_16s_19556	0.110303792	-0.443952393	3.551343276	0.491877187	NA	NA
ASV_16s_19557	0.110303792	-0.443952393	3.551343276	0.491877187	NA	NA
ASV_16s_19558	0.168007674	-0.582632909	3.550948162	0.625003528	NA	NA
ASV_16s_19559	0.035143493	-0.148695474	3.551167301	0.146033668	NA	NA
ASV_16s_19560	0.032790334	-0.145420021	3.551201011	0.130685791	0.999994172	NA
ASV_16s_19561	0.036772479	0.164102047	3.55121406	0.183517191	0.999981264	NA
ASV_16s_19562	0.031172537	-0.143023904	3.551226954	0.120024135	NA	NA
ASV_16s_19563	0.071398673	0.220239283	3.550981456	0.329784181	NA	NA
ASV_16s_19568	0.154980492	0.687544216	3.550982468	0.73000359	NA	NA
ASV_16s_19569	0.116474886	-0.124665311	3.551671358	0.596668406	0.99900994	NA
ASV_16s_19570	0.116857874	0.04855047	3.551844439	0.513913405	0.999393924	NA
ASV_16s_19571	0.145511996	-0.494215243	3.551245901	0.559377002	0.999198719	NA
ASV_16s_19572	0.119096918	0.411449836	3.551815262	0.589352252	0.999049114	NA
ASV_16s_19573	0.124199608	-0.092206076	3.551247556	0.524223905	0.99935286	NA
ASV_16s_19574	0.118289413	0.274808027	3.551713304	0.550095313	0.999241595	NA
ASV_16s_19575	0.149029693	-0.509281624	3.551086759	0.621314853	0.998870004	NA
ASV_16s_19576	0.140703746	-0.04054407	3.551553228	0.729890912	0.998095379	NA
ASV_16s_19577	0.145634566	-0.483278975	3.551091888	0.62732306	0.998833987	NA
ASV_16s_19578	0.132073151	-0.445826439	3.551233997	0.578716781	0.999104177	NA
ASV_16s_19579	0.121486618	0.311241969	3.55196193	0.380802134	0.999776574	NA
ASV_16s_19580	0.091374725	0.236490076	3.551678409	0.431524678	0.999660601	NA
ASV_16s_19581	0.151009066	-0.487286302	3.55101389	0.658651241	0.998633662	NA
ASV_16s_19582	0.142516843	-0.456172881	3.551088775	0.630536809	0.998814409	NA
ASV_16s_19583	0.137843524	-0.433148973	3.551119351	0.618123305	0.998888829	NA
ASV_16s_19584	0.115744271	-0.095092589	3.551453626	0.578208897	0.999106752	NA
ASV_16s_19585	0.132344781	-0.362974789	3.551586488	0.37470795	0.999788343	NA
ASV_16s_19586	0.147621449	-0.434082751	3.550998189	0.673145868	0.99853367	NA
ASV_16s_19587	0.177378382	0.075263423	3.551019878	0.860276123	0.996779057	NA
ASV_16s_19588	0.117844855	0.247662856	3.551363332	0.390500348	0.999756927	NA
ASV_16s_19589	0.136726725	0.330122623	3.551225239	0.694246674	0.998379553	NA
ASV_16s_19590	0.129545398	-0.363814886	3.551161392	0.614141699	0.998912018	NA
ASV_16s_19591	0.11992201	0.258219978	3.551635293	0.451961132	0.999604064	NA
ASV_16s_19592	0.124150791	0.942299031	3.551607477	0.397307236	0.999742449	NA

ASV_16s_19593	0.13450752	0.67700874	3.551462916	0.636086308	0.998780085	NA
ASV_16s_19594	0.151860807	-0.386074673	3.550987088	0.706850782	0.998282543	NA
ASV_16s_19595	0.139972141	-0.371546029	3.551058248	0.660770334	0.998619337	NA
ASV_16s_19596	0.12891346	-0.339229368	3.551156506	0.619685624	0.998879641	NA
ASV_16s_19597	0.138536582	-0.34132111	3.551062163	0.66187741	0.998611814	NA
ASV_16s_19598	0.117304281	0.096771414	3.551222764	0.55986811	0.999196406	NA
ASV_16s_19599	0.176429532	0.984878454	3.551524455	0.545254667	0.999263328	NA
ASV_16s_19600	0.142206743	-0.343414799	3.55102756	0.677397285	0.998503442	NA
ASV_16s_19601	0.143692147	-0.343423737	3.551013835	0.683920664	0.998456255	NA
ASV_16s_19602	0.147123424	-0.142678863	3.551208385	0.600227799	0.998990495	NA
ASV_16s_19603	0.145085174	-0.346607678	3.551009256	0.688809482	0.99842025	NA
ASV_16s_19604	0.145781688	-0.350576709	3.551003044	0.691172799	0.998402645	NA
ASV_16s_19605	0.146910965	-0.288661512	3.55170949	0.53441284	0.999310465	NA
ASV_16s_19606	0.13276314	-0.314791336	3.551099401	0.642675924	0.99873847	NA
ASV_16s_19607	0.150351216	-0.285230559	3.551032455	0.713426699	0.998230432	NA
ASV_16s_19608	0.148721525	-0.285229565	3.551036058	0.707383864	0.998278357	NA
ASV_16s_19609	0.139599554	-0.219790159	3.551114275	0.703027257	0.998312369	NA
ASV_16s_19610	0.226315844	-0.039123452	3.550912173	1.170564594	0.991588924	NA
ASV_16s_19611	0.143649834	-0.165679998	3.551068558	0.696894343	0.998359486	NA
ASV_16s_19612	0.136396078	-0.08667846	3.551153719	0.674065259	0.998527168	NA
ASV_16s_19613	0.140874978	0.182895483	3.550118353	0.614039745	0.998912607	NA
ASV_16s_19614	0.135054342	-0.056019066	3.551103211	0.670746686	0.998550548	NA
ASV_16s_19615	0.122867642	-0.291227536	3.551055246	0.646563162	0.998713482	NA
ASV_16s_19616	0.120978305	-0.006246537	3.551466537	0.685612185	0.99844386	NA
ASV_16s_19617	0.141949835	-0.00241075	3.551059652	0.699110014	0.998342566	NA
ASV_16s_19618	0.160978062	-0.463922857	3.551390382	0.396436263	0.999744334	NA
ASV_16s_19619	0.130215168	-0.057777507	3.551419796	0.583875987	0.999077743	NA
ASV_16s_19620	0.120914636	0.108975276	3.551753657	0.428533091	0.999668383	NA
ASV_16s_19621	0.18971174	0.729421155	3.551229438	0.858059037	0.996805313	NA
ASV_16s_19622	0.095953475	1.144997201	3.551652194	0.211878308	0.999969356	NA
ASV_16s_19623	0.162696493	0.221473219	3.551721621	0.380117353	0.999777918	NA
ASV_16s_19624	0.153457522	-0.272329173	3.551159555	0.541703565	NA	NA
ASV_16s_19625	0.206652667	0.332940819	3.551907614	0.488698141	0.999486821	NA
ASV_16s_19626	0.153318003	1.128103485	3.551243525	0.545901194	NA	NA
ASV_16s_19627	0.157417925	1.364580901	3.551196243	0.351403247	0.999829418	NA
ASV_16s_19628	0.125047271	1.056293139	3.551409746	0.479393768	0.99951849	NA
ASV_16s_19629	0.133523448	1.079183119	3.551355901	0.50024112	0.999445598	NA
ASV_16s_19630	0.137409271	1.089287466	3.551331834	0.5095627	NA	NA
ASV_16s_19631	0.155938256	1.133915082	3.551229397	0.548053074	0.999250816	NA
ASV_16s_19632	0.126072831	1.059138757	3.551403896	0.481953204	NA	NA
ASV_16s_19633	0.126072831	1.059138757	3.551403896	0.481953204	NA	NA
ASV_16s_19634	0.126072831	1.059138757	3.551403896	0.481953204	NA	NA
ASV_16s_19635	0.170199745	0.653080667	3.551251657	0.849607524	0.996904119	NA
ASV_16s_19636	0.119525707	0.339838694	3.551917201	0.500789727	NA	NA
ASV_16s_19637	0.160135506	-0.456276677	3.551407851	0.51394572	0.999393799	NA
ASV_16s_19638	0.13761346	0.348676266	3.55178417	0.331721486	0.999859517	NA
ASV_16s_19639	0.144700446	0.348296107	3.551832038	0.561798801	NA	NA
ASV_16s_19640	0.187627526	0.099344564	3.551739154	0.530870475	0.999325412	NA
ASV_16s_19641	0.15302476	0.151503634	3.551403284	0.755375098	0.997873147	NA
ASV_16s_19642	0.163943725	-0.435195271	3.551289712	0.497852438	0.999454307	NA
ASV_16s_19643	0.092625283	0.330460752	3.551988215	0.425375893	NA	NA
ASV_16s_19644	0.1254167	0.341832014	3.551897719	0.515879474	NA	NA
ASV_16s_19645	0.1254167	0.341832014	3.551897719	0.515879474	NA	NA
ASV_16s_19646	0.105395088	-0.092208985	3.551842032	0.39580233	0.999745699	NA
ASV_16s_19647	0.111376244	0.347644906	3.551893959	0.269614854	0.999930346	NA
ASV_16s_19648	0.149096785	0.349424377	3.551811582	0.569993081	NA	NA
ASV_16s_19649	0.176791888	0.36230589	3.551658514	0.62566698	NA	NA
ASV_16s_19650	0.092505909	-0.36229232	3.551610967	0.432399573	NA	NA
ASV_16s_19651	0.13608473	-0.475623239	3.551241532	0.547707888	NA	NA
ASV_16s_19652	0.13608473	-0.475623239	3.551241532	0.547707888	NA	NA
ASV_16s_19653	0.123203736	-0.368893646	3.551591783	0.417812406	0.999695266	NA
ASV_16s_19654	0.121084363	-0.442373077	3.551361725	0.511177836	NA	NA
ASV_16s_19655	0.122713318	1.089634468	3.55116779	0.415557977	NA	NA
ASV_16s_19656	0.122713318	1.089634468	3.55116779	0.415557977	NA	NA
ASV_16s_19657	0.122713318	1.089634468	3.55116779	0.415557977	NA	NA
ASV_16s_19658	0.143944174	0.366764134	3.551486789	0.496375309	NA	NA
ASV_16s_19659	0.190791026	0.388794405	3.551202617	0.590116859	NA	NA

ASV_16s_19660	0.154101733	0.036434869	3.550532942	0.875043688	0.996600572	NA
ASV_16s_19661	0.140711332	0.365534142	3.551505318	0.489187177	NA	NA
ASV_16s_19662	0.127760123	0.359952885	3.551571868	0.458996531	NA	NA
ASV_16s_19663	0.131124743	0.62063144	3.551232745	0.630703647	0.998813386	NA
ASV_16s_19664	0.105464473	0.348565185	3.551674311	0.401834247	NA	NA
ASV_16s_19665	0.105464473	0.348565185	3.551674311	0.401834247	NA	NA
ASV_16s_19666	0.114108821	0.353175552	3.551631401	0.424692493	NA	NA
ASV_16s_19667	0.141630513	0.36584406	3.551500554	0.483005862	0.999506358	NA
ASV_16s_19668	0.039731719	0.339500606	3.5519144	0.180927101	0.999982155	NA
ASV_16s_19669	0.064111094	-0.281084979	3.55151691	0.276214575	NA	NA
ASV_16s_19670	0.153596184	-0.125746042	3.550542762	0.685173743	0.998447079	NA
ASV_16s_19671	0.127055081	-0.285960946	3.550988036	0.533687796	NA	NA
ASV_16s_19672	0.193705485	-0.538453253	3.550382935	0.591613567	NA	NA
ASV_16s_19673	0.077861454	-0.337606912	3.55141891	0.323363608	NA	NA
ASV_16s_19674	0.071358347	-0.312217968	3.55146634	0.301620197	NA	NA
ASV_16s_19675	0.071358347	-0.312217968	3.55146634	0.301620197	NA	NA
ASV_16s_19676	0.127760123	-0.472262126	3.550997579	0.461599393	NA	NA
ASV_16s_19677	0.139960396	1.133055151	3.551072835	0.457148208	NA	NA
ASV_16s_19678	0.127760123	-0.078196074	3.550941934	0.723315523	NA	NA
ASV_16s_19679	0.127760123	-0.078196074	3.550941934	0.723315523	NA	NA
ASV_16s_19680	0.127760123	-0.078196074	3.550941934	0.723315523	NA	NA
ASV_16s_19681	0.127760123	-0.078196074	3.550941934	0.723315523	NA	NA
ASV_16s_19682	0.127760123	-0.078196074	3.550941934	0.723315523	NA	NA
ASV_16s_19683	0.10894251	-0.077950154	3.551015217	0.653301453	NA	NA
ASV_16s_19684	0.10894251	-0.077950154	3.551015217	0.653301453	NA	NA
ASV_16s_19685	0.10894251	-0.077950154	3.551015217	0.653301453	NA	NA
ASV_16s_19686	0.10894251	-0.077950154	3.551015217	0.653301453	NA	NA
ASV_16s_19687	0.10894251	-0.077950154	3.551015217	0.653301453	NA	NA
ASV_16s_19688	0.10894251	-0.077950154	3.551015217	0.653301453	NA	NA
ASV_16s_19689	0.10894251	-0.077950154	3.551015217	0.653301453	NA	NA
ASV_16s_19690	0.10894251	-0.077950154	3.551015217	0.653301453	NA	NA
ASV_16s_19691	0.10894251	-0.077950154	3.551015217	0.653301453	NA	NA
ASV_16s_19692	0.10894251	-0.077950154	3.551015217	0.653301453	NA	NA
ASV_16s_19693	0.10894251	-0.077950154	3.551015217	0.653301453	NA	NA
ASV_16s_19694	0.076017589	0.220220631	3.550957007	0.352151104	0.999828193	NA
ASV_16s_19695	0.075993595	0.220220743	3.550957127	0.352036925	0.99982838	NA
ASV_16s_19696	0.077407417	0.220215513	3.550950217	0.35873828	0.999817145	NA
ASV_16s_19697	0.078162316	0.220212772	3.550946629	0.362288971	0.999810991	NA
ASV_16s_19698	0.081103932	0.220202796	3.550933281	0.37598158	NA	NA
ASV_16s_19699	0.080941973	0.220203334	3.550933991	0.375235035	NA	NA
ASV_16s_19700	0.130401463	0.310953498	3.551088968	0.712882321	NA	NA
ASV_16s_19701	0.13538854	0.310952313	3.551070647	0.729156985	0.99810154	NA
ASV_16s_19702	0.138422645	0.310951633	3.551060139	0.739979969	NA	NA
ASV_16s_19703	0.138422645	0.310951633	3.551060139	0.739979969	NA	NA
ASV_16s_19704	0.138422645	0.310951633	3.551060139	0.739979969	NA	NA
ASV_16s_19705	0.138422645	0.310951633	3.551060139	0.739979969	NA	NA
ASV_16s_19706	0.138179234	0.310951686	3.551060965	0.739173351	0.998016309	NA
ASV_16s_19707	0.140759238	0.310951129	3.551052354	0.747688357	NA	NA
ASV_16s_19708	0.120698265	0.31095608	3.551128921	0.678529212	NA	NA
ASV_16s_19709	0.120698265	0.31095608	3.551128921	0.678529212	NA	NA
ASV_16s_19710	0.120698265	0.31095608	3.551128921	0.678529212	NA	NA
ASV_16s_19711	0.120698265	0.31095608	3.551128921	0.678529212	NA	NA
ASV_16s_19712	0.137984506	0.310951729	3.551061628	0.738527276	NA	NA
ASV_16s_19713	0.137984506	0.310951729	3.551061628	0.738527276	NA	NA
ASV_16s_19714	0.085436592	0.595637582	3.550871195	0.342317284	NA	NA
ASV_16s_19715	0.085436592	0.595637582	3.550871195	0.342317284	NA	NA
ASV_16s_19716	0.123804735	0.68721056	3.551089365	0.630441493	NA	NA
ASV_16s_19717	0.144368227	0.687446624	3.55101371	0.69779227	NA	NA
ASV_16s_19718	0.099131597	-0.296144865	3.551705905	0.49789519	0.999454152	NA
ASV_16s_19719	0.133937045	1.130978534	3.551566157	0.270722555	0.99992937	NA
ASV_16s_19720	0.113402175	-0.066933902	3.551135237	0.7208904	0.998170025	NA
ASV_16s_19721	0.122717928	-0.06720249	3.551051217	0.756517176	0.997862805	NA
ASV_16s_19722	0.195323675	1.482486736	3.551207573	0.283233616	0.99991767	NA
ASV_16s_19723	0.113897693	-0.06656387	3.551156854	0.727964781	0.99811152	NA
ASV_16s_19724	0.175685334	1.186292629	3.551291704	0.518405471	0.999376258	NA
ASV_16s_19725	0.181438523	-0.473018432	3.551186977	0.598482823	0.99900006	NA
ASV_16s_19726	0.182574041	-0.424497901	3.551229423	0.608345373	0.998945191	NA

ASV_16s_19727	0.137686532	1.311495445	3.551414426	0.290981955	0.999909784	NA
ASV_16s_19728	0.138971436	0.33993369	3.551801435	0.408733874	0.999716829	NA
ASV_16s_19729	0.160897768	0.729975162	3.551565271	0.765757527	0.997777908	NA
ASV_16s_19730	0.124335808	-0.052062464	3.55125041	0.769895047	0.997739184	NA
ASV_16s_19731	0.210145715	-0.165524133	3.55029635	0.837761782	0.997039212	NA
ASV_16s_19732	0.200488418	0.287561954	3.551771909	0.459879301	0.999580531	NA
ASV_16s_19733	0.12220902	0.655215181	3.551858905	0.602186489	0.998979686	NA
ASV_16s_19734	0.122497693	-0.027726873	3.551372936	0.747553847	0.997943081	NA
ASV_16s_19735	0.183080283	-0.690623768	3.551203303	0.416073837	0.999699479	NA
ASV_16s_19736	0.149490944	0.502086266	3.551160905	0.722429121	0.998157404	NA
ASV_16s_19737	0.141576868	1.028886255	3.551414204	0.514920454	NA	NA
ASV_16s_19738	0.180665465	1.105666361	3.55115411	0.592354295	NA	NA
ASV_16s_19739	0.054540577	0.292465304	3.551896381	0.292339115	0.999908351	NA
ASV_16s_19740	0.194959672	0.27946484	3.551555544	0.682190788	NA	NA
ASV_16s_19741	0.153833894	0.280294947	3.55163485	0.590192589	NA	NA
ASV_16s_19742	0.180549813	-0.304318397	3.551067119	0.85763612	0.996810306	NA
ASV_16s_19743	0.204512919	-0.128667738	3.551272995	0.932670388	0.995842787	NA
ASV_16s_19744	0.248838355	-0.562848745	3.550308968	0.782486611	NA	NA
ASV_16s_19745	0.158368105	0.450512472	3.551091882	0.664702441	0.998592491	NA
ASV_16s_19746	0.160445445	1.175824679	3.550969515	0.502086485	NA	NA
ASV_16s_19747	0.146061176	0.8936567	3.551360018	0.688739224	0.998420771	NA
ASV_16s_19748	0.091427043	-0.077631619	3.551110276	0.580938551	NA	NA
ASV_16s_19749	0.091427043	-0.077631619	3.551110276	0.580938551	NA	NA
ASV_16s_19750	0.091427043	-0.077631619	3.551110276	0.580938551	NA	NA
ASV_16s_19751	0.1032421	-0.094634497	3.551208823	0.631639559	0.99880764	NA
ASV_16s_19752	0.127804603	0.295525206	3.550853411	0.72264195	0.998155653	NA
ASV_16s_19753	0.197008491	0.359433606	3.550969498	1.397822671	0.985637861	NA
ASV_16s_19754	0.192917965	0.357452294	3.550563474	1.054231629	0.99390563	NA
ASV_16s_19755	0.120625169	-0.027941997	3.551190123	0.75669453	NA	NA
ASV_16s_19756	0.120625169	-0.027941997	3.551190123	0.75669453	NA	NA
ASV_16s_19757	0.140345975	0.355831566	3.550937409	0.836481373	0.997053579	NA
ASV_16s_19758	0.137829607	-0.073154832	3.550950292	0.781985731	NA	NA
ASV_16s_19759	0.152842843	0.317439866	3.550821067	0.891566717	0.996393373	NA
ASV_16s_19760	0.153997707	0.328850569	3.550821579	0.89251909	0.996381186	NA
ASV_16s_19761	0.100665178	-0.072592778	3.551110003	0.644196216	NA	NA
ASV_16s_19762	0.100665178	-0.072592778	3.551110003	0.644196216	NA	NA
ASV_16s_19763	0.100665178	-0.072592778	3.551110003	0.644196216	NA	NA
ASV_16s_19764	0.100665178	-0.072592778	3.551110003	0.644196216	NA	NA
ASV_16s_19765	0.1315537	0.269716114	3.550924033	0.739011702	0.998017704	NA
ASV_16s_19766	0.131041073	0.318559215	3.551040848	0.800504107	0.997438805	NA
ASV_16s_19767	0.098856392	-0.072554799	3.55112081	0.636711104	NA	NA
ASV_16s_19768	0.098856392	-0.072554799	3.55112081	0.636711104	NA	NA
ASV_16s_19769	0.098856392	-0.072554799	3.55112081	0.636711104	NA	NA
ASV_16s_19770	0.098856392	-0.072554799	3.55112081	0.636711104	NA	NA
ASV_16s_19771	0.098856392	-0.072554799	3.55112081	0.636711104	NA	NA
ASV_16s_19772	0.122241243	0.274622279	3.551068126	0.764284446	0.997791589	NA
ASV_16s_19773	0.129070289	-0.073050835	3.550979806	0.742078441	0.997991124	NA
ASV_16s_19774	0.140429459	0.368341793	3.551125049	0.816152553	0.997275596	NA
ASV_16s_19775	0.122657133	-0.072965995	3.551003899	0.729244749	NA	NA
ASV_16s_19776	0.122657133	-0.072965995	3.551003899	0.729244749	NA	NA
ASV_16s_19777	0.129853392	0.026851619	3.551029179	0.702903183	NA	NA
ASV_16s_19778	0.139825211	0.281445236	3.550920862	0.834688824	0.997073616	NA
ASV_16s_19779	0.137599841	0.36025003	3.551329451	0.798876826	NA	NA
ASV_16s_19780	0.137599841	0.36025003	3.551329451	0.798876826	NA	NA
ASV_16s_19781	0.137599841	0.36025003	3.551329451	0.798876826	NA	NA
ASV_16s_19782	0.132862199	0.340540257	3.551778311	0.573649703	0.999129638	NA
ASV_16s_19783	0.129946727	0.332228065	3.551401929	0.589074234	0.999050581	NA
ASV_16s_19784	0.173484013	0.335914027	3.55124885	0.676573764	0.998509329	NA
ASV_16s_19785	0.122321505	0.36022248	3.551420172	0.745997745	NA	NA
ASV_16s_19786	0.122321505	0.36022248	3.551420172	0.745997745	NA	NA
ASV_16s_19787	0.122321505	0.36022248	3.551420172	0.745997745	NA	NA
ASV_16s_19788	0.122321505	0.36022248	3.551420172	0.745997745	NA	NA
ASV_16s_19789	0.122321505	0.36022248	3.551420172	0.745997745	NA	NA
ASV_16s_19790	0.122321505	0.36022248	3.551420172	0.745997745	NA	NA
ASV_16s_19791	0.122321505	0.36022248	3.551420172	0.745997745	NA	NA
ASV_16s_19792	0.122321505	0.36022248	3.551420172	0.745997745	NA	NA
ASV_16s_19793	0.122321505	0.36022248	3.551420172	0.745997745	NA	NA

ASV_16s_19794	0.122321505	0.36022248	3.551420172	0.745997745	NA	NA
ASV_16s_19795	0.122321505	0.36022248	3.551420172	0.745997745	NA	NA
ASV_16s_19796	0.122321505	0.36022248	3.551420172	0.745997745	NA	NA
ASV_16s_19797	0.122321505	0.36022248	3.551420172	0.745997745	NA	NA
ASV_16s_19798	0.15849131	0.315939908	3.551039515	0.822077087	NA	NA
ASV_16s_19799	0.160892225	0.315939782	3.551032874	0.827757483	0.997150252	NA
ASV_16s_19800	0.135727468	0.31594002	3.551114755	0.750964886	0.997912771	NA
ASV_16s_19801	0.133451083	0.315940034	3.551123674	0.74394095	0.997974866	NA
ASV_16s_19802	0.136999404	0.315940013	3.551109883	0.758038457	NA	NA
ASV_16s_19803	0.136999404	0.315940013	3.551109883	0.758038457	NA	NA
ASV_16s_19804	0.153406025	0.693240345	3.551106028	0.877845626	0.996565997	NA
ASV_16s_19805	0.13186647	0.315940043	3.551130055	0.740573199	0.998004199	NA
ASV_16s_19806	0.171798447	0.315939857	3.551004785	0.861658769	0.996762612	NA
ASV_16s_19807	0.127760123	0.315940069	3.551147345	0.726505389	NA	NA
ASV_16s_19808	0.127760123	0.315940069	3.551147345	0.726505389	NA	NA
ASV_16s_19809	0.127760123	0.315940069	3.551147345	0.726505389	NA	NA
ASV_16s_19810	0.127760123	0.315940069	3.551147345	0.726505389	NA	NA
ASV_16s_19811	0.127760123	0.315940069	3.551147345	0.726505389	NA	NA
ASV_16s_19812	0.127760123	0.315940069	3.551147345	0.726505389	NA	NA
ASV_16s_19813	0.127760123	0.315940069	3.551147345	0.726505389	NA	NA
ASV_16s_19814	0.127760123	0.315940069	3.551147345	0.726505389	NA	NA
ASV_16s_19815	0.127760123	0.315940069	3.551147345	0.726505389	NA	NA
ASV_16s_19816	0.127760123	0.315940069	3.551147345	0.726505389	NA	NA
ASV_16s_19817	0.172022758	0.59605022	3.550709891	0.631999066	NA	NA
ASV_16s_19818	0.121670696	0.687181551	3.551098669	0.623076599	NA	NA
ASV_16s_19819	0.121670696	0.687181551	3.551098669	0.623076599	NA	NA
ASV_16s_19820	0.219806623	0.687937763	3.550856806	0.902497418	NA	NA
ASV_16s_19821	0.261879785	0.734190514	3.550986697	1.085708252	0.993324978	NA
ASV_16s_19822	0.221481087	1.113551465	3.551032876	0.750167342	0.997919884	NA
ASV_16s_19823	0.187222764	1.095650318	3.551130366	0.665094317	0.998589796	NA
ASV_16s_19824	0.169010943	0.733420758	3.551210361	0.832793542	NA	NA
ASV_16s_19825	0.169010943	0.733420758	3.551210361	0.832793542	NA	NA
ASV_16s_19826	0.169010943	0.733420758	3.551210361	0.832793542	NA	NA
ASV_16s_19827	0.169010943	0.733420758	3.551210361	0.832793542	NA	NA
ASV_16s_19828	0.169010943	0.733420758	3.551210361	0.832793542	NA	NA
ASV_16s_19829	0.169010943	0.733420758	3.551210361	0.832793542	NA	NA
ASV_16s_19830	0.169010943	0.733420758	3.551210361	0.832793542	NA	NA
ASV_16s_19831	0.169010943	0.733420758	3.551210361	0.832793542	NA	NA
ASV_16s_19832	0.169010943	0.733420758	3.551210361	0.832793542	NA	NA
ASV_16s_19833	0.169010943	0.733420758	3.551210361	0.832793542	NA	NA
ASV_16s_19834	0.169010943	0.733420758	3.551210361	0.832793542	NA	NA
ASV_16s_19835	0.169010943	0.733420758	3.551210361	0.832793542	NA	NA
ASV_16s_19836	0.169010943	0.733420758	3.551210361	0.832793542	NA	NA
ASV_16s_19837	0.169010943	0.733420758	3.551210361	0.832793542	NA	NA
ASV_16s_19838	0.169010943	0.733420758	3.551210361	0.832793542	NA	NA
ASV_16s_19839	0.169010943	0.733420758	3.551210361	0.832793542	NA	NA
ASV_16s_19840	0.169010943	0.733420758	3.551210361	0.832793542	NA	NA
ASV_16s_19841	0.169010943	0.733420758	3.551210361	0.832793542	NA	NA
ASV_16s_19842	0.18338381	1.043889809	3.55101079	0.65106537	0.998684128	NA
ASV_16s_19843	0.083720528	0.690144677	3.551638323	0.539347699	NA	NA
ASV_16s_19844	0.083720528	0.690144677	3.551638323	0.539347699	NA	NA
ASV_16s_19845	0.083720528	0.690144677	3.551638323	0.539347699	NA	NA
ASV_16s_19846	0.083720528	0.690144677	3.551638323	0.539347699	NA	NA
ASV_16s_19847	0.15712542	0.692571233	3.551023308	0.760942065	NA	NA
ASV_16s_19848	0.15712542	0.692571233	3.551023308	0.760942065	NA	NA
ASV_16s_19849	0.15712542	0.692571233	3.551023308	0.760942065	NA	NA
ASV_16s_19850	0.159437155	0.692594259	3.551016531	0.767175046	0.997764691	NA
ASV_16s_19851	0.161583766	0.69261506	3.551010409	0.773464652	NA	NA
ASV_16s_19852	0.161583766	0.69261506	3.551010409	0.773464652	NA	NA
ASV_16s_19853	0.161583766	0.69261506	3.551010409	0.773464652	NA	NA
ASV_16s_19854	0.161583766	0.69261506	3.551010409	0.773464652	NA	NA
ASV_16s_19855	0.161583766	0.69261506	3.551010409	0.773464652	NA	NA
ASV_16s_19856	0.149056491	0.692485277	3.551048618	0.733295268	0.998066627	NA
ASV_16s_19857	0.145298308	0.692442045	3.551061352	0.721680733	0.998163549	NA
ASV_16s_19858	0.178018741	0.69275786	3.550968405	0.815354879	0.997284076	NA
ASV_16s_19859	0.127760123	0.692208449	3.551130555	0.668481874	NA	NA
ASV_16s_19860	0.127760123	0.692208449	3.551130555	0.668481874	NA	NA

ASV_16s_19861	0.127760123	0.692208449	3.551130555	0.668481874	NA	NA
ASV_16s_19862	0.127760123	0.692208449	3.551130555	0.668481874	NA	NA
ASV_16s_19863	0.127760123	0.692208449	3.551130555	0.668481874	NA	NA
ASV_16s_19864	0.127760123	0.692208449	3.551130555	0.668481874	NA	NA
ASV_16s_19865	0.127760123	0.692208449	3.551130555	0.668481874	NA	NA
ASV_16s_19866	0.127760123	0.692208449	3.551130555	0.668481874	NA	NA
ASV_16s_19867	0.127760123	0.692208449	3.551130555	0.668481874	NA	NA
ASV_16s_19868	0.149829245	0.692493779	3.551046114	0.732739932	0.998071337	NA
ASV_16s_19869	0.156613502	0.692565874	3.551024886	0.752595645	0.997898177	NA
ASV_16s_19870	0.187346754	0.692827925	3.550947812	0.839734981	NA	NA
ASV_16s_19871	0.106070161	0.297361847	3.551909876	0.463853033	NA	NA
ASV_16s_19872	0.106070161	0.297361847	3.551909876	0.463853033	NA	NA
ASV_16s_19873	0.106070161	0.297361847	3.551909876	0.463853033	NA	NA
ASV_16s_19874	0.106070161	0.297361847	3.551909876	0.463853033	NA	NA
ASV_16s_19875	0.106070161	0.297361847	3.551909876	0.463853033	NA	NA
ASV_16s_19876	0.106070161	0.297361847	3.551909876	0.463853033	NA	NA
ASV_16s_19877	0.106070161	0.297361847	3.551909876	0.463853033	NA	NA
ASV_16s_19878	0.106070161	0.297361847	3.551909876	0.463853033	NA	NA
ASV_16s_19879	0.106070161	0.297361847	3.551909876	0.463853033	NA	NA
ASV_16s_19880	0.106070161	0.297361847	3.551909876	0.463853033	NA	NA
ASV_16s_19881	0.106070161	0.297361847	3.551909876	0.463853033	NA	NA
ASV_16s_19882	0.106070161	0.297361847	3.551909876	0.463853033	NA	NA
ASV_16s_19883	0.106070161	0.297361847	3.551909876	0.463853033	NA	NA
ASV_16s_19884	0.106070161	0.297361847	3.551909876	0.463853033	NA	NA
ASV_16s_19885	0.106070161	0.297361847	3.551909876	0.463853033	NA	NA
ASV_16s_19886	0.106070161	0.297361847	3.551909876	0.463853033	NA	NA
ASV_16s_19887	0.106070161	0.297361847	3.551909876	0.463853033	NA	NA
ASV_16s_19888	0.106070161	0.297361847	3.551909876	0.463853033	NA	NA
ASV_16s_19889	0.106070161	0.297361847	3.551909876	0.463853033	NA	NA
ASV_16s_19890	0.106070161	0.297361847	3.551909876	0.463853033	NA	NA
ASV_16s_19891	0.106070161	0.297361847	3.551909876	0.463853033	NA	NA
ASV_16s_19892	0.106070161	0.297361847	3.551909876	0.463853033	NA	NA
ASV_16s_19893	0.112272366	0.097193484	3.551712515	0.610541118	0.998932705	NA
ASV_16s_19894	0.107623674	0.068031884	3.551704544	0.624163159	0.998853024	NA
ASV_16s_19895	0.107882593	0.036201794	3.551667868	0.640083928	0.998754951	NA
ASV_16s_19896	0.106110089	0.048030588	3.55169105	0.631315203	0.998809634	NA
ASV_16s_19897	0.107882593	0.036201794	3.551667868	0.640083928	0.998754951	NA
ASV_16s_19898	0.108141512	0.00585653	3.551634401	0.653706488	0.998666702	NA
ASV_16s_19899	0.118474571	-0.054000665	3.551507546	0.706688034	0.99828382	NA
ASV_16s_19900	0.106121498	-0.0097754	3.551630486	0.654378523	0.998662243	NA
ASV_16s_19901	0.106121498	-0.0097754	3.551630486	0.654378523	0.998662243	NA
ASV_16s_19902	0.10840043	-0.023544432	3.551600822	0.665098042	0.998589771	NA
ASV_16s_19903	0.108659349	-0.051738648	3.551571108	0.674308091	0.998525447	NA
ASV_16s_19904	0.108918268	-0.078924838	3.551544759	0.681181061	0.998476191	NA
ASV_16s_19905	0.112312295	-0.096474923	3.551506756	0.696502682	0.998362465	NA
ASV_16s_19906	0.106132906	-0.063975574	3.551576264	0.668878834	0.998563597	NA
ASV_16s_19907	0.106132906	-0.063975574	3.551576264	0.668878834	0.998563597	NA
ASV_16s_19908	0.109177187	-0.105327921	3.551520895	0.685662526	0.99844349	NA
ASV_16s_19909	0.10613861	-0.089784023	3.551553447	0.672779061	0.998536259	NA
ASV_16s_19910	0.109436106	-0.131321321	3.551496287	0.687716295	0.998428349	NA
ASV_16s_19911	0.106144314	-0.114984148	3.551533288	0.67429504	0.99852554	NA
ASV_16s_19912	0.109436106	-0.131321321	3.551496287	0.687716295	0.998428349	NA
ASV_16s_19913	0.109695025	-0.156689227	3.551474506	0.687617066	0.998429083	NA
ASV_16s_19914	0.109695025	-0.156689227	3.551474506	0.687617066	0.998429083	NA
ASV_16s_19915	0.109695025	-0.156689227	3.551474506	0.687617066	0.998429083	NA
ASV_16s_19916	0.109695025	-0.156689227	3.551474506	0.687617066	0.998429083	NA
ASV_16s_19917	0.109953944	-0.18155947	3.551455321	0.68515923	0.998447186	NA
ASV_16s_19918	0.120287003	-0.224114783	3.551342862	0.725666775	0.998130659	NA
ASV_16s_19919	0.110212862	-0.205960465	3.551438154	0.68026323	0.998482831	NA
ASV_16s_19920	0.110212862	-0.205960465	3.551438154	0.68026323	0.998482831	NA
ASV_16s_19921	0.110471781	-0.230147493	3.551420143	0.673099103	0.998534	NA
ASV_16s_19922	0.134296904	-0.313920207	3.551178449	0.760796237	0.997823762	NA
ASV_16s_19923	0.110471781	-0.230147493	3.551420143	0.673099103	0.998534	NA
ASV_16s_19924	0.108446063	-0.221577192	3.551440347	0.666181601	0.998582302	NA
ASV_16s_19925	0.110471781	-0.230147493	3.551420143	0.673099103	0.998534	NA
ASV_16s_19926	0.109211411	-0.2480962	3.551416777	0.658877633	0.998632137	NA
ASV_16s_19927	0.1107307	-0.254370887	3.551401118	0.663807987	0.998598628	NA

ASV_16s_19928	0.131454327	-0.326325151	3.551198687	0.735429043	0.998048461	NA
ASV_16s_19929	0.10871639	-0.292654836	3.55139419	0.631787559	0.99880673	NA
ASV_16s_19930	0.115453326	-0.317758809	3.551331398	0.651237312	0.998682998	NA
ASV_16s_19931	0.179940261	-0.60309155	3.550880997	0.651003883	NA	NA
ASV_16s_19932	0.144713135	-0.536216766	3.551096804	0.575005197	NA	NA
ASV_16s_19933	0.144713135	-0.536216766	3.551096804	0.575005197	NA	NA
ASV_16s_19934	0.144713135	-0.536216766	3.551096804	0.575005197	NA	NA
ASV_16s_19935	0.144713135	-0.536216766	3.551096804	0.575005197	NA	NA
ASV_16s_19936	0.146976804	-0.541037247	3.551080819	0.579500919	0.999100193	NA
ASV_16s_19937	0.146976804	-0.541037247	3.551080819	0.579500919	0.999100193	NA
ASV_16s_19938	0.137758451	-0.519666141	3.551141953	0.560620097	0.999192855	NA
ASV_16s_19939	0.137758451	-0.519666141	3.551141953	0.560620097	0.999192855	NA
ASV_16s_19940	0.121024108	-0.475423736	3.551256854	0.51482269	0.999390376	NA
ASV_16s_19941	0.147499189	-0.542139678	3.551077184	0.580547499	0.999094856	NA
ASV_16s_19942	0.149240474	-0.545787164	3.551065215	0.584060196	NA	NA
ASV_16s_19943	0.149240474	-0.545787164	3.551065215	0.584060196	NA	NA
ASV_16s_19944	0.140311638	-0.52599276	3.551125832	0.565632007	0.999168919	NA
ASV_16s_19945	0.146384495	-0.539782194	3.551084965	0.578318804	NA	NA
ASV_16s_19946	0.125718375	-0.48846524	3.551222461	0.527862186	0.999337931	NA
ASV_16s_19947	0.112802051	-0.451486976	3.551322133	0.498731779	NA	NA
ASV_16s_19948	0.112802051	-0.451486976	3.551322133	0.498731779	NA	NA
ASV_16s_19949	0.112802051	-0.451486976	3.551322133	0.498731779	NA	NA
ASV_16s_19950	0.112802051	-0.451486976	3.551322133	0.498731779	NA	NA
ASV_16s_19951	0.112802051	-0.451486976	3.551322133	0.498731779	NA	NA
ASV_16s_19952	0.110016688	-0.442997757	3.551345975	0.490976633	0.999478855	NA
ASV_16s_19953	0.108750614	-0.439073077	3.551357121	0.487406389	0.9994913	NA
ASV_16s_19954	0.106218467	-0.431101664	3.55138001	0.480228371	NA	NA
ASV_16s_19955	0.106218467	-0.431101664	3.55138001	0.480228371	NA	NA
ASV_16s_19956	0.106218467	-0.431101664	3.55138001	0.480228371	NA	NA
ASV_16s_19957	0.106218467	-0.431101664	3.55138001	0.480228371	NA	NA
ASV_16s_19958	0.106218467	-0.431101664	3.55138001	0.480228371	NA	NA
ASV_16s_19959	0.106218467	-0.431101664	3.55138001	0.480228371	NA	NA
ASV_16s_19960	0.106218467	-0.431101664	3.55138001	0.480228371	NA	NA
ASV_16s_19961	0.106218467	-0.431101664	3.55138001	0.480228371	NA	NA
ASV_16s_19962	0.106218467	-0.431101664	3.55138001	0.480228371	NA	NA
ASV_16s_19963	0.106218467	-0.431101664	3.55138001	0.480228371	NA	NA
ASV_16s_19964	0.106218467	-0.431101664	3.55138001	0.480228371	NA	NA
ASV_16s_19965	0.106218467	-0.431101664	3.55138001	0.480228371	NA	NA
ASV_16s_19966	0.106218467	-0.431101664	3.55138001	0.480228371	NA	NA
ASV_16s_19967	0.106218467	-0.431101664	3.55138001	0.480228371	NA	NA
ASV_16s_19968	0.106218467	-0.431101664	3.55138001	0.480228371	NA	NA
ASV_16s_19969	0.106218467	-0.431101664	3.55138001	0.480228371	NA	NA
ASV_16s_19970	0.106218467	-0.431101664	3.55138001	0.480228371	NA	NA
ASV_16s_19971	0.106218467	-0.431101664	3.55138001	0.480228371	NA	NA
ASV_16s_19972	0.106218467	-0.431101664	3.55138001	0.480228371	NA	NA
ASV_16s_19973	0.135301428	1.067156208	3.551571396	0.370345718	0.9997965	NA
ASV_16s_19974	0.033841883	-0.146908027	3.55118545	0.137535899	NA	NA
ASV_16s_19975	0.034833439	0.052803815	3.551174453	0.210999806	0.999969788	NA
ASV_16s_19977	0.118123032	0.00574699	3.551542462	0.662601722	0.998606877	NA
ASV_16s_19978	0.108443991	-0.096052081	3.551527355	0.566775528	0.999163391	NA
ASV_16s_19979	0.11240691	0.536123738	3.551944027	0.458643347	0.999584266	NA
ASV_16s_19980	0.108678713	0.063384416	3.551620462	0.566673825	0.999163884	NA
ASV_16s_19981	0.141072014	-0.480648049	3.551285976	0.544885327	0.999264968	NA
ASV_16s_19982	0.130404746	-0.171792635	3.551456928	0.478495106	0.999521476	NA
ASV_16s_19983	0.113761479	-0.386232734	3.551467902	0.525173205	0.999348987	NA
ASV_16s_19984	0.118488755	0.007556457	3.551830737	0.509335552	0.999411575	NA
ASV_16s_19985	0.119571286	0.261253357	3.551689769	0.625402564	0.998845582	NA
ASV_16s_19986	0.142253551	-0.486295608	3.551240628	0.564909212	0.9991724	NA
ASV_16s_19987	0.137044049	-0.485312686	3.551221124	0.561041195	0.999190862	NA
ASV_16s_19988	0.144947698	-0.504379759	3.551155378	0.589928459	0.999046067	NA
ASV_16s_19989	0.143034376	-0.497735681	3.551173714	0.584282237	0.999075639	NA
ASV_16s_19990	0.147962241	-0.505638438	3.551097809	0.615780563	0.998902513	NA
ASV_16s_19991	0.113948214	0.018661957	3.551538782	0.43083449	0.999662407	NA
ASV_16s_19992	0.110218746	-0.038220607	3.551408468	0.488327833	0.999488108	NA
ASV_16s_19993	0.128455404	-0.421386389	3.551520687	0.490496822	0.99948054	NA
ASV_16s_19994	0.143575145	-0.475412639	3.551109536	0.617629017	0.998891726	NA
ASV_16s_19995	0.115699535	0.137333457	3.551477824	0.593896019	0.99902491	NA

ASV_16s_19996	0.11370464	0.293709415	3.551463078	0.577390595	0.999110889	NA
ASV_16s_19997	0.177304261	0.718112017	3.551436192	0.864034513	0.996734227	NA
ASV_16s_19998	0.112139029	0.669669649	3.551725944	0.515799156	0.99938655	NA
ASV_16s_19999	0.14192062	-0.009986703	3.55101174	0.633341428	0.998797144	NA
ASV_16s_20000	0.141881431	-0.407901298	3.55104786	0.649697051	0.998693096	NA
ASV_16s_20001	0.127257389	0.233014862	3.551209961	0.727954928	0.998111603	NA
ASV_16s_20002	0.11193062	0.329732384	3.551896291	0.42440887	0.999678909	NA
ASV_16s_20003	0.178638354	0.720220324	3.551409851	0.868221602	0.996683806	NA
ASV_16s_20004	0.138359386	0.013341023	3.551061915	0.626205198	0.998840745	NA
ASV_16s_20005	0.141268177	-0.38232571	3.551047403	0.656591938	0.998647488	NA
ASV_16s_20006	0.136768352	-0.377903216	3.551089149	0.636865391	0.998775213	NA
ASV_16s_20007	0.137496083	-0.360345122	3.55108079	0.644875255	0.998724373	NA
ASV_16s_20008	0.134102141	-0.340979207	3.551108099	0.634786222	0.998788185	NA
ASV_16s_20009	0.092366103	0.600668879	3.551882847	0.506183305	0.999423524	NA
ASV_16s_20010	0.103640741	0.331095141	3.551922471	0.248101027	0.999947501	NA
ASV_16s_20011	0.119992187	0.308313584	3.5518486	0.424660047	0.999678275	NA
ASV_16s_20012	0.146475317	-0.290861562	3.55144058	0.732248787	0.998075496	NA
ASV_16s_20013	0.146376937	-0.27257323	3.551798664	0.423389712	0.999681475	NA
ASV_16s_20014	0.136675479	0.544838463	3.551512214	0.449530036	0.999611104	NA
ASV_16s_20015	0.139247792	-0.285212479	3.551081407	0.663797061	0.998598703	NA
ASV_16s_20016	0.14004167	-0.285212808	3.551080512	0.666660443	0.998578994	NA
ASV_16s_20017	0.14004167	-0.285212808	3.551080512	0.666660443	0.998578994	NA
ASV_16s_20018	0.12274599	-0.091086442	3.551736544	0.225440514	0.999962123	NA
ASV_16s_20019	0.146825359	0.34353306	3.551284235	0.510556701	0.999406902	NA
ASV_16s_20020	0.139428416	-0.224599983	3.551091934	0.669720307	0.998557728	NA
ASV_16s_20021	0.133050676	-0.224599983	3.551091934	0.64845294	0.998701215	NA
ASV_16s_20022	0.145892641	0.147593392	3.551061892	0.650980952	0.998684683	NA
ASV_16s_20023	0.130399626	0.679937718	3.551343561	0.620448995	0.998875132	NA
ASV_16s_20024	0.140299068	0.524871916	3.551230501	0.676074484	0.998512891	NA
ASV_16s_20025	0.137680709	-0.165669909	3.5510993	0.668519669	0.998566097	NA
ASV_16s_20026	0.137152454	-0.108352875	3.551236951	0.578321487	0.999106181	NA
ASV_16s_20027	0.139127091	0.682723826	3.551221807	0.656083771	0.998650885	NA
ASV_16s_20028	0.136928161	0.310857369	3.551400147	0.534329271	0.99931082	NA
ASV_16s_20029	0.138160679	0.314504835	3.551753655	0.485321073	0.999498474	NA
ASV_16s_20030	0.134503764	0.168167222	3.550433623	0.555556657	0.99921656	NA
ASV_16s_20031	0.136168487	1.216527579	3.551460746	0.347730244	0.999835344	NA
ASV_16s_20032	0.134643358	-0.002380773	3.5511753	0.652785979	0.998672793	NA
ASV_16s_20033	0.15249074	-0.735635342	3.551205642	0.410600556	0.999712483	NA
ASV_16s_20034	0.116219165	0.369433564	3.551145856	0.567914988	0.999157858	NA
ASV_16s_20035	0.115410708	0.701986344	3.551323283	0.364635401	0.999806847	NA
ASV_16s_20036	0.134109301	0.861346108	3.551502672	0.47035444	0.999547954	NA
ASV_16s_20037	0.145382282	0.48072319	3.551047411	0.690025206	NA	NA
ASV_16s_20038	0.138724583	0.032399029	3.551876685	0.334564183	0.999855417	NA
ASV_16s_20039	0.13728654	1.323628455	3.551344265	0.283239125	0.999917664	NA
ASV_16s_20040	0.145271662	0.258147817	3.550926876	0.486786003	0.999493442	NA
ASV_16s_20041	0.121050411	0.223108934	3.551355445	0.231477394	0.99995855	NA
ASV_16s_20042	0.117594246	1.034499005	3.551452575	0.460334614	NA	NA
ASV_16s_20043	0.117594246	1.034499005	3.551452575	0.460334614	NA	NA
ASV_16s_20044	0.117594246	1.034499005	3.551452575	0.460334614	NA	NA
ASV_16s_20045	0.121492112	1.261846435	3.551415516	0.292660826	0.999908009	NA
ASV_16s_20046	0.148316716	0.682644208	3.551061822	0.715161136	0.998216514	NA
ASV_16s_20047	0.132320038	1.07593075	3.551363745	0.49723451	NA	NA
ASV_16s_20048	0.132320038	1.07593075	3.551363745	0.49723451	NA	NA
ASV_16s_20049	0.121403466	1.045751817	3.551430249	0.470086885	NA	NA
ASV_16s_20050	0.121403466	1.045751817	3.551430249	0.470086885	NA	NA
ASV_16s_20051	0.121403466	1.045751817	3.551430249	0.470086885	NA	NA
ASV_16s_20052	0.121403466	1.045751817	3.551430249	0.470086885	NA	NA
ASV_16s_20053	0.121403466	1.045751817	3.551430249	0.470086885	NA	NA
ASV_16s_20054	0.115098829	0.338271458	3.551932879	0.489071881	NA	NA
ASV_16s_20055	0.13934117	0.346609922	3.55185337	0.546130631	NA	NA
ASV_16s_20056	0.15291425	1.060963591	3.551377001	0.457404003	0.999587988	NA
ASV_16s_20057	0.203210757	-0.351028144	3.55062198	0.929055024	0.995893259	NA
ASV_16s_20058	0.154117309	0.351044895	3.551786842	0.57688464	NA	NA
ASV_16s_20059	0.201881826	0.373772295	3.551503562	0.688677903	NA	NA
ASV_16s_20060	0.145266898	0.347658514	3.551971785	0.302472203	0.999897146	NA
ASV_16s_20061	0.089194717	0.329219384	3.551997482	0.414834421	NA	NA
ASV_16s_20062	0.089194717	0.329219384	3.551997482	0.414834421	NA	NA

ASV_16s_20063	0.120771637	0.340259019	3.551913051	0.503920571	NA	NA
ASV_16s_20064	0.120771637	0.340259019	3.551913051	0.503920571	NA	NA
ASV_16s_20065	0.120771637	0.340259019	3.551913051	0.503920571	NA	NA
ASV_16s_20066	0.141904943	0.740321518	3.551822169	0.433522284	0.999655335	NA
ASV_16s_20067	0.162115851	0.354915166	3.551740199	0.59429815	NA	NA
ASV_16s_20068	0.218956991	-0.378858985	3.550894762	0.788349573	0.99756104	NA
ASV_16s_20069	0.174116806	-0.697443112	3.550827274	0.401915135	0.999732318	NA
ASV_16s_20070	0.149240474	-0.498477501	3.55113107	0.571393202	NA	NA
ASV_16s_20071	0.183948927	-0.962090399	3.550751956	0.34173749	0.999844704	NA
ASV_16s_20072	0.089079765	-0.351238566	3.551640581	0.421850136	NA	NA
ASV_16s_20073	0.149663697	1.392921459	3.551179234	0.318759052	0.999877197	NA
ASV_16s_20074	0.174752419	0.494551912	3.550987869	0.742524583	0.997987237	NA
ASV_16s_20075	0.132072034	1.113868655	3.55111413	0.438435036	NA	NA
ASV_16s_20076	0.132072034	1.113868655	3.55111413	0.438435036	NA	NA
ASV_16s_20077	0.132072034	1.113868655	3.55111413	0.438435036	NA	NA
ASV_16s_20078	0.103524865	0.465628986	3.551110134	0.564529162	0.999174226	NA
ASV_16s_20079	0.068928879	0.90712091	3.551597542	0.263248257	0.999935777	NA
ASV_16s_20080	0.068928879	0.90712091	3.551597542	0.263248257	0.999935777	NA
ASV_16s_20081	0.095442457	1.007078486	3.551369857	0.342693321	0.999843237	NA
ASV_16s_20082	0.11816838	1.077144559	3.551196517	0.404042276	NA	NA
ASV_16s_20083	0.11816838	1.077144559	3.551196517	0.404042276	NA	NA
ASV_16s_20084	0.11816838	1.077144559	3.551196517	0.404042276	NA	NA
ASV_16s_20085	0.11816838	1.077144559	3.551196517	0.404042276	NA	NA
ASV_16s_20086	0.11816838	1.077144559	3.551196517	0.404042276	NA	NA
ASV_16s_20087	0.150086956	0.037546332	3.55086573	0.761354293	0.997818636	NA
ASV_16s_20088	0.135499802	0.363314566	3.551534581	0.475117279	NA	NA
ASV_16s_20089	0.135499802	0.363314566	3.551534581	0.475117279	NA	NA
ASV_16s_20090	0.135499802	0.363314566	3.551534581	0.475117279	NA	NA
ASV_16s_20091	0.123028266	0.357644646	3.551591694	0.447259319	NA	NA
ASV_16s_20092	0.123028266	0.357644646	3.551591694	0.447259319	NA	NA
ASV_16s_20093	0.123028266	0.357644646	3.551591694	0.447259319	NA	NA
ASV_16s_20094	0.123028266	0.357644646	3.551591694	0.447259319	NA	NA
ASV_16s_20095	0.123028266	0.357644646	3.551591694	0.447259319	NA	NA
ASV_16s_20096	0.101558381	0.347323867	3.551689158	0.39099098	NA	NA
ASV_16s_20097	0.101558381	0.347323867	3.551689158	0.39099098	NA	NA
ASV_16s_20098	0.101558381	0.347323867	3.551689158	0.39099098	NA	NA
ASV_16s_20099	0.101558381	0.347323867	3.551689158	0.39099098	NA	NA
ASV_16s_20100	0.173974151	0.379978392	3.551314952	0.55600233	NA	NA
ASV_16s_20101	0.173974151	0.379978392	3.551314952	0.55600233	NA	NA
ASV_16s_20102	0.109882569	0.350942485	3.551651913	0.413552048	NA	NA
ASV_16s_20103	0.109882569	0.350942485	3.551651913	0.413552048	NA	NA
ASV_16s_20104	0.109882569	0.350942485	3.551651913	0.413552048	NA	NA
ASV_16s_20105	0.164884283	0.086609521	3.550380484	0.891229239	0.996397685	NA
ASV_16s_20106	0.038260174	0.339073382	3.551921011	0.174803271	0.999984144	NA
ASV_16s_20107	0.038260174	0.339073382	3.551921011	0.174803271	0.999984144	NA
ASV_16s_20108	0.132834506	0.194644609	3.55128242	0.631211156	0.998810273	NA
ASV_16s_20109	0.061736609	-0.270332951	3.551533258	0.267542317	NA	NA
ASV_16s_20110	0.151445153	-0.508435213	3.550757067	0.508253208	NA	NA
ASV_16s_20111	0.172679986	-0.529397558	3.550567463	0.550998429	0.999237493	NA
ASV_16s_20112	0.091295843	0.068467562	3.550976319	0.305060885	0.999894138	NA
ASV_16s_20113	0.074977696	-0.326780782	3.551441085	0.313821907	NA	NA
ASV_16s_20114	0.074977696	-0.326780782	3.551441085	0.313821907	NA	NA
ASV_16s_20115	0.074977696	-0.326780782	3.551441085	0.313821907	NA	NA
ASV_16s_20116	0.074977696	-0.326780782	3.551441085	0.313821907	NA	NA
ASV_16s_20117	0.068715445	-0.301011432	3.55148572	0.292428132	NA	NA
ASV_16s_20118	0.054473272	0.841269116	3.551729533	0.214741713	0.999967917	NA
ASV_16s_20119	0.134776678	1.12059327	3.551099699	0.44491168	NA	NA
ASV_16s_20120	0.123028266	-0.078141296	3.550958316	0.706320651	NA	NA
ASV_16s_20121	0.123028266	-0.078141296	3.550958316	0.706320651	NA	NA
ASV_16s_20122	0.123028266	-0.078141296	3.550958316	0.706320651	NA	NA
ASV_16s_20123	0.123028266	-0.078141296	3.550958316	0.706320651	NA	NA
ASV_16s_20124	0.123028266	-0.078141296	3.550958316	0.706320651	NA	NA
ASV_16s_20125	0.123028266	-0.078141296	3.550958316	0.706320651	NA	NA
ASV_16s_20126	0.123028266	-0.078141296	3.550958316	0.706320651	NA	NA
ASV_16s_20127	0.104907602	-0.077886141	3.551034382	0.637169626	NA	NA
ASV_16s_20128	0.104907602	-0.077886141	3.551034382	0.637169626	NA	NA
ASV_16s_20129	0.104907602	-0.077886141	3.551034382	0.637169626	NA	NA

ASV_16s_20130	0.104907602	-0.077886141	3.551034382	0.637169626	NA	NA
ASV_16s_20131	0.104907602	-0.077886141	3.551034382	0.637169626	NA	NA
ASV_16s_20132	0.104907602	-0.077886141	3.551034382	0.637169626	NA	NA
ASV_16s_20133	0.104907602	-0.077886141	3.551034382	0.637169626	NA	NA
ASV_16s_20134	0.104907602	-0.077886141	3.551034382	0.637169626	NA	NA
ASV_16s_20135	0.104907602	-0.077886141	3.551034382	0.637169626	NA	NA
ASV_16s_20136	0.072288833	0.220235474	3.55097655	0.334067817	0.999856139	NA
ASV_16s_20137	0.072995744	0.220232562	3.550972701	0.337518133	0.99985107	NA
ASV_16s_20138	0.0733492	0.220231126	3.550970805	0.339236686	0.999848499	NA
ASV_16s_20139	0.073702655	0.220229704	3.550968926	0.340950862	0.999845905	NA
ASV_16s_20140	0.074409567	0.220226767	3.550965219	0.344365901	0.999840645	NA
ASV_16s_20141	0.078100083	0.220213101	3.550946961	0.36191906	NA	NA
ASV_16s_20142	0.078100083	0.220213101	3.550946961	0.36191906	NA	NA
ASV_16s_20143	0.078100083	0.220213101	3.550946961	0.36191906	NA	NA
ASV_16s_20144	0.078100083	0.220213101	3.550946961	0.36191906	NA	NA
ASV_16s_20145	0.125571779	0.310954753	3.551108146	0.695906294	NA	NA
ASV_16s_20146	0.125571779	0.310954753	3.551108146	0.695906294	NA	NA
ASV_16s_20147	0.13329588	0.310952816	3.551078222	0.721566552	NA	NA
ASV_16s_20148	0.13329588	0.310952816	3.551078222	0.721566552	NA	NA
ASV_16s_20149	0.13329588	0.310952816	3.551078222	0.721566552	NA	NA
ASV_16s_20150	0.116227959	0.310957433	3.551149614	0.661949161	NA	NA
ASV_16s_20151	0.132873968	0.310952916	3.551079768	0.720335417	NA	NA
ASV_16s_20152	0.132873968	0.310952916	3.551079768	0.720335417	NA	NA
ASV_16s_20153	0.082272274	0.595606468	3.55088352	0.328927377	NA	NA
ASV_16s_20154	0.082272274	0.595606468	3.55088352	0.328927377	NA	NA
ASV_16s_20155	0.082272274	0.595606468	3.55088352	0.328927377	NA	NA
ASV_16s_20156	0.119219374	0.687146999	3.551109824	0.614440983	NA	NA
ASV_16s_20157	0.093582838	-0.226879189	3.551615635	0.294153773	0.99990641	NA
ASV_16s_20158	0.133154326	1.239124405	3.551374285	0.315021564	0.999881993	NA
ASV_16s_20159	0.113589542	0.32521934	3.551997861	0.389548333	0.999758906	NA
ASV_16s_20160	0.116645182	0.343861145	3.55189668	0.344242661	0.999840837	NA
ASV_16s_20161	0.110358304	-0.063534161	3.551231551	0.708397842	0.998270376	NA
ASV_16s_20162	0.13626256	0.298146102	3.550916579	0.788048375	0.997564019	NA
ASV_16s_20163	0.175756794	0.215722085	3.551197035	0.822733108	0.997204974	NA
ASV_16s_20164	0.118011314	-0.049443629	3.55131599	0.735534964	0.998047556	NA
ASV_16s_20165	0.126134231	0.665897873	3.551755573	0.537679618	0.999296484	NA
ASV_16s_20166	0.093713289	0.21235491	3.551507988	0.458306549	0.999585279	NA
ASV_16s_20167	0.11798929	-0.042319031	3.551370464	0.729875077	0.998095512	NA
ASV_16s_20168	0.193190841	-0.05933162	3.551551932	0.616650511	0.998897445	NA
ASV_16s_20169	0.11432245	0.011921761	3.551496996	0.700652202	0.998330721	NA
ASV_16s_20170	0.114817969	0.052100036	3.55150827	0.695547613	0.998369713	NA
ASV_16s_20171	0.11792322	0.052100036	3.55150827	0.706633546	0.998284247	NA
ASV_16s_20172	0.115313487	0.10700672	3.551514797	0.691599805	0.998399451	NA
ASV_16s_20173	0.144121951	1.034522734	3.551396805	0.520615763	NA	NA
ASV_16s_20174	0.141425873	1.028479519	3.551415434	0.514127689	0.99939309	NA
ASV_16s_20175	0.194367171	1.126015752	3.551069707	0.620123466	NA	NA
ASV_16s_20176	0.088443217	0.28570063	3.551781637	0.416093052	0.999699433	NA
ASV_16s_20177	0.088443217	0.28570063	3.551781637	0.416093052	0.999699433	NA
ASV_16s_20178	0.073646364	0.287357399	3.551835037	0.366121067	0.999804191	NA
ASV_16s_20179	0.239622119	-0.562600778	3.550371175	0.777670351	NA	NA
ASV_16s_20180	0.239622119	-0.562600778	3.550371175	0.777670351	NA	NA
ASV_16s_20181	0.239622119	-0.562600778	3.550371175	0.777670351	NA	NA
ASV_16s_20182	0.239622119	-0.562600778	3.550371175	0.777670351	NA	NA
ASV_16s_20183	0.201186699	0.046382305	3.551201049	0.884864645	0.996478379	NA
ASV_16s_20184	0.245285355	1.267292273	3.550810406	0.741519198	NA	NA
ASV_16s_20185	0.154503021	1.164483147	3.551000291	0.486169635	NA	NA
ASV_16s_20186	0.154503021	1.164483147	3.551000291	0.486169635	NA	NA
ASV_16s_20187	0.154503021	1.164483147	3.551000291	0.486169635	NA	NA
ASV_16s_20188	0.154503021	1.164483147	3.551000291	0.486169635	NA	NA
ASV_16s_20189	0.154503021	1.164483147	3.551000291	0.486169635	NA	NA
ASV_16s_20190	0.160481089	1.334594142	3.550998319	0.389738062	0.999758513	NA
ASV_16s_20191	0.135133941	0.859243981	3.551211785	0.59503337	0.999018787	NA
ASV_16s_20192	0.131502239	0.687020167	3.551218913	0.609527417	0.998938482	NA
ASV_16s_20193	0.200659052	0.618277116	3.551516551	0.688686518	0.998421162	NA
ASV_16s_20194	0.088040857	-0.077555709	3.551133042	0.565855623	NA	NA
ASV_16s_20195	0.088040857	-0.077555709	3.551133042	0.565855623	NA	NA
ASV_16s_20196	0.088040857	-0.077555709	3.551133042	0.565855623	NA	NA

ASV_16s_20197	0.088040857	-0.077555709	3.551133042	0.565855623	NA	NA
ASV_16s_20198	0.101721685	-0.064353425	3.551267478	0.66334003	0.998601832	NA
ASV_16s_20199	0.243633084	-0.028983218	3.550853178	1.145488235	NA	NA
ASV_16s_20200	0.176114538	0.332789187	3.551012416	1.287632624	0.988767283	NA
ASV_16s_20201	0.20500084	0.351832261	3.550466118	1.078230007	0.993465975	NA
ASV_16s_20202	0.153809119	0.501472913	3.551034393	0.786878456	0.997575568	NA
ASV_16s_20203	0.11615757	-0.027001622	3.551214115	0.739859682	NA	NA
ASV_16s_20204	0.11615757	-0.027001622	3.551214115	0.739859682	NA	NA
ASV_16s_20205	0.11615757	-0.027001622	3.551214115	0.739859682	NA	NA
ASV_16s_20206	0.132724806	-0.073096122	3.550967021	0.764596357	NA	NA
ASV_16s_20207	0.132724806	-0.073096122	3.550967021	0.764596357	NA	NA
ASV_16s_20208	0.132724806	-0.073096122	3.550967021	0.764596357	NA	NA
ASV_16s_20209	0.096936838	-0.072513067	3.551132784	0.628581077	NA	NA
ASV_16s_20210	0.096936838	-0.072513067	3.551132784	0.628581077	NA	NA
ASV_16s_20211	0.096936838	-0.072513067	3.551132784	0.628581077	NA	NA
ASV_16s_20212	0.096936838	-0.072513067	3.551132784	0.628581077	NA	NA
ASV_16s_20213	0.096936838	-0.072513067	3.551132784	0.628581077	NA	NA
ASV_16s_20214	0.096936838	-0.072513067	3.551132784	0.628581077	NA	NA
ASV_16s_20215	0.096936838	-0.072513067	3.551132784	0.628581077	NA	NA
ASV_16s_20216	0.127312733	0.321784319	3.551096654	0.774784406	0.997692852	NA
ASV_16s_20217	0.095195044	-0.072473676	3.551143999	0.621208984	NA	NA
ASV_16s_20218	0.095195044	-0.072473676	3.551143999	0.621208984	NA	NA
ASV_16s_20219	0.095195044	-0.072473676	3.551143999	0.621208984	NA	NA
ASV_16s_20220	0.095195044	-0.072473676	3.551143999	0.621208984	NA	NA
ASV_16s_20221	0.095195044	-0.072473676	3.551143999	0.621208984	NA	NA
ASV_16s_20222	0.102989995	0.08612062	3.551149736	0.63895675	NA	NA
ASV_16s_20223	0.160658487	-0.073372589	3.550888636	0.847099295	NA	NA
ASV_16s_20224	0.118114277	-0.072900204	3.551022662	0.712434753	NA	NA
ASV_16s_20225	0.118114277	-0.072900204	3.551022662	0.712434753	NA	NA
ASV_16s_20226	0.118114277	-0.072900204	3.551022662	0.712434753	NA	NA
ASV_16s_20227	0.118114277	-0.072900204	3.551022662	0.712434753	NA	NA
ASV_16s_20228	0.118114277	-0.072900204	3.551022662	0.712434753	NA	NA
ASV_16s_20229	0.118114277	-0.072900204	3.551022662	0.712434753	NA	NA
ASV_16s_20230	0.145583201	0.359996209	3.551026692	0.826354492	0.997165602	NA
ASV_16s_20231	0.13250355	0.360241503	3.551357499	0.780871224	NA	NA
ASV_16s_20232	0.13250355	0.360241503	3.551357499	0.780871224	NA	NA
ASV_16s_20233	0.13250355	0.360241503	3.551357499	0.780871224	NA	NA
ASV_16s_20234	0.13250355	0.360241503	3.551357499	0.780871224	NA	NA
ASV_16s_20235	0.181932214	0.360304154	3.551152162	0.932018647	0.995851915	NA
ASV_16s_20236	0.132214451	0.339248002	3.55176535	0.570191782	0.999146729	NA
ASV_16s_20237	0.13250355	0.360241503	3.551357499	0.780871224	NA	NA
ASV_16s_20238	0.117791078	0.360212851	3.551451626	0.729324349	NA	NA
ASV_16s_20239	0.117791078	0.360212851	3.551451626	0.729324349	NA	NA
ASV_16s_20240	0.117791078	0.360212851	3.551451626	0.729324349	NA	NA
ASV_16s_20241	0.117791078	0.360212851	3.551451626	0.729324349	NA	NA
ASV_16s_20242	0.117791078	0.360212851	3.551451626	0.729324349	NA	NA
ASV_16s_20243	0.117791078	0.360212851	3.551451626	0.729324349	NA	NA
ASV_16s_20244	0.117791078	0.360212851	3.551451626	0.729324349	NA	NA
ASV_16s_20245	0.117791078	0.360212851	3.551451626	0.729324349	NA	NA
ASV_16s_20246	0.117791078	0.360212851	3.551451626	0.729324349	NA	NA
ASV_16s_20247	0.117791078	0.360212851	3.551451626	0.729324349	NA	NA
ASV_16s_20248	0.117791078	0.360212851	3.551451626	0.729324349	NA	NA
ASV_16s_20249	0.117791078	0.360212851	3.551451626	0.729324349	NA	NA
ASV_16s_20250	0.117791078	0.360212851	3.551451626	0.729324349	NA	NA
ASV_16s_20251	0.117791078	0.360212851	3.551451626	0.729324349	NA	NA
ASV_16s_20252	0.117791078	0.360212851	3.551451626	0.729324349	NA	NA
ASV_16s_20253	0.117791078	0.360212851	3.551451626	0.729324349	NA	NA
ASV_16s_20254	0.152621262	0.315939948	3.551056834	0.802219646	NA	NA
ASV_16s_20255	0.152621262	0.315939948	3.551056834	0.802219646	NA	NA
ASV_16s_20256	0.142377533	0.315939999	3.551090367	0.769791507	0.997740158	NA
ASV_16s_20257	0.134410188	0.315940044	3.551119943	0.744745045	0.99796782	NA
ASV_16s_20258	0.132133803	0.315940058	3.551129041	0.737648025	0.998029449	NA
ASV_16s_20259	0.131925352	0.315940059	3.551129873	0.740360006	NA	NA
ASV_16s_20260	0.131925352	0.315940059	3.551129873	0.740360006	NA	NA
ASV_16s_20261	0.131925352	0.315940059	3.551129873	0.740360006	NA	NA
ASV_16s_20262	0.131925352	0.315940059	3.551129873	0.740360006	NA	NA
ASV_16s_20263	0.131925352	0.315940059	3.551129873	0.740360006	NA	NA

ASV_16s_20264	0.126108027	0.315940098	3.551154682	0.719677675	0.998179932	NA
ASV_16s_20265	0.125765831	0.3159401	3.551156212	0.718542585	0.998189172	NA
ASV_16s_20266	0.125765831	0.3159401	3.551156212	0.718542585	0.998189172	NA
ASV_16s_20267	0.148193023	0.315939969	3.551070853	0.787165987	0.997572733	NA
ASV_16s_20268	0.141901834	0.315940002	3.551092114	0.767394215	0.997762643	NA
ASV_16s_20269	0.123028266	0.315940119	3.551168755	0.709618104	NA	NA
ASV_16s_20270	0.123028266	0.315940119	3.551168755	0.709618104	NA	NA
ASV_16s_20271	0.123028266	0.315940119	3.551168755	0.709618104	NA	NA
ASV_16s_20272	0.123028266	0.315940119	3.551168755	0.709618104	NA	NA
ASV_16s_20273	0.123028266	0.315940119	3.551168755	0.709618104	NA	NA
ASV_16s_20274	0.123028266	0.315940119	3.551168755	0.709618104	NA	NA
ASV_16s_20275	0.123028266	0.315940119	3.551168755	0.709618104	NA	NA
ASV_16s_20276	0.123028266	0.315940119	3.551168755	0.709618104	NA	NA
ASV_16s_20277	0.123028266	0.315940119	3.551168755	0.709618104	NA	NA
ASV_16s_20278	0.123028266	0.315940119	3.551168755	0.709618104	NA	NA
ASV_16s_20279	0.145979262	0.614690079	3.550525364	0.627132219	0.998835142	NA
ASV_16s_20280	0.123028266	0.526949297	3.551104077	0.924864736	0.995951259	NA
ASV_16s_20281	0.203484961	1.078086249	3.550843368	0.667270156	0.998574773	NA
ASV_16s_20282	0.172287643	0.978740926	3.551095012	0.636671363	0.998776428	NA
ASV_16s_20283	0.192329494	0.733682879	3.551134143	0.894710594	NA	NA
ASV_16s_20284	0.192329494	0.733682879	3.551134143	0.894710594	NA	NA
ASV_16s_20285	0.170846854	0.733443472	3.551203801	0.834017475	0.997081097	NA
ASV_16s_20286	0.179815634	0.733550473	3.551172659	0.859404851	0.996789392	NA
ASV_16s_20287	0.162751279	0.733337729	3.55123459	0.812487825	NA	NA
ASV_16s_20288	0.162751279	0.733337729	3.55123459	0.812487825	NA	NA
ASV_16s_20289	0.162751279	0.733337729	3.55123459	0.812487825	NA	NA
ASV_16s_20290	0.162751279	0.733337729	3.55123459	0.812487825	NA	NA
ASV_16s_20291	0.162751279	0.733337729	3.55123459	0.812487825	NA	NA
ASV_16s_20292	0.162751279	0.733337729	3.55123459	0.812487825	NA	NA
ASV_16s_20293	0.162751279	0.733337729	3.55123459	0.812487825	NA	NA
ASV_16s_20294	0.162751279	0.733337729	3.55123459	0.812487825	NA	NA
ASV_16s_20295	0.080619768	0.684906799	3.551655714	0.525530078	NA	NA
ASV_16s_20296	0.080619768	0.684906799	3.551655714	0.525530078	NA	NA
ASV_16s_20297	0.080619768	0.684906799	3.551655714	0.525530078	NA	NA
ASV_16s_20298	0.080619768	0.684906799	3.551655714	0.525530078	NA	NA
ASV_16s_20299	0.080619768	0.684906799	3.551655714	0.525530078	NA	NA
ASV_16s_20300	0.080619768	0.684906799	3.551655714	0.525530078	NA	NA
ASV_16s_20301	0.15130596	0.692510208	3.551041335	0.741359028	NA	NA
ASV_16s_20302	0.15130596	0.692510208	3.551041335	0.741359028	NA	NA
ASV_16s_20303	0.161379738	0.692613093	3.551011044	0.768206077	0.997755045	NA
ASV_16s_20304	0.131729095	0.692265795	3.551113369	0.67869994	0.998494097	NA
ASV_16s_20305	0.127378681	0.692201458	3.551132345	0.665393865	0.998587734	NA
ASV_16s_20306	0.170680892	0.692696075	3.550986226	0.792907325	0.997515664	NA
ASV_16s_20307	0.155599182	0.692555678	3.551027946	0.752734783	NA	NA
ASV_16s_20308	0.155599182	0.692555678	3.551027946	0.752734783	NA	NA
ASV_16s_20309	0.155599182	0.692555678	3.551027946	0.752734783	NA	NA
ASV_16s_20310	0.155599182	0.692555678	3.551027946	0.752734783	NA	NA
ASV_16s_20311	0.155599182	0.692555678	3.551027946	0.752734783	NA	NA
ASV_16s_20312	0.155599182	0.692555678	3.551027946	0.752734783	NA	NA
ASV_16s_20313	0.155599182	0.692555678	3.551027946	0.752734783	NA	NA
ASV_16s_20314	0.155599182	0.692555678	3.551027946	0.752734783	NA	NA
ASV_16s_20315	0.155599182	0.692555678	3.551027946	0.752734783	NA	NA
ASV_16s_20316	0.123028266	0.692132655	3.551152645	0.652243155	NA	NA
ASV_16s_20317	0.123028266	0.692132655	3.551152645	0.652243155	NA	NA
ASV_16s_20318	0.123028266	0.692132655	3.551152645	0.652243155	NA	NA
ASV_16s_20319	0.123028266	0.692132655	3.551152645	0.652243155	NA	NA
ASV_16s_20320	0.123028266	0.692132655	3.551152645	0.652243155	NA	NA
ASV_16s_20321	0.123028266	0.692132655	3.551152645	0.652243155	NA	NA
ASV_16s_20322	0.123028266	0.692132655	3.551152645	0.652243155	NA	NA
ASV_16s_20323	0.145223174	0.692441053	3.551061707	0.717410297	0.998198358	NA
ASV_16s_20324	0.147304301	0.692465284	3.551054568	0.723618493	0.998147608	NA
ASV_16s_20325	0.149662155	0.692491932	3.551046718	0.730624927	0.998089204	NA
ASV_16s_20326	0.180735026	0.692779059	3.550962232	0.821419772	NA	NA
ASV_16s_20327	0.180735026	0.692779059	3.550962232	0.821419772	NA	NA
ASV_16s_20328	0.180596662	0.692777997	3.550962544	0.821033073	0.997223332	NA
ASV_16s_20329	0.180546348	0.692777611	3.550962657	0.820892477	0.997224847	NA
ASV_16s_20330	0.180407985	0.692776547	3.55096297	0.8205059	NA	NA

ASV_16s_20331	0.101990539	0.298113797	3.551922166	0.452334297	NA	NA
ASV_16s_20332	0.101990539	0.298113797	3.551922166	0.452334297	NA	NA
ASV_16s_20333	0.101990539	0.298113797	3.551922166	0.452334297	NA	NA
ASV_16s_20334	0.101990539	0.298113797	3.551922166	0.452334297	NA	NA
ASV_16s_20335	0.101990539	0.298113797	3.551922166	0.452334297	NA	NA
ASV_16s_20336	0.101990539	0.298113797	3.551922166	0.452334297	NA	NA
ASV_16s_20337	0.101990539	0.298113797	3.551922166	0.452334297	NA	NA
ASV_16s_20338	0.101990539	0.298113797	3.551922166	0.452334297	NA	NA
ASV_16s_20339	0.101990539	0.298113797	3.551922166	0.452334297	NA	NA
ASV_16s_20340	0.101990539	0.298113797	3.551922166	0.452334297	NA	NA
ASV_16s_20341	0.101990539	0.298113797	3.551922166	0.452334297	NA	NA
ASV_16s_20342	0.101990539	0.298113797	3.551922166	0.452334297	NA	NA
ASV_16s_20343	0.101990539	0.298113797	3.551922166	0.452334297	NA	NA
ASV_16s_20344	0.101990539	0.298113797	3.551922166	0.452334297	NA	NA
ASV_16s_20345	0.101990539	0.298113797	3.551922166	0.452334297	NA	NA
ASV_16s_20346	0.101990539	0.298113797	3.551922166	0.452334297	NA	NA
ASV_16s_20347	0.101990539	0.298113797	3.551922166	0.452334297	NA	NA
ASV_16s_20348	0.103544052	0.066134107	3.551727258	0.60380506	0.998970695	NA
ASV_16s_20349	0.10406189	0.00311402	3.551657106	0.631488561	0.998808569	NA
ASV_16s_20350	0.10406189	0.00311402	3.551657106	0.631488561	0.998808569	NA
ASV_16s_20351	0.102036172	0.0158704	3.551682253	0.621827499	0.998866961	NA
ASV_16s_20352	0.115945501	-0.090349596	3.551488169	0.699641566	0.998338489	NA
ASV_16s_20353	0.121276503	-0.115169166	3.5514199	0.727533123	0.998115125	NA
ASV_16s_20354	0.104579728	-0.055177738	3.551594936	0.650308421	0.998689094	NA
ASV_16s_20355	0.104579728	-0.055177738	3.551594936	0.650308421	0.998689094	NA
ASV_16s_20356	0.104579728	-0.055177738	3.551594936	0.650308421	0.998689094	NA
ASV_16s_20357	0.104579728	-0.055177738	3.551594936	0.650308421	0.998689094	NA
ASV_16s_20358	0.109771816	-0.08316115	3.551535931	0.67447078	0.998524294	NA
ASV_16s_20359	0.104579728	-0.055177738	3.551594936	0.650308421	0.998689094	NA
ASV_16s_20360	0.104579728	-0.055177738	3.551594936	0.650308421	0.998689094	NA
ASV_16s_20361	0.104838647	-0.082799532	3.551568151	0.656169059	0.998650315	NA
ASV_16s_20362	0.107421912	-0.096242164	3.551539993	0.667631524	0.998572268	NA
ASV_16s_20363	0.104838647	-0.082799532	3.551568151	0.656169059	0.998650315	NA
ASV_16s_20364	0.104838647	-0.082799532	3.551568151	0.656169059	0.998650315	NA
ASV_16s_20365	0.104838647	-0.082799532	3.551568151	0.656169059	0.998650315	NA
ASV_16s_20366	0.104838647	-0.082799532	3.551568151	0.656169059	0.998650315	NA
ASV_16s_20367	0.105097565	-0.109625171	3.551544503	0.659548949	0.998627606	NA
ASV_16s_20368	0.102058988	-0.094005922	3.551577686	0.646853512	0.998711602	NA
ASV_16s_20369	0.120597155	-0.180691513	3.551368331	0.728981476	0.998103012	NA
ASV_16s_20370	0.104084706	-0.104493845	3.551555323	0.655275758	0.998656275	NA
ASV_16s_20371	0.105356484	-0.135835448	3.551522913	0.660624229	0.998620328	NA
ASV_16s_20372	0.102064692	-0.119633732	3.551557234	0.647397448	0.998708076	NA
ASV_16s_20373	0.122147706	-0.208925685	3.551334447	0.733105142	0.998068241	NA
ASV_16s_20374	0.10409041	-0.129678305	3.551536434	0.655492954	0.998654828	NA
ASV_16s_20375	0.105615403	-0.161634726	3.551500786	0.659418724	0.998628486	NA
ASV_16s_20376	0.104349329	-0.155599347	3.551514486	0.6544884	0.998661513	NA
ASV_16s_20377	0.105615403	-0.161634726	3.551500786	0.659418724	0.998628486	NA
ASV_16s_20378	0.102070396	-0.144611606	3.551538656	0.645754035	0.99871871	NA
ASV_16s_20379	0.102070396	-0.144611606	3.551538656	0.645754035	0.99871871	NA
ASV_16s_20380	0.105874322	-0.186810998	3.551480737	0.655698197	0.998653459	NA
ASV_16s_20381	0.105874322	-0.186810998	3.551480737	0.655698197	0.998653459	NA
ASV_16s_20382	0.102081804	-0.19362237	3.551503372	0.635354061	0.998784651	NA
ASV_16s_20383	0.106133241	-0.211533559	3.551462948	0.649611035	0.998693659	NA
ASV_16s_20384	0.12679936	-0.290492813	3.551250071	0.727996415	0.998111256	NA
ASV_16s_20385	0.11581199	-0.274413357	3.551351832	0.673928244	0.998528138	NA
ASV_16s_20386	0.10639216	-0.236276573	3.551444753	0.641045247	0.998748855	NA
ASV_16s_20387	0.104619656	-0.228799583	3.551461944	0.635090268	0.998786294	NA
ASV_16s_20388	0.102093213	-0.242180745	3.551472053	0.616196691	0.998900091	NA
ASV_16s_20389	0.102098917	-0.266350503	3.551458533	0.603630983	0.998971665	NA
ASV_16s_20390	0.03646039	-0.103493529	3.551878194	0.224140568	0.999962863	NA
ASV_16s_20391	0.03646039	-0.103493529	3.551878194	0.224140568	0.999962863	NA
ASV_16s_20392	0.03646039	-0.103493529	3.551878194	0.224140568	0.999962863	NA
ASV_16s_20393	0.139147245	-0.523045683	3.551133319	0.558693782	NA	NA
ASV_16s_20394	0.139147245	-0.523045683	3.551133319	0.558693782	NA	NA
ASV_16s_20395	0.139147245	-0.523045683	3.551133319	0.558693782	NA	NA
ASV_16s_20396	0.140366144	-0.526052138	3.551125682	0.56134273	0.999189433	NA
ASV_16s_20397	0.140540272	-0.526479793	3.551124599	0.561722783	0.999187629	NA

ASV_16s_20398	0.140888529	-0.527333649	3.551122439	0.562484095	0.999184008	NA
ASV_16s_20399	0.133583498	-0.509096064	3.551168872	0.546816441	0.999256363	NA
ASV_16s_20400	0.142281556	-0.530729869	3.551113882	0.565545268	0.999169337	NA
ASV_16s_20401	0.127556106	-0.493399798	3.551209659	0.53443264	NA	NA
ASV_16s_20402	0.127556106	-0.493399798	3.551209659	0.53443264	NA	NA
ASV_16s_20403	0.127556106	-0.493399798	3.551209659	0.53443264	NA	NA
ASV_16s_20404	0.127556106	-0.493399798	3.551209659	0.53443264	NA	NA
ASV_16s_20405	0.127556106	-0.493399798	3.551209659	0.53443264	NA	NA
ASV_16s_20406	0.127556106	-0.493399798	3.551209659	0.53443264	NA	NA
ASV_16s_20407	0.127556106	-0.493399798	3.551209659	0.53443264	NA	NA
ASV_16s_20408	0.116100548	-0.46120546	3.551295286	0.504187315	0.999431004	NA
ASV_16s_20409	0.113681157	-0.454015881	3.551315102	0.496026099	0.999460902	NA
ASV_16s_20410	0.11363004	-0.453876655	3.551315488	0.497676342	0.999454945	NA
ASV_16s_20411	0.10846351	-0.43810611	3.551359881	0.486495537	NA	NA
ASV_16s_20412	0.10846351	-0.43810611	3.551359881	0.486495537	NA	NA
ASV_16s_20413	0.10846351	-0.43810611	3.551359881	0.486495537	NA	NA
ASV_16s_20414	0.10846351	-0.43810611	3.551359881	0.486495537	NA	NA
ASV_16s_20415	0.10846351	-0.43810611	3.551359881	0.486495537	NA	NA
ASV_16s_20416	0.10846351	-0.43810611	3.551359881	0.486495537	NA	NA
ASV_16s_20417	0.10846351	-0.43810611	3.551359881	0.486495537	NA	NA
ASV_16s_20418	0.10846351	-0.43810611	3.551359881	0.486495537	NA	NA
ASV_16s_20419	0.10846351	-0.43810611	3.551359881	0.486495537	NA	NA
ASV_16s_20420	0.10846351	-0.43810611	3.551359881	0.486495537	NA	NA
ASV_16s_20421	0.10846351	-0.43810611	3.551359881	0.486495537	NA	NA
ASV_16s_20422	0.10846351	-0.43810611	3.551359881	0.486495537	NA	NA
ASV_16s_20423	0.10846351	-0.43810611	3.551359881	0.486495537	NA	NA
ASV_16s_20424	0.10846351	-0.43810611	3.551359881	0.486495537	NA	NA
ASV_16s_20425	0.10846351	-0.43810611	3.551359881	0.486495537	NA	NA
ASV_16s_20426	0.10846351	-0.43810611	3.551359881	0.486495537	NA	NA
ASV_16s_20427	0.10846351	-0.43810611	3.551359881	0.486495537	NA	NA
ASV_16s_20428	0.106184577	-0.430917824	3.551380541	0.480032812	0.999516358	NA
ASV_16s_20429	0.104158859	-0.424411406	3.551399476	0.474236678	0.999535455	NA
ASV_16s_20430	0.102133141	-0.417789914	3.551418977	0.468400199	NA	NA
ASV_16s_20431	0.102133141	-0.417789914	3.551418977	0.468400199	NA	NA
ASV_16s_20432	0.102133141	-0.417789914	3.551418977	0.468400199	NA	NA
ASV_16s_20433	0.102133141	-0.417789914	3.551418977	0.468400199	NA	NA
ASV_16s_20434	0.102133141	-0.417789914	3.551418977	0.468400199	NA	NA
ASV_16s_20435	0.102133141	-0.417789914	3.551418977	0.468400199	NA	NA
ASV_16s_20436	0.102133141	-0.417789914	3.551418977	0.468400199	NA	NA
ASV_16s_20437	0.102133141	-0.417789914	3.551418977	0.468400199	NA	NA
ASV_16s_20438	0.02886346	-0.139363844	3.551268846	0.104607315	NA	NA
ASV_16s_20439	0.030843316	-0.071540302	3.551214051	0.15155109	0.999990292	NA
ASV_16s_20440	0.019606002	-0.099995972	3.551450587	0.041171489	0.999999894	NA
ASV_16s_20441	0.019606002	-0.099995972	3.551450587	0.041171489	0.999999894	NA
ASV_16s_20442	0.034310909	0.230658378	3.551306411	0.120499382	NA	NA
ASV_16s_20448	0.143500455	0.687438091	3.551016574	0.69016883	NA	NA
ASV_16s_20449	0.10480235	0.232448861	3.551791883	0.529198973	0.999332387	NA
ASV_16s_20450	0.144090672	-0.492929039	3.551246482	0.480921291	0.999513385	NA
ASV_16s_20451	0.106057215	0.312351883	3.551933435	0.389997867	0.999757973	NA
ASV_16s_20452	0.121896539	0.603846059	3.551841946	0.556368519	0.999212791	NA
ASV_16s_20453	0.133353757	-0.167464471	3.551254767	0.522321015	0.999360577	NA
ASV_16s_20454	0.125754733	-0.017433068	3.551668002	0.630340654	0.99881561	NA
ASV_16s_20455	0.110874054	0.029739867	3.551844061	0.469761797	0.999549842	NA
ASV_16s_20456	0.131478159	-0.464936474	3.551270934	0.541503683	0.999279875	NA
ASV_16s_20457	0.133284486	-0.126143553	3.551154607	0.534391381	0.999310556	NA
ASV_16s_20458	0.122006368	-0.629261671	3.551213258	0.398629323	0.999739569	NA
ASV_16s_20459	0.136762274	-0.446870177	3.551165086	0.59279148	0.999030831	NA
ASV_16s_20460	0.127630743	-0.407442845	3.551242817	0.564197828	0.999175817	NA
ASV_16s_20461	0.127776789	-0.407569582	3.551240818	0.564876658	0.999172557	NA
ASV_16s_20462	0.136687204	-0.581728516	3.551251302	0.46650326	0.999560125	NA
ASV_16s_20463	0.142773553	0.329367515	3.5519273	0.283787807	0.999917122	NA
ASV_16s_20464	0.133255067	-0.41012516	3.551156552	0.594978111	0.999019085	NA
ASV_16s_20465	0.108477707	0.783711838	3.551946111	0.403027501	0.999729832	NA
ASV_16s_20466	0.12338666	-0.373857273	3.551257856	0.564751143	0.99917316	NA
ASV_16s_20467	0.130185425	0.132090402	3.551543857	0.468267713	0.999554577	NA
ASV_16s_20468	0.132691945	-0.363059243	3.551162975	0.609841978	0.998936691	NA
ASV_16s_20469	0.133683014	-0.369367894	3.551118578	0.616721156	0.998897033	NA

ASV_16s_20470	0.132486806	-0.362458899	3.551129131	0.613022243	0.998918478	NA
ASV_16s_20471	0.135528159	-0.349846155	3.551104197	0.631485013	0.99880859	NA
ASV_16s_20472	0.130533385	-0.280838643	3.551495491	0.414622498	0.999702966	NA
ASV_16s_20473	0.131384254	0.939812566	3.551738819	0.451546317	0.999605272	NA
ASV_16s_20474	0.121997863	0.677973851	3.551512164	0.579389734	0.999100759	NA
ASV_16s_20475	0.132999493	-0.285194124	3.55113117	0.630622595	0.998813883	NA
ASV_16s_20476	0.124480453	-0.275384437	3.551358749	0.5008647	0.999443309	NA
ASV_16s_20477	0.142131981	-0.873316403	3.551185937	0.202462999	0.999973768	NA
ASV_16s_20478	0.111918992	0.12976676	3.55125703	0.525499789	0.999347651	NA
ASV_16s_20479	0.104212653	-0.541031465	3.551297985	0.375305376	0.999787209	NA
ASV_16s_20480	0.134336692	-0.167278072	3.551856956	0.398683837	0.99973945	NA
ASV_16s_20481	0.13444614	0.333262235	3.55185721	0.467697251	0.999556376	NA
ASV_16s_20482	0.127323021	0.672650618	3.551760086	0.480347617	0.999515306	NA
ASV_16s_20483	0.13350589	-0.16565937	3.551131452	0.645364879	0.99872122	NA
ASV_16s_20484	0.133688398	-0.165655677	3.551142765	0.644306315	0.99872803	NA
ASV_16s_20485	0.16427681	0.248389574	3.551189352	0.78512269	0.997592833	NA
ASV_16s_20486	0.140761619	-0.121476594	3.551829791	0.504454345	0.999430007	NA
ASV_16s_20487	0.172509347	-0.122696724	3.551694692	0.61197731	0.998924486	NA
ASV_16s_20488	0.149846306	0.251578108	3.551310605	0.654656794	0.998660394	NA
ASV_16s_20489	0.121587456	-0.110670209	3.551135725	0.61193056	0.998924754	NA
ASV_16s_20490	0.110105021	0.339201682	3.551789112	0.489614024	0.999483629	NA
ASV_16s_20491	0.136609896	0.371842837	3.551187005	0.646043314	0.998716843	NA
ASV_16s_20492	0.133403012	-0.056013479	3.551121945	0.651939019	0.998678381	NA
ASV_16s_20493	0.132461891	-0.056011756	3.551128252	0.648113526	0.998703424	NA
ASV_16s_20494	0.115543198	0.314811601	3.551231345	0.576825593	0.999113738	NA
ASV_16s_20495	0.117969463	-0.082142357	3.551210943	0.601804926	0.998981798	NA
ASV_16s_20496	0.109210143	0.286367075	3.551626055	0.523335432	0.999356471	NA
ASV_16s_20497	0.165617642	0.375017985	3.550998847	0.747852028	0.997940443	NA
ASV_16s_20498	0.128132551	0.136933065	3.55068655	0.485423157	0.999498125	NA
ASV_16s_20499	0.128132551	0.136933065	3.55068655	0.485423157	0.999498125	NA
ASV_16s_20500	0.115317428	0.528649434	3.551280923	0.234890555	0.999956428	NA
ASV_16s_20501	0.124628724	0.341005497	3.551726388	0.328720199	0.999863758	NA
ASV_16s_20502	0.124853837	0.086771339	3.550876502	0.452148633	0.999603518	NA
ASV_16s_20503	0.125330353	0.222703267	3.551422075	0.511816635	0.999402053	NA
ASV_16s_20504	0.133981894	1.011359902	3.551466859	0.496781427	NA	NA
ASV_16s_20505	0.164548102	0.301566317	3.551942632	0.435719474	0.99964948	NA
ASV_16s_20506	0.185658775	0.298698298	3.551883348	0.476917564	0.999526687	NA
ASV_16s_20507	0.134437735	-0.24589885	3.55115015	0.625319864	0.99884608	NA
ASV_16s_20508	0.11307139	1.020611736	3.551480675	0.448407388	NA	NA
ASV_16s_20509	0.11307139	1.020611736	3.551480675	0.448407388	NA	NA
ASV_16s_20510	0.11307139	1.020611736	3.551480675	0.448407388	NA	NA
ASV_16s_20511	0.114243458	1.024254051	3.551473305	0.451509907	0.999605377	NA
ASV_16s_20512	0.127230806	1.062117715	3.551397338	0.484632856	NA	NA
ASV_16s_20513	0.127230806	1.062117715	3.551397338	0.484632856	NA	NA
ASV_16s_20514	0.185019044	1.188458047	3.551075932	0.607059411	0.998952457	NA
ASV_16s_20515	0.129364592	0.173243364	3.551124778	0.803388017	0.997409224	NA
ASV_16s_20516	0.116734102	1.031838978	3.55145792	0.458012875	NA	NA
ASV_16s_20517	0.10375847	0.732609634	3.55178166	0.34259424	0.999843389	NA
ASV_16s_20518	0.110671951	0.33679133	3.551943749	0.476998532	NA	NA
ASV_16s_20519	0.110671951	0.33679133	3.551943749	0.476998532	NA	NA
ASV_16s_20520	0.110671951	0.33679133	3.551943749	0.476998532	NA	NA
ASV_16s_20521	0.110671951	0.33679133	3.551943749	0.476998532	NA	NA
ASV_16s_20522	0.126049242	-0.204493558	3.551710216	0.399001279	0.999738755	NA
ASV_16s_20523	0.133981894	0.344562264	3.551871852	0.53055467	NA	NA
ASV_16s_20524	0.133981894	0.344562264	3.551871852	0.53055467	NA	NA
ASV_16s_20525	0.14818972	0.349107876	3.551816527	0.562275226	NA	NA
ASV_16s_20526	0.132970564	0.096507954	3.551858241	0.466955793	0.999558706	NA
ASV_16s_20527	0.19411714	0.370654671	3.551553066	0.676646911	NA	NA
ASV_16s_20528	0.085764151	0.328273921	3.552005043	0.404021144	NA	NA
ASV_16s_20529	0.116126574	0.338628912	3.551929275	0.491685875	NA	NA
ASV_16s_20530	0.116126574	0.338628912	3.551929275	0.491685875	NA	NA
ASV_16s_20531	0.116126574	0.338628912	3.551929275	0.491685875	NA	NA
ASV_16s_20532	0.178214186	0.039646717	3.550959057	0.966250914	0.995354739	NA
ASV_16s_20533	0.123671493	-0.415177913	3.551290573	0.379124808	0.999779857	NA
ASV_16s_20534	0.129536993	-0.461962297	3.551294287	0.527660079	0.999338766	NA
ASV_16s_20535	0.148391493	0.156597213	3.551530949	0.449541943	NA	NA
ASV_16s_20536	0.171388228	-0.524744879	3.550907062	0.618638813	NA	NA

ASV_16s_20537	0.136884963	0.865046322	3.551224989	0.39294919	0.999751785	NA
ASV_16s_20538	0.12330081	-0.44760081	3.551344014	0.515760028	NA	NA
ASV_16s_20539	0.08565362	-0.339724371	3.551671504	0.411020536	NA	NA
ASV_16s_20540	0.112115151	-0.41957139	3.551441215	0.487585945	NA	NA
ASV_16s_20541	0.112115151	-0.41957139	3.551441215	0.487585945	NA	NA
ASV_16s_20542	0.235851303	1.257750005	3.550850765	0.730259068	NA	NA
ASV_16s_20543	0.12699234	1.100808257	3.551142713	0.425955884	NA	NA
ASV_16s_20544	0.12699234	1.100808257	3.551142713	0.425955884	NA	NA
ASV_16s_20545	0.149496968	1.154078719	3.551025242	0.471128498	0.999545481	NA
ASV_16s_20546	0.066277768	0.895672295	3.551622008	0.254595035	0.999942676	NA
ASV_16s_20547	0.113623443	1.06420467	3.551227074	0.392300345	NA	NA
ASV_16s_20548	0.113623443	1.06420467	3.551227074	0.392300345	NA	NA
ASV_16s_20549	0.113623443	1.06420467	3.551227074	0.392300345	NA	NA
ASV_16s_20550	0.113623443	1.06420467	3.551227074	0.392300345	NA	NA
ASV_16s_20551	0.113623443	1.06420467	3.551227074	0.392300345	NA	NA
ASV_16s_20552	0.14362299	0.366589071	3.55148918	0.487882797	NA	NA
ASV_16s_20553	0.13935588	0.364998726	3.55151359	0.478646803	0.999520973	NA
ASV_16s_20554	0.130288271	0.361000617	3.551561079	0.459739286	NA	NA
ASV_16s_20555	0.130288271	0.361000617	3.551561079	0.459739286	NA	NA
ASV_16s_20556	0.11829641	0.355274718	3.551612528	0.435245306	NA	NA
ASV_16s_20557	0.11829641	0.355274718	3.551612528	0.435245306	NA	NA
ASV_16s_20558	0.11829641	0.355274718	3.551612528	0.435245306	NA	NA
ASV_16s_20559	0.11829641	0.355274718	3.551612528	0.435245306	NA	NA
ASV_16s_20560	0.11829641	0.355274718	3.551612528	0.435245306	NA	NA
ASV_16s_20561	0.11829641	0.355274718	3.551612528	0.435245306	NA	NA
ASV_16s_20562	0.09765229	0.346543999	3.551701438	0.379870463	NA	NA
ASV_16s_20563	0.09765229	0.346543999	3.551701438	0.379870463	NA	NA
ASV_16s_20564	0.105656316	0.348628182	3.55167366	0.402176752	NA	NA
ASV_16s_20565	0.105656316	0.348628182	3.55167366	0.402176752	NA	NA
ASV_16s_20566	0.105656316	0.348628182	3.55167366	0.402176752	NA	NA
ASV_16s_20567	0.036788629	0.338633959	3.551927905	0.168622443	0.999985987	NA
ASV_16s_20568	0.036788629	0.338633959	3.551927905	0.168622443	0.999985987	NA
ASV_16s_20569	0.036788629	0.338633959	3.551927905	0.168622443	0.999985987	NA
ASV_16s_20570	0.059362124	-0.259234926	3.551550445	0.258762689	0.999939421	NA
ASV_16s_20571	0.14562034	-0.501024804	3.550813991	0.493431203	NA	NA
ASV_16s_20572	0.14562034	-0.501024804	3.550813991	0.493431203	NA	NA
ASV_16s_20573	0.187841296	-0.536059156	3.550431293	0.589949351	NA	NA
ASV_16s_20574	0.193881324	-0.538476524	3.550382306	0.604961377	NA	NA
ASV_16s_20575	0.17935693	-0.532397053	3.550504982	0.569384044	NA	NA
ASV_16s_20576	0.17935693	-0.532397053	3.550504982	0.569384044	NA	NA
ASV_16s_20577	0.097792172	-0.391839776	3.551297497	0.361336697	0.999812655	NA
ASV_16s_20578	0.066072543	-0.289571023	3.551504214	0.283096082	NA	NA
ASV_16s_20579	0.052378146	0.830629855	3.551751251	0.207344832	0.99997154	NA
ASV_16s_20580	0.12959296	1.107515388	3.551127938	0.432323407	NA	NA
ASV_16s_20581	0.11829641	-0.078082167	3.550976003	0.688843798	NA	NA
ASV_16s_20582	0.11829641	-0.078082167	3.550976003	0.688843798	NA	NA
ASV_16s_20583	0.11829641	-0.078082167	3.550976003	0.688843798	NA	NA
ASV_16s_20584	0.11829641	-0.078082167	3.550976003	0.688843798	NA	NA
ASV_16s_20585	0.11829641	-0.078082167	3.550976003	0.688843798	NA	NA
ASV_16s_20586	0.11829641	-0.078082167	3.550976003	0.688843798	NA	NA
ASV_16s_20587	0.11829641	-0.078082167	3.550976003	0.688843798	NA	NA
ASV_16s_20588	0.11829641	-0.078082167	3.550976003	0.688843798	NA	NA
ASV_16s_20589	0.11829641	-0.078082167	3.550976003	0.688843798	NA	NA
ASV_16s_20590	0.100872694	-0.077817034	3.551055072	0.620708142	NA	NA
ASV_16s_20591	0.100872694	-0.077817034	3.551055072	0.620708142	NA	NA
ASV_16s_20592	0.100872694	-0.077817034	3.551055072	0.620708142	NA	NA
ASV_16s_20593	0.100872694	-0.077817034	3.551055072	0.620708142	NA	NA
ASV_16s_20594	0.100872694	-0.077817034	3.551055072	0.620708142	NA	NA
ASV_16s_20595	0.100872694	-0.077817034	3.551055072	0.620708142	NA	NA
ASV_16s_20596	0.100872694	-0.077817034	3.551055072	0.620708142	NA	NA
ASV_16s_20597	0.100872694	-0.077817034	3.551055072	0.620708142	NA	NA
ASV_16s_20598	0.100872694	-0.077817034	3.551055072	0.620708142	NA	NA
ASV_16s_20599	0.075096233	0.220224315	3.550961732	0.347585407	NA	NA
ASV_16s_20600	0.075096233	0.220224315	3.550961732	0.347585407	NA	NA
ASV_16s_20601	0.075096233	0.220224315	3.550961732	0.347585407	NA	NA
ASV_16s_20602	0.074946271	0.220224895	3.550962498	0.346866244	NA	NA
ASV_16s_20603	0.128169116	0.310954091	3.551097746	0.700566328	NA	NA

ASV_16s_20604	0.128169116	0.310954091	3.551097746	0.700566328	NA	NA
ASV_16s_20605	0.128169116	0.310954091	3.551097746	0.700566328	NA	NA
ASV_16s_20606	0.130332628	0.310953547	3.551089346	0.707248654	NA	NA
ASV_16s_20607	0.127360632	0.310954204	3.55110096	0.696857209	0.998359768	NA
ASV_16s_20608	0.119930642	0.310956236	3.551132488	0.671723171	0.998543694	NA
ASV_16s_20609	0.122001351	0.310955645	3.551123317	0.679053716	0.998491552	NA
ASV_16s_20610	0.127763431	0.310954195	3.551099352	0.699317993	NA	NA
ASV_16s_20611	0.127763431	0.310954195	3.551099352	0.699317993	NA	NA
ASV_16s_20612	0.079107955	0.595572138	3.550896829	0.315278408	NA	NA
ASV_16s_20613	0.079107955	0.595572138	3.550896829	0.315278408	NA	NA
ASV_16s_20614	0.12488261	1.127028533	3.551521357	0.348654668	0.999833866	NA
ASV_16s_20615	0.130116213	0.712088875	3.551318591	0.504740396	0.999428938	NA
ASV_16s_20616	0.103845804	-0.065116802	3.551148168	0.646561675	0.998713491	NA
ASV_16s_20617	0.106818914	-0.057042763	3.551310071	0.685040847	0.998448054	NA
ASV_16s_20618	0.106818914	-0.057042763	3.551310071	0.685040847	0.998448054	NA
ASV_16s_20619	0.106818914	-0.057042763	3.551310071	0.685040847	0.998448054	NA
ASV_16s_20620	0.113524935	-0.058902057	3.551247917	0.713677548	0.998228423	NA
ASV_16s_20621	0.142831905	0.714835061	3.551205424	0.794175717	0.997502939	NA
ASV_16s_20622	0.107809951	-0.048335865	3.551388687	0.687042915	0.998433324	NA
ASV_16s_20623	0.113502911	-0.054388311	3.551286175	0.711793807	0.998243468	NA
ASV_16s_20624	0.146020133	1.175344696	3.551348406	0.455566785	0.999593463	NA
ASV_16s_20625	0.112415624	-0.016179504	3.551295569	0.738191962	0.99802477	NA
ASV_16s_20626	0.108305469	-0.040540512	3.551438532	0.686038312	0.998440727	NA
ASV_16s_20627	0.115073525	-0.045225583	3.551373661	0.711024591	0.998249587	NA
ASV_16s_20628	0.108800987	-0.02796613	3.551486824	0.683756835	0.998457452	NA
ASV_16s_20629	0.149064423	0.748162243	3.551446209	0.66913584	0.998561806	NA
ASV_16s_20630	0.144175822	-0.499347722	3.551035	0.420776515	0.999687988	NA
ASV_16s_20631	0.113436841	-0.028555705	3.551452379	0.697418167	0.998355496	NA
ASV_16s_20632	0.102411318	0.954481294	3.551772877	0.37485118	0.999788071	NA
ASV_16s_20633	0.134143233	0.007863479	3.551540843	0.617411128	0.998893001	NA
ASV_16s_20634	0.109792024	0.010854909	3.55153764	0.675986172	0.998513521	NA
ASV_16s_20635	0.113414817	-0.010314244	3.551503044	0.690366534	0.998408666	NA
ASV_16s_20636	0.120703654	0.319958766	3.551506711	0.528128664	0.999336828	NA
ASV_16s_20637	0.173066945	-0.627207077	3.55127848	0.412628487	0.999707711	NA
ASV_16s_20638	0.111774097	0.208160442	3.551523354	0.668827072	NA	NA
ASV_16s_20639	0.267970681	1.118527573	3.550825393	0.788465414	NA	NA
ASV_16s_20640	0.182961433	1.019033013	3.551198631	0.579176537	0.999101843	NA
ASV_16s_20641	0.1602923	0.980275875	3.551336059	0.52849883	0.999335295	NA
ASV_16s_20642	0.129316525	0.922921412	3.551491762	0.469081679	NA	NA
ASV_16s_20643	0.085041555	0.286063027	3.551792949	0.40493347	0.999725535	NA
ASV_16s_20644	0.085041555	0.286063027	3.551792949	0.40493347	0.999725535	NA
ASV_16s_20645	0.050500534	0.29361995	3.551905284	0.275091084	0.999925426	NA
ASV_16s_20646	0.050500534	0.29361995	3.551905284	0.275091084	0.999925426	NA
ASV_16s_20647	0.08594544	-0.305132624	3.551612331	0.408372764	0.999717664	NA
ASV_16s_20648	0.267291056	1.117960968	3.550827929	0.786660503	NA	NA
ASV_16s_20649	0.203948073	0.394611617	3.551114961	0.636594251	NA	NA
ASV_16s_20650	0.203948073	0.394611617	3.551114961	0.636594251	NA	NA
ASV_16s_20651	0.145777055	0.357735352	3.551735549	0.482677317	0.99950747	NA
ASV_16s_20652	0.133981894	-0.483449709	3.550932577	0.469891608	NA	NA
ASV_16s_20653	0.148560597	1.152126742	3.551029988	0.470190378	NA	NA
ASV_16s_20654	0.148560597	1.152126742	3.551029988	0.470190378	NA	NA
ASV_16s_20655	0.148560597	1.152126742	3.551029988	0.470190378	NA	NA
ASV_16s_20656	0.148560597	1.152126742	3.551029988	0.470190378	NA	NA
ASV_16s_20657	0.08465467	-0.077473746	3.551157615	0.550441599	NA	NA
ASV_16s_20658	0.08465467	-0.077473746	3.551157615	0.550441599	NA	NA
ASV_16s_20659	0.08465467	-0.077473746	3.551157615	0.550441599	NA	NA
ASV_16s_20660	0.234262581	-0.028942992	3.550866511	1.131838597	NA	NA
ASV_16s_20661	0.175156646	0.435876848	3.551043259	1.208151618	0.990737996	NA
ASV_16s_20662	0.127780884	0.260441572	3.551249469	0.947551131	0.995630826	NA
ASV_16s_20663	0.161350981	0.543527598	3.551128807	1.032046672	0.994294881	NA
ASV_16s_20664	0.186862659	-0.028679676	3.550954039	0.968374256	NA	NA
ASV_16s_20665	0.160877289	0.360355637	3.550771733	0.907954344	0.99617993	NA
ASV_16s_20666	0.111689971	-0.026021119	3.551239935	0.722598732	NA	NA
ASV_16s_20667	0.111689971	-0.026021119	3.551239935	0.722598732	NA	NA
ASV_16s_20668	0.111689971	-0.026021119	3.551239935	0.722598732	NA	NA
ASV_16s_20669	0.111689971	-0.026021119	3.551239935	0.722598732	NA	NA
ASV_16s_20670	0.111689971	-0.026021119	3.551239935	0.722598732	NA	NA

ASV_16s_20671	0.111689971	-0.026021119	3.551239935	0.722598732	NA	NA
ASV_16s_20672	0.127620006	-0.073032744	3.550985081	0.743386187	NA	NA
ASV_16s_20673	0.127620006	-0.073032744	3.550985081	0.743386187	NA	NA
ASV_16s_20674	0.142633242	0.324724732	3.550926432	0.828433034	0.997142841	NA
ASV_16s_20675	0.143788107	0.336999241	3.550944255	0.827271639	0.997155574	NA
ASV_16s_20676	0.093208498	-0.072427	3.551157373	0.612583757	NA	NA
ASV_16s_20677	0.093208498	-0.072427	3.551157373	0.612583757	NA	NA
ASV_16s_20678	0.093208498	-0.072427	3.551157373	0.612583757	NA	NA
ASV_16s_20679	0.093208498	-0.072427	3.551157373	0.612583757	NA	NA
ASV_16s_20680	0.108396446	0.217372703	3.551147922	0.678965589	0.998492186	NA
ASV_16s_20681	0.091533697	-0.072386084	3.551169029	0.60531011	NA	NA
ASV_16s_20682	0.091533697	-0.072386084	3.551169029	0.60531011	NA	NA
ASV_16s_20683	0.091533697	-0.072386084	3.551169029	0.60531011	NA	NA
ASV_16s_20684	0.091533697	-0.072386084	3.551169029	0.60531011	NA	NA
ASV_16s_20685	0.091533697	-0.072386084	3.551169029	0.60531011	NA	NA
ASV_16s_20686	0.091533697	-0.072386084	3.551169029	0.60531011	NA	NA
ASV_16s_20687	0.091533697	-0.072386084	3.551169029	0.60531011	NA	NA
ASV_16s_20688	0.091533697	-0.072386084	3.551169029	0.60531011	NA	NA
ASV_16s_20689	0.091533697	-0.072386084	3.551169029	0.60531011	NA	NA
ASV_16s_20690	0.124023823	-0.061226149	3.5511958	0.792449705	0.997520245	NA
ASV_16s_20691	0.142314563	0.550886515	3.550904721	0.655897957	0.998652126	NA
ASV_16s_20692	0.140901714	0.449325725	3.551285954	0.756145546	0.997866174	NA
ASV_16s_20693	0.11357142	-0.072829181	3.551042918	0.69516252	NA	NA
ASV_16s_20694	0.11357142	-0.072829181	3.551042918	0.69516252	NA	NA
ASV_16s_20695	0.12740726	0.360232302	3.551387769	0.759825208	NA	NA
ASV_16s_20696	0.12740726	0.360232302	3.551387769	0.759825208	NA	NA
ASV_16s_20697	0.12740726	0.360232302	3.551387769	0.759825208	NA	NA
ASV_16s_20698	0.12740726	0.360232302	3.551387769	0.759825208	NA	NA
ASV_16s_20699	0.113260652	0.360202548	3.55148557	0.71224815	NA	NA
ASV_16s_20700	0.113260652	0.360202548	3.55148557	0.71224815	NA	NA
ASV_16s_20701	0.113260652	0.360202548	3.55148557	0.71224815	NA	NA
ASV_16s_20702	0.113260652	0.360202548	3.55148557	0.71224815	NA	NA
ASV_16s_20703	0.113260652	0.360202548	3.55148557	0.71224815	NA	NA
ASV_16s_20704	0.113260652	0.360202548	3.55148557	0.71224815	NA	NA
ASV_16s_20705	0.113260652	0.360202548	3.55148557	0.71224815	NA	NA
ASV_16s_20706	0.113260652	0.360202548	3.55148557	0.71224815	NA	NA
ASV_16s_20707	0.113260652	0.360202548	3.55148557	0.71224815	NA	NA
ASV_16s_20708	0.113260652	0.360202548	3.55148557	0.71224815	NA	NA
ASV_16s_20709	0.113260652	0.360202548	3.55148557	0.71224815	NA	NA
ASV_16s_20710	0.113260652	0.360202548	3.55148557	0.71224815	NA	NA
ASV_16s_20711	0.113260652	0.360202548	3.55148557	0.71224815	NA	NA
ASV_16s_20712	0.113260652	0.360202548	3.55148557	0.71224815	NA	NA
ASV_16s_20713	0.113260652	0.360202548	3.55148557	0.71224815	NA	NA
ASV_16s_20714	0.146751213	0.315939988	3.551075532	0.781999972	NA	NA
ASV_16s_20715	0.146751213	0.315939988	3.551075532	0.781999972	NA	NA
ASV_16s_20716	0.146751213	0.315939988	3.551075532	0.781999972	NA	NA
ASV_16s_20717	0.146751213	0.315939988	3.551075532	0.781999972	NA	NA
ASV_16s_20718	0.129678331	0.315940088	3.551139279	0.727383308	0.998116375	NA
ASV_16s_20719	0.1268513	0.315940107	3.551151452	0.719300291	NA	NA
ASV_16s_20720	0.1268513	0.315940107	3.551151452	0.719300291	NA	NA
ASV_16s_20721	0.152081891	0.31593987	3.551058613	0.79909519	0.997453176	NA
ASV_16s_20722	0.122744953	0.315940137	3.551170156	0.706110145	0.998288348	NA
ASV_16s_20723	0.121033975	0.31594015	3.55117832	0.700745498	0.998330002	NA
ASV_16s_20724	0.143461167	0.315939907	3.551086741	0.770926459	0.997729461	NA
ASV_16s_20725	0.11829641	0.315940171	3.551191865	0.692270415	NA	NA
ASV_16s_20726	0.11829641	0.315940171	3.551191865	0.692270415	NA	NA
ASV_16s_20727	0.11829641	0.315940171	3.551191865	0.692270415	NA	NA
ASV_16s_20728	0.11829641	0.315940171	3.551191865	0.692270415	NA	NA
ASV_16s_20729	0.11829641	0.315940171	3.551191865	0.692270415	NA	NA
ASV_16s_20730	0.11829641	0.315940171	3.551191865	0.692270415	NA	NA
ASV_16s_20731	0.11829641	0.315940171	3.551191865	0.692270415	NA	NA
ASV_16s_20732	0.11829641	0.315940171	3.551191865	0.692270415	NA	NA
ASV_16s_20733	0.11829641	0.315940171	3.551191865	0.692270415	NA	NA
ASV_16s_20734	0.11829641	0.315940171	3.551191865	0.692270415	NA	NA
ASV_16s_20735	0.14761616	0.719501554	3.551222234	0.843727377	0.996971671	NA
ASV_16s_20736	0.11829641	0.481199717	3.551126732	0.908728317	0.996169651	NA
ASV_16s_20737	0.159280331	0.596017078	3.550722697	0.591455151	NA	NA

ASV_16s_20738	0.203524651	0.687861523	3.550880954	0.866624607	NA	NA
ASV_16s_20739	0.203524651	0.687861523	3.550880954	0.866624607	NA	NA
ASV_16s_20740	0.188474079	0.997985711	3.550933026	0.653658592	0.998667019	NA
ASV_16s_20741	0.194600277	1.091168412	3.55090698	0.649401289	0.998695029	NA
ASV_16s_20742	0.169660864	1.08681568	3.551264874	0.612123552	0.998923646	NA
ASV_16s_20743	0.185895512	1.086269756	3.551042063	0.652105708	0.998677282	NA
ASV_16s_20744	0.182784538	0.733583106	3.551163206	0.868681338	0.996678239	NA
ASV_16s_20745	0.145149748	0.801285116	3.55113456	0.620523423	0.998874692	NA
ASV_16s_20746	0.184932206	0.733606675	3.551156349	0.875368999	NA	NA
ASV_16s_20747	0.184932206	0.733606675	3.551156349	0.875368999	NA	NA
ASV_16s_20748	0.170143098	0.733434906	3.551206338	0.831426914	0.997109847	NA
ASV_16s_20749	0.169005474	0.733420471	3.55121054	0.828092537	0.997146578	NA
ASV_16s_20750	0.165592603	0.733375998	3.55122349	0.818131631	0.997254481	NA
ASV_16s_20751	0.156491614	0.733248108	3.551260742	0.791896294	NA	NA
ASV_16s_20752	0.156491614	0.733248108	3.551260742	0.791896294	NA	NA
ASV_16s_20753	0.156491614	0.733248108	3.551260742	0.791896294	NA	NA
ASV_16s_20754	0.156491614	0.733248108	3.551260742	0.791896294	NA	NA
ASV_16s_20755	0.156491614	0.733248108	3.551260742	0.791896294	NA	NA
ASV_16s_20756	0.156491614	0.733248108	3.551260742	0.791896294	NA	NA
ASV_16s_20757	0.156491614	0.733248108	3.551260742	0.791896294	NA	NA
ASV_16s_20758	0.156491614	0.733248108	3.551260742	0.791896294	NA	NA
ASV_16s_20759	0.156491614	0.733248108	3.551260742	0.791896294	NA	NA
ASV_16s_20760	0.156491614	0.733248108	3.551260742	0.791896294	NA	NA
ASV_16s_20761	0.156491614	0.733248108	3.551260742	0.791896294	NA	NA
ASV_16s_20762	0.156491614	0.733248108	3.551260742	0.791896294	NA	NA
ASV_16s_20763	0.156491614	0.733248108	3.551260742	0.791896294	NA	NA
ASV_16s_20764	0.233958909	0.734022549	3.551035522	1.026848548	NA	NA
ASV_16s_20765	0.077519007	0.679311099	3.551673351	0.511409558	NA	NA
ASV_16s_20766	0.077519007	0.679311099	3.551673351	0.511409558	NA	NA
ASV_16s_20767	0.077519007	0.679311099	3.551673351	0.511409558	NA	NA
ASV_16s_20768	0.077519007	0.679311099	3.551673351	0.511409558	NA	NA
ASV_16s_20769	0.077519007	0.679311099	3.551673351	0.511409558	NA	NA
ASV_16s_20770	0.1454865	0.692444334	3.551060797	0.72029063	NA	NA
ASV_16s_20771	0.1454865	0.692444334	3.551060797	0.72029063	NA	NA
ASV_16s_20772	0.1454865	0.692444334	3.551060797	0.72029063	NA	NA
ASV_16s_20773	0.134610464	0.692306133	3.551101532	0.68553079	0.998444458	NA
ASV_16s_20774	0.147302863	0.692465443	3.551054578	0.725361639	0.99813319	NA
ASV_16s_20775	0.148128483	0.69247487	3.551051801	0.727674429	0.998113946	NA
ASV_16s_20776	0.138271787	0.69235324	3.551087114	0.696585154	0.998361838	NA
ASV_16s_20777	0.148623855	0.692480476	3.551050149	0.729064407	0.998102316	NA
ASV_16s_20778	0.149614598	0.69249158	3.551046879	0.731849493	NA	NA
ASV_16s_20779	0.149614598	0.69249158	3.551046879	0.731849493	NA	NA
ASV_16s_20780	0.149614598	0.69249158	3.551046879	0.731849493	NA	NA
ASV_16s_20781	0.149614598	0.69249158	3.551046879	0.731849493	NA	NA
ASV_16s_20782	0.125812775	0.692177164	3.551139575	0.658130636	0.998637166	NA
ASV_16s_20783	0.11829641	0.692052098	3.551176489	0.635666605	NA	NA
ASV_16s_20784	0.11829641	0.692052098	3.551176489	0.635666605	NA	NA
ASV_16s_20785	0.11829641	0.692052098	3.551176489	0.635666605	NA	NA
ASV_16s_20786	0.11829641	0.692052098	3.551176489	0.635666605	NA	NA
ASV_16s_20787	0.11829641	0.692052098	3.551176489	0.635666605	NA	NA
ASV_16s_20788	0.11829641	0.692052098	3.551176489	0.635666605	NA	NA
ASV_16s_20789	0.11829641	0.692052098	3.551176489	0.635666605	NA	NA
ASV_16s_20790	0.11829641	0.692052098	3.551176489	0.635666605	NA	NA
ASV_16s_20791	0.11829641	0.692052098	3.551176489	0.635666605	NA	NA
ASV_16s_20792	0.11829641	0.692052098	3.551176489	0.635666605	NA	NA
ASV_16s_20793	0.11829641	0.692052098	3.551176489	0.635666605	NA	NA
ASV_16s_20794	0.11829641	0.692052098	3.551176489	0.635666605	NA	NA
ASV_16s_20795	0.11829641	0.692052098	3.551176489	0.635666605	NA	NA
ASV_16s_20796	0.136052336	0.692325549	3.551095807	0.687978955	0.998426405	NA
ASV_16s_20797	0.140365532	0.692381713	3.55107925	0.700955406	0.998328385	NA
ASV_16s_20798	0.160366106	0.692602135	3.551013997	0.761526732	0.99781705	NA
ASV_16s_20799	0.173783678	0.692723709	3.550978568	0.801599645	NA	NA
ASV_16s_20800	0.173783678	0.692723709	3.550978568	0.801599645	NA	NA
ASV_16s_20801	0.172739507	1.148714333	3.551308925	0.447072857	0.999618132	NA
ASV_16s_20802	0.16522486	0.291736712	3.551727214	0.605092857	NA	NA
ASV_16s_20803	0.097910918	0.298961729	3.55193236	0.440514457	NA	NA
ASV_16s_20804	0.097910918	0.298961729	3.55193236	0.440514457	NA	NA

ASV_16s_20805	0.097910918	0.298961729	3.55193236	0.440514457	NA	NA
ASV_16s_20806	0.097910918	0.298961729	3.55193236	0.440514457	NA	NA
ASV_16s_20807	0.097910918	0.298961729	3.55193236	0.440514457	NA	NA
ASV_16s_20808	0.097910918	0.298961729	3.55193236	0.440514457	NA	NA
ASV_16s_20809	0.097910918	0.298961729	3.55193236	0.440514457	NA	NA
ASV_16s_20810	0.097910918	0.298961729	3.55193236	0.440514457	NA	NA
ASV_16s_20811	0.097910918	0.298961729	3.55193236	0.440514457	NA	NA
ASV_16s_20812	0.097910918	0.298961729	3.55193236	0.440514457	NA	NA
ASV_16s_20813	0.097910918	0.298961729	3.55193236	0.440514457	NA	NA
ASV_16s_20814	0.097910918	0.298961729	3.55193236	0.440514457	NA	NA
ASV_16s_20815	0.097910918	0.298961729	3.55193236	0.440514457	NA	NA
ASV_16s_20816	0.097910918	0.298961729	3.55193236	0.440514457	NA	NA
ASV_16s_20817	0.097910918	0.298961729	3.55193236	0.440514457	NA	NA
ASV_16s_20818	0.097910918	0.298961729	3.55193236	0.440514457	NA	NA
ASV_16s_20819	0.097910918	0.298961729	3.55193236	0.440514457	NA	NA
ASV_16s_20820	0.097910918	0.298961729	3.55193236	0.440514457	NA	NA
ASV_16s_20821	0.097939438	0.105940349	3.55178856	0.560018666	0.999195696	NA
ASV_16s_20822	0.099464431	0.063805997	3.551748461	0.582763391	0.999083487	NA
ASV_16s_20823	0.097945142	0.074308753	3.551765289	0.575312349	0.999121338	NA
ASV_16s_20824	0.097950846	0.0433485	3.551737181	0.588311966	0.999054598	NA
ASV_16s_20825	0.099982269	9.36E-05	3.55167946	0.608814523	0.998942531	NA
ASV_16s_20826	0.097962254	-0.016099015	3.551676945	0.608000254	0.998947144	NA
ASV_16s_20827	0.097962254	-0.016099015	3.551676945	0.608000254	0.998947144	NA
ASV_16s_20828	0.097962254	-0.016099015	3.551676945	0.608000254	0.998947144	NA
ASV_16s_20829	0.100241187	-0.029710231	3.551650045	0.618425086	0.998887058	NA
ASV_16s_20830	0.100241187	-0.029710231	3.551650045	0.618425086	0.998887058	NA
ASV_16s_20831	0.100500106	-0.05880951	3.551619809	0.625572662	0.998844558	NA
ASV_16s_20832	0.100500106	-0.05880951	3.551619809	0.625572662	0.998844558	NA
ASV_16s_20833	0.100500106	-0.05880951	3.551619809	0.625572662	0.998844558	NA
ASV_16s_20834	0.100500106	-0.05880951	3.551619809	0.625572662	0.998844558	NA
ASV_16s_20835	0.097967958	-0.044150402	3.551651989	0.614343922	0.998910848	NA
ASV_16s_20836	0.097967958	-0.044150402	3.551651989	0.614343922	0.998910848	NA
ASV_16s_20837	0.100759025	-0.086958582	3.551592902	0.630346481	0.998815574	NA
ASV_16s_20838	0.100759025	-0.086958582	3.551592902	0.630346481	0.998815574	NA
ASV_16s_20839	0.100759025	-0.086958582	3.551592902	0.630346481	0.998815574	NA
ASV_16s_20840	0.106410923	-0.115948371	3.551531279	0.655338318	0.998655858	NA
ASV_16s_20841	0.100759025	-0.086958582	3.551592902	0.630346481	0.998815574	NA
ASV_16s_20842	0.101017944	-0.114174386	3.55156854	0.632713371	0.998801025	NA
ASV_16s_20843	0.10652809	-0.141311004	3.551510059	0.6561272	0.998650595	NA
ASV_16s_20844	0.107735025	-0.171317949	3.551478897	0.659247672	0.998629641	NA
ASV_16s_20845	0.101276863	-0.140647618	3.551546835	0.632664072	0.998801329	NA
ASV_16s_20846	0.101276863	-0.140647618	3.551546835	0.632664072	0.998801329	NA
ASV_16s_20847	0.097990775	-0.149684789	3.551562211	0.616748482	0.998896873	NA
ASV_16s_20848	0.101535782	-0.166567555	3.551526763	0.630194367	0.998816505	NA
ASV_16s_20849	0.116406619	-0.254278	3.551360548	0.681751399	0.998472055	NA
ASV_16s_20850	0.107746433	-0.218315068	3.551447705	0.64767064	0.998706302	NA
ASV_16s_20851	0.097996479	-0.174682696	3.551545873	0.611535322	0.99892702	NA
ASV_16s_20852	0.1017947	-0.192201194	3.551506626	0.625298749	0.998846207	NA
ASV_16s_20853	0.1017947	-0.192201194	3.551506626	0.625298749	0.998846207	NA
ASV_16s_20854	0.100534331	-0.210865715	3.551503957	0.612565136	0.998921109	NA
ASV_16s_20855	0.112645598	-0.28455854	3.551368934	0.643615088	0.998732463	NA
ASV_16s_20856	0.098007887	-0.224440948	3.55151411	0.593998381	0.99902436	NA
ASV_16s_20857	0.102571457	-0.268159198	3.551454297	0.596166298	0.999012663	NA
ASV_16s_20858	0.098013591	-0.249325189	3.551498981	0.582161192	0.999086587	NA
ASV_16s_20859	0.111498161	-0.349042629	3.551349764	0.589598031	0.999047815	NA
ASV_16s_20860	0.098030703	-0.324555694	3.551468157	0.53416381	0.999311523	NA
ASV_16s_20861	0.035001974	-0.093274796	3.551885091	0.217120445	0.999966687	NA
ASV_16s_20862	0.035001974	-0.093274796	3.551885091	0.217120445	0.999966687	NA
ASV_16s_20863	0.035001974	-0.093274796	3.551885091	0.217120445	0.999966687	NA
ASV_16s_20864	0.166098703	-0.578899217	3.550959907	0.623921317	NA	NA
ASV_16s_20865	0.134692112	-0.511803286	3.551161937	0.544871544	0.999265029	NA
ASV_16s_20866	0.133581355	-0.509008003	3.551169098	0.54230013	NA	NA
ASV_16s_20867	0.133581355	-0.509008003	3.551169098	0.54230013	NA	NA
ASV_16s_20868	0.134800254	-0.512098872	3.551161183	0.545154537	0.999263773	NA
ASV_16s_20869	0.127090317	-0.492053861	3.551213138	0.527440802	0.999339672	NA
ASV_16s_20870	0.136019153	-0.515163509	3.55115338	0.548030074	0.99925092	NA
ASV_16s_20871	0.126163026	-0.489541953	3.551219657	0.52536097	0.999348219	NA

ASV_16s_20872	0.13207712	-0.505152583	3.551179034	0.5387994	0.999291647	NA
ASV_16s_20873	0.137760437	-0.519505655	3.551142363	0.552171121	NA	NA
ASV_16s_20874	0.137760437	-0.519505655	3.551142363	0.552171121	NA	NA
ASV_16s_20875	0.137760437	-0.519505655	3.551142363	0.552171121	NA	NA
ASV_16s_20876	0.137760437	-0.519505655	3.551142363	0.552171121	NA	NA
ASV_16s_20877	0.137760437	-0.519505655	3.551142363	0.552171121	NA	NA
ASV_16s_20878	0.137760437	-0.519505655	3.551142363	0.552171121	NA	NA
ASV_16s_20879	0.130744923	-0.501706724	3.551187975	0.535729644	0.999304852	NA
ASV_16s_20880	0.122453861	-0.479324023	3.551246482	0.51718103	NA	NA
ASV_16s_20881	0.122453861	-0.479324023	3.551246482	0.51718103	NA	NA
ASV_16s_20882	0.122453861	-0.479324023	3.551246482	0.51718103	NA	NA
ASV_16s_20883	0.122453861	-0.479324023	3.551246482	0.51718103	NA	NA
ASV_16s_20884	0.135124149	-0.512915223	3.5511591	0.545917164	NA	NA
ASV_16s_20885	0.135124149	-0.512915223	3.5511591	0.545917164	NA	NA
ASV_16s_20886	0.135124149	-0.512915223	3.5511591	0.545917164	NA	NA
ASV_16s_20887	0.135124149	-0.512915223	3.5511591	0.545917164	NA	NA
ASV_16s_20888	0.10412497	-0.424223995	3.551400024	0.474038748	NA	NA
ASV_16s_20889	0.10412497	-0.424223995	3.551400024	0.474038748	NA	NA
ASV_16s_20890	0.10412497	-0.424223995	3.551400024	0.474038748	NA	NA
ASV_16s_20891	0.10412497	-0.424223995	3.551400024	0.474038748	NA	NA
ASV_16s_20892	0.10412497	-0.424223995	3.551400024	0.474038748	NA	NA
ASV_16s_20893	0.10412497	-0.424223995	3.551400024	0.474038748	NA	NA
ASV_16s_20894	0.10412497	-0.424223995	3.551400024	0.474038748	NA	NA
ASV_16s_20895	0.10412497	-0.424223995	3.551400024	0.474038748	NA	NA
ASV_16s_20896	0.10412497	-0.424223995	3.551400024	0.474038748	NA	NA
ASV_16s_20897	0.10412497	-0.424223995	3.551400024	0.474038748	NA	NA
ASV_16s_20898	0.10412497	-0.424223995	3.551400024	0.474038748	NA	NA
ASV_16s_20899	0.10412497	-0.424223995	3.551400024	0.474038748	NA	NA
ASV_16s_20900	0.10412497	-0.424223995	3.551400024	0.474038748	NA	NA
ASV_16s_20901	0.10412497	-0.424223995	3.551400024	0.474038748	NA	NA
ASV_16s_20902	0.10412497	-0.424223995	3.551400024	0.474038748	NA	NA
ASV_16s_20903	0.10412497	-0.424223995	3.551400024	0.474038748	NA	NA
ASV_16s_20904	0.101846037	-0.416762589	3.551422025	0.467463398	0.999557112	NA
ASV_16s_20905	0.109128686	-0.440040223	3.551354367	0.482432949	0.999508296	NA
ASV_16s_20906	0.100073534	-0.410912528	3.55143881	0.462285622	0.999573194	NA
ASV_16s_20907	0.098047815	-0.404217604	3.551457005	0.456303718	NA	NA
ASV_16s_20908	0.098047815	-0.404217604	3.551457005	0.456303718	NA	NA
ASV_16s_20909	0.098047815	-0.404217604	3.551457005	0.456303718	NA	NA
ASV_16s_20910	0.098047815	-0.404217604	3.551457005	0.456303718	NA	NA
ASV_16s_20911	0.098047815	-0.404217604	3.551457005	0.456303718	NA	NA
ASV_16s_20912	0.098047815	-0.404217604	3.551457005	0.456303718	NA	NA
ASV_16s_20913	0.098047815	-0.404217604	3.551457005	0.456303718	NA	NA
ASV_16s_20914	0.098047815	-0.404217604	3.551457005	0.456303718	NA	NA
ASV_16s_20915	0.098047815	-0.404217604	3.551457005	0.456303718	NA	NA
ASV_16s_20916	0.098047815	-0.404217604	3.551457005	0.456303718	NA	NA
ASV_16s_20917	0.098047815	-0.404217604	3.551457005	0.456303718	NA	NA
ASV_16s_20918	0.098047815	-0.404217604	3.551457005	0.456303718	NA	NA
ASV_16s_20919	0.098047815	-0.404217604	3.551457005	0.456303718	NA	NA
ASV_16s_20920	0.098047815	-0.404217604	3.551457005	0.456303718	NA	NA
ASV_16s_20921	0.098047815	-0.404217604	3.551457005	0.456303718	NA	NA
ASV_16s_20922	0.098047815	-0.404217604	3.551457005	0.456303718	NA	NA
ASV_16s_20923	0.098047815	-0.404217604	3.551457005	0.456303718	NA	NA
ASV_16s_20924	0.098047815	-0.404217604	3.551457005	0.456303718	NA	NA
ASV_16s_20925	0.098047815	-0.404217604	3.551457005	0.456303718	NA	NA
ASV_16s_20926	0.098047815	-0.404217604	3.551457005	0.456303718	NA	NA
ASV_16s_20927	0.167281655	1.148714333	3.551308925	0.44220191	0.999631806	NA
ASV_16s_20928	0.031238661	-0.143107748	3.551226026	0.120356366	NA	NA
ASV_16s_20929	0.031663614	-0.042595858	3.551199398	0.169746986	0.999985664	NA
ASV_16s_20930	0.032230218	0.070636385	3.55121698	0.182403006	0.999981651	NA
ASV_16s_20931	0.063465487	0.220277391	3.551031853	0.289258118	NA	NA
ASV_16s_20932	0.032938472	0.231570285	3.551328458	0.111781105	NA	NA
ASV_16s_20933	0.043547773	0.22534824	3.551192896	0.177439853	NA	NA
ASV_16s_20937	0.152909118	0.595998504	3.5507299	0.570550278	NA	NA
ASV_16s_20938	0.147991048	0.442001381	3.550737462	0.530099431	0.999328635	NA
ASV_16s_20939	0.101172092	0.063728672	3.551891436	0.427952445	0.999669879	NA
ASV_16s_20940	0.104247667	0.535150637	3.551964243	0.424938772	0.99967757	NA
ASV_16s_20941	0.107532376	0.369285566	3.551842274	0.477206278	0.999525737	NA

ASV_16s_20942	0.11739607	0.216777313	3.551691409	0.596623127	0.999010186	NA
ASV_16s_20943	0.131465847	-0.448400926	3.551346303	0.527697333	0.999338612	NA
ASV_16s_20944	0.126212784	-0.430518475	3.551389452	0.511769237	0.999402236	NA
ASV_16s_20945	0.109190405	-0.039659215	3.551567057	0.364456463	0.999807165	NA
ASV_16s_20946	0.10668084	0.026345214	3.551847751	0.451754278	0.999604667	NA
ASV_16s_20947	0.149540278	-0.04835156	3.55140844	0.794661008	0.997498058	NA
ASV_16s_20948	0.112432104	0.308960228	3.551717603	0.57639884	0.999115886	NA
ASV_16s_20949	0.13880068	-0.448475294	3.551217849	0.613937193	0.9989132	NA
ASV_16s_20950	0.127312321	1.064113643	3.551364763	0.410249835	0.999713303	NA
ASV_16s_20951	0.125435553	-0.380853175	3.551236056	0.562082364	0.99918592	NA
ASV_16s_20952	0.130581036	-0.397451046	3.551181574	0.581427232	0.999090354	NA
ASV_16s_20953	0.126861421	-0.01776025	3.551201829	0.547477311	0.999253402	NA
ASV_16s_20954	0.129702924	-0.367871549	3.551163176	0.589936041	0.999046027	NA
ASV_16s_20955	0.13143988	-0.348428606	3.551143405	0.605045555	0.998963768	NA
ASV_16s_20956	0.130206665	-0.34798656	3.5511561	0.599273937	0.998995731	NA
ASV_16s_20957	0.123786592	-0.312687884	3.551188638	0.579842032	0.999098456	NA
ASV_16s_20958	0.103771377	0.330931763	3.551892021	0.413673051	0.999705232	NA
ASV_16s_20959	0.103771377	0.330931763	3.551892021	0.413673051	0.999705232	NA
ASV_16s_20960	0.119439659	-0.283382121	3.551215538	0.567231795	0.999161179	NA
ASV_16s_20961	0.122561671	-0.284449354	3.551185515	0.581450986	0.999090232	NA
ASV_16s_20962	0.139183412	0.404315722	3.551257204	0.412168691	0.999708798	NA
ASV_16s_20963	0.125267826	-0.535616714	3.55115109	0.454654697	0.999596163	NA
ASV_16s_20964	0.119514854	-0.224541721	3.551224896	0.576119884	0.999117288	NA
ASV_16s_20965	0.116921165	0.674743583	3.551382131	0.534899857	0.999308393	NA
ASV_16s_20966	0.127367674	-0.165641897	3.551184888	0.612689801	0.998920392	NA
ASV_16s_20967	0.12263801	-0.578300617	3.551130266	0.415859761	0.999699995	NA
ASV_16s_20968	0.126295714	-0.110657633	3.551176725	0.613739651	0.998914341	NA
ASV_16s_20969	0.140510594	-0.613712194	3.551444392	0.365135247	0.999805956	NA
ASV_16s_20970	0.109651145	0.683485514	3.55141688	0.548455545	0.999249005	NA
ASV_16s_20971	0.129560216	0.581827535	3.551117173	0.467749517	0.999556211	NA
ASV_16s_20972	0.13605817	0.049849051	3.551135327	0.651496155	0.998681296	NA
ASV_16s_20973	0.118848581	1.036590478	3.551334655	0.36421206	0.999807599	NA
ASV_16s_20974	0.122749541	0.102085635	3.5511318	0.627359196	0.998833768	NA
ASV_16s_20975	0.114876384	0.883559386	3.551270994	0.360110743	0.999814783	NA
ASV_16s_20976	0.149439933	0.047518502	3.55174241	0.504531177	0.99942972	NA
ASV_16s_20977	0.128622619	0.998713161	3.551502819	0.480299452	NA	NA
ASV_16s_20978	0.128622619	0.998713161	3.551502819	0.480299452	NA	NA
ASV_16s_20979	0.128622619	0.998713161	3.551502819	0.480299452	NA	NA
ASV_16s_20980	0.128622619	0.998713161	3.551502819	0.480299452	NA	NA
ASV_16s_20981	0.128622619	0.590069803	3.551556957	0.380487934	0.999777191	NA
ASV_16s_20982	0.108544037	-0.445530921	3.551694425	0.376501132	0.999784925	NA
ASV_16s_20983	0.136282669	1.086151304	3.551339255	0.499466642	NA	NA
ASV_16s_20984	0.108835602	1.232851469	3.551504884	0.242908931	0.999951148	NA
ASV_16s_20985	0.122141574	1.047738129	3.551426356	0.471758781	NA	NA
ASV_16s_20986	0.144248061	0.942520282	3.551229682	0.590623437	0.999042384	NA
ASV_16s_20987	0.163515194	1.46501649	3.550972105	0.313094361	0.999884414	NA
ASV_16s_20988	0.112064738	1.017369684	3.551487248	0.445621954	NA	NA
ASV_16s_20989	0.17707875	1.174550638	3.551114895	0.590834848	NA	NA
ASV_16s_20990	0.106245073	0.335282417	3.551953999	0.464633358	NA	NA
ASV_16s_20991	0.106245073	0.335282417	3.551953999	0.464633358	NA	NA
ASV_16s_20992	0.106245073	0.335282417	3.551953999	0.464633358	NA	NA
ASV_16s_20993	0.106245073	0.335282417	3.551953999	0.464633358	NA	NA
ASV_16s_20994	0.106245073	0.335282417	3.551953999	0.464633358	NA	NA
ASV_16s_20995	0.122520196	0.340827029	3.551907478	0.499588081	0.999447988	NA
ASV_16s_20996	0.128622619	0.342851832	3.551887949	0.514708393	NA	NA
ASV_16s_20997	0.128622619	0.342851832	3.551887949	0.514708393	NA	NA
ASV_16s_20998	0.128622619	0.342851832	3.551887949	0.514708393	NA	NA
ASV_16s_20999	0.128622619	0.342851832	3.551887949	0.514708393	NA	NA
ASV_16s_21000	0.128622619	0.342851832	3.551887949	0.514708393	NA	NA
ASV_16s_21001	0.142262131	0.347517696	3.55184201	0.54706936	NA	NA
ASV_16s_21002	0.142262131	0.347517696	3.55184201	0.54706936	NA	NA
ASV_16s_21003	0.082333585	0.327295136	3.552013018	0.392995678	0.999751686	NA
ASV_16s_21004	0.082333585	0.327295136	3.552013018	0.392995678	0.999751686	NA
ASV_16s_21005	0.082333585	0.327295136	3.552013018	0.392995678	0.999751686	NA
ASV_16s_21006	0.082333585	0.327295136	3.552013018	0.392995678	0.999751686	NA
ASV_16s_21007	0.082333585	0.327295136	3.552013018	0.392995678	0.999751686	NA
ASV_16s_21008	0.10055165	-0.071234801	3.551351311	0.531560806	0.999322516	NA

ASV_16s_21009	0.111481511	0.337056084	3.551941973	0.477948487	NA	NA
ASV_16s_21010	0.111481511	0.337056084	3.551941973	0.477948487	NA	NA
ASV_16s_21011	0.111481511	0.337056084	3.551941973	0.477948487	NA	NA
ASV_16s_21012	0.111481511	0.337056084	3.551941973	0.477948487	NA	NA
ASV_16s_21013	0.132558671	0.333533383	3.552046192	0.296566176	0.999903786	NA
ASV_16s_21014	0.149645401	0.349566595	3.551809367	0.565364999	NA	NA
ASV_16s_21015	0.137760437	-0.478750212	3.551229497	0.540622334	NA	NA
ASV_16s_21016	0.137760437	-0.478750212	3.551229497	0.540622334	NA	NA
ASV_16s_21017	0.118368778	-0.435467187	3.551385413	0.498681901	NA	NA
ASV_16s_21018	0.082227475	-0.327430273	3.551699436	0.39992495	NA	NA
ASV_16s_21019	0.082227475	-0.327430273	3.551699436	0.39992495	NA	NA
ASV_16s_21020	0.082227475	-0.327430273	3.551699436	0.39992495	NA	NA
ASV_16s_21021	0.082227475	-0.327430273	3.551699436	0.39992495	NA	NA
ASV_16s_21022	0.082227475	-0.327430273	3.551699436	0.39992495	NA	NA
ASV_16s_21023	0.120964205	-0.441867712	3.551363446	0.503963753	NA	NA
ASV_16s_21024	0.107630545	-0.407735123	3.551479113	0.475340683	NA	NA
ASV_16s_21025	0.107630545	-0.407735123	3.551479113	0.475340683	NA	NA
ASV_16s_21026	0.107630545	-0.407735123	3.551479113	0.475340683	NA	NA
ASV_16s_21027	0.148300142	1.116030863	3.5512703	0.524866013	NA	NA
ASV_16s_21028	0.143523359	0.969339388	3.551657721	0.424660324	0.999678274	NA
ASV_16s_21029	0.121912646	1.0872589	3.551173197	0.413257499	NA	NA
ASV_16s_21030	0.121912646	1.0872589	3.551173197	0.413257499	NA	NA
ASV_16s_21031	0.063626657	0.884130008	3.551643899	0.245800091	0.99994914	NA
ASV_16s_21032	0.063626657	0.884130008	3.551643899	0.245800091	0.99994914	NA
ASV_16s_21033	0.063626657	0.884130008	3.551643899	0.245800091	0.99994914	NA
ASV_16s_21034	0.109078505	1.050782118	3.551259648	0.380288656	NA	NA
ASV_16s_21035	0.109078505	1.050782118	3.551259648	0.380288656	NA	NA
ASV_16s_21036	0.109078505	1.050782118	3.551259648	0.380288656	NA	NA
ASV_16s_21037	0.127950377	0.359999619	3.551571471	0.45041546	NA	NA
ASV_16s_21038	0.127950377	0.359999619	3.551571471	0.45041546	NA	NA
ASV_16s_21039	0.137878071	0.364424209	3.55152253	0.472911837	NA	NA
ASV_16s_21040	0.137878071	0.364424209	3.55152253	0.472911837	NA	NA
ASV_16s_21041	0.124708249	0.358434797	3.551584854	0.44330313	0.999628745	NA
ASV_16s_21042	0.12507674	0.35861796	3.551583278	0.4441224	NA	NA
ASV_16s_21043	0.12507674	0.35861796	3.551583278	0.4441224	NA	NA
ASV_16s_21044	0.12507674	0.35861796	3.551583278	0.4441224	NA	NA
ASV_16s_21045	0.113564554	0.352852693	3.551634338	0.420415172	NA	NA
ASV_16s_21046	0.113564554	0.352852693	3.551634338	0.420415172	NA	NA
ASV_16s_21047	0.113564554	0.352852693	3.551634338	0.420415172	NA	NA
ASV_16s_21048	0.113564554	0.352852693	3.551634338	0.420415172	NA	NA
ASV_16s_21049	0.122308211	0.012860984	3.551141307	0.631611787	0.998807811	NA
ASV_16s_21050	0.093746198	0.345883639	3.551713316	0.368480279	NA	NA
ASV_16s_21051	0.093746198	0.345883639	3.551713316	0.368480279	NA	NA
ASV_16s_21052	0.101430063	0.347272989	3.551689805	0.390446971	NA	NA
ASV_16s_21053	0.101430063	0.347272989	3.551689805	0.390446971	NA	NA
ASV_16s_21054	0.101430063	0.347272989	3.551689805	0.390446971	NA	NA
ASV_16s_21055	0.128622619	-0.473683567	3.550989206	0.453918495	NA	NA
ASV_16s_21056	0.139795526	-0.493083114	3.550874443	0.478456764	NA	NA
ASV_16s_21057	0.180327645	-0.532802543	3.550496684	0.578145679	NA	NA
ASV_16s_21058	0.180327645	-0.532802543	3.550496684	0.578145679	NA	NA
ASV_16s_21059	0.180327645	-0.532802543	3.550496684	0.578145679	NA	NA
ASV_16s_21060	0.151684998	-0.312194725	3.551052463	0.593606024	0.999026467	NA
ASV_16s_21061	0.117710251	-0.394154668	3.551177108	0.354848752	0.999823726	NA
ASV_16s_21062	0.069210181	-0.303000662	3.551482252	0.294005407	0.99990657	NA
ASV_16s_21063	0.113564554	-0.441964742	3.551135373	0.423923454	NA	NA
ASV_16s_21064	0.05028302	0.820068555	3.551767106	0.199798057	0.999974931	NA
ASV_16s_21065	0.05028302	0.820068555	3.551767106	0.199798057	0.999974931	NA
ASV_16s_21066	0.05028302	0.820068555	3.551767106	0.199798057	0.999974931	NA
ASV_16s_21067	0.113564554	-0.078018157	3.550995157	0.668769838	NA	NA
ASV_16s_21068	0.113564554	-0.078018157	3.550995157	0.668769838	NA	NA
ASV_16s_21069	0.113564554	-0.078018157	3.550995157	0.668769838	NA	NA
ASV_16s_21070	0.113564554	-0.078018157	3.550995157	0.668769838	NA	NA
ASV_16s_21071	0.096837787	-0.077742211	3.551077476	0.603817693	NA	NA
ASV_16s_21072	0.096837787	-0.077742211	3.551077476	0.603817693	NA	NA
ASV_16s_21073	0.096837787	-0.077742211	3.551077476	0.603817693	NA	NA
ASV_16s_21074	0.096837787	-0.077742211	3.551077476	0.603817693	NA	NA
ASV_16s_21075	0.096837787	-0.077742211	3.551077476	0.603817693	NA	NA

ASV_16s_21076	0.096837787	-0.077742211	3.551077476	0.603817693	NA	NA
ASV_16s_21077	0.096837787	-0.077742211	3.551077476	0.603817693	NA	NA
ASV_16s_21078	0.096837787	-0.077742211	3.551077476	0.603817693	NA	NA
ASV_16s_21079	0.096837787	-0.077742211	3.551077476	0.603817693	NA	NA
ASV_16s_21080	0.096837787	-0.077742211	3.551077476	0.603817693	NA	NA
ASV_16s_21081	0.102530747	-0.077844856	3.551046474	0.625684028	0.998843887	NA
ASV_16s_21082	0.109258791	-0.077955089	3.551013958	0.654185289	NA	NA
ASV_16s_21083	0.063465487	0.220277391	3.551031853	0.289258118	NA	NA
ASV_16s_21084	0.066646587	0.220260943	3.55101028	0.305691992	0.999893395	NA
ASV_16s_21085	0.072092384	0.220236456	3.550977729	0.332934614	NA	NA
ASV_16s_21086	0.072092384	0.220236456	3.550977729	0.332934614	NA	NA
ASV_16s_21087	0.0996243	0.233273728	3.551297679	0.282593683	0.999918299	NA
ASV_16s_21088	0.125119323	0.310954903	3.551110143	0.68602996	NA	NA
ASV_16s_21089	0.125119323	0.310954903	3.551110143	0.68602996	NA	NA
ASV_16s_21090	0.125119323	0.310954903	3.551110143	0.68602996	NA	NA
ASV_16s_21091	0.107287346	0.310960465	3.551196142	0.627566094	NA	NA
ASV_16s_21092	0.122652894	0.310955577	3.55112056	0.678039862	NA	NA
ASV_16s_21093	0.075943637	0.595535516	3.550911244	0.30136184	NA	NA
ASV_16s_21094	0.075943637	0.595535516	3.550911244	0.30136184	NA	NA
ASV_16s_21095	0.075943637	0.595535516	3.550911244	0.30136184	NA	NA
ASV_16s_21096	0.09881986	-0.066135452	3.55114505	0.60587454	0.998959122	NA
ASV_16s_21097	0.132025374	1.172247639	3.551459105	0.315967361	0.999880792	NA
ASV_16s_21098	0.112355972	-0.065337993	3.551078372	0.670670041	0.998551085	NA
ASV_16s_21099	0.099810896	-0.064993416	3.551187159	0.622217919	0.998864639	NA
ASV_16s_21100	0.126610559	1.029745065	3.551308556	0.464974058	0.999564895	NA
ASV_16s_21101	0.158251344	0.73806981	3.551396683	0.655477063	0.998654934	NA
ASV_16s_21102	0.14120654	0.670633652	3.55112529	0.778191033	0.997660203	NA
ASV_16s_21103	0.197688334	0.36753931	3.551651728	0.525400526	0.999348057	NA
ASV_16s_21104	0.111751681	-0.066878161	3.551153051	0.689165686	0.998417605	NA
ASV_16s_21105	0.122613705	0.343344707	3.552005057	0.41888679	0.999692641	NA
ASV_16s_21106	0.10179297	-0.060344345	3.551299247	0.650736368	0.998686288	NA
ASV_16s_21107	0.068485502	0.506374484	3.551192113	0.345501443	0.999838869	NA
ASV_16s_21108	0.103279525	-0.048477257	3.551418263	0.658366513	0.99863558	NA
ASV_16s_21109	0.103279525	-0.048477257	3.551418263	0.658366513	0.99863558	NA
ASV_16s_21110	0.098171568	0.184338899	3.55176219	0.510294879	0.999407906	NA
ASV_16s_21111	0.130209138	0.355012409	3.55117906	0.722609586	0.99815592	NA
ASV_16s_21112	0.097008275	0.976802253	3.551771821	0.355209377	0.999823123	NA
ASV_16s_21113	0.147382418	-0.720295807	3.551413119	0.372196431	0.999793066	NA
ASV_16s_21114	0.10476608	-0.012517067	3.551564619	0.654164672	0.998663663	NA
ASV_16s_21115	0.108906415	-0.028194187	3.551485545	0.671261569	0.998546937	NA
ASV_16s_21116	0.114769486	0.325840707	3.551556934	0.458512639	0.999584659	NA
ASV_16s_21117	0.109737378	0.105362378	3.551592101	0.654634464	0.998660543	NA
ASV_16s_21118	0.108840344	0.105362378	3.551592101	0.652044897	0.998677683	NA
ASV_16s_21119	0.108840344	0.105362378	3.551592101	0.652044897	0.998677683	NA
ASV_16s_21120	0.153557257	0.292510479	3.551767351	0.574326038	0.999126268	NA
ASV_16s_21121	0.148300142	0.293303611	3.551782548	0.560944342	NA	NA
ASV_16s_21122	0.148300142	0.293303611	3.551782548	0.560944342	NA	NA
ASV_16s_21123	0.125846105	0.992013445	3.551522236	0.4750941	NA	NA
ASV_16s_21124	0.125846105	0.992013445	3.551522236	0.4750941	NA	NA
ASV_16s_21125	0.121237056	0.90703393	3.551519961	0.449421244	0.999611417	NA
ASV_16s_21126	0.121237056	0.90703393	3.551519961	0.449421244	0.999611417	NA
ASV_16s_21127	0.103248908	0.284236911	3.551737943	0.448600509	0.999613773	NA
ASV_16s_21128	0.192526921	0.279446467	3.551561827	0.697126037	NA	NA
ASV_16s_21129	0.081639893	0.286438252	3.551804877	0.393542146	0.999750528	NA
ASV_16s_21130	0.029291933	0.299541861	3.551953116	0.17521716	0.999984014	NA
ASV_16s_21131	0.160591524	-0.475918616	3.55129958	0.596985426	NA	NA
ASV_16s_21132	0.095483395	-0.332217825	3.551573433	0.438071939	0.999643135	NA
ASV_16s_21133	0.096238934	0.850708737	3.551625287	0.383779007	0.999770664	NA
ASV_16s_21134	0.229185261	-0.00167603	3.55086084	1.004958252	0.994748156	NA
ASV_16s_21135	0.226417251	1.247433309	3.550892093	0.716903795	NA	NA
ASV_16s_21136	0.155911167	0.930218132	3.550912661	0.705659292	0.998291875	NA
ASV_16s_21137	0.132405864	0.32371614	3.551521536	0.437875464	0.999643668	NA
ASV_16s_21138	0.142618173	1.13901799	3.551060232	0.454133912	NA	NA
ASV_16s_21139	0.142618173	1.13901799	3.551060232	0.454133912	NA	NA
ASV_16s_21140	0.129301931	1.00472201	3.55122152	0.515404813	0.999388097	NA
ASV_16s_21141	0.081268483	-0.077384996	3.551184221	0.534710826	NA	NA
ASV_16s_21142	0.081268483	-0.077384996	3.551184221	0.534710826	NA	NA

ASV_16s_21143	0.081268483	-0.077384996	3.551184221	0.534710826	NA	NA
ASV_16s_21144	0.081268483	-0.077384996	3.551184221	0.534710826	NA	NA
ASV_16s_21145	0.186904216	-0.423504028	3.550821893	0.747262444	0.997945657	NA
ASV_16s_21146	0.170785798	0.395317813	3.55104307	1.215354163	0.990569064	NA
ASV_16s_21147	0.142822088	-0.414494571	3.551026687	0.596228276	0.999012327	NA
ASV_16s_21148	0.171474848	0.91945444	3.550975148	0.703577894	0.998308095	NA
ASV_16s_21149	0.179388153	-0.028624926	3.55097209	0.949517257	NA	NA
ASV_16s_21150	0.179388153	-0.028624926	3.55097209	0.949517257	NA	NA
ASV_16s_21151	0.179388153	-0.028624926	3.55097209	0.949517257	NA	NA
ASV_16s_21152	0.123672958	0.633138602	3.551382584	0.739061872	0.998017271	NA
ASV_16s_21153	0.122515206	-0.072964126	3.551004639	0.721036253	NA	NA
ASV_16s_21154	0.089480158	-0.072333806	3.551183995	0.596158388	NA	NA
ASV_16s_21155	0.089480158	-0.072333806	3.551183995	0.596158388	NA	NA
ASV_16s_21156	0.089480158	-0.072333806	3.551183995	0.596158388	NA	NA
ASV_16s_21157	0.089480158	-0.072333806	3.551183995	0.596158388	NA	NA
ASV_16s_21158	0.089480158	-0.072333806	3.551183995	0.596158388	NA	NA
ASV_16s_21159	0.099283489	-0.072563626	3.551118535	0.630463428	0.998814858	NA
ASV_16s_21160	0.087872349	-0.072291239	3.551196127	0.588988149	NA	NA
ASV_16s_21161	0.087872349	-0.072291239	3.551196127	0.588988149	NA	NA
ASV_16s_21162	0.087872349	-0.072291239	3.551196127	0.588988149	NA	NA
ASV_16s_21163	0.087872349	-0.072291239	3.551196127	0.588988149	NA	NA
ASV_16s_21164	0.087872349	-0.072291239	3.551196127	0.588988149	NA	NA
ASV_16s_21165	0.087872349	-0.072291239	3.551196127	0.588988149	NA	NA
ASV_16s_21166	0.113855516	0.299535192	3.551218595	0.701438535	0.998324659	NA
ASV_16s_21167	0.148300142	-0.073262246	3.550919798	0.806144724	NA	NA
ASV_16s_21168	0.148300142	-0.073262246	3.550919798	0.806144724	NA	NA
ASV_16s_21169	0.148300142	-0.073262246	3.550919798	0.806144724	NA	NA
ASV_16s_21170	0.14982949	0.473958714	3.551327956	0.776443646	0.997676987	NA
ASV_16s_21171	0.109028563	-0.072752281	3.551064853	0.677414001	NA	NA
ASV_16s_21172	0.109028563	-0.072752281	3.551064853	0.677414001	NA	NA
ASV_16s_21173	0.109028563	-0.072752281	3.551064853	0.677414001	NA	NA
ASV_16s_21174	0.127913448	0.304696541	3.551053343	0.759095462	0.997839336	NA
ASV_16s_21175	0.117208894	0.334606781	3.551785331	0.526199074	0.999344784	NA
ASV_16s_21176	0.117208894	0.334606781	3.551785331	0.526199074	0.999344784	NA
ASV_16s_21177	0.120838505	0.320433166	3.551315109	0.598614089	NA	NA
ASV_16s_21178	0.320025623	0.360377646	3.550912108	1.469726884	NA	NA
ASV_16s_21179	0.108730226	0.359591738	3.551516736	0.694679794	NA	NA
ASV_16s_21180	0.108730226	0.359591738	3.551516736	0.694679794	NA	NA
ASV_16s_21181	0.108730226	0.359591738	3.551516736	0.694679794	NA	NA
ASV_16s_21182	0.108730226	0.359591738	3.551516736	0.694679794	NA	NA
ASV_16s_21183	0.108730226	0.359591738	3.551516736	0.694679794	NA	NA
ASV_16s_21184	0.108730226	0.359591738	3.551516736	0.694679794	NA	NA
ASV_16s_21185	0.108730226	0.359591738	3.551516736	0.694679794	NA	NA
ASV_16s_21186	0.108730226	0.359591738	3.551516736	0.694679794	NA	NA
ASV_16s_21187	0.108730226	0.359591738	3.551516736	0.694679794	NA	NA
ASV_16s_21188	0.108730226	0.359591738	3.551516736	0.694679794	NA	NA
ASV_16s_21189	0.108730226	0.359591738	3.551516736	0.694679794	NA	NA
ASV_16s_21190	0.108730226	0.359591738	3.551516736	0.694679794	NA	NA
ASV_16s_21191	0.108730226	0.359591738	3.551516736	0.694679794	NA	NA
ASV_16s_21192	0.108730226	0.359591738	3.551516736	0.694679794	NA	NA
ASV_16s_21193	0.108730226	0.359591738	3.551516736	0.694679794	NA	NA
ASV_16s_21194	0.108730226	0.359591738	3.551516736	0.694679794	NA	NA
ASV_16s_21195	0.108730226	0.359591738	3.551516736	0.694679794	NA	NA
ASV_16s_21196	0.108730226	0.359591738	3.551516736	0.694679794	NA	NA
ASV_16s_21197	0.108730226	0.359591738	3.551516736	0.694679794	NA	NA
ASV_16s_21198	0.108730226	0.359591738	3.551516736	0.694679794	NA	NA
ASV_16s_21199	0.108730226	0.359591738	3.551516736	0.694679794	NA	NA
ASV_16s_21200	0.108730226	0.359591738	3.551516736	0.694679794	NA	NA
ASV_16s_21201	0.108730226	0.359591738	3.551516736	0.694679794	NA	NA
ASV_16s_21202	0.108730226	0.359591738	3.551516736	0.694679794	NA	NA
ASV_16s_21203	0.108730226	0.359591738	3.551516736	0.694679794	NA	NA
ASV_16s_21204	0.140881165	0.315940029	3.55109578	0.761154288	NA	NA
ASV_16s_21205	0.128006932	0.566827751	3.551085988	0.885269585	0.996473281	NA
ASV_16s_21206	0.121777248	0.315940157	3.551174819	0.697997118	NA	NA
ASV_16s_21207	0.129972036	0.460052174	3.551079814	0.955265751	0.995518247	NA
ASV_16s_21208	0.118355292	0.315940079	3.551191646	0.687036409	0.998433372	NA
ASV_16s_21209	0.116644314	0.315940198	3.551200428	0.681603965	0.998473125	NA

ASV_16s_21210	0.163828233	0.315939758	3.551025093	0.840342688	0.997010114	NA
ASV_16s_21211	0.115275532	0.31594021	3.551207637	0.677281848	0.998504268	NA
ASV_16s_21212	0.189058824	0.315939798	3.55096696	0.931121002	NA	NA
ASV_16s_21213	0.189058824	0.315939798	3.55096696	0.931121002	NA	NA
ASV_16s_21214	0.189058824	0.315939798	3.55096696	0.931121002	NA	NA
ASV_16s_21215	0.13872931	0.315939958	3.551103712	0.753640755	0.997888788	NA
ASV_16s_21216	0.113564554	0.315940224	3.551216889	0.671909828	NA	NA
ASV_16s_21217	0.113564554	0.315940224	3.551216889	0.671909828	NA	NA
ASV_16s_21218	0.113564554	0.315940224	3.551216889	0.671909828	NA	NA
ASV_16s_21219	0.113564554	0.315940224	3.551216889	0.671909828	NA	NA
ASV_16s_21220	0.113564554	0.315940224	3.551216889	0.671909828	NA	NA
ASV_16s_21221	0.113564554	0.315940224	3.551216889	0.671909828	NA	NA
ASV_16s_21222	0.113564554	0.315940224	3.551216889	0.671909828	NA	NA
ASV_16s_21223	0.113564554	0.315940224	3.551216889	0.671909828	NA	NA
ASV_16s_21224	0.113564554	0.315940224	3.551216889	0.671909828	NA	NA
ASV_16s_21225	0.113564554	0.315940224	3.551216889	0.671909828	NA	NA
ASV_16s_21226	0.113564554	0.315940224	3.551216889	0.671909828	NA	NA
ASV_16s_21227	0.152909118	0.595998504	3.5507299	0.570550278	NA	NA
ASV_16s_21228	0.10815173	0.686971545	3.551166273	0.574321842	NA	NA
ASV_16s_21229	0.10815173	0.686971545	3.551166273	0.574321842	NA	NA
ASV_16s_21230	0.10815173	0.686971545	3.551166273	0.574321842	NA	NA
ASV_16s_21231	0.10815173	0.686971545	3.551166273	0.574321842	NA	NA
ASV_16s_21232	0.10815173	0.686971545	3.551166273	0.574321842	NA	NA
ASV_16s_21233	0.195383665	0.687809728	3.550894533	0.846590568	NA	NA
ASV_16s_21234	0.202817798	0.733780371	3.551105711	0.935560061	0.995802158	NA
ASV_16s_21235	0.176996254	1.058081495	3.551245119	0.658074297	0.998637545	NA
ASV_16s_21236	0.177534918	0.733524192	3.551180394	0.854953408	NA	NA
ASV_16s_21237	0.177534918	0.733524192	3.551180394	0.854953408	NA	NA
ASV_16s_21238	0.153445533	1.018633702	3.551202257	0.579515446	0.999100119	NA
ASV_16s_21239	0.177534918	0.733524192	3.551180394	0.854953408	NA	NA
ASV_16s_21240	0.161608186	0.733321577	3.551239382	0.805398855	0.997388465	NA
ASV_16s_21241	0.158195315	0.733272956	3.551253545	0.794965179	0.997494996	NA
ASV_16s_21242	0.150231949	0.732693762	3.551288086	0.770852031	NA	NA
ASV_16s_21243	0.150231949	0.732693762	3.551288086	0.770852031	NA	NA
ASV_16s_21244	0.150231949	0.732693762	3.551288086	0.770852031	NA	NA
ASV_16s_21245	0.150231949	0.732693762	3.551288086	0.770852031	NA	NA
ASV_16s_21246	0.150231949	0.732693762	3.551288086	0.770852031	NA	NA
ASV_16s_21247	0.150231949	0.732693762	3.551288086	0.770852031	NA	NA
ASV_16s_21248	0.150231949	0.732693762	3.551288086	0.770852031	NA	NA
ASV_16s_21249	0.150231949	0.732693762	3.551288086	0.770852031	NA	NA
ASV_16s_21250	0.150231949	0.732693762	3.551288086	0.770852031	NA	NA
ASV_16s_21251	0.150231949	0.732693762	3.551288086	0.770852031	NA	NA
ASV_16s_21252	0.150231949	0.732693762	3.551288086	0.770852031	NA	NA
ASV_16s_21253	0.150231949	0.732693762	3.551288086	0.770852031	NA	NA
ASV_16s_21254	0.150231949	0.732693762	3.551288086	0.770852031	NA	NA
ASV_16s_21255	0.150231949	0.732693762	3.551288086	0.770852031	NA	NA
ASV_16s_21256	0.224600552	0.733957028	3.551054607	1.007394659	NA	NA
ASV_16s_21257	0.074418247	0.673502816	3.551692167	0.496984373	0.999457449	NA
ASV_16s_21258	0.074418247	0.673502816	3.551692167	0.496984373	0.999457449	NA
ASV_16s_21259	0.074418247	0.673502816	3.551692167	0.496984373	0.999457449	NA
ASV_16s_21260	0.13966704	0.69237302	3.551081871	0.699082949	NA	NA
ASV_16s_21261	0.13966704	0.69237302	3.551081871	0.699082949	NA	NA
ASV_16s_21262	0.13966704	0.69237302	3.551081871	0.699082949	NA	NA
ASV_16s_21263	0.13966704	0.69237302	3.551081871	0.699082949	NA	NA
ASV_16s_21264	0.13966704	0.69237302	3.551081871	0.699082949	NA	NA
ASV_16s_21265	0.140988031	0.692389712	3.551076951	0.702950859	0.998312961	NA
ASV_16s_21266	0.141813651	0.692399989	3.551073923	0.705375189	0.998294095	NA
ASV_16s_21267	0.141978775	0.69240203	3.551073321	0.705860684	0.9982903	NA
ASV_16s_21268	0.123352986	0.692137796	3.551151248	0.648130351	0.998703314	NA
ASV_16s_21269	0.142804395	0.692412167	3.551070334	0.708291278	0.998271216	NA
ASV_16s_21270	0.143630015	0.69242219	3.551067381	0.710727025	NA	NA
ASV_16s_21271	0.143630015	0.69242219	3.551067381	0.710727025	NA	NA
ASV_16s_21272	0.143630015	0.69242219	3.551067381	0.710727025	NA	NA
ASV_16s_21273	0.143630015	0.69242219	3.551067381	0.710727025	NA	NA
ASV_16s_21274	0.143630015	0.69242219	3.551067381	0.710727025	NA	NA
ASV_16s_21275	0.143630015	0.69242219	3.551067381	0.710727025	NA	NA
ASV_16s_21276	0.143630015	0.69242219	3.551067381	0.710727025	NA	NA

ASV_16s_21277	0.148400938	0.692477928	3.551050962	0.724880008	0.998137181	NA
ASV_16s_21278	0.127344556	0.692200797	3.551132658	0.660083965	0.998623988	NA
ASV_16s_21279	0.126091829	0.692181441	3.551138369	0.656256171	0.998649733	NA
ASV_16s_21280	0.134620935	0.692304814	3.551101586	0.681848887	0.998471347	NA
ASV_16s_21281	0.113564554	0.691964873	3.551202306	0.618697292	NA	NA
ASV_16s_21282	0.113564554	0.691964873	3.551202306	0.618697292	NA	NA
ASV_16s_21283	0.113564554	0.691964873	3.551202306	0.618697292	NA	NA
ASV_16s_21284	0.113564554	0.691964873	3.551202306	0.618697292	NA	NA
ASV_16s_21285	0.113564554	0.691964873	3.551202306	0.618697292	NA	NA
ASV_16s_21286	0.113564554	0.691964873	3.551202306	0.618697292	NA	NA
ASV_16s_21287	0.113564554	0.691964873	3.551202306	0.618697292	NA	NA
ASV_16s_21288	0.135759461	0.692321546	3.551097044	0.68481637	0.9984497	NA
ASV_16s_21289	0.093831296	0.299807533	3.551938819	0.428301189	0.999668981	NA
ASV_16s_21290	0.093831296	0.299807533	3.551938819	0.428301189	0.999668981	NA
ASV_16s_21291	0.093831296	0.299807533	3.551938819	0.428301189	0.999668981	NA
ASV_16s_21292	0.093831296	0.299807533	3.551938819	0.428301189	0.999668981	NA
ASV_16s_21293	0.093831296	0.299807533	3.551938819	0.428301189	0.999668981	NA
ASV_16s_21294	0.093831296	0.299807533	3.551938819	0.428301189	0.999668981	NA
ASV_16s_21295	0.093831296	0.299807533	3.551938819	0.428301189	0.999668981	NA
ASV_16s_21296	0.093831296	0.299807533	3.551938819	0.428301189	0.999668981	NA
ASV_16s_21297	0.093831296	0.299807533	3.551938819	0.428301189	0.999668981	NA
ASV_16s_21298	0.093831296	0.299807533	3.551938819	0.428301189	0.999668981	NA
ASV_16s_21299	0.093831296	0.299807533	3.551938819	0.428301189	0.999668981	NA
ASV_16s_21300	0.093831296	0.299807533	3.551938819	0.428301189	0.999668981	NA
ASV_16s_21301	0.093831296	0.299807533	3.551938819	0.428301189	0.999668981	NA
ASV_16s_21302	0.093831296	0.299807533	3.551938819	0.428301189	0.999668981	NA
ASV_16s_21303	0.093831296	0.299807533	3.551938819	0.428301189	0.999668981	NA
ASV_16s_21304	0.093831296	0.299807533	3.551938819	0.428301189	0.999668981	NA
ASV_16s_21305	0.093831296	0.299807533	3.551938819	0.428301189	0.999668981	NA
ASV_16s_21306	0.093831296	0.299807533	3.551938819	0.428301189	0.999668981	NA
ASV_16s_21307	0.093831296	0.299807533	3.551938819	0.428301189	0.999668981	NA
ASV_16s_21308	0.093831296	0.299807533	3.551938819	0.428301189	0.999668981	NA
ASV_16s_21309	0.093831296	0.299807533	3.551938819	0.428301189	0.999668981	NA
ASV_16s_21310	0.093831296	0.299807533	3.551938819	0.428301189	0.999668981	NA
ASV_16s_21311	0.093831296	0.299807533	3.551938819	0.428301189	0.999668981	NA
ASV_16s_21312	0.093831296	0.299807533	3.551938819	0.428301189	0.999668981	NA
ASV_16s_21313	0.093831296	0.299807533	3.551938819	0.428301189	0.999668981	NA
ASV_16s_21314	0.093831296	0.299807533	3.551938819	0.428301189	0.999668981	NA
ASV_16s_21315	0.093859817	0.103359468	3.551804454	0.539389461	0.99928909	NA
ASV_16s_21316	0.093865521	0.071207726	3.551780815	0.553797131	0.999224686	NA
ASV_16s_21317	0.095384809	0.060984337	3.551767068	0.561151531	0.99919034	NA
ASV_16s_21318	0.093865521	0.071207726	3.551780815	0.553797131	0.999224686	NA
ASV_16s_21319	0.093871225	0.040395581	3.551758439	0.566084033	0.999166737	NA
ASV_16s_21320	0.095643728	0.028573981	3.551737027	0.574470805	0.999125546	NA
ASV_16s_21321	0.106235707	-0.063942473	3.551576334	0.636460537	0.998777746	NA
ASV_16s_21322	0.095902647	-0.00312869	3.551703055	0.585399877	0.999069835	NA
ASV_16s_21323	0.095902647	-0.00312869	3.551703055	0.585399877	0.999069835	NA
ASV_16s_21324	0.096161566	-0.033393734	3.551672918	0.593977638	0.999024471	NA
ASV_16s_21325	0.096161566	-0.033393734	3.551672918	0.593977638	0.999024471	NA
ASV_16s_21326	0.096161566	-0.033393734	3.551672918	0.593977638	0.999024471	NA
ASV_16s_21327	0.093882633	-0.019547395	3.551700067	0.58371092	0.999078597	NA
ASV_16s_21328	0.096420485	-0.062549846	3.551645136	0.600119088	0.998991093	NA
ASV_16s_21329	0.093888337	-0.047975267	3.551674214	0.589049246	0.999050713	NA
ASV_16s_21330	0.096420485	-0.062549846	3.551645136	0.600119088	0.998991093	NA
ASV_16s_21331	0.096679403	-0.091101356	3.551617308	0.603776062	0.998970857	NA
ASV_16s_21332	0.110887361	-0.161692707	3.55146311	0.668099891	0.998569016	NA
ASV_16s_21333	0.110887361	-0.161692707	3.55146311	0.668099891	0.998569016	NA
ASV_16s_21334	0.093894041	-0.075543958	3.551650772	0.592066597	0.999034704	NA
ASV_16s_21335	0.096679403	-0.091101356	3.551617308	0.603776062	0.998970857	NA
ASV_16s_21336	0.096938322	-0.118746744	3.551592504	0.605015842	0.998963935	NA
ASV_16s_21337	0.093899745	-0.102680164	3.551626801	0.592704183	0.999031298	NA
ASV_16s_21338	0.096938322	-0.118746744	3.551592504	0.605015842	0.998963935	NA
ASV_16s_21339	0.101623981	-0.142126361	3.551543779	0.624619668	0.998850287	NA
ASV_16s_21340	0.097197241	-0.145678413	3.551570605	0.603710481	0.998971222	NA
ASV_16s_21341	0.093905449	-0.129161252	3.551605733	0.59089921	0.999040919	NA
ASV_16s_21342	0.097197241	-0.145678413	3.551570605	0.603710481	0.998971222	NA
ASV_16s_21343	0.095683657	-0.163684989	3.551569047	0.593418419	0.999027473	NA

ASV_16s_21344	0.095683657	-0.163684989	3.551569047	0.593418419	0.999027473	NA
ASV_16s_21345	0.097715079	-0.198018717	3.551534336	0.593876117	0.999025017	NA
ASV_16s_21346	0.093922561	-0.20603141	3.55155652	0.571476041	0.999140408	NA
ASV_16s_21347	0.10624163	-0.306261675	3.551408057	0.585161343	0.999071076	NA
ASV_16s_21348	0.033543559	-0.082760848	3.551892297	0.209987233	0.999970281	NA
ASV_16s_21349	0.033543559	-0.082760848	3.551892297	0.209987233	0.999970281	NA
ASV_16s_21350	0.137355503	-0.518349048	3.551145316	0.549409017	0.999244702	NA
ASV_16s_21351	0.135536968	-0.513808917	3.551156824	0.544689728	0.999265836	NA
ASV_16s_21352	0.128015465	-0.49445575	3.551206934	0.525737781	NA	NA
ASV_16s_21353	0.128015465	-0.49445575	3.551206934	0.525737781	NA	NA
ASV_16s_21354	0.128015465	-0.49445575	3.551206934	0.525737781	NA	NA
ASV_16s_21355	0.128015465	-0.49445575	3.551206934	0.525737781	NA	NA
ASV_16s_21356	0.128015465	-0.49445575	3.551206934	0.525737781	NA	NA
ASV_16s_21357	0.123842655	-0.483088551	3.551236541	0.515485993	0.999387779	NA
ASV_16s_21358	0.130105006	-0.499955512	3.551192541	0.530944262	0.999325102	NA
ASV_16s_21359	0.122451718	-0.47922366	3.551246749	0.512132061	0.999400835	NA
ASV_16s_21360	0.132020419	-0.504920092	3.551179636	0.535772227	NA	NA
ASV_16s_21361	0.132020419	-0.504920092	3.551179636	0.535772227	NA	NA
ASV_16s_21362	0.124367131	-0.484532088	3.551232747	0.516753891	0.999382794	NA
ASV_16s_21363	0.117351617	-0.464699411	3.551285751	0.500119053	NA	NA
ASV_16s_21364	0.117351617	-0.464699411	3.551285751	0.500119053	NA	NA
ASV_16s_21365	0.129493976	-0.498359416	3.551196716	0.529416632	NA	NA
ASV_16s_21366	0.129493976	-0.498359416	3.551196716	0.529416632	NA	NA
ASV_16s_21367	0.115590351	-0.459498524	3.551299967	0.49368274	0.999469285	NA
ASV_16s_21368	0.09978643	-0.409889216	3.551441576	0.461334701	NA	NA
ASV_16s_21369	0.09978643	-0.409889216	3.551441576	0.461334701	NA	NA
ASV_16s_21370	0.09978643	-0.409889216	3.551441576	0.461334701	NA	NA
ASV_16s_21371	0.09978643	-0.409889216	3.551441576	0.461334701	NA	NA
ASV_16s_21372	0.09978643	-0.409889216	3.551441576	0.461334701	NA	NA
ASV_16s_21373	0.09978643	-0.409889216	3.551441576	0.461334701	NA	NA
ASV_16s_21374	0.09978643	-0.409889216	3.551441576	0.461334701	NA	NA
ASV_16s_21375	0.09978643	-0.409889216	3.551441576	0.461334701	NA	NA
ASV_16s_21376	0.09978643	-0.409889216	3.551441576	0.461334701	NA	NA
ASV_16s_21377	0.09978643	-0.409889216	3.551441576	0.461334701	NA	NA
ASV_16s_21378	0.096494637	-0.398917194	3.551471571	0.450935411	0.999607045	NA
ASV_16s_21379	0.096494637	-0.398917194	3.551471571	0.450935411	0.999607045	NA
ASV_16s_21380	0.095988208	-0.397167982	3.55147615	0.449441215	0.99961136	NA
ASV_16s_21381	0.094975349	-0.393560196	3.551484964	0.446576624	0.999619541	NA
ASV_16s_21382	0.09396249	-0.389917616	3.551493919	0.44385584	0.999627202	NA
ASV_16s_21383	0.09396249	-0.389917616	3.551493919	0.44385584	0.999627202	NA
ASV_16s_21384	0.09396249	-0.389917616	3.551493919	0.44385584	0.999627202	NA
ASV_16s_21385	0.09396249	-0.389917616	3.551493919	0.44385584	0.999627202	NA
ASV_16s_21386	0.09396249	-0.389917616	3.551493919	0.44385584	0.999627202	NA
ASV_16s_21387	0.09396249	-0.389917616	3.551493919	0.44385584	0.999627202	NA
ASV_16s_21388	0.09396249	-0.389917616	3.551493919	0.44385584	0.999627202	NA
ASV_16s_21389	0.09396249	-0.389917616	3.551493919	0.44385584	0.999627202	NA
ASV_16s_21390	0.09396249	-0.389917616	3.551493919	0.44385584	0.999627202	NA
ASV_16s_21391	0.09396249	-0.389917616	3.551493919	0.44385584	0.999627202	NA
ASV_16s_21392	0.09396249	-0.389917616	3.551493919	0.44385584	0.999627202	NA
ASV_16s_21393	0.09396249	-0.389917616	3.551493919	0.44385584	0.999627202	NA
ASV_16s_21394	0.09396249	-0.389917616	3.551493919	0.44385584	0.999627202	NA
ASV_16s_21395	0.09396249	-0.389917616	3.551493919	0.44385584	0.999627202	NA
ASV_16s_21396	0.09396249	-0.389917616	3.551493919	0.44385584	0.999627202	NA
ASV_16s_21397	0.09396249	-0.389917616	3.551493919	0.44385584	0.999627202	NA
ASV_16s_21398	0.09396249	-0.389917616	3.551493919	0.44385584	0.999627202	NA
ASV_16s_21399	0.09396249	-0.389917616	3.551493919	0.44385584	0.999627202	NA
ASV_16s_21400	0.09396249	-0.389917616	3.551493919	0.44385584	0.999627202	NA
ASV_16s_21401	0.09396249	-0.389917616	3.551493919	0.44385584	0.999627202	NA
ASV_16s_21402	0.143117648	-0.532538362	3.551109109	0.564687342	NA	NA
ASV_16s_21403	0.02993705	-0.141084264	3.55124881	0.11168695	NA	NA
ASV_16s_21404	0.02993705	-0.141084264	3.55124881	0.11168695	NA	NA
ASV_16s_21405	0.032113443	0.024922034	3.551202394	0.178985344	0.999982804	NA
ASV_16s_21406	0.060821091	0.22029232	3.55105156	0.275247342	NA	NA
ASV_16s_21407	0.031566036	0.232521497	3.551352319	0.103068077	NA	NA
ASV_16s_21408	0.041733282	0.226300051	3.551211506	0.166378199	NA	NA
ASV_16s_21414	0.045920146	0.65986383	3.551816201	0.229110032	0.999959978	NA
ASV_16s_21415	0.066486949	0.595405932	3.550962343	0.258360779	NA	NA

ASV_16s_21416	0.096495521	0.01380963	3.551677975	0.514445021	0.999391852	NA
ASV_16s_21417	0.10126134	-0.227763647	3.551557106	0.416259973	0.99969903	NA
ASV_16s_21418	0.104130032	-0.468544992	3.551492709	0.39156416	0.999754702	NA
ASV_16s_21419	0.099533784	-0.209596601	3.55158752	0.376116829	0.999785661	NA
ASV_16s_21420	0.097993749	-0.127978188	3.55177416	0.470865738	0.999546321	NA
ASV_16s_21421	0.100168045	0.535010919	3.551972612	0.407376906	0.999719959	NA
ASV_16s_21422	0.120719738	-0.412235864	3.551473792	0.478029197	0.999523019	NA
ASV_16s_21423	0.124720511	-0.427104292	3.551438425	0.489716224	0.999483272	NA
ASV_16s_21424	0.101435395	0.582012614	3.551978646	0.418408804	0.999693811	NA
ASV_16s_21425	0.116789793	-0.349938158	3.55149998	0.537507414	0.999297226	NA
ASV_16s_21426	0.098959897	0.119123834	3.551691351	0.485145554	0.999499075	NA
ASV_16s_21427	0.119816967	-0.403351827	3.551446566	0.488464654	0.999487632	NA
ASV_16s_21428	0.121456221	-0.417669217	3.551417728	0.49238606	0.999473886	NA
ASV_16s_21429	0.1008318	0.644451824	3.551907299	0.334012841	0.999856219	NA
ASV_16s_21430	0.116326853	0.095065047	3.551796645	0.478563141	0.99952125	NA
ASV_16s_21431	0.131132544	-0.452245152	3.551278689	0.539263566	0.999289636	NA
ASV_16s_21432	0.120809018	-0.191337248	3.551359675	0.703141722	0.998311481	NA
ASV_16s_21433	0.100343769	0.368110671	3.551799172	0.50899157	0.999412887	NA
ASV_16s_21434	0.126984571	-0.421749972	3.551274103	0.539390574	0.999289085	NA
ASV_16s_21435	0.141797047	-0.341858761	3.551561585	0.415363904	0.999701188	NA
ASV_16s_21436	0.097962769	0.01973468	3.551536053	0.432967154	0.999656804	NA
ASV_16s_21437	0.133708554	-0.353254111	3.551650538	0.478101707	0.999522779	NA
ASV_16s_21438	0.102683914	0.212875123	3.551598156	0.517158409	0.999381197	NA
ASV_16s_21439	0.121569495	-0.354346717	3.551261979	0.545855309	0.999260654	NA
ASV_16s_21440	0.120510811	-0.363775313	3.551375128	0.560364333	0.999194064	NA
ASV_16s_21441	0.126226575	-0.346483905	3.551200977	0.572489041	0.9991354	NA
ASV_16s_21442	0.124939413	-0.289711213	3.55149772	0.622833847	0.99886097	NA
ASV_16s_21443	0.098775319	0.252506888	3.5517809	0.368366503	0.999800128	NA
ASV_16s_21444	0.146168643	0.305649009	3.551890964	0.447296356	0.999617497	NA
ASV_16s_21445	0.124661376	-0.282735311	3.551234375	0.575670562	0.999119543	NA
ASV_16s_21446	0.171001571	0.723402292	3.551387252	0.830103251	0.997124465	NA
ASV_16s_21447	0.102310349	0.666215747	3.551695944	0.492170031	0.999474649	NA
ASV_16s_21448	0.095839205	-0.505982111	3.551364565	0.355567934	0.999822522	NA
ASV_16s_21449	0.125226266	0.18414499	3.550269565	0.522083936	0.999361534	NA
ASV_16s_21450	0.121737894	-0.165623278	3.551241903	0.58111431	0.999091957	NA
ASV_16s_21451	0.121867713	-0.165623737	3.551240495	0.581698863	0.999088961	NA
ASV_16s_21452	0.147990416	0.208189803	3.551087664	0.673371955	0.998532073	NA
ASV_16s_21453	0.105292853	0.193066708	3.551346592	0.513758265	0.999394528	NA
ASV_16s_21454	0.123031345	0.333736091	3.551866771	0.445948077	0.99962132	NA
ASV_16s_21455	0.113202535	0.051592211	3.551179678	0.679902835	0.998485433	NA
ASV_16s_21456	0.136952812	0.26501673	3.551075666	0.643959854	0.998730253	NA
ASV_16s_21457	0.104267053	0.680679244	3.551521583	0.515280975	0.999388583	NA
ASV_16s_21458	0.121319242	-0.002375687	3.551194777	0.597017429	0.999008045	NA
ASV_16s_21459	0.113491924	0.095706714	3.551818767	0.440701588	0.99963595	NA
ASV_16s_21460	0.113615572	0.344104413	3.551745644	0.514544541	0.999391463	NA
ASV_16s_21461	0.125091645	0.102092728	3.551161833	0.617148978	0.998894534	NA
ASV_16s_21462	0.117797368	0.213433889	3.550953091	0.546458625	0.999257962	NA
ASV_16s_21463	0.111261629	0.347288183	3.551697506	0.544117289	0.999268372	NA
ASV_16s_21464	0.130444428	0.688176391	3.551176336	0.632392402	0.998803005	NA
ASV_16s_21465	0.123263343	0.985588584	3.551541018	0.464018503	NA	NA
ASV_16s_21466	0.123263343	0.985588584	3.551541018	0.464018503	NA	NA
ASV_16s_21467	0.125595935	1.117081434	3.551565305	0.331467165	0.99985988	NA
ASV_16s_21468	0.129964998	1.006301439	3.551690594	0.361160486	0.999812962	NA
ASV_16s_21469	0.145047581	0.585123159	3.551457899	0.413073297	0.999706657	NA
ASV_16s_21470	0.129780872	0.064477152	3.551186259	0.388975112	0.999760093	NA
ASV_16s_21471	0.142268636	0.321495943	3.55200807	0.371279449	0.999794773	NA
ASV_16s_21472	0.149411299	0.612096543	3.551445018	0.781414696	0.997629028	NA
ASV_16s_21473	0.107395374	1.002280201	3.551518346	0.432891932	0.999657003	NA
ASV_16s_21474	0.107395374	1.002280201	3.551518346	0.432891932	0.999657003	NA
ASV_16s_21475	0.107395374	1.002280201	3.551518346	0.432891932	0.999657003	NA
ASV_16s_21476	0.107395374	1.002280201	3.551518346	0.432891932	0.999657003	NA
ASV_16s_21477	0.107395374	1.002280201	3.551518346	0.432891932	0.999657003	NA
ASV_16s_21478	0.106399963	1.204072998	3.551542877	0.256680778	0.999941063	NA
ASV_16s_21479	0.112319328	1.248914553	3.551475062	0.245903665	0.999949067	NA
ASV_16s_21480	0.114256382	0.712262595	3.551345117	0.549879126	0.999242575	NA
ASV_16s_21481	0.1631644	0.898956354	3.551159071	0.725885183	0.998128845	NA
ASV_16s_21482	0.161760377	1.22464308	3.55130585	0.47409788	0.999535906	NA

ASV_16s_21483	0.101818195	0.333761856	3.551964545	0.451135323	NA	NA
ASV_16s_21484	0.101818195	0.333761856	3.551964545	0.451135323	NA	NA
ASV_16s_21485	0.101818195	0.333761856	3.551964545	0.451135323	NA	NA
ASV_16s_21486	0.101818195	0.333761856	3.551964545	0.451135323	NA	NA
ASV_16s_21487	0.101818195	0.333761856	3.551964545	0.451135323	NA	NA
ASV_16s_21488	0.123263343	0.341073812	3.551905069	0.498552857	NA	NA
ASV_16s_21489	0.123263343	0.341073812	3.551905069	0.498552857	NA	NA
ASV_16s_21490	0.134325181	0.346433668	3.551986823	0.285608182	0.999915306	NA
ASV_16s_21491	0.127909897	0.348744956	3.551925944	0.274350347	0.999926105	NA
ASV_16s_21492	0.118398556	-0.071129105	3.551299243	0.575476657	0.999120515	NA
ASV_16s_21493	0.133229166	0.348059757	3.551804825	0.306967479	0.999891884	NA
ASV_16s_21494	0.13612816	0.347230009	3.551955684	0.284957439	0.999915959	NA
ASV_16s_21495	0.161413815	0.349093714	3.551799608	0.340282779	0.99984692	NA
ASV_16s_21496	0.078903019	0.326345875	3.552020984	0.381702372	0.999774798	NA
ASV_16s_21497	0.078903019	0.326345875	3.552020984	0.381702372	0.999774798	NA
ASV_16s_21498	0.106836448	0.33547338	3.551952689	0.461449933	NA	NA
ASV_16s_21499	0.106836448	0.33547338	3.551952689	0.461449933	NA	NA
ASV_16s_21500	0.106836448	0.33547338	3.551952689	0.461449933	NA	NA
ASV_16s_21501	0.143410176	0.347865682	3.55183769	0.549705001	NA	NA
ASV_16s_21502	0.112360469	-0.148014179	3.551310738	0.347155587	0.999836258	NA
ASV_16s_21503	0.137342113	-0.477844252	3.551232975	0.537949828	NA	NA
ASV_16s_21504	0.148045254	-0.67869258	3.551316435	0.322002276	0.999872927	NA
ASV_16s_21505	0.132020419	-0.467097618	3.5512747	0.5247867	NA	NA
ASV_16s_21506	0.132020419	-0.467097618	3.5512747	0.5247867	NA	NA
ASV_16s_21507	0.132020419	-0.467097618	3.5512747	0.5247867	NA	NA
ASV_16s_21508	0.132020419	-0.467097618	3.5512747	0.5247867	NA	NA
ASV_16s_21509	0.132020419	-0.467097618	3.5512747	0.5247867	NA	NA
ASV_16s_21510	0.109170334	-0.626343418	3.551431085	0.292505202	0.999908174	NA
ASV_16s_21511	0.116897992	-0.489924314	3.551265049	0.405259911	0.999724795	NA
ASV_16s_21512	0.134019113	-0.710759448	3.551174579	0.291087297	0.999909673	NA
ASV_16s_21513	0.15767717	-0.509318576	3.551043501	0.590947042	NA	NA
ASV_16s_21514	0.128909118	0.492066043	3.551190116	0.433839689	0.999654494	NA
ASV_16s_21515	0.113436745	-0.422858897	3.551429532	0.481937764	NA	NA
ASV_16s_21516	0.113436745	-0.422858897	3.551429532	0.481937764	NA	NA
ASV_16s_21517	0.07880133	-0.314721819	3.551726259	0.388587504	0.999760893	NA
ASV_16s_21518	0.070185657	-0.463735283	3.551650758	0.190128258	0.999978847	NA
ASV_16s_21519	0.115924029	-0.429236443	3.551407072	0.487323271	NA	NA
ASV_16s_21520	0.115375057	1.19048432	3.55141378	0.270894958	0.999929217	NA
ASV_16s_21521	0.216983199	1.236357032	3.550935291	0.701228581	NA	NA
ASV_16s_21522	0.116832953	1.073177941	3.551205797	0.397204422	NA	NA
ASV_16s_21523	0.164020687	1.18196373	3.55095218	0.501478008	NA	NA
ASV_16s_21524	0.104533567	1.036756323	3.551294234	0.367991553	0.999800811	NA
ASV_16s_21525	0.104533567	1.036756323	3.551294234	0.367991553	0.999800811	NA
ASV_16s_21526	0.132133151	0.36165678	3.551552846	0.457366169	NA	NA
ASV_16s_21527	0.119865209	0.356021636	3.551605909	0.428266631	NA	NA
ASV_16s_21528	0.108832697	0.350334673	3.551657577	0.404449458	NA	NA
ASV_16s_21529	0.108832697	0.350334673	3.551657577	0.404449458	NA	NA
ASV_16s_21530	0.108832697	0.350334673	3.551657577	0.404449458	NA	NA
ASV_16s_21531	0.089840107	0.345204782	3.55172579	0.356866458	NA	NA
ASV_16s_21532	0.089840107	0.345204782	3.55172579	0.356866458	NA	NA
ASV_16s_21533	0.097203811	0.346461715	3.551702905	0.378388817	NA	NA
ASV_16s_21534	0.097203811	0.346461715	3.551702905	0.378388817	NA	NA
ASV_16s_21535	0.132815163	-0.0706739	3.551188138	0.548361078	0.99924943	NA
ASV_16s_21536	0.134614911	0.923037725	3.551118115	0.60754478	0.998949718	NA
ASV_16s_21537	0.136363614	0.363682531	3.551530622	0.467836448	NA	NA
ASV_16s_21538	0.136363614	0.363682531	3.551530622	0.467836448	NA	NA
ASV_16s_21539	0.129315334	-0.474885822	3.550982143	0.451879263	0.999604303	NA
ASV_16s_21540	0.054613154	-0.235902923	3.551587642	0.24101681	0.999952432	NA
ASV_16s_21541	0.133970713	-0.48329616	3.550933459	0.462986639	NA	NA
ASV_16s_21542	0.133970713	-0.48329616	3.550933459	0.462986639	NA	NA
ASV_16s_21543	0.133970713	-0.48329616	3.550933459	0.462986639	NA	NA
ASV_16s_21544	0.152060941	0.178304927	3.550545417	0.756605774	0.997862001	NA
ASV_16s_21545	0.145315244	0.255021334	3.550633421	0.721993643	0.998160982	NA
ASV_16s_21546	0.165008376	-0.523931517	3.550639026	0.543335741	NA	NA
ASV_16s_21547	0.06078674	-0.265780772	3.551540269	0.263878566	0.999935253	NA
ASV_16s_21548	0.119887879	1.081660494	3.551186044	0.403066513	NA	NA
ASV_16s_21549	0.119887879	1.081660494	3.551186044	0.403066513	NA	NA

ASV_16s_21550	0.119887879	1.081660494	3.551186044	0.403066513	NA	NA
ASV_16s_21551	0.119225523	1.079838455	3.551190258	0.401781895	NA	NA
ASV_16s_21552	0.119225523	1.079838455	3.551190258	0.401781895	NA	NA
ASV_16s_21553	0.108832697	-0.077947704	3.551015968	0.646158513	NA	NA
ASV_16s_21554	0.108832697	-0.077947704	3.551015968	0.646158513	NA	NA
ASV_16s_21555	0.108832697	-0.077947704	3.551015968	0.646158513	NA	NA
ASV_16s_21556	0.108832697	-0.077947704	3.551015968	0.646158513	NA	NA
ASV_16s_21557	0.108832697	-0.077947704	3.551015968	0.646158513	NA	NA
ASV_16s_21558	0.108832697	-0.077947704	3.551015968	0.646158513	NA	NA
ASV_16s_21559	0.108832697	-0.077947704	3.551015968	0.646158513	NA	NA
ASV_16s_21560	0.108832697	-0.077947704	3.551015968	0.646158513	NA	NA
ASV_16s_21561	0.108832697	-0.077947704	3.551015968	0.646158513	NA	NA
ASV_16s_21562	0.108832697	-0.077947704	3.551015968	0.646158513	NA	NA
ASV_16s_21563	0.108832697	-0.077947704	3.551015968	0.646158513	NA	NA
ASV_16s_21564	0.108832697	-0.077947704	3.551015968	0.646158513	NA	NA
ASV_16s_21565	0.106679816	-0.077909379	3.55102601	0.639170319	0.998760725	NA
ASV_16s_21566	0.092802879	-0.07766094	3.551101816	0.58647693	NA	NA
ASV_16s_21567	0.092802879	-0.07766094	3.551101816	0.58647693	NA	NA
ASV_16s_21568	0.092802879	-0.07766094	3.551101816	0.58647693	NA	NA
ASV_16s_21569	0.092802879	-0.07766094	3.551101816	0.58647693	NA	NA
ASV_16s_21570	0.092802879	-0.07766094	3.551101816	0.58647693	NA	NA
ASV_16s_21571	0.092802879	-0.07766094	3.551101816	0.58647693	NA	NA
ASV_16s_21572	0.092802879	-0.07766094	3.551101816	0.58647693	NA	NA
ASV_16s_21573	0.092802879	-0.07766094	3.551101816	0.58647693	NA	NA
ASV_16s_21574	0.092802879	-0.07766094	3.551101816	0.58647693	NA	NA
ASV_16s_21575	0.092802879	-0.07766094	3.551101816	0.58647693	NA	NA
ASV_16s_21576	0.092802879	-0.07766094	3.551101816	0.58647693	NA	NA
ASV_16s_21577	0.092802879	-0.07766094	3.551101816	0.58647693	NA	NA
ASV_16s_21578	0.092802879	-0.07766094	3.551101816	0.58647693	NA	NA
ASV_16s_21579	0.104706342	-0.077882873	3.551035576	0.632820002	NA	NA
ASV_16s_21580	0.104706342	-0.077882873	3.551035576	0.632820002	NA	NA
ASV_16s_21581	0.064709103	0.220270885	3.551023237	0.295633208	0.999904807	NA
ASV_16s_21582	0.065416014	0.220267262	3.551018445	0.299286077	0.999900766	NA
ASV_16s_21583	0.069088535	0.22024826	3.550995086	0.317950553	NA	NA
ASV_16s_21584	0.069088535	0.22024826	3.550995086	0.317950553	NA	NA
ASV_16s_21585	0.069088535	0.22024826	3.550995086	0.317950553	NA	NA
ASV_16s_21586	0.069088535	0.22024826	3.550995086	0.317950553	NA	NA
ASV_16s_21587	0.06895057	0.220248867	3.550995917	0.317259032	NA	NA
ASV_16s_21588	0.06895057	0.220248867	3.550995917	0.317259032	NA	NA
ASV_16s_21589	0.06895057	0.220248867	3.550995917	0.317259032	NA	NA
ASV_16s_21590	0.11776954	0.310956829	3.551142535	0.657149571	0.998643753	NA
ASV_16s_21591	0.118694451	0.310956554	3.55113825	0.660274978	0.998622695	NA
ASV_16s_21592	0.117688403	0.310956854	3.551142914	0.656875814	0.998645588	NA
ASV_16s_21593	0.119906018	0.31095631	3.551132737	0.664383307	NA	NA
ASV_16s_21594	0.119906018	0.31095631	3.551132737	0.664383307	NA	NA
ASV_16s_21595	0.10281704	0.310962171	3.55122242	0.608004916	NA	NA
ASV_16s_21596	0.110499814	0.31095927	3.551178708	0.632901845	0.998799861	NA
ASV_16s_21597	0.117542357	0.31095701	3.551143601	0.656383466	NA	NA
ASV_16s_21598	0.117542357	0.31095701	3.551143601	0.656383466	NA	NA
ASV_16s_21599	0.117542357	0.31095701	3.551143601	0.656383466	NA	NA
ASV_16s_21600	0.072779319	0.595486532	3.550926909	0.287168088	NA	NA
ASV_16s_21601	0.122980342	0.68719949	3.55109319	0.620461845	NA	NA
ASV_16s_21602	0.095783928	-0.091652976	3.551357845	0.405077621	0.999725208	NA
ASV_16s_21603	0.097758062	-0.056687632	3.551354891	0.623535955	0.998856778	NA
ASV_16s_21604	0.097758062	-0.056687632	3.551354891	0.623535955	0.998856778	NA
ASV_16s_21605	0.117970058	1.171163371	3.551532942	0.280408042	0.999920422	NA
ASV_16s_21606	0.150791703	0.903597222	3.551513356	0.465758508	0.999562452	NA
ASV_16s_21607	0.09825358	-0.05252177	3.551400172	0.626982877	0.998836046	NA
ASV_16s_21608	0.105697906	-0.05590906	3.551327755	0.659433227	0.998628388	NA
ASV_16s_21609	0.098749099	-0.048619023	3.551449999	0.628953789	0.99882408	NA
ASV_16s_21610	0.13017063	0.530452889	3.551671171	0.323353153	0.999871119	NA
ASV_16s_21611	0.105005521	0.155162642	3.551610852	0.625808793	0.998843136	NA
ASV_16s_21612	0.091898953	0.286293333	3.551860898	0.316023805	0.99988072	NA
ASV_16s_21613	0.127492495	0.995921947	3.551510877	0.472543127	NA	NA
ASV_16s_21614	0.142120969	0.294278877	3.551801626	0.545140707	NA	NA
ASV_16s_21615	0.173409404	-0.592033966	3.550918985	0.649958331	NA	NA
ASV_16s_21616	0.155856495	0.972025833	3.551364474	0.517264297	0.999380779	NA

ASV_16s_21617	0.081835251	0.286418338	3.551804238	0.394104182	0.999749334	NA
ASV_16s_21618	0.166076757	0.27953204	3.551617142	0.625016869	NA	NA
ASV_16s_21619	0.131043687	0.281901826	3.551674457	0.526025618	0.999345496	NA
ASV_16s_21620	0.207763142	-0.538967226	3.551096862	0.741177078	NA	NA
ASV_16s_21621	0.207763142	-0.538967226	3.551096862	0.741177078	NA	NA
ASV_16s_21622	0.211973413	-0.552012992	3.55056106	0.749258424	NA	NA
ASV_16s_21623	0.211973413	-0.552012992	3.55056106	0.749258424	NA	NA
ASV_16s_21624	0.129366116	0.321591409	3.551443904	0.464527083	0.999566282	NA
ASV_16s_21625	0.123263343	-0.462758841	3.55104304	0.437924717	NA	NA
ASV_16s_21626	0.136675749	1.124940975	3.551090245	0.437875665	NA	NA
ASV_16s_21627	0.136675749	1.124940975	3.551090245	0.437875665	NA	NA
ASV_16s_21628	0.136675749	1.124940975	3.551090245	0.437875665	NA	NA
ASV_16s_21629	0.136675749	1.124940975	3.551090245	0.437875665	NA	NA
ASV_16s_21630	0.128201777	1.053011804	3.551287027	0.449904379	0.999610026	NA
ASV_16s_21631	0.150220939	1.067021792	3.551158011	0.557855686	0.999205856	NA
ASV_16s_21632	0.117306669	0.686997727	3.551350319	0.532466197	0.999318706	NA
ASV_16s_21633	0.077882296	-0.077288597	3.551213122	0.518605897	NA	NA
ASV_16s_21634	0.077882296	-0.077288597	3.551213122	0.518605897	NA	NA
ASV_16s_21635	0.077882296	-0.077288597	3.551213122	0.518605897	NA	NA
ASV_16s_21636	0.085956314	-0.080765062	3.551379439	0.533312357	0.999315132	NA
ASV_16s_21637	0.242265708	-0.028976466	3.550855186	1.202065342	NA	NA
ASV_16s_21638	0.188058427	0.341092626	3.550477593	1.03097933	0.994313197	NA
ASV_16s_21639	0.095629312	-0.167939812	3.55128308	0.510392576	0.999407531	NA
ASV_16s_21640	0.215521574	-0.028851835	3.550896647	1.096408263	NA	NA
ASV_16s_21641	0.168774691	0.261077406	3.55101118	0.931348655	0.995861285	NA
ASV_16s_21642	0.105397349	-0.024581193	3.551502278	0.681662292	0.998472702	NA
ASV_16s_21643	0.102754773	-0.023927804	3.551297977	0.685164326	NA	NA
ASV_16s_21644	0.102754773	-0.023927804	3.551297977	0.685164326	NA	NA
ASV_16s_21645	0.102754773	-0.023927804	3.551297977	0.685164326	NA	NA
ASV_16s_21646	0.102754773	-0.023927804	3.551297977	0.685164326	NA	NA
ASV_16s_21647	0.102754773	-0.023927804	3.551297977	0.685164326	NA	NA
ASV_16s_21648	0.102754773	-0.023927804	3.551297977	0.685164326	NA	NA
ASV_16s_21649	0.117410406	-0.072889592	3.551025889	0.698697982	NA	NA
ASV_16s_21650	0.117410406	-0.072889592	3.551025889	0.698697982	NA	NA
ASV_16s_21651	0.085751818	-0.072232587	3.551212914	0.579306097	NA	NA
ASV_16s_21652	0.085751818	-0.072232587	3.551212914	0.579306097	NA	NA
ASV_16s_21653	0.085751818	-0.072232587	3.551212914	0.579306097	NA	NA
ASV_16s_21654	0.085751818	-0.072232587	3.551212914	0.579306097	NA	NA
ASV_16s_21655	0.10347109	0.245380611	3.551226517	0.644311327	0.998727997	NA
ASV_16s_21656	0.084211001	-0.072188224	3.551225563	0.572284676	NA	NA
ASV_16s_21657	0.084211001	-0.072188224	3.551225563	0.572284676	NA	NA
ASV_16s_21658	0.084211001	-0.072188224	3.551225563	0.572284676	NA	NA
ASV_16s_21659	0.084211001	-0.072188224	3.551225563	0.572284676	NA	NA
ASV_16s_21660	0.084211001	-0.072188224	3.551225563	0.572284676	NA	NA
ASV_16s_21661	0.084211001	-0.072188224	3.551225563	0.572284676	NA	NA
ASV_16s_21662	0.084211001	-0.072188224	3.551225563	0.572284676	NA	NA
ASV_16s_21663	0.084211001	-0.072188224	3.551225563	0.572284676	NA	NA
ASV_16s_21664	0.142120969	-0.073200334	3.550937404	0.784596284	NA	NA
ASV_16s_21665	0.142120969	-0.073200334	3.550937404	0.784596284	NA	NA
ASV_16s_21666	0.104485706	-0.072668754	3.551088682	0.656241599	NA	NA
ASV_16s_21667	0.104485706	-0.072668754	3.551088682	0.656241599	NA	NA
ASV_16s_21668	0.104485706	-0.072668754	3.551088682	0.656241599	NA	NA
ASV_16s_21669	0.104485706	-0.072668754	3.551088682	0.656241599	NA	NA
ASV_16s_21670	0.104485706	-0.072668754	3.551088682	0.656241599	NA	NA
ASV_16s_21671	0.104485706	-0.072668754	3.551088682	0.656241599	NA	NA
ASV_16s_21672	0.104485706	-0.072668754	3.551088682	0.656241599	NA	NA
ASV_16s_21673	0.117214679	0.360211533	3.551456122	0.716850609	NA	NA
ASV_16s_21674	0.117214679	0.360211533	3.551456122	0.716850609	NA	NA
ASV_16s_21675	0.117214679	0.360211533	3.551456122	0.716850609	NA	NA
ASV_16s_21676	0.1041998	0.358601892	3.55154715	0.673800625	NA	NA
ASV_16s_21677	0.1041998	0.358601892	3.55154715	0.673800625	NA	NA
ASV_16s_21678	0.1041998	0.358601892	3.55154715	0.673800625	NA	NA
ASV_16s_21679	0.1041998	0.358601892	3.55154715	0.673800625	NA	NA
ASV_16s_21680	0.1041998	0.358601892	3.55154715	0.673800625	NA	NA
ASV_16s_21681	0.1041998	0.358601892	3.55154715	0.673800625	NA	NA
ASV_16s_21682	0.1041998	0.358601892	3.55154715	0.673800625	NA	NA
ASV_16s_21683	0.1041998	0.358601892	3.55154715	0.673800625	NA	NA

ASV_16s_21684	0.1041998	0.358601892	3.55154715	0.673800625	NA	NA
ASV_16s_21685	0.1041998	0.358601892	3.55154715	0.673800625	NA	NA
ASV_16s_21686	0.1041998	0.358601892	3.55154715	0.673800625	NA	NA
ASV_16s_21687	0.1041998	0.358601892	3.55154715	0.673800625	NA	NA
ASV_16s_21688	0.139544984	0.805359021	3.551495146	0.760043428	0.997830665	NA
ASV_16s_21689	0.135011116	0.315940006	3.55111778	0.739563069	NA	NA
ASV_16s_21690	0.135011116	0.315940006	3.55111778	0.739563069	NA	NA
ASV_16s_21691	0.135011116	0.315940006	3.55111778	0.739563069	NA	NA
ASV_16s_21692	0.135011116	0.315940006	3.55111778	0.739563069	NA	NA
ASV_16s_21693	0.135011116	0.315940006	3.55111778	0.739563069	NA	NA
ASV_16s_21694	0.135011116	0.315940006	3.55111778	0.739563069	NA	NA
ASV_16s_21695	0.135011116	0.315940006	3.55111778	0.739563069	NA	NA
ASV_16s_21696	0.117938234	0.31594011	3.551193869	0.680461291	0.9984814	NA
ASV_16s_21697	0.11566185	0.315940126	3.551205688	0.672806928	0.998536062	NA
ASV_16s_21698	0.130251631	0.315939916	3.551137085	0.722646873	0.998155613	NA
ASV_16s_21699	0.116703196	0.315940208	3.551200205	0.676332991	NA	NA
ASV_16s_21700	0.116703196	0.315940208	3.551200205	0.676332991	NA	NA
ASV_16s_21701	0.116703196	0.315940208	3.551200205	0.676332991	NA	NA
ASV_16s_21702	0.116703196	0.315940208	3.551200205	0.676332991	NA	NA
ASV_16s_21703	0.116703196	0.315940208	3.551200205	0.676332991	NA	NA
ASV_16s_21704	0.120949923	0.315939959	3.551178972	0.690562552	0.998407203	NA
ASV_16s_21705	0.181181373	0.315939715	3.550983421	0.909678743	NA	NA
ASV_16s_21706	0.146579832	0.315939846	3.551076327	0.780668569	0.997636268	NA
ASV_16s_21707	0.108832697	0.31594028	3.551244073	0.650211777	NA	NA
ASV_16s_21708	0.108832697	0.31594028	3.551244073	0.650211777	NA	NA
ASV_16s_21709	0.108832697	0.31594028	3.551244073	0.650211777	NA	NA
ASV_16s_21710	0.108832697	0.31594028	3.551244073	0.650211777	NA	NA
ASV_16s_21711	0.108832697	0.31594028	3.551244073	0.650211777	NA	NA
ASV_16s_21712	0.108832697	0.31594028	3.551244073	0.650211777	NA	NA
ASV_16s_21713	0.108832697	0.31594028	3.551244073	0.650211777	NA	NA
ASV_16s_21714	0.108832697	0.31594028	3.551244073	0.650211777	NA	NA
ASV_16s_21715	0.108832697	0.31594028	3.551244073	0.650211777	NA	NA
ASV_16s_21716	0.108832697	0.31594028	3.551244073	0.650211777	NA	NA
ASV_16s_21717	0.108832697	0.31594028	3.551244073	0.650211777	NA	NA
ASV_16s_21718	0.114270715	0.408031295	3.551158679	0.818247415	0.997253242	NA
ASV_16s_21719	0.125638587	0.693845851	3.551430221	0.728279206	0.998108892	NA
ASV_16s_21720	0.1226127	0.499977539	3.551107109	0.910787238	0.996142222	NA
ASV_16s_21721	0.140028012	0.655885789	3.551064317	0.780128273	0.997641501	NA
ASV_16s_21722	0.103645408	0.686889468	3.5511927	0.55727442	NA	NA
ASV_16s_21723	0.187242679	0.687772435	3.550909292	0.824865769	NA	NA
ASV_16s_21724	0.187242679	0.687772435	3.550909292	0.824865769	NA	NA
ASV_16s_21725	0.187242679	0.687772435	3.550909292	0.824865769	NA	NA
ASV_16s_21726	0.177574607	1.102834299	3.551097608	0.618886213	0.998884349	NA
ASV_16s_21727	0.159022857	1.080582825	3.551265115	0.575768071	0.999119054	NA
ASV_16s_21728	0.186801338	0.99996649	3.551288657	0.723360713	0.998149734	NA
ASV_16s_21729	0.170137629	0.733434625	3.551206514	0.833306307	NA	NA
ASV_16s_21730	0.170137629	0.733434625	3.551206514	0.833306307	NA	NA
ASV_16s_21731	0.151772474	1.041235548	3.551255089	0.571295874	0.999141297	NA
ASV_16s_21732	0.152368177	1.000090965	3.551194837	0.580095213	0.999097165	NA
ASV_16s_21733	0.170137629	0.733434625	3.551206514	0.833306307	NA	NA
ASV_16s_21734	0.170137629	0.733434625	3.551206514	0.833306307	NA	NA
ASV_16s_21735	0.150798027	0.73278204	3.551285724	0.770812794	0.997730534	NA
ASV_16s_21736	0.143972285	0.7316484	3.551316704	0.749228794	NA	NA
ASV_16s_21737	0.143972285	0.7316484	3.551316704	0.749228794	NA	NA
ASV_16s_21738	0.143972285	0.7316484	3.551316704	0.749228794	NA	NA
ASV_16s_21739	0.143972285	0.7316484	3.551316704	0.749228794	NA	NA
ASV_16s_21740	0.143972285	0.7316484	3.551316704	0.749228794	NA	NA
ASV_16s_21741	0.143972285	0.7316484	3.551316704	0.749228794	NA	NA
ASV_16s_21742	0.143972285	0.7316484	3.551316704	0.749228794	NA	NA
ASV_16s_21743	0.143972285	0.7316484	3.551316704	0.749228794	NA	NA
ASV_16s_21744	0.143972285	0.7316484	3.551316704	0.749228794	NA	NA
ASV_16s_21745	0.143972285	0.7316484	3.551316704	0.749228794	NA	NA
ASV_16s_21746	0.143972285	0.7316484	3.551316704	0.749228794	NA	NA
ASV_16s_21747	0.143972285	0.7316484	3.551316704	0.749228794	NA	NA
ASV_16s_21748	0.143972285	0.7316484	3.551316704	0.749228794	NA	NA
ASV_16s_21749	0.143972285	0.7316484	3.551316704	0.749228794	NA	NA
ASV_16s_21750	0.143972285	0.7316484	3.551316704	0.749228794	NA	NA

ASV_16s_21751	0.143972285	0.7316484	3.551316704	0.749228794	NA	NA
ASV_16s_21752	0.143972285	0.7316484	3.551316704	0.749228794	NA	NA
ASV_16s_21753	0.143972285	0.7316484	3.551316704	0.749228794	NA	NA
ASV_16s_21754	0.143972285	0.7316484	3.551316704	0.749228794	NA	NA
ASV_16s_21755	0.215242196	0.73388501	3.551075343	0.985493754	NA	NA
ASV_16s_21756	0.168977196	1.040907553	3.551304141	0.641608644	0.998745274	NA
ASV_16s_21757	0.071317487	0.667466641	3.551712286	0.482226896	0.999508992	NA
ASV_16s_21758	0.071317487	0.667466641	3.551712286	0.482226896	0.999508992	NA
ASV_16s_21759	0.071317487	0.667466641	3.551712286	0.482226896	0.999508992	NA
ASV_16s_21760	0.071317487	0.667466641	3.551712286	0.482226896	0.999508992	NA
ASV_16s_21761	0.071317487	0.667466641	3.551712286	0.482226896	0.999508992	NA
ASV_16s_21762	0.071317487	0.667466641	3.551712286	0.482226896	0.999508992	NA
ASV_16s_21763	0.071317487	0.667466641	3.551712286	0.482226896	0.999508992	NA
ASV_16s_21764	0.071317487	0.667466641	3.551712286	0.482226896	0.999508992	NA
ASV_16s_21765	0.13384758	0.692295569	3.551104767	0.677627327	NA	NA
ASV_16s_21766	0.13384758	0.692295569	3.551104767	0.677627327	NA	NA
ASV_16s_21767	0.13384758	0.692295569	3.551104767	0.677627327	NA	NA
ASV_16s_21768	0.13384758	0.692295569	3.551104767	0.677627327	NA	NA
ASV_16s_21769	0.13384758	0.692295569	3.551104767	0.677627327	NA	NA
ASV_16s_21770	0.135498819	0.692317271	3.551098093	0.682667303	0.998465397	NA
ASV_16s_21771	0.135663943	0.692319507	3.551097434	0.683172576	0.998461716	NA
ASV_16s_21772	0.135829067	0.692321737	3.551096778	0.683678077	0.998458028	NA
ASV_16s_21773	0.135829067	0.692321737	3.551096778	0.683678077	0.998458028	NA
ASV_16s_21774	0.137645431	0.692346828	3.551089656	0.689253578	NA	NA
ASV_16s_21775	0.137645431	0.692346828	3.551089656	0.689253578	NA	NA
ASV_16s_21776	0.137645431	0.692346828	3.551089656	0.689253578	NA	NA
ASV_16s_21777	0.137645431	0.692346828	3.551089656	0.689253578	NA	NA
ASV_16s_21778	0.137645431	0.692346828	3.551089656	0.689253578	NA	NA
ASV_16s_21779	0.137645431	0.692346828	3.551089656	0.689253578	NA	NA
ASV_16s_21780	0.137645431	0.692346828	3.551089656	0.689253578	NA	NA
ASV_16s_21781	0.137645431	0.692346828	3.551089656	0.689253578	NA	NA
ASV_16s_21782	0.137645431	0.692346828	3.551089656	0.689253578	NA	NA
ASV_16s_21783	0.137645431	0.692346828	3.551089656	0.689253578	NA	NA
ASV_16s_21784	0.131381793	0.692260674	3.551115056	0.669538531	0.998558997	NA
ASV_16s_21785	0.126370883	0.692185713	3.551137167	0.653962166	0.998665007	NA
ASV_16s_21786	0.118854517	0.692061649	3.551173783	0.63099586	0.998811594	NA
ASV_16s_21787	0.108832697	0.691870137	3.55123035	0.601276573	NA	NA
ASV_16s_21788	0.108832697	0.691870137	3.55123035	0.601276573	NA	NA
ASV_16s_21789	0.108832697	0.691870137	3.55123035	0.601276573	NA	NA
ASV_16s_21790	0.108832697	0.691870137	3.55123035	0.601276573	NA	NA
ASV_16s_21791	0.108832697	0.691870137	3.55123035	0.601276573	NA	NA
ASV_16s_21792	0.108832697	0.691870137	3.55123035	0.601276573	NA	NA
ASV_16s_21793	0.108832697	0.691870137	3.55123035	0.601276573	NA	NA
ASV_16s_21794	0.108832697	0.691870137	3.55123035	0.601276573	NA	NA
ASV_16s_21795	0.108832697	0.691870137	3.55123035	0.601276573	NA	NA
ASV_16s_21796	0.108832697	0.691870137	3.55123035	0.601276573	NA	NA
ASV_16s_21797	0.108832697	0.691870137	3.55123035	0.601276573	NA	NA
ASV_16s_21798	0.108832697	0.691870137	3.55123035	0.601276573	NA	NA
ASV_16s_21799	0.108832697	0.691870137	3.55123035	0.601276573	NA	NA
ASV_16s_21800	0.126588623	0.692188928	3.551136219	0.654108392	0.998664037	NA
ASV_16s_21801	0.159880984	0.692598723	3.551015483	0.759386801	NA	NA
ASV_16s_21802	0.159742621	0.692596531	3.55101588	0.758940683	0.99784075	NA
ASV_16s_21803	0.159591679	0.69259589	3.551016316	0.758454283	NA	NA
ASV_16s_21804	0.129063774	0.030757859	3.551532812	0.709418301	0.99826232	NA
ASV_16s_21805	0.144271939	0.585092474	3.550864222	0.46597413	0.999561779	NA
ASV_16s_21806	0.089751675	0.300935949	3.551943873	0.415769573	0.999700213	NA
ASV_16s_21807	0.089751675	0.300935949	3.551943873	0.415769573	0.999700213	NA
ASV_16s_21808	0.089751675	0.300935949	3.551943873	0.415769573	0.999700213	NA
ASV_16s_21809	0.089751675	0.300935949	3.551943873	0.415769573	0.999700213	NA
ASV_16s_21810	0.089751675	0.300935949	3.551943873	0.415769573	0.999700213	NA
ASV_16s_21811	0.089751675	0.300935949	3.551943873	0.415769573	0.999700213	NA
ASV_16s_21812	0.089751675	0.300935949	3.551943873	0.415769573	0.999700213	NA
ASV_16s_21813	0.089751675	0.300935949	3.551943873	0.415769573	0.999700213	NA
ASV_16s_21814	0.089751675	0.300935949	3.551943873	0.415769573	0.999700213	NA
ASV_16s_21815	0.089751675	0.300935949	3.551943873	0.415769573	0.999700213	NA
ASV_16s_21816	0.089751675	0.300935949	3.551943873	0.415769573	0.999700213	NA
ASV_16s_21817	0.089751675	0.300935949	3.551943873	0.415769573	0.999700213	NA

ASV_16s_21818	0.089751675	0.300935949	3.551943873	0.415769573	0.999700213	NA
ASV_16s_21819	0.089751675	0.300935949	3.551943873	0.415769573	0.999700213	NA
ASV_16s_21820	0.089751675	0.300935949	3.551943873	0.415769573	0.999700213	NA
ASV_16s_21821	0.089751675	0.300935949	3.551943873	0.415769573	0.999700213	NA
ASV_16s_21822	0.089751675	0.300935949	3.551943873	0.415769573	0.999700213	NA
ASV_16s_21823	0.089751675	0.300935949	3.551943873	0.415769573	0.999700213	NA
ASV_16s_21824	0.089751675	0.300935949	3.551943873	0.415769573	0.999700213	NA
ASV_16s_21825	0.089751675	0.300935949	3.551943873	0.415769573	0.999700213	NA
ASV_16s_21826	0.089751675	0.300935949	3.551943873	0.415769573	0.999700213	NA
ASV_16s_21827	0.089751675	0.300935949	3.551943873	0.415769573	0.999700213	NA
ASV_16s_21828	0.089751675	0.300935949	3.551943873	0.415769573	0.999700213	NA
ASV_16s_21829	0.089751675	0.300935949	3.551943873	0.415769573	0.999700213	NA
ASV_16s_21830	0.089751675	0.300935949	3.551943873	0.415769573	0.999700213	NA
ASV_16s_21831	0.089751675	0.300935949	3.551943873	0.415769573	0.999700213	NA
ASV_16s_21832	0.089751675	0.300935949	3.551943873	0.415769573	0.999700213	NA
ASV_16s_21833	0.089751675	0.300935949	3.551943873	0.415769573	0.999700213	NA
ASV_16s_21834	0.089751675	0.300935949	3.551943873	0.415769573	0.999700213	NA
ASV_16s_21835	0.089751675	0.300935949	3.551943873	0.415769573	0.999700213	NA
ASV_16s_21836	0.089751675	0.300935949	3.551943873	0.415769573	0.999700213	NA
ASV_16s_21837	0.096180329	0.148125164	3.551843916	0.519688487	0.999371149	NA
ASV_16s_21838	0.089780195	0.100678547	3.551820921	0.518235507	0.999376933	NA
ASV_16s_21839	0.097504431	0.046924854	3.551743618	0.557226686	0.999208794	NA
ASV_16s_21840	0.089785899	0.068054594	3.551796583	0.531865157	0.999321237	NA
ASV_16s_21841	0.091305188	0.057651291	3.5517827	0.539092689	0.999290377	NA
ASV_16s_21842	0.091564107	0.02545352	3.551758117	0.551371152	0.999235796	NA
ASV_16s_21843	0.091823025	-0.006353388	3.551726424	0.561262849	0.999189812	NA
ASV_16s_21844	0.091823025	-0.006353388	3.551726424	0.561262849	0.999189812	NA
ASV_16s_21845	0.091823025	-0.006353388	3.551726424	0.561262849	0.999189812	NA
ASV_16s_21846	0.091823025	-0.006353388	3.551726424	0.561262849	0.999189812	NA
ASV_16s_21847	0.102156085	-0.067642374	3.551600584	0.611621759	0.998926524	NA
ASV_16s_21848	0.092081944	-0.037073794	3.551695582	0.568812308	0.999153484	NA
ASV_16s_21849	0.0913223	-0.032464738	3.55170469	0.565413027	0.999169975	NA
ASV_16s_21850	0.092081944	-0.037073794	3.551695582	0.568812308	0.999153484	NA
ASV_16s_21851	0.092081944	-0.037073794	3.551695582	0.568812308	0.999153484	NA
ASV_16s_21852	0.089808715	-0.051972458	3.551697213	0.56301156	0.999181493	NA
ASV_16s_21853	0.092340863	-0.066628591	3.551668395	0.573842203	0.999128679	NA
ASV_16s_21854	0.092340863	-0.066628591	3.551668395	0.573842203	0.999128679	NA
ASV_16s_21855	0.092340863	-0.066628591	3.551668395	0.573842203	0.999128679	NA
ASV_16s_21856	0.089814419	-0.080020963	3.551674203	0.564862269	0.999172626	NA
ASV_16s_21857	0.092599782	-0.095424482	3.551642551	0.576372163	0.99911602	NA
ASV_16s_21858	0.089814419	-0.080020963	3.551674203	0.564862269	0.999172626	NA
ASV_16s_21859	0.092599782	-0.095424482	3.551642551	0.576372163	0.99911602	NA
ASV_16s_21860	0.092599782	-0.095424482	3.551642551	0.576372163	0.99911602	NA
ASV_16s_21861	0.089814419	-0.080020963	3.551674203	0.564862269	0.999172626	NA
ASV_16s_21862	0.089820123	-0.107415216	3.551653279	0.564274305	0.99917545	NA
ASV_16s_21863	0.092858701	-0.123600648	3.551617749	0.576360349	0.999116079	NA
ASV_16s_21864	0.089820123	-0.107415216	3.551653279	0.564274305	0.99917545	NA
ASV_16s_21865	0.098510599	-0.15184716	3.5515577	0.599728548	0.998993238	NA
ASV_16s_21866	0.096232873	-0.166123066	3.551563877	0.586118661	0.99906609	NA
ASV_16s_21867	0.089825827	-0.134445462	3.551632349	0.561326423	0.99918951	NA
ASV_16s_21868	0.09311762	-0.151094847	3.551596039	0.573896843	0.999128407	NA
ASV_16s_21869	0.09311762	-0.151094847	3.551596039	0.573896843	0.999128407	NA
ASV_16s_21870	0.089825827	-0.134445462	3.551632349	0.561326423	0.99918951	NA
ASV_16s_21871	0.093376538	-0.178019667	3.551576989	0.568956368	0.99915278	NA
ASV_16s_21872	0.093376538	-0.178019667	3.551576989	0.568956368	0.99915278	NA
ASV_16s_21873	0.089831532	-0.160932274	3.551613953	0.556006308	0.999214474	NA
ASV_16s_21874	0.093635457	-0.20460834	3.551559644	0.561471279	0.999188823	NA
ASV_16s_21875	0.08984294	-0.213317985	3.551582723	0.538071502	0.999294794	NA
ASV_16s_21876	0.093140436	-0.253639866	3.551537051	0.535693546	0.999305006	NA
ASV_16s_21877	0.032085143	-0.072150761	3.551897667	0.202705581	0.99997366	NA
ASV_16s_21878	0.032085143	-0.072150761	3.551897667	0.202705581	0.99997366	NA
ASV_16s_21879	0.152257144	-0.551674034	3.551046083	0.592424587	NA	NA
ASV_16s_21880	0.152257144	-0.551674034	3.551046083	0.592424587	NA	NA
ASV_16s_21881	0.122449576	-0.479121111	3.55124702	0.508677845	NA	NA
ASV_16s_21882	0.122449576	-0.479121111	3.55124702	0.508677845	NA	NA
ASV_16s_21883	0.122449576	-0.479121111	3.55124702	0.508677845	NA	NA
ASV_16s_21884	0.122449576	-0.479121111	3.55124702	0.508677845	NA	NA

ASV_16s_21885	0.122449576	-0.479121111	3.55124702	0.508677845	NA	NA
ASV_16s_21886	0.122449576	-0.479121111	3.55124702	0.508677845	NA	NA
ASV_16s_21887	0.12419086	-0.483954348	3.551234266	0.513259925	0.999396466	NA
ASV_16s_21888	0.124539117	-0.484913443	3.551231748	0.514182357	0.999392876	NA
ASV_16s_21889	0.116422183	-0.461886703	3.551293422	0.493204089	0.999470987	NA
ASV_16s_21890	0.124713245	-0.485390923	3.551230495	0.5146442	0.999391074	NA
ASV_16s_21891	0.126280401	-0.489672292	3.551219319	0.518824065	NA	NA
ASV_16s_21892	0.126280401	-0.489672292	3.551219319	0.518824065	NA	NA
ASV_16s_21893	0.126280401	-0.489672292	3.551219319	0.518824065	NA	NA
ASV_16s_21894	0.106659712	-0.43212607	3.551377049	0.469543654	0.999550535	NA
ASV_16s_21895	0.112249373	-0.449476699	3.551327745	0.48288327	NA	NA
ASV_16s_21896	0.112249373	-0.449476699	3.551327745	0.48288327	NA	NA
ASV_16s_21897	0.112249373	-0.449476699	3.551327745	0.48288327	NA	NA
ASV_16s_21898	0.112249373	-0.449476699	3.551327745	0.48288327	NA	NA
ASV_16s_21899	0.112249373	-0.449476699	3.551327745	0.48288327	NA	NA
ASV_16s_21900	0.112249373	-0.449476699	3.551327745	0.48288327	NA	NA
ASV_16s_21901	0.112249373	-0.449476699	3.551327745	0.48288327	NA	NA
ASV_16s_21902	0.112249373	-0.449476699	3.551327745	0.48288327	NA	NA
ASV_16s_21903	0.123863803	-0.483050858	3.551236641	0.512396114	NA	NA
ASV_16s_21904	0.123863803	-0.483050858	3.551236641	0.512396114	NA	NA
ASV_16s_21905	0.099146248	-0.407644862	3.551447663	0.450889631	0.999607178	NA
ASV_16s_21906	0.095447889	-0.39515614	3.551481058	0.444987658	0.999624028	NA
ASV_16s_21907	0.095447889	-0.39515614	3.551481058	0.444987658	0.999624028	NA
ASV_16s_21908	0.095447889	-0.39515614	3.551481058	0.444987658	0.999624028	NA
ASV_16s_21909	0.095447889	-0.39515614	3.551481058	0.444987658	0.999624028	NA
ASV_16s_21910	0.095447889	-0.39515614	3.551481058	0.444987658	0.999624028	NA
ASV_16s_21911	0.095447889	-0.39515614	3.551481058	0.444987658	0.999624028	NA
ASV_16s_21912	0.095447889	-0.39515614	3.551481058	0.444987658	0.999624028	NA
ASV_16s_21913	0.095447889	-0.39515614	3.551481058	0.444987658	0.999624028	NA
ASV_16s_21914	0.095447889	-0.39515614	3.551481058	0.444987658	0.999624028	NA
ASV_16s_21915	0.095447889	-0.39515614	3.551481058	0.444987658	0.999624028	NA
ASV_16s_21916	0.094181815	-0.390617399	3.551492194	0.441434327	0.99963393	NA
ASV_16s_21917	0.106751685	-0.432394688	3.551376274	0.469733347	0.999549932	NA
ASV_16s_21918	0.0939286	-0.389703033	3.551494449	0.440762763	0.999635781	NA
ASV_16s_21919	0.093422171	-0.387867771	3.551498984	0.43944943	0.999639383	NA
ASV_16s_21920	0.092409312	-0.384169782	3.551508165	0.43692242	0.999646245	NA
ASV_16s_21921	0.089877164	-0.374770229	3.551531772	0.431019294	0.999661924	NA
ASV_16s_21922	0.089877164	-0.374770229	3.551531772	0.431019294	0.999661924	NA
ASV_16s_21923	0.089877164	-0.374770229	3.551531772	0.431019294	0.999661924	NA
ASV_16s_21924	0.089877164	-0.374770229	3.551531772	0.431019294	0.999661924	NA
ASV_16s_21925	0.089877164	-0.374770229	3.551531772	0.431019294	0.999661924	NA
ASV_16s_21926	0.089877164	-0.374770229	3.551531772	0.431019294	0.999661924	NA
ASV_16s_21927	0.089877164	-0.374770229	3.551531772	0.431019294	0.999661924	NA
ASV_16s_21928	0.089877164	-0.374770229	3.551531772	0.431019294	0.999661924	NA
ASV_16s_21929	0.089877164	-0.374770229	3.551531772	0.431019294	0.999661924	NA
ASV_16s_21930	0.089877164	-0.374770229	3.551531772	0.431019294	0.999661924	NA
ASV_16s_21931	0.089877164	-0.374770229	3.551531772	0.431019294	0.999661924	NA
ASV_16s_21932	0.089877164	-0.374770229	3.551531772	0.431019294	0.999661924	NA
ASV_16s_21933	0.089877164	-0.374770229	3.551531772	0.431019294	0.999661924	NA
ASV_16s_21934	0.089877164	-0.374770229	3.551531772	0.431019294	0.999661924	NA
ASV_16s_21935	0.089877164	-0.374770229	3.551531772	0.431019294	0.999661924	NA
ASV_16s_21936	0.089877164	-0.374770229	3.551531772	0.431019294	0.999661924	NA
ASV_16s_21937	0.136895142	-0.517153635	3.551148336	0.54797949	NA	NA
ASV_16s_21938	0.136895142	-0.517153635	3.551148336	0.54797949	NA	NA
ASV_16s_21939	0.136895142	-0.517153635	3.551148336	0.54797949	NA	NA
ASV_16s_21940	0.081181381	0.724576518	3.55194609	0.282505739	0.999918385	NA
ASV_16s_21941	0.028635439	-0.138967854	3.551273547	0.102974793	0.999997442	NA
ASV_16s_21942	0.058176696	0.220308354	3.551073044	0.260945167	NA	NA
ASV_16s_21943	0.051537458	0.221601797	3.551126179	0.224107988	0.999962882	NA
ASV_16s_21944	0.039918792	0.227291475	3.551231709	0.155180307	0.999989468	NA
ASV_16s_21950	0.140166692	0.595956478	3.550746271	0.526740434	NA	NA
ASV_16s_21951	0.127002367	-0.45443705	3.551368816	0.480096332	0.999516146	NA
ASV_16s_21952	0.085549892	-0.450405728	3.55154422	0.333309045	0.999857238	NA
ASV_16s_21953	0.094821074	0.488323929	3.551972821	0.381607913	0.999774985	NA
ASV_16s_21954	0.102608055	0.041257553	3.551676001	0.504328729	0.999430476	NA
ASV_16s_21955	0.123616316	-0.428220003	3.551435538	0.483775038	0.999503749	NA
ASV_16s_21956	0.11061401	-0.370372962	3.551515272	0.454880795	0.999595495	NA

ASV_16s_21957	0.092877373	-0.021339588	3.551652691	0.39550769	0.999746333	NA
ASV_16s_21958	0.117040761	-0.105747981	3.551389403	0.491869136	0.999475712	NA
ASV_16s_21959	0.09683774	0.061020216	3.551631428	0.318570822	0.999877442	NA
ASV_16s_21960	0.093377408	0.010087928	3.551608909	0.404608331	0.999726272	NA
ASV_16s_21961	0.153421584	-0.427269367	3.551405768	0.465088735	0.999564538	NA
ASV_16s_21962	0.107193868	-0.345610438	3.551481384	0.464982818	0.999564867	NA
ASV_16s_21963	0.141481165	-0.41741011	3.55125519	0.601461603	0.998983695	NA
ASV_16s_21964	0.119276313	-0.363407237	3.551313465	0.522425869	0.999360153	NA
ASV_16s_21965	0.118497449	-0.362780658	3.551324537	0.518785964	0.999374746	NA
ASV_16s_21966	0.120092687	-0.371219866	3.55131075	0.52398009	0.999353852	NA
ASV_16s_21967	0.156471344	0.063638776	3.551172864	0.734634064	0.998055242	NA
ASV_16s_21968	0.129762845	-0.340396796	3.551275915	0.570821288	0.999143635	NA
ASV_16s_21969	0.122122992	-0.337689396	3.551292099	0.542320331	0.999276294	NA
ASV_16s_21970	0.110874054	-0.314892192	3.551381421	0.499275445	0.99944913	NA
ASV_16s_21971	0.119985591	-0.343909526	3.551284361	0.534220959	0.99931128	NA
ASV_16s_21972	0.105929992	-0.304466138	3.551431393	0.48026433	0.999515585	NA
ASV_16s_21973	0.105929992	-0.304466138	3.551431393	0.48026433	0.999515585	NA
ASV_16s_21974	0.161902596	0.183286602	3.551680167	0.521433985	0.999364152	NA
ASV_16s_21975	0.097947701	0.996276478	3.5509799	0.238717261	0.999953961	NA
ASV_16s_21976	0.118921358	-0.222143571	3.551293554	0.550386982	0.999240272	NA
ASV_16s_21977	0.118823993	-0.222108501	3.551294608	0.549932845	0.999242332	NA
ASV_16s_21978	0.107638111	-0.220303701	3.551350941	0.50670556	0.999421556	NA
ASV_16s_21979	0.113582104	-0.222514237	3.551282501	0.533982568	0.999312292	NA
ASV_16s_21980	0.099303685	0.888166361	3.551819788	0.272665731	0.999927634	NA
ASV_16s_21981	0.149684472	-0.460505475	3.551200661	0.563194063	0.999180621	NA
ASV_16s_21982	0.108714201	-0.080786163	3.551377627	0.528893889	0.999333655	NA
ASV_16s_21983	0.129948887	0.316995606	3.551198124	0.599842062	0.998992615	NA
ASV_16s_21984	0.117661683	0.315866414	3.55121601	0.563159682	0.999180785	NA
ASV_16s_21985	0.115576339	0.168709261	3.550579442	0.450080952	0.999609516	NA
ASV_16s_21986	0.100187432	0.310520686	3.551719611	0.429192546	0.999666678	NA
ASV_16s_21987	0.116901046	-0.002366364	3.551231012	0.572714785	0.999134281	NA
ASV_16s_21988	0.098655176	-0.176234867	3.551253096	0.543828235	0.99926965	NA
ASV_16s_21989	0.100221656	0.002632987	3.551364155	0.542558241	0.999275249	NA
ASV_16s_21990	0.120255514	0.576908145	3.551130318	0.393121767	0.99975142	NA
ASV_16s_21991	0.119832385	1.09581515	3.551625956	0.217044508	0.999966727	NA
ASV_16s_21992	0.132454018	0.114435518	3.551446869	0.534098609	0.9993118	NA
ASV_16s_21993	0.125998894	0.159232614	3.551169194	0.659460051	0.998628207	NA
ASV_16s_21994	0.09196998	-0.235776526	3.551231347	0.453961432	0.999598207	NA
ASV_16s_21995	0.117904067	0.972264222	3.551576622	0.447384577	0.999617245	NA
ASV_16s_21996	0.141958165	0.83459297	3.551907492	0.469750781	0.999549877	NA
ASV_16s_21997	0.118141413	1.179047571	3.551654021	0.213235971	0.99996868	NA
ASV_16s_21998	0.152165296	0.292730201	3.551771533	0.57501961	NA	NA
ASV_16s_21999	0.12492578	1.055531313	3.551411215	0.46605992	0.999561511	NA
ASV_16s_22000	0.12993306	1.069185542	3.551380167	0.477369582	0.999525198	NA
ASV_16s_22001	0.136067339	0.888312899	3.551761264	0.390479059	0.999756972	NA
ASV_16s_22002	0.099502823	0.975406776	3.551575572	0.41078333	0.999712056	NA
ASV_16s_22003	0.099502823	0.975406776	3.551575572	0.41078333	0.999712056	NA
ASV_16s_22004	0.099502823	0.975406776	3.551575572	0.41078333	0.999712056	NA
ASV_16s_22005	0.099502823	0.975406776	3.551575572	0.41078333	0.999712056	NA
ASV_16s_22006	0.114409676	1.024452734	3.551472899	0.441473884	0.999633821	NA
ASV_16s_22007	0.111963109	1.016868268	3.551488268	0.438408881	0.99964222	NA
ASV_16s_22008	0.111963109	1.016868268	3.551488268	0.438408881	0.99964222	NA
ASV_16s_22009	0.111963109	1.016868268	3.551488268	0.438408881	0.99964222	NA
ASV_16s_22010	0.121525667	0.131915786	3.551406823	0.357352754	0.999819509	NA
ASV_16s_22011	0.11182003	1.226855819	3.551428411	0.251276776	0.999945178	NA
ASV_16s_22012	0.10272601	0.986597069	3.551551568	0.419878032	0.999690207	NA
ASV_16s_22013	0.10272601	0.986597069	3.551551568	0.419878032	0.999690207	NA
ASV_16s_22014	0.10272601	0.986597069	3.551551568	0.419878032	0.999690207	NA
ASV_16s_22015	0.10272601	0.986597069	3.551551568	0.419878032	0.999690207	NA
ASV_16s_22016	0.10272601	0.986597069	3.551551568	0.419878032	0.999690207	NA
ASV_16s_22017	0.10272601	0.986597069	3.551551568	0.419878032	0.999690207	NA
ASV_16s_22018	0.097391316	0.332182143	3.551975738	0.434405876	0.999652989	NA
ASV_16s_22019	0.097391316	0.332182143	3.551975738	0.434405876	0.999652989	NA
ASV_16s_22020	0.097391316	0.332182143	3.551975738	0.434405876	0.999652989	NA
ASV_16s_22021	0.116480044	0.338710573	3.551928453	0.478282783	0.99952218	NA
ASV_16s_22022	0.106435193	0.344024489	3.551989326	0.244338037	0.999950163	NA
ASV_16s_22023	0.133107506	-0.304192264	3.551566348	0.403308617	0.999729201	NA

ASV_16s_22024	0.117904067	0.339222855	3.551923324	0.481930646	NA	NA
ASV_16s_22025	0.117904067	0.339222855	3.551923324	0.481930646	NA	NA
ASV_16s_22026	0.117904067	0.339222855	3.551923324	0.481930646	NA	NA
ASV_16s_22027	0.117904067	0.339222855	3.551923324	0.481930646	NA	NA
ASV_16s_22028	0.117904067	0.339222855	3.551923324	0.481930646	NA	NA
ASV_16s_22029	0.130406954	0.343401127	3.55188274	0.514504183	NA	NA
ASV_16s_22030	0.128214923	0.347768294	3.551952266	0.271859745	0.999928358	NA
ASV_16s_22031	0.146665785	0.299661444	3.55193834	0.328215608	0.999864462	NA
ASV_16s_22032	0.148625901	0.823771734	3.551275392	0.768314732	0.997754026	NA
ASV_16s_22033	0.162004175	0.755680726	3.55173154	0.502336292	0.999437882	NA
ASV_16s_22034	0.075472453	0.325745901	3.552026889	0.370133189	0.999796892	NA
ASV_16s_22035	0.075472453	0.325745901	3.552026889	0.370133189	0.999796892	NA
ASV_16s_22036	0.075472453	0.325745901	3.552026889	0.370133189	0.999796892	NA
ASV_16s_22037	0.102191385	0.333881595	3.551963706	0.444732243	0.999624746	NA
ASV_16s_22038	0.102191385	0.333881595	3.551963706	0.444732243	0.999624746	NA
ASV_16s_22039	0.102191385	0.333881595	3.551963706	0.444732243	0.999624746	NA
ASV_16s_22040	0.102191385	0.333881595	3.551963706	0.444732243	0.999624746	NA
ASV_16s_22041	0.082496822	0.244126202	3.551507178	0.158833847	0.99998859	NA
ASV_16s_22042	0.068132715	-0.143486856	3.55176869	0.238601804	0.999954037	NA
ASV_16s_22043	0.177193872	0.362383316	3.551657681	0.651814973	NA	NA
ASV_16s_22044	0.135085715	0.342292504	3.552024447	0.303694407	0.999895733	NA
ASV_16s_22045	0.131370717	-0.46564076	3.551280236	0.52169031	NA	NA
ASV_16s_22046	0.126280401	-0.454419604	3.551321194	0.508352142	NA	NA
ASV_16s_22047	0.120477882	0.338877653	3.551479284	0.352075536	0.999828317	NA
ASV_16s_22048	0.15082164	-0.500378367	3.551116606	0.575342905	NA	NA
ASV_16s_22049	0.115091136	0.83460495	3.551418219	0.32719535	0.999865878	NA
ASV_16s_22050	0.075375185	-0.301537599	3.551754711	0.376958745	0.999784047	NA
ASV_16s_22051	0.075375185	-0.301537599	3.551754711	0.376958745	0.999784047	NA
ASV_16s_22052	0.083842875	-0.333045897	3.551687769	0.398925515	0.999738921	NA
ASV_16s_22053	0.110883854	-0.416173264	3.551452208	0.470566118	NA	NA
ASV_16s_22054	0.110883854	-0.416173264	3.551452208	0.470566118	NA	NA
ASV_16s_22055	0.117160961	-0.647073794	3.551433115	0.25781834	0.99994017	NA
ASV_16s_22056	0.098661333	-0.381356382	3.551555828	0.443875885	NA	NA
ASV_16s_22057	0.135941797	1.085077707	3.551341805	0.491409702	NA	NA
ASV_16s_22058	0.135941797	1.085077707	3.551341805	0.491409702	NA	NA
ASV_16s_22059	0.148715499	1.116825609	3.551268519	0.52273327	NA	NA
ASV_16s_22060	0.111753259	1.058524522	3.551240748	0.380527694	0.999777113	NA
ASV_16s_22061	0.111753259	1.058524522	3.551240748	0.380527694	0.999777113	NA
ASV_16s_22062	0.058324436	0.859802549	3.551691645	0.227826057	0.999960738	NA
ASV_16s_22063	0.058324436	0.859802549	3.551691645	0.227826057	0.999960738	NA
ASV_16s_22064	0.072282389	0.920785488	3.551568893	0.273699893	0.999926698	NA
ASV_16s_22065	0.072282389	0.920785488	3.551568893	0.273699893	0.999926698	NA
ASV_16s_22066	0.09998863	1.022084975	3.551331052	0.3554209	0.999822768	NA
ASV_16s_22067	0.09998863	1.022084975	3.551331052	0.3554209	0.999822768	NA
ASV_16s_22068	0.09998863	1.022084975	3.551331052	0.3554209	0.999822768	NA
ASV_16s_22069	0.09998863	1.022084975	3.551331052	0.3554209	0.999822768	NA
ASV_16s_22070	0.09998863	1.022084975	3.551331052	0.3554209	0.999822768	NA
ASV_16s_22071	0.13781125	1.127560971	3.551084591	0.437517194	NA	NA
ASV_16s_22072	0.126388231	0.359216455	3.551578144	0.441094761	NA	NA
ASV_16s_22073	0.126388231	0.359216455	3.551578144	0.441094761	NA	NA
ASV_16s_22074	0.126388231	0.359216455	3.551578144	0.441094761	NA	NA
ASV_16s_22075	0.092282464	0.273991564	3.551543573	0.128016915	0.999994573	NA
ASV_16s_22076	0.114653678	0.353380935	3.551629535	0.412034569	NA	NA
ASV_16s_22077	0.114653678	0.353380935	3.551629535	0.412034569	NA	NA
ASV_16s_22078	0.114653678	0.353380935	3.551629535	0.412034569	NA	NA
ASV_16s_22079	0.104100841	0.348019374	3.551680378	0.388221953	NA	NA
ASV_16s_22080	0.104100841	0.348019374	3.551680378	0.388221953	NA	NA
ASV_16s_22081	0.104100841	0.348019374	3.551680378	0.388221953	NA	NA
ASV_16s_22082	0.104100841	0.348019374	3.551680378	0.388221953	NA	NA
ASV_16s_22083	0.131344712	-0.046447697	3.551079999	0.679499541	0.998488342	NA
ASV_16s_22084	0.085934015	0.344509412	3.551738851	0.344991211	0.999839669	NA
ASV_16s_22085	0.085934015	0.344509412	3.551738851	0.344991211	0.999839669	NA
ASV_16s_22086	0.085934015	0.344509412	3.551738851	0.344991211	0.999839669	NA
ASV_16s_22087	0.147208897	0.368286781	3.551467205	0.497327316	NA	NA
ASV_16s_22088	0.092977558	0.345742921	3.551715881	0.365468839	0.99980536	NA
ASV_16s_22089	0.032373993	0.336892321	3.551946342	0.149565547	0.999990722	NA
ASV_16s_22090	0.130434761	0.36101367	3.551560912	0.451652826	NA	NA

ASV_16s_22091	0.052238669	-0.223869519	3.551604295	0.231923221	0.999958277	NA
ASV_16s_22092	0.052238669	-0.223869519	3.551604295	0.231923221	0.999958277	NA
ASV_16s_22093	0.052238669	-0.223869519	3.551604295	0.231923221	0.999958277	NA
ASV_16s_22094	0.128145899	-0.472655522	3.550995257	0.446943308	NA	NA
ASV_16s_22095	0.128145899	-0.472655522	3.550995257	0.446943308	NA	NA
ASV_16s_22096	0.179021487	-0.532175208	3.550509536	0.59101302	NA	NA
ASV_16s_22097	0.170615565	-0.528354982	3.550589539	0.565739221	NA	NA
ASV_16s_22098	0.170615565	-0.528354982	3.550589539	0.565739221	NA	NA
ASV_16s_22099	0.170615565	-0.528354982	3.550589539	0.565739221	NA	NA
ASV_16s_22100	0.157834099	-0.5159782	3.550700818	0.528057923	NA	NA
ASV_16s_22101	0.063442666	-0.2778015	3.551521871	0.273466092	0.99992691	NA
ASV_16s_22102	0.063442666	-0.2778015	3.551521871	0.273466092	0.99992691	NA
ASV_16s_22103	0.058143838	-0.253239959	3.551559863	0.254080008	0.999943069	NA
ASV_16s_22104	0.058143838	-0.253239959	3.551559863	0.254080008	0.999943069	NA
ASV_16s_22105	0.058143838	-0.253239959	3.551559863	0.254080008	0.999943069	NA
ASV_16s_22106	0.130733325	1.110152962	3.551122183	0.421049286	NA	NA
ASV_16s_22107	0.114675363	1.06697328	3.551220467	0.386365131	0.999765444	NA
ASV_16s_22108	0.046092769	0.797737893	3.551789865	0.184231816	0.999981012	NA
ASV_16s_22109	0.046092769	0.797737893	3.551789865	0.184231816	0.999981012	NA
ASV_16s_22110	0.114041804	1.065158195	3.551224794	0.385085935	0.999768036	NA
ASV_16s_22111	0.114041804	1.065158195	3.551224794	0.385085935	0.999768036	NA
ASV_16s_22112	0.114041804	1.065158195	3.551224794	0.385085935	0.999768036	NA
ASV_16s_22113	0.104100841	-0.077871832	3.551038663	0.623392521	NA	NA
ASV_16s_22114	0.104100841	-0.077871832	3.551038663	0.623392521	NA	NA
ASV_16s_22115	0.104100841	-0.077871832	3.551038663	0.623392521	NA	NA
ASV_16s_22116	0.104100841	-0.077871832	3.551038663	0.623392521	NA	NA
ASV_16s_22117	0.104100841	-0.077871832	3.551038663	0.623392521	NA	NA
ASV_16s_22118	0.104100841	-0.077871832	3.551038663	0.623392521	NA	NA
ASV_16s_22119	0.096434406	-0.077729134	3.551079987	0.595295183	0.999017374	NA
ASV_16s_22120	0.088767971	-0.077572355	3.551128355	0.56863218	NA	NA
ASV_16s_22121	0.088767971	-0.077572355	3.551128355	0.56863218	NA	NA
ASV_16s_22122	0.088767971	-0.077572355	3.551128355	0.56863218	NA	NA
ASV_16s_22123	0.088767971	-0.077572355	3.551128355	0.56863218	NA	NA
ASV_16s_22124	0.088767971	-0.077572355	3.551128355	0.56863218	NA	NA
ASV_16s_22125	0.088767971	-0.077572355	3.551128355	0.56863218	NA	NA
ASV_16s_22126	0.088767971	-0.077572355	3.551128355	0.56863218	NA	NA
ASV_16s_22127	0.088767971	-0.077572355	3.551128355	0.56863218	NA	NA
ASV_16s_22128	0.088767971	-0.077572355	3.551128355	0.56863218	NA	NA
ASV_16s_22129	0.088767971	-0.077572355	3.551128355	0.56863218	NA	NA
ASV_16s_22130	0.058176696	0.220308354	3.551073044	0.260945167	NA	NA
ASV_16s_22131	0.058176696	0.220308354	3.551073044	0.260945167	NA	NA
ASV_16s_22132	0.058176696	0.220308354	3.551073044	0.260945167	NA	NA
ASV_16s_22133	0.058176696	0.220308354	3.551073044	0.260945167	NA	NA
ASV_16s_22134	0.061771237	0.220286909	3.551044389	0.280176372	0.999920645	NA
ASV_16s_22135	0.063526518	0.220277063	3.551031556	0.28937928	0.999911456	NA
ASV_16s_22136	0.066084685	0.220264012	3.551014068	0.302628253	NA	NA
ASV_16s_22137	0.066084685	0.220264012	3.551014068	0.302628253	NA	NA
ASV_16s_22138	0.066084685	0.220264012	3.551014068	0.302628253	NA	NA
ASV_16s_22139	0.066024701	0.220264092	3.551014459	0.302320371	0.99989732	NA
ASV_16s_22140	0.065952719	0.220264669	3.551014937	0.301951587	NA	NA
ASV_16s_22141	0.065952719	0.220264669	3.551014937	0.301951587	NA	NA
ASV_16s_22142	0.068283262	0.444408276	3.550939413	0.426293302	0.999674128	NA
ASV_16s_22143	0.109904138	0.310959398	3.551181963	0.62530684	0.998846158	NA
ASV_16s_22144	0.112788822	0.310958541	3.551166904	0.635404714	NA	NA
ASV_16s_22145	0.112610321	0.310958599	3.551167814	0.634777147	0.998788241	NA
ASV_16s_22146	0.113740767	0.310958232	3.551162101	0.63875748	0.998763329	NA
ASV_16s_22147	0.098346734	0.310964029	3.551251071	0.585947522	NA	NA
ASV_16s_22148	0.098346734	0.310964029	3.551251071	0.585947522	NA	NA
ASV_16s_22149	0.112431819	0.310958658	3.551168727	0.634149967	NA	NA
ASV_16s_22150	0.069615001	0.595452105	3.550943994	0.272714143	NA	NA
ASV_16s_22151	0.12350419	0.998283692	3.551512305	0.41759707	0.99969579	NA
ASV_16s_22152	0.155609712	-0.738508676	3.551282541	0.387639877	0.999762841	NA
ASV_16s_22153	0.066763062	0.440714382	3.551104056	0.316473312	0.999880146	NA
ASV_16s_22154	0.143388141	0.641206555	3.551799589	0.583388228	0.999080264	NA
ASV_16s_22155	0.098401138	0.13806279	3.551524614	0.472544939	0.99954093	NA
ASV_16s_22156	0.103317211	-0.096640759	3.551624739	0.330724438	0.999860936	NA
ASV_16s_22157	0.102086538	-0.060552263	3.551296176	0.632571539	0.9988019	NA

ASV_16s_22158	0.094218673	-0.048203669	3.551475647	0.598649072	0.998999151	NA
ASV_16s_22159	0.094714191	-0.040943028	3.551526172	0.600043139	0.99899151	NA
ASV_16s_22160	0.106751607	0.318898015	3.551841133	0.453475782	0.999599634	NA
ASV_16s_22161	0.075327203	0.849877443	3.551831272	0.350728298	0.999830517	NA
ASV_16s_22162	0.080323441	0.634287559	3.551862434	0.449431634	0.999611387	NA
ASV_16s_22163	0.121949343	0.98230637	3.551549703	0.455877115	0.999592542	NA
ASV_16s_22164	0.200537467	0.488673855	3.550522039	0.993526325	0.994932291	NA
ASV_16s_22165	0.135941797	0.294966897	3.551819724	0.528530692	NA	NA
ASV_16s_22166	0.165869865	-0.578291053	3.550961829	0.633862995	NA	NA
ASV_16s_22167	0.16446453	1.076406907	3.551255899	0.558426418	NA	NA
ASV_16s_22168	0.111133968	0.885474541	3.551558723	0.417476424	0.999696083	NA
ASV_16s_22169	0.078277197	0.286824816	3.551817396	0.381931104	0.999774345	NA
ASV_16s_22170	0.078277197	0.286824816	3.551817396	0.381931104	0.999774345	NA
ASV_16s_22171	0.053961612	0.292638248	3.551897698	0.289616399	0.99991121	NA
ASV_16s_22172	0.053961612	0.292638248	3.551897698	0.289616399	0.99991121	NA
ASV_16s_22173	0.053961612	0.292638248	3.551897698	0.289616399	0.99991121	NA
ASV_16s_22174	0.074836568	0.287223869	3.55183057	0.369992218	0.999797151	NA
ASV_16s_22175	0.026850938	0.300516924	3.551955409	0.162541674	0.999987648	NA
ASV_16s_22176	0.228805462	1.078940713	3.550973851	0.721773167	NA	NA
ASV_16s_22177	0.088219023	0.830291462	3.551661034	0.360929948	0.999813363	NA
ASV_16s_22178	0.202757178	-0.546449072	3.550629783	0.73464607	NA	NA
ASV_16s_22179	0.164321757	0.248548639	3.551516606	0.719535615	0.99818109	NA
ASV_16s_22180	0.130733325	1.110152962	3.551122183	0.421049286	NA	NA
ASV_16s_22181	0.130733325	1.110152962	3.551122183	0.421049286	NA	NA
ASV_16s_22182	0.130733325	1.110152962	3.551122183	0.421049286	NA	NA
[truncated: 597,330 more chars]
