# Supplementary material for: Conserved organ-specific microbial assemblages in different populations of a terrestrial crab
Source: Front Microbiol. 2023 Jun 12;14:1113617. doi: 10.3389/fmicb.2023.1113617 (PMC10291174; doi:10.3389/fmicb.2023.1113617)
Supplement: Supplementary file 1 [file Data_Sheet_1.pdf]

Sequence variants inference

Amplicon sequences obtained from 16S rRNA gene and ITS1 region were analysed using the DADA2 pipeline (version 1.10.1) (Callahan et al., 2016) in R version 3.5.3 (http://www.R-project.org). Primers used for PCR amplification were removed using cutadapt version 1.15 in paired-end mode (Martin, 2011). Sequences that did not contain the primer were discarded removing both pairs if at least one of them (R1 or R2) did not pass the filtering criteria (96.26% and 90.58% of raw sequences coming from 16S rRNA gene and ITS1 region were retained, respectively). Only the corresponding primer was matched in bacterial 16S rRNA amplicons (341F for R1 mates and 805R for R2 mates) whereas in ITS1 regions both primers could be matched but only if identified in the correct orientation (ITS1F forward in 5' position and ITS2 reverse and complement in 3' position for R1 mates and the opposite for R2 mates; for additional information see https://benjjneb.github.io/dada2/ITS_workflow.html). Since samples were sequenced using multiple Illumina flow cells, we followed the "big data" approach (described at: https://benjjneb.github.io/dada2/bigdata.html) to infer amplicon sequence variants (ASVs) with a relatively modest memory requirement and to respect the dependence of DADA2 error models from the sequencing run. Reads coming from 16S rRNA gene amplification (V3-V4 region) were trimmed at 280bp (forward) and 200bp (reverse) to remove low quality segments while retaining enough overlapping nucleotides to reconstruct the full amplicon (Obertegger et al., 2018; Rosso et al., 2018). Fungal ITS1 amplicons were not trimmed to a fixed length to avoid losses of longer fragments that could be present due to the high variability in length of this region, as also reported by the authors of the DADA2 pipeline (https://benjjneb.github.io/dada2/ITS_workflow.html). On the other side, to reduce the number of small fragments, fungal reads shorter than 70bp were removed from subsequent analyses. Reads (and their respective mate) that contained ambiguous bases or more than two expected errors were filtered out to minimize errors due to the sequencing process. Sequences were denoised using the DADA2 algorithm with default parameters. The denoised mates were merged and all reads with any mismatches and an overlap length shorter than 20bp were removed (87.18% and 96.65% of trimmed reads coming from 16S rRNA gene and ITS1 region were correctly merged, respectively). Chimeric sequences were identified and removed using the removeBimeraDenovo function with "consensus" method (5.51% and 0.89% of analysed sequence coming from 16S rRNA gene and ITS1 region were flagged as chimeric and removed from subsequent analyses). Samples with a low coverage (expressed as the number of reads assigned to an ASV) where re-sequenced and added to the inference pipeline (Table S1). To respect the dependence of DADA2 error models from the run, counts from re-sequenced samples were summed together after the variant inference step.

Classification of amplicon sequence variants

Sequence variants were taxonomically annotated using the DECIPHER package (version 2.10.2) against two widely used databases: Silva SSU (version 138) (Quast et al., 2012; Wright, 2016) and Warcup v2 ITS database (Deshpande et al., 2016). The IDTAXA algorithm implemented in the package (Murali et al., 2018) needs a trained database for the correct classification of query sequences so we used the trained version of both databases available from the DECIPHER site (http://www2.decipher.codes/Downloads.html). Sequence variants not assigned to Bacterial, Archaea, or Fungal domains were removed from subsequent analyses to minimize biases due to DNA contamination, or to sequencing errors. Even if the primers used in this work are not reported

to cross-amplify chloroplast or mitochondrial DNA (Beckers et al., 2016; Herlemann et al., 2011; Klindworth et al., 2013) we removed any 16S rRNA amplicon sequence variant (ASV) that was assigned to these taxa to avoid additional biases during downstream analyses. Two fungal samples, namely samples "GI_TKP_5" and sample "MG_SH_1", were removed from downstream analyses since their ASV count dropped to zero. The number of reads retained after each step was reported in Figure S1.

Fungal amplicon sequence variant evaluation

Fungal variants showed a massive loss of reads in crab's organs (Figure S1) so we sequenced two samples for each crab's organ independently in order to validate the workflow used in an unsupervised fashion (Table S1). DNA was extracted and amplified independently for each replicate before sequencing. Replicates were included in the DADA2 pipeline together with the other samples and they were used to evaluate the overall accuracy. We considered two main parameters: a) the Spearman's rank correlation coefficient (ρ) between replicates, and b) the overall accuracy. The accuracy was computed as:

$$\frac{(TP + TN)}{(TP + TN + FP + FN)}$$

where, TP was the number of ASVs detected in both replicates (true positives); TN was the number of ASVs not detected in both replicates (true negatives); $FP + FN$ was the number of ASVs detected in one replicate but not in the other one (false negatives). Accuracy was estimated by comparing replicates in pairs (two pairs per organ for a total of eight contrasts). We used a non-parametric correlation coefficient to measure monotonic relationships between the abundance of ASVs in all replicates. To reduce differences due to the total size of different replicates, ASV counts were transformed into "parts per thousand" by dividing them by the total read count and then multiplied by 1000. A $\log_2$ transformation was performed before correlation coefficient calculation (Bulgarelli et al., 2012). Values of accuracy and ρ for each sample sequenced were reported in Table S2 whereas the $\log_2$-transformed counts of ASVs in each replicate was reported in Figure S2.

Microbial diversity within sample types and sampling sites

The coverage of microbiome data obtained through amplicon sequencing is extremely variable across samples due to differences in PCR amplification, sequencing yields, and clustering algorithms. This variability may induce over/underestimation biases when comparing microbial diversity, especially when using indices that rely on the abundance of single species. To minimize possible biases, we normalized counts by using the "median ratio method" implemented in the counts function of the DESeq2 R package (version 1.28.1) (Anders and Huber, 2010; Love et al., 2014). Differently from the "variance stabilizing transformation" (VST)–mainly used within the same package to transform count data into approximately homoskedastic data that can be used for clustering and machine learning approaches–the "median ratio method" produces positive abundance values that can be directly used to estimate differences within- and between-samples (also called alpha- and beta-diversity) (Weiss et al., 2017).

Normalized counts were used to estimate differences in the total microbial diversity across sample types by using three alpha-diversity metrics, all based on the inverse Simpson index. This index is

defined as the reciprocal of the Simpson index D (D') but it can be also defined as a diversity measure with a Hill number of order two–a definition mostly used in ecological studies (Alberdi and Gilbert, 2019). Hill numbers (^qD) are represented with the equation:

$$^qD = \left(\sum_{i=1}^{R} p_i^q\right)^{1/(1-q)}$$

were q is the order of the Hill number ^qD. For $q = 2$ we can write the equation as:

$$^2D = \frac{1}{\sum_{i=1}^{R} p_i^2}$$

Since $\sum_{i=1}^{R} p_i^2$ corresponds to the definition of the Simpson index, the equation corresponds to the inverse of the Simpson index. This index is particularly useful since it transforms the Simpson concentration–that is by definition the probability of drawing two equal species taken at random from the dataset–into a measure of diversity so that the higher the index, the higher the microbial diversity. We used the iNEXT R package (version 2.0.20) (Hsieh et al., 2016) to compute interpolated, observed and extrapolated values of alpha diversity with Hill number of order 2 (inverse Simpson index). All values were computed separately for 16S rRNA and ITS1 amplicons by using 30 bootstrap replications each. In order to compare different samples, we choose a fixed range of sizes (number of read sequenced) for the two amplicons so to obtain vectors of diversity of the same size. Since the number of reads collected for 16S rRNA amplicons was higher than that obtained for ITS1 amplicons (a maximum of roughly 460'000 normalized counts for 16S amplicons against 60'000 for ITS1) we computed alpha diversity along 300 and 100 size knots, respectively. The iNEXT package calculate interpolated and extrapolated values of diversity as a function of the sample size (called m) using the formula reported in the paper from Chao and colleagues (Chao et al., 2014). An interpolated diversity estimator ($^q\widehat{D}(m)$) is derived for any size $m < n$ where n is the total counts in the sample. Similarly, an extrapolated diversity estimator is derived ($^q\widehat{D}(m+n)$) for any enlarged sample of size $m + n > n$. Observed microbial diversity is computed by using the diversity formula described above. Confidence intervals were constructed using the bootstrap method and joined to recreate a smooth curve (Chao and Jost, 2012). Generated curves were extended to the maximum sampling size of all samples in order to check if the sequencing effort could be considered enough for the comparison of alpha-diversity across different samples (namely, the inverse Simpson index). Results were reported for each sample type (Figure 2 panels a and c in the main text) and for each sample site (Figure S3 panels a and c). Average differences between extrapolated and observed inverse Simpson index (Table 1 in the main text) were really low, ranging from 0.05 to 2.74 indicating that the sequencing effort was enough to explore the alpha diversity of sample coming from different biological matrices (both crab's organs and environmental samples). Observed inverse Simpson index and Good's coverage values were reported for each sample in Table S3. Good's coverage index was computed following its standard definition (Good, 1953):

$$(1 - \frac{n}{N}) \cdot 100$$

where, n is the number of sequences found once in a specimen (also called singletons) and N is the total number of counts in that specimen. Good's coverage estimator ranged between 99.89% and 100.00% across all samples indicating that roughly 0.11% of the reads in a sample came from ASVs

that appear only once in that sample. To detect differences across sample types and sampling sites, we used the non-parametric pairwise Wilcoxon test with Benjamini–Hochberg p-value correction. Statistical significance was then reported using alphabetical letters obtained with the multcompLetters function of the multcompView package (version 0.1.8). Results were reported for each sample type (Figure 2 panels b and d in the main text) and for each sample site (Figure S3 panels b and d).

Microbial diversity between sample types and sampling sites

The variation of microbial community between samples (beta diversity) was assessed using different approaches on microbial counts generated by 16S rRNA and ITS1 amplicon sequencing separately. Before computing any beta diversity index, samples were normalized as explained in the previous section and transformed into relative abundances to smooth differences in coverage across samples. Sample distribution was reported using principal coordinates analysis (PCoA, also called classical multidimensional scaling or MDS) on Bray-Curtis diversity index (cmdscale function of stats package, and vegdist function of vegan package (Oksanen et al., 2019), respectively). We used the quantitative version of the Bray-Curtis index with formula:

$$d_{jk} = \frac{\sum_{i=1}^{N} |x_{ij} - x_{ik}|}{\sum_{i=1}^{N} x_{ij} + x_{ik}}$$

where d_{ij} is the Bray-Curtis diversity between sample i and j, N is the total number of ASVs in the two samples, x_{ij} is the abundance of the ASV i in sample j, and x_{ik} is the abundance of the same ASV in sample k. Permutational multivariate analysis of variance (PERMANOVA) was used to inspect differences between sample types and sampling sites (adonis2 function of the vegan R package, version 2.5.6) with 1000 permutations and Bray-Curtis diversity index. Sample types and sampling sites were tested in all samples whereas for crab's organs the crab was added as a covariate for controlling variance due to the crab itself (also called batch effect). The proportion of variance explained by each factor (R squared) was reported in Table S4. Approaches based on permutations, such as PERMANOVA, rely on the assumption of homoscedasticity–the dispersion should be equal in all groups otherwise it is not possible to discern a location effect (the groups) from a dispersion effect (the difference in dispersion). To test this assumption, we used the betadisper function (vegan package) to compute distances between each entity (samples) and group centroids. Differences in dispersion were then tested using the analysis of variance (ANOVA) and results were reported in Table S5. Pairwise PERMANOVA was used to assess whether sample types and sampling sites differed based on ASV counts obtained from crab's organs and environmental samples. The r-squared value of each contrast was used to inspect the amount of variance explained by crab's organs and environmental samples on the separation between sites and sample types. Principal coordinates analysis was reported in panel a of Figure 3 (by sample type) and Figure S4 (by sampling sites) whereas results of pairwise PERMANOVA were reported in panel b of the same figures.

To detect the number of species present/absent in all sample types and in all their intersections we used a graphical visualization called "upset plot" (upset function of the UpSetR package version 1.4.0 (Conway et al., 2017)). This type of representation is extremely useful to display a large number of intersections that cannot be easily grasped with Venn plots (usually four or more sets)–the number of intersections corresponds to the binomial coefficient which is 15 for four intersections and it

roughly doubles passing from four to five (31 intersections). Upset plots were introduced to address this issue by reporting set intersections in a matrix layout where each row is a different set and each intersection–indicated with linked dots–is reported column wise. The size of each intersection is reported with a bar on the top of the corresponding column and is usually ordered according to the degree of the intersection (namely, the number of sets included) and the size (Lex et al., 2014). The number of ASVs shared across different sample types was reported for 16S and ITS1 amplicons (Figure S5) both according to sampling sites (panels a and c) and sample type (panels b and d).

Clustering of sequence variants

To detect pattern of ASVs across sample types we used the likelihood ratio test (LRT) implemented in the DESeq2 package (version 1.28.1). The main advantage of this approach is that it tests the effect of all levels of one (or more) factor/s at the same time by comparing the likelihood of the full model against a model lacking the factor/s of interest. Since we wanted to identify patterns across different sample types and given that we had eight distinct sample types (namely a factor with eight levels), we tested a full model including sampling sites and sample types against a reduced model including only sampling sites. This way we accounted for dispersion due to different sampling sites while testing for the effect of all sample types at the same time. Singletons–amplicon sequence variants detected only once in the whole community–were removed before model fitting to reduce computational time and to prevent ungrounded speculations based on sporadic species. To select the model that best fitted our data, we fitted all the possible fit types implemented in the DESeq2 package (namely, "local", "mean", and "parametric"). The choice of the best model was based on the median absolute value of the log-residuals (MALR), defined as:

$$MALR = median(|log(\alpha_i^{gw}) - log(\alpha_{tr})|)$$

where α_i^{gw} is the dispersion estimate of ASV i obtained *a priori* from the data and α_{tr} is the fitted dispersion value obtained from the model (Figure S6, for in-depth definitions of parameters reported see the original DESeq2 paper (Love et al., 2014)). The model with the lowest MALR value was selected and used for subsequent analyses (fit type "local"). Results from the LRT were reported in Table S6 using the standard DESeq2 notation even if the log_2-fold changes reported in the LRT test are not directly associated to the test.

Since the LRT determines p-values by comparing dispersion between the full and the reduced model and not by a given pairwise comparison, we did not set a threshold based on fold change. The only criterion that was used to select ASVs significantly influenced by sample types was the p-value adjusted using the Benjamini–Hochberg method (namely an adjusted p-value lower than 0.05). A total of 250 ASVs were selected accounting for 50.92% of the total microbial abundance (mean equals to 56.55% with a standard error of 2.11%). Variance stabilizing transformation (VST, implemented in the vst function of the DESeq2 package) was then used to transform counts into approximately homoskedastic data for clustering. Since abundances of microbial ASVs inferred through amplicon sequencing may have extremely different ranges of values–they reflect actual differences in abundance throughout the community where some species are extremely common, and some others can be extremely rare–transformed counts (VST) were also centered and scaled by subtracting the mean of each ASV and dividing results by their standard deviation (a process also known as standardization or z-score transformation). We used a divisive clustering approach to

separate ASVs into groups called DIvisive ANAlysis Clustering (diana clustering, implemented in the diana function of the cluster package (Maechler et al., 2021)). To group together ASVs with the same pattern across sample types, the Kendall rank correlation coefficient (τ) was transformed into a distance matrix using the formula:

$$d_{ij} = (1 - \tau_{ij})^2$$

where d_{ij} is the distance between ASV i and ASV j and τ_{ij} is the value of the Kendall correlation. Doing so, the distance of positively correlated ASVs ranged between 0 and 1 whereas the distance of negatively correlated ASVs ranged between 4 and 1, emphasizing divergences and similarities across ASVs.

The resulting hierarchical clustering was then split into groups by using the divisive coefficient obtained from diana analysis (0.99). Groups containing less than 15 ASVs (roughly 5% of the significant ASVs detected with the LRT) were discarded to reduce spurious clusters (Figure 4 reported in the main text).

Differences in mean across sample types were tested using the non-parametric Wilcoxon signed-rank test. Clusters were tested using all possible combinations of levels (pairwise test) and significance was reported using letters (Figure S7 panel a). P-values were adjusted using the Benjamini–Hochberg method (Figure S7 panel b).

Enrichment analysis of taxa and functions

To identify enriched/depleted taxa in the clusters produced we used the hypergeometric distribution. Since bacterial lineages were reported at different taxonomic levels (namely, from Domain to Genus/Species), we tested enriched taxa at all levels available to provide a full overview of the clusters defined in the previous section. The hypergeometric test is based on the hypergeometric distribution to measure the probability of obtaining a given number of successes with a specific number of trials in a defined population (where the number of success and failure are known) (Falcon and Gentleman, 2008). This definition can be applied to a microbial community with known taxonomic composition. Indeed, we tested the probability of having a given number of ASVs assigned to a specific taxon in a group (cluster) of ASVs in respect with the total community. Following this definition, the probability of having x ASVs defined as a specific taxon of interest can be expressed as:

$$P[X = x] = \frac{\binom{m}{x}\binom{n}{k-x}}{\binom{m+n}{k}}$$

where m is the number of ASVs assigned to the taxon of interest in the whole community, n is the number of taxa outside the group that is being tested, and k is the total number of ASVs in the group. Since we don't know *a priori* if the taxon is over- or under-represented, we calculated the observed probability of drawing an ASVs belonging to that taxon in the group (p_{obs}) and we compare it with the expected probability calculated in the whole community (p_{exp}):

$$p_{obs} = \frac{x}{k} \quad ; \quad p_{exp} = \frac{m}{n+k}$$

Then, if $p_{obs} > p_{exp}$, we calculated the probability of having an equal or greater (enriched) number of ASVs of the given taxon. Alternatively, if $p_{obs} < p_{exp}$, we calculated the probability of having an equal or lower (depleted) number of ASVs of the given taxon. This probability represents the p-value of the test obtained using the phyper function of the stats package following the definition reported below:

$$P_{hyper} = \begin{cases} \sum_{x=q}^{k} P\,[X = x] \quad ; \quad P_{hyper} = P[X \geq x] \quad \text{if} \quad p_{obs} > p_{exp} \\ \sum_{x=1}^{q} P\,[X = x] \quad ; \quad P_{hyper} = P[X \leq x] \quad \text{if} \quad p_{obs} < p_{exp} \end{cases}$$

We express the magnitude of the enrichment/depletion of a given taxon in a specific group using the $\log_2$ of fold changes between p_{obs} and p_{exp} (LFC) so that positive values correspond to enriched taxa and negative values correspond to depleted taxa. Obtained p-values were corrected using the Benjamini–Hochberg method to reduce the number of false positives (Table S7). Differently from LRT analysis described above–where we detected the effect of a factor on the overall community without directly comparing the levels of the factor–here we compared the observed probability of having a given number of ASVs assigned to the taxon of interest in a cluster against the same probability calculated in the whole population, hence, we selected significant taxa using both p-value and LFC. Only taxa with a p-value lower than 0.05 and with an LFC value higher than 1 (or less than -1 for depleted taxa) were considered significantly enriched/depleted and used for subsequent analyses.

Similarly to taxonomic enrichment, we detected gene enrichment by using hypergeometric test on gene ontology terms (GO terms) assigned to genes inferred by the PICRUSt2 pipeline (Douglas et al., 2020). Enzyme Commission numbers were converted into GO terms following the mapping file available at: http://www.geneontology.org/external2go/ec2go. We detected over- and under-represented terms by weighting the number of GO terms found in the inferred genome of ASVs in each group based on genome copy number provided by PICRUSt2 (“genome_function_count” of stratified output). Following the definitions reported above, m represents the number of genes with a given GO term in the whole community, n is the number of genes with the same GO term in the inferred genome content of ASVs outside the group that is being tested, and k is the size of the genome content of ASVs included in the group. Terms significantly enriched or depleted were considered to be shared between ASV groups only if they reported the same effect in the two groups considered (both enriched and depleted). The number of shared terms between groups was reported in Figure S8 and Table S8 whereas the results of gene enrichment analysis were reported in Table S9.

Figures

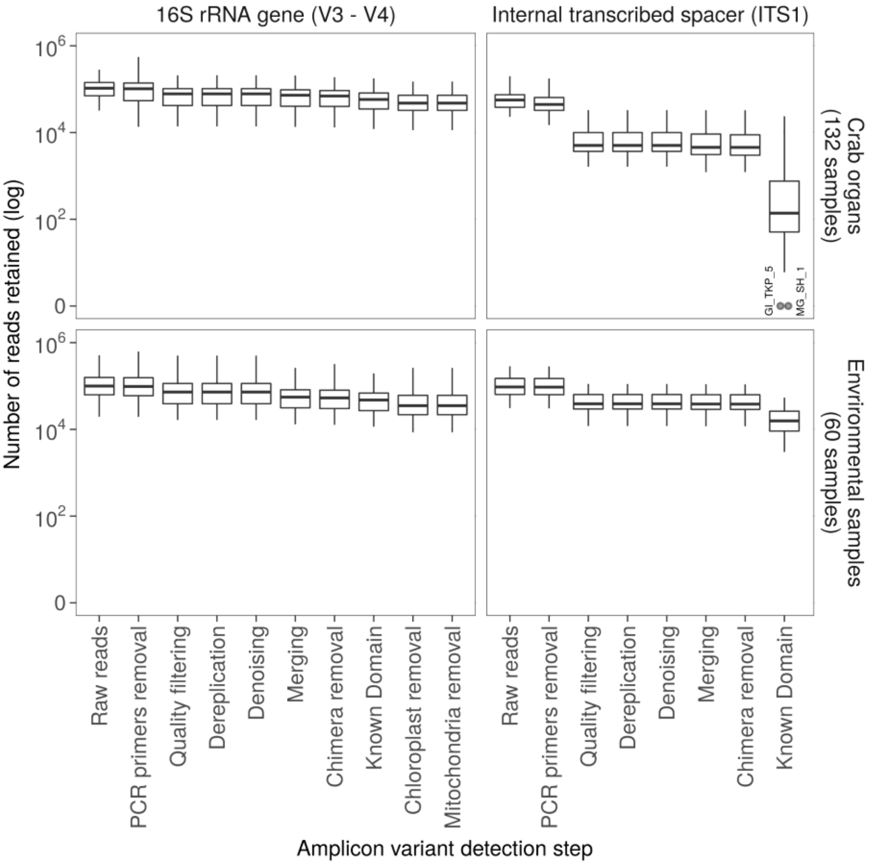

Figure S1: Number of reads retained after each step of the DADA2 workflow. The number of reads processed and retained after each step of the DADA2 workflow (x axis) is reported in the y axis. Results obtained from 16S rRNA gene the ITS1 region amplicons were reported in two different panels and divided according to the sample type (crab's organs or environmental samples). The sample ID of two fungal samples (ITS1 region) which resulted in zero counts after DADA2 pipeline were reported in the plot.

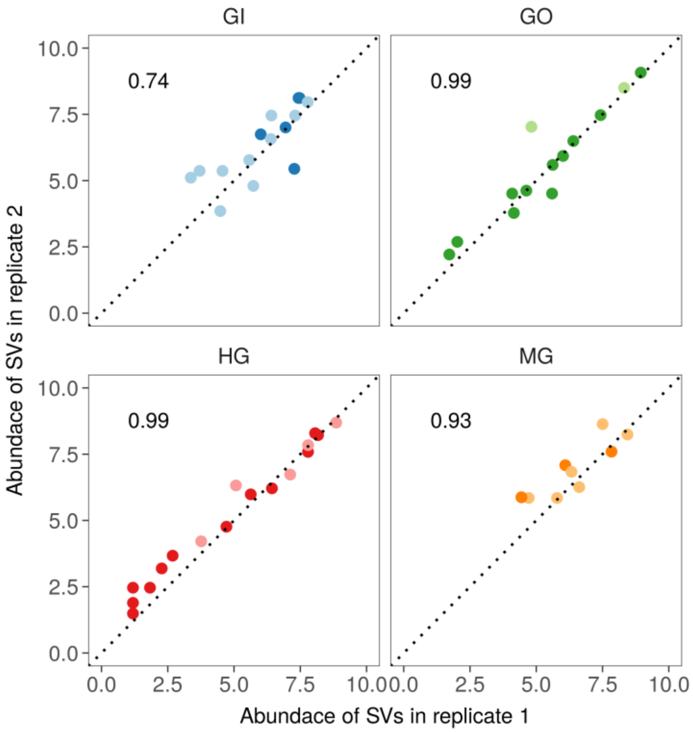

Figure S2: Counts of SVs for each replicate in different crab's organs. The abundance of every ASV is reported for the eight pairwise combinations using the log2-scale (2 contrast and four crab's organs). Colors represent crab's organs (GI, gills, blue; GO, gonads, green; HG, hindgut, red; MG, midgut, orange) whereas samples were reported using different shades of colors (two samples per organ, one darker than the other). Dotted lines represent a perfect correlation (namely a line with slope equal to one and intercept equal to zero) whereas the average value of the Spearman's correlation coefficient was reported at the top left of each panel.

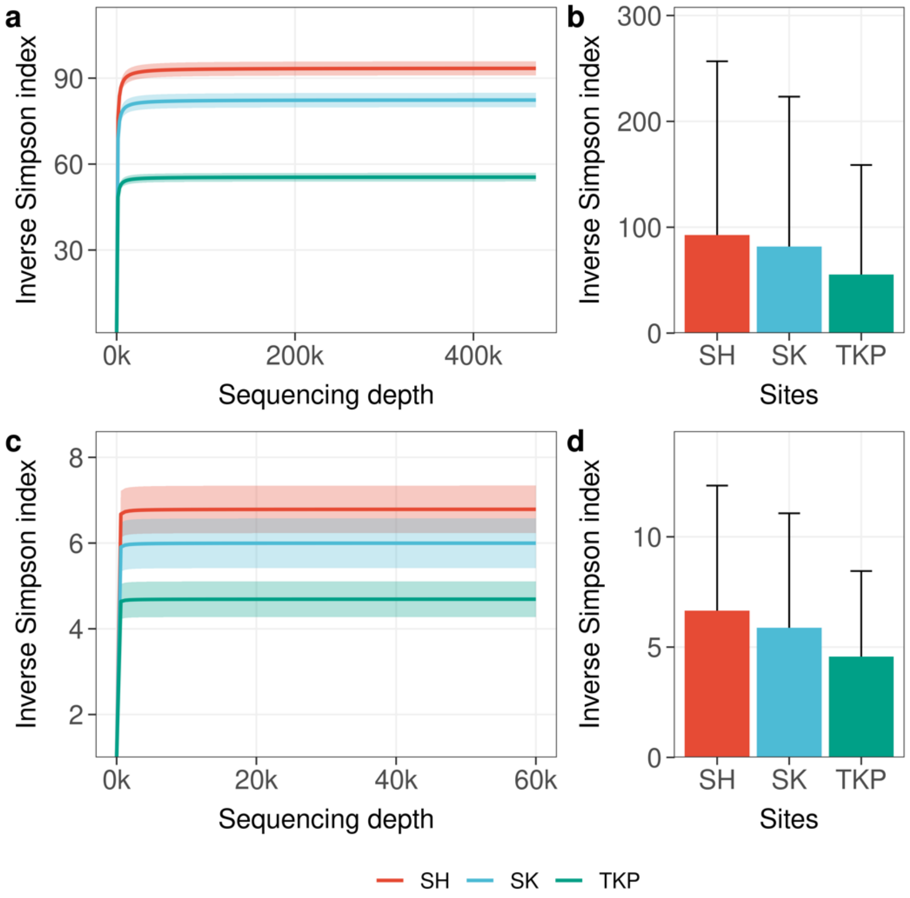

Figure S3: Microbial diversity in different sampling sites. The average of the inverse Simpson index was reported with increasing sampling effort for all sampling sites (SH, Shui Hau; SK, Sai Keng; TKP, To Kwa Peng). Interpolated and extrapolated diversity was reported in panel a and c (16S rRNA gene and ITS1 region, respectively) together with the 98% confidence limits of diversity (iNEXT package). Observed diversity was reported in panel b and d (16S rRNA gene and ITS1 region respectively) with error bars representing the standard deviation of the observed values of diversity.

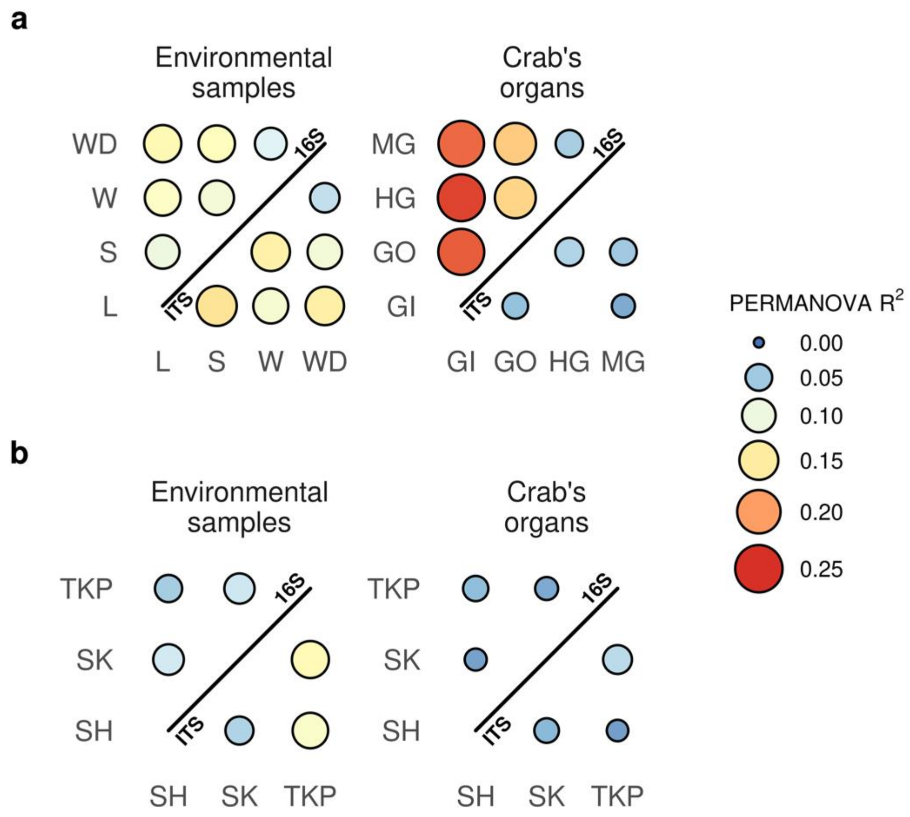

Figure S4: Microbial distribution according to Bray-Curtis distance. Permutational analysis of variance (PERMANOVA) on ordinations reported in Figure 3. A permutational analysis was performed for each pair of organs and environmental samples (panel a) and for each sampling site (panel b). R-squared values of significant contrasts were reported for both 16S (upper triangle) and ITS (lower triangle) counts.

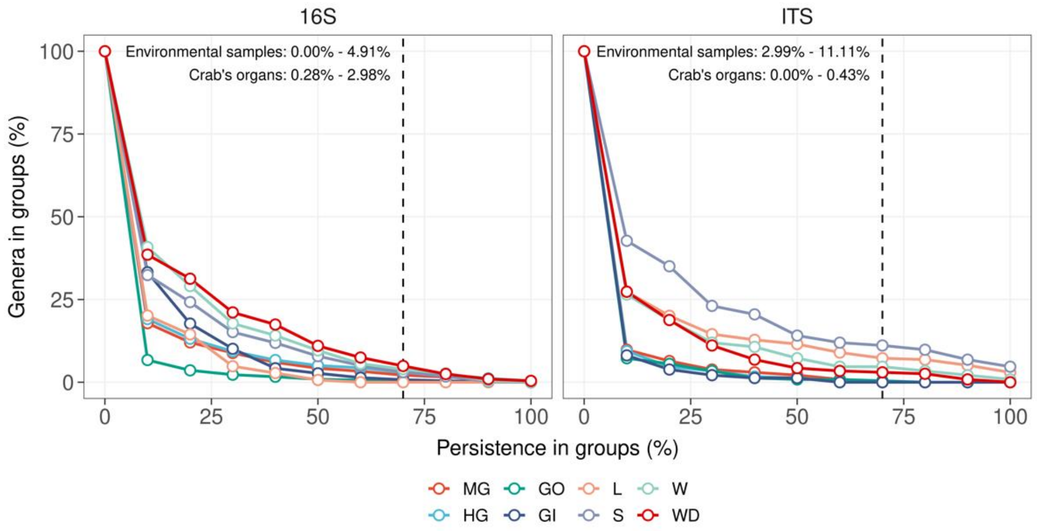

Figure S5: Persistence of genera detected in different groups. The percentage of genera detected in each group (both crab's organs and environmental samples) was plotted against increasing persistence (namely the percentage of samples in which a specific genus has an abundance higher than one). The percentage of genera composing a "soft-core" microbiome (70% of samples with abundance higher than one) was reported in the top part of each panel according to the type of samples considered (crab's organs or environmental samples). The type of amplicon used (16S rRNA gene or ITS-1 region) was reported on the top of each panel. Sample types were abbreviated as follows: MG, midgut; HG, hindgut; GO, gonads; GI, gills; L, litter; S, soil; W, water; WD, water debris.

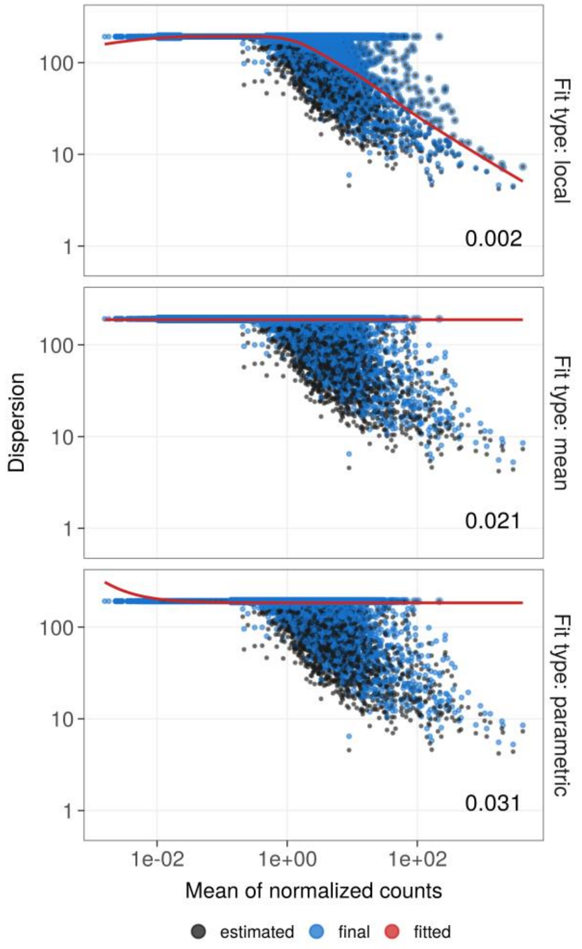

Figure S6: DESeq2 model selection based on the median of residuals on the log scale. Per-ASV dispersion estimates (x-axis) were plotted together with the mean of normalized counts obtained from DESeq2 (y-axis). Black dots represent the maximum-likelihood estimates (MLEs) for each ASVs using only the respective data, fitted models were reported using a red line, and the final estimates used for testing were reported using blue dots. Outliers were highlighted with blue circles. All fit types available were tested and the median absolute log residuals obtained were reported in the bottom right corner of each plot.

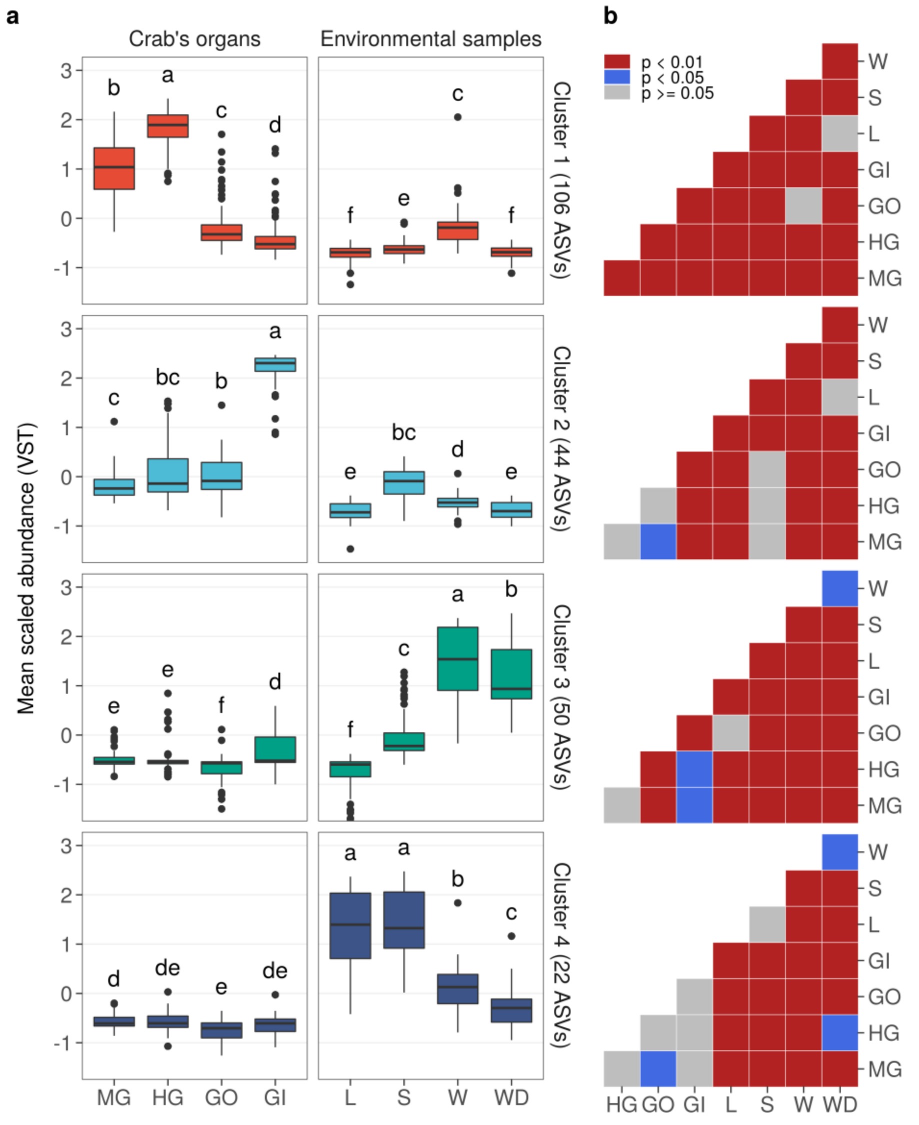

Figure S7: Differences across sample types in the four clusters detected with divisive hierarchical clustering. a) The abundance values of all ASVs included in clusters was reported using the box and whisker visualization where: the interquantile range (IQR, the space between Q_3 and Q_1) is represented by the "box", the "whiskers" extend to the most extreme data point which is no more than 1.5 times the IQR away from the box, and the median (Q_2) is reported with a black horizontal line. Outliers, namely those observations exceeding 1.5 times the IQR, were reported using black dots. Lowercase letters on top of each box represent significant differences across sample types: if two means were significantly different, all letters on top of the two boxes must be different; if two means were equal, at least one letter must be the same. Crab's organs and environmental samples were reported in two different panels (vertically) but all pairwise contrasts were included in the analysis. b) Adjusted p-values between pairwise contrasts. All contrasts were tested using Wilcoxon signed-rank test and results were reported as an heatmap where red and blue cells represent two levels of significance ($p < 0.01$ and $p < 0.05$, respectively) whereas gray cells indicate non statistical significance ($p > 0.05$).

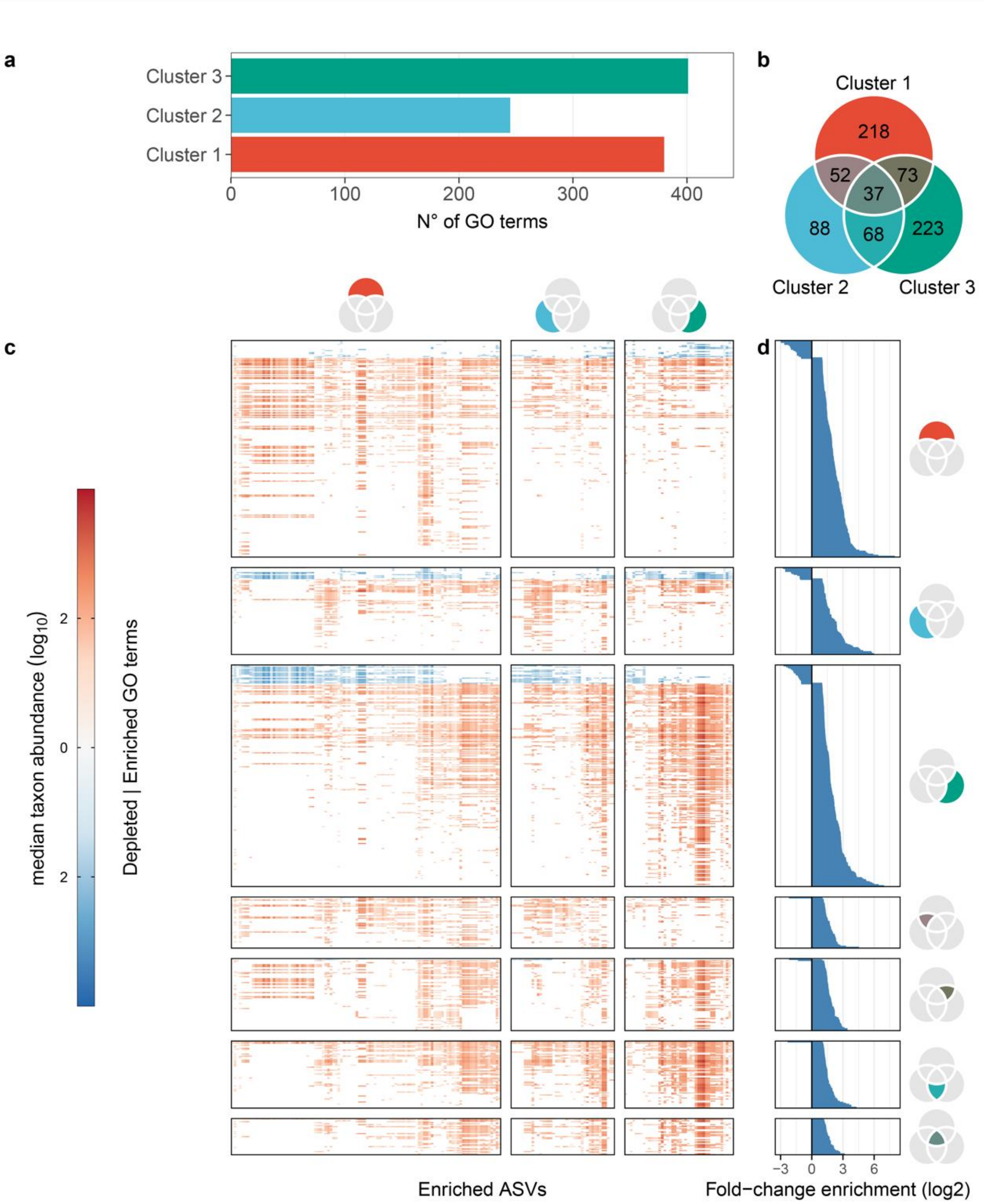

Figure S8: Enrichment analysis of "Gene Ontology" terms associated with detected functions in ASV clusters. a) Number of GO terms associated to bacterial functions detected in each ASV cluster (cluster 4 was not reported since it was entirely composed of ASVs belonging to Fungal domain and so was not functionally profiled). b) Venn diagram of GO terms significantly enriched in ASV clusters. Sets correspond to ASV clusters whereas the number of GO terms significantly enriched was reported in each intersection. c) Median abundance of GO terms significantly enriched (y-axis) in the predicted genome of ASVs (x-axis) assigned to different clusters. The median taxon abundance was reported using different shades of red for enriched terms—namely those detected with a higher frequency in respect to the whole population–and blue for depleted terms—namely those detected with a lower frequency than the rest of the population. The plot was vertically divided according to

clusters whereas it was horizontally divided according to Venn diagram sections reported in panel b. d) Mean enrichment fold changes associated to each term. Fold-changes were transformed using the logarithmic function (with base equal two) to report enriched and depleted terms symmetrically around the zero.

References

Alberdi, A., Gilbert, M.T.P., 2019. A guide to the application of Hill numbers to DNA-based diversity analyses. Mol Ecol Resour 19, 804–817. https://doi.org/10.1111/1755-0998.13014

Anders, S., Huber, W., 2010. Differential expression analysis for sequence count data. Genome Biol 11, R106. https://doi.org/10.1186/gb-2010-11-10-r106

Beckers, B., Op De Beeck, M., Thijs, S., Truyens, S., Weyens, N., Boerjan, W., Vangronsveld, J., 2016. Performance of 16s rDNA Primer Pairs in the Study of Rhizosphere and Endosphere Bacterial Microbiomes in Metabarcoding Studies. Front. Microbiol. 7. https://doi.org/10.3389/fmicb.2016.00650

Bulgarelli, D., Rott, M., Schlaeppi, K., Ver Loren van Themaat, E., Ahmadinejad, N., Assenza, F., Rauf, P., Huettel, B., Reinhardt, R., Schmelzer, E., Peplies, J., Gloeckner, F.O., Amann, R., Eickhorst, T., Schulze-Lefert, P., 2012. Revealing structure and assembly cues for Arabidopsis root-inhabiting bacterial microbiota. Nature 488, 91–95. https://doi.org/10.1038/nature11336

Callahan, B.J., McMurdie, P.J., Rosen, M.J., Han, A.W., Johnson, A.J.A., Holmes, S.P., 2016. DADA2: High-resolution sample inference from Illumina amplicon data. Nature Methods 13, 581–583. https://doi.org/10.1038/nmeth.3869

Chao, A., Gotelli, N.J., Hsieh, T.C., Sander, E.L., Ma, K.H., Colwell, R.K., Ellison, A.M., 2014. Rarefaction and extrapolation with Hill numbers: a framework for sampling and estimation in species diversity studies. Ecological Monographs 84, 45–67. https://doi.org/10.1890/13-0133.1

Chao, A., Jost, L., 2012. Coverage-based rarefaction and extrapolation: standardizing samples by completeness rather than size. Ecology 93, 2533–2547. https://doi.org/10.1890/11-1952.1

Conway, J.R., Lex, A., Gehlenborg, N., 2017. UpSetR: an R package for the visualization of intersecting sets and their properties. Bioinformatics 33, 2938–2940. https://doi.org/10.1093/bioinformatics/btx364

Deshpande, V., Wang, Q., Greenfield, P., Charleston, M., Porras-Alfaro, A., Kuske, C.R., Cole, J.R., Midgley, D.J., Tran-Dinh, N., 2016. Fungal identification using a Bayesian classifier and the Warcup training set of internal transcribed spacer sequences. Mycologia 108, 1–5. https://doi.org/10.3852/14-293

Douglas, G.M., Maffei, V.J., Zaneveld, J.R., Yurgel, S.N., Brown, J.R., Taylor, C.M., Huttenhower, C., Langille, M.G.I., 2020. PICRUSt2 for prediction of metagenome functions. Nat Biotechnol 38, 685–688. https://doi.org/10.1038/s41587-020-0548-6

Falcon, S., Gentleman, R., 2008. Hypergeometric testing used for gene set enrichment analysis, in: Bioconductor Case Studies. Springer New York, New York, NY, pp. 207–220. https://doi.org/10.1007/978-0-387-77240-0$_1$4

Good, I.J., 1953. The Population Frequencies of Species and the Estimation of Population Parameters. Biometrika 40, 237. https://doi.org/10.2307/2333344

Herlemann, D.P., Labrenz, M., Jürgens, K., Bertilsson, S., Waniek, J.J., Andersson, A.F., 2011. Transitions in bacterial communities along the 2000 km salinity gradient of the Baltic Sea. ISME J 5, 1571–1579. https://doi.org/10.1038/ismej.2011.41

Hsieh, T.C., Ma, K.H., Chao, A., 2016. iNEXT: an R package for rarefaction and extrapolation of species diversity (H ill numbers). Methods Ecol Evol 7, 1451–1456. https://doi.org/10.1111/2041-210X.12613

Klindworth, A., Pruesse, E., Schweer, T., Peplies, J., Quast, C., Horn, M., Glöckner, F.O., 2013. Evaluation of general 16S ribosomal RNA gene PCR primers for classical and next-generation sequencing-based diversity studies. Nucleic Acids Research 41, e1–e1. https://doi.org/10.1093/nar/gks808

Lex, A., Gehlenborg, N., Strobelt, H., Vuillemot, R., Pfister, H., 2014. UpSet: Visualization of Intersecting Sets. IEEE Trans. Visual. Comput. Graphics 20, 1983–1992. https://doi.org/10.1109/TVCG.2014.2346248

Love, M.I., Huber, W., Anders, S., 2014. Moderated estimation of fold change and dispersion for RNA-seq data with DESeq2. Genome Biol 15, 550. https://doi.org/10.1186/s13059-014-0550-8

Maechler, M., Rousseeuw, P., Struyf, A., Hubert, M., Hornik, K., 2021. cluster: Cluster analysis basics and extensions (manual).

Martin, M., 2011. Cutadapt removes adapter sequences from high-throughput sequencing reads. EMBnet j. 17, 10. https://doi.org/10.14806/ej.17.1.200

Murali, A., Bhargava, A., Wright, E.S., 2018. IDTAXA: a novel approach for accurate taxonomic classification of microbiome sequences. Microbiome 6, 140. https://doi.org/10.1186/s40168-018-0521-5

Obertegger, U., Bertilsson, S., Pindo, M., Larger, S., Flaim, G., 2018. Temporal variability of bacterioplankton is habitat driven. Mol Ecol 27, 4322–4335. https://doi.org/10.1111/mec.14855

Oksanen, J., Blanchet, F.G., Friendly, M., Kindt, R., Legendre, P., McGlinn, D., Minchin, P.R., O'Hara, R.B., Simpson, G.L., Solymos, P., Stevens, M.H.H., Szoecs, E., Wagner, H., 2019. vegan: Community ecology package (manual).

Quast, C., Pruesse, E., Yilmaz, P., Gerken, J., Schweer, T., Yarza, P., Peplies, J., Glöckner, F.O., 2012. The SILVA ribosomal RNA gene database project: improved data processing and web-based tools. Nucleic acids research 41, D590–D596.

Rosso, F., Tagliapietra, V., Albanese, D., Pindo, M., Baldacchino, F., Arnoldi, D., Donati, C., Rizzoli, A., 2018. Reduced diversity of gut microbiota in two Aedes mosquitoes species in areas of recent invasion. Sci Rep 8, 16091. https://doi.org/10.1038/s41598-018-34640-z

Weiss, S., Xu, Z.Z., Peddada, S., Amir, A., Bittinger, K., Gonzalez, A., Lozupone, C., Zaneveld, J.R., Vázquez-Baeza, Y., Birmingham, A., Hyde, E.R., Knight, R., 2017. Normalization and microbial differential abundance strategies depend upon data characteristics. Microbiome 5, 27. https://doi.org/10.1186/s40168-017-0237-y

Wright, E.S., 2016. Using DECIPHER v2. 0 to analyze big biological sequence data in R. R Journal 8.
